# Influence of Light and Temperature on Gene Expression Leading to Accumulation of Specific Flavonol Glycosides and Hydroxycinnamic Acid Derivatives in Kale (*Brassica oleracea* var. *sabellica*)

**DOI:** 10.3389/fpls.2016.00326

**Published:** 2016-03-30

**Authors:** Susanne Neugart, Angelika Krumbein, Rita Zrenner

**Affiliations:** Leibniz Institute of Vegetable and Ornamental Crops Großbeeren/Erfurt e.V.Großbeeren, Germany

**Keywords:** *Brassica*, flavonol glycosides, temperature, photosynthetically active radiation (PAR), gene expression, microarray

## Abstract

Light intensity and temperature are very important signals for the regulation of plant growth and development. Plants subjected to less favorable light or temperature conditions often respond with accumulation of secondary metabolites. Some of these metabolites have been identified as bioactive compounds, considered to exert positive effects on human health when consumed regularly. In order to test a typical range of growth parameters for the winter crop *Brassica oleracea* var. *sabellica*, plants were grown either at 400 μmol m^−2^ s^−1^ or 100 μmol m^−2^ s^−1^ at 10°C, or at 400 μmol m^−2^ s^−1^ with 5 or 15°C. The higher light intensity overall increased flavonol content of leaves, favoring the main quercetin glycosides, a caffeic acid monoacylated kaempferol triglycoside, and disinapoyl-gentiobiose. The higher temperature mainly increased the hydroxycinnamic acid derivative disinapoyl-gentiobiose, while at lower temperature synthesis is in favor of very complex sinapic acid acylated flavonol tetraglycosides such as kaempferol-3-*O*-sinapoyl-sophoroside-7-*O*-diglucoside. A global analysis of light and temperature dependent alterations of gene expression in *B. oleracea* var. *sabellica* leaves was performed with the most comprehensive *Brassica* microarray. When compared to the light experiment much less genes were differentially expressed in kale leaves grown at 5 or 15°C. A structured evaluation of differentially expressed genes revealed the expected enrichment in the functional categories of e.g. protein degradation at different light intensities or phytohormone metabolism at different temperature. Genes of the secondary metabolism namely phenylpropanoids are significantly enriched with both treatments. Thus, the genome of *B. oleracea* was screened for predicted genes putatively involved in the biosynthesis of flavonoids and hydroxycinnamic acid derivatives. All identified *B. oleracea* genes were analyzed for their most specific 60-mer oligonucleotides present on the 2 × 105 K format *Brassica* microarray. Expression differences were correlated to the structure-dependent response of flavonoid glycosides and hydroxycinnamic acid derivatives to alterations in either light or temperature. The altered metabolite accumulation was mainly reflected on gene expression level of core biosynthetic pathway genes and gave further hints to an isoform specific functional specialization.

## Introduction

Kale (*Brassica oleracea* var. *sabellica*) is an increasingly popular leafy vegetable of the Brassicaceae, the plant family with many important food crops. Eating these vegetables is supposed to be health-supporting since a number of epidemiological studies have identified a positive association between its consumption and human health (Verkerk et al., 2009). These positive effects have been mainly attributed to secondary metabolites like glucosinolates and flavonoids present in plant tissues (Calderón-Montaño et al., 2011; Maalik et al., 2014). Kale as a typical winter crop is mainly cultivated in Northern and Central Europe, e.g., in Germany as well as in North America. During cultivation kale is exposed to low or even freezing temperature conditions and it also has to grow at low levels of photosynthetically active radiation (PAR).

It is undoubtable that light and temperature are most important signals for development, growth and metabolism. The light environment is perceived by several receptors, controlling acclimation and avoidance responses to limiting or excess light (Chen et al., 2004; Li et al., 2009) thus creating a complex network of light response (Casal, 2013). Upon temperature changes signaling pathways trigger lower temperature response (Yamaguchi-Shinozaki and Shinozaki, 2006) finally contributing to cold adaptation and freezing tolerance (Knight and Knight, 2012). Furthermore, light and temperature signaling networks are integrated in an interconnected system that may be evolutionarily favorable (Franklin et al., 2014).

**Figure 1:**
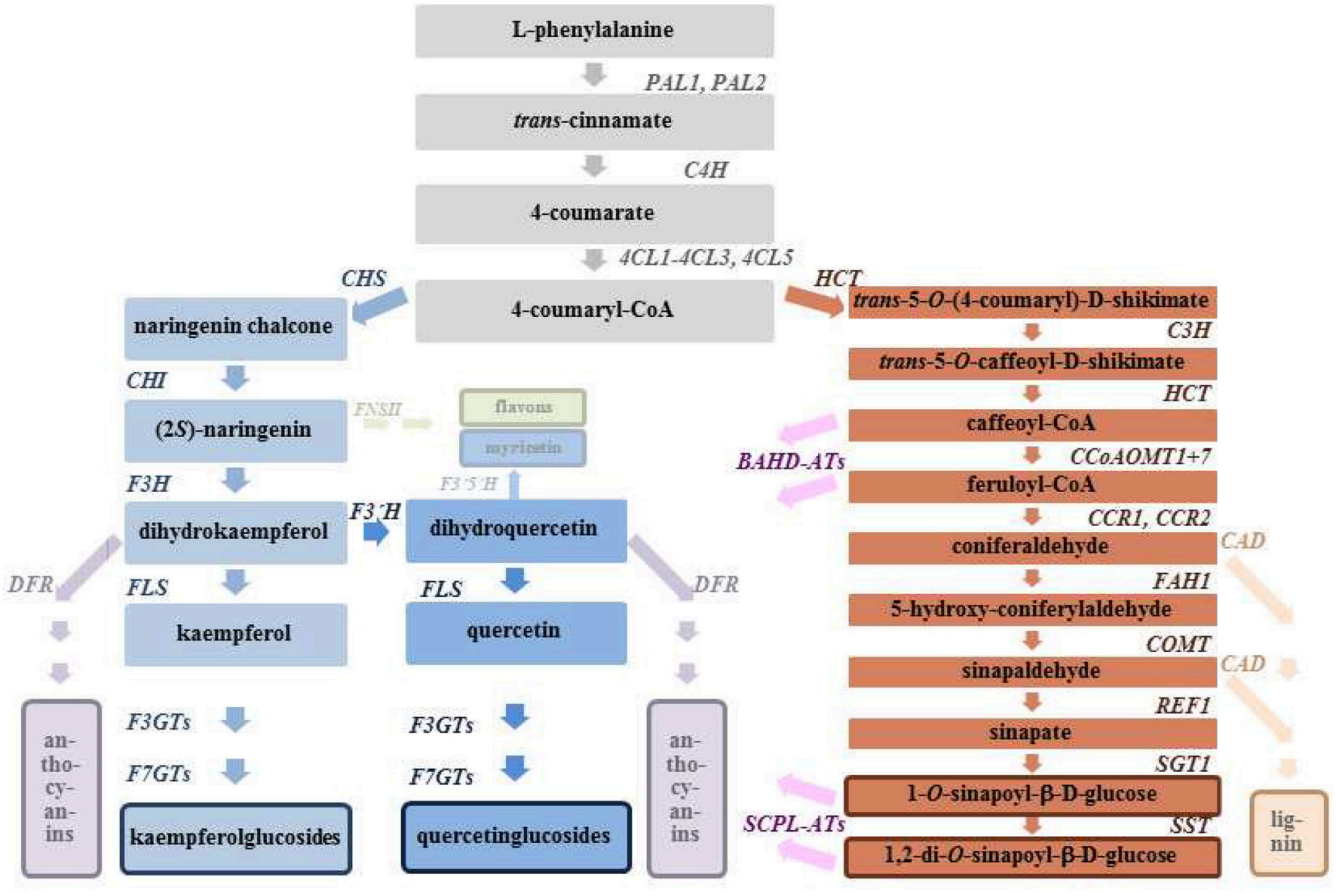
**Phenylpropanoid metabolism and related pathways**. Identified *Arabidopsis* genes coding for respective enzyme activities are listed. *4CL*, 4-coumarate-CoA ligase (*CL1, At1g51680; 4CL2, At3g21240; 4CL3, At1g65060; 4CL5, At3g21230*)*; BAHD-ATs*, acyl-CoA dependent acyltransferase (e.g., *AT5MAT, At3g29590*); *C3H*, coumarate 3′-hydroxylase (*REF8, At2g40890*)*; C4H*, cinnamate 4-hydroxylase (*C4H, At2g30490*); *CAD*, cinnamylalcohol dehydrogenase (*CAD4, At3g19450; CAD5, At4g34230*); *CCoAOMT*, caffeoyl-CoA *O*-methyltransferase (*CCoAOMT1, At4g34050; CCoAOMT7, At4g26220*); *CCR*, cinnamoyl-CoA reductase (*CCR1, At1g15950; CCR2, At1g15950*); *CHI*, chalcone isomerase (*TT5, At3g55120*); *CHS*, chalcone synthase (*TT4, At5g13930*); *COMT*, flavone 3′-*O*-methyltransferase (*COMT1, At5g54160*); *DFR*, dihydroflavonol 4-reductase (*DFR, At5g42800*); *F3GTs*, flavonol 3-*O*-glucosyltransferase (e.g., *UGT73B2, At 4g34135*); *F3H*, flavanone 3-hydroxylase (*TT6, At3g51240*); *F3*′*H*, flavonoid 3′-hydroxylase (*TT7, At5g07990*); *F3*′*5*′*H*, flavonoid 3′5′-hydroxylase (*not present in A. thaliana*); *F7GTs*, flavonol 7-*O*-glucosyltransferase (e.g., *UGT73C6, At2g36790*); *FAH1*, ferulate 5-hydroxylase (*FAH1, At4g36220*); *FLS*, flavonol synthase (*FLS1, At5g08640; FLS3, At5g63590*); *FNSII*, flavone synthase (*not present in A. thaliana*); *HCT*, shikimate *O*-hydroxycinnamoyltransferase (*HCT, At5g48930*); *PAL*, phenylalanine ammonia lyase (*PAL1, At2g37040; PAL2, At3g53260*); *REF1*, sinapaldehyde dehydrogenase (*ALDH2C4, At3g24503*); *SCPL-ATs*, acylglucose dependent acyltransferases (e.g., SCPL10, *AtSAT, At2g23000*); *SGT1*, sinapate 1-glucosyltransferase (*UGT84A2, At3g21560; UGT84A1, At4g15480; UGT84A3, At4g15490; UGT84A4, At4g15500*); *SST*, sinapoylglucose *O*-sinapoyltransferase (*SCPL9, At2g23010; SCPL13, At2g22980*).

When subjected to stress plants often respond with accumulation of secondary metabolites, especially flavonoids (Falcone Ferreyra et al., 2012). These metabolites may play a role in adaptation to the environment and in overcoming stress conditions (Ramakrishna and Ravishankar, 2011). Mutant phenotypes can be detected through altered pigmentation and corresponding genes involved in the flavonoid biosynthetic pathway have been characterized in several plant species (Lepiniec et al., 2006). A scheme of the phenylpropanoid pathways and *Arabidopsis* genes coding for respective enzyme activities are listed in Figure 1.

It is known that flavonoids and hydroxycinnamic acid derivatives protect plants from damage caused by high levels of sunlight. Besides acting as shielding components due to their absorption maxima phenolic compounds are also scavengers of reactive oxygen species (ROS) (Edreva, 2005). The reduction of sunlight has been associated with lower concentrations of flavonoids for the perennial woody species *Ligustrum vulgare* and *Phillyrea latifoilia* (Agati and Tattini, 2010) as well as for the annual leafy species *B. rapa* and *B. juncea* (Fallovo et al., 2011). The effects of different light conditions on the control of flavonoid biosynthesis is mainly exerted through R2R3 MYB transcription factors, regulating the biosynthesis of distinct flavonoids for example in fruits (Zoratti et al., 2014). It was demonstrated that low temperature enhances phenolic compounds and total flavonoids due to an enzymatic repair inhibition combined with higher quantities of ROS (Bilger et al., 2007; Klimov et al., 2008; Moheb et al., 2011; Lavola et al., 2013). Moreover, a decrease in quercetin glycosides and caffeic acid derivatives with a temperature increase of 2°C was shown for willow (*Salix myrsinifolia*) (Nybakken et al., 2012).

Compared to other leafy vegetables such as rocket salads or lettuce, kale leaves have high concentrations of a large number of naturally occurring flavonol glycosides and hydroxycinnamic acid derivatives (Ferreres et al., 2009; Lin and Harnly, 2009; Olsen et al., 2009a; Schmidt et al., 2010b; Fiol et al., 2012). Up to now, a total number of 71 flavonol glycosides has been identified in kale by HPLC-DAD-MS^n^. So far, 27 non-acylated, 30 monoacylated and 14 diacylated glycosides have been described based on the flavonol aglycones quercetin, kaempferol and isorhamnetin (Schmidt et al., 2010b). The main flavonol glycosides in kale are non-acylated and monoacylated quercetin and kaempferol glucosides, with the majority of flavonol glucosides being acylated with hydroxycinnamic acids (Schmidt et al., 2010b). Unlike *Arabidopsis thaliana* containing only non acylated flavonol glycosides (Saito et al., 2013), the acylation pattern makes kale a very useful Brassicaceae model plant to analyze the biosynthesis and function of hydroxycinnamic acid acylated flavonol glycosides. The main free hydroxycinnamic acids derivatives in kale are caffeoylquinic acid (chlorogenic acid), disinapoyl-gentiobiose and sinapoyl-feruloyl-gentiobiose (Zietz et al., 2010; Fiol et al., 2012). This is also very different from *A. thaliana* that mainly contains sinapoylcholine, sinapoylglucose, and sinapoylmalate as free hydroxycinnamic acid derivatives (Vogt, 2010; Fraser and Chapple, 2011).

Based on field experiments in a typical growing season in Germany multiple regressions with the factors temperature and radiation were calculated for kale's flavonol glycosides and underlined a structure-specific response (Schmidt et al., 2010a; Neugart et al., 2012a). In this field experiment the effect of PAR remained remarkably low thus indicating that the influence of PAR on the investigated structures might be suppressed due to the decreasing temperatures. In a consecutive experiment with young kale plants exposed for 1 week to low temperatures (5 and 10°C) with diverse PAR levels (200 to 800 μmol m^−2^ s^−1^) in growth chambers the specific effect of low temperature and moderate PAR levels on structurally different flavonoid glycosides was established further (Neugart et al., 2013). Under these constant conditions the specific impact of PAR on the accumulation of flavonoid glycosides was mainly exerted on the levels of quercetin triglycosides, however, expression of respective genes was unaffected assuming an adaptation response of the rather young plants (Neugart et al., 2013).

The aim of this study was to (I) distinguish the effect of ecologically relevant PAR (400 μmol m^−2^ s^−1^ or 100 μmol m^−2^ s^−1^ at 10°C) and temperature levels (5 or 15°C at 400 μmol m^−2^ s^−1^) on structurally different flavonoid glycosides and hydroxycinnamic acid derivatives in adult kale plants under defined conditions in climate chambers. These conditions are comparable to conditions in the field when kale is cultivated during central European winter thus resembling typical light and temperature conditions in a growing season. We want to (II) further differentiate the plant's response to PAR and temperature on expression level using the most comprehensive 2 × 104 k format *Brassica* Array. Consequently, the focus of the transcriptome analysis will be directed toward genes putatively involved in the phenylpropanoid pathway.

## Materials and methods

### Plant material and experimental design

Kale (*B. oleracea* var. *sabellica*) plants of the cultivar Winterbor (by Bruno Nebelung, Norken, Germany) were grown in pots under greenhouse conditions for 12 weeks. For both experiments, the plants were cultivated in potting soil (Standard soil Type 1, Fitz Kausek GmbH & Co.KG, Mittenwalde, Germany). Water supply was given as needed by the plants and fertilization was given as nutrient solution (pH 6.4, P 41 mg L^−1^, Mg 43 mg L^−1^, NO_3_154 mg L^−1^, Ca 205 mg L^−1^, K 230 mg L^−1^, SO_4_ 280 mg L^−1^, Zn 0.82 mg L^−1^) every 4 weeks. The adult kale plants were then transferred to climate chambers. The plants were treated either with (I) different PAR, with 100 μmol m^−2^ s^−1^ and 400 μmol m^−2^ s^−1^, measured with light meter Li-250 (Li-COR, USA), at 10°C or with (II) different temperatures, at 5 and 15°C with 400 μmol m^−2^ s^−1^. The PAR radiation was applied from 8 am to 6 pm each day. These conditions are comparable to conditions in the field during cultivation in central European winter season. The temperature was measured with a Pt1000 sensor integrated in the climate chamber with a fluctuation range of ±0.3°C independent from the PAR level. The relative humidity in the climate chambers was 70%. The duration of each experiment was 4 weeks. Leaves of six fully developed plants were harvested using three repetitions per treatment.

### Sample preparation and extraction of flavonol glycosides and hydroxycinnamic acid derivatives

A mixed sample of leaves (without petiole and midrib) taken from six fully developed plants per replication was frozen (−40°C), lyophilized for 72 h and subsequently ground to a fine powder. The samples were either stored in darkness at room temperature less than 6 months until required for analysis or they were immediately used for total RNA extraction. To analyze flavonol glycosides and hydroxycinnamic acid derivatives samples were extracted with 60% aqueous methanol as described (Neugart et al., 2012b).

### HPLC-DAD-ESI-MS^n^ measurement of flavonol glycosides and hydroxycinnamic acid derivatives

An HPLC series 1100 by Agilent (Waldbronn, Germany), consisting of a degaser, binary pump, autosampler, column oven and photodiode array detector, was used to quantify the flavonol glycosides and hydroxycinnamic acid derivatives. An ion trap (Agilent series 1100 MSD) with an electrospray ionization (ESI) ion source in negative ionization mode was used as the mass spectrometer. Nitrogen was used as the dry gas (12 L min^−1^, 350°C) and nebulizer gas (40 psi). Helium was the inert, collision gas in the ion trap. The flavonol glycosides and hydroxycinnamic acid derivatives were separated on a Phenomenex Prodigy column (150 × 3.0 mm, ODS 3, 5 ⌈m, 100 A°) with a C18 security guard column (4 × 3.0 mm, ODS 3, 5 ⌈m, 100 A°) at a temperature of 30°C using a water/acetonitrile gradient (Schmidt et al., 2010a). Solvent A consisted of 99.5% water and 0.5% acetic acid; solvent B was 100% acetonitrile. The following gradient was used for eluent B: 5–7% (0–12 min), 7–9% (12–25 min), 9–12% (25–45 min), 12–15% (45–100 min), 15% isocratic (100–150 min), 15–50% (150–155 min), 50% isocratic (155–165 min), 50–5% (165–170 min) and 5% isocratic (170–175 min). Separation was performed using a flow of 0.4 ml min^−1^; the measured detector wavelength for quantification was set at 370 nm for non-acylated flavonol glycosides, 330 nm for acylated flavonol glycosides and 320 nm for hydroxycinnamic acid derivatives (Olsen et al., 2009a; Schmidt et al., 2010a). The standards quercetin-3-*O*-glucoside, kaempferol-3-*O*-glucoside and chlorogenic acid (Carl Roth GmbH, Karlsruhe, Germany) were used to obtain an external calibration curve ranging from 0.1 to 10 mg 100 ml^−1^. Mass optimization for the ion optics of the mass spectrometer was performed for quercetin m/z 301. In addition, arbitrary m/z 1000 was used as the target mass in the auto mode to include higher mass fragments. The MS^n^ experiments were performed in auto or manual mode up to MS4 in a scan from m/z 200 to 2000.

### Microarray analysis

The microarray analysis was performed as described (Mewis et al., 2012). Lyophilized and ground leaf material was immediately used for total RNA extraction with the RNeasy Plant Mini Kit including the on-column DNase digestion step (Qiagen GmbH, Hilden, Germany). Microarray analysis was done with 1 mg of total RNA isolated from each of three to four replicates of plants grown at 10°C and 100 μmol m^−2^ s^−1^ (LL, low light) or at 10°C and 400 μmol m^−2^ s^−1^ (HL, high light), and plants grown at 400 μmol m^−2^ s^−1^ and 5°C (LT, low temperature) or at 400 μmol m^−2^ s^−1^ and 15°C (HT, high temperature). Agilent One-Color Gene Expression Microarray analysis following the recommendation of MIAME (www.mged.org) was performed at Beckman Coulter Genomics (Morrisville, NC, United States; www.beckmangenomics.com/) using their 2 × 105 K format *Brassica* Array (Custom Gene Expression Microarray G4503A, 2 × 105 K; (Trick et al., 2009); brassica.bbsrc.ac.uk/). Raw data files are available with accession number E-MTAB-2677 at ArrayExpress (www.ebi.ac.uk/arrayexpress/). The Open Source Microarray Processing Software Robin was used (Lohse et al., 2010; mapman.gabipd.org/web/guest/home) including quality control tools using the default settings. Here the data were shifted to be zero centered and scaled to have unit variance. The PCA was performed by singular value decomposition. The analysis settings were as follows: Normalization with background correction “subtraction” and between array normalization method “scale” with the analysis strategy of linear models (package limma). Results were given as log fold change of expression in relation to different light or temperature treatment. The assignment of the different genes was done by comparison of the translated protein sequences of the 95 K *Brassica* unigene set with the Arabidopsis TAIR9 database using the newly developed Mercator pipeline for automated sequence annotation (Lohse et al., 2014; mapman.gabipd.org/web/guest/app/mercator). For each identifier the gene with the highest homology was provided with identifier and description. The respective bitscores were classified as follows: very weakly similar (bitscore smaller than 100); weakly similar (bitscore 101–200); moderately similar (bitscore 201–500); highly similar (bitscore greater than 500). Assignment of the different genes represented by identifiers to respective bins and visualization of data sets was realized using MapMan 3.5.1 (Thimm et al., 2004; http://mapman.gabipd.org/web/guest/mapman-download). Complete lists of significant alterations of mRNA abundance induced by either LL, or HL, and either LT, or HT are given as Supplementary Tables 1–4.

### Statistical analysis

Statistical analyses were conducted with the software STATISTICA 9 for Windows (version 9, Statsoft Inc.). Differences due to temperature or radiation were statistically analyzed by a one-factorial ANOVA by a comparison of the factor levels using Tukey's HSD test. The individual values of flavonol gylcosides and hydroxycinnamic acid derivative represent the mean of 3 biological replicates (each biological replicate contained 6 fully developed plants). All tests were conducted at a significance level of 5%.

## Results and discussion

As a distinctive winter crop, kale has to handle low levels of PAR and also low temperature. However, under field conditions there was a certain interaction of PAR and temperature as previously shown for the formation of flavonol glycosides (Neugart et al., 2012a). Therefore, the present study used defined conditions in climate chambers to elucidate the specific effect of either low to moderate PAR levels or typical winter temperature. In order to gain a deeper insight into the adaptation processes when exposed to different PAR levels (100 μmol m^−2^ s^−1^ and 400 μmol m^−2^ s^−1^ at 10°C) and temperatures (5 and 15°C, at 400 μmol m^−2^ s^−1^), the plant's response was evaluated on expression level using microarrays. In addition to the global analysis of transcriptomic alterations, a specific focus of the expression analysis was directed toward genes putatively involved in the phenylpropanoid pathway of *B. oleracea*.

**Figure 2:**
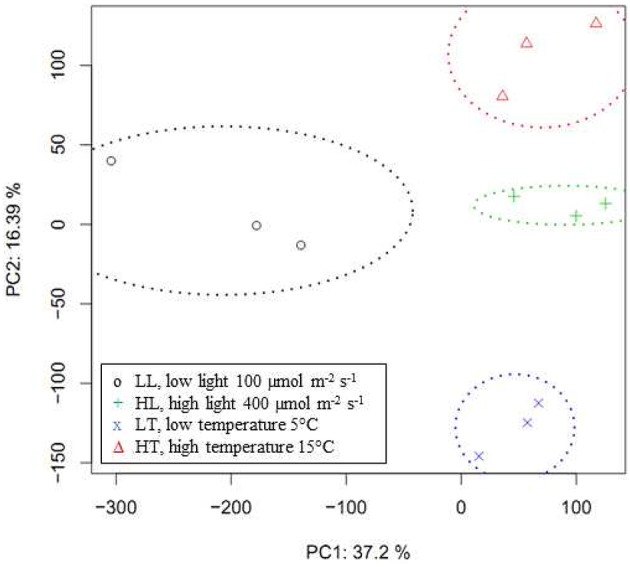
**Principal components analysis of expression data**. Score plot of the principal components analysis of normalized *Brassica* Array expression values analyzed with open source microarray processing software Robin. PC1 with standard deviation of 134 explaining a proportion of variance of 0.372, and PC2 with standard deviation of 89, explaining a proportion of variance of 0.164 and a cumulative proportion of 0.536 are shown. PC3, standard deviation 75, proportion of variance 0.115, and PC4, standard deviation 60, proportion of variance 0.075 with a cumulative proportion of 0.726 are not shown.

The comprehensive gene expression analysis was performed using the 2 × 105 K format *Brassica* Array based on the 95 K *Brassica* unigene set (Trick et al., 2009) and the microarray processing software Robin (Lohse et al., 2010; mapman.gabipd.org/web/guest/home) using default settings for quality assessment and normalization of data. The principal component analysis of all expression data demonstrates that 54% of expression variation can be explained by the two components PC1 and PC2 (Figure 2). The clear separation of the LL data (100 μmol m^−2^ s^−1^, 10°C) and HL data (400 μmol m^−2^ s^−1^, 10°C) indicated that light is the factor that mainly determines PC1. On the other hand the rather good separation of the LT data (5°C, 400 μmol m^−2^ s^−1^) and the HT data (15°C, 400 μmol m^−2^ s^−1^) indicated that temperature is the factor that mainly determines PC2. This was further supported by the fact that the HL data of the other experiments at 10°C and 400 μmol m^−2^ s^−1^ positioned between LT and HT data. This data reduction shows that in the two experimental setups with different PAR or different temperature typical expression patterns can be received for the respective situation.

### Differences in gene expression in kale leaves at low and moderate levels of photosynthetic active radiation

Since light is a very important environmental factor it does not astonish that the expression of almost 18% of the genes represented on the *Brassica* chip were significantly altered in kale leaves when plants were grown either at 400 μmol m^−2^ s^−1^ or 100 μmol m^−2^ s^−1^. Gene expression was reliably detectable for 89,621 identifier represented on the 105 K format *Brassica* Array. Among these, 16,000 reliably detectable elements showed significant differences in expression between growth at 400 μmol m^−2^ s^−1^ and 100 μmol m^−2^ s^−1^, with 12,666 upregulated at 400 PAR and 3334 upregulated at 100 PAR. The pure numbers of differentially expressed genes in this experiment showed that higher light intensities require almost 4 times more genes to be induced than at low light intensity.

At low light conditions, plants obviously need to absorb sufficient light for photosynthesis, survival, and growth, while at excess light conditions plants have to prevent photo-oxidative damages. Photosynthetic organisms have developed direct and indirect mechanisms for sensing and responding to limiting or excess light (Li et al., 2009; Casal, 2013). For a structured evaluation of differentially expressed genes a functional gene enrichment analysis was performed using the Wilcoxon Rank Sum Test including Benjamini Hochberg correction as integrated in MapMan (Thimm et al., 2004). This analysis revealed that most significant differences in gene expression between plant leaves grown at 400 μmol m^−2^ s^−1^ and 100 μmol m^−2^ s^−1^ were in the functional categories of protein degradation, transport processes, amino acid metabolism, and in secondary metabolism (Table 1). The huge size of the MapMan bin containing members involved in protein degradation indicates the most important role of E3 ubiquitin ligation in controlling plant processes including vegetative growth, light response and stress tolerance (Mazzucotelli et al., 2006). Up to now only a few of these members have been characterized with a known biological function. Amongst them is COP1, the conserved regulator that has established the ubiquitin-proteasome system as a central subject in light signal transduction (Lau and Deng, 2012). Additionally, the significant enrichment of differentially expressed genes belonging to the categories of sugar and amino acid transport and amino acid metabolism was well expected since tissue and phloem concentrations are not static; these metabolite concentrations are modulated by factors such as light and nutritional and developmental stage (Coruzzi et al., 2015).

**Table 1:** **Significantly altered gene expression in functional categories at 100 PAR-400 PAR light intensity difference**.

**MapMan bin**	**Name**	**Elements**	***p*-value**
29	Protein	2098	< 1.00E-20
29.5	Protein.degradation	1063	< 1.00E-20
29.5.11	Protein.degradation.ubiquitin	753	2.35E-12
29.5.11.4	Protein.degradation.ubiquitin.e3	567	1.42E-08
29.5.11.4.3	Protein.degradation.ubiquitin.e3.skp,cullin,f-box containing (scf) complex	227	8.62E-05
29.5.11.4.3.2	Protein.degradation.ubiquitin.E3.SCF.F-box	224	9.94E-05
29.5.11.4.2	Protein.degradation.ubiquitin.E3.RING finger domain protein	299	5.60E-04
29.5.11.20	Protein.degradation.ubiquitin.proteasom	65	4.81E-04
34	Transport	763	< 1.00E-20
34.3	Transport.amino acids	71	4.88E-10
34.2	Transporter.sugars	75	2.34E-05
34.19	Transport.Major Intrinsic Proteins	31	4.83E-04
34.19.1	Transport.Major Intrinsic Proteins.plasma membrane intrinsic proteins (PIP)	23	7.11E-04
34.99	Transport.misc	141	5.18E-04
34.8	Transport.metabolite transporters at the envelope membrane	22	4.25E-03
34.11	Transport.nucleoside diphosphate-sugars at the endoplasmic reticulum	7	3.00E-02
13	Amino acid metabolism	273	4.21E-12
13.1	Amino acid metabolism.synthesis	188	1.08E-06
13.1.1	Amino acid metabolism.synthesis.central amino acid metabolism	38	8.82E-03
13.1.3	Amino acid metabolism.synthesis.aspartate family	45	3.97E-02
13.2	Amino acid metabolism.degradation	82	7.60E-06
13.2.5	Amino acid metabolism.degradation.serine-glycine-cysteine group	15	7.56E-03
13.2.6	Amino acid metabolism.degradation.aromatic aa	24	2.36E-02
13.2.4	Amino acid metabolism.degradation.branched chain group	14	3.00E-02
16	Secondary metabolism	195	1.64E-07
16.2	Secondary metabolism.phenylpropanoids	55	5.27E-03
16.2.1	Secondary metabolism.phenylpropanoids.lignin biosynthesis	36	6.39E-06
16.8	Secondary metabolism.flavonoids	47	3.43E-05
16.8.3	Secondary metabolism.flavonoids.dihydroflavonols	8	2.37E-02
16.8.2	Secondary metabolism.flavonoids.chalcones	13	2.37E-02
33	Development	341	7.80E-07
1	PS	147	1.41E-06
1.3	PS.calvin cyle	46	2.91E-07
1.1.1	PS.lightreaction.photosystem II	35	2.41E-02
11	Lipid metabolism	290	2.98E-04
11.1.3	Lipid metabolism.fatty acid synthesis.ketoacyl acyl-carrier-protein (ACP) synthase	9	9.86E-03
11.9.4.5	Lipid metabolism.lipid degradation.beta-oxidation.acyl-CoA thioesterase	9	2.59E-02
14	S-assimilation	30	4.32E-04
14.2	S-assimilation.adenosine 5′-phosphosulphate reductase (APR)	9	4.95E-02
21	Redox.regulation	148	7.11E-04
20	Stress	360	6.49E-03
20.1	Stress.biotic	139	3.19E-04
35	Not assigned	7060	< 1.00E-20
26	Misc	690	7.20E-08
26.9	Misc.glutathione S transferases	36	2.34E-05
26.2	Misc.UDP glucosyl and glucoronyl transferases	133	3.16E-02

Functional gene enrichment analysis was performed using the Wilcoxon Rank Sum Test including Benjamini Hochberg correction as integrated in MapMan.

Light as an environmental factor with strong effects on particular metabolites expects expression differences of genes belonging to the category of secondary metabolism in plants grown at different light intensities. Only the bins with genes of phenylpropanoid and flavonoid metabolism were significantly enriched within this large group of genes coding for enzymes of various secondary pathways (Table 1). This clearly shows that light intensity is a key regulator of gene expression of flavonoid biosynthesis. This further supports the hypothesis that flavonoids and hydroxycinnamic acid derivatives may act as shielding components and scavengers of ROS, thus protecting plants from damage caused by high levels of sunlight (Edreva, 2005).

### Temperature effects on overall gene expression in kale leaves

Less genes are differentially expressed in kale leaves grown at 5 or 15°C when compared to the light experiment. Among the reliably detectable 89,621 identifier represented on the 105 K format *Brassica* Array 3332 elements or 3.7% showed significant differences in expression between growth at 5 and 15°C, with 1525 upregulated at 15°C and 1807 upregulated at 5°C. The pure numbers of differentially expressed genes in this experiment showed that lower temperature require a few more genes to be induced. Functional gene enrichment analysis revealed that most significant differences in gene expression between plant leaves at 5 and 15°C were in the functional categories of phytohormone metabolism, amino acid synthesis, stress and again secondary metabolism (Table 2). Differentially expressed genes were also found in the functional category RNA.regulation of transcription with specific enrichment of certain classes of transcriptional regulators (Table 2).

**Table 2:** **Significantly altered gene expression in functional categories at 5–15°C temperature difference**.

**MapMan bin**	**Name**	**Elements**	***p*-value**
17	Hormone metabolism	96	149E-05
17.7	Hormone metabolism.jasmonate	12	156E-03
13	Amino acid metabolism	47	1.67E-02
13.1	Amino acid metabolism.synthesis	41	2.98E-02
13.1.6	Amino acid metabolism.synthesis.aromatic aa	16	2.00E-03
20	Stress	126	2.63E-02
16	Secondary metabolism	48	4.19E-02
16.2	Secondary metabolism.phenylpropanoids	27	2.12E-02
27	RNA	373	1.20E-01
27.3.11	RNA.regulation of transcription.C2H2 zinc finger family	19	1.90E-03
27.3.64	RNA.regulation of transcription.PHOR1	11	1.10E-02
27.3.12	RNA.regulation of transcription.C3H zinc finger family	13	5.19E-02
27.3.6	RNA.regulation of transcription.bHLH,Basic Helix-Loop-Helix family	24	9.70E-03
26	Misc	234	2.48E-03
35	Not assigned	1533	3.45E-02

Functional gene enrichment analysis was performed using the Wilcoxon Rank Sum Test including Benjamini Hochberg correction as integrated in MapMan.

Plants from temperate regions show a variable degree of chilling tolerance and can increase their freezing tolerance during exposure to chilling and non-freezing temperatures. This process is known as cold acclimation and involves a complex and interactive relationship between different pathways (Miura and Furumoto, 2013). The significant enrichment of differentially expressed genes belonging to the functional category of jasmonate hormone metabolism supports recent findings that plant hormones are also involved in responses to cold stress (Shi et al., 2015). Additionally, the significant alterations in the category of amino acid synthesis especially aromatic amino acid synthesis point to alterations in phenolic compound production, a prerequisite of the increased lignification and suberin deposition at cold temperatures (Ramakrishna and Ravishankar, 2011). This is further supported by significant enrichment of altered gene expression between plant leaves at 5 and 15°C in the functional category of phenylpropanoid secondary metabolism. Differentially expressed genes were also found in the functional category RNA.regulation of transcription with specific enrichment of transcriptional regulators of the C2H2 and the C3H zink finger families, which are mainly induced at 5°C and bHLH basic helix-loop-helix family members, which are mainly induced at 15°C.

### Content of flavonoid glycosides and hydroxycinnamic acid derivatives in kale leaves specifically influenced by light or temperature

Kale as a leafy *Brassica* vegetable contains a high number of structurally different flavonol glycosides and hydroxycinnamic acid derivatives. It is therefore a suitable model plant for investigating the structure-specific formation of these phenolic compounds with respect to temperature and PAR, especially for acylated flavonol glycosides. In the present study defined conditions in climate chambers can clarify the specific effect PAR or temperature can have on the formation of structurally different flavonol glycosides (17 compounds) and hydroxycinnamic acid derivatives (3 compounds) (Table 3).

**Table 3:** **Influence of light or temperature on the content of flavonoid glycosides and hydroxycinnamic acid derivatives in kale plants**.

	**Light**		**Temperature**	
	**LL**	**HL**	**LT**	**HT**
**Quercetin glycosides as quercetin-3-*****O*****-glucoside equivalents**				
Quercetin-3-*O*-sophoroside-7-*O*-D-glucoside	0.11	0.20	0.25	0.11
Quercetin-3-*O*-feruloyl-sophoroside-7-*O*-D-glucoside	0.09	0.12	0.18	0.15
Quercetin-3-*O*-hydoxyferuloyl-sophoroside-7-*O*-D-glucoside	0.14	0.10	0.17	0.13
Quercetin-3-*O*-sinapoyl-sophoroside-7-*O*-D-glucoside / quercetin-3-*O*-sophoroside-7-*O*-sinapoyl-diglucoside	0.72	2.39	1.19	1.05
Quercetin-3-*O*-disinapoyl-triglucoside-7-*O*-D-glucoside	0.09	0.07	0.20	0.08
**Kaempferol glycosides as kaempferol-3-*****O*****-glucoside equivalents**				
Kaempferol-feruloyl-3-*O*-sophoroside	0.07	0.05	0.07	0.04
Kaempferol-hydroxyferuloyl-3-*O*-sophoroside	0.03	0.00	0.01	0.02
Kaempferol-sinapoyl-3-*O*-sophoroside	0.08	0.03	0.05	0.07
Kaempferol-3-*O*-sophoroside-7-*O*-D-glucoside	0.38	0.20	0.45	0.36
Kaempferol-3-*O*-caffeoyl-sophoroside-7-*O*-D-glucoside	0.85	2.72	1.05	1.02
Kaempferol-3-*O*-feruloyl-sophoroside-7-*O*-D-glucoside	0.60	0.35	0.60	0.68
Kaempferol-3-*O*-hydoxyferuloyl-sophoroside-7-*O*-D-glucoside	0.75	0.45	0.68	0.84
Kaempferol-3-*O*-sinaopyl-sophoroside-7-*O*-D-glucoside	1.87	2.07	1.64	2.21
Kaempferol-3-*O*-hydoxyferuloyl-sophoroside-7-*O*-diglucoside	0.64	0.40	0.72	0.37
Kaempferol-3-*O*-sinapoyl-sophoroside-7-*O*-diglucoside	2.28	1.80	2.34	1.41
Kaempferol-3-*O*-disinapoyl-triglucoside-7-*O*-D-glucoside	0.13	0.07	0.17	0.07
**Caffeoylquinic acid and hydroxycinnamic acid glycosides as caffeoylquinic acid equivalents**				
Caffeoylquinic acid	1.05	0.70	1.27	1.08
Disinapoyl-gentiobiose	1.09	2.18	0.78	1.65
Sinapoyl-feruloyl-gentiobiose	1.00	0.93	1.13	1.12

The concentration of glycosides was quantitated either as quercetin-3-O-glucoside equivalents, kaemferol-3-O-glucoside equivalents or as caffeoylquinic acid equivalents respectively. Values are given as mg × g^−1^ of dry weight. Significant changes with temperature or light (Tukey's HSD test (n = 3) at p ≤ 0.05) are visualized with HL induced in yellow, LL induced in gray, HT induced in red, and LT induced in blue. LL, low light 100 μmol m^−2^ s^−1^; HL, high light 400 μmol m^−2^ s^−1^; LT, low temperature 5°C; HT, high temperature 15°C.

Higher sunlight due to an 8-fold higher reflection caused by a reflective mulch resulted in higher concentrations of flavonoids and anthocyanins in apple fruit (Overbeck et al., 2013). Li and co-worker reported higher concentrations of quercetin glycosides independent of the glycosylated sugar moiety in sun-exposed peel of apple fruits (Li et al., 2013). In kale we found structure-dependent responses of flavonoid glycosides between low and moderate light intensities (Table 3). The main quercetin glycosides quercetin-3-*O*-sinapoyl-sophoroside-7-*O*-D-glucoside/quercetin-3-*O*-sophoroside-7-*O*-sinapoyl-diglucoside and quercetin-3-*O*-sophoroside-7-*O*-D-glucoside were increased with increasing PAR while the main kaempferol glycosides (kaempferol-3-*O*-sophoroside-7-*O*-D-glucoside, kaempferol-3-*O*-feruloyl-sophoroside-7-*O*-D-glucoside, kaempferol-3-*O*-hydroxyferuloyl-sophoroside-7-*O*-D-glucoside, kaempferol-3-*O*-sinapoyl-sophoroside-7-*O*-D-glucoside, kaempferol-3-*O*-hydroxyferuloyl-sophoroside-7-*O*-diglucose, and kaempferol-3-*O*-sinapoyl-sophoroside-7-*O*-diglucoside) were decreased or not affected. The concentrations of other investigated minor flavonol glycosides were also decreased or unchanged at higher PAR levels. It is known that quercetin glycosides have a higher antioxidant activity than their corresponding kaempferol glycosides (Zietz et al., 2010) and are better scavengers of ROS (Edreva, 2005). Out of these feruloyl monoacylated quercetin triglycoside and sinapic acid diacylated quercetin tetraglycoside showed high anti-oxidant activity with 2.54 and 4.19, respectively (Zietz et al., 2010). This implies that even low dosages of PAR result in ROS production and consequences a shift to B-ring polyhydroxylated flavonoids (Agati and Tattini, 2010; Majer et al., 2014). In conclusion, this underlines that ROS concentration presumably at low levels in the present experiment is not damaging the plant's cells. In addition, kaempferol-3-*O*-caffeoyl-sophoroside-7-*O*-D-glucoside was increased with higher PAR level whereas caffeoylquinic acid was decreased. This reflects previous investigations where increased UV-B (Neugart et al., 2012b, 2014) lead to the same results. Flavonoids are well-known antioxidants in plants but also act as shielding components (Edreva, 2005). We assume that kaempferol-3-*O*-caffeoyl-sophoroside-7-*O*-D-glucoside has a higher shielding activity in the plant than caffeoylquinic acid due to the bigger π-electron system. In contrast, disinapoyl-gentiobiose, which has a high antioxidative activity (4.51), increased at 400 μmol m^−2^ s^−1^ while sinapoyl-feruloyl-gentiobiose remained unaffected. In summary, the higher light intensity resulted in structure-dependent responses favoring the main quercetin glycoside quercetin-3-*O*-sinapoyl-sophoroside-7-*O*-D-glucoside/quercetin-3-*O*-sophoroside-7-*O*-sinapoyl-diglucoside, the caffeic acid monoacylated kaempferol triglycoside kaempferol-3-*O*-caffeoyl-sophoroside-7-*O*-D-glucoside and the disinapoyl-gentiobiose.

Lower temperatures resulted in higher concentrations of quercetin glycosides in the investigated kale. Especially the minor compounds quercetin-3-*O*-hydroxyferuloyl-sophoroside-7-*O*-D-glucoside and quercetin-3-*O*-disnapoyl-triglucoside-7-*O*-D-glucoside that have a high antioxidant activity (2.54 and 4.19, respectively) (Zietz et al., 2010) were increased assuming a higher level of ROS in kale plants at 5°C compared to 15°C. This reflects the shift to B-ring polyhydroxylated glycosides in different plant species due to higher ROS levels in plants caused by lower temperatures (Bilger et al., 2007; Klimov et al., 2008; Moheb et al., 2011; Lavola et al., 2013). Lower temperatures were shown to increase the flavonoids and free phenolic acids in cucumber leaves grown at day/night temperatures of 15/10°C under defined conditions (Chen et al., 2013). Concomitantly, the activity of polyphenol oxidase and different peroxidases was increased at lower day/night temperatures (Chen et al., 2013). However, in kale, different specific flavonol glycosides were increased at 5°C suggesting different ROS to be produced or different plant defense mechanisms to interact with the flavonol glycosides and respective enzymes (catalase, polyphenol oxidase, peroxidase and superoxid dismutase) and vitamins (vitamin C and vitamin E). Additionally, Steindal and co-workers reported higher concentrations of quercetin and kaempferol in broccoli grown at lower day/night temperatures of 15/9°C under defied conditions (Steindal et al., 2013). Interestingly, in kale low temperature of 5°C resulted in higher concentrations of kaempferol tetraglycosides (kaempferol-3-*O*-hydroxyferuloyl-sophoroside-7-*O*-diglucoside, kaempferol-3-*O*-sinapoyl-sophoroside-7-*O*-diglucoside, kaempferol-3-*O*-disinapoyl-trigluco-side-7-*O*-D-glucoside) independent of the acylation pattern. In general, glycosylation causes better water solubility and higher concentrations of sugars act as cryo-protectans in plants at lower temperatures (Theocharis et al., 2012). These kaempferol tetraglycosides can act as antioxidants comparably to their corresponding kaempferol triglycosides that are main flavonol glycosides in kale. The main hydroxycinnamic acid derivatives of kale were not affected or decreased (disinapoyl-gentiobiose) with decreasing temperatures. This was also shown for caffeoylquinic acid in box tree that was not affected by higher altitude including lower temperatures and higher UV-B radiation (Bernal et al., 2013). Even though disinapoyl-gentiobioside which has a high antioxidant potential (4.51) was decreased at lower temperature the total antioxidant activity in the kale plants was increased at lower temperatures (Zietz et al., 2010). Nevertheless, Dong and co-worker found for *Eucommia ulmoides* a higher correlation of flavonoids and chlorogenic acids with the annual average temperature than with the annual average sunshine duration (Dong et al., 2011). To summarize, in response to low temperatures highly complex sinapic acid acylated flavonol glycosides are favored (quercetin-3-*O*-disinapoyl-triglucoside-7-*O*-D-glucoside, kaempferol-3-*O*-sinapoyl-sophoroside-7-*O*-diglucoside, kaempferol-3-*O*-disinapoyl-triglucoside-7-*O*-D-glucoside) whereas disinapoyl-gentiobiose was decreased at the same time assuming them to be better cryo-protectants.

### Identification of the *B. oleracea* genes putatively involved in the biosynthesis of flavonoids and hydroxycinnamic acids

Thanks to the identification of genes involved in the phenylpropanoid biosynthetic pathway in several plant species (Milkowski and Strack, 2010; Vogt, 2010; Fraser and Chapple, 2011) the *B. oleracea* genome could be screened for respective homologs. All relevant genes (Figure 1) were selected by sequence comparison of predicted genes from the *B. oleracea* genome using the respective coding sequences of *A. thaliana* and the genome annotation system in *e!EnsemblGenomes* (Kersey et al., 2014). Genes coding for flavone synthase (*FNSII*) of *Glycine max* and flavonoid 3′5′-hydroxylase (*F3*′*5*′*H*) of *Vitis vinifera* were also included, however, putative homologs to *FNSII* and *F3*′*5*′*H* were not found in the *B. oleracea* genome. Predicted *B. oleracea* genes putatively involved in the biosynthesis of flavonol aglycons (gray and blue pathways in Figure 1) are presented in Table 4, and genes putatively involved in the biosynthesis of phenylpropanoids (orange pathway in Figure 1) are shown in Table 5. Because during evolution the *Brassica* genomes experienced whole genome triplication followed by massive gene loss and frequent reshuffling of triplicated genomic blocks (Liu et al., 2014), we expected to find in most cases more than one gene homolog in *B. oleracea* when compared to *A. thaliana*. In case of flavonoid 3′-hydroxylase *F3*′*H* and sinapaldehyde dehydrogenase *REF1* only single genes could be found in the *B. oleracea genome*, while for cinnamate 4-hydroxylase *C4H* and isoform 5 of 4-coumarate-CoA ligase *4CL5* five homologous genes are present in the *B. oleracea genome*.

**Table 4:** **Expression difference of genes putatively involved in the biosynthesis of flavonol aglycons in *Brassica oleracea***.

**Enzyme step**	***Brassica oleracea* predicted genes**	**Gene abbreviation**	**Microarray element**	**Light Log2-difference LL-HL**	**Temperature Log2-difference LT-HT**
Phenylalanine ammonia lyase EC 4.3.1.24	*Bo4g030910*	*PAL1*	EV152862	−3.43	−0.04
	*Bo4g186590*	*PAL1*	JCVI_13216	−3.63	0.09
	*Bo6g067250*	*PAL2*	JCVI_32380	−0.79	1.61
	*Bo8g082620*	*PAL2*	JCVI_32380	−0.79	1.61
Cinnamate 4-hydroxylase EC 1.14.13.11	*Bo3g024650*	*C4H*	JCVI_1296	−3.34	−2.07
	*Bo3g024670*	*C4H*	JCVI_1296	−3.34	−2.07
	*Bo4g173070*	*C4H*	AM386090	−1.96	−1.59
	*Bo4g173080*	*C4H*	JCVI_1296	−3.34	−2.07
	*Bo5g052100*	*C4H*	EX137858	−2.50	−0.13
4-coumarate-CoA ligase EC 6.2.1.12	*Bo6g042520*	*4CL1*	JCVI_1086	0.20	−1.74
	*Bo5g102340*	*4CL2*	JCVI_32600	0.10	0.21
	*Bo5g102350*	*4CL2*	JCVI_32600	0.10	0.21
	*Bo6g099190*	*4CL3*	JCVI_26641	−2.68	1.03
	*Bo3g077430*	*4CL5*	JCVI_38583	−1.55	−1.30
	*Bo3g077450*	*4CL5*	EX043451	−2.32	−2.53
	*Bo5g103380*	*4CL5*	n		
	*Bo5g103390*	*4CL5*	n		
	*Bo5g103450*	*4CL5*	JCVI_32600	0.10	0.21
Chalcone synthase EC 2.3.1.74	*Bo9g166290*	*CHS*	JCVI_6210	−3.47	0.72
	*Bo9g004350*	*CHS*	JCVI_2058	−3.94	0.39
	*Bo3g009440*	*CHS*	JCVI_24111	−4.20	1.07
Chalcone isomerase EC 5.5.1.6	*Bo8g088480*	*CHI*	JCVI_31142	−1.58	−0.11
	*Bo6g068550*	*CHI*	JCVI_20189	−2.45	0.85
	*Bo7g117570*	*CHI*	CD834583	−3.27	−0.05
	*Bo8g089480*	*CHI*	n		
	Bo9g177250	*CHI-like*	JCVI_2577	−0.93	−0.19
Flavanone 3-hydroxylase EC 1.14.11.9	*Bo8g081770*	*F3H*	JCVI_17031	−3.33	0.26
	*Bo7g100840*	*F3H*	JCVI_17031	−3.33	0.26
	*Bo8g099330*	*F3H*	JCVI_17031	−3.33	0.26
	*Bo4g120170*	*F3H*	JCVI_17031	−3.33	0.26
Flavonol synthase EC 1.14.11.23	*Bo9g174290*	*FLS1*	JCVI_2934	−1.25	1.12
	*Bo3g103270*	*FLS2*	JCVI_41972	−2.62	−0.26
	*Bo3g103260*	*FLS3*	JCVI_2239	−2.67	−0.36
	*Bo2g165770*	*FLS3*	CV432242	−3.07	−0.31
	*Bo9g017780*	*FLS3*	n		
Flavonoid 3′-hydroxylase EC 1.14.13.88	*Bo9g174880*	*F3′H*	JCVI_15282	−4.74	1.51

Enzyme steps, predicted genes and respective microarray elements with highest homology are listed. Less specific but highly homologous elements are listed repeatedly. Log2-fold changes are calculated as 100 PAR-400 PAR light intensity difference (LL-HL), or 5–15°C temperature difference (LT-HT). Significant changes are visualized, HL induced in yellow, HT induced in red, and LT induced in blue. Color intensity indicates respective expression differences. n, no microarray element with homology to the respective gene is present on the chip.

**Table 5:** **Expression difference of genes putatively involved in the phenylpropanoid biosynthesis in *Brassica oleracea***.

**Enzyme step**	***Brassica oleracea* predicted genes**	**Gene abbreviation**	**Microarray element**	**Light Log2-difference LL-HL**	**Temperature Log2-difference LT-HT**
Hydroxycinnamoyl-transferase EC 2.3.1.133	*Bo1g055210*	*HCT*	JCVI_34867	−3.42	−2.07
	*Bo1g055210*	*HCT*	EX042932	−3.10	−1.88
	*Bo7g003460*	*HCT*	JCVI_24862	−1.09	−1.44
Coumarate 3′-hydroxylase EC 1.14.-.-	*Bo3g035250*	*C3H*	n		
	*Bo4g012820*	*C3H*	JCVI_9357	−0.41	−1.04
	*Bo4g190570*	*C3H*	JCVI_9357	−0.41	−1.04
Caffeoyl-CoA *O*-methyltransferase EC 2.1.1.104	*Bo3g169210*	*CCoAOMT1*	JCVI_1016	−3.67	−1.40
	*Bo7g116590*	*CCoAOMT1*	JCVI_1016	−3.67	−1.40
	*Bo1g043050*	*CCoAOMT7*	n		
	*Bo1g043060*	*CCoAOMT7*	JCVI_31825	0.01	1.32
	*Bo3g176030*	*CCoAOMT7*	JCVI_11293	0.88	1.18
Cinnamoyl-CoA reductase EC 1.2.1.44	*Bo8g105700*	*CCR1*	JCVI_814	−1.49	−0.34
	*Bo8g105700*	*CCR1*	EE559396	−2.65	−0.17
	*no gene present*	*CCR2*	n		
Ferulate 5-hydroxylase EC 1.14.-.-	*Bo1g005770*	*FAH1*	JCVI_13253	−1.85	−0.73
	*Bo3g093960*	*FAH1*	n		
	*Bo7g117840*	*FAH1*	JCVI_17274	−1.15	−0.49
	*Bo7g119430*	*FAH1*	JCVI_26392	−0.02	−0.12
Flavone 3′-*O*-methyltransferase EC 2.1.1.-	*Bo2g041880*	*COMT*	JCVI_4160	−3.60	0.13
	*Bo2g041890*	*COMT*	n		
	*Bo3g022270*	*COMT*	JCVI_4160	−3.60	0.13
	*Bo9g115100*	*COMT*	JCVI_4160	−3.60	0.13
Sinapaldehyde dehydrogenase EC 1.2.1.3	*Bo3g083290*	*REF1*	JCVI_1718	−0.85	−0.86
Sinapate 1-glucosyltransferase EC 2.4.1.120	*Bo1g105850*	*UGT84A2*	JCVI_9170	−2.38	1.43
	*Bo5g100950*	*UGT84A2*	n		
Sinapoylglucose-acyltransferase EC 2.3.1.-	*Bo3g042330*	*SCPL8-10*	n		
	*Bo8g100760*	*SCPL8-10*	EE503308	−1.16	1.14

Enzyme steps, predicted genes and respective microarray elements with highest homology are listed. Less specific but highly homologous elements are listed repeatedly. Multiple listings of B. oleracea genes with more than one specific element are written in gray. Log2-fold changes are calculated as 100 PAR-400 PAR light intensity difference (LL-HL), or 5–15°C temperature difference (LT-HT). Significant changes are visualized, HL induced in yellow, HT induced in red, and LT induced in blue. Color intensity indicates respective expression differences. n, no microarray element with homology to the respective gene is present on the chip.

The *Brassica* community microarray resource was built with the 95K *Brassica* unigene set assembled with sequences originating from *B. napus, B. rapa*, and *B. oleracea* (Trick et al., 2009). Therefore, all identified *B. oleracea* genes putatively involved in flavonoid biosynthesis were analyzed for their most specific 60-mer oligonucleotides present on the 2 × 105 K format *Brassica* Array. In some cases, e.g. for the three *CHS* genes, gene specific microarray elements could be obtained for all individual isogenes (Table 4). While in other cases, only a single microarray element was present that is highly identical to all selected homologs thus hampering differentiation, e.g. between the four different genes of *F3H* (Table 5). In addition, there are individual genes in *B. oleracea* where there is no highly homologous, specific 60-mer oligonucleotide present on this most comprehensive 105 K *Brassica* Array.

### Specific expression changes of genes putatively involved in the biosynthesis of flavonoids induced by light or temperature

As already shown, different light intensities lead to significant enrichment of differentially expressed genes of secondary metabolism (Table 1), especially predicted genes putatively involved in the biosynthesis of flavonoids and phenylpropanoids. As evident from Table 4 most of the predicted genes putatively involved in the core biosynthesis of flavonol aglycons in kale were strongly increased with increasing light. It is known from various studies that light is one of the most important environmental factors affecting flavonoid biosynthesis, involving R2R3 MYB transcription factors and a COP1 mediated signaling pathways (Zoratti et al., 2014). In fact, a closer analysis of array elements with high homology to MYB transcription factors revealed light-increased expression of CX267652 (log2-difference LL-HL -2.30), specific for the predicted two *B. oleracea MYB1* genes, *Bo1g144680* and *Bo7g117880*. This underpins a coordinated light-increased expression of at least one gene coding for each step of flavonoid biosynthesis in kale, resulting in accumulation of higher levels of flavonoids (Table 3). Moreover, the higher light intensity caused a structure-dependent response favoring the main quercetin glycosides quercetin-3-*O*-sinapoyl-sophoroside-7-*O*-D-glucoside/ quercetin-3-*O*-sophoroside-7-*O*-sinapoyl-diglucoside. This preferential formation of quercetin as the flavonoid aglycone at higher light intensity is further supported by the very strong induction of *Bo9g174880*, coding for the flavonoid 3′-hydroxylase. This gene is among the most significantly, differentially expressed genes in this analysis. It further underlines the light dependent increased synthesis of shielding components and scavengers protecting plants from damage caused by high levels of sunlight (Edreva, 2005).

As shown previously, different temperature lead to significant enrichment of differentially expressed genes of secondary metabolism (Table 2), especially predicted genes putatively involved in the biosynthesis of phenylpropanoids. As evident from Table 4, significant temperature specific responses were only found in predicted genes putatively involved in the first three steps of core biosynthesis, phenylalanine ammonia lyase (*PAL*), cinnamate 4-hydroxylase (*C4H*) and 4-coumarate-CoA ligase (*4CL*) (Table 4). These first three steps are in common in flavonoid and phenylpropanoid biosynthesis. Thus, temperature effects on gene expression are also exerted on the level of genes involved in hydroxycinnamic acid synthesis, what is discussed in more detail in the next chapter.

### Specific expression changes of genes putatively involved in the biosynthesis of hydroxycinnamic acids induced by light or temperature

At higher light intensities, the main quercetin glycosides and kaempferol-3-*O*-caffeoyl-sophoroside-7-*O*-D-glucoside were significantly accumulating (Table 3), together with disinapoyl-gentiobiose, which has a high antioxidant potential (4.51). This elevated formation of hydroxycinnamic acid derivatives is accompanied by an increased expression of genes presumably involved in the synthesis of hydroxycinnamic acid derivatives at higher PAR levels (Table 5). Up to now, only a few genes involved in these biosynthetic pathways are known from research in *Arabidopsis* (Fraser and Chapple, 2011). For example, genes directly involved in disinapoyl-gentiobiose biosynthesis, still needs to be discovered, as well as isoforms catalyzing the acylation of flavonoids. Gene identification becomes so difficult, because the model organism *Arabidopsis* is lacking disinapoyl-gentiobiose, and also acylation of flavonols (Saito et al., 2013). In addition, candidate genes involved in the biosynthetic steps all belong to huge gene families that need further detailed exploration (Sasaki et al., 2014).

As shown previously, different temperature lead to significant enrichment of differentially expressed genes putatively involved in the biosynthesis of phenylpropanoids (Table 2). As evident from Tables 4, 5, significant temperature specific responses were found in predicted genes putatively involved in the first three steps of biosynthesis (*PAL, C4H*, and *4CL*) (Table 4), and hydroxycinnamoyl-transferase (*HCT*) (Table 5).

Within the identified genes there is evidence of an isoform specific response to different stimuli: while both phenylalanine ammonia lyase1 genes (*PAL1, Bo4g03091, Bo4g186590*) are strongly induced with higher light and unaffected by temperature changes, one or both of the *PAL2* genes (*Bo6g067250, Bo8g082620*) were unaffected by light and stronger expressed at low temperature. A functional specialization has been shown previously for *PAL1* and *PAL2* in *Arabidopsis* for an abiotic environmental-triggered flavonoid biosynthesis (Olsen et al., 2008, 2009b). The isoform specific expression changes to different stimuli proved functional specialization of *PAL* also in kale. Furthermore, a specific metabolic function of individual isoforms has recently also been discovered for 4-coumarate-CoA ligase *4CL* (Li et al., 2015). The four isoforms of *4CL* in *Arabidopsis* have overlapping yet distinct roles in phenylpropanoid metabolism, with *4CL1* and *4CL2* predominant in lignifying cells (Ehlting et al., 1999; Saito et al., 2013), and *4CL3* with a distinct role in flavonoid metabolism (Li et al., 2015). Comparable roles might also be expected in kale, as *4CL1* and *4CL2* isoform expression is unaffected by different light and temperature, while expression of the single *4CL3* isoform is significantly upregulated with high light, thus co-expressing with the other genes of core biosynthesis of flavonol aglycons. Two isoforms of *4CL5* were significantly increased at high temperature and light. It might be speculated that this is related to increased levels of disinapoyl-gentiobiose at high temperature and light. It was demonstrated previously in *Arabidopsis*, that a specific *4CL* gene product is able to activate sinapic acid (Hamberger and Hahlbrock, 2004). Together with specific isoforms of cinnamate 4-hydroxylase *C4H* and hydroxycinnamoyl-transferase *HCT* co-expression of the hydroxycinnamic acid pathway at high temperature and high light was detectable.

### Expression pattern of gene family members putatively involved in flavonoid modification

In kale flavonoid glycosylation occurs preferentially at the C-3 and the C-7 position of the aglycons, with only glucose residues being detected as conjugates (Table 3). So far no arabinose or rhamnose was found to be conjugated with flavonols in kale leaves. In addition, diversity is increased by a variety of acylations (Schmidt et al., 2010b), a modification not present in *A. thaliana*. Another differentiation to *Arabidopsis* is the accumulation of other hydroxycinnamic acid derivatives: while disinapoyl-gentiobiose and sinapoyl-feruloyl-gentiobiose are highly abundant in kale, the major hydroxycinnamic acid derivative in *Arabidopsis*, the sinapoylmalate is absent in kale.

Huge gene families of glycosyltransferases (Bowles et al., 2005) and acyltransferases (Sasaki et al., 2014) are involved in the synthesis of chemically diverse flavonoids and hydroxycinnamic acid derivatives. Dependent on their subcellular location within the plant cell glycosylation is either performed by UDP-sugar dependent glycosyltransferases (UGT) in the cytosol, or by acyl-glucose dependent glucosyltransferases in the vacuole (glycoside hydrolase family 1 type glucosyltransferases, GH1-GT). Also dependent on localization acylation is either performed by acyl-CoA dependent acyltransferases in the cytosol (BAHD type family of acyltransferases, BAHD-AT), or by acyl-glucose dependent acyltransferases (serine carboxypeptidase-like family of acyltransferases, SCPL-AT) in the vacuole. In the *Arabidopsis* genome 116 UGT genes are present while the *B. oleracea* genome harbors at least 189 genes coding for predicted UDP-sugar glycosyltransferases. The gene family of vacuolar glycoside hydrolase family 1 type glucosyltransferases is a little smaller with 48 members in *Arabidopsis* and 75 GH1-GT present in the *B. oleracea* genome. While for the BAHD type family of acyltransferases in *Arabidopsis* 64 genes are known, at least 99 genes encoding putative BAHD-AT can be extracted from the *B. oleracea* genome. Finally the 54 genes coding for serine carboxypeptidase-like acyltransferases in *Arabidopsis* are exceeded by the 64 SCPL-AT present in the *B. oleracea* genome. Up to now a couple of these glycosyltransferases and acyltransferases have been determined with their functional preferences, and very few of these can be linked to flavonoid and anthocyanin modifications or hydroxycinnamic acid derivative biosynthesis (Fraser and Chapple, 2011; Saito et al., 2013).

Since these gene families are so large and most of their members still lacking functional characterization a differentiation or distinct assignment to certain pathways or enzymatic steps will not be possible at that point. However, extraction of some isoforms with correlated expression patterns might be possible and give hints to some promising candidates. For example, candidates of the UGT73B and UGT73C family with predicted functions as flavonol 3-*O*-glucosyltransferase and flavonol 7-*O*-glucosyltransferase (Table 6) are among the most induced UGT family members at high light intensities. Furthermore, the two microarray elements EV108083 and JCVI_27911 are among the top 100 most cold responsive elements. These microarray elements correspond two adjacent genes on chromosome 4 predicted as UGT family members of UGT73C5/6. Whether one or both of these genes are responsible for the enhanced accumulation of kaempferol tetraglycosides catalyzing the transfer of the second glucose residue to the flavonol 7-*O* position in kale at low temperature will be the focus of further studies.

**Table 6:** **Expression difference of genes putatively involved in glucosylation and acylation of flavonoids in *Brassica oleracea***.

**Enzyme step**	***Brassica oleracea* predicted genes**	**Gene abbreviation**	**Microarray element**	**Light Log2-difference LL-HL**	**Temperature Log2-difference LT-HT**
Flavonol 3-*O*-GT Flavonol 7-*O*-GT UGT73B	*Bo1g007620*	*UGT73B2/3*	JCVI_35049	−2.33	−0.49
	*Bo1g007630*	*UGT73B2/3*	n		
	*Bo3g169080*	*UGT73B2/3*	JCVI_28429	−2.53	0.59
	*Bo3g169090*	*UGT73B2/3*	JCVI_3139	−2.00	−1.77
Quercetin 3-*O*-GT Flavonol 7-*O*-GT UGT76E	*Bo2g026640*	*UGT76E1*	ES942112	0.49	0.4346
	*Bo9g139510*	*UGT76E2*	JCVI_42206	−0.38	0.4533
	*Bo3g132780*	*UGT76E11/12*	JCVI_33139	1.01	0.6658
	*Bo1g070870*	*UGT76E11/12*	CA992143	0.28	0.9358
Flavonol 3-*O*-GT UGT78D	*no gene present*	*UGT78D2*	n		
	*Bo3g003590*	*UGT78Drelated*	JCVI_6551	0.40	−1.45
Flavonol 7-*O*-GT UGT73C	*Bo3g030310*	*UGT73C5/6*	EV108354	−3.16	2.93
	*Bo4g031140*	*UGT73C5/6*	EV108083	−2.03	4.31
	*Bo4g031150*	*UGT73C5/6*	JCVI_27911	−2.67	4.58
*Sinapate 1-GT*	*Bo1g105850*	*UGT84A2*	JCVI_9170	−2.38	1.43
	*Bo5g100950*	*UGT84A2*	n		
	*Bo1g056800*	*UGT84A1*	n		
	*Bo7g103790*	*UGT84A1*	JCVI_13790	1.26	0.23
	*Bo1g056770*	*UGT84A3*	JCVI_15448	−2.76	0.32
	*Bo1g056740/50/60*	*UGT84A4*	n/n/n		
Acyl-glucose dependent glucosyltransferase (BGLU)	*Bo3g018610*	*BGLU1-6*	EV102326	−1.21	1.53
	*Bo3g018620*	*BGLU1-6*	JCVI_28076	−2.27	1.66
	*Bo3g039750*	*BGLU1-6*	JCVI_25479	−1.07	2.28
	*Bo4g199780*	*BGLU1-6*	JCVI_28076	−2.27	1.66
	*Bo6g089150*	*BGLU1-6*	JCVI_34674	−2.29	−0.0696
	*Bo7g082150*	*BGLU1-6*	EV102326	−1.21	1.53
	*Bo7g082190*	*BGLU1-6*	JCVI_25479	−1.07	2.28
	*Bo4g098540*	*BGLU7-10*	EX063486	−2.14	−0.70
	*Bo5g132800*	*BGLU7-10*	JCVI_11645	−2.51	−0.13
	*Bo8g098070*	*BGLU7-10*	JCVI_17890	−1.53	−1.80
	*Bo8g098080*	*BGLU7-10*	JCVI_34674	−2.29	−0.07
	*Bo5g003420*	*BGLU11*	CD837111	−2.75	−0.40
Sinapoylglucose-AT (SCPL-AT)	*Bo3g042330*	*SCPL8-10*	n		
	*Bo8g100760*	*SCPL8-10*	EE503308	−1.16	1.14
BAHD-AT	*Bo8g002690*	*3AT*	n		
	*Bo9g008920*	*5AT*	n		

Enzyme steps, predicted genes and respective microarray elements with highest homology are listed. Less specific but highly homologous elements are listed repeatedly. Log2-fold changes are calculated as 100 PAR-400 PAR light intensity difference (LL-HL), or 5–15°C temperature difference (LT-HT). Significant changes are visualized, HL induced in yellow, HT induced in red, and LT induced in blue. Color intensity indicates respective expression differences. n, no microarray element with homology to the respective gene is present on the chip.

In general, additional experimental evidence will be needed to unequivocally show the functional involvement of specific genes and gene products in flavonoid related pathways in kale. This can be done only with some degree of success in *in vitro* experiments using recombinant gene products analyzed for their enzyme activity with only limiting substrate availability. An *in vivo* functional analysis will be complicated by the fact that knock-out mutants of kale are not available and gene editing techniques in Brassicaceae are still in its infancies (Lawrenson et al., 2015). Furthermore, related knock-out mutants in *Arabidopsis* are not very useful, because specific flavonoid modifications present in kale are absent in *Arabidopsis.* However, this latter point might also be adventitious, since it allows functional *in vivo* characterization by overexpressing the respective *B. oleracea* gene candidates in transgenic *Arabidopsis* plants. A subsequent analysis of the flavonoids in these plants may result in more kale like flavonoid profiles and may give valuable hints on the enzyme specificities.

## Conclusion

Kale as a typical winter crop is exposed to less favorable growth conditions. Having to deal with the disadvantageous light and/or temperature conditions kale leaves accumulate high concentrations of a large number of naturally occurring flavonol glycosides and hydroxycinnamic acid derivatives. This makes kale an increasingly important vegetable, since its secondary metabolites are considered as health promoting substances. To understand the underlying processes and expression networks, the temperature and light dependent alterations of gene expression were analyzed in *B. oleracea* var. *sabellica* using the most comprehensive *Brassica* microarray. Expression differences were correlated to the structure-dependent response of flavonoid glycosides and hydroxycinnamic acid derivatives to alterations in either temperature or PAR. Altered metabolite accumulation was mainly reflected on gene expression level of core biosynthetic pathway genes and gave hints to an isoform specific functional specialization. However, additional experimental evidence will be needed to unequivocally show the functional involvement of specific genes and gene products in flavonoid modification pathways. On the one hand such an analysis will be complicated by the fact that knock-out mutants are not available and some flavonoid modifications are specific in kale and absent in *Arabidopsis*. On the other hand this will be adventitious since it allows functional *in vivo* characterization by overexpressing the respective *B. oleracea* gene candidates in transgenic *Arabidopsis* plants with subsequent flavonoid profiling.

## Author contributions

SN and AK designed the study, carried out the metabolite analysis, helped in data interpretation, and made a draft of parts of the manuscript. RZ carried out the expression analysis, data interpretation and wrote the manuscript. All authors have given final approval for this version to be published.

## Funding

We sincerely thank the DFG (Deutsche Forschungsgemeinschaft) for funding (Projects KR-2066/3-2). The Leibniz Association is gratefully acknowledged for supporting this work by an additional grant.

### Conflict of interest statement

The authors declare that the research was conducted in the absence of any commercial or financial relationships that could be construed as a potential conflict of interest.
