# Supplementary material for: Influence of Light and Temperature on Gene Expression Leading to Accumulation of Specific Flavonol Glycosides and Hydroxycinnamic Acid Derivatives in Kale (Brassica oleracea var. sabellica)
Source: Front Plant Sci. 2016 Mar 30;7:326. doi: 10.3389/fpls.2016.00326 (PMC4812050; doi:10.3389/fpls.2016.00326)
Supplement: Supplementary file 1 [file Table1.PDF]

Identifier	log2-fold LL induced	Description	log2-fold LT-HT
JCVI_4003	10.823	no original description	
JCVI_27160	7.157	no original description	
JCVI_30920	6.017	very weakly similar to (97.4)AT4G22460| Symbols: | protease inhibitor/seed storage/lipid transfer protein (LTP) family protein | chr4:11	
JCVI_37606	5.844	no original description	
EE568426	5.474	no similarity	
AM385398	5.312	no similarity	
DY002991	5.299	no similarity	4.410
CD843990	5.281	no similarity	5.208
JCVI_18611	5.192	moderately similar to (381)AT1G04780| Symbols: | ankyrin repeat family protein | chr1:1340890-1342964 REVERSE no original desc	
JCVI_28808	5.100	weakly similar to (106)AT3G19030| Symbols: | similar to unknown protein [Arabidopsis thaliana] (TAIR:AT1G49500.1) | chr3:65641	
JCVI_41372	5.049	no original description	
JCVI_26301	5.015	moderately similar to (240)AT1G19210| Symbols: | AP2 domain-containing transcription factor, putative | chr1:6626964-6627521 RE	
RC_EE564899	5.013	no similarity	
JCVI_33125	5.013	moderately similar to (229)AT4G32900| Symbols: | similar to unknown protein [Arabidopsis thaliana] (TAIR:AT3G03010.1); similar t	4.453
ES906294	4.970	moderately similar to (293)AT1G62660| Symbols: | beta-fructosidase (BFRUCT3) / beta-fructofuranosidase / invertase, vacuolar | chr1	3.171
EV161824	4.953	weakly similar to (122)AT4G17510| Symbols: UCH3 | UCH3; ubiquitin thiolesterase | chr4:9767127-9768661 REVERSE [21484]	
EV176011	4.905	no similarity	
DT317662	4.891	very weakly similar to (80.1)AT3G19030| Symbols: | similar to unknown protein [Arabidopsis thaliana] (TAIR:AT1G49500.1) | chr3:6	
EV224869	4.882	very weakly similar to (96.3)AT1G74930| Symbols: ORA47 | ORA47; DNA binding / transcription factor | chr1:28147900-28148487 F	5.609
H07416	4.808	no similarity	2.138
EE505143	4.796	no similarity	
CX191734	4.737	no similarity	
JCVI_31442	4.646	weakly similar to (144)AT1G59910| Symbols: | formin homology 2 domain-containing protein / FH2 domain-containing protein | chr1:	5.289
ES899985	4.637	no similarity	
JCVI_4881	4.541	very weakly similar to (87.0)AT5G15110| Symbols: | pectate lyase family protein | chr5:4895969-4897685 FORWARD no original des	4.252
EV218294	4.492	moderately similar to (322)AT4G37610| Symbols: BT5 | BT5 (BTB and TAZ domain protein 5); protein binding / transcription regulato	
JCVI_27237	4.471	highly similar to (593)AT5G53570| Symbols: | RabGAP/TBC domain-containing protein | chr5:21778150-21780308 REVERSE no or	
RC_H07284	4.469	no similarity	2.801
AM395641	4.467	very weakly similar to (82.8)AT4G27657| Symbols: | similar to unknown protein [Arabidopsis thaliana] (TAIR:AT4G27652.1) | chr4:1	2.550
JCVI_33577	4.465	no original description	
EX132042	4.465	weakly similar to (172)AT5G17350| Symbols: | similar to unknown protein [Arabidopsis thaliana] (TAIR:AT3G03280.1); similar to un	
EE554963	4.464	weakly similar to (175)AT5G52860| Symbols: | ABC transporter family protein | chr5:21437002-21438771 REVERSE [20184] 1 382	5.546
EV183787	4.422	no similarity	
RC_EE556375	4.415	no similarity	
EE521586	4.415	no similarity	
JCVI_14413	4.415	no original description	
JCVI_33843	4.415	no original description	
CN728417	4.402	no similarity	
RC_H07168	4.393	no similarity	
JCVI_20042	4.368	moderately similar to (349)AT5G45340| Symbols: CYP707A3 | CYP707A3 (cytochrome P450, family 707, subfamily A, polypeptide	
JCVI_29313	4.333	no original description	
EE567997	4.319	no similarity	
EE502320	4.319	weakly similar to (108)AT1G09010| Symbols: | glycoside hydrolase family 2 protein | chr1:2895262-2899290 REVERSEweakly simil	
JCVI_17766	4.310	weakly similar to (178)AT4G38060| Symbols: | similar to unknown protein [Arabidopsis thaliana] (TAIR:AT5G65480.1); similar to un	
JCVI_33410	4.275	moderately similar to (209)AT1G12800| Symbols: | S1 RNA-binding domain-containing protein | chr1:4361776-4365187 REVERSE	
JCVI_13118	4.241	moderately similar to (205)AT1G06900| Symbols: | metalloendopeptidase | chr1:2115154-2120634 REVERSE no original description	6.217
JCVI_2853	4.220	moderately similar to (246)AT4G26420| Symbols: GAMT1 | GAMT1 | chr4:13351210-13352459 REVERSE no original description	1.986
EV152439	4.208	no similarity	
DT317685	4.182	no similarity	
ES907514	4.181	weakly similar to (177)AT2G46750| Symbols: | FAD-binding domain-containing protein | chr2:19215513-19217979 REVERSE [2143	
JCVI_32334	4.172	no original description	
EX123581	4.161	no similarity	
RC_ES965433	4.146	no similarity	
EE559982	4.137	no similarity	
EX088739	4.137	moderately similar to (224)AT5G19190| Symbols: | similar to unknown protein [Arabidopsis thaliana] (TAIR:AT3G06070.1); similar t	
ES967782	4.130	moderately similar to (249)AT5G38700| Symbols: | similar to unknown protein [Arabidopsis thaliana] (TAIR:AT4G02170.1); similar t	4.630
JCVI_13314	4.115	highly similar to (554)AT4G37610| Symbols: BT5 | BT5 (BTB and TAZ domain protein 5); protein binding / transcription regulator | c	
EV193345	4.115	no similarity	
JCVI_4014	4.114	no original description	
JCVI_25215	4.113	no original description	
JCVI_26377	4.107	moderately similar to (350)AT5G60390| Symbols: | elongation factor 1-alpha / EF-1-alpha | chr5:24306452-24307901 FORWARDmo	
JCVI_33491	4.100	highly similar to (629)AT5G25900| Symbols: CYP701A3, GA3 | GA3 (GA REQUIRING 3); oxygen binding | chr5:9036076-9038281	
EV111846	4.096	no similarity	
JCVI_40573	4.096	no original description	
DY021387	4.091	weakly similar to (183)AT2G47220| Symbols: | 3' exoribonuclease family domain 1 protein-related | chr2:19391129-19393542 FORW	
EE474947	4.088	no similarity	
EE421759	4.079	no similarity	
JCVI_20277	4.068	no original description	
JCVI_8342	4.039	moderately similar to (408)AT4G32480| Symbols: | similar to unknown protein [Arabidopsis thaliana] (TAIR:AT2G20670.1); similar t	
EX068239	4.038	weakly similar to (122)AT1G29120| Symbols: | similar to unknown protein [Arabidopsis thaliana] (TAIR:AT4G25770.1); similar to un	
JCVI_3842	4.013	moderately similar to (246)AT1G13880| Symbols: | ELM2 domain-containing protein | chr1:4749600-4750964 FORWARD no origina	
JCVI_8946	4.012	moderately similar to (247)AT4G16265| Symbols: | DNA-directed RNA polymerase II, putative | chr4:9202545-9203991 REVERSE n	
EV214441	4.000	weakly similar to (118)AT2G33380| Symbols: RD20 | RD20 (RESPONSIVE TO DESSICATION 20); calcium ion binding | chr2:1415	
AM385947	3.987	no similarity	
JCVI_23559	3.981	weakly similar to (135)AT1G72110| Symbols: | similar to unknown protein [Arabidopsis thaliana] (TAIR:AT2G38995.1); similar to un	
JCVI_9210	3.977	weakly similar to (196)AT2G21790| Symbols: R1, RNR1 | R1/RNR1 (RIBONUCLEOTIDE REDUCTASE 1); ribonucleoside-diphosp	4.761
CV546620	3.965	no similarity	
JCVI_21499	3.965	no original description	
JCVI_38610	3.952	no original description	3.454
EV157970	3.951	weakly similar to (115)AT1G75910| Symbols: EXL4 | EXL4 (extracellular lipase 4); acyltransferase/ carboxylesterase/ lipase | chr1:285	
EV062201	3.951	no similarity	

JCVI_36371	3.926	weakly similar to (174)AT1G04645| Symbols: | self-incompatibility protein-related | chr1:1293852-1294238 REVERSE no original de	
JCVI_29256	3.915	no original description	
JCVI_36938	3.915	weakly similar to (129)AT1G15140| Symbols: | oxidoreductase NAD-binding domain-containing protein | chr1:5210637-5212132 REV	
EE543852	3.905	no similarity	
JCVI_7003	3.891	weakly similar to (134)AT5G22875| Symbols: | similar to Os04g0165500 [Oryza sativa (japonica cultivar-group)] (GB:NP_00105213	
JCVI_28578	3.889	no original description	
EE468019	3.878	weakly similar to (104)AT2G20670| Symbols: | similar to unknown protein [Arabidopsis thaliana] (TAIR:AT4G32480.1); similar to un	1.842
EV184492	3.877	no similarity	
EV167245	3.868	no similarity	
EX120603	3.859	weakly similar to (155)AT5G10100| Symbols: | trehalose-6-phosphate phosphatase, putative | chr5:3157981-3160276 FORWARD [21	
EX037832	3.830	no similarity	
JCVI_7320	3.830	no original description	
JCVI_30258	3.818	no original description	3.175
EV220855	3.817	no similarity	
JCVI_18251	3.805	moderately similar to (256)AT4G30640| Symbols: | F-box family protein (FBL19) | chr4:14952676-14953688 FORWARD no original	3.457
JCVI_39781	3.804	moderately similar to (389)AT3G54800| Symbols: | pleckstrin homology (PH) domain-containing protein / lipid-binding START doma	
EV140410	3.803	no similarity	
JCVI_37336	3.798	weakly similar to (146)AT4G08950| Symbols: | phosphate-responsive protein, putative (EXO) | chr4:5740375-5741319 FORWARD no	
JCVI_35228	3.782	no original description	
EV206048	3.777	no similarity	1.925
EV135474	3.774	no similarity	
JCVI_41856	3.763	moderately similar to (224)AT1G74930| Symbols: ORA47 | ORA47; DNA binding / transcription factor | chr1:28147900-28148487 FO	
H07737	3.757	no similarity	
BQ704822	3.753	no similarity	
EE567293	3.751	no similarity	
JCVI_36016	3.748	no original description	
JCVI_822	3.747	moderately similar to (237)AT1G72510| Symbols: | similar to unknown protein [Arabidopsis thaliana] (TAIR:AT2G09970.1); similar t	3.982
JCVI_21629	3.744	no original description	
EV225281	3.739	weakly similar to (193)AT1G10790| Symbols: | similar to hydroxyproline-rich glycoprotein family protein [Arabidopsis thaliana] (TAI	
RC_EX127879	3.722	no similarity	
JCVI_23445	3.719	moderately similar to (381)AT5G15110| Symbols: | pectate lyase family protein | chr5:4895969-4897685 FORWARDmoderately simil	
CD814264	3.717	no similarity	
EX015947	3.715	no similarity	5.005
JCVI_31632	3.708	no original description	
EX047708	3.708	weakly similar to (153)AT3G25940| Symbols: | transcription factor S-II (TFIIS) domain-containing protein | chr3:9496341-9497179 FO	
JCVI_5431	3.705	moderately similar to (449)AT4G37610| Symbols: BT5 | BT5 (BTB and TAZ domain protein 5); protein binding / transcription regulato	3.488
JCVI_42542	3.701	moderately similar to (217)AT2G32070| Symbols: | CCR4-NOT transcription complex protein, putative | chr2:13647906-13648733 RE	
EV155581	3.695	no similarity	
JCVI_23584	3.690	no original description	
EV098651	3.680	very weakly similar to (97.4)AT1G79380| Symbols: | copine-related | chr1:29865705-29867916 FORWARD [21477]	4.279
EV170843	3.646	no similarity	
EX136223	3.623	no similarity	
EX092440	3.623	no similarity	
EV211780	3.608	no similarity	
JCVI_14336	3.606	weakly similar to (125)AT2G42560| Symbols: | late embryogenesis abundant domain-containing protein / LEA domain-containing prot	
ES968179	3.602	no similarity	
JCVI_38371	3.597	moderately similar to (228)AT1G74930| Symbols: ORA47 | ORA47; DNA binding / transcription factor | chr1:28147900-28148487 FO	
JCVI_36473	3.595	no original description	
EV142173	3.588	no similarity	
RC_ES960896	3.586	no similarity	
JCVI_15742	3.579	weakly similar to (175)AT1G09370| Symbols: | enzyme inhibitor/ pectinesterase | chr1:3024903-3025427 FORWARD no original desc	
JCVI_22116	3.565	no original description	
EE491125	3.561	no similarity	
EV218517	3.553	moderately similar to (248)AT4G37610| Symbols: BT5 | BT5 (BTB and TAZ domain protein 5); protein binding / transcription regulato	
JCVI_36509	3.548	no original description	
EE554371	3.547	no similarity	
JCVI_39141	3.536	no original description	
JCVI_245	3.534	moderately similar to (202)AT5G20230| Symbols: BCB, ATBCB | ATBCB (ARABIDOPSIS BLUE-COPPER-BINDING PROTEIN);	-5.903
JCVI_39139	3.529	highly similar to (502)AT4G00290| Symbols: | mechanosensitive ion channel domain-containing protein / MS ion channel domain-cont	
EV110172	3.522	no similarity	
JCVI_14519	3.522	highly similar to (871)AT5G45340| Symbols: CYP707A3 | CYP707A3 (cytochrome P450, family 707, subfamily A, polypeptide 3); ox	2.437
JCVI_621	3.515	moderately similar to (377)AT3G53280| Symbols: CYP71B5 | CYP71B5 (CYTOCHROME P450 71B5); oxygen binding | chr3:19766	
JCVI_40564	3.511	very weakly similar to (82.0)AT1G10740| Symbols: | similar to unknown protein [Arabidopsis thaliana] (TAIR:AT1G23330.1); similar	
EV065101	3.506	no similarity	
JCVI_29891	3.500	no original description	
EV116639	3.487	weakly similar to (152)AT5G17920| Symbols: ATMETS, ATMS1, ATCIMS | ATCIMS (COBALAMIN-INDEPENDENT METHIONI	
JCVI_1677	3.487	moderately similar to (310)AT5G02040| Symbols: | prenylated rab acceptor (PRA1) family protein | chr5:401160-402468 FORWARD	
CO749260	3.479	no similarity	3.035
JCVI_42541	3.478	no original description	3.280
EV227530	3.476	no similarity	
EE517585	3.476	weakly similar to (115)AT2G32930| Symbols: ZFN2 | ZFN2 (ZINC FINGER PROTEIN 2); nucleic acid binding | chr2:13973067-1397	
DY005416	3.474	no similarity	
JCVI_32474	3.474	moderately similar to (221)AT3G19595| Symbols: | phosphoprotein phosphatase | chr3:6808591-6809514 REVERSE no original descr	
JCVI_32631	3.467	no original description	
JCVI_22123	3.466	no original description	
JCVI_8332	3.465	moderately similar to (267)AT1G14345| Symbols: | oxidoreductase | chr1:4899171-4899761 FORWARD no original description	
JCVI_17114	3.465	moderately similar to (344)AT1G18300| Symbols: ATNUDT4 | ATNUDT4 (Arabidopsis thaliana Nudix hydrolase homolog 4); hydrola	
EV109854	3.465	very weakly similar to (95.9)AT4G32910| Symbols: | similar to nucleoporin [Lotus japonicus] (GB:BAF45348.1); similar to putative nu	
JCVI_30009	3.465	weakly similar to (131)AT3G12830| Symbols: | auxin-responsive family protein | chr3:4079124-4079522 REVERSE no original descri	
JCVI_21587	3.459	weakly similar to (160)AT2G15580| Symbols: | zinc finger (C3HC4-type RING finger) family protein | chr2:6804769-6805897 FORW	
EV066619	3.453	no similarity	
EX055317	3.453	no similarity	
JCVI_6276	3.453	moderately similar to (264)AT2G01110| Symbols: UNE3, PGA2, TATC, APG2 | APG2 (ALBINO AND PALE GREEN 2) | chr2:8378	

H74559	3.449	weakly similar to (124)AT4G18780| Symbols: CESA8, IRX1, ATCESA8, LEW2 | CESA8 (CELLULOSE SYNTHASE 8); cellulose sy	
JCVI_258	3.442	highly similar to (521)AT2G37250| Symbols: ADK, ATPADK1 | ADK/ATPADK1 (ADENOSINE KINASE); adenylate kinase/ nucleo	
JCVI_20374	3.441	weakly similar to (177)AT2G25420| Symbols: | similar to TPR4/WSIP2 (TOPLESS-RELATED 4) [Arabidopsis thaliana] (TAIR:AT3(	
JCVI_691	3.441	weakly similar to (190)AT1G47200| Symbols: WPP2 | WPP2 (WPP domain protein 2) | chr1:17300621-17301163 REVERSE no origi	
EV225615	3.438	no similarity	
EE556267	3.435	no similarity	
CV973936	3.428	no similarity	
JCVI_40410	3.415	no original description	
JCVI_11803	3.415	highly similar to (753)AT4G20850| Symbols: TPP2 | TPP2 (TRIPEPTIDYL PEPTIDASE II); subtilase | chr4:11160946-11169900 RE	
CD827268	3.415	moderately similar to (215)AT5G12120| Symbols: | ubiquitin-associated (UBA)/TS-N domain-containing protein | chr5:3916230-3918	
AM395338	3.413	no similarity	
EE560203	3.403	weakly similar to (107)AT1G62740| Symbols: | stress-inducible protein, putative | chr1:23234691-23237045 FORWARDvery weakly s	
EV145756	3.402	no similarity	
JCVI_39793	3.394	moderately similar to (230)AT5G19190| Symbols: | similar to unknown protein [Arabidopsis thaliana] (TAIR:AT3G06070.1); similar t	
EE458789	3.393	no similarity	
EV134778	3.391	no similarity	
JCVI_2073	3.390	moderately similar to (209)AT5G19190| Symbols: | similar to unknown protein [Arabidopsis thaliana] (TAIR:AT3G06070.1); similar t	
EV068783	3.390	no similarity	
EV220406	3.386	weakly similar to (177)AT5G23450| Symbols: ATLCBK1 | ATLCBK1 (A. THALIANA LONG-CHAIN BASE (LCB) KINASE 1); dia	5.395
JCVI_3072	3.384	moderately similar to (312)AT3G22640| Symbols: | cupin family protein | chr3:8011909-8013890 REVERSEweakly similar to (129)V	
EV098037	3.381	weakly similar to (166)AT5G07200| Symbols: GA20OX3, ATGA20OX3 | YAP169 (GIBBERELLIN 20 OXIDASE 3); gibberellin 20-	
EE559752	3.376	weakly similar to (167)AT5G10630| Symbols: | elongation factor 1-alpha, putative / EF-1-alpha, putative | chr5:3360562-3364415 FOI	
EE567148	3.376	no similarity	4.581
RC_EE566066	3.374	no similarity	
EV171552	3.373	no similarity	
JCVI_25231	3.368	no original description	
EE562045	3.368	weakly similar to (151)AT2G38400| Symbols: AGT3 | AGT3 (ALANINE:GLYOXYLATE AMINOTRANSFERASE 3); alanine-glyox	
EL589415	3.362	very weakly similar to (99.4)AT3G13800| Symbols: | metallo-beta-lactamase family protein | chr3:4539379-4541373 FORWARD [208	
EV176799	3.361	no similarity	
JCVI_41073	3.357	weakly similar to (196)AT5G16260| Symbols: | RNA recognition motif (RRM)-containing protein | chr5:5311366-5315499 FORWAR	
RC_ES917680	3.357	no similarity	
JCVI_13622	3.356	no original description	
JCVI_37026	3.347	highly similar to (521)AT3G10030| Symbols: | aspartate/glutamate/uridylate kinase family protein | chr3:3092623-3094836 REVERSE	
AM394323	3.345	no similarity	
EV141354	3.343	no similarity	
EV190074	3.343	moderately similar to (249)AT2G41640| Symbols: | similar to unknown protein [Arabidopsis thaliana] (TAIR:AT3G57380.1); similar t	
JCVI_15308	3.342	moderately similar to (240)AT4G29340| Symbols: PRF4 | PRF4 (PROFILIN 4); actin binding | chr4:14447724-14448473 FORWARDı	
EV130893	3.333	no similarity	
JCVI_23734	3.333	no original description	
ES939469	3.333	no similarity	
JCVI_38985	3.330	weakly similar to (108)AT5G16590| Symbols: | leucine-rich repeat transmembrane protein kinase, putative | chr5:5431865-5433924 FC	
JCVI_23932	3.329	no original description	
JCVI_878	3.321	no original description	
EE560052	3.319	no similarity	
EE549860	3.318	no similarity	3.773
EV104652	3.317	no similarity	
JCVI_32464	3.315	no original description	
JCVI_26603	3.315	no original description	
EV123720	3.313	moderately similar to (315)AT5G65530| Symbols: | protein kinase, putative | chr5:26208070-26210052 REVERSEvery weakly similar	
EE418549	3.313	no similarity	3.239
JCVI_29432	3.308	very weakly similar to (99.0)AT4G08670| Symbols: | protease inhibitor/seed storage/lipid transfer protein (LTP) family protein | chr4:5	
EX104774	3.307	weakly similar to (124)AT1G06870| Symbols: | signal peptidase, putative | chr1:2108831-2110641 FORWARD [21826]	
JCVI_14218	3.306	moderately similar to (406)AT1G01050| Symbols: ATPPA1 | ATPPA1 (ARABIDOPSIS THALIANA PYROPHOSPHORYLASE 1); i	
JCVI_31922	3.304	highly similar to (568)AT5G13670| Symbols: | nodulin MtN21 family protein | chr5:4407208-4408958 REVERSE no original descript	
EV178873	3.304	moderately similar to (277)AT4G27500| Symbols: PPI1 | PPI1 (PROTON PUMP INTERACTOR 1) | chr4:13743620-13745906 FORV	
EV008705	3.304	very weakly similar to (96.7)AT5G13470| Symbols: | similar to hypothetical protein [Vitis vinifera] (GB:CAN76949.1) | chr5:4318565-	
EV006275	3.296	no similarity	2.053
ES965867	3.292	no similarity	
JCVI_29354	3.285	very weakly similar to (94.0)AT5G38820| Symbols: | amino acid transporter family protein | chr5:15560711-15562412 FORWARD no	
L33507	3.284	weakly similar to (127)AT1G11410| Symbols: | S-locus protein kinase, putative | chr1:3841286-3844284 FORWARD [132]	
JCVI_13550	3.279	moderately similar to (233)AT3G07350| Symbols: | similar to unknown protein [Arabidopsis thaliana] (TAIR:AT3G25240.1); similar t	
JCVI_37596	3.279	no original description	
EV134134	3.278	no similarity	
RC_ES966233	3.276	no similarity	
ES905207	3.275	weakly similar to (125)AT1G15170| Symbols: | MATE efflux family protein | chr1:5220685-5222751 FORWARD [21432]	
RC_EV090085	3.273	no similarity	
EX131063	3.271	no similarity	
JCVI_28495	3.270	weakly similar to (136)AT4G03090| Symbols: | similar to unnamed protein product [Vitis vinifera] (GB:CAO22433.1); contains InterP	3.446
EV130706	3.269	no similarity	
JCVI_32412	3.268	moderately similar to (419)AT5G28680| Symbols: | protein kinase family protein | chr5:10719441-10722017 REVERSEvery weakly si	
JCVI_4664	3.261	no original description	
ES983718	3.257	no similarity	
EV119333	3.257	no similarity	
EV178393	3.257	no similarity	
EV124923	3.257	moderately similar to (367)AT1G11080| Symbols: SCPL31 | SCPL31 (serine carboxypeptidase-like 31); serine carboxypeptidase | chr1:	
H07278	3.248	no similarity	
EV146828	3.242	no similarity	
EV091201	3.237	no similarity	
JCVI_11606	3.236	moderately similar to (352)AT1G66080| Symbols: | similar to hypothetical protein [Vitis vinifera] (GB:CAN77663.1); contains InterPr	4.215
EV096022	3.235	weakly similar to (134)AT5G42070| Symbols: | similar to unnamed protein product [Vitis vinifera] (GB:CAO69519.1) | chr5:1683634(	
JCVI_36344	3.231	weakly similar to (121)AT4G14450| Symbols: | Identical to Uncharacterized protein At4g14450, chloroplast precursor [Arabidopsis Th	
RC_ES969110	3.224	no similarity	
EX092285	3.220	no similarity	

JCVI_6476	3.219	no original description	
ES265746	3.218	very weakly similar to (97.4)AT4G28700| Symbols: | ammonium transporter, putative | chr4:14161687-14163201 FORWARD [21031]	
EE556375	3.218	no similarity	
ES983821	3.217	no similarity	
EX053177	3.212	no similarity	
CX189498	3.212	no similarity	
RC_EE557251	3.211	no similarity	
JCVI_10861	3.211	no original description	
EV203559	3.210	no similarity	
JCVI_5976	3.208	moderately similar to (398)AT2G37130| Symbols: | peroxidase 21 (PER21) (P21) (PRXR5) | chr2:15605304-15606813 REVERSEwe	
JCVI_37665	3.207	weakly similar to (166)AT5G47910| Symbols: ATRBOHD, RBOHD | RBOHD (RESPIRATORY BURST OXIDASE PROTEIN D) | c	
CD827326	3.202	moderately similar to (216)AT1G27040| Symbols: | nitrate transporter, putative | chr1:9386880-9390005 REVERSE [13979]	
EX083667	3.198	no similarity	3.237
RC_T18376	3.195	no similarity	
RC_ES967186	3.195	no similarity	
JCVI_35832	3.193	moderately similar to (237)AT3G21910| Symbols: | receptor-like protein kinase-related | chr3:7717379-7718412 FORWARD no origir	
JCVI_25170	3.189	no original description	
EE444936	3.184	weakly similar to (108)AT3G61550| Symbols: | zinc finger (C3HC4-type RING finger) family protein | chr3:22787419-22788057 FOF	
JCVI_4727	3.181	moderately similar to (258)AT1G10500| Symbols: ATCPISCA | ATCPISCA (CHLOROPLAST-LOCALIZED ISCA-LIKE PROTEIN)	
DY016083	3.180	no similarity	
JCVI_31874	3.173	weakly similar to (101)AT5G11480| Symbols: | GTP binding | chr5:3669351-3671472 REVERSE no original description	
JCVI_38399	3.172	no original description	
AM386974	3.172	no similarity	
EV149893	3.165	no similarity	
DY023218	3.162	weakly similar to (122)AT5G54320| Symbols: | similar to unknown protein [Arabidopsis thaliana] (TAIR:AT5G54330.1); contains Inte	
JCVI_39779	3.156	moderately similar to (259)AT1G79010| Symbols: | NADH-ubiquinone oxidoreductase 23 kDa subunit, mitochondrial (TYKY) | chr1:2	
EE451938	3.154	no similarity	
RC_EX133020	3.152	no similarity	
JCVI_8603	3.152	very weakly similar to (99.0)AT2G42580| Symbols: TTL3 | TTL3 (TETRATRICOPETIDE-REPEAT THIOREDOXIN-LIKE 3); protei	
JCVI_40501	3.151	no original description	
ES963726	3.149	no similarity	4.124
JCVI_36285	3.146	highly similar to (723)AT2G30800| Symbols: ATVT-1, HVT1 | HVT1 (HELICASE IN VASCULAR TISSUE AND TAPETUM); ATP	
JCVI_39477	3.141	no original description	
ES959392	3.141	weakly similar to (137)AT1G52930| Symbols: | brix domain-containing protein | chr1:19714929-19716771 FORWARD [21423] 1 419	
EV118479	3.138	no similarity	
JCVI_40884	3.136	weakly similar to (146)AT4G29780| Symbols: | similar to unknown protein [Arabidopsis thaliana] (TAIR:AT5G12010.1); similar to un	
EV102837	3.135	moderately similar to (201)AT3G15870| Symbols: | fatty acid desaturase family protein | chr3:5363124-5364431 FORWARD [21477]	
EV143562	3.135	no similarity	
H07682	3.134	no similarity	
EV035805	3.130	no similarity	
JCVI_38476	3.130	no original description	
EE557464	3.130	no similarity	
EV201717	3.130	no similarity	
JCVI_40442	3.128	no original description	
JCVI_33744	3.126	no original description	
EE558266	3.125	no similarity	
H07235	3.123	no similarity	
EV123208	3.116	no similarity	
EV088046	3.113	no similarity	
BQ792023	3.113	moderately similar to (214)AT3G28340| Symbols: GATL10 | GATL10 (Galacturonosyltransferase-like 10); polygalacturonate 4-alpha-g	
CX266483	3.113	no similarity	
EV127293	3.111	no similarity	
ES967428	3.111	no similarity	4.943
RC_ES966572	3.110	no similarity	
EV109388	3.107	no similarity	
EV058563	3.107	weakly similar to (192)AT4G22540| Symbols: | oxysterol-binding family protein | chr4:11860980-11863897 REVERSE [21442]	
EE470787	3.104	no similarity	
ES906042	3.103	moderately similar to (249)AT1G72130| Symbols: | proton-dependent oligopeptide transport (POT) family protein | chr1:27141425-27	
RC_CD838931	3.102	no similarity	5.419
EV062941	3.100	no similarity	
AM387385	3.099	no similarity	
EV129042	3.096	no similarity	
JCVI_40989	3.096	no original description	
JCVI_19330	3.096	weakly similar to (107)AT1G32920| Symbols: | similar to unknown protein [Arabidopsis thaliana] (TAIR:AT1G32928.1) | chr1:11928	
RC_EE565485	3.096	no similarity	
EE455325	3.093	moderately similar to (283)AT4G21630| Symbols: | subtilase family protein | chr4:11492260-11495512 REVERSE [20178]	
DY009530	3.093	weakly similar to (146)AT1G10020| Symbols: | similar to unknown protein [Arabidopsis thaliana] (TAIR:AT4G29310.1); similar to un	
JCVI_116	3.092	moderately similar to (444)AT4G23920| Symbols: UGE2 | UGE2 (UDP-D-GLUCOSE/UDP-D-GALACTOSE 4-EPIMERASE 2); UD	
EV221567	3.090	moderately similar to (204)AT1G24510| Symbols: | T-complex protein 1 epsilon subunit, putative / TCP-1-epsilon, putative / chaperon	
JCVI_15779	3.090	weakly similar to (119)AT3G27640| Symbols: | transducin family protein / WD-40 repeat family protein | chr3:10233544-10236704 FO	
JCVI_39723	3.082	very weakly similar to (84.7)AT1G12940| Symbols: ATNRT2.5 | ATNRT2.5 (NITRATE TRANSPORTER2.5); nitrate transmembrane	
EX019133	3.082	moderately similar to (266)AT5G05970| Symbols: | transducin family protein / WD-40 repeat family protein | chr5:1795465-1799314 I	
JCVI_4883	3.081	moderately similar to (253)AT1G47980| Symbols: | similar to unknown protein [Arabidopsis thaliana] (TAIR:AT3G62730.1); similar t	
H07167	3.081	no similarity	
ES934693	3.080	no similarity	
L37981	3.076	no similarity	
RC_EE562785	3.076	no similarity	
JCVI_7486	3.076	moderately similar to (255)AT5G30495| Symbols: | similar to unknown protein [Arabidopsis thaliana] (TAIR:AT1G54770.1); similar t	
ES985657	3.076	moderately similar to (345)AT1G25240| Symbols: | epsin N-terminal homology (ENTH) domain-containing protein / clathrin assembly	
EX108697	3.075	moderately similar to (386)AT1G13300| Symbols: | myb family transcription factor | chr1:4556974-4558588 FORWARD [21827]	
EV063849	3.074	no similarity	3.089
JCVI_24649	3.071	moderately similar to (204)AT4G25490| Symbols: DREB1B, CBF1 | CBF1 (C-REPEAT/DRE BINDING FACTOR 1); DNA binding /	5.442
JCVI_24545	3.070	moderately similar to (311)AT1G18370| Symbols: HIK | HIK (HINKEL); microtubule motor | chr1:6319725-6323813 REVERSE no o	

H07236	3.064	no similarity	
JCVI_13066	3.057	moderately similar to (216)AT4G30130| Symbols: | similar to unknown protein [Arabidopsis thaliana] (TAIR:AT2G19090.1); similar t	
JCVI_30387	3.057	no original description	
EV197141	3.056	no similarity	
RC_ES902576	3.055	no similarity	
JCVI_10033	3.053	moderately similar to (312)AT2G28250| Symbols: NCRK | protein kinase family protein | chr2:12051081-12053416 FORWARD no or	
EV225682	3.051	no similarity	
JCVI_11929	3.051	weakly similar to (189)AT1G17920| Symbols: HDG12 | HDG12 (HOMEODOMAIN GLABROUS12); transcription factor | chr1:6162	
EE558921	3.050	no similarity	
JCVI_40231	3.039	weakly similar to (175)AT5G13200| Symbols: | GRAM domain-containing protein / ABA-responsive protein-related | chr5:4207084-42	
EE569167	3.035	no similarity	
JCVI_15806	3.034	moderately similar to (238)AT1G05180| Symbols: AXR1 | AXR1 (AUXIN RESISTANT 1); small protein activating enzyme | chr1:149	
EE502065	3.032	weakly similar to (155)AT3G11930| Symbols: | universal stress protein (USP) family protein | chr3:3776377-3777399 FORWARD [20	
AT001747	3.031	no similarity	
JCVI_5276	3.031	weakly similar to (150)AT4G00780| Symbols: | meprin and TRAF homology domain-containing protein / MATH domain-containing p	
JCVI_18058	3.030	weakly similar to (194)AT1G76990| Symbols: ACR3 | ACR3 (ACT Domain Repeat 3) | chr1:28938281-28940073 FORWARD no orig	
EV107976	3.029	no similarity	
AM395367	3.028	no similarity	
JCVI_35707	3.028	highly similar to (540)AT5G22760| Symbols: | PHD finger family protein | chr5:7571638-7577665 FORWARD no original description	
RC_EE505338	3.026	no similarity	
EV210657	3.026	no similarity	
RC_EE567017	3.026	no similarity	
JCVI_23023	3.025	very weakly similar to (99.0)AT5G57990| Symbols: UBP23 | UBP23 (UBIQUITIN-SPECIFIC PROTEASE 23); ubiquitin-specific prot	2.413
EE558102	3.024	no similarity	
EV089328	3.024	no similarity	
JCVI_25666	3.023	highly similar to (599)AT1G79630| Symbols: | protein phosphatase 2C family protein / PP2C family protein | chr1:29967824-2996981	
JCVI_3719	3.018	weakly similar to (164)AT3G29000| Symbols: | calcium-binding EF hand family protein | chr3:11007028-11007612 FORWARD no or	
EV143537	3.017	no similarity	
ES942636	3.015	no similarity	
RC_JCVI_42160	3.013	no original description	
JCVI_20571	3.013	weakly similar to (117)AT3G07940| Symbols: | zinc finger and C2 domain protein, putative | chr3:2529548-2531374 FORWARD no o	
CD819340	3.012	no similarity	
EX016181	3.011	no similarity	
JCVI_32543	3.011	weakly similar to (107)AT1G60720| Symbols: | similar to unknown protein [Arabidopsis thaliana] (TAIR:AT1G33710.1); similar to pu	
EV131863	3.010	very weakly similar to (94.7)AT4G24380| Symbols: | similar to unknown protein [Arabidopsis thaliana] (TAIR:AT5G65400.1); similar	
JCVI_38228	3.001	no original description	2.783
EE392272	3.000	no similarity	
ES265006	3.000	moderately similar to (219)AT5G11820| Symbols: | similar to unknown protein [Arabidopsis thaliana] (TAIR:AT5G26050.1); contains	
EV145403	2.998	no similarity	
JCVI_35083	2.998	no original description	
JCVI_33374	2.996	no original description	
EV066024	2.993	moderately similar to (244)AT1G78400| Symbols: | glycoside hydrolase family 28 protein / polygalacturonase (pectinase) family protei	4.777
CN729994	2.992	no similarity	
CV433774	2.991	no similarity	
JCVI_5052	2.989	moderately similar to (496)AT3G21560| Symbols: UGT84A2 | UGT84A2; UDP-glycosyltransferase/ sinapate 1-glucosyltransferase | ch	4.673
EV151455	2.988	no similarity	
JCVI_30257	2.988	no original description	
EV151244	2.987	no similarity	
JCVI_16434	2.987	no original description	
EX112711	2.985	weakly similar to (118)AT3G14690| Symbols: CYP72A15 | CYP72A15 (cytochrome P450, family 72, subfamily A, polypeptide 15); o	
JCVI_16400	2.984	moderately similar to (201)AT5G54850| Symbols: | unknown protein | chr5:22297981-22298589 REVERSE no original description	
EE568357	2.984	weakly similar to (119)AT3G57870| Symbols: EMB1637, SCE1, AHUS5 | AHUS5 (SUMO CONJUGATION ENZYME 1); ubiquitin-	3.059
DY009096	2.984	no similarity	
EV130798	2.980	no similarity	
JCVI_4761	2.978	moderately similar to (341)AT1G01980| Symbols: SEC1A, ATSEC1A | ATSEC1A; electron carrier | chr1:340374-341999 REVERSE	
EV200335	2.977	very weakly similar to (96.7)AT5G43100| Symbols: | aspartyl protease family protein | chr5:17316492-17319946 FORWARD [21490]	3.196
EV138423	2.977	no similarity	
EE521171	2.977	no similarity	
JCVI_28651	2.977	weakly similar to (171)AT1G66120| Symbols: | acyl-activating enzyme 11 (AAE11) | chr1:24616303-24618353 FORWARD no origin	
EV006648	2.976	no similarity	
EX132070	2.975	moderately similar to (232)AT4G25810| Symbols: XTH23, XTR6 | XTR6 (XYLOGLUCAN ENDOTRANSGLYCOSYLASE 6); hydr	
EV132473	2.974	no similarity	
JCVI_21519	2.972	highly similar to (503)AT4G21326| Symbols: ATSBT3.12 | ATSBT3.12; subtilase | chr4:11346991-11349664 FORWARD no original	3.975
EX125813	2.969	weakly similar to (164)AT5G45280| Symbols: | pectinacetylesterase, putative | chr5:18364089-18366715 FORWARD [21831]	
ES961646	2.969	no similarity	
JCVI_38197	2.967	no original description	
JCVI_38756	2.965	moderately similar to (404)AT5G15130| Symbols: ATWRKY72, WRKY72 | WRKY72 (WRKY DNA-binding protein 72); transcriptio	
CD840069	2.962	no similarity	
RC_EX044727	2.959	no similarity	
DW997719	2.955	no similarity	
EV028688	2.955	no similarity	
EV125134	2.955	no similarity	
EV225683	2.951	no similarity	3.483
EV165309	2.946	moderately similar to (215)AT1G22380| Symbols: ATUGT85A3 | ATUGT85A3 (UDP-GLUCOSYL TRANSFERASE 85A3); glucuro	
EX098294	2.946	moderately similar to (276)AT1G27100| Symbols: | similar to unknown protein [Arabidopsis thaliana] (TAIR:AT1G69900.1); similar t	
JCVI_39943	2.946	moderately similar to (214)AT5G24860| Symbols: FPF, FPF1 | FPF1 (FLOWERING PROMOTING FACTOR 1) | chr5:8541825-8542	
JCVI_11930	2.946	moderately similar to (236)AT2G01080| Symbols: | similar to unknown protein [Arabidopsis thaliana] (TAIR:AT3G54200.1); similar t	
JCVI_17080	2.946	weakly similar to (136)AT4G04780| Symbols: | similar to unknown protein [Arabidopsis thaliana] (TAIR:AT5G15690.1); similar to pr	
JCVI_10679	2.946	weakly similar to (187)AT4G34790| Symbols: | auxin-responsive family protein | chr4:16594544-16594870 FORWARDvery weakly s	
EE556656	2.944	no similarity	4.962
CX192412	2.943	moderately similar to (208)AT1G28130| Symbols: GH3.17 | GH3.17 | chr1:9826366-9827870 FORWARDweakly similar to (107)GH	
JCVI_26441	2.943	highly similar to (635)AT5G59160| Symbols: PPO, TOPP2 | TOPP2 (Type one serine/threonine protein phosphatase 2); protein serine/t	4.993
EX044341	2.943	weakly similar to (112)AT3G58850| Symbols: PAR2 | PAR2 (PHY RAPIDLY REGULATED 2); transcription regulator | chr3:217705	

JCVI_3834	2.942	moderately similar to (402)AT1G70710| Symbols: CEL1, AtGH9B1 | AtGH9B1 (ARABIDOPSIS THALIANA GLYCOSYL HYDRO	2.045
EX091671	2.939	very weakly similar to (97.4)AT4G34430| Symbols: ATSWI3D, CHB3 | CHB3 (Arabidopsis thaliana switch 3D); DNA binding / transcr	
EV184017	2.939	no similarity	
ES264932	2.929	weakly similar to (168)AT5G55060| Symbols: | catalytic | chr5:22359305-22363626 FORWARD [15723]	3.501
EV025072	2.928	no similarity	
RC_ES967621	2.927	no similarity	
JCVI_29369	2.927	highly similar to (504)AT5G25510| Symbols: | serine/threonine protein phosphatase 2A (PP2A) regulatory subunit B', putative | chr5:8	
JCVI_36732	2.926	moderately similar to (398)AT4G01950| Symbols: ATGPAT3, GPAT3 | ATGPAT3/GPAT3 (GLYCEROL-3-PHOSPHATE ACYLTR	
JCVI_31135	2.925	no original description	
JCVI_18331	2.919	highly similar to (928)AT1G68560| Symbols: ATXYL1, XYL1 | ATXYL1/XYL1 (ALPHA-XYLOSIDASE 1); alpha-N-arabinofurano	
EV225843	2.915	weakly similar to (125)AT1G22710| Symbols: SUT1, ATSUC2, SUC2 | SUC2 (SUCROSE-PROTON SYMPORTER 2); carbohydrate	
JCVI_33140	2.915	no original description	
ES269078	2.915	moderately similar to (213)AT4G19230| Symbols: CYP707A1 | CYP707A1 (cytochrome P450, family 707, subfamily A, polypeptide	
EV090893	2.915	moderately similar to (214)ATCG01000| Symbols: YCF1.1 | hypothetical protein | chrC:109405-110436 FORWARDweakly similar to	
EV179477	2.915	moderately similar to (234)AT1G67830| Symbols: ATFXG1 | ATFXG1 (ALPHA-FUCOSIDASE 1); alpha-L-fucosidase/ carboxylester	
JCVI_17081	2.915	weakly similar to (108)AT5G17350| Symbols: | similar to unknown protein [Arabidopsis thaliana] (TAIR:AT3G03280.1); similar to un	
EV069452	2.914	weakly similar to (147)AT5G25470| Symbols: | DNA binding | chr5:8865857-8867237 REVERSE [21443]	3.346
ES954165	2.912	no similarity	
JCVI_13878	2.912	moderately similar to (466)AT5G45340| Symbols: CYP707A3 | CYP707A3 (cytochrome P450, family 707, subfamily A, polypeptide	
CD815798	2.912	weakly similar to (105)AT1G13730| Symbols: | nuclear transport factor 2 (NTF2) family protein / RNA recognition motif (RRM)-conta	
EE554795	2.903	weakly similar to (116)AT1G80790| Symbols: | XH/XS domain-containing protein / XS zinc finger domain-containing protein | chr1:3	
JCVI_40146	2.902	moderately similar to (229)AT1G33240| Symbols: AT-GTL2 | AT-GTL1 (Arabidopsis thaliana GT2-like 1); transcription factor | chr1:1	
JCVI_31926	2.901	no original description	
EE502096	2.900	weakly similar to (195)AT5G55050| Symbols: | GDSL-motif lipase/hydrolase family protein | chr5:22354971-22356967 FORWARDw	
ES950157	2.900	no similarity	
EE526539	2.898	no similarity	
EX119892	2.897	no similarity	
EV145334	2.895	weakly similar to (199)AT3G15990| Symbols: SULTR3;4 | SULTR3;4; sulfate transmembrane transporter | chr3:5427087-5430685 FO	
JCVI_29889	2.893	no original description	3.057
EV211227	2.890	very weakly similar to (99.0)AT3G26460| Symbols: | major latex protein-related / MLP-related | chr3:9685290-9685844 REVERSE [2	
EV144897	2.889	no similarity	
JCVI_27048	2.889	moderately similar to (367)AT1G69940| Symbols: ATPPME1 | ATPPME1; pectinesterase | chr1:26347212-26348634 REVERSEweak	
JCVI_23849	2.888	no original description	
EE567864	2.887	no similarity	
EE561199	2.886	no similarity	
EX091761	2.882	moderately similar to (260)AT1G08990| Symbols: PGSIP5 | PGSIP5 (PLANT GLYCOGENIN-LIKE STARCH INITIATION PROTE	
JCVI_34276	2.882	weakly similar to (172)AT1G52680| Symbols: | late embryogenesis abundant protein-related / LEA protein-related | chr1:19622087-19	
ES988392	2.878	weakly similar to (150)AT2G32460| Symbols: MYB101, AtM1, AtMYB101 | AtM1/AtMYB101/MYB101 (myb domain protein 101)	
EV103809	2.876	weakly similar to (153)AT4G13540| Symbols: | similar to unknown protein [Arabidopsis thaliana] (TAIR:AT3G23930.1); similar to un	
JCVI_19414	2.875	no original description	
JCVI_40938	2.875	no original description	
JCVI_33601	2.873	no original description	
EX066487	2.872	no similarity	
CX190441	2.868	no similarity	
ES911338	2.868	no similarity	
EX016747	2.868	no similarity	
EE554692	2.860	no similarity	
EX103459	2.860	moderately similar to (301)AT4G33730| Symbols: | pathogenesis-related protein, putative | chr4:16185101-16185619 FORWARDwea	
JCVI_31867	2.858	no original description	
JCVI_23644	2.852	weakly similar to (120)AT4G29780| Symbols: | similar to unknown protein [Arabidopsis thaliana] (TAIR:AT5G12010.1); similar to un	
EV224979	2.850	weakly similar to (182)AT5G64370| Symbols: BETA-UP | BETA-UP (BETA-UREIDOPROPIONASE); beta-ureidopropionase | chr5:2	
JCVI_20804	2.848	weakly similar to (113)AT3G19580| Symbols: AZF2 | AZF2 (ARABIDOPSIS ZINC-FINGER PROTEIN 2) | chr3:6803299-6804120 F	
JCVI_27647	2.848	weakly similar to (171)AT3G20520| Symbols: | glycerophosphoryl diester phosphodiesterase family protein | chr3:7162851-7165748 F	
RC_JCVI_41988	2.842	no original description	
EX137129	2.840	no similarity	2.226
CB617591	2.839	no similarity	3.317
ES969249	2.838	no similarity	
EX103165	2.838	weakly similar to (148)AT3G52960| Symbols: | peroxiredoxin type 2, putative | chr3:19650677-19651381 FORWARD [21826]	
EE568624	2.838	no similarity	
JCVI_37878	2.837	no original description	4.222
JCVI_26070	2.836	no original description	
CV973925	2.835	no similarity	
EL590006	2.833	no similarity	
EX130669	2.833	no similarity	
AM062480	2.833	weakly similar to (134)AT1G43040| Symbols: | auxin-responsive protein, putative | chr1:16186649-16186963 FORWARD [17712] 1	
ES986655	2.832	no similarity	
JCVI_35330	2.831	no original description	
RC_EE542508	2.830	no similarity	
BG543659	2.830	no similarity	
EV179248	2.830	moderately similar to (211)AT1G67230| Symbols: LINC1 | LINC1 (LITTLE NUCLEI1) | chr1:25155224-25159695 REVERSE [2148	
EV135166	2.829	weakly similar to (148)AT3G55280| Symbols: | 60S ribosomal protein L23A (RPL23aB) | chr3:20511644-20512496 FORWARDweak	
JCVI_33988	2.829	no original description	
JCVI_39493	2.828	moderately similar to (375)AT4G21640| Symbols: | subtilase family protein | chr4:11496846-11500630 REVERSE no original descrip	
JCVI_34341	2.825	very weakly similar to (82.4)AT1G62020| Symbols: | coatomer protein complex, subunit alpha, putative | chr1:22923479-22927393 FO	
EV125286	2.825	weakly similar to (174)AT1G02900| Symbols: RALFL1 | RALFL1 (RALF-LIKE 1) | chr1:653976-654338 REVERSE [21479]	
EV194079	2.824	moderately similar to (226)AT1G55620| Symbols: ATCLC-F, CLC-F | CLC-F (CHLORIDE CHANNEL F); voltage-gated chloride cha	
DY030160	2.822	no similarity	
EV211479	2.818	no similarity	
BQ704860	2.815	no similarity	
BG543383	2.814	weakly similar to (105)AT5G61460| Symbols: MIM | MIM (HYPERSENSITIVE TO MMS, IRRADIATION AND MMC); ATP bindi	
JCVI_7633	2.814	moderately similar to (213)AT3G54190| Symbols: | similar to unknown protein [Arabidopsis thaliana] (TAIR:AT2G38630.1); similar t	
JCVI_17406	2.813	highly similar to (858)AT1G74960| Symbols: KAS2, FAB1 | FAB1 (FATTY ACID BIOSYNTHESIS 1); fatty-acid synthase | chr1:281	
ES947718	2.813	no similarity	

JCVI_3227	2.810	no original description	
JCVI_19496	2.809	weakly similar to (110)AT5G50460| Symbols: | protein transport protein SEC61 gamma subunit, putative | chr5:20569394-20569735 F	
EV112414	2.809	no similarity	
JCVI_3208	2.808	no original description	
CV545114	2.808	moderately similar to (223)AT5G09590| Symbols: HSC70-5, mtHSC70-2 | mtHSC70-2 (HEAT SHOCK PROTEIN 70); ATP binding /	
ES898193	2.807	no similarity	
EV130046	2.806	no similarity	
DY001809	2.806	no similarity	
EE564722	2.804	no similarity	
EV197862	2.804	no similarity	
EV216622	2.804	no similarity	
JCVI_13374	2.804	moderately similar to (303)AT1G70260| Symbols: | nodulin MtN21 family protein | chr1:26460730-26463001 REVERSE no original c	
EE560668	2.803	no similarity	
JCVI_23275	2.803	moderately similar to (218)AT1G21280| Symbols: | similar to hypothetical protein [Vitis vinifera] (GB:CAN81001.1); contains InterPr	
JCVI_20749	2.800	moderately similar to (497)AT4G35850| Symbols: | pentatricopeptide (PPR) repeat-containing protein | chr4:16983641-16986684 FOR	
JCVI_7567	2.798	no original description	3.413
JCVI_20088	2.794	moderately similar to (441)AT4G19540| Symbols: | similar to HCF101 (HIGH-CHLOROPHYLL-FLUORESCENCE 101), ATP bindi	
EV143743	2.791	no similarity	
EV126936	2.791	no similarity	-1.053
EV147210	2.790	no similarity	
JCVI_41628	2.790	moderately similar to (229)AT5G18460| Symbols: | similar to unknown protein [Arabidopsis thaliana] (TAIR:AT1G23340.1); similar t	
EE529933	2.790	moderately similar to (211)AT1G66880| Symbols: | serine/threonine protein kinase family protein | chr1:24950591-24959101 FORWA	-1.029
JCVI_19284	2.790	very weakly similar to (88.6)AT1G23550| Symbols: SRO2 | SRO2 (SIMILAR TO RCD ONE 2); NAD+ ADP-ribosyltransferase | chr1:8	
JCVI_9252	2.788	highly similar to (693)AT1G29980| Symbols: | similar to unknown protein [Arabidopsis thaliana] (TAIR:AT2G34510.1); similar to unk	
JCVI_735	2.787	no original description	
RC_EE564048	2.787	no similarity	
EE420291	2.786	no similarity	
JCVI_33906	2.786	highly similar to (771)AT2G32860| Symbols: | glycosyl hydrolase family 1 protein | chr2:13947310-13950673 FORWARDmoderately	
JCVI_37840	2.784	no original description	
JCVI_888	2.784	moderately similar to (386)AT4G00040| Symbols: | chalcone and stilbene synthase family protein | chr4:14653-15897 FORWARDwea	
JCVI_38737	2.782	moderately similar to (435)AT4G12420| Symbols: SKU5 | SKU5 (skewed 5); copper ion binding | chr4:7349938-7352865 REVERSEn	
EV219578	2.782	moderately similar to (335)AT2G30210| Symbols: LAC3 | LAC3 (laccase 3); copper ion binding / oxidoreductase | chr2:12894812-128	
EV128384	2.782	no similarity	
JCVI_33673	2.782	very weakly similar to (92.0)AT3G44870| Symbols: | S-adenosyl-L-methionine:carboxyl methyltransferase family protein | chr3:163932	
H07270	2.780	no similarity	
JCVI_39393	2.778	weakly similar to (186)AT1G75140| Symbols: | Identical to Uncharacterized membrane protein At1g75140 [Arabidopsis Thaliana] (GE	
CN728444	2.778	weakly similar to (120)AT5G15470| Symbols: GAUT14 | GAUT14 (Galacturonosyltransferase 14); polygalacturonate 4-alpha-galacturo	
EE408514	2.774	no similarity	
EV165860	2.774	no similarity	
JCVI_26896	2.774	moderately similar to (436)AT1G68020| Symbols: TPS6, ATTPS6 | ATTPS6 (Arabidopsis thaliana trehalose phosphatase/synthase 6); t	
EE530227	2.773	no similarity	3.812
EE567771	2.773	no similarity	
JCVI_32957	2.773	moderately similar to (270)AT3G09230| Symbols: ATMYB1 | ATMYB1 (MYB DOMAIN PROTEIN 1); DNA binding / transcription	
JCVI_22325	2.767	weakly similar to (196)AT3G19680| Symbols: | similar to unknown protein [Arabidopsis thaliana] (TAIR:AT1G50040.1); similar to un	
EE558033	2.767	no similarity	
JCVI_22321	2.767	no original description	
ES968425	2.761	no similarity	
EE534462	2.760	no similarity	
H07351	2.759	very weakly similar to (85.9)AT5G17350| Symbols: | similar to unknown protein [Arabidopsis thaliana] (TAIR:AT3G03280.1); similar	
CD836896	2.757	weakly similar to (155)AT3G29670| Symbols: | transferase family protein | chr3:11530431-11531786 FORWARD [13981]	
JCVI_2034	2.755	no original description	4.045
EE565488	2.753	no similarity	
ES966938	2.751	no similarity	
EE560192	2.751	no similarity	
JCVI_20970	2.751	no original description	
JCVI_33737	2.751	no original description	
JCVI_5358	2.750	weakly similar to (169)AT1G30120| Symbols: PDH-E1 BETA | PDH-E1 BETA (PYRUVATE DEHYDROGENASE E1 BETA); pyru	
AM058692	2.750	weakly similar to (179)AT4G22250| Symbols: | zinc finger (C3HC4-type RING finger) family protein | chr4:11767697-11768341 REV	
JCVI_16557	2.747	moderately similar to (273)AT3G50060| Symbols: MYB77 | MYB77; DNA binding / transcription factor | chr3:18569129-18570034 R	
EX116638	2.743	no similarity	
JCVI_33378	2.743	moderately similar to (445)AT1G22760| Symbols: PAB3 | PAB3 (POLY(A) BINDING PROTEIN 3); RNA binding | chr1:8055588-80	
EE501925	2.737	no similarity	2.738
JCVI_19644	2.734	weakly similar to (128)AT1G48140| Symbols: | dolichol-phosphate mannosyltransferase-related | chr1:17787071-17787583 FORWAR	
EV083865	2.734	no similarity	
EV128635	2.732	no similarity	2.819
CO749390	2.732	no similarity	
EV112275	2.732	no similarity	
EV226371	2.731	no similarity	
CD819972	2.731	no similarity	
JCVI_16590	2.729	moderately similar to (372)AT3G10480| Symbols: ANAC050 | ANAC050 | chr3:3264415-3266786 FORWARDweakly similar to (18	
JCVI_32778	2.729	weakly similar to (180)AT5G45970| Symbols: ROP7, ATRAC2, ARAC2, ATROP7 | ARAC2 (RHO-RELATED PROTEIN FROM PL	
CD840408	2.727	very weakly similar to (84.3)AT5G07550| Symbols: ATGRP19, GRP19 | GRP19 (Glycine rich protein 19) | chr5:2388619-2389185 RE	
JCVI_24817	2.721	weakly similar to (188)AT2G37980| Symbols: | similar to unknown protein [Arabidopsis thaliana] (TAIR:AT5G01100.1); similar to un	
JCVI_38370	2.720	moderately similar to (416)AT4G29780| Symbols: | similar to unknown protein [Arabidopsis thaliana] (TAIR:AT5G12010.1); similar t	
DY004458	2.717	moderately similar to (308)AT4G20200| Symbols: | terpene synthase/cyclase family protein | chr4:10908682-10911161 REVERSEwea	
EE460675	2.716	no similarity	
EV066585	2.712	weakly similar to (148)AT3G15810| Symbols: | similar to unknown protein [Arabidopsis thaliana] (TAIR:AT1G80120.1); similar to un	
EE562412	2.711	no similarity	
JCVI_28897	2.710	weakly similar to (101)AT1G67030| Symbols: ZFP6 | ZFP6 (ZINC FINGER PROTEIN 6); nucleic acid binding / transcription factor/ z	
JCVI_32592	2.709	moderately similar to (294)AT2G46960| Symbols: CYP709B1 | CYP709B1 (cytochrome P450, family 709, subfamily B, polypeptide 1	
RC_EX083955	2.709	no similarity	
RC_EV010251	2.708	no similarity	
EV174730	2.707	very weakly similar to (95.9)CYB_SPEAN [21486] 62 439 786	3.344

EV131193	2.701	very weakly similar to (95.9)AT2G19880| Symbols: | ceramide glucosyltransferase, putative | chr2:8588660-8592200 FORWARD [214	
EX056651	2.701	no similarity	
JCVI_12359	2.696	moderately similar to (457)AT4G01810| Symbols: | protein transport protein-related | chr4:776734-779802 REVERSE no original desc	
EV111301	2.692	no similarity	2.674
EV018309	2.690	no similarity	
ES901996	2.688	weakly similar to (137)AT1G04590| Symbols: | similar to pentatricopeptide (PPR) repeat-containing protein [Arabidopsis thaliana] (TA	
JCVI_16560	2.688	weakly similar to (112)AT5G10660| Symbols: | calmodulin-binding protein-related | chr5:3370554-3371777 FORWARD no original d	
EE478991	2.687	moderately similar to (278)AT5G25170| Symbols: | similar to unknown protein [Arabidopsis thaliana] (TAIR:AT2G25190.1); similar t	
ES967033	2.685	no similarity	5.132
JCVI_25518	2.684	highly similar to (528)AT5G15130| Symbols: ATWRKY72, WRKY72 | WRKY72 (WRKY DNA-binding protein 72); transcription fac	
EE561031	2.683	no similarity	
JCVI_16974	2.681	very weakly similar to (84.7)AT3G19680| Symbols: | similar to unknown protein [Arabidopsis thaliana] (TAIR:AT1G50040.1); similar	
JCVI_30949	2.680	no original description	3.888
ES954371	2.678	no similarity	
CD840090	2.677	no similarity	
EE551115	2.676	no similarity	
JCVI_40144	2.676	no original description	
EV194343	2.675	weakly similar to (140)AT4G32551| Symbols: RON2, LUG | LUG (LEUNIG) | chr4:15707869-15713365 FORWARD [21489] 21 709	5.118
RC_ES954436	2.675	no similarity	3.067
CD816610	2.674	moderately similar to (218)AT2G24220| Symbols: ATPUP5 | ATPUP5 (Arabidopsis thaliana purine permease 5); purine transmembran	
EE466624	2.672	weakly similar to (101)AT1G52695| Symbols: | phospholipase/carboxylesterase family protein | chr1:19625079-19627056 REVERSE	
EV101236	2.672	no similarity	
EV182486	2.672	very weakly similar to (100)AT5G57560| Symbols: XTH22, TCH4 | TCH4 (TOUCH 4); hydrolase, acting on glycosyl bonds / xylogluc	
JCVI_21618	2.672	weakly similar to (168)AT5G02240| Symbols: | binding / catalytic/ coenzyme binding | chr5:451500-452982 FORWARD no original d	
EV170947	2.671	moderately similar to (311)AT1G76130| Symbols: ATAMY2, AMY2 | AMY2/ATAMY2 (ALPHA-AMYLASE-LIKE 2); alpha-amylas	
JCVI_38749	2.669	moderately similar to (348)AT5G04710| Symbols: | aspartyl aminopeptidase, putative | chr5:1357274-1360129 REVERSE no original	
JCVI_40532	2.669	moderately similar to (297)AT2G21790| Symbols: R1, RNR1 | R1/RNR1 (RIBONUCLEOTIDE REDUCTASE 1); ribonucleoside-dipl	1.773
EX131720	2.665	no similarity	5.534
CX270831	2.665	no similarity	
ES942692	2.663	no similarity	
EX078964	2.663	no similarity	
JCVI_21884	2.663	weakly similar to (108)AT4G00730| Symbols: AHDP, ANL2 | ANL2 (ANTHOCYANINLESS 2) | chr4:301071-304103 REVERSE nc	
EV166537	2.662	no similarity	
EE502315	2.661	no similarity	
EV177552	2.661	no similarity	
JCVI_27398	2.661	very weakly similar to (100)AT1G63670| Symbols: | similar to unknown protein [Arabidopsis thaliana] (TAIR:AT2G45900.1); similar	
EV141703	2.661	very weakly similar to (92.0)AT3G44870| Symbols: | S-adenosyl-L-methionine:carboxyl methyltransferase family protein | chr3:163932	
EV091861	2.661	no similarity	
DY029976	2.660	no similarity	
JCVI_20274	2.659	weakly similar to (182)AT2G30020| Symbols: | protein phosphatase 2C, putative / PP2C, putative | chr2:12821514-12822981 FORWA	
ES984449	2.658	no similarity	
CD821578	2.657	very weakly similar to (86.7)AT2G23148| Symbols: | similar to unknown protein [Arabidopsis thaliana] (TAIR:AT2G23142.1); contain	
ES968363	2.653	no similarity	
JCVI_18078	2.653	weakly similar to (194)AT4G30070| Symbols: LCR59 | LCR59 (Low-molecular-weight cysteine-rich 59) | chr4:14697555-14698321 F(	
JCVI_32239	2.653	no original description	
EX065570	2.649	no similarity	4.486
EV015620	2.649	no similarity	
EV098177	2.648	moderately similar to (248)AT5G53490| Symbols: | thylakoid lumenal 17.4 kDa protein, chloroplast | chr5:21740714-21741847 REVE	3.916
EV148318	2.648	no similarity	
EE563880	2.646	no similarity	
JCVI_32289	2.646	moderately similar to (329)AT5G54280| Symbols: ATATM2, ATMYOS1, ATM4, ATM2 | ATM2 (ARABIDOPSIS THALIANA MY(	
JCVI_37760	2.643	no original description	
EV142725	2.642	moderately similar to (259)AT4G26870| Symbols: | aspartyl-tRNA synthetase, putative / aspartate--tRNA ligase, putative | chr4:135053	
EE561569	2.642	no similarity	3.758
JCVI_1715	2.642	weakly similar to (196)AT1G54010| Symbols: | myrosinase-associated protein, putative | chr1:20162522-20164415 REVERSE no orig	
EE549460	2.641	no similarity	
JCVI_18525	2.641	weakly similar to (125)AT3G61980| Symbols: | serine protease inhibitor, Kazal-type family protein | chr3:22967319-22967672 REVEI	
JCVI_33576	2.641	moderately similar to (322)AT4G27480| Symbols: | glycosyltransferase family 14 protein / core-2/I-branching enzyme family protein | (	
EV142990	2.635	weakly similar to (119)AT2G40000| Symbols: HSPRO2, ATHSPRO2 | similar to unknown protein [Arabidopsis thaliana] (TAIR:AT3C	
RC_EE560830	2.633	no similarity	
EV160672	2.632	moderately similar to (346)AT4G19170| Symbols: NCED4 | NCED4 (NINE-CIS-EPOXYCAROTENOID DIOXYGENASE 4) | chr4:1	
EV153595	2.632	very weakly similar to (100)AT5G07670| Symbols: | F-box family protein | chr5:2430422-2432066 FORWARD [21484]	
JCVI_30766	2.630	no original description	
EV223305	2.629	moderately similar to (207)AT1G02310| Symbols: | glycosyl hydrolase family protein 5 / cellulase family protein / (1-4)-beta-mannan e	
ES998196	2.629	no similarity	
ES913930	2.628	moderately similar to (285)AT3G25560| Symbols: NIK2 | NIK2 (NSP-INTERACTING KINASE 2); protein kinase | chr3:9280919-928	2.529
EV059775	2.626	no similarity	
EX106682	2.626	no similarity	
JCVI_36799	2.626	no original description	
JCVI_38738	2.626	weakly similar to (185)AT1G20870| Symbols: | similar to unknown protein [Arabidopsis thaliana] (TAIR:AT1G54850.1); similar to un	
RC_EV146547	2.625	no similarity	
ES264508	2.625	no similarity	
EX072529	2.625	moderately similar to (259)AT4G17220| Symbols: ATMAP70-5 | ATMAP70-5 (microtubule-associated proteins 70-5); microtubule bir	
EV000049	2.625	no similarity	
JCVI_29724	2.623	weakly similar to (174)AT1G05000| Symbols: | tyrosine specific protein phosphatase family protein | chr1:1425659-1428392 FORWA	
JCVI_35306	2.623	very weakly similar to (89.4)AT1G18610| Symbols: | kelch repeat-containing protein | chr1:6405771-6408823 FORWARD no original	
EV200364	2.623	no similarity	
EV066988	2.622	no similarity	
EE501957	2.621	weakly similar to (142)AT5G25610| Symbols: RD22 | RD22 (RESPONSIVE TO DESSICATION 22) | chr5:8914501-8916687 REVEI	
JCVI_16483	2.620	moderately similar to (310)AT3G48080| Symbols: | lipase class 3 family protein / disease resistance protein-related | chr3:17764089-17	
JCVI_40900	2.619	highly similar to (572)AT5G64120| Symbols: | peroxidase, putative | chr5:25676777-25678172 REVERSEmoderately similar to (393)	
EV181783	2.616	weakly similar to (187)AT1G12200| Symbols: | flavin-containing monooxygenase family protein / FMO family protein | chr1:4137625	
JCVI_11277	2.616	moderately similar to (379)AT3G23250| Symbols: MYB15, AtY19, AtMYB15 | AtMYB15/AtY19/MYB15 (myb domain protein 15);	

JCVI_32712	2.615	no original description	
EV218651	2.615	moderately similar to (422)AT3G53480| Symbols: PDR9, ATPDR9 | ATPDR9/PDR9 (PLEIOTROPIC DRUG RESISTANCE 9); ATP	
AM390906	2.614	no similarity	
EV109714	2.612	no similarity	
EX118712	2.611	no similarity	
JCVI_8780	2.609	moderately similar to (455)AT5G61820| Symbols: | similar to MtN19-like protein [Pisum sativum] (GB:AAU14999.2); contains InterP	
CD814116	2.609	moderately similar to (350)AT1G43860| Symbols: | transcription factor | chr1:16624684-16626825 REVERSE [13977]	
EV135073	2.609	moderately similar to (240)AT4G28680| Symbols: | tyrosine decarboxylase, putative | chr4:14155254-14158552 FORWARDweakly si	
EV123693	2.608	no similarity	4.427
EE402903	2.605	no similarity	
JCVI_33190	2.605	no original description	
EV105906	2.604	no similarity	
EV175012	2.603	no similarity	2.019
JCVI_18635	2.602	moderately similar to (225)AT1G14420| Symbols: AT59 | AT59 (Arabidopsis homolog of tomato LAT59); lyase/ pectate lyase | chr1:4	-3.239
RC_EE568847	2.602	no similarity	
CD828816	2.601	weakly similar to (123)AT4G30210| Symbols: AR2, ATR2 | ATR2 (ARABIDOPSIS P450 REDUCTASE 2) | chr4:14796906-1480058	
JCVI_15552	2.599	weakly similar to (134)AT5G65490| Symbols: | Identical to SGT1 protein homolog At5g65490 [Arabidopsis Thaliana] (GB:Q9LSM5)	
JCVI_2697	2.599	moderately similar to (263)AT4G23600| Symbols: JR2, CORI3 | CORI3 (CORONATINE INDUCED 1, JASMONIC ACID RESPONS	3.306
EE565377	2.599	no similarity	
JCVI_17709	2.597	moderately similar to (349)AT5G58430| Symbols: ATEXO70B1 | ATEXO70B1 (exocyst subunit EXO70 family protein B1); protein b	
EV204447	2.596	no similarity	
EE567461	2.596	very weakly similar to (83.2)AT5G58375| Symbols: | similar to unknown protein [Arabidopsis thaliana] (TAIR:AT5G14602.1); similar	
JCVI_4347	2.596	weakly similar to (155)AT1G21090| Symbols: | hydroxyproline-rich glycoprotein family protein | chr1:7384843-7386188 FORWARD	
EV225681	2.596	no similarity	
EV111282	2.596	no similarity	
RC_H74834	2.593	no similarity	
ES991417	2.589	no similarity	
EE505236	2.587	no similarity	
JCVI_42567	2.586	very weakly similar to (88.6)TRAC9_MAIZE no original description	
JCVI_11379	2.586	no original description	
EV197309	2.584	no similarity	
AM386194	2.582	no similarity	1.873
EX079964	2.579	weakly similar to (171)AT4G31115| Symbols: | similar to unknown protein [Arabidopsis thaliana] (TAIR:AT5G04440.1); similar to pr	
EX089596	2.579	no similarity	
JCVI_35042	2.579	no original description	
ES967274	2.578	no similarity	
JCVI_29618	2.576	no original description	
JCVI_8926	2.575	very weakly similar to (84.3)AT4G09030| Symbols: ATAGP10, AGP10 | AGP10 (Arabinogalactan protein 10) | chr4:5792246-579262	
JCVI_14001	2.574	no original description	3.670
RC_EE567222	2.573	no similarity	
CV546475	2.573	no similarity	
EE564626	2.572	weakly similar to (200)AT3G28570| Symbols: | AAA-type ATPase family protein | chr3:10711771-10713126 FORWARD [20153] 62	
AT000748	2.572	no similarity	
JCVI_26942	2.570	moderately similar to (279)AT2G46960| Symbols: CYP709B1 | CYP709B1 (cytochrome P450, family 709, subfamily B, polypeptide 1	
EV147827	2.569	no similarity	
JCVI_26015	2.566	moderately similar to (391)AT1G61360| Symbols: | S-locus lectin protein kinase family protein | chr1:22641532-22644639 REVERSE	
JCVI_7955	2.563	moderately similar to (319)AT1G25280| Symbols: AtTLP10 | AtTLP10 (TUBBY LIKE PROTEIN 10) | chr1:8865663-8866595 FORW	
EE504791	2.563	weakly similar to (103)AT5G56670| Symbols: | 40S ribosomal protein S30 (RPS30C) | chr5:22952639-22953207 REVERSE [20159]	
EE552012	2.561	no similarity	2.413
JCVI_6555	2.559	moderately similar to (339)AT5G55530| Symbols: | C2 domain-containing protein | chr5:22511665-22512882 FORWARD no original	
EV209618	2.556	no similarity	
EE567593	2.556	no similarity	
CD820975	2.556	moderately similar to (298)AT1G63090| Symbols: ATPP2-A11 | ATPP2-A11 (Phloem protein 2-A11); carbohydrate binding | chr1:233	
JCVI_26699	2.556	weakly similar to (157)AT3G50240| Symbols: KICP-02 | KICP-02; microtubule motor | chr3:18634362-18639766 REVERSE no origi	
JCVI_10706	2.553	moderately similar to (434)AT5G20710| Symbols: BGAL7 | BGAL7 (beta-galactosidase 7); beta-galactosidase | chr5:7010538-701399	
L38061	2.551	no similarity	
JCVI_5171	2.551	no original description	
EX016624	2.550	no similarity	
EV227113	2.550	no similarity	
EX123468	2.550	very weakly similar to (84.7)AT4G09060| Symbols: | structural molecule | chr4:5797778-5800250 REVERSE [21830]	
DN961174	2.550	moderately similar to (218)AT4G16260| Symbols: | glycosyl hydrolase family 17 protein | chr4:9200310-9201457 REVERSEweakly s	
EV189060	2.549	no similarity	
JCVI_20438	2.548	no original description	
JCVI_12687	2.548	no original description	
JCVI_35269	2.543	no original description	
RC_ES968472	2.541	no similarity	2.562
JCVI_19090	2.540	highly similar to (602)AT3G50270| Symbols: | transferase family protein | chr3:18646908-18648260 FORWARDvery weakly similar t	
JCVI_38132	2.536	no original description	
JCVI_42207	2.536	no original description	
CV546580	2.536	weakly similar to (113)AT5G40010| Symbols: AATP1 | AATP1 (AAA-ATPASE 1); ATP binding / ATPase | chr5:16037446-1603899	
EE567670	2.534	no similarity	1.736
RC_EV010268	2.534	no similarity	3.010
ES937722	2.534	weakly similar to (107)AT4G21050| Symbols: | Dof-type zinc finger domain-containing protein | chr4:11238452-11239084 FORWAR	
JCVI_25351	2.532	no original description	
EX135989	2.530	moderately similar to (209)AT5G07050| Symbols: | nodulin MtN21 family protein | chr5:2191534-2193417 REVERSE [21833] 1 622	
JCVI_8551	2.527	moderately similar to (225)AT5G42380| Symbols: CML39, CML37 | CML37/CML39; calcium ion binding | chr5:16959986-1696054	
ES968335	2.526	no similarity	
JCVI_38998	2.524	moderately similar to (214)AT2G37430| Symbols: | zinc finger (C2H2 type) family protein (ZAT11) | chr2:15713533-15714069 FORW	
EX055496	2.523	moderately similar to (286)AT4G28500| Symbols: ANAC073 | ANAC073 (Arabidopsis NAC domain containing protein 73); transcript	
RC_ES998081	2.522	no similarity	
EE438838	2.521	no similarity	
EE505147	2.519	no similarity	3.882
JCVI_35	2.518	moderately similar to (318)AT1G27730| Symbols: ZAT10, STZ | STZ (SALT TOLERANCE ZINC FINGER); nucleic acid binding / tra	

EV081248	2.514	no similarity	
CD836392	2.512	no similarity	
EE565961	2.511	no similarity	
JCVI_41847	2.511	moderately similar to (360)AT5G40250| Symbols: | zinc finger (C3HC4-type RING finger) family protein | chr5:16103284-16104414	
EE520604	2.511	weakly similar to (107)AT2G36695| Symbols: | unknown protein | chr2:15389781-15390377 FORWARD [20185] 1 412 466	
JCVI_36083	2.510	no original description	
JCVI_22141	2.510	moderately similar to (290)AT5G42240| Symbols: SCPL42 | SCPL42 (serine carboxypeptidase-like 42); serine carboxypeptidase | chr5:	
EV222836	2.510	weakly similar to (112)AT3G15450| Symbols: | similar to unknown protein [Arabidopsis thaliana] (TAIR:AT4G27450.1); similar to un	
EV108550	2.506	very weakly similar to (95.9)AT4G21060| Symbols: | galactosyltransferase family protein | chr4:11240741-11244871 FORWARD [214	
AM395149	2.506	no similarity	
JCVI_23592	2.505	no original description	
ES968374	2.504	no similarity	
EE566355	2.502	no similarity	
CB617607	2.501	no similarity	
EV052696	2.500	no similarity	
EV108926	2.499	no similarity	
EX117651	2.499	no similarity	
JCVI_37499	2.497	weakly similar to (166)AT1G11260| Symbols: STP1 | STP1 (SUGAR TRANSPORTER 1); carbohydrate transmembrane transporter/ su	
JCVI_35120	2.496	no original description	
JCVI_40364	2.496	very weakly similar to (86.7)AT1G04100| Symbols: IAA10 | IAA10 (indoleacetic acid-induced protein 10); transcription factor | chr1:10	
EE411223	2.496	moderately similar to (251)AT2G21410| Symbols: VHA-A2 | VHA-A2 (VACUOLAR PROTON ATPASE A2); ATPase | chr2:916978	
RC_EX039565	2.494	no similarity	
RC_EV086118	2.493	no similarity	
JCVI_16640	2.493	moderately similar to (359)AT1G74000| Symbols: SS3 | SS3 (STRICTOSIDINE SYNTHASE 3) | chr1:27832927-27835062 REVERS	
ES937886	2.493	weakly similar to (115)AT2G34400| Symbols: | pentatricopeptide (PPR) repeat-containing protein | chr2:14523317-14525265 FORWA	
EV148460	2.492	no similarity	
CA992539	2.491	no similarity	
JCVI_28795	2.491	weakly similar to (130)AT3G53500| Symbols: RSZ32 | RSZ32; nucleic acid binding | chr3:19845535-19847485 REVERSE no original	
JCVI_39852	2.491	moderately similar to (238)AT4G28980| Symbols: CDKF;1, CAK1AT | CAK1AT/CDKF;1 (CDK-ACTIVATING KINASE 1AT); cycl	3.288
EV207489	2.489	no similarity	
ES992292	2.489	no similarity	
RC_H07491	2.489	no similarity	
EV128764	2.487	no similarity	
EE411627	2.484	no similarity	
ES956265	2.484	no similarity	
AM394984	2.481	no similarity	
EX101229	2.481	no similarity	
EV170707	2.480	no similarity	
JCVI_11944	2.479	moderately similar to (479)AT3G16430| Symbols: | jacalin lectin family protein | chr3:5581836-5582965 FORWARD no original desc	3.354
EE530249	2.479	weakly similar to (137)AT3G51290| Symbols: | proline-rich family protein | chr3:19050959-19053416 FORWARD [16815]	
ES968293	2.479	no similarity	
JCVI_23109	2.478	highly similar to (830)AT1G14610| Symbols: VALRS, TWN2 | TWN2 (TWIN 2); ATP binding / aminoacyl-tRNA ligase | chr1:50084	
JCVI_10308	2.477	no original description	
EE462905	2.476	no similarity	
EE566253	2.476	no similarity	
RC_ES965015	2.475	no similarity	
EE519672	2.474	weakly similar to (167)AT1G13570| Symbols: | F-box family protein | chr1:4642525-4643927 REVERSE [20185]	
JCVI_10627	2.474	moderately similar to (206)AT3G57860| Symbols: UVI4-LIKE | UVI4-LIKE (UV-B-INSENSITIVE 4-LIKE) | chr3:21437710-214387	
JCVI_39807	2.474	very weakly similar to (89.0)AT1G61310| Symbols: | disease resistance protein (CC-NBS-LRR class), putative | chr1:22616831-226196	
JCVI_6755	2.472	highly similar to (610)AT1G19640| Symbols: JMT | JMT (JASMONIC ACID CARBOXYL METHYLTRANSFERASE); jasmonate O	
EE567417	2.470	no similarity	
EE567519	2.467	no similarity	
EV110425	2.467	no similarity	
ES923362	2.465	no similarity	
JCVI_24975	2.465	no original description	-1.978
JCVI_18683	2.465	no original description	
JCVI_23743	2.465	no original description	
JCVI_3775	2.465	moderately similar to (384)AT2G02790| Symbols: IQD29 | IQD29 (IQ-domain 29); calmodulin binding | chr2:788707-790945 FORWA	2.102
JCVI_25743	2.465	weakly similar to (119)AT5G23610| Symbols: | similar to SWI1 (SWITCH1), phospholipase C [Arabidopsis thaliana] (TAIR:AT5G51	
EV218262	2.464	no similarity	
CV432107	2.462	very weakly similar to (100)AT4G14130| Symbols: XTR7 | XTR7 (XYLOGLUCAN ENDOTRANSGLYCOSYLASE 7); hydrolase, ac	4.266
EX136112	2.461	no similarity	
JCVI_21020	2.461	weakly similar to (141)AT2G40000| Symbols: HSPRO2, ATHSPRO2 | similar to unknown protein [Arabidopsis thaliana] (TAIR:AT3C	
DY003910	2.460	no similarity	
EV127421	2.460	weakly similar to (115)AT1G74930| Symbols: ORA47 | ORA47; DNA binding / transcription factor | chr1:28147900-28148487 FORW	3.516
BG543778	2.459	weakly similar to (140)NIA1_BRANA [8791]	
EV041604	2.456	no similarity	
EV109384	2.455	no similarity	
JCVI_3778	2.454	very weakly similar to (87.0)AT2G37980| Symbols: | similar to unknown protein [Arabidopsis thaliana] (TAIR:AT5G01100.1); similar	
JCVI_30202	2.454	weakly similar to (118)AT5G02490| Symbols: | heat shock cognate 70 kDa protein 2 (HSC70-2) (HSP70-2) | chr5:550294-552563 RE	
RC_ES969166	2.454	no similarity	
ES966630	2.454	no similarity	
JCVI_34162	2.453	highly similar to (551)AT1G05500| Symbols: SYTE, ATSYTE, NTMC2TYPE2.1, NTMC2T2.1 | ATSYTE/NTMC2T2.1/NTMC2TYF	
EV223131	2.453	no similarity	
EE446048	2.450	weakly similar to (130)AT5G36290| Symbols: | similar to unknown protein [Arabidopsis thaliana] (TAIR:AT1G25520.1); similar to un	1.841
AM395058	2.448	no similarity	
ES991160	2.448	no similarity	
CX189649	2.448	moderately similar to (211)AT5G09920| Symbols: ATRPB15.9, RPB15.9.9, RPB15.9 | RPB15.9 (RNA polymerase II 15.9) | chr5:309	
JCVI_17461	2.446	no original description	
JCVI_21988	2.445	moderately similar to (332)AT3G60830| Symbols: ARP7, ATARP7 | ATARP7 (ACTIN-RELATED PROTEIN 7); structural constituen	
JCVI_12908	2.444	weakly similar to (176)ATMG00310| Symbols: ORF154 | hypothetical protein | chrM:90883-91347 REVERSE no original description	
EV098664	2.444	no similarity	
JCVI_26516	2.441	weakly similar to (196)AT2G17440| Symbols: | leucine-rich repeat family protein | chr2:7578413-7580488 FORWARD no original des	

ES932617	2.439	no similarity	
JCVI_42191	2.438	weakly similar to (112)AT5G23570| Symbols: SGS3 | SGS3 (SUPPRESSOR OF GENE SILENCING 3) | chr5:7943624-7945877 FOR	
EE505116	2.438	no similarity	
JCVI_17690	2.437	weakly similar to (164)AT4G37650| Symbols: SGR7, SHR | SHR (SHORT ROOT); transcription factor | chr4:17691865-17693460 FC	
JCVI_38436	2.436	moderately similar to (314)AT5G11350| Symbols: | endonuclease/exonuclease/phosphatase family protein | chr5:3621584-3625413 FC	
ES968067	2.436	no similarity	
JCVI_32393	2.435	moderately similar to (259)AT2G45760| Symbols: BAL, BAP2 | BAP2 (BON ASSOCIATION PROTEIN 2) | chr2:18854199-1885482	
EV174995	2.435	no similarity	5.155
EX115617	2.434	no similarity	2.240
EV106869	2.434	no similarity	
JCVI_12231	2.434	moderately similar to (334)AT4G20130| Symbols: PTAC14 | PTAC14 (PLASTID TRANSCRIPTIONALLY ACTIVE14) | chr4:10878	
EE546376	2.434	weakly similar to (153)AT1G51990| Symbols: | O-methyltransferase family 2 protein | chr1:19334618-19336336 FORWARD [20128]	
EX088769	2.434	weakly similar to (180)AT4G26830| Symbols: | hydrolase, hydrolyzing O-glycosyl compounds | chr4:13494732-13496493 REVERSE	
JCVI_35155	2.432	no original description	
EX092993	2.432	no similarity	
RC_CD843352	2.431	no similarity	
EX126068	2.429	weakly similar to (144)AT2G29670| Symbols: | binding | chr2:12689386-12691822 REVERSE [21831] 1 664 683	
EX131397	2.429	no similarity	
EV138028	2.425	no similarity	
JCVI_40297	2.424	moderately similar to (486)AT2G44830| Symbols: | protein kinase, putative | chr2:18497473-18499854 FORWARDweakly similar to (	
JCVI_40943	2.423	moderately similar to (275)AT3G17920| Symbols: | similar to leucine-rich repeat family protein [Arabidopsis thaliana] (TAIR:AT1G48	
AM058773	2.421	weakly similar to (124)AT2G43190| Symbols: | ribonuclease P family protein | chr2:17963297-17964910 FORWARD [17712]	
EV088129	2.420	no similarity	
EX045444	2.419	weakly similar to (157)AT3G21580| Symbols: | cobalt ion transmembrane transporter | chr3:7602015-7603727 REVERSE [21811] 1 5	
JCVI_21564	2.418	no original description	
JCVI_32644	2.418	moderately similar to (345)AT3G28340| Symbols: GATL10 | GATL10 (Galacturonosyltransferase-like 10); polygalacturonate 4-alpha-g	
EX066516	2.417	no similarity	
JCVI_21982	2.417	moderately similar to (313)AT2G41960| Symbols: | similar to unknown protein [Arabidopsis thaliana] (TAIR:AT3G58050.1); similar t	
JCVI_30655	2.417	moderately similar to (294)AT2G44560| Symbols: ATGH9B11 | ATGH9B11 (ARABIDOPSIS THALIANA GLYCOSYL HYDROLA	
JCVI_33032	2.416	very weakly similar to (90.5)AT1G05805| Symbols: | basic helix-loop-helix (bHLH) family protein | chr1:1744842-1747426 FORWAR	
EV191476	2.416	weakly similar to (121)AT1G77760| Symbols: GNR1, NR1, NIA1 | NIA1 (NITRATE REDUCTASE 1) | chr1:29240899-29244261 RE	1.120
RC_EE484085	2.415	no similarity	
EV107135	2.415	no similarity	
EX038291	2.415	no similarity	
JCVI_11680	2.415	no original description	
EE455949	2.415	very weakly similar to (98.6)AT2G20340| Symbols: | tyrosine decarboxylase, putative | chr2:8786885-8789571 FORWARDvery weakl	
EE533202	2.415	weakly similar to (157)AT3G59200| Symbols: | F-box family protein | chr3:21898461-21900191 FORWARD [20175]	
JCVI_16660	2.414	moderately similar to (213)AT5G39420| Symbols: CDC2CAT | CDC2CAT (ARABIDOPSIS THALIANA CDC2C); kinase | chr5:1578	
RC_EE438795	2.413	no similarity	
EX094012	2.413	moderately similar to (360)AT5G47100| Symbols: CBL9 | CBL9 (Calcineurin B-like protein 9); calcium ion binding | chr5:19147123-1	
EE421886	2.413	no similarity	
CV973866	2.413	no similarity	
EE563018	2.411	no similarity	
JCVI_1641	2.410	weakly similar to (129)AT4G28610| Symbols: ATPHR1, PHR1 | PHR1 (PHOSPHATE STARVATION RESPONSE 1); transcription f	
EV060076	2.410	very weakly similar to (83.6)AT2G23220| Symbols: CYP81D6 | CYP81D6 (cytochrome P450, family 81, subfamily D, polypeptide 6);	
ES978243	2.409	no similarity	
CX193134	2.409	no similarity	
CN730171	2.409	no similarity	
RC_EE565639	2.408	no similarity	
JCVI_40407	2.407	highly similar to (523)AT5G57560| Symbols: XTH22, TCH4 | TCH4 (TOUCH 4); hydrolase, acting on glycosyl bonds / xyloglucan:xy	
EV190703	2.406	no similarity	
JCVI_14028	2.406	highly similar to (509)AT2G28780| Symbols: | similar to unknown protein [Arabidopsis thaliana] (TAIR:AT3G09450.1); similar to unr	
EV104190	2.405	moderately similar to (266)AT4G29010| Symbols: AIM1 | AIM1 (ABNORMAL INFLORESCENCE MERISTEM); enoyl-CoA hydrat	
JCVI_41986	2.405	no original description	
JCVI_35443	2.405	no original description	
JCVI_39843	2.404	no original description	
EV220439	2.404	no similarity	
JCVI_20037	2.404	moderately similar to (262)AT3G47940| Symbols: | DNAJ heat shock protein, putative | chr3:17699217-17700387 REVERSE no origi	
EE525061	2.403	moderately similar to (230)AT3G59490| Symbols: | similar to unnamed protein product [Vitis vinifera] (GB:CAO64819.1) | chr3:2199	
EV205088	2.402	no similarity	
JCVI_23616	2.401	no original description	
JCVI_38904	2.400	moderately similar to (219)AT5G07500| Symbols: PEI1 | PEI1; nucleic acid binding / transcription factor | chr5:2372717-2373454 FOF	
CX278854	2.400	moderately similar to (252)AT1G52030| Symbols: MBP1.2, F-ATMBP, MBP2 | MBP2 (MYROSINASE-BINDING PROTEIN 2) | chr	
JCVI_29265	2.400	weakly similar to (129)AT5G05340| Symbols: | peroxidase, putative | chr5:1579143-1580820 REVERSEweakly similar to (126)PERI	
EX117947	2.398	no similarity	
JCVI_31778	2.397	weakly similar to (110)AT5G66600| Symbols: | similar to unknown protein [Arabidopsis thaliana] (TAIR:AT2G23700.1); similar to un	
JCVI_22917	2.396	no original description	
BG543536	2.395	no similarity	
EE481088	2.390	no similarity	2.067
ES968160	2.389	no similarity	
JCVI_41074	2.389	no original description	
JCVI_11033	2.389	no original description	
EE569252	2.389	no similarity	3.399
JCVI_26891	2.388	moderately similar to (427)AT1G12640| Symbols: | membrane bound O-acyl transferase (MBOAT) family protein | chr1:4303584-430	
JCVI_41728	2.387	no original description	
EV091863	2.386	no similarity	
JCVI_27789	2.384	no original description	2.872
EV131203	2.384	no similarity	
JCVI_14004	2.384	moderately similar to (203)AT3G61680| Symbols: | lipase class 3 family protein | chr3:22835605-22837901 FORWARD no original de	
JCVI_32588	2.384	moderately similar to (471)AT5G49520| Symbols: ATWRKY48, WRKY48 | WRKY48 (WRKY DNA-binding protein 48); transcriptic	
JCVI_33886	2.384	weakly similar to (147)AT3G05490| Symbols: RALFL22 | RALFL22 (RALF-LIKE 22) | chr3:1591387-1591746 FORWARD no origin	
JCVI_19223	2.383	moderately similar to (268)AT4G04750| Symbols: | carbohydrate transmembrane transporter/ sugar:hydrogen ion symporter | chr4:241	
EX102546	2.383	weakly similar to (200)AT4G01670| Symbols: | similar to unknown protein [Arabidopsis thaliana] (TAIR:AT3G62070.1); similar to un	

JCVI_19679	2.383	moderately similar to (419)AT4G05050| Symbols: UBQ11 | UBQ11 (UBIQUITIN 11) | chr4:2588269-2588958 REVERSEweakly sim	
AM395435	2.382	weakly similar to (115)RRPO_OENBE [20346]	
JCVI_28305	2.381	no original description	
EE558622	2.381	no similarity	2.530
EE556619	2.379	no similarity	
JCVI_33213	2.379	no original description	
JCVI_4463	2.378	weakly similar to (138)AT5G01520| Symbols: | zinc finger (C3HC4-type RING finger) family protein | chr5:206796-208398 FORWA	
CX270301	2.378	weakly similar to (167)AT2G24700| Symbols: | transcriptional factor B3 family protein | chr2:10520128-10522367 REVERSE [16815	
JCVI_2589	2.377	no original description	2.662
EE566497	2.376	no similarity	
EV190711	2.375	very weakly similar to (89.7)AT1G29724| Symbols: | protein binding | chr1:10397726-10400438 REVERSE [21489] 34 802 802	
EV150955	2.374	no similarity	
EX065809	2.373	very weakly similar to (85.5)AT5G02110| Symbols: CYCD7;1, CYCD7 | CYCD7/CYCD7;1 (CYCLIN D7;1); cyclin-dependent protei	
EV204290	2.373	no similarity	
JCVI_23159	2.372	weakly similar to (157)AT4G18620| Symbols: | similar to Bet v I allergen family protein [Arabidopsis thaliana] (TAIR:AT5G45870.1);	
EV131705	2.371	no similarity	3.782
ES944040	2.370	no similarity	
EV079131	2.367	no similarity	
JCVI_32793	2.367	moderately similar to (218)AT3G62680| Symbols: ATPRP3, PRP3 | PRP3 (PROLINE-RICH PROTEIN 3); structural constituent of ce	
EV215803	2.365	weakly similar to (157)AT2G34430| Symbols: LHCB1.4, LHB1B1 | LHB1B1 (Photosystem II light harvesting complex gene 1.4); chlo	
EV166697	2.364	no similarity	
JCVI_7324	2.363	no original description	
JCVI_34932	2.363	very weakly similar to (98.6)AT1G01260| Symbols: | basic helix-loop-helix (bHLH) family protein | chr1:109595-111367 FORWARD	1.697
JCVI_10086	2.363	moderately similar to (256)AT3G08720| Symbols: ATS6K2, S6K2, ATPK2, ATPK19 | ATPK19 (ARABIDOPSIS THALIANA PROT	
EV163212	2.361	very weakly similar to (99.4)AT5G47620| Symbols: | heterogeneous nuclear ribonucleoprotein, putative / hnRNP, putative | chr5:19319	
RC_H07215	2.361	no similarity	
EV127009	2.361	no similarity	
JCVI_1692	2.361	very weakly similar to (100)AT1G62780| Symbols: | similar to hypothetical protein [Vitis vinifera] (GB:CAN83165.1) | chr1:2325301	
ES997948	2.361	weakly similar to (167)AT1G53710| Symbols: | similar to unnamed protein product [Vitis vinifera] (GB:CAO68485.1); contains InterP	
EE392422	2.359	no similarity	
JCVI_16801	2.357	moderately similar to (216)AT4G32600| Symbols: | zinc finger (C3HC4-type RING finger) family protein | chr4:15724016-15725743	
RC_EE558820	2.355	no similarity	
EE568965	2.355	no similarity	
EV137343	2.354	no similarity	
EV187017	2.353	no similarity	
CD811951	2.352	no similarity	
JCVI_4844	2.351	no original description	
JCVI_39097	2.351	no original description	
JCVI_21471	2.350	weakly similar to (104)AT1G69170| Symbols: | squamosa promoter-binding protein-like 6 (SPL6) | chr1:26009289-26010704 FORW	
H07447	2.349	no similarity	
JCVI_6023	2.349	moderately similar to (388)AT3G54150| Symbols: | embryo-abundant protein-related | chr3:20061780-20063720 REVERSE no origin	
JCVI_30610	2.348	weakly similar to (189)AT2G40150| Symbols: | similar to unknown protein [Arabidopsis thaliana] (TAIR:AT3G55990.1); similar to un	
EE468068	2.347	no similarity	
EV098616	2.347	weakly similar to (116)AT3G55260| Symbols: HEXO1, ATHEX2 | ATHEX2/HEXO1 (BETA-HEXOSAMINIDASE 1); beta-N-acetyl	
JCVI_38231	2.345	no original description	
JCVI_37463	2.343	very weakly similar to (98.2)AT5G53890| Symbols: | leucine-rich repeat transmembrane protein kinase, putative | chr5:21894461-2189	2.878
JCVI_15939	2.342	highly similar to (608)AT5G14700| Symbols: | cinnamoyl-CoA reductase-related | chr5:4740505-4743330 REVERSEvery weakly simi	
EE525908	2.341	no similarity	
JCVI_8746	2.339	no original description	
DN964770	2.339	moderately similar to (228)AT2G35930| Symbols: | U-box domain-containing protein | chr2:15090180-15091415 REVERSE [17359]	
EE478859	2.339	weakly similar to (106)AT1G50510| Symbols: | indigoidine synthase A family protein | chr1:18720246-18722728 FORWARD [20132	
EV055041	2.339	no similarity	
JCVI_16092	2.339	moderately similar to (236)AT5G46830| Symbols: | basic helix-loop-helix (bHLH) family protein | chr5:19019946-19021481 FORWA	0.694
ES901052	2.338	weakly similar to (193)AT5G18070| Symbols: | DRT101 (DNA-DAMAGE-REPAIR/TOLERATION 101); intramolecular transferase,	
RC_EE568158	2.336	no similarity	
EV127579	2.335	moderately similar to (207)AT5G18120| Symbols: ATAPRL7 | ATAPRL7 (APR-LIKE 7) | chr5:5991387-5993698 FORWARD [2148	
CD817457	2.334	no similarity	
JCVI_37219	2.334	no original description	
EV106022	2.333	no similarity	
ES904917	2.333	moderately similar to (203)AT1G30270| Symbols: SnRK3.23, ATCIPK23, LKS1, CIPK23 | CIPK23 (CBL-INTERACTING PROTEI	
CV973980	2.333	no similarity	
JCVI_5055	2.332	moderately similar to (394)AT1G69930| Symbols: ATGSTU11 | ATGSTU11 (Arabidopsis thaliana Glutathione S-transferase (class tau	
JCVI_17862	2.332	moderately similar to (403)AT5G13200| Symbols: | GRAM domain-containing protein / ABA-responsive protein-related | chr5:420708	
EV223079	2.331	no similarity	2.511
JCVI_9907	2.330	moderately similar to (286)AT1G76650| Symbols: CML38 | CML38 | chr1:28771803-28772336 REVERSEvery weakly similar to (82.	
JCVI_26200	2.329	moderately similar to (385)AT2G03760| Symbols: RAR047, ST | ST (steroid sulfotransferase); sulfotransferase | chr2:1149472-115045	
JCVI_24700	2.328	no original description	
EV165777	2.326	moderately similar to (201)AT4G20200| Symbols: | terpene synthase/cyclase family protein | chr4:10908682-10911161 REVERSEvery	
JCVI_4224	2.325	very weakly similar to (96.3)AT2G13770| Symbols: | similar to ribosomal protein-like [Oryza sativa (japonica cultivar-group)] (GB:BA	
CX191063	2.324	no similarity	
EV039389	2.324	moderately similar to (249)AT1G27080| Symbols: | transporter | chr1:9400651-9403776 FORWARD [21442]	
EE417789	2.323	moderately similar to (232)AT5G07200| Symbols: GA20OX3, ATGA20OX3 | YAP169 (GIBBERELLIN 20 OXIDASE 3); gibberellin	
JCVI_5855	2.322	moderately similar to (252)AT1G27190| Symbols: | leucine-rich repeat transmembrane protein kinase, putative | chr1:9446910-944871	
JCVI_24153	2.322	no original description	-2.559
RC_ES947980	2.320	no similarity	
ES962072	2.320	no similarity	
JCVI_39122	2.320	no original description	
JCVI_25760	2.319	moderately similar to (475)AT4G24570| Symbols: | mitochondrial substrate carrier family protein | chr4:12686556-12687497 FORWA	2.173
EV221283	2.319	no similarity	
EE464425	2.318	weakly similar to (130)AT1G68790| Symbols: LINC3 | LINC3 (LITTLE NUCLEI3) | chr1:25838595-25842820 REVERSE [20171]	4.329
EE472183	2.317	no similarity	
EV100262	2.314	no similarity	
EV136479	2.312	no similarity	2.671

EE559838	2.310	no similarity	
EX130622	2.310	moderately similar to (210)AT2G01300| Symbols: | similar to unknown protein [Arabidopsis thaliana] (TAIR:AT1G15010.1); similar t	2.169
EE545610	2.310	weakly similar to (138)AT5G46930| Symbols: | invertase/pectin methylesterase inhibitor family protein | chr5:19073882-19074418 RE	
EV104577	2.310	no similarity	
JCVI_32877	2.310	no original description	
EV217196	2.310	moderately similar to (270)AT2G36880| Symbols: MAT3 | MAT3 (METHIONINE ADENOSYLTRANSFERASE 3) | chr2:15486800-	
JCVI_23583	2.310	no original description	
JCVI_16158	2.309	no original description	
JCVI_8360	2.308	moderately similar to (367)AT2G32240| Symbols: | similar to unknown protein [Arabidopsis thaliana] (TAIR:AT1G05320.1); similar t	
JCVI_8722	2.306	moderately similar to (295)AT2G34500| Symbols: CYP710A1 | CYP710A1 (cytochrome P450, family 710, subfamily A, polypeptide	
JCVI_4114	2.305	moderately similar to (387)AT2G22300| Symbols: | ethylene-responsive calmodulin-binding protein, putative (SR1) | chr2:9478679-94	
JCVI_20638	2.305	weakly similar to (145)AT2G20835| Symbols: | similar to unknown protein [Arabidopsis thaliana] (TAIR:AT3G15534.1); similar to un	
EV142911	2.304	no similarity	
JCVI_6893	2.304	weakly similar to (151)AT3G50800| Symbols: | similar to unknown protein [Arabidopsis thaliana] (TAIR:AT5G66580.1); similar to un	
JCVI_17037	2.304	weakly similar to (166)AT1G78080| Symbols: RAP2.4 | RAP2.4 (related to AP2 4); DNA binding / transcription factor | chr1:2936968	
EV004192	2.303	no similarity	
JCVI_2174	2.302	no original description	
AM391923	2.302	very weakly similar to (100)AT5G58000| Symbols: | CPL4 (C-TERMINAL DOMAIN PHOSPHATASE-LIKE 4) | chr5:23494642-23	2.689
L38213	2.301	no similarity	
EV223686	2.301	no similarity	
EV087750	2.300	no similarity	
EV121875	2.300	no similarity	
EV000534	2.299	no similarity	
EX025522	2.299	very weakly similar to (97.1)AT3G25720| Symbols: | similar to unknown protein [Arabidopsis thaliana] (TAIR:AT4G10613.1); similar	1.916
JCVI_29006	2.299	moderately similar to (236)AT2G03980| Symbols: | GDSL-motif lipase/hydrolase family protein | chr2:1260904-1262405 FORWARD	
JCVI_1841	2.298	moderately similar to (223)AT4G37260| Symbols: MYB73, AtMYB73 | AtMYB73/MYB73 (myb domain protein 73); DNA binding /	
EV015332	2.297	no similarity	
JCVI_530	2.296	highly similar to (611)AT2G26200| Symbols: | similar to unknown protein [Arabidopsis thaliana] (TAIR:AT1G54650.1); similar to unk	
JCVI_29421	2.296	very weakly similar to (89.4)AT1G35435| Symbols: | Encodes a defensin-like (DEFL) family protein. | chr1:13032784-13033225 REV	
BG543634	2.296	weakly similar to (127)AT3G45850| Symbols: | kinesin motor protein-related | chr3:16866799-16871935 REVERSE [8791] 1 444 458	
JCVI_14437	2.296	moderately similar to (248)AT4G20140| Symbols: GSO1 | leucine-rich repeat transmembrane protein kinase, putative | chr4:10884230-	
EV141857	2.296	weakly similar to (160)AT5G60170| Symbols: | RNA binding | chr5:24245404-24249620 FORWARD [21482] 1 600 736	
JCVI_36962	2.293	weakly similar to (110)AT3G01250| Symbols: | unknown protein | chr3:79411-79905 FORWARD no original description	
EE546514	2.292	no similarity	
JCVI_9412	2.291	highly similar to (551)AT4G14130| Symbols: XTR7 | XTR7 (XYLOGLUCAN ENDOTRANSGLYCOSYLASE 7); hydrolase, acting	
JCVI_31006	2.291	no original description	
EV152303	2.291	no similarity	
JCVI_22385	2.288	moderately similar to (303)AT2G41640| Symbols: | similar to unknown protein [Arabidopsis thaliana] (TAIR:AT3G57380.1); similar t	
JCVI_13338	2.288	moderately similar to (223)AT2G37500| Symbols: | arginine biosynthesis protein ArgJ family | chr2:15746982-15749508 REVERSE n	
EE465917	2.287	no similarity	
JCVI_9514	2.286	moderately similar to (334)AT1G04770| Symbols: | male sterility MS5 family protein | chr1:1336563-1337766 REVERSE no original	
JCVI_7505	2.286	moderately similar to (355)AT1G18980| Symbols: | germin-like protein, putative | chr1:6557355-6558017 REVERSEmoderately simil	
RC_EE402513	2.285	no similarity	
EV053773	2.285	weakly similar to (104)AT2G13770| Symbols: | similar to ribosomal protein-like [Oryza sativa (japonica cultivar-group)] (GB:BAD37	
JCVI_3148	2.284	moderately similar to (240)AT1G06280| Symbols: LBD2 | LBD2 (LOB DOMAIN-CONTAINING PROTEIN 2) | chr1:1920326-1920	
EE527347	2.284	no similarity	
CV973934	2.284	no similarity	2.165
EX035886	2.282	no similarity	
EG019381	2.282	weakly similar to (173)AT3G11830| Symbols: | chaperonin, putative | chr3:3732740-3736162 FORWARD [20440] 1 459 475	
ES914598	2.282	no similarity	
ES964741	2.282	no similarity	
RC_CD823283	2.280	no similarity	
JCVI_39875	2.280	no original description	
EE535482	2.280	moderately similar to (317)AT1G04150| Symbols: | C2 domain-containing protein | chr1:1081207-1084245 REVERSE [20150]	
JCVI_31263	2.280	weakly similar to (117)AT1G10000| Symbols: | nucleic acid binding / ribonuclease H | chr1:3263879-3264790 REVERSE no original	
EE559930	2.280	weakly similar to (102)AT1G27120| Symbols: | galactosyltransferase family protein | chr1:9421376-9423897 FORWARD [20153] 21	
EV171267	2.279	very weakly similar to (95.5)AT2G45200| Symbols: ATGOS12, GOS12 | GOS12 (GOLGI SNARE 12); SNARE binding | chr2:186447	
ES962911	2.278	no similarity	
RC_JCVI_29195	2.277	no original description	
EE556745	2.277	no similarity	
EE504784	2.276	no similarity	
RC_ES965361	2.276	no similarity	
EE410680	2.276	no similarity	
RC_EX016639	2.276	no similarity	
EV141922	2.275	no similarity	
EV209165	2.274	very weakly similar to (95.9)ATCG00740| Symbols: RPOA | RNA polymerase alpha subunit | chrC:77901-78890 REVERSEvery weakl	2.894
JCVI_33562	2.273	no original description	1.624
EE535370	2.273	very weakly similar to (100)AT1G06420| Symbols: | unknown protein | chr1:1958986-1959651 FORWARD [20150]	
JCVI_38766	2.272	highly similar to (932)AT2G21540| Symbols: | SEC14 cytosolic factor, putative / phosphoglyceride transfer protein, putative | chr2:922	
ES956956	2.270	no similarity	
JCVI_15271	2.269	moderately similar to (255)AT3G47580| Symbols: | leucine-rich repeat transmembrane protein kinase, putative | chr3:17543672-17546	
EE541187	2.269	no similarity	
EV121879	2.269	no similarity	
EV108573	2.269	no similarity	
EV146452	2.268	no similarity	
JCVI_30459	2.263	no original description	
EV223098	2.263	no similarity	
EE392342	2.263	very weakly similar to (81.3)AT3G51190| Symbols: | 60S ribosomal protein L8 (RPL8B) | chr3:19027585-19028526 REVERSEvery w	
EE566653	2.263	no similarity	
JCVI_17725	2.263	no original description	
EE558644	2.262	no similarity	
JCVI_15099	2.262	no original description	
EE527375	2.259	no similarity	

JCVI_15636	2.259	moderately similar to (255)AT3G09910| Symbols: AtRab18C, AtRABC2b | AtRABC2b/AtRab18C (Arabidopsis Rab GTPase homolog	
JCVI_35641	2.258	no original description	
H06422	2.257	no similarity	
EX066286	2.257	moderately similar to (221)AT2G19050| Symbols: | GDSL-motif lipase/hydrolase family protein | chr2:8260498-8262616 FORWARD	
JCVI_9810	2.257	moderately similar to (477)AT3G29670| Symbols: | transferase family protein | chr3:11530431-11531786 FORWARD no original desc	
H07256	2.256	no similarity	
EE433603	2.256	weakly similar to (107)AT1G44830| Symbols: | AP2 domain-containing transcription factor TINY, putative | chr1:16936232-16936867	
CD841406	2.255	weakly similar to (150)AT5G61610| Symbols: | glycine-rich protein / oleosin | chr5:24787325-24788139 FORWARD [13982]	
ES966738	2.252	no similarity	
EV219168	2.251	no similarity	
EV021400	2.251	weakly similar to (189)AT2G18600| Symbols: | RUB1-conjugating enzyme, putative | chr2:8080419-8081647 REVERSE [21441]	2.749
JCVI_26031	2.248	weakly similar to (116)AT4G37610| Symbols: BT5 | BT5 (BTB and TAZ domain protein 5); protein binding / transcription regulator | c	3.630
ES900145	2.247	no similarity	2.783
AB012650	2.246	no similarity	
EE450743	2.246	no similarity	
JCVI_37168	2.246	no original description	
ES996255	2.246	moderately similar to (259)AT5G60600| Symbols: ISPG, CSB3, CLB4, GcpE | GcpE (CHLOROPLAST BIOGENESIS 4) | chr5:24376	
EV194609	2.246	weakly similar to (192)AT4G28820| Symbols: | zinc finger (HIT type) family protein | chr4:14230821-14232293 REVERSE [21489]	
EX073155	2.245	no similarity	1.250
EV126887	2.245	no similarity	4.009
EE442803	2.244	no similarity	
AM395139	2.242	no similarity	
ES993840	2.240	no similarity	
JCVI_1399	2.239	moderately similar to (244)AT5G11270| Symbols: OCP3 | OCP3 (OVEREXPRESSOR OF CATIONIC PEROXIDASE 3) | chr5:35955	
EV112898	2.239	no similarity	
CK991425	2.237	no similarity	
JCVI_24047	2.237	moderately similar to (371)AT1G07630| Symbols: PLL5 | PLL5 (POL-like 5); protein serine/threonine phosphatase | chr1:2349186-235	
EV177226	2.237	no similarity	
JCVI_39766	2.237	no original description	
EV098633	2.235	no similarity	1.798
EV186706	2.233	weakly similar to (128)AT2G26600| Symbols: | glycosyl hydrolase family 17 protein | chr2:11324292-11325293 FORWARD [21488]	1.281
JCVI_15437	2.233	moderately similar to (456)AT2G43770| Symbols: | transducin family protein / WD-40 repeat family protein | chr2:18141349-1814238	2.040
EV164663	2.232	moderately similar to (202)AT2G38370| Symbols: | similar to unknown protein [Arabidopsis thaliana] (TAIR:AT3G51720.1); similar t	
CX189791	2.231	moderately similar to (259)AT2G13570| Symbols: | CCAAT-box binding transcription factor, putative | chr2:5662924-5663571 REVE	
EE402854	2.230	moderately similar to (210)AT5G16460| Symbols: | similar to unknown protein [Arabidopsis thaliana] (TAIR:AT1G29760.1); similar t	4.297
JCVI_32857	2.228	moderately similar to (258)AT3G28980| Symbols: | similar to unknown protein [Arabidopsis thaliana] (TAIR:AT3G28810.1); contains	
EV165339	2.227	no similarity	
EX137311	2.225	moderately similar to (323)AT1G14370| Symbols: APK2A | APK2A (PROTEIN KINASE 2A); kinase | chr1:4915854-4917954 FORW	
CV545055	2.225	weakly similar to (184)AT3G49670| Symbols: BAM2 | BAM2 (big apical meristem 2); ATP binding / protein serine/threonine kinase | c	2.155
JCVI_16916	2.225	moderately similar to (270)AT1G73540| Symbols: ATNUDT21 | ATNUDT21 (Arabidopsis thaliana Nudix hydrolase homolog 21); hyd	
JCVI_38126	2.224	very weakly similar to (91.3)AT4G18570| Symbols: | proline-rich family protein | chr4:10231450-10234545 FORWARD no original de	
JCVI_12318	2.223	highly similar to (550)AT2G03800| Symbols: GEK1 | GEK1 (GEKO1) | chr2:1156779-1158692 FORWARD no original description	4.113
EV135372	2.223	weakly similar to (122)AT3G60750| Symbols: | transketolase, putative | chr3:22464979-22467799 FORWARDweakly similar to (123)	
EV118798	2.222	no similarity	
EV161446	2.222	no similarity	
EV210107	2.222	moderately similar to (294)AT1G44760| Symbols: | universal stress protein (USP) family protein | chr1:16899334-16900867 REVERS	
EV109671	2.222	very weakly similar to (89.0)AT4G15530| Symbols: PPDK | PPDK (PYRUVATE ORTHOPHOSPHATE DIKINASE) | chr4:8864826-8	
EE421652	2.221	no similarity	
JCVI_34463	2.220	no original description	4.589
EV131594	2.219	no similarity	
JCVI_21172	2.218	no original description	
JCVI_40529	2.218	no original description	
JCVI_15094	2.217	weakly similar to (101)AT2G40900| Symbols: | nodulin MtN21 family protein | chr2:17070474-17072592 REVERSE no original desc	
EE563168	2.217	no similarity	
EV087239	2.215	no similarity	3.316
JCVI_2696	2.215	moderately similar to (260)AT1G17470| Symbols: ATDRG, ATDRG1 | ATDRG1 (ARABIDOPSIS THALIANA DEVELOPMENTAL	
JCVI_14811	2.214	no original description	2.431
JCVI_34427	2.211	moderately similar to (343)AT1G73020| Symbols: | similar to unnamed protein product [Vitis vinifera] (GB:CAO45737.1); similar to h	
EV108841	2.211	no similarity	
JCVI_23960	2.211	no original description	
EE548612	2.211	no similarity	2.652
JCVI_14606	2.208	moderately similar to (448)AT2G37700| Symbols: | similar to CER1 protein, putative [Arabidopsis thaliana] (TAIR:AT1G02190.1); si	
EE555323	2.208	no similarity	
EE549729	2.208	no similarity	
EV163200	2.208	moderately similar to (307)AT2G40850| Symbols: | phosphatidylinositol 3- and 4-kinase family protein | chr2:17058653-17060338 FO	
JCVI_21273	2.207	weakly similar to (189)AT4G34410| Symbols: | AP2 domain-containing transcription factor, putative | chr4:16451996-16452802 FORW	4.104
EV071596	2.207	no similarity	
JCVI_23351	2.204	moderately similar to (333)AT1G60980| Symbols: ATGA20OX4 | ATGA20OX4 (GIBBERELLIN 20-OXIDASE 4); gibberellin 20-ox	
JCVI_2286	2.204	moderately similar to (217)AT4G27170| Symbols: | 2S seed storage protein 4 / 2S albumin storage protein / NWMU2-2S albumin 4 | cl	
EV205494	2.204	no similarity	
JCVI_37300	2.203	weakly similar to (198)AT4G19230| Symbols: CYP707A1 | CYP707A1 (cytochrome P450, family 707, subfamily A, polypeptide 1); o	
JCVI_31904	2.203	no original description	
JCVI_38051	2.201	moderately similar to (234)AT5G41330| Symbols: | potassium channel tetramerisation domain-containing protein | chr5:16553680-165	
JCVI_24324	2.200	no original description	
JCVI_38962	2.200	weakly similar to (166)AT3G02080| Symbols: | 40S ribosomal protein S19 (RPS19A) | chr3:364145-365168 REVERSEweakly similar	
EV180517	2.200	weakly similar to (107)AT3G49430| Symbols: SRP34A | SRP34A (SER/ARG-RICH PROTEIN 34A) | chr3:18343653-18345602 FOR	
EE502208	2.198	no similarity	
EV098884	2.197	no similarity	3.377
EV194141	2.196	no similarity	
JCVI_16333	2.195	weakly similar to (183)AT5G17710| Symbols: EMB1241 | EMB1241 (EMBRYO DEFECTIVE 1241); adenyl-nucleotide exchange fac	
JCVI_28458	2.195	highly similar to (704)AT5G26310| Symbols: UGT72E3 | UGT72E3; UDP-glycosyltransferase/ coniferyl-alcohol glucosyltransferase/ t	
JCVI_17548	2.195	moderately similar to (292)AT1G78815| Symbols: | similar to unknown protein [Arabidopsis thaliana] (TAIR:AT1G16910.1); similar t	
EV146975	2.194	no similarity	

JCVI_27993	2.194	weakly similar to (117)AT1G56560| Symbols: | beta-fructofuranosidase, putative / invertase, putative / saccharase, putative / beta-fructo	
EV178209	2.193	no similarity	
ES938309	2.191	no similarity	
JCVI_38772	2.189	no original description	2.762
EV144440	2.189	no similarity	
JCVI_26223	2.189	moderately similar to (300)AT5G51990| Symbols: CBF4, DREB1D | CBF4/DREB1D (C- REPEAT-BINDING FACTOR 4); DNA bin	
EE562569	2.189	no similarity	
JCVI_22409	2.189	no original description	
JCVI_11605	2.189	moderately similar to (230)AT1G79340| Symbols: ATMC4 | ATMC4 (METACASPASE 4); caspase/ cysteine-type peptidase | chr1:298	
ES266187	2.189	moderately similar to (361)AT2G33100| Symbols: CSLD1, ATCSLD1 | ATCSLD1 (Cellulose synthase-like D1); cellulose synthase/ tra	
CX189187	2.189	no similarity	
JCVI_696	2.185	no original description	
JCVI_2054	2.185	no original description	
JCVI_30284	2.184	no original description	
JCVI_20083	2.184	no original description	
EX087049	2.183	very weakly similar to (100)AT1G12290| Symbols: | disease resistance protein (CC-NBS-LRR class), putative | chr1:4178591-4181245	2.436
JCVI_9348	2.182	no original description	
JCVI_38243	2.182	no original description	
JCVI_18453	2.182	moderately similar to (258)AT1G17620| Symbols: | similar to unknown protein [Arabidopsis thaliana] (TAIR:AT5G11890.1); similar t	
EH430271	2.180	no similarity	
EV219606	2.180	weakly similar to (180)AT2G26690| Symbols: | nitrate transporter (NTP2) | chr2:11354425-11357994 REVERSE [21492] 52 704 704	
EX089551	2.179	no similarity	
EV111696	2.178	no similarity	
JCVI_5240	2.176	moderately similar to (272)AT3G03950| Symbols: ECT1 | ECT1 | chr3:1021509-1023774 FORWARD no original description	
JCVI_21087	2.175	moderately similar to (217)AT3G53190| Symbols: | pectate lyase family protein | chr3:19725149-19728515 FORWARD no original de	
EV218736	2.175	no similarity	
EV088740	2.174	no similarity	
EE534620	2.172	no similarity	
ES950859	2.172	no similarity	
H74438	2.172	no similarity	
EX102092	2.172	moderately similar to (314)AT4G29780| Symbols: | similar to unknown protein [Arabidopsis thaliana] (TAIR:AT5G12010.1); similar t	
JCVI_14845	2.172	moderately similar to (471)ATCG01110| Symbols: NDHH | Encodes the 49KDa plastid NAD(P)H dehydrogenase subunit H protein. It	
JCVI_24538	2.172	weakly similar to (103)AT1G12000| Symbols: | pyrophosphate--fructose-6-phosphate 1-phosphotransferase beta subunit, putative / pyro	
EV098641	2.172	weakly similar to (169)AT5G10450| Symbols: AFT1, GRF6 | GRF6 (G-BOX REGULATING FACTOR 6); protein phosphorylated am	
EV151827	2.172	no similarity	
EV221722	2.172	very weakly similar to (87.0)AT2G37180| Symbols: PIP2;3, PIP2C, RD28 | RD28 (plasma membrane intrinsic protein 2;3); water chann	4.699
AM059925	2.172	no similarity	
EE559738	2.172	no similarity	
EX046962	2.172	no similarity	
JCVI_31746	2.172	no original description	
JCVI_11988	2.170	no original description	
ES968632	2.169	no similarity	
EX071243	2.168	weakly similar to (139)AT2G39855| Symbols: | similar to unknown protein [Arabidopsis thaliana] (TAIR:AT3G55646.1); similar to un	
JCVI_14937	2.167	no original description	
JCVI_27655	2.167	no original description	
JCVI_15016	2.166	no original description	
EH415296	2.166	no similarity	
CV432076	2.166	no similarity	
EE556297	2.165	no similarity	
JCVI_19674	2.162	no original description	
AT002205	2.162	no similarity	
EV167224	2.162	no similarity	
EE504774	2.161	no similarity	
JCVI_37340	2.161	moderately similar to (249)AT5G58580| Symbols: ATL63 | ATL63; protein binding / zinc ion binding | chr5:23694132-23695058 REV	
JCVI_32803	2.161	no original description	
DY029251	2.160	no similarity	
CX189531	2.158	weakly similar to (121)AT3G22040| Symbols: | receptor-like protein kinase-related | chr3:7761814-7762682 FORWARD [16807]	
RC_ES958682	2.158	no similarity	
EE424329	2.156	weakly similar to (106)AT1G24460| Symbols: | myosin-related | chr1:8666060-8672346 FORWARD [20158]	
JCVI_42319	2.155	no original description	
DN964809	2.155	weakly similar to (188)AT5G50420| Symbols: | similar to unknown protein [Arabidopsis thaliana] (TAIR:AT1G53770.1); similar to un	2.187
EV100550	2.155	weakly similar to (189)AT1G11440| Symbols: | similar to glycine-rich protein [Arabidopsis thaliana] (TAIR:AT3G29075.1); similar to	
RC_EE567818	2.154	no similarity	
EX130042	2.153	weakly similar to (127)AT5G14940| Symbols: | proton-dependent oligopeptide transport (POT) family protein | chr5:4831751-4834315	
EV147401	2.152	no similarity	
CN735960	2.152	no similarity	
JCVI_38623	2.150	highly similar to (556)AT3G55840| Symbols: | similar to unknown protein [Arabidopsis thaliana] (TAIR:AT2G40000.1); similar to unr	
RC_ES962063	2.150	no similarity	4.530
JCVI_35611	2.149	moderately similar to (235)AT3G61150| Symbols: HDG1 | HDG1 (HOMEODOMAIN GLABROUS1); DNA binding / transcription fa	
JCVI_28996	2.148	no original description	
JCVI_32830	2.148	highly similar to (592)AT5G18525| Symbols: | WD-40 repeat family protein | chr5:6146934-6149732 REVERSE no original descriptio	
DY021832	2.147	no similarity	
JCVI_29379	2.147	no original description	
EV144812	2.146	no similarity	
EV059840	2.146	no similarity	
ES266564	2.146	no similarity	
RC_EV032029	2.146	no similarity	
EV135225	2.146	no similarity	
JCVI_2317	2.146	no original description	
CD830742	2.145	no similarity	
JCVI_24387	2.145	moderately similar to (241)AT5G07070| Symbols: SnRK3.2, CIPK2 | CIPK2 (CBL-INTERACTING PROTEIN KINASE 2); kinase | cl	
ES966580	2.145	no similarity	
BG544771	2.143	no similarity	

JCVI_4691	2.143	no original description	
EV087233	2.143	no similarity	
EE471788	2.142	very weakly similar to (99.4)AT5G21930| Symbols: PAA2, HMA8 | HMA8/PAA2 (P-TYPE ATPASE OF ARABIDOPSIS 2); ATPase,	
JCVI_14317	2.141	no original description	
BG544701	2.141	no similarity	1.562
JCVI_21363	2.141	highly similar to (690)AT2G22400| Symbols: | NOL1/NOP2/sun family protein | chr2:9511903-9515868 REVERSE no original descri	
RC_CV973903	2.140	no similarity	
DN965542	2.140	no similarity	
ES966406	2.139	no similarity	
JCVI_16044	2.138	moderately similar to (225)AT3G21175| Symbols: TIFY2B, ZML1 | ZML1 (ZIM-LIKE 1) | chr3:7422838-7423771 FORWARD no ori	3.479
EE551761	2.137	no similarity	
JCVI_28686	2.137	no original description	3.195
JCVI_18700	2.137	moderately similar to (423)AT1G70590| Symbols: | F-box family protein | chr1:26622066-26623822 FORWARD no original descripti	
JCVI_7142	2.136	moderately similar to (377)AT2G42810| Symbols: PAPP5, PP5 | PAPP5/PP5 (PROTEIN PHOSPHATASE 5); phosphoprotein phospha	
EE557970	2.136	very weakly similar to (82.4)AT5G10010| Symbols: | similar to unknown protein [Arabidopsis thaliana] (TAIR:AT5G64910.1); similar	2.923
JCVI_41173	2.136	weakly similar to (181)AT5G02900| Symbols: CYP96A13 | CYP96A13 (cytochrome P450, family 96, subfamily A, polypeptide 13); o	
JCVI_17336	2.136	no original description	
JCVI_21850	2.135	moderately similar to (239)AT1G29090| Symbols: | peptidase C1A papain family protein | chr1:10163089-10164371 REVERSEvery w	
AM387464	2.135	no similarity	
JCVI_33167	2.135	no original description	
EV088344	2.135	no similarity	
EV091831	2.134	no similarity	
EE561329	2.134	no similarity	
EV193910	2.132	no similarity	
JCVI_8771	2.132	highly similar to (862)AT5G18840| Symbols: | sugar transporter, putative | chr5:6282956-6286401 FORWARDweakly similar to (166	4.527
JCVI_21979	2.130	weakly similar to (169)AT3G60800| Symbols: | zinc finger (DHHC type) family protein | chr3:22478461-22480248 REVERSE no orig	
DN961328	2.129	no similarity	
EV090686	2.128	weakly similar to (176)AT3G24420| Symbols: | hydrolase, alpha/beta fold family protein | chr3:8863118-8864890 REVERSE [21476]	
JCVI_38500	2.127	weakly similar to (124)AT5G14130| Symbols: | peroxidase, putative | chr5:4558864-4560030 REVERSEvery weakly similar to (82.8)l	
JCVI_33648	2.126	moderately similar to (290)AT2G42450| Symbols: | lipase class 3 family protein | chr2:17679503-17681932 REVERSE no original des	
JCVI_18452	2.126	no original description	
JCVI_42141	2.126	no original description	
JCVI_29693	2.126	moderately similar to (337)AT1G08630| Symbols: THA1 | THA1 (THREONINE ALDOLASE 1) | chr1:2743951-2745688 REVERSE	2.632
EE569691	2.126	no similarity	
JCVI_21714	2.125	weakly similar to (147)AT4G39840| Symbols: | similar to unnamed protein product [Vitis vinifera] (GB:CAO21162.1); similar to unna	
EX047424	2.124	no similarity	
ES912517	2.123	highly similar to (565)AT3G19820| Symbols: DIM, EVE1, DW1, DIM1, CBB1, DWF1 | DWF1 (DIMINUTO 1); catalytic | chr3:68798	
JCVI_29229	2.122	weakly similar to (119)AT5G52510| Symbols: | scarecrow-like transcription factor 8 (SCL8) | chr5:21324422-21326344 FORWARD r	
EV150562	2.116	no similarity	
JCVI_3065	2.115	moderately similar to (443)AT5G04180| Symbols: | carbonic anhydrase family protein | chr5:1147908-1149238 REVERSE no original	
CX279977	2.115	moderately similar to (235)AT2G42560| Symbols: | late embryogenesis abundant domain-containing protein / LEA domain-containing	
RC_EE567416	2.115	no similarity	
JCVI_40915	2.115	no original description	
JCVI_26660	2.115	moderately similar to (467)AT5G61510| Symbols: | NADP-dependent oxidoreductase, putative | chr5:24754310-24756201 REVERSE	
JCVI_40041	2.114	moderately similar to (234)AT2G39400| Symbols: | hydrolase, alpha/beta fold family protein | chr2:16459797-16461728 FORWARD	
JCVI_38816	2.113	no original description	
DY026036	2.113	weakly similar to (154)AT3G16490| Symbols: IQD26 | IQD26 (IQ-domain 26); calmodulin binding | chr3:5603968-5605495 REVERS	
EE494544	2.113	no similarity	
EV149501	2.112	no similarity	
JCVI_16662	2.111	moderately similar to (224)AT5G21170| Symbols: | 5'-AMP-activated protein kinase beta-2 subunit, putative | chr5:7205721-7208242	
JCVI_22827	2.111	highly similar to (518)AT1G21790| Symbols: | similar to unnamed protein product [Vitis vinifera] (GB:CAO61872.1); contains InterPr	
JCVI_30122	2.110	no original description	
RC_EE563070	2.109	no similarity	
CX269865	2.107	moderately similar to (231)AT4G10400| Symbols: | F-box family protein | chr4:6446331-6447711 REVERSE [16815]	
CD843764	2.107	no similarity	3.564
EV138924	2.106	no similarity	
EV111582	2.106	no similarity	
ES993543	2.106	no similarity	2.121
EL590485	2.106	weakly similar to (146)AT1G70770| Symbols: | similar to unknown protein [Arabidopsis thaliana] (TAIR:AT1G23170.1); similar to hy	
EV130012	2.105	no similarity	
EV140465	2.104	no similarity	
JCVI_21809	2.104	no original description	
EX134202	2.103	no similarity	1.739
EE567286	2.103	no similarity	
CV546780	2.102	no similarity	
EV218215	2.101	no similarity	
JCVI_8099	2.101	no original description	
JCVI_26192	2.101	moderately similar to (438)AT3G21090| Symbols: | ABC transporter family protein | chr3:7391503-7394939 REVERSE no original de	
H07242	2.101	no similarity	
EE562117	2.100	no similarity	2.733
JCVI_33113	2.099	no original description	
EE472195	2.099	no similarity	1.277
EV159783	2.098	weakly similar to (145)AT4G30810| Symbols: SCPL29 | SCPL29 (serine carboxypeptidase-like 29); serine carboxypeptidase | chr4:150	
RC_ES962408	2.097	no similarity	
EV132861	2.097	no similarity	
ES917057	2.097	moderately similar to (208)AT5G22920| Symbols: | zinc finger (C3HC4-type RING finger) family protein | chr5:7665146-7667034 FC	2.262
EV112391	2.097	no similarity	
EX091753	2.096	no similarity	
JCVI_33259	2.096	weakly similar to (191)AT1G62120| Symbols: | mitochondrial transcription termination factor-related / mTERF-related | chr1:2296399	3.079
JCVI_19706	2.095	no original description	
EE565674	2.094	no similarity	
JCVI_1184	2.093	moderately similar to (280)AT2G35960| Symbols: NHL12 | NHL12 (NDR1/HIN1-like 12) | chr2:15114229-15114861 FORWARD no	
EV098433	2.093	no similarity	

JCVI_34493	2.090	weakly similar to (150)AT3G26910| Symbols: | hydroxyproline-rich glycoprotein family protein | chr3:9916541-9919748 REVERSE r	
EE545727	2.089	no similarity	
EV132933	2.088	no similarity	
JCVI_113	2.087	moderately similar to (371)AT2G43130| Symbols: AtRab11F, AtRABA5c, Ara-4 | ARA4 (Arabidopsis Rab GTPase homolog A5c); G7	
JCVI_42470	2.087	weakly similar to (171)AT3G28200| Symbols: | peroxidase, putative | chr3:10519319-10520269 FORWARD no original description	
JCVI_6426	2.087	moderately similar to (240)AT1G80830| Symbols: PMIT1, ATNRAMP1, NRAMP1 | NRAMP1 (NRAMP metal ion transporter 1); mar	
CD843729	2.085	no similarity	
EV199280	2.085	no similarity	
EV165341	2.085	moderately similar to (261)AT5G57190| Symbols: PSD2 | PSD2 (PHOSPHATIDYLSERINE DECARBOXYLASE 2); phosphatidylser	
RC_EE543346	2.084	no similarity	
EE562825	2.084	no similarity	
JCVI_8102	2.082	moderately similar to (235)AT3G17850| Symbols: | protein kinase, putative | chr3:6109860-6116251 REVERSE no original descriptio	
EV111479	2.082	very weakly similar to (96.7)AT4G23700| Symbols: CHX17, ATCHX17 | ATCHX17 (CATION/H+ EXCHANGER 17); monovalent ca	2.348
EX023620	2.082	weakly similar to (182)AT1G04830| Symbols: | RabGAP/TBC domain-containing protein | chr1:1359086-1361843 REVERSE [21809	
EX086430	2.080	very weakly similar to (99.0)AT5G36228| Symbols: | nucleic acid binding / zinc ion binding | chr5:14288921-14290006 FORWARD [2	
JCVI_35107	2.080	weakly similar to (191)AT3G62170| Symbols: VGDH2 | VGDH2 (VANGUARD 1 HOMOLOG 2); pectinesterase | chr3:23027470-23	
RC_EX055797	2.078	no similarity	2.546
RC_EE559778	2.077	no similarity	
ES930022	2.077	weakly similar to (132)AT5G18800| Symbols: | NADH-ubiquinone oxidoreductase 19 kDa subunit (NDUFA8) family protein | chr5:62	
JCVI_9869	2.076	moderately similar to (326)AT2G34590| Symbols: | transketolase family protein | chr2:14576035-14577923 REVERSEmoderately sim	
CN733031	2.076	no similarity	
EE548763	2.076	no similarity	
JCVI_28834	2.076	highly similar to (689)AT4G28650| Symbols: | leucine-rich repeat transmembrane protein kinase, putative | chr4:14144161-14147282 I	
JCVI_41666	2.076	weakly similar to (151)AT2G45120| Symbols: | zinc finger (C2H2 type) family protein | chr2:18610755-18611699 FORWARD no orig	
JCVI_40985	2.076	weakly similar to (159)AT3G59080| Symbols: | aspartyl protease family protein | chr3:21847789-21849396 FORWARD no original de	
JCVI_25460	2.076	weakly similar to (157)AT5G23700| Symbols: | similar to unknown protein [Arabidopsis thaliana] (TAIR:AT3G48860.2); similar to hy	
EV110529	2.075	no similarity	
EE561790	2.075	no similarity	
EE419429	2.075	no similarity	
JCVI_31482	2.074	no original description	
JCVI_21105	2.073	very weakly similar to (84.7)AT2G35765| Symbols: | similar to unknown protein [Arabidopsis thaliana] (TAIR:AT1G19500.1) | chr2:1	
JCVI_23169	2.073	no original description	
JCVI_18757	2.073	weakly similar to (196)AT1G24260| Symbols: AGL9, SEP3 | SEP3 (SEPALLATA3); transcription factor | chr1:8593779-8595851 RE	
EX096357	2.072	no similarity	
JCVI_25363	2.070	moderately similar to (324)AT1G18460| Symbols: | lipase family protein | chr1:6352675-6355964 FORWARD no original description	
EE565238	2.070	no similarity	
EE427194	2.069	no similarity	
EV019444	2.069	weakly similar to (122)AT1G69290| Symbols: | pentatricopeptide (PPR) repeat-containing protein | chr1:26051035-26053011 REVER	
EV144814	2.068	no similarity	
JCVI_24568	2.068	no original description	
EV141577	2.067	weakly similar to (132)AT4G37990| Symbols: ELI3, ELI3-2 | ELI3-2 (ELICITOR-ACTIVATED GENE 3) | chr4:17855958-17857382	
CX267764	2.067	no similarity	3.541
CD828534	2.067	no similarity	3.078
JCVI_30997	2.066	moderately similar to (420)AT1G15210| Symbols: PDR7, ATPDR7 | ATPDR7/PDR7 (PLEIOTROPIC DRUG RESISTANCE 7); ATP	
EV219183	2.066	no similarity	
ES269370	2.065	no similarity	
CX267453	2.064	no similarity	
JCVI_30388	2.064	no original description	
JCVI_13509	2.064	highly similar to (671)AT5G63930| Symbols: | leucine-rich repeat transmembrane protein kinase, putative | chr5:25600232-25603618 I	
AM389116	2.064	no similarity	
EV046827	2.061	no similarity	
JCVI_7161	2.061	moderately similar to (337)AT3G50060| Symbols: MYB77 | MYB77; DNA binding / transcription factor | chr3:18569129-18570034 R	
EE563466	2.061	no similarity	
JCVI_37344	2.060	moderately similar to (421)AT2G44220| Symbols: | similar to unknown protein [Arabidopsis thaliana] (TAIR:AT2G44240.1); similar t	3.696
JCVI_11305	2.059	highly similar to (827)AT3G22520| Symbols: | similar to unknown protein [Arabidopsis thaliana] (TAIR:AT4G14840.1); similar to unk	
CX270847	2.059	no similarity	
EX131682	2.058	weakly similar to (163)AT5G44730| Symbols: | haloacid dehalogenase-like hydrolase family protein | chr5:18062815-18063746 REVE	
JCVI_35972	2.057	very weakly similar to (93.2)AT2G31945| Symbols: | similar to unknown protein [Arabidopsis thaliana] (TAIR:AT1G05575.1); similar	
EV215352	2.056	no similarity	
JCVI_32780	2.053	no original description	
JCVI_41102	2.053	no original description	
RC_EE558774	2.053	no similarity	
CD819016	2.051	moderately similar to (204)AT5G04530| Symbols: | beta-ketoacyl-CoA synthase family protein | chr5:1292223-1293617 REVERSE [1	
CV432234	2.051	very weakly similar to (87.4)AT2G01450| Symbols: ATMPK17 | ATMPK17 (Arabidopsis thaliana MAP kinase 17); MAP kinase | chr2:	
JCVI_29355	2.050	no original description	
JCVI_13749	2.049	moderately similar to (340)AT1G80070| Symbols: EMB158, EMB33, EMB177, EMB14, SUS2 | SUS2 (ABNORMAL SUSPENSOR	
EV064850	2.049	no similarity	
EV173253	2.048	moderately similar to (432)AT3G27970| Symbols: | exonuclease | chr3:10390846-10392781 FORWARD [21486] 102 877 877	
ES269021	2.048	no similarity	
JCVI_35831	2.048	weakly similar to (103)AT4G15280| Symbols: | UDP-glucoronosyl/UDP-glucosyl transferase family protein | chr4:8719182-8720618 I	
EE559030	2.047	no similarity	
EV164136	2.045	weakly similar to (172)AT4G27080| Symbols: ATPDIL5-4 | ATPDIL5-4 (PDI-LIKE 5-4) | chr4:13589162-13593341 FORWARD [214	
JCVI_23106	2.045	no original description	
EE438020	2.045	weakly similar to (102)AT5G51210| Symbols: OLEO3 | OLEO3 (OLEOSIN3) | chr5:20837397-20837932 FORWARDvery weakly sin	
EE439045	2.043	no similarity	
EE547503	2.043	no similarity	1.198
EV150094	2.042	weakly similar to (151)AT5G51220| Symbols: | ubiquinol-cytochrome C chaperone family protein | chr5:20838631-20840351 REVER	2.021
RC_L46444	2.042	no similarity	
EV113522	2.041	moderately similar to (210)AT4G17800| Symbols: | DNA-binding protein-related | chr4:9895562-9896440 REVERSE [21479]	
JCVI_9305	2.040	highly similar to (692)AT4G19120| Symbols: ERD3 | ERD3 (EARLY-RESPONSIVE TO DEHYDRATION 3) | chr4:10460676-10463	
JCVI_21660	2.039	weakly similar to (148)ATMG01360| Symbols: COX1 | cytochrome c oxidase subunit 1 | chrM:349830-351413 REVERSEweakly simi	
JCVI_26941	2.039	moderately similar to (248)AT5G02960| Symbols: | 40S ribosomal protein S23 (RPS23B) | chr5:693278-694394 REVERSEmoderatel	
EX088920	2.038	moderately similar to (303)AT2G31580| Symbols: | similar to unknown protein [Arabidopsis thaliana] (TAIR:AT2G32320.1); similar t	

JCVI_12862	2.038	moderately similar to (211)AT5G37790| Symbols: | protein kinase family protein | chr5:15025663-15028255 REVERSE no original de	
JCVI_25462	2.037	no original description	
JCVI_13618	2.037	moderately similar to (227)AT4G01280| Symbols: | myb family transcription factor | chr4:535288-536854 FORWARD no original des	
JCVI_37995	2.037	highly similar to (508)AT4G24250| Symbols: ATMLO13, MLO13 | MLO13 (MILDEW RESISTANCE LOCUS O 13); calmodulin bin	
EV194567	2.036	no similarity	
EV090075	2.036	no similarity	
H07212	2.035	no similarity	2.695
JCVI_21495	2.035	weakly similar to (140)AT1G24160| Symbols: | similar to unknown protein [Arabidopsis thaliana] (TAIR:AT1G70100.3); similar to un	
JCVI_21658	2.035	no original description	
JCVI_19565	2.034	no original description	
RC_EV064436	2.033	no similarity	
EL589202	2.033	moderately similar to (373)AT1G55810| Symbols: | uracil phosphoribosyltransferase, putative / UMP pyrophosphorylase, putative / UP	
ES269387	2.033	moderately similar to (473)AT2G25600| Symbols: AKT6, SPIK | SPIK (SHAKER POLLEN INWARD K+ CHANNEL); cyclic nucleo	
JCVI_25123	2.033	weakly similar to (176)AT5G20380| Symbols: | transporter-related | chr5:6887938-6892360 REVERSE no original description	
EE559382	2.033	very weakly similar to (92.4)AT3G60040| Symbols: | F-box family protein | chr3:22186914-22190705 REVERSE [20153] 18 563 563	
EX088005	2.032	no similarity	
EV163883	2.032	no similarity	
JCVI_4292	2.031	moderately similar to (205)AT4G14420| Symbols: | lesion inducing protein-related | chr4:8302167-8303734 REVERSE no original des	
JCVI_18863	2.031	weakly similar to (150)AT3G58850| Symbols: PAR2 | PAR2 (PHY RAPIDLY REGULATED 2); transcription regulator | chr3:21770	
JCVI_3379	2.031	moderately similar to (469)AT5G39320| Symbols: | UDP-glucose 6-dehydrogenase, putative | chr5:15760482-15761924 FORWARDn	
CX266960	2.028	no similarity	
EB041738	2.027	no similarity	
JCVI_21520	2.027	no original description	
EE431349	2.027	moderately similar to (222)AT1G47600| Symbols: | glycosyl hydrolase family 1 protein | chr1:17494211-17497029 FORWARDweakl	
JCVI_27606	2.027	moderately similar to (270)AT1G59660| Symbols: | nucleoporin family protein | chr1:21928358-21932898 FORWARD no original des	
JCVI_6200	2.027	moderately similar to (432)AT3G15450| Symbols: | similar to unknown protein [Arabidopsis thaliana] (TAIR:AT4G27450.1); similar t	
JCVI_20045	2.027	moderately similar to (399)AT4G38530| Symbols: ATPLC1 | ATPLC1 (PHOSPHOLIPASE C 1); phospholipase C | chr4:18020702-18	
EV216785	2.027	no similarity	
JCVI_40202	2.026	very weakly similar to (83.6)AT4G27657| Symbols: | similar to unknown protein [Arabidopsis thaliana] (TAIR:AT4G27652.1) | chr4:1	
JCVI_19632	2.025	moderately similar to (421)AT5G12080| Symbols: | mechanosensitive ion channel domain-containing protein / MS ion channel domain	
JCVI_12561	2.024	no original description	
EE448803	2.024	weakly similar to (150)AT1G11480| Symbols: | eukaryotic translation initiation factor-related | chr1:3864368-3866707 REVERSE [20	4.507
EE453480	2.022	no similarity	
JCVI_21756	2.022	moderately similar to (263)AT1G72520| Symbols: | lipoxygenase, putative | chr1:27312273-27316251 FORWARDweakly similar to (	
EV132818	2.022	no similarity	
JCVI_38351	2.022	no original description	
JCVI_6543	2.022	very weakly similar to (96.3)AT3G24080| Symbols: | KRR1 family protein | chr3:8695198-8697114 REVERSE no original description	
JCVI_30203	2.019	no original description	
AM057474	2.019	no similarity	
JCVI_27328	2.018	moderately similar to (269)AT1G78160| Symbols: APUM7 | APUM7 (ARABIDOPSIS PUMILIO 7); RNA binding | chr1:29412793-2	
RC_ES951332	2.018	no similarity	
ES938339	2.018	weakly similar to (173)AT3G12820| Symbols: AtMYB10 | AtMYB10 (myb domain protein 10); DNA binding / transcription factor | ch	
JCVI_13863	2.017	moderately similar to (398)AT5G13050| Symbols: 5-FCL | 5-FCL; 5-formyltetrahydrofolate cyclo-ligase | chr5:4137265-4138714 REV	
EB041739	2.017	no similarity	
EV182652	2.016	no similarity	
EE485895	2.015	no similarity	
EV088245	2.014	weakly similar to (129)AT4G35600| Symbols: CONNEXIN 32 | CONNEXIN 32; kinase | chr4:16896453-16898719 FORWARD [214	
EE558058	2.014	no similarity	
EV109748	2.013	no similarity	
EV222413	2.013	no similarity	
ES962947	2.012	no similarity	
JCVI_42135	2.012	moderately similar to (356)AT1G59900| Symbols: AT-E1 ALPHA | AT-E1 ALPHA (pyruvate dehydrogenase complex E1 alpha subun	
RC_EE462682	2.011	no similarity	
RC_JCVI_19560	2.011	no original description	
ES998460	2.011	no similarity	
EE531816	2.011	very weakly similar to (96.3)AT4G29090| Symbols: | reverse transcriptase, putative / RNA-dependent DNA polymerase, putative | chr4:	
EX086786	2.010	moderately similar to (283)AT3G28940| Symbols: | avirulence-responsive protein, putative / avirulence induced gene (AIG) protein, pu	
EX045585	2.010	no similarity	
CX270567	2.009	weakly similar to (109)AT5G15540| Symbols: EMB2773 | EMB2773 (EMBRYO DEFECTIVE 2773); binding / protein binding / zinc	
JCVI_10122	2.009	weakly similar to (171)AT5G57790| Symbols: | unknown protein | chr5:23429469-23430668 REVERSE no original description	
EV068125	2.008	no similarity	
EV129846	2.008	no similarity	
JCVI_38684	2.007	no original description	
ES969123	2.007	no similarity	
JCVI_3752	2.006	moderately similar to (307)AT1G31880| Symbols: NIP3;1, NLM9, BRX | BRX/NIP3;1/NLM9 (BREVIS RADIX); identical protein bi	
EE524530	2.006	moderately similar to (202)AT3G26780| Symbols: | catalytic | chr3:9849984-9851514 FORWARD [20143]	
EH419213	2.005	no similarity	
JCVI_29307	2.005	weakly similar to (125)AT5G13300| Symbols: VAN3, SFC | SFC (SCARFACE) | chr5:4255926-4262021 REVERSE no original descr	
ES959432	2.005	very weakly similar to (100)AT1G08520| Symbols: PDE166, CHLD | CHLD/PDE166 (PIGMENT DEFECTIVE 166); magnesium che	
RC_JCVI_24506	2.004	no original description	
H07426	2.004	no similarity	
EV226604	2.004	no similarity	
JCVI_36867	2.003	very weakly similar to (94.7)AT2G26850| Symbols: | F-box family protein | chr2:11456447-11457967 REVERSE no original descripti	
EV192330	2.002	no similarity	
EE473381	2.001	weakly similar to (130)AT1G72570| Symbols: | DNA binding / transcription factor | chr1:27335043-27337360 FORWARD [20163]	2.714
JCVI_23621	1.999	no original description	3.157
EX137551	1.999	moderately similar to (236)AT3G04370| Symbols: | Identical to Cysteine-rich repeat secretory protein 39 precursor (CRRSP39) [Arabi	
EE508434	1.998	weakly similar to (111)AT3G03410| Symbols: | calmodulin-related protein, putative | chr3:811331-811726 REVERSE [15718] 1 490 5	
JCVI_35686	1.998	no original description	
BG543161	1.997	no similarity	
CN727560	1.995	very weakly similar to (99.0)AT1G08750| Symbols: | GPI-anchor transamidase, putative | chr1:2801286-2804395 FORWARD [15722]	
EX119983	1.995	no similarity	2.573
JCVI_13982	1.994	no original description	

JCVI_18023	1.994	weakly similar to (186)AT3G24550| Symbols: ATPERK1 | ATPERK1 (PROLINE EXTENSIN-LIKE RECEPTOR KINASE 1); ATP b	
EX016696	1.994	no similarity	
JCVI_36742	1.994	no original description	
EV160261	1.993	weakly similar to (107)AT1G32920| Symbols: | similar to unknown protein [Arabidopsis thaliana] (TAIR:AT1G32928.1) | chr1:11928	
ES968137	1.991	no similarity	
EV094248	1.991	very weakly similar to (82.4)AT3G57260| Symbols: PR2, BG2, PR-2, BGL2 | BGL2 (PATHOGENESIS-RELATED PROTEIN 2); gluc	
DY009744	1.991	no similarity	
JCVI_4868	1.991	moderately similar to (325)AT1G67730| Symbols: YBR159 | YBR159; ketoreductase/ oxidoreductase | chr1:25395339-25397028 FOR	
JCVI_3657	1.989	moderately similar to (240)AT2G46030| Symbols: UBC6 | UBC6 (UBIQUITIN-CONJUGATING ENZYME 6); ubiquitin-protein ligas	
EV060838	1.988	moderately similar to (209)AT1G71070| Symbols: | glycosyltransferase family 14 protein / core-2/I-branching enzyme family protein |	
JCVI_7741	1.986	no original description	
RC_JCVI_42038	1.985	no original description	
AM396053	1.985	no similarity	
JCVI_171	1.984	moderately similar to (229)AT5G67420| Symbols: LBD37 | LBD37 (LOB DOMAIN-CONTAINING PROTEIN 37) | chr5:26921802-2	
CD815607	1.984	weakly similar to (181)AT4G24970| Symbols: | ATP-binding region, ATPase-like domain-containing protein | chr4:12831135-1283545	
EX093682	1.984	no similarity	
EV191670	1.983	no similarity	
EE423429	1.983	no similarity	
JCVI_14630	1.983	no original description	
JCVI_42282	1.982	weakly similar to (162)AT3G25585| Symbols: AAPT2 | AAPT2 (AMINOALCOHOLPHOSPHOTRANSFERASE); phosphatidyltransf	
JCVI_39310	1.982	no original description	2.019
EV091050	1.981	moderately similar to (203)AT1G14620| Symbols: DECOY | DECOY (endoxyloglucan transferase A2) | chr1:5014943-5016496 REVE	
CN727410	1.980	moderately similar to (262)AT5G64190| Symbols: | similar to unknown protein [Arabidopsis thaliana] (TAIR:AT2G40390.1); similar t	2.341
EE534969	1.980	no similarity	
EX083822	1.977	very weakly similar to (91.7)AT1G54040| Symbols: ESR, TASTY, ESP | ESP (EPITHIOSPECIFIER PROTEIN) | chr1:20174663-2017	
EV134209	1.977	no similarity	
EV197150	1.977	no similarity	
EV108126	1.977	weakly similar to (127)AT3G03100| Symbols: | NADH:ubiquinone oxidoreductase family protein | chr3:705571-707585 REVERSE [2	
JCVI_23044	1.977	weakly similar to (155)AT1G13390| Symbols: | similar to unknown protein [Arabidopsis thaliana] (TAIR:AT1G68490.1); similar to un	
RC_ES967962	1.977	no similarity	
ES965114	1.975	no similarity	
ES967006	1.975	no similarity	
EE568004	1.975	no similarity	
EE569332	1.975	weakly similar to (129)AT1G54070| Symbols: | dormancy/auxin associated protein-related | chr1:20186351-20186910 FORWARD [20	
JCVI_33502	1.973	moderately similar to (450)AT4G31940| Symbols: CYP82C4 | CYP82C4 (cytochrome P450, family 82, subfamily C, polypeptide 4); o	
JCVI_10773	1.973	no original description	
ES967676	1.973	no similarity	
EV105096	1.971	no similarity	
EE557906	1.971	no similarity	
JCVI_7498	1.971	no original description	
EV193158	1.971	moderately similar to (206)AT1G09932| Symbols: | phosphoglycerate/bisphosphoglycerate mutase-related | chr1:3230718-3232926 RE	
EV100424	1.969	moderately similar to (385)AT1G60590| Symbols: | polygalacturonase, putative / pectinase, putative | chr1:22318091-22320532 REVE	
AM394903	1.968	no similarity	3.560
RC_JCVI_40485	1.968	no original description	
JCVI_32942	1.967	no original description	
EV170896	1.967	moderately similar to (254)AT5G25900| Symbols: CYP701A3, GA3 | GA3 (GA REQUIRING 3); oxygen binding | chr5:9036076-9038	
EV085654	1.966	no similarity	2.081
EV209930	1.966	no similarity	
EX040735	1.966	weakly similar to (160)AT2G17800| Symbols: ARAC1, ATGP2, ATRAC1, RAC1, ROP3, AtROP3 | ARAC1/ATGP2/ATRAC1/AtRO	
EX097145	1.966	weakly similar to (144)AT5G41690| Symbols: | RNA binding | chr5:16687354-16691417 REVERSE [21824]	
JCVI_16727	1.965	moderately similar to (207)AT1G19730| Symbols: ATH4, ATTRX4 | ATTRX4 (thioredoxin H-type 4); thiol-disulfide exchange interm	
CV432081	1.964	no similarity	2.596
JCVI_30734	1.964	moderately similar to (208)AT1G71400| Symbols: | disease resistance family protein / LRR family protein | chr1:26913567-26916110	
JCVI_31836	1.964	no original description	
CV973910	1.964	no similarity	
CV973916	1.964	no similarity	
EV098722	1.964	moderately similar to (270)AT1G14740| Symbols: | similar to unknown protein [Arabidopsis thaliana] (TAIR:AT3G63500.2); similar t	
JCVI_11504	1.963	moderately similar to (361)AT1G18300| Symbols: ATNUDT4 | ATNUDT4 (Arabidopsis thaliana Nudix hydrolase homolog 4); hydrola	
JCVI_22028	1.962	highly similar to (506)AT1G61130| Symbols: SCPL32 | SCPL32; serine carboxypeptidase | chr1:22532689-22535449 REVERSEmode	
EV208608	1.961	no similarity	
JCVI_15928	1.961	weakly similar to (103)AT3G12750| Symbols: ZIP1 | ZIP1 (ZINC TRANSPORTER 1 PRECURSOR); zinc ion transmembrane transpo	1.672
ES936686	1.958	no similarity	
JCVI_16840	1.957	weakly similar to (163)AT1G27100| Symbols: | similar to unknown protein [Arabidopsis thaliana] (TAIR:AT1G69900.1); similar to hy	
JCVI_17852	1.955	moderately similar to (461)AT3G22104| Symbols: | phototropic-responsive NPH3 protein-related | chr3:7789821-7792186 FORWARI	1.775
EE467749	1.954	no similarity	2.632
JCVI_34813	1.954	no original description	
EE550610	1.953	no similarity	
EV142231	1.953	no similarity	
JCVI_10689	1.953	no original description	4.871
JCVI_5423	1.951	moderately similar to (325)AT3G61880| Symbols: CYP78A9 | CYP78A9 (CYTOCHROME P450 78A9); oxygen binding | chr3:22917	
JCVI_28609	1.951	no original description	
JCVI_12043	1.950	weakly similar to (164)AT4G32090| Symbols: | galactosyltransferase | chr4:15509996-15510826 REVERSE no original description	
EV063153	1.948	no similarity	
JCVI_13279	1.948	moderately similar to (385)AT1G10010| Symbols: AAP8 | AAP8 (amino acid permease 8); amino acid transmembrane transporter | chr1	
ES997598	1.948	no similarity	
EV109865	1.947	no similarity	
EX067093	1.946	no similarity	2.732
JCVI_20223	1.946	no original description	3.518
JCVI_10128	1.946	no original description	
EV100308	1.946	weakly similar to (189)AT5G59660| Symbols: | leucine-rich repeat protein kinase, putative | chr5:24052913-24057205 FORWARDver	
DY015548	1.946	weakly similar to (192)AT1G09570| Symbols: FHY2, FRE1, HY8, PHYA | PHYA (PHYTOCHROME A) | chr1:3095500-3098894 RI	1.724
EE568833	1.946	weakly similar to (103)AT5G06070| Symbols: RAB, RBE | RBE (RABBIT EARS); nucleic acid binding / transcription factor/ zinc ion	
AM386883	1.945	weakly similar to (191)AT1G76600| Symbols: | similar to unknown protein [Arabidopsis thaliana] (TAIR:AT1G21010.1); similar to hy	

CD828547	1.945	no similarity	
EE392293	1.943	weakly similar to (131)AT2G42480| Symbols: | meprin and TRAF homology domain-containing protein / MATH domain-containing p	
EV176999	1.943	very weakly similar to (95.9)AT5G23630| Symbols: | (MALE GAMETOGENESIS IMPAIRED ANTHERS); cation-transporting ATPa	
EX101625	1.943	no similarity	
JCVI_4372	1.943	moderately similar to (303)AT4G26720| Symbols: EP124, PPX-1, EP129, PPX1 | PPX1 (protein phosphatase x-1); protein serine/threo	
JCVI_30567	1.942	weakly similar to (166)AT2G43810| Symbols: | small nuclear ribonucleoprotein F, putative / U6 snRNA-associated Sm-like protein, pu	
EV089696	1.942	no similarity	
EV134137	1.942	no similarity	
EE413685	1.940	moderately similar to (277)AT1G27020| Symbols: | similar to unknown protein [Arabidopsis thaliana] (TAIR:AT1G27030.1); similar t	
EV192716	1.940	weakly similar to (198)AT5G02490| Symbols: | heat shock cognate 70 kDa protein 2 (HSC70-2) (HSP70-2) | chr5:550294-552563 RE	
CD823627	1.940	no similarity	
ES938422	1.940	no similarity	
EE568889	1.939	no similarity	
EE445726	1.939	no similarity	
RC_ES899340	1.939	no similarity	3.337
JCVI_7980	1.938	no original description	
JCVI_35395	1.936	no original description	
EV144299	1.935	no similarity	
EE530127	1.934	no similarity	
DY025489	1.934	moderately similar to (467)AT5G51810| Symbols: GA20OX2, AT2353, ATGA20OX2 | AT2353/ATGA20OX2/GA20OX2 (GIBBERI	
CX268053	1.933	weakly similar to (127)AT1G60830| Symbols: | U2 snRNP auxiliary factor large subunit, putative | chr1:22398723-22399268 REVERS	
EE471019	1.933	no similarity	2.554
ES914604	1.932	no similarity	
EV163400	1.931	no similarity	
JCVI_21881	1.929	no original description	2.722
H74867	1.927	no similarity	
CD830177	1.926	no similarity	
EE454397	1.926	moderately similar to (207)AT3G11460| Symbols: | pentatricopeptide (PPR) repeat-containing protein | chr3:3608256-3610127 FORW	
JCVI_7542	1.925	very weakly similar to (82.0)AT2G24450| Symbols: FLA3 | FLA3 (FASCICLIN-LIKE ARABINOGALACTAN PROTEIN 3 PRECUR	
JCVI_24052	1.925	moderately similar to (215)AT1G09660| Symbols: | KH domain-containing quaking protein, putative | chr1:3128207-3130793 REVER	
RC_DY014259	1.925	no similarity	
EE561338	1.924	no similarity	
ES965210	1.923	no similarity	1.897
EV151707	1.923	no similarity	
JCVI_11805	1.922	highly similar to (863)AT5G40270| Symbols: | metal-dependent phosphohydrolase HD domain-containing protein | chr5:16110622-16	4.838
EV147666	1.920	no similarity	
JCVI_15183	1.920	highly similar to (608)AT3G52200| Symbols: LTA3 | LTA3 (Dihydrolipoamide S-acetyltransferase 3); dihydrolipoyllysine-residue acet	
EV142230	1.920	moderately similar to (246)AT3G47610| Symbols: | transcription regulator/ zinc ion binding | chr3:17560163-17562189 FORWARD [	
JCVI_34418	1.920	weakly similar to (169)AT2G21470| Symbols: ATSAE2, EMB2764, SAE2 | SAE2 (SUMO-ACTIVATING ENZYME 2) | chr2:92058	
JCVI_11409	1.920	no original description	
CX193796	1.919	no similarity	
JCVI_6677	1.919	moderately similar to (387)AT4G29700| Symbols: | type I phosphodiesterase/nucleotide pyrophosphatase family protein | chr4:145437	
ES901797	1.919	moderately similar to (467)AT5G09640| Symbols: SCPL19, SNG2 | SNG2 (SINAPOYLGLUCOSE ACCUMULATOR 2); serine carb	
JCVI_32770	1.918	no original description	
JCVI_34440	1.917	moderately similar to (214)AT4G13350| Symbols: | human Rev interacting-like protein-related / hRIP protein-related | chr4:7770166-7	
EX055332	1.916	moderately similar to (228)AT5G18880| Symbols: | glucose transmembrane transporter | chr5:6300537-6301424 REVERSE [21813]	
EV220133	1.916	no similarity	
EE442548	1.916	no similarity	
EE560050	1.915	no similarity	
EV127979	1.915	no similarity	
ES906146	1.915	moderately similar to (362)AT2G17080| Symbols: | similar to unknown protein [Arabidopsis thaliana] (TAIR:AT2G17070.1); similar t	
JCVI_28934	1.915	moderately similar to (376)AT2G29740| Symbols: | UDP-glucoronosyl/UDP-glucosyl transferase family protein | chr2:12713824-1271	
JCVI_3404	1.915	moderately similar to (384)AT3G50060| Symbols: MYB77 | MYB77; DNA binding / transcription factor | chr3:18569129-18570034 R	
JCVI_23195	1.914	weakly similar to (177)AT4G23550| Symbols: WRKY29 | WRKY29 (WRKY DNA-binding protein 29); transcription factor | chr4:122	
RC_H07302	1.912	no similarity	
JCVI_6241	1.912	highly similar to (521)AT4G37320| Symbols: CYP81D5 | CYP81D5 (cytochrome P450, family 81, subfamily D, polypeptide 5); oxyg	
JCVI_39267	1.912	highly similar to (649)AT4G00660| Symbols: | DEAD/DEAH box helicase, putative | chr4:274638-277438 FORWARDweakly similar	
JCVI_16889	1.912	weakly similar to (103)AT4G23496| Symbols: SP1L5 | SP1L5 (SPIRAL1-LIKE5) | chr4:12257924-12258307 REVERSE no original d	2.202
EV032701	1.911	no similarity	
EX043377	1.911	weakly similar to (118)AT3G09530| Symbols: ATEXO70H3 | ATEXO70H3 (exocyst subunit EXO70 family protein H3); protein bind	
EV026904	1.908	moderately similar to (317)AT2G37700| Symbols: | similar to CER1 protein, putative [Arabidopsis thaliana] (TAIR:AT1G02190.1); si	
EV146041	1.908	no similarity	
JCVI_39230	1.908	no original description	
EE564724	1.908	no similarity	
ES899313	1.907	no similarity	
EE532266	1.906	moderately similar to (305)AT3G14490| Symbols: | terpene synthase/cyclase family protein | chr3:4863638-4865956 REVERSEweakl	
JCVI_35529	1.904	no original description	3.477
JCVI_25830	1.903	moderately similar to (248)AT1G07570| Symbols: APK1, APK1A | APK1A (Arabidopsis protein kinase 1A); kinase | chr1:2331366-23	
EX135316	1.903	no similarity	2.964
EV108280	1.903	no similarity	
EE558691	1.902	no similarity	
EV152555	1.902	no similarity	
EV130895	1.902	no similarity	
JCVI_15023	1.902	moderately similar to (262)AT4G14350| Symbols: | protein kinase family protein | chr4:8256349-8259930 REVERSE no original desc	-1.952
EV092671	1.902	moderately similar to (264)AT2G24765| Symbols: ARF3, ARL1, ATARL1 | ARF3/ARL1/ATARL1 (ADP-RIBOSYLATION FACTO	
ES968428	1.901	no similarity	
EV142567	1.900	no similarity	
EE565008	1.899	no similarity	
EE474833	1.899	no similarity	
EE559048	1.898	no similarity	
EE550318	1.898	weakly similar to (101)AT1G74680| Symbols: | exostosin family protein | chr1:28063189-28064645 FORWARD [20184] 1 349 407	
JCVI_42268	1.897	no original description	
CN729010	1.897	no similarity	

RC_CO750204	1.896	no similarity	2.769
JCVI_42216	1.894	no original description	
EV075089	1.894	no similarity	
ES920481	1.894	weakly similar to (182)AT5G49330| Symbols: AtMYB111 | AtMYB111 (myb domain protein 111); DNA binding / transcription factor	
EE558707	1.893	no similarity	
JCVI_16518	1.893	moderately similar to (232)AT1G75450| Symbols: ATCKX5, ATCKX6, CKX5 | CKX5 (CYTOKININ OXIDASE 5); cytokinin dehyd	
EE502213	1.892	weakly similar to (199)AT2G04520| Symbols: | eukaryotic translation initiation factor 1A, putative / eIF-1A, putative / eIF-4C, putative	
JCVI_23825	1.890	no original description	
ES968980	1.890	no similarity	
EX016437	1.890	weakly similar to (115)AT5G59510| Symbols: RTFL5, DVL18 | DVL18/RTFL5 (ROTUNDIFOLIA LIKE 5) | chr5:24007315-240077	
JCVI_36328	1.890	nearly identical (1134)AT5G41770| Symbols: | crooked neck protein, putative / cell cycle protein, putative | chr5:16735249-16738164 F	
EE561047	1.889	no similarity	
DR697814	1.889	no similarity	
EE557936	1.889	no similarity	
JCVI_27478	1.888	weakly similar to (117)AT3G22270| Symbols: | similar to unknown protein [Arabidopsis thaliana] (TAIR:AT4G14990.1); similar to un	
EV110410	1.887	no similarity	
JCVI_5721	1.887	moderately similar to (332)AT1G17810| Symbols: BETA-TIP | BETA-TIP (BETA-TONOPLAST INTRINSIC PROTEIN); water chan	
EX030008	1.887	no similarity	
EV212682	1.887	no similarity	
EE566996	1.886	no similarity	
EV198952	1.884	weakly similar to (127)AT3G57810| Symbols: | OTU-like cysteine protease family protein | chr3:21427313-21428786 FORWARD [21	
ES968507	1.882	no similarity	
EV002408	1.882	no similarity	
EL587213	1.882	no similarity	
AM059211	1.882	no similarity	
EE567172	1.881	no similarity	
EE561058	1.881	no similarity	
EE559320	1.880	no similarity	
EE441925	1.880	no similarity	-1.496
EV091905	1.880	no similarity	
EV224947	1.880	moderately similar to (281)AT5G56190| Symbols: | WD-40 repeat family protein | chr5:22759880-22762135 FORWARD [21493] 16	1.502
JCVI_2893	1.880	moderately similar to (402)AT5G62180| Symbols: ATCXE20 | ATCXE20 (ARABIDOPSIS THALIANA CARBOXYESTERASE 20);	
JCVI_29162	1.880	highly similar to (594)AT1G24530| Symbols: | transducin family protein / WD-40 repeat family protein | chr1:8693274-8694530 FOR	
JCVI_33081	1.880	moderately similar to (238)AT1G29900| Symbols: CARB | CARB (CARBAMOYL PHOSPHATE SYNTHETASE B); ATP binding /	
CN827556	1.879	moderately similar to (350)AT1G30810| Symbols: | transcription factor jumonji (jmj) family protein / zinc finger (C5HC2 type) family	
RC_EV169393	1.879	no similarity	
JCVI_35396	1.878	no original description	
EE555609	1.877	no similarity	
EE535427	1.877	no similarity	
RC_L38119	1.875	no similarity	1.290
DY006402	1.875	weakly similar to (115)AT5G08260| Symbols: SCPL35 | SCPL35 (serine carboxypeptidase-like 35); serine carboxypeptidase | chr5:265	
JCVI_8107	1.875	no original description	
JCVI_15635	1.874	moderately similar to (275)AT5G62020| Symbols: HSFB2A, AT-HSFB2A | AT-HSFB2A (Arabidopsis thaliana heat shock transcriptio	
EV116513	1.873	weakly similar to (150)AT1G19770| Symbols: ATPUP14 | ATPUP14 (Arabidopsis thaliana purine permease 14); purine transmembran	
EX067845	1.871	weakly similar to (150)AT5G67010| Symbols: | AP2 domain-containing transcription factor, putative | chr5:26766284-26766842 REV	
JCVI_40120	1.871	no original description	
RC_ES969136	1.870	no similarity	
JCVI_23844	1.869	no original description	
EV165796	1.866	no similarity	
DY024008	1.865	no similarity	
JCVI_11671	1.865	no original description	
EX020287	1.865	weakly similar to (133)AT1G09420| Symbols: G6PD4 | G6PD4 (GLUCOSE-6-PHOSPHATE DEHYDROGENASE 4); glucose-6-pho	
JCVI_39013	1.865	weakly similar to (177)AT1G69935| Symbols: | similar to unknown protein [Arabidopsis thaliana] (TAIR:AT4G33780.1); similar to un	
EV145554	1.864	no similarity	2.527
EV091919	1.864	weakly similar to (123)AT1G14410| Symbols: ATWHY1, PTAC1 | ATWHY1/PTAC1 (A. THALIANA WHIRLY 1); DNA binding / t	
RC_EV001684	1.864	no similarity	
RC_L46581	1.864	no similarity	
EV095136	1.863	no similarity	2.508
EV101256	1.861	no similarity	
EV226837	1.861	no similarity	
JCVI_20838	1.860	no original description	
JCVI_23101	1.860	no original description	
EV136808	1.860	no similarity	
ES911858	1.858	moderately similar to (461)AT4G19680| Symbols: IRT2 | IRT2 (iron-responsive transporter 2); iron ion transmembrane transporter/ zin	
JCVI_26452	1.858	weakly similar to (184)AT3G26120| Symbols: TEL1 | TEL1 (TERMINAL EAR1-LIKE 1); RNA binding | chr3:9547635-9550423 FO	
ES901851	1.858	moderately similar to (390)AT5G05570| Symbols: | transducin family protein / WD-40 repeat family protein | chr5:1656767-1663729	
JCVI_592	1.856	moderately similar to (252)AT1G15860| Symbols: | calcium ion binding | chr1:5455050-5456735 FORWARD no original description	
EE556882	1.856	no similarity	
EX032619	1.856	no similarity	
JCVI_35354	1.855	no original description	
JCVI_14118	1.855	moderately similar to (284)AT4G23050| Symbols: | protein kinase, putative | chr4:12080123-12083719 FORWARD no original descri	
EE564522	1.854	no similarity	
EV065223	1.853	no similarity	
EV157628	1.852	weakly similar to (182)AT1G14460| Symbols: | DNA polymerase-related | chr1:4948957-4952745 REVERSE [21484]	
JCVI_41752	1.852	weakly similar to (135)AT3G13350| Symbols: | high mobility group (HMG1/2) family protein / ARID/BRIGHT DNA-binding domain	
CX272501	1.852	no similarity	4.669
JCVI_31911	1.851	no original description	
JCVI_25083	1.851	no original description	
JCVI_5217	1.851	moderately similar to (223)AT5G04150| Symbols: BHLH101 | BHLH101; DNA binding / transcription factor | chr5:1138560-1139477	1.185
EV068666	1.849	moderately similar to (313)AT3G09090| Symbols: DEX1 | DEX1 (DEFECTIVE IN EXINE FORMATION 1) | chr3:2783170-2787557	
EE566491	1.848	no similarity	
JCVI_23973	1.847	no original description	
JCVI_31530	1.846	no original description	2.297

JCVI_18647	1.845	weakly similar to (179)AT3G02550| Symbols: LBD41 | LBD41 (LOB DOMAIN-CONTAINING PROTEIN 41) | chr3:536754-537657	
JCVI_17337	1.845	moderately similar to (210)AT1G32750| Symbols: TAF1, HAF1, HAC13, GTD1, HAF01 | HAF01 (HISTONE ACETYLTRANSFER/	2.302
JCVI_5778	1.845	moderately similar to (392)AT2G37210| Symbols: | Encodes a protein of unknown function. It has been crystallized and shown to be s	
EE502373	1.844	no similarity	
EV135177	1.843	weakly similar to (144)AT4G16530| Symbols: | binding | chr4:9311378-9315576 FORWARD [21481] 1 461 751	
JCVI_1506	1.843	weakly similar to (181)AT3G02470| Symbols: SAMDC | SAMDC (S-ADENOSYLMETHIONINE DECARBOXYLASE) | chr3:51023	
CD817935	1.843	no similarity	
ES982268	1.843	no similarity	
EV146998	1.842	no similarity	
JCVI_20973	1.841	weakly similar to (150)AT2G45140| Symbols: | vesicle-associated membrane protein, putative / VAMP, putative | chr2:18618104-1862	
ES982756	1.839	no similarity	
JCVI_37318	1.838	moderately similar to (267)AT5G63370| Symbols: | protein kinase family protein | chr5:25402180-25403616 REVERSEweakly simila	
ES930133	1.838	very weakly similar to (100)AT4G20880| Symbols: | ethylene-responsive nuclear protein / ethylene-regulated nuclear protein (ERT2) | c	
EX099443	1.838	no similarity	
CN827568	1.838	moderately similar to (252)AT5G23720| Symbols: PHS1 | PHS1 (PROPYZAMIDE-HYPERSENSITIVE 1); protein tyrosine/serine/thr	
EV221042	1.837	no similarity	
EV205064	1.837	no similarity	
JCVI_32240	1.836	no original description	
JCVI_14542	1.836	no original description	3.435
EV112284	1.836	no similarity	2.498
EE560671	1.835	no similarity	
H07703	1.833	no similarity	3.341
JCVI_7791	1.832	no original description	
CD815331	1.831	no similarity	
JCVI_36009	1.831	no original description	
JCVI_23709	1.830	moderately similar to (301)AT2G36020| Symbols: HVA22J | HVA22J (HVA22-LIKE PROTEIN J) | chr2:15130503-15132219 REVE	3.316
ES267439	1.829	no similarity	
JCVI_15545	1.829	no original description	
EE557056	1.828	no similarity	3.742
JCVI_12764	1.828	moderately similar to (265)AT4G17730| Symbols: ATSYP23, SYP23 | SYP23 (syntaxin 23) | chr4:9865364-9866730 FORWARD no	
JCVI_24351	1.828	no original description	
EE559717	1.827	no similarity	
RC_EV109019	1.827	no similarity	
CD839772	1.827	weakly similar to (140)AT2G22170| Symbols: | lipid-associated family protein | chr2:9434090-9434822 REVERSE [13982]	
EV159377	1.827	moderately similar to (420)AT3G28430| Symbols: | similar to unnamed protein product [Vitis vinifera] (GB:CAO69571.1); similar to (	
EE463115	1.827	no similarity	3.436
EV144905	1.826	no similarity	
EV130759	1.826	no similarity	2.779
ES939515	1.824	moderately similar to (231)AT1G76250| Symbols: | similar to unnamed protein product [Vitis vinifera] (GB:CAO62018.1) | chr1:2861	
JCVI_18184	1.824	no original description	
AM394657	1.824	no similarity	
JCVI_11535	1.824	moderately similar to (447)AT2G22490| Symbols: CYCD2;1 | CYCD2;1 (CYCLIN D2;1); cyclin-dependent protein kinase regulator/ p	
EV146941	1.823	no similarity	
H74903	1.822	no similarity	
EX127610	1.822	no similarity	
EV123932	1.821	moderately similar to (412)AT2G26440| Symbols: | pectinesterase family protein | chr2:11254485-11256485 FORWARDmoderately s	
EV176822	1.820	moderately similar to (347)AT5G11490| Symbols: | adaptin family protein | chr5:3671965-3676145 FORWARD [21487] 80 1092 109	
EV101497	1.818	no similarity	-1.186
JCVI_27423	1.818	weakly similar to (149)AT1G14910| Symbols: | epsin N-terminal homology (ENTH) domain-containing protein | chr1:5139923-51435	
JCVI_37307	1.818	very weakly similar to (100)AT3G29000| Symbols: | calcium-binding EF hand family protein | chr3:11007028-11007612 FORWARD	
JCVI_29635	1.817	moderately similar to (327)AT3G47360| Symbols: ATHSD3 | ATHSD3 (HYDROXYSTEROID DEHYDROGENASE 3); oxidoreduct	
EE413535	1.817	very weakly similar to (82.0)AT3G17520| Symbols: | late embryogenesis abundant domain-containing protein / LEA domain-containing	2.782
ES933407	1.816	weakly similar to (119)AT5G47690| Symbols: | binding | chr5:19335125-19344240 FORWARD [20143] 1 357 371	
EE439537	1.815	no similarity	
EE563418	1.814	no similarity	
EX089824	1.814	no similarity	2.195
JCVI_34109	1.814	no original description	
BQ704694	1.813	no similarity	
JCVI_38866	1.812	no original description	1.893
EE502258	1.812	no similarity	
EV180758	1.812	weakly similar to (144)AT1G21890| Symbols: | nodulin MtN21 family protein | chr1:7682797-7685570 REVERSE [21487] 16 547 73	
EV223885	1.811	weakly similar to (105)AT5G13820| Symbols: ATBP-1, ATBP1, ATTBP1, HPPBF-1, TBP1 | TBP1 (TELOMERIC DNA BINDING P	
H07316	1.811	no similarity	
EV098557	1.811	no similarity	3.483
JCVI_16389	1.811	weakly similar to (142)AT4G08670| Symbols: | protease inhibitor/seed storage/lipid transfer protein (LTP) family protein | chr4:55367	
JCVI_29569	1.810	moderately similar to (296)AT4G26720| Symbols: EP124, PPX-1, EP129, PPX1 | PPX1 (protein phosphatase x-1); protein serine/threo	
CX271936	1.810	moderately similar to (222)AT3G58820| Symbols: | F-box family protein | chr3:21763937-21765592 FORWARD [16815]	
ES968707	1.809	no similarity	
JCVI_20770	1.809	weakly similar to (145)AT2G20835| Symbols: | similar to unknown protein [Arabidopsis thaliana] (TAIR:AT3G15534.1); similar to un	
RC_ES967741	1.808	no similarity	
JCVI_28364	1.808	moderately similar to (461)AT5G45780| Symbols: | leucine-rich repeat transmembrane protein kinase, putative | chr5:18584173-18586	4.279
RC_EE558983	1.808	no similarity	
EE426054	1.808	weakly similar to (179)AT1G72570| Symbols: | DNA binding / transcription factor | chr1:27335043-27337360 FORWARD [20189]	
JCVI_10440	1.807	weakly similar to (198)AT1G47270| Symbols: AtTLP6 | AtTLP6 (TUBBY LIKE PROTEIN 6); phosphoric diester hydrolase/ transcript	
CO750001	1.807	moderately similar to (217)AT4G04570| Symbols: | protein kinase family protein | chr4:2290043-2292253 FORWARD [16161]	
JCVI_21051	1.806	no original description	
JCVI_9657	1.804	moderately similar to (355)AT3G03420| Symbols: | Ku70-binding family protein | chr3:812534-813651 FORWARD no original descri	
EE527123	1.801	no similarity	
JCVI_4685	1.801	moderately similar to (277)AT5G11160| Symbols: APT5 | APT5 (ADENINE PHOSPHORIBOSYLTRANSFERASE 5); adenine phosp	
JCVI_27186	1.801	weakly similar to (199)AT4G27280| Symbols: | calcium-binding EF hand family protein | chr4:13663776-13664168 REVERSE no ori	
RC_JCVI_34456	1.801	no original description	
JCVI_15360	1.800	weakly similar to (155)AT5G01960| Symbols: | zinc finger (C3HC4-type RING finger) family protein | chr5:370808-372772 FORWA	
EE559149	1.799	no similarity	-1.554

JCVI_10145	1.799	highly similar to (831)AT3G25840| Symbols: | protein kinase family protein | chr3:9454230-9457559 REVERSEweakly similar to (12	
JCVI_3479	1.799	moderately similar to (325)AT2G25150| Symbols: | transferase family protein | chr2:10709661-10711345 REVERSE no original descr	
RC_EE501888	1.798	no similarity	
EV063739	1.798	no similarity	
JCVI_27803	1.798	moderately similar to (281)AT2G24210| Symbols: TPS10 | TPS10 (TERPENE SYNTHASE 10); myrcene/(E)-beta-ocimene synthase |	
RC_CN735168	1.798	no similarity	
EE547059	1.794	no similarity	2.271
EE546377	1.793	no similarity	
JCVI_22353	1.792	very weakly similar to (85.5)AT5G48140| Symbols: | polygalacturonase, putative / pectinase, putative | chr5:19536133-19537638 REV	
EV110965	1.792	no similarity	
EE558884	1.791	no similarity	
JCVI_545	1.791	no original description	
RC_AM057182	1.790	no similarity	
JCVI_30427	1.788	moderately similar to (234)AT1G18740| Symbols: | similar to unknown protein [Arabidopsis thaliana] (TAIR:AT1G74450.1); similar t	
JCVI_4001	1.787	highly similar to (642)AT5G62790| Symbols: PDE129, DXR | DXR (1-DEOXY-D-XYLULOSE 5-PHOSPHATE REDUCTOISOMEI	
EX038628	1.786	moderately similar to (364)AT1G51410| Symbols: | cinnamyl-alcohol dehydrogenase, putative (CAD) | chr1:19063553-19065092 FOR	
EE502143	1.783	moderately similar to (277)AT4G02080| Symbols: ATSARA1C, ATSAR2, ASAR1 | ASAR1 (Arabidopsis thaliana secretion-associated	1.774
JCVI_31190	1.783	no original description	
EE392256	1.783	moderately similar to (216)AT1G02690| Symbols: | importin alpha-2 subunit, putative | chr1:584397-587036 FORWARDweakly simil	3.133
EV047330	1.782	no similarity	
EV226389	1.782	no similarity	
EE568299	1.781	no similarity	1.751
CD828081	1.781	no similarity	
JCVI_9944	1.780	very weakly similar to (95.9)AT2G33520| Symbols: | similar to proline-rich family protein [Arabidopsis thaliana] (TAIR:AT1G12810.1	
EE473185	1.780	no similarity	
JCVI_15249	1.779	moderately similar to (325)AT3G17810| Symbols: | dihydroorotate dehydrogenase family protein / dihydroorotate oxidase family prote	
JCVI_39917	1.779	weakly similar to (121)AT1G70410| Symbols: | carbonic anhydrase, putative / carbonate dehydratase, putative | chr1:26537830-265411	
EV135881	1.778	no similarity	
JCVI_29424	1.777	no original description	
EV149959	1.777	no similarity	2.858
CD812606	1.777	weakly similar to (129)AT3G44110| Symbols: ATJ, ATJ3 | ATJ3 (Arabidopsis thaliana DnaJ homologue 3) | chr3:15880166-15882046	
EE564414	1.776	no similarity	
JCVI_27747	1.776	moderately similar to (425)AT5G25430| Symbols: | anion exchange protein family | chr5:8851254-8854262 FORWARD no original de	
EE568267	1.776	no similarity	
ES954012	1.775	no similarity	
AM387291	1.774	no similarity	
JCVI_10057	1.773	moderately similar to (396)AT5G48100| Symbols: LAC15, TT10 | TT10 (TRANSPARENT TESTA 10); laccase | chr5:19506756-1950	
JCVI_32184	1.771	weakly similar to (135)AT2G28430| Symbols: | similar to unknown [Populus trichocarpa] (GB:ABK94614.1) | chr2:12166813-121674	
JCVI_12087	1.770	no original description	
JCVI_630	1.770	no original description	
EV215200	1.768	no similarity	
EV151095	1.768	no similarity	
JCVI_27431	1.766	no original description	
JCVI_41634	1.765	no original description	
EV075020	1.765	no similarity	
JCVI_32346	1.764	moderately similar to (456)AT5G37020| Symbols: ARF8 | ARF8 (AUXIN RESPONSE FACTOR 8) | chr5:14647381-14651146 FORV	4.621
EE559769	1.763	no similarity	
AM394870	1.763	no similarity	
EV165884	1.763	weakly similar to (126)AT4G38170| Symbols: FRS9 | FRS9 (FAR1-related sequence 9); zinc ion binding | chr4:17904607-17906433 F	
EE502259	1.762	no similarity	
EE567435	1.761	weakly similar to (117)AT5G22880| Symbols: H2B, HTB2 | H2B/HTB2 (HISTONE H2B); DNA binding | chr5:7652133-7652570 RE	
JCVI_12315	1.760	moderately similar to (216)AT1G05600| Symbols: | pentatricopeptide (PPR) repeat-containing protein | chr1:1672160-1673674 FORW	
ES964533	1.760	no similarity	
JCVI_12680	1.760	moderately similar to (327)AT1G52600| Symbols: | signal peptidase, putative | chr1:19594281-19596155 FORWARD no original desc	
EV128496	1.759	no similarity	
ES966380	1.758	no similarity	
CD826220	1.758	no similarity	
JCVI_31755	1.757	moderately similar to (391)AT1G18800| Symbols: NRP2 | NRP2 (NAP1-RELATED PROTEIN 2); DNA binding / chromatin binding /	
EE564779	1.757	no similarity	
JCVI_11316	1.756	weakly similar to (130)AT5G44540| Symbols: | tapetum-specific protein-related | chr5:17958514-17958873 REVERSE no original des	
JCVI_38387	1.755	moderately similar to (225)AT2G36460| Symbols: | fructose-bisphosphate aldolase, putative | chr2:15304008-15304811 REVERSEmo	2.523
RC_AT000660	1.754	no similarity	
EX019686	1.753	weakly similar to (169)AT1G02730| Symbols: CSLD5, ATCSLD5 | ATCSLD5 (CELLULOSE SYNTHASE-LIKE D5); 1,4-beta-D-xy	
EE491541	1.752	no similarity	
JCVI_11306	1.752	moderately similar to (466)AT1G17840| Symbols: WBC11, ABCG11, DSO, COF1 | ABCG11/COF1/DSO/WBC11 (DESPERADO);	
EE562280	1.751	no similarity	2.478
EE479477	1.751	no similarity	
H06424	1.751	no similarity	
CD825434	1.751	no similarity	
JCVI_32964	1.751	no original description	
JCVI_21823	1.750	no original description	
JCVI_41609	1.750	weakly similar to (155)AT4G04614| Symbols: | similar to unnamed protein product [Vitis vinifera] (GB:CAO22561.1); similar to hypo	1.279
EV225197	1.750	no similarity	
EX045323	1.749	moderately similar to (296)AT3G05580| Symbols: | serine/threonine protein phosphatase, putative | chr3:1618222-1619856 REVERSE	
EE449338	1.749	no similarity	
EV060159	1.749	no similarity	
EV125321	1.748	moderately similar to (464)AT2G22070| Symbols: | pentatricopeptide (PPR) repeat-containing protein | chr2:9390682-9393042 FORW	
CV546087	1.748	no similarity	
EV085386	1.748	no similarity	
EV147391	1.748	no similarity	
ES947980	1.747	no similarity	
EV103116	1.746	no similarity	
EE403898	1.745	weakly similar to (119)AT5G10820| Symbols: | integral membrane transporter family protein | chr5:3420877-3423167 REVERSE [16	

JCVI_11723	1.744	no original description	
EV176810	1.744	no similarity	
EV057037	1.744	moderately similar to (234)AT4G11850| Symbols: MEE54 | PLDGAMMA1 (maternal effect embryo arrest 54); phospholipase D | chr4	
AM387499	1.743	no similarity	1.791
JCVI_292	1.742	moderately similar to (284)AT2G17480| Symbols: ATMLO8, MLO8 | MLO8 (MILDEW RESISTANCE LOCUS O 8); calmodulin bin	
EE468849	1.741	very weakly similar to (95.9)AT4G23160| Symbols: | protein kinase family protein | chr4:12129496-12134097 FORWARD [20156]	
JCVI_40662	1.740	moderately similar to (253)AT5G24470| Symbols: PRR5, APRR5 | APRR5 (PSEUDO-RESPONSE REGULATOR 5); transcription re	
JCVI_23700	1.739	no original description	
JCVI_29668	1.737	no original description	
EV091712	1.737	weakly similar to (182)AT1G72050| Symbols: | zinc finger (C2H2 type) family protein | chr1:27119470-27120890 FORWARD [2147	
EE562464	1.735	no similarity	
EE568820	1.734	no similarity	
EV178478	1.734	no similarity	
JCVI_41484	1.733	no original description	
EV108402	1.733	no similarity	3.158
EV101393	1.732	moderately similar to (201)AT4G02500| Symbols: ATXT2 | ATXT2; UDP-xylosyltransferase/ transferase/ transferase, transferring glyc	
EE533412	1.732	no similarity	
JCVI_4697	1.732	weakly similar to (193)AT2G47460| Symbols: MYB12, ATMYB12 | ATMYB12/MYB12 (MYB DOMAIN PROTEIN 12); DNA bind	2.682
EE563050	1.732	no similarity	
EV104299	1.731	no similarity	
EV104369	1.731	weakly similar to (113)AT1G77920| Symbols: | bZIP family transcription factor | chr1:29303853-29305501 FORWARD [21478] 1 38	
RC_EE462179	1.730	no similarity	
ES979266	1.730	no similarity	
JCVI_3468	1.730	moderately similar to (499)AT1G68530| Symbols: CER6, G2, POP1, CUT1 | CUT1 (CUTICULAR 1); catalytic | chr1:25717263-2571	
JCVI_12686	1.728	very weakly similar to (92.0)AT3G18295| Symbols: | similar to unknown protein [Arabidopsis thaliana] (TAIR:AT1G48770.1); similar	
CD832055	1.727	no similarity	
ES992740	1.727	very weakly similar to (95.5)AT5G51150| Symbols: | similar to unknown protein [Arabidopsis thaliana] (TAIR:AT1G34630.1); similar	
H07440	1.727	no similarity	
EX044792	1.726	weakly similar to (144)AT4G13710| Symbols: | pectate lyase family protein | chr4:7962546-7966008 FORWARDvery weakly similar t	
CX188493	1.726	no similarity	
EV033347	1.726	no similarity	
JCVI_19655	1.723	no original description	
JCVI_13277	1.723	no original description	
ES904390	1.722	moderately similar to (331)AT3G51340| Symbols: | pepsin A | chr3:19067992-19070767 REVERSE [21432]	
EE561202	1.722	no similarity	
EV152311	1.722	no similarity	
EE502361	1.722	no similarity	
JCVI_33258	1.721	moderately similar to (204)AT3G07150| Symbols: | similar to hypothetical protein [Vitis vinifera] (GB:CAN68821.1) | chr3:2263812-2	
EE562017	1.721	no similarity	
JCVI_20854	1.721	moderately similar to (416)AT3G27170| Symbols: ATCLC-B, CLC-B | CLC-B (chloride channel protein B); anion channel/ voltage-gat	
RC_EV171149	1.720	no similarity	
JCVI_36881	1.720	very weakly similar to (83.6)AT4G20870| Symbols: | fatty acid hydroxylase, putative | chr4:11174631-11175889 REVERSE no origina	
EE469983	1.720	no similarity	
ES912522	1.719	weakly similar to (103)AT4G22640| Symbols: | similar to unknown protein [Arabidopsis thaliana] (TAIR:AT4G22666.1); similar to un	
EV217391	1.719	no similarity	
EV145829	1.719	no similarity	
EV227344	1.718	very weakly similar to (94.0)AT3G57050| Symbols: CBL | CBL (CYSTATHIONINE BETA-LYASE) | chr3:21122918-21125500 REV	
EV115639	1.717	weakly similar to (170)AT2G46860| Symbols: ATPPA3 | ATPPA3 (ARABIDOPSIS THALIANA PYROPHOSPHORYLASE 3); inorg	
RC_CV974015	1.717	no similarity	
H07193	1.717	no similarity	
EV024333	1.717	no similarity	
EV102580	1.716	no similarity	
JCVI_40354	1.715	no original description	
EV189801	1.714	moderately similar to (217)AT1G18420| Symbols: | similar to ATALMT9 (ALUMINUM-ACTIVATED MALATE TRANSPORTER 9	
CN728159	1.712	no similarity	
EV223123	1.712	no similarity	
JCVI_29879	1.712	moderately similar to (253)AT2G17510| Symbols: EMB2763 | EMB2763 (EMBRYO DEFECTIVE 2763); RNA binding / ribonuclease	
EV043014	1.711	no similarity	1.780
EE557448	1.711	no similarity	
JCVI_12956	1.710	no original description	2.235
JCVI_25865	1.708	weakly similar to (154)AT5G22070| Symbols: | similar to unknown protein [Arabidopsis thaliana] (TAIR:AT3G52060.2); similar to un	
JCVI_29079	1.707	no original description	
JCVI_448	1.707	moderately similar to (362)AT3G23080| Symbols: | similar to unknown protein [Arabidopsis thaliana] (TAIR:AT4G14500.1); similar t	
DY014788	1.707	very weakly similar to (99.0)AT1G03830| Symbols: | guanylate-binding family protein | chr1:962127-966621 REVERSE [18966]	
JCVI_32596	1.707	moderately similar to (316)AT5G46910| Symbols: | transcription factor jumonji (jmj) family protein | chr5:19065007-19068107 FORW	
JCVI_8823	1.706	weakly similar to (182)AT5G20830| Symbols: SUS1, ASUS1, ATSUS1 | SUS1 (SUCROSE SYNTHASE 1); UDP-glycosyltransferase/	
RC_CD836255	1.706	no similarity	1.402
JCVI_1810	1.705	moderately similar to (416)AT3G07680| Symbols: | emp24/gp25L/p24 family protein | chr3:2455633-2456658 FORWARD no origina	
EV191047	1.705	no similarity	
JCVI_12735	1.704	moderately similar to (457)AT4G28520| Symbols: CRC, CRU3 | CRU3 (CRUCIFERIN 3) | chr4:14087602-14089235 FORWARDhig	
JCVI_8469	1.703	moderately similar to (401)AT4G38160| Symbols: PDE191 | PDE191 (PIGMENT DEFECTIVE 191) | chr4:17902406-17903778 FOR	
BG544144	1.701	very weakly similar to (97.1)AT5G05670| Symbols: | signal recognition particle binding | chr5:1695917-1697535 REVERSE [8791] 1 4	
JCVI_24437	1.700	moderately similar to (296)AT4G33920| Symbols: | protein phosphatase 2C family protein / PP2C family protein | chr4:16260881-162	
CX192092	1.700	weakly similar to (125)AT2G33690| Symbols: | late embryogenesis abundant protein, putative / LEA protein, putative | chr2:14259275-	
EE559528	1.700	no similarity	
EE559687	1.699	no similarity	
JCVI_10809	1.698	moderately similar to (253)AT1G28590| Symbols: | lipase, putative | chr1:10047495-10049286 REVERSEvery weakly similar to (84.0	2.475
CX270121	1.698	weakly similar to (176)AT1G18190| Symbols: GC2 | GC2 (GOLGIN CANDIDATE 2) | chr1:6257956-6261318 REVERSE [16815] 1:	
EX132152	1.698	moderately similar to (242)AT1G80820| Symbols: CCR2 | CCR2 (CINNAMOYL COA REDUCTASE) | chr1:30375538-30377352 FO	
EV089702	1.697	weakly similar to (114)AT3G19170| Symbols: ATPREP1, ATZNMP | ATPREP1/ATZNMP (PRESEQUENCE PROTEASE 1); metall	
EE502180	1.697	weakly similar to (193)AT5G51040| Symbols: | similar to unnamed protein product [Vitis vinifera] (GB:CAO23083.1); contains InterP	
JCVI_38753	1.695	moderately similar to (479)AT4G26010| Symbols: | peroxidase, putative | chr4:13200662-13201697 FORWARDmoderately similar to	
EB041761	1.694	no similarity	

EE501998	1.694	weakly similar to (137)AT2G23090| Symbols: | Identical to Uncharacterized protein At2g23090 [Arabidopsis Thaliana] (GB:O64818);	-1.959
RC_ES948491	1.694	no similarity	
JCVI_27817	1.694	moderately similar to (362)AT3G44050| Symbols: | kinesin motor protein-related | chr3:15829725-15835779 FORWARD no original	
EX138069	1.693	moderately similar to (471)AT4G08040| Symbols: ACS11 | ACS11 (1-Amino-cyclopropane-1-carboxylate synthase 11); 1-aminocyclo	
EV128803	1.693	no similarity	1.910
EE566677	1.691	no similarity	
EV207729	1.691	no similarity	
RC_EE559934	1.691	no similarity	2.191
EE561150	1.690	no similarity	6.596
CO749673	1.689	no similarity	
JCVI_36133	1.689	moderately similar to (416)AT3G49600| Symbols: UBP26 | UBP26 (ubiquitin-specific protease 26); ubiquitin-specific protease | chr3:1	
EV132438	1.689	no similarity	
RC_JCVI_42214	1.689	no original description	
JCVI_15454	1.688	weakly similar to (163)AT2G18300| Symbols: | basic helix-loop-helix (bHLH) family protein | chr2:7960104-7961528 REVERSE no	
EE563413	1.688	no similarity	2.896
EV054789	1.688	no similarity	
H74693	1.688	no similarity	
EV227465	1.687	no similarity	
EV226810	1.686	weakly similar to (167)AT4G25630| Symbols: ATFIB2, FIB2 | FIB2 (FIBRILLARIN 2) | chr4:13074248-13076214 FORWARD [214	
JCVI_25142	1.686	no original description	
JCVI_2540	1.686	very weakly similar to (84.0)AT5G59960| Symbols: | similar to unnamed protein product [Vitis vinifera] (GB:CAO21698.1) | chr5:241	
CX267699	1.686	weakly similar to (156)AT1G11880| Symbols: | similar to unnamed protein product [Vitis vinifera] (GB:CAO22037.1); contains InterP	
DN962582	1.684	no similarity	
DN961527	1.683	moderately similar to (243)AT5G48790| Symbols: | similar to unknown protein [Arabidopsis thaliana] (TAIR:AT1G73060.1); similar t	
RC_EX016781	1.683	no similarity	
JCVI_39273	1.682	no original description	
EE415349	1.682	no similarity	
JCVI_6044	1.681	no original description	
EV111606	1.681	no similarity	
JCVI_38215	1.679	moderately similar to (228)AT3G54770| Symbols: | RNA recognition motif (RRM)-containing protein | chr3:20284841-20286709 RE	
EE567363	1.677	no similarity	5.290
JCVI_32610	1.676	weakly similar to (184)AT2G25360| Symbols: | zinc finger protein-related | chr2:10811328-10812840 FORWARD no original descript	
JCVI_1222	1.674	highly similar to (583)AT1G13260| Symbols: RAV1 | RAV1 (Related to ABI3/VP1 1); DNA binding / transcription factor | chr1:45423	
JCVI_27148	1.674	moderately similar to (218)AT4G21710| Symbols: EMB1989, RPB2, NRPB2 | NRPB2 (EMBRYO DEFECTIVE 1989); DNA binding	-1.574
JCVI_41873	1.673	weakly similar to (110)AT3G13677| Symbols: | similar to unknown [Populus trichocarpa] (GB:ABK93532.1) | chr3:4476303-4477099	
JCVI_6876	1.672	moderately similar to (283)AT3G46550| Symbols: SOS5 | SOS5 (SALT OVERLY SENSITIVE 5) | chr3:17147597-17148859 REVER	
EV122790	1.671	very weakly similar to (82.4)AT2G03120| Symbols: | signal peptide peptidase family protein | chr2:937551-940080 FORWARD [2147	
JCVI_31056	1.671	weakly similar to (126)AT4G05010| Symbols: | F-box family protein | chr4:2567472-2568160 FORWARD no original description	
EV218355	1.669	no similarity	
JCVI_9205	1.669	weakly similar to (106)AT2G14900| Symbols: | gibberellin-regulated family protein | chr2:6411292-6412125 FORWARD no original	
JCVI_40876	1.667	no original description	
EE566526	1.666	no similarity	
JCVI_17594	1.666	weakly similar to (132)AT3G01500| Symbols: CA1 | CA1 (CARBONIC ANHYDRASE 1); carbonate dehydratase/ zinc ion binding | c	
ES993383	1.665	no similarity	1.274
EX025934	1.664	moderately similar to (264)AT5G38100| Symbols: | methyltransferase-related | chr5:15217014-15218398 REVERSE [21810] 1 661 67	
ES980004	1.664	no similarity	
JCVI_23839	1.663	weakly similar to (137)AT1G65020| Symbols: | similar to unnamed protein product [Vitis vinifera] (GB:CAO62149.1); contains InterP	
CD822668	1.662	no similarity	
CX280196	1.662	no similarity	
EV005095	1.661	weakly similar to (129)AT3G19100| Symbols: | calcium-dependent protein kinase, putative / CDPK, putative | chr3:6605687-6608986	
EV099418	1.661	moderately similar to (211)AT1G74170| Symbols: | protein binding | chr1:27895216-27899102 REVERSE [21477]	2.601
JCVI_18828	1.661	moderately similar to (350)AT2G35610| Symbols: | similar to unknown protein [Arabidopsis thaliana] (TAIR:AT1G70630.1); similar t	
AM390087	1.661	weakly similar to (130)ATMG00730| Symbols: COX3 | Encodes cytochrome c oxidase subunit 3. | chrM:218280-219077 FORWARDv	
EV159516	1.661	moderately similar to (318)AT2G35320| Symbols: ATEYA | ATEYA (ARABIDOPSIS THALIANA EYES ABSENT HOMOLOG); p	
EV056990	1.661	weakly similar to (127)AT5G01860| Symbols: | zinc finger (C2H2 type) family protein | chr5:335627-336274 FORWARD [21442]	
JCVI_31810	1.655	moderately similar to (241)AT5G61170| Symbols: | 40S ribosomal protein S19 (RPS19C) | chr5:24628384-24629428 FORWARDwea	
JCVI_10781	1.655	moderately similar to (294)AT1G20816| Symbols: | similar to unknown protein [Arabidopsis thaliana] (TAIR:AT1G76405.2); similar t	
EE564467	1.654	no similarity	
RC_EE567283	1.653	no similarity	
JCVI_20231	1.653	weakly similar to (101)AT3G10800| Symbols: BZIP28 | BZIP28; DNA binding / transcription factor | chr3:3379331-3381435 FORWA	
ES980826	1.653	weakly similar to (118)AT5G08460| Symbols: | GDSL-motif lipase/hydrolase family protein | chr5:2733221-2735352 FORWARD [21	
EV099882	1.652	weakly similar to (119)AT1G31810| Symbols: | actin binding | chr1:11399903-11405742 REVERSE [21477]	
AT000440	1.652	no similarity	
EV165402	1.652	no similarity	
EV213685	1.651	moderately similar to (210)AT4G24500| Symbols: | hydroxyproline-rich glycoprotein family protein | chr4:12658742-12660221 FOR	3.189
JCVI_27326	1.649	moderately similar to (406)AT3G33530| Symbols: | transducin family protein / WD-40 repeat family protein | chr3:14096340-1410443	
EV166336	1.648	moderately similar to (235)AT5G57100| Symbols: | transporter-related | chr5:23123789-23125666 REVERSE [21486]	
EE450638	1.648	weakly similar to (155)AT3G07340| Symbols: | basic helix-loop-helix (bHLH) family protein | chr3:2341194-2343294 REVERSE [20	
JCVI_31999	1.648	no original description	2.049
JCVI_36145	1.646	weakly similar to (166)AT5G08460| Symbols: | GDSL-motif lipase/hydrolase family protein | chr5:2733221-2735352 FORWARD no	
JCVI_3357	1.646	moderately similar to (236)AT5G20130| Symbols: | similar to unnamed protein product [Vitis vinifera] (GB:CAO21131.1) | chr5:6797	
EV172610	1.645	no similarity	
RC_ES965115	1.644	no similarity	
JCVI_17427	1.644	moderately similar to (230)AT4G37240| Symbols: | similar to unknown protein [Arabidopsis thaliana] (TAIR:AT2G23690.1); similar t	
EV225312	1.643	weakly similar to (148)AT2G39720| Symbols: RHC2A | RHC2A (RING-H2 finger C2A); protein binding / zinc ion binding | chr2:1657	
JCVI_5701	1.643	highly similar to (815)AT3G05910| Symbols: | pectinacetylesterase, putative | chr3:1764515-1767246 REVERSE no original descriptic	
JCVI_1424	1.643	weakly similar to (101)AT3G54360| Symbols: | protein binding / zinc ion binding | chr3:20139548-20142559 REVERSE no original d	
EX063324	1.641	very weakly similar to (81.6)AT1G60500| Symbols: | dynamin family protein | chr1:22295247-22297487 FORWARD [21814]	
JCVI_24368	1.641	no original description	
EV213935	1.641	no similarity	
JCVI_1046	1.641	no original description	
JCVI_27292	1.640	highly similar to (504)AT1G20150| Symbols: | subtilase family protein | chr1:6987323-6990352 REVERSE no original description	2.496
JCVI_487	1.638	highly similar to (586)AT1G18740| Symbols: | similar to unknown protein [Arabidopsis thaliana] (TAIR:AT1G74450.1); similar to un	1.220

DY030758	1.637	weakly similar to (111)AT1G17360| Symbols: | COP1-interacting protein-related | chr1:5947434-5951210 FORWARD [18973]	
JCVI_23476	1.637	moderately similar to (312)AT4G18050| Symbols: PGP9 | PGP9 (P-GLYCOPROTEIN 9); ATPase, coupled to transmembrane movem	2.150
EE529641	1.637	weakly similar to (104)AT5G59710| Symbols: ATVIP2, VIP2 | VIP2 (VIRE2 INTERACTING PROTEIN2); transcription regulator | ch	3.096
JCVI_5942	1.637	moderately similar to (296)AT5G01160| Symbols: | e-cadherin binding protein-related | chr5:54279-55726 FORWARD no original des	
EX134973	1.636	very weakly similar to (92.8)AT5G17920| Symbols: ATMETS, ATMS1, ATCIMS | ATCIMS (COBALAMIN-INDEPENDENT METH	
RC_EE557370	1.635	no similarity	2.225
JCVI_32278	1.635	no original description	
RC_EE566596	1.635	no similarity	
EX094943	1.635	very weakly similar to (81.6)AT1G19770| Symbols: ATPUP14 | ATPUP14 (Arabidopsis thaliana purine permease 14); purine transmem	2.582
ES952392	1.634	no similarity	
EV109547	1.634	no similarity	
EE565219	1.633	no similarity	
RC_JCVI_22611	1.632	no original description	
EV099239	1.632	weakly similar to (135)AT3G55110| Symbols: | ABC transporter family protein | chr3:20435743-20437869 REVERSE [21477]	
EE568265	1.631	no similarity	
RC_EE565891	1.631	no similarity	
JCVI_33452	1.630	moderately similar to (234)AT1G68580| Symbols: | agenet domain-containing protein / bromo-adjacent homology (BAH) domain-cont	
EV187726	1.630	no similarity	
JCVI_38563	1.630	weakly similar to (138)AT3G19580| Symbols: AZF2 | AZF2 (ARABIDOPSIS ZINC-FINGER PROTEIN 2) | chr3:6803299-6804120 F	
JCVI_35689	1.629	weakly similar to (104)AT1G17970| Symbols: | zinc finger (C3HC4-type RING finger) family protein | chr1:6185025-6187195 FORW	
AM058095	1.624	weakly similar to (113)AT1G16130| Symbols: WAKL2 | WAKL2 (WALL ASSOCIATED KINASE-LIKE 2); kinase | chr1:5525628-55	1.831
EE449463	1.624	no similarity	
JCVI_15891	1.624	no original description	
JCVI_7849	1.623	no original description	
JCVI_17175	1.623	no original description	
RC_EX018585	1.623	no similarity	
JCVI_27236	1.622	moderately similar to (234)AT3G05960| Symbols: | sugar transporter, putative | chr3:1783593-1785340 REVERSEmoderately similar	
RC_ES967426	1.622	no similarity	1.161
JCVI_19599	1.620	weakly similar to (133)AT2G35880| Symbols: | similar to unknown protein [Arabidopsis thaliana] (TAIR:AT4G32330.3); similar to un	2.524
JCVI_25017	1.620	nearly identical (1139)AT1G50200| Symbols: ACD, ALATS | ALATS (ALANYL-TRNA SYNTHETASE); ATP binding / alanine-tRN	
JCVI_14685	1.620	no original description	
JCVI_24319	1.619	no original description	
JCVI_38747	1.618	no original description	
EE561010	1.616	no similarity	
RC_JCVI_41110	1.616	no original description	
RC_JCVI_30899	1.615	no original description	
ES949733	1.614	weakly similar to (102)AT2G34320| Symbols: | nucleic acid binding | chr2:14492038-14492916 FORWARD [21393] 1 528 543	
CD842452	1.614	no similarity	
JCVI_13591	1.614	weakly similar to (184)AT2G30020| Symbols: | protein phosphatase 2C, putative / PP2C, putative | chr2:12821514-12822981 FORWA	1.692
JCVI_9015	1.614	weakly similar to (174)AT5G47320| Symbols: RPS19 | RPS19 (40S ribosomal protein S19); RNA binding | chr5:19221028-19222178	
EE567109	1.613	no similarity	
EE408517	1.612	no similarity	3.702
JCVI_6399	1.609	moderately similar to (269)AT1G19770| Symbols: ATPUP14 | ATPUP14 (Arabidopsis thaliana purine permease 14); purine transmemb	2.378
JCVI_42480	1.609	no original description	
EV178524	1.608	no similarity	
EE562863	1.606	no similarity	
JCVI_35554	1.606	moderately similar to (483)AT3G16630| Symbols: ATKINESIN-13A, KINESIN-13A | ATKINESIN-13A/KINESIN-13A; microtubule	
DN961326	1.605	moderately similar to (297)AT2G27600| Symbols: SKD1, VPS4 | SKD1/VPS4; ATP binding | chr2:11788303-11790807 FORWARDv	
EV013733	1.605	weakly similar to (157)AT5G54650| Symbols: ATFH5, Fh5 | Fh5 (FORMIN HOMOLOGY5); actin binding | chr5:22215082-2221887	
JCVI_491	1.604	moderately similar to (202)AT3G48860| Symbols: | similar to unknown protein [Arabidopsis thaliana] (TAIR:AT5G23700.1); similar t	
CV973973	1.603	no similarity	
JCVI_27908	1.602	no original description	
EE483198	1.601	no similarity	
JCVI_41234	1.601	moderately similar to (376)AT4G31910| Symbols: | transferase family protein | chr4:15441131-15443702 FORWARD no original desc	
JCVI_33249	1.601	weakly similar to (186)AT2G42110| Symbols: | similar to unknown [Populus trichocarpa] (GB:ABK94541.1); similar to unknown [Pop	
EE556047	1.600	no similarity	
EV142282	1.600	no similarity	
JCVI_25794	1.600	no original description	3.388
EV132091	1.600	no similarity	
RC_AM395286	1.598	no similarity	
EE465690	1.597	no similarity	
ES956530	1.597	no similarity	
CX188266	1.597	no similarity	
JCVI_13557	1.597	no original description	
EV146460	1.597	no similarity	
JCVI_37999	1.597	no original description	
JCVI_32042	1.596	highly similar to (572)AT4G10710| Symbols: SPT16 | SPT16 (GLOBAL TRANSCRIPTION FACTOR C); metalloexopeptidase | chr4:	
JCVI_22377	1.596	no original description	
JCVI_41374	1.596	no original description	
EV107977	1.595	no similarity	
JCVI_35945	1.595	no original description	1.634
ES941720	1.594	no similarity	2.185
JCVI_37468	1.594	very weakly similar to (81.6)AT5G60710| Symbols: | zinc finger (C3HC4-type RING finger) family protein | chr5:24428179-24432075	
JCVI_6962	1.593	moderately similar to (324)AT3G16840| Symbols: | ATP-dependent helicase | chr3:5738026-5743048 REVERSE no original descriptio	
CD813496	1.593	no similarity	
EV160049	1.593	weakly similar to (186)AT3G19360| Symbols: | zinc finger (CCCH-type) family protein | chr3:6707550-6708870 FORWARD [21484]	
JCVI_22420	1.592	no original description	
JCVI_37547	1.592	no original description	
EX094245	1.592	no similarity	
ES902651	1.590	weakly similar to (142)AT3G05490| Symbols: RALFL22 | RALFL22 (RALF-LIKE 22) | chr3:1591387-1591746 FORWARD [21432]	
EE502207	1.589	moderately similar to (246)AT3G60820| Symbols: PBF1 | PBF1 (20S proteasome beta subunit F1); peptidase | chr3:22483013-2248478	
EV041949	1.588	no similarity	1.205
EV104580	1.587	no similarity	

EV221331	1.587	weakly similar to (178)AT3G08530| Symbols: | clathrin heavy chain, putative | chr3:2587177-2595417 REVERSE [21492]	
EE558473	1.587	no similarity	
JCVI_24542	1.587	very weakly similar to (85.1)AT3G05480| Symbols: | cell cycle checkpoint control protein family | chr3:1585389-1588244 FORWARD	
JCVI_35960	1.584	highly similar to (612)AT3G54090| Symbols: | pfkB-type carbohydrate kinase family protein | chr3:20039123-20040813 FORWARDv	
EE445045	1.582	no similarity	
DQ023570	1.582	moderately similar to (268)AT3G06483| Symbols: ATPDHK, PDK | PDK (PYRUVATE DEHYDROGENASE KINASE); ATP bindin	
RC_EE561263	1.581	no similarity	
EE459120	1.581	no similarity	
JCVI_22446	1.579	moderately similar to (271)AT4G22050| Symbols: | aspartyl protease family protein | chr4:11683877-11685518 FORWARDmoderatel	
EV150296	1.579	no similarity	2.449
EV215705	1.579	no similarity	
JCVI_34831	1.579	no original description	2.501
EE562727	1.579	no similarity	
JCVI_22260	1.578	no original description	
EV109961	1.578	no similarity	
CV973968	1.578	no similarity	1.246
JCVI_41216	1.578	no original description	
EV069942	1.575	no similarity	2.939
JCVI_9130	1.574	highly similar to (517)AT1G62280| Symbols: SLAH1 | SLAH1 (SLAC1 HOMOLOGUE 1); transporter | chr1:23010974-23012205 RE	
EE563043	1.574	no similarity	
EV008788	1.574	no similarity	
EE446245	1.574	no similarity	
JCVI_42231	1.574	no original description	
CX279538	1.574	no similarity	
EV107311	1.574	moderately similar to (230)AT3G11080| Symbols: | disease resistance family protein | chr3:3470487-3473318 FORWARD [21478] 51	
EE472497	1.572	no similarity	
JCVI_41664	1.571	no original description	
JCVI_20891	1.571	moderately similar to (250)AT1G05230| Symbols: HDG2 | homeobox-leucine zipper family protein / lipid-binding START domain-con	
EE561932	1.571	no similarity	2.129
AM393924	1.570	no similarity	2.557
EE568263	1.570	no similarity	
EV178531	1.570	no similarity	
JCVI_30630	1.570	highly similar to (520)AT1G14040| Symbols: | ATP binding / ATPase, coupled to transmembrane movement of substances | chr1:4810	
EX047601	1.570	no similarity	
EV106366	1.570	no similarity	
EV094759	1.569	no similarity	
DY020476	1.568	no similarity	2.961
RC_H07300	1.568	no similarity	
EE431728	1.568	very weakly similar to (82.4)AT4G08395| Symbols: | unknown protein | chr4:5319631-5320709 FORWARD [20151]	
CX272790	1.567	no similarity	
JCVI_38889	1.567	moderately similar to (264)AT1G17620| Symbols: | similar to unknown protein [Arabidopsis thaliana] (TAIR:AT5G11890.1); similar t	
EB041727	1.566	no similarity	
RC_EV141714	1.566	no similarity	
EX119499	1.566	no similarity	
EX079614	1.565	moderately similar to (239)AT2G22260| Symbols: | oxidoreductase, 2OG-Fe(II) oxygenase family protein | chr2:9468422-9470133 FO	
JCVI_39775	1.565	weakly similar to (171)AT1G29350| Symbols: | similar to kinase-related [Arabidopsis thaliana] (TAIR:AT1G29370.1); similar to unnar	
JCVI_665	1.565	very weakly similar to (83.2)AT2G41260| Symbols: ATM17, M17 | M17 | chr2:17213619-17214570 FORWARD no original descriptio	
RC_JCVI_25003	1.563	no original description	1.707
EE489964	1.563	no similarity	1.819
CD835540	1.562	weakly similar to (194)AT4G15240| Symbols: | fringe-related protein | chr4:8703518-8705378 REVERSE [13981]	
JCVI_18612	1.561	moderately similar to (336)AT5G59520| Symbols: ZIP2 | ZIP2 (ZINC TRANSPORTER 2 PRECURSOR); transferase, transferring glyc	1.713
JCVI_25257	1.561	no original description	
ES962397	1.560	no similarity	
EV105033	1.559	no similarity	
AM057728	1.557	no similarity	
EV140579	1.557	no similarity	3.953
JCVI_7222	1.557	weakly similar to (115)AT5G22650| Symbols: HDT02, HDT2, ATHD2B, HDA4, HD2, HD2B | HD2B (HISTONE DEACETYLASE 2	
EX117630	1.557	very weakly similar to (88.2)AT3G57520| Symbols: ATSIP2 | ATSIP2 (ARABIDOPSIS THALIANA SEED IMBIBITION 2); hydrolase	
EV206812	1.556	no similarity	
EV108197	1.556	no similarity	
EV104958	1.556	no similarity	
JCVI_3284	1.556	no original description	
JCVI_25402	1.556	moderately similar to (233)AT1G75510| Symbols: | transcription initiation factor IIF beta subunit (TFIIF-beta) family protein | chr1:283	
CD840477	1.556	weakly similar to (126)AT4G37610| Symbols: BT5 | BT5 (BTB and TAZ domain protein 5); protein binding / transcription regulator | c	
JCVI_21215	1.556	weakly similar to (127)AT3G21160| Symbols: | mannosyl-oligosaccharide 1,2-alpha-mannosidase, putative | chr3:7414135-7418334 R	
JCVI_4933	1.555	moderately similar to (407)AT5G55240| Symbols: | caleosin-related family protein / embryo-specific protein, putative | chr5:22423189	
JCVI_10739	1.555	moderately similar to (263)AT1G73500| Symbols: ATMKK9 | ATMKK9 (Arabidopsis thaliana MAP kinase kinase 9); kinase | chr1:276	
JCVI_24886	1.554	weakly similar to (169)AT1G24330| Symbols: | armadillo/beta-catenin repeat family protein / U-box domain-containing family protein	
JCVI_6423	1.554	moderately similar to (385)AT5G25060| Symbols: | RNA recognition motif (RRM)-containing protein | chr5:8634222-8639987 REVE	
ES952546	1.553	no similarity	
EE532855	1.553	no similarity	
ES967248	1.551	no similarity	
JCVI_18064	1.551	no original description	
JCVI_18420	1.551	moderately similar to (253)AT1G77840| Symbols: | eukaryotic translation initiation factor 5, putative / eIF-5, putative | chr1:29273981-	
EE559789	1.550	no similarity	4.052
JCVI_14836	1.550	no original description	2.083
JCVI_6820	1.548	no original description	
EE562330	1.547	no similarity	
EE438788	1.547	no similarity	
EE558591	1.547	no similarity	
RC_EV035662	1.546	no similarity	1.704
EX133841	1.546	no similarity	
JCVI_42244	1.544	no original description	

CD843660	1.544	weakly similar to (191)AT2G19070| Symbols: | transferase family protein | chr2:8267267-8269040 REVERSE [13982]	
EV000713	1.542	moderately similar to (223)AT5G46420| Symbols: | 16S rRNA processing protein RimM family | chr5:18847226-18850120 FORWAF	2.217
JCVI_27020	1.540	weakly similar to (134)AT5G62090| Symbols: | similar to SLK1 (SEUSS-LIKE 1), transcription regulator [Arabidopsis thaliana] (TAIF	
EV144618	1.540	no similarity	
EV142183	1.539	no similarity	
EV173421	1.539	no similarity	
EE565935	1.539	no similarity	
EV226774	1.537	no similarity	3.259
RC_EV141915	1.537	no similarity	
RC_EE564588	1.536	no similarity	
JCVI_169	1.535	moderately similar to (256)AT3G26070| Symbols: | plastid-lipid associated protein PAP / fibrillin family protein | chr3:9528141-95294	
JCVI_40949	1.534	no original description	
DW997333	1.533	no similarity	
RC_EE568828	1.533	no similarity	
EV078734	1.533	no similarity	3.329
JCVI_41494	1.533	no original description	
EE406554	1.532	no similarity	
EV064568	1.531	no similarity	2.238
EX055179	1.531	weakly similar to (134)AT2G32360| Symbols: | ubiquitin family protein | chr2:13744096-13744623 REVERSE [21812]	
JCVI_38972	1.530	no original description	
JCVI_42289	1.529	weakly similar to (137)AT5G12380| Symbols: | annexin, putative | chr5:4009224-4010688 FORWARDvery weakly similar to (94.0)AI	
EV040325	1.528	no similarity	
RC_EV128855	1.527	no similarity	
ES216611	1.526	no similarity	
JCVI_24995	1.526	no original description	
ES979003	1.526	no similarity	
JCVI_40220	1.526	no original description	
JCVI_21007	1.525	no original description	
RC_EE557313	1.524	no similarity	
CD835852	1.523	no similarity	
JCVI_24854	1.522	moderately similar to (273)AT2G40940| Symbols: ERS, ERS1 | ERS1 (ETHYLENE RESPONSE SENSOR 1); receptor | chr2:170917	
ES907952	1.522	weakly similar to (142)AT5G41761| Symbols: | similar to unknown protein [Arabidopsis thaliana] (TAIR:AT3G55570.1); similar to un	2.742
JCVI_35060	1.521	moderately similar to (410)AT5G55050| Symbols: | GDSL-motif lipase/hydrolase family protein | chr5:22354971-22356967 FORWAI	
EV192239	1.520	moderately similar to (298)AT3G10540| Symbols: | 3-phosphoinositide-dependent protein kinase, putative | chr3:3289921-3292434 FC	
JCVI_12379	1.520	no original description	
EV110091	1.520	no similarity	
EV221831	1.520	moderately similar to (328)AT3G61240| Symbols: | DEAD/DEAH box helicase, putative (RH12) | chr3:22677565-22680129 FORWA	3.094
EV013227	1.519	no similarity	
EV185534	1.518	no similarity	3.141
EX038903	1.517	moderately similar to (363)AT1G06250| Symbols: | lipase class 3 family protein | chr1:1913338-1914849 REVERSE [21811]	
RC_EH425397	1.517	no similarity	
RC_CX188526	1.517	no similarity	
JCVI_36167	1.516	weakly similar to (127)AT5G14090| Symbols: | similar to hypothetical protein [Vitis vinifera] (GB:CAN72196.1) | chr5:4547232-4549	
EE457779	1.514	moderately similar to (270)AT3G04480| Symbols: | endoribonuclease | chr3:1193994-1197326 REVERSE [20179]	
JCVI_13327	1.514	moderately similar to (266)AT1G73180| Symbols: | eukaryotic translation initiation factor-related | chr1:27521996-27524675 FORWA	
JCVI_32326	1.513	weakly similar to (152)AT5G24400| Symbols: EMB2024 | EMB2024 (EMBRYO DEFECTIVE 2024); catalytic | chr5:8330535-83317	
H07192	1.512	no similarity	
JCVI_14371	1.511	moderately similar to (244)AT4G34490| Symbols: CAP 1, CAP1, ATCAP1 | ATCAP1 (CYCLASE ASSOCIATED PROTEIN 1) | chr4	2.881
RC_EX125674	1.511	no similarity	
ES906075	1.510	moderately similar to (390)AT3G14260| Symbols: | similar to unknown protein [Arabidopsis thaliana] (TAIR:AT1G53870.1); similar t	
EE415842	1.509	no similarity	
EV107180	1.507	no similarity	
EE484017	1.506	weakly similar to (119)AT5G39220| Symbols: | hydrolase, alpha/beta fold family protein | chr5:15723141-15725267 FORWARD [201	
EV103018	1.504	no similarity	
EV129642	1.503	no similarity	
EV178946	1.503	moderately similar to (390)AT3G59770| Symbols: SAC9 | SAC9 (suppressor of actin 9) | chr3:22090258-22095762 REVERSE [21487	
RC_ES965522	1.503	no similarity	
JCVI_40706	1.502	no original description	
EE433092	1.502	moderately similar to (225)AT1G21340| Symbols: | Dof-type zinc finger domain-containing protein | chr1:7476075-7476857 FORWA	
EV177360	1.502	moderately similar to (204)AT2G21390| Symbols: | coatomer protein complex, subunit alpha, putative | chr2:9159508-9163657 FORW	
EX118448	1.502	moderately similar to (252)AT1G03220| Symbols: | extracellular dermal glycoprotein, putative / EDGP, putative | chr1:787143-788444	
EV107159	1.502	no similarity	
CV546376	1.501	no similarity	
DY017085	1.500	no similarity	
ES271455	1.500	weakly similar to (168)AT4G32630| Symbols: | ARF GTPase activator/ zinc ion binding | chr4:15738321-15741418 FORWARD [210	
EV000620	1.500	moderately similar to (353)AT3G14630| Symbols: CYP72A9 | CYP72A9 (cytochrome P450, family 72, subfamily A, polypeptide 9); c	
ES958185	1.499	no similarity	
AT000633	1.499	no similarity	3.835
JCVI_30925	1.499	moderately similar to (323)AT2G41340| Symbols: | eukaryotic rpb5 RNA polymerase subunit family protein | chr2:17236104-1723752	
JCVI_13859	1.498	moderately similar to (210)AT4G08670| Symbols: | protease inhibitor/seed storage/lipid transfer protein (LTP) family protein | chr4:553	
JCVI_26057	1.497	moderately similar to (303)AT5G60390| Symbols: | elongation factor 1-alpha / EF-1-alpha | chr5:24306452-24307901 FORWARDmo	
AT000459	1.496	no similarity	2.297
ES918306	1.496	weakly similar to (156)AT2G02170| Symbols: | remorin family protein | chr2:556594-558609 REVERSE [15718]	
JCVI_5783	1.495	moderately similar to (342)AT1G04640| Symbols: LIP2 | LIP2 (LIPOYLTRANSFERASE 2) | chr1:1292540-1293247 FORWARD no	
JCVI_31055	1.494	highly similar to (631)AT5G08280| Symbols: HEMC | HEMC (HYDROXYMETHYLBILANE SYNTHASE); hydroxymethylbilane sy	
JCVI_36426	1.493	moderately similar to (209)AT2G23200| Symbols: | protein kinase family protein | chr2:9886431-9888935 FORWARD no original des	
EX136226	1.493	weakly similar to (108)AT3G13720| Symbols: | prenylated rab acceptor (PRA1) family protein | chr3:4495209-4495775 REVERSE [2	
CX190389	1.491	weakly similar to (194)AT5G58400| Symbols: | peroxidase, putative | chr5:23622583-23624193 REVERSEweakly similar to (159)PE	
EV090258	1.490	very weakly similar to (87.4)AT3G17120| Symbols: | similar to unknown protein [Arabidopsis thaliana] (TAIR:AT4G01960.1); similar	-1.130
JCVI_29503	1.490	very weakly similar to (86.3)AT1G16850| Symbols: | unknown protein | chr1:5765055-5765611 REVERSE no original description	-1.896
JCVI_32755	1.489	moderately similar to (259)AT4G01360| Symbols: | similar to BPS1 (BYPASS 1) [Arabidopsis thaliana] (TAIR:AT1G01550.2); simila	1.520
EV111104	1.488	no similarity	
JCVI_11120	1.488	very weakly similar to (92.4)AT5G44510| Symbols: | disease resistance protein (TIR-NBS-LRR class), putative | chr5:17946900-17951	

JCVI_36219	1.488	no original description	
RC_EE556547	1.487	no similarity	1.849
EE509045	1.487	no similarity	
CX189964	1.487	weakly similar to (126)AT1G30820| Symbols: | CTP synthase, putative / UTP--ammonia ligase, putative | chr1:10945232-10948806 R	
EE556555	1.486	no similarity	
CD833602	1.486	no similarity	
JCVI_10814	1.485	moderately similar to (387)AT5G36260| Symbols: | aspartyl protease family protein | chr5:14302298-14305409 REVERSE no original	
JCVI_39869	1.485	no original description	
ES909140	1.484	moderately similar to (329)AT2G41700| Symbols: | similar to ATATH11 (ABC2 homolog 11), ATPase, coupled to transmembrane mo	
JCVI_8380	1.483	no original description	
ES967290	1.483	no similarity	
EV110348	1.483	no similarity	
EX070150	1.482	no similarity	
RC_JCVI_42143	1.482	no original description	
JCVI_28003	1.481	moderately similar to (377)AT5G13680| Symbols: ELO2, ABO1 | ABO1/ELO2 (ABA-OVERLY SENSITIVE 1); transcription elongat	1.504
EV130288	1.479	weakly similar to (138)AT3G58780| Symbols: AGL1, SHP1 | SHP1 (SHATTERPROOF 1) | chr3:21750127-21752743 FORWARDve	
EV084867	1.478	moderately similar to (237)AT1G03080| Symbols: | kinase interacting family protein | chr1:731794-737332 REVERSE [21444]	
AM060558	1.477	no similarity	
JCVI_11707	1.477	moderately similar to (317)AT1G10870| Symbols: AGD4 | AGD4 (ARF-GAP DOMAIN 4); ARF GTPase activator/ protein binding / z	
EV103854	1.477	no similarity	
JCVI_22538	1.476	moderately similar to (258)AT5G26830| Symbols: | threonyl-tRNA synthetase / threonine--tRNA ligase (THRRS) | chr5:9437354-9441	
JCVI_20186	1.475	no original description	
EV130305	1.475	no similarity	
EV111694	1.475	no similarity	
JCVI_32927	1.475	moderately similar to (390)AT3G14040| Symbols: | exopolygalacturonase / galacturan 1,4-alpha-galacturonidase / pectinase | chr3:4648	3.555
EE467127	1.474	moderately similar to (261)AT1G51640| Symbols: ATEXO70G2 | ATEXO70G2 (exocyst subunit EXO70 family protein G2); protein b	
EL590556	1.474	weakly similar to (134)AT2G18193| Symbols: | AAA-type ATPase family protein | chr2:7924703-7926266 REVERSE [20863]	
EV051986	1.473	weakly similar to (103)AT2G46240| Symbols: BAG6, ATBAG6 | BAG6 (ARABIDOPSIS THALIANA BCL-2-ASSOCIATED ATHA	
JCVI_23286	1.469	very weakly similar to (81.6)AT1G03850| Symbols: | glutaredoxin family protein | chr1:977232-977684 REVERSE no original descrip	
EE557335	1.469	no similarity	
JCVI_34163	1.468	weakly similar to (132)AT3G56910| Symbols: PSRP5 | PSRP5 (PLASTID-SPECIFIC 50S RIBOSOMAL PROTEIN 5) | chr3:2108053	
EV221306	1.467	moderately similar to (290)AT5G53320| Symbols: | leucine-rich repeat transmembrane protein kinase, putative | chr5:21653679-21655	
ES978384	1.467	no similarity	
CA992531	1.466	no similarity	
JCVI_40057	1.465	no original description	
EV197949	1.465	no similarity	
JCVI_13973	1.463	no original description	
CD842998	1.463	no similarity	
RC_EH429864	1.463	no similarity	
EE566128	1.462	no similarity	
JCVI_36756	1.461	highly similar to (561)AT2G34357| Symbols: | binding | chr2:14506315-14512634 FORWARD no original description	
ES915996	1.461	weakly similar to (169)AT1G04990| Symbols: | zinc finger (CCCH-type) family protein | chr1:1419367-1421453 REVERSEvery weak	
EH429137	1.461	no similarity	-2.615
EE567756	1.461	no similarity	
JCVI_33816	1.460	no original description	
JCVI_4603	1.458	no original description	
AT000953	1.457	no similarity	
EH430386	1.457	weakly similar to (107)AT1G29120| Symbols: | similar to unknown protein [Arabidopsis thaliana] (TAIR:AT4G25770.1); similar to un	
ES956344	1.457	no similarity	4.109
JCVI_26037	1.456	moderately similar to (475)AT1G06250| Symbols: | lipase class 3 family protein | chr1:1913338-1914849 REVERSE no original descr	
EV099378	1.455	no similarity	
CD822982	1.455	no similarity	
JCVI_8059	1.455	very weakly similar to (91.3)AT4G28990| Symbols: | RNA-binding protein-related | chr4:14291211-14293024 FORWARD no original	
EX055974	1.455	no similarity	
JCVI_26814	1.454	moderately similar to (333)AT5G55090| Symbols: MAPKKK15 | MAPKKK15 (Mitogen-activated protein kinase kinase kinase 15); ki	
JCVI_23536	1.453	weakly similar to (193)AT3G17350| Symbols: | similar to unknown protein [Arabidopsis thaliana] (TAIR:AT5G50290.1); similar to un	
EE559322	1.453	no similarity	
EV028305	1.452	moderately similar to (279)AT5G12350| Symbols: | Ran GTPase binding / chromatin binding / zinc ion binding | chr5:3995790-400054	
RC_EV151856	1.452	no similarity	
EV098567	1.451	moderately similar to (256)AT4G17530| Symbols: ATRAB1C, ATRABD2C, RAB1C | RAB1C; GTP binding | chr4:9773734-9775437	
JCVI_20203	1.451	moderately similar to (245)AT4G37670| Symbols: | GCN5-related N-acetyltransferase (GNAT) family protein / amino acid kinase fam	-1.794
EE567709	1.449	moderately similar to (318)AT4G15040| Symbols: | subtilase family protein | chr4:8581368-8584117 REVERSE [20153] 16 639 639	
CD827731	1.448	no similarity	
EV145901	1.448	no similarity	
JCVI_35650	1.448	no original description	
EX125576	1.448	moderately similar to (332)AT4G35860| Symbols: ATRABB1B, ATGB2, ATRAB2C | ATGB2/ATRAB2C/ATRABB1B (GTP-BINDI	
EV186410	1.448	weakly similar to (115)AT1G08200| Symbols: AXS2 | AXS2 (UDP-D-APIOSE/UDP-D-XYLOSE SYNTHASE 2) | chr1:2574256-257	
JCVI_36160	1.448	weakly similar to (188)AT5G67620| Symbols: | similar to unknown protein [Arabidopsis thaliana] (TAIR:AT5G62900.1); similar to un	
JCVI_32997	1.448	no original description	
JCVI_26862	1.447	weakly similar to (163)AT5G14370| Symbols: | similar to CIL [Arabidopsis thaliana] (TAIR:AT4G25990.1); similar to unnamed prote	
JCVI_27777	1.446	moderately similar to (213)AT2G40030| Symbols: DRD3, ATNRPD1B, NRPD1b | NRPD1b (nuclear RNA polymerase D 1b); DNA b	
JCVI_4641	1.445	very weakly similar to (99.0)AT1G12970| Symbols: | leucine-rich repeat family protein | chr1:4423725-4425630 FORWARD no origin	
H07209	1.445	no similarity	
EE407583	1.444	no similarity	
RC_EV069141	1.444	no similarity	
JCVI_26895	1.443	no original description	
EX135527	1.441	no similarity	
JCVI_1129	1.440	moderately similar to (278)AT2G39960| Symbols: | microsomal signal peptidase 25 kDa subunit, putative (SPC25) | chr2:16688744-16	
AM060232	1.438	no similarity	
AT000698	1.438	no similarity	
JCVI_9908	1.436	no original description	
EV147742	1.436	no similarity	
JCVI_40930	1.434	no original description	

EV105948	1.430	no similarity	
JCVI_39709	1.429	no original description	
EV134099	1.429	no similarity	
EE439107	1.429	no similarity	
JCVI_5282	1.429	moderately similar to (270)AT3G62300| Symbols: | agenet domain-containing protein | chr3:23065222-23068056 FORWARD no orig	
EV140700	1.427	no similarity	
JCVI_10013	1.427	moderately similar to (292)AT1G73260| Symbols: | trypsin and protease inhibitor family protein / Kunitz family protein | chr1:275510	
JCVI_41334	1.427	weakly similar to (195)AT3G53750| Symbols: ACT3 | ACT3 (ACTIN 3); structural constituent of cytoskeleton | chr3:19926902-199283	
EV024953	1.426	weakly similar to (192)AT1G63730| Symbols: | disease resistance protein (TIR-NBS-LRR class), putative | chr1:23645433-23648795 I	2.567
EV092812	1.425	weakly similar to (121)AT5G67420| Symbols: LBD37 | LBD37 (LOB DOMAIN-CONTAINING PROTEIN 37) | chr5:26921802-2692	
ES900535	1.424	moderately similar to (349)AT4G32650| Symbols: KAT3, ATKC1 | ATKC1 (ARABIDOPSIS THALIANA K+ RECTIFYING CHANN	
ES945936	1.424	no similarity	
H07240	1.424	no similarity	
EV109794	1.424	weakly similar to (116)AT4G29480| Symbols: | mitochondrial ATP synthase g subunit family protein | chr4:14486271-14487263 REV	
EL590531	1.424	weakly similar to (196)AT3G11060| Symbols: | similar to unknown protein [Arabidopsis thaliana] (TAIR:AT3G06545.1) | chr3:34663	
JCVI_22813	1.423	no original description	
JCVI_30785	1.421	no original description	
CD822362	1.421	no similarity	
JCVI_2457	1.421	weakly similar to (185)AT5G54170| Symbols: | similar to CP5 [Arabidopsis thaliana] (TAIR:AT1G64720.1); similar to putative nodule	
EV015734	1.421	no similarity	
EE561281	1.420	no similarity	
EV136405	1.420	weakly similar to (118)AT3G13400| Symbols: SKS13 | SKS13 (SKU5 Similar 13); copper ion binding / oxidoreductase | chr3:4355264	
EE450741	1.419	weakly similar to (105)AT3G07350| Symbols: | similar to unknown protein [Arabidopsis thaliana] (TAIR:AT3G25240.1); similar to un	
JCVI_31903	1.419	weakly similar to (148)AT2G45460| Symbols: | forkhead-associated domain-containing protein / FHA domain-containing protein | chr2	
JCVI_20194	1.418	moderately similar to (452)AT3G55250| Symbols: | similar to unnamed protein product [Vitis vinifera] (GB:CAO14780.1) | chr3:2049	
JCVI_38968	1.417	weakly similar to (150)AT5G59950| Symbols: | RNA and export factor-binding protein, putative | chr5:24157461-24158636 FORWAI	
JCVI_33747	1.416	moderately similar to (208)AT3G28790| Symbols: | similar to unknown protein [Arabidopsis thaliana] (TAIR:AT3G28830.1); contains	
EL590055	1.415	moderately similar to (335)AT1G09580| Symbols: | emp24/gp25L/p24 family protein | chr1:3104659-3106094 FORWARD [20863]	
ES969282	1.415	no similarity	
JCVI_11321	1.414	no original description	
JCVI_32388	1.414	moderately similar to (330)AT5G01890| Symbols: | leucine-rich repeat transmembrane protein kinase, putative | chr5:341658-344647 F	
JCVI_31189	1.414	weakly similar to (104)AT1G43722| Symbols: | similar to unknown protein [Arabidopsis thaliana] (TAIR:AT5G28730.1); similar to hy	2.036
EV093388	1.414	no similarity	
JCVI_27345	1.414	moderately similar to (211)AT5G04420| Symbols: | kelch repeat-containing protein | chr5:1246868-1249456 REVERSE no original de	
CX187929	1.414	no similarity	
ES966917	1.411	no similarity	
RC_EX070870	1.411	no similarity	
EV089036	1.410	no similarity	
ES921286	1.410	no similarity	
JCVI_38310	1.409	no original description	
EX015585	1.408	no similarity	
RC_ES979021	1.408	no similarity	2.761
EV118247	1.408	no similarity	
CB617669	1.407	no similarity	
DT469162	1.407	very weakly similar to (81.6)AT1G76580| Symbols: | transcription factor | chr1:28740222-28743345 FORWARD [17811]	
AM394346	1.407	moderately similar to (306)AT3G28340| Symbols: GATL10 | GATL10 (Galacturonosyltransferase-like 10); polygalacturonate 4-alpha-g	1.019
EV122886	1.406	no similarity	
EV220108	1.406	no similarity	
JCVI_7932	1.406	very weakly similar to (87.0)AT5G64310| Symbols: ATAGP1, AGP1 | AGP1 (ARABINOGALACTAN-PROTEIN 1) | chr5:25739244-	
EV150383	1.406	very weakly similar to (97.8)AT5G15640| Symbols: | mitochondrial substrate carrier family protein | chr5:5087593-5089680 FORWAR	
EV170073	1.406	moderately similar to (352)AT4G21990| Symbols: PRH-26, PRH26, ATAPR3, APR3 | APR3 (APS REDUCTASE 3) | chr4:11657296-	1.672
EV116362	1.405	no similarity	
JCVI_37124	1.405	weakly similar to (107)AT3G31430| Symbols: | similar to unknown protein [Arabidopsis thaliana] (TAIR:AT5G18636.1); similar to 80	2.602
JCVI_25347	1.405	no original description	
EV098586	1.405	weakly similar to (112)AT2G35860| Symbols: FLA16 | FLA16 (FASCICLIN-LIKE ARABINOGALACTAN PROTEIN 16 PRECURS	
RC_ES968445	1.404	no similarity	
EX055334	1.404	no similarity	
JCVI_19533	1.404	no original description	
EV186109	1.402	very weakly similar to (98.2)AT2G15620| Symbols: NIR, ATHNIR, NIR1 | NIR1 (NITRITE REDUCTASE); ferredoxin-nitrate reductas	
EV105960	1.402	no similarity	3.413
JCVI_40367	1.401	no original description	
RC_ES966556	1.401	no similarity	
ES900108	1.400	weakly similar to (157)AT2G45430| Symbols: | DNA-binding protein-related | chr2:18734922-18735875 FORWARD [21428] 1 678 7	
RC_EE565489	1.399	no similarity	
ES937199	1.399	weakly similar to (124)AT3G09360| Symbols: | RNA polymerase II transcription factor | chr3:2873802-2878438 FORWARD [16815]	
EE559472	1.398	no similarity	
CV433373	1.398	very weakly similar to (85.9)AT3G21510| Symbols: AHP1 | AHP1 (HISTIDINE-CONTAINING PHOSPHOTRANSMITTER 3); histid	
EV177441	1.398	moderately similar to (283)AT1G06890| Symbols: | transporter-related | chr1:2111727-2114037 REVERSE [21487]	
CX194834	1.397	no similarity	
DY019070	1.397	no similarity	
JCVI_7693	1.396	moderately similar to (225)AT5G54490| Symbols: PBP1 | PBP1 (PINOID-BINDING PROTEIN 1); calcium ion binding | chr5:221386	
EX093528	1.396	weakly similar to (130)AT5G13340| Symbols: | similar to F-box family protein [Arabidopsis thaliana] (TAIR:AT1G10890.1); similar t	
JCVI_30328	1.395	moderately similar to (270)AT1G44750| Symbols: ATPUP11 | ATPUP11 (Arabidopsis thaliana purine permease 11) | chr1:16896697-1	1.423
EX110845	1.394	no similarity	
EV086171	1.393	no similarity	
EV098661	1.393	very weakly similar to (85.5)TRAC9_MAIZE [21477]	
ES898836	1.392	no similarity	
EV131945	1.392	no similarity	
EE566632	1.392	no similarity	
EE567536	1.392	no similarity	
JCVI_26497	1.391	no original description	
JCVI_40060	1.391	no original description	2.532
EE513336	1.390	no similarity	

EV223121	1.390	no similarity	
EE562204	1.389	no similarity	
EE423276	1.389	no similarity	
ES995596	1.389	no similarity	
EV220623	1.388	no similarity	
EV174416	1.388	weakly similar to (121)AT3G15450| Symbols: | similar to unknown protein [Arabidopsis thaliana] (TAIR:AT4G27450.1); similar to un	
EV091063	1.388	no similarity	
ETG10_236652	1.387	no similarity	
ES966626	1.387	no similarity	
CD843597	1.387	no similarity	
JCVI_622	1.386	moderately similar to (464)AT2G34770| Symbols: FAH1 | FAH1 (FATTY ACID HYDROXYLASE 1); catalytic | chr2:14673855-1467	
EV067830	1.385	no similarity	
JCVI_38585	1.384	no original description	
JCVI_31383	1.384	weakly similar to (199)AT2G40600| Symbols: | appr-1-p processing enzyme family protein | chr2:16954090-16955615 REVERSE no	1.693
ES910429	1.384	weakly similar to (174)AT1G34010| Symbols: | similar to unknown protein [Arabidopsis thaliana] (TAIR:AT1G22790.2); similar to un	
JCVI_29049	1.384	weakly similar to (130)AT1G58360| Symbols: NAT2, AAP1 | AAP1 (AMINO ACID PERMEASE 1); amino acid transmembrane trans	
JCVI_2595	1.384	highly similar to (646)AT1G71770| Symbols: PAB5 | PAB5 (POLY(A)-BINDING PROTEIN); RNA binding | chr1:26994439-2699710	
EE501845	1.383	no similarity	
DY011593	1.383	very weakly similar to (86.3)AT4G30240| Symbols: | protein binding | chr4:14808365-14809751 REVERSE [18980]	
EV146475	1.382	weakly similar to (147)AT4G21120| Symbols: CAT1 | AAT1 (CATIONIC AMINO ACID TRANSPORTER 1); cationic amino acid tra	
ES960234	1.382	no similarity	
JCVI_35527	1.382	moderately similar to (261)AT5G23190| Symbols: CYP86B1 | CYP86B1 (cytochrome P450, family 86, subfamily B, polypeptide 1); o	
EV096246	1.382	no similarity	
JCVI_33758	1.380	moderately similar to (354)AT1G69940| Symbols: ATPPME1 | ATPPME1; pectinesterase | chr1:26347212-26348634 REVERSEvery	
EX103391	1.380	weakly similar to (155)AT3G05920| Symbols: | heavy-metal-associated domain-containing protein | chr3:1768997-1769528 REVERSI	
RC_CV973879	1.380	no similarity	
EV183877	1.379	weakly similar to (130)AT1G11880| Symbols: | similar to unnamed protein product [Vitis vinifera] (GB:CAO22037.1); contains InterP	
AM395320	1.379	no similarity	
RC_EV167123	1.379	no similarity	
JCVI_6453	1.378	no original description	
BQ791333	1.377	no similarity	1.770
JCVI_4244	1.377	moderately similar to (261)AT3G20740| Symbols: FIS3, FIE1, FIE | FIE (FERTILIZATION-INDEPENDENT ENDOSPERM 1); nucle	
CX266638	1.377	moderately similar to (230)AT1G55550| Symbols: | microtubule motor | chr1:20752581-20756528 FORWARD [16816] 26 522 522	
CO750216	1.377	no similarity	1.715
JCVI_3837	1.374	moderately similar to (326)AT2G23640| Symbols: | reticulon family protein (RTNLB13) | chr2:10064634-10066122 FORWARD no o	
JCVI_181	1.371	highly similar to (524)AT5G67400| Symbols: | peroxidase 73 (PER73) (P73) (PRXR11) | chr5:26912122-26913526 FORWARDmode	1.278
EE558079	1.371	no similarity	
DN962793	1.370	weakly similar to (128)AT2G40000| Symbols: HSPRO2, ATHSPRO2 | similar to unknown protein [Arabidopsis thaliana] (TAIR:AT3C	
EV165539	1.370	moderately similar to (228)AT2G46140| Symbols: | late embryogenesis abundant protein, putative / LEA protein, putative | chr2:18966	
EE531349	1.369	no similarity	
JCVI_25169	1.369	weakly similar to (199)AT2G43900| Symbols: | endonuclease/exonuclease/phosphatase family protein | chr2:18185910-18190899 REV	
EE547386	1.369	no similarity	
JCVI_6384	1.367	no original description	
ES265492	1.367	weakly similar to (108)AT4G16745| Symbols: | exostosin family protein | chr4:9412207-9414075 FORWARD [21031]	
JCVI_28178	1.366	very weakly similar to (99.0)AT5G26760| Symbols: | similar to hypothetical protein OsI_017683 [Oryza sativa (indica cultivar-group)]	
JCVI_4494	1.364	no original description	
EV145751	1.363	no similarity	
DY009187	1.363	no similarity	
EV049289	1.361	no similarity	
RC_EV216502	1.361	no similarity	2.065
EE552225	1.361	no similarity	
RC_EV017894	1.361	no similarity	
EE471402	1.361	moderately similar to (252)AT4G24790| Symbols: | ATP binding / DNA binding / DNA-directed DNA polymerase | chr4:12778232-12	
EX125037	1.359	weakly similar to (126)AT4G17615| Symbols: ATCBL1, SCABP5 | CBL1 (CALCINEURIN B-LIKE PROTEIN 1); calcium ion bindir	
EV177542	1.359	no similarity	
JCVI_11488	1.358	moderately similar to (364)AT4G29240| Symbols: | leucine-rich repeat family protein / extensin family protein | chr4:14418832-14420	
EV135306	1.357	no similarity	
JCVI_33127	1.356	moderately similar to (334)AT1G80410| Symbols: EMB2753 | EMB2753 (EMBRYO DEFECTIVE 2753); binding | chr1:30232855-3	
AM389713	1.356	no similarity	
JCVI_7609	1.354	weakly similar to (137)AT1G12390| Symbols: | cornichon family protein | chr1:4220345-4221479 FORWARD no original description	
JCVI_33753	1.354	no original description	
EV065424	1.353	no similarity	
EE521237	1.352	no similarity	
JCVI_47	1.352	moderately similar to (246)AT1G77640| Symbols: | AP2 domain-containing transcription factor, putative | chr1:29183690-29184424 F	
RC_JCVI_29494	1.351	no original description	2.068
JCVI_40366	1.350	moderately similar to (435)AT4G03070| Symbols: AOP, AOP1.1, AOP1 | AOP1 (2-oxoglutarate?dependent dioxygenase 1.1); oxidore	
ES948565	1.350	no similarity	
RC_EE565901	1.349	no similarity	
JCVI_25786	1.348	no original description	
JCVI_39967	1.348	moderately similar to (397)AT2G18480| Symbols: | mannitol transporter, putative | chr2:8016664-8018325 REVERSEweakly similar t	
JCVI_22350	1.347	moderately similar to (327)AT5G12900| Symbols: | similar to unknown protein [Arabidopsis thaliana] (TAIR:AT1G12330.1); similar t	
EV090195	1.346	weakly similar to (176)AT3G52340| Symbols: SPP2 | SPP2; sucrose-phosphatase | chr3:19418390-19420160 FORWARD [21444]	
DY028090	1.346	no similarity	1.769
ES903612	1.344	no similarity	
JCVI_24754	1.344	very weakly similar to (89.0)AT1G02720| Symbols: GATL5 | GATL5; transferase, transferring glycosyl groups / transferase, transferring	
JCVI_32832	1.343	weakly similar to (145)AT1G14510| Symbols: | PHD finger family protein | chr1:4962166-4964149 REVERSE no original description	
EE565995	1.343	no similarity	-1.715
EE501795	1.343	weakly similar to (120)AT1G70260| Symbols: | nodulin MtN21 family protein | chr1:26460730-26463001 REVERSE [20133]	
JCVI_32977	1.343	weakly similar to (101)AT2G28390| Symbols: | SAND family protein | chr2:12146913-12150452 REVERSE no original description	
CD825490	1.341	no similarity	
JCVI_25567	1.341	moderately similar to (295)AT5G44560| Symbols: VPS2.2 | VPS2.2 | chr5:17963743-17965449 FORWARD no original description	3.892
ES960175	1.341	no similarity	
JCVI_30718	1.340	moderately similar to (293)AT5G59340| Symbols: WOX2 | WOX2 (WUSCHEL-related homeobox 2); transcription factor | chr5:2395	

EE550965	1.340	no similarity	
JCVI_41811	1.340	weakly similar to (140)AT4G27470| Symbols: | zinc finger (C3HC4-type RING finger) family protein | chr4:13735582-13736313 FOR	
JCVI_1372	1.340	moderately similar to (322)AT1G47600| Symbols: | glycosyl hydrolase family 1 protein | chr1:17494211-17497029 FORWARDweakly	
JCVI_18975	1.338	highly similar to (511)AT1G31440| Symbols: | SH3 domain-containing protein 1 (SH3P1) | chr1:11256131-11258460 REVERSE no o	
RC_EB041735	1.336	no similarity	
EV110999	1.336	no similarity	
JCVI_8848	1.336	no original description	1.622
EE560278	1.334	no similarity	
EV109636	1.334	no similarity	1.781
EX047794	1.332	no similarity	
EV106052	1.332	no similarity	
JCVI_12010	1.332	weakly similar to (197)AT3G48800| Symbols: | sterile alpha motif (SAM) domain-containing protein | chr3:18106810-18107646 REV	1.251
RC_AM394852	1.332	no similarity	
ES963527	1.331	no similarity	
EV018052	1.330	no similarity	
JCVI_38459	1.330	moderately similar to (374)AT3G60320| Symbols: | DNA binding | chr3:22303048-22306203 REVERSE no original description	2.114
EE549718	1.330	no similarity	
EH424674	1.329	no similarity	
RC_JCVI_40422	1.329	no original description	
EE526882	1.329	no similarity	
JCVI_28456	1.329	no original description	
CV973884	1.328	no similarity	
JCVI_34878	1.328	weakly similar to (175)AT5G59670| Symbols: | leucine-rich repeat protein kinase, putative | chr5:24058764-24062704 FORWARD no	3.178
DN965455	1.327	no similarity	
JCVI_27380	1.327	moderately similar to (232)AT3G53020| Symbols: RPL24B, RPL24, STV1 | STV1 (SHORT VALVE1); structural constituent of ribosc	1.500
JCVI_6966	1.326	moderately similar to (343)AT1G01360| Symbols: | similar to unknown protein [Arabidopsis thaliana] (TAIR:AT4G01026.1); similar t	
JCVI_7640	1.325	moderately similar to (384)AT3G47500| Symbols: CDF3 | CDF3 (CYCLING DOF FACTOR 3); DNA binding / protein binding / trans	2.716
EV147794	1.324	no similarity	
EX133335	1.324	weakly similar to (127)AT4G23460| Symbols: | beta-adaptin, putative | chr4:12243909-12248908 REVERSE [21833] 20 585 585	
JCVI_41079	1.323	highly similar to (893)AT5G39590| Symbols: | similar to unnamed protein product [Vitis vinifera] (GB:CAO61019.1); contains InterPr	
ES967608	1.322	no similarity	
JCVI_9461	1.321	highly similar to (503)AT3G50100| Symbols: | exonuclease | chr3:18589394-18591577 FORWARD no original description	
RC_ES960829	1.321	no similarity	
EV173566	1.320	moderately similar to (439)AT1G66830| Symbols: | leucine-rich repeat transmembrane protein kinase, putative | chr1:24934363-24936	
EX092831	1.320	moderately similar to (297)AT5G56370| Symbols: | F-box family protein | chr5:22853426-22854845 REVERSE [21823] 18 852 852	
EE558537	1.320	no similarity	
EE565726	1.319	no similarity	1.130
JCVI_27002	1.318	no original description	
DY003536	1.317	very weakly similar to (99.8)AT5G25540| Symbols: CID6 | CID6 (CTC-Interacting Domain 6); protein binding | chr5:8891773-8892365	
EE567175	1.316	no similarity	
JCVI_23541	1.316	very weakly similar to (99.0)AT4G34410| Symbols: | AP2 domain-containing transcription factor, putative | chr4:16451996-16452802 I	
JCVI_5591	1.314	moderately similar to (334)AT1G74820| Symbols: | cupin family protein | chr1:28115543-28116226 REVERSEweakly similar to (157	
JCVI_38439	1.314	no original description	
EL591783	1.313	moderately similar to (353)AT1G08340| Symbols: | rac GTPase activating protein, putative | chr1:2631305-2632666 FORWARD [208	
RC_EE563974	1.313	no similarity	2.230
EE505235	1.313	no similarity	1.228
JCVI_35716	1.313	moderately similar to (408)AT4G36030| Symbols: | armadillo/beta-catenin repeat family protein | chr4:17045087-17047099 REVERSI	
EE543610	1.312	no similarity	
EV171540	1.312	no similarity	
JCVI_12486	1.311	moderately similar to (263)AT3G48080| Symbols: | lipase class 3 family protein / disease resistance protein-related | chr3:17764089-17	
JCVI_35549	1.311	no original description	
JCVI_34556	1.310	moderately similar to (316)AT5G02370| Symbols: | kinesin motor protein-related | chr5:503442-506386 FORWARD no original descri	
EV163857	1.310	weakly similar to (109)AT2G33810| Symbols: SPL3 | SPL3 (SQUAMOSA PROMOTER BINDING PROTEIN-LIKE 3); transcription	
JCVI_28643	1.309	weakly similar to (105)AT5G56460| Symbols: | protein kinase, putative | chr5:22882735-22885092 FORWARD no original descriptio	
ES953772	1.309	no similarity	
EE569283	1.309	no similarity	
EV221837	1.308	no similarity	
JCVI_15874	1.308	no original description	
JCVI_34123	1.306	moderately similar to (234)AT5G04010| Symbols: | similar to unknown protein [Arabidopsis thaliana] (TAIR:AT5G03920.1); similar t	
JCVI_27017	1.306	weakly similar to (180)AT1G17530| Symbols: ATTIM23-1 | ATTIM23-1 (TRANSLOCASE OF INNER MITOCHONDRIAL MEMBI	
EX093418	1.306	weakly similar to (183)AT1G65160| Symbols: | ubiquitin carboxyl-terminal hydrolase family protein | chr1:24209887-24211401 FORV	
JCVI_31159	1.305	highly similar to (580)AT2G46750| Symbols: | FAD-binding domain-containing protein | chr2:19215513-19217979 REVERSE no orig	
EE546195	1.305	no similarity	
JCVI_33419	1.305	very weakly similar to (95.9)AT5G11810| Symbols: | similar to Os02g0135600 [Oryza sativa (japonica cultivar-group)] (GB:NP_00104	
JCVI_11741	1.303	no original description	
JCVI_958	1.303	no original description	
JCVI_9018	1.302	weakly similar to (169)AT5G23860| Symbols: TUB8 | TUB8 (tubulin beta-8) | chr5:8042965-8044531 FORWARDweakly similar to (	
EE567384	1.302	no similarity	
JCVI_18326	1.300	moderately similar to (384)AT4G01360| Symbols: | similar to BPS1 (BYPASS 1) [Arabidopsis thaliana] (TAIR:AT1G01550.2); simila	
JCVI_195	1.300	no original description	
EV006157	1.300	weakly similar to (105)AT5G58640| Symbols: | selenoprotein-related | chr5:23715222-23716731 FORWARD [21427]	
JCVI_18920	1.300	no original description	
JCVI_32318	1.299	moderately similar to (206)AT3G18440| Symbols: ATALMT9 | ATALMT9 (ALUMINUM-ACTIVATED MALATE TRANSPORTER	
JCVI_39291	1.299	moderately similar to (226)AT5G16890| Symbols: | exostosin family protein | chr5:5551667-5554744 FORWARD no original descript	
EE559994	1.298	no similarity	
EX125056	1.297	weakly similar to (167)AT3G57010| Symbols: | strictosidine synthase family protein | chr3:21106833-21108295 REVERSE [21830]	
EV223015	1.297	no similarity	4.448
ES908593	1.296	weakly similar to (139)AT4G05150| Symbols: | octicosapeptide/Phox/Bem1p (PB1) domain-containing protein | chr4:2660337-266267	
RC_EE565714	1.295	no similarity	
EV217656	1.294	no similarity	
H07284	1.294	no similarity	
ES961512	1.294	no similarity	2.110
ES996702	1.293	no similarity	

RC_JCVI_42381	1.292	no original description	
EV190691	1.292	no similarity	
ES939524	1.292	no similarity	
EV150322	1.290	very weakly similar to (100)AT5G14995| Symbols: | Encodes a ECA1 gametogenesis related family protein | chr5:4853416-4853721 F	
JCVI_27796	1.289	weakly similar to (107)AT4G27657| Symbols: | similar to unknown protein [Arabidopsis thaliana] (TAIR:AT4G27652.1) | chr4:13813	
EE417919	1.289	no similarity	
EE558422	1.288	no similarity	
JCVI_18930	1.288	weakly similar to (170)AT3G16000| Symbols: MFP1 | MFP1 (MAR BINDING FILAMENT-LIKE PROTEIN 1) | chr3:5431047-5433	
EV099338	1.287	no similarity	2.066
JCVI_38236	1.287	weakly similar to (159)AT1G14860| Symbols: ATNUDT18 | ATNUDT18 (Arabidopsis thaliana Nudix hydrolase homolog 18); hydrola	
CX278446	1.287	moderately similar to (298)AT4G37050| Symbols: PLP4, PLA V | PLA V/PLP4 (Patatin-like protein 4); nutrient reservoir | chr4:17457	1.324
JCVI_13997	1.285	no original description	
JCVI_15772	1.285	weakly similar to (177)AT4G30180| Symbols: | transcription factor/ transcription regulator | chr4:14769034-14769510 FORWARD no	
EV128968	1.283	no similarity	
JCVI_35464	1.283	moderately similar to (334)AT4G33210| Symbols: | F-box family protein (FBL15) | chr4:16015974-16020700 REVERSE no original	
EE419268	1.283	no similarity	1.862
JCVI_26806	1.283	no original description	
EV148098	1.282	no similarity	
JCVI_7083	1.282	no original description	
JCVI_12330	1.282	weakly similar to (148)AT5G50915| Symbols: | basic helix-loop-helix (bHLH) family protein | chr5:20727990-20729478 REVERSE n	
JCVI_5729	1.281	no original description	
EV107221	1.280	no similarity	
EE569647	1.279	no similarity	
EV175792	1.279	weakly similar to (148)AT5G27150| Symbols: ATNHX, AT-NHX1, ATNHX1, NHX1 | NHX1 (NA+/H+ EXCHANGER); sodium ion	1.355
EE449364	1.277	weakly similar to (144)AT1G12540| Symbols: | basic helix-loop-helix (bHLH) family protein | chr1:4273135-4273978 FORWARD [2	
EE566939	1.275	no similarity	
EV178131	1.275	no similarity	
EE472687	1.274	no similarity	
RC_ES955688	1.273	no similarity	
RC_EE569610	1.273	no similarity	
EV064370	1.273	no similarity	
EV224957	1.272	very weakly similar to (99.8)AT5G57800| Symbols: FLP1, YRE, CER3, WAX2 | CER3/FLP1/WAX2/YRE (ECERIFERUM 3); catalyt	
EE438184	1.272	moderately similar to (257)AT4G13310| Symbols: CYP71A20 | CYP71A20 (cytochrome P450, family 71, subfamily A, polypeptide 2	
JCVI_18418	1.271	highly similar to (579)AT4G13020| Symbols: MHK | MHK | chr4:7603944-7606729 FORWARDweakly similar to (189)CDC2_MAI	
EE549228	1.270	very weakly similar to (91.3)AT3G50060| Symbols: MYB77 | MYB77; DNA binding / transcription factor | chr3:18569129-18570034 I	
RC_EE461708	1.270	no similarity	
JCVI_3627	1.270	moderately similar to (330)AT4G03960| Symbols: | tyrosine specific protein phosphatase family protein | chr4:1887671-1888998 FOR	
RC_EV015302	1.270	no similarity	
JCVI_27951	1.269	moderately similar to (202)AT4G33400| Symbols: | dem protein-related / defective embryo and meristems protein-related | chr4:16078	
JCVI_218	1.269	moderately similar to (365)AT5G27390| Symbols: | similar to unnamed protein product [Vitis vinifera] (GB:CAO17664.1); contains In	
JCVI_10805	1.269	weakly similar to (134)AT1G51650| Symbols: | ATP synthase epsilon chain, mitochondrial | chr1:19156348-19157309 FORWARDwe	
JCVI_16808	1.268	weakly similar to (152)AT1G76740| Symbols: | similar to unknown protein [Arabidopsis thaliana] (TAIR:AT1G76840.1); similar to ce	
JCVI_39221	1.267	no original description	
EE408376	1.267	weakly similar to (162)AT2G03520| Symbols: ATUPS4 | ATUPS4 (ARABIDOPSIS THALIANA UREIDE PERMEASE 4) | chr2:106	
JCVI_28642	1.267	no original description	
EE518209	1.267	very weakly similar to (89.0)AT1G63100| Symbols: | scarecrow transcription factor family protein | chr1:23403056-23405032 REVER	
AM394544	1.266	no similarity	
EE560298	1.266	no similarity	5.045
CO750106	1.266	no similarity	
EE446770	1.265	no similarity	
RC_ES958812	1.265	no similarity	
CV544986	1.265	no similarity	
EX037129	1.264	weakly similar to (183)AT1G03050| Symbols: | epsin N-terminal homology (ENTH) domain-containing protein / clathrin assembly pro	
EV103962	1.264	weakly similar to (146)AT4G13540| Symbols: | similar to unknown protein [Arabidopsis thaliana] (TAIR:AT3G23930.1); similar to un	
JCVI_12210	1.263	no original description	
JCVI_13709	1.263	no original description	
JCVI_38694	1.263	weakly similar to (168)AT2G34480| Symbols: | 60S ribosomal protein L18A (RPL18aB) | chr2:14539995-14541240 REVERSEweakl	
JCVI_34334	1.262	weakly similar to (145)AT2G35930| Symbols: | U-box domain-containing protein | chr2:15090180-15091415 REVERSE no original d	
DY026611	1.259	no similarity	
JCVI_38780	1.259	no original description	
EE551048	1.258	no similarity	-1.240
CD842919	1.258	no similarity	1.001
AM058087	1.257	weakly similar to (172)AT2G17550| Symbols: | similar to unknown protein [Arabidopsis thaliana] (TAIR:AT5G02390.1); similar to un	
JCVI_36197	1.257	moderately similar to (233)AT4G00350| Symbols: | MATE efflux family protein | chr4:151978-153988 FORWARD no original descri	
JCVI_21009	1.257	no original description	
RC_CX272297	1.256	no similarity	2.986
JCVI_10234	1.255	moderately similar to (244)AT4G30410| Symbols: | transcription factor | chr4:14871315-14871857 REVERSE no original description	
EV226155	1.255	no similarity	3.589
RC_EE567596	1.255	no similarity	
JCVI_432	1.254	moderately similar to (231)AT4G04780| Symbols: | similar to unknown protein [Arabidopsis thaliana] (TAIR:AT5G15690.1); similar t	
DW999676	1.253	very weakly similar to (89.7)AT5G12050| Symbols: | similar to unnamed protein product [Vitis vinifera] (GB:CAO45643.1) | chr5:389	
JCVI_31414	1.253	weakly similar to (191)AT4G29710| Symbols: | phosphodiesterase/nucleotide pyrophosphatase-related | chr4:14547007-14547408 RE	
JCVI_25393	1.253	no original description	
RC_H07474	1.253	no similarity	
JCVI_34834	1.252	moderately similar to (303)AT3G05630| Symbols: PDLZ2, PLDP2 | PLDP2 (PHOSPHOLIPASE D ZETA 2); phospholipase D | chr3:	
JCVI_9651	1.252	weakly similar to (146)AT4G25760| Symbols: ATGDU2 | ATGDU2 (ARABIDOPSIS THALIANA GLUTAMINE DUMPER 2) | chr4	
JCVI_24883	1.251	weakly similar to (101)AT2G20100| Symbols: | ethylene-responsive family protein | chr2:8685266-8687997 FORWARD no original d	
EE483558	1.250	no similarity	1.667
JCVI_7689	1.250	moderately similar to (400)AT3G24550| Symbols: ATPERK1 | ATPERK1 (PROLINE EXTENSIN-LIKE RECEPTOR KINASE 1); A	
EV128726	1.249	no similarity	
EE505233	1.248	no similarity	
JCVI_2497	1.248	no original description	
EE556940	1.248	no similarity	

JCVI_40151	1.248	weakly similar to (136)AT5G22920| Symbols: | zinc finger (C3HC4-type RING finger) family protein | chr5:7665146-7667034 FORW	
EL586758	1.248	no similarity	
EV217824	1.247	moderately similar to (390)AT3G51360| Symbols: | aspartyl protease family protein | chr3:19075273-19077539 REVERSE [21492] 41	
CD832107	1.247	weakly similar to (116)AT3G33520| Symbols: ARP6, SUF3, ESD1, ATARP6 | ATARP6; structural constituent of cytoskeleton | chr3:1	
ES961244	1.246	no similarity	
JCVI_20201	1.246	moderately similar to (352)AT5G24400| Symbols: EMB2024 | EMB2024 (EMBRYO DEFECTIVE 2024); catalytic | chr5:8330535-83	
EV086515	1.245	no similarity	
EE501848	1.245	no similarity	
EV146237	1.245	weakly similar to (152)AT4G21120| Symbols: CAT1 | AAT1 (CATIONIC AMINO ACID TRANSPORTER 1); cationic amino acid tra	
JCVI_41582	1.244	very weakly similar to (85.5)AT3G49370| Symbols: | calcium-dependent protein kinase, putative / CDPK, putative | chr3:18315939-183	
EV169287	1.243	no similarity	
EE569976	1.243	no similarity	
JCVI_27856	1.242	weakly similar to (155)AT4G31430| Symbols: | similar to unnamed protein product [Vitis vinifera] (GB:CAO43934.1) | chr4:1524851	
EE564834	1.242	no similarity	
EE566081	1.240	no similarity	
CN733443	1.240	no similarity	
CX190646	1.240	no similarity	
EE568186	1.239	very weakly similar to (89.4)AT2G42910| Symbols: | ribose-phosphate pyrophosphokinase 4 / phosphoribosyl diphosphate synthetase 4	
CD829818	1.239	no similarity	-1.695
EV099841	1.239	no similarity	
EV181239	1.239	no similarity	
EE567251	1.238	no similarity	1.307
EE567468	1.238	no similarity	
DY017020	1.237	no similarity	
CX194309	1.237	no similarity	
RC_EE565477	1.237	no similarity	
CN733048	1.236	no similarity	
JCVI_21024	1.235	weakly similar to (159)AT2G30150| Symbols: | UDP-glucoronosyl/UDP-glucosyl transferase family protein | chr2:12881783-1288319	
JCVI_9495	1.234	moderately similar to (230)AT5G61780| Symbols: | tudor domain-containing protein / nuclease family protein | chr5:24839238-248438	
CD827908	1.234	no similarity	
JCVI_35754	1.233	no original description	
EE548376	1.233	no similarity	
JCVI_41169	1.233	very weakly similar to (92.0)AT4G32300| Symbols: | lectin protein kinase family protein | chr4:15599976-15602441 FORWARD no or	
JCVI_34278	1.232	no original description	
RC_ES957422	1.232	no similarity	
ES938120	1.232	no similarity	
DY021547	1.231	no similarity	
EB041749	1.231	no similarity	
EE453815	1.231	very weakly similar to (84.3)AT1G25350| Symbols: OVA9 | OVA9 (OVULE ABORTION 9); glutamine-tRNA ligase | chr1:8889267-8	
H07287	1.231	no similarity	
JCVI_39802	1.229	no original description	
EV152480	1.229	very weakly similar to (85.5)AT1G11840| Symbols: ATGLX1 | ATGLX1 (GLYOXALASE I HOMOLOG); lactoylglutathione lyase | ch	
JCVI_23697	1.227	moderately similar to (229)AT1G73030| Symbols: VPS46.2 | VPS46.2 | chr1:27477599-27478509 FORWARD no original description	
JCVI_20622	1.226	no original description	1.891
CV432552	1.226	very weakly similar to (100)AT4G25980| Symbols: | cationic peroxidase, putative | chr4:13189402-13191516 FORWARD [16490] 40	
EV210294	1.224	no similarity	1.490
JCVI_39874	1.224	moderately similar to (261)AT2G20030| Symbols: | zinc finger (C3HC4-type RING finger) family protein | chr2:8654894-8656066 FC	
EE503950	1.224	no similarity	
JCVI_35218	1.223	moderately similar to (216)AT5G67060| Symbols: HEC1 | HEC1 (HECATE 1); transcription factor | chr5:26783502-26784227 FORW	
JCVI_13157	1.222	moderately similar to (320)AT1G18830| Symbols: | transducin family protein / WD-40 repeat family protein | chr1:6489300-6494209 I	
ES967984	1.221	no similarity	1.320
JCVI_42478	1.220	very weakly similar to (96.3)AT5G60390| Symbols: | elongation factor 1-alpha / EF-1-alpha | chr5:24306452-24307901 FORWARDve	
EV101103	1.220	weakly similar to (188)AT2G34420| Symbols: LHCB1.5, LHB1B2 | LHB1B2 (Photosystem II light harvesting complex gene 1.5); chlo	
JCVI_22578	1.220	nearly identical (1211)AT2G25800| Symbols: | similar to unknown protein [Arabidopsis thaliana] (TAIR:AT2G20010.2); similar to unk	
RC_AM060878	1.219	no similarity	1.046
JCVI_21427	1.219	no original description	
ES951218	1.218	no similarity	
JCVI_22340	1.218	no original description	
EV105059	1.218	no similarity	
EV178195	1.217	moderately similar to (309)AT1G22870| Symbols: | protein kinase family protein | chr1:8089490-8094162 FORWARD [21487] 82 99	
EV110475	1.217	no similarity	-1.217
JCVI_3538	1.217	moderately similar to (326)AT1G75910| Symbols: EXL4 | EXL4 (extracellular lipase 4); acyltransferase/ carboxylesterase/ lipase | chr1:	
EE552044	1.216	very weakly similar to (81.6)RL7A_ORYSA [20184] 1 221 257	1.613
AM395789	1.216	weakly similar to (143)AT5G35930| Symbols: | AMP-dependent synthetase and ligase family protein | chr5:14084427-14091634 REV	1.094
JCVI_13220	1.215	moderately similar to (252)AT3G07840| Symbols: | polygalacturonase, putative / pectinase, putative | chr3:2501899-2503481 REVERS	
JCVI_8900	1.214	moderately similar to (209)AT4G22310| Symbols: | similar to unknown protein [Arabidopsis thaliana] (TAIR:AT4G14695.1); similar t	
JCVI_17093	1.214	very weakly similar to (92.0)AT5G54690| Symbols: GAUT12, LGT6, IRX8 | GAUT12/IRX8/LGT6 (GALACTURONOSYLTRANSFE	
EV225710	1.213	no similarity	
EV090756	1.212	no similarity	
JCVI_33407	1.212	weakly similar to (187)AT1G48360| Symbols: | similar to unnamed protein product [Vitis vinifera] (GB:CAO45274.1); contains InterP	
ES967449	1.212	no similarity	
JCVI_9638	1.211	weakly similar to (183)AT1G52415| Symbols: | similar to Expressed protein [Arabidopsis thaliana] (TAIR:AT4G28405.1); contains Int	
EV037482	1.211	no similarity	
JCVI_1957	1.211	weakly similar to (117)AT5G48480| Symbols: | Identical to Uncharacterized protein At5g48480 [Arabidopsis Thaliana] (GB:Q9LV66)	
EV065172	1.210	no similarity	
JCVI_36018	1.210	no original description	
JCVI_39538	1.210	moderately similar to (257)AT4G20030| Symbols: | RNA recognition motif (RRM)-containing protein | chr4:10846372-10847256 FOI	
JCVI_30471	1.210	no original description	
JCVI_11395	1.210	moderately similar to (217)AT3G46480| Symbols: | oxidoreductase, acting on paired donors, with incorporation or reduction of molecu	
JCVI_42352	1.208	moderately similar to (342)AT3G09630| Symbols: | 60S ribosomal protein L4/L1 (RPL4A) | chr3:2953818-2955449 FORWARD no o	
EV157622	1.207	no similarity	
JCVI_18484	1.207	moderately similar to (347)AT5G58784| Symbols: | dehydrodolichyl diphosphate synthase, putative / DEDOL-PP synthase, putative | c	
JCVI_3808	1.207	weakly similar to (168)AT5G59310| Symbols: LTP4 | LTP4 (LIPID TRANSFER PROTEIN 4); lipid binding | chr5:23942522-2394299	

EV011894	1.206	no similarity	
JCVI_32121	1.206	no original description	
EX037308	1.205	moderately similar to (314)AT1G26310| Symbols: CAL1, AGL10, CAL | CAL (CAULIFLOWER); DNA binding / transcription factor	
JCVI_32296	1.205	moderately similar to (224)AT3G01440| Symbols: | oxygen evolving enhancer 3 (PsbQ) family protein | chr3:168485-169414 FORWA	
JCVI_16072	1.204	moderately similar to (214)AT5G66800| Symbols: | similar to unknown protein [Arabidopsis thaliana] (TAIR:AT3G50640.1); similar t	
EE516834	1.204	weakly similar to (161)AT1G66620| Symbols: | seven in absentia (SINA) protein, putative | chr1:24856469-24857707 REVERSEvery	
AT000438	1.203	no similarity	
JCVI_36165	1.202	no original description	
EE564606	1.201	no similarity	1.813
JCVI_21906	1.200	no original description	
EE546397	1.199	no similarity	3.205
JCVI_5915	1.198	moderately similar to (228)AT3G50270| Symbols: | transferase family protein | chr3:18646908-18648260 FORWARD no original desc	
JCVI_27059	1.198	moderately similar to (291)AT3G56620| Symbols: | integral membrane family protein / nodulin MtN21-related | chr3:20983675-20985	
JCVI_12374	1.197	weakly similar to (175)AT4G30720| Symbols: | oxidoreductase | chr4:14972218-14975467 REVERSE no original description	
EE556970	1.197	no similarity	
DN961164	1.195	no similarity	0.308
JCVI_17941	1.194	no original description	
JCVI_28053	1.194	no original description	
ES912768	1.193	moderately similar to (248)AT5G19880| Symbols: | peroxidase, putative | chr5:6720580-6722413 REVERSEweakly similar to (150)P	
EV149614	1.193	very weakly similar to (92.8)AT5G42980| Symbols: ATH3, ATTRXH3, TRXH3, ATTRX3 | ATTRX3 (thioredoxin H-type 3); thiol-dis	
JCVI_10422	1.193	no original description	
EV127720	1.193	no similarity	
JCVI_34489	1.191	moderately similar to (346)AT5G09540| Symbols: | DNAJ heat shock N-terminal domain-containing protein | chr5:2962423-2963265 I	
JCVI_3134	1.191	highly similar to (526)AT1G04770| Symbols: | male sterility MS5 family protein | chr1:1336563-1337766 REVERSE no original desc	
EE481259	1.190	weakly similar to (165)AT5G58230| Symbols: MEE70, MSI1 | MSI1 (MULTICOPY SUPRESSOR OF IRA1) | chr5:23573338-235752	
JCVI_8705	1.190	moderately similar to (294)AT5G54080| Symbols: HGO | HGO (HOMOGENTISATE 1,2-DIOXYGENASE); homogentisate 1,2-dioxy	2.093
JCVI_20091	1.190	weakly similar to (164)AT4G36120| Symbols: | similar to unknown protein [Arabidopsis thaliana] (TAIR:AT1G19835.1); similar to un	
EE552450	1.190	no similarity	
EE516683	1.189	weakly similar to (115)AT2G33130| Symbols: RALFL18 | RALFL18 (RALF-LIKE 18) | chr2:14053598-14053909 REVERSE [20185	
JCVI_28244	1.189	moderately similar to (244)AT5G37770| Symbols: CML24, TCH2 | TCH2 (TOUCH 2); calcium ion binding | chr5:15016305-1501679	
JCVI_32418	1.188	very weakly similar to (80.5)AT3G28770| Symbols: | similar to unknown protein [Arabidopsis thaliana] (TAIR:AT3G28810.1); similar	1.278
EE476768	1.188	moderately similar to (254)AT5G17350| Symbols: | similar to unknown protein [Arabidopsis thaliana] (TAIR:AT3G03280.1); similar t	
JCVI_39321	1.188	very weakly similar to (82.0)AT5G13000| Symbols: GSL12, ATGSL12 | ATGSL12 (GLUCAN SYNTHASE-LIKE 12); 1,3-beta-glucar	
EV150273	1.188	no similarity	
JCVI_22208	1.188	very weakly similar to (81.3)AT2G43710| Symbols: FAB2, SSI2 | SSI2 (fatty acid biosynthesis 2); acyl-[acyl-carrier-protein] desaturase	
JCVI_27767	1.188	moderately similar to (254)AT1G22360| Symbols: ATUGT85A2 | ATUGT85A2 (UDP-GLUCOSYL TRANSFERASE 85A2); UDP-gl	3.359
JCVI_5733	1.186	moderately similar to (204)AT1G21910| Symbols: | AP2 domain-containing transcription factor family protein | chr1:7696644-769733	
EE444230	1.186	moderately similar to (225)AT1G04110| Symbols: SDD1 | SDD1 (STOMATAL DENSITY AND DISTRIBUTION); subtilase | chr1:10	
JCVI_39320	1.185	weakly similar to (143)AT2G44440| Symbols: | emsy N terminus domain-containing protein / ENT domain-containing protein | chr2:1	
EV078713	1.185	no similarity	
JCVI_27559	1.185	highly similar to (972)AT4G33330| Symbols: PGSIP3 | PGSIP3 (PLANT GLYCOGENIN-LIKE STARCH INITIATION PROTEIN 3)	
EV141101	1.184	no similarity	
JCVI_17229	1.183	weakly similar to (105)AT1G30090| Symbols: | kelch repeat-containing F-box family protein | chr1:10559730-10560926 REVERSE n	
JCVI_24981	1.183	no original description	2.685
ES930799	1.182	very weakly similar to (100)AT5G02010| Symbols: ATROPGEF7, ROPGEF7 | ATROPGEF7/ROPGEF7 (KINASE PARTNER PROT	
EV092818	1.182	no similarity	
JCVI_19370	1.181	no original description	
EE460363	1.181	no similarity	
EV163624	1.179	weakly similar to (170)AT3G22270| Symbols: | similar to unknown protein [Arabidopsis thaliana] (TAIR:AT4G14990.1); similar to un	
JCVI_21812	1.178	no original description	
JCVI_36563	1.178	no original description	1.433
EV104180	1.176	weakly similar to (160)AT2G01600| Symbols: | epsin N-terminal homology (ENTH) domain-containing protein | chr2:268974-272355	
JCVI_40106	1.176	moderately similar to (225)AT5G26250| Symbols: | sugar transporter, putative | chr5:9196761-9198684 FORWARDweakly similar to	
EV205459	1.175	no similarity	
EV107555	1.174	very weakly similar to (87.0)AT1G74940| Symbols: | senescence-associated protein-related | chr1:28149945-28150726 FORWARD [21	
JCVI_7629	1.174	moderately similar to (203)AT2G47880| Symbols: | glutaredoxin family protein | chr2:19612194-19612502 FORWARD no original de	
EV106984	1.173	no similarity	
JCVI_8132	1.173	moderately similar to (210)AT3G23910| Symbols: | similar to unknown protein [Arabidopsis thaliana] (TAIR:AT3G24255.1); similar t	
CD829816	1.172	no similarity	
EV128714	1.172	no similarity	
JCVI_25373	1.171	moderately similar to (321)AT3G46960| Symbols: | ATP-dependent helicase | chr3:17301989-17309052 REVERSE no original descri	
RC_ES967585	1.169	no similarity	
RC_EE558216	1.168	no similarity	
L46475	1.167	no similarity	
EX131157	1.167	weakly similar to (147)AT5G42310| Symbols: | pentatricopeptide (PPR) repeat-containing protein | chr5:16933088-16935466 FORWA	
JCVI_22967	1.167	weakly similar to (179)AT3G58430| Symbols: | similar to meprin and TRAF homology domain-containing protein / MATH domain-co	
EE414049	1.166	no similarity	
EE410226	1.166	no similarity	
EV063338	1.166	no similarity	1.720
RC_EE526833	1.166	no similarity	
JCVI_19079	1.165	weakly similar to (106)AT4G27657| Symbols: | similar to unknown protein [Arabidopsis thaliana] (TAIR:AT4G27652.1) | chr4:13813	
EV214081	1.164	weakly similar to (195)AT1G49470| Symbols: | similar to unknown protein [Arabidopsis thaliana] (TAIR:AT1G55230.1); similar to hy	
CX192459	1.164	no similarity	
JCVI_12482	1.164	weakly similar to (129)AT2G19110| Symbols: HMA4 | HMA4 (Heavy metal ATPase 4); cadmium-transporting ATPase | chr2:8286560	
EV104851	1.162	no similarity	1.837
JCVI_2170	1.160	moderately similar to (332)AT5G64840| Symbols: ATGCN5 | ATGCN5 (Arabidopsis thaliana general control non-repressible 5) | chr5:	
EV171239	1.159	no similarity	
EV184925	1.159	no similarity	
JCVI_20977	1.159	weakly similar to (115)AT5G46370| Symbols: ATTPK2, KCO2, ATKCO2 | KCO2 (CA2+ ACTIVATED OUTWARD RECTIFYING	
JCVI_12760	1.158	very weakly similar to (82.0)AT5G14620| Symbols: DMT7, DRM2 | DRM2 (DOMAINS REARRANGED METHYLTRANSFERASE	
EV146900	1.157	no similarity	
JCVI_17711	1.157	no original description	
JCVI_14258	1.157	very weakly similar to (85.5)AT5G05830| Symbols: | zinc finger (C3HC4-type RING finger) family protein | chr5:1755911-1756826 F	

EE468341	1.157	no similarity	
EE566453	1.157	weakly similar to (140)AT4G35750| Symbols: | Rho-GTPase-activating protein-related | chr4:16940870-16941679 REVERSE [20153]	
CD812594	1.157	weakly similar to (143)AT2G47330| Symbols: | DEAD/DEAH box helicase, putative | chr2:19436153-19438687 REVERSE [13976]	
EV124157	1.157	moderately similar to (216)AT1G01790| Symbols: ATKEA1, KEA1 | KEA1 (K EFFLUX ANTIPORTER 1); potassium:hydrogen antip	
CX193946	1.156	weakly similar to (155)AT4G25630| Symbols: ATFIB2, FIB2 | FIB2 (FIBRILLARIN 2) | chr4:13074248-13076214 FORWARD [1680	
EE470884	1.156	no similarity	
EV152808	1.156	weakly similar to (101)AT4G04690| Symbols: | F-box family protein (FBX15) | chr4:2373997-2375133 REVERSE [21484]	
ES951296	1.155	moderately similar to (233)AT4G10630| Symbols: | glutaredoxin family protein | chr4:6566621-6567625 REVERSE [21423]	
ES978926	1.155	no similarity	
EE470870	1.155	no similarity	
EV151880	1.154	weakly similar to (147)AT4G21560| Symbols: | vacuolar protein sorting-associated protein 28 family protein / VPS28 family protein | c	
JCVI_38305	1.154	moderately similar to (288)AT1G55870| Symbols: ATPARN, AHG2 | AHG2/ATPARN | chr1:20899850-20901710 FORWARD no ori	
EH426427	1.154	moderately similar to (203)AT5G20490| Symbols: ATXIK, XIK | XIK (Myosin-like protein XIK); motor/ protein binding | chr5:69270	1.388
EX132052	1.153	moderately similar to (254)AT5G13590| Symbols: | similar to unnamed protein product [Vitis vinifera] (GB:CAO41555.1) | chr5:4374	
JCVI_19138	1.153	no original description	
EV104616	1.152	no similarity	
ES968782	1.152	no similarity	
EL587353	1.151	weakly similar to (108)AT1G15790| Symbols: | similar to protein binding / transcription cofactor [Arabidopsis thaliana] (TAIR:AT1G1	
JCVI_19076	1.151	no original description	3.098
EE561884	1.150	weakly similar to (107)AT1G22240| Symbols: APUM8 | APUM8 (ARABIDOPSIS PUMILIO 8); RNA binding | chr1:7853073-785495	-1.097
AM395567	1.150	no similarity	
EV226946	1.149	no similarity	
EE562182	1.149	no similarity	
EV130747	1.149	no similarity	
JCVI_16629	1.148	moderately similar to (248)AT3G51780| Symbols: ATBAG4 | ATBAG4 (ARABIDOPSIS THALIANA BCL-2-ASSOCIATED ATHAI	
EV210662	1.147	no similarity	
EV099499	1.146	no similarity	
EX124506	1.145	weakly similar to (144)AT4G29090| Symbols: | reverse transcriptase, putative / RNA-dependent DNA polymerase, putative | chr4:1433	
EE538327	1.144	no similarity	
EV207690	1.143	no similarity	
RC_JCVI_32770	1.143	no original description	
EX036222	1.143	moderately similar to (409)AT4G22730| Symbols: | leucine-rich repeat transmembrane protein kinase, putative | chr4:11941395-11943	
RC_ES965944	1.142	no similarity	
EE563724	1.141	weakly similar to (166)AT5G60615| Symbols: | Encodes a defensin-like (DEFL) family protein. | chr5:24383179-24383545 REVERSI	
RC_EX015383	1.140	no similarity	
JCVI_24416	1.139	highly similar to (707)AT3G46520| Symbols: ACT12 | ACT12 (ACTIN-12); structural constituent of cytoskeleton | chr3:17139552-171	
EE444120	1.139	weakly similar to (186)AT3G05670| Symbols: | PHD finger family protein | chr3:1653894-1657028 FORWARD [20160]	
JCVI_36364	1.138	highly similar to (505)AT3G20350| Symbols: | similar to unknown protein [Arabidopsis thaliana] (TAIR:AT1G50660.1); similar to unr	
EV156212	1.134	no similarity	
EX035128	1.134	moderately similar to (393)AT2G36780| Symbols: | UDP-glucoronosyl/UDP-glucosyl transferase family protein | chr2:15424697-1542	1.439
EX031510	1.134	no similarity	
EE546863	1.134	no similarity	
JCVI_198	1.132	moderately similar to (292)AT3G25910| Symbols: | zinc ion binding | chr3:9491073-9492191 FORWARD no original description	
JCVI_8079	1.132	weakly similar to (155)AT5G61880| Symbols: | signaling molecule-related | chr5:24867824-24868738 FORWARD no original descrip	
JCVI_14185	1.132	no original description	
EV111276	1.131	no similarity	
JCVI_35629	1.130	no original description	
CX191134	1.130	no similarity	
EV104642	1.129	no similarity	
EE560434	1.125	no similarity	
JCVI_34940	1.123	no original description	
RC_ES963926	1.121	no similarity	
EX089618	1.121	very weakly similar to (84.0)AT2G33000| Symbols: | ubiquitin-associated (UBA)/TS-N domain-containing protein-related | chr2:14013	
AM395361	1.121	no similarity	
EE546528	1.120	no similarity	
EV166563	1.119	no similarity	
EE417103	1.118	moderately similar to (253)AT3G55720| Symbols: | similar to unknown protein [Arabidopsis thaliana] (TAIR:AT5G05840.1); similar t	
EX041917	1.118	no similarity	
EV035083	1.115	weakly similar to (135)AT4G31560| Symbols: HCF153 | HCF153 | chr4:15295225-15296034 FORWARD [21441]	
JCVI_9275	1.115	no original description	
EE410492	1.115	no similarity	
AT000499	1.115	no similarity	
JCVI_5468	1.115	moderately similar to (296)AT5G67300| Symbols: ATMYBR1, ATMYB44, MYBR1 | ATMYB44/ATMYBR1/MYBR1 (MYB DOM/	
JCVI_39718	1.115	very weakly similar to (85.9)AT5G09680| Symbols: | cytochrome b5 domain-containing protein | chr5:2999363-3000186 REVERSE nc	
EV142971	1.114	no similarity	
JCVI_28912	1.114	moderately similar to (233)AT2G45110| Symbols: EXPB4, ATHEXP BETA 1.1, ATEXPB4 | ATEXPB4 (ARABIDOPSIS THALIAN	
RC_H07276	1.113	no similarity	
JCVI_8672	1.112	weakly similar to (136)AT2G25720| Symbols: | similar to hypothetical protein SDM1_56t00007 [Solanum demissum] (GB:AAU9032	
EX016787	1.111	weakly similar to (136)AT5G09630| Symbols: | protein binding / zinc ion binding | chr5:2986017-2987177 REVERSE [21808]	
JCVI_28425	1.111	no original description	1.256
ES264714	1.110	moderately similar to (306)AT3G13850| Symbols: LBD22 | LBD22 (LOB DOMAIN-CONTAINING PROTEIN 22) | chr3:4559917-45	
EE414902	1.109	no similarity	-1.004
JCVI_31954	1.109	weakly similar to (124)AT1G26320| Symbols: | NADP-dependent oxidoreductase, putative | chr1:9105227-9107016 FORWARD no or	
EE518095	1.108	no similarity	
RC_JCVI_42177	1.107	no original description	
CV973890	1.107	no similarity	1.186
RC_EX083949	1.106	no similarity	
RC_EE544274	1.106	no similarity	
CX190870	1.105	weakly similar to (114)AT1G02335| Symbols: GL22 | GL22 (GERMIN-LIKE PROTEIN SUBFAMILY 2 MEMBER 2 PRECURSOR	
JCVI_10850	1.105	moderately similar to (225)AT1G15010| Symbols: | similar to unknown protein [Arabidopsis thaliana] (TAIR:AT2G01300.1); similar t	
RC_EE561316	1.104	no similarity	
EX073784	1.103	no similarity	
EE568516	1.102	no similarity	

JCVI_16325	1.101	no original description	
EV221580	1.101	no similarity	
EV100923	1.100	no similarity	
ES930632	1.099	no similarity	
EV144282	1.099	no similarity	
ES991430	1.099	moderately similar to (307)AT5G13600| Symbols: | phototropic-responsive NPH3 family protein | chr5:4380435-4382500 FORWARD	
CV546281	1.097	weakly similar to (150)AT3G10610| Symbols: | 40S ribosomal protein S17 (RPS17C) | chr3:3319464-3319886 FORWARD [16551] 1	
RC_JCVI_31046	1.097	no original description	
EV216499	1.095	no similarity	
JCVI_39149	1.095	no original description	2.105
ES959318	1.094	no similarity	
JCVI_37107	1.094	no original description	
CD834522	1.094	weakly similar to (185)AT4G15350| Symbols: CYP705A2 | CYP705A2 (cytochrome P450, family 705, subfamily A, polypeptide 2); o	
JCVI_30071	1.093	moderately similar to (416)AT4G24150| Symbols: AtGRF8 | AtGRF8 (GROWTH-REGULATING FACTOR 8) | chr4:12535982-1253	
EV112906	1.092	no similarity	
EE563564	1.092	no similarity	
EV088013	1.092	weakly similar to (191)POLX_TOBAC [21444]	
JCVI_38437	1.091	weakly similar to (118)AT1G23600| Symbols: | similar to unknown protein [Arabidopsis thaliana] (TAIR:AT1G23570.2); similar to un	
EE418004	1.090	weakly similar to (135)AT5G14670| Symbols: ATARFA1B | ATARFA1B (ADP-RIBOSYLATION FACTOR A1B); GTP binding / ph	
EV205151	1.090	no similarity	
ES968154	1.089	no similarity	
EX022131	1.089	no similarity	
ES968127	1.087	no similarity	
JCVI_39772	1.087	weakly similar to (182)AT2G47270| Symbols: | transcription factor/ transcription regulator | chr2:19418811-19419119 REVERSE no c	
JCVI_8777	1.087	moderately similar to (234)AT1G04250| Symbols: IAA17, AXR3 | AXR3 (AUXIN RESISTANT 3); transcription factor | chr1:1136381	
JCVI_41343	1.086	no original description	
EE563352	1.085	no similarity	
EX050638	1.085	weakly similar to (150)AT2G13770| Symbols: | similar to ribosomal protein-like [Oryza sativa (japonica cultivar-group)] (GB:BAD379	
JCVI_38473	1.085	very weakly similar to (80.5)AT1G76920| Symbols: | F-box family protein (FBX3) | chr1:28897189-28898313 FORWARD no original	
JCVI_20996	1.085	no original description	
JCVI_22562	1.084	very weakly similar to (82.0)AT2G22440| Symbols: | similar to reverse transcriptase, putative / RNA-dependent DNA polymerase, puta	
EE561437	1.084	no similarity	
RC_EL591193	1.083	no similarity	
EV110683	1.082	no similarity	
JCVI_17733	1.081	moderately similar to (218)AT4G22390| Symbols: | F-box family protein-related | chr4:11813769-11815086 REVERSE no original de	
ES960748	1.081	no similarity	
EE567830	1.079	no similarity	
JCVI_19775	1.079	moderately similar to (466)AT5G13530| Symbols: KEG | KEG (KEEP ON GOING); protein binding / protein kinase/ ubiquitin-protein	
JCVI_28778	1.077	no original description	2.222
JCVI_21849	1.076	moderately similar to (486)AT5G41080| Symbols: | glycerophosphoryl diester phosphodiesterase family protein | chr5:16459217-1646	
RC_EV130845	1.075	no similarity	
CX271629	1.075	weakly similar to (118)AT1G60720| Symbols: | similar to unknown protein [Arabidopsis thaliana] (TAIR:AT1G33710.1); similar to pu	
EV140246	1.074	no similarity	
JCVI_17398	1.073	moderately similar to (288)AT4G14210| Symbols: PDS, PDE226, PDS3 | PDS3 (PHYTOENE DESATURASE) | chr4:8190421-81947	
EV220592	1.073	no similarity	
JCVI_16185	1.071	weakly similar to (193)AT1G20220| Symbols: | nucleic acid binding | chr1:7005081-7007276 REVERSE no original description	
ES966548	1.070	no similarity	
JCVI_35557	1.070	moderately similar to (213)AT1G01260| Symbols: | basic helix-loop-helix (bHLH) family protein | chr1:109595-111367 FORWARD r	
JCVI_293	1.069	weakly similar to (169)AT3G12480| Symbols: | transcription factor, putative | chr3:3958072-3960285 FORWARD no original descript	
EV152233	1.069	no similarity	1.294
JCVI_38516	1.069	no original description	
EV194445	1.067	weakly similar to (120)AT1G31550| Symbols: | GDSL-motif lipase, putative | chr1:11295616-11297265 REVERSE [21489] 41 722 7	
EE567416	1.066	no similarity	
JCVI_11761	1.065	no original description	1.254
DY003886	1.064	weakly similar to (140)AT3G63350| Symbols: HSFA7B, AT-HSFA7B | AT-HSFA7B (Arabidopsis thaliana heat shock transcription fac	
ES933334	1.063	no similarity	
JCVI_4571	1.062	moderately similar to (266)AT5G59820| Symbols: ZAT12, RHL41 | RHL41 (RESPONSIVE TO HIGH LIGHT 41); nucleic acid bindi	
JCVI_3696	1.062	moderately similar to (420)AT5G26110| Symbols: | ATP binding / protein kinase | chr5:9118242-9118993 REVERSE no original desc	
JCVI_5445	1.061	no original description	
DW997179	1.061	no similarity	
CD826637	1.060	moderately similar to (350)AT4G02350| Symbols: | exocyst complex subunit Sec15-like family protein | chr4:1038157-1040571 FORV	
JCVI_40879	1.060	very weakly similar to (83.6)AT5G46910| Symbols: | transcription factor jumonji (jmj) family protein | chr5:19065007-19068107 FORV	
DT317682	1.060	no similarity	
JCVI_16376	1.060	moderately similar to (464)AT5G65910| Symbols: | BSD domain-containing protein | chr5:26378818-26380567 REVERSE no origina	
JCVI_13274	1.056	moderately similar to (369)AT3G24800| Symbols: PRT1 | PRT1 (PROTEOLYSIS 1); ubiquitin-protein ligase | chr3:9055660-9057802	
EV134884	1.056	no similarity	
EE530681	1.055	no similarity	
CX188787	1.055	no similarity	
RC_ES978013	1.055	no similarity	
EV131420	1.054	no similarity	
EE559481	1.054	no similarity	
JCVI_15747	1.054	weakly similar to (148)AT4G27280| Symbols: | calcium-binding EF hand family protein | chr4:13663776-13664168 REVERSE no orig	
JCVI_31294	1.052	moderately similar to (459)AT4G38890| Symbols: | dihydrouridine synthase family protein | chr4:18135903-18139094 REVERSE no	-1.448
JCVI_39499	1.052	weakly similar to (108)AT2G24650| Symbols: | transcriptional factor B3 family protein | chr2:10487807-10494650 REVERSE no orig	
EE567405	1.051	no similarity	
EV107687	1.050	no similarity	
JCVI_7424	1.050	no original description	
EV039920	1.049	moderately similar to (239)AT2G42550| Symbols: | protein kinase family protein | chr2:17720274-17721308 FORWARDvery weakly	
JCVI_14583	1.049	weakly similar to (118)AT5G24490| Symbols: | 30S ribosomal protein, putative | chr5:8365693-8367181 FORWARDvery weakly simi	
EE524452	1.048	weakly similar to (169)AT1G14560| Symbols: | mitochondrial substrate carrier family protein | chr1:4981295-4983077 FORWARD [2	
EV100862	1.048	very weakly similar to (85.9)AT1G57750| Symbols: MAH1, CYP96A15 | CYP96A15/MAH1 (MID-CHAIN ALKANE HYDROXYLA	
JCVI_40	1.048	moderately similar to (432)AT1G78630| Symbols: EMB1473 | EMB1473 (EMBRYO DEFECTIVE 1473); structural constituent of ribo	
EE504672	1.047	no similarity	

JCVI_18195	1.047	moderately similar to (215)AT3G15210| Symbols: ATERF-4, ERF4, RAP2.5, ATERF4 | ATERF-4/ATERF4/ERF4/RAP2.5 (ETHYLE	
JCVI_12621	1.044	no original description	
JCVI_30114	1.042	no original description	
EV099936	1.041	moderately similar to (241)AT1G20020| Symbols: ATLFNR2 | ATLFNR2 (LEAF FNR 2); poly(U) binding | chr1:6942842-6944859 F	
JCVI_33471	1.041	no original description	2.085
EV178823	1.038	no similarity	
EV098082	1.037	no similarity	1.317
JCVI_39622	1.037	weakly similar to (157)AT2G15910| Symbols: | CSL zinc finger domain-containing protein | chr2:6939999-6942415 REVERSE no ori	
JCVI_6413	1.037	weakly similar to (102)AT1G08920| Symbols: | sugar transporter, putative | chr1:2867449-2870193 FORWARD no original descriptio	
EV091597	1.035	moderately similar to (261)AT5G23890| Symbols: | similar to unknown protein [Arabidopsis thaliana] (TAIR:AT5G52410.2); similar t	1.086
CA991582	1.035	very weakly similar to (83.6)AT4G38800| Symbols: ATMTN1 | ATMTN1; catalytic/ methylthioadenosine nucleosidase | chr4:1811334	
RC_JCVI_35546	1.034	no original description	
EE568839	1.034	no similarity	
JCVI_20936	1.032	no original description	
EV218157	1.032	no similarity	
BG543914	1.031	weakly similar to (102)AT4G31700| Symbols: RPS6 | RPS6 (RIBOSOMAL PROTEIN S6) | chr4:15346312-15347081 REVERSEvery	
JCVI_26963	1.029	very weakly similar to (88.2)AT2G38500| Symbols: | similar to DTA4 (DOWNSTREAM TARGET OF AGL15-4) [Arabidopsis thaliar	
JCVI_15983	1.027	moderately similar to (394)AT1G10910| Symbols: | similar to PTAC2 (PLASTID TRANSCRIPTIONALLY ACTIVE2) [Arabidopsis t	
ES965081	1.027	no similarity	
JCVI_19002	1.026	weakly similar to (124)AT4G02075| Symbols: PIT1 | PIT1 (PITCHOUN 1); protein binding / zinc ion binding | chr4:913555-916414 R	
CX268927	1.025	no similarity	
EV103978	1.025	no similarity	
EV208389	1.025	no similarity	
EX016865	1.024	no similarity	
JCVI_33662	1.024	no original description	
EV135772	1.024	no similarity	
EV204179	1.022	no similarity	
EE401649	1.022	weakly similar to (103)AT2G42380| Symbols: | bZIP transcription factor family protein | chr2:17654283-17655738 REVERSE [20197	
JCVI_20087	1.022	weakly similar to (182)AT1G19230| Symbols: | respiratory burst oxidase protein E (RbohE) / NADPH oxidase | chr1:6644180-664914	
EV148759	1.020	no similarity	
JCVI_18052	1.020	moderately similar to (254)AT3G11980| Symbols: MS2 | MS2 (MALE STERILITY 2) | chr3:3814490-3816933 FORWARD no origin	
EV218684	1.019	no similarity	
JCVI_41904	1.018	no original description	
JCVI_29660	1.018	moderately similar to (262)AT2G44520| Symbols: COX10 | COX10 (CYTOCHROME C OXIDASE 10); prenyltransferase | chr2:1838	
JCVI_10099	1.018	moderately similar to (225)AT5G51750| Symbols: ATSBT1.3 | ATSBT1.3; subtilase | chr5:21037492-21039834 FORWARD no origin	
JCVI_18456	1.017	weakly similar to (180)AT1G07150| Symbols: MAPKKK13 | MAPKKK13 (Mitogen-activated protein kinase kinase kinase 13); kinase	
RC_H07682	1.017	no similarity	
RC_EV112452	1.016	no similarity	
EV206757	1.015	weakly similar to (102)AT3G27690| Symbols: LHCB2.3, LHCB2, LHCB2:4 | LHCB2:4 (Photosystem II light harvesting complex gene	
EV197442	1.015	very weakly similar to (99.0)AT4G38970| Symbols: | fructose-bisphosphate aldolase, putative | chr4:18163763-18165653 REVERSEve	
JCVI_39268	1.015	moderately similar to (245)AT5G41270| Symbols: | similar to Os01g0541600 [Oryza sativa (japonica cultivar-group)] (GB:NP_00104	
EE472257	1.013	weakly similar to (159)AT4G11580| Symbols: | F-box family protein | chr4:7006642-7007732 REVERSE [20163]	
JCVI_5111	1.011	no original description	
RC_JCVI_41878	1.011	no original description	
EV222917	1.011	moderately similar to (237)AT5G60390| Symbols: | elongation factor 1-alpha / EF-1-alpha | chr5:24306452-24307901 FORWARDmo	
EX087640	1.009	moderately similar to (212)AT1G23380| Symbols: KNAT6L, KNAT6S, KNAT6 | KNAT6 (Knotted-like Arabidopsis thaliana 6); DNA	
JCVI_39590	1.008	moderately similar to (366)AT4G23460| Symbols: | beta-adaptin, putative | chr4:12243909-12248908 REVERSE no original descriptio	
EX065651	1.008	moderately similar to (298)AT1G55570| Symbols: SKS12 | SKS12 (SKU5 Similar 12); copper ion binding / oxidoreductase | chr1:2076	
EX093982	1.007	moderately similar to (378)AT1G12260| Symbols: EMB2749, ANAC007, VND4 | VND4 (VASCULAR RELATED NAC-DOMAIN P	
EV226859	1.006	moderately similar to (326)AT5G24470| Symbols: PRR5, APRR5 | APRR5 (PSEUDO-RESPONSE REGULATOR 5); transcription re	
EV170364	1.004	weakly similar to (174)AT5G06950| Symbols: TGA2, AHBP-1B | AHBP-1B (bZIP transcription factor HBP-1b homolog) | chr5:21523	
EL590426	1.002	moderately similar to (276)AT5G02010| Symbols: ATROPGEF7, ROPGEF7 | ATROPGEF7/ROPGEF7 (KINASE PARTNER PROTE	
EV058225	1.002	weakly similar to (179)AT5G25320| Symbols: | ACT domain-containing protein | chr5:8787406-8789533 REVERSE [21442]	
EE449456	0.999	no similarity	
EL587091	0.998	no similarity	
JCVI_14015	0.997	moderately similar to (370)AT5G37300| Symbols: | similar to unknown protein [Arabidopsis thaliana] (TAIR:AT2G38995.1); similar t	
EV195092	0.996	very weakly similar to (97.1)AT1G69800| Symbols: | CBS domain-containing protein | chr1:26278079-26279988 REVERSE [21489] 3	
EV209409	0.988	weakly similar to (169)AT5G25150| Symbols: TAF5 | TAF5 (TBP-ASSOCIATED FACTOR 5); nucleotide binding | chr5:8677120-866	
EV158470	0.988	no similarity	
JCVI_41467	0.983	no original description	
JCVI_4595	0.979	moderately similar to (266)AT4G34970| Symbols: | actin binding | chr4:16653789-16654598 FORWARDweakly similar to (176)ADF	
JCVI_5890	0.977	weakly similar to (192)AT2G22500| Symbols: | mitochondrial substrate carrier family protein | chr2:9570611-9571552 REVERSE no	
JCVI_12349	0.976	no original description	1.277
EE565485	0.971	no similarity	
RC_EV012511	0.967	no similarity	
EE392280	0.954	very weakly similar to (96.7)AT1G45190| Symbols: | similar to unknown protein [Arabidopsis thaliana] (TAIR:AT3G11990.1); contain	
JCVI_2142	0.950	no original description	
JCVI_14932	0.945	no original description	
EV220691	0.943	no similarity	
EV142319	0.943	no similarity	
JCVI_27062	0.936	no original description	
JCVI_13988	0.936	moderately similar to (213)AT4G21895| Symbols: | similar to AT hook motif-containing protein [Arabidopsis thaliana] (TAIR:AT5G5	
EV130062	0.932	no similarity	
EE535293	0.929	moderately similar to (240)AT5G11560| Symbols: | catalytic | chr5:3709735-3713995 REVERSE [20150]	
JCVI_35720	0.921	moderately similar to (305)AT4G32510| Symbols: | anion exchanger | chr4:15685909-15688817 REVERSE no original description	
EV098714	0.920	moderately similar to (266)AT2G35610| Symbols: | similar to unknown protein [Arabidopsis thaliana] (TAIR:AT1G70630.1); similar t	
H07700	0.919	no similarity	
JCVI_15621	0.919	no original description	
JCVI_34192	0.914	no original description	
JCVI_25545	0.911	moderately similar to (248)AT1G11020| Symbols: | zinc finger (C3HC4-type RING finger) family protein | chr1:3676968-3678350 FC	
JCVI_16737	0.897	no original description	
JCVI_19949	0.896	moderately similar to (297)AT2G19050| Symbols: | GDSL-motif lipase/hydrolase family protein | chr2:8260498-8262616 FORWARD	
ES966296	0.878	no similarity	

RC_EV204033	0.878	no similarity	
JCVI_26199	0.867	no original description	
JCVI_19464	0.860	highly similar to (565)AT1G04990| Symbols: | zinc finger (CCCH-type) family protein | chr1:1419367-1421453 REVERSEmoderately	
JCVI_41069	0.859	highly similar to (556)AT2G38010| Symbols: | ceramidase family protein | chr2:15913940-15916945 FORWARD no original descripti	
JCVI_31489	0.858	moderately similar to (308)AT2G41840| Symbols: | 40S ribosomal protein S2 (RPS2C) | chr2:17467094-17468476 REVERSE no orig	-0.964
EE568162	0.854	no similarity	
EV143884	0.853	no similarity	
JCVI_23627	0.846	moderately similar to (295)AT5G67310| Symbols: CYP81G1 | CYP81G1 (cytochrome P450, family 81, subfamily G, polypeptide 1); c	
JCVI_23654	0.846	no original description	-1.526
JCVI_15762	0.845	moderately similar to (462)AT4G18480| Symbols: CH42, CH-42, CHL11, CHLI-1, CHLI1 | CHLI1 (CHLORINA 42); magnesium che	
EE558080	0.842	no similarity	
CV433940	0.831	no similarity	
JCVI_11165	0.817	no original description	
RC_EV013640	0.815	no similarity	
EV127571	0.815	no similarity	
JCVI_15980	0.805	weakly similar to (112)AT1G54050| Symbols: | 17.4 kDa class III heat shock protein (HSP17.4-CIII) | chr1:20183226-20183790 REVI	
EV176914	0.802	no similarity	
RC_EE565963	0.802	no similarity	
JCVI_36562	0.801	no original description	
EE560924	0.786	no similarity	
EV123380	0.775	weakly similar to (135)AT4G00440| Symbols: | similar to unknown protein [Arabidopsis thaliana] (TAIR:AT2G45900.1); similar to un	
ES960782	0.772	no similarity	
JCVI_3653	0.770	moderately similar to (473)AT1G26480| Symbols: GF14 IOTA, GRF12 | GRF12 (GENERAL REGULATORY FACTOR 12); protein	
ES983837	0.766	no similarity	
RC_EX064898	0.765	no similarity	
DY010873	0.756	no similarity	
JCVI_1068	0.745	moderately similar to (212)AT3G04320| Symbols: | endopeptidase inhibitor | chr3:1143822-1144433 FORWARD no original descripti	
JCVI_37982	0.740	no original description	
CX271816	0.729	weakly similar to (102)AT1G04150| Symbols: | C2 domain-containing protein | chr1:1081207-1084245 REVERSE [16815]	
JCVI_33335	0.728	weakly similar to (187)AT3G06390| Symbols: | integral membrane family protein | chr3:1938919-1939713 REVERSE no original desc	
DN965438	0.723	no similarity	
JCVI_33057	0.719	weakly similar to (111)AT5G60850| Symbols: OBP4 | OBP4 (OBF BINDING PROTEIN 4); DNA binding / transcription factor | chr5:2	
RC_ES966655	0.718	no similarity	
CX190760	0.706	no similarity	
EE562726	0.702	very weakly similar to (93.6)AT2G01450| Symbols: ATMPK17 | ATMPK17 (Arabidopsis thaliana MAP kinase 17); MAP kinase | chr2:	
JCVI_19732	0.696	moderately similar to (270)AT1G47610| Symbols: | transducin family protein / WD-40 repeat family protein | chr1:17507276-1750833	
DY007829	0.683	weakly similar to (113)AT4G14695| Symbols: | similar to unknown protein [Arabidopsis thaliana] (TAIR:AT4G22310.1); similar to un	
EV132032	0.672	no similarity	
CV432515	0.644	weakly similar to (138)AT3G10400| Symbols: | RNA recognition motif (RRM)-containing protein | chr3:3232641-3233426 FORWAR	
JCVI_41442	0.643	no original description	
EX134503	0.634	no similarity	
EX133342	0.631	no similarity	
JCVI_14485	0.604	no original description	
EV107694	0.594	no similarity	
JCVI_18802	0.577	moderately similar to (350)AT4G28040| Symbols: | nodulin MtN21 family protein | chr4:13940887-13942207 FORWARD no original	
CD823053	0.528	no similarity	
EX095814	0.509	weakly similar to (180)AT1G34220| Symbols: | similar to unknown protein [Arabidopsis thaliana] (TAIR:AT4G35730.1); similar to hy	
EX110598	0.502	no similarity	
JCVI_34124	0.497	moderately similar to (370)AT5G51760| Symbols: AHG1 | AHG1 (ABA-HYPERSENSITIVE GERMINATION 1); protein serine/threo	
JCVI_22401	0.470	moderately similar to (340)AT5G06930| Symbols: | similar to nucleolar protein gar2-related [Arabidopsis thaliana] (TAIR:AT2G42320	
JCVI_39816	0.469	no original description	
EX070812	0.410	no similarity	
JCVI_9447	0.250	no original description	
EE523558	0.206	moderately similar to (209)AT1G17890| Symbols: GER2 | GER2; catalytic | chr1:6154471-6155589 REVERSEweakly similar to (158	
JCVI_25196	0.172	moderately similar to (272)AT2G28880| Symbols: EMB1997 | EMB1997 (EMBRYO DEFECTIVE 1997); anthranilate synthase/ catal	
JCVI_35033	0.110	weakly similar to (184)AT5G56290| Symbols: PEX5 | PEX5 (PEROXIN 5); peroxisome matrix targeting signal-1 binding | chr5:22803	
RC_ES979191	0.069	no similarity	
RC_ES955671	0.033	no similarity	
