# Supplementary material for: Influence of Light and Temperature on Gene Expression Leading to Accumulation of Specific Flavonol Glycosides and Hydroxycinnamic Acid Derivatives in Kale (Brassica oleracea var. sabellica)
Source: Front Plant Sci. 2016 Mar 30;7:326. doi: 10.3389/fpls.2016.00326 (PMC4812050; doi:10.3389/fpls.2016.00326)
Supplement: Supplementary file 2 [file Table2.PDF]

Identifier	log2-fold HL induced	Description	log2-fold LT-HT
EV107953	9.609	weakly similar to (144)AT5G45890| Symbols: SAG12 | SAG12 (SENESCENCE-ASSOCIATED GENE 12); cysteine-type peptidase | chr5	
EV108057	9.267	no similarity	
EE465824	6.500	weakly similar to (193)AT1G62975| Symbols: | basic helix-loop-helix (bHLH) family protein (bHLH125) | chr1:23332566-23334187 FOR	-5.262
JCVI_26670	5.719	moderately similar to (269)AT1G02470| Symbols: | similar to unknown protein [Arabidopsis thaliana] (TAIR:AT1G02475.1); similar to u	
JCVI_36187	5.675	no original description	
EV107830	5.639	no similarity	3.057
EE568322	5.540	weakly similar to (124)AT3G08860| Symbols: | alanine--glyoxylate aminotransferase, putative / beta-alanine-pyruvate aminotransferase, p	
EV111066	5.508	no similarity	3.038
JCVI_2085	5.501	moderately similar to (258)AT3G22840| Symbols: ELIP, ELIP1 | ELIP1 (EARLY LIGHT-INDUCABLE PROTEIN); chlorophyll binding	3.152
JCVI_17490	5.416	moderately similar to (304)AT4G29930| Symbols: | basic helix-loop-helix (bHLH) family protein | chr4:14644114-14645174 FORWARD	-7.488
JCVI_342	5.381	moderately similar to (240)AT1G72290| Symbols: | trypsin and protease inhibitor family protein / Kunitz family protein | chr1:27219514-2	-4.634
EV065788	5.268	weakly similar to (159)AT5G04200| Symbols: ATMC9 | ATMC9 (METACASPASE 9); caspase/ cysteine-type peptidase | chr5:1153894-1	
EV112363	5.203	no similarity	
JCVI_10557	5.128	moderately similar to (270)AT5G39610| Symbols: ANAC092, ATNAC6, ATNAC2 | ANAC092/ATNAC2/ATNAC6 (Arabidopsis NAC do	
CD814689	5.063	moderately similar to (284)AT1G71880| Symbols: ATSUC1, SUC1 | SUC1 (SUCROSE-PROTON SYMPORTER 1); carbohydrate transm	-2.358
JCVI_213	5.014	highly similar to (532)AT5G37600| Symbols: ATGSR1 | ATGSR1 (Arabidopsis thaliana glutamine synthase clone R1); glutamate-ammoni	
EV193206	4.987	weakly similar to (113)AT2G37180| Symbols: PIP2;3, PIP2C, RD28 | RD28 (plasma membrane intrinsic protein 2;3); water channel | chr2	
EV114900	4.939	moderately similar to (214)AT4G13930| Symbols: SHM4 | SHM4 (SERINE HYDROXYMETHYLTRANSFERASE 4); glycine hydroxym	
EV111322	4.902	no similarity	3.040
JCVI_2879	4.825	weakly similar to (109)AT4G32940| Symbols: GAMMAVPE, GAMMA-VPE | GAMMA-VPE (Vacuolar processing enzyme gamma); cys	
EE451932	4.816	very weakly similar to (87.8)AT3G08860| Symbols: | alanine--glyoxylate aminotransferase, putative / beta-alanine-pyruvate aminotransfera	
JCVI_22811	4.810	moderately similar to (202)AT4G14690| Symbols: ELIP2 | ELIP2 (EARLY LIGHT-INDUCIBLE PROTEIN 2); chlorophyll binding | chr4	2.413
JCVI_5021	4.785	moderately similar to (379)AT1G17170| Symbols: GST, ATGSTU24 | ATGSTU24 (ARABIDOPSIS THALIANA GLUTATHIONE S-TR	-3.748
DN964631	4.772	moderately similar to (246)AT1G42970| Symbols: GAPB | GAPB (GLYCERALDEHYDE-3-PHOSPHATE DEHYDROGENASE B SUBU	
JCVI_21173	4.768	moderately similar to (330)AT1G04350| Symbols: | 2-oxoglutarate-dependent dioxygenase, putative | chr1:1165295-1166537 FORWARD	
ES930029	4.747	weakly similar to (150)AT3G05280| Symbols: | integral membrane Yip1 family protein | chr3:1503998-1505560 REVERSE [20185]	
JCVI_15282	4.743	moderately similar to (493)AT5G07990| Symbols: CYP75B1, D501, TT7 | TT7 (TRANSPARENT TESTA 7); flavonoid 3'-monooxygenas	
EE531023	4.711	weakly similar to (152)AT4G35060| Symbols: | heavy-metal-associated domain-containing protein / copper chaperone (CCH)-related | chr	
JCVI_34200	4.680	weakly similar to (187)AT5G48300| Symbols: APS1, ADG1 | ADG1 (ADP GLUCOSE PYROPHOSPHORYLASE SMALL SUBUNIT 1);	
DY025170	4.646	moderately similar to (404)AT5G54060| Symbols: UF3GT | UF3GT (UDP-GLUCOSE:FLAVONOID 3-O-GLUCOSYLTRANSFERASE);	
JCVI_6670	4.606	moderately similar to (382)AT2G02990| Symbols: RNS1 | RNS1 (RIBONUCLEASE 1); endoribonuclease | chr2:873713-874666 FORWA	-3.953
JCVI_13315	4.604	moderately similar to (404)AT5G26220| Symbols: | ChaC-like family protein | chr5:9163181-9164616 REVERSE no original description	
JCVI_14458	4.588	moderately similar to (477)AT1G71880| Symbols: ATSUC1, SUC1 | SUC1 (SUCROSE-PROTON SYMPORTER 1); carbohydrate transm	-1.544
ES951266	4.580	no similarity	
EX131200	4.518	moderately similar to (446)AT5G49690| Symbols: | UDP-glucoronosyl/UDP-glucosyl transferase family protein | chr5:20207194-2020857	
JCVI_8160	4.510	moderately similar to (274)AT1G62760| Symbols: | invertase/pectin methylesterase inhibitor family protein | chr1:23241239-23242177 RI	
JCVI_21470	4.506	moderately similar to (382)AT1G65680| Symbols: EXPB2, ATHEXP BETA 1.4, ATEXPB2 | ATEXPB2 (ARABIDOPSIS THALIANA E	-3.386
JCVI_13432	4.461	moderately similar to (392)AT1G17180| Symbols: ATGSTU25 | ATGSTU25 (Arabidopsis thaliana Glutathione S-transferase (class tau) 2	-3.141
AM394020	4.460	very weakly similar to (85.5)AT5G28237| Symbols: | tryptophan synthase, beta subunit, putative | chr5:10207481-10213546 REVERSE [2	-5.531
EX020681	4.454	very weakly similar to (81.3)AT5G61340| Symbols: | similar to unknown protein [Arabidopsis thaliana] (TAIR:AT1G26650.1); similar to l	
ES940777	4.439	moderately similar to (244)AT1G06790| Symbols: | RNA polymerase Rpb7 N-terminal domain-containing protein | chr1:2087644-208928	
AI352931	4.425	weakly similar to (152)AT1G02360| Symbols: | chitinase, putative | chr1:472138-473116 REVERSEweakly similar to (116)CHI2_BRAN	
DY023111	4.416	moderately similar to (209)AT5G61890| Symbols: | AP2 domain-containing transcription factor family protein | chr5:24869865-24870970	
JCVI_29081	4.414	weakly similar to (123)AT5G13180| Symbols: ANAC083 | ANAC083 (Arabidopsis NAC domain containing protein 83); transcription fact	
EV118424	4.405	weakly similar to (125)AT1G69523| Symbols: | UbiE/COQ5 methyltransferase family protein | chr1:26133490-26134820 FORWARD [21	
JCVI_36089	4.398	moderately similar to (250)AT3G54040| Symbols: | photoassimilate-responsive protein-related | chr3:20025010-20026277 REVERSE no	
EE406338	4.390	moderately similar to (317)AT4G35090| Symbols: CAT2 | CAT2 (CATALASE 2); catalase | chr4:16701110-16703220 REVERSEmoderat	
JCVI_18529	4.382	highly similar to (910)AT2G45220| Symbols: | pectinesterase family protein | chr2:18651355-18653468 REVERSEhighly similar to (655)	
JCVI_8697	4.377	moderately similar to (292)AT2G30140| Symbols: | UDP-glucoronosyl/UDP-glucosyl transferase family protein | chr2:12879277-1288076	-2.302
ES988324	4.373	very weakly similar to (95.9)AT1G71400| Symbols: | disease resistance family protein / LRR family protein | chr1:26913567-26916110 FC	
ES909993	4.361	moderately similar to (266)AT4G27950| Symbols: CRF4 | CRF4 (CYTOKININ RESPONSE FACTOR 4); DNA binding / transcription fac	-3.328
EE565021	4.345	weakly similar to (110)AT1G20850| Symbols: XCP2 | XCP2 (XYLEM CYSTEINE PEPTIDASE 2); cysteine-type peptidase/ peptidase | c	
AM385209	4.340	moderately similar to (207)AT5G52470| Symbols: FBR1, ATFIB1, ATFBR1, SKIP7, FIB1 | FIB1 (FIBRILLARIN 1) | chr5:21311516-213	
EV061088	4.318	weakly similar to (129)AT3G61180| Symbols: | zinc finger (C3HC4-type RING finger) family protein | chr3:22656655-22658265 FORWA	
EV116303	4.310	moderately similar to (272)AT5G39320| Symbols: | UDP-glucose 6-dehydrogenase, putative | chr5:15760482-15761924 FORWARDmode	
JCVI_20453	4.308	moderately similar to (337)AT1G69523| Symbols: | UbiE/COQ5 methyltransferase family protein | chr1:26133490-26134820 FORWARD	
JCVI_2015	4.298	moderately similar to (300)AT3G21710| Symbols: | unknown protein | chr3:7648387-7649540 FORWARD no original description	2.972
EX112823	4.286	weakly similar to (158)AT2G37180| Symbols: PIP2;3, PIP2C, RD28 | RD28 (plasma membrane intrinsic protein 2;3); water channel | chr2	
JCVI_501	4.267	highly similar to (528)AT2G38380| Symbols: | peroxidase 22 (PER22) (P22) (PRXEA) / basic peroxidase E | chr2:16083521-16085392 F	
AM388781	4.257	moderately similar to (275)AT1G10070| Symbols: ATBCAT-2 | ATBCAT-2; catalytic | chr1:3288674-3290166 FORWARD [20118]	
JCVI_39156	4.252	moderately similar to (293)AT4G35060| Symbols: | heavy-metal-associated domain-containing protein / copper chaperone (CCH)-related	
JCVI_13834	4.223	moderately similar to (276)AT1G71880| Symbols: ATSUC1, SUC1 | SUC1 (SUCROSE-PROTON SYMPORTER 1); carbohydrate transm	
CO749461	4.215	no similarity	
JCVI_2411	4.196	moderately similar to (468)AT5G13930| Symbols: CHS, TT4, ATCHS | ATCHS/CHS/TT4 (CHALCONE SYNTHASE); naringenin-chalc	
ES902248	4.191	moderately similar to (286)AT1G28230| Symbols: ATPUP1, PUP1 | PUP1 (PURINE PERMEASE 1); purine transmembrane transporter |	2.909
CN728781	4.187	moderately similar to (429)AT3G13620| Symbols: | amino acid permease family protein | chr3:4450911-4452563 REVERSE [15719]	
EE566908	4.174	no similarity	
JCVI_24588	4.173	moderately similar to (341)AT5G17220| Symbols: GST26, TT19, ATGSTF12 | ATGSTF12 (GLUTATHIONE S-TRANSFERASE 26); glu	
JCVI_14407	4.169	no original description	
EV077483	4.155	very weakly similar to (98.2)AT2G43800| Symbols: | formin homology 2 domain-containing protein / FH2 domain-containing protein | chr	
EV110702	4.138	no similarity	
JCVI_331	4.135	highly similar to (503)AT1G09780| Symbols: | 2,3-biphosphoglycerate-independent phosphoglycerate mutase, putative / phosphoglyceron	
JCVI_16148	4.132	weakly similar to (140)AT1G02930| Symbols: GST1, ERD11, ATGSTF3, GSTF6, ATGSTF6 | ATGSTF6 (EARLY RESPONSIVE TO DI	
JCVI_36523	4.130	no original description	
AM390499	4.129	moderately similar to (246)AT3G22370| Symbols: ATAOX1A, AOX1A | AOX1A (alternative oxidase 1A); alternative oxidase | chr3:7906	
JCVI_35682	4.128	no original description	3.248
CV546988	4.123	very weakly similar to (80.5)AT4G32150| Symbols: VAMP711, ATVAMP711, VAMP7C | VAMP7C (VESICLE-ASSOCIATED MEMBR	
JCVI_19167	4.118	weakly similar to (182)AT5G28237| Symbols: | tryptophan synthase, beta subunit, putative | chr5:10207481-10213546 REVERSEweakly s	-5.942
JCVI_518	4.114	moderately similar to (208)AT5G13420| Symbols: | transaldolase, putative | chr5:4302083-4304215 REVERSE no original description	

JCVI_24058	4.114	nearly identical (1036)AT5G40390| Symbols: SIP1 | SIP1 (SEED IMBIBITION 1-LIKE); galactinol-sucrose galactosyltransferase/ hydrola	
EV176859	4.092	moderately similar to (308)AT5G63050| Symbols: EMB2759 | EMB2759 (EMBRYO DEFECTIVE 2759) | chr5:25310335-25312025 FOF	
JCVI_18573	4.084	moderately similar to (435)AT1G71880| Symbols: ATSUC1, SUC1 | SUC1 (SUCROSE-PROTON SYMPORTER 1); carbohydrate transm	
EE404313	4.073	no similarity	
JCVI_7134	4.072	moderately similar to (459)AT4G33010| Symbols: ATGLDP1 | ATGLDP1 (ARABIDOPSIS THALIANA GLYCINE DECARBOXYLASE	
JCVI_4417	4.069	moderately similar to (323)AT4G37880| Symbols: | protein binding / zinc ion binding | chr4:17810177-17811343 FORWARD no original	
JCVI_9149	4.067	moderately similar to (347)AT3G21550| Symbols: | similar to unknown protein [Arabidopsis thaliana] (TAIR:AT3G21520.1); similar to u	
JCVI_3927	4.042	moderately similar to (251)AT5G66140| Symbols: PAD2 | PAD2 (20S proteasome alpha subunit D2); peptidase | chr5:26454671-2645590	
EV203669	4.041	no similarity	
JCVI_2844	4.031	moderately similar to (430)AT2G04400| Symbols: | indole-3-glycerol phosphate synthase (IGPS) | chr2:1531205-1533575 FORWARD no	
JCVI_18736	4.029	moderately similar to (368)AT1G73010| Symbols: | phosphoric monoester hydrolase | chr1:27468441-27469841 REVERSE no original de	
JCVI_3497	4.023	no original description	
JCVI_4	4.014	moderately similar to (448)AT1G42970| Symbols: GAPB | GAPB (GLYCERALDEHYDE-3-PHOSPHATE DEHYDROGENASE B SUBU	
H07450	4.014	no similarity	
CX278439	4.014	weakly similar to (189)AT2G14095| Symbols: | unknown protein | chr2:5939588-5940834 REVERSE [16821]	
JCVI_4769	4.013	moderately similar to (365)AT1G35160| Symbols: GF14 PHI, GRF4 | GRF4 (GENERAL REGULATORY FACTOR 4); protein phosphory	
DY015167	4.011	moderately similar to (252)AT3G08010| Symbols: ATAB2 | ATAB2; RNA binding | chr3:2556052-2557432 FORWARD [18966]	1.070
JCVI_3318	4.010	moderately similar to (468)AT5G60390| Symbols: | elongation factor 1-alpha / EF-1-alpha | chr5:24306452-24307901 FORWARDmodera	
JCVI_20373	4.003	moderately similar to (255)AT1G80390| Symbols: IAA15 | IAA15 (indoleacetic acid-induced protein 15); transcription factor | chr1:30226	5.057
JCVI_4291	3.999	weakly similar to (150)AT4G17890| Symbols: AGD8 | AGD8 (ARF-GAP DOMAIN 8); DNA binding | chr4:9937134-9939001 FORWAR	-1.982
JCVI_13697	3.998	moderately similar to (436)AT2G03760| Symbols: RAR047, ST | ST (steroid sulfotransferase); sulfotransferase | chr2:1149472-1150452 R	
JCVI_25678	3.980	no original description	-2.429
DN965962	3.979	moderately similar to (265)AT1G74640| Symbols: | similar to unnamed protein product [Vitis vinifera] (GB:CAO64553.1); contains doma	
EV020179	3.978	no similarity	
DY014595	3.971	moderately similar to (241)AT3G02580| Symbols: DWF7, BUL1 | STE1 (STEROL 1); C-5 sterol desaturase | chr3:547055-548622 FORW	
ES909769	3.971	weakly similar to (101)AT1G69250| Symbols: | nuclear transport factor 2 (NTF2) family protein / RNA recognition motif (RRM)-containi	
JCVI_12930	3.966	highly similar to (756)AT4G37430| Symbols: CYP81F1, CYP91A2 | CYP91A2 (CYTOCHROME P450 MONOOXYGENASE 91A2); ox	
JCVI_28231	3.964	highly similar to (702)AT1G80460| Symbols: GLI1, NHO1 | NHO1 (NONHOST RESISTANCE TO P. S. PHASEOLICOLA 1); carbohydr	
EE476492	3.963	moderately similar to (232)AT1G10960| Symbols: ATFD1 | ATFD1 (FERREDOXIN 1); 2 iron, 2 sulfur cluster binding / electron carrier/ i	
L46441	3.962	no similarity	-3.210
JCVI_36520	3.961	weakly similar to (134)AT5G26340| Symbols: STP13, MSS1 | MSS1 (SUGAR TRANSPORT PROTEIN 13); carbohydrate transmembran	
JCVI_2610	3.953	moderately similar to (446)AT4G13930| Symbols: SHM4 | SHM4 (SERINE HYDROXYMETHYLTRANSFERASE 4); glycine hydroxym	
JCVI_38595	3.950	moderately similar to (236)AT5G57050| Symbols: ABI2 | ABI2 (ABA INSENSITIVE 2) | chr5:23104946-23106529 FORWARD no origir	
JCVI_12232	3.948	moderately similar to (322)AT5G47860| Symbols: | similar to unknown protein [Arabidopsis thaliana] (TAIR:AT3G43540.1); similar to u	
JCVI_2058	3.943	moderately similar to (454)AT5G13930| Symbols: CHS, TT4, ATCHS | ATCHS/CHS/TT4 (CHALCONE SYNTHASE); naringenin-chalc	
EX046595	3.938	no similarity	
JCVI_35980	3.937	moderately similar to (489)AT1G28230| Symbols: ATPUP1, PUP1 | PUP1 (PURINE PERMEASE 1); purine transmembrane transporter |	2.960
JCVI_1058	3.936	moderately similar to (374)AT5G30510| Symbols: ARRPS1, RPS1 | RPS1 (ribosomal protein S1); RNA binding | chr5:11636492-1163845	
EG019987	3.935	moderately similar to (248)AT5G15650| Symbols: RGP2 | RGP2 (REVERSIBLY GLYCOSYLATED POLYPEPTIDE 2); alpha-1,4-gluca	
JCVI_3086	3.935	moderately similar to (308)AT5G40670| Symbols: | PQ-loop repeat family protein / transmembrane family protein | chr5:16303196-16304	
JCVI_5955	3.933	moderately similar to (299)AT5G33370| Symbols: | GDSL-motif lipase/hydrolase family protein | chr5:12620094-12621900 REVERSEve	
JCVI_1191	3.925	moderately similar to (216)AT3G24290| Symbols: | ammonium transporter, putative | chr3:8801407-8802897 REVERSE no original descr	
EV017317	3.924	weakly similar to (116)AT2G34260| Symbols: | transducin family protein / WD-40 repeat family protein | chr2:14473416-14475495 FORV	
H07761	3.923	no similarity	
DY029056	3.919	weakly similar to (194)AT3G09035| Symbols: | legume lectin family protein | chr3:2759072-2760088 FORWARD [18978]	
EX041283	3.917	weakly similar to (174)AT3G12520| Symbols: SULTR4;2 | SULTR4;2 (sulfate transporter 4;2); sulfate transmembrane transporter | chr3:3	
JCVI_22761	3.915	moderately similar to (424)AT4G13510| Symbols: ATAMT1, ATAMT1;1, AMT1;1 | AMT1;1 (AMMONIUM TRANSPORT 1); ammoni	
JCVI_16018	3.913	moderately similar to (416)AT3G56200| Symbols: | amino acid transporter family protein | chr3:20861066-20862758 FORWARD no orig	
JCVI_18544	3.910	weakly similar to (108)AT3G23490| Symbols: CYN | CYN (CYANASE); cyanate hydratase | chr3:8423245-8424422 REVERSEvery weak	
JCVI_6563	3.909	moderately similar to (371)AT1G74020| Symbols: SS2 | SS2 (STRICTOSIDINE SYNTHASE 2); strictosidine synthase | chr1:27838950-2	-3.034
CD828777	3.904	weakly similar to (181)AT5G65020| Symbols: ANNAT2 | ANNAT2 (ANNEXIN ARABIDOPSIS 2); calcium ion binding / calcium-depen	
JCVI_33803	3.903	moderately similar to (332)AT2G15560| Symbols: | similar to unknown protein [Arabidopsis thaliana] (TAIR:AT3G62200.1); similar to E	
JCVI_9956	3.901	moderately similar to (375)AT5G39520| Symbols: | similar to unknown protein [Arabidopsis thaliana] (TAIR:AT5G39530.1); similar to u	-2.289
ES902026	3.895	moderately similar to (239)AT1G20850| Symbols: XCP2 | XCP2 (XYLEM CYSTEINE PEPTIDASE 2); cysteine-type peptidase/ peptidas	
JCVI_42035	3.893	moderately similar to (271)AT1G73260| Symbols: | trypsin and protease inhibitor family protein / Kunitz family protein | chr1:27551071-2	
EX129599	3.887	moderately similar to (248)AT5G48380| Symbols: | leucine-rich repeat family protein / protein kinase family protein | chr5:19621810-1962	
JCVI_16186	3.886	very weakly similar to (94.4)AT2G29650| Symbols: | inorganic phosphate transporter, putative | chr2:12680762-12682799 REVERSE no o	-3.819
JCVI_2785	3.885	moderately similar to (361)AT5G47860| Symbols: | similar to unknown protein [Arabidopsis thaliana] (TAIR:AT3G43540.1); similar to u	
EX123908	3.884	moderately similar to (249)AT1G25340| Symbols: AtMYB116, MYB116 | MYB116 (myb domain protein 116); DNA binding / transcripti	
JCVI_7995	3.882	moderately similar to (393)AT3G60140| Symbols: SRG2, DIN2 | DIN2 (DARK INDUCIBLE 2); hydrolase, hydrolyzing O-glycosyl comp	
JCVI_24300	3.881	very weakly similar to (96.3)AT5G54770| Symbols: TZ, THI1 | THI1 (THIAZOLE REQUIRING) | chr5:22263860-22265117 FORWARD	
EE445766	3.880	weakly similar to (130)AT3G56580| Symbols: | zinc finger (C3HC4-type RING finger) family protein | chr3:20973542-20974504 FORWA	
JCVI_6183	3.879	moderately similar to (364)AT4G27070| Symbols: TSB2 | TSB2 (TRYPTOPHAN SYNTHASE BETA-SUBUNIT); tryptophan synthase |	
JCVI_39752	3.872	weakly similar to (194)AT1G05210| Symbols: | similar to unknown protein [Arabidopsis thaliana] (TAIR:AT2G32380.1); similar to unnar	
JCVI_11413	3.868	moderately similar to (438)AT1G67300| Symbols: | hexose transporter, putative | chr1:25197495-25200414 REVERSEvery weakly simila	
JCVI_13134	3.868	moderately similar to (451)AT1G23730| Symbols: | carbonic anhydrase, putative / carbonate dehydratase, putative | chr1:8395954-839800	
JCVI_2629	3.867	moderately similar to (299)AT5G15800| Symbols: AGL2, SEP1 | SEP1 (SEPALLATA1) | chr5:5151597-5153770 REVERSEmoderately s	
JCVI_32286	3.867	moderately similar to (254)AT3G28730| Symbols: SSRP1, NFD, ATHMG | ATHMG (HIGH MOBILITY GROUP); transcription factor |	
EE504385	3.865	very weakly similar to (87.0)AT4G35350| Symbols: XCP1 | XCP1 (XYLEM CYSTEINE PEPTIDASE 1); cysteine-type peptidase | chr4:1	1.722
JCVI_25964	3.857	moderately similar to (354)AT1G78820| Symbols: | curculin-like (mannose-binding) lectin family protein / PAN domain-containing protei	
JCVI_26965	3.857	highly similar to (851)AT2G41190| Symbols: | amino acid transporter family protein | chr2:17174639-17177223 REVERSE no original de	
EX121951	3.854	moderately similar to (234)AT2G33030| Symbols: | leucine-rich repeat family protein | chr2:14024761-14025417 REVERSE [21829] 19 6	-1.519
JCVI_16491	3.853	moderately similar to (367)AT3G08860| Symbols: | alanine--glyoxylate aminotransferase, putative / beta-alanine-pyruvate aminotransferas	
JCVI_18354	3.853	moderately similar to (254)AT2G38940| Symbols: PHT1;4, ATPT2 | ATPT2 (PHOSPHATE TRANSPORTER 2); carbohydrate transmem	
EV204583	3.849	moderately similar to (229)AT1G07430| Symbols: | protein phosphatase 2C, putative / PP2C, putative | chr1:2281148-2282653 REVERSE	
JCVI_39726	3.849	weakly similar to (135)AT1G72290| Symbols: | trypsin and protease inhibitor family protein / Kunitz family protein | chr1:27219514-2722	
CV545028	3.848	moderately similar to (300)AT2G43580| Symbols: | chitinase, putative | chr2:18085894-18087090 REVERSEmoderately similar to (285)	
CN737494	3.840	no similarity	-2.301
EE434787	3.839	weakly similar to (143)AT4G17750| Symbols: HSFA1A, ATHSF1, ATHSFA1A, HSF1 | HSF1 (ARABIDOPSIS HEAT SHOCK FACTOR	
JCVI_19403	3.838	very weakly similar to (93.2)AT5G24660| Symbols: | similar to unknown protein [Arabidopsis thaliana] (TAIR:AT5G24655.1); similar to	
JCVI_14449	3.833	moderately similar to (337)AT4G23690| Symbols: | disease resistance-responsive family protein / dirigent family protein | chr4:12339162-	

JCVI_10518	3.833	moderately similar to (217)AT2G04240| Symbols: XERICO | XERICO; protein binding / zinc ion binding | chr2:1461813-1462301 REVE	
ES978129	3.826	very weakly similar to (91.3)AT5G63350| Symbols: | similar to unknown protein [Arabidopsis thaliana] (TAIR:AT3G48510.1); similar to	
JCVI_38093	3.818	no original description	
JCVI_18136	3.816	moderately similar to (409)AT5G49360| Symbols: ATBXL1, BXL1 | BXL1 (BETA-XYLOSIDASE 1); hydrolase, hydrolyzing O-glycosyl	
EE565736	3.814	no similarity	
EE546229	3.808	weakly similar to (117)AT5G38430| Symbols: | ribulose bisphosphate carboxylase small chain 1B / RuBisCO small subunit 1B (RBCS-1E	
JCVI_34646	3.803	moderately similar to (213)AT3G30390| Symbols: | amino acid transporter family protein | chr3:11979591-11981306 REVERSE no origin	
EV128620	3.797	moderately similar to (344)AT5G62790| Symbols: PDE129, DXR | DXR (1-DEOXY-D-XYLULOSE 5-PHOSPHATE REDUCTOISOME	
JCVI_34245	3.795	moderately similar to (281)AT1G07240| Symbols: | UDP-glucoronosyl/UDP-glucosyl transferase family protein | chr1:2223888-2225330	
EE546866	3.794	moderately similar to (219)AT1G69530| Symbols: EXP1, AT-EXP1, ATEXP1, ATHEXP ALPHA 1.2, ATEXPA1 | ATEXPA1 (ARABID	
EV205380	3.794	moderately similar to (244)AT1G34190| Symbols: ANAC017 | ANAC017 (Arabidopsis NAC domain containing protein 17); transcription	
JCVI_36140	3.787	moderately similar to (423)AT1G22300| Symbols: GF14 EPSILON, GRF10 | GRF10 (GENERAL REGULATORY FACTOR 10); protein	
JCVI_613	3.786	moderately similar to (434)AT3G22890| Symbols: APS1 | APS1 (ATP sulfurylase 3) | chr3:8112844-8114741 FORWARD no original des	-2.354
JCVI_4733	3.783	moderately similar to (325)AT4G30950| Symbols: FADC, SFD4, FAD6 | FAD6 (FATTY ACID DESATURASE 6); omega-6 fatty acid de	
JCVI_30154	3.779	moderately similar to (328)AT5G42480| Symbols: ARC6 | ARC6 (ACCUMULATION AND REPLICATION OF CHLOROPLASTS 6) | c	
JCVI_15204	3.777	moderately similar to (413)AT5G64250| Symbols: | 2-nitropropane dioxygenase family / NPD family | chr5:25714849-25716171 REVERS	
EV167917	3.776	no similarity	
JCVI_190	3.775	moderately similar to (475)AT5G61410| Symbols: EMB2728, RPE | RPE (EMBRYO DEFECTIVE 2728); ribulose-phosphate 3-epimeras	
JCVI_4345	3.774	moderately similar to (403)AT3G15500| Symbols: ANAC055, ATNAC3 | ATNAC3 (ARABIDOPSIS NAC DOMAIN CONTAINING PR	
JCVI_39110	3.774	no original description	
JCVI_8459	3.773	moderately similar to (357)AT1G74590| Symbols: ATGSTU10 | ATGSTU10 (Arabidopsis thaliana Glutathione S-transferase (class tau) 1	
JCVI_923	3.772	moderately similar to (420)AT4G04770| Symbols: LAF6, ATNAP1, ATABC1 | ATABC1 (ARABIDOPSIS THALIANA NUCLEOSOME	-3.747
JCVI_26342	3.760	moderately similar to (475)AT4G24780| Symbols: | pectate lyase family protein | chr4:12770641-12772237 REVERSEmoderately similar	
JCVI_3882	3.753	moderately similar to (431)AT1G77450| Symbols: ANAC032 | ANAC032 (Arabidopsis NAC domain containing protein 32); transcription	
AM395252	3.751	moderately similar to (238)AT2G43590| Symbols: | chitinase, putative | chr2:18088669-18089826 REVERSEmoderately similar to (249)	
JCVI_14516	3.745	weakly similar to (170)AT1G19540| Symbols: | isoflavone reductase, putative | chr1:6765704-6767238 FORWARDweakly similar to (12	
ES942221	3.745	moderately similar to (365)AT1G52780| Symbols: | similar to unknown protein [Arabidopsis thaliana] (TAIR:AT4G21700.1); similar to h	
DY021932	3.745	weakly similar to (150)AT2G34340| Symbols: | similar to unknown protein [Arabidopsis thaliana] (TAIR:AT1G29640.1); similar to unkn	
JCVI_856	3.742	moderately similar to (441)AT4G39660| Symbols: AGT2 | AGT2 (ALANINE:GLYOXYLATE AMINOTRANSFERASE 2); alanine-glyo	
CD814040	3.740	moderately similar to (211)AT4G37130| Symbols: | hydroxyproline-rich glycoprotein family protein | chr4:17489561-17491767 FORWA	
JCVI_4833	3.739	weakly similar to (149)AT5G12470| Symbols: | similar to unknown protein [Arabidopsis thaliana] (TAIR:AT2G40400.2); similar to unkn	
EV221263	3.733	weakly similar to (159)AT2G45960| Symbols: TMP-A, ATHH2, PIP1;2, PIP1B | PIP1B (plasma membrane intrinsic protein 1;2) | chr2:18	
AM062464	3.723	moderately similar to (223)AT3G13120| Symbols: | 30S ribosomal protein S10, chloroplast, putative | chr3:4220317-4221533 REVERSE	
EV100379	3.721	moderately similar to (432)AT4G38180| Symbols: FRS5 | FRS5 (FAR1-RELATED SEQUENCE 5); zinc ion binding | chr4:17906696-179	
JCVI_34233	3.719	moderately similar to (207)AT5G52300| Symbols: RD29B, LTI65 | LTI65/RD29B (RESPONSIVE TO DESSICATION 29B) | chr5:21254	
EH413893	3.718	no similarity	
JCVI_16999	3.717	moderately similar to (370)AT3G55800| Symbols: SBPASE | SBPASE (SEDOHEPTULOSE-BISPHOSPHATASE); phosphoric ester hyd	
JCVI_29134	3.714	moderately similar to (213)AT1G09500| Symbols: | cinnamyl-alcohol dehydrogenase family / CAD family | chr1:3066813-3068246 FORV	
JCVI_6340	3.713	moderately similar to (329)AT2G22480| Symbols: | phosphofructokinase family protein | chr2:9552750-9555494 FORWARD no original	-1.657
EV042145	3.713	weakly similar to (195)AT1G34130| Symbols: STT3B | STT3B (STAUROSPORIN AND TEMPERATURE SENSITIVE 3-LIKE B); olig	
JCVI_10129	3.712	moderately similar to (315)AT3G18830| Symbols: ATPLT5 | ATPLT5 (POLYOL TRANSPORTER 5); D-ribose transmembrane transport	
EX025411	3.711	weakly similar to (190)AT1G59700| Symbols: ATGSTU16 | ATGSTU16 (Arabidopsis thaliana Glutathione S-transferase (class tau) 16); g	
JCVI_2135	3.706	moderately similar to (394)AT3G18490| Symbols: | aspartyl protease family protein | chr3:6349096-6350598 REVERSE no original descr	
JCVI_2841	3.706	moderately similar to (373)AT1G69530| Symbols: EXP1, AT-EXP1, ATEXP1, ATHEXP ALPHA 1.2, ATEXPA1 | ATEXPA1 (ARABID	
JCVI_12378	3.704	very weakly similar to (100)AT1G12900| Symbols: GAPA-2 | GAPA-2 | chr1:4392632-4393848 REVERSEvery weakly similar to (100)G	
JCVI_26367	3.701	moderately similar to (340)AT4G17740| Symbols: | C-terminal processing protease, putative | chr4:9867101-9869732 REVERSE no origi	
JCVI_40019	3.695	no original description	
EE422930	3.694	very weakly similar to (99.8)AT5G19750| Symbols: | peroxisomal membrane 22 kDa family protein | chr5:6677126-6679129 FORWARD	
JCVI_39761	3.691	weakly similar to (121)AT1G78560| Symbols: | bile acid:sodium symporter family protein | chr1:29551739-29553657 REVERSE no origi	
JCVI_14406	3.691	no original description	
CX191896	3.687	no similarity	
EV018798	3.687	weakly similar to (109)AT5G27430| Symbols: | signal peptidase subunit family protein | chr5:9687473-9689188 FORWARD [21441]	
JCVI_26115	3.685	moderately similar to (230)AT2G32840| Symbols: | proline-rich family protein | chr2:13940505-13941913 REVERSE no original descript	
DN961666	3.680	weakly similar to (140)AT2G43590| Symbols: | chitinase, putative | chr2:18088669-18089826 REVERSEweakly similar to (128)CHI4_B	
L35790	3.678	very weakly similar to (82.4)AT1G63970| Symbols: MECPS, ISPF | ISPF (Homolog of E. coli ispF (isoprenoids F)); 2-C-methyl-D-erythrit	
EV215619	3.678	weakly similar to (147)AT2G43020| Symbols: ATPAO2 | ATPAO2 (POLYAMINE OXIDASE 2); amine oxidase | chr2:17899022-179015	
JCVI_12511	3.677	moderately similar to (246)AT1G63840| Symbols: | zinc finger (C3HC4-type RING finger) family protein | chr1:23693654-23694154 RE	
EV025291	3.675	weakly similar to (157)AT4G31530| Symbols: | binding / catalytic/ coenzyme binding | chr4:15282287-15284070 FORWARD [21441]	
JCVI_9289	3.672	highly similar to (536)AT5G60600| Symbols: ISPG, CSB3, CLB4, GcpE | GcpE (CHLOROPLAST BIOGENESIS 4) | chr5:24376673-243	
EV051898	3.672	moderately similar to (306)AT4G36640| Symbols: | SEC14 cytosolic factor family protein / phosphoglyceride transfer family protein | chr	
CD831726	3.669	moderately similar to (460)AT2G41770| Symbols: | similar to unknown protein [Arabidopsis thaliana] (TAIR:AT3G57420.1); similar to u	
JCVI_3392	3.667	moderately similar to (442)AT3G12780| Symbols: PGK1 | PGK1 (PHOSPHOGLYCERATE KINASE 1); phosphoglycerate kinase | chr3:	
JCVI_9984	3.666	moderately similar to (325)AT5G63570| Symbols: GSA1 | GSA1 (GLUTAMATE-1-SEMIALDEHYDE-2,1-AMINOMUTASE); glutamat	
JCVI_1016	3.664	moderately similar to (482)AT4G34050| Symbols: | caffeoyl-CoA 3-O-methyltransferase, putative | chr4:16310849-16311978 FORWARD	
JCVI_1839	3.664	moderately similar to (286)AT5G12860| Symbols: DIT1 | DIT1 (DICARBOXYLATE TRANSPORTER 1) | chr5:4059853-4061922 REV	
EV034631	3.661	no similarity	
JCVI_26352	3.660	moderately similar to (332)AT3G60240| Symbols: CUM2, EIF4G | EIF4G (EUKARYOTIC TRANSLATION INITIATION FACTOR 4G	-1.548
JCVI_2726	3.657	moderately similar to (318)AT3G04090| Symbols: SIP1;1, SIP1A | SIP1;1 (SMALL AND BASIC INTRINSIC PROTEIN 1A) | chr3:1072	
EG020206	3.653	moderately similar to (256)AT3G21550| Symbols: | similar to unknown protein [Arabidopsis thaliana] (TAIR:AT3G21520.1); similar to u	
JCVI_38803	3.652	moderately similar to (265)AT3G58620| Symbols: TTL4 | TTL4 (TETRATRICOPETIDE-REPEAT THIOREDOXIN-LIKE 4); binding |	
JCVI_37003	3.643	weakly similar to (198)AT3G56200| Symbols: | amino acid transporter family protein | chr3:20861066-20862758 FORWARD no original	
JCVI_7831	3.641	highly similar to (523)AT4G38790| Symbols: | ER lumen protein retaining receptor family protein | chr4:18111420-18112971 FORWARD	
EV145485	3.641	weakly similar to (139)AT5G52550| Symbols: | similar to unknown protein [Arabidopsis thaliana] (TAIR:AT4G25670.1); similar to hypo	
JCVI_23309	3.640	moderately similar to (368)AT4G24040| Symbols: ATTRE1, TRE1 | ATTRE1/TRE1 (TREHALASE 1); alpha,alpha-trehalase/ trehalase |	
EX099357	3.636	moderately similar to (317)AT3G13672| Symbols: | seven in absentia (SINA) family protein | chr3:4473548-4474350 REVERSE [21825]	
EV194315	3.634	weakly similar to (120)AT3G11930| Symbols: | universal stress protein (USP) family protein | chr3:3776377-3777399 FORWARD [2148	
JCVI_35906	3.629	moderately similar to (324)AT3G02230| Symbols: ATRGP1, ATRGP, RGP1 | RGP1 (REVERSIBLY GLYCOSYLATED POLYPEPTIDE	
EX094331	3.623	no similarity	
EV012188	3.622	no similarity	-5.852
JCVI_13216	3.622	highly similar to (868)AT2G37040| Symbols: PAL1 | PAL1 (PHE AMMONIA LYASE 1); phenylalanine ammonia-lyase | chr2:15564681-	1.146
JCVI_322	3.621	highly similar to (879)AT1G20620| Symbols: SEN2, CAT3 | CAT3 (CATALASE 3); catalase | chr1:7143132-7146183 FORWARDhighly	

DY016454	3.621	weakly similar to (176)AT5G28237| Symbols: | tryptophan synthase, beta subunit, putative | chr5:10207481-10213546 REVERSEweakly s	
JCVI_5140	3.621	moderately similar to (426)AT5G61820| Symbols: | similar to MtN19-like protein [Pisum sativum] (GB:AAU14999.2); contains InterPro d	
EV226213	3.620	weakly similar to (176)AT1G19580| Symbols: GAMMA CA1 | GAMMA CA1 (GAMMA CARBONIC ANHYDRASE 1); carbonate dehyd	
DY029651	3.618	weakly similar to (199)AT1G79900| Symbols: ATMBAC2, BAC2 | ATMBAC2/BAC2 (Arabidopsis mitochondrial basic amino acid carrie	
JCVI_35737	3.617	weakly similar to (110)AT1G68552| Symbols: CPuORF53 | CPuORF53 (Conserved peptide upstream open reading frame 53) | chr1:25730	-1.755
JCVI_19823	3.617	highly similar to (608)AT3G16150| Symbols: | L-asparaginase, putative / L-asparagine amidohydrolase, putative | chr3:5471800-5473039	
JCVI_2499	3.616	moderately similar to (338)AT2G32080| Symbols: PUR ALPHA-1 | PUR ALPHA-1 (purin-rich alpha 1); nucleic acid binding | chr2:13649	
JCVI_1514	3.615	moderately similar to (479)AT2G36880| Symbols: MAT3 | MAT3 (METHIONINE ADENOSYLTRANSFERASE 3) | chr2:15486800-154	
JCVI_40711	3.614	weakly similar to (119)AT3G48990| Symbols: | AMP-dependent synthetase and ligase family protein | chr3:18170016-18172279 REVERS	-3.266
EE438761	3.614	weakly similar to (192)AT2G37180| Symbols: PIP2;3, PIP2C, RD28 | RD28 (plasma membrane intrinsic protein 2;3); water channel | chr2	
JCVI_26587	3.610	weakly similar to (157)AT3G15760| Symbols: | similar to unknown protein [Arabidopsis thaliana] (TAIR:AT1G52565.1); similar to unnam	
DN962854	3.606	moderately similar to (306)AT5G67360| Symbols: ARA12 | ARA12; subtilase | chr5:26889418-26891691 REVERSE [17359]	
JCVI_7146	3.606	highly similar to (592)AT3G12780| Symbols: PGK1 | PGK1 (PHOSPHOGLYCERATE KINASE 1); phosphoglycerate kinase | chr3:40611	-3.347
JCVI_27049	3.605	moderately similar to (261)AT1G32080| Symbols: | membrane protein, putative | chr1:11537552-11539736 REVERSE no original descrip	
JCVI_24041	3.603	weakly similar to (160)AT4G33480| Symbols: | similar to unknown [Picea sitchensis] (GB:ABK24826.1); similar to Os04g0692200 [Oryz	
JCVI_8333	3.603	moderately similar to (394)AT5G58310| Symbols: | hydrolase, alpha/beta fold family protein | chr5:23592353-23593233 REVERSEweakl	
JCVI_40921	3.602	weakly similar to (126)AT3G25870| Symbols: | similar to unknown protein [Arabidopsis thaliana] (TAIR:AT1G13360.1); similar to unkno	
EE435156	3.600	very weakly similar to (98.2)AT2G21250| Symbols: | mannose 6-phosphate reductase (NADPH-dependent), putative | chr2:9110489-91121	
JCVI_4160	3.600	moderately similar to (348)AT5G54160| Symbols: OMT1, ATOMT1 | ATOMT1 (O-METHYLTRANSFERASE 1) | chr5:21999301-22001	
JCVI_19235	3.600	moderately similar to (217)AT3G22370| Symbols: ATAOX1A, AOX1A | AOX1A (alternative oxidase 1A); alternative oxidase | chr3:7906	
JCVI_22843	3.600	weakly similar to (126)AT2G45960| Symbols: TMP-A, ATHH2, PIP1;2, PIP1B | PIP1B (plasma membrane intrinsic protein 1;2) | chr2:18	
JCVI_7398	3.598	highly similar to (576)AT5G57020| Symbols: ATNMT1, NMT1 | NMT1 (N-MYRISTOYLTRANSFERASE 1) | chr5:23092677-23093981	-2.965
JCVI_32783	3.597	moderately similar to (327)AT4G26080| Symbols: ABI1 | ABI1 (ABA INSENSITIVE 1); calcium ion binding / protein serine/threonine ph	-1.951
JCVI_28241	3.596	moderately similar to (329)AT1G01710| Symbols: | acyl-CoA thioesterase family protein | chr1:262950-266029 FORWARD no original de	
JCVI_32452	3.594	highly similar to (514)AT5G65020| Symbols: ANNAT2 | ANNAT2 (ANNEXIN ARABIDOPSIS 2); calcium ion binding / calcium-depend	
AT000471	3.590	no similarity	
JCVI_11279	3.589	moderately similar to (262)AT1G04350| Symbols: | 2-oxoglutarate-dependent dioxygenase, putative | chr1:1165295-1166537 FORWARD	
JCVI_1620	3.589	moderately similar to (305)AT3G52880| Symbols: ATMDAR1 | ATMDAR1 (MONODEHYDROASCORBATE REDUCTASE 1); monod	
EX022159	3.585	weakly similar to (131)AT4G00490| Symbols: BMY9, BAM2 | BAM2/BMY9 (BETA-AMYLASE 2); beta-amylase | chr4:222422-224862	
JCVI_15488	3.585	very weakly similar to (99.8)AT1G55210| Symbols: | disease resistance response | chr1:20601724-20602287 REVERSE no original descrip	
EE544606	3.580	weakly similar to (169)AT1G18600| Symbols: | rhomboid family protein | chr1:6400639-6402579 FORWARD [20124]	
EE562600	3.580	weakly similar to (153)AT3G54670| Symbols: SMC1, ATSMC1, TTN8 | TTN8 (TITAN8); ATP binding | chr3:20251406-20254679 FORW	
JCVI_1513	3.579	moderately similar to (401)AT5G14040| Symbols: | mitochondrial phosphate transporter | chr5:4531061-4532967 REVERSE no original d	
JCVI_9825	3.578	no original description	
ES928478	3.575	weakly similar to (187)AT1G68260| Symbols: | thioesterase family protein | chr1:25589639-25591264 REVERSE [15713]	
JCVI_27602	3.575	moderately similar to (261)AT1G05300| Symbols: ZIP5 | ZIP5 (ZINC TRANSPORTER 5 PRECURSOR); cation transmembrane transpor	-2.097
JCVI_35271	3.572	weakly similar to (118)AT5G05560| Symbols: EMB2771, APC1 | APC1 (EMBRYO DEFECTIVE 2771); ubiquitin-protein ligase | chr5:16	
JCVI_14831	3.570	moderately similar to (333)AT4G08520| Symbols: | clathrin adaptor complex small chain family protein | chr4:5417884-5420292 FORWA	
JCVI_2485	3.569	moderately similar to (499)AT3G17390| Symbols: SAMS3, MAT4, MTO3 | MTO3 (S-adenosylmethionine synthase 3); methionine adeno	
JCVI_390	3.563	moderately similar to (389)AT4G23430| Symbols: | short-chain dehydrogenase/reductase (SDR) family protein | chr4:12229181-12231503	
JCVI_34985	3.562	moderately similar to (222)AT1G80510| Symbols: | amino acid transporter family protein | chr1:30277992-30279461 FORWARD no orig	
EX140016	3.559	no similarity	
JCVI_13194	3.558	weakly similar to (110)AT5G23750| Symbols: | remorin family protein | chr5:8010007-8011456 REVERSE no original description	
JCVI_18727	3.558	moderately similar to (443)AT1G76130| Symbols: ATAMY2, AMY2 | AMY2/ATAMY2 (ALPHA-AMYLASE-LIKE 2); alpha-amylase | c	
JCVI_28577	3.556	moderately similar to (350)AT3G61890| Symbols: ATHB12, ATHB-12 | ATHB-12 (ARABIDOPSIS THALIANA HOMEOBOX PROTEI	
EV108754	3.553	no similarity	
JCVI_5412	3.553	moderately similar to (387)AT4G38370| Symbols: | phosphoglycerate/bisphosphoglycerate mutase family protein | chr4:17970106-179712	-1.748
JCVI_2864	3.551	moderately similar to (320)AT4G38460| Symbols: GGR | GGR (GERANYLGERANYL REDUCTASE); farnesyltranstransferase | chr4:17	1.788
EV185660	3.548	weakly similar to (104)TRXH_BRAOL [21488] 32 371 730	-3.550
ES265763	3.548	moderately similar to (301)AT1G80510| Symbols: | amino acid transporter family protein | chr1:30277992-30279461 FORWARD [21031]	
JCVI_4356	3.544	moderately similar to (467)AT3G24190| Symbols: | ABC1 family protein | chr3:8743326-8747710 FORWARD no original description	
DW997085	3.540	moderately similar to (352)AT5G24420| Symbols: | glucosamine/galactosamine-6-phosphate isomerase-related | chr5:8336946-8337882 R	1.740
JCVI_8178	3.539	weakly similar to (105)AT3G44840| Symbols: | S-adenosyl-L-methionine:carboxyl methyltransferase family protein | chr3:16383484-1638	
JCVI_15259	3.538	moderately similar to (404)AT2G30360| Symbols: SIP4, SNRK3.22, PKS5, CIPK11 | CIPK11 (SOS3-INTERACTING PROTEIN 4); kina	
JCVI_36229	3.537	highly similar to (524)AT4G31390| Symbols: | ABC1 family protein | chr4:15233132-15236770 FORWARD no original description	
JCVI_2869	3.536	moderately similar to (305)AT1G12550| Symbols: | oxidoreductase family protein | chr1:4274647-4275829 FORWARD no original descri	
EV216858	3.534	no similarity	
JCVI_25744	3.533	highly similar to (606)AT3G23810| Symbols: SAHH2 | SAHH2 (S-ADENOSYL-L-HOMOCYSTEINE (SAH) HYDROLASE 2); adenosyl	
JCVI_9166	3.530	weakly similar to (143)AT3G02540| Symbols: RAD23-3 | RAD23-3 (PUTATIVE DNA REPAIR PROTEIN RAD23-3) | chr3:533913-536	
JCVI_38843	3.529	weakly similar to (162)AT5G62150| Symbols: | peptidoglycan-binding LysM domain-containing protein | chr5:24975551-24975859 FORW	
EX042516	3.529	weakly similar to (122)AT4G01900| Symbols: P11, GLB1 | GLB1 (glutamine synthetase B1) | chr4:821736-823294 FORWARD [21811]	
RC_CB617683	3.528	no similarity	-1.379
JCVI_27514	3.527	moderately similar to (327)AT1G34130| Symbols: STT3B | STT3B (STAUROSPORIN AND TEMPERATURE SENSITIVE 3-LIKE B); c	
JCVI_39957	3.527	moderately similar to (233)AT1G53550| Symbols: | F-box family protein | chr1:19987079-19988305 FORWARD no original description	
JCVI_2565	3.526	moderately similar to (333)AT4G27080| Symbols: ATPDIL5-4 | ATPDIL5-4 (PDI-LIKE 5-4) | chr4:13589162-13593341 FORWARD no o	-1.351
JCVI_1756	3.526	highly similar to (531)AT2G36880| Symbols: MAT3 | MAT3 (METHIONINE ADENOSYLTRANSFERASE 3) | chr2:15486800-1548797	
EV118552	3.524	moderately similar to (396)AT4G14030| Symbols: SBP1 | selenium-binding protein, putative | chr4:8098116-8100160 REVERSE [21479]	
EV215033	3.523	no similarity	-2.120
JCVI_30132	3.522	moderately similar to (364)AT4G00970| Symbols: | protein kinase family protein | chr4:418437-421694 FORWARD no original descriptio	-2.876
DY023394	3.521	very weakly similar to (99.0)AT4G39860| Symbols: | similar to unknown protein [Arabidopsis thaliana] (TAIR:AT2G22270.1); similar to u	
JCVI_3663	3.521	moderately similar to (379)AT1G49970| Symbols: NCLPP5, CLPR1 | CLPR1 (Clp protease proteolytic subunit 5); endopeptidase Clp | chr	
JCVI_41709	3.518	weakly similar to (180)AT2G01150| Symbols: RHA2B | RHA2B (RING-H2 FINGER PROTEIN 2B); protein binding / zinc ion binding | c	
ES997334	3.518	weakly similar to (136)AT5G65020| Symbols: ANNAT2 | ANNAT2 (ANNEXIN ARABIDOPSIS 2); calcium ion binding / calcium-depen	
JCVI_39275	3.517	moderately similar to (290)AT3G14070| Symbols: CAX9 | CAX9 (CATION EXCHANGER 9); cation:cation antiporter | chr3:4661150-46	
JCVI_35692	3.516	moderately similar to (260)AT1G67600| Symbols: | similar to catalytic [Arabidopsis thaliana] (TAIR:AT1G24350.1); similar to unknown	
JCVI_19390	3.516	weakly similar to (169)AT2G01110| Symbols: UNE3, PGA2, TATC, APG2 | APG2 (ALBINO AND PALE GREEN 2) | chr2:83786-85088	
JCVI_26334	3.515	highly similar to (535)AT1G75280| Symbols: | isoflavone reductase, putative | chr1:28255691-28257016 FORWARDmoderately similar to	
JCVI_16755	3.513	moderately similar to (334)AT1G34190| Symbols: ANAC017 | ANAC017 (Arabidopsis NAC domain containing protein 17); transcription	
JCVI_13	3.512	highly similar to (530)AT1G12900| Symbols: GAPA-2 | GAPA-2 | chr1:4392632-4393848 REVERSEmoderately similar to (468)G3PA_P	
JCVI_1924	3.510	moderately similar to (288)AT3G02360| Symbols: | 6-phosphogluconate dehydrogenase family protein | chr3:482505-483965 FORWARD	
JCVI_20331	3.509	moderately similar to (436)AT4G18810| Symbols: | binding / catalytic/ transcription repressor | chr4:10322633-10325746 REVERSE no o	

JCVI_2139	3.507	moderately similar to (385)AT5G23660| Symbols: MTN3 | MTN3 (ARABIDOPSIS HOMOLOG OF MEDICAGO TRUNCATULA MTN	
CV433051	3.505	weakly similar to (153)AT1G16470| Symbols: PAB1 | PAB1 (PROTEASOME SUBUNIT PAB1); peptidase | chr1:5623116-5625433 FOR	
AM385165	3.505	very weakly similar to (84.7)AT1G08630| Symbols: THA1 | THA1 (THREONINE ALDOLASE 1) | chr1:2743951-2745688 REVERSE [20	
JCVI_4419	3.504	moderately similar to (345)AT1G15140| Symbols: | oxidoreductase NAD-binding domain-containing protein | chr1:5210637-5212132 RE	
JCVI_5131	3.502	moderately similar to (391)AT5G48880| Symbols: PKT1, KAT5, PKT2 | KAT5/PKT1/PKT2 (PEROXISOMAL 3-KETO-ACYL-COA TH	
EL592315	3.502	no similarity	
JCVI_6754	3.502	moderately similar to (387)AT1G29150| Symbols: RPN6, ATS9 | ATS9 (19S PROTEOSOME SUBUNIT 9) | chr1:10181226-10182485 F	
JCVI_16867	3.500	weakly similar to (175)AT5G13440| Symbols: | ubiquinol-cytochrome C reductase iron-sulfur subunit, mitochondrial, putative / Rieske iro	
CX195042	3.500	moderately similar to (304)AT4G17830| Symbols: | peptidase M20/M25/M40 family protein | chr4:9915929-9918062 FORWARD [16807	
JCVI_30885	3.498	moderately similar to (443)AT1G11860| Symbols: | aminomethyltransferase, putative | chr1:4001800-4003244 FORWARDmoderately sim	
EX053499	3.498	moderately similar to (204)AT4G37110| Symbols: | protein binding / zinc ion binding | chr4:17484337-17486191 REVERSE [21812]	
JCVI_14197	3.496	moderately similar to (261)AT2G18240| Symbols: | RER1 protein, putative | chr2:7942548-7943546 FORWARD no original description	
JCVI_38072	3.494	highly similar to (625)AT1G71040| Symbols: LPR2 | LPR2 (LOW PHOSPHATE ROOT2); copper ion binding | chr1:26800863-26803886	
JCVI_1144	3.494	moderately similar to (332)AT3G01440| Symbols: | oxygen evolving enhancer 3 (PsbQ) family protein | chr3:168485-169414 FORWARD	
EV164073	3.494	no similarity	-1.949
EE441781	3.493	weakly similar to (176)AT4G30930| Symbols: NFD1 | NFD1 (NUCLEAR FUSION DEFECTIVE 1); structural constituent of ribosome | c	1.507
JCVI_3850	3.491	no original description	
EX093993	3.489	moderately similar to (233)AT3G25410| Symbols: | bile acid:sodium symporter family protein | chr3:9215802-9217568 REVERSE [2182	
JCVI_21940	3.489	weakly similar to (191)AT2G38940| Symbols: PHT1;4, ATPT2 | ATPT2 (PHOSPHATE TRANSPORTER 2); carbohydrate transmembra	
JCVI_12278	3.487	weakly similar to (182)AT5G53970| Symbols: | aminotransferase, putative | chr5:21927902-21929820 FORWARD no original description	
JCVI_25128	3.486	highly similar to (629)AT2G42520| Symbols: | DEAD box RNA helicase, putative | chr2:17712460-17715822 FORWARDweakly similar	
AM057102	3.483	no similarity	
JCVI_26927	3.483	moderately similar to (392)AT1G60160| Symbols: | potassium transporter family protein | chr1:22191995-22195060 REVERSEmoderatel	
JCVI_9739	3.483	moderately similar to (234)AT4G33110| Symbols: | coclaurine N-methyltransferase, putative | chr4:15972500-15974534 REVERSE no or	
JCVI_36572	3.482	highly similar to (555)AT1G34190| Symbols: ANAC017 | ANAC017 (Arabidopsis NAC domain containing protein 17); transcription facto	
EX053692	3.480	moderately similar to (370)AT3G17240| Symbols: LPD2 | LPD2 (LIPOAMIDE DEHYDROGENASE 2); FAD binding / dihydrolipoyl deh	
ES980774	3.477	weakly similar to (145)AT2G22370| Symbols: | similar to unnamed protein product [Vitis vinifera] (GB:CAO61240.1) | chr2:9508099-95	
JCVI_4046	3.477	moderately similar to (430)AT4G14030| Symbols: SBP1 | selenium-binding protein, putative | chr4:8098116-8100160 REVERSE no origir	
ES905731	3.474	no similarity	
JCVI_2593	3.473	moderately similar to (473)AT1G72480| Symbols: | similar to unknown protein [Arabidopsis thaliana] (TAIR:AT2G01070.1); similar to h	
JCVI_28302	3.471	no original description	
ES930276	3.471	very weakly similar to (81.6)AT2G45010| Symbols: | similar to unknown protein [Arabidopsis thaliana] (TAIR:AT5G51400.1); similar to u	
EE462208	3.471	moderately similar to (449)AT4G14030| Symbols: SBP1 | selenium-binding protein, putative | chr4:8098116-8100160 REVERSE [15722]	
JCVI_14088	3.471	moderately similar to (392)AT3G14690| Symbols: CYP72A15 | CYP72A15 (cytochrome P450, family 72, subfamily A, polypeptide 15); c	
ES995275	3.470	moderately similar to (278)AT3G30390| Symbols: | amino acid transporter family protein | chr3:11979591-11981306 REVERSE [21427]	
EV041972	3.468	moderately similar to (344)AT4G01400| Symbols: | pentatricopeptide (PPR) repeat-containing protein | chr4:573098-577243 REVERSE [	
CV432363	3.468	weakly similar to (118)AT1G74100| Symbols: | sulfotransferase family protein | chr1:27868150-27869166 REVERSE [16490] 190 723 13	
JCVI_6210	3.466	moderately similar to (303)AT5G13930| Symbols: CHS, TT4, ATCHS | ATCHS/CHS/TT4 (CHALCONE SYNTHASE); naringenin-chalc	
AM060805	3.464	moderately similar to (202)AT3G03780| Symbols: AtMS2 | AtMS2 (Arabidopsis thaliana methionine synthase 2) | chr3:957609-960747 F	
JCVI_541	3.460	moderately similar to (398)AT4G30850| Symbols: HHP2 | HHP2 (HEPTAHELICAL TRANSMEMBRANE PROTEIN2) | chr4:15020546	
JCVI_40910	3.459	no original description	
JCVI_7296	3.456	weakly similar to (173)AT2G40420| Symbols: | amino acid transporter family protein | chr2:16884378-16886060 FORWARD no original	
JCVI_39288	3.456	very weakly similar to (98.2)AT3G09880| Symbols: ATB' BETA | ATB' BETA (Arabidopsis thaliana serine/threonine protein phosphatase	
JCVI_30861	3.456	moderately similar to (490)AT5G08280| Symbols: HEMC | HEMC (HYDROXYMETHYLBILANE SYNTHASE); hydroxymethylbilane s	
JCVI_37849	3.455	moderately similar to (243)AT1G54350| Symbols: | ABC transporter family protein | chr1:20290584-20293912 FORWARD no original de	
JCVI_38070	3.455	moderately similar to (249)AT4G27600| Symbols: | pfkB-type carbohydrate kinase family protein | chr4:13782759-13785011 REVERSE r	
EX030851	3.453	moderately similar to (241)AT1G29860| Symbols: ATWRKY71, WRKY71 | WRKY71 (WRKY DNA-binding protein 71); transcription fa	
JCVI_15196	3.448	moderately similar to (399)AT3G23920| Symbols: BMY7, TR-BAMY, BAM1 | BAM1/BMY7/TR-BAMY (BETA-AMYLASE 1); beta-an	
JCVI_2754	3.447	moderately similar to (443)AT3G60750| Symbols: | transketolase, putative | chr3:22464979-22467799 FORWARDmoderately similar to (	-2.287
JCVI_305	3.447	weakly similar to (176)AT5G24010| Symbols: | protein kinase family protein | chr5:8113913-8116387 FORWARD no original description	
EX125854	3.447	weakly similar to (105)AT1G42970| Symbols: GAPB | GAPB (GLYCERALDEHYDE-3-PHOSPHATE DEHYDROGENASE B SUBUNI	-2.310
JCVI_33484	3.444	moderately similar to (345)AT3G51430| Symbols: YLS2 | YLS2 (yellow-leaf-specific gene 2); strictosidine synthase | chr3:19097527-190	
JCVI_445	3.443	moderately similar to (435)AT2G38750| Symbols: ANNAT4 | ANNAT4 (ANNEXIN ARABIDOPSIS 4); calcium ion binding / calcium-de	
BQ704374	3.443	no similarity	
JCVI_41998	3.443	weakly similar to (195)AT3G27400| Symbols: | pectate lyase family protein | chr3:10141560-10144260 FORWARDweakly similar to (12	
DY014304	3.442	moderately similar to (240)AT5G62160| Symbols: ATZIP12 | XLG2/ZIP12 (ZINC TRANSPORTER 12 PRECURSOR); cation transmem	
JCVI_21716	3.441	very weakly similar to (94.0)AT1G24600| Symbols: | similar to unknown protein [Arabidopsis thaliana] (TAIR:AT1G67920.1) | chr1:8720	
ES264149	3.438	moderately similar to (310)AT4G01320| Symbols: STE24, ATSTE24 | ATSTE24 | chr4:545905-549002 FORWARD [15723] 1 596 612	
JCVI_2799	3.437	moderately similar to (247)AT2G35490| Symbols: | plastid-lipid associated protein PAP, putative | chr2:14919388-14920876 REVERSEm	
JCVI_23949	3.435	moderately similar to (206)AT3G20920| Symbols: | translocation protein-related | chr3:7328769-7330704 REVERSE no original descripti	
JCVI_6795	3.433	moderately similar to (417)AT1G15980| Symbols: | similar to unnamed protein product [Vitis vinifera] (GB:CAO49411.1); contains doma	
EV100817	3.433	very weakly similar to (80.1)AT2G30695| Symbols: | similar to unnamed protein product [Vitis vinifera] (GB:CAO46271.1); contains Inte	
JCVI_18373	3.431	moderately similar to (290)AT5G03880| Symbols: | similar to unknown protein [Arabidopsis thaliana] (TAIR:AT4G10000.2); similar to u	
JCVI_17764	3.430	highly similar to (561)AT4G05390| Symbols: ATRFNR1 | ATRFNR1 (ROOT FNR 1); oxidoreductase | chr4:2738836-2740480 REVERSI	
JCVI_33805	3.429	very weakly similar to (81.3)AT5G08139| Symbols: | zinc finger (C3HC4-type RING finger) family protein | chr5:2616488-2617618 FOR	
EV046638	3.429	moderately similar to (270)AT5G44460| Symbols: | calcium-binding protein, putative | chr5:17934513-17935058 FORWARDweakly simi	
EG020012	3.426	moderately similar to (208)AT1G50450| Symbols: | binding / catalytic | chr1:18691570-18694016 REVERSE [20440]	
EE532602	3.426	no similarity	
JCVI_2591	3.425	moderately similar to (351)AT3G61580| Symbols: | delta-8 sphingolipid desaturase (SLD1) | chr3:22797228-22798577 FORWARD no or	
ES269525	3.423	no similarity	
EV152862	3.423	moderately similar to (381)AT2G37040| Symbols: PAL1 | PAL1 (PHE AMMONIA LYASE 1); phenylalanine ammonia-lyase | chr2:15564	
DW997756	3.420	moderately similar to (336)AT1G11750| Symbols: NCLPP1, NCLPP6, CLPP6 | CLPP6 (Clp protease proteolytic subunit 6); endopeptidas	
AM389802	3.418	moderately similar to (229)AT1G68440| Symbols: | similar to unknown protein [Arabidopsis thaliana] (TAIR:AT1G25400.1); similar to u	-2.070
JCVI_2938	3.416	highly similar to (513)AT3G59970| Symbols: MTHFR1 | MTHFR1 (METHYLENETETRAHYDROFOLATE REDUCTASE 1); methylen	
EH430102	3.414	moderately similar to (354)AT4G24120| Symbols: YSL1 | YSL1 (YELLOW STRIPE LIKE 1); oligopeptide transporter | chr4:12524591-1	
JCVI_30115	3.413	weakly similar to (114)AT3G20920| Symbols: | translocation protein-related | chr3:7328769-7330704 REVERSE no original description	
JCVI_34867	3.413	moderately similar to (363)AT5G48930| Symbols: HCT | transferase family protein | chr5:19853880-19855318 REVERSEweakly similar t	
JCVI_4173	3.410	moderately similar to (406)AT1G52760| Symbols: | esterase/lipase/thioesterase family protein | chr1:19655046-19656244 FORWARD no	
EE530917	3.408	moderately similar to (206)AT2G41900| Symbols: | zinc finger (CCCH-type) family protein | chr2:17498430-17500580 FORWARD [201	
EE527184	3.407	moderately similar to (217)AT4G01883| Symbols: | similar to unknown protein [Arabidopsis thaliana] (TAIR:AT1G02475.1); similar to u	
EE418087	3.407	moderately similar to (216)AT5G25100| Symbols: | endomembrane protein 70, putative | chr5:8648377-8651018 REVERSE [20146]	

DY014158	3.405	weakly similar to (121)AT2G37170| Symbols: PIP2;2, PIP2B | PIP2B (plasma membrane intrinsic protein 2;2); water channel | chr2:1562(	
EE426107	3.405	no similarity	
AM388974	3.401	no similarity	
JCVI_2526	3.400	highly similar to (501)AT5G46290| Symbols: KAS I | KAS I (3-KETOACYL-ACYL CARRIER PROTEIN SYNTHASE I); fatty-acid synt	
JCVI_1516	3.399	moderately similar to (390)AT4G17510| Symbols: UCH3 | UCH3; ubiquitin thiolesterase | chr4:9767127-9768661 REVERSE no original c	
JCVI_34247	3.395	moderately similar to (336)AT2G44380| Symbols: | DC1 domain-containing protein | chr2:18330666-18331409 REVERSE no original des	
EV170666	3.394	weakly similar to (129)AT2G41870| Symbols: | remorin family protein | chr2:17478197-17479597 REVERSE [21486] 156 862 862	
JCVI_3301	3.394	moderately similar to (292)AT5G07470| Symbols: PMSR3 | PMSR3 (PEPTIDEMETHIONINE SULFOXIDE REDUCTASE 3); peptide-m	
JCVI_27243	3.393	moderately similar to (322)AT1G67190| Symbols: | F-box family protein | chr1:25136717-25137976 FORWARD no original description	
JCVI_3269	3.393	weakly similar to (188)AT3G51840| Symbols: ATSCX, ATG6, ACX4 | ACX4 (ACYL-COA OXIDASE 4); oxidoreductase | chr3:1923663	
JCVI_14766	3.393	moderately similar to (365)AT5G11880| Symbols: | diaminopimelate decarboxylase, putative / DAP carboxylase, putative | chr5:3827807-	
EV197533	3.393	weakly similar to (175)AT5G26780| Symbols: SHM2 | SHM2 (SERINE HYDROXYMETHYLTRANSFERASE 2); glycine hydroxymethy	
EX088236	3.392	moderately similar to (216)AT4G36440| Symbols: | similar to unnamed protein product [Vitis vinifera] (GB:CAO48295.1) | chr4:1720721	
JCVI_6125	3.391	moderately similar to (327)AT2G01110| Symbols: UNE3, PGA2, TATC, APG2 | APG2 (ALBINO AND PALE GREEN 2) | chr2:83786-8:	
JCVI_37855	3.387	weakly similar to (151)AT1G51660| Symbols: MKK4, ATMKK4 | ATMKK4 (MITOGEN-ACTIVATED PROTEIN KINASE KINASE 4)	-3.120
JCVI_15517	3.387	moderately similar to (387)AT4G26430| Symbols: CSN6B | CSN6B (COP9 SIGNALOSOME SUBUNIT 6B) | chr4:13355237-13357334 I	
H07719	3.385	no similarity	
JCVI_30201	3.385	moderately similar to (497)AT5G63110| Symbols: AXE1, ATHDA6, RTS1, RPD3B, SIL1, HDA6 | HDA6 (HISTONE DEACETYLASE 6	-1.971
EV210392	3.382	no similarity	-2.716
JCVI_9724	3.381	moderately similar to (329)AT1G14290| Symbols: | acid phosphatase, putative | chr1:4880302-4881779 REVERSE no original descriptior	
JCVI_121	3.381	moderately similar to (434)AT2G30490| Symbols: ATC4H, C4H, CYP73A5 | ATC4H/C4H/CYP73A5 (CINNAMATE 4-HYDROXYLAS	
JCVI_1644	3.378	moderately similar to (323)AT4G08780| Symbols: | peroxidase, putative | chr4:5604150-5608199 FORWARDweakly similar to (180)PEF	
JCVI_13130	3.378	moderately similar to (447)AT2G24100| Symbols: | similar to unknown protein [Arabidopsis thaliana] (TAIR:AT4G30780.1); similar to u	
JCVI_8783	3.378	moderately similar to (475)AT5G07590| Symbols: | WD-40 repeat protein family | chr5:2401713-2403732 REVERSE no original descript	
EE546223	3.373	very weakly similar to (87.0)AT2G45960| Symbols: TMP-A, ATHH2, PIP1;2, PIP1B | PIP1B (plasma membrane intrinsic protein 1;2) | chr	
JCVI_29661	3.373	moderately similar to (365)AT3G03480| Symbols: CHAT | CHAT (ACETYL COA:(Z)-3-HEXEN-1-OL ACETYLTRANSFERASE); acet	
JCVI_3895	3.372	moderately similar to (269)AT1G52870| Symbols: | peroxisomal membrane protein-related | chr1:19689524-19691346 FORWARD no ori	
CN726635	3.372	no similarity	
ES265414	3.372	no similarity	
JCVI_14569	3.370	weakly similar to (129)AT1G02470| Symbols: | similar to unknown protein [Arabidopsis thaliana] (TAIR:AT1G02475.1); similar to unnar	
JCVI_39765	3.368	moderately similar to (258)AT2G18950| Symbols: TPT1, ATHPT, HPT, VTE2, HPT1 | HPT1 (HOMOGENTISATE PHYTYLTRANSFE	
JCVI_41280	3.368	no original description	
JCVI_12311	3.368	moderately similar to (338)AT1G55910| Symbols: ZIP11 | ZIP11 (ZINC TRANSPORTER 11 PRECURSOR); cation transmembrane trans	
CD814776	3.365	weakly similar to (122)AT5G49000| Symbols: | Identical to F-box/Kelch-repeat protein At5g49000 [Arabidopsis Thaliana] (GB:Q9FI70;C	
EV215440	3.364	weakly similar to (187)AT5G54080| Symbols: HGO | HGO (HOMOGENTISATE 1,2-DIOXYGENASE); homogentisate 1,2-dioxygenase	
JCVI_5035	3.362	moderately similar to (429)AT5G38100| Symbols: | methyltransferase-related | chr5:15217014-15218398 REVERSEweakly similar to (14	
CN830519	3.361	moderately similar to (243)AT1G31600| Symbols: | oxidoreductase, 2OG-Fe(II) oxygenase family protein | chr1:11313357-11315180 REV	
JCVI_9941	3.358	no original description	-2.153
JCVI_13390	3.358	moderately similar to (290)AT5G11410| Symbols: | protein kinase family protein | chr5:3638432-3639884 REVERSEvery weakly similar	
JCVI_125	3.358	highly similar to (540)AT5G58290| Symbols: RPT3 | RPT3 (root phototropism 3); ATPase | chr5:23586381-23588342 FORWARDmodera	
JCVI_41377	3.357	moderately similar to (303)AT1G02930| Symbols: GST1, ERD11, ATGSTF3, GSTF6, ATGSTF6 | ATGSTF6 (EARLY RESPONSIVE TC	
EH413915	3.357	moderately similar to (302)AT3G21760| Symbols: | UDP-glucoronosyl/UDP-glucosyl transferase family protein | chr3:7667106-7668563	
EV015432	3.356	no similarity	
CO729360	3.355	no similarity	
JCVI_23817	3.354	very weakly similar to (80.1)AT3G61470| Symbols: LHCA2 | LHCA2 (Photosystem I light harvesting complex gene 2); chlorophyll binding	
EX125015	3.354	moderately similar to (357)AT5G40240| Symbols: | nodulin MtN21 family protein | chr5:16099553-16101694 REVERSE [21830]	
EV111111	3.353	no similarity	
JCVI_2819	3.352	moderately similar to (357)AT1G29150| Symbols: RPN6, ATS9 | ATS9 (19S PROTEOSOME SUBUNIT 9) | chr1:10181226-10182485 F(	
JCVI_35063	3.350	moderately similar to (284)AT2G40950| Symbols: BZIP17 | BZIP17; DNA binding / transcription activator/ transcription factor | chr2:170	
BQ704699	3.349	weakly similar to (177)AT5G46050| Symbols: ATPTR3, PTR3 | ATPTR3/PTR3 (PEPTIDE TRANSPORTER PROTEIN 3); transporter |	1.201
EX124070	3.347	weakly similar to (200)AT4G33565| Symbols: | zinc finger (C3HC4-type RING finger) family protein | chr4:16137312-16137926 FORWA	
JCVI_39239	3.346	moderately similar to (345)AT2G44380| Symbols: | DC1 domain-containing protein | chr2:18330666-18331409 REVERSE no original des	
JCVI_4115	3.345	highly similar to (753)AT3G13920| Symbols: RH4, TIF4A1, EIF4A1 | EIF4A1 (eukaryotic translation initiation factor 4A-1) | chr3:459264	
JCVI_11359	3.344	moderately similar to (226)AT5G61820| Symbols: | similar to MtN19-like protein [Pisum sativum] (GB:AAU14999.2); contains InterPro (	
ES940671	3.343	moderately similar to (246)AT3G60590| Symbols: | similar to unknown protein [Arabidopsis thaliana] (TAIR:AT1G48460.1); similar to u	
JCVI_25852	3.341	moderately similar to (329)AT2G35390| Symbols: | ribose-phosphate pyrophosphokinase 1 / phosphoribosyl diphosphate synthetase 1 (PF	-2.073
JCVI_7747	3.338	highly similar to (923)AT3G14067| Symbols: | subtilase family protein | chr3:4658428-4660761 REVERSE no original description	
JCVI_5478	3.337	highly similar to (521)AT3G27740| Symbols: CARA | CARA (CARBAMOYL PHOSPHATE SYNTHETASE A) | chr3:10282707-102850	
JCVI_14474	3.337	moderately similar to (323)AT1G07230| Symbols: | phosphoesterase family protein | chr1:2220508-2222777 REVERSE no original descri	
JCVI_1296	3.335	highly similar to (518)AT2G30490| Symbols: ATC4H, C4H, CYP73A5 | ATC4H/C4H/CYP73A5 (CINNAMATE 4-HYDROXYLASE, CI	
JCVI_22023	3.332	moderately similar to (322)AT1G02335| Symbols: GL22 | GL22 (GERMIN-LIKE PROTEIN SUBFAMILY 2 MEMBER 2 PRECURSOR	
JCVI_5728	3.332	moderately similar to (381)AT1G32080| Symbols: | membrane protein, putative | chr1:11537552-11539736 REVERSE no original descrip	
JCVI_7727	3.331	highly similar to (504)AT3G04520| Symbols: THA2 | THA2 (THREONINE ALDOLASE 2); threonine aldolase | chr3:1217403-1219577 F	
JCVI_27554	3.330	moderately similar to (402)AT3G62020| Symbols: GLP10 | GLP10 (GERMIN-LIKE PROTEIN 10); manganese ion binding / metal ion bir	
JCVI_17031	3.329	moderately similar to (444)AT3G51240| Symbols: TT6, F3'H, F3H | F3H (TRANSPARENT TESTA 6); naringenin 3-dioxygenase | chr3:1	
CX190952	3.328	moderately similar to (269)AT2G24490| Symbols: RPA2, ATRPA2, ROR1 | ATRPA2/ROR1/RPA2 (REPLICON PROTEIN A) | chr2:104	
L38156	3.328	no similarity	
JCVI_36094	3.327	moderately similar to (329)AT4G24690| Symbols: | ubiquitin-associated (UBA)/TS-N domain-containing protein / octicosapeptide/Phox/I	
EE548064	3.326	no similarity	
EV131109	3.326	no similarity	
JCVI_4153	3.326	moderately similar to (394)AT5G59540| Symbols: | oxidoreductase, 2OG-Fe(II) oxygenase family protein | chr5:24013841-24014802 REV	
JCVI_38555	3.325	moderately similar to (277)AT3G49210| Symbols: | similar to unknown protein [Arabidopsis thaliana] (TAIR:AT3G49200.1); similar to c	
JCVI_9369	3.323	weakly similar to (113)AT5G03480| Symbols: | nucleic acid binding / nucleotide binding | chr5:869207-870854 REVERSE no original des	
JCVI_46	3.323	moderately similar to (370)AT4G01850| Symbols: SAM-2, MAT2 | MAT2/SAM-2 (S-adenosylmethionine synthetase 2) | chr4:796298-79	-1.992
JCVI_31051	3.323	highly similar to (640)AT1G14360| Symbols: ATUTR3, UTR3 | ATUTR3/UTR3 (UDP-GALACTOSE TRANSPORTER 3); pyrimidine n	
JCVI_4434	3.322	moderately similar to (370)AT3G56460| Symbols: | oxidoreductase, zinc-binding dehydrogenase family protein | chr3:20944008-2094540	
JCVI_721	3.322	moderately similar to (384)AT4G29040| Symbols: RPT2A | RPT2A (REGULATORY PARTICLE AAA-ATPASE 2A); ATPase | chr4:143	
JCVI_6699	3.321	weakly similar to (194)AT1G70700| Symbols: JAZ9, TIFY7 | JAZ9/TIFY7 (JASMONATE-ZIM-DOMAIN PROTEIN 9) | chr1:26658614	
EE535363	3.320	moderately similar to (343)AT3G26230| Symbols: CYP71B24 | CYP71B24 (cytochrome P450, family 71, subfamily B, polypeptide 24); o	
ES943509	3.320	moderately similar to (313)AT5G46290| Symbols: KAS I | KAS I (3-KETOACYL-ACYL CARRIER PROTEIN SYNTHASE I); fatty-acid	
JCVI_8135	3.320	moderately similar to (355)AT5G10600| Symbols: CYP81K2 | CYP81K2 (cytochrome P450, family 81, subfamily K, polypeptide 2); oxyg	

EE407914	3.319	moderately similar to (354)AT5G16050| Symbols: GF14 UPSILON, GRF5 | GRF5 (GENERAL REGULATORY FACTOR 5); protein pho	
JCVI_22305	3.319	moderately similar to (473)AT2G40420| Symbols: | amino acid transporter family protein | chr2:16884378-16886060 FORWARD no orig	
JCVI_886	3.319	moderately similar to (448)AT2G42590| Symbols: GF14 MU, GRF9 | GRF9 (GENERAL REGULATORY FACTOR 9); protein phosphory	
JCVI_40516	3.317	weakly similar to (119)AT1G13440| Symbols: GAPC-2 | GAPC-2 | chr1:4608462-4610491 REVERSEweakly similar to (119)G3PC_SINA	
JCVI_407	3.317	moderately similar to (382)AT2G26900| Symbols: | bile acid:sodium symporter family protein | chr2:11482234-11484948 REVERSE no o	
EV107352	3.317	weakly similar to (139)AT3G47110| Symbols: | leucine-rich repeat transmembrane protein kinase, putative | chr3:17358088-17361281 RE	
JCVI_2150	3.317	moderately similar to (223)AT4G00430| Symbols: PIP1;4, PIP1E, TMP-C | TMP-C (PLASMA MEMBRANE INTRINSIC PROTEIN 1;4	
EE561774	3.316	no similarity	
EE527043	3.316	no similarity	
JCVI_32257	3.316	highly similar to (514)AT4G28480| Symbols: | DNAJ heat shock family protein | chr4:14073316-14075097 FORWARDvery weakly simila	-2.188
ES920297	3.314	moderately similar to (207)AT2G02030| Symbols: | F-box family protein | chr2:482334-483828 FORWARD [15718]	
EE475977	3.311	moderately similar to (367)AT2G22240| Symbols: | inositol-3-phosphate synthase isozyme 2 / myo-inositol-1-phosphate synthase 2 / MI-1	-2.366
JCVI_30812	3.311	moderately similar to (235)AT1G08630| Symbols: THA1 | THA1 (THREONINE ALDOLASE 1) | chr1:2743951-2745688 REVERSE no o	-2.417
AM058964	3.309	no similarity	
JCVI_6774	3.309	weakly similar to (122)AT2G18230| Symbols: ATPPA2 | ATPPA2 (ARABIDOPSIS THALIANA PYROPHOSPHORYLASE 2); inorgani	
CV546026	3.307	no similarity	
EH430551	3.304	weakly similar to (189)AT3G56710| Symbols: SIB1 | SIB1 (SIGMA FACTOR BINDING PROTEIN 1); binding | chr3:21018032-2101848	
JCVI_41195	3.304	no original description	
JCVI_28668	3.303	moderately similar to (209)AT2G37970| Symbols: SOUL-1 | SOUL-1; binding | chr2:15898105-15898782 FORWARD no original descript	
JCVI_9864	3.302	highly similar to (508)AT1G15690| Symbols: ATAVP3, AVP-3, AVP1 | AVP1 (vacuolar-type H+-pumping pyrophosphatase 1) | chr1:539	
EX089812	3.302	moderately similar to (282)AT1G23230| Symbols: | similar to hypothetical protein OsJ_007998 [Oryza sativa (japonica cultivar-group)] (C	
DQ023575	3.301	very weakly similar to (85.9)AT5G03880| Symbols: | similar to unknown protein [Arabidopsis thaliana] (TAIR:AT4G10000.2); similar to u	
JCVI_7626	3.300	moderately similar to (216)AT3G16800| Symbols: | protein phosphatase 2C, putative / PP2C, putative | chr3:5721300-5722725 FORWAR	
JCVI_16283	3.299	moderately similar to (327)AT2G29900| Symbols: | presenilin family protein | chr2:12756914-12758107 FORWARD no original descripti	
JCVI_4638	3.299	moderately similar to (488)AT3G53420| Symbols: PIP2, PIP2;1, PIP2A | PIP2A (PLASMA MEMBRANE INTRINSIC PROTEIN 2A) | c	
JCVI_7716	3.298	moderately similar to (342)AT4G38250| Symbols: | amino acid transporter family protein | chr4:17935527-17936837 FORWARD no orig	
EE447713	3.297	moderately similar to (287)AT4G38160| Symbols: PDE191 | PDE191 (PIGMENT DEFECTIVE 191) | chr4:17902406-17903778 FORWA	
EX059093	3.297	moderately similar to (226)AT4G23160| Symbols: | protein kinase family protein | chr4:12129496-12134097 FORWARD [21813]	
JCVI_624	3.295	moderately similar to (291)AT5G48850| Symbols: | male sterility MS5 family protein | chr5:19822802-19824925 REVERSE no original d	
JCVI_3368	3.295	moderately similar to (285)AT5G35220| Symbols: EGY1 | EGY1 (ETHYLENE-DEPENDENT GRAVITROPISM-DEFICIENT AND YE	
JCVI_16058	3.294	weakly similar to (174)AT5G62150| Symbols: | peptidoglycan-binding LysM domain-containing protein | chr5:24975551-24975859 FORV	
JCVI_5822	3.294	moderately similar to (412)AT5G02240| Symbols: | binding / catalytic/ coenzyme binding | chr5:451500-452982 FORWARD no original d	
DT317691	3.294	weakly similar to (104)AT3G53260| Symbols: PAL2 | PAL2 (phenylalanine ammonia-lyase 2); phenylalanine ammonia-lyase | chr3:19755	
JCVI_20710	3.291	moderately similar to (293)AT4G31780| Symbols: MGDA, MGD1 | MGD1 (MONOGALACTOSYLDIACYLGLYCEROL SYNTHASE 1	
JCVI_3558	3.290	moderately similar to (437)AT4G38790| Symbols: | ER lumen protein retaining receptor family protein | chr4:18111420-18112971 FORW	
JCVI_34845	3.289	moderately similar to (312)AT1G19050| Symbols: ARR7 | ARR7 (RESPONSE REGULATOR 7); transcription regulator/ two-component	
JCVI_39731	3.289	moderately similar to (248)AT5G24380| Symbols: ATYSL2, YSL2 | YSL2 (YELLOW STRIPE LIKE 2); oligopeptide transporter | chr5:83	
ES268747	3.289	moderately similar to (227)AT1G11880| Symbols: | similar to unnamed protein product [Vitis vinifera] (GB:CAO22037.1); contains Inter	
JCVI_32553	3.288	moderately similar to (266)AT2G26570| Symbols: | similar to unknown protein [Arabidopsis thaliana] (TAIR:AT4G33390.1); similar to u	
JCVI_634	3.287	moderately similar to (367)AT1G78380| Symbols: GST8, ATGSTU19 | ATGSTU19 (GLUTATHIONE TRANSFERASE 8); glutathione tr	
JCVI_37876	3.286	moderately similar to (274)AT1G08570| Symbols: | thioredoxin family protein | chr1:2713607-2714315 FORWARD no original descriptio	
JCVI_27129	3.286	moderately similar to (357)AT4G29400| Symbols: | oxidoreductase/ transition metal ion binding | chr4:14466356-14467986 FORWARD n	
JCVI_9795	3.286	moderately similar to (268)AT3G47520| Symbols: MDH | MDH (MALATE DEHYDROGENASE); malate dehydrogenase | chr3:1752464	
JCVI_15420	3.286	moderately similar to (447)AT1G77510| Symbols: ATPDIL1-2 | ATPDIL1-2 (PDI-LIKE 1-2); protein disulfide isomerase | chr1:2913163	
DT317694	3.284	no similarity	
EV119497	3.284	moderately similar to (363)AT3G24290| Symbols: | ammonium transporter, putative | chr3:8801407-8802897 REVERSE [21479] 44 766	
EX116683	3.283	moderately similar to (225)AT5G05110| Symbols: | cysteine protease inhibitor, putative / cystatin, putative | chr5:1507616-1508766 REV	
JCVI_8544	3.282	weakly similar to (177)AT1G63900| Symbols: | zinc finger (C3HC4-type RING finger) family protein | chr1:23720719-23722749 FORWA	
JCVI_30461	3.282	highly similar to (829)AT1G04730| Symbols: | AAA-type ATPase family protein | chr1:1325384-1331085 REVERSE no original descripti	
JCVI_3896	3.280	weakly similar to (198)AT5G18130| Symbols: | similar to unknown protein [Arabidopsis thaliana] (TAIR:AT3G03870.2); similar to unkno	
JCVI_25986	3.278	moderately similar to (317)AT2G40970| Symbols: | myb family transcription factor | chr2:17104850-17105596 REVERSE no original des	
JCVI_7853	3.278	moderately similar to (281)AT1G62180| Symbols: APSR, PRH43, PRH, ATAPR2, APR2 | APR2 (5'ADENYLYLPHOSPHOSULFATE R	
JCVI_11213	3.278	moderately similar to (319)AT2G38640| Symbols: | similar to unknown protein [Arabidopsis thaliana] (TAIR:AT5G41590.1); similar to u	
JCVI_23989	3.277	weakly similar to (122)AT3G07560| Symbols: APM2, PEX13 | APM2/PEX13 (ABERRANT PEROXISOME MORPHOLOGY 2); protein	
CD836978	3.276	no similarity	
JCVI_819	3.275	moderately similar to (319)AT4G01610| Symbols: | cathepsin B-like cysteine protease, putative | chr4:694857-696937 FORWARD no orig	
JCVI_12909	3.274	moderately similar to (359)AT3G17000| Symbols: UBC32 | UBC32 (UBIQUITIN-CONJUGATING ENZYME 32); ubiquitin-protein ligas	
JCVI_9139	3.274	moderately similar to (385)AT1G65960| Symbols: GAD2 | GAD2 (GLUTAMATE DECARBOXYLASE 2) | chr1:24555757-24560916 FC	
JCVI_1139	3.272	moderately similar to (381)AT2G33150| Symbols: PED1, KAT2 | KAT2/PED1 (PEROXISOME DEFECTIVE 1); acetyl-CoA C-acyltrans	
EV103504	3.272	moderately similar to (469)AT5G05200| Symbols: | ABC1 family protein | chr5:1544207-1547083 REVERSE [21477] 41 784 784	
CD834583	3.270	moderately similar to (283)AT3G55120| Symbols: TT5, A11, CFI | A11/CFI/TT5 (TRANSPARENT TESTA 5); chalcone isomerase | chr3	
JCVI_26921	3.270	moderately similar to (412)AT4G20830| Symbols: | FAD-binding domain-containing protein | chr4:11155497-11157119 FORWARD no o	
EV052322	3.269	moderately similar to (263)AT5G28840| Symbols: GME | GME (GDP-D-MANNOSE 3',5'-EPIMERASE); GDP-mannose 3,5-epimerase/ N	3.445
CV432063	3.266	no similarity	
CD820575	3.265	weakly similar to (161)AT2G34340| Symbols: | similar to unknown protein [Arabidopsis thaliana] (TAIR:AT1G29640.1); similar to unkno	
JCVI_19222	3.265	weakly similar to (130)AT5G36910| Symbols: THI2.2 | THI2.2 (THIONIN 2.2); toxin receptor binding | chr5:14579283-14579903 REVEI	
JCVI_13423	3.265	highly similar to (545)AT4G21320| Symbols: HSA32 | HSA32 (HEAT-STRESS-ASSOCIATED 32); catalytic | chr4:11340502-11341742	-1.632
JCVI_11498	3.264	highly similar to (513)AT4G23400| Symbols: PIP1D, PIP1;5 | PIP1;5/PIP1D (plasma membrane intrinsic protein 1;5); water channel | chr4	
JCVI_4683	3.263	highly similar to (561)AT1G11910| Symbols: | aspartyl protease family protein | chr1:4017117-4019872 REVERSEmoderately similar to (	
JCVI_34074	3.262	moderately similar to (413)AT1G11190| Symbols: ENDO1, BFN1 | BFN1 (BIFUNCTIONAL NUCLEASE I); nucleic acid binding | chr1:3	
JCVI_15992	3.262	moderately similar to (370)AT1G08650| Symbols: PPCK1 | PPCK1 (PHOSPHOENOLPYRUVATE CARBOXYLASE KINASE); kinase |	
EX060979	3.261	no similarity	1.465
JCVI_12543	3.261	moderately similar to (224)AT5G13860| Symbols: ELC-LIKE | ATELC-LIKE/ELC-LIKE; small conjugating protein ligase | chr5:4473214	
EE560202	3.260	very weakly similar to (90.5)AT4G25225| Symbols: | similar to unknown protein [Arabidopsis thaliana] (TAIR:AT2G35736.1); similar to u	
EG020050	3.259	very weakly similar to (100)AT4G27900| Symbols: | similar to unknown protein [Arabidopsis thaliana] (TAIR:AT5G53420.1); similar to u	
JCVI_8318	3.256	moderately similar to (358)AT3G10420| Symbols: | sporulation protein-related | chr3:3239312-3241576 FORWARD no original descriptio	
ES980361	3.254	weakly similar to (139)AT5G56420| Symbols: | F-box family protein | chr5:22868089-22869560 REVERSE [21388]	
JCVI_2309	3.254	moderately similar to (348)AT2G44050| Symbols: COS1 | COS1 (COI1 SUPPRESSOR1); 6,7-dimethyl-8-ribityllumazine synthase | chr2:1	
JCVI_17176	3.254	moderately similar to (492)AT1G02100| Symbols: | leucine carboxyl methyltransferase family protein | chr1:389876-392448 FORWARD	
JCVI_8820	3.250	moderately similar to (478)AT1G53710| Symbols: | similar to unnamed protein product [Vitis vinifera] (GB:CAO68485.1); contains Inter	
EV052055	3.249	moderately similar to (342)AT4G04750| Symbols: | carbohydrate transmembrane transporter/ sugar:hydrogen ion symporter | chr4:241810	

JCVI_42105	3.249	highly similar to (521)AT3G51430| Symbols: YLS2 | YLS2 (yellow-leaf-specific gene 2); strictosidine synthase | chr3:19097527-19098888	
EX066757	3.249	moderately similar to (358)AT5G53830| Symbols: | VQ motif-containing protein | chr5:21874283-21875014 FORWARD [21815]	
JCVI_13500	3.248	moderately similar to (310)AT1G29160| Symbols: | Dof-type zinc finger domain-containing protein | chr1:10183783-10184310 REVERSE	
EX073124	3.248	very weakly similar to (85.1)AT5G54840| Symbols: | GTP-binding family protein | chr5:22294587-22295554 REVERSE [21817]	
EE509320	3.248	weakly similar to (198)AT4G31600| Symbols: | UDP-glucuronic acid/UDP-N-acetylgalactosamine transporter-related | chr4:15315371-15	-4.972
JCVI_42533	3.247	no original description	
JCVI_5438	3.246	moderately similar to (469)AT4G02930| Symbols: | elongation factor Tu, putative / EF-Tu, putative | chr4:1295751-1298354 REVERSEm	
EV216602	3.245	moderately similar to (301)AT2G01490| Symbols: | phytanoyl-CoA dioxygenase (PhyH) family protein | chr2:221315-223186 FORWARD	
JCVI_18858	3.243	moderately similar to (365)AT5G67210| Symbols: | nucleic acid binding / pancreatic ribonuclease | chr5:26836245-26837198 FORWARD	
JCVI_4592	3.242	moderately similar to (310)AT2G41040| Symbols: | methyltransferase-related | chr2:17128577-17130142 FORWARD no original descript	1.495
JCVI_4687	3.241	moderately similar to (283)AT5G33320| Symbols: PPT, ARAPPT, CUE1 | CUE1 (CAB UNDEREXPRESSED 1); antiporter/ triose-phosp	-3.349
JCVI_853	3.240	moderately similar to (454)AT3G24170| Symbols: ATGR1 | ATGR1 (GLUTATHIONE-DISULFIDE REDUCTASE) | chr3:8729769-8734	
JCVI_2473	3.240	moderately similar to (308)AT5G41210| Symbols: GST10, ATGSTT1 | ATGSTT1 (Arabidopsis thaliana Glutathione S-transferase (class t	
JCVI_6032	3.238	moderately similar to (388)AT3G01060| Symbols: | similar to unnamed protein product [Vitis vinifera] (GB:CAO15045.1); similar to unk	
JCVI_38481	3.237	moderately similar to (288)AT1G75280| Symbols: | isoflavone reductase, putative | chr1:28255691-28257016 FORWARDmoderately sim	
EE481364	3.236	weakly similar to (153)AT3G28460| Symbols: | similar to hypothetical protein [Vitis vinifera] (GB:CAN74970.1); contains InterPro doma	
JCVI_13173	3.236	moderately similar to (455)AT1G80820| Symbols: CCR2 | CCR2 (CINNAMOYL COA REDUCTASE) | chr1:30375538-30377352 FORW	-3.626
CV432250	3.235	no similarity	
EX113523	3.232	weakly similar to (162)AT4G23150| Symbols: | protein kinase family protein | chr4:12125742-12128312 FORWARD [21827]	-2.000
JCVI_31371	3.232	weakly similar to (191)AT4G17840| Symbols: | similar to unknown protein [Arabidopsis thaliana] (TAIR:AT2G35260.1); similar to hypot	
AI352922	3.231	very weakly similar to (83.2)AT2G39530| Symbols: | integral membrane protein, putative | chr2:16505737-16506479 REVERSE [1285]	
JCVI_21684	3.230	weakly similar to (141)AT3G10450| Symbols: SCPL7 | SCPL7; serine carboxypeptidase | chr3:3249775-3252325 FORWARDvery weakly	
JCVI_9022	3.228	moderately similar to (337)AT1G51860| Symbols: | leucine-rich repeat protein kinase, putative | chr1:19261303-19265148 REVERSEwea	
DY021272	3.227	moderately similar to (249)AT5G23070| Symbols: | thymidine kinase, putative | chr5:7741982-7742830 FORWARDmoderately similar to	
EX093233	3.227	weakly similar to (128)AT2G45140| Symbols: | vesicle-associated membrane protein, putative / VAMP, putative | chr2:18618104-186200	
JCVI_1948	3.227	moderately similar to (328)AT4G18480| Symbols: CH42, CH-42, CHL11, CHLI-1, CHLI1 | CHLI1 (CHLORINA 42); magnesium chelatas	
CX195418	3.227	weakly similar to (111)AT5G59840| Symbols: | Ras-related GTP-binding family protein | chr5:24124676-24126275 REVERSEweakly sim	
EV111174	3.227	no similarity	
JCVI_3780	3.226	moderately similar to (441)AT1G80560| Symbols: | 3-isopropylmalate dehydrogenase, chloroplast, putative | chr1:30292725-30295018 FO	
JCVI_4445	3.225	moderately similar to (425)AT1G65560| Symbols: | allyl alcohol dehydrogenase, putative | chr1:24375271-24377336 REVERSE no origin	
JCVI_3867	3.224	moderately similar to (384)AT3G12780| Symbols: PGK1 | PGK1 (PHOSPHOGLYCERATE KINASE 1); phosphoglycerate kinase | chr3:4	
EX101988	3.224	weakly similar to (157)AT4G10890| Symbols: | similar to unknown protein [Arabidopsis thaliana] (TAIR:AT1G43722.1); similar to unnar	
JCVI_27492	3.223	weakly similar to (152)AT3G60966| Symbols: | protein binding / zinc ion binding | chr3:22563693-22564112 FORWARD no original desc	
JCVI_4917	3.222	moderately similar to (422)AT3G11900| Symbols: ANT1 | ANT1 (AROMATIC AND NEUTRAL TRANSPORTER 1); amino acid transm	
JCVI_5787	3.220	very weakly similar to (94.7)AT5G03480| Symbols: | nucleic acid binding / nucleotide binding | chr5:869207-870854 REVERSE no origina	
EE526300	3.220	no similarity	
JCVI_35597	3.219	no original description	
JCVI_3462	3.219	moderately similar to (426)AT1G22920| Symbols: CSN5A, JAB1, AJH1 | AJH1/CSN5A/JAB1 (COP9 SIGNALOSOME 5A) | chr1:81099	
DN962755	3.217	moderately similar to (418)AT2G39210| Symbols: | nodulin family protein | chr2:16373365-16375309 REVERSE [17359]	-1.890
DV643295	3.217	no similarity	
JCVI_24842	3.217	moderately similar to (239)AT5G57030| Symbols: LUT2 | LUT2 (LUTEIN DEFICIENT 2); lycopene epsilon cyclase | chr5:23094624-230	
ES936675	3.215	no similarity	
JCVI_21306	3.214	moderately similar to (330)AT3G09035| Symbols: | legume lectin family protein | chr3:2759072-2760088 FORWARD no original descrip	
JCVI_7700	3.212	highly similar to (518)AT5G02500| Symbols: HSP70-1, AT-HSC70-1, HSC70, HSC70-1 | HSC70-1 (heat shock cognate 70 kDa protein 1)	
JCVI_15647	3.211	highly similar to (719)AT1G21680| Symbols: | similar to unknown protein [Arabidopsis thaliana] (TAIR:AT1G21670.1); similar to hypotl	
JCVI_4329	3.210	highly similar to (590)AT1G11910| Symbols: | aspartyl protease family protein | chr1:4017117-4019872 REVERSEmoderately similar to (	
EV067986	3.208	no similarity	-2.145
DN964006	3.208	weakly similar to (108)AT3G48850| Symbols: | mitochondrial phosphate transporter, putative | chr3:18125744-18127405 REVERSE [173	-4.268
JCVI_34968	3.207	weakly similar to (141)AT5G08730| Symbols: | IBR domain-containing protein | chr5:2845825-2847416 REVERSE no original descriptio	
JCVI_15034	3.205	highly similar to (540)AT2G30950| Symbols: FTSH2, VAR2 | VAR2 (VARIEGATED 2); ATP-dependent peptidase/ ATPase/ metallopept	
EE429349	3.205	moderately similar to (233)AT1G10960| Symbols: ATFD1 | ATFD1 (FERREDOXIN 1); 2 iron, 2 sulfur cluster binding / electron carrier/ i	
JCVI_26448	3.205	highly similar to (720)AT3G45140| Symbols: ATLOX2, LOX2 | LOX2 (LIPOXYGENASE 2) | chr3:16536422-16540218 FORWARDhigh	
JCVI_30233	3.203	moderately similar to (363)AT3G14790| Symbols: RHM3 | RHM3 (RHAMNOSE BIOSYNTHESIS 3); catalytic | chr3:4964798-4966882	
JCVI_13489	3.203	moderately similar to (333)AT1G28280| Symbols: | VQ motif-containing protein | chr1:9886284-9887382 REVERSE no original descripti	
JCVI_20631	3.203	very weakly similar to (91.3)AT3G49570| Symbols: | similar to unknown protein [Arabidopsis thaliana] (TAIR:AT5G24660.1); similar to u	
JCVI_15520	3.202	moderately similar to (214)AT2G40475| Symbols: | unknown protein | chr2:16914300-16914881 REVERSE no original description	-4.166
JCVI_22703	3.202	moderately similar to (380)AT3G49560| Symbols: | mitochondrial import inner membrane translocase subunit Tim17/Tim22/Tim23 famil	
JCVI_2712	3.201	moderately similar to (417)AT5G62740| Symbols: | band 7 family protein | chr5:25218546-25219761 FORWARD no original description	
CB617689	3.201	no similarity	
JCVI_39565	3.201	weakly similar to (142)AT4G29905| Symbols: | similar to unknown protein [Arabidopsis thaliana] (TAIR:AT5G57123.1); similar to hypot	
JCVI_887	3.198	moderately similar to (377)AT2G28000| Symbols: CH-CPN60A, SLP, CPN60A | CPN60A (chloroplast / 60 kDa chaperonin alpha subunit	
JCVI_744	3.196	moderately similar to (257)AT4G28910| Symbols: | similar to nuclear transport factor 2 (NTF2) family protein / RNA recognition motif (I	
JCVI_18577	3.196	highly similar to (704)AT3G51250| Symbols: | senescence/dehydration-associated protein-related | chr3:19039206-19041421 FORWARD	
JCVI_28856	3.196	moderately similar to (275)AT5G65990| Symbols: | amino acid transporter family protein | chr5:26412181-26413547 FORWARD no orig	
AM391152	3.196	weakly similar to (142)AT2G24530| Symbols: | similar to unknown protein [Arabidopsis thaliana] (TAIR:AT4G31440.1); similar to unnai	
L38165	3.196	no similarity	
JCVI_368	3.195	moderately similar to (410)AT5G11520| Symbols: YLS4, ASP3 | ASP3 (ASPARTATE AMINOTRANSFERASE 3) | chr5:3685258-36877	
JCVI_2632	3.195	moderately similar to (328)AT5G46290| Symbols: KAS I | KAS I (3-KETOACYL-ACYL CARRIER PROTEIN SYNTHASE I); fatty-acid	
JCVI_38642	3.194	moderately similar to (300)AT2G14860| Symbols: | peroxisomal membrane protein 22 kDa, putative | chr2:6394902-6396645 REVERSE	
JCVI_16268	3.194	moderately similar to (253)AT1G02470| Symbols: | similar to unknown protein [Arabidopsis thaliana] (TAIR:AT1G02475.1); similar to u	
JCVI_10817	3.193	highly similar to (643)AT1G52730| Symbols: | transducin family protein / WD-40 repeat family protein | chr1:19646534-19648646 FORW	
EX100651	3.193	moderately similar to (232)AT4G35060| Symbols: | heavy-metal-associated domain-containing protein / copper chaperone (CCH)-related	
JCVI_6438	3.193	moderately similar to (406)AT3G59970| Symbols: MTHFR1 | MTHFR1 (METHYLENETETRAHYDROFOLATE REDUCTASE 1); metl	
JCVI_4117	3.191	moderately similar to (282)AT5G16360| Symbols: | NC domain-containing protein | chr5:5355239-5356198 REVERSE no original descrip	
AM391161	3.191	moderately similar to (220)AT1G69920| Symbols: ATGSTU12 | ATGSTU12 (Arabidopsis thaliana Glutathione S-transferase (class tau) 1	
JCVI_15631	3.191	moderately similar to (327)AT2G40490| Symbols: HEME2 | HEME2; uroporphyrinogen decarboxylase | chr2:16920039-16922066 FORW	
EX124350	3.190	moderately similar to (348)AT1G35660| Symbols: | binding | chr1:13192721-13197262 FORWARD [21830] 14 744 744	
JCVI_11854	3.190	moderately similar to (317)AT1G14370| Symbols: APK2A | APK2A (PROTEIN KINASE 2A); kinase | chr1:4915854-4917954 FORWAR	
EX021577	3.188	weakly similar to (140)AT5G19875| Symbols: | similar to unknown protein [Arabidopsis thaliana] (TAIR:AT2G31940.1); similar to hypot	-1.285
EE439077	3.186	moderately similar to (219)AT2G38230| Symbols: ATPDX1.1 | ATPDX1.1 (PYRIDOXINE BIOSYNTHESIS 1.1); protein heterodimeriza	
AM394745	3.186	weakly similar to (184)AT2G47800| Symbols: EST3, ATMRP4 | ATMRP4 (Arabidopsis thaliana multidrug resistance-associated protein 4	

EV215047	3.186	moderately similar to (261)AT1G19920| Symbols: ASA1, APS2 | APS2 (ATP SULFURYLASE PRECURSOR) | chr1:6914826-6916648 R	
L38152	3.186	weakly similar to (102)AT1G63000| Symbols: UER1, NRS/ER | NRS/ER (NUCLEOTIDE-RHAMNOSE SYNTHASE/EPIMERASE-RED	
JCVI_35866	3.185	moderately similar to (390)AT2G19540| Symbols: | transducin family protein / WD-40 repeat family protein | chr2:8468886-8471429 FOI	
CV433548	3.184	very weakly similar to (93.2)AT5G15650| Symbols: RGP2 | RGP2 (REVERSIBLY GLYCOSYLATED POLYPEPTIDE 2); alpha-1,4-gluc	
EE467670	3.184	moderately similar to (256)AT5G48380| Symbols: | leucine-rich repeat family protein / protein kinase family protein | chr5:19621810-196	-3.233
JCVI_10029	3.183	moderately similar to (457)AT3G13860| Symbols: | chaperonin, putative | chr3:4561711-4565140 REVERSEmoderately similar to (376)(	
CD828780	3.182	moderately similar to (219)AT5G47400| Symbols: | similar to hypothetical protein 25.t00068 [Brassica oleracea] (GB:ABD64961.1); simi	
JCVI_18753	3.182	weakly similar to (169)AT2G28060| Symbols: | protein kinase-related | chr2:11957251-11957695 REVERSE no original description	
JCVI_12384	3.182	weakly similar to (108)AT3G02885| Symbols: GASA5 | GASA5 (GAST1 PROTEIN HOMOLOG 5) | chr3:638330-639018 REVERSE no	-2.297
JCVI_27014	3.181	highly similar to (588)AT3G16150| Symbols: | L-asparaginase, putative / L-asparagine amidohydrolase, putative | chr3:5471800-5473039	
JCVI_27461	3.180	moderately similar to (226)AT4G01370| Symbols: MPK4, ATMPK4 | ATMPK4 (MAP KINASE 4); MAP kinase/ kinase | chr4:567219-56	
ES928565	3.179	no similarity	
JCVI_25800	3.179	weakly similar to (169)AT2G01150| Symbols: RHA2B | RHA2B (RING-H2 FINGER PROTEIN 2B); protein binding / zinc ion binding | c	
EX127222	3.178	moderately similar to (214)AT5G60390| Symbols: | elongation factor 1-alpha / EF-1-alpha | chr5:24306452-24307901 FORWARDmodera	
EG019832	3.177	weakly similar to (118)AT2G33070| Symbols: | jacalin lectin family protein | chr2:14036427-14038011 REVERSE [20440]	
JCVI_10983	3.177	moderately similar to (454)AT4G00370| Symbols: ANTR2 | ANTR2 (anion transporter 2); organic anion transmembrane transporter | chr4	
JCVI_30617	3.175	moderately similar to (290)AT2G25620| Symbols: | protein phosphatase 2C, putative / PP2C, putative | chr2:10910232-10912056 REVER	
EX043682	3.174	moderately similar to (312)AT4G21570| Symbols: | similar to unknown protein [Arabidopsis thaliana] (TAIR:AT1G11200.1); similar to h	
JCVI_16804	3.174	moderately similar to (308)AT3G50620| Symbols: | nodulation protein-related | chr3:18795974-18797728 REVERSE no original descripti	
JCVI_33723	3.172	highly similar to (521)AT5G16370| Symbols: | AMP-binding protein, putative | chr5:5356826-5358484 REVERSE no original description	
JCVI_5259	3.171	moderately similar to (442)AT2G45300| Symbols: | 3-phosphoshikimate 1-carboxyvinyltransferase / 5-enolpyruvylshikimate-3-phosphate	
JCVI_3631	3.169	moderately similar to (320)AT5G25760| Symbols: UBC21, PEX4 | PEX4 (PEROXIN4); ubiquitin-protein ligase | chr5:8967986-8969176	
EV119139	3.169	moderately similar to (392)AT5G35160| Symbols: | endomembrane protein 70, putative | chr5:13432175-13434151 FORWARD [21479]	
JCVI_212	3.168	moderately similar to (429)AT2G25450| Symbols: | 2-oxoglutarate-dependent dioxygenase, putative | chr2:10837364-10838641 REVERS	
JCVI_17129	3.168	weakly similar to (192)AT4G29960| Symbols: | similar to unnamed protein product [Vitis vinifera] (GB:CAO15556.1) | chr4:14660759-14	
JCVI_26765	3.167	moderately similar to (375)AT5G21050| Symbols: | similar to unknown protein [Arabidopsis thaliana] (TAIR:AT5G64090.1); similar to u	
JCVI_6624	3.167	moderately similar to (335)AT3G07870| Symbols: | F-box family protein | chr3:2510877-2512130 FORWARD no original description	
JCVI_18490	3.166	very weakly similar to (88.2)AT5G54970| Symbols: | similar to unknown protein [Arabidopsis thaliana] (TAIR:AT4G26960.1) | chr5:2233	
JCVI_29877	3.166	weakly similar to (187)AT2G21190| Symbols: | ER lumen protein retaining receptor family protein | chr2:9087997-9089723 FORWARD	
JCVI_22477	3.166	moderately similar to (347)AT1G12550| Symbols: | oxidoreductase family protein | chr1:4274647-4275829 FORWARD no original descri	
EV174588	3.164	weakly similar to (151)AT2G39730| Symbols: RCA | RCA (RUBISCO ACTIVASE) | chr2:16578252-16580423 REVERSEweakly similar	
JCVI_15168	3.163	moderately similar to (432)AT5G04520| Symbols: | oxidoreductase/ transition metal ion binding | chr5:1290013-1291064 REVERSE no o	
JCVI_15899	3.163	moderately similar to (376)AT2G30050| Symbols: | transducin family protein / WD-40 repeat family protein | chr2:12832617-12833525 F	-3.319
EE416388	3.162	moderately similar to (305)AT1G75140| Symbols: | Identical to Uncharacterized membrane protein At1g75140 [Arabidopsis Thaliana] (G	
JCVI_27515	3.162	moderately similar to (325)AT2G32020| Symbols: | GCN5-related N-acetyltransferase (GNAT) family protein | chr2:13638270-13638821	
EV184224	3.160	weakly similar to (132)AT2G45960| Symbols: TMP-A, ATHH2, PIP1;2, PIP1B | PIP1B (plasma membrane intrinsic protein 1;2) | chr2:18	
JCVI_30218	3.160	no original description	
JCVI_369	3.160	highly similar to (528)AT2G28380| Symbols: DRB2 | DRB2 (DSRNA-BINDING PROTEIN 2); double-stranded RNA binding | chr2:1214	
ES904578	3.160	moderately similar to (218)AT4G38790| Symbols: | ER lumen protein retaining receptor family protein | chr4:18111420-18112971 FORW	
JCVI_21741	3.160	highly similar to (654)AT5G22510| Symbols: | beta-fructofuranosidase, putative / invertase, putative / saccharase, putative / beta-fructosic	
JCVI_31756	3.159	very weakly similar to (97.1)AT4G14030| Symbols: SBP1 | selenium-binding protein, putative | chr4:8098116-8100160 REVERSE no origi	1.954
JCVI_737	3.159	moderately similar to (313)AT3G30280| Symbols: | transferase family protein | chr3:11916845-11918176 FORWARD no original descript	
JCVI_28281	3.158	moderately similar to (397)AT5G44320| Symbols: | eukaryotic translation initiation factor 3 subunit 7, putative / eIF-3 zeta, putative / eIF	
CV432550	3.158	no similarity	
JCVI_263	3.157	moderately similar to (460)AT5G58770| Symbols: | dehydrodolichyl diphosphate synthase, putative / DEDOL-PP synthase, putative | chr5	2.931
JCVI_13533	3.156	moderately similar to (350)AT5G27830| Symbols: | similar to hypothetical protein [Vitis vinifera] (GB:CAN74239.1) | chr5:9861356-986	
JCVI_225	3.156	moderately similar to (483)AT4G39330| Symbols: | mannitol dehydrogenase, putative | chr4:18291262-18292734 FORWARDmoderately	
EX078051	3.156	no similarity	
EV108354	3.156	weakly similar to (141)AT2G36780| Symbols: | UDP-glucoronosyl/UDP-glucosyl transferase family protein | chr2:15424697-15426187 R	
ES941013	3.155	moderately similar to (282)AT3G53750| Symbols: ACT3 | ACT3 (ACTIN 3); structural constituent of cytoskeleton | chr3:19926902-1992	
ES959658	3.154	moderately similar to (457)AT4G17260| Symbols: | L-lactate dehydrogenase, putative | chr4:9674070-9675322 FORWARDmoderately sir	
JCVI_964	3.154	moderately similar to (322)AT5G14040| Symbols: | mitochondrial phosphate transporter | chr5:4531061-4532967 REVERSE no original c	
JCVI_33692	3.153	moderately similar to (327)AT1G69370| Symbols: CM-3, CM3 | CM3 (CHORISMATE MUTASE 3); chorismate mutase | chr1:26083761-	
EE392264	3.152	no similarity	
ES938111	3.152	moderately similar to (259)AT4G36680| Symbols: | pentatricopeptide (PPR) repeat-containing protein | chr4:17292483-17293721 REVEF	
JCVI_32074	3.151	highly similar to (503)AT1G23880| Symbols: | NHL repeat-containing protein | chr1:8436114-8438625 FORWARD no original descriptio	
JCVI_40506	3.150	weakly similar to (148)AT1G64890| Symbols: | integral membrane transporter family protein | chr1:24113415-24114828 FORWARD no c	-1.575
AM387567	3.149	weakly similar to (125)AT5G49360| Symbols: ATBXL1, BXL1 | BXL1 (BETA-XYLOSIDASE 1); hydrolase, hydrolyzing O-glycosyl com	
JCVI_32843	3.149	weakly similar to (115)AT2G41190| Symbols: | amino acid transporter family protein | chr2:17174639-17177223 REVERSE no original d	2.487
AM394162	3.149	moderately similar to (249)AT2G20320| Symbols: | DENN (AEX-3) domain-containing protein | chr2:8774187-8779217 FORWARD [20	
JCVI_10069	3.148	moderately similar to (315)AT5G19980| Symbols: | integral membrane family protein | chr5:6749909-6750934 REVERSE no original des	
JCVI_38388	3.148	weakly similar to (128)AT2G03600| Symbols: ATUPS3 | ATUPS3 (Arabidopsis thaliana ureide permease 3) | chr2:1097606-1098364 FOR	
JCVI_22830	3.148	weakly similar to (168)AT2G34340| Symbols: | similar to unknown protein [Arabidopsis thaliana] (TAIR:AT1G29640.1); similar to unkno	
EE560072	3.148	moderately similar to (204)AT1G11780| Symbols: | oxidoreductase, 2OG-Fe(II) oxygenase family protein | chr1:3977613-3979176 REVE	
JCVI_33256	3.148	moderately similar to (364)AT1G67410| Symbols: | exostosin family protein | chr1:25255269-25257231 REVERSE no original descriptio	
BG544873	3.147	very weakly similar to (90.5)AT1G66580| Symbols: | 60S ribosomal protein L10 (RPL10C) | chr1:24842871-24844102 FORWARDvery w	
JCVI_28197	3.146	moderately similar to (311)AT5G63890| Symbols: ATHDH | ATHDH (HISTIDINOL DEHYDROGENASE) | chr5:25582826-25585330 R	
JCVI_2577	3.145	moderately similar to (264)AT5G05270| Symbols: | chalcone-flavanone isomerase family protein | chr5:1563544-1564828 FORWARD no	
EX134529	3.145	no similarity	
JCVI_41926	3.145	moderately similar to (486)AT5G35970| Symbols: | DNA-binding protein, putative | chr5:14136290-14140308 REVERSE no original desc	
JCVI_25645	3.144	moderately similar to (291)AT2G36750| Symbols: UGT72C1 | UGT72C1 (UDP-GLUCOSYL TRANSFERASE 72C1); UDP-glycosyltrans	
JCVI_4179	3.143	moderately similar to (422)AT5G46180| Symbols: delta-OAT | delta-OAT (ornithine- delta-aminotransferase); ornithine-oxo-acid transami	
AM389957	3.140	moderately similar to (261)AT3G27380| Symbols: SDH2-1 | SDH2-1 (succinate dehydrogenase 2-1) | chr3:10132446-10133910 REVERSI	
JCVI_32521	3.140	moderately similar to (385)AT1G75760| Symbols: | ER lumen protein retaining receptor family protein | chr1:28450601-28452082 REVE	
JCVI_4760	3.139	moderately similar to (373)AT3G23600| Symbols: | dienelactone hydrolase family protein | chr3:8473840-8475662 FORWARDweakly sir	
EX088011	3.139	no similarity	
JCVI_18140	3.138	no original description	
ES264223	3.136	moderately similar to (307)AT1G06210| Symbols: | VHS domain-containing protein / GAT domain-containing protein | chr1:1897566-189	
EX030558	3.135	weakly similar to (194)AT1G14370| Symbols: APK2A | APK2A (PROTEIN KINASE 2A); kinase | chr1:4915854-4917954 FORWARD [2	
JCVI_2145	3.131	moderately similar to (407)AT4G30810| Symbols: SCPL29 | SCPL29 (serine carboxypeptidase-like 29); serine carboxypeptidase | chr4:15	
JCVI_38130	3.131	no original description	

JCVI_3370	3.131	moderately similar to (305)AT5G39250| Symbols: | F-box family protein | chr5:15742088-15742846 FORWARD no original description	
JCVI_28295	3.130	moderately similar to (341)AT5G64370| Symbols: BETA-UP | BETA-UP (BETA-UREIDOPROPIONASE); beta-ureidopropionase | chr5:	
AM391990	3.129	moderately similar to (266)AT2G43330| Symbols: ATINT1 | ATINT1 (INOSITOL TRANSPORTER 1); carbohydrate transmembrane tran	
EE439403	3.129	weakly similar to (122)AT3G11510| Symbols: | 40S ribosomal protein S14 (RPS14B) | chr3:3623763-3624872 REVERSEweakly similar t	
JCVI_22482	3.126	moderately similar to (347)AT2G37760| Symbols: | aldo/keto reductase family protein | chr2:15839073-15840756 FORWARDweakly sim	
JCVI_15654	3.126	moderately similar to (263)AT2G35410| Symbols: | 33 kDa ribonucleoprotein, chloroplast, putative / RNA-binding protein cp33, putative	
JCVI_7538	3.126	moderately similar to (209)AT5G54940| Symbols: | eukaryotic translation initiation factor SUI1, putative | chr5:22325646-22325984 REV	
JCVI_196	3.125	moderately similar to (306)AT5G03880| Symbols: | similar to unknown protein [Arabidopsis thaliana] (TAIR:AT4G10000.2); similar to u	-2.138
JCVI_3334	3.123	moderately similar to (325)AT1G70980| Symbols: SYNC3 | SYNC3; ATP binding / aminoacyl-tRNA ligase/ asparagine-tRNA ligase/ aspa	
JCVI_399	3.123	moderately similar to (432)AT4G21990| Symbols: PRH-26, PRH26, ATAPR3, APR3 | APR3 (APS REDUCTASE 3) | chr4:11657296-116	
CV546570	3.123	weakly similar to (107)AT1G69920| Symbols: ATGSTU12 | ATGSTU12 (Arabidopsis thaliana Glutathione S-transferase (class tau) 12); g	
JCVI_24781	3.123	moderately similar to (249)AT3G11260| Symbols: WOX5 | WOX5 (WUSCHEL-related homeobox 5); transcription factor | chr3:3527612-	
JCVI_13368	3.123	moderately similar to (291)AT1G73260| Symbols: | trypsin and protease inhibitor family protein / Kunitz family protein | chr1:27551071-2	
EX055765	3.121	very weakly similar to (97.1)AT1G80510| Symbols: | amino acid transporter family protein | chr1:30277992-30279461 FORWARD [21813	
JCVI_4510	3.121	moderately similar to (342)AT1G69390| Symbols: ATMINE1 | ATMINE1 (ARABIDOPSIS HOMOLOGUE OF BACTERIAL MINE 1); p	
EV061160	3.120	moderately similar to (348)AT5G20960| Symbols: AO1, ATAO, ATAO1, AAO1 | AAO1 (ALDEHYDE OXIDASE 1) | chr5:7116785-712	
JCVI_3365	3.119	moderately similar to (422)AT4G38600| Symbols: UPL3, KAK | KAK (KAKTUS) | chr4:18041497-18049286 REVERSE no original desc	
JCVI_2621	3.117	moderately similar to (498)AT5G54080| Symbols: HGO | HGO (HOMOGENTISATE 1,2-DIOXYGENASE); homogentisate 1,2-dioxygen	
EX135525	3.117	moderately similar to (235)AT4G18430| Symbols: AtRABA1e | AtRABA1e (Arabidopsis Rab GTPase homolog A1e); GTP binding | chr4:	
JCVI_31333	3.115	very weakly similar to (86.7)AT3G51990| Symbols: | protein kinase family protein | chr3:19298967-19300055 FORWARD no original desc	
JCVI_211	3.113	highly similar to (528)AT5G44720| Symbols: | molybdenum cofactor sulfurase family protein | chr5:18060313-18062502 FORWARDvery	-1.461
EE542585	3.113	no similarity	
EV164093	3.113	moderately similar to (331)AT5G10180| Symbols: SULTR2;1, AST68 | AST68 (Sulfate transporter 2.1) | chr5:3193226-3196819 FORWA	
JCVI_6326	3.111	highly similar to (739)AT2G20580| Symbols: RPN1A, AtRPN1a | AtRPN1a/RPN1A (26S proteasome regulatory subunit S2 1A); binding	
JCVI_16591	3.111	moderately similar to (343)AT1G34750| Symbols: | protein phosphatase 2C, putative / PP2C, putative | chr1:12736364-12737705 REVER	
CD824533	3.110	moderately similar to (382)AT1G09350| Symbols: ATGOLS3 | ATGOLS3 (ARABIDOPSIS THALIANA GALACTINOL SYNTHASE 3);	-1.337
JCVI_9741	3.109	highly similar to (714)AT2G43790| Symbols: MPK6, MAPK6, ATMPK6 | ATMPK6 (MAP KINASE 6); MAP kinase/ kinase | chr2:18145	
EV055366	3.108	weakly similar to (105)AT1G01380| Symbols: ETC1 | ETC1 (ENHANCER OF TRY AND CPC 1); DNA binding / transcription factor | ch	
JCVI_41905	3.106	moderately similar to (235)AT4G39940| Symbols: AKN2 | AKN2 (APS-KINASE 2); ATP binding / kinase/ transferase, transferring phosp	
EX117276	3.105	weakly similar to (165)AT1G19200| Symbols: | senescence-associated protein-related | chr1:6625095-6625847 REVERSE [21828]	2.023
ES930665	3.104	no similarity	
ES901208	3.102	no similarity	-1.783
JCVI_13681	3.101	moderately similar to (219)AT1G78340| Symbols: ATGSTU22 | ATGSTU22 (Arabidopsis thaliana Glutathione S-transferase (class tau) 2	
EE439056	3.101	weakly similar to (129)AT5G19780| Symbols: TUA5 | TUA5 (tubulin alpha-5) | chr5:6687214-6688928 FORWARDweakly similar to (12	
EV193181	3.100	moderately similar to (257)AT4G18210| Symbols: ATPUP10 | ATPUP10 (Arabidopsis thaliana purine permease 10); purine transmembra	
ES938920	3.099	no similarity	
DY026572	3.099	very weakly similar to (87.8)AT3G07680| Symbols: | emp24/gp25L/p24 family protein | chr3:2455633-2456658 FORWARD [18978]	
EX017039	3.099	weakly similar to (148)AT1G80790| Symbols: | XH/XS domain-containing protein / XS zinc finger domain-containing protein | chr1:3036	
JCVI_34541	3.098	moderately similar to (455)AT3G25020| Symbols: | disease resistance family protein | chr3:9116875-9119547 REVERSEweakly similar to	
EV037870	3.098	moderately similar to (214)AT4G03020| Symbols: | transducin family protein / WD-40 repeat family protein | chr4:1331704-1334472 REV	-3.503
JCVI_8561	3.097	moderately similar to (460)AT1G72770| Symbols: HAB1 | HAB1 (HOMOLOGY TO ABI1) | chr1:27394660-27396075 FORWARD no or	
JCVI_56	3.096	highly similar to (523)AT4G13940| Symbols: EMB1395, MEE58, SAHH, SAHH1, HOG1 | HOG1 (HOMOLOGY-DEPENDENT GENE S	
JCVI_35964	3.096	very weakly similar to (89.4)AT2G39805| Symbols: | integral membrane Yip1 family protein | chr2:16617294-16619431 REVERSE no ori	
JCVI_13234	3.095	moderately similar to (380)AT4G25680| Symbols: | similar to unknown protein [Arabidopsis thaliana] (TAIR:AT4G25660.1); similar to u	-1.883
DN962602	3.095	moderately similar to (234)AT5G38430| Symbols: | ribulose bisphosphate carboxylase small chain 1B / RuBisCO small subunit 1B (RBCS	
EE568090	3.095	moderately similar to (258)AT1G04180| Symbols: | flavin-containing monooxygenase family protein / FMO family protein | chr1:1104622	-4.481
JCVI_14139	3.094	moderately similar to (448)AT1G44820| Symbols: | aminoacylase, putative / N-acyl-L-amino-acid amidohydrolase, putative | chr1:169287	
JCVI_4992	3.094	moderately similar to (371)AT5G22300| Symbols: NIT4 | NIT4 (NITRILASE 4) | chr5:7379404-7381767 FORWARDmoderately similar t	
EE534598	3.091	weakly similar to (107)AT3G13050| Symbols: | transporter-related | chr3:4176873-4178875 FORWARD [20150]	
EX042932	3.091	weakly similar to (166)AT5G48930| Symbols: HCT | transferase family protein | chr5:19853880-19855318 REVERSE [21811]	
JCVI_23492	3.090	highly similar to (518)AT4G34740| Symbols: CIA1, ATPURF2, ATD2, ATASE2 | ATASE2 (GLN PHOSPHORIBOSYL PYROPHOSPH	
ES911771	3.089	weakly similar to (102)AT1G64405| Symbols: | unknown protein | chr1:23927463-23927819 FORWARD [21431] 1 383 459	
EG020408	3.088	moderately similar to (407)AT1G65070| Symbols: | DNA mismatch repair MutS family protein | chr1:24176795-24179907 REVERSE [20	
JCVI_5481	3.086	moderately similar to (492)AT3G24170| Symbols: ATGR1 | ATGR1 (GLUTATHIONE-DISULFIDE REDUCTASE) | chr3:8729769-8734	
EX098712	3.085	very weakly similar to (89.4)AT3G02885| Symbols: GASA5 | GASA5 (GAST1 PROTEIN HOMOLOG 5) | chr3:638330-639018 REVERS	-1.559
JCVI_14056	3.085	moderately similar to (350)AT5G17520| Symbols: MEX1, RCP1 | RCP1 (ROOT CAP 1) | chr5:5772798-5775233 REVERSEmoderately s	
EV048869	3.084	moderately similar to (251)AT1G68260| Symbols: | thioesterase family protein | chr1:25589639-25591264 REVERSE [21442]	
JCVI_15496	3.084	moderately similar to (231)AT5G47180| Symbols: | vesicle-associated membrane family protein / VAMP family protein | chr5:19178611-1	
CB331870	3.083	no similarity	-1.586
JCVI_10849	3.083	moderately similar to (338)AT3G03910| Symbols: | glutamate dehydrogenase, putative | chr3:1006920-1008853 FORWARDmoderately si	
AM395628	3.082	moderately similar to (242)AT1G66620| Symbols: | seven in absentia (SINA) protein, putative | chr1:24856469-24857707 REVERSE [20	
EE450891	3.082	no similarity	
JCVI_30794	3.080	weakly similar to (153)AT1G19050| Symbols: ARR7 | ARR7 (RESPONSE REGULATOR 7); transcription regulator/ two-component resp	
JCVI_6509	3.079	moderately similar to (286)AT5G38660| Symbols: APE1 | APE1 (ACCLIMATION OF PHOTOSYNTHESIS TO ENVIRONMENT) | chr5	
JCVI_2809	3.076	moderately similar to (456)AT4G22530| Symbols: | embryo-abundant protein-related | chr4:11859258-11860140 REVERSE no original de	
JCVI_27231	3.075	moderately similar to (260)AT1G71015| Symbols: | similar to unknown protein [Arabidopsis thaliana] (TAIR:AT2G01340.1); similar to u	
DN961931	3.075	moderately similar to (369)AT2G45510| Symbols: CYP704A2 | CYP704A2 (cytochrome P450, family 704, subfamily A, polypeptide 2); c	
JCVI_18043	3.075	weakly similar to (135)AT1G63540| Symbols: | hydroxyproline-rich glycoprotein family protein | chr1:23571158-23573158 FORWARD n	
CX279159	3.074	moderately similar to (286)AT3G19490| Symbols: NHD1, ATNHD1 | ATNHD1 (Arabidopsis thaliana Na/H antiporter 1); sodium:hydroge	
JCVI_9402	3.074	weakly similar to (169)AT5G55280| Symbols: ATFTSZ1-1, CPFTSZ, FTSZ1-1 | FTSZ1-1 (FtsZ1-1); structural molecule | chr5:22437966-	
EE567168	3.073	no similarity	
EV169702	3.073	no similarity	-4.066
EX107275	3.073	moderately similar to (210)AT1G07210| Symbols: | 30S ribosomal protein S18 family | chr1:2215319-2216667 FORWARD [21827]	
EX088423	3.071	moderately similar to (261)AT1G26440| Symbols: ATUPS5 | ATUPS5 (ARABIDOPSIS THALIANA UREIDE PERMEASE 5) | chr1:914	
CV432242	3.071	very weakly similar to (85.9)AT5G63590| Symbols: FLS | FLS (Flavonol synthase); flavonol synthase | chr5:25474398-25475653 REVERS	
JCVI_40731	3.071	weakly similar to (130)AT5G25290| Symbols: | F-box family protein | chr5:8778595-8779788 FORWARD no original description	
JCVI_27283	3.070	moderately similar to (261)AT1G10270| Symbols: GRP23 | GRP23 (GLUTAMINE-RICH PROTEIN23); binding | chr1:3363537-3366278	
JCVI_4710	3.070	moderately similar to (222)AT3G21610| Symbols: | similar to unknown protein [Arabidopsis thaliana] (TAIR:AT1G67600.1); similar to u	
JCVI_2772	3.069	moderately similar to (265)AT5G65720| Symbols: ATNIFS1, NIFS1, NFS1, ATNFS1 | ATNFS1/ATNIFS1/NFS1/NIFS1 (ARABIOPSIS T	
EV144805	3.068	no similarity	
JCVI_533	3.068	moderately similar to (337)AT4G22920| Symbols: ATNYE1, NYE1 | ATNYE1/NYE1 (NON-YELLOWING 1) | chr4:12016787-12017980	1.093

ES959847	3.067	moderately similar to (348)AT4G30110| Symbols: HMA2 | HMA2 (Heavy metal ATPase 2); cadmium-transporting ATPase | chr4:147202	
JCVI_34823	3.066	highly similar to (530)AT5G25370| Symbols: PLDALPHA3 | PLDALPHA3 (PHOSPHLIPASE D ALPHA 3); phospholipase D | chr5:8804	
JCVI_29699	3.066	no original description	
JCVI_30859	3.065	weakly similar to (127)AT1G09070| Symbols: SRC2, (AT)SRC2 | (AT)SRC2/SRC2 (SOYBEAN GENE REGULATED BY COLD-2); pro	
EV007978	3.064	weakly similar to (189)AT4G04490| Symbols: | protein kinase family protein | chr4:2231955-2234636 REVERSEvery weakly similar to (9	-2.260
L33615	3.063	weakly similar to (177)AT5G48760| Symbols: | 60S ribosomal protein L13A (RPL13aD) | chr5:19788541-19789912 REVERSEweakly sir	
JCVI_232	3.063	moderately similar to (471)AT3G12610| Symbols: DRT100 | DRT100 (DNA-DAMAGE REPAIR/TOLERATION 100); protein binding | c	-1.964
JCVI_41907	3.063	highly similar to (538)AT5G51890| Symbols: | peroxidase | chr5:21108389-21109561 REVERSEmoderately similar to (241)PER1_ORY!	-1.668
JCVI_3802	3.062	weakly similar to (166)AT5G13440| Symbols: | ubiquinol-cytochrome C reductase iron-sulfur subunit, mitochondrial, putative / Rieske iro	
JCVI_10479	3.060	moderately similar to (244)AT5G41050| Symbols: | similar to unknown protein [Arabidopsis thaliana] (TAIR:AT3G26960.1); similar to u	-1.935
JCVI_16602	3.060	moderately similar to (302)AT5G64430| Symbols: | octicosapeptide/Phox/Bem1p (PB1) domain-containing protein | chr5:25779766-2578	
EV193675	3.059	moderately similar to (300)AT1G75760| Symbols: | ER lumen protein retaining receptor family protein | chr1:28450601-28452082 REVE	-2.331
EX043287	3.059	weakly similar to (131)AT5G13180| Symbols: ANAC083 | ANAC083 (Arabidopsis NAC domain containing protein 83); transcription fact	
JCVI_39277	3.056	weakly similar to (149)AT5G05540| Symbols: | exonuclease family protein | chr5:1636420-1638257 FORWARD no original description	
EX096295	3.055	no similarity	
JCVI_39107	3.054	moderately similar to (480)AT3G03440| Symbols: | armadillo/beta-catenin repeat family protein | chr3:815716-818575 FORWARD no ori	
EV179544	3.052	weakly similar to (145)AT1G77220| Symbols: | similar to unknown protein [Arabidopsis thaliana] (TAIR:AT4G38360.2); similar to unnar	
EV182009	3.052	moderately similar to (273)AT3G12920| Symbols: | protein binding / zinc ion binding | chr3:4122134-4123330 REVERSE [21487]	
JCVI_10526	3.052	moderately similar to (212)AT4G31450| Symbols: | zinc finger (C3HC4-type RING finger) family protein | chr4:15255961-15257983 REV	
DY005339	3.052	weakly similar to (130)AT2G34925| Symbols: CLE42 | CLE42 (CLAVATA3/ESR-RELATED 42) | chr2:14741349-14741615 FORWARD	
JCVI_23804	3.052	no original description	
EX047982	3.051	no similarity	
JCVI_3125	3.051	moderately similar to (464)AT3G50080| Symbols: VFB2 | VFB2 (VIER F-BOX PROTEINE 2); ubiquitin-protein ligase | chr3:18583771-1	
EV200765	3.051	moderately similar to (350)AT1G11860| Symbols: | aminomethyltransferase, putative | chr1:4001800-4003244 FORWARDmoderately sin	
JCVI_14632	3.051	moderately similar to (308)AT1G52630| Symbols: | similar to unknown protein [Arabidopsis thaliana] (TAIR:AT5G35570.1); similar to u	
JCVI_956	3.050	moderately similar to (437)AT1G10070| Symbols: ATBCAT-2 | ATBCAT-2; catalytic | chr1:3288674-3290166 FORWARD no original de	
JCVI_25644	3.050	moderately similar to (401)AT4G15960| Symbols: | epoxide hydrolase, putative | chr4:9045777-9047213 REVERSE no original descriptic	
BG543520	3.049	weakly similar to (102)AT1G64470| Symbols: | ubiquitin family protein | chr1:23948448-23949089 REVERSE [8791]	
DN962932	3.048	no similarity	
JCVI_6693	3.048	weakly similar to (182)AT5G32440| Symbols: | similar to unknown [Populus trichocarpa] (GB:ABK93674.1); contains InterPro domain U	
JCVI_9204	3.046	weakly similar to (150)AT3G16090| Symbols: | zinc finger (C3HC4-type RING finger) family protein | chr3:5456519-5458700 FORWAR	-2.964
JCVI_16911	3.045	moderately similar to (326)AT1G27420| Symbols: | kelch repeat-containing F-box family protein | chr1:9519095-9520507 FORWARD no	
JCVI_35916	3.044	no original description	
JCVI_30968	3.044	very weakly similar to (97.1)AT1G04985| Symbols: | similar to unnamed protein product [Vitis vinifera] (GB:CAO24071.1) | chr1:141656	
JCVI_13029	3.042	weakly similar to (119)AT5G42890| Symbols: | sterol carrier protein 2 (SCP-2) family protein | chr5:17211686-17213138 REVERSE no o	
EX138365	3.042	very weakly similar to (100)AT1G55300| Symbols: TAF7 | TAF7 (TBP-ASSOCIATED FACTOR 7); general RNA polymerase II transcrip	
JCVI_38953	3.042	very weakly similar to (89.7)AT1G05710| Symbols: | ethylene-responsive protein, putative | chr1:1716197-1717022 FORWARD no origina	
JCVI_27400	3.041	highly similar to (625)AT2G37710| Symbols: RLK | RLK (RECEPTOR LECTIN KINASE); kinase | chr2:15822012-15824039 REVERSE	
JCVI_19038	3.039	moderately similar to (412)AT3G08030| Symbols: | similar to unknown protein [Arabidopsis thaliana] (TAIR:AT2G41800.1); similar to u	
JCVI_14096	3.037	moderately similar to (387)AT1G21870| Symbols: | glucose-6-phosphate/phosphate translocator-related | chr1:7678197-7679686 FORWA	
CV546297	3.037	no similarity	
JCVI_26265	3.036	moderately similar to (367)AT1G11870| Symbols: OVA7, SRS, AtSRS | AtSRS (OVULE ABORTION 7); serine-tRNA ligase | chr1:40038	
EG020579	3.035	very weakly similar to (95.5)AT5G42790| Symbols: PAF1 | PAF1 (proteasome alpha subunit F1); peptidase | chr5:17176498-17178203 RE	
JCVI_15768	3.035	moderately similar to (404)AT1G67190| Symbols: | F-box family protein | chr1:25136717-25137976 FORWARD no original description	
EX098226	3.034	no similarity	
EV201070	3.034	moderately similar to (273)AT3G23120| Symbols: | leucine-rich repeat family protein | chr3:8227229-8229583 REVERSEweakly similar t	
JCVI_10412	3.033	moderately similar to (499)AT3G24430| Symbols: HCF101 | HCF101 (HIGH-CHLOROPHYLL-FLUORESCENCE 101); ATP binding | c	
EX041028	3.032	no similarity	-1.799
EX097006	3.031	moderately similar to (213)AT5G46800| Symbols: BOU | BOU (A BOUT DE SOUFFLE); binding | chr5:19006006-19007037 REVERSE	
CX195560	3.030	moderately similar to (217)AT1G17840| Symbols: WBC11, ABCG11, DSO, COF1 | ABCG11/COF1/DSO/WBC11 (DESPERADO); ATP	
JCVI_12689	3.030	moderately similar to (288)AT1G19800| Symbols: TGD1 | TGD1 (TRIGALACTOSYLDIACYLGLYCEROL 1) | chr1:6846803-6847945	
JCVI_9443	3.030	moderately similar to (392)AT4G36680| Symbols: | pentatricopeptide (PPR) repeat-containing protein | chr4:17292483-17293721 REVEF	-6.243
JCVI_14148	3.029	moderately similar to (316)AT1G68620| Symbols: | hydrolase | chr1:25769681-25770691 FORWARD no original description	
JCVI_6765	3.029	moderately similar to (348)AT5G37360| Symbols: | similar to unnamed protein product [Vitis vinifera] (GB:CAO38751.1) | chr5:1482266	
JCVI_17998	3.028	moderately similar to (311)AT5G61760| Symbols: IPK2B, ATIPK2BETA | ATIPK2BETA (Arabidopsis thaliana inositol hexakisphosphat	
JCVI_37161	3.028	moderately similar to (410)AT1G15520| Symbols: PDR12, ATPDR12 | ATPDR12/PDR12 (PLEIOTROPIC DRUG RESISTANCE 12); A	
BG543176	3.027	very weakly similar to (85.9)AT1G13440| Symbols: GAPC-2 | GAPC-2 | chr1:4608462-4610491 REVERSEvery weakly similar to (86.7)G:	-2.409
EE526847	3.026	very weakly similar to (83.2)AT1G35620| Symbols: ATPDIL5-2 | ATPDIL5-2 (PDI-LIKE 5-2); thiol-disulfide exchange intermediate | chr1	
EE417749	3.026	moderately similar to (333)AT5G60790| Symbols: ATGCN1 | ATGCN1 (Arabidopsis thaliana general control non-repressible 1) | chr5:24	-3.325
JCVI_11499	3.026	moderately similar to (278)AT5G64750| Symbols: ABR1 | ABR1 (ABA REPRESSOR1); DNA binding / transcription factor | chr5:259089	
JCVI_1703	3.024	moderately similar to (201)AT1G08630| Symbols: THA1 | THA1 (THREONINE ALDOLASE 1) | chr1:2743951-2745688 REVERSE no c	
JCVI_12096	3.023	moderately similar to (377)AT5G67370| Symbols: | similar to unknown protein [Arabidopsis thaliana] (TAIR:AT5G11840.1); similar to h	
JCVI_11240	3.023	moderately similar to (329)AT4G29690| Symbols: | type I phosphodiesterase/nucleotide pyrophosphatase family protein | chr4:14541090-	
JCVI_1967	3.023	moderately similar to (275)AT2G01110| Symbols: UNE3, PGA2, TATC, APG2 | APG2 (ALBINO AND PALE GREEN 2) | chr2:83786-8	
ES943377	3.022	no similarity	
JCVI_17459	3.021	weakly similar to (101)AT4G17670| Symbols: | senescence-associated protein-related | chr4:9833961-9834676 REVERSE no original des	-2.873
JCVI_20908	3.021	moderately similar to (377)AT1G61800| Symbols: GPT2 | GPT2 (glucose-6-phosphate/phosphate translocator 2); antiporter/ glucose-6-ph	
EV179235	3.021	weakly similar to (146)AT1G75050| Symbols: | similar to ATLP-3 (Arabidopsis thaumatin-like protein 3) [Arabidopsis thaliana] (TAIR:A	
JCVI_470	3.021	highly similar to (508)AT1G60810| Symbols: ACLA-2 | ACLA-2 (ATP-citrate lyase A-2) | chr1:22392356-22394658 REVERSE no origina	-3.354
JCVI_21423	3.021	weakly similar to (200)AT5G01850| Symbols: | protein kinase, putative | chr5:332826-334177 FORWARD no original description	
JCVI_363	3.020	moderately similar to (428)AT4G21210| Symbols: | Identical to Protein At4g21210 [Arabidopsis Thaliana] (GB:O49562); similar to unkn	
EV155072	3.020	weakly similar to (107)AT5G16960| Symbols: | NADP-dependent oxidoreductase, putative | chr5:5574540-5575887 REVERSE [21484] 1	
JCVI_39205	3.019	moderately similar to (214)AT3G10840| Symbols: | hydrolase, alpha/beta fold family protein | chr3:3391212-3393286 REVERSE no origi	
JCVI_24371	3.018	no original description	
JCVI_21352	3.017	moderately similar to (282)AT5G10130| Symbols: | pollen Ole e 1 allergen and extensin family protein | chr5:3171552-3172429 REVERS	
JCVI_36924	3.017	highly similar to (640)AT5G58230| Symbols: MEE70, MSI1 | MSI1 (MULTICOPY SUPRESSOR OF IRA1) | chr5:23573338-23575220 F	
JCVI_3693	3.016	moderately similar to (343)AT5G20910| Symbols: | zinc finger (C3HC4-type RING finger) family protein | chr5:7092665-7094312 REVE	
JCVI_9028	3.016	weakly similar to (197)AT1G30320| Symbols: | remorin family protein | chr1:10680330-10682834 FORWARD no original description	
EX021227	3.015	moderately similar to (232)AT5G06510| Symbols: | CCAAT-binding transcription factor (CBF-B/NF-YA) family protein | chr5:1985440-	
JCVI_4656	3.015	highly similar to (564)AT5G48300| Symbols: APS1, ADG1 | ADG1 (ADP GLUCOSE PYROPHOSPHORYLASE SMALL SUBUNIT 1);	
JCVI_8104	3.015	moderately similar to (367)AT5G30510| Symbols: ARRPS1, RPS1 | RPS1 (ribosomal protein S1); RNA binding | chr5:11636492-1163845	

CD833333	3.015	no similarity	
JCVI_2436	3.014	moderately similar to (377)AT1G54040| Symbols: ESR, TASTY, ESP | ESP (EPITHIOSPECIFIER PROTEIN) | chr1:20174663-2017755	
JCVI_7247	3.013	moderately similar to (267)AT2G41060| Symbols: | RNA recognition motif (RRM)-containing protein | chr2:17134216-17135571 FORW	
CX195912	3.013	no similarity	
JCVI_7318	3.012	weakly similar to (154)AT3G57680| Symbols: | peptidase S41 family protein | chr3:21392031-21394606 FORWARD no original descripti	
JCVI_9355	3.012	moderately similar to (353)AT1G15960| Symbols: ATNRAMP6, NRAMP6 | NRAMP6 (NRAMP metal ion transporter 6); metal ion trans	
EV100878	3.011	weakly similar to (134)AT5G46800| Symbols: BOU | BOU (A BOUT DE SOUFFLE); binding | chr5:19006006-19007037 REVERSE [214	
JCVI_28828	3.011	moderately similar to (308)AT5G19000| Symbols: ATBPM1 | ATBPM1 (BTB-POZ AND MATH DOMAIN 1); protein binding | chr5:634	
JCVI_9014	3.010	moderately similar to (229)AT4G36970| Symbols: | remorin family protein | chr4:17429821-17431454 REVERSE no original description	
JCVI_7738	3.010	moderately similar to (392)AT5G58220| Symbols: TTL | TTL (TRANSTHYRETIN-LIKE PROTEIN); steroid binding | chr5:23571772-23	
EE434577	3.010	weakly similar to (164)AT5G05330| Symbols: | DNA binding | chr5:1577290-1578600 REVERSE [15720]	
EE516446	3.009	weakly similar to (142)AT1G10790| Symbols: | similar to hydroxyproline-rich glycoprotein family protein [Arabidopsis thaliana] (TAIR:A	
EX086915	3.008	moderately similar to (441)AT1G72710| Symbols: CKL2 | CKL2; casein kinase I/ kinase | chr1:27376215-27379840 FORWARD [21823]	
JCVI_3698	3.008	moderately similar to (312)AT1G71840| Symbols: | transducin family protein / WD-40 repeat family protein | chr1:27026086-27028042 F	
JCVI_39952	3.008	no original description	
DY028533	3.007	no similarity	
EE413488	3.007	weakly similar to (191)AT1G80910| Symbols: | similar to unknown protein [Arabidopsis thaliana] (TAIR:AT1G16020.1); similar to unkn	
JCVI_12754	3.007	highly similar to (577)AT1G79600| Symbols: | ABC1 family protein | chr1:29954998-29957409 REVERSE no original description	
JCVI_5453	3.006	moderately similar to (304)AT5G11500| Symbols: | similar to unknown [Brassica juncea] (GB:ABX10747.1); contains InterPro domain P	
JCVI_2880	3.006	weakly similar to (179)AT1G54520| Symbols: | similar to unknown [Populus trichocarpa x Populus deltoides] (GB:ABK96363.1); contain	-3.448
JCVI_1849	3.005	moderately similar to (438)AT1G08200| Symbols: AXS2 | AXS2 (UDP-D-APIOSE/UDP-D-XYLOSE SYNTHASE 2) | chr1:2574256-257	
EE561736	3.004	no similarity	
JCVI_10359	3.004	moderately similar to (402)AT1G50430| Symbols: PA, LE, ST7R, 7RED, DWF5 | DWF5 (DWARF 5) | chr1:18685843-18689223 REVER	
EE505194	3.003	very weakly similar to (94.7)AT3G23800| Symbols: SBP3 | selenium-binding family protein | chr3:8581708-8583711 FORWARD [20139]	
JCVI_29565	3.003	weakly similar to (176)AT4G25880| Symbols: APUM6 | APUM6 (ARABIDOPSIS PUMILIO 6); RNA binding | chr4:13155527-1315907	
EV208956	2.998	very weakly similar to (87.0)AT3G12780| Symbols: PGK1 | PGK1 (PHOSPHOGLYCERATE KINASE 1); phosphoglycerate kinase | chr3:	
EV107181	2.998	moderately similar to (310)AT1G05560| Symbols: UGT75B1, UGT1 | UGT1 (UDP-glucosyl transferase 75B1); UDP-glycosyltransferase/	
JCVI_39885	2.995	moderately similar to (348)AT1G78920| Symbols: AVPL1, AVP2 | AVP2 (ARABIDOPSIS VACUOLAR H+-PYROPHOSPHATASE 2) |	
EE468644	2.995	very weakly similar to (87.0)AT3G04300| Symbols: | similar to unknown protein [Arabidopsis thaliana] (TAIR:AT4G10300.1); similar to	
JCVI_3852	2.994	moderately similar to (405)AT3G61140| Symbols: ATFUS6, CSN1, COP11, EMB78, FUS6 | FUS6 (FUSCA 6) | chr3:22637310-2263987	
JCVI_1838	2.994	highly similar to (533)AT1G15690| Symbols: ATAVP3, AVP-3, AVP1 | AVP1 (vacuolar-type H+-pumping pyrophosphatase 1) | chr1:539	
JCVI_37824	2.993	moderately similar to (389)AT1G31814| Symbols: FRL2 | FRL2 (FRIGIDA LIKE 2) | chr1:11412966-11414387 REVERSE no original de	
EX070106	2.993	moderately similar to (206)AT5G45900| Symbols: ATAPG7, ATG7, APG7 | APG7 (AUTOPHAGY 7) | chr5:18632531-18635663 FORW	
JCVI_8816	2.992	moderately similar to (459)AT3G14850| Symbols: | similar to unknown protein [Arabidopsis thaliana] (TAIR:AT1G29050.1); similar to u	
EV084776	2.992	weakly similar to (161)AT1G02930| Symbols: GST1, ERD11, ATGSTF3, GSTF6, ATGSTF6 | ATGSTF6 (EARLY RESPONSIVE TO DE	
JCVI_5577	2.991	moderately similar to (307)AT1G51730| Symbols: | RWD domain-containing protein | chr1:19190480-19192306 REVERSE no original de	
JCVI_2414	2.991	moderately similar to (441)AT5G13930| Symbols: CHS, TT4, ATCHS | ATCHS/CHS/TT4 (CHALCONE SYNTHASE); naringenin-chalc	
JCVI_27434	2.990	highly similar to (688)AT4G25450| Symbols: ATNAP8 | ATNAP8 (Arabidopsis thaliana non-intrinsic ABC protein 8) | chr4:13009855-13	
JCVI_15495	2.990	moderately similar to (381)AT1G63120| Symbols: ATRBL2 | ATRBL2 (ARABIDOPSIS THALIANA RHOMBOID-LIKE 2); serine-type	
BG544183	2.990	no similarity	
JCVI_20230	2.989	moderately similar to (340)AT2G22290| Symbols: AtRABH1d | AtRABH1d (Arabidopsis Rab GTPase homolog H1d); GTP binding | chr2	
JCVI_26547	2.989	moderately similar to (402)AT1G71170| Symbols: | 6-phosphogluconate dehydrogenase NAD-binding domain-containing protein | chr1:2	
CD822278	2.989	moderately similar to (246)AT1G23780| Symbols: | F-box family protein | chr1:8407042-8408469 REVERSE [13979]	
CD844359	2.987	moderately similar to (294)AT2G03360| Symbols: | similar to serine carboxypeptidase [Arabidopsis thaliana] (TAIR:AT2G03370.1); simi	
EV140916	2.987	very weakly similar to (91.7)AT5G53710| Symbols: | unknown protein | chr5:21822635-21822979 REVERSE [21482] 1 430 724	
JCVI_29478	2.987	moderately similar to (321)AT5G40020| Symbols: | pathogenesis-related thaumatin family protein | chr5:16040124-16041522 REVERSEv	-1.949
JCVI_12273	2.987	moderately similar to (299)AT2G47910| Symbols: CRR6 | CRR6 (CHLORORESPIRATORY REDUCTION 6) | chr2:19622033-1962262	
JCVI_8441	2.985	highly similar to (501)AT3G13920| Symbols: RH4, TIF4A1, EIF4A1 | EIF4A1 (eukaryotic translation initiation factor 4A-1) | chr3:459264	
JCVI_13588	2.984	moderately similar to (286)AT4G31450| Symbols: | zinc finger (C3HC4-type RING finger) family protein | chr4:15255961-15257983 RE	-1.491
JCVI_8988	2.984	moderately similar to (318)AT3G27090| Symbols: | similar to unknown protein [Arabidopsis thaliana] (TAIR:AT5G42050.1); similar to h	
JCVI_28451	2.983	very weakly similar to (82.8)AT5G47210| Symbols: | nuclear RNA-binding protein, putative | chr5:19186615-19188239 REVERSE no orig	
JCVI_32808	2.981	moderately similar to (268)AT5G54930| Symbols: | AT hook motif-containing protein | chr5:22323123-22324065 REVERSE no original d	
AM061351	2.980	moderately similar to (404)AT5G44820| Symbols: | similar to unknown protein [Arabidopsis thaliana] (TAIR:AT4G19970.1); similar to u	
JCVI_36779	2.980	weakly similar to (170)AT1G49660| Symbols: ATCXE5 | ATCXE5 (ARABIDOPSIS THALIANA CARBOXYESTERASE 5); carboxyles	-1.586
EV223761	2.980	no similarity	
CV544596	2.980	moderately similar to (228)AT2G17120| Symbols: LYM2 | LYM2 (LYSM DOMAIN GPI-ANCHORED PROTEIN 2 PRECURSOR) | chr	
JCVI_20942	2.980	very weakly similar to (83.6)AT5G11230| Symbols: | phosphate translocator-related | chr5:3580563-3581618 FORWARD no original desc	
CN729751	2.980	weakly similar to (115)AT1G02150| Symbols: | pentatricopeptide (PPR) repeat-containing protein | chr1:408779-410433 FORWARD [15	
EX097213	2.980	moderately similar to (305)AT4G23470| Symbols: | hydroxyproline-rich glycoprotein family protein | chr4:12249299-12251089 FORWA	-1.768
JCVI_30749	2.980	weakly similar to (135)AT5G08260| Symbols: SCPL35 | SCPL35 (serine carboxypeptidase-like 35); serine carboxypeptidase | chr5:265723	
JCVI_41914	2.980	moderately similar to (341)AT5G42420| Symbols: | transporter-related | chr5:16986047-16987453 FORWARD no original description	
JCVI_2146	2.980	moderately similar to (444)AT5G56280| Symbols: CSN6A | CSN6A (COP9 SIGNALOSOME SUBUNIT 6A) | chr5:22800843-22802756	
CX280463	2.978	moderately similar to (381)AT5G11330| Symbols: | monooxygenase family protein | chr5:3617343-3618862 REVERSE [16820]	
AM390427	2.977	moderately similar to (283)AT2G17120| Symbols: LYM2 | LYM2 (LYSM DOMAIN GPI-ANCHORED PROTEIN 2 PRECURSOR) | chr	
CX271981	2.977	no similarity	
EH416882	2.977	no similarity	
JCVI_35384	2.977	moderately similar to (274)AT3G19640| Symbols: | magnesium transporter CorA-like family protein (MRS2-3) | chr3:6820975-6823110 F	
EV011875	2.976	no similarity	
JCVI_19587	2.976	moderately similar to (363)AT1G66620| Symbols: | seven in absentia (SINA) protein, putative | chr1:24856469-24857707 REVERSEvery	-3.076
EX095196	2.975	weakly similar to (163)AT2G26030| Symbols: | F-box family protein | chr2:11098583-11100078 REVERSE [21824]	
JCVI_5076	2.974	moderately similar to (466)AT1G29700| Symbols: | similar to unnamed protein product [Vitis vinifera] (GB:CAO42101.1); contains doma	
EV213099	2.974	weakly similar to (187)AT3G08590| Symbols: | 2,3-biphosphoglycerate-independent phosphoglycerate mutase, putative / phosphoglycero	
EV048522	2.973	weakly similar to (124)AT4G03820| Symbols: | similar to unknown protein [Arabidopsis thaliana] (TAIR:AT4G22270.1); similar to unna	-3.498
JCVI_41039	2.973	weakly similar to (113)AT4G35440| Symbols: ATCLC-E, CLC-E | CLC-E (CHLORIDE CHANNEL E); voltage-gated chloride channel | c	
EV211396	2.973	weakly similar to (150)AT2G25070| Symbols: | protein phosphatase 2C, putative / PP2C, putative | chr2:10670596-10672445 REVERSE |	
JCVI_39023	2.973	weakly similar to (197)AT5G40680| Symbols: | kelch repeat-containing F-box family protein | chr5:16305982-16307229 FORWARD no o	
JCVI_13297	2.972	highly similar to (518)AT3G19760| Symbols: | eukaryotic translation initiation factor 4A, putative / eIF-4A, putative / DEAD box RNA he	
JCVI_4602	2.972	moderately similar to (255)AT4G37200| Symbols: HCF164 | HCF164 (High chlorophyll fluorescence 164); thiol-disulfide exchange intern	
AM059713	2.971	moderately similar to (214)AT1G56423| Symbols: | similar to unnamed protein product [Vitis vinifera] (GB:CAO17431.1) | chr1:2113274	
EH429905	2.970	moderately similar to (293)AT5G67360| Symbols: ARA12 | ARA12; subtilase | chr5:26889418-26891691 REVERSE [20767]	-2.594
ES944499	2.970	no similarity	

EG020082	2.970	no similarity	
JCVI_2438	2.970	moderately similar to (336)AT5G12040| Symbols: | carbon-nitrogen hydrolase family protein | chr5:3885163-3887773 FORWARD no ori	
JCVI_25824	2.969	moderately similar to (434)AT5G44050| Symbols: | MATE efflux family protein | chr5:17739711-17743436 FORWARD no original desc	
EE443692	2.969	moderately similar to (280)AT5G64500| Symbols: | membrane protein-related | chr5:25797737-25800700 FORWARD [20160]	
CN726483	2.968	no similarity	
JCVI_22402	2.968	moderately similar to (480)AT1G77510| Symbols: ATPDIL1-2 | ATPDIL1-2 (PDI-LIKE 1-2); protein disulfide isomerase | chr1:2913163	
AM392204	2.967	moderately similar to (207)AT2G02030| Symbols: | F-box family protein | chr2:482334-483828 FORWARD [20118]	
JCVI_3749	2.966	moderately similar to (345)AT3G04780| Symbols: | Encodes a protein with little sequence identity with any other protein of known structu	
JCVI_8397	2.965	moderately similar to (372)AT4G27760| Symbols: FEY3, FEY | FEY (FOREVER YOUNG); oxidoreductase | chr4:13844157-13846569 F	
EV110950	2.965	weakly similar to (146)AT1G12200| Symbols: | flavin-containing monooxygenase family protein / FMO family protein | chr1:4137625-41	
L38091	2.964	weakly similar to (105)AT1G68230| Symbols: | reticulon family protein (RTNLB14) | chr1:25575848-25576584 FORWARD [132]	
JCVI_13276	2.964	highly similar to (510)AT2G31110| Symbols: | similar to unknown protein [Arabidopsis thaliana] (TAIR:AT2G42570.1); similar to unnam	
EE560695	2.964	moderately similar to (207)AT5G61240| Symbols: | protein binding | chr5:24646711-24649184 FORWARD [20153] 21 435 435	
JCVI_4503	2.963	weakly similar to (148)AT5G06760| Symbols: | late embryogenesis abundant group 1 domain-containing protein / LEA group 1 domain-co	
JCVI_30418	2.963	moderately similar to (300)AT3G04790| Symbols: | ribose 5-phosphate isomerase-related | chr3:1313371-1314201 FORWARD no origina	
EE569999	2.963	weakly similar to (143)AT5G40390| Symbols: SIP1 | SIP1 (SEED IMBIBITION 1-LIKE); galactinol-sucrose galactosyltransferase/ hydrol	
AM057806	2.963	no similarity	
JCVI_13206	2.962	moderately similar to (481)AT2G45300| Symbols: | 3-phosphoshikimate 1-carboxyvinyltransferase / 5-enolpyruvylshikimate-3-phosphate	
JCVI_8618	2.961	moderately similar to (411)AT1G35720| Symbols: OXY5, ATOXY5, ANNAT1 | ANNAT1 (ANNEXIN ARABIDOPSIS 1); calcium ion b	
JCVI_26536	2.961	moderately similar to (273)AT1G10970| Symbols: ZIP4 | ZIP4 (ZINC TRANSPORTER 4 PRECURSOR); cation transmembrane transpor	
CD836376	2.961	moderately similar to (397)AT2G42120| Symbols: POLD2 | POLD2 (DNA POLYMERASE DELTA SMALL SUBUNIT); DNA binding /	
H07833	2.960	no similarity	
JCVI_5672	2.960	moderately similar to (365)AT1G68820| Symbols: | membrane protein, putative | chr1:25869515-25872463 FORWARD no original descri	
JCVI_19010	2.960	moderately similar to (341)AT1G26770| Symbols: AT-EXP10, ATEXP10, ATHEXP ALPHA 1.1, EXP10, ATEXPA10 | ATEXPA10 (AR	
JCVI_15083	2.958	moderately similar to (368)AT3G13410| Symbols: | similar to unknown protein [Arabidopsis thaliana] (TAIR:AT1G55546.1); similar to h	
DY020133	2.958	weakly similar to (196)AT3G62960| Symbols: | glutaredoxin family protein | chr3:23279755-23280063 FORWARD [18974] 1 603 621	
JCVI_20886	2.957	moderately similar to (496)AT5G10840| Symbols: | endomembrane protein 70, putative | chr5:3424911-3427798 REVERSE no original d	
JCVI_8236	2.957	moderately similar to (226)AT1G04530| Symbols: | binding | chr1:1234455-1235894 REVERSE no original description	
JCVI_3033	2.956	weakly similar to (174)AT1G69530| Symbols: EXP1, AT-EXP1, ATEXP1, ATHEXP ALPHA 1.2, ATEXPA1 | ATEXPA1 (ARABIDOPS	
EX106370	2.955	weakly similar to (188)AT1G12410| Symbols: CLPP2, NCLPP2, CLPR2 | CLPR2 (Clp protease proteolytic subunit 2); endopeptidase Clp	
JCVI_13028	2.954	moderately similar to (280)AT5G02820| Symbols: BIN5, RHL2 | RHL2 (ROOT HAIRLESS 2); ATP binding / DNA binding / DNA topois	
JCVI_3744	2.952	moderately similar to (452)AT2G34250| Symbols: | protein transport protein sec61, putative | chr2:14469714-14471651 FORWARD no o	
EX103900	2.952	moderately similar to (237)AT3G30390| Symbols: | amino acid transporter family protein | chr3:11979591-11981306 REVERSE [21826]	
EV037147	2.951	moderately similar to (224)AT5G10790| Symbols: UBP22 | UBP22 (UBIQUITIN-SPECIFIC PROTEASE 22); ubiquitin-specific protease	
JCVI_25189	2.950	moderately similar to (300)AT2G19450| Symbols: AS11, ABX45, DGAT1, RDS1, ATDGAT, TAG1 | TAG1 (TRIACYLGLYCEROL BIO	
EE564241	2.950	no similarity	
JCVI_1993	2.949	moderately similar to (407)AT4G12980| Symbols: | auxin-responsive protein, putative | chr4:7589667-7591071 REVERSE no original des	
EE419343	2.949	no similarity	
JCVI_8487	2.949	moderately similar to (399)AT5G04410| Symbols: ANAC078, NAC2 | NAC2 (Arabidopsis NAC domain containing protein 78); transcript	
CN729714	2.949	weakly similar to (172)AT2G21340| Symbols: | enhanced disease susceptibility protein, putative / salicylic acid induction deficient protein	
JCVI_6229	2.949	moderately similar to (345)AT4G17480| Symbols: | palmitoyl protein thioesterase family protein | chr4:9745325-9746859 REVERSE no c	
EV217100	2.948	moderately similar to (340)AT3G06620| Symbols: | protein kinase family protein | chr3:2062839-2067144 REVERSEvery weakly similar	
JCVI_9492	2.948	moderately similar to (366)AT5G16880| Symbols: | VHS domain-containing protein / GAT domain-containing protein | chr5:5549661-555	
JCVI_21394	2.946	weakly similar to (105)AT3G25585| Symbols: AAPT2 | AAPT2 (AMINOALCOHOLPHOSPHOTRANSFERASE); phosphatidyltransferas	
EV189237	2.946	weakly similar to (162)AT5G10770| Symbols: | chloroplast nucleoid DNA-binding protein, putative | chr5:3403332-3405332 REVERSE [	
CV432973	2.945	moderately similar to (269)AT1G20190| Symbols: EXP11, ATEXP11, ATHEXP ALPHA 1.14, ATEXPA11 | ATEXPA11 (ARABIDOPSI	
EE527978	2.944	weakly similar to (121)AT1G53320| Symbols: AtTLP7 | AtTLP7 (TUBBY LIKE PROTEIN 7); phosphoric diester hydrolase/ transcription	
JCVI_19087	2.944	moderately similar to (408)AT1G78770| Symbols: | cell division cycle family protein | chr1:29622314-29626166 FORWARD no original	
JCVI_1167	2.944	moderately similar to (381)AT1G67950| Symbols: | RNA recognition motif (RRM)-containing protein | chr1:25482547-25483735 REVER	
EX036932	2.942	weakly similar to (155)AT5G47880| Symbols: ERF1, ERF1-1 | ERF1-1 (EUKARYOTIC RELEASE FACTOR 1-1); translation release fac	
JCVI_9650	2.942	moderately similar to (280)AT1G69920| Symbols: ATGSTU12 | ATGSTU12 (Arabidopsis thaliana Glutathione S-transferase (class tau) 1	
JCVI_18084	2.942	moderately similar to (325)AT5G55130| Symbols: SIR1, CNX5 | CNX5 (SIRTINOL RESISTANT 1); Mo-molybdopterin cofactor sulfura	
JCVI_9666	2.942	no original description	
JCVI_3669	2.942	moderately similar to (333)AT4G03320| Symbols: TIC20-IV | TIC20-IV (TRANSLOCON AT THE INNER ENVELOPE MEMBRANE C	
JCVI_38990	2.941	weakly similar to (154)AT1G55900| Symbols: EMB1860, TIM50 | TIM50 (EMBRYO DEFECTIVE 1860) | chr1:20906828-20909085 FO	
JCVI_1175	2.939	moderately similar to (454)AT1G65660| Symbols: SMP1 | SMP1 (swellmap 1); nucleic acid binding | chr1:24421958-24424894 REVERS	-1.603
JCVI_24832	2.938	highly similar to (546)AT4G32320| Symbols: APX6 | APX6 (ASCORBATE PEROXIDASE 6); L-ascorbate peroxidase | chr4:15602783-1	
DN962440	2.938	moderately similar to (236)AT1G23090| Symbols: SULTR3;3, AST91 | AST91 (SULFATE TRANSPORTER 91); sulfate transmembrane	
EV109972	2.938	no similarity	
JCVI_19347	2.938	moderately similar to (402)AT4G16190| Symbols: | cysteine proteinase, putative | chr4:9171527-9172892 FORWARDmoderately similar	
JCVI_3679	2.938	moderately similar to (326)AT3G52460| Symbols: | hydroxyproline-rich glycoprotein family protein | chr3:19457948-19458850 FORWA	
CX195205	2.937	moderately similar to (244)AT5G42480| Symbols: ARC6 | ARC6 (ACCUMULATION AND REPLICATION OF CHLOROPLASTS 6) | c	
JCVI_11813	2.936	moderately similar to (483)AT5G13640| Symbols: ATPDAT | ATPDAT (Arabidopsis thaliana phospholipid:diacylglycerol acyltransferase	
EV214464	2.936	moderately similar to (342)AT3G48195| Symbols: | phox (PX) domain-containing protein | chr3:17839691-17843373 REVERSE [21491]	
CN729016	2.936	no similarity	
EE414030	2.936	moderately similar to (347)AT5G16370| Symbols: | AMP-binding protein, putative | chr5:5356826-5358484 REVERSE [20145]	-1.791
JCVI_10877	2.935	highly similar to (915)AT5G10840| Symbols: | endomembrane protein 70, putative | chr5:3424911-3427798 REVERSE no original descri	
JCVI_30685	2.935	moderately similar to (468)AT1G24610| Symbols: | SET domain-containing protein | chr1:8720892-8722697 REVERSE no original descr	
JCVI_488	2.934	moderately similar to (426)AT3G17390| Symbols: SAMS3, MAT4, MTO3 | MTO3 (S-adenosylmethionine synthase 3); methionine adeno	1.491
EX021647	2.934	moderately similar to (213)AT3G22890| Symbols: APS1 | APS1 (ATP sulfurylase 3) | chr3:8112844-8114741 FORWARD [21809]	
EV123005	2.934	very weakly similar to (84.3)AT1G51680| Symbols: 4CL.1, AT4CL1, 4CL1 | 4CL1 (4-COUMARATE:COA LIGASE 1) | chr1:19162748-1	
EE556478	2.934	weakly similar to (144)AT2G16405| Symbols: | transducin family protein / WD-40 repeat family protein | chr2:7112997-7115869 REVER	
ES901787	2.933	moderately similar to (479)AT1G11660| Symbols: | heat shock protein, putative | chr1:3921056-3924347 FORWARDweakly similar to (1	
JCVI_18092	2.933	highly similar to (930)AT5G63680| Symbols: | pyruvate kinase, putative | chr5:25507733-25509756 FORWARDhighly similar to (837)K	
JCVI_33918	2.932	moderately similar to (315)AT5G01220| Symbols: SQD2 | SQD2 (SULFOQUINOVOSYLDIACYLGLYCEROL 2); UDP-sulfoquinovose:	
EE460761	2.932	moderately similar to (343)AT2G40190| Symbols: | glycosyl transferase family 1 protein | chr2:16792310-16794335 FORWARD [20152]	
ES978070	2.932	no similarity	
JCVI_15838	2.932	moderately similar to (454)AT1G02090| Symbols: CSN7, COP15, CSN7II, FUS5 | FUS5 (FUSCA 5); MAP kinase kinase | chr1:388223-3	
JCVI_34836	2.931	highly similar to (838)AT2G24240| Symbols: | potassium channel tetramerisation domain-containing protein | chr2:10317918-10319243 F	-2.809
JCVI_16730	2.930	moderately similar to (226)AT5G17990| Symbols: PAT1, TRP1 | TRP1 (TRYPTOPHAN BIOSYNTHESIS 1); anthranilate phosphoribos	
EV089994	2.930	moderately similar to (260)AT4G12410| Symbols: | auxin-responsive family protein | chr4:7342953-7343426 REVERSE [21444]	

JCVI_25868	2.930	moderately similar to (433)AT5G22020| Symbols: | strictosidine synthase family protein | chr5:7287881-7289360 REVERSEweakly simil	
JCVI_3929	2.930	moderately similar to (334)AT3G48410| Symbols: | hydrolase, alpha/beta fold family protein | chr3:17936771-17939085 REVERSE no or	
EV175001	2.930	weakly similar to (151)AT5G03240| Symbols: UBQ3 | UBQ3 (POLYUBIQUITIN 3); protein binding | chr5:771975-772895 REVERSEwe	
EE564706	2.930	very weakly similar to (89.7)AT5G59480| Symbols: | haloacid dehalogenase-like hydrolase family protein | chr5:23996845-23998382 REV	
JCVI_33	2.929	highly similar to (545)AT2G20060| Symbols: | ribosomal protein L4 family protein | chr2:8666465-8668308 FORWARDvery weakly simi	
JCVI_192	2.929	moderately similar to (260)AT5G13490| Symbols: AAC2 | AAC2 (ADP/ATP CARRIER 2); binding | chr5:4336037-4337382 FORWARD	
JCVI_5757	2.929	moderately similar to (469)AT5G49730| Symbols: ATFRO6, FRO6 | ATFRO6/FRO6 (FERRIC REDUCTION OXIDASE 6); ferric-chelat	
JCVI_695	2.929	highly similar to (548)AT3G13920| Symbols: RH4, TIF4A1, EIF4A1 | EIF4A1 (eukaryotic translation initiation factor 4A-1) | chr3:459264	-2.991
JCVI_28039	2.929	moderately similar to (455)AT1G64680| Symbols: | similar to unknown protein [Arabidopsis thaliana] (TAIR:AT1G03055.1); similar to u	
RC_CB617675	2.929	no similarity	
JCVI_39138	2.929	weakly similar to (145)AT5G50760| Symbols: | auxin-responsive family protein | chr5:20662006-20662557 FORWARD no original descri	
EL588841	2.929	no similarity	
CD826897	2.928	weakly similar to (184)AT2G13650| Symbols: GONST1 | GONST1 (GOLGI NUCLEOTIDE SUGAR TRANSPORTER 1) | chr2:5695067	
EV110183	2.927	weakly similar to (167)AT1G59830| Symbols: PP2A-1 | PP2A-1 (protein phosphatase 2A-2); protein serine/threonine phosphatase | chr1:2	
BQ704516	2.926	weakly similar to (123)AT2G47880| Symbols: | glutaredoxin family protein | chr2:19612194-19612502 FORWARD [11009] 54 546 546	
JCVI_17997	2.926	moderately similar to (209)AT3G13680| Symbols: | F-box family protein | chr3:4477541-4478728 REVERSE no original description	
CV432364	2.926	no similarity	
CV432499	2.925	weakly similar to (149)AT1G52910| Symbols: | similar to unknown protein [Arabidopsis thaliana] (TAIR:AT3G15480.1); similar to unkn	
CV433816	2.925	moderately similar to (202)AT2G18915| Symbols: ADO2, LKP2 | LKP2 (LOV KELCH PROTEIN 2); ubiquitin-protein ligase | chr2:8201	
JCVI_4604	2.924	moderately similar to (399)AT3G05280| Symbols: | integral membrane Yip1 family protein | chr3:1503998-1505560 REVERSE no origin	
JCVI_34923	2.924	no original description	
EV033290	2.923	moderately similar to (207)AT1G19700| Symbols: BEL10, BLH10A | BEL10/BLH10A (BEL1-LIKE HOMEODOMAIN 10); DNA bindin	
JCVI_14539	2.923	moderately similar to (413)AT3G54820| Symbols: PIP2D, PIP2;5 | PIP2;5/PIP2D (plasma membrane intrinsic protein 2;5); water channel	
BQ704437	2.922	no similarity	
JCVI_13679	2.922	highly similar to (759)AT5G38530| Symbols: | tryptophan synthase-related | chr5:15441327-15443524 FORWARD no original descriptio	
JCVI_1256	2.922	moderately similar to (363)AT4G14110| Symbols: CSN8, FUS7, EMB143, COP9 | COP9 (CONSTITUTIVE PHOTOMORPHOGENIC 9)	-2.580
JCVI_14141	2.922	moderately similar to (297)AT5G64510| Symbols: | similar to unnamed protein product [Vitis vinifera] (GB:CAO49799.1) | chr5:2580181	
JCVI_27652	2.921	weakly similar to (152)AT1G21370| Symbols: | similar to unnamed protein product [Vitis vinifera] (GB:CAO60871.1) | chr1:7484239-748	
EV167409	2.921	moderately similar to (215)AT1G71800| Symbols: | cleavage stimulation factor, putative | chr1:27003268-27005512 FORWARD [21486]	
JCVI_2659	2.920	weakly similar to (197)AT1G72220| Symbols: | zinc finger (C3HC4-type RING finger) family protein | chr1:27188050-27189291 REVER	
JCVI_9430	2.920	moderately similar to (270)AT5G53030| Symbols: | similar to unknown protein [Arabidopsis thaliana] (TAIR:AT4G27810.1); similar to h	
JCVI_20670	2.920	weakly similar to (198)AT1G64385| Symbols: | similar to unnamed protein product [Vitis vinifera] (GB:CAO69552.1) | chr1:23903972-2	
EE454334	2.920	weakly similar to (184)AT2G35410| Symbols: | 33 kDa ribonucleoprotein, chloroplast, putative / RNA-binding protein cp33, putative | chr	
JCVI_11445	2.919	weakly similar to (135)AT3G21055| Symbols: PSBTN | PSBTN (photosystem II subunit T) | chr3:7376767-7377078 REVERSE no origina	-1.480
JCVI_36953	2.918	very weakly similar to (99.8)AT1G60950| Symbols: ATFD2, FED A | FED A (FERREDOXIN 2); 2 iron, 2 sulfur cluster binding / electron	
JCVI_17633	2.918	moderately similar to (364)AT1G10430| Symbols: PP2A-2 | PP2A-2 (protein phosphatase 2a-2); protein serine/threonine phosphatase | chr	
EE547452	2.917	no similarity	
JCVI_29141	2.916	moderately similar to (337)AT1G63110| Symbols: | cell division cycle protein-related | chr1:23408810-23411422 FORWARD no original	
JCVI_24271	2.916	weakly similar to (150)AT5G12250| Symbols: TUB6 | TUB6 (BETA-6 TUBULIN) | chr5:3961318-3962972 REVERSEweakly similar to (	
JCVI_20557	2.916	moderately similar to (381)AT5G53660| Symbols: AtGRF7 | AtGRF7 (GROWTH-REGULATING FACTOR 7) | chr5:21811862-2181315	-2.137
JCVI_16317	2.916	moderately similar to (343)AT4G29120| Symbols: | 6-phosphogluconate dehydrogenase NAD-binding domain-containing protein | chr4:1	
JCVI_1014	2.916	moderately similar to (257)AT3G48990| Symbols: | AMP-dependent synthetase and ligase family protein | chr3:18170016-18172279 REV	
EV113416	2.915	moderately similar to (290)AT3G16830| Symbols: TPR2 | TPR2 (TOPLESS-RELATED 2) | chr3:5731715-5737537 FORWARD [21479]	
JCVI_42068	2.915	weakly similar to (189)AT4G26580| Symbols: | protein binding / zinc ion binding | chr4:13411711-13412895 REVERSE no original descr	
JCVI_9004	2.915	weakly similar to (168)AT3G23400| Symbols: | plastid-lipid associated protein PAP / fibrillin family protein | chr3:8376643-8378232 REV	
JCVI_3607	2.913	moderately similar to (389)AT3G22630| Symbols: PRCGB, PBD1 | PBD1 (PROTEASOME SUBUNIT PRGB); peptidase | chr3:8009716-	
JCVI_40028	2.913	moderately similar to (491)AT1G77405| Symbols: | binding | chr1:29092039-29093415 FORWARD no original description	
JCVI_21131	2.912	highly similar to (593)AT3G55640| Symbols: | mitochondrial substrate carrier family protein | chr3:20651027-20653390 FORWARDweak	
JCVI_37658	2.911	very weakly similar to (81.3)AT1G32550| Symbols: | ferredoxin family protein | chr1:11771949-11774097 REVERSE no original descripti	
JCVI_41190	2.911	moderately similar to (274)AT5G48900| Symbols: | pectate lyase family protein | chr5:19842466-19846135 FORWARDweakly similar to	
JCVI_1556	2.911	moderately similar to (459)AT3G20820| Symbols: | leucine-rich repeat family protein | chr3:7280936-7282033 FORWARDweakly similar	
JCVI_21515	2.910	moderately similar to (251)AT2G30590| Symbols: WRKY21 | WRKY21 (WRKY DNA-binding protein 21); transcription factor | chr2:130	
EV210872	2.909	moderately similar to (312)AT1G21640| Symbols: ATNADK2, NADK2 | NADK2 (NAD KINASE 2); NAD+ kinase/ calmodulin binding |	
EV167296	2.909	no similarity	
JCVI_5517	2.909	moderately similar to (386)AT4G03250| Symbols: | homeobox-leucine zipper family protein | chr4:1425622-1427744 FORWARD no orig	-2.729
EV012279	2.908	no similarity	
EX017821	2.908	moderately similar to (350)AT1G79680| Symbols: | wall-associated kinase, putative | chr1:29985081-29987642 REVERSEweakly similar	
JCVI_14933	2.906	moderately similar to (455)AT2G36310| Symbols: | inosine-uridine preferring nucleoside hydrolase family protein | chr2:15231771-15233	
JCVI_8543	2.905	moderately similar to (440)AT1G17290| Symbols: ALAAT1 | ALAAT1 (ALANINE AMINOTRANSFERAS); alanine transaminase | chr1:	
EV185511	2.905	moderately similar to (366)AT1G32080| Symbols: | membrane protein, putative | chr1:11537552-11539736 REVERSE [21488]	
EX021381	2.905	very weakly similar to (90.9)AT1G07280| Symbols: | binding | chr1:2238504-2240990 FORWARD [21809]	
JCVI_11083	2.904	moderately similar to (337)AT4G15545| Symbols: | similar to unknown protein [Arabidopsis thaliana] (TAIR:AT1G16520.1); similar to u	
EV227478	2.904	moderately similar to (273)AT4G28660| Symbols: PSB28 | PSB28 (PHOTOSYSTEM II REACTION CENTER PSB28 PROTEIN) | chr4:	
JCVI_3994	2.904	moderately similar to (290)AT1G60170| Symbols: EMB1220 | EMB1220 (EMBRYO DEFECTIVE 1220) | chr1:22196673-22198842 FOR	
JCVI_30615	2.904	weakly similar to (176)AT3G62980| Symbols: TIR1 | TIR1 (TRANSPORT INHIBITOR RESPONSE 1); ubiquitin-protein ligase | chr3:23	
CX196105	2.904	moderately similar to (382)AT3G11950| Symbols: | homogentisate farnesyltransferase/ homogentisate geranylgeranyltransferase/ homoge	-4.117
JCVI_9052	2.903	moderately similar to (373)AT5G15440| Symbols: EDL1 | EDL1 (EID1-LIKE 1) | chr5:5013161-5014134 FORWARD no original descript	
JCVI_13894	2.903	moderately similar to (234)AT1G07280| Symbols: | binding | chr1:2238504-2240990 FORWARD no original description	
EV160033	2.902	moderately similar to (346)AT1G11600| Symbols: CYP77B1 | CYP77B1 (cytochrome P450, family 77, subfamily B, polypeptide 1); oxyg	-4.364
AM390211	2.902	moderately similar to (309)AT1G08920| Symbols: | sugar transporter, putative | chr1:2867449-2870193 FORWARD [20118]	
L38208	2.901	no similarity	
EG019713	2.901	weakly similar to (103)AT2G03690| Symbols: | coenzyme Q biosynthesis Coq4 family protein / ubiquinone biosynthesis Coq4 family prot	
CV432365	2.899	very weakly similar to (84.0)AT2G18770| Symbols: | signal recognition particle binding | chr2:8142066-8143442 FORWARD [16490] 30 4	
JCVI_30584	2.897	highly similar to (892)AT3G27020| Symbols: YSL6 | YSL6 (YELLOW STRIPE LIKE 6); oligopeptide transporter | chr3:9962860-996569	
AM386208	2.896	moderately similar to (315)AT3G07790| Symbols: | DGCR14-related | chr3:2487497-2489023 FORWARD [20118]	1.951
AT000817	2.896	no similarity	
AT000838	2.896	no similarity	
DY005491	2.895	no similarity	
JCVI_14967	2.895	moderately similar to (493)AT1G36370| Symbols: SHM7 | SHM7 (serine hydroxymethyltransferase 7); glycine hydroxymethyltransferase	
JCVI_20291	2.895	no original description	
JCVI_23683	2.894	moderately similar to (224)AT2G15530| Symbols: | zinc finger (C3HC4-type RING finger) family protein | chr2:6781232-6784150 FORW	

JCVI_10290	2.894	moderately similar to (421)AT1G32380| Symbols: | ribose-phosphate pyrophosphokinase 2 / phosphoribosyl diphosphate synthetase 2 (PF	
JCVI_26899	2.893	weakly similar to (193)AT1G69760| Symbols: | similar to unknown protein [Arabidopsis thaliana] (TAIR:AT1G26920.1); similar to hypot	
EE484523	2.893	weakly similar to (163)AT1G13830| Symbols: | beta-1,3-glucanase-related | chr1:4739996-4740923 REVERSE [20174]	
EX089759	2.892	moderately similar to (296)AT1G14360| Symbols: ATUTR3, UTR3 | ATUTR3/UTR3 (UDP-GALACTOSE TRANSPORTER 3); pyrimid	
JCVI_6512	2.892	very weakly similar to (92.8)AT1G30380| Symbols: PSAK | PSAK (PHOTOSYSTEM I SUBUNIT K) | chr1:10722307-10722995 FORWA	
JCVI_6474	2.892	moderately similar to (266)AT2G34460| Symbols: | flavin reductase-related | chr2:14536714-14537811 FORWARD no original descriptio	
JCVI_40007	2.892	weakly similar to (154)AT1G52230| Symbols: PSAH2, PSAH-2, PSI-H | PSAH-2/PSAH2/PSI-H (PHOTOSYSTEM I SUBUNIT H-2) | ch	
EL587715	2.891	moderately similar to (384)AT2G26150| Symbols: HSFA2, ATHSFA2 | ATHSFA2 (Arabidopsis thaliana heat shock transcription factor A	
CV432537	2.888	no similarity	
DY003227	2.888	very weakly similar to (89.7)AT5G07460| Symbols: PMSR2 | PMSR2 (PEPTIDEMETHIONINE SULFOXIDE REDUCTASE 2); peptide-r	
JCVI_16297	2.887	highly similar to (726)AT1G34580| Symbols: | monosaccharide transporter, putative | chr1:12660609-12663531 FORWARDmoderately si	
JCVI_1067	2.887	highly similar to (534)AT1G23820| Symbols: SPDS1 | SPDS1 (SPERMIDINE SYNTHASE 1) | chr1:8420267-8422713 FORWARDmoder	
JCVI_39034	2.886	moderately similar to (270)AT2G29150| Symbols: | tropinone reductase, putative / tropine dehydrogenase, putative | chr2:12542792-1254	
EV129466	2.885	weakly similar to (105)AT2G47590| Symbols: PHR2 | PHR2 (PHOTOLYASE/BLUE-LIGHT RECEPTOR 2) | chr2:19528958-19530802	
JCVI_3600	2.884	moderately similar to (457)AT1G68620| Symbols: | hydrolase | chr1:25769681-25770691 FORWARDweakly similar to (120)GID1_ORY	
JCVI_22330	2.884	highly similar to (524)AT5G42240| Symbols: SCPL42 | SCPL42 (serine carboxypeptidase-like 42); serine carboxypeptidase | chr5:169059	
DY018026	2.884	weakly similar to (156)AT1G60570| Symbols: | kelch repeat-containing F-box family protein | chr1:22315501-22316646 FORWARD [18	
JCVI_5917	2.883	moderately similar to (379)AT2G31955| Symbols: CNX2 | CNX2 (COFACTOR OF NITRATE REDUCTASE AND XANTHINE DEHYD	
EE556264	2.883	no similarity	
EL592828	2.882	no similarity	
EV101414	2.882	weakly similar to (107)AT1G09340| Symbols: CRB | CRB; binding / catalytic/ coenzyme binding | chr1:3015475-3018037 FORWARD [2	
EV071808	2.881	moderately similar to (236)AT3G05545| Symbols: | transcription factor, putative / zinc finger (C3HC4 type RING finger) family protein |	-2.198
JCVI_19837	2.880	no original description	
JCVI_1019	2.880	weakly similar to (196)AT4G15610| Symbols: | integral membrane family protein | chr4:8909160-8910639 FORWARD no original descri	-2.450
EE456068	2.880	no similarity	
JCVI_3974	2.880	moderately similar to (380)AT5G13930| Symbols: CHS, TT4, ATCHS | ATCHS/CHS/TT4 (CHALCONE SYNTHASE); naringenin-chalc	
JCVI_32236	2.880	very weakly similar to (82.4)AT1G72290| Symbols: | trypsin and protease inhibitor family protein / Kunitz family protein | chr1:27219514-	
AT000675	2.878	no similarity	
EX020356	2.877	weakly similar to (145)AT1G31300| Symbols: | similar to unknown protein [Arabidopsis thaliana] (TAIR:AT4G19645.2); similar to unkn	
JCVI_19337	2.877	moderately similar to (345)AT5G64430| Symbols: | octicosapeptide/Phox/Bem1p (PB1) domain-containing protein | chr5:25779766-2578	
JCVI_41572	2.877	weakly similar to (102)AT5G67480| Symbols: BT4 | BT4 (BTB AND TAZ DOMAIN PROTEIN 4); protein binding | chr5:26948281-2694	
JCVI_408	2.877	moderately similar to (471)AT3G12290| Symbols: | tetrahydrofolate dehydrogenase/cyclohydrolase, putative | chr3:3919598-3921333 FO	-2.363
DW997607	2.876	moderately similar to (288)AT1G65020| Symbols: | similar to unnamed protein product [Vitis vinifera] (GB:CAO62149.1); contains Inter	
EV045597	2.876	weakly similar to (179)AT1G42470| Symbols: | patched family protein | chr1:15929427-15937568 FORWARD [21442]	
JCVI_836	2.876	moderately similar to (333)AT5G40670| Symbols: | PQ-loop repeat family protein / transmembrane family protein | chr5:16303196-16304	
RC_T18361	2.875	no similarity	-2.407
CN728982	2.875	moderately similar to (223)AT3G05360| Symbols: | disease resistance family protein / LRR family protein | chr3:1530906-1533266 REVE	
JCVI_39663	2.875	weakly similar to (176)AT1G55530| Symbols: | zinc finger (C3HC4-type RING finger) family protein | chr1:20733138-20734193 REVER	
JCVI_16332	2.875	highly similar to (516)AT2G24170| Symbols: | endomembrane protein 70, putative | chr2:10281387-10283974 REVERSE no original desc	
L38037	2.874	weakly similar to (152)AT1G68910| Symbols: | similar to unknown protein [Arabidopsis thaliana] (TAIR:AT5G11390.1); similar to unna	
JCVI_3236	2.873	moderately similar to (440)AT3G53140| Symbols: | O-diphenol-O-methyl transferase, putative | chr3:19706670-19708333 FORWARDwe	
EV203657	2.873	no similarity	
EX138606	2.873	moderately similar to (491)AT3G50930| Symbols: | AAA-type ATPase family protein | chr3:18940798-18942528 FORWARD [21833] 1	-4.698
EX130650	2.873	no similarity	
EE434133	2.873	weakly similar to (124)AT1G32660| Symbols: | F-box family protein | chr1:11811020-11812360 FORWARD [15720]	-4.703
JCVI_7672	2.872	moderately similar to (223)AT3G11220| Symbols: ELO1 | ELO1 (ELONGATA 1) | chr3:3513836-3515996 REVERSE no original descrip	
JCVI_34222	2.871	moderately similar to (245)AT2G27420| Symbols: | cysteine proteinase, putative | chr2:11733389-11734597 REVERSEweakly similar to (	
L37988	2.871	weakly similar to (132)AT1G56345| Symbols: | pseudouridine synthase family protein | chr1:21097074-21098119 REVERSE [132]	
RC_EX063694	2.871	no similarity	
JCVI_17929	2.871	moderately similar to (397)AT5G41670| Symbols: | 6-phosphogluconate dehydrogenase family protein | chr5:16682875-16684338 REVE	
EX120674	2.869	weakly similar to (134)AT4G23160| Symbols: | protein kinase family protein | chr4:12129496-12134097 FORWARD [21829] 19 518 518	-2.318
EV112934	2.869	moderately similar to (315)AT3G19553| Symbols: | amino acid permease family protein | chr3:6790994-6792513 REVERSE [21479] 45	-1.442
JCVI_40504	2.868	weakly similar to (158)AT5G19140| Symbols: | auxin/aluminum-responsive protein, putative | chr5:6423400-6425787 FORWARD no orig	
EE418726	2.868	moderately similar to (307)AT2G37040| Symbols: PAL1 | PAL1 (PHE AMMONIA LYASE 1); phenylalanine ammonia-lyase | chr2:15564	
JCVI_3979	2.867	moderately similar to (454)AT3G18140| Symbols: | transducin family protein / WD-40 repeat family protein | chr3:6213231-6214573 RE	-4.188
ES944113	2.866	weakly similar to (147)AT3G51140| Symbols: | heat shock protein binding | chr3:19009161-19010416 FORWARD [21392]	
JCVI_3169	2.866	moderately similar to (266)AT4G15960| Symbols: | epoxide hydrolase, putative | chr4:9045777-9047213 REVERSE no original descriptio	
JCVI_21653	2.866	weakly similar to (142)AT1G10370| Symbols: GST30, ATGSTU17, GST30B, ERD9 | ATGSTU17/ERD9/GST30/GST30B (EARLY-RES	
EE559363	2.866	no similarity	
EV186492	2.866	weakly similar to (138)AT2G32150| Symbols: | haloacid dehalogenase-like hydrolase family protein | chr2:13666172-13667608 FORWA	
EH416153	2.866	no similarity	
JCVI_12186	2.866	weakly similar to (118)AT4G17840| Symbols: | similar to unknown protein [Arabidopsis thaliana] (TAIR:AT2G35260.1); similar to hypot	
JCVI_8941	2.866	moderately similar to (222)AT1G03090| Symbols: MCCA | MCCA (3-methylcrotonyl-CoA carboxylase 1) | chr1:739715-743819 FORWA	
JCVI_27342	2.865	moderately similar to (344)AT3G57150| Symbols: ATNAP57, CBF5, ATCBF5, NAP57 | NAP57 (ARABIDOPSIS THALIANA HOMOLO	
JCVI_34379	2.864	moderately similar to (249)AT3G59500| Symbols: | integral membrane HRF1 family protein | chr3:21998087-21999146 FORWARD no o	
EX043766	2.864	moderately similar to (222)AT3G21230| Symbols: 4CL5 | 4CL5 (4-COUMARATE:COA LIGASE 5); 4-coumarate-CoA ligase | chr3:7448	
EV156162	2.864	moderately similar to (271)AT2G30140| Symbols: | UDP-glucoronosyl/UDP-glucosyl transferase family protein | chr2:12879277-1288076	
JCVI_11966	2.863	highly similar to (664)AT3G18830| Symbols: ATPLT5 | ATPLT5 (POLYOL TRANSPORTER 5); D-ribose transmembrane transporter/ D	
JCVI_4940	2.863	moderately similar to (334)AT1G20200| Symbols: EMB2719 | EMB2719 (EMBRYO DEFECTIVE 2719) | chr1:7001400-7004145 REVE	
JCVI_24499	2.863	moderately similar to (305)AT4G30920| Symbols: | cytosol aminopeptidase family protein | chr4:15046595-15049310 REVERSEmoderat	
JCVI_6435	2.863	weakly similar to (141)AT4G29905| Symbols: | similar to unknown protein [Arabidopsis thaliana] (TAIR:AT5G57123.1); similar to hypot	
JCVI_3629	2.862	highly similar to (571)AT4G22690| Symbols: CYP706A1 | CYP706A1 (cytochrome P450, family 706, subfamily A, polypeptide 1); oxyge	
EV186095	2.862	moderately similar to (370)AT3G13080| Symbols: EST2, MRP3, ATMRP3 | ATMRP3 (Arabidopsis thaliana multidrug resistance-associat	
DN961112	2.860	weakly similar to (108)AT5G27120| Symbols: | SAR DNA-binding protein, putative | chr5:9541290-9543687 FORWARD [17359]	-2.008
EE478250	2.860	no similarity	
JCVI_12520	2.859	moderately similar to (270)AT4G34640| Symbols: ERG9, SQS1 | SQS1 (SQUALENE SYNTHASE 1); farnesyl-diphosphate farnesyltransf	
JCVI_4136	2.859	moderately similar to (431)AT5G06060| Symbols: | tropinone reductase, putative / tropine dehydrogenase, putative | chr5:1824067-18258	
JCVI_839	2.859	moderately similar to (400)AT2G19520| Symbols: ACG1, MSI4, NFC4, NFC04, ATMSI4, FVE | FVE | chr2:8463088-8466317 FORWAR	
JCVI_8741	2.859	moderately similar to (476)AT3G24670| Symbols: | pectate lyase family protein | chr3:9006212-9008808 REVERSEmoderately similar to	
JCVI_41809	2.858	moderately similar to (405)AT1G07230| Symbols: | phosphoesterase family protein | chr1:2220508-2222777 REVERSE no original descri	
JCVI_8645	2.858	moderately similar to (382)AT1G35470| Symbols: | SPla/RYanodine receptor (SPRY) domain-containing protein | chr1:13051614-13054	

EX070220	2.857	no similarity	
JCVI_9838	2.857	moderately similar to (387)AT3G09630| Symbols: | 60S ribosomal protein L4/L1 (RPL4A) | chr3:2953818-2955449 FORWARD no origi	
JCVI_412	2.857	moderately similar to (317)AT4G23100| Symbols: GSH1, CAD2, PAD2, RML1 | RML1 (ROOT MERISTEMLESS 1) | chr4:12103790-12	
JCVI_29708	2.856	moderately similar to (395)AT2G20980| Symbols: MCM10 | MCM10 | chr2:9018735-9020937 REVERSE no original description	
JCVI_11872	2.856	weakly similar to (187)AT1G06830| Symbols: | glutaredoxin family protein | chr1:2097188-2097487 FORWARD no original description	
EE556263	2.855	no similarity	
EE420004	2.854	moderately similar to (244)AT3G26560| Symbols: | ATP-dependent RNA helicase, putative | chr3:9751359-9754956 REVERSE [20149]	
JCVI_3124	2.854	moderately similar to (399)AT3G05530| Symbols: ATS6A.2, RPT5A | RPT5A (regulatory particle triple-A 5A); ATPase/ calmodulin bind	
JCVI_1394	2.854	moderately similar to (432)AT4G25640| Symbols: | MATE efflux family protein | chr4:13076962-13078974 REVERSE no original descri	-1.998
EX094798	2.853	very weakly similar to (85.9)AT3G30460| Symbols: | zinc finger (C3HC4-type RING finger) family protein | chr3:12106912-12107355 FO	
JCVI_14917	2.853	weakly similar to (195)AT1G09130| Symbols: | ATP-dependent Clp protease proteolytic subunit, putative | chr1:2940065-2942219 REVE	
JCVI_26052	2.853	highly similar to (545)AT3G11480| Symbols: BSMT1 | BSMT1; S-adenosylmethionine-dependent methyltransferase | chr3:3614550-3617	1.647
CV432616	2.852	weakly similar to (152)AT3G14790| Symbols: RHM3 | RHM3 (RHAMNOSE BIOSYNTHESIS 3); catalytic | chr3:4964798-4966882 FOR	
EE568125	2.852	no similarity	
JCVI_14394	2.851	weakly similar to (199)AT4G34270| Symbols: | TIP41-like family protein | chr4:16404142-16406157 REVERSE no original description	
JCVI_7341	2.851	moderately similar to (374)AT1G03905| Symbols: | ABC transporter family protein | chr1:993477-995593 FORWARD no original descrip	
EX112446	2.851	moderately similar to (386)AT2G20610| Symbols: HLS3, RTY, ALF1, RTY1, SUR1 | SUR1 (SUPERROOT 1); transaminase | chr2:88853	
EE437351	2.850	moderately similar to (290)AT1G77130| Symbols: PGSIP2 | PGSIP2 (PLANT GLYCOGENIN-LIKE STARCH INITIATION PROTEIN 2	
JCVI_20062	2.849	highly similar to (511)AT1G07670| Symbols: | calcium-transporting ATPase | chr1:2370302-2374193 REVERSEweakly similar to (181)A	
JCVI_17235	2.849	moderately similar to (231)AT2G37250| Symbols: ADK, ATPADK1 | ADK/ATPADK1 (ADENOSINE KINASE); adenylate kinase/ nucle	
JCVI_23618	2.849	weakly similar to (182)AT1G72830| Symbols: ATHAP2C, HAP2, HAP2C | HAP2C (Heme activator protein (yeast) homolog 2C); transcri	
CB686379	2.848	no similarity	-2.186
JCVI_34612	2.848	weakly similar to (141)AT3G05545| Symbols: | transcription factor, putative / zinc finger (C3HC4 type RING finger) family protein | chr3	
JCVI_3179	2.847	weakly similar to (187)AT4G38770| Symbols: ATPRP4, PRP4 | PRP4 (PROLINE-RICH PROTEIN 4) | chr4:18097003-18098442 REVER	
EV021162	2.847	weakly similar to (186)AT5G23810| Symbols: AAP7 | AAP7 (amino acid permease 7) | chr5:8028464-8030141 FORWARD [21441] 1 471	-1.998
JCVI_18334	2.845	moderately similar to (417)AT2G30490| Symbols: ATC4H, C4H, CYP73A5 | ATC4H/C4H/CYP73A5 (CINNAMATE 4-HYDROXYLAS	
EE522061	2.845	no similarity	
EV167413	2.844	moderately similar to (206)AT2G45140| Symbols: | vesicle-associated membrane protein, putative / VAMP, putative | chr2:18618104-186	
JCVI_5209	2.844	moderately similar to (374)AT3G26210| Symbols: CYP71B23 | CYP71B23 (cytochrome P450, family 71, subfamily B, polypeptide 23); o	
JCVI_12904	2.844	moderately similar to (446)AT3G19170| Symbols: ATPREP1, ATZNMP | ATPREP1/ATZNMP (PRESEQUENCE PROTEASE 1); metall	1.656
JCVI_15749	2.844	moderately similar to (397)AT3G57020| Symbols: | strictosidine synthase family protein | chr3:21109494-21111282 REVERSEvery weak	
JCVI_3412	2.843	moderately similar to (482)AT2G13360| Symbols: AGT1, AGT | AGT (ALANINE:GLYOXYLATE AMINOTRANSFERASE) | chr2:5546	
EV142997	2.842	moderately similar to (270)AT4G05020| Symbols: NDB2 | NDB2 (NAD(P)H DEHYDROGENASE B2); disulfide oxidoreductase | chr4:2	
JCVI_29524	2.842	moderately similar to (357)AT1G59900| Symbols: AT-E1 ALPHA | AT-E1 ALPHA (pyruvate dehydrogenase complex E1 alpha subunit);	
JCVI_5215	2.841	moderately similar to (342)AT5G20150| Symbols: | SPX (SYG1/Pho81/XPR1) domain-containing protein | chr5:6802431-6803369 FORV	
JCVI_6997	2.841	moderately similar to (333)AT3G03080| Symbols: | NADP-dependent oxidoreductase, putative | chr3:698537-700285 REVERSE no origi	
JCVI_26816	2.841	moderately similar to (307)AT1G71840| Symbols: | transducin family protein / WD-40 repeat family protein | chr1:27026086-27028042 F	
JCVI_40287	2.840	moderately similar to (380)AT4G38630| Symbols: MCB1, ATMCB1, MBP1, RPN10 | RPN10 (REGULATORY PARTICLE NON-ATPA	
EV125249	2.840	weakly similar to (178)AT2G17420| Symbols: ATNTRA, NTR2, NTRA | NTRA (NADPH-dependent thioredoxin reductase 2) | chr2:7571	-1.789
JCVI_19483	2.840	moderately similar to (379)AT2G30140| Symbols: | UDP-glucoronosyl/UDP-glucosyl transferase family protein | chr2:12879277-1288076	
CV432692	2.839	no similarity	
JCVI_32779	2.839	moderately similar to (221)AT5G48900| Symbols: | pectate lyase family protein | chr5:19842466-19846135 FORWARDweakly similar to	
JCVI_12958	2.838	moderately similar to (244)AT3G03440| Symbols: | armadillo/beta-catenin repeat family protein | chr3:815716-818575 FORWARD no ori	
JCVI_5542	2.838	moderately similar to (422)AT3G11400| Symbols: ATEIF3G1, EIF3G1 | EIF3G1 (eukaryotic translation initiation factor 3G1); RNA bindi	
JCVI_14287	2.838	moderately similar to (361)AT2G17970| Symbols: | oxidoreductase, 2OG-Fe(II) oxygenase family protein | chr2:7826198-7827869 REVE	
JCVI_40857	2.838	highly similar to (506)AT1G66910| Symbols: | protein kinase, putative | chr1:24965297-24967604 REVERSEweakly similar to (189)KPF	
JCVI_33361	2.838	moderately similar to (228)AT1G11480| Symbols: | eukaryotic translation initiation factor-related | chr1:3864368-3866707 REVERSE no	
JCVI_30378	2.836	moderately similar to (382)AT1G21210| Symbols: WAK4 | WAK4 (WALL ASSOCIATED KINASE 4); protein serine/threonine kinase | c	
JCVI_27097	2.835	moderately similar to (313)AT3G21790| Symbols: | UDP-glucoronosyl/UDP-glucosyl transferase family protein | chr3:7676934-7678421	
JCVI_18791	2.835	moderately similar to (493)AT3G03780| Symbols: AtMS2 | AtMS2 (Arabidopsis thaliana methionine synthase 2) | chr3:957609-960747 F	
EV223498	2.835	moderately similar to (248)AT5G45890| Symbols: SAG12 | SAG12 (SENESCENCE-ASSOCIATED GENE 12); cysteine-type peptidase |	
EX094581	2.834	moderately similar to (254)AT3G05200| Symbols: ATL6 | ATL6 (Arabidopsis T?xicos en Levadura 6); protein binding / zinc ion binding |	
ES948682	2.834	weakly similar to (181)AT4G05460| Symbols: | F-box family protein (FBL20) | chr4:2761103-2762397 REVERSE [21393]	
JCVI_38890	2.834	moderately similar to (241)AT3G22600| Symbols: | protease inhibitor/seed storage/lipid transfer protein (LTP) family protein | chr3:8006	
JCVI_24184	2.833	moderately similar to (405)AT1G24180| Symbols: IAR4 | IAR4 (IAA-conjugate-resistant 4); pyruvate dehydrogenase (acetyl-transferring)	
JCVI_4520	2.833	moderately similar to (233)AT4G33140| Symbols: | similar to unnamed protein product [Vitis vinifera] (GB:CAO65164.1); contains doma	
JCVI_27206	2.832	very weakly similar to (93.2)AT5G61240| Symbols: | protein binding | chr5:24646711-24649184 FORWARD no original description	
JCVI_9945	2.832	moderately similar to (334)AT2G37770| Symbols: | aldo/keto reductase family protein | chr2:15841966-15843737 FORWARDweakly sim	
JCVI_4168	2.832	moderately similar to (290)AT3G12920| Symbols: | protein binding / zinc ion binding | chr3:4122134-4123330 REVERSE no original desc	
JCVI_19928	2.829	weakly similar to (162)AT5G62150| Symbols: | peptidoglycan-binding LysM domain-containing protein | chr5:24975551-24975859 FORV	
JCVI_15325	2.829	moderately similar to (374)AT3G01150| Symbols: PTB | PTB (POLYPYRIMIDINE TRACT-BINDING) | chr3:51739-54351 FORWARD	
JCVI_37069	2.828	very weakly similar to (95.5)AT1G74870| Symbols: | protein binding / zinc ion binding | chr1:28130754-28131936 FORWARD no original	
DN966088	2.828	moderately similar to (204)AT3G53810| Symbols: | lectin protein kinase, putative | chr3:19944131-19946164 REVERSEvery weakly simi	-1.746
JCVI_32681	2.828	weakly similar to (129)AT1G70330| Symbols: ENT1,AT | ENT1,AT (EQUILIBRATIVE NUCLEOTIDE TRANSPORTER 1); nucleoside	
JCVI_29609	2.828	weakly similar to (194)AT5G61730| Symbols: ATATH11 | ATATH11 (ABC2 homolog 11); ATPase, coupled to transmembrane movemer	
EX097379	2.828	moderately similar to (391)AT1G50420| Symbols: SCL-3, SCL3 | SCL3 (SCARECROW-LIKE 3); transcription factor | chr1:18681845-18	
JCVI_21247	2.827	moderately similar to (227)AT4G19970| Symbols: | similar to unknown protein [Arabidopsis thaliana] (TAIR:AT5G44820.1); similar to u	
JCVI_32477	2.827	highly similar to (525)AT1G66750| Symbols: CDKD1;2, CAK4AT, AT;CDKD;2, CDKD;2 | AT;CDKD;2/CAK4AT/CDKD1;2/CDKD;2 (	
EE429874	2.827	weakly similar to (147)AT3G53460| Symbols: CP29 | CP29 (chloroplast 29 kDa ribonucleoprotein); RNA binding / poly(U) binding | chr3	
DN965398	2.827	weakly similar to (162)AT5G08690| Symbols: | ATP synthase beta chain 2, mitochondrial | chr5:2825740-2828353 FORWARDweakly sir	
RC_H07525	2.826	no similarity	
EG020802	2.826	weakly similar to (169)AT2G02370| Symbols: | similar to unknown protein [Arabidopsis thaliana] (TAIR:AT1G12450.1); similar to unnar	
JCVI_17243	2.826	moderately similar to (400)AT1G74090| Symbols: | sulfotransferase family protein | chr1:27866664-27867716 FORWARD no original de	
JCVI_23694	2.826	weakly similar to (160)AT3G21250| Symbols: ATMRP6 | ATMRP6 (Arabidopsis thaliana multidrug resistance-associated protein 6) | chr3	
JCVI_32759	2.826	moderately similar to (408)AT5G16230| Symbols: | acyl-(acyl-carrier-protein) desaturase, putative / stearoyl-ACP desaturase, putative | ch	
JCVI_7571	2.825	no original description	
JCVI_3714	2.825	moderately similar to (445)AT3G13870| Symbols: RHD3 | RHD3 (ROOT HAIR DEFECTIVE 3) | chr3:4565769-4571116 REVERSE no	
EV225319	2.823	no similarity	
DY013636	2.822	no similarity	
JCVI_5821	2.822	highly similar to (530)AT1G15690| Symbols: ATAVP3, AVP-3, AVP1 | AVP1 (vacuolar-type H+-pumping pyrophosphatase 1) | chr1:539	
EX064019	2.821	weakly similar to (138)AT5G58330| Symbols: | malate dehydrogenase (NADP), chloroplast, putative | chr5:23597236-23599513 REVERS	

JCVI_14508	2.821	moderately similar to (364)AT3G54360| Symbols: | protein binding / zinc ion binding | chr3:20139548-20142559 REVERSE no original d	
CA991761	2.821	no similarity	
JCVI_8973	2.821	moderately similar to (462)AT5G44070| Symbols: ARA8, ATPCS1, PCS1, CAD1 | CAD1 (CADMIUM SENSITIVE 1) | chr5:17752103-1	
JCVI_11078	2.821	moderately similar to (406)AT4G24880| Symbols: | similar to unnamed protein product [Vitis vinifera] (GB:CAO68295.1); contains doma	
EV194821	2.820	moderately similar to (352)AT5G28840| Symbols: GME | GME (GDP-D-MANNOSE 3',5'-EPIMERASE); GDP-mannose 3,5-epimerase/ N	-1.671
DY024177	2.820	no similarity	
EV079795	2.820	moderately similar to (412)AT1G69870| Symbols: | proton-dependent oligopeptide transport (POT) family protein | chr1:26319871-26323	
JCVI_14882	2.819	moderately similar to (307)AT2G35840| Symbols: | sucrose-phosphatase 1 (SPP1) | chr2:15061031-15062855 FORWARD no original des	
JCVI_33591	2.819	moderately similar to (271)AT5G19580| Symbols: | glyoxal oxidase-related | chr5:6607597-6609519 REVERSE no original description	
JCVI_37702	2.819	highly similar to (555)AT5G11880| Symbols: | diaminopimelate decarboxylase, putative / DAP carboxylase, putative | chr5:3827807-3829	
JCVI_37897	2.818	moderately similar to (476)AT1G09240| Symbols: | nicotianamine synthase, putative | chr1:2984952-2985914 FORWARDmoderately sim	
JCVI_14392	2.818	moderately similar to (363)AT3G62770| Symbols: AtATG18a | AtATG18a (Arabidopsis thaliana homolog of yeast autophagy 18 (ATG18)	
EV116254	2.817	moderately similar to (283)AT2G45260| Symbols: | similar to unknown protein [Arabidopsis thaliana] (TAIR:AT4G34080.1); similar to u	
EV191175	2.817	moderately similar to (326)AT1G26850| Symbols: | dehydration-responsive family protein | chr1:9301133-9303419 REVERSE [21489]	
EV066456	2.817	moderately similar to (244)AT2G34840| Symbols: | coatomer protein epsilon subunit family protein / COPE family protein | chr2:1471001	
EX096770	2.816	no similarity	
JCVI_7071	2.816	moderately similar to (256)AT2G05600| Symbols: | similar to F-box family protein [Arabidopsis thaliana] (TAIR:AT2G02030.1); similar	
ES944496	2.815	no similarity	
JCVI_18806	2.815	moderately similar to (245)AT3G12750| Symbols: ZIP1 | ZIP1 (ZINC TRANSPORTER 1 PRECURSOR); zinc ion transmembrane transpo	
JCVI_19715	2.815	moderately similar to (275)AT4G35760| Symbols: | electron carrier/ protein disulfide oxidoreductase | chr4:16942738-16944627 REVERS	
EV035283	2.815	very weakly similar to (99.8)AT5G09680| Symbols: | cytochrome b5 domain-containing protein | chr5:2999363-3000186 REVERSE [2144	
EV145123	2.814	weakly similar to (146)AT5G52550| Symbols: | similar to unknown protein [Arabidopsis thaliana] (TAIR:AT4G25670.1); similar to hypot	
EV114803	2.814	highly similar to (506)AT4G07410| Symbols: | transducin family protein / WD-40 repeat family protein | chr4:4201462-4204573 REVERS	-1.308
JCVI_1658	2.814	moderately similar to (428)AT2G46280| Symbols: TIF3I1, TRIP-1 | TRIP-1 (TGF-BETA RECEPTOR INTERACTING PROTEIN 1); nuc	
JCVI_36012	2.813	weakly similar to (169)AT3G61180| Symbols: | zinc finger (C3HC4-type RING finger) family protein | chr3:22656655-22658265 FORWA	
JCVI_14161	2.813	moderately similar to (308)AT4G24080| Symbols: ALL1 | ALL1 (ALDOLASE LIKE); carbon-carbon lyase/ catalytic | chr4:12511394-125	
JCVI_2486	2.813	moderately similar to (397)AT3G03890| Symbols: | FMN binding | chr3:999844-1002003 REVERSE no original description	
JCVI_35252	2.813	moderately similar to (404)AT3G13224| Symbols: | RNA recognition motif (RRM)-containing protein | chr3:4254855-4256628 FORWAR	
JCVI_1675	2.813	highly similar to (501)AT1G08200| Symbols: AXS2 | AXS2 (UDP-D-APIOSE/UDP-D-XYLOSE SYNTHASE 2) | chr1:2574256-2576606	
JCVI_13507	2.813	moderately similar to (405)AT1G54040| Symbols: ESR, TASTY, ESP | ESP (EPITHIOSPECIFIER PROTEIN) | chr1:20174663-2017755	
JCVI_23355	2.812	highly similar to (687)AT2G36530| Symbols: LOS2 | LOS2 (Low expression of osmotically responsive genes 1); phosphopyruvate hydrata	
EX020406	2.812	no similarity	
DY008143	2.812	no similarity	
JCVI_17430	2.810	moderately similar to (251)AT4G03410| Symbols: | peroxisomal membrane protein-related | chr4:1501906-1503502 FORWARD no origin	
EV193869	2.810	weakly similar to (168)AT5G09620| Symbols: | octicosapeptide/Phox/Bem1p (PB1) domain-containing protein | chr5:2983758-2985353 F	
EV106086	2.809	weakly similar to (140)AT3G50830| Symbols: ATCOR413-PM2 | COR413-PM2 (cold regulated 413 plasma membrane 2) | chr3:1890509(	
JCVI_13426	2.808	moderately similar to (274)AT4G25660| Symbols: | similar to unknown protein [Arabidopsis thaliana] (TAIR:AT4G25680.1); similar to u	
JCVI_11868	2.808	weakly similar to (147)AT3G21055| Symbols: PSBTN | PSBTN (photosystem II subunit T) | chr3:7376767-7377078 REVERSE no origina	
JCVI_21321	2.808	highly similar to (770)AT3G26560| Symbols: | ATP-dependent RNA helicase, putative | chr3:9751359-9754956 REVERSE no original de	
JCVI_39946	2.808	moderately similar to (204)AT3G13950| Symbols: | similar to unknown protein [Arabidopsis thaliana] (TAIR:AT4G13266.1); similar to A	
JCVI_3089	2.807	moderately similar to (293)AT4G11600| Symbols: PHGPX, LSC803, ATGPX6 | ATGPX6 (GLUTATHIONE PEROXIDASE 6); glutathio	1.202
EX056191	2.807	very weakly similar to (100)AT3G57070| Symbols: | glutaredoxin family protein | chr3:21135153-21136406 FORWARD [21813]	4.581
EE528733	2.807	very weakly similar to (82.0)AT5G58230| Symbols: MEE70, MSI1 | MSI1 (MULTICOPY SUPRESSOR OF IRA1) | chr5:23573338-23575	
JCVI_29508	2.806	moderately similar to (215)AT4G03610| Symbols: | phosphonate metabolism protein-related | chr4:1605037-1606857 FORWARD no orig	
CX190307	2.806	moderately similar to (462)AT3G53180| Symbols: | glutamate-ammonia ligase | chr3:19718046-19722166 FORWARD [16807]	
JCVI_40567	2.806	weakly similar to (142)AT4G16360| Symbols: | 5'-AMP-activated protein kinase beta-2 subunit, putative | chr4:9245681-9246852 FORWA	
JCVI_34835	2.805	moderately similar to (209)AT4G19700| Symbols: | protein binding / zinc ion binding | chr4:10713643-10714645 REVERSE no original d	
JCVI_26196	2.805	moderately similar to (410)AT3G57750| Symbols: | protein kinase, putative | chr3:21405027-21406031 FORWARDweakly similar to (11	
JCVI_9029	2.805	highly similar to (956)AT1G62750| Symbols: ATSCO1, ATSCO1/CPEF-G, SCO1 | ATSCO1/ATSCO1/CPEF-G/SCO1 (SNOWY COTYL	
AM059109	2.805	weakly similar to (108)AT3G07950| Symbols: | rhomboid protein-related | chr3:2531988-2534283 FORWARD [17712]	
JCVI_26619	2.804	moderately similar to (228)AT3G13120| Symbols: | 30S ribosomal protein S10, chloroplast, putative | chr3:4220317-4221533 REVERSEn	
JCVI_1875	2.804	moderately similar to (389)AT5G25265| Symbols: | similar to unknown protein [Arabidopsis thaliana] (TAIR:AT2G25260.1); similar to u	
EV004039	2.804	no similarity	-2.755
JCVI_5364	2.804	highly similar to (618)AT4G29810| Symbols: MKK2, MK1, ATMKK2 | ATMKK2 (MAP KINASE KINASE 2) | chr4:14593485-1459524	
ES901370	2.804	moderately similar to (368)AT1G79680| Symbols: | wall-associated kinase, putative | chr1:29985081-29987642 REVERSEweakly similar	
EX089281	2.803	very weakly similar to (94.0)AT4G39260| Symbols: GR-RBP8, ATGRP8, CCR1 | ATGRP8/GR-RBP8 (COLD, CIRCADIAN RHYTHM, A	
JCVI_19	2.802	moderately similar to (326)AT1G16720| Symbols: HCF173 | HCF173 (HIGH CHLOROPHYLL FLUORESCENCE PHENOTYPE 173); b	
EE486087	2.801	very weakly similar to (99.4)AT1G10830| Symbols: | sodium symporter-related | chr1:3606412-3607450 REVERSE [20165]	
JCVI_13745	2.800	moderately similar to (426)AT3G51130| Symbols: | Identical to UPF0183 protein At3g51130 [Arabidopsis Thaliana] (GB:Q9SD33;GB:Q	
AM396012	2.800	no similarity	
JCVI_36305	2.799	highly similar to (542)AT2G02800| Symbols: APK2B | APK2B (PROTEIN KINASE 2B); kinase | chr2:796888-799249 REVERSEweakly	-3.070
EH420911	2.799	moderately similar to (328)AT1G26440| Symbols: ATUPS5 | ATUPS5 (ARABIDOPSIS THALIANA UREIDE PERMEASE 5) | chr1:914	
JCVI_35335	2.799	highly similar to (536)AT4G16390| Symbols: | chloroplastic RNA-binding protein P67, putative | chr4:9258050-9260116 FORWARDwea	
JCVI_6142	2.799	moderately similar to (439)AT3G61320| Symbols: | Identical to UPF0187 protein At3g61320, chloroplast precursor [Arabidopsis Thaliana	
JCVI_3712	2.799	weakly similar to (141)AT1G75390| Symbols: ATBZIP44 | ATBZIP44 (ARABIDOPSIS THALIANA BASIC LEUCINE-ZIPPER 44) | chr	
EX101231	2.798	moderately similar to (311)AT3G50950| Symbols: | disease resistance protein (CC-NBS-LRR class), putative | chr3:18947108-18949666 I	
JCVI_18128	2.798	moderately similar to (224)AT5G57170| Symbols: | macrophage migration inhibitory factor family protein / MIF family protein | chr5:231	
EE560776	2.797	weakly similar to (107)AT3G54910| Symbols: | similar to F-box family protein [Arabidopsis thaliana] (TAIR:AT4G10400.2); similar to F	
EV105678	2.796	no similarity	
DY012593	2.796	moderately similar to (342)AT3G26090| Symbols: ATRGS1, RGS1 | RGS1 (REGULATOR OF G-PROTEIN SIGNALING 1) | chr3:95341	
EE433660	2.796	moderately similar to (316)AT3G49740| Symbols: | pentatricopeptide (PPR) repeat-containing protein | chr3:18458773-18460986 FORWA	
EX124763	2.796	moderately similar to (418)AT3G59040| Symbols: | pentatricopeptide (PPR) repeat-containing protein | chr3:21832472-21835210 REVER	
JCVI_4566	2.795	moderately similar to (298)AT3G12930| Symbols: | similar to unknown [Populus trichocarpa] (GB:ABK94112.1); contains InterPro doma	
JCVI_9813	2.795	moderately similar to (403)AT3G26710| Symbols: | similar to unnamed protein product [Vitis vinifera] (GB:CAO49107.1) | chr3:9814787	
ES900811	2.795	moderately similar to (318)AT1G67570| Symbols: | similar to unknown protein [Arabidopsis thaliana] (TAIR:AT1G50630.1); similar to u	
JCVI_53	2.794	moderately similar to (378)AT3G12920| Symbols: | protein binding / zinc ion binding | chr3:4122134-4123330 REVERSE no original desc	
EE434856	2.794	moderately similar to (318)AT2G30740| Symbols: | serine/threonine protein kinase, putative | chr2:13103476-13105362 FORWARDvery	
JCVI_8851	2.793	moderately similar to (317)AT4G34120| Symbols: LEJ1 | LEJ1 (LOSS OF THE TIMING OF ET AND JA BIOSYNTHESIS 1) | chr4:1634	
CB686234	2.793	no similarity	
EE439042	2.792	weakly similar to (131)AT1G80040| Symbols: | similar to unknown protein [Arabidopsis thaliana] (TAIR:AT5G32440.1); similar to hypot	
JCVI_13875	2.792	moderately similar to (362)AT1G31130| Symbols: | similar to unknown protein [Arabidopsis thaliana] (TAIR:AT5G44860.1); similar to u	

EX114227	2.792	moderately similar to (253)AT2G40113| Symbols: | similar to unknown protein [Arabidopsis thaliana] (TAIR:AT5G47635.1); similar to h	
EE446777	2.791	weakly similar to (107)AT3G12740| Symbols: ALIS1 | LEM3 (ligand-effect modulator 3) family protein / CDC50 family protein | chr3:404	
EV070097	2.791	moderately similar to (321)AT3G07650| Symbols: COL9 | COL9 (CONSTANS-LIKE 9) | chr3:2442500-2443907 FORWARD [21443]	1.158
DY017647	2.791	weakly similar to (135)AT3G17580| Symbols: | similar to unknown protein [Arabidopsis thaliana] (TAIR:AT1G48330.1); similar to unkno	
JCVI_10852	2.791	moderately similar to (404)AT5G64610| Symbols: HAM1 | HAM1 (HISTONE ACETYLTRANSFERASE OF THE MYST FAMILY 1); H	
JCVI_28672	2.791	moderately similar to (482)AT5G61010| Symbols: ATEXO70E2 | ATEXO70E2 (EXOCYST SUBUNIT EXO70 FAMILY PROTEIN E2);	
JCVI_5551	2.790	moderately similar to (211)AT2G16600| Symbols: ROC3 | ROC3 (rotamase CyP 3) | chr2:7207944-7208465 FORWARDmoderately simil	
EV069563	2.788	weakly similar to (114)AT3G52910| Symbols: AtGRF4 | AtGRF4 (GROWTH-REGULATING FACTOR 4) | chr3:19627155-19629246 RE	
EV209468	2.788	moderately similar to (374)AT3G06170| Symbols: | TMS membrane family protein / tumour differentially expressed (TDE) family protein	
JCVI_14989	2.787	moderately similar to (451)AT4G02990| Symbols: | mitochondrial transcription termination factor family protein / mTERF family protein	
JCVI_16678	2.786	highly similar to (838)AT5G26742| Symbols: EMB1138 | EMB1138 (EMBRYO DEFECTIVE 1138); ATP binding / ATP-dependent helic	
JCVI_9217	2.786	moderately similar to (204)AT3G62960| Symbols: | glutaredoxin family protein | chr3:23279755-23280063 FORWARD no original descri	
JCVI_22606	2.786	no original description	
JCVI_1661	2.786	moderately similar to (297)AT4G11260| Symbols: ATSGT1B, ETA3, RPR1, EDM1, SGT1B | SGT1B (enhanced downy mildew 1b); prot	-2.490
AM386167	2.786	moderately similar to (385)AT4G27880| Symbols: | seven in absentia (SINA) family protein | chr4:13883629-13884933 FORWARDweak	
JCVI_167	2.786	highly similar to (516)AT4G02730| Symbols: | transducin family protein / WD-40 repeat family protein | chr4:1207759-1209066 FORWA	-2.219
JCVI_27720	2.786	moderately similar to (325)AT1G17160| Symbols: | pfkB-type carbohydrate kinase family protein | chr1:5867671-5869168 FORWARD no	
JCVI_3087	2.786	moderately similar to (258)AT3G12250| Symbols: BZIP45, TGA6 | TGA6 (TGA1a-related gene 6) | chr3:3906643-3908590 FORWARDw	
JCVI_18836	2.784	moderately similar to (464)AT1G06890| Symbols: | transporter-related | chr1:2111727-2114037 REVERSE no original description	
EV031551	2.784	no similarity	
EH425403	2.784	weakly similar to (181)AT4G10890| Symbols: | similar to unknown protein [Arabidopsis thaliana] (TAIR:AT1G43722.1); similar to unnar	
JCVI_4407	2.783	moderately similar to (402)AT5G62700| Symbols: ATGCP3, TUB3 | TUB3 (TUBULIN BETA-3); structural molecule | chr5:25201727-25	
RC_H74774	2.783	no similarity	-2.523
JCVI_5224	2.783	moderately similar to (212)AT4G31450| Symbols: | zinc finger (C3HC4-type RING finger) family protein | chr4:15255961-15257983 REV	-1.650
JCVI_2254	2.782	moderately similar to (275)AT3G53460| Symbols: CP29 | CP29 (chloroplast 29 kDa ribonucleoprotein); RNA binding / poly(U) binding | c	
EX091301	2.782	weakly similar to (144)AT4G10890| Symbols: | similar to unknown protein [Arabidopsis thaliana] (TAIR:AT1G43722.1); similar to unnar	
JCVI_607	2.782	moderately similar to (498)AT5G03240| Symbols: UBQ3 | UBQ3 (POLYUBIQUITIN 3); protein binding | chr5:771975-772895 REVERS	
JCVI_17664	2.781	highly similar to (798)AT2G01320| Symbols: | ABC transporter family protein | chr2:154668-158062 REVERSEweakly similar to (181)P	
JCVI_768	2.781	weakly similar to (139)AT5G08300| Symbols: | succinyl-CoA ligase (GDP-forming) alpha-chain, mitochondrial, putative / succinyl-CoA s	
ES902339	2.781	moderately similar to (293)AT2G42230| Symbols: | tubulin-specific chaperone C-related | chr2:17600885-17603641 REVERSE [21428]	
CD817556	2.781	moderately similar to (285)AT4G01220| Symbols: | similar to RGXT1 (RHAMNOGALACTURONAN XYLOSYLTRANSFERASE 1), U	-3.167
JCVI_10361	2.781	moderately similar to (385)AT4G27680| Symbols: | MSP1 protein, putative / intramitochondrial sorting protein, putative | chr4:13821269-	
EE522481	2.780	moderately similar to (256)AT1G72130| Symbols: | proton-dependent oligopeptide transport (POT) family protein | chr1:27141425-27142	
EE457777	2.780	weakly similar to (130)AT1G07410| Symbols: AtRABA2b | AtRABA2b (Arabidopsis Rab GTPase homolog A2b); GTP binding | chr1:227	
JCVI_12230	2.780	highly similar to (597)AT3G57890| Symbols: | tubulin-specific chaperone C-related | chr3:21449248-21452672 FORWARD no original de	
EE475896	2.780	moderately similar to (202)AT5G63000| Symbols: | similar to hypothetical protein OsJ_001206 [Oryza sativa (japonica cultivar-group)] ((	
EV055474	2.780	moderately similar to (301)AT1G47230| Symbols: CYCA3;4 | CYCA3;4; cyclin-dependent protein kinase regulator | chr1:17309192-1731	
JCVI_13236	2.780	moderately similar to (350)AT5G25050| Symbols: | integral membrane transporter family protein | chr5:8632025-8633831 FORWARD no	
EH413811	2.780	moderately similar to (214)AT1G35710| Symbols: | leucine-rich repeat transmembrane protein kinase, putative | chr1:13222152-13225598	
ES944547	2.780	very weakly similar to (97.4)AT3G15690| Symbols: | biotin carboxyl carrier protein of acetyl-CoA carboxylase-related | chr3:5317115-531	
JCVI_8843	2.780	moderately similar to (391)AT2G17120| Symbols: LYM2 | LYM2 (LYSM DOMAIN GPI-ANCHORED PROTEIN 2 PRECURSOR) | chr	
EE519641	2.779	very weakly similar to (92.4)AT4G23060| Symbols: IQD22 | IQD22 (IQ-domain 22); calmodulin binding | chr4:12087294-12090419 FORV	
JCVI_8910	2.777	moderately similar to (205)AT4G13590| Symbols: | similar to unknown protein [Arabidopsis thaliana] (TAIR:AT1G64150.1); similar to u	
JCVI_12092	2.775	moderately similar to (442)AT1G67680| Symbols: | 7S RNA binding | chr1:25369625-25372127 REVERSE no original description	
JCVI_843	2.774	moderately similar to (388)AT4G11150| Symbols: EMB2448, TUFF, VHA-E1, TUF | TUF (VACUOLAR ATP SYNTHASE SUBUNIT E	
EV127386	2.774	no similarity	
EV127408	2.774	no similarity	
JCVI_13020	2.774	weakly similar to (108)AT3G52360| Symbols: | similar to unknown protein [Arabidopsis thaliana] (TAIR:AT2G35850.1); similar to unnar	-1.696
JCVI_41510	2.773	moderately similar to (270)AT5G19980| Symbols: | integral membrane family protein | chr5:6749909-6750934 REVERSE no original des	
EE419087	2.772	weakly similar to (184)AT3G02650| Symbols: | pentatricopeptide (PPR) repeat-containing protein | chr3:566278-569872 FORWARD [20	
JCVI_8225	2.772	moderately similar to (440)AT3G26290| Symbols: CYP71B26 | CYP71B26 (cytochrome P450, family 71, subfamily B, polypeptide 26); o	
JCVI_8428	2.771	nearly identical (1172)AT4G36760| Symbols: APP1, ATAPP1 | ATAPP1 (aminopeptidase P1) | chr4:17326727-17329461 FORWARD no	
JCVI_15147	2.771	weakly similar to (131)AT5G42050| Symbols: | similar to unknown protein [Arabidopsis thaliana] (TAIR:AT3G27090.1); similar to unkno	
JCVI_21348	2.771	no original description	
JCVI_5885	2.771	moderately similar to (205)AT4G27990| Symbols: | YGGT family protein | chr4:13923997-13924653 REVERSE no original description	
JCVI_33757	2.770	moderately similar to (218)AT1G69760| Symbols: | similar to unknown protein [Arabidopsis thaliana] (TAIR:AT1G26920.1); similar to h	
JCVI_2625	2.770	moderately similar to (468)AT3G02360| Symbols: | 6-phosphogluconate dehydrogenase family protein | chr3:482505-483965 FORWARD	
EV120350	2.770	moderately similar to (425)AT3G54420| Symbols: ATCHITIV, CHIV, ATEP3 | ATEP3 (Arabidopsis thaliana chitinase class IV); chitinase	
EG021120	2.769	very weakly similar to (80.9)AT1G67360| Symbols: | rubber elongation factor (REF) family protein | chr1:25240735-25241576 REVERSE	
JCVI_36249	2.769	weakly similar to (139)AT1G22970| Symbols: | protein binding | chr1:8130829-8132188 REVERSE no original description	
JCVI_15758	2.769	moderately similar to (379)AT5G17220| Symbols: GST26, TT19, ATGSTF12 | ATGSTF12 (GLUTATHIONE S-TRANSFERASE 26); glu	
DY014492	2.768	no similarity	
EE480029	2.768	moderately similar to (309)AT1G74090| Symbols: | sulfotransferase family protein | chr1:27866664-27867716 FORWARD [20132]	
JCVI_16355	2.768	moderately similar to (338)AT2G17265| Symbols: HSK | HSK (HOMOSERINE KINASE); homoserine kinase | chr2:7515688-7516800 FO	-2.184
EX019682	2.768	moderately similar to (357)AT4G29420| Symbols: | F-box family protein | chr4:14470721-14472210 REVERSE [21809]	
JCVI_8128	2.767	moderately similar to (298)AT3G50830| Symbols: ATCOR413-PM2 | COR413-PM2 (cold regulated 413 plasma membrane 2) | chr3:1890	
JCVI_24376	2.767	moderately similar to (296)AT3G27190| Symbols: | uracil phosphoribosyltransferase, putative / UMP pyrophosphorylase, putative / UPRT	
JCVI_22837	2.767	highly similar to (602)AT5G23300| Symbols: PYRD | PYRD (PYRIMIDINE D); dihydroorotate dehydrogenase | chr5:7847795-7850246 I	
EE431142	2.767	no similarity	
EV181200	2.767	weakly similar to (108)AT1G08050| Symbols: | zinc finger (C3HC4-type RING finger) family protein | chr1:2499085-2501308 REVERSE	
AM388629	2.767	moderately similar to (338)AT1G33110| Symbols: | MATE efflux family protein | chr1:12005064-12008020 FORWARD [20118]	
CN727352	2.766	very weakly similar to (83.2)GLNAC_BRANA [15722] 15 412 447	-1.793
CV546991	2.766	no similarity	
EV109814	2.766	weakly similar to (130)AT3G20770| Symbols: EIN3 | EIN3 (ETHYLENE-INSENSITIVE3); transcription factor | chr3:7260708-7262594 I	
JCVI_37237	2.765	moderately similar to (456)AT2G47390| Symbols: | serine-type endopeptidase/ serine-type peptidase | chr2:19449348-19453323 REVERS	
EV217149	2.764	no similarity	
JCVI_35839	2.764	weakly similar to (133)AT3G20890| Symbols: | RNA binding | chr3:7320224-7321060 FORWARD no original description	
JCVI_19232	2.763	moderately similar to (316)AT1G58030| Symbols: CAT2 | CAT2 (CATIONIC AMINO ACID TRANSPORTER 2); amino acid transmeml	
JCVI_3253	2.763	moderately similar to (246)AT5G49945| Symbols: | Identical to Uncharacterized protein At5g49945 precursor [Arabidopsis Thaliana] (GI	
DW998013	2.762	very weakly similar to (87.0)AT3G11630| Symbols: | 2-cys peroxiredoxin, chloroplast (BAS1) | chr3:3672195-3673943 FORWARDvery w	
JCVI_13181	2.761	moderately similar to (497)AT4G38020| Symbols: | tRNA/rRNA methyltransferase (SpoU) family protein | chr4:17861562-17863004 FOI	

EV135146	2.761	no similarity	
EV136083	2.761	no similarity	
JCVI_820	2.760	moderately similar to (348)AT3G61870| Symbols: | similar to unknown [Populus trichocarpa] (GB:ABK95718.1) | chr3:22913677-229148	
CV432604	2.760	weakly similar to (151)AT5G10840| Symbols: | endomembrane protein 70, putative | chr5:3424911-3427798 REVERSE [16490] 1 621 65	
JCVI_24428	2.760	very weakly similar to (89.7)AT2G42880| Symbols: ATMPK20 | ATMPK20 (Arabidopsis thaliana MAP kinase 20); MAP kinase | chr2:178	
JCVI_145	2.760	highly similar to (543)AT1G76680| Symbols: OPR1 | OPR1 (12-oxophytodienoate reductase 1); 12-oxophytodienoate reductase | chr1:287	
JCVI_37985	2.760	very weakly similar to (94.0)AT3G02730| Symbols: TRXF1, ATF1 | ATF1/TRXF1 (THIOREDOXIN F-TYPE 1); thiol-disulfide exchange	
JCVI_2079	2.760	moderately similar to (251)AT5G05140| Symbols: | transcription elongation factor-related | chr5:1520354-1522298 FORWARD no origina	
EE539425	2.760	moderately similar to (266)AT1G22700| Symbols: | tetratricopeptide repeat (TPR)-containing protein | chr1:8028312-8029278 REVERSE	
JCVI_991	2.760	moderately similar to (397)AT5G53400| Symbols: | nuclear movement family protein | chr5:21678814-21680609 FORWARD no original	
JCVI_35175	2.759	weakly similar to (107)AT1G67920| Symbols: | similar to unknown protein [Arabidopsis thaliana] (TAIR:AT1G24600.1) | chr1:25477394	
JCVI_14716	2.759	moderately similar to (262)AT4G20360| Symbols: AtRab8D, AtRABE1b | AtRABE1b/AtRab8D (Arabidopsis Rab GTPase homolog E1b)	
CX194724	2.759	moderately similar to (269)AT1G51610| Symbols: | cation efflux family protein / metal tolerance protein, putative (MTPc4) | chr1:191402	
JCVI_39460	2.758	moderately similar to (324)AT3G09180| Symbols: | similar to unnamed protein product [Vitis vinifera] (GB:CAO45433.1); contains doma	
EE415429	2.756	weakly similar to (157)AT3G29400| Symbols: ATEXO70E1 | ATEXO70E1 (exocyst subunit EXO70 family protein E1); protein binding |	
JCVI_38143	2.755	very weakly similar to (97.4)AT1G18640| Symbols: PSP | PSP (3-PHOSPHOSERINE PHOSPHATASE) | chr1:6416516-6418237 REVERS	
ES949725	2.755	weakly similar to (136)AT1G30380| Symbols: PSAK | PSAK (PHOTOSYSTEM I SUBUNIT K) | chr1:10722307-10722995 FORWARDw	
JCVI_10992	2.755	no original description	
JCVI_14140	2.755	moderately similar to (418)AT1G78140| Symbols: | methyltransferase-related | chr1:29406830-29408771 REVERSE no original descriptio	
JCVI_39868	2.755	highly similar to (861)AT2G30110| Symbols: MOS5, ATUBA1 | ATUBA1 (ARABIDOPSIS THALIANA UBIQUITIN-ACTIVATING EN	
JCVI_1334	2.754	weakly similar to (172)AT5G13930| Symbols: CHS, TT4, ATCHS | ATCHS/CHS/TT4 (CHALCONE SYNTHASE); naringenin-chalcone	
EX028455	2.753	moderately similar to (288)AT1G01050| Symbols: ATPPA1 | ATPPA1 (ARABIDOPSIS THALIANA PYROPHOSPHORYLASE 1); inorg	
EX136860	2.753	no similarity	
EX066154	2.753	moderately similar to (350)AT2G38400| Symbols: AGT3 | AGT3 (ALANINE:GLYOXYLATE AMINOTRANSFERASE 3); alanine-glyox	-3.396
EX091686	2.753	weakly similar to (105)AT5G50850| Symbols: MAB1 | MAB1 (MACCI-BOU); pyruvate dehydrogenase (acetyl-transferring) | chr5:20706	
JCVI_36023	2.752	no original description	
JCVI_35963	2.751	no original description	
DN965884	2.751	moderately similar to (217)AT2G04050| Symbols: | MATE efflux family protein | chr2:1337383-1339267 REVERSE [17359]	
ES946572	2.750	no similarity	
JCVI_40558	2.750	highly similar to (775)AT4G04610| Symbols: APR, PRH19, ATAPR1, APR1 | APR1 (PAPS REDUCTASE HOMOLOG 19) | chr4:23250	
JCVI_2294	2.750	highly similar to (525)AT1G32900| Symbols: | starch synthase, putative | chr1:11920562-11923486 REVERSEmoderately similar to (458	
JCVI_40692	2.750	weakly similar to (112)AT2G40830| Symbols: RHC1A | RHC1A (RING-H2 finger C1A); protein binding / zinc ion binding | chr2:1705072	
JCVI_33232	2.750	weakly similar to (178)AT5G39990| Symbols: | glycosyltransferase family 14 protein / core-2/I-branching enzyme family protein | chr5:16	
EE442066	2.748	weakly similar to (182)AT5G40240| Symbols: | nodulin MtN21 family protein | chr5:16099553-16101694 REVERSE [20164]	-2.814
AM391722	2.748	no similarity	
JCVI_1260	2.748	moderately similar to (274)AT1G72310| Symbols: ATL3 | ATL3 (Arabidopsis T?xicos en Levadura 3); protein binding / zinc ion binding |	
CD815377	2.747	weakly similar to (110)AT1G70770| Symbols: | similar to unknown protein [Arabidopsis thaliana] (TAIR:AT1G23170.1); similar to hypot	
EX102010	2.747	no similarity	
EE443035	2.746	weakly similar to (105)AT1G66100| Symbols: | thionin, putative | chr1:24609539-24610136 REVERSE [20160] 1 430 450	
JCVI_41455	2.746	no original description	
JCVI_15448	2.745	moderately similar to (356)AT4G15490| Symbols: UGT84A3 | UGT84A3; UDP-glycosyltransferase/ sinapate 1-glucosyltransferase/ transf	
JCVI_15152	2.745	moderately similar to (380)AT2G46100| Symbols: | similar to unknown protein [Arabidopsis thaliana] (TAIR:AT3G04890.2); similar to u	
EX122546	2.745	moderately similar to (284)AT2G33490| Symbols: | hydroxyproline-rich glycoprotein family protein | chr2:14190628-14194742 FORWAI	
JCVI_39654	2.745	moderately similar to (416)AT4G15093| Symbols: | catalytic LigB subunit of aromatic ring-opening dioxygenase family | chr4:8618451-86	
JCVI_31689	2.744	no original description	
JCVI_14278	2.744	highly similar to (547)AT1G20850| Symbols: XCP2 | XCP2 (XYLEM CYSTEINE PEPTIDASE 2); cysteine-type peptidase/ peptidase | ch	
EX118910	2.744	no similarity	
EX025904	2.744	moderately similar to (222)AT1G59730| Symbols: ATH7 | ATH7 (thioredoxin H-type 7); thiol-disulfide exchange intermediate | chr1:2195	
JCVI_16324	2.742	very weakly similar to (93.6)AT5G55135| Symbols: | unknown protein | chr5:22397185-22397612 FORWARD no original description	
ES906219	2.742	weakly similar to (161)AT1G15380| Symbols: | lactoylglutathione lyase family protein / glyoxalase I family protein | chr1:5290950-52922	-1.812
JCVI_14319	2.742	moderately similar to (303)AT5G39080| Symbols: | transferase family protein | chr5:15658909-15660300 FORWARD no original descrip	
JCVI_36209	2.742	no original description	
EV177030	2.742	moderately similar to (214)AT2G01670| Symbols: ATNUDT17 | ATNUDT17 (Arabidopsis thaliana Nudix hydrolase homolog 17); hydrol	
JCVI_16866	2.742	moderately similar to (321)AT2G45550| Symbols: CYP76C4 | CYP76C4 (cytochrome P450, family 76, subfamily C, polypeptide 4); oxyg	
JCVI_33772	2.742	moderately similar to (277)AT1G21750| Symbols: ATPDIL1-1 | ATPDIL1-1 (PDI-LIKE 1-1); protein disulfide isomerase | chr1:7645756-	
JCVI_27053	2.742	moderately similar to (438)AT4G38690| Symbols: | 1-phosphatidylinositol phosphodiesterase-related | chr4:18074737-18075693 REVERS	-2.417
CO749935	2.741	weakly similar to (136)AT5G64550| Symbols: | loricrin-related | chr5:25819020-25820924 REVERSE [16161]	
ES938639	2.739	no similarity	
DY019353	2.739	moderately similar to (345)AT4G11650| Symbols: ATOSM34 | ATOSM34 (OSMOTIN 34) | chr4:7025121-7026107 REVERSEmoderatel	
AM394730	2.738	weakly similar to (160)AT1G32520| Symbols: | similar to unnamed protein product [Vitis vinifera] (GB:CAO63428.1) | chr1:11758402-11	
JCVI_3747	2.738	moderately similar to (414)AT3G60970| Symbols: ATMRP15 | ATMRP15 (Arabidopsis thaliana multidrug resistance-associated protein 1	
JCVI_7383	2.738	highly similar to (567)AT1G75200| Symbols: | flavodoxin family protein / radical SAM domain-containing protein | chr1:28224510-28227	
JCVI_26494	2.737	moderately similar to (367)AT5G18520| Symbols: | similar to unknown protein [Arabidopsis thaliana] (TAIR:AT3G09570.1); similar to u	
EE456190	2.737	weakly similar to (190)AT5G58030| Symbols: | transport protein particle (TRAPP) component Bet3 family protein | chr5:23504247-23505	
JCVI_8150	2.736	moderately similar to (457)AT2G37110| Symbols: | similar to unknown protein [Arabidopsis thaliana] (TAIR:AT2G40935.1); similar to u	-2.478
JCVI_59	2.735	moderately similar to (411)AT5G23060| Symbols: CAS | similar to unknown protein [Arabidopsis thaliana] (TAIR:AT3G59780.1); similar	-1.388
EE452917	2.735	moderately similar to (220)AT1G72830| Symbols: ATHAP2C, HAP2, HAP2C | HAP2C (Heme activator protein (yeast) homolog 2C); tran	
JCVI_39176	2.735	moderately similar to (332)AT1G47230| Symbols: CYCA3;4 | CYCA3;4; cyclin-dependent protein kinase regulator | chr1:17309192-1731	
EX094228	2.734	weakly similar to (131)AT3G18850| Symbols: LPAT5 | LPAT5 | chr3:6499535-6500846 REVERSE [21823]	
JCVI_34165	2.733	moderately similar to (417)AT5G06570| Symbols: | hydrolase | chr5:2008076-2011014 REVERSEweakly similar to (121)GID1_ORYSA	
JCVI_26224	2.733	moderately similar to (320)AT5G05790| Symbols: | myb family transcription factor | chr5:1740725-1741672 REVERSE no original descri	
EX020740	2.732	weakly similar to (172)AT2G37770| Symbols: | aldo/keto reductase family protein | chr2:15841966-15843737 FORWARDvery weakly sin	-2.237
CD825199	2.731	moderately similar to (247)AT2G47115| Symbols: | similar to unknown protein [Arabidopsis thaliana] (TAIR:AT1G10660.3); similar to u	
AT000862	2.731	no similarity	
JCVI_15435	2.730	moderately similar to (311)AT1G47240| Symbols: ATNRAMP2, NRAMP2 | NRAMP2 (NRAMP metal ion transporter 2); metal ion transı	
JCVI_1882	2.729	moderately similar to (481)AT3G57890| Symbols: | tubulin-specific chaperone C-related | chr3:21449248-21452672 FORWARD no origi	-3.719
JCVI_10696	2.729	moderately similar to (302)AT1G10070| Symbols: ATBCAT-2 | ATBCAT-2; catalytic | chr1:3288674-3290166 FORWARD no original de	1.909
CD837111	2.728	moderately similar to (215)AT1G02850| Symbols: | glycosyl hydrolase family 1 protein | chr1:630569-633085 FORWARDweakly similar	
JCVI_19776	2.727	highly similar to (538)AT4G27790| Symbols: | calcium-binding EF hand family protein | chr4:13850072-13851494 FORWARD no origina	
JCVI_1607	2.726	moderately similar to (345)AT4G12560| Symbols: | F-box family protein | chr4:7441812-7443154 FORWARD no original description	
EV076456	2.726	weakly similar to (127)AT3G02990| Symbols: HSFA1E, ATHSFA1E | ATHSFA1E (Arabidopsis thaliana heat shock transcription factor A	

ES965233	2.726	no similarity	
JCVI_577	2.726	very weakly similar to (92.8)AT1G48620| Symbols: HON5 | HON5 (HIGH MOBILITY GROUP FAMILY A 5); DNA binding | chr1:1797[illegible]	
JCVI_16954	2.725	moderately similar to (388)AT1G76030| Symbols: | (VACUOLAR ATP SYNTHASE SUBUNIT B1); hydrogen ion transporting ATP syn[illegible]	
JCVI_9251	2.725	weakly similar to (200)AT3G15520| Symbols: | peptidyl-prolyl cis-trans isomerase TLP38, chloroplast / thylakoid lumen PPIase of 38 kDa[illegible]	
JCVI_6723	2.725	moderately similar to (406)AT5G06610| Symbols: | similar to unknown protein [Arabidopsis thaliana] (TAIR:AT1G27690.1); similar to u[illegible]	
JCVI_8424	2.725	moderately similar to (416)AT1G08940| Symbols: | phosphoglycerate/bisphosphoglycerate mutase family protein | chr1:2877697-287910[illegible]	
JCVI_1748	2.724	moderately similar to (442)AT1G80560| Symbols: | 3-isopropylmalate dehydrogenase, chloroplast, putative | chr1:30292725-30295018 F[illegible]	
CV650361	2.724	weakly similar to (160)AT5G23810| Symbols: AAP7 | AAP7 (amino acid permease 7) | chr5:8028464-8030141 FORWARD [16632] 1 30[illegible]	
JCVI_27144	2.724	moderately similar to (411)AT3G06850| Symbols: LTA1, DIN3, BCE2 | DIN3/LTA1 (DARK INDUCIBLE 3); alpha-ketoacid dehydrogen[illegible]	
JCVI_21062	2.723	highly similar to (784)AT5G19690| Symbols: STT3A | STT3A (STAUROSPORIN AND TEMPERATURE SENSITIVE 3-LIKE A); oligo	-2.085
EV181968	2.723	no similarity	
JCVI_27410	2.722	moderately similar to (338)AT4G37520| Symbols: | peroxidase 50 (PER50) (P50) (PRXR2) | chr4:17631698-17633054 FORWARDweak[illegible]	
EE439379	2.722	weakly similar to (140)AT1G67250| Symbols: | proteasome maturation factor UMP1 family protein | chr1:25167471-25168630 REVERS[illegible]	
JCVI_16900	2.720	moderately similar to (281)AT5G67030| Symbols: LOS6, NPQ2, ATABA1, ZEP, IBS3, ATZEP, ABA1 | ABA1 (ABA DEFICIENT 1); ze[illegible]	
JCVI_40242	2.720	weakly similar to (178)AT5G12230| Symbols: | similar to unknown protein [Arabidopsis thaliana] (TAIR:AT5G19480.1); similar to unkn[illegible]	
JCVI_4161	2.719	moderately similar to (230)AT1G72010| Symbols: | TCP family transcription factor, putative | chr1:27111506-27112633 FORWARD no [illegible]	
JCVI_41005	2.719	moderately similar to (314)AT5G26340| Symbols: STP13, MSS1 | MSS1 (SUGAR TRANSPORT PROTEIN 13); carbohydrate transmem[illegible]	
JCVI_35145	2.719	weakly similar to (137)AT5G47140| Symbols: | zinc finger (GATA type) family protein | chr5:19162335-19164530 FORWARD no origin[illegible]	
L37495	2.719	very weakly similar to (93.6)AT3G02090| Symbols: MPPBETA | MPPBETA; metalloendopeptidase | chr3:365631-368541 FORWARD [1[illegible]	-3.588
JCVI_3397	2.718	moderately similar to (381)AT2G46280| Symbols: TIF3I1, TRIP-1 | TRIP-1 (TGF-BETA RECEPTOR INTERACTING PROTEIN 1); nu[illegible]	2.799
AM060364	2.717	no similarity	
JCVI_26445	2.717	moderately similar to (366)AT1G62740| Symbols: | stress-inducible protein, putative | chr1:23234691-23237045 FORWARDmoderately s[illegible]	
JCVI_23871	2.717	highly similar to (829)AT4G08850| Symbols: | leucine-rich repeat family protein / protein kinase family protein | chr4:5637464-5640493 [illegible]	
JCVI_42522	2.717	moderately similar to (460)AT1G74680| Symbols: | exostosin family protein | chr1:28063189-28064645 FORWARD no original descripti[illegible]	
JCVI_3748	2.717	moderately similar to (366)AT2G23580| Symbols: | hydrolase, alpha/beta fold family protein | chr2:10040440-10041390 REVERSEweak[illegible]	
AM395626	2.716	weakly similar to (103)AT5G55740| Symbols: CRR21 | CRR21 (CHLORORESPIRATORY REDUCTION 21) | chr5:22579167-22581659	
JCVI_11922	2.716	moderately similar to (428)AT2G41530| Symbols: ATSFGH | ATSFGH (ARABIDOPSIS THALIANA S-FORMYLGLUTATHIONE HY[illegible]	
EV171128	2.716	weakly similar to (129)AT1G72290| Symbols: | trypsin and protease inhibitor family protein / Kunitz family protein | chr1:27219514-2722[illegible]	
JCVI_18478	2.715	moderately similar to (400)AT1G53990| Symbols: GLIP3 | GLIP3 (GDSL-motif lipase 3); carboxylesterase/ lipase | chr1:20154684-20156[illegible]	
JCVI_13747	2.715	moderately similar to (477)AT3G04830| Symbols: | binding | chr3:1326295-1329138 FORWARD no original description	
JCVI_5541	2.715	highly similar to (511)AT5G46290| Symbols: KAS I | KAS I (3-KETOACYL-ACYL CARRIER PROTEIN SYNTHASE I); fatty-acid synt[illegible]	
ES267277	2.714	moderately similar to (336)AT2G41800| Symbols: | similar to unknown protein [Arabidopsis thaliana] (TAIR:AT2G41810.1); similar to u[illegible]	
JCVI_16106	2.713	moderately similar to (457)AT5G22830| Symbols: GMN10, ATMGT10 | GMN10 (Arabidopsis thaliana Mg transporter 10) | chr5:762767[illegible]	
EH420554	2.713	no similarity	
JCVI_207	2.713	moderately similar to (464)AT1G69490| Symbols: ANAC029, ATNAP, NAP | NAP (NAC-LIKE, ACTIVATED BY AP3/PI); transcriptio[illegible]	
AM058289	2.713	moderately similar to (278)AT1G08050| Symbols: | zinc finger (C3HC4-type RING finger) family protein | chr1:2499085-2501308 REVE[illegible]	
JCVI_20803	2.713	moderately similar to (451)AT2G19880| Symbols: | ceramide glucosyltransferase, putative | chr2:8588660-8592200 FORWARD no origin[illegible]	
EX056642	2.713	moderately similar to (242)AT3G15300| Symbols: | VQ motif-containing protein | chr3:5147558-5148217 REVERSE [21813]	
JCVI_31537	2.713	moderately similar to (432)AT2G41540| Symbols: GPDHC1 | GPDHC1; glycerol-3-phosphate dehydrogenase (NAD+) | chr2:17333879-1[illegible]	
EV017026	2.713	weakly similar to (107)AT1G15670| Symbols: | kelch repeat-containing F-box family protein | chr1:5390114-5391193 FORWARD [2144[illegible]	
EE567335	2.712	weakly similar to (103)AT1G60720| Symbols: | similar to unknown protein [Arabidopsis thaliana] (TAIR:AT1G33710.1); similar to putat[illegible]	
JCVI_19780	2.712	moderately similar to (212)AT5G13610| Symbols: | similar to unknown protein [Arabidopsis thaliana] (TAIR:AT1G69380.1); similar to u[illegible]	
JCVI_9377	2.712	moderately similar to (211)AT3G20310| Symbols: ATERF-7, ATERF7, ERF7 | ATERF-7/ATERF7/ERF7 (ETHYLINE RESPONSE FAC[illegible]	
EV173241	2.710	moderately similar to (284)AT3G27970| Symbols: | exonuclease | chr3:10390846-10392781 FORWARD [21486] 79 865 865	
JCVI_31373	2.710	highly similar to (631)AT5G54200| Symbols: | WD-40 repeat family protein | chr5:22010791-22014302 REVERSE no original descriptio[illegible]	
EV219985	2.710	no similarity	1.149
JCVI_36722	2.709	moderately similar to (473)AT1G55590| Symbols: | F-box family protein | chr1:20773142-20775422 REVERSE no original description	
JCVI_33175	2.709	moderately similar to (221)AT1G58470| Symbols: RBP1, XF41, ATRBP1 | ATRBP1 (ARABIDOPSIS THALIANA RNA-BINDING PR[illegible]	
JCVI_11495	2.709	moderately similar to (404)AT4G24740| Symbols: AME1, AFC2 | AFC2 (ARABIDOPSIS FUS3-COMPLEMENTING GENE 1); kinase | [illegible]	
JCVI_2627	2.709	moderately similar to (305)AT5G64090| Symbols: | similar to unknown protein [Arabidopsis thaliana] (TAIR:AT5G21050.1); similar to u[illegible]	
ES908452	2.708	moderately similar to (338)AT5G63410| Symbols: | leucine-rich repeat transmembrane protein kinase, putative | chr5:25412399-2541499[illegible]	
JCVI_32995	2.708	moderately similar to (297)AT2G20780| Symbols: | mannitol transporter, putative | chr2:8954577-8956251 REVERSE no original descrip[illegible]	
EE532464	2.707	no similarity	
JCVI_6152	2.707	moderately similar to (434)AT5G22060| Symbols: ATJ2 | ATJ2 (Arabidopsis thaliana DnaJ homologue 2) | chr5:7303801-7305671 REVE[illegible]	
JCVI_8093	2.706	moderately similar to (350)AT3G44330| Symbols: | similar to hypothetical protein OsJ_024705 [Oryza sativa (japonica cultivar-group)] ([illegible]	
EV049288	2.705	weakly similar to (169)AT3G09430| Symbols: | similar to unnamed protein product [Vitis vinifera] (GB:CAO15639.1) | chr3:2901286-29[illegible]	
JCVI_5975	2.704	moderately similar to (355)AT2G34250| Symbols: | protein transport protein sec61, putative | chr2:14469714-14471651 FORWARD no o[illegible]	
JCVI_34682	2.704	weakly similar to (121)AT1G12710| Symbols: ATPP2-A12 | ATPP2-A12 (PHLOEM PROTEIN 2-A12); carbohydrate binding | chr1:4326[illegible]	
EX076183	2.704	no similarity	
DW998114	2.703	moderately similar to (406)AT4G16590| Symbols: CSLA01, ATCSLA1, ATCSLA01 | ATCSLA01 (Cellulose synthase-like A1); glucosylt[illegible]	
JCVI_1936	2.703	moderately similar to (385)AT4G18040| Symbols: EIF4E, LSP1, CUM1, AT.EIF4E1 | EIF4E (EUKARYOTIC TRANSLATION INITATI[illegible]	
JCVI_4813	2.703	moderately similar to (416)AT4G15130| Symbols: | cholinephosphate cytidylyltransferase, putative / phosphorylcholine transferase, putati	-1.272
JCVI_2238	2.702	moderately similar to (407)AT1G53320| Symbols: AtTLP7 | AtTLP7 (TUBBY LIKE PROTEIN 7); phosphoric diester hydrolase/ transcri[illegible]	
JCVI_18824	2.702	moderately similar to (439)AT2G43820| Symbols: GT, UGT74F2 | GT/UGT74F2 (UDP-GLUCOSYLTRANSFERASE 74F2); UDP-gluco[illegible]	
JCVI_16852	2.702	moderately similar to (446)AT1G34780| Symbols: ATAPRL4 | ATAPRL4 (APR-LIKE 4) | chr1:12748813-12750102 REVERSE no origin[illegible]	
EX136215	2.702	weakly similar to (111)AT5G27840| Symbols: TOPP8 | TOPP8 (Type one serine/threonine protein phosphatase 8); protein serine/threonin[illegible]	
JCVI_3573	2.702	moderately similar to (359)AT1G58030| Symbols: CAT2 | CAT2 (CATIONIC AMINO ACID TRANSPORTER 2); amino acid transmem[illegible]	
JCVI_16505	2.701	weakly similar to (159)AT5G13340| Symbols: | similar to F-box family protein [Arabidopsis thaliana] (TAIR:AT1G10890.1); similar to u[illegible]	
ES966664	2.701	no similarity	
JCVI_21572	2.701	moderately similar to (263)AT1G72160| Symbols: | SEC14 cytosolic factor family protein / phosphoglyceride transfer family protein | chr[illegible]	
JCVI_22924	2.700	no original description	
AM385064	2.700	moderately similar to (269)AT4G13350| Symbols: | human Rev interacting-like protein-related / hRIP protein-related | chr4:7770166-777[illegible]	
JCVI_12903	2.700	moderately similar to (221)AT5G44050| Symbols: | MATE efflux family protein | chr5:17739711-17743436 FORWARD no original desc[illegible]	
JCVI_34065	2.699	weakly similar to (159)AT5G18980| Symbols: | binding | chr5:6334946-6337945 REVERSE no original description	
JCVI_12027	2.699	no original description	-2.052
JCVI_37303	2.699	moderately similar to (373)AT1G64890| Symbols: | integral membrane transporter family protein | chr1:24113415-24114828 FORWARD	
JCVI_38855	2.698	moderately similar to (399)AT2G25620| Symbols: | protein phosphatase 2C, putative / PP2C, putative | chr2:10910232-10912056 REVER[illegible]	
JCVI_5723	2.698	moderately similar to (348)AT5G36160| Symbols: | aminotransferase-related | chr5:14250491-14252359 REVERSE no original descriptio[illegible]	
JCVI_9870	2.698	moderately similar to (297)AT4G31300| Symbols: PBA1 | PBA1 (20S proteasome beta subunit A 1); peptidase | chr4:15188933-15190941	
JCVI_26460	2.698	moderately similar to (245)AT5G19750| Symbols: | peroxisomal membrane 22 kDa family protein | chr5:6677126-6679129 FORWARD n[illegible]	
EV160597	2.697	moderately similar to (290)AT1G01740| Symbols: | protein kinase family protein | chr1:272111-274239 REVERSE [21484]	

JCVI_25577	2.697	highly similar to (679)AT5G54250| Symbols: CNGC4, HLM1, DND2, ATCNGC4 | ATCNGC4 (DEFENSE, NO DEATH 2); calmodulin b	
JCVI_6631	2.696	highly similar to (835)AT4G10840| Symbols: | kinesin light chain-related | chr4:6656610-6658698 FORWARD no original description	
JCVI_16952	2.696	moderately similar to (316)AT4G12560| Symbols: | F-box family protein | chr4:7441812-7443154 FORWARD no original description	
JCVI_3470	2.695	moderately similar to (326)AT4G17830| Symbols: | peptidase M20/M25/M40 family protein | chr4:9915929-9918062 FORWARD no orig	
AM062099	2.695	moderately similar to (285)AT3G06470| Symbols: | GNS1/SUR4 membrane family protein | chr3:1984212-1985048 FORWARD [17712]	
EX042285	2.694	no similarity	
JCVI_3692	2.694	moderately similar to (352)AT5G11150| Symbols: VAMP713, ATVAMP713 | ATVAMP713 (Arabidopsis thaliana vesicle-associated mer	
JCVI_7189	2.693	moderately similar to (351)AT1G77590| Symbols: LACS9 | LACS9 (LONG CHAIN ACYL-COA SYNTHETASE 9); long-chain-fatty-acid	
EE452160	2.692	moderately similar to (242)AT2G34460| Symbols: | flavin reductase-related | chr2:14536714-14537811 FORWARD [20194]	
CD813053	2.692	moderately similar to (208)AT4G29670| Symbols: | thioredoxin family protein | chr4:14535989-14537114 REVERSE [13977]	
JCVI_2462	2.691	moderately similar to (366)AT3G47810| Symbols: MAG1 | MAG1 (MAIGO 1); protein serine/threonine phosphatase | chr3:17648241-176	-1.608
CV433876	2.691	very weakly similar to (95.1)AT5G14330| Symbols: | unknown protein | chr5:4619950-4620554 REVERSE [16490]	
AM394310	2.691	moderately similar to (249)AT5G60410| Symbols: ATSIZ1, SIZ1 | ATSIZ1/SIZ1 | chr5:24312452-24318018 FORWARD [20346]	2.194
EV035294	2.690	no similarity	
EE515211	2.690	moderately similar to (314)AT1G12350| Symbols: ATCOAB | ATCOAB (4-PHOSPHO-PANTO-THENOYLCYSTEINE SYNTHETASE)	
JCVI_22603	2.690	moderately similar to (436)AT1G76320| Symbols: FRS4 | FRS4 (FAR1-RELATED SEQUENCE 4); zinc ion binding | chr1:28636298-286	
JCVI_39667	2.690	moderately similar to (479)AT4G21585| Symbols: ENDO4 | ENDO4 (ENDONUCLEASE 4); T/G mismatch-specific endonuclease/ endon	
JCVI_9297	2.690	moderately similar to (366)AT5G04180| Symbols: | carbonic anhydrase family protein | chr5:1147908-1149238 REVERSE no original des	
AM056872	2.690	no similarity	
ES953651	2.690	very weakly similar to (81.6)AT5G04350| Symbols: | self-incompatibility protein-related | chr5:1220830-1221270 FORWARD [21423]	
JCVI_7825	2.690	moderately similar to (342)AT5G42420| Symbols: | transporter-related | chr5:16986047-16987453 FORWARD no original description	
JCVI_9727	2.689	weakly similar to (169)AT3G08040| Symbols: MAN1, FRD3 | FRD3 (FERRIC REDUCTASE DEFECTIVE 3); antiporter | chr3:2566599-	
JCVI_5567	2.689	moderately similar to (343)AT2G46170| Symbols: | reticulon family protein (RTNLB5) | chr2:18972483-18973559 FORWARD no origina	
EV116601	2.689	moderately similar to (431)AT1G80320| Symbols: | oxidoreductase, 2OG-Fe(II) oxygenase family protein | chr1:30201674-30202788 FOF	-4.232
ES980563	2.688	moderately similar to (351)AT1G12600| Symbols: | similar to ATUTR2/UTR2 (UDP-GALACTOSE TRANSPORTER 2) [Arabidopsis tha	
JCVI_829	2.688	moderately similar to (292)AT1G64770| Symbols: | carbohydrate binding / catalytic | chr1:24061212-24062228 FORWARD no original de	
JCVI_37258	2.687	weakly similar to (136)AT1G19000| Symbols: | myb family transcription factor | chr1:6561326-6562675 REVERSE no original descriptio	
JCVI_39200	2.687	very weakly similar to (96.3)AT3G28690| Symbols: | protein kinase, putative | chr3:10756718-10758731 FORWARD no original descriptio	2.105
JCVI_17561	2.687	highly similar to (661)AT1G76850| Symbols: SEC5A | SEC5A (EXOCYST COMPLEX COMPONENT SEC5) | chr1:28852907-2885917(	
EE439352	2.687	very weakly similar to (80.1)AT5G13490| Symbols: AAC2 | AAC2 (ADP/ATP CARRIER 2); binding | chr5:4336037-4337382 FORWARD	
EE474627	2.687	no similarity	
JCVI_965	2.686	moderately similar to (468)AT1G23800| Symbols: ALDH2B, ALDH2B7 | ALDH2B7 (Aldehyde dehydrogenase 2B7); 3-chloroallyl aldehy	
JCVI_9756	2.685	moderately similar to (363)AT1G14720| Symbols: EXGT-A2, XTR2 | XTR2 (XYLOGLUCAN ENDOTRANSGLYCOSYLASE RELATE	
EE439115	2.685	weakly similar to (170)AT4G05410| Symbols: | transducin family protein / WD-40 repeat family protein | chr4:2743226-2745518 REVER	
JCVI_28258	2.685	moderately similar to (313)AT2G28930| Symbols: APK1B | APK1B (Arabidopsis protein kinase 1B); kinase | chr2:12431628-12433642 F(	
EE459209	2.684	very weakly similar to (92.0)AT5G03900| Symbols: | Identical to Uncharacterized protein At5g03900 precursor [Arabidopsis Thaliana] (G	
JCVI_23208	2.684	moderately similar to (491)AT1G35190| Symbols: | oxidoreductase, 2OG-Fe(II) oxygenase family protein | chr1:12890522-12892610 FOF	
CD813547	2.683	moderately similar to (254)AT3G26170| Symbols: CYP71B19 | CYP71B19 (cytochrome P450, family 71, subfamily B, polypeptide 19); o	
CX189022	2.683	moderately similar to (222)AT5G35220| Symbols: EGY1 | EGY1 (ETHYLENE-DEPENDENT GRAVITROPISM-DEFICIENT AND YEI	
CD812314	2.683	no similarity	
EE474581	2.682	weakly similar to (118)AT3G12320| Symbols: | similar to unknown protein [Arabidopsis thaliana] (TAIR:AT5G06980.1); similar to hypot	
JCVI_19649	2.682	weakly similar to (149)AT3G46020| Symbols: | RNA-binding protein, putative | chr3:16923496-16924235 REVERSE no original descript	
JCVI_27751	2.681	moderately similar to (250)AT2G39290| Symbols: PGPS1, PGP1, PGS1 | PGP1/PGPS1/PGS1 (PHOSPHATIDYLGLYCEROLPHOSPHA	-2.891
JCVI_9415	2.681	moderately similar to (219)AT1G65090| Symbols: | similar to unknown protein [Arabidopsis thaliana] (TAIR:AT5G36100.1); similar to u	
JCVI_26261	2.681	moderately similar to (267)AT1G15700| Symbols: ATPC2 | ATPC2 (ATP synthase gamma chain 2) | chr1:5402624-5403784 REVERSEm	
JCVI_5968	2.681	moderately similar to (385)AT1G15700| Symbols: ATPC2 | ATPC2 (ATP synthase gamma chain 2) | chr1:5402624-5403784 REVERSEm	
EX061091	2.681	weakly similar to (107)AT5G22120| Symbols: | nucleotide binding | chr5:7334615-7336758 REVERSE [21813]	
EE426243	2.680	no similarity	
JCVI_17869	2.680	moderately similar to (476)AT2G01720| Symbols: | ribophorin I family protein | chr2:317192-320015 REVERSE no original description	
JCVI_26321	2.680	moderately similar to (336)AT2G21270| Symbols: | ubiquitin fusion degradation UFD1 family protein | chr2:9114922-9117093 FORWAR	
JCVI_14253	2.680	moderately similar to (357)AT3G23760| Symbols: | similar to transferase, transferring glycosyl groups [Arabidopsis thaliana] (TAIR:AT4	
AM390334	2.679	no similarity	
JCVI_41419	2.679	moderately similar to (269)AT2G41810| Symbols: | similar to unknown protein [Arabidopsis thaliana] (TAIR:AT2G41800.1); similar to u	-1.116
EE516867	2.679	moderately similar to (248)AT4G20890| Symbols: TUB9 | TUB9 (tubulin beta-9 chain); structural molecule | chr4:11182229-11183851 FC	
JCVI_4555	2.679	moderately similar to (377)AT2G27530| Symbols: PGY1 | 60S ribosomal protein L10A (RPL10aB) | chr2:11770520-11771647 REVERSE	
JCVI_27038	2.678	moderately similar to (374)AT3G04080| Symbols: ATAPY1 | ATAPY1 (APYRASE 1); calmodulin binding | chr3:1068075-1070924 REV	
JCVI_33635	2.678	highly similar to (674)AT3G09020| Symbols: | alpha 1,4-glycosyltransferase family protein / glycosyltransferase sugar-binding DXD moti	
JCVI_9965	2.678	weakly similar to (102)AT3G61980| Symbols: | serine protease inhibitor, Kazal-type family protein | chr3:22967319-22967672 REVERSE	
DY006582	2.678	weakly similar to (123)AT2G34925| Symbols: CLE42 | CLE42 (CLAVATA3/ESR-RELATED 42) | chr2:14741349-14741615 FORWARD	
JCVI_15413	2.677	moderately similar to (255)AT3G19280| Symbols: ATFUT11, FUCT1, FUCTA, FUT11 | FUT11 (Fucosyltransferase 11); 4-galactosyl-N-a	
AM391952	2.677	moderately similar to (333)AT1G07520| Symbols: | scarecrow transcription factor family protein | chr1:2309715-2311802 REVERSEvery	
JCVI_1300	2.677	highly similar to (538)AT4G33360| Symbols: | terpene cyclase/mutase-related | chr4:16068126-16069377 REVERSEvery weakly similar t	
CV545939	2.677	weakly similar to (122)AT5G20010| Symbols: RAN1, ATRAN1, RAN-1 | RAN-1 (RAS RELATED NUCLEAR PROTEIN); GTP binding	
EV161825	2.677	moderately similar to (203)AT3G08030| Symbols: | similar to unknown protein [Arabidopsis thaliana] (TAIR:AT2G41800.1); similar to u	
CV547055	2.676	no similarity	
AM059885	2.676	weakly similar to (174)AT1G06870| Symbols: | signal peptidase, putative | chr1:2108831-2110641 FORWARD [17712]	
JCVI_33990	2.676	moderately similar to (416)AT5G62650| Symbols: | similar to hypothetical protein OsI_000940 [Oryza sativa (indica cultivar-group)] (GB	
EX132676	2.675	weakly similar to (103)AT5G19780| Symbols: TUA5 | TUA5 (tubulin alpha-5) | chr5:6687214-6688928 FORWARDweakly similar to (10	
JCVI_13224	2.674	moderately similar to (427)AT3G29160| Symbols: SNRK1.2, AKIN11 | AKIN11 (ARABIDOPSIS SNF1 KINASE HOMOLOG 11); prote	
JCVI_29515	2.674	no original description	
JCVI_39712	2.674	moderately similar to (259)AT1G51950| Symbols: IAA18 | IAA18 (indoleacetic acid-induced protein 18); transcription factor | chr1:19309	
JCVI_22632	2.674	no original description	
CN737725	2.673	moderately similar to (208)AT4G18975| Symbols: | pentatricopeptide (PPR) repeat-containing protein | chr4:10392181-10393676 REVEF	
EE541499	2.673	no similarity	
EX133519	2.673	weakly similar to (182)AT1G71880| Symbols: ATSUC1, SUC1 | SUC1 (SUCROSE-PROTON SYMPORTER 1); carbohydrate transmemb	
JCVI_10924	2.672	highly similar to (522)AT1G56600| Symbols: ATGOLS2 | ATGOLS2 (ARABIDOPSIS THALIANA GALACTINOL SYNTHASE 2); tran	
JCVI_39359	2.672	moderately similar to (306)AT3G05630| Symbols: PDLZ2, PLDP2 | PLDP2 (PHOSPHOLIPASE D ZETA 2); phospholipase D | chr3:163	
JCVI_41223	2.672	moderately similar to (421)AT5G67250| Symbols: VFB4, SKIP2 | SKIP2 (SKP1 INTERACTING PARTNER 2); ubiquitin-protein ligase |	1.584
JCVI_17312	2.671	moderately similar to (288)AT1G27000| Symbols: | bZIP family transcription factor | chr1:9374055-9376409 FORWARD no original desc	
EX079191	2.671	no similarity	
JCVI_26888	2.670	moderately similar to (320)AT3G54970| Symbols: | catalytic | chr3:20379550-20381047 REVERSE no original description	

JCVI_33476	2.670	moderately similar to (461)AT5G19550| Symbols: AAT2, ASP2 | ASP2 (ASPARTATE AMINOTRANSFERASE 2) | chr5:6598203-66015	
EE541186	2.670	weakly similar to (179)AT1G27385| Symbols: | similar to unnamed protein product [Vitis vinifera] (GB:CAO66165.1); contains InterPro	
DN964670	2.670	weakly similar to (131)AT2G44080| Symbols: ARL | ARL (ARGOS-LIKE) | chr2:18244802-18245209 FORWARD [17359]	
JCVI_12712	2.669	moderately similar to (451)AT1G07280| Symbols: | binding | chr1:2238504-2240990 FORWARD no original description	
EE438841	2.669	weakly similar to (102)AT3G19553| Symbols: | amino acid permease family protein | chr3:6790994-6792513 REVERSE [20173] 1 329 42	
AM390021	2.669	moderately similar to (288)AT5G60230| Symbols: ATSEN2, SEN2 | SEN2 (SPLICING ENDONUCLEASE 2); tRNA-intron endonuclease	
JCVI_17625	2.669	moderately similar to (271)AT1G62370| Symbols: | zinc finger (C3HC4-type RING finger) family protein | chr1:23076240-23076854 FOI	
JCVI_22602	2.668	nearly identical (1085)AT5G26710| Symbols: | glutamate-tRNA ligase, putative / glutamyl-tRNA synthetase, putatuve / GluRS, putative | c	
EV035571	2.668	weakly similar to (177)AT3G19550| Symbols: | similar to hypothetical protein [Vitis vinifera] (GB:CAN60715.1) | chr3:6787468-6788171	
JCVI_10647	2.668	moderately similar to (367)AT5G26280| Symbols: | meprin and TRAF homology domain-containing protein / MATH domain-containing	
JCVI_25546	2.668	weakly similar to (161)AT3G13790| Symbols: ATCWINV1, ATBFRUCT1 | ATBFRUCT1/ATCWINV1 (ARABIDOPSIS THALIANA CI	
JCVI_37875	2.667	no original description	
JCVI_27302	2.667	highly similar to (924)AT5G63840| Symbols: RSW3 | RSW3 (RADIAL SWELLING 3); hydrolase, hydrolyzing O-glycosyl compounds | cl	4.581
JCVI_5985	2.666	moderately similar to (318)AT5G23340| Symbols: | protein binding | chr5:7856317-7857986 FORWARD no original description	
JCVI_11014	2.666	weakly similar to (176)AT4G17910| Symbols: | zinc finger (C3HC4-type RING finger) family protein / pentatricopeptide (PPR) repeat-co	
JCVI_5628	2.666	weakly similar to (177)AT5G04170| Symbols: | calcium-binding EF hand family protein | chr5:1145580-1147520 FORWARD no original	
JCVI_16603	2.665	weakly similar to (192)AT4G32570| Symbols: TIFY8 | TIFY8 | chr4:15716439-15718708 REVERSE no original description	
EV220668	2.664	no similarity	
RC_EE560455	2.664	no similarity	-1.847
JCVI_26641	2.664	highly similar to (677)AT1G65060| Symbols: 4CL3 | 4CL3 (4-coumarate:CoA ligase 3); 4-coumarate-CoA ligase | chr1:24171590-241751	
EE469282	2.664	no similarity	
JCVI_9429	2.664	moderately similar to (354)AT1G71940| Symbols: | similar to unknown protein [Arabidopsis thaliana] (TAIR:AT4G09580.1); similar to u	
DN961661	2.663	no similarity	
JCVI_6273	2.663	moderately similar to (496)AT2G24200| Symbols: | cytosol aminopeptidase | chr2:10294097-10296530 REVERSEmoderately similar to (	
ES988352	2.663	weakly similar to (107)AT4G37260| Symbols: MYB73, AtMYB73 | AtMYB73/MYB73 (myb domain protein 73); DNA binding / transcrip	
JCVI_35883	2.663	very weakly similar to (91.7)AT5G53710| Symbols: | unknown protein | chr5:21822635-21822979 REVERSE no original description	
EV115188	2.663	moderately similar to (412)AT3G22060| Symbols: | receptor protein kinase-related | chr3:7771072-7772144 FORWARD [21479] 43 884 8	
JCVI_1700	2.663	moderately similar to (324)AT5G58070| Symbols: | lipocalin, putative | chr5:23517738-23518382 REVERSE no original description	
EE565426	2.663	weakly similar to (160)AT3G61700| Symbols: | similar to unknown protein [Arabidopsis thaliana] (TAIR:AT2G46420.1); similar to unnai	-3.235
JCVI_3457	2.662	moderately similar to (371)AT1G08370| Symbols: DCP1 | DCP1 (DECAPPING 1); m7G(5')pppN diphosphatase/ protein homodimerizatic	
JCVI_28637	2.662	moderately similar to (328)AT4G20760| Symbols: | short-chain dehydrogenase/reductase (SDR) family protein | chr4:11129054-1113055	
ES938739	2.662	weakly similar to (147)AT4G08350| Symbols: | KOW domain-containing transcription factor family protein | chr4:5286348-5292069 FOR	
JCVI_41902	2.661	weakly similar to (186)AT1G67940| Symbols: ATNAP3 | ATNAP3 (Arabidopsis thaliana non-intrinsic ABC protein 3) | chr1:25481468-25	
JCVI_4301	2.661	highly similar to (667)AT3G13235| Symbols: | ubiquitin family protein | chr3:4271499-4274355 REVERSE no original description	
JCVI_5768	2.661	very weakly similar to (97.1)AT1G76810| Symbols: | eukaryotic translation initiation factor 2 family protein / eIF-2 family protein | chr1:2	
EE435950	2.660	no similarity	
JCVI_17452	2.660	highly similar to (502)AT3G19630| Symbols: | radical SAM domain-containing protein | chr3:6818682-6820680 REVERSE no original de	
JCVI_21241	2.660	moderately similar to (296)AT4G26340| Symbols: | F-box family protein | chr4:13324139-13325568 FORWARD no original description	-2.476
CD818904	2.660	moderately similar to (327)AT5G61780| Symbols: | tudor domain-containing protein / nuclease family protein | chr5:24839238-24843867	
JCVI_10648	2.659	moderately similar to (228)AT4G39940| Symbols: AKN2 | AKN2 (APS-KINASE 2); ATP binding / kinase/ transferase, transferring phosp	-2.877
EX089345	2.659	moderately similar to (242)AT3G53490| Symbols: | similar to unknown protein [Arabidopsis thaliana] (TAIR:AT5G02720.1); similar to C	
JCVI_3314	2.659	weakly similar to (186)AT2G43050| Symbols: ATPMEPCRD | ATPMEPCRD; pectinesterase | chr2:17909585-17911251 FORWARD no	
EX037727	2.658	weakly similar to (115)AT3G56170| Symbols: CAN | CAN (CA-2+ DEPENDENT NUCLEASE); nuclease | chr3:20853593-20855298 FOI	
EV178498	2.657	no similarity	
JCVI_2115	2.657	moderately similar to (282)AT1G45233| Symbols: | similar to unknown protein [Arabidopsis thaliana] (TAIR:AT5G42920.2); similar to u	
JCVI_33233	2.657	moderately similar to (269)AT3G06410| Symbols: | nucleic acid binding | chr3:1947477-1949534 REVERSEweakly similar to (170)ZFNI	
JCVI_32403	2.657	moderately similar to (459)AT1G20560| Symbols: | AMP-dependent synthetase and ligase family protein | chr1:7119917-7121353 REVEI	
JCVI_1662	2.656	moderately similar to (312)AT5G59500| Symbols: | protein-S-isoprenylcysteine O-methyltransferase | chr5:24003250-24004440 FORWA	
JCVI_3315	2.656	moderately similar to (330)AT5G65840| Symbols: | similar to antioxidant/ oxidoreductase [Arabidopsis thaliana] (TAIR:AT2G37240.1); s	
JCVI_16993	2.655	highly similar to (792)AT1G47840| Symbols: | hexokinase, putative | chr1:17618683-17621299 REVERSEhighly similar to (579)HXK2_	
DT317726	2.655	no similarity	
JCVI_21298	2.654	moderately similar to (303)AT2G45690| Symbols: SSE, PEX16, SSE1 | SSE1 (SHRUNKEN SEED 1) | chr2:18830539-18832675 REVER	
JCVI_13798	2.654	moderately similar to (463)AT5G40780| Symbols: LTH1, LHT1 | LHT1 (LYSINE HISTIDINE TRANSPORTER 1); amino acid transmen	
BG543655	2.654	very weakly similar to (84.7)AT4G21580| Symbols: | oxidoreductase, zinc-binding dehydrogenase family protein | chr4:11475834-1147752	
JCVI_1982	2.653	weakly similar to (186)AT3G29575| Symbols: | similar to TMAC2 (TWO OR MORE ABRES-CONTAINING GENE 2) [Arabidopsis thal	
JCVI_15288	2.653	moderately similar to (264)AT4G24460| Symbols: | similar to unknown protein [Arabidopsis thaliana] (TAIR:AT5G19380.1); similar to u	
EE527198	2.653	moderately similar to (220)AT3G52380| Symbols: PDE322, CP33 | CP33 (PIGMENT DEFECTIVE 322); RNA binding | chr3:19432597-1	
JCVI_19693	2.653	weakly similar to (156)AT1G42990| Symbols: ATBZIP60 | ATBZIP60 (BASIC REGION/LEUCINE ZIPPER MOTIF 60); DNA binding /	
ES932177	2.653	weakly similar to (112)AT3G56250| Symbols: | unknown protein | chr3:20877099-20878819 FORWARD [20143]	
JCVI_8909	2.652	moderately similar to (312)AT5G58950| Symbols: | protein kinase family protein | chr5:23818362-23820251 REVERSE no original descri	
JCVI_15568	2.652	moderately similar to (451)AT3G61960| Symbols: | protein kinase family protein | chr3:22952941-22955971 REVERSE no original descri	2.065
ES946497	2.652	very weakly similar to (88.2)AT5G18400| Symbols: | similar to unknown protein [Arabidopsis thaliana] (TAIR:AT5G18362.1); similar to	
JCVI_6497	2.652	moderately similar to (327)AT4G17260| Symbols: | L-lactate dehydrogenase, putative | chr4:9674070-9675322 FORWARDmoderately si	
JCVI_2299	2.652	moderately similar to (394)AT3G61600| Symbols: ATPOB1 | ATPOB1 (Arabidopsis thaliana POZ/BTB containing-protein 1); protein bin	
JCVI_27911	2.651	moderately similar to (416)AT2G36800| Symbols: UGT73C5, DOGT1 | DOGT1 (DON-GLUCOSYLTRANSFERASE); UDP-glycosyltran	
JCVI_2239	2.651	weakly similar to (196)AT5G63590| Symbols: FLS | FLS (Flavonol synthase); flavonol synthase | chr5:25474398-25475653 REVERSEwe	-1.719
JCVI_28879	2.651	weakly similar to (109)AT3G03550| Symbols: | zinc finger (C3HC4-type RING finger) family protein | chr3:850398-851468 REVERSE n	
EV211746	2.650	no similarity	-3.294
JCVI_20682	2.650	moderately similar to (455)AT2G44160| Symbols: MTHFR2 | MTHFR2 (METHYLENETETRAHYDROFOLATE REDUCTASE 2); metl	
JCVI_24056	2.650	weakly similar to (129)AT1G22550| Symbols: | proton-dependent oligopeptide transport (POT) family protein | chr1:7966597-7968541 RI	
EX114325	2.649	weakly similar to (132)AT1G14010| Symbols: | emp24/gp25L/p24 family protein | chr1:4800382-4801787 REVERSE [21827]	
JCVI_9668	2.649	moderately similar to (451)AT3G25860| Symbols: PLE2, LTA2 | LTA2 (PLASTID E2 SUBUNIT OF PYRUVATE DECARBOXYLASE)	
JCVI_3702	2.649	moderately similar to (443)AT4G24820| Symbols: | 26S proteasome regulatory subunit, putative (RPN7) | chr4:12790481-12792609 REV	
CK991390	2.649	weakly similar to (159)AT3G55440| Symbols: TPI, ATCTIMC | ATCTIMC (CYTOSOLIC TRIOSE PHOSPHATE ISOMERASE); triose	
JCVI_1382	2.648	moderately similar to (447)AT1G43190| Symbols: | polypyrimidine tract-binding protein, putative / heterogeneous nuclear ribonucleoprot	
CD836959	2.648	weakly similar to (160)AT4G36195| Symbols: | serine carboxypeptidase S28 family protein | chr4:17127205-17129790 FORWARD [1398	
JCVI_7829	2.647	moderately similar to (312)AT2G01620| Symbols: MEE11 | MEE11 (maternal effect embryo arrest 11) | chr2:278203-279225 FORWARD	
EE441260	2.646	moderately similar to (302)AT5G26010| Symbols: | protein serine/threonine phosphatase | chr5:9085515-9087375 REVERSE [20167]	
JCVI_41448	2.646	no original description	
ES271775	2.646	moderately similar to (306)AT3G61580| Symbols: | delta-8 sphingolipid desaturase (SLD1) | chr3:22797228-22798577 FORWARD [2103	
EX106705	2.645	moderately similar to (493)AT5G28237| Symbols: | tryptophan synthase, beta subunit, putative | chr5:10207481-10213546 REVERSEmo	

EV057452	2.645	very weakly similar to (82.0)AT4G25550| Symbols: | protein binding | chr4:13048528-13050882 FORWARD [21442]	
JCVI_2530	2.645	moderately similar to (340)AT5G38430| Symbols: | ribulose bisphosphate carboxylase small chain 1B / RuBisCO small subunit 1B (RBCS	
JCVI_30814	2.645	highly similar to (533)AT1G20840| Symbols: TMT1 | TMT1 (TONOPLAST MONOSACCHARIDE TRANSPORTER1); carbohydrate tra	
JCVI_5326	2.645	highly similar to (503)AT5G58330| Symbols: | malate dehydrogenase (NADP), chloroplast, putative | chr5:23597236-23599513 REVERS	
JCVI_35989	2.645	moderately similar to (322)AT5G24680| Symbols: | similar to unnamed protein product [Vitis vinifera] (GB:CAO48536.1); contains Inter	
JCVI_7598	2.644	moderately similar to (286)AT3G14770| Symbols: | nodulin MtN3 family protein | chr3:4957794-4959209 REVERSE no original descript	
JCVI_3545	2.644	moderately similar to (443)AT1G56450| Symbols: PBG1 | PBG1 (20S proteasome beta subunit G1); peptidase | chr1:21145635-21147851	
JCVI_435	2.643	moderately similar to (431)AT5G15090| Symbols: | porin, putative / voltage-dependent anion-selective channel protein, putative | chr5:48	
EV086376	2.643	no similarity	
JCVI_15206	2.643	moderately similar to (253)AT5G23940| Symbols: EMB3009 | EMB3009 (EMBRYO DEFECTIVE 3009); transferase | chr5:8076619-807	
JCVI_19231	2.643	moderately similar to (218)AT5G01750| Symbols: | Identical to Uncharacterized protein At5g01750 [Arabidopsis Thaliana] (GB:Q9LZX1	
EE439152	2.643	weakly similar to (127)AT5G45775| Symbols: | 60S ribosomal protein L11 (RPL11D) | chr5:18582508-18583723 REVERSEweakly simil	-3.146
H74394	2.643	weakly similar to (115)AT1G12630| Symbols: | DNA binding / transcription activator/ transcription factor | chr1:4298895-4299473 FORW	
JCVI_1252	2.642	moderately similar to (355)AT5G61410| Symbols: EMB2728, RPE | RPE (EMBRYO DEFECTIVE 2728); ribulose-phosphate 3-epimeras	
EE541798	2.642	moderately similar to (288)AT1G11940| Symbols: | similar to unknown protein [Arabidopsis thaliana] (TAIR:AT1G62305.1); similar to u	
JCVI_5995	2.642	moderately similar to (476)AT1G20260| Symbols: | (VACUOLAR ATP SYNTHASE SUBUNIT B3); hydrogen ion transmembrane transp	
JCVI_24145	2.642	moderately similar to (385)AT4G21810| Symbols: DER2.1 | DER2.1 (DERLIN-2.1) | chr4:11575357-11577015 REVERSEmoderately sim	
EX017340	2.642	moderately similar to (377)AT5G61760| Symbols: IPK2B, ATIPK2BETA | ATIPK2BETA (Arabidopsis thaliana inositol hexakisphosphat	
JCVI_25992	2.641	very weakly similar to (85.1)AT4G24780| Symbols: | pectate lyase family protein | chr4:12770641-12772237 REVERSE no original descri	
JCVI_9389	2.641	weakly similar to (102)AT1G02770| Symbols: | similar to unknown protein [Arabidopsis thaliana] (TAIR:AT1G19060.1); contains InterPr	
CN826718	2.641	moderately similar to (434)AT1G09910| Symbols: | lyase | chr1:3220153-3224453 REVERSE [15793]	
JCVI_7753	2.640	moderately similar to (267)AT5G05520| Symbols: | outer membrane OMP85 family protein | chr5:1632913-1635105 FORWARD no origi	
DY017873	2.640	moderately similar to (338)AT5G43960| Symbols: | nuclear transport factor 2 (NTF2) family protein / RNA recognition motif (RRM)-con	
JCVI_19833	2.640	moderately similar to (259)AT1G22280| Symbols: | protein phosphatase 2C, putative / PP2C, putative | chr1:7874225-7875485 FORWAR	
JCVI_1010	2.640	weakly similar to (182)AT4G31990| Symbols: AAT3, ATAAT1 | ASP5 (ASPARTATE AMINOTRANSFERASE 5) | chr4:15470882-1547	
EX134910	2.639	weakly similar to (164)AT5G64000| Symbols: ATSAL2, SAL2 | SAL2; 3'(2'),5'-bisphosphate nucleotidase/ inositol or phosphatidylinositol	
JCVI_18169	2.638	moderately similar to (333)AT5G59250| Symbols: | sugar transporter family protein | chr5:23921184-23924079 FORWARD no original de	
JCVI_3640	2.638	weakly similar to (131)AT1G72160| Symbols: | SEC14 cytosolic factor family protein / phosphoglyceride transfer family protein | chr1:27	
JCVI_4199	2.638	moderately similar to (412)AT1G22800| Symbols: | methyltransferase | chr1:8072009-8074028 FORWARD no original description	
ES903348	2.638	moderately similar to (277)AT1G74080| Symbols: ATMYB122, MYB122 | MYB122 (myb domain protein 122); DNA binding / transcript	
EV147283	2.638	weakly similar to (109)AT3G63460| Symbols: | WD-40 repeat family protein | chr3:23441984-23448216 REVERSE [21482]	
JCVI_27164	2.638	weakly similar to (163)AT1G29640| Symbols: | similar to unknown protein [Arabidopsis thaliana] (TAIR:AT2G34340.1); similar to unkno	
DY009108	2.638	moderately similar to (340)AT4G34350| Symbols: ISPH, CLB6 | CLB6 (CHLOROPLAST BIOGENESIS 6); 4-hydroxy-3-methylbut-2-en-	
EE460834	2.637	moderately similar to (271)AT5G12040| Symbols: | carbon-nitrogen hydrolase family protein | chr5:3885163-3887773 FORWARD [2015	1.453
JCVI_8448	2.636	moderately similar to (300)AT5G52420| Symbols: | similar to unknown protein [Arabidopsis thaliana] (TAIR:AT5G23920.1); similar to u	-2.785
JCVI_20405	2.636	moderately similar to (390)AT1G15690| Symbols: ATAVP3, AVP-3, AVP1 | AVP1 (vacuolar-type H+-pumping pyrophosphatase 1) | chr1	
JCVI_27438	2.636	moderately similar to (405)AT5G44070| Symbols: ARA8, ATPCS1, PCS1, CAD1 | CAD1 (CADMIUM SENSITIVE 1) | chr5:17752103-1	
CX190417	2.636	weakly similar to (196)AT2G47170| Symbols: ARF1A1c | ARF1A1c (ADP-RIBOSYLATION FACTOR 1); GTP binding / phospholipase	
EX038890	2.635	weakly similar to (111)AT5G15270| Symbols: | KH domain-containing protein | chr5:4958741-4960950 FORWARD [21811]	-1.931
JCVI_109	2.635	moderately similar to (493)AT4G31500| Symbols: SUR2, RNT1, RED1, ATR4, CYP83B1 | CYP83B1 (CYTOCHROME P450 MONOOX	
JCVI_39281	2.635	weakly similar to (155)AT3G02650| Symbols: | pentatricopeptide (PPR) repeat-containing protein | chr3:566278-569872 FORWARD no o	-2.373
EE525938	2.634	no similarity	
JCVI_1794	2.634	moderately similar to (206)AT4G32940| Symbols: GAMMAVPE, GAMMA-VPE | GAMMA-VPE (Vacuolar processing enzyme gamma);	
ES983479	2.634	weakly similar to (184)AT2G37560| Symbols: ORC2, ATORC2 | ATORC2/ORC2 (ORIGIN RECOGNITION COMPLEX SECOND LAR	
JCVI_30269	2.633	very weakly similar to (91.3)AT4G21990| Symbols: PRH-26, PRH26, ATAPR3, APR3 | APR3 (APS REDUCTASE 3) | chr4:11657296-11	
JCVI_5118	2.633	moderately similar to (272)AT2G38740| Symbols: | haloacid dehalogenase-like hydrolase family protein | chr2:16201717-16203073 REVE	
JCVI_16041	2.633	moderately similar to (422)AT3G03740| Symbols: ATBPM4 | ATBPM4 (BTB-POZ AND MATH DOMAIN 4); protein binding | chr3:937	
JCVI_39533	2.633	moderately similar to (206)AT1G25055| Symbols: | Identical to F-box/Kelch-repeat protein At1g24795/At1g24885/At1g25056/At1g2514	-1.929
JCVI_17411	2.632	moderately similar to (310)AT3G61530| Symbols: PANB2 | PANB2; 3-methyl-2-oxobutanoate hydroxymethyltransferase | chr3:22782667	
EE559396	2.631	weakly similar to (103)AT1G15950| Symbols: IRX4, ATCCR1, CCR1 | CCR1 (CINNAMOYL COA REDUCTASE 1) | chr1:5478849-548	
ES939527	2.631	moderately similar to (354)AT1G18370| Symbols: HIK | HIK (HINKEL); microtubule motor | chr1:6319725-6323813 REVERSE [21390]	
ES899667	2.631	no similarity	
EX015773	2.631	weakly similar to (155)AT4G01883| Symbols: | similar to unknown protein [Arabidopsis thaliana] (TAIR:AT1G02475.1); similar to unnam	
JCVI_38041	2.631	moderately similar to (481)AT3G51120| Symbols: | zinc finger (CCCH-type) family protein | chr3:18997006-19002680 REVERSE no orig	
JCVI_23796	2.630	moderately similar to (201)AT3G60620| Symbols: | phosphatidate cytidylyltransferase family protein | chr3:22417512-22419214 FORWA	
JCVI_1414	2.630	very weakly similar to (89.4)AT2G30620| Symbols: | histone H1.2 | chr2:13052437-13053344 FORWARD no original description	
AI352890	2.630	weakly similar to (187)AT1G35720| Symbols: OXY5, ATOXY5, ANNAT1 | ANNAT1 (ANNEXIN ARABIDOPSIS 1); calcium ion bindin	
ES934104	2.630	no similarity	
EX112778	2.630	weakly similar to (157)AT4G19140| Symbols: | similar to unnamed protein product [Vitis vinifera] (GB:CAO66715.1) | chr4:10469841-10	-2.480
JCVI_7822	2.630	moderately similar to (256)AT1G16040| Symbols: | similar to unknown [Populus trichocarpa] (GB:ABK92967.1); contains InterPro doma	
CV432184	2.630	no similarity	
JCVI_450	2.630	highly similar to (600)AT1G11260| Symbols: STP1 | STP1 (SUGAR TRANSPORTER 1); carbohydrate transmembrane transporter/ sugar	
CD814629	2.629	no similarity	
JCVI_32522	2.629	moderately similar to (312)AT5G06150| Symbols: CYCB1;2, CYC1BAT | CYC1BAT (CYCLIN B 1;2); cyclin-dependent protein kinase r	
ES928197	2.629	weakly similar to (130)AT4G17170| Symbols: AtRABB1c, AtRab2A, AT-RAB2 | AT-RAB2 (Arabidopsis Rab GTPase homolog B1c); GT	
CV544754	2.628	weakly similar to (160)AT3G52580| Symbols: | 40S ribosomal protein S14 (RPS14C) | chr3:19514302-19515679 FORWARDweakly simi	
EH414568	2.628	weakly similar to (105)AT1G51890| Symbols: | leucine-rich repeat protein kinase, putative | chr1:19278471-19282197 REVERSE [20767]	
EX063745	2.627	weakly similar to (181)AT1G58360| Symbols: NAT2, AAP1 | AAP1 (AMINO ACID PERMEASE 1); amino acid transmembrane transpor	
AT000794	2.627	no similarity	
EV052278	2.627	weakly similar to (164)AT5G40210| Symbols: | nodulin MtN21 family protein | chr5:16090953-16093316 REVERSE [21442]	
JCVI_23045	2.627	highly similar to (914)AT2G41080| Symbols: | pentatricopeptide (PPR) repeat-containing protein | chr2:17139935-17141632 FORWARD	
EX113526	2.626	moderately similar to (254)AT1G29660| Symbols: | GDSL-motif lipase/hydrolase family protein | chr1:10371941-10373610 FORWARD [	
EE564614	2.626	no similarity	
CV433991	2.625	weakly similar to (140)AT2G40590| Symbols: | 40S ribosomal protein S26 (RPS26B) | chr2:16952293-16953423 REVERSEweakly simila	
JCVI_3883	2.625	moderately similar to (315)AT1G80160| Symbols: | lactoylglutathione lyase family protein / glyoxalase I family protein | chr1:30156172-3	
JCVI_12706	2.625	moderately similar to (310)AT5G17760| Symbols: | AAA-type ATPase family protein | chr5:5861278-5862303 REVERSE no original des	-2.257
JCVI_4601	2.624	moderately similar to (301)AT4G00026| Symbols: | similar to unnamed protein product [Vitis vinifera] (GB:CAO66702.1); contains Inter	
JCVI_10204	2.623	moderately similar to (236)AT4G27000| Symbols: ATRBP45C | ATRBP45C; RNA binding | chr4:13554989-13557769 REVERSE no orig	
JCVI_18735	2.623	moderately similar to (219)AT1G24040| Symbols: | GCN5-related N-acetyltransferase (GNAT) family protein | chr1:8505783-8506742 R	
EH423183	2.623	weakly similar to (127)AT1G80530| Symbols: | nodulin family protein | chr1:30283116-30285139 REVERSE [20767]	
DN964531	2.623	moderately similar to (395)AT5G03340| Symbols: | (Cell division control protein 48 homolog E); ATPase | chr5:810090-813132 REVERS	

EX040361	2.623	weakly similar to (104)AT2G23910| Symbols: | cinnamoyl-CoA reductase-related | chr2:10184982-10186869 FORWARD [21811]	
JCVI_2284	2.622	moderately similar to (400)AT3G01280| Symbols: | porin, putative | chr3:85761-87619 FORWARDmoderately similar to (342)VDAC1_S	
JCVI_30562	2.622	weakly similar to (189)AT4G26550| Symbols: | similar to unknown protein [Arabidopsis thaliana] (TAIR:AT5G56020.1); similar to unnam	
EV049361	2.621	moderately similar to (244)AT3G26070| Symbols: | plastid-lipid associated protein PAP / fibrillin family protein | chr3:9528141-9529436	
JCVI_5527	2.621	moderately similar to (227)AT2G18630| Symbols: | Identical to UPF0496 protein At2g18630 [Arabidopsis Thaliana] (GB:Q56XQ0;GB:Q	
EE477555	2.621	weakly similar to (168)AT2G31390| Symbols: | pfkB-type carbohydrate kinase family protein | chr2:13390712-13393193 REVERSEweak	
AT002090	2.620	weakly similar to (121)AT5G59320| Symbols: LTP3 | LTP3 (LIPID TRANSFER PROTEIN 3); lipid binding | chr5:23946277-23946718 F	
JCVI_23833	2.620	moderately similar to (395)AT1G06430| Symbols: FTSH8 | FTSH8 (FtsH protease 8); ATP-dependent peptidase/ ATPase/ metallopeptidas	-2.450
EE503492	2.620	no similarity	
JCVI_26679	2.619	moderately similar to (292)AT5G58560| Symbols: | phosphatidate cytidylyltransferase family protein | chr5:23687739-23689796 FORWA	
JCVI_7752	2.619	moderately similar to (227)AT3G55410| Symbols: | 2-oxoglutarate dehydrogenase E1 component, putative / oxoglutarate decarboxylase, p	
JCVI_40933	2.619	highly similar to (645)AT1G35670| Symbols: CPK11, ATCDPK2 | ATCDPK2 (CALCIUM-DEPENDENT PROTEIN KINASE 2); calmod	
JCVI_23253	2.619	moderately similar to (455)AT5G18570| Symbols: | GTP1/OBG family protein | chr5:6171841-6174825 REVERSE no original description	
JCVI_18314	2.619	moderately similar to (448)AT2G45510| Symbols: CYP704A2 | CYP704A2 (cytochrome P450, family 704, subfamily A, polypeptide 2); c	
JCVI_22844	2.618	weakly similar to (156)AT1G70700| Symbols: JAZ9, TIFY7 | JAZ9/TIFY7 (JASMONATE-ZIM-DOMAIN PROTEIN 9) | chr1:26658614	
EV110418	2.618	moderately similar to (211)AT1G59830| Symbols: PP2A-1 | PP2A-1 (protein phosphatase 2A-2); protein serine/threonine phosphatase | ch	
EX084095	2.618	no similarity	
JCVI_19575	2.618	moderately similar to (308)AT2G41680| Symbols: | thioredoxin reductase, putative / NADPH-dependent thioredoxin reductase, putative |	
ES948294	2.618	moderately similar to (358)AT3G06550| Symbols: | similar to O-acetyltransferase family protein [Arabidopsis thaliana] (TAIR:AT2G344	
JCVI_12223	2.617	moderately similar to (397)AT1G21700| Symbols: CHB4, ATSWI3C | ATSWI3C (Arabidopsis thaliana switching protein 3C); DNA bindi	-2.964
JCVI_13942	2.617	highly similar to (769)AT2G38940| Symbols: PHT1;4, ATPT2 | ATPT2 (PHOSPHATE TRANSPORTER 2); carbohydrate transmembrane	
JCVI_16169	2.617	moderately similar to (251)AT3G51980| Symbols: | binding | chr3:19296789-19298480 REVERSE no original description	
EE526681	2.616	weakly similar to (127)AT1G15940| Symbols: | binding | chr1:5473666-5478044 FORWARD [20143] 1 659 688	
ES946978	2.616	no similarity	
JCVI_10174	2.616	no original description	
JCVI_16454	2.616	moderately similar to (340)AT3G53750| Symbols: ACT3 | ACT3 (ACTIN 3); structural constituent of cytoskeleton | chr3:19926902-19928	
CV433126	2.616	no similarity	
JCVI_83	2.616	moderately similar to (325)AT1G53580| Symbols: GLX2-3, ETHE1, GLY3 | ETHE1/GLX2-3/GLY3 (GLYOXALASE 2-3); hydroxyacylg	
JCVI_36533	2.615	moderately similar to (219)AT1G10960| Symbols: ATFD1 | ATFD1 (FERREDOXIN 1); 2 iron, 2 sulfur cluster binding / electron carrier/ i	
JCVI_15408	2.615	moderately similar to (311)AT2G29110| Symbols: GLR2.8, ATGLR2.8 | ATGLR2.8 (Arabidopsis thaliana glutamate receptor 2.8) | chr2:1	
EV109284	2.615	no similarity	
AM390284	2.615	weakly similar to (118)AT5G63680| Symbols: | pyruvate kinase, putative | chr5:25507733-25509756 FORWARDweakly similar to (117)I	
EV019169	2.614	no similarity	
JCVI_27577	2.614	moderately similar to (494)AT4G39080| Symbols: VHA-A3 | VHA-A3 (VACUOLAR PROTON ATPASE A3); ATPase | chr4:18209507-1	
JCVI_749	2.613	highly similar to (574)AT5G23860| Symbols: TUB8 | TUB8 (tubulin beta-8) | chr5:8042965-8044531 FORWARDhighly similar to (572)T	
JCVI_12404	2.613	moderately similar to (460)AT4G24550| Symbols: | clathrin adaptor complexes medium subunit family protein | chr4:12675883-12678913	
JCVI_32618	2.612	moderately similar to (419)AT1G74040| Symbols: MAML-3, IMS1, IPMS2 | IMS1; 2-isopropylmalate synthase | chr1:27845919-2784922	
EE546474	2.612	weakly similar to (150)AT1G42970| Symbols: GAPB | GAPB (GLYCERALDEHYDE-3-PHOSPHATE DEHYDROGENASE B SUBUNIT	
EV134810	2.612	moderately similar to (386)AT1G53710| Symbols: | similar to unnamed protein product [Vitis vinifera] (GB:CAO68485.1); contains Inter	
EE439057	2.612	moderately similar to (231)AT4G38040| Symbols: | exostosin family protein | chr4:17867495-17869125 FORWARD [20173]	
JCVI_8504	2.612	moderately similar to (281)AT5G64250| Symbols: | 2-nitropropane dioxygenase family / NPD family | chr5:25714849-25716171 REVERS	
EX032967	2.612	no similarity	
JCVI_4211	2.612	moderately similar to (432)AT5G09650| Symbols: ATPPA6 | ATPPA6 (ARABIDOPSIS THALIANA PYROPHOSPHORYLASE 6); inorg	1.219
JCVI_19738	2.611	moderately similar to (444)AT1G28690| Symbols: | pentatricopeptide (PPR) repeat-containing protein | chr1:10080028-10081590 REVER	
JCVI_27166	2.611	moderately similar to (234)AT2G43790| Symbols: MPK6, MAPK6, ATMPK6 | ATMPK6 (MAP KINASE 6); MAP kinase/ kinase | chr2:1	-3.078
BQ704274	2.611	weakly similar to (132)AT5G02050| Symbols: | mitochondrial glycoprotein family protein / MAM33 family protein | chr5:403236-404241	
EE442947	2.610	weakly similar to (176)AT1G18480| Symbols: | calcineurin-like phosphoesterase family protein | chr1:6361632-6362807 FORWARD [20	
EE440508	2.608	no similarity	
AM394180	2.608	weakly similar to (137)AT3G43600| Symbols: AAO2 | AAO2 (ALDEHYDE OXIDASE 2) | chr3:15523766-15528363 REVERSEvery wea	
JCVI_22782	2.608	highly similar to (622)AT5G22980| Symbols: SCPL47 | SCPL47 (serine carboxypeptidase-like 47); serine carboxypeptidase | chr5:768808	
JCVI_19306	2.608	moderately similar to (210)AT1G62040| Symbols: ATG8C | ATG8C (AUTOPHAGY 8C); microtubule binding | chr1:22936881-2293791	
JCVI_3970	2.606	moderately similar to (363)AT2G25920| Symbols: | similar to 3'-5' exonuclease domain-containing protein / K homology domain-containi	-2.946
CV433818	2.606	weakly similar to (136)AT5G12850| Symbols: | zinc finger (CCCH-type) family protein | chr5:4057071-4059191 FORWARD [16490]	-4.004
JCVI_16872	2.605	moderately similar to (304)AT2G23093| Symbols: | similar to unknown protein [Arabidopsis thaliana] (TAIR:AT3G49310.1); similar to u	-2.284
JCVI_21955	2.605	highly similar to (519)AT2G47600| Symbols: MHX1, ATMHX1, ATMHX | ATMHX (MAGNESIUM/PROTON EXCHANGER); cation:c	
CD816241	2.605	moderately similar to (351)AT3G18730| Symbols: MGO3, BRU1, TSK | TSK (TONSOKU) | chr3:6446068-6453051 REVERSE [13977]	
JCVI_16409	2.604	moderately similar to (296)AT2G33480| Symbols: ANAC041 | ANAC041 (Arabidopsis NAC domain containing protein 41) | chr2:141883	
EX120389	2.603	weakly similar to (167)AT3G05360| Symbols: | disease resistance family protein / LRR family protein | chr3:1530906-1533266 REVERSI	-2.947
JCVI_8691	2.603	moderately similar to (420)AT3G51000| Symbols: | epoxide hydrolase, putative | chr3:18956239-18957480 REVERSE no original descrip	
JCVI_36663	2.603	moderately similar to (341)AT4G05460| Symbols: | F-box family protein (FBL20) | chr4:2761103-2762397 REVERSE no original descrip	
EV002137	2.603	no similarity	-6.330
JCVI_39063	2.603	highly similar to (882)AT4G17770| Symbols: TPS5, ATTPS5 | ATTPS5 (Arabidopsis thaliana trehalose phosphatase/synthase 5); transfera	
EE544917	2.603	no similarity	
ES946004	2.602	moderately similar to (276)AT5G45500| Symbols: | similar to unknown protein [Arabidopsis thaliana] (TAIR:AT5G45520.1); similar to u	
BQ704506	2.602	very weakly similar to (90.5)AT1G22850| Symbols: | similar to unknown protein [Arabidopsis thaliana] (TAIR:AT1G03260.1); similar to u	-1.624
JCVI_41972	2.602	moderately similar to (312)AT5G63580| Symbols: | flavonol synthase, putative | chr5:25471956-25473318 FORWARDweakly similar to (	
EX125145	2.601	no similarity	
EX090158	2.601	weakly similar to (129)AT1G03340| Symbols: | similar to unknown protein [Arabidopsis thaliana] (TAIR:AT4G02920.1); similar to unnar	
EV180493	2.600	moderately similar to (286)AT2G30360| Symbols: SIP4, SNRK3.22, PKS5, CIPK11 | CIPK11 (SOS3-INTERACTING PROTEIN 4); kina	
EH419049	2.600	moderately similar to (327)AT1G01860| Symbols: PFC1 | PFC1 (PALEFACE 1) | chr1:304439-306275 REVERSE [20767]	
JCVI_1850	2.600	moderately similar to (348)AT1G64980| Symbols: | similar to unnamed protein product [Vitis vinifera] (GB:CAO62125.1); contains doma	
JCVI_1591	2.600	moderately similar to (460)AT2G46620| Symbols: | AAA-type ATPase family protein | chr2:19146141-19147616 REVERSE no original d	
JCVI_37762	2.599	no original description	
BG544106	2.599	weakly similar to (123)AT5G06130| Symbols: | chaperone protein dnaJ-related | chr5:1853755-1855764 REVERSE [8791]	
JCVI_26810	2.599	moderately similar to (301)AT2G25830| Symbols: | YebC-related | chr2:11026169-11028743 REVERSE no original description	
JCVI_22914	2.599	highly similar to (628)AT3G10370| Symbols: | glycerol-3-phosphate dehydrogenase, putative | chr3:3216507-3219032 FORWARD no orig	
JCVI_8012	2.599	highly similar to (515)AT5G02500| Symbols: HSP70-1, AT-HSC70-1, HSC70, HSC70-1 | HSC70-1 (heat shock cognate 70 kDa protein 1)	
EX036268	2.598	moderately similar to (262)AT5G13480| Symbols: FY | FY | chr5:4326641-4331560 REVERSE [21811]	
CD814818	2.598	moderately similar to (282)AT1G73700| Symbols: | MATE efflux family protein | chr1:27721215-27723291 REVERSE [13977]	
JCVI_19326	2.598	weakly similar to (155)AT5G51250| Symbols: | kelch repeat-containing F-box family protein | chr5:20847969-20849075 FORWARD no o	-2.002
AT000776	2.598	no similarity	

EV078164	2.597	no similarity	
AM058765	2.597	moderately similar to (290)AT5G26742| Symbols: EMB1138 | EMB1138 (EMBRYO DEFECTIVE 1138); ATP binding / ATP-dependent	
EV026425	2.597	no similarity	
JCVI_26569	2.596	moderately similar to (335)AT1G13130| Symbols: | glycosyl hydrolase family 5 protein / cellulase family protein | chr1:4474724-4477818	
L38176	2.595	no similarity	
EV039671	2.595	moderately similar to (351)AT5G60170| Symbols: | RNA binding | chr5:24245404-24249620 FORWARD [21442] 19 694 694	
JCVI_27430	2.595	moderately similar to (265)AT1G05940| Symbols: CAT9 | CAT9 (CATIONIC AMINO ACID TRANSPORTER 9); cationic amino acid tra	
JCVI_2016	2.594	moderately similar to (381)AT3G03120| Symbols: ATARFB1C | ATARFB1C (ADP-ribosylation factor B1C); GTP binding | chr3:717352-	
JCVI_8112	2.594	moderately similar to (427)AT4G39170| Symbols: | SEC14 cytosolic factor, putative / phosphoglyceride transfer protein, putative | chr4:18	
EX089859	2.594	weakly similar to (142)AT4G04860| Symbols: DER2.2 | DER2.2 (DERLIN-2.2) | chr4:2460133-2461376 FORWARDweakly similar to (1	
JCVI_1352	2.593	moderately similar to (399)AT2G22240| Symbols: | inositol-3-phosphate synthase isozyme 2 / myo-inositol-1-phosphate synthase 2 / MI-1	
JCVI_6419	2.593	moderately similar to (486)AT4G35260| Symbols: IDH1 | IDH1 (ISOCITRATE DEHYDROGENASE 1); isocitrate dehydrogenase (NAD-	
DY019580	2.593	moderately similar to (224)AT4G10890| Symbols: | similar to unknown protein [Arabidopsis thaliana] (TAIR:AT1G43722.1); similar to u	
JCVI_4851	2.593	moderately similar to (359)AT1G35190| Symbols: | oxidoreductase, 2OG-Fe(II) oxygenase family protein | chr1:12890522-12892610 FOI	
EX132512	2.593	moderately similar to (233)AT3G07570| Symbols: | membrane protein, putative | chr3:2418211-2420212 REVERSE [21833] 1 680 702	
JCVI_4203	2.593	moderately similar to (454)AT4G33010| Symbols: ATGLDP1 | ATGLDP1 (ARABIDOPSIS THALIANA GLYCINE DECARBOXYLASE	-1.818
JCVI_1154	2.592	no original description	
EE407447	2.592	weakly similar to (137)AT3G04840| Symbols: | 40S ribosomal protein S3A (RPS3aA) | chr3:1329757-1331424 FORWARDweakly similar	
JCVI_14753	2.592	weakly similar to (191)AT3G12650| Symbols: | similar to unnamed protein product [Vitis vinifera] (GB:CAO15069.1) | chr3:4017888-401	
JCVI_4777	2.592	moderately similar to (370)AT3G19930| Symbols: STP4 | STP4 (SUGAR TRANSPORTER 4); carbohydrate transmembrane transporter/ s	
JCVI_16385	2.592	moderately similar to (249)AT1G05270| Symbols: | TraB family protein | chr1:1531805-1534304 REVERSE no original description	
EX021895	2.591	very weakly similar to (86.3)AT1G19660| Symbols: | wound-responsive family protein | chr1:6800352-6802231 REVERSE [21809] 1 225	
DV643279	2.591	highly similar to (555)AT5G44820| Symbols: | similar to unknown protein [Arabidopsis thaliana] (TAIR:AT4G19970.1); similar to unnam	
EV227471	2.591	weakly similar to (115)AT1G75290| Symbols: | isoflavone reductase, putative | chr1:28257551-28258834 FORWARDweakly similar to (1	
JCVI_16994	2.590	moderately similar to (334)AT4G29590| Symbols: | methyltransferase | chr4:14512742-14514410 REVERSE no original description	
JCVI_15519	2.590	highly similar to (877)AT4G36195| Symbols: | serine carboxypeptidase S28 family protein | chr4:17127205-17129790 FORWARD no orig	
JCVI_8920	2.590	moderately similar to (304)AT1G06650| Symbols: | 2-oxoglutarate-dependent dioxygenase, putative | chr1:2035908-2037185 FORWARD	
ES909228	2.590	weakly similar to (107)AT5G16570| Symbols: GLN1;4 | GLN1;4 (Glutamine synthetase 1;4); glutamate-ammonia ligase | chr5:5421901-54	
ES901375	2.589	no similarity	
JCVI_13996	2.589	weakly similar to (171)AT4G01070| Symbols: GT72B1 | GT72B1; UDP-glucosyltransferase/ UDP-glycosyltransferase/ transferase, transfe	
ES910257	2.589	moderately similar to (259)AT4G02850| Symbols: | phenazine biosynthesis PhzC/PhzF family protein | chr4:1266535-1268569 REVERSE	
JCVI_13904	2.589	moderately similar to (203)AT1G78820| Symbols: | curculin-like (mannose-binding) lectin family protein / PAN domain-containing protei	
JCVI_3843	2.588	weakly similar to (135)AT4G39860| Symbols: | similar to unknown protein [Arabidopsis thaliana] (TAIR:AT2G22270.1); similar to unnar	-1.979
EE553719	2.588	weakly similar to (137)ATMG00160| Symbols: COX2 | cytochrome c oxidase subunit 2 | chrM:40502-42628 REVERSEweakly similar to (	
EV180694	2.588	moderately similar to (315)AT4G14170| Symbols: | pentatricopeptide (PPR) repeat-containing protein | chr4:8176704-8178080 REVERSE	
EX095729	2.588	very weakly similar to (97.8)AT3G14290| Symbols: PAE2 | PAE2 (20S proteasome alpha subunit E2); peptidase | chr3:4764371-4766388 F	-4.602
JCVI_21038	2.588	moderately similar to (259)AT1G72310| Symbols: ATL3 | ATL3 (Arabidopsis T?xicos en Levadura 3); protein binding / zinc ion binding |	
JCVI_2695	2.588	weakly similar to (102)AT5G64080| Symbols: | protease inhibitor/seed storage/lipid transfer protein (LTP) family protein | chr5:25662701	
JCVI_23685	2.587	no original description	
JCVI_42051	2.587	moderately similar to (393)AT5G61190| Symbols: | zinc finger protein-related | chr5:24632706-24637112 FORWARD no original descrip	-1.864
EX137380	2.587	highly similar to (516)AT1G71850| Symbols: | similar to unknown protein [Arabidopsis thaliana] (TAIR:AT4G24320.1); similar to unnam	
EX134092	2.587	no similarity	
JCVI_39467	2.586	moderately similar to (343)AT4G32980| Symbols: ATH1 | ATH1 (ARABIDOPSIS THALIANA HOMEOBOX GENE 1); transcription fac	
JCVI_30141	2.586	highly similar to (666)AT2G24850| Symbols: TAT, TAT3 | TAT3 (TYROSINE AMINOTRANSFERASE 3); transaminase | chr2:1059014	
JCVI_19308	2.586	weakly similar to (134)AT1G29260| Symbols: PEX7 | PEX7 (peroxin 7) | chr1:10224909-10225862 FORWARD no original description	
EE535098	2.586	moderately similar to (311)AT2G35060| Symbols: KUP11 | KUP11 (K+ uptake permease 11); potassium ion transmembrane transporter |	
EX121359	2.585	no similarity	
JCVI_13438	2.585	highly similar to (869)AT3G46970| Symbols: ATPHS2, PHS2 | ATPHS2/PHS2 (ALPHA-GLUCAN PHOSPHORYLASE 2); phosphorylas	
JCVI_37629	2.585	weakly similar to (135)AT4G28990| Symbols: | RNA-binding protein-related | chr4:14291211-14293024 FORWARD no original descripti	
EX059821	2.585	weakly similar to (101)AT3G14290| Symbols: PAE2 | PAE2 (20S proteasome alpha subunit E2); peptidase | chr3:4764371-4766388 FORV	
JCVI_11133	2.585	moderately similar to (259)AT1G76980| Symbols: | similar to EMB2170 (EMBRYO DEFECTIVE 2170) [Arabidopsis thaliana] (TAIR:A	-2.118
JCVI_5091	2.584	moderately similar to (231)AT5G14040| Symbols: | mitochondrial phosphate transporter | chr5:4531061-4532967 REVERSE no original c	
EE461082	2.584	weakly similar to (138)AT5G05010| Symbols: | clathrin adaptor complexes medium subunit-related | chr5:1477138-1479873 FORWARD	
EV058843	2.584	weakly similar to (186)AT2G25280| Symbols: | similar to unknown [Populus trichocarpa] (GB:ABK93585.1); contains InterPro domain P	
JCVI_38341	2.584	very weakly similar to (96.7)AT1G23440| Symbols: | pyrrolidone-carboxylate peptidase family protein | chr1:8321929-8324008 FORWAR	
ES954018	2.584	no similarity	
EE451261	2.583	moderately similar to (281)AT5G21482| Symbols: ATCKX5, CKX7 | CKX7 (CYTOKININ OXIDASE 7); oxidoreductase | chr5:7226845-	
JCVI_28246	2.583	moderately similar to (466)AT2G17200| Symbols: | ubiquitin family protein | chr2:7489215-7492172 REVERSE no original description	
EE512958	2.582	weakly similar to (151)AT1G75810| Symbols: | similar to unnamed protein product [Vitis vinifera] (GB:CAO60969.1) | chr1:28465408-28	
ES910611	2.582	moderately similar to (310)AT5G39450| Symbols: | F-box family protein | chr5:15803273-15805105 FORWARD [21430]	
EE439958	2.582	weakly similar to (185)AT1G25260| Symbols: | acidic ribosomal protein P0-related | chr1:8854150-8855753 REVERSE [20167]	
JCVI_25172	2.582	moderately similar to (233)AT5G07250| Symbols: | rhomboid family protein | chr5:2274438-2275936 REVERSE no original description	
JCVI_4415	2.582	moderately similar to (300)AT3G55800| Symbols: SBPASE | SBPASE (SEDOHEPTULOSE-BISPHOSPHATASE); phosphoric ester hydr	
EE547508	2.582	no similarity	
EV036424	2.581	weakly similar to (137)AT3G13180| Symbols: | NOL1/NOP2/sun family protein / antitermination NusB domain-containing protein | chr3:4	
JCVI_34249	2.581	highly similar to (531)AT1G08660| Symbols: | glycosyl transferase family 29 protein / sialyltransferase family protein | chr1:2757587-275	
JCVI_3606	2.581	highly similar to (627)AT3G42050| Symbols: | vacuolar ATP synthase subunit H family protein | chr3:14239832-14243214 REVERSEhig	-4.205
JCVI_35478	2.581	weakly similar to (133)AT5G20910| Symbols: | zinc finger (C3HC4-type RING finger) family protein | chr5:7092665-7094312 REVERSE	
JCVI_13503	2.581	moderately similar to (385)AT4G25310| Symbols: | oxidoreductase, 2OG-Fe(II) oxygenase family protein | chr4:12949763-12951148 FOI	
CX189486	2.580	moderately similar to (276)AT1G58210| Symbols: EMB1674 | EMB1674 (EMBRYO DEFECTIVE 1674) | chr1:21557286-21561721 FOF	
JCVI_40801	2.580	very weakly similar to (86.3)AT1G26190| Symbols: | phosphoribulokinase/uridine kinase family protein | chr1:9057272-9060420 REVERS	
JCVI_5645	2.580	moderately similar to (335)AT4G17270| Symbols: | Mo25 family protein | chr4:9676419-9678581 FORWARD no original description	
JCVI_5441	2.580	moderately similar to (330)AT3G16857| Symbols: ARR1 | ARR1 (ARABIDOPSIS RESPONSE REGULATOR 1); transcription factor/ tw	
JCVI_34829	2.579	weakly similar to (102)AT4G17560| Symbols: | ribosomal protein L19 family protein | chr4:9780356-9781765 FORWARD no original des	
EV175841	2.579	weakly similar to (115)AT1G71880| Symbols: ATSUC1, SUC1 | SUC1 (SUCROSE-PROTON SYMPORTER 1); carbohydrate transmemb	
JCVI_17752	2.579	highly similar to (532)AT2G17200| Symbols: | ubiquitin family protein | chr2:7489215-7492172 REVERSE no original description	
EV177798	2.578	weakly similar to (171)AT4G23910| Symbols: | similar to unknown protein [Arabidopsis thaliana] (TAIR:AT4G10970.4); similar to unkno	-1.600
EV066774	2.578	moderately similar to (358)AT3G62890| Symbols: | binding | chr3:23257143-23258948 FORWARDvery weakly similar to (91.3)RF1_OR	
JCVI_15056	2.578	moderately similar to (328)AT4G38970| Symbols: | fructose-bisphosphate aldolase, putative | chr4:18163763-18165653 REVERSEmoder	
EE471368	2.578	no similarity	
JCVI_27167	2.578	moderately similar to (236)AT3G56220| Symbols: | transcription regulator | chr3:20869920-20871281 FORWARD no original description	

ES926228	2.577	very weakly similar to (95.5)AT2G23800| Symbols: GGPS5, GGPS2 | GGPS2 (GERANYLGERANYL PYROPHOSPHATE SYNTHASE	
EE445845	2.577	moderately similar to (224)AT4G27540| Symbols: | prenylated rab acceptor (PRA1) protein-related | chr4:13753455-13754666 REVERS	
JCVI_14430	2.577	moderately similar to (385)AT4G30470| Symbols: | cinnamoyl-CoA reductase-related | chr4:14894269-14896512 FORWARDvery weakly	
JCVI_15911	2.576	moderately similar to (368)AT2G39200| Symbols: ATMLO12, MLO12 | MLO12 (MILDEW RESISTANCE LOCUS O 12); calmodulin bi	
JCVI_39125	2.575	moderately similar to (280)AT3G05580| Symbols: | serine/threonine protein phosphatase, putative | chr3:1618222-1619856 REVERSEmo	
JCVI_9169	2.575	no original description	
JCVI_10367	2.575	moderately similar to (387)AT1G04620| Symbols: | coenzyme F420 hydrogenase family / dehydrogenase, beta subunit family | chr1:1282	
JCVI_7437	2.575	moderately similar to (234)AT1G48320| Symbols: | thioesterase family protein | chr1:17858692-17859245 REVERSE no original descript	-3.305
JCVI_35348	2.575	no original description	
EV041240	2.574	moderately similar to (230)AT2G32980| Symbols: | similar to unnamed protein product [Vitis vinifera] (GB:CAO24056.1) | chr2:1400464	
EV022865	2.574	weakly similar to (157)AT3G52640| Symbols: | nicastrin-related | chr3:19526603-19531268 REVERSE [21441]	
JCVI_14529	2.573	moderately similar to (493)AT1G28090| Symbols: | polynucleotide adenylyltransferase family protein | chr1:9796223-9798878 FORWAR	
EE523169	2.573	weakly similar to (161)AT4G12110| Symbols: ATSMO1, ATSMO1-1, SMO1-1 | SMO1-1 (STEROL-4ALPHA-METHYL OXIDASE 1-1)	
JCVI_39705	2.572	moderately similar to (419)AT3G20740| Symbols: FIS3, FIE1, FIE | FIE (FERTILIZATION-INDEPENDENT ENDOSPERM 1); nucleoti	
JCVI_40982	2.572	moderately similar to (458)AT4G20960| Symbols: | cytidine/deoxycytidylate deaminase family protein | chr4:11212088-11213541 FORW	
JCVI_8042	2.572	highly similar to (589)AT1G16560| Symbols: | Per1-like family protein | chr1:5669227-5670336 FORWARD no original description	
EE504908	2.572	no similarity	
JCVI_9463	2.572	weakly similar to (192)AT4G10430| Symbols: | similar to unknown protein [Arabidopsis thaliana] (TAIR:AT1G33230.1); similar to unna	
EX117312	2.572	moderately similar to (287)AT5G54600| Symbols: | 50S ribosomal protein L24, chloroplast (CL24) | chr5:22200272-22201629 FORWAR	
JCVI_33364	2.572	moderately similar to (247)AT1G24470| Symbols: | short-chain dehydrogenase/reductase (SDR) family protein | chr1:8674044-8676265 F	
JCVI_35344	2.572	weakly similar to (150)AT1G13280| Symbols: AOC4 | AOC4 (ALLENE OXIDE CYCLASE 4) | chr1:4547621-4548549 FORWARD no o	
AM389003	2.571	moderately similar to (319)AT2G04040| Symbols: ATDTX1 | ATDTX1; antiporter/ multidrug efflux pump/ multidrug transporter/ transpo	-2.103
JCVI_36086	2.571	moderately similar to (214)AT5G14780| Symbols: FDH | FDH (FORMATE DEHYDROGENASE); NAD binding / binding / catalytic/ cof	
JCVI_1207	2.571	highly similar to (599)AT3G11170| Symbols: FADD, FAD7 | FAD7 (FATTY ACID DESATURASE 7); omega-3 fatty acid desaturase | ch	
AM395575	2.570	no similarity	
EV171174	2.570	very weakly similar to (87.4)AT5G64220| Symbols: | calmodulin-binding protein | chr5:25703660-25709129 FORWARD [21486] 89 1010	
EX133091	2.569	very weakly similar to (80.9)AT3G14990| Symbols: | 4-methyl-5(b-hydroxyethyl)-thiazole monophosphate biosynthesis protein, putative |	
JCVI_19275	2.569	weakly similar to (167)AT3G13720| Symbols: | prenylated rab acceptor (PRA1) family protein | chr3:4495209-4495775 REVERSE no ori	
EE545201	2.569	no similarity	
JCVI_3385	2.569	moderately similar to (459)AT1G25220| Symbols: TRP4, ASB1 | ASB1 (ANTHRANILATE SYNTHASE BETA SUBUNIT 1); anthranila	
JCVI_23248	2.568	moderately similar to (223)AT3G01500| Symbols: CA1 | CA1 (CARBONIC ANHYDRASE 1); carbonate dehydratase/ zinc ion binding | c	
CN726413	2.568	weakly similar to (152)AT1G08110| Symbols: | lactoylglutathione lyase, putative / glyoxalase I, putative | chr1:2535460-2537627 FORWA	
JCVI_16498	2.567	moderately similar to (218)AT2G35860| Symbols: FLA16 | FLA16 (FASCICLIN-LIKE ARABINOGALACTAN PROTEIN 16 PRECURS	
CD828517	2.567	moderately similar to (381)AT1G13770| Symbols: | similar to unknown protein [Arabidopsis thaliana] (TAIR:AT3G45890.1); similar to u	
JCVI_13744	2.567	moderately similar to (412)AT5G51400| Symbols: | similar to unknown protein [Arabidopsis thaliana] (TAIR:AT2G45010.1); similar to u	
EV213995	2.567	moderately similar to (273)AT1G11860| Symbols: | aminomethyltransferase, putative | chr1:4001800-4003244 FORWARDmoderately sin	
JCVI_10020	2.566	moderately similar to (400)AT4G15410| Symbols: PUX5 | UBX domain-containing protein | chr4:8814868-8816596 FORWARD no origir	
JCVI_18380	2.566	moderately similar to (243)AT2G43820| Symbols: GT, UGT74F2 | GT/UGT74F2 (UDP-GLUCOSYLTRANSFERASE 74F2); UDP-gluco	
JCVI_21342	2.566	very weakly similar to (94.4)AT3G56910| Symbols: PSRP5 | PSRP5 (PLASTID-SPECIFIC 50S RIBOSOMAL PROTEIN 5) | chr3:210805	
JCVI_15827	2.565	highly similar to (762)AT2G18700| Symbols: TPS11, ATTPSB, ATTPS11 | ATTPS11 (Arabidopsis thaliana trehalose phosphatase/syntha	
EE481174	2.565	weakly similar to (182)AT3G16565| Symbols: | ATP binding / alanine-tRNA ligase/ ligase, forming aminoacyl-tRNA and related compou	
DY021213	2.565	weakly similar to (160)AT1G32120| Symbols: | similar to unknown protein [Arabidopsis thaliana] (TAIR:AT4G16050.1); similar to unkno	
JCVI_576	2.565	moderately similar to (272)AT3G06270| Symbols: | protein phosphatase 2C, putative / PP2C, putative | chr3:1896769-1897893 FORWAR	
EV204509	2.565	no similarity	
EV059456	2.564	no similarity	
EE529708	2.564	moderately similar to (204)AT1G68030| Symbols: | PHD finger protein-related | chr1:25504041-25505207 REVERSE [16815]	
EE457962	2.564	moderately similar to (318)AT3G55250| Symbols: | similar to unnamed protein product [Vitis vinifera] (GB:CAO14780.1) | chr3:2049027	
JCVI_14940	2.564	very weakly similar to (98.6)AT4G34640| Symbols: ERG9, SQS1 | SQS1 (SQUALENE SYNTHASE 1); farnesyl-diphosphate farnesyltrans	
EX089358	2.564	moderately similar to (310)AT1G20200| Symbols: EMB2719 | EMB2719 (EMBRYO DEFECTIVE 2719) | chr1:7001400-7004145 REVE	
EE455164	2.564	moderately similar to (207)AT2G28650| Symbols: ATEXO70H8 | ATEXO70H8 (exocyst subunit EXO70 family protein H8); protein bind	
JCVI_7208	2.563	moderately similar to (244)AT3G46900| Symbols: COPT2 | COPT2 (Copper transporter 2); copper ion transmembrane transporter | chr3:1	
RC_ES216586	2.563	no similarity	
JCVI_28177	2.563	moderately similar to (471)AT1G26830| Symbols: CUL3A, ATCUL3A, ATCUL3, CUL3 | ATCUL3/ATCUL3A/CUL3/CUL3A (Cullin 3A	
EE462377	2.563	no similarity	
JCVI_25684	2.563	weakly similar to (138)AT1G06570| Symbols: HPD, PDS1 | PDS1 (PHYTOENE DESATURATION 1) | chr1:2012014-2013542 REVERS	1.682
JCVI_28229	2.563	moderately similar to (252)AT5G46800| Symbols: BOU | BOU (A BOUT DE SOUFFLE); binding | chr5:19006006-19007037 REVERSE	
JCVI_18244	2.562	highly similar to (814)AT5G42310| Symbols: | pentatricopeptide (PPR) repeat-containing protein | chr5:16933088-16935466 FORWARD	
RC_AM386768	2.562	no similarity	
CX279615	2.562	moderately similar to (311)AT1G09155| Symbols: ATPP2-B15 | ATPP2-B15 (Phloem protein 2-B15); carbohydrate binding | chr1:294983	
L47854	2.561	no similarity	
JCVI_17907	2.561	highly similar to (664)AT3G48780| Symbols: | serine C-palmitoyltransferase, putative | chr3:18100331-18103261 FORWARD no original	
JCVI_10293	2.561	moderately similar to (363)AT4G03400| Symbols: GH3-10, DFL2 | DFL2 (DWARF IN LIGHT 2) | chr4:1497674-1499728 REVERSEwea	
JCVI_26729	2.561	moderately similar to (266)AT4G10050| Symbols: | hydrolase, alpha/beta fold family protein | chr4:6284617-6287183 REVERSE no origi	
DY016897	2.560	moderately similar to (233)AT5G61730| Symbols: ATATH11 | ATATH11 (ABC2 homolog 11); ATPase, coupled to transmembrane move	
EV087491	2.560	moderately similar to (278)AT5G13000| Symbols: GSL12, ATGSL12 | ATGSL12 (GLUCAN SYNTHASE-LIKE 12); 1,3-beta-glucan syn	
JCVI_1263	2.560	moderately similar to (491)AT2G01140| Symbols: | fructose-bisphosphate aldolase, putative | chr2:95005-96490 REVERSEmoderately sir	
ES949134	2.560	moderately similar to (277)AT2G29480| Symbols: GST20, ATGSTU2 | ATGSTU2 (GLUTATHIONE S-TRANSFERASE 20); glutathione	
JCVI_14738	2.560	moderately similar to (458)AT2G30710| Symbols: | RabGAP/TBC domain-containing protein | chr2:13093224-13096068 REVERSE no o	
JCVI_38112	2.559	no original description	
EV134150	2.559	moderately similar to (353)AT5G55930| Symbols: ATOPT1 | ATOPT1 (oligopeptide transporter 1); oligopeptide transporter | chr5:226702	
EX063904	2.558	no similarity	1.478
JCVI_7154	2.558	moderately similar to (249)AT5G62700| Symbols: ATGCP3, TUB3 | TUB3 (TUBULIN BETA-3); structural molecule | chr5:25201727-25	
JCVI_16672	2.558	moderately similar to (441)AT5G10830| Symbols: | embryo-abundant protein-related | chr5:3423732-3424650 FORWARD no original de	
EV138017	2.558	moderately similar to (317)AT3G06540| Symbols: | GDP dissociation inhibitor family protein / Rab GTPase activator family protein | chr3	
DY016304	2.557	weakly similar to (148)AT4G24330| Symbols: | similar to unknown protein [Arabidopsis thaliana] (TAIR:AT5G49945.1); similar to hypot	
EV209525	2.557	very weakly similar to (85.1)AT2G16890| Symbols: | UDP-glucoronosyl/UDP-glucosyl transferase family protein | chr2:7324020-7326104	-3.012
EE455917	2.557	no similarity	
JCVI_1345	2.557	moderately similar to (348)AT5G43190| Symbols: | F-box family protein (FBX6) | chr5:17357528-17358739 REVERSE no original descr	
JCVI_2042	2.557	highly similar to (531)AT2G20370| Symbols: KAM1, MUR3 | KAM1/MUR3 (MURUS 3); catalytic/ transferase, transferring glycosyl gro	
JCVI_2564	2.557	weakly similar to (125)AT2G28000| Symbols: CH-CPN60A, SLP, CPN60A | CPN60A (chloroplast / 60 kDa chaperonin alpha subunit); A	
ES985667	2.556	moderately similar to (226)AT5G55380| Symbols: | membrane bound O-acyl transferase (MBOAT) family protein / wax synthase-related	

EV042839	2.556	no similarity	
JCVI_17275	2.556	highly similar to (801)AT4G30190| Symbols: PMA2, AHA2 | AHA2 (Arabidopsis H(+)-ATPase 2); ATPase | chr4:14770826-14775926 R	
EE530829	2.556	moderately similar to (201)AT1G79610| Symbols: | sodium proton exchanger, putative (NHX6) | chr1:29957982-29961963 REVERSE [2	
JCVI_37498	2.556	moderately similar to (244)AT5G64290| Symbols: DCT, DIT2.1 | DCT/DIT2.1 (DICARBOXYLATE TRANSPORT); oxoglutarate:malate	
JCVI_1186	2.556	weakly similar to (193)AT2G39530| Symbols: | integral membrane protein, putative | chr2:16505737-16506479 REVERSE no original des	
JCVI_16699	2.555	weakly similar to (153)AT5G51260| Symbols: | acid phosphatase, putative | chr5:20849448-20850692 REVERSE no original description	-1.892
CX189592	2.554	no similarity	
JCVI_36926	2.554	moderately similar to (233)AT5G11710| Symbols: | (EPSIN1); binding | chr5:3772982-3776317 FORWARD no original description	
JCVI_27593	2.554	highly similar to (572)AT2G37710| Symbols: RLK | RLK (RECEPTOR LECTIN KINASE); kinase | chr2:15822012-15824039 REVERSE	
JCVI_5558	2.553	highly similar to (723)AT5G60160| Symbols: | aspartyl aminopeptidase, putative | chr5:24241113-24244009 REVERSEvery weakly simila	
JCVI_27659	2.552	very weakly similar to (93.2)AT1G72290| Symbols: | trypsin and protease inhibitor family protein / Kunitz family protein | chr1:27219514-	
JCVI_10236	2.552	moderately similar to (305)AT1G70580| Symbols: GGT2, AOAT2 | AOAT2 (GLUTAMATE:GLYOXYLATE AMINOTRANSFERASE 2	
EE439366	2.552	very weakly similar to (88.6)AT3G49350| Symbols: | RAB GTPase activator | chr3:18308648-18310831 REVERSE [20176] 19 256 256	
JCVI_37590	2.552	weakly similar to (181)AT1G77920| Symbols: | bZIP family transcription factor | chr1:29303853-29305501 FORWARD no original descr	
JCVI_28589	2.552	moderately similar to (392)AT2G41740| Symbols: VLN2 | VLN2 (VILLIN 2); actin binding | chr2:17418040-17423956 REVERSE no orig	2.555
EX088249	2.552	moderately similar to (274)AT2G40310| Symbols: | glycoside hydrolase family 28 protein / polygalacturonase (pectinase) family protein |	
CX192375	2.552	no similarity	
JCVI_33945	2.551	moderately similar to (294)AT5G05110| Symbols: | cysteine protease inhibitor, putative / cystatin, putative | chr5:1507616-1508766 REV	
JCVI_26170	2.551	highly similar to (660)AT1G79550| Symbols: PGK | PGK (PHOSPHOGLYCERATE KINASE) | chr1:29929240-29931188 REVERSEhigl	
ES903435	2.551	moderately similar to (354)AT1G79720| Symbols: | aspartyl protease family protein | chr1:30002152-30003844 REVERSE [21432] 16 78	
EX131453	2.551	moderately similar to (308)AT1G68370| Symbols: ARG1 | ARG1 (ALTERED RESPONSE TO GRAVITY 1) | chr1:25635709-25638190 I	
JCVI_3858	2.550	highly similar to (504)AT5G53350| Symbols: CLPX | CLPX (Clp protease regulatory subunit X); ATPase | chr5:21661286-21664729 FOR	
JCVI_17163	2.550	moderately similar to (389)AT4G35360| Symbols: | pantothenate kinase family protein | chr4:16812308-16814661 REVERSE no original	
JCVI_1881	2.549	moderately similar to (436)AT3G26650| Symbols: GAPA-1, GAPA | GAPA (GLYCERALDEHYDE 3-PHOSPHATE DEHYDROGENAS	
CN727135	2.549	moderately similar to (356)AT5G05460| Symbols: | hydrolase, acting on glycosyl bonds | chr5:1615616-1618772 FORWARD [15722]	
ES980906	2.548	moderately similar to (325)AT4G34100| Symbols: | similar to protein binding / zinc ion binding [Arabidopsis thaliana] (TAIR:AT4G3267	
JCVI_55	2.547	moderately similar to (413)AT4G04950| Symbols: | thioredoxin family protein | chr4:2517880-2519922 REVERSE no original description	
CD843565	2.547	no similarity	
DW997423	2.547	moderately similar to (248)AT5G20030| Symbols: | agenet domain-containing protein | chr5:6764973-6766038 REVERSE [18977]	
AM385243	2.547	no similarity	
EV217621	2.547	weakly similar to (101)AT5G66850| Symbols: MAPKKK5 | MAPKKK5 (Mitogen-activated protein kinase kinase kinase 5); kinase | chr5:	
AM394295	2.547	weakly similar to (149)AT5G59660| Symbols: | leucine-rich repeat protein kinase, putative | chr5:24052913-24057205 FORWARD [2034	
JCVI_26297	2.547	moderately similar to (207)AT4G29100| Symbols: | ethylene-responsive family protein | chr4:14341146-14344581 FORWARD no origina	-2.157
JCVI_19376	2.547	moderately similar to (304)AT2G21590| Symbols: APL4 | APL4 (large subunit of AGP 4); glucose-1-phosphate adenylyltransferase | chr2:	
EV194447	2.546	moderately similar to (302)AT1G73960| Symbols: TAF2 | TAF2 (TBP-ASSOCIATED FACTOR 2); binding / metallopeptidase/ zinc ion b	1.619
JCVI_834	2.546	moderately similar to (347)AT4G15093| Symbols: | catalytic LigB subunit of aromatic ring-opening dioxygenase family | chr4:8618451-8	
EX094705	2.545	weakly similar to (139)AT1G01640| Symbols: | speckle-type POZ protein-related | chr1:231164-231915 REVERSE [21824]	
JCVI_21385	2.545	moderately similar to (371)AT1G35720| Symbols: OXY5, ATOXY5, ANNAT1 | ANNAT1 (ANNEXIN ARABIDOPSIS 1); calcium ion b	
EV020846	2.545	moderately similar to (365)AT5G66360| Symbols: | ribosomal RNA adenine dimethylase family protein | chr5:26527555-26528937 FORV	
EV010667	2.544	no similarity	
EV044820	2.544	moderately similar to (376)AT1G76580| Symbols: | transcription factor | chr1:28740222-28743345 FORWARD [21442]	
EE418904	2.544	weakly similar to (140)AT3G12010| Symbols: | similar to Colon cancer-associated protein Mic1-like containing protein, expressed [Oryza	
JCVI_9931	2.543	moderately similar to (498)AT3G57330| Symbols: ACA11 | ACA11 (AUTOINHIBITED CA2+-ATPASE 11); calcium-transporting ATPa	
EV013306	2.543	no similarity	
JCVI_15279	2.543	moderately similar to (323)AT4G34490| Symbols: CAP 1, CAP1, ATCAP1 | ATCAP1 (CYCLASE ASSOCIATED PROTEIN 1) | chr4:16	
JCVI_20068	2.543	moderately similar to (254)AT2G36300| Symbols: | integral membrane Yip1 family protein | chr2:15220441-15221208 REVERSE no orig	
JCVI_1548	2.543	moderately similar to (412)AT3G21690| Symbols: | MATE efflux family protein | chr3:7638757-7641868 FORWARD no original descrip	
JCVI_29411	2.542	weakly similar to (156)AT4G26080| Symbols: ABI1 | ABI1 (ABA INSENSITIVE 1); calcium ion binding / protein serine/threonine phospl	
JCVI_14178	2.542	moderately similar to (424)AT3G14940| Symbols: ATPPC3 | ATPPC3 (PHOSPHOENOLPYRUVATE CARBOXYLASE 3); phosphoeno	
JCVI_20613	2.542	moderately similar to (206)AT3G06580| Symbols: GALK, GAL1 | GAL1 (GALACTOSE KINASE 1); ATP binding / galactokinase | chr3:	
ES904633	2.542	weakly similar to (129)AT5G66570| Symbols: PSBO-1, OEE1, OEE33, OE33, PSBO1 | OE33/OEE1/OEE33/PSBO-1/PSBO1 (OXYGEN-	
JCVI_3154	2.541	moderately similar to (406)AT1G17650| Symbols: | phosphogluconate dehydrogenase (decarboxylating) | chr1:6069587-6071957 REVER	
JCVI_163	2.541	moderately similar to (416)AT5G43280| Symbols: ATDCI1 | ATDCI1 (DELTA(3,5),DELTA(2,4)-DIENOYL-COA ISOMERASE 1) | chr	
JCVI_24093	2.540	very weakly similar to (81.3)AT2G23550| Symbols: | hydrolase | chr2:10034893-10035847 REVERSE no original description	
JCVI_24129	2.540	no original description	
EV182445	2.540	weakly similar to (156)AT3G08650| Symbols: | metal transporter family protein | chr3:2624700-2627314 REVERSE [21487] 39 727 727	
CD828441	2.540	weakly similar to (124)AT5G62930| Symbols: | GDSL-motif lipase/hydrolase family protein | chr5:25272138-25273583 FORWARD [139	
EX051667	2.539	no similarity	
H74917	2.539	very weakly similar to (84.7)AT5G42080| Symbols: ADL1A, AG68, DRP1A | ADL1 (ARABIDOPSIS DYNAMIN-LIKE PROTEIN); GTF	
EV092581	2.539	weakly similar to (145)AT3G49620| Symbols: DIN11 | DIN11 (DARK INDUCIBLE 11); oxidoreductase | chr3:18404808-18407647 FOR	
JCVI_4696	2.539	moderately similar to (329)AT1G55210| Symbols: | disease resistance response | chr1:20601724-20602287 REVERSE no original descrip	
JCVI_11292	2.538	moderately similar to (390)AT5G05730| Symbols: TRP5, AMT1, WEI2, ASA1 | ASA1 (ANTHRANILATE SYNTHASE ALPHA SUBUN	
EE460681	2.538	weakly similar to (132)AT5G25540| Symbols: CID6 | CID6 (CTC-Interacting Domain 6); protein binding | chr5:8891773-8892365 REVEI	-2.771
EE479356	2.538	no similarity	
EV049663	2.537	very weakly similar to (91.3)AT2G31470| Symbols: | F-box family protein | chr2:13414569-13415732 REVERSE [21442]	
JCVI_2681	2.537	moderately similar to (440)AT1G58360| Symbols: NAT2, AAP1 | AAP1 (AMINO ACID PERMEASE 1); amino acid transmembrane tran	
JCVI_9698	2.537	moderately similar to (312)AT2G29300| Symbols: | tropinone reductase, putative / tropine dehydrogenase, putative | chr2:12595291-1259	-2.849
JCVI_40271	2.537	moderately similar to (279)AT5G19050| Symbols: | similar to unnamed protein product [Vitis vinifera] (GB:CAO71093.1); contains Inter	
JCVI_36578	2.536	moderately similar to (422)AT4G24140| Symbols: | hydrolase, alpha/beta fold family protein | chr4:12530042-12533674 REVERSE no or	
JCVI_16189	2.536	moderately similar to (321)AT3G62970| Symbols: | protein binding / zinc ion binding | chr3:23281611-23283673 FORWARD no original	
JCVI_6581	2.536	moderately similar to (233)AT4G24090| Symbols: | similar to hypothetical protein [Vitis vinifera] (GB:CAN62286.1) | chr4:12512752-12	-1.391
JCVI_3793	2.535	moderately similar to (378)AT3G16990| Symbols: | TENA/THI-4 family protein | chr3:5795943-5796789 REVERSEmoderately similar to	
JCVI_2812	2.535	moderately similar to (226)AT5G44340| Symbols: TUB4 | TUB4 (tubulin beta-4 chain) | chr5:17876669-17878221 REVERSEmoderately	
JCVI_7703	2.534	weakly similar to (138)AT1G22090| Symbols: EMB2204 | EMB2204 (EMBRYO DEFECTIVE 2204) | chr1:7795710-7797241 FORWAR	
JCVI_38435	2.534	moderately similar to (264)AT3G11850| Symbols: | similar to unknown protein [Arabidopsis thaliana] (TAIR:AT5G06560.1); similar to u	
JCVI_24985	2.534	moderately similar to (222)AT2G31560| Symbols: | similar to unknown protein [Arabidopsis thaliana] (TAIR:AT1G05870.1); similar to u	
EV041296	2.534	moderately similar to (351)AT3G13490| Symbols: OVA5 | OVA5 (OVULE ABORTION 5); ATP binding / aminoacyl-tRNA ligase | chr3:	
JCVI_841	2.534	moderately similar to (454)AT1G21750| Symbols: ATPDIL1-1 | ATPDIL1-1 (PDI-LIKE 1-1); protein disulfide isomerase | chr1:7645756-	
AT000721	2.533	no similarity	
CD826627	2.533	moderately similar to (289)AT5G61390| Symbols: | exonuclease family protein | chr5:24696028-24698083 REVERSE [13979]	
JCVI_38154	2.533	moderately similar to (427)AT1G26640| Symbols: | aspartate/glutamate/uridylate kinase family protein | chr1:9207607-9209753 REVERS	

JCVI_12154	2.532	highly similar to (659)AT4G29010| Symbols: AIM1 | AIM1 (ABNORMAL INFLORESCENCE MERISTEM); enoyl-CoA hydratase | chr4	
JCVI_17962	2.532	weakly similar to (162)AT5G44930| Symbols: ARAD2 | ARAD2 (ARABINAN DEFICIENT 2); catalytic | chr5:18157853-18159371 REV	
EV027253	2.532	moderately similar to (322)AT1G77770| Symbols: | protein binding / zinc ion binding | chr1:29251375-29252169 REVERSE [21441]	
EV038679	2.532	weakly similar to (182)AT1G54150| Symbols: | zinc finger (C3HC4-type RING finger) family protein | chr1:20219147-20220970 FORWA	
JCVI_1950	2.532	moderately similar to (402)AT2G43710| Symbols: FAB2, SSI2 | SSI2 (fatty acid biosynthesis 2); acyl-[acyl-carrier-protein] desaturase | ch	1.915
EV214299	2.531	no similarity	
JCVI_11575	2.531	moderately similar to (393)AT5G10910| Symbols: | mraW methylase family protein | chr5:3439589-3441518 FORWARD no original desc	
EH420923	2.531	moderately similar to (297)AT3G09580| Symbols: | amine oxidase family protein | chr3:2942619-2944052 REVERSE [20767]	
JCVI_3707	2.531	moderately similar to (361)AT3G60880| Symbols: | dihydrodipicolinate synthase 1 (DHDPS1) (DHDPS) (DHPS1) | chr3:22506061-22507	
JCVI_583	2.530	moderately similar to (352)AT3G04790| Symbols: | ribose 5-phosphate isomerase-related | chr3:1313371-1314201 FORWARD no origina	-2.394
JCVI_18301	2.530	moderately similar to (262)AT2G04040| Symbols: ATDTX1 | ATDTX1; antiporter/ multidrug efflux pump/ multidrug transporter/ transpo	
JCVI_18375	2.529	highly similar to (504)AT3G07790| Symbols: | DGCR14-related | chr3:2487497-2489023 FORWARD no original description	
JCVI_16108	2.529	moderately similar to (401)AT4G39820| Symbols: | binding | chr4:18476692-18477918 REVERSE no original description	
AM386444	2.529	very weakly similar to (94.0)AT2G45680| Symbols: | TCP family transcription factor, putative | chr2:18827791-18828861 REVERSE [201	
JCVI_42224	2.529	no original description	
EV115684	2.529	moderately similar to (206)AT1G01580| Symbols: FRD1, ATFRO2, FRO2 | FRO2 (FERRIC REDUCTION OXIDASE 2); ferric-chelate re	
JCVI_20648	2.528	highly similar to (581)AT5G60390| Symbols: | elongation factor 1-alpha / EF-1-alpha | chr5:24306452-24307901 FORWARDhighly simila	
JCVI_26500	2.528	moderately similar to (407)AT1G50920| Symbols: | GTP-binding protein-related | chr1:18874223-18876238 FORWARD no original descr	
EX092717	2.528	moderately similar to (409)AT5G45170| Symbols: | CbbY protein-related | chr5:18287782-18290356 REVERSE [21823]	
JCVI_101	2.528	moderately similar to (477)AT3G48170| Symbols: ALDH10A9 | ALDH10A9 (Aldehyde dehydrogenase 10A9); 3-chloroallyl aldehyde deh	
JCVI_16496	2.528	moderately similar to (446)AT1G64710| Symbols: | alcohol dehydrogenase, putative | chr1:24048499-24049976 FORWARDmoderately si	
DN964807	2.528	weakly similar to (178)AT3G15650| Symbols: | phospholipase/carboxylesterase family protein | chr3:5306013-5307771 FORWARD [173	
EE507345	2.528	weakly similar to (112)AT5G22440| Symbols: | 60S ribosomal protein L10A (RPL10aC) | chr5:7435331-7436489 REVERSE [15718]	
JCVI_12084	2.527	moderately similar to (233)AT5G65940| Symbols: CHY1 | CHY1 (BETA-HYDROXYISOBUTYRYL-COA HYDROLASE 1) | chr5:2639	
JCVI_29834	2.527	no original description	
JCVI_29372	2.527	weakly similar to (154)AT4G35300| Symbols: TMT2 | TMT2 (TONOPLAST MONOSACCHARIDE TRANSPORTER2); carbohydrate tr	
JCVI_5997	2.527	moderately similar to (417)AT3G59360| Symbols: UTR6, ATUTR6 | ATUTR6/UTR6 (UDP-GALACTOSE TRANSPORTER 6); nucleoti	
EV012575	2.527	no similarity	
JCVI_5568	2.527	moderately similar to (476)AT3G22200| Symbols: HER1, POP2, GABA-T | POP2 (POLLEN-PISTIL INCOMPATIBILITY 2); 4-aminobu	5.272
JCVI_8412	2.526	weakly similar to (116)AT4G17520| Symbols: | nuclear RNA-binding protein, putative | chr4:9771509-9773326 FORWARD no original d	
JCVI_14120	2.526	moderately similar to (344)AT4G32960| Symbols: | similar to unknown protein [Arabidopsis thaliana] (TAIR:AT4G32970.1); similar to u	-3.892
JCVI_17463	2.526	moderately similar to (389)AT5G49650| Symbols: | xylulose kinase, putative | chr5:20170124-20172800 FORWARD no original descripti	
JCVI_13395	2.526	moderately similar to (330)AT5G52390| Symbols: | photoassimilate-responsive protein, putative | chr5:21281507-21282399 REVERSE no	
ES952545	2.526	no similarity	
JCVI_20165	2.526	moderately similar to (436)AT1G77990| Symbols: SULTR2;2, AST56 | AST56 (sulphate transporter 2;2); sulfate transmembrane transpor	
JCVI_4174	2.525	moderately similar to (392)AT5G60620| Symbols: | phospholipid/glycerol acyltransferase family protein | chr5:24384492-24386873 FORV	
JCVI_9591	2.524	moderately similar to (396)AT5G33370| Symbols: | GDSL-motif lipase/hydrolase family protein | chr5:12620094-12621900 REVERSEwe	
JCVI_5826	2.524	weakly similar to (102)AT5G16010| Symbols: | 3-oxo-5-alpha-steroid 4-dehydrogenase family protein / steroid 5-alpha-reductase family p	
EE531751	2.524	moderately similar to (375)AT2G41140| Symbols: CRK1 | CRK1 (CDPK-RELATED KINASE 1); calcium ion binding / calcium-depender	
ES993348	2.524	very weakly similar to (99.4)AT1G64850| Symbols: | calcium-binding EF hand family protein | chr1:24100211-24101237 REVERSE [2142	
DY013648	2.524	no similarity	
JCVI_7179	2.524	moderately similar to (367)AT5G48230| Symbols: EMB1276, ACAT2 | ACAT2/EMB1276 (ACETOACETYL-COA THIOLASE 2); acety	
JCVI_14137	2.524	highly similar to (725)AT1G09020| Symbols: ATSNF4, SNF4 | SNF4 (Sucrose NonFermenting 4) | chr1:2900152-2904215 REVERSE no	
DY016151	2.524	weakly similar to (105)AT5G50340| Symbols: | ATP binding / ATP-dependent peptidase/ damaged DNA binding / nucleoside-triphosphat	
JCVI_28028	2.523	no original description	
EX099372	2.523	moderately similar to (204)AT1G13190| Symbols: | RNA recognition motif (RRM)-containing protein | chr1:4499630-4501351 FORWAR	
JCVI_13298	2.522	moderately similar to (252)AT4G04610| Symbols: APR, PRH19, ATAPR1, APR1 | APR1 (PAPS REDUCTASE HOMOLOG 19) | chr4:2	
JCVI_4440	2.522	moderately similar to (329)AT1G04980| Symbols: ATPDIL2-2 | ATPDIL2-2 (PDI-LIKE 2-2); thiol-disulfide exchange intermediate | chr1	
JCVI_3820	2.522	moderately similar to (456)AT5G03290| Symbols: | isocitrate dehydrogenase, putative / NAD+ isocitrate dehydrogenase, putative | chr5:79	
ES981667	2.522	weakly similar to (136)AT1G12760| Symbols: | protein binding / zinc ion binding | chr1:4348939-4350510 FORWARD [21388]	
JCVI_10411	2.522	moderately similar to (452)AT5G43340| Symbols: PHT6 | PHT6 (phosphate transporter 6); carbohydrate transmembrane transporter/ phos	
CV433253	2.522	no similarity	-3.905
JCVI_39833	2.521	moderately similar to (272)AT3G16740| Symbols: | F-box family protein | chr3:5699482-5700657 FORWARD no original description	1.340
JCVI_8579	2.521	weakly similar to (126)AT4G14020| Symbols: | rapid alkalinization factor (RALF) family protein | chr4:8095365-8095700 REVERSE no	2.349
JCVI_28781	2.521	weakly similar to (104)AT1G09645| Symbols: | similar to unknown protein [Arabidopsis thaliana] (TAIR:AT1G57765.1); similar to unnar	
JCVI_14700	2.521	highly similar to (502)AT3G62830| Symbols: AUD1, ATUXS2, UXS2 | UXS2 (UDP-GLUCURONIC ACID DECARBOXYLASE 2) | chr	
JCVI_24359	2.521	moderately similar to (328)AT3G26200| Symbols: CYP71B22 | CYP71B22 (cytochrome P450, family 71, subfamily B, polypeptide 22); o	
JCVI_16011	2.521	moderately similar to (421)AT2G15240| Symbols: | UNC-50 family protein | chr2:6623062-6625944 REVERSE no original description	
CV432133	2.521	weakly similar to (165)AT1G79810| Symbols: PEX2, TED3 | TED3 (REVERSAL OF THE DET PHENOTYPE); protein binding / zinc ion	
JCVI_30449	2.520	moderately similar to (408)AT1G19190| Symbols: | hydrolase | chr1:6623867-6624823 FORWARDvery weakly similar to (89.0)GID1_OI	
JCVI_25411	2.520	no original description	
JCVI_9896	2.519	weakly similar to (198)AT3G56240| Symbols: CCH | CCH (COPPER CHAPERONE) | chr3:20874439-20875381 REVERSE no original d	
EV180370	2.519	weakly similar to (118)AT5G52840| Symbols: | NADH-ubiquinone oxidoreductase-related | chr5:21430944-21432020 FORWARD [2148	
EV227118	2.519	weakly similar to (115)AT2G45220| Symbols: | pectinesterase family protein | chr2:18651355-18653468 REVERSEvery weakly similar to	
JCVI_33145	2.518	very weakly similar to (94.0)AT1G29380| Symbols: | similar to unknown protein [Arabidopsis thaliana] (TAIR:AT2G30933.2); similar to u	
EV205431	2.518	no similarity	
JCVI_25114	2.517	no original description	
JCVI_4867	2.517	moderately similar to (235)AT1G70690| Symbols: | kinase-related | chr1:26655762-26657044 FORWARD no original description	
JCVI_4536	2.517	no original description	
JCVI_40691	2.517	very weakly similar to (87.0)AT4G24770| Symbols: ATRBP31, CP31, ATRBP33, RBP31 | RBP31 (31-KDA RNA BINDING PROTEIN);	
JCVI_38511	2.517	weakly similar to (118)AT2G01520| Symbols: MLP328 | MLP328 (MLP-LIKE PROTEIN 328) | chr2:235991-236880 FORWARD no orig	
EE424791	2.517	moderately similar to (271)AT3G06450| Symbols: | anion exchange family protein | chr3:1976091-1979309 REVERSE [20158]	
EV205596	2.516	weakly similar to (184)AT2G17150| Symbols: | RWP-RK domain-containing protein | chr2:7475213-7477615 REVERSE [21491] 51 736	
EX042317	2.516	weakly similar to (119)AT2G33620| Symbols: | DNA-binding family protein / AT-hook protein 1 (AHP1) | chr2:14241825-14243639 FOF	-3.772
RC_EE562372	2.516	no similarity	
JCVI_27801	2.516	moderately similar to (317)AT2G16530| Symbols: | 3-oxo-5-alpha-steroid 4-dehydrogenase family protein / steroid 5-alpha-reductase fam	
EV138903	2.515	no similarity	-1.854
JCVI_32876	2.515	moderately similar to (499)AT4G00550| Symbols: DGD2 | DGD2 (digalactosyldiacylglycerol synthase 2); digalactosyldiacylglycerol synth	
JCVI_28009	2.514	moderately similar to (241)AT3G19950| Symbols: | zinc finger (C3HC4-type RING finger) family protein | chr3:6942859-6943845 FORW	
JCVI_12450	2.514	highly similar to (560)AT1G70770| Symbols: | similar to unknown protein [Arabidopsis thaliana] (TAIR:AT1G23170.1); similar to hypotl	
JCVI_29633	2.514	moderately similar to (483)AT3G26820| Symbols: | esterase/lipase/thioesterase family protein | chr3:9882365-9886304 FORWARD no or	

AM385389	2.514	very weakly similar to (85.5)AT1G35340| Symbols: | ATP-dependent protease La (LON) domain-containing protein | chr1:12977746-1297	
JCVI_28788	2.514	very weakly similar to (99.0)AT5G43570| Symbols: | serine-type endopeptidase inhibitor | chr5:17523681-17524683 FORWARD no origin	
EE448492	2.513	weakly similar to (124)AT5G54980| Symbols: | integral membrane family protein | chr5:22333025-22333786 FORWARD [20172]	
CK991375	2.513	weakly similar to (185)AT5G20850| Symbols: RAD51, ATRAD51 | ATRAD51 (Arabidopsis thaliana Ras Associated with Diabetes protein	
JCVI_27961	2.512	moderately similar to (310)AT5G53280| Symbols: PDV1 | PDV1 (PLASTID DIVISION1) | chr5:21624812-21626306 FORWARD no orig	
JCVI_28429	2.512	highly similar to (543)AT4G34131| Symbols: UGT73B3 | UGT73B3 (UDP-GLUCOSYL TRANSFERASE 73B3); UDP-glycosyltransferas	
EX110829	2.512	weakly similar to (180)AT4G04830| Symbols: | methionine sulfoxide reductase domain-containing protein / SeIR domain-containing prote	
JCVI_5252	2.512	moderately similar to (393)AT4G18910| Symbols: NIP1;2, NLM2 | NIP1;2/NLM2 (NOD26-like intrinsic protein 1;2); water channel | chr4	-2.500
EX032103	2.512	no similarity	
JCVI_6758	2.512	moderately similar to (221)AT2G39795| Symbols: | mitochondrial glycoprotein family protein / MAM33 family protein | chr2:16604104-1	
ES264159	2.511	moderately similar to (378)AT4G37640| Symbols: ACA2 | ACA2 (CALCIUM ATPASE 2); calmodulin binding | chr4:17683219-1768680	
RC_AM386400	2.511	no similarity	
JCVI_39171	2.511	moderately similar to (489)AT4G31160| Symbols: | transducin family protein / WD-40 repeat family protein | chr4:15145942-15152945 F	-2.748
JCVI_13837	2.511	no original description	
JCVI_18969	2.510	moderately similar to (348)AT1G22540| Symbols: | proton-dependent oligopeptide transport (POT) family protein | chr1:7964191-796621	
JCVI_2144	2.510	moderately similar to (379)AT2G43750| Symbols: ACS1, CPACS1, ATCS-B, OASB | OASB (O-ACETYLSERINE (THIOL) LYASE B);	-2.987
RC_EE559385	2.510	no similarity	
JCVI_13139	2.510	highly similar to (861)AT1G12200| Symbols: | flavin-containing monooxygenase family protein / FMO family protein | chr1:4137625-413	
EX131692	2.510	moderately similar to (332)AT1G72120| Symbols: | transporter | chr1:27135795-27137637 FORWARD [21833]	
JCVI_11711	2.509	highly similar to (541)AT2G26600| Symbols: | glycosyl hydrolase family 17 protein | chr2:11324292-11325293 FORWARDweakly simila	
EV193597	2.508	no similarity	-2.614
JCVI_39045	2.508	moderately similar to (227)AT1G77920| Symbols: | bZIP family transcription factor | chr1:29303853-29305501 FORWARDvery weakly s	
JCVI_27607	2.508	moderately similar to (351)AT1G77120| Symbols: ADH, ATADH, ADH1 | ADH1 (ALCOHOL DEHYDROGENASE 1) | chr1:28980403-	
EX118563	2.508	no similarity	-2.544
JCVI_39356	2.508	weakly similar to (152)AT3G59520| Symbols: | rhomboid family protein | chr3:22003031-22003840 FORWARD no original description	
EE520875	2.508	moderately similar to (231)AT3G10770| Symbols: | nucleic acid binding | chr3:3372466-3374031 REVERSE [20185]	
EV187288	2.507	moderately similar to (336)AT1G74780| Symbols: | nodulin family protein | chr1:28099577-28101573 FORWARD [21488] 49 732 732	
JCVI_37116	2.507	highly similar to (542)AT5G17520| Symbols: MEX1, RCP1 | RCP1 (ROOT CAP 1) | chr5:5772798-5775233 REVERSEmoderately simila	
JCVI_33071	2.507	moderately similar to (347)AT1G34130| Symbols: STT3B | STT3B (STAUROSPORIN AND TEMPERATURE SENSITIVE 3-LIKE B);	
ES911636	2.507	weakly similar to (164)AT5G05500| Symbols: | pollen Ole e 1 allergen and extensin family protein | chr5:1629717-1630268 FORWARD [	
JCVI_23784	2.507	no original description	
JCVI_12679	2.506	no original description	
JCVI_35617	2.506	very weakly similar to (82.4)AT3G22560| Symbols: | GCN5-related N-acetyltransferase (GNAT) family protein | chr3:7998922-7999449 F	
EE473027	2.506	very weakly similar to (91.3)AT1G02450| Symbols: NIMIN1, NIMIN-1 | NIMIN-1/NIMIN1; protein binding | chr1:498052-498480 REVE	1.415
ES969649	2.506	no similarity	
EV189195	2.506	moderately similar to (270)AT2G21410| Symbols: VHA-A2 | VHA-A2 (VACUOLAR PROTON ATPASE A2); ATPase | chr2:9169783-9	
JCVI_6113	2.505	moderately similar to (268)AT1G12550| Symbols: | oxidoreductase family protein | chr1:4274647-4275829 FORWARD no original descri	
EX037271	2.505	weakly similar to (181)AT1G18750| Symbols: AGL65 | AGL65; DNA binding / transcription factor | chr1:6467395-6469689 FORWARD	
JCVI_8198	2.505	moderately similar to (216)AT5G54800| Symbols: GPT1 | GPT1 (glucose-6-phosphate transporter 1); antiporter/ glucose-6-phosphate tran	
EV025924	2.504	moderately similar to (335)AT5G60300| Symbols: | lectin protein kinase family protein | chr5:24282088-24285199 FORWARD [21441]	
JCVI_39821	2.504	highly similar to (553)AT3G03300| Symbols: DCL2 | DCL2 (DICER-LIKE 2) | chr3:768027-774532 REVERSE no original description	
AM388932	2.504	moderately similar to (320)AT2G48010| Symbols: RKF3 | RKF3 (RECEPTOR-LIKE KINASE IN IN FLOWERS 3); kinase | chr2:196485	
JCVI_36754	2.504	moderately similar to (249)AT5G25500| Symbols: | similar to unnamed protein product [Vitis vinifera] (GB:CAO43931.1) | chr5:8881121	
ES900619	2.504	moderately similar to (232)AT1G54060| Symbols: | transcription factor | chr1:20184640-20185791 FORWARD [21428]	
JCVI_15136	2.503	moderately similar to (230)AT5G07990| Symbols: CYP75B1, D501, TT7 | TT7 (TRANSPARENT TESTA 7); flavonoid 3'-monooxygenas	
EE531709	2.503	no similarity	
JCVI_5600	2.503	weakly similar to (188)AT4G10790| Symbols: | UBX domain-containing protein | chr4:6640748-6643031 REVERSE no original descripti	
EX029905	2.502	weakly similar to (101)AT2G32870| Symbols: | meprin and TRAF homology domain-containing protein / MATH domain-containing prote	
JCVI_5923	2.502	moderately similar to (428)AT2G26060| Symbols: EMB1345 | EMB1345 (EMBRYO DEFECTIVE 1345); nucleotide binding | chr2:1110	
ES899535	2.502	no similarity	
EE522738	2.502	weakly similar to (193)AT3G20930| Symbols: | RNA recognition motif (RRM)-containing protein | chr3:7331745-7333755 FORWARD [	
EX136527	2.502	weakly similar to (168)AT1G17290| Symbols: ALAAT1 | ALAAT1 (ALANINE AMINOTRANSFERAS); alanine transaminase | chr1:592	
JCVI_15723	2.502	moderately similar to (370)AT1G30540| Symbols: | ATPase, BadF/BadG/BcrA/BcrD-type family | chr1:10816933-10819154 REVERSE	
JCVI_19896	2.502	moderately similar to (374)AT1G02150| Symbols: | pentatricopeptide (PPR) repeat-containing protein | chr1:408779-410433 FORWARD	
EE432166	2.502	weakly similar to (130)AT1G12890| Symbols: | AP2 domain-containing transcription factor, putative | chr1:4391732-4392391 FORWARI	
JCVI_11796	2.501	moderately similar to (271)AT5G19500| Symbols: | tryptophan/tyrosine permease family protein | chr5:6579021-6581701 FORWARD no	
EE404244	2.501	moderately similar to (236)AT3G22410| Symbols: | similar to unknown protein [Arabidopsis thaliana] (TAIR:AT1G05370.1); similar to u	
JCVI_38508	2.501	moderately similar to (259)AT3G05200| Symbols: ATL6 | ATL6 (Arabidopsis T?xicos en Levadura 6); protein binding / zinc ion binding |	
EE435290	2.501	weakly similar to (124)AT3G47850| Symbols: | similar to hypothetical protein [Vitis vinifera] (GB:CAN75379.1) | chr3:17665241-17666	
AM062037	2.501	moderately similar to (286)AT1G01220| Symbols: | GHMP kinase-related | chr1:91750-95552 FORWARD [17712]	
JCVI_2430	2.500	moderately similar to (454)AT3G03250| Symbols: UGP | UGP (UDP-glucose pyrophosphorylase); UTP:glucose-1-phosphate uridylyltrans	
JCVI_1602	2.500	moderately similar to (263)AT4G15630| Symbols: | integral membrane family protein | chr4:8917525-8918681 FORWARD no original de	
JCVI_4027	2.500	moderately similar to (285)AT1G55530| Symbols: | zinc finger (C3HC4-type RING finger) family protein | chr1:20733138-20734193 RE	
EG020475	2.500	moderately similar to (206)AT5G64000| Symbols: ATSAL2, SAL2 | SAL2; 3'(2'),5'-bisphosphate nucleotidase/ inositol or phosphatidylino	
JCVI_11826	2.500	highly similar to (612)AT3G10230| Symbols: LYC | LYC (LYCOPENE CYCLASE) | chr3:3164345-3165454 REVERSEmoderately simila	
JCVI_2839	2.499	weakly similar to (105)AT5G03495| Symbols: | nucleotide binding | chr5:873803-875024 REVERSE no original description	
JCVI_20808	2.499	highly similar to (836)AT4G34100| Symbols: | similar to protein binding / zinc ion binding [Arabidopsis thaliana] (TAIR:AT4G32670.1);	
JCVI_5685	2.499	weakly similar to (180)AT5G22650| Symbols: HDT02, HDT2, ATHD2B, HDA4, HD2, HD2B | HD2B (HISTONE DEACETYLASE 2B) |	
JCVI_10256	2.499	no original description	1.653
EE473563	2.498	moderately similar to (324)AT1G17120| Symbols: CAT8 | CAT8 (CATIONIC AMINO ACID TRANSPORTER 8); cationic amino acid tra	
JCVI_13419	2.498	highly similar to (570)AT1G16870| Symbols: | mitochondrial 28S ribosomal protein S29-related | chr1:5770957-5773150 REVERSE no o	
JCVI_42312	2.498	very weakly similar to (96.3)AT5G64550| Symbols: | loricrin-related | chr5:25819020-25820924 REVERSE no original description	
JCVI_4448	2.497	highly similar to (536)AT5G44070| Symbols: ARA8, ATPCS1, PCS1, CAD1 | CAD1 (CADMIUM SENSITIVE 1) | chr5:17752103-17754	
EE557221	2.497	no similarity	-4.852
EV142121	2.497	moderately similar to (273)AT5G10770| Symbols: | chloroplast nucleoid DNA-binding protein, putative | chr5:3403332-3405332 REVERS	
JCVI_33046	2.496	highly similar to (627)AT1G43670| Symbols: | fructose-1,6-bisphosphatase, putative / D-fructose-1,6-bisphosphate 1-phosphohydrolase, p	
EX125153	2.496	moderately similar to (468)AT1G74210| Symbols: | glycerophosphoryl diester phosphodiesterase family protein | chr1:27914057-2791644	
JCVI_40180	2.495	weakly similar to (140)AT4G23600| Symbols: JR2, CORI3 | CORI3 (CORONATINE INDUCED 1, JASMONIC ACID RESPONSIVE 2);	
JCVI_19427	2.495	no original description	-1.838
JCVI_4473	2.495	moderately similar to (353)AT5G23240| Symbols: | DNAJ heat shock N-terminal domain-containing protein | chr5:7826860-7828537 RE	
CD827548	2.495	very weakly similar to (94.4)AT5G04560| Symbols: DME1, DME | DME (DEMETER) | chr5:1309787-1318092 FORWARD [13979]	

JCVI_38433	2.495	moderately similar to (332)AT2G17640| Symbols: SAT-106, AtSerat3;1 | AtSerat3;1 (SERINE ACETYLTRANSFERASE-106); acetyltran	-1.862
EX087334	2.494	moderately similar to (269)AT5G22370| Symbols: EMB1705, QQT1 | EMB1705/QQT1 (QUATRE-QUART1) | chr5:7406590-7408512 F	
JCVI_25161	2.494	moderately similar to (277)AT4G22180| Symbols: | F-box family protein | chr4:11738586-11739794 FORWARD no original description	
EE448719	2.494	moderately similar to (240)AT3G59210| Symbols: | F-box family protein | chr3:21900919-21902530 FORWARD [20172]	
JCVI_27613	2.494	moderately similar to (361)AT5G56840| Symbols: | DNA-binding family protein | chr5:22998015-22999378 FORWARD no original descr	
JCVI_884	2.494	moderately similar to (485)AT3G20500| Symbols: ATPAP18, PAP18 | ATPAP18/PAP18 (purple acid phosphatase 18); acid phosphatase/	
EV177308	2.494	weakly similar to (200)AT4G10080| Symbols: | similar to unknown protein [Arabidopsis thaliana] (TAIR:AT4G13530.1); similar to unnan	
JCVI_9921	2.494	highly similar to (702)AT5G23880| Symbols: EMB1265, CPSF100, ESP5, ATCPSF100 | ATCPSF100/CPSF100/EMB1265/ESP5 (CLEAV	
EV189475	2.493	weakly similar to (108)AT5G44130| Symbols: FLA13 | FLA13 (FASCICLIN-LIKE ARABINOGALACTAN PROTEIN 13 PRECURSOR)	
EV133596	2.493	weakly similar to (196)AT5G52990| Symbols: | vesicle-associated membrane protein-related | chr5:21501414-21502232 FORWARD [214	
JCVI_12829	2.493	moderately similar to (288)AT5G12010| Symbols: | similar to unknown protein [Arabidopsis thaliana] (TAIR:AT4G29780.1); similar to u	
JCVI_99	2.493	moderately similar to (457)AT1G19150| Symbols: LHCA2*1, LHCA6 | LHCA6 (Photosystem I light harvesting complex gene 6); chloropl	
JCVI_40979	2.493	very weakly similar to (100)AT3G20340| Symbols: | Expression of the gene is downregulated in the presence of paraquat, an inducer of pl	
EE462145	2.493	very weakly similar to (83.6)AT5G10810| Symbols: ATER | ATER (Arabidopsis thaliana enhancer of rudimentary homologue) | chr5:3418	
JCVI_18322	2.492	moderately similar to (434)AT4G01630| Symbols: ATEXP17, ATHEXP ALPHA 1.13, ATEXPA17 | ATEXPA17 (ARABIDOPSIS THAL	
EX103360	2.491	moderately similar to (324)AT3G51250| Symbols: | senescence/dehydration-associated protein-related | chr3:19039206-19041421 FORW	
ES991355	2.491	weakly similar to (118)AT4G27040| Symbols: | similar to unknown protein [Arabidopsis thaliana] (TAIR:AT3G31960.1); similar to unnan	
CX194419	2.491	no similarity	
JCVI_16819	2.490	weakly similar to (173)AT5G65060| Symbols: AGL70, FCL3, MAF3 | MAF3 (MADS AFFECTING FLOWERING 3); transcription factor	
JCVI_35402	2.490	highly similar to (559)AT1G14080| Symbols: ATFUT6, FUT6 | FUT6 (fucosyltransferase 6); fucosyltransferase/ transferase, transferring g	
EV165776	2.490	very weakly similar to (84.3)AT5G48850| Symbols: | male sterility MS5 family protein | chr5:19822802-19824925 REVERSE [21486] 88	
JCVI_8923	2.490	moderately similar to (457)AT2G26510| Symbols: PDE135 | PDE135 (PIGMENT DEFECTIVE EMBRYO 135); transmembrane transpor	
JCVI_6090	2.490	moderately similar to (360)AT4G27650| Symbols: PEL1 | PEL1 (PELOTA); translation release factor | chr4:13803465-13807562 REVER	
JCVI_2904	2.489	moderately similar to (298)AT5G11810| Symbols: | similar to Os02g0135600 [Oryza sativa (japonica cultivar-group)] (GB:NP_00104581	
JCVI_5780	2.489	moderately similar to (386)AT2G39420| Symbols: | esterase/lipase/thioesterase family protein | chr2:16467520-16469950 FORWARD no	
JCVI_37420	2.488	moderately similar to (206)AT5G11150| Symbols: VAMP713, ATVAMP713 | ATVAMP713 (Arabidopsis thaliana vesicle-associated mer	-1.826
EV097689	2.488	weakly similar to (165)AT1G71030| Symbols: ATMYBL2 | ATMYBL2 (Arabidopsis myb-like 2); DNA binding / transcription factor | chr	
ES981692	2.488	weakly similar to (194)AT4G24280| Symbols: CPHSC70-1 | CPHSC70-1 (chloroplast heat shock protein 70-1); ATP binding / unfolded pr	
JCVI_20476	2.488	weakly similar to (142)AT5G48490| Symbols: | protease inhibitor/seed storage/lipid transfer protein (LTP) family protein | chr5:19665158	
JCVI_25925	2.487	moderately similar to (288)AT1G20132| Symbols: | hydrolase, acting on ester bonds / lipase | chr1:6981349-6983486 FORWARDmoderat	
JCVI_28171	2.487	highly similar to (620)AT3G55350| Symbols: | similar to unknown protein [Arabidopsis thaliana] (TAIR:AT3G63270.1); similar to unnam	
JCVI_26553	2.487	moderately similar to (253)AT3G21710| Symbols: | unknown protein | chr3:7648387-7649540 FORWARD no original description	
EL592778	2.487	no similarity	
CD828788	2.487	moderately similar to (413)AT1G09910| Symbols: | lyase | chr1:3220153-3224453 REVERSE [13979]	
ES919067	2.487	moderately similar to (305)AT4G15240| Symbols: | fringe-related protein | chr4:8703518-8705378 REVERSE [15718] 1 746 762	
EE426477	2.486	moderately similar to (287)AT5G51100| Symbols: FSD2 | FSD2 (FE SUPEROXIDE DISMUTASE 2); iron superoxide dismutase | chr5:20	
JCVI_3940	2.485	moderately similar to (407)AT1G80670| Symbols: | transducin family protein / WD-40 repeat family protein | chr1:30325701-30328435 R	
JCVI_27638	2.485	moderately similar to (361)AT5G03350| Symbols: | legume lectin family protein | chr5:815803-816627 REVERSE no original description	
JCVI_11645	2.485	moderately similar to (328)AT3G62750| Symbols: | hydrolase, hydrolyzing O-glycosyl compounds | chr3:23225351-23227876 FORWARI	
JCVI_29563	2.484	moderately similar to (319)AT3G54910| Symbols: | similar to F-box family protein [Arabidopsis thaliana] (TAIR:AT4G10400.2); similar	
ES990272	2.484	no similarity	
JCVI_24292	2.483	moderately similar to (490)AT1G30160| Symbols: | similar to unknown protein [Arabidopsis thaliana] (TAIR:AT1G05540.1); contains Int	
JCVI_32372	2.483	highly similar to (572)AT5G02500| Symbols: HSP70-1, AT-HSC70-1, HSC70, HSC70-1 | HSC70-1 (heat shock cognate 70 kDa protein 1)	
EE468587	2.483	moderately similar to (363)AT5G11480| Symbols: | GTP binding | chr5:3669351-3671472 REVERSE [20156]	
JCVI_8091	2.483	moderately similar to (343)AT5G55130| Symbols: SIR1, CNX5 | CNX5 (SIRTINOL RESISTANT 1); Mo-molybdopterin cofactor sulfuras	
JCVI_12640	2.483	highly similar to (718)AT4G24280| Symbols: CPHSC70-1 | CPHSC70-1 (chloroplast heat shock protein 70-1); ATP binding / unfolded pro	
EE486039	2.483	weakly similar to (119)AT5G05760| Symbols: ATSED5, ATSYP31, SED5, SYP31 | SYP31 (T-SNARE SED 5); SNAP receptor | chr5:172	
JCVI_23715	2.482	moderately similar to (339)AT3G62130| Symbols: | epimerase-related | chr3:23015969-23017333 FORWARD no original description	
JCVI_5063	2.482	highly similar to (513)AT5G44340| Symbols: TUB4 | TUB4 (tubulin beta-4 chain) | chr5:17876669-17878221 REVERSEmoderately simil	
JCVI_40369	2.482	moderately similar to (236)AT2G34960| Symbols: CAT5 | CAT5 (CATIONIC AMINO ACID TRANSPORTER 5); cationic amino acid tra	
EX037410	2.482	moderately similar to (314)AT1G17030| Symbols: | similar to unknown protein [Arabidopsis thaliana] (TAIR:AT2G47010.2); similar to u	
EV110385	2.481	no similarity	
JCVI_22708	2.481	very weakly similar to (89.7)AT4G27880| Symbols: | seven in absentia (SINA) family protein | chr4:13883629-13884933 FORWARD no c	-2.967
JCVI_34475	2.481	no original description	
EV178576	2.481	very weakly similar to (81.3)AT3G54840| Symbols: AtRABF1, Ara-6, AtRab5C, ARA6 | ARA6 | chr3:20329575-20331715 FORWARD [2	
EE437788	2.480	moderately similar to (231)AT3G11890| Symbols: | similar to unknown protein [Arabidopsis thaliana] (TAIR:AT3G11860.1); similar to u	-3.275
JCVI_11418	2.480	moderately similar to (307)AT3G26710| Symbols: | similar to unnamed protein product [Vitis vinifera] (GB:CAO49107.1) | chr3:9814787	
CN727180	2.480	weakly similar to (150)AT1G05850| Symbols: ERH2, ELP1, CTL1, ELP, HOT2, POM1 | POM1 (POM-POM1); chitinase | chr1:1766832-	
JCVI_1852	2.479	moderately similar to (384)AT5G47200| Symbols: AtRABD2b, AtRab1A | AtRABD2b/AtRab1A (Arabidopsis Rab GTPase homolog D2b)	
EE480071	2.479	weakly similar to (177)AT2G46890| Symbols: | oxidoreductase, acting on the CH-CH group of donors | chr2:19273948-19275203 REVER	
JCVI_5324	2.479	moderately similar to (280)AT1G11650| Symbols: ATRBP45B | ATRBP45B; RNA binding | chr1:3914895-3917301 FORWARD no origi	
CD828560	2.479	moderately similar to (209)AT3G10800| Symbols: BZIP28 | BZIP28; DNA binding / transcription factor | chr3:3379331-3381435 FORWA	
EX107867	2.479	weakly similar to (158)AT2G28060| Symbols: | protein kinase-related | chr2:11957251-11957695 REVERSE [21827]	
EE468972	2.479	very weakly similar to (88.2)AT4G12040| Symbols: | zinc finger (AN1-like) family protein | chr4:7215335-7215862 FORWARD [20156]	
EV152780	2.479	moderately similar to (382)AT3G05960| Symbols: | sugar transporter, putative | chr3:1783593-1785340 REVERSEmoderately similar to (	
JCVI_42273	2.479	moderately similar to (322)AT5G22620| Symbols: | phosphoglycerate/bisphosphoglycerate mutase family protein | chr5:7517734-7520226	
JCVI_5172	2.479	weakly similar to (123)AT3G03490| Symbols: PEX19-1 | PEX19-1 (PEROXIN 19-1) | chr3:830309-831766 REVERSE no original descrip	1.568
JCVI_4829	2.479	highly similar to (852)AT2G20340| Symbols: | tyrosine decarboxylase, putative | chr2:8786885-8789571 FORWARDhighly similar to (71	
EX137858	2.479	moderately similar to (276)AT2G30490| Symbols: ATC4H, C4H, CYP73A5 | ATC4H/C4H/CYP73A5 (CINNAMATE 4-HYDROXYLAS	
JCVI_2302	2.478	moderately similar to (336)AT3G45600| Symbols: TET3 | TET3 (TETRASPANIN3) | chr3:16744958-16746839 REVERSE no original de	
JCVI_1590	2.478	weakly similar to (111)AT1G22300| Symbols: GF14 EPSILON, GRF10 | GRF10 (GENERAL REGULATORY FACTOR 10); protein phos	
EX023634	2.478	weakly similar to (134)AT4G33300| Symbols: ADR1-L1 | ADR1-L1 (ADR1-LIKE 1); ATP binding / protein binding | chr4:16051166-160	
JCVI_19903	2.478	moderately similar to (217)AT1G08060| Symbols: MOM1, MOM | MOM (MAINTENANCE OF METHYLATION) | chr1:2501978-2510	
EE556752	2.478	weakly similar to (103)AT5G06150| Symbols: CYCB1;2, CYC1BAT | CYC1BAT (CYCLIN B 1;2); cyclin-dependent protein kinase regul	
CV544524	2.478	very weakly similar to (82.8)AT2G33580| Symbols: | protein kinase family protein / peptidoglycan-binding LysM domain-containing prote	
H07826	2.478	no similarity	
JCVI_17931	2.478	moderately similar to (296)AT1G31260| Symbols: ZIP10 | ZIP10 (ZINC TRANSPORTER 10 PRECURSOR); cation transmembrane trans	
JCVI_14162	2.477	moderately similar to (266)AT1G20140| Symbols: ASK4 | ASK4 (ARABIDOPSIS SKP1-LIKE 4); ubiquitin-protein ligase | chr1:6986421-	
EV044211	2.477	moderately similar to (285)AT1G67930| Symbols: | Golgi transport complex protein-related | chr1:25477881-25480995 REVERSE [2144	
JCVI_7918	2.476	moderately similar to (405)AT1G77090| Symbols: | thylakoid lumenal 29.8 kDa protein | chr1:28965470-28966769 REVERSE no original	
JCVI_12620	2.476	moderately similar to (345)AT3G28730| Symbols: SSRP1, NFD, ATHMG | ATHMG (HIGH MOBILITY GROUP); transcription factor | c	

JCVI_36231	2.476	highly similar to (759)AT2G41770| Symbols: | similar to unknown protein [Arabidopsis thaliana] (TAIR:AT3G57420.1); similar to unnam	
JCVI_955	2.476	weakly similar to (191)AT3G24160| Symbols: PMP | PMP (PUTATIVE TYPE 1 MEMBRANE PROTEIN) | chr3:8726248-8729030 FOR	
EE475863	2.475	moderately similar to (249)AT5G49460| Symbols: ACLB-2 | ACLB-2 (ATP-citrate lyase B-2) | chr5:20072274-20075421 FORWARD [20	
EX123246	2.474	moderately similar to (360)AT5G47330| Symbols: | palmitoyl protein thioesterase family protein | chr5:19224397-19225938 FORWARD	-1.782
ES926505	2.474	moderately similar to (239)AT3G53560| Symbols: | chloroplast lumen common family protein | chr3:19870932-19871954 REVERSE [15	2.095
EE460831	2.474	weakly similar to (157)AT1G06060| Symbols: | RanBPM-related | chr1:1833070-1834515 FORWARD [20152]	
JCVI_7203	2.474	moderately similar to (333)AT3G01790| Symbols: | ribosomal protein L13 family protein | chr3:283887-285590 REVERSEvery weakly si	
JCVI_33955	2.474	highly similar to (704)AT5G49030| Symbols: OVA2 | OVA2 (OVULE ABORTION 2); ATP binding / aminoacyl-tRNA ligase | chr5:1989	
JCVI_36728	2.473	moderately similar to (267)AT2G40230| Symbols: | transferase family protein | chr2:16810363-16811664 REVERSE no original descripti	
JCVI_26542	2.473	moderately similar to (278)AT2G13440| Symbols: | glucose-inhibited division family A protein | chr2:5600352-5605378 REVERSE no or	
EE534972	2.472	very weakly similar to (93.6)AT3G05890| Symbols: RCI2B | RCI2B (RARE-COLD-INDUCIBLE 2B) | chr3:1758185-1758458 REVERSE	
EX134666	2.472	weakly similar to (167)AT1G71400| Symbols: | disease resistance family protein / LRR family protein | chr1:26913567-26916110 FORW	
JCVI_41497	2.472	moderately similar to (439)AT4G00740| Symbols: | dehydration-responsive protein-related | chr4:307815-310298 REVERSE no original d	
EX128653	2.472	moderately similar to (347)AT3G53840| Symbols: | protein kinase family protein | chr3:19956549-19958697 FORWARDweakly similar to	
JCVI_27581	2.472	weakly similar to (137)AT5G49410| Symbols: | similar to unknown protein [Arabidopsis thaliana] (TAIR:AT1G73940.1); similar to unnai	
JCVI_19323	2.472	moderately similar to (273)AT2G19130| Symbols: | S-locus lectin protein kinase family protein | chr2:8300871-8303357 FORWARDweak	
JCVI_20940	2.472	highly similar to (599)AT5G03340| Symbols: | (Cell division control protein 48 homolog E); ATPase | chr5:810090-813132 REVERSEhig	
DY023353	2.471	weakly similar to (149)AT4G25320| Symbols: | DNA-binding protein-related | chr4:12954498-12956352 FORWARD [18979]	
JCVI_24943	2.471	highly similar to (771)AT3G13772| Symbols: | endomembrane protein 70, putative | chr3:4521719-4524401 REVERSE no original descrip	
JCVI_24894	2.470	highly similar to (528)AT5G64280| Symbols: DIT2.2 | DIT2.2 (DICARBOXYLATE TRANSPORTER 2.2); oxoglutarate:malate antiporte	
JCVI_6112	2.470	moderately similar to (496)AT3G28730| Symbols: SSRP1, NFD, ATHMG | ATHMG (HIGH MOBILITY GROUP); transcription factor | c	
JCVI_8693	2.470	weakly similar to (174)AT3G02650| Symbols: | pentatricopeptide (PPR) repeat-containing protein | chr3:566278-569872 FORWARD no c	
CD815541	2.470	moderately similar to (399)AT5G61990| Symbols: | pentatricopeptide (PPR) repeat-containing protein | chr5:24917412-24920336 REVER	
EV011443	2.469	no similarity	
JCVI_30936	2.469	moderately similar to (420)AT5G63830| Symbols: | zinc finger (HIT type) family protein | chr5:25560811-25562028 REVERSE no origin	
JCVI_18576	2.469	moderately similar to (411)AT2G25950| Symbols: | similar to unknown protein [Arabidopsis thaliana] (TAIR:AT3G04780.1); similar to u	
CX280902	2.468	weakly similar to (165)AT4G23370| Symbols: | similar to unknown protein [Arabidopsis thaliana] (TAIR:AT4G23360.1); similar to hypot	
EX049029	2.468	moderately similar to (268)AT1G20880| Symbols: | RNA recognition motif (RRM)-containing protein | chr1:7262868-7264823 REVERSI	
EV211405	2.468	moderately similar to (271)AT3G01180| Symbols: ATSS2 | ATSS2 (STARCH SYNTHASE 2); transferase, transferring glycosyl groups | c	
JCVI_28141	2.468	moderately similar to (257)AT1G57610| Symbols: | similar to unknown protein [Arabidopsis thaliana] (TAIR:AT1G09575.1); similar to u	
BQ704755	2.468	no similarity	
EE462169	2.468	no similarity	
EE521174	2.467	moderately similar to (219)AT4G24120| Symbols: YSL1 | YSL1 (YELLOW STRIPE LIKE 1); oligopeptide transporter | chr4:12524591-1	
EX044155	2.467	no similarity	
JCVI_19048	2.466	no original description	
JCVI_1537	2.466	moderately similar to (460)AT4G35600| Symbols: CONNEXIN 32 | CONNEXIN 32; kinase | chr4:16896453-16898719 FORWARDweakl	
ES911157	2.466	moderately similar to (278)AT2G41050| Symbols: | PQ-loop repeat family protein / transmembrane family protein | chr2:17130466-17132	-2.362
EV225362	2.466	no similarity	
JCVI_39970	2.466	no original description	
EE439300	2.465	no similarity	
EV103815	2.465	weakly similar to (182)AT1G02410| Symbols: | cytochrome c oxidase assembly protein CtaG / Cox11 family | chr1:491300-492762 FORV	
JCVI_2341	2.465	moderately similar to (389)AT3G51610| Symbols: | similar to unnamed protein product [Vitis vinifera] (GB:CAO48858.1) | chr3:1915069	
EV030436	2.464	very weakly similar to (80.5)AT1G48350| Symbols: | ribosomal protein L18 family protein | chr1:17870937-17871883 FORWARD [21441	
JCVI_38835	2.464	moderately similar to (327)AT3G10630| Symbols: | glycosyl transferase family 1 protein | chr3:3321727-3323190 REVERSE no original c	
JCVI_7585	2.463	moderately similar to (320)AT4G00905| Symbols: | similar to NC domain-containing protein-related [Arabidopsis thaliana] (TAIR:AT1G	
JCVI_19743	2.463	moderately similar to (340)AT5G19855| Symbols: | similar to unnamed protein product [Vitis vinifera] (GB:CAO21907.1) | chr5:6712168	
JCVI_18443	2.463	moderately similar to (382)AT2G38400| Symbols: AGT3 | AGT3 (ALANINE:GLYOXYLATE AMINOTRANSFERASE 3); alanine-glyox	
JCVI_33925	2.462	moderately similar to (453)AT1G14360| Symbols: ATUTR3, UTR3 | ATUTR3/UTR3 (UDP-GALACTOSE TRANSPORTER 3); pyrimid	
EE479063	2.461	moderately similar to (256)AT5G10450| Symbols: AFT1, GRF6 | GRF6 (G-BOX REGULATING FACTOR 6); protein phosphorylated am	
AM395240	2.461	weakly similar to (180)AT4G29360| Symbols: | glycosyl hydrolase family 17 protein | chr4:14451568-14453693 REVERSE [20346]	
JCVI_17060	2.461	moderately similar to (276)AT1G60080| Symbols: | 3' exoribonuclease family domain 1-containing protein | chr1:22156270-22158004 RE	
JCVI_15842	2.461	weakly similar to (139)AT5G17990| Symbols: PAT1, TRP1 | TRP1 (TRYPTOPHAN BIOSYNTHESIS 1); anthranilate phosphoribosyltra	
JCVI_8429	2.461	highly similar to (578)AT5G49360| Symbols: ATBXL1, BXL1 | BXL1 (BETA-XYLOSIDASE 1); hydrolase, hydrolyzing O-glycosyl comp	
JCVI_42010	2.461	moderately similar to (372)AT1G22020| Symbols: SHM6 | SHM6 (serine hydroxymethyltransferase 6); glycine hydroxymethyltransferase	-2.197
JCVI_22045	2.461	moderately similar to (289)AT5G35630| Symbols: GLN2, ATGSL1, GS2 | GS2 (GLUTAMINE SYNTHETASE 2) | chr5:13848450-13850	-3.620
CN737738	2.460	weakly similar to (136)AT3G32930| Symbols: | similar to unnamed protein product [Vitis vinifera] (GB:CAO22035.1) | chr3:13493308-1	
JCVI_8162	2.460	moderately similar to (369)AT5G57490| Symbols: | porin, putative | chr5:23301121-23302561 REVERSEweakly similar to (199)VDAC2	
EE473736	2.460	no similarity	
JCVI_15922	2.460	moderately similar to (311)AT5G12040| Symbols: | carbon-nitrogen hydrolase family protein | chr5:3885163-3887773 FORWARD no orig	
JCVI_21845	2.460	moderately similar to (241)AT3G52160| Symbols: | beta-ketoacyl-CoA synthase family protein | chr3:19356047-19357569 REVERSE no	-1.866
JCVI_5977	2.459	moderately similar to (384)AT1G32050| Symbols: | secretory carrier membrane protein (SCAMP) family protein | chr1:11528596-115309	
JCVI_1203	2.459	moderately similar to (377)AT5G65430| Symbols: GF14 KAPPA, GRF8 | GRF8 (GENERAL REGULATORY FACTOR 8); protein phosp	
JCVI_5592	2.458	moderately similar to (397)AT1G23890| Symbols: | NHL repeat-containing protein | chr1:8439310-8440792 REVERSE no original descri	2.167
ES945317	2.458	no similarity	
RC_H07812	2.458	no similarity	
JCVI_9108	2.457	nearly identical (1061)AT1G48410| Symbols: AGO1 | AGO1 (ARGONAUTE 1) | chr1:17889953-17895560 REVERSE no original descrip	-1.982
ES913234	2.457	no similarity	
JCVI_24513	2.456	moderately similar to (241)AT2G45980| Symbols: | similar to unknown protein [Arabidopsis thaliana] (TAIR:AT4G00355.2); similar to u	
EV209608	2.456	moderately similar to (334)AT5G11040| Symbols: | similar to hypothetical protein OsI_015984 [Oryza sativa (indica cultivar-group)] (GB	
JCVI_4711	2.456	moderately similar to (424)AT1G71190| Symbols: SAG18 | SAG18 (SENESCENCE ASSOCIATED GENE 18) | chr1:26837259-2683864	-3.880
EX115134	2.456	moderately similar to (276)AT5G17990| Symbols: PAT1, TRP1 | TRP1 (TRYPTOPHAN BIOSYNTHESIS 1); anthranilate phosphoribosy	
EV207957	2.456	weakly similar to (141)AT5G51020| Symbols: | similar to hypothetical protein [Vitis vinifera] (GB:CAN83158.1); contains InterPro doma	
JCVI_1969	2.456	moderately similar to (329)AT4G21990| Symbols: PRH-26, PRH26, ATAPR3, APR3 | APR3 (APS REDUCTASE 3) | chr4:11657296-116	
JCVI_2883	2.455	moderately similar to (399)AT3G13490| Symbols: OVA5 | OVA5 (OVULE ABORTION 5); ATP binding / aminoacyl-tRNA ligase | chr3:	
CV433909	2.455	no similarity	
EX138631	2.455	no similarity	
DY013767	2.454	weakly similar to (174)AT2G17420| Symbols: ATNTRA, NTR2, NTRA | NTRA (NADPH-dependent thioredoxin reductase 2) | chr2:7571	
JCVI_25528	2.454	moderately similar to (422)AT3G57260| Symbols: PR2, BG2, PR-2, BGL2 | BGL2 (PATHOGENESIS-RELATED PROTEIN 2); glucan 1	
RC_EV011983	2.454	no similarity	
JCVI_34432	2.454	moderately similar to (401)AT3G07650| Symbols: COL9 | COL9 (CONSTANS-LIKE 9) | chr3:2442500-2443907 FORWARD no original	
JCVI_10672	2.454	weakly similar to (147)AT3G21055| Symbols: PSBTN | PSBTN (photosystem II subunit T) | chr3:7376767-7377078 REVERSE no origina	

JCVI_6131	2.454	very weakly similar to (90.1)AT2G43750| Symbols: ACS1, CPACS1, ATCS-B, OASB | OASB (O-ACETYLSERINE (THIOL) LYASE B);	
ES938820	2.453	moderately similar to (228)AT5G14420| Symbols: RGLG2 | RGLG2 (RING DOMAIN LIGASE2) | chr5:4648358-4650566 REVERSE [21	
JCVI_21948	2.453	highly similar to (658)AT4G03560| Symbols: TPC1, ATCCH1, FOU2, ATTPC1 | ATTPC1 (TWO-PORE CHANNEL 1); calcium channel	
JCVI_5987	2.453	moderately similar to (399)AT2G35860| Symbols: FLA16 | FLA16 (FASCICLIN-LIKE ARABINOGALACTAN PROTEIN 16 PRECURS	
JCVI_35714	2.453	highly similar to (548)AT2G36290| Symbols: | hydrolase, alpha/beta fold family protein | chr2:15215946-15217847 REVERSE no origina	
ES913262	2.453	moderately similar to (216)AT4G21910| Symbols: | MATE efflux family protein | chr4:11625833-11630976 REVERSE [21431] 1 577 59	
EE402587	2.452	weakly similar to (150)AT5G40720| Symbols: | similar to zinc finger (C3HC4-type RING finger) family protein [Arabidopsis thaliana] (T	
JCVI_38632	2.452	moderately similar to (457)AT5G44110| Symbols: ATPOP1, ATNAP2, POP1 | POP1 | chr5:17771619-17772899 REVERSE no original de	
CD821580	2.452	moderately similar to (226)AT4G17650| Symbols: | aromatic-rich family protein | chr4:9827762-9829456 FORWARD [13979]	
EV086417	2.451	no similarity	
JCVI_23220	2.451	moderately similar to (207)AT3G21690| Symbols: | MATE efflux family protein | chr3:7638757-7641868 FORWARD no original descrip	
CN728640	2.451	moderately similar to (452)AT2G35690| Symbols: ACX5 | ACX5 (ACYL-COA OXIDASE 5); acyl-CoA oxidase | chr2:15007041-1500991	-1.925
EV192836	2.451	weakly similar to (124)AT5G66190| Symbols: ATLFNR1 | ATLFNR1 (LEAF FNR 1); poly(U) binding | chr5:26468429-26469842 REVEI	
EE449470	2.450	no similarity	
JCVI_18223	2.450	moderately similar to (297)AT3G12780| Symbols: PGK1 | PGK1 (PHOSPHOGLYCERATE KINASE 1); phosphoglycerate kinase | chr3:4	2.880
JCVI_23333	2.450	moderately similar to (454)AT1G19920| Symbols: ASA1, APS2 | APS2 (ATP SULFURYLASE PRECURSOR) | chr1:6914826-6916648 R	
JCVI_5853	2.450	highly similar to (518)AT5G53370| Symbols: ATPMEPCRF | ATPMEPCRF; pectinesterase | chr5:21666909-21668756 REVERSEweakly	
EE566299	2.450	weakly similar to (115)AT3G27110| Symbols: | peptidase M48 family protein | chr3:9999243-10001129 FORWARD [20153] 61 689 709	
EE522768	2.449	moderately similar to (206)AT3G08740| Symbols: | elongation factor P (EF-P) family protein | chr3:2654794-2656160 REVERSE [20143	
EV011998	2.449	no similarity	
JCVI_34159	2.449	moderately similar to (238)AT3G17540| Symbols: | F-box family protein | chr3:6002789-6003979 FORWARD no original description	
DY026369	2.449	weakly similar to (174)AT4G29960| Symbols: | similar to unnamed protein product [Vitis vinifera] (GB:CAO15556.1) | chr4:14660759-14	
EE540455	2.449	very weakly similar to (87.4)AT1G48240| Symbols: NPSN12, ATNPSN12 | ATNPSN12 (novel plant SNARE 12); protein transporter | chr1	
EV024762	2.448	moderately similar to (279)AT3G02140| Symbols: TMAC2 | TMAC2 (TWO OR MORE ABRES-CONTAINING GENE 2) | chr3:385485-	
JCVI_37809	2.448	weakly similar to (109)AT2G28900| Symbols: ATOEP16-L, ATOEP16-1, OEP16 | OEP16 (OUTER ENVELOPE PROTEIN 16); P-P-bon	
JCVI_5865	2.448	moderately similar to (396)AT5G17760| Symbols: | AAA-type ATPase family protein | chr5:5861278-5862303 REVERSE no original des	-3.332
JCVI_11679	2.447	moderately similar to (352)AT1G73480| Symbols: | hydrolase, alpha/beta fold family protein | chr1:27632927-27636147 FORWARD no c	
JCVI_20499	2.447	weakly similar to (104)AT4G26080| Symbols: ABI1 | ABI1 (ABA INSENSITIVE 1); calcium ion binding / protein serine/threonine phosp	
JCVI_18526	2.447	moderately similar to (226)AT1G79530| Symbols: GAPCP-1 | GAPCP-1; glyceraldehyde-3-phosphate dehydrogenase | chr1:29921125-299	
JCVI_16319	2.446	weakly similar to (163)AT4G15160| Symbols: | protease inhibitor/seed storage/lipid transfer protein (LTP) family protein | chr4:8646193-	-3.156
JCVI_24140	2.446	very weakly similar to (97.4)AT5G48760| Symbols: | 60S ribosomal protein L13A (RPL13aD) | chr5:19788541-19789912 REVERSEvery	
CB686335	2.446	weakly similar to (149)AT2G33380| Symbols: RD20 | RD20 (RESPONSIVE TO DESSICATION 20); calcium ion binding | chr2:1415206	
JCVI_13009	2.446	moderately similar to (254)AT4G35260| Symbols: IDH1 | IDH1 (ISOCITRATE DEHYDROGENASE 1); isocitrate dehydrogenase (NAD-	
JCVI_18416	2.446	highly similar to (506)AT3G54050| Symbols: | fructose-1,6-bisphosphatase, putative / D-fructose-1,6-bisphosphate 1-phosphohydrolase, p	
JCVI_3773	2.446	moderately similar to (257)AT5G64040| Symbols: PSAN | PSAN (photosystem I reaction center subunit PSI-N); calmodulin binding | chr5	
RC_EX063904	2.446	no similarity	
JCVI_15261	2.446	weakly similar to (158)AT5G14540| Symbols: | proline-rich family protein | chr5:4687336-4689627 REVERSE no original description	-2.149
DY030350	2.445	no similarity	
EV084218	2.445	moderately similar to (255)AT3G13235| Symbols: | ubiquitin family protein | chr3:4271499-4274355 REVERSE [21444]	
EV079087	2.445	weakly similar to (197)AT5G10550| Symbols: GTE2 | GTE2 (GLOBAL TRANSCRIPTION FACTOR GROUP E 2); DNA binding | chr5:	
DY030106	2.445	weakly similar to (194)AT3G50410| Symbols: OBP1 | OBP1 (OBF BINDING PROTEIN 1); DNA binding / transcription factor | chr3:187	
EE419570	2.444	weakly similar to (108)AT1G55365| Symbols: | similar to unknown protein [Arabidopsis thaliana] (TAIR:AT5G56520.1) | chr1:20677649	
JCVI_26054	2.444	moderately similar to (340)AT1G55000| Symbols: | peptidoglycan-binding LysM domain-containing protein | chr1:20518429-20519833 F	
JCVI_26398	2.444	moderately similar to (497)AT5G50210| Symbols: QS | QS (QUINOLINATE SYNTHASE); quinolinate synthetase A | chr5:20460029-204	
JCVI_1152	2.443	moderately similar to (220)AT2G23790| Symbols: | similar to unknown protein [Arabidopsis thaliana] (TAIR:AT4G36820.1); similar to u	
JCVI_34964	2.443	moderately similar to (384)AT1G71900| Symbols: | similar to permease-related [Arabidopsis thaliana] (TAIR:AT1G34470.1); similar to h	
EX040831	2.443	moderately similar to (405)AT2G16430| Symbols: PAP10, ATPAP10 | ATPAP10/PAP10; acid phosphatase/ protein serine/threonine phos	
JCVI_5022	2.443	moderately similar to (381)AT3G49720| Symbols: | Identical to Uncharacterized protein At3g49720 [Arabidopsis Thaliana] (GB:Q9M2Y(	
AT000451	2.443	no similarity	
JCVI_15537	2.443	moderately similar to (321)AT3G27770| Symbols: | similar to unknown protein [Arabidopsis thaliana] (TAIR:AT5G62960.1); similar to u	
JCVI_28407	2.443	moderately similar to (429)AT3G07050| Symbols: | GTP-binding family protein | chr3:2229608-2232285 REVERSE no original descriptio	-2.826
JCVI_39974	2.443	weakly similar to (200)AT5G38060| Symbols: | similar to unknown protein [Arabidopsis thaliana] (TAIR:AT1G65000.1); similar to unnar	
JCVI_15613	2.442	no original description	
JCVI_7524	2.442	moderately similar to (356)AT5G20520| Symbols: WAV2 | WAV2 (WAVY GROWTH 2) | chr5:6943538-6946317 REVERSE no original	
JCVI_19749	2.442	highly similar to (785)AT1G62430| Symbols: ATCDS1 | ATCDS1 (CDP-diacylglycerol synthase 1); phosphatidate cytidylyltransferase | ch	-2.173
EE439251	2.442	weakly similar to (149)AT1G74050| Symbols: | 60S ribosomal protein L6 (RPL6C) | chr1:27850917-27852341 REVERSEweakly similar t	
EX040551	2.441	moderately similar to (258)AT3G16180| Symbols: | proton-dependent oligopeptide transport (POT) family protein | chr3:5481483-548494	
JCVI_20073	2.441	moderately similar to (218)AT2G16770| Symbols: | DNA binding / transcription factor | chr2:7286941-7287899 FORWARD no original d	
EV035282	2.441	no similarity	
EE517306	2.441	weakly similar to (107)AT1G05270| Symbols: | TraB family protein | chr1:1531805-1534304 REVERSE [20185]	-1.616
EV213100	2.441	no similarity	
JCVI_16114	2.440	very weakly similar to (99.4)AT5G11930| Symbols: | glutaredoxin family protein | chr5:3845166-3845612 REVERSE no original descriptio	
JCVI_6239	2.440	moderately similar to (225)AT3G04090| Symbols: SIP1;1, SIP1A | SIP1;1 (SMALL AND BASIC INTRINSIC PROTEIN 1A) | chr3:10723	
EV206727	2.440	no similarity	
EX095985	2.440	moderately similar to (239)AT1G10580| Symbols: | transducin family protein / WD-40 repeat family protein | chr1:3491561-3493666 REV	
JCVI_2720	2.439	moderately similar to (221)AT4G34720| Symbols: VHA-C1, ATVHA-C1, AVA-P1 | AVA-P1 (vacuolar H+-pumping ATPase 16 kDa prot	
JCVI_32615	2.439	weakly similar to (155)AT1G50250| Symbols: FTSH1 | FTSH1 (FtsH protease 1); ATP-dependent peptidase/ ATPase/ metallopeptidase | c	
EX093127	2.439	moderately similar to (346)AT1G31500| Symbols: | endonuclease/exonuclease/phosphatase family protein | chr1:11273802-11276515 RE	-2.839
JCVI_10443	2.439	highly similar to (597)AT5G57020| Symbols: ATNMT1, NMT1 | NMT1 (N-MYRISTOYLTRANSFERASE 1) | chr5:23092677-23093981	
JCVI_20243	2.438	highly similar to (600)AT1G26390| Symbols: | FAD-binding domain-containing protein | chr1:9130151-9131743 REVERSE no original de	
JCVI_16547	2.438	highly similar to (590)AT5G16520| Symbols: | similar to unknown [Populus trichocarpa] (GB:ABK95080.1) | chr5:5394743-5397374 REV	
EV047544	2.438	moderately similar to (336)AT4G18975| Symbols: | pentatricopeptide (PPR) repeat-containing protein | chr4:10392181-10393676 REVEF	1.993
JCVI_21231	2.438	weakly similar to (171)AT5G25752| Symbols: | rhomboid family protein | chr5:8951506-8953326 REVERSE no original description	
EE560871	2.437	no similarity	
EE544718	2.437	moderately similar to (374)AT4G16390| Symbols: | chloroplastic RNA-binding protein P67, putative | chr4:9258050-9260116 FORWARD	
JCVI_17325	2.437	weakly similar to (192)AT2G39270| Symbols: | adenylate kinase family protein | chr2:16407061-16408486 FORWARDweakly similar to (	
JCVI_30480	2.437	no original description	
EV204694	2.437	no similarity	
JCVI_36474	2.436	moderately similar to (413)AT5G58950| Symbols: | protein kinase family protein | chr5:23818362-23820251 REVERSE no original descri	
RC_CK991405	2.436	no similarity	
JCVI_25365	2.436	moderately similar to (431)AT4G13810| Symbols: | disease resistance family protein / LRR family protein | chr4:8005058-8007283 REVE	

JCVI_1001	2.436	moderately similar to (320)AT1G78170| Symbols: | similar to unknown protein [Arabidopsis thaliana] (TAIR:AT1G22250.1); similar to u	
JCVI_15006	2.435	moderately similar to (290)AT4G00220| Symbols: LBD30, JLO | JLO/LBD30 (JAGGED LATERAL ORGANS) | chr4:90147-92087 FORW	
JCVI_2770	2.435	moderately similar to (384)AT5G46110| Symbols: TPT, APE2 | APE2 (ACCLIMATION OF PHOTOSYNTHESIS TO ENVIRONMENT)	
JCVI_11390	2.435	weakly similar to (117)AT2G14900| Symbols: | gibberellin-regulated family protein | chr2:6411292-6412125 FORWARD no original desc	
JCVI_16589	2.435	no original description	
JCVI_2647	2.435	moderately similar to (315)AT3G02230| Symbols: ATRGP1, ATRGP, RGP1 | RGP1 (REVERSIBLY GLYCOSYLATED POLYPEPTIDE	
EV218755	2.435	moderately similar to (321)AT1G79410| Symbols: ATOCT5 | ATOCT5 (ARABIDOPSIS THALIANA ORGANIC CATION/CARNITINE	
JCVI_8221	2.435	highly similar to (563)AT5G61840| Symbols: GUT1 | GUT1; catalytic | chr5:24856591-24858929 REVERSE no original description	
JCVI_6434	2.434	weakly similar to (187)AT2G20825| Symbols: ULT2 | ULT2 (ULTRAPETALA 2); DNA binding | chr2:8972926-8973876 REVERSE no c	
EX132980	2.434	weakly similar to (167)AT4G36640| Symbols: | SEC14 cytosolic factor family protein / phosphoglyceride transfer family protein | chr4:17	
JCVI_28234	2.434	moderately similar to (387)AT1G77290| Symbols: | tetrachloro-p-hydroquinone reductive dehalogenase-related | chr1:29043811-2904470	
JCVI_15215	2.434	moderately similar to (473)AT5G64410| Symbols: ATOPT4 | ATOPT4 (oligopeptide transporter 4); oligopeptide transporter | chr5:257681	
JCVI_20049	2.434	moderately similar to (249)AT5G13080| Symbols: ATWRKY75, WRKY75 | WRKY75 (WRKY DNA-BINDING PROTEIN 75); transcrip	
JCVI_15020	2.433	moderately similar to (318)AT3G03860| Symbols: ATAPRL5 | ATAPRL5 (APR-LIKE 5) | chr3:992472-994322 FORWARD no original d	
JCVI_28501	2.433	moderately similar to (298)AT1G50480| Symbols: THFS | THFS (10-FORMYLTETRAHYDROFOLATE SYNTHETASE); ATP binding	
JCVI_1912	2.433	moderately similar to (250)AT1G72290| Symbols: | trypsin and protease inhibitor family protein / Kunitz family protein | chr1:27219514-2	
EE518590	2.433	moderately similar to (217)AT4G22140| Symbols: | DNA binding | chr4:11728105-11730242 REVERSE [20185]	
AT000465	2.432	weakly similar to (132)AT1G30400| Symbols: EST1, ATMRP1 | ATMRP1 (Arabidopsis thaliana multidrug resistance-associated protein 1	
JCVI_2919	2.432	highly similar to (573)AT4G04640| Symbols: ATPC1 | ATPC1 (ATP synthase gamma chain 1) | chr4:2350759-2351880 REVERSEhighly	
JCVI_14121	2.432	moderately similar to (363)AT1G31830| Symbols: | amino acid permease family protein | chr1:11418856-11420295 REVERSE no origina	
EE530360	2.432	moderately similar to (228)AT3G28720| Symbols: | similar to unknown protein [Arabidopsis thaliana] (TAIR:AT5G58100.1); similar to h	
JCVI_26242	2.431	moderately similar to (385)AT3G18310| Symbols: | similar to hypothetical protein [Vitis vinifera] (GB:CAN64638.1) | chr3:6284529-628	
JCVI_20189	2.431	moderately similar to (279)AT3G55120| Symbols: TT5, A11, CFI | A11/CFI/TT5 (TRANSPARENT TESTA 5); chalcone isomerase | chr3	
JCVI_14110	2.430	moderately similar to (470)AT2G05120| Symbols: | transporter | chr2:1842066-1846855 REVERSE no original description	
EX113975	2.430	moderately similar to (266)AT3G57240| Symbols: BG3 | BG3 (BETA-1,3-GLUCANASE 3); hydrolase, hydrolyzing O-glycosyl compounc	
JCVI_2435	2.430	highly similar to (535)AT3G05530| Symbols: ATS6A.2, RPT5A | RPT5A (regulatory particle triple-A 5A); ATPase/ calmodulin binding |	
JCVI_6721	2.430	moderately similar to (269)AT1G51710| Symbols: UBP6 | UBP6 (UBIQUITIN-SPECIFIC PROTEASE 6) | chr1:19179473-19183067 RE	-4.349
ES911333	2.429	moderately similar to (223)AT4G19670| Symbols: | zinc finger (C3HC4-type RING finger) family protein | chr4:10699393-10701352 RE	
EX127485	2.429	moderately similar to (362)AT4G33080| Symbols: | protein kinase, putative | chr4:15960149-15964299 FORWARDweakly similar to (10	
JCVI_9582	2.429	moderately similar to (305)AT3G09180| Symbols: | similar to unnamed protein product [Vitis vinifera] (GB:CAO45433.1); contains doma	
EV058990	2.429	moderately similar to (322)AT5G44070| Symbols: ARA8, ATPCS1, PCS1, CAD1 | CAD1 (CADMIUM SENSITIVE 1) | chr5:17752103-1	
JCVI_595	2.428	moderately similar to (294)AT1G76090| Symbols: SMT3 | SMT3 (S-adenosyl-methionine-sterol-C-methyltransferase 3); S-adenosylmethi	-3.850
JCVI_39446	2.428	highly similar to (605)AT1G73370| Symbols: SUS6 | SUS6; UDP-glycosyltransferase/ sucrose synthase | chr1:27588194-27591987 REVE	
EE436665	2.428	moderately similar to (209)AT5G11310| Symbols: | pentatricopeptide (PPR) repeat-containing protein | chr5:3606491-3608410 FORWAR	
EV047540	2.428	moderately similar to (280)AT2G03450| Symbols: PAP9, ATPAP9 | ATPAP9/PAP9 (purple acid phosphatase 9); acid phosphatase/ protei	
EE439918	2.428	weakly similar to (176)AT5G09270| Symbols: | similar to hypothetical protein OsI_004420 [Oryza sativa (indica cultivar-group)] (GB:EA	
JCVI_28176	2.427	weakly similar to (115)AT2G26150| Symbols: HSFA2, ATHSFA2 | ATHSFA2 (Arabidopsis thaliana heat shock transcription factor A2) |	
JCVI_30137	2.427	moderately similar to (260)AT5G08580| Symbols: | calcium-binding EF hand family protein | chr5:2780977-2782962 REVERSE no origir	
JCVI_13041	2.427	weakly similar to (132)AT5G61910| Symbols: | similar to unknown protein [Arabidopsis thaliana] (TAIR:AT2G32910.1); similar to unnai	
JCVI_33515	2.427	moderately similar to (354)AT5G13280| Symbols: AK, AK1, AK-LYS1 | AK-LYS1 (ASPARTATE KINASE 1) | chr5:4249519-4252657 F	
JCVI_33706	2.427	very weakly similar to (82.4)AT2G29020| Symbols: | Rab5-interacting family protein | chr2:12476946-12478505 FORWARD no original c	-1.824
JCVI_32907	2.427	moderately similar to (372)AT5G64000| Symbols: ATSAL2, SAL2 | SAL2; 3'(2'),5'-bisphosphate nucleotidase/ inositol or phosphatidylino	
JCVI_13330	2.427	highly similar to (693)AT1G69790| Symbols: | protein kinase, putative | chr1:26270501-26272481 FORWARDmoderately similar to (215	
ES940745	2.427	moderately similar to (215)AT3G07210| Symbols: | catalytic | chr3:2293006-2295125 REVERSE [21391]	
EV164965	2.427	moderately similar to (413)AT5G13320| Symbols: GDG1, WIN3, PBS3 | PBS3 (AVRPPHB SUSCEPTIBLE 3) | chr5:4268905-4270899 F	
JCVI_21106	2.426	highly similar to (560)AT5G56630| Symbols: | phosphofructokinase family protein | chr5:22941537-22943954 FORWARDvery weakly sir	
JCVI_20280	2.426	very weakly similar to (89.7)AT1G55310| Symbols: ATSCL33, SCL33, SR33 | SR33 (SC35-like splicing factor 33); RNA binding | chr1:20	
JCVI_6321	2.426	weakly similar to (155)AT5G25290| Symbols: | F-box family protein | chr5:8778595-8779788 FORWARD no original description	
JCVI_10241	2.426	weakly similar to (142)AT5G55125| Symbols: | similar to hypothetical protein OsI_035046 [Oryza sativa (indica cultivar-group)] (GB:EA	
JCVI_1994	2.426	moderately similar to (276)AT3G22320| Symbols: ATRPABC24.3 | ATRPABC24.3 (ARABIDOPSIS THALIANA RNA POLYMERASE	
JCVI_34320	2.426	moderately similar to (224)AT1G53900| Symbols: | GTP binding / translation initiation factor | chr1:20131302-20134502 FORWARD no	
ES907000	2.425	weakly similar to (127)AT5G19990| Symbols: ATSUG1, RPT6A | RPT6A; ATPase | chr5:6752146-6754920 FORWARD [21429]	
JCVI_2252	2.425	highly similar to (536)AT1G09640| Symbols: | elongation factor 1B-gamma, putative / eEF-1B gamma, putative | chr1:3120164-3122154	
JCVI_27025	2.424	moderately similar to (357)AT4G24630| Symbols: | receptor/ zinc ion binding | chr4:12714929-12717121 FORWARD no original descript	
JCVI_25735	2.423	highly similar to (853)AT1G32640| Symbols: RD22BP1, JAI1, JIN1, MYC2, ZBF1, ATMYC2 | ATMYC2 (JASMONATE INSENSITIVE	-1.666
JCVI_21244	2.423	weakly similar to (173)AT3G51370| Symbols: | protein phosphatase 2C, putative / PP2C, putative | chr3:19081366-19082954 FORWARD	
JCVI_40841	2.423	moderately similar to (247)AT1G04830| Symbols: | RabGAP/TBC domain-containing protein | chr1:1359086-1361843 REVERSE no orig	
JCVI_11764	2.423	moderately similar to (288)AT3G07565| Symbols: | DNA binding | chr3:2413829-2415878 FORWARD no original description	
JCVI_1160	2.423	weakly similar to (166)AT5G43830| Symbols: | similar to unknown protein [Arabidopsis thaliana] (TAIR:AT3G22850.1); similar to alumi	-2.251
EV017499	2.422	no similarity	
JCVI_18269	2.422	highly similar to (691)AT1G68750| Symbols: ATPPC4 | ATPPC4 (Arabidopsis thaliana phosphoenolpyruvate carboxylase 4); phosphoeno	
CD828756	2.422	no similarity	
CD814504	2.421	weakly similar to (145)AT1G64840| Symbols: | F-box family protein | chr1:24097673-24098827 FORWARD [13977]	
EE550992	2.421	weakly similar to (168)AT5G17600| Symbols: | zinc finger (C3HC4-type RING finger) family protein | chr5:5800031-5801119 REVERSE	
JCVI_32761	2.421	moderately similar to (231)AT3G07550| Symbols: | F-box family protein (FBL12) | chr3:2409952-2411139 FORWARD no original descri	
JCVI_8194	2.421	moderately similar to (424)AT2G20420| Symbols: | succinyl-CoA ligase (GDP-forming) beta-chain, mitochondrial, putative / succinyl-Co	
JCVI_38868	2.420	moderately similar to (343)AT2G38760| Symbols: ANNAT3 | ANNAT3 (ANNEXIN ARABIDOPSIS 3); calcium ion binding / calcium-de	
JCVI_25097	2.420	moderately similar to (223)AT4G29190| Symbols: | zinc finger (CCCH-type) family protein | chr4:14392239-14393309 REVERSE no orig	
JCVI_18699	2.420	moderately similar to (257)AT3G04070| Symbols: ANAC047 | ANAC047 (Arabidopsis NAC domain containing protein 47) | chr3:106158	-2.519
CV432548	2.420	moderately similar to (244)AT5G20170| Symbols: | similar to hypothetical protein [Vitis vinifera] (GB:CAN63500.1) | chr5:6807556-681	
EV214637	2.419	weakly similar to (149)AT1G74470| Symbols: | geranylgeranyl reductase | chr1:27994909-27996506 FORWARD [21491]	
JCVI_8068	2.419	weakly similar to (194)AT4G12480| Symbols: pEARLI 1 | pEARLI 1; lipid binding | chr4:7406368-7406874 REVERSEweakly similar to (	
JCVI_14629	2.419	moderately similar to (257)AT1G62262| Symbols: SLAH4 | SLAH4 (SLAC1 HOMOLOGUE 4) | chr1:23003983-23005156 REVERSE no	-1.482
JCVI_33915	2.419	moderately similar to (468)AT3G61150| Symbols: HDG1 | HDG1 (HOMEODOMAIN GLABROUS1); DNA binding / transcription factor	
JCVI_8330	2.419	very weakly similar to (98.6)AT3G51800| Symbols: ATG2 | ATG2 (G2p-related protein); metalloexopeptidase | chr3:19222238-19224545	
EX050590	2.419	moderately similar to (313)AT3G21865| Symbols: PEX22 | PEX22 (PEROXIN 22); protein binding | chr3:7701315-7703225 REVERSE [	
JCVI_11245	2.419	weakly similar to (105)AT4G16500| Symbols: | cysteine protease inhibitor family protein / cystatin family protein | chr4:9301553-930190	
EE526157	2.418	no similarity	
JCVI_6527	2.418	moderately similar to (205)AT3G09035| Symbols: | legume lectin family protein | chr3:2759072-2760088 FORWARD no original descript	
JCVI_10176	2.418	highly similar to (680)AT2G43230| Symbols: | serine/threonine protein kinase, putative | chr2:17973552-17975523 FORWARDweakly sir	
EV155563	2.417	moderately similar to (326)AT4G33410| Symbols: | signal peptide peptidase family protein | chr4:16081643-16083122 FORWARD [2148	

JCVI_38096	2.417	weakly similar to (169)AT5G13630| Symbols: CCH, CHLH, CCH1, GUN5 | GUN5 (GENOMES UNCOUPLED 5) | chr5:4387923-439208	
JCVI_40470	2.417	weakly similar to (115)AT1G58225| Symbols: | unknown protein | chr1:21567870-21568660 FORWARD no original description	
JCVI_2068	2.417	moderately similar to (391)AT5G26280| Symbols: | meprin and TRAF homology domain-containing protein / MATH domain-containing	
EV017477	2.417	weakly similar to (184)AT5G10625| Symbols: | similar to FPF1 (FLOWERING PROMOTING FACTOR 1) [Arabidopsis thaliana] (TAIR	
JCVI_18994	2.417	moderately similar to (394)AT2G43570| Symbols: | chitinase, putative | chr2:18083466-18084512 REVERSEmoderately similar to (243)	
JCVI_13195	2.417	weakly similar to (117)AT2G37435| Symbols: | cysteine protease inhibitor | chr2:15716902-15717607 FORWARD no original description	
JCVI_16665	2.416	moderately similar to (333)AT5G51980| Symbols: | WD-40 repeat family protein / zfwd2 protein (ZFWD2), putative | chr5:21130876-211	
EX091151	2.416	moderately similar to (342)AT5G22740| Symbols: CSLA02, ATCSLA2, ATCSLA02 | ATCSLA02 (Cellulose synthase-like A2); transferas	
JCVI_7015	2.416	moderately similar to (228)AT2G21170| Symbols: TIM | TIM (TRIOSEPHOSPHATE ISOMERASE) | chr2:9078128-9080187 REVERSE	
JCVI_6668	2.415	moderately similar to (319)AT5G41950| Symbols: | binding | chr5:16803053-16806588 FORWARD no original description	
EE521910	2.415	moderately similar to (250)AT5G19000| Symbols: ATBPM1 | ATBPM1 (BTB-POZ AND MATH DOMAIN 1); protein binding | chr5:634	
JCVI_6564	2.415	moderately similar to (361)AT3G13740| Symbols: | URF 4-related | chr3:4504317-4505944 FORWARD no original description	
EV216595	2.415	moderately similar to (322)AT3G56060| Symbols: | glucose-methanol-choline (GMC) oxidoreductase family protein | chr3:20814307-208	
JCVI_13369	2.415	moderately similar to (339)AT5G40230| Symbols: | nodulin-related | chr5:16097042-16098963 REVERSE no original description	2.999
JCVI_26247	2.415	moderately similar to (335)AT5G20200| Symbols: | nucleoporin-related | chr5:6816778-6821622 FORWARD no original description	
JCVI_11429	2.415	moderately similar to (457)AT3G22370| Symbols: ATAOX1A, AOX1A | AOX1A (alternative oxidase 1A); alternative oxidase | chr3:7906	-2.173
JCVI_7736	2.415	moderately similar to (388)AT3G10940| Symbols: | protein phosphatase-related | chr3:3422264-3423399 REVERSE no original descriptio	
EH413892	2.415	moderately similar to (302)AT3G28890| Symbols: | leucine-rich repeat family protein | chr3:10897943-10900078 REVERSEvery weakly s	
JCVI_9438	2.415	moderately similar to (372)AT5G57930| Symbols: EMB1629, APO2 | APO2 (ACCUMULATION OF PHOTOSYSTEM ONE 2) | chr5:23	
JCVI_18377	2.414	no original description	
CV546025	2.414	very weakly similar to (91.3)AT2G43590| Symbols: | chitinase, putative | chr2:18088669-18089826 REVERSEweakly similar to (106)CH	
EH415075	2.414	weakly similar to (113)AT1G01640| Symbols: | speckle-type POZ protein-related | chr1:231164-231915 REVERSE [20767]	
EV224667	2.414	moderately similar to (239)AT5G56030| Symbols: ERD8, HSP81-2 | HSP81-2 (EARLY-RESPONSIVE TO DEHYDRATION 8); ATP bin	
EV024533	2.414	weakly similar to (135)AT3G01350| Symbols: | proton-dependent oligopeptide transport (POT) family protein | chr3:135031-137467 FOR	
ES931762	2.414	moderately similar to (283)AT3G04890| Symbols: | similar to unknown protein [Arabidopsis thaliana] (TAIR:AT2G46100.1); similar to u	
JCVI_25636	2.414	highly similar to (669)AT2G13100| Symbols: | glycerol-3-phosphate transporter, putative / glycerol 3-phosphate permease, putative | chr2	
JCVI_12859	2.413	moderately similar to (395)AT4G36640| Symbols: | SEC14 cytosolic factor family protein / phosphoglyceride transfer family protein | chr	
ES960799	2.413	moderately similar to (232)AT1G22280| Symbols: | protein phosphatase 2C, putative / PP2C, putative | chr1:7874225-7875485 FORWAR	
JCVI_1843	2.413	moderately similar to (288)AT2G28160| Symbols: FIT1, ATBHLH029, FRU, BHLH029 | ATBHLH029/BHLH029/FIT1/FRU (FE-DEFIC	
JCVI_37572	2.413	very weakly similar to (81.3)AT5G64770| Symbols: | similar to 80C09_10 [Brassica rapa subsp. pekinensis] (GB:AAZ41821.1) | chr5:259	2.770
JCVI_13351	2.413	moderately similar to (319)AT1G34370| Symbols: STOP1 | STOP1 (SENSITIVE TO PROTON RHIZOTOXICITY 1); nucleic acid bindir	1.896
JCVI_31657	2.412	moderately similar to (424)AT2G21340| Symbols: | enhanced disease susceptibility protein, putative / salicylic acid induction deficient pro	
CD827796	2.412	weakly similar to (127)AT2G34500| Symbols: CYP710A1 | CYP710A1 (cytochrome P450, family 710, subfamily A, polypeptide 1); C-22	
JCVI_16422	2.412	weakly similar to (166)AT3G58800| Symbols: | similar to unnamed protein product [Vitis vinifera] (GB:CAO70032.1); contains domain a	
JCVI_24409	2.412	weakly similar to (104)AT3G62200| Symbols: | similar to EDA32 (embryo sac development arrest 32) [Arabidopsis thaliana] (TAIR:AT3	2.452
DY020148	2.412	very weakly similar to (87.0)AT1G08500| Symbols: | plastocyanin-like domain-containing protein | chr1:2689113-2689884 FORWARD [1	
JCVI_37788	2.412	moderately similar to (297)AT4G27950| Symbols: CRF4 | CRF4 (CYTOKININ RESPONSE FACTOR 4); DNA binding / transcription fac	
EV109633	2.411	very weakly similar to (94.7)AT2G28500| Symbols: LBD11 | LBD11 (LOB DOMAIN-CONTAINING PROTEIN 11) | chr2:12193680-121	
JCVI_4562	2.411	moderately similar to (454)AT1G30740| Symbols: | FAD-binding domain-containing protein | chr1:10903011-10904612 FORWARD no o	
JCVI_1134	2.411	moderately similar to (357)AT1G04940| Symbols: TIC20 | TIC20; P-P-bond-hydrolysis-driven protein transmembrane transporter | chr1:1	
JCVI_1181	2.411	moderately similar to (363)AT5G43780| Symbols: APS4 | APS4 | chr5:17606858-17608707 REVERSE no original description	
EX044059	2.410	no similarity	
JCVI_8480	2.410	moderately similar to (265)AT3G17100| Symbols: | transcription factor | chr3:5831558-5832250 FORWARD no original description	
EE448866	2.410	weakly similar to (168)AT5G17700| Symbols: | MATE efflux family protein | chr5:5831027-5833417 REVERSE [20172]	
EV101949	2.410	moderately similar to (380)AT2G17320| Symbols: | pantothenate kinase-related | chr2:7540619-7542958 REVERSE [21477] 46 893 893	
JCVI_7619	2.410	highly similar to (567)AT3G57220| Symbols: | UDP-GlcNAc:dolichol phosphate N-acetylglucosamine-1-phosphate transferase, putative |	
JCVI_10615	2.410	no original description	
EV085976	2.410	no similarity	
CX190108	2.410	weakly similar to (132)AT1G75400| Symbols: | protein binding / zinc ion binding | chr1:28301270-28303125 FORWARD [16807]	
JCVI_33455	2.410	no original description	
JCVI_11453	2.410	moderately similar to (382)AT1G56500| Symbols: | haloacid dehalogenase-like hydrolase family protein | chr1:21163440-21170757 FORV	
JCVI_36311	2.409	weakly similar to (112)AT3G06580| Symbols: GALK, GAL1 | GAL1 (GALACTOSE KINASE 1); ATP binding / galactokinase | chr3:2049	
JCVI_40387	2.409	weakly similar to (108)AT5G08380| Symbols: ATAGAL1 | ATAGAL1 (ARABIDOPSIS THALIANA ALPHA-GALACTOSIDASE 1); alp	
JCVI_33959	2.409	weakly similar to (158)AT1G70790| Symbols: | C2 domain-containing protein | chr1:26704386-26705789 FORWARD no original descrip	
JCVI_13149	2.408	highly similar to (634)AT3G54960| Symbols: ATPDIL1-3 | ATPDIL1-3 (PDI-LIKE 1-3) | chr3:20374872-20377799 REVERSEweakly sin	
JCVI_40598	2.407	no original description	
EV199715	2.407	no similarity	
JCVI_5112	2.407	moderately similar to (392)AT4G13770| Symbols: REF2, CYP83A1 | CYP83A1 (CYTOCHROME P450 83A1); oxygen binding | chr4:79	
EV042601	2.406	moderately similar to (360)AT5G40250| Symbols: | zinc finger (C3HC4-type RING finger) family protein | chr5:16103284-16104414 FOI	
CV544619	2.406	very weakly similar to (82.4)AT3G27880| Symbols: | similar to unknown protein [Arabidopsis thaliana] (TAIR:AT1G23710.1); similar to l	
JCVI_24481	2.406	weakly similar to (158)AT2G40820| Symbols: | 3'-5'-exoribonuclease/ RNA binding | chr2:17041814-17046552 REVERSE no original de	
EV199049	2.406	very weakly similar to (82.0)AT4G36220| Symbols: CYP84A1, FAH1 | FAH1 (FERULATE-5-HYDROXYLASE 1); ferulate 5-hydroxylas	
EX089355	2.406	weakly similar to (109)AT2G37220| Symbols: | 29 kDa ribonucleoprotein, chloroplast, putative / RNA-binding protein cp29, putative | chr	
JCVI_29146	2.405	moderately similar to (250)AT5G13220| Symbols: JAZ10, TIFY9, JAS1, AT5G13220 | JAS1/JAZ10/TIFY9 (JASMONATE-ZIM-DOMAI	
EV203370	2.405	weakly similar to (105)AT2G01890| Symbols: PAP8, ATPAP8 | PAP8 (PURPLE ACID PHOSPHATASE PRECURSOR); protein serine/t	
JCVI_40373	2.405	highly similar to (643)AT4G23940| Symbols: | FtsH protease, putative | chr4:12437118-12441851 FORWARDweakly similar to (121)FTS	
EV040829	2.405	moderately similar to (209)AT5G14420| Symbols: RGLG2 | RGLG2 (RING DOMAIN LIGASE2) | chr5:4648358-4650566 REVERSE [2	
JCVI_406	2.404	moderately similar to (471)AT3G52720| Symbols: | carbonic anhydrase family protein | chr3:19550595-19552094 REVERSE no original	
JCVI_15229	2.404	moderately similar to (202)AT1G75580| Symbols: | auxin-responsive protein, putative | chr1:28381191-28381517 FORWARDvery weakly	
JCVI_24936	2.404	no original description	1.467
JCVI_33269	2.404	moderately similar to (349)AT3G59580| Symbols: | RWP-RK domain-containing protein | chr3:22020629-22023698 FORWARD no origi	
ES994488	2.403	moderately similar to (208)AT5G17570| Symbols: | tatD-related deoxyribonuclease family protein | chr5:5792950-5794582 FORWARD [	
JCVI_19298	2.403	moderately similar to (297)AT5G50860| Symbols: | protein kinase family protein | chr5:20711004-20714209 REVERSEvery weakly simil	
EG020308	2.403	moderately similar to (239)AT2G44740| Symbols: CYCP4;1 | CYCP4;1 (cyclin p4;1); cyclin-dependent protein kinase | chr2:18449362-1	
JCVI_4249	2.403	moderately similar to (313)AT1G03475| Symbols: HEMF1, ATCPO-I, LIN2 | LIN2 (LESION INITIATION 2); coproporphyrinogen oxida	-5.154
EV202861	2.403	moderately similar to (473)AT1G77740| Symbols: | 1-phosphatidylinositol-4-phosphate 5-kinase, putative / PIP kinase, putative / PtdIns(4	
CD816058	2.402	weakly similar to (137)AT4G24230| Symbols: ACBP3 | ACBP3 (ACYL-COA-BINDING DOMAIN 3) | chr4:12567132-12568764 REVEF	
JCVI_39469	2.401	moderately similar to (286)AT1G80510| Symbols: | amino acid transporter family protein | chr1:30277992-30279461 FORWARD no orig	
EV226804	2.401	weakly similar to (118)AT3G61860| Symbols: RSP31, ATRSP31 | ATRSP31 (ARGININE/SERINE-RICH SPLICING FACTOR 31); RNA	
AM389607	2.401	weakly similar to (191)AT5G53050| Symbols: | hydrolase, alpha/beta fold family protein | chr5:21527760-21530658 REVERSE [20118] 3	
JCVI_15135	2.401	weakly similar to (158)AT1G63610| Symbols: | similar to unknown protein [Arabidopsis thaliana] (TAIR:AT2G14910.1); similar to unnar	

JCVI_7904	2.400	weakly similar to (161)AT2G16070| Symbols: PDV2 | PDV2 (PLASTID DIVISION2) | chr2:6991154-6992438 REVERSE no original des	
JCVI_26402	2.400	moderately similar to (291)AT3G09580| Symbols: | amine oxidase family protein | chr3:2942619-2944052 REVERSE no original descript	
JCVI_13143	2.400	moderately similar to (402)AT1G34200| Symbols: | oxidoreductase family protein | chr1:12455771-12456913 FORWARD no original des	
JCVI_26597	2.400	moderately similar to (349)AT5G13760| Symbols: | similar to unknown protein [Arabidopsis thaliana] (TAIR:AT3G04440.1); similar to u	
JCVI_6378	2.400	weakly similar to (134)AT4G29550| Symbols: | similar to unknown protein [Arabidopsis thaliana] (TAIR:AT2G20620.1); contains InterPr	
JCVI_2267	2.400	moderately similar to (444)AT4G22756| Symbols: ATSMO1-2, ATSMO1, SMO1, SMO1-2 | SMO1-2 (STEROL C4-METHYL OXIDASE	
JCVI_19126	2.400	highly similar to (546)AT4G31120| Symbols: SKB1, ATPRMT5, PRMT5 | ATPRMT5/PRMT5/SKB1 (SHK1 BINDING PROTEIN 1); pr	
ES981427	2.400	moderately similar to (234)AT3G50660| Symbols: CYP90B1, CLM, SNP2, DWF4 | DWF4 (DWARF 4) | chr3:18825243-18828149 REVE	
EV022238	2.400	weakly similar to (194)AT1G69250| Symbols: | nuclear transport factor 2 (NTF2) family protein / RNA recognition motif (RRM)-containi	
JCVI_16907	2.399	highly similar to (503)AT4G15475| Symbols: | F-box family protein (FBL4) | chr4:8845925-8848699 FORWARD no original description	
JCVI_32209	2.399	highly similar to (810)AT1G19440| Symbols: | very-long-chain fatty acid condensing enzyme, putative | chr1:6729110-6730660 FORWAF	
JCVI_26430	2.399	moderately similar to (304)AT3G03220| Symbols: EXP13, ATEXP13, ATHEXP ALPHA 1.22, ATEXPA13 | ATEXPA13 (ARABIDOPSI	
JCVI_39425	2.399	moderately similar to (338)AT1G27650| Symbols: ATU2AF35A | ATU2AF35A | chr1:9615289-9616029 FORWARD no original descripti	
JCVI_10692	2.398	highly similar to (519)AT4G18130| Symbols: PHYE | PHYE (PHYTOCHROME DEFECTIVE E); G-protein coupled photoreceptor/ signa	
ES952569	2.398	no similarity	
ES907013	2.398	weakly similar to (124)AT1G13900| Symbols: | calcineurin-like phosphoesterase family protein | chr1:4753491-4755551 REVERSE [2142	
JCVI_3196	2.398	moderately similar to (337)AT1G51780| Symbols: ILL5 | ILL5 (IAA-leucine resistant (ILR)-like gene 5); metallopeptidase | chr1:1920827	
JCVI_20842	2.397	moderately similar to (334)AT5G22630| Symbols: ADT5 | ADT5 (AROGENATE DEHYDRATASE 5); arogenate dehydratase/ prephenat	
ES967768	2.397	no similarity	
JCVI_37493	2.397	no original description	
JCVI_22318	2.397	moderately similar to (265)AT3G15610| Symbols: | transducin family protein / WD-40 repeat family protein | chr3:5291083-5292803 REV	
JCVI_12860	2.397	weakly similar to (144)AT3G09770| Symbols: | zinc finger (C3HC4-type RING finger) family protein | chr3:2996634-2997840 REVERSE	
AT000779	2.396	no similarity	
EV086314	2.396	no similarity	
JCVI_31998	2.396	highly similar to (634)AT5G19580| Symbols: | glyoxal oxidase-related | chr5:6607597-6609519 REVERSE no original description	
EX087775	2.396	very weakly similar to (99.8)AT5G37410| Symbols: | binding | chr5:14853891-14855738 FORWARD [21823]	
JCVI_34204	2.396	no original description	
JCVI_22832	2.395	very weakly similar to (88.6)AT5G55450| Symbols: | protease inhibitor/seed storage/lipid transfer protein (LTP) family protein | chr5:2248	
JCVI_39168	2.395	moderately similar to (414)AT2G31750| Symbols: UGT74D1 | UGT74D1 (UDP-GLUCOSYL TRANSFERASE 74D1); UDP-glycosyltran	
JCVI_3710	2.395	highly similar to (774)AT4G21120| Symbols: CAT1 | AAT1 (CATIONIC AMINO ACID TRANSPORTER 1); cationic amino acid transm	
JCVI_27780	2.394	weakly similar to (184)AT4G24590| Symbols: | similar to unknown protein [Arabidopsis thaliana] (TAIR:AT5G49710.3); similar to unnar	
JCVI_33355	2.394	moderately similar to (397)AT1G22410| Symbols: | 2-dehydro-3-deoxyphosphoheptonate aldolase, putative / 3-deoxy-D-arabino-heptulos	
JCVI_41149	2.393	highly similar to (612)AT1G63050| Symbols: | membrane bound O-acyl transferase (MBOAT) family protein | chr1:23379726-23381843	-2.628
JCVI_10309	2.393	moderately similar to (478)AT5G15680| Symbols: | binding | chr5:5101191-5110796 REVERSE no original description	
EX122757	2.393	moderately similar to (297)AT4G05100| Symbols: AtMYB74 | AtMYB74 (myb domain protein 74); DNA binding / transcription factor | c	
JCVI_11542	2.392	moderately similar to (360)AT4G29490| Symbols: | X-Pro dipeptidase | chr4:14487949-14491327 FORWARD no original description	
JCVI_21416	2.392	weakly similar to (186)AT1G54060| Symbols: | transcription factor | chr1:20184640-20185791 FORWARD no original description	
AM062638	2.392	moderately similar to (320)AT1G12780| Symbols: UGE1 | UGE1 (UDP-D-GLUCOSE/UDP-D-GALACTOSE 4-EPIMERASE 1); UDP-gl	
EV124225	2.392	moderately similar to (328)AT2G37585| Symbols: | glycosyltransferase family 14 protein / core-2/I-branching enzyme family protein | chr	
ES902515	2.392	moderately similar to (484)AT1G53280| Symbols: | DJ-1 family protein | chr1:19868610-19871009 REVERSE [21428]	
CN727990	2.392	moderately similar to (369)AT4G38360| Symbols: | similar to unknown protein [Arabidopsis thaliana] (TAIR:AT1G77220.1); similar to M	
EV115375	2.392	moderately similar to (261)AT3G11010| Symbols: | disease resistance family protein / LRR family protein | chr3:3450994-3453678 REVE	
JCVI_1407	2.392	highly similar to (577)AT5G09810| Symbols: ACT2/7, ACT7 | ACT7 (actin 7) | chr5:3052810-3054221 FORWARDhighly similar to (566	
JCVI_3121	2.391	moderately similar to (254)AT4G22890| Symbols: PGR5-LIKE A | PGR5-LIKE A | chr4:12007168-12009186 FORWARD no original des	
JCVI_6994	2.391	highly similar to (620)AT4G16190| Symbols: | cysteine proteinase, putative | chr4:9171527-9172892 FORWARDmoderately similar to (5	
JCVI_5579	2.391	moderately similar to (392)AT3G16370| Symbols: | GDSL-motif lipase/hydrolase family protein | chr3:5556934-5558357 FORWARDwea	
EE566052	2.391	no similarity	
JCVI_75	2.391	moderately similar to (488)AT3G17410| Symbols: | serine/threonine protein kinase, putative | chr3:5956607-5958888 FORWARDweakly	
JCVI_33268	2.390	moderately similar to (382)AT3G02680| Symbols: ATNBS1, NBS1 | NBS1 (NIJMEGEN BREAKAGE SYNDROME 1) | chr3:576385-579	
CD813609	2.390	moderately similar to (390)AT3G18860| Symbols: | transducin family protein / WD-40 repeat family protein | chr3:6501780-6508358 FOF	
JCVI_25918	2.390	highly similar to (529)AT1G68530| Symbols: CER6, G2, POP1, CUT1 | CUT1 (CUTICULAR 1); catalytic | chr1:25717263-25718396 RE	
JCVI_21634	2.390	weakly similar to (119)AT1G51920| Symbols: | unknown protein | chr1:19297898-19298248 FORWARD no original description	
JCVI_11072	2.390	weakly similar to (145)AT4G39630| Symbols: | similar to unnamed protein product [Vitis vinifera] (GB:CAO71785.1) | chr4:18397743-18	
EX091519	2.389	very weakly similar to (83.2)AT4G03460| Symbols: | ankyrin repeat family protein | chr4:1536402-1540109 REVERSE [21823]	
JCVI_4490	2.389	moderately similar to (320)AT4G40010| Symbols: SNRK2-7, SNRK2.7, SRK2F | SNRK2-7/SNRK2.7/SRK2F (SNF1-RELATED PROTE	
JCVI_11356	2.389	moderately similar to (236)AT1G18440| Symbols: | peptidyl-tRNA hydrolase family protein | chr1:6345987-6347679 FORWARD no orig	
EV165473	2.389	weakly similar to (174)AT5G60920| Symbols: COB | COB (COBRA) | chr5:24528692-24531158 REVERSEweakly similar to (134)COBI	
EX056378	2.388	moderately similar to (289)AT4G28320| Symbols: | glycosyl hydrolase family 5 protein / cellulase family protein | chr4:14018299-140199	
JCVI_33427	2.388	moderately similar to (217)AT1G23780| Symbols: | F-box family protein | chr1:8407042-8408469 REVERSE no original description	
CX188207	2.388	very weakly similar to (80.1)AT5G12140| Symbols: ATCYS1 | ATCYS1 (A. THALIANA CYSTATIN-1); cysteine protease inhibitor | chr5	
EV108729	2.388	weakly similar to (150)AT2G21240| Symbols: BPC4, BBR/BPC4, ATBPC4 | ATBPC4/BBR/BPC4/BPC4 (BASIC PENTACYSTEINE 4)	
EX130273	2.388	moderately similar to (304)AT5G21482| Symbols: ATCKX5, CKX7 | CKX7 (CYTOKININ OXIDASE 7); oxidoreductase | chr5:7226845-	
EV203686	2.388	no similarity	
JCVI_35273	2.387	weakly similar to (109)AT3G21610| Symbols: | similar to unknown protein [Arabidopsis thaliana] (TAIR:AT1G67600.1); similar to unnar	
EV020480	2.387	moderately similar to (323)AT4G34490| Symbols: CAP 1, CAP1, ATCAP1 | ATCAP1 (CYCLASE ASSOCIATED PROTEIN 1) | chr4:16	2.229
EV176191	2.387	weakly similar to (105)AT4G33905| Symbols: | peroxisomal membrane protein 22 kDa, putative | chr4:16254070-16255597 REVERSE [2	
JCVI_4634	2.386	moderately similar to (296)AT3G21865| Symbols: PEX22 | PEX22 (PEROXIN 22); protein binding | chr3:7701315-7703225 REVERSE n	
JCVI_35573	2.386	weakly similar to (139)AT3G13430| Symbols: | zinc finger (C3HC4-type RING finger) family protein | chr3:4367761-4368708 FORWAR	
EV124708	2.386	moderately similar to (283)AT1G01790| Symbols: ATKEA1, KEA1 | KEA1 (K EFFLUX ANTIPORTER 1); potassium:hydrogen antiporte	
AM387285	2.386	no similarity	
CV546572	2.385	no similarity	
AM388362	2.385	moderately similar to (263)AT1G13520| Symbols: | similar to unknown protein [Arabidopsis thaliana] (TAIR:AT1G13480.1); similar to u	-1.917
JCVI_9704	2.385	moderately similar to (496)AT1G73180| Symbols: | eukaryotic translation initiation factor-related | chr1:27521996-27524675 FORWARD	
ES917663	2.385	weakly similar to (199)AT2G33620| Symbols: | DNA-binding family protein / AT-hook protein 1 (AHP1) | chr2:14241825-14243639 FOF	
EV197817	2.385	no similarity	
JCVI_37887	2.385	weakly similar to (188)AT4G21895| Symbols: | similar to AT hook motif-containing protein [Arabidopsis thaliana] (TAIR:AT5G52890.1)	
JCVI_15326	2.385	highly similar to (744)AT5G16150| Symbols: PGLCT, GLT1 | GLT1/PGLCT (GLUCOSE TRANSPORTER 1); carbohydrate transmembr	
DN965500	2.385	very weakly similar to (87.8)AT1G06630| Symbols: | F-box family protein | chr1:2028070-2029441 FORWARD [17359]	
JCVI_41821	2.384	highly similar to (682)AT4G01400| Symbols: | pentatricopeptide (PPR) repeat-containing protein | chr4:573098-577243 REVERSE no ori	
EE460090	2.384	no similarity	
JCVI_15978	2.384	moderately similar to (271)AT1G76760| Symbols: ATY1 | ATY1 (Arabidopsis thioredoxin y1); thiol-disulfide exchange intermediate | chr	

JCVI_17205	2.384	no original description	
JCVI_35035	2.384	moderately similar to (283)AT1G12320| Symbols: | similar to unknown protein [Arabidopsis thaliana] (TAIR:AT1G62840.1); similar to u	
JCVI_41459	2.383	weakly similar to (200)AT5G18830| Symbols: SPL7 | SPL7 (SQUAMOSA PROMOTER BINDING PROTEIN-LIKE 7); DNA binding / tr	
JCVI_27519	2.383	moderately similar to (306)AT4G10760| Symbols: EMB1706 | EMB1706 (EMBRYO DEFECTIVE 1706); S-adenosylmethionine-depende	
DY020582	2.383	moderately similar to (278)AT3G11850| Symbols: | similar to unknown protein [Arabidopsis thaliana] (TAIR:AT5G06560.1); similar to u	
JCVI_18372	2.383	moderately similar to (456)AT4G18905| Symbols: | transducin family protein / WD-40 repeat family protein | chr4:10360245-10363002 F	
JCVI_40994	2.383	moderately similar to (268)AT5G12930| Symbols: | similar to unnamed protein product [Vitis vinifera] (GB:CAO21945.1); similar to hyp	
ES995288	2.382	no similarity	
JCVI_2933	2.382	moderately similar to (262)AT5G45710| Symbols: HSFA4C, RHA1, AT-HSFA4C | AT-HSFA4C (Arabidopsis thaliana heat shock transcri	
JCVI_4625	2.382	moderately similar to (420)AT5G19990| Symbols: ATSUG1, RPT6A | RPT6A; ATPase | chr5:6752146-6754920 FORWARDweakly simil	
EV191661	2.381	no similarity	
BQ704587	2.381	moderately similar to (204)AT5G39960| Symbols: | GTP-binding family protein | chr5:16011423-16014048 FORWARD [11009]	
JCVI_12989	2.381	moderately similar to (372)AT4G33420| Symbols: | peroxidase, putative | chr4:16084859-16086108 FORWARDweakly similar to (184)P	
JCVI_8001	2.380	moderately similar to (333)AT4G31130| Symbols: | similar to hypothetical protein [Vitis vinifera] (GB:CAN64246.1); contains InterPro d	
JCVI_33382	2.380	moderately similar to (254)AT1G01790| Symbols: ATKEA1, KEA1 | KEA1 (K EFFLUX ANTIPORTER 1); potassium:hydrogen antiporte	
ES952449	2.380	no similarity	
EX078411	2.379	moderately similar to (442)AT1G59720| Symbols: | pentatricopeptide (PPR) repeat-containing protein | chr1:21943533-21945449 REVER	
JCVI_37042	2.379	no original description	
EV101710	2.379	moderately similar to (307)AT2G20790| Symbols: | protein binding / protein transporter | chr2:8957243-8959694 REVERSE [21477] 45 7	
JCVI_6867	2.379	highly similar to (580)AT1G65030| Symbols: | transducin family protein / WD-40 repeat family protein | chr1:24160461-24162174 FORW	
EV226248	2.379	weakly similar to (124)AT2G36630| Symbols: | similar to unknown protein [Arabidopsis thaliana] (TAIR:AT2G25737.1); similar to unnar	
CV432188	2.379	very weakly similar to (93.6)AT1G30580| Symbols: | GTP binding | chr1:10831935-10835436 REVERSE [16490] 1 451 521	
CV545450	2.379	moderately similar to (218)AT4G17970| Symbols: | similar to unknown protein [Arabidopsis thaliana] (TAIR:AT5G46600.1); similar to u	
JCVI_29943	2.379	moderately similar to (341)AT3G17670| Symbols: | binding | chr3:6040863-6042018 FORWARD no original description	
JCVI_2477	2.379	moderately similar to (419)AT3G10210| Symbols: | similar to Rho-GTPase-activating protein-related [Arabidopsis thaliana] (TAIR:AT4G	
EE535139	2.378	moderately similar to (214)AT3G20320| Symbols: TGD2 | TGD2 (TRIGALACTOSYLDIACYLGLYCEROL2) | chr3:7087663-7089646 F	
EV084161	2.378	no similarity	-3.869
JCVI_4572	2.378	moderately similar to (425)AT4G21800| Symbols: QQT2 | QQT2 (QUATRE-QUART2); ATP binding | chr4:11573301-11574988 FORW	
JCVI_18276	2.378	moderately similar to (244)AT5G05987| Symbols: | prenylated rab acceptor (PRA1) family protein | chr5:1804882-1806560 FORWARD r	
JCVI_20414	2.377	moderately similar to (447)AT3G60440| Symbols: | similar to unknown protein [Arabidopsis thaliana] (TAIR:AT3G60450.1); similar to u	
JCVI_40405	2.377	moderately similar to (259)AT2G44140| Symbols: | autophagy 4a (APG4a) | chr2:18262182-18264282 REVERSE no original description	
JCVI_21083	2.377	moderately similar to (431)AT5G47730| Symbols: | SEC14 cytosolic factor, putative / polyphosphoinositide-binding protein, putative | chr	
JCVI_37471	2.376	weakly similar to (122)AT2G23580| Symbols: | hydrolase, alpha/beta fold family protein | chr2:10040440-10041390 REVERSEvery weak	
JCVI_14736	2.375	weakly similar to (199)AT1G74730| Symbols: | similar to unknown [Populus trichocarpa x Populus deltoides] (GB:ABK96654.1); contain	
DY016648	2.375	moderately similar to (294)AT5G41700| Symbols: ATUBC8, UBC8 | UBC8 (UBIQUITIN CONJUGATING ENZYME 8); ubiquitin-prote	
JCVI_40323	2.375	highly similar to (544)AT3G17470| Symbols: | RelA/SpoT domain-containing protein / calcium-binding EF-hand family protein | chr3:597	
JCVI_41246	2.375	no original description	
AM062272	2.375	no similarity	
ES949832	2.375	no similarity	
JCVI_5312	2.375	weakly similar to (135)AT5G25290| Symbols: | F-box family protein | chr5:8778595-8779788 FORWARD no original description	
EV125785	2.375	weakly similar to (117)AT1G07930| Symbols: | elongation factor 1-alpha / EF-1-alpha | chr1:2459011-2460455 FORWARDweakly simila	-1.276
EX131054	2.375	weakly similar to (169)AT5G13490| Symbols: AAC2 | AAC2 (ADP/ATP CARRIER 2); binding | chr5:4336037-4337382 FORWARDweal	
JCVI_14909	2.374	moderately similar to (263)AT5G57030| Symbols: LUT2 | LUT2 (LUTEIN DEFICIENT 2); lycopene epsilon cyclase | chr5:23094624-230	
ES940812	2.374	weakly similar to (135)AT5G15710| Symbols: | F-box family protein | chr5:5122794-5124140 FORWARD [21391]	
JCVI_7755	2.374	moderately similar to (352)AT3G53520| Symbols: ATUXS1, UXS1 | UXS1 (UDP-GLUCURONIC ACID DECARBOXYLASE 1) | chr3:1	
JCVI_41046	2.374	no original description	
JCVI_33080	2.374	moderately similar to (246)AT3G55960| Symbols: | NLI interacting factor (NIF) family protein | chr3:20771776-20773871 REVERSE no	
JCVI_5872	2.374	moderately similar to (413)AT4G01070| Symbols: GT72B1 | GT72B1; UDP-glucosyltransferase/ UDP-glycosyltransferase/ transferase, tra	
EE521658	2.373	moderately similar to (290)AT2G36290| Symbols: | hydrolase, alpha/beta fold family protein | chr2:15215946-15217847 REVERSE [2018	
JCVI_4535	2.373	moderately similar to (367)AT5G28500| Symbols: | similar to unknown protein [Arabidopsis thaliana] (TAIR:AT3G04550.1); similar to u	
JCVI_776	2.373	moderately similar to (399)AT1G03210| Symbols: | phenazine biosynthesis PhzC/PhzF family protein | chr1:782948-784240 FORWARD	
JCVI_34309	2.373	weakly similar to (107)AT5G48640| Symbols: | cyclin family protein | chr5:19740879-19742867 REVERSEvery weakly similar to (81.6)(	
ES994973	2.373	no similarity	
JCVI_14756	2.373	moderately similar to (319)AT3G08860| Symbols: | alanine--glyoxylate aminotransferase, putative / beta-alanine-pyruvate aminotransfera:	
JCVI_6640	2.372	moderately similar to (332)AT1G07470| Symbols: | transcription factor IIA large subunit, putative / TFIIA large subunit, putative | chr1:2	
AM395956	2.372	weakly similar to (109)AT3G20770| Symbols: EIN3 | EIN3 (ETHYLENE-INSENSITIVE3); transcription factor | chr3:7260708-7262594 I	
EV193959	2.371	weakly similar to (139)AT4G16770| Symbols: | oxidoreductase, 2OG-Fe(II) oxygenase family protein | chr4:9434590-9437187 REVERSE	
JCVI_5219	2.371	moderately similar to (351)AT1G07840| Symbols: | leucine zipper factor-related | chr1:2424600-2426128 FORWARD no original descrip	
EE433406	2.371	moderately similar to (207)AT1G70180| Symbols: | sterile alpha motif (SAM) domain-containing protein | chr1:26430431-26432760 FOR	
JCVI_2318	2.371	moderately similar to (213)AT1G74230| Symbols: GR-RBP5 | GR-RBP5 (glycine-rich RNA-binding protein 5); RNA binding | chr1:2791	-2.617
JCVI_5194	2.371	moderately similar to (291)AT2G25830| Symbols: | YebC-related | chr2:11026169-11028743 REVERSE no original description	
JCVI_877	2.370	moderately similar to (365)AT5G44200| Symbols: ATCBP20, CBP20 | CBP20 (CAP-BINDING PROTEIN 20) | chr5:17819289-1782110	
JCVI_7764	2.370	moderately similar to (223)AT5G16200| Symbols: | 50S ribosomal protein-related | chr5:5289662-5290153 REVERSE no original descrip	
EX055750	2.370	moderately similar to (211)AT4G12030| Symbols: | bile acid:sodium symporter family protein | chr4:7211710-7212707 FORWARD [218	
EX137918	2.370	moderately similar to (479)AT3G24180| Symbols: | catalytic | chr3:8735018-8741154 REVERSE [21833]	
CX193620	2.369	weakly similar to (155)AT1G31350| Symbols: | F-box family protein | chr1:11221500-11222687 REVERSE [16807]	
JCVI_25676	2.369	very weakly similar to (89.7)AT1G71130| Symbols: | AP2 domain-containing transcription factor, putative | chr1:26826539-26827024 FOI	
EX134698	2.369	weakly similar to (179)AT4G30830| Symbols: | similar to unknown protein [Arabidopsis thaliana] (TAIR:AT2G24140.1); similar to unnar	
JCVI_18697	2.369	moderately similar to (341)AT5G41610| Symbols: CHX18, ATCHX18 | ATCHX18 (cation/hydrogen exchanger 18); monovalent cation:pr	
JCVI_31597	2.369	moderately similar to (297)AT4G29070| Symbols: | similar to unnamed protein product [Vitis vinifera] (GB:CAO16862.1); contains Inter	
JCVI_3662	2.368	highly similar to (523)AT5G54840| Symbols: | GTP-binding family protein | chr5:22294587-22295554 REVERSE no original description	
JCVI_34889	2.368	moderately similar to (386)AT3G48560| Symbols: ALS, AHAS, TZP5, IMR1, CSR1 | CSR1 (CHLORSULFURON/IMIDAZOLINONE RI	
EX037560	2.368	moderately similar to (315)AT2G43080| Symbols: AT-P4H-1 | AT-P4H-1 (A. THALIANA P4H ISOFORM 1); oxidoreductase, acting on p	
AM057953	2.368	moderately similar to (301)AT5G55990| Symbols: ATCBL2, CBL2 | CBL2 (calcineurin B-like protein 2); calcium ion binding | chr5:2268	
CD835512	2.368	moderately similar to (368)AT5G10180| Symbols: SULTR2;1, AST68 | AST68 (Sulfate transporter 2.1) | chr5:3193226-3196819 FORWA	
JCVI_17332	2.367	moderately similar to (387)AT1G30300| Symbols: | similar to phosphonate metabolism protein-related [Arabidopsis thaliana] (TAIR:AT4	
JCVI_2491	2.366	moderately similar to (372)AT1G10360| Symbols: GST29, ATGSTU18 | ATGSTU18 (GLUTATHIONE S-TRANSFERASE 29); glutathio	
JCVI_39389	2.366	moderately similar to (340)AT4G14950| Symbols: | similar to unknown protein [Arabidopsis thaliana] (TAIR:AT1G05360.1); similar to u	
JCVI_15797	2.366	weakly similar to (111)AT4G06676| Symbols: | similar to unnamed protein product [Vitis vinifera] (GB:CAO46991.1); contains InterPro	
JCVI_37223	2.366	moderately similar to (286)AT1G75340| Symbols: | zinc finger (CCCH-type) family protein | chr1:28272814-28275534 REVERSE no orig	
EX122808	2.366	no similarity	

JCVI_13325	2.366	highly similar to (857)AT3G03570| Symbols: | similar to signal transducer [Arabidopsis thaliana] (TAIR:AT4G40050.1); similar to unnam	
RC_ES966375	2.366	no similarity	
EE547962	2.366	moderately similar to (301)AT5G27120| Symbols: | SAR DNA-binding protein, putative | chr5:9541290-9543687 FORWARD [20128] 45	
JCVI_5255	2.366	moderately similar to (219)AT5G40420| Symbols: PA23, OLEO2 | OLEO2 (OLEOSIN 2) | chr5:16190850-16191968 REVERSEvery weal	
JCVI_16627	2.365	moderately similar to (446)AT3G14830| Symbols: | similar to unknown protein [Arabidopsis thaliana] (TAIR:AT1G53450.2); similar to u	
EV156912	2.365	weakly similar to (173)AT1G27930| Symbols: | similar to unknown protein [Arabidopsis thaliana] (TAIR:AT1G67330.1); similar to unkno	
JCVI_36442	2.365	weakly similar to (143)AT5G08240| Symbols: | similar to unknown protein [Arabidopsis thaliana] (TAIR:AT5G23160.1) | chr5:2651383-2	
ES909141	2.365	weakly similar to (189)AT5G42655| Symbols: | similar to disease resistance-responsive family protein [Arabidopsis thaliana] (TAIR:AT5(	
JCVI_17198	2.365	moderately similar to (263)AT4G38630| Symbols: MCB1, ATMCB1, MBP1, RPN10 | RPN10 (REGULATORY PARTICLE NON-ATPA	
DN963370	2.364	weakly similar to (143)AT3G07560| Symbols: APM2, PEX13 | APM2/PEX13 (ABERRANT PEROXISOME MORPHOLOGY 2); protein	
JCVI_21225	2.364	weakly similar to (126)AT4G23580| Symbols: | kelch repeat-containing F-box family protein | chr4:12304256-12305407 REVERSE no or	
JCVI_3005	2.364	moderately similar to (342)AT5G03290| Symbols: | isocitrate dehydrogenase, putative / NAD+ isocitrate dehydrogenase, putative | chr5:79	
EE433644	2.364	no similarity	
EX106564	2.364	moderately similar to (232)AT5G20820| Symbols: | auxin-responsive protein-related | chr5:7046913-7047296 REVERSE [21827] 1 573 5	
EE538699	2.363	weakly similar to (146)AT4G05460| Symbols: | F-box family protein (FBL20) | chr4:2761103-2762397 REVERSE [20161]	
JCVI_1951	2.363	moderately similar to (295)AT2G21970| Symbols: SEP2 | SEP2 (STRESS ENHANCED PROTEIN 2) | chr2:9364268-9364954 REVERSE	
ES903641	2.363	moderately similar to (392)AT4G20840| Symbols: | FAD-binding domain-containing protein | chr4:11157927-11159546 FORWARD [214	
JCVI_17134	2.363	moderately similar to (412)AT3G63130| Symbols: RANGAP1 | RANGAP1 (RAN GTPASE ACTIVATING PROTEIN 1); RAN GTPase a	
EH414064	2.363	moderately similar to (330)AT3G06380| Symbols: ATTLP9 | ATTLP9 (TUBBY-LIKE PROTEIN 9); phosphoric diester hydrolase/ protein	
JCVI_3000	2.363	weakly similar to (175)AT5G61240| Symbols: | protein binding | chr5:24646711-24649184 FORWARD no original description	-1.714
DY026614	2.363	moderately similar to (231)AT4G22890| Symbols: PGR5-LIKE A | PGR5-LIKE A | chr4:12007168-12009186 FORWARD [18978]	
EE525789	2.362	weakly similar to (174)AT3G50410| Symbols: OBP1 | OBP1 (OBF BINDING PROTEIN 1); DNA binding / transcription factor | chr3:187	
JCVI_37759	2.362	weakly similar to (121)AT1G27420| Symbols: | kelch repeat-containing F-box family protein | chr1:9519095-9520507 FORWARD no orig	
CX273018	2.362	weakly similar to (196)AT2G24150| Symbols: HHP3 | HHP3 (heptahelical protein 3); receptor | chr2:10272712-10274425 REVERSE [168	
EV203692	2.362	no similarity	
JCVI_19151	2.361	moderately similar to (291)AT3G43600| Symbols: AAO2 | AAO2 (ALDEHYDE OXIDASE 2) | chr3:15523766-15528363 REVERSEmod	2.057
JCVI_33326	2.361	moderately similar to (305)AT4G21680| Symbols: | proton-dependent oligopeptide transport (POT) family protein | chr4:11517552-11519	
EE415626	2.361	weakly similar to (133)AT3G16990| Symbols: | TENA/THI-4 family protein | chr3:5795943-5796789 REVERSEweakly similar to (104)A	
CD832214	2.361	weakly similar to (143)AT5G65940| Symbols: CHY1 | CHY1 (BETA-HYDROXYISOBUTYRYL-COA HYDROLASE 1) | chr5:26394153	
JCVI_9079	2.360	moderately similar to (239)AT2G20930| Symbols: | similar to unknown [Populus trichocarpa] (GB:ABK93191.1); contains InterPro doma	
JCVI_20172	2.360	moderately similar to (411)AT3G08970| Symbols: | DNAJ heat shock N-terminal domain-containing protein | chr3:2737594-2740270 FOR	
JCVI_4272	2.360	moderately similar to (295)AT1G14570| Symbols: | UBX domain-containing protein | chr1:4983765-4987187 FORWARD no original des	
JCVI_33225	2.360	moderately similar to (299)AT4G01220| Symbols: | similar to RGXT1 (RHAMNOGALACTURONAN XYLOSYLTRANSFERASE 1), U	
JCVI_16279	2.360	moderately similar to (209)AT4G33040| Symbols: | glutaredoxin family protein | chr4:15940782-15941216 REVERSE no original descrip	
EE550763	2.359	no similarity	
JCVI_29295	2.359	weakly similar to (104)AT3G10760| Symbols: | myb family transcription factor | chr3:3369819-3370826 FORWARD no original descripti	
JCVI_28122	2.359	moderately similar to (440)AT2G31340| Symbols: EMB1381 | EMB1381 (EMBRYO DEFECTIVE 1381) | chr2:13368691-13371710 FOR	
JCVI_30712	2.359	weakly similar to (167)AT4G15248| Symbols: | DNA binding / zinc ion binding | chr4:8708881-8709234 FORWARD no original descripti	
JCVI_41656	2.358	very weakly similar to (93.6)AT2G04515| Symbols: | similar to unknown protein [Arabidopsis thaliana] (TAIR:AT2G04495.1) | chr2:1573	
JCVI_774	2.358	moderately similar to (345)AT1G44575| Symbols: PSBS, NPQ4 | NPQ4 (NONPHOTOCHEMICAL QUENCHING) | chr1:16874208-1687	
JCVI_20836	2.358	highly similar to (551)AT1G74960| Symbols: KAS2, FAB1 | FAB1 (FATTY ACID BIOSYNTHESIS 1); fatty-acid synthase | chr1:281562	
EX042384	2.358	weakly similar to (176)AT3G53230| Symbols: | cell division cycle protein 48, putative / CDC48, putative | chr3:19734394-19737467 FOR	
EE430303	2.357	weakly similar to (117)AT5G58250| Symbols: | similar to hypothetical protein [Vitis vinifera] (GB:CAN75840.1); contains domain PD02(	
JCVI_4966	2.357	moderately similar to (401)AT5G47120| Symbols: BI-1, ATBI1, ATBI-1 | ATBI-1 (ARABIDOPSIS BAX INHIBITOR 1) | chr5:19153298	
ES948539	2.357	no similarity	
CV546251	2.357	moderately similar to (255)AT1G18480| Symbols: | calcineurin-like phosphoesterase family protein | chr1:6361632-6362807 FORWARD	
EV191337	2.357	weakly similar to (149)AT2G22790| Symbols: | similar to unknown protein [Arabidopsis thaliana] (TAIR:AT5G67020.1); similar to unnar	-1.949
JCVI_8854	2.356	moderately similar to (365)AT5G56190| Symbols: | WD-40 repeat family protein | chr5:22759880-22762135 FORWARD no original desc	
JCVI_16261	2.356	moderately similar to (375)AT5G22100| Symbols: | RNA cyclase family protein | chr5:7329018-7330721 FORWARD no original descript	
JCVI_30611	2.356	highly similar to (581)AT4G27830| Symbols: | glycosyl hydrolase family 1 protein | chr4:13861800-13864495 REVERSEmoderately simi	
JCVI_20500	2.356	highly similar to (792)AT2G37230| Symbols: | pentatricopeptide (PPR) repeat-containing protein | chr2:15644256-15646529 REVERSEw	
JCVI_28016	2.356	weakly similar to (130)AT5G56050| Symbols: | similar to unknown protein [Arabidopsis thaliana] (TAIR:AT4G26490.1); similar to hypot	
JCVI_28065	2.356	moderately similar to (402)AT4G27100| Symbols: | RAB GTPase activator | chr4:13595851-13598626 FORWARD no original description	
JCVI_5129	2.355	moderately similar to (451)AT1G26820| Symbols: RNS3 | RNS3 (RIBONUCLEASE 3); endoribonuclease | chr1:9292747-9293709 REVE	
EE531224	2.355	no similarity	
EE505257	2.355	weakly similar to (120)AT5G27420| Symbols: | zinc finger (C3HC4-type RING finger) family protein | chr5:9684122-9685228 FORWAR	
JCVI_2214	2.355	moderately similar to (374)AT1G29660| Symbols: | GDSL-motif lipase/hydrolase family protein | chr1:10371941-10373610 FORWARDv	
JCVI_37338	2.355	highly similar to (728)AT4G10770| Symbols: ATOPT7 | ATOPT7 (oligopeptide transporter 7); oligopeptide transporter | chr4:6628642-66	2.322
JCVI_1772	2.355	moderately similar to (465)AT5G04740| Symbols: | ACT domain-containing protein | chr5:1368714-1371392 REVERSE no original descr	-2.371
JCVI_39349	2.354	highly similar to (508)AT2G21410| Symbols: VHA-A2 | VHA-A2 (VACUOLAR PROTON ATPASE A2); ATPase | chr2:9169783-917522	
JCVI_38460	2.354	moderately similar to (321)AT1G75960| Symbols: | AMP-binding protein, putative | chr1:28521848-28523482 FORWARD no original de	
JCVI_39102	2.354	moderately similar to (233)AT3G21760| Symbols: | UDP-glucoronosyl/UDP-glucosyl transferase family protein | chr3:7667106-7668563	
RC_JCVI_32011	2.354	no original description	
EV178328	2.354	weakly similar to (103)AT2G44730| Symbols: | transcription factor | chr2:18444522-18445640 REVERSE [21487] 85 916 916	
EE533808	2.354	weakly similar to (197)AT2G41720| Symbols: EMB2654 | EMB2654 (EMBRYO DEFECTIVE 2654) | chr2:17410822-17414205 REVER	-3.925
JCVI_21692	2.354	highly similar to (510)AT4G17550| Symbols: | transporter-related | chr4:9777951-9779751 REVERSE no original description	
JCVI_22836	2.354	moderately similar to (224)AT5G40770| Symbols: ATPHB3 | ATPHB3 (PROHIBITIN 3) | chr5:16332817-16333849 REVERSE no origin	
EE517501	2.354	weakly similar to (142)AT1G74910| Symbols: | ADP-glucose pyrophosphorylase family protein | chr1:28139431-28142117 REVERSE [2(	1.703
JCVI_39038	2.353	moderately similar to (357)AT2G45320| Symbols: | binding / catalytic | chr2:18691256-18693181 REVERSE no original description	
EX132897	2.353	highly similar to (563)AT5G20680| Symbols: | similar to unknown protein [Arabidopsis thaliana] (TAIR:AT5G64020.1); similar to Os11g	
JCVI_34773	2.353	weakly similar to (135)AT3G20300| Symbols: | extracellular ligand-gated ion channel | chr3:7079838-7081815 REVERSE no original desc	
EE401455	2.353	no similarity	
JCVI_19118	2.352	moderately similar to (434)AT2G46260| Symbols: | BTB/POZ domain-containing protein | chr2:19003184-19005536 FORWARD no orig	
CX195692	2.352	weakly similar to (167)AT5G19930| Symbols: | integral membrane family protein | chr5:6737874-6739285 REVERSE [16807]	
EV190427	2.352	weakly similar to (187)AT2G01260| Symbols: | similar to unknown protein [Arabidopsis thaliana] (TAIR:AT1G15030.1); similar to unkno	
EX060307	2.352	weakly similar to (171)AT2G36050| Symbols: ATOFP15, OFP15 | ATOFP15/OFP15 (Arabidopsis thaliana ovate family protein 15) | chr2	
JCVI_20516	2.352	moderately similar to (221)AT3G48740| Symbols: | nodulin MtN3 family protein | chr3:18063799-18065648 REVERSE no original descr	
CX280365	2.352	moderately similar to (370)AT3G10200| Symbols: | dehydration-responsive protein-related | chr3:3157623-3160021 FORWARD [16820]	
AM395464	2.351	no similarity	
JCVI_300	2.351	moderately similar to (418)AT5G13490| Symbols: AAC2 | AAC2 (ADP/ATP CARRIER 2); binding | chr5:4336037-4337382 FORWARD	
EV021169	2.351	moderately similar to (212)AT4G23515| Symbols: | Toll-Interleukin-Resistance (TIR) domain-containing protein | chr4:12271392-122728	

JCVI_4502	2.350	highly similar to (560)AT1G80820| Symbols: CCR2 | CCR2 (CINNAMOYL COA REDUCTASE) | chr1:30375538-30377352 FORWARD	
RC_CV432550	2.350	no similarity	
JCVI_11862	2.350	weakly similar to (132)AT5G40670| Symbols: | PQ-loop repeat family protein / transmembrane family protein | chr5:16303196-16304819	
JCVI_32973	2.350	moderately similar to (361)AT2G45050| Symbols: | zinc finger (GATA type) family protein | chr2:18590033-18590920 FORWARD no or	
JCVI_36350	2.350	highly similar to (614)AT1G73930| Symbols: | similar to unnamed protein product [Vitis vinifera] (GB:CAO68016.1); similar to hypothet	
EE446248	2.350	weakly similar to (150)AT1G26270| Symbols: | phosphatidylinositol 3- and 4-kinase family protein | chr1:9089809-9091701 REVERSE [2	
AM060040	2.350	moderately similar to (236)AT1G69610| Symbols: | structural constituent of ribosome | chr1:26190617-26193012 FORWARD [17712]	
JCVI_2091	2.350	moderately similar to (239)AT5G02870| Symbols: | 60S ribosomal protein L4/L1 (RPL4D) | chr5:657828-659524 FORWARD no original	
JCVI_1492	2.349	moderately similar to (357)AT1G27000| Symbols: | bZIP family transcription factor | chr1:9374055-9376409 FORWARD no original desc	
JCVI_2441	2.349	moderately similar to (292)AT2G14860| Symbols: | peroxisomal membrane protein 22 kDa, putative | chr2:6394902-6396645 REVERSE	1.865
EE464318	2.348	moderately similar to (334)AT5G48330| Symbols: | regulator of chromosome condensation (RCC1) family protein | chr5:19603215-19604	
JCVI_9170	2.348	weakly similar to (159)AT3G21560| Symbols: UGT84A2 | UGT84A2; UDP-glycosyltransferase/ sinapate 1-glucosyltransferase | chr3:759	
JCVI_11193	2.348	moderately similar to (340)AT5G05160| Symbols: | leucine-rich repeat transmembrane protein kinase, putative | chr5:1528001-1530018 F	
EV082186	2.348	weakly similar to (162)AT1G17780| Symbols: | similar to unknown protein [Arabidopsis thaliana] (TAIR:AT2G16575.1) | chr1:6124310-6	
ES981677	2.348	no similarity	
ES901120	2.347	weakly similar to (122)AT2G33170| Symbols: | leucine-rich repeat transmembrane protein kinase, putative | chr2:14063448-14066906 RE	
JCVI_30659	2.347	no original description	-2.964
JCVI_21111	2.347	moderately similar to (240)AT4G34000| Symbols: ABF3, DPBF5 | ABF3/DPBF5 (ABSCISIC ACID RESPONSIVE ELEMENTS-BINDIN	
JCVI_42234	2.346	highly similar to (662)AT1G12680| Symbols: PEPKR2 | PEPKR2 (PHOSPHOENOLPYRUVATE CARBOXYLASE-RELATED KINASE	
JCVI_106	2.346	moderately similar to (395)AT2G37170| Symbols: PIP2;2, PIP2B | PIP2B (plasma membrane intrinsic protein 2;2); water channel | chr2:1	
JCVI_19880	2.346	highly similar to (738)AT2G18900| Symbols: | transducin family protein / WD-40 repeat family protein | chr2:8195573-8199491 REVERS	
JCVI_10521	2.346	moderately similar to (301)AT4G20020| Symbols: | similar to unknown protein [Arabidopsis thaliana] (TAIR:AT5G44780.1); similar to C	
JCVI_12260	2.345	weakly similar to (167)AT1G22690| Symbols: | gibberellin-responsive protein, putative | chr1:8027317-8027950 FORWARD no original d	
JCVI_14922	2.345	moderately similar to (310)AT4G28660| Symbols: PSB28 | PSB28 (PHOTOSYSTEM II REACTION CENTER PSB28 PROTEIN) | chr4:	
CX280917	2.345	weakly similar to (131)AT1G78550| Symbols: | oxidoreductase, 2OG-Fe(II) oxygenase family protein | chr1:29549924-29551253 REVER	
JCVI_9294	2.344	highly similar to (665)AT2G38860| Symbols: YLS5 | YLS5 (yellow-leaf-specific gene 5) | chr2:16240707-16242285 REVERSE no origina	
JCVI_9715	2.344	weakly similar to (117)AT1G63120| Symbols: ATRBL2 | ATRBL2 (ARABIDOPSIS THALIANA RHOMBOID-LIKE 2); serine-type endo	
JCVI_37785	2.344	no original description	
ES907341	2.344	weakly similar to (162)AT5G48970| Symbols: | mitochondrial substrate carrier family protein | chr5:19874254-19876600 REVERSE [214	
CK991398	2.344	moderately similar to (330)AT5G63180| Symbols: | pectate lyase family protein | chr5:25358332-25360339 REVERSEmoderately similar	
CX194214	2.344	no similarity	
JCVI_42365	2.344	no original description	
JCVI_21372	2.344	no original description	
JCVI_25267	2.344	moderately similar to (354)AT1G58360| Symbols: NAT2, AAP1 | AAP1 (AMINO ACID PERMEASE 1); amino acid transmembrane tran	-1.575
DY018335	2.343	moderately similar to (259)AT3G04450| Symbols: | transcription factor | chr3:1184308-1186270 FORWARD [18979]	
JCVI_11789	2.343	highly similar to (644)AT4G14160| Symbols: | transport protein, putative | chr4:8167569-8173021 FORWARD no original description	
CN728748	2.342	weakly similar to (103)AT1G06980| Symbols: | similar to unknown protein [Arabidopsis thaliana] (TAIR:AT2G30230.1); similar to unnar	
JCVI_7690	2.342	moderately similar to (396)AT1G70320| Symbols: UPL2 | UPL2 (UBIQUITIN-PROTEIN LIGASE 2); ubiquitin-protein ligase | chr1:2649	
EE431674	2.342	weakly similar to (132)AT1G47530| Symbols: | ripening-responsive protein, putative | chr1:17454164-17456550 FORWARD [20151] 1 3	
CV432667	2.342	no similarity	
JCVI_22455	2.341	moderately similar to (221)AT1G33110| Symbols: | MATE efflux family protein | chr1:12005064-12008020 FORWARD no original descr	
JCVI_3142	2.341	moderately similar to (470)AT1G63940| Symbols: | monodehydroascorbate reductase, putative | chr1:23733758-23737197 FORWARDwe	
EE530693	2.341	weakly similar to (107)AT3G16740| Symbols: | F-box family protein | chr3:5699482-5700657 FORWARD [20175]	
JCVI_12642	2.341	moderately similar to (473)AT3G13700| Symbols: | RNA-binding protein, putative | chr3:4490866-4492639 REVERSE no original descrip	
JCVI_37711	2.341	highly similar to (551)AT1G43620| Symbols: | UDP-glucose:sterol glucosyltransferase, putative | chr1:16428094-16431940 REVERSE no	
EX094199	2.341	moderately similar to (350)AT5G01470| Symbols: | similar to unnamed protein product [Vitis vinifera] (GB:CAO15817.1); contains doma	
JCVI_41790	2.341	moderately similar to (343)AT1G04945| Symbols: | similar to hypothetical protein [Vitis vinifera] (GB:CAN60314.1); contains InterPro d	
JCVI_16379	2.340	highly similar to (564)AT4G39950| Symbols: CYP79B2 | CYP79B2 (cytochrome P450, family 79, subfamily B, polypeptide 2); oxygen bi	
ES948135	2.340	moderately similar to (311)AT5G64960| Symbols: CDKC;2 | CDKC;2 (CYCLIN-DEPENDENT KINASE C;2) | chr5:25973376-25975653	
JCVI_9070	2.340	moderately similar to (347)AT1G33110| Symbols: | MATE efflux family protein | chr1:12005064-12008020 FORWARD no original descr	
JCVI_11769	2.340	highly similar to (564)AT1G26830| Symbols: CUL3A, ATCUL3A, ATCUL3, CUL3 | ATCUL3/ATCUL3A/CUL3/CUL3A (Cullin 3A); pr	
JCVI_4065	2.340	moderately similar to (414)AT1G62640| Symbols: KAS III | KAS III (3-KETOACYL-ACYL CARRIER PROTEIN SYNTHASE III); 3-ox	
EX018573	2.339	weakly similar to (139)AT2G12480| Symbols: SCPL43 | SCPL43; serine carboxypeptidase | chr2:5076877-5079309 REVERSEvery weakl	
EV042682	2.339	weakly similar to (192)AT4G17640| Symbols: CKB2 | CKB2 (casein kinase II beta chain 2); protein kinase CK2 regulator | chr4:9825451-	
JCVI_2801	2.339	moderately similar to (392)AT4G33620| Symbols: | Ulp1 protease family protein | chr4:16147695-16152856 FORWARD no original desc	
ES997484	2.339	very weakly similar to (84.7)AT3G62870| Symbols: | 60S ribosomal protein L7A (RPL7aB) | chr3:23253837-23255248 REVERSE [21427	-1.960
CD818380	2.339	moderately similar to (390)AT4G36830| Symbols: | GNS1/SUR4 membrane family protein | chr4:17349515-17350384 FORWARD [1397	
JCVI_758	2.338	highly similar to (531)AT2G33040| Symbols: | ATP synthase gamma chain, mitochondrial (ATPC) | chr2:14026055-14028124 REVERSE	
JCVI_32827	2.338	moderately similar to (444)AT4G12230| Symbols: | esterase/lipase/thioesterase family protein | chr4:7284639-7287342 FORWARD no or	
JCVI_1155	2.338	moderately similar to (454)AT1G03630| Symbols: PORC, POR C | POR C (PROTOCHLOROPHYLLIDE OXIDOREDUCTASE); oxidor	
EV170490	2.338	moderately similar to (344)AT1G23460| Symbols: | polygalacturonase | chr1:8327371-8329611 FORWARDweakly similar to (151)PGLR	
EE533271	2.338	moderately similar to (250)AT2G39740| Symbols: | similar to unknown protein [Arabidopsis thaliana] (TAIR:AT3G45750.1); similar to u	
CV546578	2.338	no similarity	
JCVI_2159	2.337	very weakly similar to (90.9)AT5G03495| Symbols: | nucleotide binding | chr5:873803-875024 REVERSE no original description	
EE484242	2.337	no similarity	
JCVI_4005	2.337	weakly similar to (118)AT3G27690| Symbols: LHCB2.3, LHCB2, LHCB2:4 | LHCB2:4 (Photosystem II light harvesting complex gene 2.3	
JCVI_17023	2.336	moderately similar to (339)AT1G74960| Symbols: KAS2, FAB1 | FAB1 (FATTY ACID BIOSYNTHESIS 1); fatty-acid synthase | chr1:28	-1.914
DY024996	2.336	weakly similar to (142)AT3G30520| Symbols: | similar to unknown protein [Arabidopsis thaliana] (TAIR:AT3G42870.1) | chr3:12132819	
BG544888	2.336	no similarity	
ES940202	2.335	weakly similar to (121)AT3G05910| Symbols: | pectinacetylesterase, putative | chr3:1764515-1767246 REVERSE [21391]	
EV162217	2.335	moderately similar to (233)AT1G10290| Symbols: DRP2A, ADL6 | ADL6 (DYNAMIN-LIKE PROTEIN 6) | chr1:3370776-3377122 FOR	
RC_EE417205	2.334	no similarity	
EE531535	2.334	weakly similar to (103)AT5G57610| Symbols: | protein kinase family protein | chr5:23342533-23346325 FORWARD [20175]	
EX085856	2.334	moderately similar to (339)AT2G37890| Symbols: | mitochondrial substrate carrier family protein | chr2:15869095-15870927 REVERSEv	
JCVI_1298	2.334	moderately similar to (283)AT3G02910| Symbols: | Identical to UPF0131 protein At3g02910 [Arabidopsis Thaliana] (GB:Q9M8T3); simi	
EX071451	2.334	moderately similar to (499)AT3G13080| Symbols: EST2, MRP3, ATMRP3 | ATMRP3 (Arabidopsis thaliana multidrug resistance-associat	
JCVI_23550	2.333	moderately similar to (274)AT2G24200| Symbols: | cytosol aminopeptidase | chr2:10294097-10296530 REVERSEmoderately similar to (	
JCVI_6598	2.333	weakly similar to (164)AT1G75750| Symbols: GASA1 | GASA1 (GAST1 PROTEIN HOMOLOG 1) | chr1:28445474-28445945 REVERS	
EE466887	2.333	weakly similar to (179)AT1G51730| Symbols: | RWD domain-containing protein | chr1:19190480-19192306 REVERSE [20188]	
JCVI_16746	2.333	moderately similar to (258)AT5G01990| Symbols: | auxin efflux carrier family protein | chr5:377370-379597 REVERSE no original descr	
JCVI_15353	2.332	moderately similar to (338)AT3G02230| Symbols: ATRGP1, ATRGP, RGP1 | RGP1 (REVERSIBLY GLYCOSYLATED POLYPEPTIDE	

JCVI_10875	2.332	moderately similar to (317)AT4G17720| Symbols: | RNA recognition motif (RRM)-containing protein | chr4:9862673-9864511 REVERSI	-1.918
EE452964	2.332	no similarity	
ES912869	2.332	moderately similar to (340)AT5G17330| Symbols: GAD1, GAD | GAD (Glutamate decarboxylase 1); calmodulin binding | chr5:5711143-5	
JCVI_15396	2.332	no original description	
JCVI_22607	2.331	weakly similar to (173)AT4G33040| Symbols: | glutaredoxin family protein | chr4:15940782-15941216 REVERSE no original description	
EV198220	2.331	no similarity	
EE490643	2.331	moderately similar to (225)AT5G05580| Symbols: SH1, FAD8 | FAD8 (FATTY ACID DESATURASE 8); omega-3 fatty acid desaturase |	
EV109304	2.331	very weakly similar to (94.7)AT2G28500| Symbols: LBD11 | LBD11 (LOB DOMAIN-CONTAINING PROTEIN 11) | chr2:12193680-121	
EE449786	2.330	weakly similar to (150)AT5G51710| Symbols: ATKEA5, KEA5 | KEA5 (K+ efflux antiporter 5) | chr5:21021792-21025806 REVERSE [2	
JCVI_135	2.330	highly similar to (770)AT5G05730| Symbols: TRP5, AMT1, WEI2, ASA1 | ASA1 (ANTHRANILATE SYNTHASE ALPHA SUBUNIT 1	
JCVI_14920	2.329	moderately similar to (421)AT1G66900| Symbols: | similar to unknown protein [Arabidopsis thaliana] (TAIR:AT5G38220.1); similar to u	
EE504042	2.329	weakly similar to (137)AT3G10770| Symbols: | nucleic acid binding | chr3:3372466-3374031 REVERSE [20129]	
JCVI_26098	2.329	moderately similar to (302)AT3G03610| Symbols: | phagocytosis and cell motility protein ELMO1-related | chr3:869486-871694 FORWA	
JCVI_24361	2.329	weakly similar to (142)AT5G18140| Symbols: | DNAJ heat shock N-terminal domain-containing protein | chr5:5998237-5999701 FORWA	
JCVI_15356	2.329	weakly similar to (133)AT1G22090| Symbols: EMB2204 | EMB2204 (EMBRYO DEFECTIVE 2204) | chr1:7795710-7797241 FORWAR	
JCVI_27087	2.329	moderately similar to (253)AT3G27090| Symbols: | similar to unknown protein [Arabidopsis thaliana] (TAIR:AT5G42050.1); similar to h	
JCVI_7808	2.329	highly similar to (615)AT3G14225| Symbols: GLIP4 | GLIP4; carboxylesterase | chr3:4734623-4736000 REVERSEweakly similar to (119	
CX269057	2.329	moderately similar to (239)AT3G18630| Symbols: | uracil DNA glycosylase family protein | chr3:6411331-6413008 REVERSE [16816]	
JCVI_15323	2.329	moderately similar to (429)AT1G74710| Symbols: ICS1, EDS16, SID2 | ICS1 (ISOCHORISMATE SYNTHASEI); isochorismate synthase	
EV100858	2.328	weakly similar to (136)AT3G18430| Symbols: | calcium-binding EF hand family protein | chr3:6326186-6327482 FORWARD [21477]	
JCVI_2348	2.328	moderately similar to (256)AT4G35080| Symbols: | high-affinity nickel-transport family protein | chr4:16698389-16700017 FORWARD n	
ES933700	2.328	weakly similar to (139)AT3G11510| Symbols: | 40S ribosomal protein S14 (RPS14B) | chr3:3623763-3624872 REVERSEweakly similar t	
JCVI_2921	2.328	weakly similar to (164)AT2G43235| Symbols: | sugar porter | chr2:17975987-17977899 REVERSE no original description	
JCVI_26146	2.327	weakly similar to (170)AT1G29760| Symbols: | similar to unknown protein [Arabidopsis thaliana] (TAIR:AT2G34380.1); similar to unnar	
ES954484	2.327	no similarity	
EE533086	2.326	moderately similar to (330)AT2G41790| Symbols: | peptidase M16 family protein / insulinase family protein | chr2:17436531-17443188 R	
JCVI_15303	2.325	highly similar to (787)AT4G29840| Symbols: TS, MTO2 | MTO2 (METHIONINE OVER-ACCUMULATOR); threonine synthase | chr4:1	
JCVI_2588	2.325	moderately similar to (375)AT1G50940| Symbols: ETFALPHA | ETFALPHA (ELECTRON TRANSFER FLAVOPROTEIN ALPHA); FA	
EV167051	2.325	moderately similar to (326)AT2G38010| Symbols: | ceramidase family protein | chr2:15913940-15916945 FORWARD [21486] 88 1104 1	-1.153
JCVI_14059	2.324	moderately similar to (380)AT1G69640| Symbols: | acid phosphatase, putative | chr1:26197596-26199129 REVERSE no original descript	
DY015048	2.324	no similarity	
JCVI_16691	2.324	moderately similar to (416)AT1G52930| Symbols: | brix domain-containing protein | chr1:19714929-19716771 FORWARD no original de	
JCVI_25584	2.324	highly similar to (550)AT2G28100| Symbols: ATFUC1 | ATFUC1 (ALPHA-L-FUCOSIDASE 1); alpha-L-fucosidase | chr2:11981880-119	
EE532455	2.324	moderately similar to (283)AT5G09420| Symbols: ATTOC64-V, MTOM64 | ATTOC64-V/MTOM64 (ARABIDOPSIS THALIANA TRA	
JCVI_16255	2.323	weakly similar to (157)AT3G03470| Symbols: CYP89A9 | CYP89A9 (cytochrome P450, family 87, subfamily A, polypeptide 9); oxygen b	
EV112954	2.323	moderately similar to (266)AT1G49050| Symbols: | aspartyl protease family protein | chr1:18154829-18156854 FORWARD [21479] 45 8	
ES932251	2.323	weakly similar to (103)AT4G12610| Symbols: RAP74, ATRAP74 | ATRAP74/RAP74; transcription initiation factor | chr4:7454967-74574	
JCVI_25077	2.323	moderately similar to (464)AT3G52850| Symbols: VSR1, BP-80, ATELP, VSR-1, BP80, BP80B, ATELP1 | ATELP/ATELP1/BP-80/BP8	-1.091
JCVI_15837	2.323	moderately similar to (365)AT5G51100| Symbols: FSD2 | FSD2 (FE SUPEROXIDE DISMUTASE 2); iron superoxide dismutase | chr5:20	
JCVI_27468	2.323	moderately similar to (324)AT5G15890| Symbols: | similar to unknown protein [Arabidopsis thaliana] (TAIR:AT5G15900.1); similar to h	
JCVI_13451	2.323	highly similar to (531)AT5G06570| Symbols: | hydrolase | chr5:2008076-2011014 REVERSEweakly similar to (131)GID1_ORYSA no or	
L35799	2.322	weakly similar to (129)AT3G11200| Symbols: | PHD finger family protein | chr3:3508393-3510201 REVERSE [132]	
JCVI_29193	2.322	moderately similar to (226)AT1G08510| Symbols: FATB | FATB (FATTY ACYL-ACP THIOESTERASES B); acyl carrier/ acyl-ACP thic	
JCVI_13078	2.322	moderately similar to (298)AT3G14850| Symbols: | similar to unknown protein [Arabidopsis thaliana] (TAIR:AT1G29050.1); similar to u	
JCVI_26048	2.322	moderately similar to (388)AT5G12010| Symbols: | similar to unknown protein [Arabidopsis thaliana] (TAIR:AT4G29780.1); similar to u	
JCVI_24824	2.322	no original description	
JCVI_2208	2.322	moderately similar to (270)AT1G75050| Symbols: | similar to ATLP-3 (Arabidopsis thaumatin-like protein 3) [Arabidopsis thaliana] (TAI	
AT002101	2.321	weakly similar to (125)AT4G16720| Symbols: | 60S ribosomal protein L15 (RPL15A) | chr4:9400178-9401337 REVERSEweakly similar t	
EX091107	2.321	weakly similar to (160)AT3G12180| Symbols: | cornichon family protein | chr3:3883471-3884577 FORWARD [21823]	-2.530
EV152128	2.321	weakly similar to (184)AT5G22020| Symbols: | strictosidine synthase family protein | chr5:7287881-7289360 REVERSE [21483]	
JCVI_18175	2.320	moderately similar to (404)AT3G56840| Symbols: | FAD-dependent oxidoreductase family protein | chr3:21055296-21057156 FORWARI	
JCVI_119	2.320	highly similar to (527)AT1G04410| Symbols: | malate dehydrogenase, cytosolic, putative | chr1:1189417-1191266 REVERSEhighly simila	
JCVI_40645	2.319	highly similar to (835)AT3G10160| Symbols: ATDFC | ATDFC (A. THALIANA DHFS-FPGS HOMOLOG C); dihydrofolate synthase | ch	
EV024079	2.319	moderately similar to (310)AT3G03860| Symbols: ATAPRL5 | ATAPRL5 (APR-LIKE 5) | chr3:992472-994322 FORWARD [21441] 17 5	
AM391280	2.319	weakly similar to (126)AT5G32440| Symbols: | similar to unknown [Populus trichocarpa] (GB:ABK93674.1); contains InterPro domain U	
JCVI_2450	2.319	highly similar to (531)AT3G57220| Symbols: | UDP-GlcNAc:dolichol phosphate N-acetylglucosamine-1-phosphate transferase, putative |	
JCVI_34081	2.319	weakly similar to (150)AT2G02060| Symbols: | transcription factor | chr2:495690-497608 FORWARD no original description	2.190
JCVI_27855	2.319	moderately similar to (316)AT4G36940| Symbols: | nicotinate phosphoribosyltransferase | chr4:17416935-17419880 FORWARD no origi	
EV080493	2.319	very weakly similar to (89.7)AT1G53580| Symbols: GLX2-3, ETHE1, GLY3 | ETHE1/GLX2-3/GLY3 (GLYOXALASE 2-3); hydroxyacyl	
JCVI_26001	2.318	moderately similar to (421)AT5G18580| Symbols: TON2, EMB40, FS1, GDO, FASS | FASS (FASS 1) | chr5:6175156-6178216 FORWAR	
EE424067	2.318	weakly similar to (163)AT5G49300| Symbols: | zinc finger (GATA type) family protein | chr5:20002075-20002701 REVERSE [20158]	
CD826421	2.318	no similarity	
JCVI_11689	2.318	moderately similar to (275)AT1G54150| Symbols: | zinc finger (C3HC4-type RING finger) family protein | chr1:20219147-20220970 FOI	
EV111901	2.318	weakly similar to (114)AT4G17170| Symbols: AtRABB1c, AtRab2A, AT-RAB2 | AT-RAB2 (Arabidopsis Rab GTPase homolog B1c); GT	
EX057954	2.318	weakly similar to (171)AT5G13640| Symbols: ATPDAT | ATPDAT (Arabidopsis thaliana phospholipid:diacylglycerol acyltransferase); ph	
JCVI_11830	2.317	moderately similar to (332)AT5G56340| Symbols: | zinc finger (C3HC4-type RING finger) family protein | chr5:22835480-22836670 FOI	
JCVI_3829	2.317	highly similar to (565)AT3G20770| Symbols: EIN3 | EIN3 (ETHYLENE-INSENSITIVE3); transcription factor | chr3:7260708-7262594 R	-1.817
JCVI_23290	2.317	moderately similar to (305)AT4G27435| Symbols: | similar to unknown protein [Arabidopsis thaliana] (TAIR:AT3G15480.1); similar to fi	
JCVI_7001	2.317	weakly similar to (170)AT2G25690| Symbols: | senescence-associated protein-related | chr2:10947608-10948727 REVERSE no original d	
JCVI_17088	2.317	moderately similar to (341)AT1G06200| Symbols: | serine-type peptidase/ signal peptidase | chr1:1894603-1896851 REVERSE no origina	
JCVI_27466	2.317	moderately similar to (448)AT3G18940| Symbols: | clast3-related | chr3:6527087-6529056 REVERSE no original description	
JCVI_8950	2.316	moderately similar to (338)AT4G02980| Symbols: ABP, ABP1 | ABP1 (ENDOPLASMIC RETICULUM AUXIN BINDING PROTEIN 1)	
EX040803	2.316	very weakly similar to (91.3)AT1G24050| Symbols: | similar to unknown protein [Arabidopsis thaliana] (TAIR:AT1G70220.1); similar to u	
JCVI_7149	2.316	highly similar to (672)AT4G28780| Symbols: | GDSL-motif lipase/hydrolase family protein | chr4:14215609-14217165 FORWARDweakl	
JCVI_16081	2.316	moderately similar to (207)AT1G77920| Symbols: | bZIP family transcription factor | chr1:29303853-29305501 FORWARD no original d	
EV184093	2.316	no similarity	-4.240
JCVI_10429	2.315	weakly similar to (120)AT2G43535| Symbols: | trypsin inhibitor, putative | chr2:18078207-18078637 FORWARDweakly similar to (116)	
EX039029	2.315	weakly similar to (117)AT1G66070| Symbols: | translation initiation factor-related | chr1:24599857-24601183 REVERSE [21811]	
JCVI_2913	2.315	moderately similar to (266)AT1G73630| Symbols: | calcium-binding protein, putative | chr1:27688409-27688900 FORWARDweakly simi	
JCVI_40097	2.315	moderately similar to (330)AT1G72370| Symbols: AP40, RP40, RPSAA, P40 | P40 (40S ribosomal protein SA); structural constituent of r	
EE460859	2.315	moderately similar to (205)AT5G63870| Symbols: PP7 | PP7 (protein phosphatase 7); protein serine/threonine phosphatase | chr5:2557856	

ES265498	2.315	moderately similar to (256)AT4G38360| Symbols: | similar to unknown protein [Arabidopsis thaliana] (TAIR:AT1G77220.1); similar to M	
EX037042	2.315	moderately similar to (290)AT5G04660| Symbols: CYP77A4 | CYP77A4 (cytochrome P450, family 77, subfamily A, polypeptide 4); oxyg	
DW998584	2.315	no similarity	
CD827507	2.314	moderately similar to (264)AT3G10350| Symbols: | anion-transporting ATPase family protein | chr3:3208315-3210683 FORWARD [1397	
JCVI_21303	2.314	moderately similar to (479)AT5G24300| Symbols: SSI, ATSS1 | ATSS1/SSI (STARCH SYNTHASE I); transferase, transferring glycosyl g	
EV211580	2.314	weakly similar to (143)AT2G41040| Symbols: | methyltransferase-related | chr2:17128577-17130142 FORWARD [21491]	
JCVI_9689	2.314	very weakly similar to (97.1)AT1G31330| Symbols: PSAF | PSAF (photosystem I subunit F) | chr1:11214992-11215920 REVERSEvery we	
JCVI_1290	2.313	moderately similar to (495)AT1G76150| Symbols: | maoC-like dehydratase domain-containing protein | chr1:28580130-28582531 REVER	
EV202673	2.313	moderately similar to (292)AT1G69690| Symbols: | TCP family transcription factor, putative | chr1:26220112-26221089 FORWARD [214	-2.559
JCVI_471	2.312	moderately similar to (268)AT3G46000| Symbols: ADF2 | ADF2 (ACTIN DEPOLYMERIZING FACTOR 2); actin binding | chr3:169187	
JCVI_10637	2.312	highly similar to (635)AT2G35610| Symbols: | similar to unknown protein [Arabidopsis thaliana] (TAIR:AT1G70630.1); similar to unnam	
CO750619	2.312	moderately similar to (216)AT3G12977| Symbols: | DNA binding | chr3:4143839-4145867 FORWARDweakly similar to (133)NAC77_O	
ES949367	2.312	moderately similar to (219)AT1G68030| Symbols: | PHD finger protein-related | chr1:25504041-25505207 REVERSE [21393]	-2.368
JCVI_37015	2.311	highly similar to (863)AT1G49340| Symbols: ATPI4K ALPHA | ATPI4K ALPHA (Arabidopsis thaliana phosphatidylinositol 4-kinase alph	
JCVI_84	2.311	moderately similar to (230)AT4G24770| Symbols: ATRBP31, CP31, ATRBP33, RBP31 | RBP31 (31-KDA RNA BINDING PROTEIN); I	
EX135252	2.311	moderately similar to (363)AT4G25070| Symbols: | similar to unknown protein [Arabidopsis thaliana] (TAIR:AT3G48860.2); similar to u	
JCVI_3398	2.311	moderately similar to (398)AT4G28390| Symbols: ATAAC3, AAC3 | AAC3 (ADP/ATP CARRIER 3); ATP:ADP antiporter/ binding | chr4	
EE467954	2.311	weakly similar to (117)AT5G49890| Symbols: ATCLC-C, CLC-C | CLC-C (chloride channel C); anion channel/ voltage-gated chloride cha	
JCVI_563	2.311	weakly similar to (191)AT4G27520| Symbols: | plastocyanin-like domain-containing protein | chr4:13750674-13751825 REVERSE no ori	
ES917670	2.310	moderately similar to (333)AT1G78550| Symbols: | oxidoreductase, 2OG-Fe(II) oxygenase family protein | chr1:29549924-29551253 REV	
EE448341	2.310	no similarity	
CX189577	2.310	moderately similar to (254)AT4G35520| Symbols: MLH3, ATMLH3 | ATMLH3/MLH3 (MUTL PROTEIN HOMOLOG 3); ATP binding	
DY015719	2.310	no similarity	
EE440420	2.310	moderately similar to (438)AT1G18900| Symbols: | pentatricopeptide (PPR) repeat-containing protein | chr1:6529769-6532351 FORWAR	
CD826338	2.309	moderately similar to (329)AT1G60470| Symbols: ATGOLS4 | ATGOLS4 (ARABIDOPSIS THALIANA GALACTINOL SYNTHASE 4);	
JCVI_7322	2.309	moderately similar to (249)AT3G26125| Symbols: CYP86C2 | CYP86C2 (cytochrome P450, family 86, subfamily C, polypeptide 2); oxyg	
CX192032	2.309	moderately similar to (300)AT4G17480| Symbols: | palmitoyl protein thioesterase family protein | chr4:9745325-9746859 REVERSE [168	
JCVI_38455	2.309	moderately similar to (325)AT4G28610| Symbols: ATPHR1, PHR1 | PHR1 (PHOSPHATE STARVATION RESPONSE 1); transcription	
ES922772	2.308	very weakly similar to (83.6)AT5G67320| Symbols: HOS15 | WD-40 repeat family protein | chr5:26874494-26878200 FORWARD [15718	
JCVI_18241	2.308	moderately similar to (423)AT5G04590| Symbols: SIR | SIR (sulfite reductase); sulfite reductase (ferredoxin) | chr5:1319405-1322299 FO	
CD832636	2.308	moderately similar to (419)AT3G18750| Symbols: ZIK5, WNK6 | WNK6 (Arabidopsis WNK kinase 6); kinase | chr3:6454313-6456836 R	
BG544702	2.308	no similarity	
JCVI_25669	2.308	weakly similar to (164)AT1G17610| Symbols: | disease resistance protein-related | chr1:6056888-6058150 FORWARD no original descrip	
JCVI_960	2.308	weakly similar to (176)AT1G22410| Symbols: | 2-dehydro-3-deoxyphosphoheptonate aldolase, putative / 3-deoxy-D-arabino-heptulosonat	
EX020133	2.308	weakly similar to (107)AT2G46450| Symbols: CNGC12, ATCNGC12 | ATCNGC12 (cyclic nucleotide gated channel 12); cyclic nucleotid	
EX138232	2.307	moderately similar to (221)AT3G29270| Symbols: | similar to zinc finger (C3HC4-type RING finger) family protein [Arabidopsis thaliana	
JCVI_183	2.307	moderately similar to (437)AT3G02090| Symbols: MPPBETA | MPPBETA; metalloendopeptidase | chr3:365631-368541 FORWARDvery	-1.993
JCVI_24237	2.307	no original description	
EV038203	2.307	weakly similar to (167)AT2G38480| Symbols: | integral membrane protein, putative | chr2:16118038-16118772 REVERSE [21441]	
EV027485	2.306	very weakly similar to (84.3)AT5G41210| Symbols: GST10, ATGSTT1 | ATGSTT1 (Arabidopsis thaliana Glutathione S-transferase (class	
JCVI_25131	2.306	no original description	
DN960858	2.306	no similarity	
JCVI_14374	2.306	moderately similar to (416)AT1G56010| Symbols: ANAC021, ANAC022, NAC1 | NAC1 (Arabidopsis NAC domain containing protein 21	
JCVI_32979	2.306	no original description	
EE547020	2.306	no similarity	
EV187159	2.306	moderately similar to (418)AT3G04760| Symbols: | pentatricopeptide (PPR) repeat-containing protein | chr3:1303890-1305698 REVERSI	
JCVI_22902	2.305	highly similar to (715)AT2G20020| Symbols: | Identical to CRS2-associated factor 1, chloroplast precursor [Arabidopsis Thaliana] (GB:C	
ES984681	2.305	moderately similar to (212)AT2G16730| Symbols: BGAL13 | BGAL13 (beta-galactosidase 13); beta-galactosidase | chr2:7269068-727318	
EX018015	2.305	weakly similar to (159)AT5G38260| Symbols: | serine/threonine protein kinase, putative | chr5:15300922-15303067 REVERSE [21809]	
JCVI_37097	2.304	moderately similar to (309)AT1G66700| Symbols: PXMT1 | PXMT1; S-adenosylmethionine-dependent methyltransferase | chr1:2487748	
JCVI_28724	2.304	moderately similar to (353)AT3G04710| Symbols: | ankyrin repeat family protein | chr3:1278235-1280948 FORWARD no original descrip	
JCVI_14741	2.304	moderately similar to (389)AT1G10340| Symbols: | ankyrin repeat family protein | chr1:3390477-3392483 REVERSE no original descript	
EE535151	2.304	no similarity	
CV432391	2.304	no similarity	
AM388906	2.304	no similarity	
EE439388	2.304	no similarity	
JCVI_2670	2.304	moderately similar to (275)AT5G48680| Symbols: | sterile alpha motif (SAM) domain-containing protein | chr5:19761951-19763279 REV	
JCVI_28881	2.303	moderately similar to (209)AT3G23950| Symbols: | F-box family protein | chr3:8652915-8654171 REVERSE no original description	
EV089098	2.303	no similarity	
JCVI_36481	2.303	moderately similar to (220)AT4G26080| Symbols: ABI1 | ABI1 (ABA INSENSITIVE 1); calcium ion binding / protein serine/threonine ph	
JCVI_19943	2.303	weakly similar to (199)AT5G63870| Symbols: PP7 | PP7 (protein phosphatase 7); protein serine/threonine phosphatase | chr5:25578562-25	
JCVI_32110	2.302	no original description	
EE482857	2.302	weakly similar to (128)AT1G10390| Symbols: | nucleoporin family protein | chr1:3407266-3412046 REVERSE [20154]	
ES960184	2.302	no similarity	
JCVI_6008	2.302	moderately similar to (397)AT5G43690| Symbols: | sulfotransferase family protein | chr5:17563500-17564495 FORWARD no original de	
EX042209	2.302	moderately similar to (426)AT4G19120| Symbols: ERD3 | ERD3 (EARLY-RESPONSIVE TO DEHYDRATION 3) | chr4:10460676-1046	-1.459
AM388966	2.302	moderately similar to (331)AT3G26170| Symbols: CYP71B19 | CYP71B19 (cytochrome P450, family 71, subfamily B, polypeptide 19); o	
CK991399	2.301	moderately similar to (222)AT4G28940| Symbols: | catalytic | chr4:14274420-14276926 FORWARD [15500] 20 407 434	-3.059
EV207492	2.300	moderately similar to (361)AT1G60160| Symbols: | potassium transporter family protein | chr1:22191995-22195060 REVERSEweakly sir	
ES948870	2.300	very weakly similar to (91.3)AT2G25605| Symbols: | similar to unnamed protein product [Vitis vinifera] (GB:CAO44153.1) | chr2:109066	
JCVI_38308	2.300	weakly similar to (164)AT1G22510| Symbols: | zinc finger (C3HC4-type RING finger) family protein | chr1:7950992-7952586 REVERSE	
EE452989	2.300	weakly similar to (181)AT1G67250| Symbols: | proteasome maturation factor UMP1 family protein | chr1:25167471-25168630 REVERSI	
DY006789	2.299	weakly similar to (180)AT1G29240| Symbols: | similar to unknown protein [Arabidopsis thaliana] (TAIR:AT2G34170.1); similar to unnar	
JCVI_4845	2.299	moderately similar to (254)AT5G10960| Symbols: | CCR4-NOT transcription complex protein, putative | chr5:3464582-3465415 FORWA	
JCVI_26687	2.299	moderately similar to (441)AT1G54115| Symbols: | cation exchanger, putative | chr1:20205786-20207720 FORWARD no original descrip	
JCVI_4078	2.299	moderately similar to (399)AT3G57890| Symbols: | tubulin-specific chaperone C-related | chr3:21449248-21452672 FORWARD no origi	-6.237
JCVI_26599	2.299	moderately similar to (427)AT4G12070| Symbols: | protein binding | chr4:7231935-7234266 FORWARD no original description	1.827
JCVI_9328	2.299	weakly similar to (144)AT5G65480| Symbols: | similar to unknown protein [Arabidopsis thaliana] (TAIR:AT4G38060.2); similar to unnar	
EV076094	2.299	weakly similar to (126)AT5G17670| Symbols: | hydrolase, acting on ester bonds | chr5:5821084-5822602 FORWARD [21443]	
EX123326	2.298	moderately similar to (258)AT5G62460| Symbols: | zinc finger (C3HC4-type RING finger) family protein | chr5:25092771-25094298 FOI	-1.173
JCVI_17	2.298	moderately similar to (418)AT1G07890| Symbols: MEE6, CS1, APX1 | APX1 (ASCORBATE PEROXIDASE 1) | chr1:2438002-2439432	

EE564103	2.298	very weakly similar to (86.7)AT4G30110| Symbols: HMA2 | HMA2 (Heavy metal ATPase 2); cadmium-transporting ATPase | chr4:14720	
JCVI_8089	2.298	highly similar to (563)AT4G39980| Symbols: DHS1 | DHS1 (3-DEOXY-D-ARABINO-HEPTULOSONATE 7-PHOSPHATE SYNTHASE	
BQ704508	2.298	no similarity	
CD833322	2.297	moderately similar to (261)AT4G23950| Symbols: | similar to unknown protein [Arabidopsis thaliana] (TAIR:AT1G22882.1); similar to h	
EE448026	2.297	weakly similar to (134)AT5G66280| Symbols: GMD1 | GMD1 (GDP-D-MANNOSE 4,6-DEHYDRATASE 1); GDP-mannose 4,6-dehydra	
JCVI_18248	2.297	moderately similar to (231)AT2G01270| Symbols: ATQSOX2 | ATQSOX2 (QUIESCIN-SULFHYDRYL OXIDASE 2); thiol-disulfide exc	
L33621	2.297	weakly similar to (101)AT2G44610| Symbols: AtRABH1b, AtRab6A, RAB6 | RAB6; GTP binding | chr2:18418854-18420959 REVERSE	
JCVI_36381	2.297	highly similar to (518)AT3G52890| Symbols: KIPK | KIPK (KCBP-INTERACTING PROTEIN KINASE); kinase | chr3:19620128-19623	
JCVI_32384	2.297	moderately similar to (322)AT2G31210| Symbols: | basic helix-loop-helix (bHLH) family protein | chr2:13303732-13305216 FORWARD	
JCVI_38844	2.296	moderately similar to (278)AT3G09690| Symbols: | hydrolase, alpha/beta fold family protein | chr3:2972361-2974597 FORWARD no orig	
EV192037	2.296	moderately similar to (402)AT5G09880| Symbols: | RNA recognition motif (RRM)-containing protein | chr5:3081647-3085180 REVERSE	
JCVI_9670	2.296	moderately similar to (409)AT3G63250| Symbols: HMT-2, ATHMT-2, HMT2 | ATHMT-2/HMT-2/HMT2 (HOMOCYSTEINE METHYL	
JCVI_3324	2.296	highly similar to (944)AT4G02290| Symbols: ATGH9B13 | ATGH9B13 (ARABIDOPSIS THALIANA GLYCOSYL HYDROLASE 9B13	
JCVI_38539	2.296	no original description	
CX195832	2.296	no similarity	
EE441548	2.295	moderately similar to (204)AT4G29220| Symbols: | phosphofructokinase family protein | chr4:14403627-14406077 REVERSE [20164]	
EE559533	2.295	very weakly similar to (97.4)AT5G10740| Symbols: | protein phosphatase 2C-related / PP2C-related | chr5:3393798-3395849 REVERSE [	
EX055398	2.295	weakly similar to (172)AT1G53500| Symbols: RHM2, MUM4 | MUM4 (MUCILAGE-MODIFIED 4); catalytic | chr1:19970825-1997290	-3.838
JCVI_24540	2.295	weakly similar to (136)AT3G06190| Symbols: ATBPM2 | ATBPM2; protein binding | chr3:1874583-1876581 REVERSE no original desc	
JCVI_35372	2.295	moderately similar to (274)AT4G36210| Symbols: | similar to unknown protein [Arabidopsis thaliana] (TAIR:AT2G18100.1); similar to u	
JCVI_11034	2.295	moderately similar to (421)AT1G15470| Symbols: | transducin family protein / WD-40 repeat family protein | chr1:5315833-5317691 FOR	
JCVI_29309	2.295	moderately similar to (286)AT5G66450| Symbols: | phosphatidic acid phosphatase-related / PAP2-related | chr5:26552204-26553177 FOR	
JCVI_35049	2.295	weakly similar to (154)AT4G34135| Symbols: UGT73B2 | UGT73B2; UDP-glycosyltransferase | chr4:16346013-16347020 REVERSE no	
EE426287	2.295	weakly similar to (182)AT1G27600| Symbols: | glycosyl transferase family 43 protein | chr1:9604070-9605868 REVERSE [20189]	
JCVI_2833	2.295	moderately similar to (328)AT4G12790| Symbols: | ATP-binding family protein | chr4:7517277-7518889 REVERSE no original descriptio	1.845
ES268544	2.294	moderately similar to (234)AT2G16630| Symbols: | proline-rich family protein | chr2:7216368-7217954 FORWARD [21032]	-2.039
JCVI_28628	2.293	no original description	
EE435993	2.293	moderately similar to (311)AT2G24280| Symbols: | serine carboxypeptidase S28 family protein | chr2:10341702-10344007 FORWARD [2	-1.193
JCVI_23556	2.293	moderately similar to (281)AT1G11270| Symbols: | F-box family protein | chr1:3785833-3786653 REVERSE no original description	
DY018930	2.293	weakly similar to (155)AT2G16900| Symbols: | similar to unknown protein [Arabidopsis thaliana] (TAIR:AT4G35110.2); similar to unkn	
JCVI_16396	2.293	highly similar to (587)AT4G02790| Symbols: | GTP-binding family protein | chr4:1247416-1249351 FORWARD no original description	
EH416725	2.292	very weakly similar to (95.1)AT3G12345| Symbols: | similar to Os06g0484500 [Oryza sativa (japonica cultivar-group)] (GB:NP_0010576	
EX047678	2.292	very weakly similar to (100)AT4G16950| Symbols: RPP5 | RPP5 (RECOGNITION OF PERONOSPORA PARASITICA 5) | chr4:953918	
EX134079	2.292	moderately similar to (482)AT2G35030| Symbols: | pentatricopeptide (PPR) repeat-containing protein | chr2:14768159-14770042 REVER	
EE482913	2.292	weakly similar to (139)AT4G22760| Symbols: | pentatricopeptide (PPR) repeat-containing protein | chr4:11958488-11962881 FORWARD	
JCVI_5347	2.291	highly similar to (786)AT4G15530| Symbols: PPDK | PPDK (PYRUVATE ORTHOPHOSPHATE DIKINASE) | chr4:8864826-8870746 F	
JCVI_4775	2.291	moderately similar to (217)AT3G27460| Symbols: | similar to unknown protein [Arabidopsis thaliana] (TAIR:AT5G40550.1); similar to u	
EV009961	2.291	very weakly similar to (86.3)AT1G75150| Symbols: | similar to unnamed protein product [Vitis vinifera] (GB:CAO61179.1) | chr1:282083	
JCVI_8151	2.291	moderately similar to (301)AT3G15090| Symbols: | oxidoreductase, zinc-binding dehydrogenase family protein | chr3:5076854-5078877 F	
EV197623	2.291	moderately similar to (357)AT5G60900| Symbols: RLK1 | RLK1 (RECEPTOR-LIKE PROTEIN KINASE 1); carbohydrate binding / kinas	
EV067184	2.290	moderately similar to (463)AT4G38190| Symbols: CSLD4, ATCSLD4 | ATCSLD4 (Cellulose synthase-like D4); cellulose synthase/ transf	
EV048449	2.290	moderately similar to (226)AT5G14790| Symbols: | binding | chr5:4784061-4785511 FORWARD [21442]	
JCVI_29080	2.290	weakly similar to (155)AT5G65380| Symbols: | ripening-responsive protein, putative | chr5:26140467-26143578 REVERSE no original de	
JCVI_23042	2.289	highly similar to (642)AT5G24150| Symbols: SQP1 | SQP1 (Squalene monooxygenase 1) | chr5:8172673-8175398 REVERSEhighly simila	
JCVI_18474	2.289	moderately similar to (430)AT5G14790| Symbols: | binding | chr5:4784061-4785511 FORWARD no original description	
JCVI_14835	2.289	moderately similar to (366)AT1G23800| Symbols: ALDH2B, ALDH2B7 | ALDH2B7 (Aldehyde dehydrogenase 2B7); 3-chloroallyl aldehy	
EE455095	2.289	moderately similar to (222)AT1G79870| Symbols: | oxidoreductase family protein | chr1:30049687-30050744 FORWARD [20178]	
EV043583	2.288	moderately similar to (266)AT2G46150| Symbols: | similar to unknown protein [Arabidopsis thaliana] (TAIR:AT3G54200.1); similar to p	
JCVI_33171	2.288	moderately similar to (390)AT4G38250| Symbols: | amino acid transporter family protein | chr4:17935527-17936837 FORWARD no orig	
EE483537	2.288	weakly similar to (154)AT1G79090| Symbols: | similar to unknown protein [Arabidopsis thaliana] (TAIR:AT3G22270.1); similar to unnar	
JCVI_24768	2.288	highly similar to (686)AT2G43870| Symbols: | polygalacturonase, putative / pectinase, putative | chr2:18173692-18175341 REVERSEmod	-1.832
JCVI_2117	2.288	weakly similar to (160)AT4G31720| Symbols: TAF10, STG1, TAFII15 | TAFII15 (SALT TOLERANCE DURING GERMINATION 1); tr	
EV020982	2.288	weakly similar to (186)AT4G09720| Symbols: AtRABG3a | AtRABG3a; GTP binding | chr4:6133402-6134956 FORWARDweakly similar	
EX136039	2.288	very weakly similar to (82.0)AT5G44720| Symbols: | molybdenum cofactor sulfurase family protein | chr5:18060313-18062502 FORWAR	
EX038636	2.287	moderately similar to (240)AT3G01650| Symbols: RGLG1 | RGLG1 (RING DOMAIN LIGASE1); protein binding / zinc ion binding | chr3	
EE454373	2.287	moderately similar to (246)AT3G61690| Symbols: | nucleotidyltransferase | chr3:22839324-22844452 FORWARD [20178]	
EX043451	2.286	weakly similar to (136)AT3G21230| Symbols: 4CL5 | 4CL5 (4-COUMARATE:COA LIGASE 5); 4-coumarate-CoA ligase | chr3:7448237-	
EE531049	2.286	moderately similar to (288)AT2G38400| Symbols: AGT3 | AGT3 (ALANINE:GLYOXYLATE AMINOTRANSFERASE 3); alanine-glyox	
JCVI_14608	2.286	highly similar to (702)AT1G76990| Symbols: ACR3 | ACR3 (ACT Domain Repeat 3) | chr1:28938281-28940073 FORWARD no original	
JCVI_3187	2.286	moderately similar to (358)AT3G57790| Symbols: | glycoside hydrolase family 28 protein / polygalacturonase (pectinase) family protein |	
CD818956	2.286	weakly similar to (183)AT3G63190| Symbols: RRF | RRF (RIBOSOME RECYCLING FACTOR, CHLOROPLAST PRECURSOR) | chr3	
EX069208	2.286	moderately similar to (217)AT3G54630| Symbols: | similar to kinetochore protein [Capsella rubella] (GB:BAF63163.1); similar to kinetoc	
JCVI_25389	2.286	moderately similar to (238)AT5G66570| Symbols: PSBO-1, OEE1, OEE33, OE33, PSBO1 | OE33/OEE1/OEE33/PSBO-1/PSBO1 (OXYG	-2.529
EV050053	2.286	moderately similar to (338)AT2G39710| Symbols: | aspartyl protease family protein | chr2:16569129-16570457 REVERSE [21442]	
EE560233	2.285	no similarity	
JCVI_24326	2.285	moderately similar to (442)AT1G73240| Symbols: | similar to unnamed protein product [Vitis vinifera] (GB:CAO69427.1) | chr1:2754605	
EV049769	2.285	moderately similar to (310)AT5G55560| Symbols: | protein kinase family protein | chr5:22523703-22524983 REVERSE [21442]	
JCVI_3951	2.285	moderately similar to (381)AT5G67630| Symbols: | DNA helicase, putative | chr5:26984761-26986532 REVERSE no original description	
JCVI_25232	2.285	moderately similar to (389)AT5G13030| Symbols: | similar to hypothetical protein OsI_021963 [Oryza sativa (indica cultivar-group)] (GB	
CN829660	2.285	weakly similar to (158)AT1G47310| Symbols: | similar to hypothetical protein [Oryza sativa (japonica cultivar-group)] (GB:AAP50957.1)	-3.681
EV076669	2.285	very weakly similar to (96.7)AT1G60995| Symbols: | similar to S3 self-incompatibility locus-linked pollen 3.15 protein [Petunia integrifoli	-6.392
JCVI_1276	2.284	moderately similar to (466)AT1G47250| Symbols: PAF2 | PAF2 (20S proteasome alpha subunit F2); peptidase | chr1:17321660-17323340	
ES268822	2.284	very weakly similar to (95.5)AT3G24515| Symbols: UBC37 | UBC37 (UBIQUITIN-CONJUGATING ENZYME 37); ubiquitin-protein liga	
EV182211	2.284	weakly similar to (125)AT1G71380| Symbols: ATGH9B3, ATCEL3 | ATCEL3/ATGH9B3 (ARABIDOPSIS THALIANA GLYCOSYL HY	
JCVI_20310	2.284	weakly similar to (150)AT5G02580| Symbols: | similar to unknown protein [Arabidopsis thaliana] (TAIR:AT3G55240.1); similar to hypot	
JCVI_4803	2.284	no original description	
JCVI_12921	2.283	moderately similar to (245)AT1G18340| Symbols: | basal transcription factor complex subunit-related | chr1:6311605-6313727 REVERSE	
EE533233	2.283	moderately similar to (209)AT2G43820| Symbols: GT, UGT74F2 | GT/UGT74F2 (UDP-GLUCOSYLTRANSFERASE 74F2); UDP-gluco	
RC_EL586758	2.283	no similarity	
JCVI_10716	2.282	moderately similar to (326)AT3G02540| Symbols: RAD23-3 | RAD23-3 (PUTATIVE DNA REPAIR PROTEIN RAD23-3) | chr3:533913-	
EE534551	2.282	no similarity	

JCVI_8985	2.281	moderately similar to (318)AT2G03050| Symbols: | mitochondrial transcription termination factor-related / mTERF-related | chr2:900093-	
JCVI_648	2.281	moderately similar to (398)AT4G05530| Symbols: | short-chain dehydrogenase/reductase (SDR) family protein | chr4:2816459-2818071 F	-3.336
JCVI_11567	2.281	highly similar to (701)AT5G23630| Symbols: | (MALE GAMETOGENESIS IMPAIRED ANTHERS); cation-transporting ATPase | chr5:	
EE546653	2.281	moderately similar to (342)AT5G48300| Symbols: APS1, ADG1 | ADG1 (ADP GLUCOSE PYROPHOSPHORYLASE SMALL SUBUNIT	
EV155363	2.281	weakly similar to (193)AT5G61790| Symbols: | calnexin 1 (CNX1) | chr5:24844620-24846868 REVERSEweakly similar to (172)CALX_	
JCVI_22403	2.281	moderately similar to (302)AT2G46970| Symbols: PIL1 | PIL1 (PHYTOCHROME INTERACTING FACTOR 3-LIKE 1); transcription fa	
JCVI_28552	2.280	no original description	
JCVI_18451	2.280	highly similar to (867)AT1G53310| Symbols: ATPPC1 | ATPPC1 (PHOSPHOENOLPYRUVATE CARBOXYLASE 1); phosphoenolpyru	
EV070919	2.280	weakly similar to (108)AT1G10270| Symbols: GRP23 | GRP23 (GLUTAMINE-RICH PROTEIN23); binding | chr1:3363537-3366278 FO	
EE556987	2.280	weakly similar to (151)AT5G51440| Symbols: | 23.5 kDa mitochondrial small heat shock protein (HSP23.5-M) | chr5:20908468-2090923	
JCVI_4799	2.280	moderately similar to (335)AT2G37570| Symbols: SLT1 | SLT1 (SODIUM- AND LITHIUM-TOLERANT 1) | chr2:15768544-15770028 I	
EE448174	2.280	weakly similar to (165)AT5G21280| Symbols: | hydroxyproline-rich glycoprotein family protein | chr5:7264097-7265517 REVERSE [201	
JCVI_9053	2.280	moderately similar to (468)AT2G15480| Symbols: UGT73B5 | UGT73B5 (UDP-GLUCOSYL TRANSFERASE 73B5); UDP-glycosyltrans	
EE449787	2.279	no similarity	
JCVI_18308	2.279	moderately similar to (296)AT2G01620| Symbols: MEE11 | MEE11 (maternal effect embryo arrest 11) | chr2:278203-279225 FORWARD	
JCVI_42429	2.279	moderately similar to (474)AT3G20920| Symbols: | translocation protein-related | chr3:7328769-7330704 REVERSE no original descripti	
JCVI_22299	2.279	moderately similar to (413)AT5G35630| Symbols: GLN2, ATGSL1, GS2 | GS2 (GLUTAMINE SYNTHETASE 2) | chr5:13848450-13850	
JCVI_23780	2.279	no original description	
ES267526	2.279	weakly similar to (169)AT3G19950| Symbols: | zinc finger (C3HC4-type RING finger) family protein | chr3:6942859-6943845 FORWAR	
ES903134	2.279	moderately similar to (402)AT5G12940| Symbols: | leucine-rich repeat family protein | chr5:4087785-4088900 FORWARDweakly similar	
JCVI_36228	2.279	weakly similar to (186)AT3G53850| Symbols: | Identical to UPF0497 membrane protein At3g53850 [Arabidopsis Thaliana] (GB:Q945M	
JCVI_26823	2.278	very weakly similar to (98.2)ATMG01275| Symbols: NAD1A, ND1 | Encodes subunit of mitochondrial NAD(P)H dehydrogenase that is tra	
JCVI_17698	2.278	weakly similar to (177)AT4G22750| Symbols: | zinc finger (DHHC type) family protein | chr4:11949377-11951346 REVERSE no origina	
JCVI_13532	2.278	moderately similar to (478)AT5G61530| Symbols: | small G protein family protein / RhoGAP family protein | chr5:24759856-24761812 F	
JCVI_34323	2.278	weakly similar to (189)AT1G22690| Symbols: | gibberellin-responsive protein, putative | chr1:8027317-8027950 FORWARD no original c	-1.723
EE420303	2.278	weakly similar to (190)AT5G25190| Symbols: | ethylene-responsive element-binding protein, putative | chr5:8707010-8707658 REVERSE	
JCVI_37489	2.278	moderately similar to (423)AT5G35160| Symbols: | endomembrane protein 70, putative | chr5:13432175-13434151 FORWARD no origin	
CX195151	2.278	moderately similar to (423)AT2G22475| Symbols: GEM | GEM | chr2:9548603-9551858 FORWARD [16807]	
JCVI_35677	2.278	weakly similar to (123)AT4G25100| Symbols: FSD1 | FSD1 (FE SUPEROXIDE DISMUTASE 1); iron superoxide dismutase | chr4:12884	
JCVI_6849	2.278	highly similar to (640)AT3G50790| Symbols: | late embryogenesis abundant protein, putative / LEA protein, putative | chr3:18891055-188	
EX096583	2.278	weakly similar to (141)AT3G04880| Symbols: DRT102 | DRT102 (DNA-DAMAGE-REPAIR/TOLERATION 2) | chr3:1346437-1347369	
EE409593	2.278	weakly similar to (119)AT1G55365| Symbols: | similar to unknown protein [Arabidopsis thaliana] (TAIR:AT5G56520.1) | chr1:20677649	2.691
EE471565	2.278	no similarity	
EE529543	2.278	weakly similar to (159)AT1G15430| Symbols: | similar to zinc ion binding [Arabidopsis thaliana] (TAIR:AT1G80220.1); similar to Os01g	
JCVI_39944	2.277	moderately similar to (202)AT4G37210| Symbols: | tetratricopeptide repeat (TPR)-containing protein | chr4:17512370-17514415 FORWA	
JCVI_35804	2.277	no original description	
AT002103	2.277	no similarity	
JCVI_6404	2.277	moderately similar to (461)AT2G02180| Symbols: TOM3 | TOM3 (tobamovirus multiplication protein 3) | chr2:560975-562960 FORWAR	
JCVI_20601	2.277	very weakly similar to (94.4)AT2G33410| Symbols: | heterogeneous nuclear ribonucleoprotein, putative / hnRNP, putative | chr2:14163161	
EV149149	2.277	no similarity	
JCVI_22153	2.277	highly similar to (624)AT5G63850| Symbols: AAP4 | AAP4 (amino acid permease 4); amino acid transmembrane transporter | chr5:25568	
DY001519	2.277	moderately similar to (293)AT5G16710| Symbols: DHAR3 | DHAR3 (DEHYDROASCORBATE REDUCTASE 1); glutathione dehydroge	
JCVI_42324	2.277	highly similar to (561)AT5G15810| Symbols: | N2,N2-dimethylguanosine tRNA methyltransferase family protein | chr5:5157801-5161124	
JCVI_13647	2.276	moderately similar to (286)AT5G24090| Symbols: | acidic endochitinase (CHIB1) | chr5:8143808-8145156 REVERSEmoderately similar	
JCVI_2566	2.276	no original description	
EV120738	2.276	moderately similar to (318)AT1G77990| Symbols: SULTR2;2, AST56 | AST56 (sulphate transporter 2;2); sulfate transmembrane transpor	
EX131375	2.276	weakly similar to (101)AT2G03800| Symbols: GEK1 | GEK1 (GEKO1) | chr2:1156779-1158692 FORWARD [21833]	
AM059869	2.276	moderately similar to (266)AT4G13870| Symbols: WRNEXO, ATWRNEXO, WEX, ATWEX | WRNEXO (WERNER SYNDROME-LIKE	
JCVI_13269	2.276	no original description	
EE555983	2.276	weakly similar to (130)AT5G65560| Symbols: | pentatricopeptide (PPR) repeat-containing protein | chr5:26218238-26220985 REVERSE	
JCVI_39250	2.275	moderately similar to (496)AT5G36880| Symbols: | acetyl-CoA synthetase, putative / acetate-CoA ligase, putative | chr5:14552191-14557	
JCVI_38709	2.275	moderately similar to (317)AT1G52200| Symbols: | similar to unknown protein [Arabidopsis thaliana] (TAIR:AT3G18470.1); similar to u	
ES978074	2.275	no similarity	
JCVI_27411	2.275	weakly similar to (186)AT1G51600| Symbols: TIFY2A, ZML2 | ZML2 (ZIM-LIKE 2); transcription factor | chr1:19136844-19138920 FO	
JCVI_17749	2.274	moderately similar to (338)AT2G46930| Symbols: | pectinacetylesterase, putative | chr2:19290694-19293159 FORWARD no original desc	
JCVI_18260	2.274	moderately similar to (286)AT5G07090| Symbols: | 40S ribosomal protein S4 (RPS4B) | chr5:2202784-2203806 FORWARDmoderately s	
JCVI_30112	2.274	highly similar to (598)AT1G73110| Symbols: | ribulose bisphosphate carboxylase/oxygenase activase, putative / RuBisCO activase, putati	
JCVI_23869	2.274	moderately similar to (379)AT4G24840| Symbols: | similar to unnamed protein product [Vitis vinifera] (GB:CAO68282.1); similar to hyp	
EE479752	2.274	very weakly similar to (82.4)AT1G54320| Symbols: | LEM3 (ligand-effect modulator 3) family protein / CDC50 family protein | chr1:2027	
JCVI_1619	2.274	moderately similar to (375)AT2G21170| Symbols: TIM | TIM (TRIOSEPHOSPHATE ISOMERASE) | chr2:9078128-9080187 REVERSE	1.663
EX047533	2.274	moderately similar to (293)AT4G30560| Symbols: CNGC9, ATCNGC9 | ATCNGC9 (CYCLIC NUCLEOTIDE GATED CHANNEL 9); c	
ES939680	2.273	moderately similar to (302)AT1G57820| Symbols: VIM1, ORTH2 | ORTH2/VIM1 (VARIANT IN METHYLATION 1); DNA binding | ch	
JCVI_12771	2.273	weakly similar to (128)AT1G04930| Symbols: | hydroxyproline-rich glycoprotein family protein | chr1:1396545-1398987 REVERSE no or	
JCVI_30826	2.273	highly similar to (644)AT4G35350| Symbols: XCP1 | XCP1 (XYLEM CYSTEINE PEPTIDASE 1); cysteine-type peptidase | chr4:168105	
EX090024	2.273	weakly similar to (140)AT5G50960| Symbols: | nucleotide-binding family protein | chr5:20751493-20753050 FORWARD [21823]	
ES918410	2.273	moderately similar to (291)AT5G19910| Symbols: | SOH1 family protein | chr5:6731486-6732823 REVERSE [15718]	
RC_JCVI_38423	2.273	no original description	
JCVI_24912	2.273	moderately similar to (382)AT1G05520| Symbols: | transport protein, putative | chr1:1631125-1635702 REVERSE no original description	
JCVI_37856	2.273	moderately similar to (298)AT5G40510| Symbols: | similar to unknown protein [Arabidopsis thaliana] (TAIR:AT3G27570.1); similar to u	
JCVI_28548	2.273	moderately similar to (473)AT3G53030| Symbols: SRPK4 | SRPK4 (SER/ARG-RICH PROTEIN KINASE 4); kinase/ protein kinase | chr3	-3.743
JCVI_662	2.272	moderately similar to (415)AT2G44350| Symbols: CSY4, ATCS | ATCS (CITRATE SYNTHASE 4); citrate (SI)-synthase | chr2:1832374	
DY024653	2.272	moderately similar to (310)AT1G77770| Symbols: | protein binding / zinc ion binding | chr1:29251375-29252169 REVERSE [18971]	1.212
JCVI_1553	2.271	moderately similar to (422)AT2G17420| Symbols: ATNTRA, NTR2, NTRA | NTRA (NADPH-dependent thioredoxin reductase 2) | chr2:	
JCVI_27689	2.271	moderately similar to (435)AT1G14140| Symbols: | mitochondrial substrate carrier family protein | chr1:4838128-4839599 REVERSE no	
JCVI_25595	2.271	weakly similar to (180)AT3G14990| Symbols: | 4-methyl-5(b-hydroxyethyl)-thiazole monophosphate biosynthesis protein, putative | chr3:	
EE521576	2.271	moderately similar to (283)AT1G06140| Symbols: | pentatricopeptide (PPR) repeat-containing protein | chr1:1864795-1866471 FORWAR	
EE553181	2.271	weakly similar to (184)AT2G41980| Symbols: | seven in absentia (SINA) family protein | chr2:17530572-17531770 REVERSE [20184]	
JCVI_20637	2.271	no original description	
EV139347	2.270	weakly similar to (174)AT2G46040| Symbols: | ARID/BRIGHT DNA-binding domain-containing protein / ELM2 domain-containing prot	
EX118493	2.270	weakly similar to (161)AT2G01320| Symbols: | ABC transporter family protein | chr2:154668-158062 REVERSE [21828]	
JCVI_9339	2.270	moderately similar to (368)AT2G35040| Symbols: | AICARFT/IMPCHase bienzyme family protein | chr2:14772426-14775348 REVERSE	

ES942291	2.270	moderately similar to (248)AT2G27150| Symbols: AAO3 | AAO3 (ABSCISIC ALDEHYDE OXIDASE 3) | chr2:11609030-11614092 FOI	
JCVI_23221	2.269	moderately similar to (355)AT2G40130| Symbols: | heat shock protein-related | chr2:16773108-16776152 FORWARD no original descrip	
JCVI_36650	2.269	highly similar to (695)AT1G07720| Symbols: | beta-ketoacyl-CoA synthase family protein | chr1:2390967-2392403 REVERSE no original	
JCVI_30251	2.268	weakly similar to (107)AT4G19500| Symbols: | disease resistance protein (TIR-NBS-LRR class), putative | chr4:10625798-10630150 FOI	
EV129286	2.268	no similarity	
EV152963	2.268	weakly similar to (191)AT5G45510| Symbols: | leucine-rich repeat family protein | chr5:18462025-18466179 FORWARD [21484] 97 106	
EX043503	2.266	no similarity	
JCVI_33777	2.266	highly similar to (586)AT5G05660| Symbols: | transcription factor | chr5:1691114-1695465 REVERSE no original description	
JCVI_22173	2.266	moderately similar to (307)AT3G21740| Symbols: APO4 | APO4 (ACCUMULATION OF PHOTOSYSTEM ONE 4) | chr3:7662549-7663	
JCVI_7493	2.265	highly similar to (566)AT1G76140| Symbols: | similar to prolyl oligopeptidase, putative / prolyl endopeptidase, putative / post-proline clea	
EV085813	2.265	weakly similar to (139)AT3G55250| Symbols: | similar to unnamed protein product [Vitis vinifera] (GB:CAO14780.1) | chr3:20490279-2(	
EE430543	2.265	moderately similar to (374)AT1G10950| Symbols: | endomembrane protein 70, putative | chr1:3659322-3663622 FORWARD [20136] 18	
EX117019	2.265	moderately similar to (245)AT3G03480| Symbols: CHAT | CHAT (ACETYL COA:(Z)-3-HEXEN-1-OL ACETYLTRANSFERASE); acet	
JCVI_27383	2.264	moderately similar to (252)AT3G11090| Symbols: LBD21 | LBD21 (LOB DOMAIN-CONTAINING PROTEIN 21) | chr3:3475042-34755	
EX125835	2.264	moderately similar to (474)AT2G37650| Symbols: | scarecrow-like transcription factor 9 (SCL9) | chr2:15799701-15801857 FORWARDv	
JCVI_30902	2.264	highly similar to (503)AT5G05600| Symbols: | oxidoreductase, 2OG-Fe(II) oxygenase family protein | chr5:1672267-1674603 FORWARI	
EV164211	2.264	no similarity	
EE534175	2.263	no similarity	
JCVI_26136	2.263	moderately similar to (464)AT2G02080| Symbols: ATIDD4 | ATIDD4 (ARABIDOPSIS THALIANA INDETERMINATE(ID)-DOMAIN	
EX118978	2.263	no similarity	
JCVI_12980	2.263	moderately similar to (327)AT2G19410| Symbols: | protein kinase family protein | chr2:8411983-8416094 REVERSEvery weakly similar	-4.217
EE423439	2.262	very weakly similar to (93.6)AT1G71140| Symbols: | MATE efflux family protein | chr1:26828424-26830410 FORWARD [20146]	
EV100888	2.262	weakly similar to (152)AT5G46750| Symbols: AGD8 | AGD8 (ARF-GAP DOMAIN 8); DNA binding | chr5:18987177-18989044 REVER	
JCVI_14955	2.262	highly similar to (602)AT2G36250| Symbols: ATFTSZ2-1, FTSZ2-1 | FTSZ2-1 (FtsZ homolog 2-1); structural molecule | chr2:15204740-1	
JCVI_1481	2.262	moderately similar to (275)AT4G26780| Symbols: AR192 | AR192; adenyl-nucleotide exchange factor/ chaperone binding / protein bindin	
DY027996	2.262	no similarity	
CX267652	2.262	moderately similar to (224)AT3G09230| Symbols: ATMYB1 | ATMYB1 (MYB DOMAIN PROTEIN 1); DNA binding / transcription fact	
JCVI_8295	2.262	moderately similar to (259)AT1G44000| Symbols: | similar to unknown protein [Arabidopsis thaliana] (TAIR:AT4G11911.1); similar to h	
EE520866	2.261	moderately similar to (231)AT5G63120| Symbols: | ethylene-responsive DEAD box RNA helicase, putative (RH30) | chr5:25337024-2533	
DN961478	2.261	weakly similar to (181)AT1G13470| Symbols: | similar to unknown protein [Arabidopsis thaliana] (TAIR:AT1G13520.1); similar to unnar	-2.508
JCVI_13611	2.260	moderately similar to (460)AT5G16070| Symbols: | chaperonin, putative | chr5:5247552-5251053 REVERSEweakly similar to (127)TCP	
EE519484	2.260	moderately similar to (244)AT5G63870| Symbols: PP7 | PP7 (protein phosphatase 7); protein serine/threonine phosphatase | chr5:2557856	
JCVI_19210	2.260	highly similar to (527)AT2G03620| Symbols: | magnesium transporter CorA-like family protein (MRS2-5) | chr2:1100486-1102165 REVI	
JCVI_29260	2.260	weakly similar to (122)AT5G17690| Symbols: LHP1, TFL2 | TFL2 (TERMINAL FLOWER 2) | chr5:5827506-5829539 REVERSEvery we	-1.236
JCVI_35953	2.260	moderately similar to (218)AT5G22820| Symbols: | binding | chr5:7624116-7626946 REVERSE no original description	3.194
JCVI_40177	2.260	no original description	
JCVI_25230	2.259	highly similar to (1000)AT3G07160| Symbols: GSL10, ATGSL10 | ATGSL10 (GLUCAN SYNTHASE-LIKE 10); 1,3-beta-glucan synthase	
JCVI_12721	2.259	moderately similar to (251)AT5G67590| Symbols: FRO1 | FRO1 (FROSTBITE1) | chr5:26975299-26976582 FORWARD no original desc	
AM390102	2.259	weakly similar to (120)AT2G14850| Symbols: | similar to unknown protein [Arabidopsis thaliana] (TAIR:AT4G33890.2); similar to unkn	
BQ704543	2.259	no similarity	
JCVI_17996	2.259	moderately similar to (391)AT2G41790| Symbols: | peptidase M16 family protein / insulinase family protein | chr2:17436531-17443188 R	
JCVI_6138	2.259	moderately similar to (209)AT1G75380| Symbols: | wound-responsive protein-related | chr1:28285450-28287292 REVERSE no original d	
H07418	2.259	no similarity	
JCVI_11777	2.259	moderately similar to (361)AT3G59030| Symbols: TT12 | TT12 (TRANSPARENT TESTA 12); antiporter/ solute:hydrogen antiporter/ tra	
DN965915	2.259	moderately similar to (310)AT2G16950| Symbols: | protein transporter | chr2:7361021-7367719 FORWARD [17359]	
JCVI_9237	2.258	moderately similar to (233)AT1G03350| Symbols: | BSD domain-containing protein | chr1:822834-824246 REVERSE no original descript	
EH427320	2.258	moderately similar to (362)AT3G61320| Symbols: | Identical to UPF0187 protein At3g61320, chloroplast precursor [Arabidopsis Thaliana	
JCVI_27760	2.257	highly similar to (553)AT4G15560| Symbols: DEF, CLA, DXS, DXPS2, CLA1 | CLA1 (CLOROPLASTOS ALTERADOS 1) | chr4:88842	
JCVI_38285	2.257	moderately similar to (337)AT4G21910| Symbols: | MATE efflux family protein | chr4:11625833-11630976 REVERSE no original descri	
JCVI_34674	2.257	moderately similar to (217)AT3G62750| Symbols: | hydrolase, hydrolyzing O-glycosyl compounds | chr3:23225351-23227876 FORWARI	
JCVI_25334	2.257	moderately similar to (256)AT1G80510| Symbols: | amino acid transporter family protein | chr1:30277992-30279461 FORWARD no orig	
JCVI_4786	2.257	weakly similar to (118)AT4G33110| Symbols: | coclaurine N-methyltransferase, putative | chr4:15972500-15974534 REVERSE no origina	
JCVI_22468	2.257	moderately similar to (307)AT1G68570| Symbols: | proton-dependent oligopeptide transport (POT) family protein | chr1:25750474-25753	
CN732606	2.257	very weakly similar to (81.3)AT4G26860| Symbols: | alanine racemase family protein | chr4:13503297-13504699 REVERSE [15714]	
EL592104	2.257	no similarity	
JCVI_8205	2.256	moderately similar to (339)AT3G19990| Symbols: | binding | chr3:6965677-6967108 FORWARD no original description	
EV029370	2.256	weakly similar to (153)AT1G33490| Symbols: | similar to unknown protein [Arabidopsis thaliana] (TAIR:AT4G10140.1); similar to unnar	1.809
ES264702	2.256	moderately similar to (432)AT4G37680| Symbols: HHP4 | HHP4 (heptahelical protein 4); receptor | chr4:17701225-17702562 FORWARI	
JCVI_2030	2.256	moderately similar to (383)AT2G30100| Symbols: | ubiquitin family protein | chr2:12854905-12858985 FORWARD no original descriptic	
JCVI_3296	2.256	weakly similar to (199)AT4G02450| Symbols: | glycine-rich protein | chr4:1073987-1075765 REVERSE no original description	
JCVI_22769	2.255	moderately similar to (500)AT3G21690| Symbols: | MATE efflux family protein | chr3:7638757-7641868 FORWARD no original descrip	
AM389939	2.255	moderately similar to (330)AT5G08650| Symbols: | GTP-binding protein LepA, putative | chr5:2806534-2813221 REVERSE [20118]	
EE565903	2.255	no similarity	
EX089406	2.255	no similarity	
JCVI_29936	2.255	weakly similar to (128)AT4G23130| Symbols: RLK6, CRK5 | CRK5 (CYSTEINE-RICH RLK5) | chr4:12117699-12120145 REVERSE no	
JCVI_3578	2.255	highly similar to (762)AT3G46740| Symbols: TOC75-III | TOC75-III (translocon outer membrane complex 75-III); P-P-bond-hydrolysis-d	
JCVI_23771	2.254	moderately similar to (402)AT5G13630| Symbols: CCH, CHLH, CCH1, GUN5 | GUN5 (GENOMES UNCOUPLED 5) | chr5:4387923-43	
JCVI_35940	2.254	moderately similar to (453)AT4G32180| Symbols: ATPANK2 | ATPANK2 (PANTOTHENATE KINASE 2) | chr4:15538346-15543721 R	
EE410380	2.254	no similarity	
JCVI_33739	2.254	weakly similar to (161)AT1G13460| Symbols: | serine/threonine protein phosphatase 2A (PP2A) regulatory subunit B', putative | chr1:461	
JCVI_17772	2.254	moderately similar to (311)AT2G45980| Symbols: | similar to unknown protein [Arabidopsis thaliana] (TAIR:AT4G00355.2); similar to u	1.633
JCVI_23199	2.254	moderately similar to (295)AT2G21240| Symbols: BPC4, BBR/BPC4, ATBPC4 | ATBPC4/BBR/BPC4/BPC4 (BASIC PENTACYSTEIN	
ES907958	2.253	moderately similar to (327)AT1G67570| Symbols: | similar to unknown protein [Arabidopsis thaliana] (TAIR:AT1G50630.1); similar to u	
CV434041	2.253	weakly similar to (140)AT1G72280| Symbols: AERO1 | AERO1 (ARABIDOPSIS ENDOPLASMIC RETICULUM OXIDOREDUCTINS	
JCVI_40047	2.253	weakly similar to (134)AT3G58140| Symbols: | phenylalanyl-tRNA synthetase class IIc family protein | chr3:21540965-21543363 REVEF	
JCVI_25541	2.253	nearly identical (1014)AT5G50320| Symbols: ELO3, HAG3, HAC8 | ELO3/HAC8/HAG3 (ELONGATA 3); N-acetyltransferase/ catalytic/	
BQ790929	2.253	weakly similar to (138)AT1G74120| Symbols: | mitochondrial transcription termination factor-related / mTERF-related | chr1:27875584-2	
JCVI_812	2.253	moderately similar to (449)AT3G51840| Symbols: ATSCX, ATG6, ACX4 | ACX4 (ACYL-COA OXIDASE 4); oxidoreductase | chr3:1923	
JCVI_5461	2.253	moderately similar to (393)AT5G08100| Symbols: | L-asparaginase / L-asparagine amidohydrolase | chr5:2593243-2594587 REVERSEmo	
JCVI_31243	2.252	no original description	1.925
JCVI_18392	2.252	no original description	

EX040748	2.252	weakly similar to (198)AT1G57790| Symbols: | F-box family protein | chr1:21408243-21409301 REVERSE [21811]	
EV124985	2.252	moderately similar to (253)AT5G39460| Symbols: | F-box family protein | chr5:15805517-15807232 REVERSE [21479]	
JCVI_8340	2.252	highly similar to (632)AT3G19590| Symbols: | WD-40 repeat family protein / mitotic checkpoint protein, putative | chr3:6805804-680838	
JCVI_49	2.252	highly similar to (541)AT5G17000| Symbols: | NADP-dependent oxidoreductase, putative | chr5:5584986-5586994 REVERSE no original	
JCVI_5906	2.252	moderately similar to (353)AT5G58020| Symbols: | similar to predicted protein [Physcomitrella patens subsp. patens] (GB:EDQ76028.1);	
JCVI_26437	2.252	moderately similar to (341)AT1G14130| Symbols: | 2-oxoglutarate-dependent dioxygenase, putative | chr1:4836038-4837037 REVERSEv	
CD816018	2.252	weakly similar to (167)AT1G67970| Symbols: HSFA8, AT-HSFA8 | AT-HSFA8 (Arabidopsis thaliana heat shock transcription factor A8);	
JCVI_20056	2.251	moderately similar to (382)AT3G62030| Symbols: ROC4 | ROC4 (ROTAMASE CYP 4); peptidyl-prolyl cis-trans isomerase | chr3:229846	
JCVI_38919	2.251	no original description	
JCVI_15111	2.251	highly similar to (540)AT3G62360| Symbols: | carbohydrate binding | chr3:23083995-23091430 REVERSE no original description	
JCVI_11459	2.251	moderately similar to (487)AT5G57740| Symbols: XBAT32 | XBAT32 (XB3 ortholog 2 in Arabidopsis thaliana 32); protein binding / zinc	
EV197905	2.251	no similarity	
JCVI_14821	2.250	highly similar to (504)AT4G16143| Symbols: | importin alpha-2, putative (IMPA-2) | chr4:9134736-9137147 REVERSEmoderately simila	
EX126494	2.250	weakly similar to (152)AT1G66700| Symbols: PXMT1 | PXMT1; S-adenosylmethionine-dependent methyltransferase | chr1:24877489-24	
JCVI_6255	2.250	moderately similar to (365)AT1G75900| Symbols: | family II extracellular lipase 3 (EXL3) | chr1:28502840-28504604 FORWARDweakly	
JCVI_23517	2.250	weakly similar to (104)AT1G10585| Symbols: | transcription factor | chr1:3494116-3495105 REVERSE no original description	-2.730
EE417983	2.250	weakly similar to (199)AT1G75710| Symbols: | zinc finger (C2H2 type) family protein | chr1:28432467-28434789 FORWARD [20146]	-1.644
JCVI_12574	2.249	moderately similar to (344)AT5G06620| Symbols: | similar to ASHR2 (ASH1-RELATED PROTEIN 2) [Arabidopsis thaliana] (TAIR:AT	
DN965268	2.249	moderately similar to (253)AT5G49460| Symbols: ACLB-2 | ACLB-2 (ATP-citrate lyase B-2) | chr5:20072274-20075421 FORWARD [17	
JCVI_8035	2.249	moderately similar to (490)AT1G11800| Symbols: | endonuclease/exonuclease/phosphatase family protein | chr1:3985519-3987262 REVE	
JCVI_14169	2.249	moderately similar to (245)AT4G39980| Symbols: DHS1 | DHS1 (3-DEOXY-D-ARABINO-HEPTULOSONATE 7-PHOSPHATE SYNTH	
JCVI_9409	2.249	moderately similar to (325)AT5G08300| Symbols: | succinyl-CoA ligase (GDP-forming) alpha-chain, mitochondrial, putative / succinyl-C	1.671
JCVI_38696	2.248	highly similar to (572)AT5G13800| Symbols: | hydrolase, alpha/beta fold family protein | chr5:4452066-4454144 REVERSE no original d	
EE526961	2.248	weakly similar to (194)AT3G02460| Symbols: | plant adhesion molecule, putative | chr3:506117-508099 FORWARD [20143]	
JCVI_14757	2.248	moderately similar to (334)AT3G08890| Symbols: | similar to unknown protein [Arabidopsis thaliana] (TAIR:AT5G37070.1); similar to h	
JCVI_37253	2.248	weakly similar to (181)AT1G45050| Symbols: UBC15, ATUBC2-1 | ATUBC2-1 (ARABIDOPSIS THALIANA UBIQUITIN-CONJUGAT	1.359
EX131101	2.248	highly similar to (501)AT5G01400| Symbols: ESP4 | ESP4 (ENHANCED SILENCING PHENOTYPE 4); binding | chr5:162802-171071 F	
RC_T18349	2.248	no similarity	
AM059719	2.247	no similarity	
EV078256	2.247	moderately similar to (413)AT5G18490| Symbols: | similar to unknown protein [Arabidopsis thaliana] (TAIR:AT3G04350.1); similar to h	
JCVI_36602	2.247	moderately similar to (445)AT3G51530| Symbols: | F-box family protein | chr3:19123852-19125476 REVERSE no original description	
JCVI_11163	2.247	moderately similar to (410)AT1G76570| Symbols: | chlorophyll A-B binding family protein | chr1:28734026-28735648 FORWARDweakl	
JCVI_17581	2.247	moderately similar to (434)AT4G37910| Symbols: MTHSC70-1 | MTHSC70-1 (mitochondrial heat shock protein 70-1); ATP binding / unf	
JCVI_16551	2.247	highly similar to (590)AT5G06830| Symbols: | Identical to CDK5RAP3-like protein [Arabidopsis Thaliana] (GB:Q9FG23); similar to unn	
JCVI_5287	2.246	moderately similar to (374)AT1G69640| Symbols: | acid phosphatase, putative | chr1:26197596-26199129 REVERSE no original descript	
JCVI_25451	2.246	weakly similar to (102)AT2G02390| Symbols: GST18, ATGSTZ1 | ATGSTZ1 (GLUTATHIONE S-TRANSFERASE 18); glutathione tran	
ES906697	2.245	moderately similar to (238)AT1G79680| Symbols: | wall-associated kinase, putative | chr1:29985081-29987642 REVERSE [21429]	
BG543222	2.245	no similarity	
JCVI_37208	2.245	highly similar to (560)AT1G06950| Symbols: ATTIC110, TIC110 | ATTIC110/TIC110 (TRANSLOCON AT THE INNER ENVELOPE M	
JCVI_21550	2.245	moderately similar to (312)AT5G03170| Symbols: FLA11 | FLA11 (fasciclin-like arabinogalactan-protein 11) | chr5:752897-753637 REVI	
ES944030	2.245	moderately similar to (320)AT1G17160| Symbols: | pfkB-type carbohydrate kinase family protein | chr1:5867671-5869168 FORWARD [2	
JCVI_35858	2.244	weakly similar to (155)AT1G18360| Symbols: | hydrolase, alpha/beta fold family protein | chr1:6316989-6319197 REVERSE no original c	
EE478856	2.244	very weakly similar to (84.7)AT4G37608| Symbols: | similar to hypothetical protein OsI_010192 [Oryza sativa (indica cultivar-group)] (GI	
EV174979	2.244	moderately similar to (258)AT1G62750| Symbols: ATSCO1, ATSCO1/CPEF-G, SCO1 | ATSCO1/ATSCO1/CPEF-G/SCO1 (SNOWY CC	
JCVI_11952	2.244	no original description	
JCVI_26065	2.244	highly similar to (511)AT4G35790| Symbols: PLDDELTA, ATPLDDELTA | ATPLDDELTA (Arabidopsis thaliana phospholipase D delta	1.172
EE435336	2.244	weakly similar to (134)AT3G51250| Symbols: | senescence/dehydration-associated protein-related | chr3:19039206-19041421 FORWARD	
JCVI_34784	2.244	highly similar to (603)AT1G15440| Symbols: | transducin family protein / WD-40 repeat family protein | chr1:5306154-5309455 REVERS	
JCVI_38903	2.244	moderately similar to (410)AT5G44450| Symbols: | similar to hypothetical protein OsI_013284 [Oryza sativa (indica cultivar-group)] (GB	
JCVI_13889	2.243	weakly similar to (103)AT1G49920| Symbols: | zinc finger protein-related | chr1:18485466-18487901 REVERSE no original description	
ES967789	2.243	weakly similar to (131)AT5G51470| Symbols: | auxin-responsive GH3 family protein | chr5:20924513-20926566 FORWARDvery weakly	
JCVI_11989	2.243	moderately similar to (314)AT5G10450| Symbols: AFT1, GRF6 | GRF6 (G-BOX REGULATING FACTOR 6); protein phosphorylated am	
JCVI_30795	2.243	no original description	
JCVI_26784	2.243	moderately similar to (297)AT3G22660| Symbols: | rRNA processing protein-related | chr3:8016244-8017125 REVERSE no original desc	-3.123
JCVI_15684	2.242	moderately similar to (306)AT4G34950| Symbols: | nodulin family protein | chr4:16642549-16644764 REVERSE no original description	
ES912845	2.242	moderately similar to (357)AT2G37150| Symbols: | zinc finger (C3HC4-type RING finger) family protein | chr2:15610827-15613067 REV	-2.407
JCVI_23260	2.242	highly similar to (562)AT1G48470| Symbols: GLN1;5 | GLN1;5 (GLUTAMINE SYNTHETASE 1;5); glutamate-ammonia ligase | chr1:17	
JCVI_26069	2.241	moderately similar to (376)AT1G44820| Symbols: | aminoacylase, putative / N-acyl-L-amino-acid amidohydrolase, putative | chr1:169287	
EV095541	2.241	no similarity	
JCVI_17864	2.241	moderately similar to (319)AT1G07780| Symbols: TRP6, PAI1 | PAI1 (PHOSPHORIBOSYLANTHRANILATE ISOMERASE 1) | chr1:2	
JCVI_18927	2.240	highly similar to (884)AT2G18960| Symbols: PMA, OST2, AHA1 | AHA1 (ARABIDOPSIS H+ ATPASE 1); ATPase | chr2:8228940-823	
ES953631	2.240	no similarity	
JCVI_7663	2.240	moderately similar to (368)AT2G02180| Symbols: TOM3 | TOM3 (tobamovirus multiplication protein 3) | chr2:560975-562960 FORWAR	
EV070876	2.240	moderately similar to (363)AT4G01880| Symbols: | similar to unnamed protein product [Vitis vinifera] (GB:CAO40179.1); contains Inter	
EX137378	2.240	moderately similar to (466)AT1G61360| Symbols: | S-locus lectin protein kinase family protein | chr1:22641532-22644639 REVERSEwea	
JCVI_18856	2.240	moderately similar to (338)AT5G19930| Symbols: | integral membrane family protein | chr5:6737874-6739285 REVERSE no original des	
JCVI_39935	2.240	moderately similar to (388)AT5G55700| Symbols: BMY6, BAM4 | BAM4/BMY6 (BETA-AMYLASE 4); beta-amylase | chr5:22569099-2	
EE557895	2.240	no similarity	
JCVI_19865	2.240	moderately similar to (263)AT1G08490| Symbols: ATSUFS, SUFS, ATCPNIFS, ATNFS2, CPNIFS | ATCPNIFS/ATNFS2/ATSUFS/CPN	
JCVI_37166	2.239	highly similar to (756)AT2G45570| Symbols: CYP76C2 | CYP76C2 (cytochrome P450, family 76, subfamily C, polypeptide 2); oxygen bi	
DY029096	2.239	no similarity	
JCVI_37812	2.239	highly similar to (523)AT5G05200| Symbols: | ABC1 family protein | chr5:1544207-1547083 REVERSE no original description	
AM062591	2.238	moderately similar to (385)AT5G02500| Symbols: HSP70-1, AT-HSC70-1, HSC70, HSC70-1 | HSC70-1 (heat shock cognate 70 kDa prote	
JCVI_25833	2.238	moderately similar to (230)AT5G25520| Symbols: | transcription elongation factor-related | chr5:8885553-8889487 FORWARD no origina	
JCVI_26769	2.238	very weakly similar to (94.0)AT3G63260| Symbols: ATMRK1 | ATMRK1 (Arabidopsis thaliana MLK/Raf-related protein kinase 1); kinase	
ES900874	2.237	moderately similar to (439)AT5G56630| Symbols: | phosphofructokinase family protein | chr5:22941537-22943954 FORWARD [21428]	
JCVI_28843	2.237	highly similar to (701)AT2G45910| Symbols: | protein kinase family protein / U-box domain-containing protein | chr2:18901593-1890528	
JCVI_30681	2.237	no original description	
JCVI_5422	2.236	moderately similar to (350)AT2G47730| Symbols: GST6, ATGSTF5, GSTF8, ATGSTF8 | ATGSTF8 (GLUTATHIONE S-TRANSFERAS	
EV093526	2.235	no similarity	
JCVI_20185	2.235	weakly similar to (103)AT3G59640| Symbols: | glycine-rich protein | chr3:22040078-22041277 FORWARD no original description	

CD828750	2.235	moderately similar to (263)AT3G02750| Symbols: | protein phosphatase 2C family protein / PP2C family protein | chr3:593608-595464 R	
JCVI_41030	2.235	no original description	
EX120841	2.235	moderately similar to (214)AT4G00970| Symbols: | protein kinase family protein | chr4:418437-421694 FORWARD [21829] 28 588 588	
JCVI_16073	2.235	moderately similar to (301)AT2G21600| Symbols: ATRER1B | ATRER1B (Arabidopsis thaliana endoplasmatic reticulum retrieval protein	
JCVI_12887	2.235	moderately similar to (283)AT2G32930| Symbols: ZFN2 | ZFN2 (ZINC FINGER PROTEIN 2); nucleic acid binding | chr2:13973067-1397	
JCVI_27879	2.234	moderately similar to (241)AT5G47390| Symbols: | myb family transcription factor | chr5:19244228-19245773 FORWARD no original de	
JCVI_6194	2.234	moderately similar to (377)AT3G02740| Symbols: | aspartyl protease family protein | chr3:590568-593096 FORWARDvery weakly simila	
EE454202	2.234	no similarity	
JCVI_8617	2.234	weakly similar to (169)AT5G13730| Symbols: SIGD, SIG4 | SIG4 (SIGMA FACTOR 4); DNA binding / DNA-directed RNA polymerase/	
JCVI_910	2.234	moderately similar to (286)AT5G57280| Symbols: | methyltransferase | chr5:23221759-23223711 FORWARD no original description	
EV119208	2.234	weakly similar to (175)AT1G24180| Symbols: IAR4 | IAR4 (IAA-conjugate-resistant 4); pyruvate dehydrogenase (acetyl-transferring) | chr	
JCVI_31840	2.234	weakly similar to (126)AT3G20970| Symbols: ATNFU2, NFU4 | NFU4 (NFU domain protein 4) | chr3:7348283-7349633 FORWARD no	
JCVI_28076	2.234	moderately similar to (335)AT4G22100| Symbols: | glycosyl hydrolase family 1 protein | chr4:11707382-11709944 REVERSEweakly sim	
JCVI_32459	2.233	highly similar to (529)AT4G39800| Symbols: MI-1-P SYNTHASE | MI-1-P SYNTHASE (Myo-inositol-1-phosphate synthase); inositol-3-	
JCVI_31022	2.233	highly similar to (505)AT3G06510| Symbols: SFR2 | SFR2 (SENSITIVE TO FREEZING 2) | chr3:2016456-2019539 FORWARD no origi	-2.722
EV139662	2.233	no similarity	
CV432443	2.233	no similarity	
JCVI_14711	2.233	highly similar to (754)AT1G65430| Symbols: | zinc finger protein-related | chr1:24304828-24309822 REVERSE no original description	
JCVI_33797	2.232	weakly similar to (103)AT5G11730| Symbols: | similar to unknown protein [Arabidopsis thaliana] (TAIR:AT5G25970.1); similar to hypot	
EV090690	2.232	moderately similar to (204)AT3G23760| Symbols: | similar to transferase, transferring glycosyl groups [Arabidopsis thaliana] (TAIR:AT4	
ES929862	2.232	weakly similar to (118)AT5G46230| Symbols: | similar to unknown protein [Arabidopsis thaliana] (TAIR:AT1G09310.1); similar to unnar	
JCVI_26650	2.232	moderately similar to (434)AT5G03240| Symbols: UBQ3 | UBQ3 (POLYUBIQUITIN 3); protein binding | chr5:771975-772895 REVERS	
EX120515	2.232	no similarity	
BQ791001	2.231	moderately similar to (204)AT5G09590| Symbols: HSC70-5, mtHSC70-2 | mtHSC70-2 (HEAT SHOCK PROTEIN 70); ATP binding / un	
EV110599	2.231	weakly similar to (118)AT4G21910| Symbols: | MATE efflux family protein | chr4:11625833-11630976 REVERSE [21478] 1 367 755	
JCVI_13964	2.230	weakly similar to (181)AT1G11270| Symbols: | F-box family protein | chr1:3785833-3786653 REVERSE no original description	
EE531296	2.230	no similarity	
CD813922	2.230	moderately similar to (258)AT5G66670| Symbols: | Identical to UPF0496 protein At5g66670 [Arabidopsis Thaliana] (GB:Q9LVR3); simi	1.385
JCVI_1901	2.230	weakly similar to (186)AT5G46110| Symbols: TPT, APE2 | APE2 (ACCLIMATION OF PHOTOSYNTHESIS TO ENVIRONMENT) | ch	
JCVI_25400	2.230	highly similar to (699)AT2G25737| Symbols: | similar to unknown protein [Arabidopsis thaliana] (TAIR:AT2G36630.1); similar to unnam	
JCVI_13415	2.230	moderately similar to (244)AT3G18300| Symbols: | similar to unknown protein [Arabidopsis thaliana] (TAIR:AT1G48780.1); similar to h	
JCVI_40765	2.229	no original description	
CO749970	2.229	very weakly similar to (99.0)AT1G68000| Symbols: ATPIS, ATPIS1 | ATPIS1 (Arabidopsis thaliana phosphatidylinositol synthase 1); CDI	
EX087529	2.229	weakly similar to (137)AT1G11510| Symbols: | DNA-binding storekeeper protein-related | chr1:3871778-3872836 REVERSE [21823]	
EE535249	2.229	no similarity	
JCVI_9117	2.228	highly similar to (547)AT4G24620| Symbols: PGI, PGI1 | PGI1 (CHLOROPLASTIC PHOSPHOGLUCOSE ISOMERASE) | chr4:127091	-1.844
JCVI_29272	2.228	moderately similar to (239)AT5G18830| Symbols: SPL7 | SPL7 (SQUAMOSA PROMOTER BINDING PROTEIN-LIKE 7); DNA binding	
JCVI_8261	2.228	highly similar to (508)AT5G58900| Symbols: | myb family transcription factor | chr5:23800501-23801893 REVERSE no original descripti	
EG019236	2.228	weakly similar to (114)AT5G15190| Symbols: | unknown protein | chr5:4933151-4933501 REVERSE [20440]	
DW999857	2.227	no similarity	
JCVI_15241	2.227	moderately similar to (300)AT1G53210| Symbols: | sodium/calcium exchanger family protein / calcium-binding EF hand family protein | c	
JCVI_25113	2.227	moderately similar to (380)AT1G79500| Symbols: AtkdsA1 | AtkdsA1 (Arabidopsis thaliana KDO-8-phosphate synthase A1); 3-deoxy-8-p	
EE501690	2.227	very weakly similar to (95.9)AT3G05590| Symbols: RPL18 | RPL18 (RIBOSOMAL PROTEIN L18); structural constituent of ribosome | cl	
EV107679	2.227	no similarity	
JCVI_1242	2.226	moderately similar to (317)AT1G01720| Symbols: ANAC002, ATAF1 | ATAF1 (Arabidopsis NAC domain containing protein 2); transcrip	
JCVI_32000	2.226	moderately similar to (479)AT5G21050| Symbols: | similar to unknown protein [Arabidopsis thaliana] (TAIR:AT5G64090.1); similar to u	1.064
EX092188	2.226	moderately similar to (384)AT2G04360| Symbols: | similar to unnamed protein product [Vitis vinifera] (GB:CAO41137.1) | chr2:1519642	
JCVI_9871	2.226	highly similar to (578)AT4G16143| Symbols: | importin alpha-2, putative (IMPA-2) | chr4:9134736-9137147 REVERSEhighly similar to (	
JCVI_20381	2.226	no original description	
EV026388	2.226	moderately similar to (302)AT1G51600| Symbols: TIFY2A, ZML2 | ZML2 (ZIM-LIKE 2); transcription factor | chr1:19136844-19138920	
EV025504	2.226	moderately similar to (354)AT3G08510| Symbols: ATPLC2 | ATPLC2 (PHOSPHOLIPASE C 2); phospholipase C | chr3:2582632-258556	3.390
JCVI_61	2.226	moderately similar to (295)AT2G47890| Symbols: | zinc finger (B-box type) family protein | chr2:19615315-19616546 FORWARD no ori	
JCVI_35767	2.226	very weakly similar to (96.3)AT5G44310| Symbols: | late embryogenesis abundant domain-containing protein / LEA domain-containing pr	
JCVI_35559	2.226	highly similar to (542)AT5G58100| Symbols: | similar to unknown protein [Arabidopsis thaliana] (TAIR:AT3G28720.1); similar to unnam	
EV206617	2.226	moderately similar to (358)AT5G51340| Symbols: | binding | chr5:20880820-20884397 FORWARD [21491] 53 761 761	
JCVI_3732	2.225	weakly similar to (200)AT5G13220| Symbols: JAZ10, TIFY9, JAS1, AT5G13220 | JAS1/JAZ10/TIFY9 (JASMONATE-ZIM-DOMAIN P	
JCVI_30967	2.225	highly similar to (548)AT5G38530| Symbols: | tryptophan synthase-related | chr5:15441327-15443524 FORWARD no original descriptior	
JCVI_2900	2.225	moderately similar to (318)AT3G04940| Symbols: ATCYSD1 | ATCYSD1 (Arabidopsis thaliana cysteine synthase D1); cysteine synthase	
JCVI_22027	2.225	no original description	
JCVI_3160	2.224	weakly similar to (178)AT4G29270| Symbols: | acid phosphatase class B family protein | chr4:14423803-14424854 REVERSEvery weakly	
AM059161	2.224	very weakly similar to (85.1)AT2G41670| Symbols: | GTP-binding family protein | chr2:17381196-17383255 FORWARD [17712]	
EV156914	2.224	moderately similar to (258)AT1G17790| Symbols: | DNA-binding bromodomain-containing protein | chr1:6125525-6127269 REVERSE [	
EX021884	2.223	weakly similar to (106)AT1G14120| Symbols: | 2-oxoglutarate-dependent dioxygenase, putative | chr1:4833645-4834830 REVERSE [218	
AM396094	2.223	moderately similar to (228)AT5G13740| Symbols: ZIF1 | ZIF1 (ZINC INDUCED FACILITATOR 1); carbohydrate transmembrane transp	
JCVI_4103	2.223	moderately similar to (364)AT2G47470| Symbols: ATPDIL2-1, UNE5, MEE30 | ATPDIL2-1/MEE30/UNE5 (PDI-LIKE 2-1) | chr2:19488	
JCVI_20334	2.222	weakly similar to (120)AT4G17713| Symbols: | Encodes a defensin-like (DEFL) family protein. | chr4:9859808-9860221 REVERSE no or	
JCVI_14560	2.222	moderately similar to (337)AT1G59700| Symbols: ATGSTU16 | ATGSTU16 (Arabidopsis thaliana Glutathione S-transferase (class tau) 1(	
JCVI_29269	2.222	moderately similar to (374)AT2G32120| Symbols: HSP70T-2 | HSP70T-2; ATP binding | chr2:13658797-13660488 REVERSEweakly sim	
EX024817	2.222	very weakly similar to (82.8)AT1G51390| Symbols: ATNFU1, NFU5 | NFU5 (NFU domain protein 5) | chr1:19054095-19055421 FORWA	
EV033128	2.222	moderately similar to (401)AT2G17470| Symbols: | similar to unknown protein [Arabidopsis thaliana] (TAIR:AT1G25480.1); similar to u	
JCVI_35894	2.221	weakly similar to (186)AT2G44940| Symbols: | AP2 domain-containing transcription factor TINY, putative | chr2:18544369-18545256 F(	
JCVI_925	2.221	weakly similar to (140)AT3G15670| Symbols: | late embryogenesis abundant protein, putative / LEA protein, putative | chr3:5310148-531	
EE463002	2.221	weakly similar to (114)AT4G12900| Symbols: | gamma interferon responsive lysosomal thiol reductase family protein / GILT family prote	
EE456112	2.221	very weakly similar to (91.7)AT3G03920| Symbols: | Gar1 RNA-binding region family protein | chr3:1009130-1010386 REVERSE [2017	
RC_AM388062	2.221	no similarity	
EV165075	2.221	very weakly similar to (100)AT3G17810| Symbols: | dihydroorotate dehydrogenase family protein / dihydroorotate oxidase family protein	-1.928
JCVI_35612	2.221	moderately similar to (220)AT2G37690| Symbols: | phosphoribosylaminoimidazole carboxylase, putative / AIR carboxylase, putative | chr	
JCVI_19826	2.220	moderately similar to (212)AT3G06420| Symbols: ATG8H | ATG8H (AUTOPHAGY 8H); microtubule binding | chr3:1955225-1956280 F	
JCVI_28859	2.220	moderately similar to (296)AT5G46630| Symbols: | clathrin adaptor complexes medium subunit family protein | chr5:18937807-18940287	
JCVI_36168	2.220	highly similar to (639)AT2G40540| Symbols: ATKT2, SHY3, KUP2, ATKUP2, TRK2, KT2 | KT2 (POTASSIUM TRANSPORTER 2) | c	
JCVI_18222	2.220	weakly similar to (148)AT1G20070| Symbols: | unknown protein | chr1:6958235-6958816 REVERSE no original description	

JCVI_2815	2.219	moderately similar to (211)AT4G13530| Symbols: | similar to unknown protein [Arabidopsis thaliana] (TAIR:AT4G10080.1); similar to u	
EV049417	2.219	weakly similar to (154)AT1G54320| Symbols: | LEM3 (ligand-effect modulator 3) family protein / CDC50 family protein | chr1:20279399	
EE513249	2.219	no similarity	
EG019503	2.219	no similarity	
EV191362	2.218	weakly similar to (110)AT3G27690| Symbols: LHCB2.3, LHCB2, LHCB2:4 | LHCB2:4 (Photosystem II light harvesting complex gene 2.3	
JCVI_22905	2.218	weakly similar to (113)AT5G48880| Symbols: PKT1, KAT5, PKT2 | KAT5/PKT1/PKT2 (PEROXISOMAL 3-KETO-ACYL-COA THIOI	
JCVI_17604	2.218	highly similar to (592)AT3G17790| Symbols: ATPAP17, PAP17, ATACP5 | ATACP5 (acid phosphatase 5); acid phosphatase/ protein seri	
ES946042	2.218	moderately similar to (210)AT5G55920| Symbols: | nucleolar protein, putative | chr5:22662968-22666609 REVERSE [21393]	
JCVI_3957	2.217	moderately similar to (201)AT2G37660| Symbols: | binding / catalytic/ coenzyme binding | chr2:15802559-15804055 REVERSE no origi	
JCVI_34076	2.217	no original description	
ES948962	2.216	moderately similar to (277)AT3G10730| Symbols: | sad1/unc-84-like 2 family protein | chr3:3358561-3360527 REVERSE [21393]	
CN727903	2.216	moderately similar to (204)AT5G40610| Symbols: | glycerol-3-phosphate dehydrogenase (NAD+) / GPDH | chr5:16282299-16284486 RE	
JCVI_14381	2.216	moderately similar to (375)AT4G31410| Symbols: | similar to unknown protein [Arabidopsis thaliana] (TAIR:AT3G24740.2); similar to u	
JCVI_22326	2.216	moderately similar to (347)AT1G44100| Symbols: AAP5 | AAP5 (amino acid permease 5); amino acid transmembrane transporter | chr1:1	
BQ791311	2.216	no similarity	-1.981
BG544855	2.216	no similarity	
ES944550	2.216	no similarity	
JCVI_2536	2.216	highly similar to (668)AT5G60640| Symbols: ATPDIL1-4 | ATPDIL1-4 (PDI-LIKE 1-4); thiol-disulfide exchange intermediate | chr5:243	
JCVI_38734	2.216	moderately similar to (337)AT2G33610| Symbols: CHB2, ATSWI3B | ATSWI3B (Arabidopsis thaliana switching protein 3B); DNA bindi	
JCVI_27001	2.215	moderately similar to (285)AT5G02310| Symbols: PRT6 | PRT6 (PROTEOLYSIS 6); ubiquitin-protein ligase | chr5:474277-482550 FORV	
EV205951	2.215	moderately similar to (301)AT2G01680| Symbols: | ankyrin repeat family protein | chr2:306596-308426 FORWARD [21491] 53 751 751	-1.712
EX078025	2.215	weakly similar to (151)AT3G25470| Symbols: | bacterial hemolysin-related | chr3:9234276-9236259 FORWARD [21818]	
JCVI_13806	2.215	moderately similar to (490)AT5G11670| Symbols: ATNADP-ME2 | ATNADP-ME2 (NADP-MALIC ENZYME 2); malate dehydrogenase	
JCVI_35555	2.215	moderately similar to (229)AT5G49760| Symbols: | leucine-rich repeat family protein / protein kinase family protein | chr5:20233905-202	
JCVI_23893	2.214	weakly similar to (146)AT1G24350| Symbols: | similar to unknown protein [Arabidopsis thaliana] (TAIR:AT1G67600.1); similar to unna	
EX136121	2.214	moderately similar to (319)AT4G14630| Symbols: GLP9 | GLP9 (GERMIN-LIKE PROTEIN 9); manganese ion binding / metal ion bindin	
JCVI_821	2.214	moderately similar to (403)AT5G04430| Symbols: | KH domain-containing protein NOVA, putative | chr5:1250603-1253524 REVERSE	
EX113570	2.214	weakly similar to (112)AT1G22740| Symbols: AtRABG3b, RAB7 | RAB7 (Ras-related protein 7); GTP binding | chr1:8049236-8050483 F	
JCVI_34756	2.214	moderately similar to (419)AT5G51050| Symbols: | mitochondrial substrate carrier family protein | chr5:20770607-20772940 FORWARD	
JCVI_30460	2.214	highly similar to (672)AT3G02875| Symbols: ILR1 | ILR1 (IAA-LEUCINE RESISTANT 1); metallopeptidase | chr3:632000-633866 FOR	
JCVI_11662	2.213	highly similar to (527)AT1G10290| Symbols: DRP2A, ADL6 | ADL6 (DYNAMIN-LIKE PROTEIN 6) | chr1:3370776-3377122 FORWA	
EE439966	2.213	weakly similar to (170)AT3G13226| Symbols: | regulatory protein RecX family protein | chr3:4264449-4266293 REVERSE [20167]	-2.624
JCVI_41365	2.213	no original description	
JCVI_6896	2.213	highly similar to (535)AT4G25300| Symbols: | oxidoreductase, 2OG-Fe(II) oxygenase family protein | chr4:12945790-12946652 FORWA	
JCVI_6567	2.213	moderately similar to (390)AT4G02405| Symbols: | similar to unnamed protein product [Vitis vinifera] (GB:CAO40030.1); similar to hyp	
JCVI_15063	2.213	moderately similar to (230)AT3G16230| Symbols: | RNA binding / catalytic | chr3:5500569-5503309 FORWARD no original description	
JCVI_32967	2.213	moderately similar to (415)AT1G31280| Symbols: AGO2 | AGO2 (ARGONAUTE 2); nucleic acid binding | chr1:11181758-11185093 FO	
EE520236	2.213	weakly similar to (158)AT5G13120| Symbols: | peptidyl-prolyl cis-trans isomerase cyclophilin-type family protein | chr5:4162717-416472	
JCVI_14498	2.212	moderately similar to (498)AT4G28740| Symbols: | similar to LPA1 (LOW PSII ACCUMULATION1), binding [Arabidopsis thaliana] (T	
EE412722	2.212	weakly similar to (164)AT4G11740| Symbols: SAY1 | SAY1 | chr4:7071949-7075250 FORWARD [20145]	-3.334
EV008065	2.212	no similarity	
EL589995	2.212	moderately similar to (431)AT3G04530| Symbols: PEPCK2, PPCK2 | PPCK2 (PHOSPHOENOLPYRUVATE CARBOXYLASE KINASE	
JCVI_21234	2.211	no original description	
JCVI_41832	2.210	moderately similar to (351)AT1G78880| Symbols: | balbiani ring 1-related / BR1-related | chr1:29657961-29659712 REVERSE no origina	
EV109263	2.210	moderately similar to (233)AT2G29990| Symbols: NDA2 | NDA2 (ALTERNATIVE NAD(P)H DEHYDROGENASE 2); NADH dehydrog	
JCVI_8742	2.210	moderately similar to (331)AT1G55150| Symbols: | DEAD box RNA helicase, putative (RH20) | chr1:20578301-20580808 FORWARDw	
DV643325	2.209	very weakly similar to (94.7)AT1G29290| Symbols: | similar to hypothetical protein [Vitis vinifera] (GB:CAN69942.1) | chr1:10245041-1	
JCVI_5520	2.209	moderately similar to (417)AT2G20830| Symbols: | folic acid binding / transferase | chr2:8975451-8976879 REVERSE no original descri	
JCVI_33448	2.209	moderately similar to (386)AT1G05590| Symbols: HEXO2, ATHEX3 | ATHEX3/HEXO2 (BETA-HEXOSAMINIDASE 2); beta-N-acetyl	
JCVI_4444	2.209	weakly similar to (187)AT3G58490| Symbols: | phosphatidic acid phosphatase family protein / PAP2 family protein | chr3:21644339-2164	
EX128380	2.208	no similarity	
JCVI_5226	2.208	weakly similar to (130)AT5G07240| Symbols: IQD24 | IQD24 (IQ-domain 24); calmodulin binding | chr5:2272029-2274052 FORWARD	
JCVI_11202	2.208	moderately similar to (207)AT5G13330| Symbols: RAP2.6L | RAP2.6L (related to AP2 6L); DNA binding / transcription factor | chr5:427	
JCVI_17079	2.208	highly similar to (598)AT5G03160| Symbols: | DNAJ heat shock N-terminal domain-containing protein | chr5:750285-752670 FORWARI	
JCVI_8341	2.208	moderately similar to (207)AT4G30820| Symbols: | cyclin-dependent kinase-activating kinase assembly factor-related / CDK-activating ki	
BG544141	2.208	weakly similar to (150)AT4G31040| Symbols: | proton extrusion protein-related | chr4:15111817-15113887 REVERSE [8791]	
JCVI_11505	2.207	moderately similar to (366)AT1G12200| Symbols: | flavin-containing monooxygenase family protein / FMO family protein | chr1:413762	
JCVI_40012	2.207	moderately similar to (437)AT1G26390| Symbols: | FAD-binding domain-containing protein | chr1:9130151-9131743 REVERSE no origi	
JCVI_22962	2.207	no original description	
EX096588	2.207	moderately similar to (306)AT1G78130| Symbols: UNE2 | UNE2 (unfertilized embryo sac 2); carbohydrate transmembrane transporter/ su	
EV121921	2.207	moderately similar to (365)AT1G19520| Symbols: NFD5 | NFD5 (NUCLEAR FUSION DEFECTIVE 5); transcription factor | chr1:67600	
JCVI_37456	2.207	moderately similar to (473)AT1G69450| Symbols: | similar to HYP1 (HYPOTHETICAL PROTEIN 1) [Arabidopsis thaliana] (TAIR:AT3	
CN727303	2.207	no similarity	-2.413
EV052565	2.207	moderately similar to (279)AT3G48510| Symbols: | similar to unknown protein [Arabidopsis thaliana] (TAIR:AT5G63350.1); similar to u	
JCVI_22158	2.206	moderately similar to (291)AT5G11980| Symbols: | conserved oligomeric Golgi complex component-related / COG complex component-	
EX067148	2.206	weakly similar to (116)AT5G19000| Symbols: ATBPM1 | ATBPM1 (BTB-POZ AND MATH DOMAIN 1); protein binding | chr5:634256	
EE470630	2.206	no similarity	
EV172678	2.205	moderately similar to (224)AT1G71695| Symbols: | peroxidase 12 (PER12) (P12) (PRXR6) | chr1:26968021-26970219 FORWARDweak	-1.734
JCVI_28793	2.205	moderately similar to (430)AT3G06510| Symbols: SFR2 | SFR2 (SENSITIVE TO FREEZING 2) | chr3:2016456-2019539 FORWARD no	
EX021546	2.204	no similarity	
EV012598	2.204	no similarity	
EV053692	2.204	moderately similar to (312)AT4G22140| Symbols: | DNA binding | chr4:11728105-11730242 REVERSE [21442]	
JCVI_18621	2.204	moderately similar to (343)AT5G19050| Symbols: | similar to unnamed protein product [Vitis vinifera] (GB:CAO71093.1); contains Inter	
EV154920	2.204	weakly similar to (187)AT5G61790| Symbols: | calnexin 1 (CNX1) | chr5:24844620-24846868 REVERSEweakly similar to (119)CALX_	
DN960767	2.204	weakly similar to (155)AT4G23150| Symbols: | protein kinase family protein | chr4:12125742-12128312 FORWARD [17359]	
JCVI_1215	2.204	weakly similar to (122)AT1G18800| Symbols: NRP2 | NRP2 (NAP1-RELATED PROTEIN 2); DNA binding / chromatin binding / histone	
EE530347	2.203	no similarity	
JCVI_11684	2.203	moderately similar to (287)AT5G21060| Symbols: | homoserine dehydrogenase family protein | chr5:7149155-7152747 REVERSE no orig	
JCVI_32379	2.203	moderately similar to (429)AT3G53520| Symbols: ATUXS1, UXS1 | UXS1 (UDP-GLUCURONIC ACID DECARBOXYLASE 1) | chr3:1	
EX036423	2.203	no similarity	
AM393903	2.203	no similarity	

CD828207	2.203	moderately similar to (368)AT4G23640| Symbols: ATKT3, KUP4, TRH1 | TRH1 (TINY ROOT HAIR 1); potassium ion transmembrane t	
JCVI_42342	2.202	highly similar to (607)AT5G07320| Symbols: | mitochondrial substrate carrier family protein | chr5:2310249-2312083 FORWARD no orig	
H07429	2.201	no similarity	
EV202611	2.201	moderately similar to (238)AT2G36750| Symbols: UGT72C1 | UGT72C1 (UDP-GLUCOSYL TRANSFERASE 72C1); UDP-glycosyltrans	1.625
EV109105	2.201	no similarity	
DY016415	2.201	moderately similar to (283)AT1G12990| Symbols: | glycosyl transferase family 17 protein | chr1:4433971-4435550 FORWARD [18975]	
JCVI_14361	2.201	no original description	
JCVI_7801	2.201	moderately similar to (237)AT3G51890| Symbols: | protein binding / protein transporter/ structural molecule | chr3:19260663-19261804 F	
EE564226	2.201	moderately similar to (251)AT5G14280| Symbols: | DNA-binding storekeeper protein-related | chr5:4609126-4611447 FORWARD [2015	
JCVI_1000	2.200	highly similar to (706)AT5G51970| Symbols: | sorbitol dehydrogenase, putative / L-iditol 2-dehydrogenase, putative | chr5:21129046-211	
JCVI_3571	2.200	moderately similar to (408)AT4G27720| Symbols: | similar to unknown protein [Arabidopsis thaliana] (TAIR:AT1G64650.2); similar to u	-2.630
JCVI_13106	2.200	highly similar to (529)AT5G24150| Symbols: SQP1 | SQP1 (Squalene monooxygenase 1) | chr5:8172673-8175398 REVERSEhighly simila	
EE562255	2.200	no similarity	
EV084959	2.200	no similarity	
JCVI_30918	2.199	very weakly similar to (84.7)AT2G36050| Symbols: ATOFP15, OFP15 | ATOFP15/OFP15 (Arabidopsis thaliana ovate family protein 15) |	
JCVI_34393	2.199	moderately similar to (483)AT5G13640| Symbols: ATPDAT | ATPDAT (Arabidopsis thaliana phospholipid:diacylglycerol acyltransferase	
JCVI_17987	2.199	moderately similar to (431)AT5G11580| Symbols: | UVB-resistance protein-related / regulator of chromosome condensation (RCC1) fami	
ES943599	2.199	no similarity	-2.453
ES987713	2.199	weakly similar to (115)AT4G29480| Symbols: | mitochondrial ATP synthase g subunit family protein | chr4:14486271-14487263 REVERS	
JCVI_21179	2.199	moderately similar to (253)AT3G51440| Symbols: | strictosidine synthase family protein | chr3:19100093-19101516 FORWARD no origin	-5.488
JCVI_35126	2.199	moderately similar to (336)AT1G09830| Symbols: | phosphoribosylamine--glycine ligase (PUR2) | chr1:3192785-3194938 REVERSEmod	
EV143849	2.199	no similarity	
EE434650	2.198	no similarity	
AM062438	2.198	moderately similar to (269)AT5G64410| Symbols: ATOPT4 | ATOPT4 (oligopeptide transporter 4); oligopeptide transporter | chr5:257681	
JCVI_13377	2.198	moderately similar to (214)AT3G27200| Symbols: | plastocyanin-like domain-containing protein | chr3:10044975-10045577 REVERSE no	
JCVI_42500	2.198	no original description	
JCVI_1723	2.198	moderately similar to (385)AT2G34560| Symbols: | katanin, putative | chr2:14567345-14569774 FORWARDweakly similar to (126)CDC	
JCVI_1975	2.198	highly similar to (602)AT4G34450| Symbols: | coatomer gamma-2 subunit, putative / gamma-2 coat protein, putative / gamma-2 COP, put	
ES990696	2.197	weakly similar to (138)AT1G12800| Symbols: | S1 RNA-binding domain-containing protein | chr1:4361776-4365187 REVERSE [21425]	
EX092638	2.197	moderately similar to (291)AT5G56420| Symbols: | F-box family protein | chr5:22868089-22869560 REVERSE [21823]	
CX194291	2.196	moderately similar to (355)AT3G27820| Symbols: ATMDAR4, MDAR4 | ATMDAR4/MDAR4 (MONODEHYDROASCORBATE REDU	
RC_EV147794	2.196	no similarity	
EV205683	2.196	no similarity	
JCVI_18015	2.196	moderately similar to (320)AT3G16100| Symbols: AtRABG3c, AtRab7D | AtRABG3c/AtRab7D (Arabidopsis Rab GTPase homolog G3c)	
JCVI_32413	2.195	moderately similar to (372)AT5G01450| Symbols: | protein binding / zinc ion binding | chr5:183692-186243 REVERSE no original descri	
CV544545	2.195	weakly similar to (109)AT3G27270| Symbols: | similar to DNA-binding storekeeper protein-related [Arabidopsis thaliana] (TAIR:AT5G1	
CX268151	2.195	moderately similar to (201)AT4G28480| Symbols: | DNAJ heat shock family protein | chr4:14073316-14075097 FORWARD [16816]	-2.326
JCVI_7998	2.194	moderately similar to (254)AT3G05545| Symbols: | transcription factor, putative / zinc finger (C3HC4 type RING finger) family protein |	-3.090
JCVI_42209	2.194	weakly similar to (106)AT2G24270| Symbols: ALDH11A3 | ALDH11A3 (Aldehyde dehydrogenase 11A3); 3-chloroallyl aldehyde dehydro	
JCVI_36583	2.194	no original description	
JCVI_18158	2.194	moderately similar to (219)AT4G22880| Symbols: TDS4, TT18, ANS, LDOX | LDOX (TANNIN DEFICIENT SEED 4) | chr4:12004916-	
EX021068	2.193	moderately similar to (310)AT4G04570| Symbols: | protein kinase family protein | chr4:2290043-2292253 FORWARDweakly similar to (	
JCVI_8098	2.193	moderately similar to (320)AT4G01690| Symbols: HEMG1, PPOX | PPOX (PROTOPORPHYRINOGEN OXIDASE); protoporphyrinoge	
JCVI_26704	2.193	moderately similar to (311)AT3G56160| Symbols: | bile acid:sodium symporter | chr3:20848712-20852520 REVERSE no original descrip	
CX192838	2.193	moderately similar to (318)AT1G54790| Symbols: | GDSL-motif lipase/hydrolase family protein | chr1:20444791-20447664 REVERSEwe	
EE408450	2.193	moderately similar to (244)AT4G37210| Symbols: | tetratricopeptide repeat (TPR)-containing protein | chr4:17512370-17514415 FORWA	
JCVI_6464	2.193	moderately similar to (260)AT2G25070| Symbols: | protein phosphatase 2C, putative / PP2C, putative | chr2:10670596-10672445 REVER	
EE533435	2.192	weakly similar to (171)AT2G25430| Symbols: | epsin N-terminal homology (ENTH) domain-containing protein | chr2:10829795-1083175	
ES988396	2.192	no similarity	
JCVI_24763	2.192	no original description	
EV131122	2.192	moderately similar to (250)AT1G31300| Symbols: | similar to unknown protein [Arabidopsis thaliana] (TAIR:AT4G19645.2); similar to u	
JCVI_35724	2.192	very weakly similar to (90.9)AT3G16350| Symbols: | myb family transcription factor | chr3:5547834-5549403 FORWARD no original des	
EH419432	2.191	weakly similar to (123)AT2G25300| Symbols: | transferase, transferring glycosyl groups / transferase, transferring hexosyl groups | chr2:1	
JCVI_609	2.191	moderately similar to (400)AT1G65040| Symbols: | similar to zinc finger (C3HC4-type RING finger) family protein [Arabidopsis thaliana	
JCVI_38492	2.191	no original description	
JCVI_5986	2.191	moderately similar to (347)AT1G17200| Symbols: | integral membrane family protein | chr1:5878486-5879864 FORWARD no original de	
JCVI_12149	2.191	weakly similar to (140)AT5G56740| Symbols: HAG2, HAC7, HAG02, HAC07 | HAG2 (HISTONE ACETYLTRANSFERASE OF THE G	
JCVI_18763	2.190	weakly similar to (146)AT3G10185| Symbols: | Encodes a Gibberellin-regulated GASA/GAST/Snakin family protein | chr3:3145584-3146	
EV113345	2.190	weakly similar to (160)AT4G34138| Symbols: UGT73B1 | UGT73B1 (UDP-GLUCOSYL TRANSFERASE 73B1); UDP-glycosyltransfera	
JCVI_32166	2.190	moderately similar to (233)AT4G21990| Symbols: PRH-26, PRH26, ATAPR3, APR3 | APR3 (APS REDUCTASE 3) | chr4:11657296-116	-1.635
JCVI_19484	2.190	highly similar to (581)AT5G35970| Symbols: | DNA-binding protein, putative | chr5:14136290-14140308 REVERSE no original descripti	
JCVI_16935	2.190	moderately similar to (486)AT3G10350| Symbols: | anion-transporting ATPase family protein | chr3:3208315-3210683 FORWARD no or	
JCVI_33391	2.190	highly similar to (630)AT4G39950| Symbols: CYP79B2 | CYP79B2 (cytochrome P450, family 79, subfamily B, polypeptide 2); oxygen bi	
JCVI_17320	2.189	moderately similar to (355)AT1G13870| Symbols: DRL1 | DRL1 (DEFORMED ROOTS AND LEAVES 1) | chr1:4747434-4748342 REV	
EV049953	2.189	moderately similar to (474)AT5G15450| Symbols: APG6, CLPB3, CLPB-P | APG6/CLPB-P/CLPB3 (ALBINO AND PALE GREEN 6); A	
EE533151	2.189	moderately similar to (340)AT3G60860| Symbols: | guanine nucleotide exchange family protein | chr3:22495779-22502485 FORWARD [	
EV220134	2.189	weakly similar to (200)AT3G23160| Symbols: | similar to unknown protein [Arabidopsis thaliana] (TAIR:AT5G04550.1); similar to unna	-3.636
JCVI_40281	2.189	moderately similar to (380)AT1G22360| Symbols: ATUGT85A2 | ATUGT85A2 (UDP-GLUCOSYL TRANSFERASE 85A2); UDP-glyco	
JCVI_16386	2.189	moderately similar to (230)AT1G69870| Symbols: | proton-dependent oligopeptide transport (POT) family protein | chr1:26319871-26323	
JCVI_38335	2.189	weakly similar to (148)AT4G01220| Symbols: | similar to RGXT1 (RHAMNOGALACTURONAN XYLOSYLTRANSFERASE 1), UDP-	
JCVI_17571	2.188	no original description	
JCVI_15951	2.188	moderately similar to (268)AT1G68610| Symbols: | similar to unknown protein [Arabidopsis thaliana] (TAIR:AT1G14870.1); similar to u	
JCVI_41339	2.188	weakly similar to (182)AT3G12090| Symbols: TET6 | TET6 (TETRASPANIN6) | chr3:3852332-3853720 REVERSE no original descripti	
EX052567	2.188	weakly similar to (139)AT5G59900| Symbols: | pentatricopeptide (PPR) repeat-containing protein | chr5:24141209-24143932 REVERSE	
CD833806	2.188	moderately similar to (209)AT3G45040| Symbols: | phosphatidate cytidylyltransferase family protein | chr3:16483786-16486687 REVERS	
JCVI_11424	2.187	weakly similar to (195)AT5G35330| Symbols: MBD2, MBD02 | MBD02 (methyl-CpG-binding domain 2); DNA binding | chr5:13540955-	
EE505392	2.187	weakly similar to (108)AT5G25500| Symbols: | similar to unnamed protein product [Vitis vinifera] (GB:CAO43931.1) | chr5:8881121-888	
ES269865	2.187	no similarity	
EH414462	2.187	no similarity	
JCVI_25066	2.187	weakly similar to (127)AT1G64385| Symbols: | similar to unnamed protein product [Vitis vinifera] (GB:CAO69552.1) | chr1:23903972-2	
JCVI_6515	2.187	moderately similar to (283)AT3G51920| Symbols: CAM9 | CAM9 (CALMODULIN 9); calcium ion binding | chr3:19279156-19280292 R	

JCVI_303	2.187	moderately similar to (422)AT5G13180| Symbols: ANAC083 | ANAC083 (Arabidopsis NAC domain containing protein 83); transcription	
JCVI_7433	2.187	moderately similar to (313)AT2G27680| Symbols: | aldo/keto reductase family protein | chr2:11811058-11813042 REVERSE no original	
ES913954	2.187	moderately similar to (410)AT4G23190| Symbols: AT-RLK3, CRK11 | CRK11 (CYSTEINE-RICH RLK11); kinase | chr4:12141208-1214	
ES995954	2.187	weakly similar to (178)AT5G52650| Symbols: | 40S ribosomal protein S10 (RPS10C) | chr5:21373007-21374229 REVERSEweakly simila	
AM388387	2.187	no similarity	
JCVI_23774	2.187	moderately similar to (279)AT1G04250| Symbols: IAA17, AXR3 | AXR3 (AUXIN RESISTANT 3); transcription factor | chr1:1136381-1	
JCVI_7650	2.186	highly similar to (667)AT4G22880| Symbols: TDS4, TT18, ANS, LDOX | LDOX (TANNIN DEFICIENT SEED 4) | chr4:12004916-1200	
JCVI_26985	2.186	moderately similar to (213)AT1G06190| Symbols: | ATP binding / ATPase, coupled to transmembrane movement of ions, phosphorylative	
JCVI_896	2.186	very weakly similar to (96.3)AT1G61150| Symbols: | similar to unknown protein [Arabidopsis thaliana] (TAIR:AT4G09300.1); similar to	
EV211657	2.185	moderately similar to (250)AT1G54080| Symbols: UBP1A | UBP1A; mRNA 3'-UTR binding | chr1:20187591-20190492 REVERSE [214	
ES913294	2.185	moderately similar to (315)AT1G51860| Symbols: | leucine-rich repeat protein kinase, putative | chr1:19261303-19265148 REVERSE [21	
JCVI_32210	2.185	moderately similar to (202)AT5G18770| Symbols: | F-box family protein | chr5:6261428-6263174 FORWARD no original description	
JCVI_22888	2.185	moderately similar to (277)AT5G59830| Symbols: | similar to unknown protein [Arabidopsis thaliana] (TAIR:AT5G13660.2); similar to u	
DY019614	2.185	weakly similar to (139)AT1G14900| Symbols: HMGA | HMGA (HIGH MOBILITY GROUP A); DNA binding | chr1:5138660-5139348 R	
JCVI_39406	2.185	moderately similar to (482)AT3G44340| Symbols: CEF | CEF (CLONE EIGHTY-FOUR); transporter | chr3:16023071-16030860 REVER	
CX195673	2.184	weakly similar to (131)AT5G04490| Symbols: VTE5 | VTE5 (VITAMIN E PATHWAY GENE5); phosphatidate cytidylyltransferase/ phyt	
JCVI_21099	2.184	moderately similar to (333)AT1G14810| Symbols: | semialdehyde dehydrogenase family protein | chr1:5102679-5104628 REVERSE no o	
JCVI_22776	2.184	moderately similar to (379)AT3G10370| Symbols: | glycerol-3-phosphate dehydrogenase, putative | chr3:3216507-3219032 FORWARD n	-2.646
EX132805	2.184	moderately similar to (229)AT4G02500| Symbols: ATXT2 | ATXT2; UDP-xylosyltransferase/ transferase, transferring glycos	
CX189551	2.184	moderately similar to (466)AT4G25970| Symbols: PSD3 | PSD3 (PHOSPHATIDYLSERINE DECARBOXYLASE 3); calcium ion binding	
EV195218	2.184	moderately similar to (250)AT3G01910| Symbols: AT-SO, ATSO, SOX | SOX (SULFITE OXIDASE) | chr3:314926-317060 REVERSEve	
JCVI_7350	2.184	highly similar to (640)AT1G21780| Symbols: | BTB/POZ domain-containing protein | chr1:7652465-7653855 FORWARD no original des	
JCVI_33275	2.183	no original description	
JCVI_33503	2.183	moderately similar to (453)AT2G23540| Symbols: | GDSL-motif lipase/hydrolase family protein | chr2:10031446-10033138 FORWARDv	
JCVI_6947	2.183	weakly similar to (184)AT5G38590| Symbols: | F-box family protein | chr5:15469816-15470685 REVERSE no original description	
CD833451	2.183	moderately similar to (407)AT2G20710| Symbols: | pentatricopeptide (PPR) repeat-containing protein | chr2:8933616-8934803 FORWAR	
JCVI_18790	2.183	moderately similar to (342)AT3G14680| Symbols: CYP72A14 | CYP72A14 (cytochrome P450, family 72, subfamily A, polypeptide 14); c	
DY012632	2.182	moderately similar to (202)AT5G27750| Symbols: | F-box family protein | chr5:9828434-9829985 FORWARD [18975] 15 696 713	
EE506027	2.182	very weakly similar to (91.7)AT5G48630| Symbols: | cyclin family protein | chr5:19738889-19740426 REVERSE [20200]	
JCVI_1884	2.182	moderately similar to (316)AT5G05010| Symbols: | clathrin adaptor complexes medium subunit-related | chr5:1477138-1479873 FORWA	
EE568745	2.182	moderately similar to (223)AT2G16370| Symbols: THY-1 | THY-1 (THYMIDYLATE SYNTHASE 1) | chr2:7089120-7091415 REVERS	
CD828659	2.181	moderately similar to (416)AT5G13110| Symbols: G6PD2 | G6PD2 (GLUCOSE-6-PHOSPHATE DEHYDROGENASE 2); glucose-6-pho	
EV101101	2.181	weakly similar to (160)AT3G48740| Symbols: | nodulin MtN3 family protein | chr3:18063799-18065648 REVERSE [21477] 96 868 868	
JCVI_19411	2.181	moderately similar to (308)AT2G46590| Symbols: DAG2 | DAG2 (DOF AFFECTING GERMINATION 2); DNA binding | chr2:1914023	
JCVI_35987	2.181	moderately similar to (369)AT2G26250| Symbols: FDH | FDH (FIDDLEHEAD); acyltransferase | chr2:11177877-11180137 REVERSE n	
JCVI_42212	2.181	weakly similar to (191)AT1G11755| Symbols: | transferase | chr1:3969987-3971488 REVERSE no original description	
JCVI_21861	2.180	moderately similar to (391)AT3G62130| Symbols: | epimerase-related | chr3:23015969-23017333 FORWARD no original description	
JCVI_33593	2.180	moderately similar to (348)AT1G65900| Symbols: | similar to unnamed protein product [Vitis vinifera] (GB:CAO22185.1); contains doma	
JCVI_34304	2.179	moderately similar to (475)AT5G28150| Symbols: | similar to unknown protein [Arabidopsis thaliana] (TAIR:AT3G04860.1); similar to h	
JCVI_26864	2.179	highly similar to (580)AT2G21510| Symbols: | DNAJ heat shock N-terminal domain-containing protein | chr2:9217921-9219779 REVERS	
JCVI_34015	2.179	highly similar to (548)AT3G53950| Symbols: | glyoxal oxidase-related | chr3:19986682-19988319 FORWARD no original description	
JCVI_9816	2.179	very weakly similar to (100)AT2G47520| Symbols: | AP2 domain-containing transcription factor, putative | chr2:19509961-19510560 RE	
EE415409	2.179	moderately similar to (314)AT1G71960| Symbols: | ABC transporter family protein | chr1:27086249-27091825 REVERSE [20141]	
DY012193	2.179	weakly similar to (174)AT5G46350| Symbols: ATWRKY8, WRKY8 | WRKY8 (WRKY DNA-binding protein 8); transcription factor | chr	
CD817765	2.179	moderately similar to (258)AT5G63060| Symbols: | transporter | chr5:25312308-25313940 REVERSE [13978]	
EX135464	2.179	moderately similar to (295)AT2G36780| Symbols: | UDP-glucoronosyl/UDP-glucosyl transferase family protein | chr2:15424697-1542618	
JCVI_11595	2.179	highly similar to (516)AT1G06090| Symbols: | fatty acid desaturase family protein | chr1:1847920-1849499 FORWARD no original descr	
CV433374	2.178	weakly similar to (147)AT2G31940| Symbols: | oxidoreductase/ transition metal ion binding | chr2:13587677-13588039 FORWARD [164	
CV650388	2.178	no similarity	
EE450373	2.178	moderately similar to (202)AT4G12570| Symbols: UPL5 | UPL5 (UBIQUITIN PROTEIN LIGASE 5); ubiquitin-protein ligase | chr4:7445	
JCVI_8981	2.178	very weakly similar to (93.6)AT3G51820| Symbols: ATG4, G4, CHLG | ATG4/CHLG/G4 (CHLOROPHYLL SYNTHASE); chlorophyll sy	
JCVI_11658	2.178	moderately similar to (336)AT1G79550| Symbols: PGK | PGK (PHOSPHOGLYCERATE KINASE) | chr1:29929240-29931188 REVERS	
DY016844	2.178	weakly similar to (147)AT1G52200| Symbols: | similar to unknown protein [Arabidopsis thaliana] (TAIR:AT3G18470.1); similar to unna	
JCVI_26826	2.178	weakly similar to (115)AT4G23640| Symbols: ATKT3, KUP4, TRH1 | TRH1 (TINY ROOT HAIR 1); potassium ion transmembrane trans	
EX114054	2.177	weakly similar to (186)AT1G68140| Symbols: | similar to protein binding / zinc ion binding [Arabidopsis thaliana] (TAIR:AT4G08460.1);	
EV055604	2.177	weakly similar to (147)AT1G19650| Symbols: | SEC14 cytosolic factor, putative / phosphoglyceride transfer protein, putative | chr1:6796	
JCVI_675	2.177	moderately similar to (368)AT3G08940| Symbols: LHCB4.2 | LHCB4.2 (LIGHT HARVESTING COMPLEX PSII) | chr3:2717722-27186	
JCVI_23852	2.177	no original description	
JCVI_9620	2.177	weakly similar to (182)AT4G26480| Symbols: | KH domain-containing protein | chr4:13372893-13378398 REVERSE no original descript	
JCVI_41269	2.177	moderately similar to (258)AT5G02230| Symbols: | haloacid dehalogenase-like hydrolase family protein | chr5:449130-450505 FORWAR	
EE516785	2.177	moderately similar to (323)AT3G07050| Symbols: | GTP-binding family protein | chr3:2229608-2232285 REVERSE [20185] 1 650 663	
JCVI_26506	2.177	moderately similar to (336)AT3G60680| Symbols: | similar to unknown protein [Arabidopsis thaliana] (TAIR:AT2G45260.1); similar to u	-5.925
JCVI_4618	2.176	moderately similar to (480)AT4G19410| Symbols: | pectinacetylesterase, putative | chr4:10582199-10584777 REVERSE no original descr	
JCVI_26728	2.176	moderately similar to (479)AT2G47760| Symbols: | ALG3 family protein | chr2:19573003-19575291 REVERSE no original description	
JCVI_26870	2.175	moderately similar to (301)AT1G26180| Symbols: | similar to unnamed protein product [Vitis vinifera] (GB:CAO42035.1) | chr1:9054811	
JCVI_26256	2.175	moderately similar to (258)AT3G01550| Symbols: | triose phosphate/phosphate translocator, putative | chr3:216954-218863 REVERSEve	
ES266065	2.175	no similarity	
CV433026	2.175	very weakly similar to (80.5)AT3G45140| Symbols: ATLOX2, LOX2 | LOX2 (LIPOXYGENASE 2) | chr3:16536422-16540218 FORWAR	
JCVI_15239	2.175	moderately similar to (298)AT1G19320| Symbols: | pathogenesis-related thaumatin family protein | chr1:6679318-6680169 FORWARDm	
JCVI_39518	2.174	moderately similar to (332)AT1G30200| Symbols: | F-box family protein | chr1:10625131-10626270 FORWARD no original description	-3.092
JCVI_27202	2.174	weakly similar to (187)AT2G42460| Symbols: | meprin and TRAF homology domain-containing protein / MATH domain-containing prote	
JCVI_1798	2.174	highly similar to (505)AT5G23540| Symbols: | 26S proteasome regulatory subunit, putative | chr5:7938112-7939342 FORWARD no origi	
EE407728	2.173	weakly similar to (130)AT2G39670| Symbols: | radical SAM domain-containing protein | chr2:16541381-16544064 FORWARD [16821]	
JCVI_10975	2.173	moderately similar to (387)AT3G53260| Symbols: PAL2 | PAL2 (phenylalanine ammonia-lyase 2); phenylalanine ammonia-lyase | chr3:19	
JCVI_405	2.173	moderately similar to (360)AT4G35000| Symbols: APX3 | APX3 (ASCORBATE PEROXIDASE 3); L-ascorbate peroxidase | chr4:16665	-2.158
EV166326	2.173	no similarity	
JCVI_13128	2.173	moderately similar to (431)AT1G19650| Symbols: | SEC14 cytosolic factor, putative / phosphoglyceride transfer protein, putative | chr1:6	
JCVI_26733	2.172	weakly similar to (198)AT5G42440| Symbols: | protein kinase family protein | chr5:16990662-16991741 REVERSEweakly similar to (11	1.700
EV189764	2.172	weakly similar to (117)AT3G51950| Symbols: | zinc finger (CCCH-type) family protein / RNA recognition motif (RRM)-containing prote	
JCVI_30165	2.172	highly similar to (975)AT3G17310| Symbols: | methyltransferase family protein | chr3:5909300-5912844 REVERSE no original descriptio	
ES937691	2.172	weakly similar to (164)AT1G62040| Symbols: ATG8C | ATG8C (AUTOPHAGY 8C); microtubule binding | chr1:22936881-22937918 FO	

JCVI_28784	2.172	moderately similar to (262)AT1G60440| Symbols: ATCOAA, ATPANK1 | ATCOAA/ATPANK1 (PANTOTHENATE KINASE 1); panto	
CD833051	2.171	weakly similar to (200)AT5G11790| Symbols: | Ndr family protein | chr5:3799683-3802497 FORWARDweakly similar to (130)SF21_HE	
EV051367	2.171	weakly similar to (176)AT4G14820| Symbols: | pentatricopeptide (PPR) repeat-containing protein | chr4:8507789-8510033 REVERSE [2	
ES270872	2.170	no similarity	
JCVI_31729	2.170	weakly similar to (196)AT3G58510| Symbols: | DEAD box RNA helicase, putative (RH11) | chr3:21651585-21654441 FORWARD no ori	
EE569326	2.170	no similarity	
RC_EE564909	2.169	no similarity	
EV022188	2.169	weakly similar to (112)AT1G53560| Symbols: | similar to unknown protein [Arabidopsis thaliana] (TAIR:AT1G17080.1); similar to unkn	1.796
JCVI_17012	2.169	moderately similar to (418)AT2G35680| Symbols: | dual specificity protein phosphatase family protein | chr2:15004083-15005669 REVE	-5.354
JCVI_216	2.169	moderately similar to (362)AT1G48470| Symbols: GLN1;5 | GLN1;5 (GLUTAMINE SYNTHETASE 1;5); glutamate-ammonia ligase | ch	
JCVI_8139	2.169	weakly similar to (166)AT5G54310| Symbols: AGD5 | AGD5 (ARF-GAP DOMAIN 5); DNA binding | chr5:22074488-22078292 REVER	2.289
JCVI_7173	2.169	moderately similar to (422)AT1G11200| Symbols: | similar to unknown protein [Arabidopsis thaliana] (TAIR:AT4G21570.1); similar to h	
JCVI_11068	2.169	moderately similar to (249)AT1G10710| Symbols: | Encodes a Maternally expressed gene (MEG) family protein | chr1:3558424-3560408	
JCVI_173	2.168	moderately similar to (313)AT5G43780| Symbols: APS4 | APS4 | chr5:17606858-17608707 REVERSE no original description	
JCVI_41214	2.168	highly similar to (555)AT5G51430| Symbols: | conserved oligomeric Golgi complex component-related / COG complex component-relate	
EE440908	2.168	moderately similar to (318)AT5G12010| Symbols: | similar to unknown protein [Arabidopsis thaliana] (TAIR:AT4G29780.1); similar to u	
JCVI_39157	2.168	weakly similar to (197)AT2G01060| Symbols: | myb family transcription factor | chr2:73456-74902 REVERSE no original description	
CX193955	2.168	weakly similar to (139)AT3G06350| Symbols: EMB3004, MEE32 | EMB3004/MEE32 (EMBRYO DEFECTIVE 3004); 3-dehydroquinate	
JCVI_13126	2.167	moderately similar to (375)AT3G25140| Symbols: GAUT8, QUA1 | GAUT8/QUA1 (GALACTURONOSYLTRANSFERASE 8, QUASIM	
JCVI_17897	2.167	weakly similar to (134)AT1G71865| Symbols: | similar to unnamed protein product [Vitis vinifera] (GB:CAO64617.1) | chr1:27033779-27	
JCVI_35285	2.167	moderately similar to (204)AT2G38670| Symbols: PECT1 | PECT1 (PHOSPHORYLETHANOLAMINE CYTIDYLYLTRANSFERASE 1	
JCVI_25689	2.166	highly similar to (590)AT1G32330| Symbols: HSFA1D, ATHSFA1D | ATHSFA1D (Arabidopsis thaliana heat shock transcription factor A	
JCVI_1351	2.166	moderately similar to (455)AT1G07440| Symbols: | tropinone reductase, putative / tropine dehydrogenase, putative | chr1:2286697-22876	
JCVI_36485	2.166	moderately similar to (353)AT3G11340| Symbols: | UDP-glucoronosyl/UDP-glucosyl transferase family protein | chr3:3556734-3558155	
JCVI_39700	2.166	moderately similar to (384)AT5G16990| Symbols: | NADP-dependent oxidoreductase, putative | chr5:5581834-5583852 REVERSE no ori	
CX196139	2.165	no similarity	
EE442205	2.165	weakly similar to (159)AT1G56560| Symbols: | beta-fructofuranosidase, putative / invertase, putative / saccharase, putative / beta-fructosi	
EV226350	2.165	no similarity	
EX131852	2.165	no similarity	
JCVI_24503	2.165	no original description	
JCVI_15065	2.165	weakly similar to (162)AT1G51538| Symbols: | similar to unknown protein [Arabidopsis thaliana] (TAIR:AT5G18510.1); similar to unkn	
JCVI_14490	2.164	no original description	
CX194088	2.164	no similarity	
AM061109	2.163	weakly similar to (162)AT4G30190| Symbols: PMA2, AHA2 | AHA2 (Arabidopsis H(+)-ATPase 2); ATPase | chr4:14770826-14775926 F	-3.888
JCVI_28370	2.163	moderately similar to (224)AT3G04080| Symbols: ATAPY1 | ATAPY1 (APYRASE 1); calmodulin binding | chr3:1068075-1070924 REV	-2.809
EV031085	2.163	moderately similar to (229)AT2G28310| Symbols: | similar to unknown protein [Arabidopsis thaliana] (TAIR:AT1G08040.1); similar to u	
JCVI_30542	2.163	no original description	
JCVI_4981	2.163	moderately similar to (437)AT5G24600| Symbols: | similar to unknown protein [Arabidopsis thaliana] (TAIR:AT3G18215.1); similar to u	
JCVI_28848	2.163	moderately similar to (245)AT5G21140| Symbols: EMB1379 | EMB1379 (EMBRYO DEFECTIVE 1379) | chr5:7187419-7189524 REVE	
JCVI_22129	2.163	highly similar to (527)AT5G13550| Symbols: SULTR4;1 | SULTR4;1 (Sulfate transporter 4.1); sulfate transmembrane transporter | chr5:4	
EV208994	2.162	weakly similar to (164)AT3G62800| Symbols: DRB4 | DRB4 (double-stranded RNA-binding domain (DsRBD)-containing protein 4); doul	
JCVI_25484	2.162	moderately similar to (370)AT4G17640| Symbols: CKB2 | CKB2 (casein kinase II beta chain 2); protein kinase CK2 regulator | chr4:9825	
JCVI_3309	2.162	weakly similar to (105)AT5G44320| Symbols: | eukaryotic translation initiation factor 3 subunit 7, putative / eIF-3 zeta, putative / eIF3d, p	
JCVI_19225	2.162	moderately similar to (282)AT1G77000| Symbols: ATSKP2;2, SKP2B | ATSKP2;2/SKP2B (ARABIDOPSIS HOMOLOG OF HOMOLO	
JCVI_40697	2.162	moderately similar to (201)AT2G46430| Symbols: CNGC3, CNGC3.C, ATCNGC3 | ATCNGC3 (CYCLIC NUCLEOTIDE GATED CHA	
EX064394	2.162	weakly similar to (121)AT5G54080| Symbols: HGO | HGO (HOMOGENTISATE 1,2-DIOXYGENASE); homogentisate 1,2-dioxygenase	
JCVI_22379	2.161	moderately similar to (340)AT2G47050| Symbols: | invertase/pectin methylesterase inhibitor family protein | chr2:19338997-19339647 R	
JCVI_16553	2.161	moderately similar to (345)AT3G18630| Symbols: | uracil DNA glycosylase family protein | chr3:6411331-6413008 REVERSE no origina	
JCVI_12013	2.161	weakly similar to (185)AT1G71900| Symbols: | similar to permease-related [Arabidopsis thaliana] (TAIR:AT1G34470.1); similar to hypo	
CX193639	2.161	no similarity	
JCVI_21987	2.161	no original description	
L38159	2.160	weakly similar to (105)AT2G27810| Symbols: | xanthine/uracil permease family protein | chr2:11859415-11862488 FORWARD [132]	
EE534102	2.160	weakly similar to (125)AT4G29420| Symbols: | F-box family protein | chr4:14470721-14472210 REVERSE [20150]	
EE566788	2.160	very weakly similar to (81.3)AT1G24360| Symbols: | 3-oxoacyl-(acyl-carrier protein) reductase, chloroplast / 3-ketoacyl-acyl carrier protei	
DY027407	2.160	weakly similar to (120)AT1G36280| Symbols: | adenylosuccinate lyase, putative / adenylosuccinase, putative | chr1:13641812-13644120 F	
JCVI_5561	2.160	moderately similar to (259)AT2G28400| Symbols: | similar to unknown protein [Arabidopsis thaliana] (TAIR:AT5G60680.1); similar to u	
EE415154	2.160	very weakly similar to (95.9)AT1G32660| Symbols: | F-box family protein | chr1:11811020-11812360 FORWARD [20141]	
JCVI_24581	2.160	no original description	
JCVI_20730	2.159	moderately similar to (323)AT2G24050| Symbols: | MIF4G domain-containing protein / MA3 domain-containing protein | chr2:10232580	
JCVI_25033	2.159	moderately similar to (493)AT5G01220| Symbols: SQD2 | SQD2 (SULFOQUINOVOSYLDIACYLGLYCEROL 2); UDP-sulfoquinovose:	
ES919362	2.158	weakly similar to (165)AT2G29700| Symbols: ATPH1 | ATPH1 (ARABIDOPSIS THALIANA PLECKSTRIN HOMOLOGUE 1) | chr2:1	
JCVI_19805	2.158	moderately similar to (343)AT1G03910| Symbols: | similar to unnamed protein product [Vitis vinifera] (GB:CAO62957.1); similar to unk	
JCVI_13948	2.158	weakly similar to (151)AT3G19260| Symbols: LAG1 HOMOLOG 2 | LAG1 HOMOLOG 2 (LONGEVITY ASSURANCE GENE1 HOMC	
CO749479	2.158	no similarity	
JCVI_30875	2.158	moderately similar to (318)AT1G10470| Symbols: MEE7, ATRR1, IBC7, ARR4 | ARR4 (RESPONSE REGULATOR 4); transcription reg	
EV116120	2.158	moderately similar to (214)AT1G04790| Symbols: | zinc finger (C3HC4-type RING finger) family protein | chr1:1345468-1348142 FORW	
EV033998	2.157	weakly similar to (147)AT3G17310| Symbols: | methyltransferase family protein | chr3:5909300-5912844 REVERSE [21441]	
EE532929	2.157	weakly similar to (170)AT1G58220| Symbols: | myb family transcription factor | chr1:21562053-21566334 REVERSE [20175]	
JCVI_15771	2.157	weakly similar to (186)AT4G36020| Symbols: CSDP1 | CSDP1 (COLD SHOCK DOMAIN PROTEIN 1); RNA binding / double-stranded	
JCVI_39304	2.157	moderately similar to (337)AT1G47240| Symbols: ATNRAMP2, NRAMP2 | NRAMP2 (NRAMP metal ion transporter 2); metal ion trans	
EE502312	2.156	weakly similar to (174)AT3G57090| Symbols: BIGYIN | BIGYIN; binding | chr3:21139606-21140960 FORWARD [20193] 35 740 740	
EE451883	2.156	moderately similar to (236)AT4G34450| Symbols: | coatomer gamma-2 subunit, putative / gamma-2 coat protein, putative / gamma-2 COF	
EV197855	2.156	very weakly similar to (80.1)AT1G17550| Symbols: HAB2 | HAB2 (Homology to ABI2); protein serine/threonine phosphatase | chr1:6034	
EE568740	2.156	no similarity	
JCVI_13270	2.156	moderately similar to (287)AT1G72820| Symbols: | mitochondrial substrate carrier family protein | chr1:27407119-27408168 FORWARD	
EV048144	2.156	moderately similar to (330)AT5G09400| Symbols: KUP7 | KUP7 (K+ uptake permease 7); potassium ion transmembrane transporter | chr5	
JCVI_2356	2.155	moderately similar to (481)AT3G63520| Symbols: ATCCD1, ATNCED1, NCED1, CCD1 | CCD1 (CAROTENOID CLEAVAGE DIOXY	
JCVI_3650	2.155	moderately similar to (319)AT3G61610| Symbols: | aldose 1-epimerase family protein | chr3:22810455-22812004 FORWARD no original	
JCVI_39848	2.154	weakly similar to (155)AT4G27830| Symbols: | glycosyl hydrolase family 1 protein | chr4:13861800-13864495 REVERSEvery weakly sin	-2.540
EE403510	2.154	very weakly similar to (81.6)AT5G49120| Symbols: | senescence-associated protein-related | chr5:19926026-19926558 REVERSE [16820]	
JCVI_29684	2.154	moderately similar to (410)AT5G57830| Symbols: | similar to unknown protein [Arabidopsis thaliana] (TAIR:AT4G30830.1); similar to u	

JCVI_28172	2.154	weakly similar to (184)AT2G24280| Symbols: | serine carboxypeptidase S28 family protein | chr2:10341702-10344007 FORWARD no or	
JCVI_10800	2.154	moderately similar to (421)AT5G01340| Symbols: | mitochondrial substrate carrier family protein | chr5:143239-144560 REVERSE no or	
EV111864	2.154	no similarity	
JCVI_37425	2.154	moderately similar to (284)AT2G30970| Symbols: ASP1 | ASP1 (ASPARTATE AMINOTRANSFERASE 1) | chr2:13186089-13188763 F	
JCVI_25615	2.153	highly similar to (573)AT1G79490| Symbols: EMB2217 | EMB2217 (EMBRYO DEFECTIVE 2217) | chr1:29905510-29908020 FORWA	
EV134461	2.153	no similarity	
JCVI_33619	2.153	moderately similar to (455)AT3G23300| Symbols: | dehydration-responsive protein-related | chr3:8333528-8335909 FORWARD no origi	
JCVI_10096	2.153	weakly similar to (193)AT1G23730| Symbols: | carbonic anhydrase, putative / carbonate dehydratase, putative | chr1:8395954-8398003 F	-2.280
JCVI_28130	2.153	weakly similar to (181)AT5G48655| Symbols: | zinc finger (C3HC4-type RING finger) family protein | chr5:19748801-19749712 REVER	
JCVI_7069	2.153	highly similar to (816)AT2G46370| Symbols: JAR, FIN219, JAR1 | JAR1 (JASMONATE RESISTANT 1) | chr2:19041652-19043442 FO	
JCVI_19504	2.153	highly similar to (609)AT3G02350| Symbols: GAUT9 | GAUT9 (Galacturonosyltransferase 9); polygalacturonate 4-alpha-galacturonosyltr	
JCVI_12331	2.152	moderately similar to (390)AT3G21175| Symbols: TIFY2B, ZML1 | ZML1 (ZIM-LIKE 1) | chr3:7422838-7423771 FORWARD no origin	
JCVI_10876	2.152	moderately similar to (242)AT2G38380| Symbols: | peroxidase 22 (PER22) (P22) (PRXEA) / basic peroxidase E | chr2:16083521-160853	
JCVI_16738	2.152	moderately similar to (269)AT2G39210| Symbols: | nodulin family protein | chr2:16373365-16375309 REVERSE no original description	
EX040602	2.152	weakly similar to (113)AT1G22090| Symbols: EMB2204 | EMB2204 (EMBRYO DEFECTIVE 2204) | chr1:7795710-7797241 FORWAR	
EX062926	2.152	no similarity	
EX117897	2.152	weakly similar to (124)AT1G17070| Symbols: | D111/G-patch domain-containing protein | chr1:5837646-5840195 FORWARD [21828]	
JCVI_2905	2.152	moderately similar to (404)AT5G56630| Symbols: | phosphofructokinase family protein | chr5:22941537-22943954 FORWARD no origin	
JCVI_7704	2.152	moderately similar to (411)AT1G60770| Symbols: | pentatricopeptide (PPR) repeat-containing protein | chr1:22370624-22372313 REVEF	
CX278350	2.152	weakly similar to (114)AT1G73130| Symbols: | similar to unknown protein [Arabidopsis thaliana] (TAIR:AT1G17780.2); similar to hypot	
DY016190	2.151	moderately similar to (400)AT3G19100| Symbols: | calcium-dependent protein kinase, putative / CDPK, putative | chr3:6605687-6608986	-3.584
JCVI_32151	2.151	weakly similar to (165)AT5G23850| Symbols: | similar to unknown protein [Arabidopsis thaliana] (TAIR:AT3G48980.1); similar to unna	
DY010224	2.150	no similarity	
EV086653	2.150	weakly similar to (140)AT2G44530| Symbols: | ribose-phosphate pyrophosphokinase, putative / phosphoribosyl diphosphate synthetase, p	
AM390068	2.150	no similarity	
ES941750	2.150	moderately similar to (385)AT3G24320| Symbols: MSH1, CHM1, ATMSH1, CHM | ATMSH1/CHM/CHM1/MSH1 (MUTL PROTEIN H	1.261
EV049152	2.150	moderately similar to (354)AT3G48420| Symbols: | haloacid dehalogenase-like hydrolase family protein | chr3:17940728-17942536 FORV	
JCVI_22466	2.150	very weakly similar to (95.1)AT2G45010| Symbols: | similar to unknown protein [Arabidopsis thaliana] (TAIR:AT5G51400.1); similar to u	
JCVI_38869	2.149	moderately similar to (239)AT5G04610| Symbols: | spermidine synthase-related / putrescine aminopropyltransferase-related | chr5:132561	
JCVI_12198	2.149	moderately similar to (301)AT2G02360| Symbols: ATPP2-B10 | ATPP2-B10 (Phloem protein 2-B10); carbohydrate binding | chr2:619698	
JCVI_27502	2.149	moderately similar to (422)AT4G12130| Symbols: | aminomethyltransferase | chr4:7263634-7265419 FORWARD no original description	
EV110392	2.149	no similarity	
JCVI_38850	2.148	weakly similar to (143)AT5G03380| Symbols: | heavy-metal-associated domain-containing protein | chr5:832399-834127 REVERSE no o	
JCVI_10149	2.148	moderately similar to (250)AT1G19320| Symbols: | pathogenesis-related thaumatin family protein | chr1:6679318-6680169 FORWARDw	
CX188349	2.148	moderately similar to (246)AT5G21482| Symbols: ATCKX5, CKX7 | CKX7 (CYTOKININ OXIDASE 7); oxidoreductase | chr5:7226845-	
JCVI_10835	2.148	moderately similar to (278)AT1G73320| Symbols: | similar to Expressed protein [Arabidopsis thaliana] (TAIR:AT1G08125.1); similar to	
JCVI_10077	2.148	weakly similar to (179)AT1G10230| Symbols: ASK18 | ASK18 (ARABIDOPSIS SKP1-LIKE 18); protein binding / ubiquitin-protein ligas	
EE467059	2.148	moderately similar to (256)AT3G18210| Symbols: | oxidoreductase, 2OG-Fe(II) oxygenase family protein | chr3:6238270-6240402 REVE	
JCVI_17626	2.148	moderately similar to (484)AT1G27930| Symbols: | similar to unknown protein [Arabidopsis thaliana] (TAIR:AT1G67330.1); similar to u	
JCVI_14712	2.148	moderately similar to (455)AT3G07670| Symbols: | SET domain-containing protein | chr3:2451657-2454623 FORWARDvery weakly sim	
JCVI_7594	2.147	moderately similar to (342)AT1G80300| Symbols: ATNTT1 | ATNTT1; ATP:ADP antiporter | chr1:30196846-30199172 FORWARDmod	
EE541109	2.147	weakly similar to (177)AT4G30480| Symbols: | tetratricopeptide repeat (TPR)-containing protein | chr4:14897506-14898260 FORWARD	
DY019109	2.147	no similarity	
JCVI_27182	2.147	weakly similar to (144)AT5G54270| Symbols: LHCB3*1, LHCB3 | LHCB3 (LIGHT-HARVESTING CHLOROPHYLL BINDING PROT	
CV545213	2.147	very weakly similar to (83.2)AT5G10930| Symbols: SnRK3.24, CIPK5 | CIPK5 (CBL-INTERACTING PROTEIN KINASE 5); kinase | ch	
JCVI_38964	2.147	highly similar to (548)AT4G04955| Symbols: ATALN | ATALN (ARABIDOPSIS ALLANTOINASE); allantoinase/ hydrolase | chr4:2522	
ES967717	2.147	no similarity	
EV205928	2.146	no similarity	
JCVI_27460	2.146	moderately similar to (480)AT4G23180| Symbols: RLK4, CRK10 | CRK10 (CYSTEINE-RICH RLK10); kinase | chr4:12138182-1214079	
CX189377	2.146	no similarity	
EE521260	2.146	weakly similar to (175)AT5G12120| Symbols: | ubiquitin-associated (UBA)/TS-N domain-containing protein | chr5:3916230-3918089 RE	
EV194475	2.146	moderately similar to (352)AT3G28450| Symbols: | leucine-rich repeat transmembrane protein kinase, putative | chr3:10668596-1067041	
JCVI_40630	2.145	weakly similar to (178)AT1G52200| Symbols: | similar to unknown protein [Arabidopsis thaliana] (TAIR:AT3G18470.1); similar to unna	
EX093580	2.145	weakly similar to (124)AT4G05460| Symbols: | F-box family protein (FBL20) | chr4:2761103-2762397 REVERSE [21823]	
EX060476	2.145	moderately similar to (250)AT1G79460| Symbols: KS, ATKS, GA2 | GA2 (GA REQUIRING 2); ent-kaurene synthase | chr1:29895461-29	
JCVI_15405	2.145	highly similar to (640)AT3G04140| Symbols: | ankyrin repeat family protein | chr3:1087070-1089113 FORWARD no original description	
JCVI_16239	2.145	no original description	
JCVI_22727	2.145	moderately similar to (395)AT2G47510| Symbols: FUM1 | FUM1 (FUMARASE 1) | chr2:19505684-19509090 FORWARD no original de	
JCVI_16000	2.145	moderately similar to (321)AT2G35980| Symbols: NHL10, YLS9 | YLS9 (YELLOW-LEAF-SPECIFIC GENE 9) | chr2:15117714-151183	
JCVI_12556	2.144	moderately similar to (422)AT5G05920| Symbols: EDA22, DHS | DHS (embryo sac development arrest 22) | chr5:1777903-1779700 REV	
EX044080	2.144	moderately similar to (244)AT4G18990| Symbols: | xyloglucan:xyloglucosyl transferase, putative / xyloglucan endotransglycosylase, puta	
JCVI_12250	2.144	weakly similar to (147)AT1G80510| Symbols: | amino acid transporter family protein | chr1:30277992-30279461 FORWARD no original	
JCVI_5731	2.144	weakly similar to (176)AT1G03260| Symbols: | similar to unknown protein [Arabidopsis thaliana] (TAIR:AT5G19070.1); similar to unna	
JCVI_17219	2.144	moderately similar to (226)AT2G19430| Symbols: | transducin family protein / WD-40 repeat family protein | chr2:8422299-8424822 FO	
ES934366	2.144	moderately similar to (406)AT1G60710| Symbols: ATB2 | ATB2; oxidoreductase | chr1:22358738-22360292 REVERSEmoderately simila	
JCVI_26408	2.144	moderately similar to (410)AT5G36220| Symbols: CYP91A1, CYP81D1 | CYP81D1 (CYTOCHROME P450 91A1); oxygen binding | chr	
JCVI_32001	2.143	moderately similar to (452)AT1G79750| Symbols: ATNADP-ME4 | ATNADP-ME4 (NADP-MALIC ENZYME 4); malate dehydrogenase	
JCVI_7260	2.143	moderately similar to (278)AT1G71140| Symbols: | MATE efflux family protein | chr1:26828424-26830410 FORWARD no original desc	
JCVI_11955	2.143	moderately similar to (480)AT4G37270| Symbols: HMA1 | HMA1 (Heavy metal ATPase 1); copper-exporting ATPase | chr4:17541981-1	
EV100081	2.143	weakly similar to (181)AT3G59940| Symbols: | kelch repeat-containing F-box family protein | chr3:22153986-22155242 FORWARD [21	
JCVI_32438	2.143	highly similar to (587)AT2G29560| Symbols: | enolase, putative | chr2:12653712-12656771 FORWARDmoderately similar to (318)ENO_	
JCVI_41694	2.143	moderately similar to (258)AT3G09320| Symbols: | zinc finger (DHHC type) family protein | chr3:2862148-2864119 REVERSE no origin	
JCVI_10831	2.143	weakly similar to (111)AT2G44280| Symbols: | similar to lactose permease-related [Arabidopsis thaliana] (TAIR:AT3G60070.1); similar	
JCVI_19770	2.143	highly similar to (933)AT3G17650| Symbols: PDE321, YSL5 | YSL5 (YELLOW STRIPE LIKE 5); oligopeptide transporter | chr3:603431	
JCVI_762	2.142	highly similar to (523)AT2G26740| Symbols: ATSEH | ATSEH (Arabidopsis thaliana soluble epoxide hydrolase); epoxide hydrolase | chr2	
JCVI_35875	2.142	weakly similar to (194)AT3G10070| Symbols: TAF12, TAFII58 | TAF12/TAFII58 (TBP-ASSOCIATED FACTOR 12); DNA binding / tra	
BG543782	2.142	weakly similar to (132)AT5G50520| Symbols: | nodulin family protein | chr5:20586906-20588813 REVERSE [8791]	
JCVI_1640	2.142	moderately similar to (367)AT5G27350| Symbols: SFP1 | SFP1; carbohydrate transmembrane transporter/ sugar:hydrogen ion symporter |	
JCVI_33992	2.142	no original description	
JCVI_4358	2.141	moderately similar to (477)AT5G26280| Symbols: | meprin and TRAF homology domain-containing protein / MATH domain-containing	
JCVI_7568	2.141	moderately similar to (320)AT1G17180| Symbols: ATGSTU25 | ATGSTU25 (Arabidopsis thaliana Glutathione S-transferase (class tau) 2	

DY027965	2.141	moderately similar to (216)AT3G10915| Symbols: | reticulon family protein | chr3:3416106-3417502 REVERSE [18978]	
JCVI_41440	2.141	highly similar to (546)AT4G27440| Symbols: PORB | PORB (PROTOCHLOROPHYLLIDE OXIDOREDUCTASE B); oxidoreductase/ pr	
JCVI_14860	2.141	moderately similar to (432)AT2G45990| Symbols: | similar to hypothetical protein [Vitis vinifera] (GB:CAN70694.1); similar to unnamed	
JCVI_38506	2.140	moderately similar to (333)AT2G36330| Symbols: | Identical to UPF0497 membrane protein At2g36330 [Arabidopsis Thaliana] (GB:Q84	
JCVI_22937	2.140	highly similar to (625)AT1G04200| Symbols: | similar to hypothetical protein OsJ_003532 [Oryza sativa (japonica cultivar-group)] (GB:E	
EX036265	2.140	weakly similar to (124)AT3G49350| Symbols: | RAB GTPase activator | chr3:18308648-18310831 REVERSE [21811]	
JCVI_389	2.139	weakly similar to (115)AT5G47210| Symbols: | nuclear RNA-binding protein, putative | chr5:19186615-19188239 REVERSE no original	
JCVI_4077	2.139	moderately similar to (466)AT3G52180| Symbols: ATPTPKIS1, DSP4, SEX4 | ATPTPKIS1/DSP4/SEX4 (STARCH-EXCESS 4); protein	
JCVI_9725	2.139	weakly similar to (127)AT5G59970| Symbols: | histone H4 | chr5:24163578-24163889 REVERSEweakly similar to (127)H43_MAIZE nc	
JCVI_23231	2.139	highly similar to (525)AT3G11670| Symbols: DGD1 | DGD1 (DIGALACTOSYL DIACYLGLYCEROL DEFICIENT 1); galactolipid gala	
JCVI_8080	2.138	moderately similar to (247)AT3G11230| Symbols: | yippee family protein | chr3:3516689-3518199 FORWARDweakly similar to (173)YI	
EE557329	2.138	no similarity	
EE435362	2.138	no similarity	
JCVI_27512	2.138	moderately similar to (362)AT5G03080| Symbols: | phosphatidic acid phosphatase-related / PAP2-related | chr5:721975-722655 FORWA	
JCVI_40444	2.138	moderately similar to (340)AT5G24000| Symbols: | similar to unknown protein [Arabidopsis thaliana] (TAIR:AT5G52540.1); similar to h	
EV183277	2.138	moderately similar to (333)AT3G58620| Symbols: TTL4 | TTL4 (TETRATRICOPETIDE-REPEAT THIOREDOXIN-LIKE 4); binding | c	
JCVI_11727	2.138	moderately similar to (414)AT2G21520| Symbols: | similar to SEC14 cytosolic factor, putative / phosphoglyceride transfer protein, putativ	
CD837356	2.138	no similarity	
CN827085	2.137	moderately similar to (438)AT1G19300| Symbols: GATL1, PARVUS, GLZ1 | GATL1/GLZ1/PARVUS (GALACTURONOSYLTRANSFI	
EH415978	2.137	moderately similar to (306)AT1G08465| Symbols: YAB2 | YAB2 (YABBY 2); transcription factor | chr1:2676030-2679385 FORWARDw	
CX188279	2.137	no similarity	
JCVI_7507	2.137	moderately similar to (344)AT2G43050| Symbols: ATPMEPCRD | ATPMEPCRD; pectinesterase | chr2:17909585-17911251 FORWARD	3.455
JCVI_18979	2.136	weakly similar to (156)AT3G60470| Symbols: | similar to unknown protein [Arabidopsis thaliana] (TAIR:AT2G44930.1); similar to hypot	
ES945217	2.136	very weakly similar to (87.0)AT5G03160| Symbols: | DNAJ heat shock N-terminal domain-containing protein | chr5:750285-752670 FORV	
EX087642	2.136	weakly similar to (158)AT4G01500| Symbols: NGA4 | NGA4 (NGATHA4); transcription factor | chr4:639806-640792 FORWARD [2182	
CX193997	2.136	very weakly similar to (92.0)AT4G00430| Symbols: PIP1;4, PIP1E, TMP-C | TMP-C (PLASMA MEMBRANE INTRINSIC PROTEIN 1;4	
JCVI_31341	2.136	weakly similar to (124)AT5G15400| Symbols: | U-box domain-containing protein | chr5:4997767-5002910 REVERSE no original descript	-1.507
BG544889	2.136	weakly similar to (181)AT3G09150| Symbols: GUN3, HY2 | HY2 (ELONGATED HYPOCOTYL 2); phytochromobilin:ferredoxin oxido	1.824
JCVI_35392	2.136	weakly similar to (150)AT1G71460| Symbols: | pentatricopeptide (PPR) repeat-containing protein | chr1:26931909-26933978 REVERSE	
JCVI_6253	2.136	moderately similar to (316)AT5G58540| Symbols: | protein kinase family protein | chr5:23681132-23682408 FORWARD no original desc	
EE457804	2.136	no similarity	
JCVI_5828	2.135	moderately similar to (428)AT4G33970| Symbols: | leucine-rich repeat family protein / extensin family protein | chr4:16279800-1628189	
CD813352	2.135	moderately similar to (263)AT4G29680| Symbols: | type I phosphodiesterase/nucleotide pyrophosphatase family protein | chr4:14538073-	-1.749
JCVI_23489	2.135	moderately similar to (355)AT2G17980| Symbols: ATSLY1 | ATSLY1; protein transporter | chr2:7831434-7833486 FORWARDmoderate	
JCVI_4236	2.134	moderately similar to (355)AT4G39470| Symbols: | chloroplast lumen common family protein | chr4:18359729-18361159 REVERSE no c	
JCVI_22685	2.134	moderately similar to (490)AT1G52260| Symbols: ATPDIL1-5 | ATPDIL1-5 (PDI-LIKE 1-5); thiol-disulfide exchange intermediate | chr1	
JCVI_5385	2.134	very weakly similar to (100)AT2G19270| Symbols: | similar to unnamed protein product [Vitis vinifera] (GB:CAO69581.1) | chr2:836743	
EE503065	2.134	no similarity	
JCVI_35775	2.133	very weakly similar to (97.1)AT1G60680| Symbols: AGD2 | AGD2 (ARF-GAP DOMAIN 2); aldo-keto reductase | chr1:22351489-2235290	
JCVI_9060	2.133	moderately similar to (376)AT5G05580| Symbols: SH1, FAD8 | FAD8 (FATTY ACID DESATURASE 8); omega-3 fatty acid desaturase |	
ES900309	2.133	moderately similar to (324)AT1G48560| Symbols: | unknown protein | chr1:17956603-17958776 FORWARD [21428]	
EX137376	2.133	weakly similar to (129)AT4G39220| Symbols: ATRER1A | ATRER1A (Arabidopsis thaliana endoplasmic reticulum retrieval protein 1A) |	
JCVI_13552	2.133	no original description	-1.944
EX133093	2.133	highly similar to (523)AT3G22400| Symbols: LOX5 | LOX5; lipoxygenase | chr3:7927018-7931174 FORWARDmoderately similar to (36	
JCVI_36656	2.133	moderately similar to (252)AT3G58030| Symbols: | zinc finger (C3HC4-type RING finger) family protein | chr3:21496504-21497814 FOI	
EL591208	2.132	no similarity	
RC_T18372	2.132	no similarity	
ES926626	2.132	moderately similar to (204)AT1G71200| Symbols: | basic helix-loop-helix (bHLH) family protein | chr1:26840203-26840970 REVERSE [	
JCVI_9220	2.131	moderately similar to (402)AT5G15640| Symbols: | mitochondrial substrate carrier family protein | chr5:5087593-5089680 FORWARD no	
JCVI_11070	2.131	moderately similar to (431)AT1G74910| Symbols: | ADP-glucose pyrophosphorylase family protein | chr1:28139431-28142117 REVERSI	
EE566565	2.131	no similarity	
JCVI_21272	2.131	moderately similar to (263)AT3G54170| Symbols: FIP37, ATFIP37 | ATFIP37 (ARABIDOPSIS THALIANA FKBP12 INTERACTING P	
ES984346	2.131	weakly similar to (188)AT1G12750| Symbols: | rhomboid family protein | chr1:4345098-4346686 REVERSE [21389]	
DY026772	2.131	weakly similar to (124)AT4G25870| Symbols: | similar to unknown protein [Arabidopsis thaliana] (TAIR:AT5G57270.3); similar to unkno	
JCVI_39459	2.131	moderately similar to (387)AT2G03510| Symbols: | band 7 family protein | chr2:1066714-1068931 FORWARD no original description	
JCVI_32196	2.131	no original description	
JCVI_38002	2.130	no original description	
JCVI_31282	2.130	highly similar to (761)AT2G26140| Symbols: FTSH4 | FTSH4 (FtsH protease 4); ATP-dependent peptidase/ ATPase/ metallopeptidase | cl	
EE535394	2.130	moderately similar to (244)AT1G06130| Symbols: GLX2-4 | GLX2-4 (GLYOXALASE 2-4); hydroxyacylglutathione hydrolase | chr1:1858	
JCVI_41281	2.130	moderately similar to (384)AT5G57270| Symbols: | similar to unknown protein [Arabidopsis thaliana] (TAIR:AT4G25870.1); similar to u	
EE458295	2.130	moderately similar to (252)AT2G17150| Symbols: | RWP-RK domain-containing protein | chr2:7475213-7477615 REVERSE [20179]	
JCVI_16531	2.130	highly similar to (721)AT4G25970| Symbols: PSD3 | PSD3 (PHOSPHATIDYLSERINE DECARBOXYLASE 3); calcium ion binding / ph	
JCVI_6129	2.130	moderately similar to (299)AT1G21000| Symbols: | zinc-binding family protein | chr1:7338002-7339077 FORWARD no original descripti	
DY014738	2.129	moderately similar to (227)AT1G07380| Symbols: | ceramidase family protein | chr1:2264827-2268304 REVERSE [18966] 24 519 519	
EV007966	2.129	moderately similar to (268)AT4G17950| Symbols: | DNA-binding family protein | chr4:9967307-9969019 REVERSE [21427]	
JCVI_5563	2.129	moderately similar to (214)AT4G16790| Symbols: | hydroxyproline-rich glycoprotein family protein | chr4:9451766-9453187 REVERSE r	
DY022607	2.128	moderately similar to (296)AT2G20370| Symbols: KAM1, MUR3 | KAM1/MUR3 (MURUS 3); catalytic/ transferase, transferring glycosy	2.168
JCVI_7957	2.128	moderately similar to (338)AT3G26630| Symbols: | pentatricopeptide (PPR) repeat-containing protein | chr3:9792809-9794176 REVERSI	
JCVI_31071	2.128	no original description	
JCVI_1498	2.128	moderately similar to (452)AT5G05600| Symbols: | oxidoreductase, 2OG-Fe(II) oxygenase family protein | chr5:1672267-1674603 FORW	
EE434801	2.128	moderately similar to (364)AT3G11540| Symbols: SPY | SPY (SPINDLY) | chr3:3632848-3637553 FORWARDmoderately similar to (33	
JCVI_343	2.128	highly similar to (506)AT5G02870| Symbols: | 60S ribosomal protein L4/L1 (RPL4D) | chr5:657828-659524 FORWARD no original desc	
EV073580	2.127	moderately similar to (249)AT3G60580| Symbols: | zinc finger (C2H2 type) family protein | chr3:22404982-22405848 FORWARD [2144	
JCVI_38206	2.127	weakly similar to (124)AT5G20720| Symbols: CPN10, CHCPN10, ATCPN21, CPN21, CPN20 | CPN20 (CHAPERONIN 20); calmodulin	
CX193065	2.127	no similarity	
CD827928	2.127	moderately similar to (375)AT1G60160| Symbols: | potassium transporter family protein | chr1:22191995-22195060 REVERSEmoderatel	
JCVI_1999	2.127	moderately similar to (419)AT5G08620| Symbols: STRS2 | STRS2 (STRESS RESPONSE SUPPRESSOR 2); ATP-dependent helicase | ch	
JCVI_35621	2.127	weakly similar to (157)AT3G63290| Symbols: | similar to unknown protein [Arabidopsis thaliana] (TAIR:AT4G13400.1); similar to unnar	
RC_EV011846	2.127	no similarity	
JCVI_35502	2.127	moderately similar to (390)AT2G47130| Symbols: | short-chain dehydrogenase/reductase (SDR) family protein | chr2:19356697-1935755	
JCVI_24613	2.126	moderately similar to (464)AT2G42400| Symbols: | similar to unknown protein [Arabidopsis thaliana] (TAIR:AT1G28520.2); similar to u	

JCVI_6069	2.126	moderately similar to (436)AT4G34200| Symbols: EDA9 | EDA9 (embryo sac development arrest 9); NAD binding / amino acid binding /	
JCVI_26149	2.126	weakly similar to (176)AT1G13830| Symbols: | beta-1,3-glucanase-related | chr1:4739996-4740923 REVERSE no original description	-1.313
JCVI_14282	2.126	moderately similar to (326)AT3G27050| Symbols: | similar to unnamed protein product [Vitis vinifera] (GB:CAO49017.1) | chr3:9979523	
JCVI_4518	2.126	moderately similar to (340)AT4G02980| Symbols: ABP, ABP1 | ABP1 (ENDOPLASMIC RETICULUM AUXIN BINDING PROTEIN 1)	
AM060493	2.126	moderately similar to (375)AT5G42830| Symbols: | transferase family protein | chr5:17193612-17195134 FORWARD [17712]	
JCVI_21335	2.126	weakly similar to (158)AT1G71980| Symbols: | protease-associated zinc finger (C3HC4-type RING finger) family protein | chr1:27101912	3.713
JCVI_12995	2.126	highly similar to (588)AT2G31390| Symbols: | pfkB-type carbohydrate kinase family protein | chr2:13390712-13393193 REVERSEmoder	
EV116653	2.126	weakly similar to (176)AT3G50070| Symbols: CYCD3;3 | CYCD3;3 (CYCLIN D3;3); cyclin-dependent protein kinase | chr3:18576305-18	
JCVI_13190	2.126	weakly similar to (115)AT2G31470| Symbols: | F-box family protein | chr2:13414569-13415732 REVERSE no original description	1.906
JCVI_7479	2.125	moderately similar to (261)AT1G21010| Symbols: | similar to unknown protein [Arabidopsis thaliana] (TAIR:AT1G76600.1); similar to u	2.527
ES979732	2.125	no similarity	
CV432258	2.125	no similarity	
JCVI_591	2.125	no original description	
EE446055	2.125	moderately similar to (276)AT5G23050| Symbols: | acyl-activating enzyme 17 (AAE17) | chr5:7731516-7735402 REVERSE [20201]	
JCVI_25804	2.125	moderately similar to (338)AT1G13090| Symbols: CYP71B28 | CYP71B28 (cytochrome P450, family 71, subfamily B, polypeptide 28); o	
JCVI_2175	2.125	moderately similar to (450)AT5G09650| Symbols: ATPPA6 | ATPPA6 (ARABIDOPSIS THALIANA PYROPHOSPHORYLASE 6); inorg	
JCVI_7453	2.124	moderately similar to (345)AT1G75270| Symbols: DHAR2 | DHAR2; glutathione dehydrogenase (ascorbate) | chr1:28253916-28254898 R	
JCVI_16288	2.124	moderately similar to (253)AT1G27600| Symbols: | glycosyl transferase family 43 protein | chr1:9604070-9605868 REVERSE no original	
JCVI_2268	2.123	weakly similar to (142)AT1G76140| Symbols: | similar to prolyl oligopeptidase, putative / prolyl endopeptidase, putative / post-proline cle	
EV219315	2.123	moderately similar to (322)AT4G26140| Symbols: BGAL12 | BGAL12 (beta-galactosidase 12); beta-galactosidase | chr4:13243683-13247	
CN727520	2.123	weakly similar to (129)AT1G75690| Symbols: | chaperone protein dnaJ-related | chr1:28425934-28426831 REVERSE [15722]	
JCVI_5539	2.123	moderately similar to (281)AT5G67150| Symbols: | transferase family protein | chr5:26813106-26814452 REVERSE no original descripti	
JCVI_37043	2.123	very weakly similar to (80.9)AT1G18450| Symbols: ARP4, ATARP4 | ATARP4 (ACTIN-RELATED PROTEIN 4); structural constituent c	
RC_JCVI_21968	2.123	no original description	
EE547409	2.122	no similarity	
EV151501	2.122	weakly similar to (126)AT3G51690| Symbols: | DNA helicase homolog PIF1. | chr3:19187710-19189086 REVERSE [21483] 38 741 741	
H07297	2.122	no similarity	
JCVI_36639	2.122	weakly similar to (170)AT3G07850| Symbols: | exopolygalacturonase / galacturan 1,4-alpha-galacturonidase / pectinase | chr3:2505819-25	
CN731378	2.122	no similarity	
EV114165	2.121	weakly similar to (134)AT1G08680| Symbols: AGD14, ZIGA4 | ZIGA4 (ARF GAP-LIKE ZINC FINGER-CONTAINING PROTEIN ZIG	
ES903501	2.121	moderately similar to (239)AT1G32360| Symbols: | zinc finger (CCCH-type) family protein | chr1:11673305-11675142 FORWARD [214	
JCVI_20288	2.120	moderately similar to (362)AT5G63100| Symbols: | similar to unknown protein [Arabidopsis thaliana] (TAIR:AT5G44600.1); similar to u	
DY025265	2.120	weakly similar to (179)AT2G39890| Symbols: ATPROT1, ProT1 | ProT1 (PROLINE TRANSPORTER 1) | chr2:16663100-16665280 FOI	-1.908
JCVI_28525	2.120	moderately similar to (302)AT3G22850| Symbols: | similar to unknown protein [Arabidopsis thaliana] (TAIR:AT5G43830.1); similar to h	
EV168076	2.119	no similarity	
ES966261	2.119	no similarity	
EX058742	2.119	no similarity	2.035
CV433836	2.119	no similarity	
CD817301	2.119	weakly similar to (196)AT2G26680| Symbols: | similar to unnamed protein product [Vitis vinifera] (GB:CAO65660.1); contains InterPro	
JCVI_1448	2.119	moderately similar to (435)AT5G48850| Symbols: | male sterility MS5 family protein | chr5:19822802-19824925 REVERSE no original d	
JCVI_14563	2.118	moderately similar to (361)AT1G18980| Symbols: | germin-like protein, putative | chr1:6557355-6558017 REVERSEweakly similar to (1	
EE418768	2.118	very weakly similar to (99.8)AT3G22190| Symbols: IQD5 | IQD5 (IQ-domain 5); calmodulin binding | chr3:7831668-7833519 REVERSE [	
JCVI_31960	2.118	moderately similar to (468)AT3G51640| Symbols: | similar to unknown protein [Arabidopsis thaliana] (TAIR:AT3G51650.1); similar to u	
L46436	2.118	no similarity	
JCVI_18811	2.117	moderately similar to (274)AT2G41160| Symbols: | ubiquitin-associated (UBA)/TS-N domain-containing protein | chr2:17163392-171657	
JCVI_12901	2.117	weakly similar to (115)AT2G46250| Symbols: | myosin heavy chain-related | chr2:18998459-19000274 FORWARD no original descriptio	
JCVI_7328	2.116	moderately similar to (390)AT5G25757| Symbols: | similar to unknown protein [Arabidopsis thaliana] (TAIR:AT5G25754.1); similar to p	
EV160842	2.116	moderately similar to (454)AT3G08850| Symbols: RAPTOR1B, ATRAPTOR1B | ATRAPTOR1B/RAPTOR1B (RAPTOR1); nucleotide b	
EV042418	2.116	weakly similar to (184)AT3G57040| Symbols: ATRR4, ARR9 | ARR9 (RESPONSE REACTOR 4); transcription regulator | chr3:2112103	
JCVI_34656	2.116	moderately similar to (327)AT3G62940| Symbols: | OTU-like cysteine protease family protein | chr3:23274081-23275220 REVERSE no	
JCVI_3107	2.115	moderately similar to (392)AT5G34850| Symbols: ATPAP26, PAP26 | ATPAP26/PAP26 (purple acid phosphatase 26); acid phosphatase/	
CD827541	2.115	moderately similar to (304)AT5G09880| Symbols: | RNA recognition motif (RRM)-containing protein | chr5:3081647-3085180 REVERSI	
ES966409	2.115	no similarity	
JCVI_9939	2.115	moderately similar to (258)AT1G67250| Symbols: | proteasome maturation factor UMP1 family protein | chr1:25167471-25168630 REVE	
JCVI_13255	2.115	moderately similar to (374)AT1G78180| Symbols: | binding | chr1:29421812-29423418 FORWARD no original description	
JCVI_4385	2.114	moderately similar to (380)AT1G14620| Symbols: DECOY | DECOY (endoxyloglucan transferase A2) | chr1:5014943-5016496 REVERS	
EV141180	2.114	weakly similar to (174)AT2G43410| Symbols: FPA | FPA | chr2:18033474-18038066 REVERSE [21482]	
EX062433	2.114	no similarity	
EE455805	2.114	moderately similar to (253)AT5G12960| Symbols: | catalytic | chr5:4097791-4101521 FORWARD [20178]	
JCVI_31839	2.114	highly similar to (561)AT3G26680| Symbols: SNM1 | SNM1 (SENSITIVE TO NITROGEN MUSTARD 1) | chr3:9802546-9804754 FOR	
JCVI_28164	2.114	moderately similar to (405)AT2G46500| Symbols: | phosphatidylinositol 3- and 4-kinase family protein / ubiquitin family protein | chr2:19	
EE472284	2.114	no similarity	
JCVI_13753	2.114	moderately similar to (312)AT4G31890| Symbols: | armadillo/beta-catenin repeat family protein | chr4:15427296-15429055 REVERSE no	
JCVI_22820	2.114	moderately similar to (258)AT3G21230| Symbols: 4CL5 | 4CL5 (4-COUMARATE:COA LIGASE 5); 4-coumarate-CoA ligase | chr3:7448	
JCVI_23917	2.114	weakly similar to (103)AT5G65020| Symbols: ANNAT2 | ANNAT2 (ANNEXIN ARABIDOPSIS 2); calcium ion binding / calcium-depen	
JCVI_42096	2.114	moderately similar to (236)AT1G78310| Symbols: | VQ motif-containing protein | chr1:29468896-29469831 REVERSE no original descri	
ES988933	2.113	no similarity	
CX268826	2.113	moderately similar to (249)AT2G26830| Symbols: EMB1187 | EMB1187 (EMBRYO DEFECTIVE 1187); kinase/ phosphotransferase, alc	
EV200901	2.113	moderately similar to (469)AT5G67030| Symbols: LOS6, NPQ2, ATABA1, ZEP, IBS3, ATZEP, ABA1 | ABA1 (ABA DEFICIENT 1); ze	
JCVI_2104	2.113	highly similar to (606)AT3G06410| Symbols: | nucleic acid binding | chr3:1947477-1949534 REVERSEmoderately similar to (301)ZFNL	
ES933020	2.113	weakly similar to (138)AT3G12685| Symbols: | similar to catalytic [Arabidopsis thaliana] (TAIR:AT1G24350.1); similar to unknown [Po	-3.448
AM060929	2.112	moderately similar to (241)AT3G20570| Symbols: | plastocyanin-like domain-containing protein | chr3:7186760-7187459 REVERSE [177	
EE444404	2.112	weakly similar to (172)AT1G55915| Symbols: | zinc ion binding | chr1:20911328-20913094 FORWARD [20160]	
JCVI_365	2.112	moderately similar to (333)AT4G20330| Symbols: | transcription initiation factor-related | chr4:10982694-10984050 REVERSE no origina	
JCVI_344	2.112	moderately similar to (386)AT5G05780| Symbols: RPN8A, AE3, ATHMOV34 | AE3/ATHMOV34 (ASYMMETRIC LEAVES ENHANC	
EV146787	2.112	no similarity	
ES970970	2.112	no similarity	
EX105780	2.112	moderately similar to (262)AT1G08500| Symbols: | plastocyanin-like domain-containing protein | chr1:2689113-2689884 FORWARD [21	
JCVI_41705	2.111	moderately similar to (408)AT1G05540| Symbols: | similar to unknown protein [Arabidopsis thaliana] (TAIR:AT1G30160.2); contains In	
BG544270	2.111	weakly similar to (102)AT5G09590| Symbols: HSC70-5, mtHSC70-2 | mtHSC70-2 (HEAT SHOCK PROTEIN 70); ATP binding / unfold	
EE420195	2.111	very weakly similar to (91.3)AT2G04660| Symbols: APC2 | APC2 (anaphase-promoting complex/cyclosome 2); ubiquitin-protein ligase | cl	

<table>
<tr><th></th><th></th><th></th><th></th></tr>
<tr><td>JCVI_16327</td><td>2.111</td><td>moderately similar to (352)AT2G45900| Symbols: | similar to unknown protein [Arabidopsis thaliana] (TAIR:AT3G61380.1); similar to</td><td></td></tr>
<tr><td>JCVI_14984</td><td>2.111</td><td>moderately similar to (375)AT1G12350| Symbols: ATCOAB | ATCOAB (4-PHOSPHO-PANTO-THENOYLCYSTEINE SYNTHETASE)</td><td></td></tr>
<tr><td>JCVI_12481</td><td>2.111</td><td>moderately similar to (422)AT1G07670| Symbols: | calcium-transporting ATPase | chr1:2370302-2374193 REVERSE no original descript</td><td></td></tr>
<tr><td>JCVI_39409</td><td>2.111</td><td>no original description</td><td></td></tr>
<tr><td>JCVI_39434</td><td>2.110</td><td>weakly similar to (129)AT3G21000| Symbols: | similar to unknown protein [Arabidopsis thaliana] (TAIR:AT3G20980.1); similar to putati</td><td></td></tr>
<tr><td>AM385523</td><td>2.110</td><td>moderately similar to (239)AT1G80460| Symbols: GLI1, NHO1 | NHO1 (NONHOST RESISTANCE TO P. S. PHASEOLICOLA 1); carb</td><td></td></tr>
<tr><td>JCVI_39385</td><td>2.110</td><td>moderately similar to (235)AT5G02770| Symbols: | similar to unnamed protein product [Vitis vinifera] (GB:CAO18065.1) | chr5:628099-</td><td></td></tr>
<tr><td>AM395565</td><td>2.110</td><td>very weakly similar to (85.5)AT5G08520| Symbols: | myb family transcription factor | chr5:2755471-2757742 REVERSE [20346]</td><td></td></tr>
<tr><td>JCVI_4978</td><td>2.110</td><td>moderately similar to (313)AT3G51100| Symbols: | similar to Os06g0713900 [Oryza sativa (japonica cultivar-group)] (GB:NP_00105856</td><td></td></tr>
<tr><td>EV218973</td><td>2.110</td><td>very weakly similar to (86.7)AT5G54160| Symbols: OMT1, ATOMT1 | ATOMT1 (O-METHYLTRANSFERASE 1) | chr5:21999301-2200</td><td></td></tr>
<tr><td>EX141526</td><td>2.110</td><td>very weakly similar to (84.7)AT3G03570| Symbols: | similar to signal transducer [Arabidopsis thaliana] (TAIR:AT4G40050.1); similar to</td><td></td></tr>
<tr><td>EE467329</td><td>2.110</td><td>moderately similar to (241)AT4G02920| Symbols: | similar to unknown protein [Arabidopsis thaliana] (TAIR:AT1G03340.1); similar to h</td><td></td></tr>
<tr><td>EE419372</td><td>2.109</td><td>very weakly similar to (100)AT5G27970| Symbols: | binding | chr5:10004724-10015433 FORWARD [20146]</td><td></td></tr>
<tr><td>EE516168</td><td>2.109</td><td>moderately similar to (270)AT1G04850| Symbols: | ubiquitin-associated (UBA)/TS-N domain-containing protein | chr1:1365310-1368705</td><td></td></tr>
<tr><td>DY010262</td><td>2.109</td><td>very weakly similar to (82.4)AT5G50190| Symbols: | similar to EMB1353 (EMBRYO DEFECTIVE 1353) [Arabidopsis thaliana] (TAIR:</td><td></td></tr>
<tr><td>JCVI_16726</td><td>2.109</td><td>moderately similar to (358)AT1G72770| Symbols: HAB1 | HAB1 (HOMOLOGY TO ABI1) | chr1:27394660-27396075 FORWARD no or</td><td></td></tr>
<tr><td>JCVI_30136</td><td>2.109</td><td>moderately similar to (311)AT3G24150| Symbols: | similar to unknown protein [Arabidopsis thaliana] (TAIR:AT4G32295.1); similar to u</td><td></td></tr>
<tr><td>EV143962</td><td>2.109</td><td>weakly similar to (108)AT2G03470| Symbols: | myb family transcription factor / ELM2 domain-containing protein | chr2:1045691-104712</td><td></td></tr>
<tr><td>JCVI_15352</td><td>2.109</td><td>moderately similar to (417)AT2G02810| Symbols: ATUTR1, UTR1 | ATUTR1/UTR1 (UDP-GALACTOSE TRANSPORTER 1); UDP-ga</td><td></td></tr>
<tr><td>JCVI_3270</td><td>2.108</td><td>moderately similar to (263)AT3G18690| Symbols: MKS1 | MKS1 (MAP KINASE SUBSTRATE 1) | chr3:6429761-6430429 REVERSE n</td><td>2.963</td></tr>
<tr><td>JCVI_4230</td><td>2.108</td><td>no original description</td><td></td></tr>
<tr><td>EE560146</td><td>2.108</td><td>weakly similar to (103)AT3G10915| Symbols: | reticulon family protein | chr3:3416106-3417502 REVERSE [20153] 21 581 624</td><td></td></tr>
<tr><td>JCVI_38885</td><td>2.108</td><td>highly similar to (825)AT1G26850| Symbols: | dehydration-responsive family protein | chr1:9301133-9303419 REVERSE no original desc</td><td></td></tr>
<tr><td>CX271340</td><td>2.108</td><td>moderately similar to (324)AT1G80770| Symbols: PDE318 | PDE318 (PIGMENT DEFECTIVE 318); GTP binding | chr1:30360158-3036</td><td></td></tr>
<tr><td>EV147708</td><td>2.108</td><td>weakly similar to (168)AT1G70580| Symbols: GGT2, AOAT2 | AOAT2 (GLUTAMATE:GLYOXYLATE AMINOTRANSFERASE 2); al</td><td></td></tr>
<tr><td>JCVI_18581</td><td>2.108</td><td>weakly similar to (181)AT3G02310| Symbols: AGL4, SEP2 | SEP2 (SEPALLATA2); DNA binding / transcription factor | chr3:464561-46</td><td></td></tr>
<tr><td>DN237913</td><td>2.107</td><td>no similarity</td><td></td></tr>
<tr><td>JCVI_11270</td><td>2.107</td><td>moderately similar to (457)AT3G13330| Symbols: | binding | chr3:4319811-4330068 REVERSE no original description</td><td></td></tr>
<tr><td>JCVI_12243</td><td>2.107</td><td>weakly similar to (124)AT3G47070| Symbols: | similar to unknown [Populus trichocarpa] (GB:ABK95428.1) | chr3:17348190-17348492</td><td></td></tr>
<tr><td>JCVI_27824</td><td>2.107</td><td>weakly similar to (135)AT4G32600| Symbols: | zinc finger (C3HC4-type RING finger) family protein | chr4:15724016-15725743 FORW</td><td></td></tr>
<tr><td>JCVI_21261</td><td>2.107</td><td>very weakly similar to (85.1)AT1G14800| Symbols: | similar to F-box family protein [Arabidopsis thaliana] (TAIR:AT2G35280.1); similar</td><td></td></tr>
<tr><td>JCVI_14930</td><td>2.107</td><td>weakly similar to (153)AT2G41870| Symbols: | remorin family protein | chr2:17478197-17479597 REVERSE no original description</td><td></td></tr>
<tr><td>DY023190</td><td>2.107</td><td>weakly similar to (160)AT2G02870| Symbols: | kelch repeat-containing F-box family protein | chr2:838377-839780 FORWARD [18979]</td><td></td></tr>
<tr><td>JCVI_833</td><td>2.107</td><td>moderately similar to (499)AT1G78850| Symbols: | curculin-like (mannose-binding) lectin family protein | chr1:29646965-29648290 REV</td><td></td></tr>
<tr><td>JCVI_24216</td><td>2.106</td><td>weakly similar to (174)AT3G21140| Symbols: | FMN binding | chr3:7409702-7412092 REVERSE no original description</td><td></td></tr>
<tr><td>JCVI_27293</td><td>2.106</td><td>highly similar to (577)AT3G21760| Symbols: | UDP-glucoronosyl/UDP-glucosyl transferase family protein | chr3:7667106-7668563 FOR</td><td></td></tr>
<tr><td>JCVI_4584</td><td>2.106</td><td>weakly similar to (197)AT4G28140| Symbols: | AP2 domain-containing transcription factor, putative | chr4:13974911-13975789 REVERS</td><td></td></tr>
<tr><td>EV045942</td><td>2.106</td><td>weakly similar to (120)AT1G16680| Symbols: | DNAJ heat shock N-terminal domain-containing protein / S-locus protein, putative | chr1:</td><td></td></tr>
<tr><td>EV160420</td><td>2.106</td><td>weakly similar to (168)AT4G31420| Symbols: | zinc finger (C2H2 type) family protein | chr4:15245993-15247769 REVERSE [21484] 52</td><td></td></tr>
<tr><td>JCVI_8369</td><td>2.106</td><td>moderately similar to (276)AT5G62440| Symbols: | Encodes a protein DOMINO1 that belongs to a plant-specific gene family sharing a co</td><td></td></tr>
<tr><td>JCVI_30497</td><td>2.105</td><td>moderately similar to (329)AT5G12410| Symbols: | THUMP domain-containing protein | chr5:4021975-4023951 REVERSE no original d</td><td></td></tr>
<tr><td>JCVI_22653</td><td>2.105</td><td>moderately similar to (301)AT2G48020| Symbols: | sugar transporter, putative | chr2:19651511-19654077 FORWARD no original descrip</td><td></td></tr>
<tr><td>EV210637</td><td>2.105</td><td>weakly similar to (160)AT3G18290| Symbols: EMB2454 | EMB2454 (EMBRYO DEFECTIVE 2454); protein binding / zinc ion binding |</td><td>1.480</td></tr>
<tr><td>JCVI_27406</td><td>2.105</td><td>weakly similar to (127)AT5G51180| Symbols: | similar to unknown protein [Arabidopsis thaliana] (TAIR:AT4G25770.1); similar to unkn</td><td></td></tr>
<tr><td>CX188570</td><td>2.105</td><td>moderately similar to (201)AT3G02420| Symbols: | similar to hypothetical protein [Cleome spinosa] (GB:ABD96906.1) | chr3:496186-49</td><td></td></tr>
<tr><td>JCVI_14916</td><td>2.105</td><td>highly similar to (526)AT5G50960| Symbols: | nucleotide-binding family protein | chr5:20751493-20753050 FORWARD no original desc</td><td>-2.491</td></tr>
<tr><td>JCVI_28604</td><td>2.105</td><td>highly similar to (576)AT3G63380| Symbols: | calcium-transporting ATPase, plasma membrane-type, putative / Ca(2+)-ATPase, putative</td><td></td></tr>
<tr><td>JCVI_14194</td><td>2.105</td><td>moderately similar to (450)AT1G19270| Symbols: | ubiquitin interaction motif-containing protein / LIM domain-containing protein | chr1:</td><td>-2.745</td></tr>
<tr><td>JCVI_11416</td><td>2.105</td><td>weakly similar to (169)AT1G53000| Symbols: | cytidylyltransferase family | chr1:19748998-19750801 REVERSE no original description</td><td></td></tr>
<tr><td>JCVI_35785</td><td>2.105</td><td>weakly similar to (122)AT3G61530| Symbols: PANB2 | PANB2; 3-methyl-2-oxobutanoate hydroxymethyltransferase | chr3:22782667-227</td><td></td></tr>
<tr><td>EG020087</td><td>2.104</td><td>no similarity</td><td></td></tr>
<tr><td>JCVI_6911</td><td>2.104</td><td>moderately similar to (441)AT1G53580| Symbols: GLX2-3, ETHE1, GLY3 | ETHE1/GLX2-3/GLY3 (GLYOXALASE 2-3); hydroxyacylg</td><td></td></tr>
<tr><td>JCVI_27082</td><td>2.104</td><td>highly similar to (569)AT4G11380| Symbols: | beta-adaptin, putative | chr4:6920603-6925439 FORWARD no original description</td><td></td></tr>
<tr><td>JCVI_28824</td><td>2.104</td><td>highly similar to (748)AT2G39940| Symbols: COI1 | COI1 (CORONATINE INSENSITIVE 1); ubiquitin-protein ligase | chr2:16679926-1</td><td></td></tr>
<tr><td>JCVI_22249</td><td>2.104</td><td>highly similar to (516)AT1G72650| Symbols: TRFL6 | TRFL6 (TRF-LIKE 6); DNA binding / transcription factor | chr1:27353915-273571</td><td></td></tr>
<tr><td>BQ790913</td><td>2.104</td><td>weakly similar to (112)AT5G57610| Symbols: | protein kinase family protein | chr5:23342533-23346325 FORWARD [8791]</td><td></td></tr>
<tr><td>JCVI_12623</td><td>2.104</td><td>moderately similar to (295)AT4G14090| Symbols: | UDP-glucoronosyl/UDP-glucosyl transferase family protein | chr4:8122429-8123799</td><td></td></tr>
<tr><td>JCVI_13529</td><td>2.104</td><td>moderately similar to (297)AT2G30700| Symbols: | similar to unknown protein [Arabidopsis thaliana] (TAIR:AT1G61900.1); similar to h</td><td></td></tr>
<tr><td>EE548132</td><td>2.103</td><td>weakly similar to (126)AT2G45950| Symbols: ASK20 | ASK20 (ARABIDOPSIS SKP1-LIKE 20) | chr2:18911746-18914040 REVERSE [</td><td>-5.194</td></tr>
<tr><td>JCVI_18914</td><td>2.103</td><td>no original description</td><td></td></tr>
<tr><td>JCVI_28986</td><td>2.103</td><td>moderately similar to (356)AT4G03400| Symbols: GH3-10, DFL2 | DFL2 (DWARF IN LIGHT 2) | chr4:1497674-1499728 REVERSEmo</td><td></td></tr>
<tr><td>EE454424</td><td>2.103</td><td>moderately similar to (314)AT3G25800| Symbols: PR 65, PR65, PDF1 | PDF1 (65 KDA REGULATORY SUBUNIT OF PROTEIN PHO</td><td></td></tr>
<tr><td>JCVI_27107</td><td>2.103</td><td>moderately similar to (299)AT5G18070| Symbols: | DRT101 (DNA-DAMAGE-REPAIR/TOLERATION 101); intramolecular transferase</td><td></td></tr>
<tr><td>JCVI_14193</td><td>2.103</td><td>weakly similar to (191)AT2G01190| Symbols: | octicosapeptide/Phox/Bem1p (PB1) domain-containing protein | chr2:115022-117295 FO</td><td></td></tr>
<tr><td>JCVI_20527</td><td>2.103</td><td>highly similar to (669)AT1G49040| Symbols: SCD1 | SCD1 (STOMATAL CYTOKINESIS-DEFECTIVE 1) | chr1:18145675-18152494 R</td><td></td></tr>
<tr><td>BG543306</td><td>2.102</td><td>weakly similar to (147)AT2G33430| Symbols: | plastid developmental protein DAG, putative | chr2:14169808-14171805 FORWARD [87</td><td></td></tr>
<tr><td>DY026413</td><td>2.102</td><td>moderately similar to (223)AT5G28530| Symbols: FRS10 | FRS10 (FAR1-RELATED SEQUENCE 10); zinc ion binding | chr5:10525082-</td><td></td></tr>
<tr><td>EE525041</td><td>2.102</td><td>moderately similar to (283)AT1G06380| Symbols: | ribosomal protein-related | chr1:1945123-1945887 FORWARD [20143]</td><td></td></tr>
<tr><td>AM390276</td><td>2.102</td><td>no similarity</td><td></td></tr>
<tr><td>JCVI_7028</td><td>2.102</td><td>moderately similar to (308)AT4G36910| Symbols: LEJ2 | LEJ2 (LOSS OF THE TIMING OF ET AND JA BIOSYNTHESIS 2) | chr4:173</td><td></td></tr>
<tr><td>EE421187</td><td>2.102</td><td>moderately similar to (276)AT4G34412| Symbols: | similar to unnamed protein product [Vitis vinifera] (GB:CAO47805.1); contains Inter</td><td></td></tr>
<tr><td>EX091311</td><td>2.101</td><td>weakly similar to (104)AT4G39080| Symbols: VHA-A3 | VHA-A3 (VACUOLAR PROTON ATPASE A3); ATPase | chr4:18209507-1821</td><td></td></tr>
<tr><td>JCVI_31099</td><td>2.101</td><td>moderately similar to (481)AT5G17380| Symbols: | pyruvate decarboxylase family protein | chr5:5724922-5726722 REVERSEweakly sim</td><td></td></tr>
<tr><td>JCVI_15446</td><td>2.101</td><td>moderately similar to (432)AT3G55040| Symbols: | In2-1 protein, putative | chr3:20409695-20411282 REVERSEmoderately similar to (</td><td></td></tr>
<tr><td>JCVI_19965</td><td>2.101</td><td>moderately similar to (252)AT1G74020| Symbols: SS2 | SS2 (STRICTOSIDINE SYNTHASE 2); strictosidine synthase | chr1:27838950-2</td><td></td></tr>
<tr><td>JCVI_42235</td><td>2.101</td><td>moderately similar to (399)AT3G06980| Symbols: | DEAD/DEAH box helicase, putative | chr3:2201537-2204668 FORWARD no original</td><td></td></tr>
<tr><td>EV112610</td><td>2.100</td><td>weakly similar to (114)AT1G73750| Symbols: | similar to unknown protein [Arabidopsis thaliana] (TAIR:AT1G15060.1); similar to unna</td><td></td></tr>
<tr><td>JCVI_21578</td><td>2.099</td><td>weakly similar to (165)AT5G51860| Symbols: | MADS-box protein (AGL72) | chr5:21099070-21101352 REVERSEweakly similar to (10</td><td></td></tr>
<tr><td>RC_ES977217</td><td>2.099</td><td>no similarity</td><td></td></tr>
<tr><td>EV194950</td><td>2.099</td><td>weakly similar to (197)AT5G38530| Symbols: | tryptophan synthase-related | chr5:15441327-15443524 FORWARD [21489] 30 745 745</td><td></td></tr>
<tr><td>JCVI_13676</td><td>2.099</td><td>moderately similar to (328)AT1G45976| Symbols: SBP1 | SBP1 (S-RIBONUCLEASE BINDING PROTEIN 1); protein binding / zinc ion</td><td></td></tr>
</table>

JCVI_13477	2.099	moderately similar to (279)AT3G63460| Symbols: | WD-40 repeat family protein | chr3:23441984-23448216 REVERSE no original descr	
JCVI_25306	2.099	weakly similar to (118)AT5G42940| Symbols: | zinc finger (C3HC4-type RING finger) family protein | chr5:17233877-17236399 REVER	-1.804
JCVI_367	2.099	moderately similar to (306)AT1G78300| Symbols: GF14 OMEGA, GRF2 | GRF2 (GENERAL REGULATORY FACTOR 2); protein phos	
JCVI_12604	2.099	weakly similar to (190)AT2G21960| Symbols: | similar to unknown protein [Arabidopsis thaliana] (TAIR:AT1G56180.1); similar to unna	
JCVI_42400	2.098	highly similar to (814)AT3G12980| Symbols: HAC5, ATHPCAT4 | ATHPCAT4/HAC5 (HISTONE ACETYLTRANSFERASE OF THE C	
JCVI_19158	2.098	no original description	
JCVI_2197	2.098	moderately similar to (281)AT5G20190| Symbols: | binding | chr5:6814095-6815173 FORWARD no original description	
EE517285	2.098	moderately similar to (246)AT5G61210| Symbols: ATSNAP33, SNP33, ATSNAP33B, SNAP33 | SNAP33 (synaptosomal-associated prote	-1.850
EX130518	2.098	moderately similar to (212)AT1G47840| Symbols: | hexokinase, putative | chr1:17618683-17621299 REVERSEweakly similar to (158)H	
ES953099	2.098	moderately similar to (330)AT5G57300| Symbols: | UbiE/COQ5 methyltransferase family protein | chr5:23225931-23227837 REVERSE |	
EX094629	2.098	moderately similar to (275)AT1G60360| Symbols: | zinc finger (C3HC4-type RING finger) family protein | chr1:22246413-22247396 RE	
DN961678	2.097	moderately similar to (325)AT1G78900| Symbols: VHA-A | VHA-A; ATP binding / hydrogen ion transporting ATP synthase, rotational m	
EX103795	2.097	moderately similar to (391)AT5G45275| Symbols: | similar to nodulin-related [Arabidopsis thaliana] (TAIR:AT4G19450.1); similar to NF	-1.056
EX043880	2.097	no similarity	
EE502966	2.097	no similarity	
EE524518	2.097	moderately similar to (359)AT3G54720| Symbols: COP2, HPT, PT, AMP1 | AMP1 (ALTERED MERISTEM PROGRAM 1); dipeptidase	
ES968928	2.097	no similarity	
JCVI_23254	2.097	moderately similar to (432)AT1G10410| Symbols: | similar to CW14 [Arabidopsis thaliana] (TAIR:AT1G59650.1); similar to expressed p	
EE567494	2.097	very weakly similar to (83.2)AT4G34670| Symbols: | 40S ribosomal protein S3A (RPS3aB) | chr4:16548729-16550227 FORWARD [2015	
EE532069	2.097	moderately similar to (400)AT5G03340| Symbols: | (Cell division control protein 48 homolog E); ATPase | chr5:810090-813132 REVERS	
JCVI_37171	2.096	weakly similar to (164)AT2G35240| Symbols: | plastid developmental protein DAG, putative | chr2:14852178-14853341 REVERSE no or	
JCVI_10201	2.096	moderately similar to (446)AT1G74260| Symbols: | catalytic | chr1:27926666-27931425 REVERSE no original description	
EV121238	2.095	moderately similar to (340)AT1G54710| Symbols: AtATG18h | AtATG18h (Arabidopsis thaliana homolog of yeast autophagy 18 (ATG18	
JCVI_16149	2.095	moderately similar to (472)AT1G10290| Symbols: DRP2A, ADL6 | ADL6 (DYNAMIN-LIKE PROTEIN 6) | chr1:3370776-3377122 FOR	
JCVI_20365	2.095	moderately similar to (214)AT4G33440| Symbols: | glycoside hydrolase family 28 protein / polygalacturonase (pectinase) family protein |	
JCVI_4793	2.095	moderately similar to (471)AT1G28340| Symbols: | leucine-rich repeat family protein | chr1:9940162-9943239 FORWARDvery weakly si	
EE410402	2.094	weakly similar to (194)AT5G62760| Symbols: | nuclear protein ZAP-related | chr5:25223400-25226619 REVERSE [20140]	
JCVI_25370	2.094	moderately similar to (211)AT2G22100| Symbols: | RNA recognition motif (RRM)-containing protein | chr2:9399477-9400625 REVERSI	
EV129112	2.094	no similarity	
JCVI_20036	2.093	moderately similar to (336)AT5G67610| Symbols: | similar to unknown protein [Arabidopsis thaliana] (TAIR:AT3G49840.1); similar to u	
EX094877	2.093	moderately similar to (267)AT5G47390| Symbols: | myb family transcription factor | chr5:19244228-19245773 FORWARD [21824] 16 7	
EL588515	2.093	moderately similar to (262)AT5G25190| Symbols: | ethylene-responsive element-binding protein, putative | chr5:8707010-8707658 REVE	
EH416010	2.092	moderately similar to (232)AT5G47070| Symbols: | protein kinase, putative | chr5:19135910-19137755 REVERSE [20767]	
EV021741	2.092	moderately similar to (247)AT5G04850| Symbols: VPS60.2 | VPS60.2 | chr5:1408247-1409807 REVERSE [21441]	1.407
JCVI_13511	2.092	moderately similar to (220)AT3G53400| Symbols: | similar to CPuORF47 (Conserved peptide upstream open reading frame 47) [Arabidop	
EV043086	2.091	weakly similar to (119)AT3G21710| Symbols: | unknown protein | chr3:7648387-7649540 FORWARD [21442]	
CB686397	2.091	no similarity	
JCVI_36957	2.091	weakly similar to (150)AT1G55910| Symbols: ZIP11 | ZIP11 (ZINC TRANSPORTER 11 PRECURSOR); cation transmembrane transport	
EH413816	2.091	moderately similar to (204)AT1G14870| Symbols: | Identical to Uncharacterized protein At1g14870 [Arabidopsis Thaliana] (GB:Q9LQU	
JCVI_16191	2.091	moderately similar to (353)AT1G07470| Symbols: | transcription factor IIA large subunit, putative / TFIIA large subunit, putative | chr1:2	
EE409569	2.091	moderately similar to (269)AT3G07310| Symbols: | similar to unknown protein [Arabidopsis thaliana] (TAIR:AT5G48590.1); similar to e	
JCVI_34086	2.091	moderately similar to (421)AT5G02270| Symbols: ATNAP9 | ATNAP9 (Non-intrinsic ABC protein 9) | chr5:467267-469039 REVERSE n	
JCVI_13166	2.091	moderately similar to (251)AT2G46890| Symbols: | oxidoreductase, acting on the CH-CH group of donors | chr2:19273948-19275203 RE	
JCVI_15320	2.090	no original description	
EX021402	2.090	weakly similar to (185)AT1G51610| Symbols: | cation efflux family protein / metal tolerance protein, putative (MTPc4) | chr1:19140293-1	
AM390139	2.090	moderately similar to (250)AT5G35200| Symbols: | epsin N-terminal homology (ENTH) domain-containing protein | chr5:13479693-1348	
JCVI_1062	2.090	highly similar to (530)AT2G18960| Symbols: PMA, OST2, AHA1 | AHA1 (ARABIDOPSIS H+ ATPASE 1); ATPase | chr2:8228940-823	
JCVI_3442	2.090	weakly similar to (137)AT4G15440| Symbols: CYP74B2, HPL1 | HPL1 (HYDROPEROXIDE LYASE 1); heme binding / iron ion binding	
CD822440	2.090	weakly similar to (135)AT2G24200| Symbols: | cytosol aminopeptidase | chr2:10294097-10296530 REVERSEweakly similar to (103)AM	
ES966078	2.089	no similarity	-1.597
JCVI_26922	2.089	moderately similar to (219)AT3G26510| Symbols: | octicosapeptide/Phox/Bem1p (PB1) domain-containing protein | chr3:9713123-97137	
EE423206	2.089	moderately similar to (266)AT5G53060| Symbols: | KH domain-containing protein | chr5:21532581-21535423 FORWARD [20146]	
EE455301	2.089	very weakly similar to (88.2)AT1G60990| Symbols: | similar to aminomethyltransferase, putative [Arabidopsis thaliana] (TAIR:AT1G118	
EX091728	2.088	moderately similar to (260)AT5G21900| Symbols: | similar to unknown protein [Arabidopsis thaliana] (TAIR:AT2G06040.1); similar to u	
JCVI_40059	2.088	moderately similar to (466)AT5G14430| Symbols: | dehydration-responsive protein-related | chr5:4653095-4655744 FORWARD no origi	
JCVI_13472	2.088	highly similar to (854)AT5G60920| Symbols: COB | COB (COBRA) | chr5:24528692-24531158 REVERSEhighly similar to (688)COBL1	
EX119850	2.088	moderately similar to (466)AT3G52210| Symbols: | mRNA capping enzyme family protein | chr3:19378013-19380271 FORWARDmoder	
JCVI_24933	2.087	weakly similar to (172)AT2G44940| Symbols: | AP2 domain-containing transcription factor TINY, putative | chr2:18544369-18545256 F	
EV140441	2.087	moderately similar to (226)AT4G33870| Symbols: | peroxidase, putative | chr4:16234675-16236497 REVERSEweakly similar to (137)PE	
JCVI_4961	2.087	moderately similar to (201)AT1G55265| Symbols: | similar to unknown protein [Arabidopsis thaliana] (TAIR:AT5G19860.1); similar to h	
EE442622	2.087	no similarity	-3.377
EV157813	2.087	weakly similar to (101)AT1G04510| Symbols: | transducin family protein / WD-40 repeat family protein | chr1:1226748-1230591 FORWA	
EE458494	2.087	moderately similar to (224)AT3G60800| Symbols: | zinc finger (DHHC type) family protein | chr3:22478461-22480248 REVERSE [2017	
JCVI_20021	2.087	weakly similar to (130)AT1G48330| Symbols: | similar to unknown protein [Arabidopsis thaliana] (TAIR:AT3G17580.1); similar to unkn	
JCVI_6570	2.087	moderately similar to (454)AT4G15900| Symbols: PRL1 | PRL1 (PLEIOTROPIC REGULATORY LOCUS 1); nucleotide binding | chr4:9	
JCVI_33027	2.086	weakly similar to (199)AT1G79060| Symbols: | similar to unknown protein [Arabidopsis thaliana] (TAIR:AT1G56020.1); similar to hypot	
JCVI_13294	2.086	weakly similar to (159)AT4G01550| Symbols: ANAC069 | ANAC069 (Arabidopsis NAC domain containing protein 69) | chr4:674025-675	
EX121358	2.086	weakly similar to (132)AT1G70250| Symbols: | receptor serine/threonine kinase, putative | chr1:26456638-26459751 FORWARD [21829	
JCVI_5491	2.086	moderately similar to (256)AT3G01910| Symbols: AT-SO, ATSO, SOX | SOX (SULFITE OXIDASE) | chr3:314926-317060 REVERSE n	
JCVI_16645	2.086	moderately similar to (448)AT2G18960| Symbols: PMA, OST2, AHA1 | AHA1 (ARABIDOPSIS H+ ATPASE 1); ATPase | chr2:8228940	
JCVI_38895	2.086	moderately similar to (302)AT3G12120| Symbols: FAD2 | FAD2 (FATTY ACID DESATURASE 2) | chr3:3860598-3861749 REVERSEm	1.665
AM060984	2.086	no similarity	
JCVI_11883	2.086	highly similar to (620)AT5G53850| Symbols: | haloacid dehalogenase-like hydrolase family protein | chr5:21878843-21882043 REVERSE	-3.113
ES911424	2.086	moderately similar to (322)AT1G12110| Symbols: CHL1-1, NRT1, B-1, ATNRT1, CHL1, NRT1.1 | NRT1.1 (NITRATE TRANSPORTEI	
JCVI_36119	2.085	weakly similar to (115)AT3G13062| Symbols: | similar to unknown protein [Arabidopsis thaliana] (TAIR:AT1G55960.1); similar to unna	
BQ791425	2.085	moderately similar to (267)AT5G13750| Symbols: ZIFL1 | ZIFL1 (ZINC INDUCED FACILITATOR-LIKE 1); tetracycline:hydrogen antip	
JCVI_14754	2.085	moderately similar to (249)AT1G61730| Symbols: | DNA-binding storekeeper protein-related | chr1:22797112-22798242 REVERSE no o	
JCVI_2638	2.085	moderately similar to (452)AT3G49680| Symbols: ATBCAT-3, BCAT3 | ATBCAT-3/BCAT3 (BRANCHED-CHAIN AMINOTRANSFEI	
EX063486	2.084	moderately similar to (223)AT4G27820| Symbols: | glycosyl hydrolase family 1 protein | chr4:13857879-13860577 REVERSEweakly sim	
JCVI_17810	2.084	moderately similar to (216)AT2G35290| Symbols: | similar to unnamed protein product [Vitis vinifera] (GB:CAO63442.1) | chr2:1486871	
JCVI_18896	2.084	moderately similar to (242)AT3G12860| Symbols: | nucleolar protein Nop56, putative | chr3:4091685-4093928 FORWARD no original de	

CD828156	2.084	moderately similar to (275)AT3G01510| Symbols: | 5'-AMP-activated protein kinase beta-1 subunit-related | chr3:198862-201689 REVER	
EX126468	2.084	no similarity	
JCVI_27382	2.083	moderately similar to (309)AT4G00260| Symbols: MEE45 | MEE45 (maternal effect embryo arrest 45); DNA binding / transcription facto	
JCVI_3461	2.083	moderately similar to (433)AT2G33040| Symbols: | ATP synthase gamma chain, mitochondrial (ATPC) | chr2:14026055-14028124 REVI	
EV203693	2.083	no similarity	
EH413958	2.083	weakly similar to (114)AT3G21760| Symbols: | UDP-glucoronosyl/UDP-glucosyl transferase family protein | chr3:7667106-7668563 FOF	
JCVI_15973	2.082	highly similar to (672)AT4G10960| Symbols: UGE5 | UGE5 (UDP-D-GLUCOSE/UDP-D-GALACTOSE 4-EPIMERASE 5); UDP-glucos	
JCVI_42230	2.082	highly similar to (639)AT5G09760| Symbols: | pectinesterase family protein | chr5:3032447-3034365 FORWARDweakly similar to (198)	
EV103883	2.082	moderately similar to (299)AT3G18850| Symbols: LPAT5 | LPAT5 | chr3:6499535-6500846 REVERSE [21477]	
CX189671	2.082	moderately similar to (216)AT5G66810| Symbols: | similar to unknown protein [Arabidopsis thaliana] (TAIR:AT1G61150.6); similar to u	
JCVI_10860	2.082	weakly similar to (111)AT2G37760| Symbols: | aldo/keto reductase family protein | chr2:15839073-15840756 FORWARD no original des	
JCVI_5840	2.082	highly similar to (832)AT5G08570| Symbols: | pyruvate kinase, putative | chr5:2778434-2780301 FORWARDhighly similar to (763)KPY	
CD828630	2.082	moderately similar to (291)AT3G20630| Symbols: TTN6, ATUBP14, UBP14 | UBP14 (UBIQUITIN-SPECIFIC PROTEASE 14); ubiquiti	
ES934887	2.082	no similarity	
JCVI_23756	2.081	no original description	
JCVI_21918	2.081	moderately similar to (320)AT4G27890| Symbols: | nuclear movement family protein | chr4:13886039-13887226 FORWARD no original	-1.540
JCVI_9734	2.081	moderately similar to (231)AT5G15330| Symbols: | SPX (SYG1/Pho81/XPR1) domain-containing protein | chr5:4980598-4982046 FORV	-1.518
JCVI_16126	2.081	moderately similar to (273)AT3G26670| Symbols: | similar to permease-related [Arabidopsis thaliana] (TAIR:AT3G23870.1); similar to u	
EX050343	2.081	weakly similar to (118)AT4G24190| Symbols: SHD | SHD (SHEPHERD); ATP binding | chr4:12551912-12555861 REVERSEweakly simi	
RC_JCVI_42182	2.081	no original description	
JCVI_4969	2.080	moderately similar to (247)AT1G67250| Symbols: | proteasome maturation factor UMP1 family protein | chr1:25167471-25168630 REVE	
JCVI_14163	2.080	very weakly similar to (82.8)AT1G04000| Symbols: | similar to unknown protein [Arabidopsis thaliana] (TAIR:AT5G44060.1); similar to	
JCVI_10269	2.080	moderately similar to (298)AT2G25840| Symbols: OVA4 | OVA4 (OVULE ABORTION 4); ATP binding / aminoacyl-tRNA ligase | chr2:	
EE502951	2.080	weakly similar to (152)AT4G14040| Symbols: EDA38, SBP2 | EDA38 (embryo sac development arrest 38); selenium binding | chr4:81006	
JCVI_6856	2.080	moderately similar to (403)AT5G49970| Symbols: ATPPOX | ATPPOX (A. THALIANA PYRIDOXIN (PYRODOXAMINE) 5'-PHOSPH	
EH419260	2.080	very weakly similar to (87.0)AT3G13200| Symbols: EMB2769 | EMB2769 (EMBRYO DEFECTIVE 2769) | chr3:4242246-4243983 FORV	
ES907751	2.080	moderately similar to (397)AT5G63610| Symbols: CDKE;1, HEN3 | HEN3 (HUA ENHANCER 3); kinase | chr5:25480871-25482283 RE	
JCVI_34631	2.080	no original description	
EV129812	2.080	no similarity	-1.558
EV093273	2.079	no similarity	
AM391106	2.079	moderately similar to (267)AT2G32800| Symbols: AP4.3A | AP4.3A; ATP binding / protein kinase | chr2:13923555-13926110 FORWARI	
JCVI_17247	2.079	highly similar to (502)AT4G24290| Symbols: | similar to NSL1 (NECROTIC SPOTTED LESIONS 1) [Arabidopsis thaliana] (TAIR:AT1	
JCVI_38915	2.078	moderately similar to (311)AT4G28680| Symbols: | tyrosine decarboxylase, putative | chr4:14155254-14158552 FORWARDmoderately s	
DY027795	2.078	moderately similar to (334)AT3G07330| Symbols: CSLC06, ATCSLC6, ATCSLC06 | ATCSLC06 (Cellulose synthase-like C6); transferas	
JCVI_5655	2.078	moderately similar to (265)AT5G66055| Symbols: EMB2036, AKRP | AKRP/EMB2036 (EMBRYO DEFECTIVE 2036); protein binding	-2.315
JCVI_76	2.078	weakly similar to (104)AT4G32260| Symbols: | ATP synthase family | chr4:15573865-15574592 REVERSEvery weakly similar to (95.1)	
JCVI_41874	2.078	highly similar to (516)AT3G26100| Symbols: | regulator of chromosome condensation (RCC1) family protein | chr3:9539695-9541386 FC	
JCVI_11246	2.078	moderately similar to (437)AT3G02580| Symbols: DWF7, BUL1 | STE1 (STEROL 1); C-5 sterol desaturase | chr3:547055-548622 FORW	
JCVI_28371	2.078	highly similar to (513)AT3G21230| Symbols: 4CL5 | 4CL5 (4-COUMARATE:COA LIGASE 5); 4-coumarate-CoA ligase | chr3:7448237-	
AM395293	2.078	no similarity	
JCVI_8685	2.077	moderately similar to (285)AT2G24200| Symbols: | cytosol aminopeptidase | chr2:10294097-10296530 REVERSEmoderately similar to (	
JCVI_42459	2.077	moderately similar to (271)AT2G32240| Symbols: | similar to unknown protein [Arabidopsis thaliana] (TAIR:AT1G05320.1); similar to u	
JCVI_7552	2.077	moderately similar to (205)AT4G00750| Symbols: | dehydration-responsive family protein | chr4:314405-317507 FORWARD no original	
JCVI_784	2.077	highly similar to (506)AT1G13470| Symbols: | similar to unknown protein [Arabidopsis thaliana] (TAIR:AT1G13520.1); similar to unnam	
BG544660	2.077	no similarity	
EX131922	2.076	highly similar to (537)AT3G21110| Symbols: ATPURC, PUR7, PURC | PUR7 (PURIN 7); phosphoribosylaminoimidazolesuccinocarboxa	
JCVI_14422	2.076	moderately similar to (347)AT1G21400| Symbols: | 2-oxoisovalerate dehydrogenase, putative / 3-methyl-2-oxobutanoate dehydrogenase,	
JCVI_4405	2.076	moderately similar to (379)AT1G15060| Symbols: | similar to unknown protein [Arabidopsis thaliana] (TAIR:AT1G73750.1); similar to C	-1.348
EG020533	2.076	no similarity	
JCVI_35841	2.076	weakly similar to (181)AT3G49250| Symbols: | similar to ATP binding [Arabidopsis thaliana] (TAIR:AT5G24280.1); similar to unnamed	
EV128932	2.076	moderately similar to (312)AT3G48720| Symbols: | transferase family protein | chr3:18057512-18060280 FORWARDweakly similar to (	
JCVI_33785	2.075	moderately similar to (338)AT5G55530| Symbols: | C2 domain-containing protein | chr5:22511665-22512882 FORWARD no original des	
JCVI_20026	2.075	moderately similar to (276)AT1G69170| Symbols: | squamosa promoter-binding protein-like 6 (SPL6) | chr1:26009289-26010704 FORW	-1.214
JCVI_34668	2.075	moderately similar to (218)AT3G05545| Symbols: | transcription factor, putative / zinc finger (C3HC4 type RING finger) family protein |	
JCVI_38485	2.075	moderately similar to (278)AT5G24680| Symbols: | similar to unnamed protein product [Vitis vinifera] (GB:CAO48536.1); contains Inter	
JCVI_31050	2.074	highly similar to (674)AT4G02940| Symbols: | oxidoreductase, 2OG-Fe(II) oxygenase family protein | chr4:1306658-1310699 FORWARI	
JCVI_39119	2.074	moderately similar to (301)AT4G24330| Symbols: | similar to unknown protein [Arabidopsis thaliana] (TAIR:AT5G49945.1); similar to h	
ES910517	2.074	moderately similar to (242)AT4G27990| Symbols: | YGGT family protein | chr4:13923997-13924653 REVERSE [21430] 1 602 627	
L47922	2.074	moderately similar to (208)AT2G19400| Symbols: | protein kinase, putative | chr2:8406605-8409563 REVERSE [132]	
JCVI_8580	2.074	no original description	
EV136620	2.074	no similarity	
JCVI_8859	2.074	moderately similar to (235)AT4G25370| Symbols: | Clp amino terminal domain-containing protein | chr4:12972757-12974590 FORWARI	
JCVI_19497	2.074	moderately similar to (266)AT2G21600| Symbols: ATRER1B | ATRER1B (Arabidopsis thaliana endoplasmatic reticulum retrieval protein	3.004
JCVI_22598	2.074	moderately similar to (280)AT1G30860| Symbols: | protein binding / zinc ion binding | chr1:10986677-10989227 REVERSE no original d	
JCVI_6892	2.073	weakly similar to (138)AT2G40170| Symbols: ATEM6, GEA6 | ATEM6/GEA6 (ARABIDOPSIS EARLY METHIONINE-LABELLED 6)	
AM388979	2.073	no similarity	
EV157418	2.073	moderately similar to (208)AT4G26850| Symbols: VTC2 | VTC2 (VITAMIN C DEFECTIVE 2) | chr4:13499268-13501151 REVERSE [2	
ES912597	2.073	no similarity	
JCVI_36003	2.073	moderately similar to (374)AT2G19430| Symbols: | transducin family protein / WD-40 repeat family protein | chr2:8422299-8424822 FOF	
JCVI_18563	2.073	moderately similar to (336)AT5G54500| Symbols: FQR1 | FQR1 (FLAVODOXIN-LIKE QUINONE REDUCTASE 1) | chr5:22141900-22	
JCVI_4269	2.072	moderately similar to (367)AT3G58140| Symbols: | phenylalanyl-tRNA synthetase class IIc family protein | chr3:21540965-21543363 RE	
JCVI_15302	2.072	moderately similar to (458)AT1G25530| Symbols: | lysine and histidine specific transporter, putative | chr1:8964814-8967378 REVERSE	1.674
CD832266	2.072	moderately similar to (360)AT3G03110| Symbols: CRM1B, XPO1B | XPO1B (exportin 1B); protein transporter | chr3:708973-716886 FO	
JCVI_11030	2.072	moderately similar to (291)AT1G55920| Symbols: SAT5, SAT1, AtSerat2;1 | AtSerat2;1 (SERINE ACETYLTRANSFERASE 1) | chr1:20	
JCVI_24485	2.072	moderately similar to (247)AT3G10730| Symbols: | sad1/unc-84-like 2 family protein | chr3:3358561-3360527 REVERSE no original des	
AM060125	2.072	no similarity	
EX092992	2.072	moderately similar to (270)AT4G16480| Symbols: ATINT4 | ATINT4 (INOSITOL TRANSPORTER 4); carbohydrate transmembrane tra	
EE536652	2.071	weakly similar to (132)AT1G17440| Symbols: EER4, TAF12B | EER4/TAF12B (TBP-ASSOCIATED FACTOR 12B); transcription initiat	
EX116542	2.071	weakly similar to (122)AT2G30370| Symbols: | allergen-related | chr2:12947654-12949244 REVERSE [21822] 1 683 699	
DN237936	2.071	weakly similar to (132)AT1G14530| Symbols: | (TOM THREE HOMOLOG); virion binding | chr1:4971415-4973592 REVERSE [17056]	
EV198663	2.071	moderately similar to (365)AT1G73110| Symbols: | ribulose bisphosphate carboxylase/oxygenase activase, putative / RuBisCO activase, p	

JCVI_2151	2.071	moderately similar to (345)AT5G11450| Symbols: | oxygen-evolving complex-related | chr5:3654476-3656358 FORWARD no original de	
JCVI_26911	2.071	moderately similar to (429)AT3G45620| Symbols: | transducin family protein / WD-40 repeat family protein | chr3:16756903-16758978 F	
JCVI_7970	2.071	moderately similar to (261)AT3G59090| Symbols: | similar to TOM1 (TOBAMOVIRUS MULTIPLICATION 1) [Arabidopsis thaliana] (T	
EX085964	2.070	no similarity	
EE546783	2.070	weakly similar to (128)AT5G35530| Symbols: | 40S ribosomal protein S3 (RPS3C) | chr5:13727585-13729422 REVERSE [20128] 1 253	
JCVI_5525	2.070	moderately similar to (389)AT2G15290| Symbols: ATTIC21, TIC21, CIA5, PIC1 | ATTIC21/CIA5/PIC1/TIC21 (CHLOROPLAST IMPC	
EV074099	2.070	moderately similar to (383)AT5G63680| Symbols: | pyruvate kinase, putative | chr5:25507733-25509756 FORWARDmoderately similar to	1.472
JCVI_28822	2.070	moderately similar to (308)AT1G11910| Symbols: | aspartyl protease family protein | chr1:4017117-4019872 REVERSEmoderately simila	
JCVI_15523	2.070	no original description	
JCVI_27944	2.069	moderately similar to (385)AT5G43930| Symbols: | transducin family protein / WD-40 repeat family protein | chr5:17694274-17697804 F	
DY029458	2.069	moderately similar to (337)AT3G53760| Symbols: | tubulin family protein | chr3:19929160-19933242 REVERSEmoderately similar to (2	
EX038707	2.069	moderately similar to (284)AT2G22830| Symbols: SQE2 | SQE2 (SQUALENE EPOXIDASE 2); oxidoreductase | chr2:9731027-9733350	
JCVI_17839	2.069	moderately similar to (459)AT3G59140| Symbols: ATMRP14 | ATMRP14 (Arabidopsis thaliana multidrug resistance-associated protein 1	
JCVI_17841	2.069	weakly similar to (182)AT3G11840| Symbols: | U-box domain-containing protein | chr3:3736584-3738256 REVERSE no original descript	
EX096121	2.069	moderately similar to (221)AT1G71030| Symbols: ATMYBL2 | ATMYBL2 (Arabidopsis myb-like 2); DNA binding / transcription factor	-2.860
EX131806	2.069	no similarity	
JCVI_37379	2.069	moderately similar to (211)AT1G52280| Symbols: AtRABG3d | AtRABG3d (Arabidopsis Rab GTPase homolog G3d); GTP binding | chr1	
EE558820	2.069	no similarity	
JCVI_8153	2.069	no original description	
RC_ES968200	2.068	no similarity	
JCVI_14586	2.068	moderately similar to (256)AT5G46750| Symbols: AGD8 | AGD8 (ARF-GAP DOMAIN 8); DNA binding | chr5:18987177-18989044 REV	
JCVI_30619	2.067	no original description	
EE439649	2.067	weakly similar to (109)AT4G21990| Symbols: PRH-26, PRH26, ATAPR3, APR3 | APR3 (APS REDUCTASE 3) | chr4:11657296-116589	
JCVI_33555	2.067	moderately similar to (465)AT1G61810| Symbols: BGLU45 | BGLU45; hydrolase, hydrolyzing O-glycosyl compounds | chr1:22833700-22	
CX280236	2.067	weakly similar to (151)AT2G38570| Symbols: | similar to hypothetical protein [Vitis vinifera] (GB:CAN71020.1); similar to unnamed pro	
EV178521	2.067	moderately similar to (278)AT2G45740| Symbols: PEX11D | PEX11D | chr2:18846939-18848176 FORWARD [21487] 136 928 943	
EL587973	2.067	no similarity	
JCVI_23057	2.067	moderately similar to (434)AT2G29050| Symbols: ATRBL1 | ATRBL1 (ARABIDOPSIS THALIANA RHOMBOID-LIKE 1) | chr2:12485	
EX074826	2.067	moderately similar to (388)AT4G23340| Symbols: | oxidoreductase, 2OG-Fe(II) oxygenase family protein | chr4:12195729-12196803 RE	
EV043488	2.066	weakly similar to (141)AT1G53450| Symbols: | similar to unknown protein [Arabidopsis thaliana] (TAIR:AT3G14830.1); similar to unkn	
JCVI_19348	2.065	moderately similar to (411)AT5G35735| Symbols: | auxin-responsive family protein | chr5:13918143-13920164 REVERSE no original de	
JCVI_7144	2.065	moderately similar to (391)AT2G48020| Symbols: | sugar transporter, putative | chr2:19651511-19654077 FORWARD no original descrip	
CX280968	2.065	weakly similar to (188)AT2G28930| Symbols: APK1B | APK1B (Arabidopsis protein kinase 1B); kinase | chr2:12431628-12433642 FORV	
JCVI_10968	2.065	moderately similar to (384)AT1G61800| Symbols: GPT2 | GPT2 (glucose-6-phosphate/phosphate translocator 2); antiporter/ glucose-6-ph	
JCVI_7084	2.065	moderately similar to (228)AT5G02910| Symbols: | F-box family protein | chr5:677118-678905 FORWARD no original description	1.666
EE475329	2.064	moderately similar to (233)AT1G21390| Symbols: EMB2170 | EMB2170 (EMBRYO DEFECTIVE 2170) | chr1:7489374-7490543 REVE	
ES951677	2.064	no similarity	
JCVI_10830	2.064	moderately similar to (358)AT1G11930| Symbols: | alanine racemase family protein | chr1:4028781-4030296 FORWARD no original desc	
EH429408	2.064	weakly similar to (112)AT5G43450| Symbols: | 2-oxoglutarate-dependent dioxygenase, putative | chr5:17474549-17475942 REVERSE [2	
JCVI_26571	2.064	highly similar to (770)AT2G26170| Symbols: MAX1, CYP711A1 | CYP711A1 (MORE AXILLARY BRANCHES 1); oxygen binding | ch	
JCVI_8492	2.063	moderately similar to (397)AT1G72100| Symbols: | late embryogenesis abundant domain-containing protein / LEA domain-containing pro	
JCVI_7103	2.063	moderately similar to (336)AT1G02330| Symbols: | similar to unnamed protein product [Vitis vinifera] (GB:CAO40200.1); contains Inter	
JCVI_19548	2.063	moderately similar to (211)AT2G03470| Symbols: | myb family transcription factor / ELM2 domain-containing protein | chr2:1045691-10	
AM395136	2.063	no similarity	
EX042814	2.062	moderately similar to (210)AT5G20270| Symbols: HHP1 | HHP1 (HEPTAHELICAL TRANSMEMBRANE PROTEIN1) | chr5:6841027-6	
JCVI_39838	2.062	moderately similar to (447)AT5G49665| Symbols: | zinc finger (C3HC4-type RING finger) family protein | chr5:20184345-20186646 RE	
JCVI_32526	2.062	moderately similar to (362)AT1G22040| Symbols: | kelch repeat-containing F-box family protein | chr1:7768359-7769786 FORWARD no	
AM058900	2.062	very weakly similar to (91.7)AT3G24535| Symbols: | unknown protein | chr3:8952013-8952579 FORWARD [17712]	
DY029532	2.062	moderately similar to (358)AT2G30780| Symbols: | pentatricopeptide (PPR) repeat-containing protein | chr2:13123624-13125136 FORW	
JCVI_40216	2.062	no original description	
JCVI_10326	2.062	highly similar to (573)AT1G04690| Symbols: KV-BETA1, KAB1 | KAB1 (POTASSIUM CHANNEL BETA SUBUNIT); potassium chan	
CN827392	2.062	weakly similar to (199)AT4G03010| Symbols: | leucine-rich repeat family protein | chr4:1329952-1331139 FORWARD [15793]	
JCVI_30514	2.062	moderately similar to (492)AT3G05960| Symbols: | sugar transporter, putative | chr3:1783593-1785340 REVERSEmoderately similar to (	
EV218832	2.061	no similarity	
L38167	2.061	no similarity	
EE433819	2.061	no similarity	
ES968864	2.061	weakly similar to (132)AT4G02120| Symbols: | CTP synthase, putative / UTP--ammonia ligase, putative | chr4:940873-944097 FORWAR	
JCVI_34839	2.061	moderately similar to (239)AT3G08980| Symbols: | signal peptidase I family protein | chr3:2741284-2742380 FORWARD no original des	
JCVI_9953	2.061	moderately similar to (282)AT3G47940| Symbols: | DNAJ heat shock protein, putative | chr3:17699217-17700387 REVERSEvery weakly	
JCVI_37074	2.060	highly similar to (623)AT1G74100| Symbols: | sulfotransferase family protein | chr1:27868150-27869166 REVERSE no original descripti	
JCVI_11651	2.060	moderately similar to (330)AT5G50520| Symbols: | nodulin family protein | chr5:20586906-20588813 REVERSE no original description	
JCVI_22236	2.060	no original description	
JCVI_32437	2.060	highly similar to (917)AT3G23640| Symbols: HGL1 | HGL1 (HETEROGLYCAN GLUCOSIDASE 1) | chr3:8502362-8509365 FORWAR	
AM386985	2.059	weakly similar to (127)AT5G37990| Symbols: | S-adenosylmethionine-dependent methyltransferase | chr5:15151097-15152678 REVERSI	
JCVI_8039	2.059	weakly similar to (182)AT3G14010| Symbols: CID4 | CID4 (CTC-Interacting Domain 4) | chr3:4637171-4640698 FORWARD no original	
JCVI_32859	2.059	moderately similar to (226)AT2G30250| Symbols: ATWRKY25, WRKY25 | WRKY25 (WRKY DNA-binding protein 25); transcription fa	
EV195520	2.059	weakly similar to (134)AT1G19050| Symbols: ARR7 | ARR7 (RESPONSE REGULATOR 7); transcription regulator/ two-component resp	-2.986
JCVI_219	2.059	highly similar to (536)AT5G51570| Symbols: | band 7 family protein | chr5:20966737-20968460 FORWARD no original description	
JCVI_42046	2.058	highly similar to (563)AT5G19970| Symbols: | similar to unnamed protein product [Vitis vinifera] (GB:CAO65601.1) | chr5:6747552-674	
JCVI_10333	2.058	moderately similar to (312)AT2G33020| Symbols: | leucine-rich repeat family protein | chr2:14020951-14023593 REVERSEvery weakly s	
EE534204	2.058	no similarity	
JCVI_26537	2.058	moderately similar to (370)AT1G07260| Symbols: | UDP-glucoronosyl/UDP-glucosyl transferase family protein | chr1:2227747-2229177	
ES968111	2.058	weakly similar to (184)AT3G44530| Symbols: HIRA | HIRA (ARABIDOPSIS HOMOLOG OF HISTONE CHAPERONE HIRA); nucleoti	
JCVI_14833	2.058	moderately similar to (420)AT1G73840| Symbols: ESP1 | ESP1 (ENHANCED SILENCING PHENOTYPE 1) | chr1:27767598-27769989	
JCVI_27096	2.057	moderately similar to (250)AT1G32750| Symbols: TAF1, HAF1, HAC13, GTD1, HAF01 | HAF01 (HISTONE ACETYLTRANSFERASE	
JCVI_1489	2.057	highly similar to (582)AT3G23810| Symbols: SAHH2 | SAHH2 (S-ADENOSYL-L-HOMOCYSTEINE (SAH) HYDROLASE 2); adenosyl	
EV030761	2.057	no similarity	
CX190615	2.057	weakly similar to (156)AT3G19760| Symbols: | eukaryotic translation initiation factor 4A, putative / eIF-4A, putative / DEAD box RNA h	
RC_EV205431	2.057	no similarity	
JCVI_14696	2.057	moderately similar to (232)AT3G26200| Symbols: CYP71B22 | CYP71B22 (cytochrome P450, family 71, subfamily B, polypeptide 22); o	
JCVI_10356	2.057	weakly similar to (155)AT4G17640| Symbols: CKB2 | CKB2 (casein kinase II beta chain 2); protein kinase CK2 regulator | chr4:9825451-	

JCVI_2834	2.056	moderately similar to (305)AT3G18560| Symbols: | similar to unknown protein [Arabidopsis thaliana] (TAIR:AT1G49000.1); similar to u	
JCVI_17880	2.056	moderately similar to (430)AT4G31140| Symbols: | glycosyl hydrolase family 17 protein | chr4:15141587-15143194 FORWARDweakly s	
JCVI_42506	2.056	moderately similar to (391)AT3G14620| Symbols: CYP72A8 | CYP72A8 (cytochrome P450, family 72, subfamily A, polypeptide 8); oxyg	
JCVI_30554	2.056	no original description	
JCVI_4451	2.056	moderately similar to (246)AT3G52950| Symbols: | CBS domain-containing protein / octicosapeptide/Phox/Bemp1 (PB1) domain-contain	
ES909500	2.056	moderately similar to (345)AT3G01300| Symbols: | protein kinase, putative | chr3:90824-93342 REVERSEweakly similar to (106)NORK	
EE483620	2.056	weakly similar to (108)AT3G07800| Symbols: | thymidine kinase, putative | chr3:2489950-2490941 REVERSE [20174] 1 299 313	
JCVI_17630	2.056	moderately similar to (487)AT5G50340| Symbols: | ATP binding / ATP-dependent peptidase/ damaged DNA binding / nucleoside-triphos	
EV098356	2.056	moderately similar to (266)AT1G48370| Symbols: YSL8 | YSL8 (YELLOW STRIPE LIKE 8); oligopeptide transporter | chr1:17878228-1	
JCVI_26988	2.056	moderately similar to (330)AT1G05900| Symbols: | endonuclease-related | chr1:1786895-1789501 FORWARD no original description	
AM059924	2.056	weakly similar to (178)AT1G50120| Symbols: | similar to unnamed protein product [Vitis vinifera] (GB:CAO42021.1); contains InterPro	
RC_AM386729	2.056	no similarity	
JCVI_20598	2.055	moderately similar to (263)AT3G01150| Symbols: PTB | PTB (POLYPYRIMIDINE TRACT-BINDING) | chr3:51739-54351 FORWARD	
CD835778	2.055	moderately similar to (356)AT3G03380| Symbols: DEGP7 | DEGP7 (DEGP PROTEASE 7); serine-type peptidase/ trypsin | chr3:799727-8	
EX063757	2.055	weakly similar to (150)AT4G38020| Symbols: | tRNA/rRNA methyltransferase (SpoU) family protein | chr4:17861562-17863004 FORWA	
EV207659	2.055	no similarity	
JCVI_23775	2.055	highly similar to (641)AT3G24460| Symbols: | TMS membrane family protein / tumour differentially expressed (TDE) family protein | chr	-1.171
JCVI_30930	2.054	moderately similar to (322)AT4G28210| Symbols: EMB1923 | EMB1923 (EMBRYO DEFECTIVE 1923) | chr4:13990640-13991937 FOR	-2.770
JCVI_13852	2.054	nearly identical (1093)AT5G49810| Symbols: MMT | MMT (methionine S-methyltransferase); S-adenosylmethionine-dependent methyltra	
JCVI_6249	2.054	moderately similar to (230)AT3G13310| Symbols: | DNAJ heat shock N-terminal domain-containing protein | chr3:4310834-4311307 REV	
JCVI_38927	2.053	moderately similar to (387)AT5G04990| Symbols: | sad1/unc-84 protein-related | chr5:1471699-1473771 REVERSE no original descriptio	
JCVI_38363	2.053	highly similar to (686)AT5G64870| Symbols: | similar to unknown protein [Arabidopsis thaliana] (TAIR:AT5G25250.1); similar to unkno	
JCVI_31205	2.053	moderately similar to (334)AT2G33210| Symbols: | chaperonin, putative | chr2:14082170-14085645 REVERSEmoderately similar to (33	
JCVI_37729	2.053	highly similar to (583)AT1G18590| Symbols: | sulfotransferase family protein | chr1:6398626-6399666 FORWARD no original descriptio	
JCVI_12946	2.053	very weakly similar to (87.4)AT1G11680| Symbols: EMB1738, CYP51A2, CYP51, CYP51G1 | CYP51G1 (CYTOCHROME P450 51); ox	
JCVI_3144	2.053	moderately similar to (217)AT2G36880| Symbols: MAT3 | MAT3 (METHIONINE ADENOSYLTRANSFERASE 3) | chr2:15486800-154	
EE407950	2.052	moderately similar to (390)AT4G31120| Symbols: SKB1, ATPRMT5, PRMT5 | ATPRMT5/PRMT5/SKB1 (SHK1 BINDING PROTEIN	
JCVI_3393	2.052	moderately similar to (276)AT4G31180| Symbols: | aspartyl-tRNA synthetase, putative / aspartate--tRNA ligase, putative | chr4:15156702	
JCVI_32625	2.052	no original description	
JCVI_27444	2.052	no original description	
EV225738	2.052	moderately similar to (335)AT5G39110| Symbols: | germin-like protein, putative | chr5:15675030-15675812 REVERSEweakly similar to (	
EV080335	2.052	moderately similar to (240)AT3G21140| Symbols: | FMN binding | chr3:7409702-7412092 REVERSE [21443]	-1.833
JCVI_11666	2.051	moderately similar to (362)AT1G13560| Symbols: AAPT1 | AAPT1 (AMINOALCOHOLPHOSPHOTRANSFERASE 1); phosphatidyltran	
EX133171	2.051	weakly similar to (123)AT4G29960| Symbols: | similar to unnamed protein product [Vitis vinifera] (GB:CAO15556.1) | chr4:14660759-14	
EV194440	2.051	very weakly similar to (92.4)AT2G21170| Symbols: TIM | TIM (TRIOSEPHOSPHATE ISOMERASE) | chr2:9078128-9080187 REVERS	
EV125555	2.051	moderately similar to (266)AT5G25050| Symbols: | integral membrane transporter family protein | chr5:8632025-8633831 FORWARD [2	
JCVI_15258	2.051	no original description	
ES935564	2.051	no similarity	
JCVI_2537	2.051	weakly similar to (167)AT3G05450| Symbols: | similar to unknown protein [Arabidopsis thaliana] (TAIR:AT3G05685.1); contains InterPr	
JCVI_33060	2.050	moderately similar to (386)AT1G05620| Symbols: | inosine-uridine preferring nucleoside hydrolase family protein | chr1:1679608-168152	
JCVI_36446	2.050	highly similar to (565)AT3G57940| Symbols: | Identical to UPF0202 protein At3g57940 [Arabidopsis Thaliana] (GB:Q9M2Q4;GB:Q0WN	
JCVI_12696	2.050	highly similar to (640)AT4G35090| Symbols: CAT2 | CAT2 (CATALASE 2); catalase | chr4:16701110-16703220 REVERSEhighly simila	
EX070115	2.050	very weakly similar to (83.6)AT5G11580| Symbols: | UVB-resistance protein-related / regulator of chromosome condensation (RCC1) fam	
EV051919	2.050	moderately similar to (288)AT1G55535| Symbols: | similar to unknown protein [Arabidopsis thaliana] (TAIR:AT3G13420.1); similar to u	
JCVI_10776	2.050	highly similar to (781)AT1G26420| Symbols: | FAD-binding domain-containing protein | chr1:9141702-9143291 REVERSE no original de	1.193
EX093834	2.050	moderately similar to (217)AT5G10570| Symbols: | basic helix-loop-helix (bHLH) family protein | chr5:3341358-3342878 FORWARD [2	
JCVI_11612	2.050	highly similar to (752)AT2G35020| Symbols: | UTP--glucose-1-phosphate uridylyltransferase family protein | chr2:14763882-14767556 F	
JCVI_22135	2.049	weakly similar to (195)AT4G17080| Symbols: | MORN (Membrane Occupation and Recognition Nexus) repeat-containing protein /phosp	
JCVI_28610	2.049	highly similar to (522)AT5G20040| Symbols: ATIPT9 | ATIPT9 (Arabidopsis thaliana isopentenyltransferase 9); ATP binding / tRNA isop	
EV013260	2.049	no similarity	
JCVI_41627	2.049	moderately similar to (336)AT5G14760| Symbols: AO | AO (L-ASPARTATE OXIDASE); L-aspartate oxidase | chr5:4769136-4772015 FO	
EX052317	2.049	very weakly similar to (93.2)AT1G80670| Symbols: | transducin family protein / WD-40 repeat family protein | chr1:30325701-30328435 I	-1.650
JCVI_13874	2.049	weakly similar to (198)AT3G10640| Symbols: VPS60.1 | VPS60.1 | chr3:3323506-3324489 REVERSE no original description	
JCVI_5505	2.049	highly similar to (650)AT4G27940| Symbols: | mitochondrial substrate carrier family protein | chr4:13904751-13907042 FORWARDvery	
JCVI_35622	2.049	weakly similar to (102)AT3G48660| Symbols: | similar to unknown protein [Arabidopsis thaliana] (TAIR:AT5G63500.1); similar to hypot	
EX140689	2.049	moderately similar to (394)AT1G08070| Symbols: | pentatricopeptide (PPR) repeat-containing protein | chr1:2514371-2516596 REVERSI	
ES951100	2.048	no similarity	
JCVI_13710	2.048	moderately similar to (218)AT2G34690| Symbols: ACD11 | ACD11 (ACCELERATED CELL DEATH 11) | chr2:14637504-14638858 FO	
JCVI_14475	2.048	moderately similar to (253)AT1G62600| Symbols: | flavin-containing monooxygenase family protein / FMO family protein | chr1:231832	
JCVI_27341	2.048	moderately similar to (220)AT2G22980| Symbols: SCPL13 | SCPL13 | chr2:9786109-9790168 FORWARDvery weakly similar to (96.7)Cl	
EV220125	2.048	moderately similar to (279)AT2G39435| Symbols: | similar to unknown protein [Arabidopsis thaliana] (TAIR:AT3G53540.1); similar to u	
JCVI_22968	2.048	highly similar to (571)AT5G36160| Symbols: | aminotransferase-related | chr5:14250491-14252359 REVERSE no original description	
JCVI_29817	2.048	moderately similar to (313)AT3G48080| Symbols: | lipase class 3 family protein / disease resistance protein-related | chr3:17764089-1776	
JCVI_14326	2.048	moderately similar to (365)AT4G17300| Symbols: OVA8, ATNS1, NS1 | NS1 (OVULE ABORTION 8) | chr4:9681570-9684845 FORWA	
EX100536	2.048	moderately similar to (432)AT5G15270| Symbols: | KH domain-containing protein | chr5:4958741-4960950 FORWARD [21825] 1 800 8	
JCVI_29714	2.047	weakly similar to (152)AT3G20370| Symbols: | meprin and TRAF homology domain-containing protein / MATH domain-containing prote	
JCVI_30145	2.047	weakly similar to (151)AT5G13970| Symbols: | similar to unknown protein [Arabidopsis thaliana] (TAIR:AT5G13310.1); similar to OSJN	
EX060016	2.047	weakly similar to (145)AT1G03055| Symbols: | similar to unknown protein [Arabidopsis thaliana] (TAIR:AT1G64680.1); similar to hypot	
EV157334	2.047	moderately similar to (314)AT2G35350| Symbols: PLL1 | PLL1 (POLTERGEIST LIKE 1); protein serine/threonine phosphatase | chr2:14	-1.189
JCVI_26283	2.047	no original description	
ES900651	2.047	moderately similar to (436)AT2G06000| Symbols: | pentatricopeptide (PPR) repeat-containing protein | chr2:2327997-2329607 REVERSI	-1.760
EE559427	2.047	no similarity	
JCVI_4939	2.047	moderately similar to (398)AT3G24360| Symbols: | enoyl-CoA hydratase/isomerase family protein | chr3:8839839-8842806 REVERSE no	
JCVI_19660	2.047	weakly similar to (152)AT5G46900| Symbols: | protease inhibitor/seed storage/lipid transfer protein (LTP) family protein | chr5:19057181	
JCVI_28276	2.046	moderately similar to (295)AT5G19900| Symbols: | PRLI-interacting factor, putative | chr5:6728563-6730047 REVERSE no original desc	
JCVI_23753	2.046	weakly similar to (196)AT2G33120| Symbols: VAMP722, ATVAMP722, SAR1 | SAR1 (SYNAPTOBREVIN-RELATED PROTEIN 1) | c	
EV158355	2.046	moderately similar to (221)AT3G56150| Symbols: ATEIF3C-1, EIF3C-1, ATTIF3C1, TIF3C1, EIF3C | EIF3C (EUKARYOTIC TRANSL	
JCVI_21800	2.046	no original description	
JCVI_21645	2.046	moderately similar to (258)AT3G21700| Symbols: | GTP binding | chr3:7644588-7645961 FORWARD no original description	
JCVI_35834	2.046	weakly similar to (158)AT3G63290| Symbols: | similar to unknown protein [Arabidopsis thaliana] (TAIR:AT4G13400.1); similar to unnar	
JCVI_15	2.046	highly similar to (562)AT3G14990| Symbols: | 4-methyl-5(b-hydroxyethyl)-thiazole monophosphate biosynthesis protein, putative | chr3:5	

JCVI_3076	2.046	moderately similar to (290)AT5G09810| Symbols: ACT2/7, ACT7 | ACT7 (actin 7) | chr5:3052810-3054221 FORWARDmoderately simil	
EE423356	2.045	moderately similar to (323)AT4G15960| Symbols: | epoxide hydrolase, putative | chr4:9045777-9047213 REVERSE [20146]	
JCVI_38185	2.045	moderately similar to (471)AT2G23420| Symbols: | nicotinate phosphoribosyltransferase family protein / NAPRTase family protein | chr2	
JCVI_41002	2.045	moderately similar to (446)AT3G53810| Symbols: | lectin protein kinase, putative | chr3:19944131-19946164 REVERSE no original descr	
EE467521	2.045	no similarity	
JCVI_29663	2.045	weakly similar to (117)AT3G03550| Symbols: | zinc finger (C3HC4-type RING finger) family protein | chr3:850398-851468 REVERSE n	
JCVI_8166	2.045	moderately similar to (230)AT5G46850| Symbols: | similar to unknown [Populus trichocarpa] (GB:ABK96208.1); contains InterPro doma	
JCVI_2165	2.044	highly similar to (613)AT3G01480| Symbols: CYP38 | CYP38 (CYCLOPHILIN 38) | chr3:188576-190259 FORWARDhighly similar to (	
EV168011	2.044	weakly similar to (200)AT2G20360| Symbols: | binding / catalytic/ coenzyme binding | chr2:8793151-8796179 FORWARD [21486]	
JCVI_5152	2.044	moderately similar to (248)AT5G14040| Symbols: | mitochondrial phosphate transporter | chr5:4531061-4532967 REVERSE no original c	
JCVI_42447	2.044	moderately similar to (356)AT4G00500| Symbols: | lipase class 3 family protein / calmodulin-binding heat-shock protein-related | chr4:22	
JCVI_9493	2.044	moderately similar to (209)AT5G61030| Symbols: GR-RBP3 | GR-RBP3 (glycine-rich RNA-binding protein 3); RNA binding | chr5:24578	
AI352861	2.044	no similarity	
JCVI_37898	2.043	moderately similar to (273)AT3G47620| Symbols: ATTCP14 | TCP family transcription factor, putative | chr3:17569989-17571458 FORW	
JCVI_9397	2.043	moderately similar to (345)AT5G20040| Symbols: ATIPT9 | ATIPT9 (Arabidopsis thaliana isopentenyltransferase 9); ATP binding / tRNA	
JCVI_28120	2.043	moderately similar to (244)AT5G56230| Symbols: | prenylated rab acceptor (PRA1) family protein | chr5:22775994-22776554 REVERSE	
JCVI_3063	2.043	weakly similar to (187)AT5G32440| Symbols: | similar to unknown [Populus trichocarpa] (GB:ABK93674.1); contains InterPro domain U	2.019
EV010243	2.043	no similarity	-2.272
JCVI_6690	2.042	moderately similar to (367)AT2G33470| Symbols: GLTP1 | GLTP1 (GLYCOLIPID TRANSFER PROTEIN 1); glycolipid binding / glycol	
EV194958	2.042	weakly similar to (185)AT5G10770| Symbols: | chloroplast nucleoid DNA-binding protein, putative | chr5:3403332-3405332 REVERSE [	
JCVI_15143	2.042	highly similar to (513)AT2G16900| Symbols: | similar to unknown protein [Arabidopsis thaliana] (TAIR:AT4G35110.2); similar to unkno	
JCVI_14805	2.042	moderately similar to (389)AT4G34460| Symbols: ELK4, AGB1 | AGB1 (GTP BINDING PROTEIN BETA 1) | chr4:16477397-1647927C	
EV017292	2.042	weakly similar to (129)AT2G44660| Symbols: | transferase, transferring glycosyl groups | chr2:18428171-18429902 REVERSE [21440]	
JCVI_40590	2.042	very weakly similar to (80.9)AT4G15210| Symbols: BMY1, BAM5, AT-BETA-AMY, RAM1, ATBETA-AMY | ATBETA-AMY (BETA-A	
JCVI_2358	2.041	moderately similar to (333)AT2G33070| Symbols: | jacalin lectin family protein | chr2:14036427-14038011 REVERSE no original descrip	
EV049492	2.041	no similarity	-1.210
JCVI_41594	2.041	moderately similar to (239)AT1G31470| Symbols: NFD4 | NFD4 (NUCLEAR FUSION DEFECTIVE 4) | chr1:11262918-11264925 REVE	
ES980351	2.041	moderately similar to (266)AT3G59950| Symbols: | autophagy 4b (APG4b) | chr3:22156536-22157606 REVERSE [21388]	
JCVI_22691	2.041	weakly similar to (175)AT1G76680| Symbols: OPR1 | OPR1 (12-oxophytodienoate reductase 1); 12-oxophytodienoate reductase | chr1:28	
EX125596	2.041	highly similar to (542)AT5G19130| Symbols: | GPI transamidase component family protein / Gaa1-like family protein | chr5:6416130-641	
JCVI_42016	2.040	no original description	-3.329
EE418728	2.040	no similarity	
JCVI_40338	2.040	highly similar to (865)AT5G20990| Symbols: SIR4, CNX, CHL6, CNX1, B73 | B73 (CHLORATE RESISTANT 6); molybdenum ion bind	
JCVI_2392	2.039	moderately similar to (390)AT5G52660| Symbols: | myb family transcription factor | chr5:21376649-21379263 REVERSE no original des	
JCVI_7194	2.039	moderately similar to (333)AT1G49480| Symbols: RTV1 | RTV1 (RELATED TO VERNALIZATION1 1); DNA binding / transcription fa	
JCVI_25940	2.039	moderately similar to (398)AT3G53110| Symbols: LOS4 | LOS4 (Low expression of osmotically responsive genes 1); ATP-dependent heli	
JCVI_38554	2.039	weakly similar to (155)AT3G07810| Symbols: | heterogeneous nuclear ribonucleoprotein, putative / hnRNP, putative | chr3:2492881-2495	
JCVI_37631	2.039	no original description	
EV034152	2.039	moderately similar to (320)AT3G20330| Symbols: | aspartate carabmoyltransferase, chloroplast / aspartate transcarbamylase / ATCase (P	
CV650659	2.039	weakly similar to (139)AT2G23290| Symbols: AtMYB70 | AtMYB70 (myb domain protein 70); DNA binding / transcription factor | chr2:9	
JCVI_30076	2.039	highly similar to (713)AT3G07130| Symbols: ATPAP15, PAP15 | ATPAP15/PAP15 (purple acid phosphatase 15); acid phosphatase/ prote	
JCVI_969	2.039	moderately similar to (216)AT1G12520| Symbols: ATCCS, CCS1 | CCS1 (copper chaperone for superoxide dismutase 1); superoxide dism	
JCVI_34549	2.038	weakly similar to (115)AT2G02030| Symbols: | F-box family protein | chr2:482334-483828 FORWARD no original description	
ES939871	2.038	no similarity	-3.224
JCVI_39073	2.038	highly similar to (535)AT2G39670| Symbols: | radical SAM domain-containing protein | chr2:16541381-16544064 FORWARD no origina	
ES942798	2.038	weakly similar to (138)AT3G12140| Symbols: | emsy N terminus domain-containing protein / ENT domain-containing protein | chr3:3869	
JCVI_8826	2.038	moderately similar to (256)AT4G35335| Symbols: | nucleotide-sugar transporter family protein | chr4:16807291-16809809 FORWARD no	
JCVI_26997	2.038	moderately similar to (452)AT3G19010| Symbols: | oxidoreductase, 2OG-Fe(II) oxygenase family protein | chr3:6556573-6557868 REVE	
JCVI_7840	2.037	moderately similar to (226)AT5G15800| Symbols: AGL2, SEP1 | SEP1 (SEPALLATA1) | chr5:5151597-5153770 REVERSEweakly simila	
EX127632	2.037	moderately similar to (238)AT5G18550| Symbols: | nucleic acid binding | chr5:6160517-6162731 FORWARDweakly similar to (127)ZFN	
EX020714	2.037	very weakly similar to (93.6)AT4G23280| Symbols: | protein kinase, putative | chr4:12174750-12177481 FORWARD [21809]	
EV142796	2.036	weakly similar to (135)AT1G76680| Symbols: OPR1 | OPR1 (12-oxophytodienoate reductase 1); 12-oxophytodienoate reductase | chr1:28	
EV048621	2.036	moderately similar to (262)AT5G17520| Symbols: MEX1, RCP1 | RCP1 (ROOT CAP 1) | chr5:5772798-5775233 REVERSEweakly simil	
JCVI_2293	2.036	weakly similar to (106)AT3G14230| Symbols: RAP2.2 | RAP2.2; DNA binding / transcription factor | chr3:4737623-4739007 REVERSE r	
JCVI_16141	2.036	no original description	
JCVI_10163	2.036	moderately similar to (348)AT5G10940| Symbols: | transducin family protein / WD-40 repeat family protein | chr5:3448891-3454128 REV	-1.245
DY023511	2.036	weakly similar to (157)AT1G69980| Symbols: | similar to unnamed protein product [Vitis vinifera] (GB:CAO40983.1) | chr1:26360222-26	
EE420526	2.036	moderately similar to (328)AT5G13750| Symbols: ZIFL1 | ZIFL1 (ZINC INDUCED FACILITATOR-LIKE 1); tetracycline:hydrogen antip	
JCVI_12830	2.035	no original description	
JCVI_913	2.035	weakly similar to (119)AT1G70760| Symbols: | inorganic carbon transport protein-related | chr1:26690929-26691863 FORWARD no orig	
EV100276	2.035	moderately similar to (337)AT3G01550| Symbols: | triose phosphate/phosphate translocator, putative | chr3:216954-218863 REVERSEwe	2.896
JCVI_5920	2.035	weakly similar to (169)AT3G19130| Symbols: ATRBP47B | ATRBP47B (RNA-BINDING PROTEIN 47B); RNA binding | chr3:6611404-	
JCVI_14946	2.035	moderately similar to (356)AT3G07800| Symbols: | thymidine kinase, putative | chr3:2489950-2490941 REVERSEmoderately similar to (	
AM394517	2.035	weakly similar to (105)AT4G35420| Symbols: | dihydroflavonol 4-reductase family / dihydrokaempferol 4-reductase family | chr4:168340	
JCVI_27632	2.035	weakly similar to (187)AT3G56620| Symbols: | integral membrane family protein / nodulin MtN21-related | chr3:20983675-20985474 RE	
EX134016	2.035	moderately similar to (340)AT5G01900| Symbols: ATWRKY62, WRKY62 | WRKY62 (WRKY DNA-binding protein 62); transcription fa	
EX093884	2.034	very weakly similar to (97.1)AT1G19130| Symbols: | similar to unknown [Populus trichocarpa] (GB:ABK94529.1); contains InterPro dom	
JCVI_37317	2.034	highly similar to (738)AT1G34630| Symbols: | similar to unknown protein [Arabidopsis thaliana] (TAIR:AT5G51150.1); similar to Os03g	
JCVI_20698	2.033	moderately similar to (343)AT3G10840| Symbols: | hydrolase, alpha/beta fold family protein | chr3:3391212-3393286 REVERSE no origi	
JCVI_115	2.033	moderately similar to (381)AT1G58270| Symbols: ZW9 | ZW9 | chr1:21616059-21617754 REVERSE no original description	
JCVI_21285	2.033	moderately similar to (267)AT2G29580| Symbols: | zinc finger (CCCH-type) family protein / RNA recognition motif (RRM)-containing p	
JCVI_2773	2.032	highly similar to (706)AT4G18360| Symbols: | (S)-2-hydroxy-acid oxidase, peroxisomal, putative / glycolate oxidase, putative / short chai	
ES958750	2.032	no similarity	
EX138487	2.032	moderately similar to (334)AT1G18580| Symbols: GAUT11 | GAUT11 (Galacturonosyltransferase 11); polygalacturonate 4-alpha-galactu	
JCVI_7463	2.032	moderately similar to (304)AT1G52930| Symbols: | brix domain-containing protein | chr1:19714929-19716771 FORWARD no original de	
EH414686	2.032	no similarity	
EE430459	2.031	moderately similar to (202)AT2G43330| Symbols: ATINT1 | ATINT1 (INOSITOL TRANSPORTER 1); carbohydrate transmembrane tran	
JCVI_16115	2.031	highly similar to (635)AT3G48150| Symbols: CDC23, APC8 | APC8 (anaphase-promoting complex/cyclosome 8); binding | chr3:1779078	
ES955165	2.031	moderately similar to (209)AT5G35730| Symbols: | EXS family protein / ERD1/XPR1/SYG1 family protein | chr5:13911171-13914051 F	
JCVI_13176	2.030	highly similar to (697)AT2G21790| Symbols: R1, RNR1 | R1/RNR1 (RIBONUCLEOTIDE REDUCTASE 1); ribonucleoside-diphosphate	
JCVI_19681	2.030	moderately similar to (314)AT1G08630| Symbols: THA1 | THA1 (THREONINE ALDOLASE 1) | chr1:2743951-2745688 REVERSE no c	

JCVI_28396	2.030	moderately similar to (328)AT5G22860| Symbols: | serine carboxypeptidase S28 family protein | chr5:7639910-7642948 REVERSE no or	
BQ790702	2.030	weakly similar to (123)AT4G13160| Symbols: | similar to unknown protein [Arabidopsis thaliana] (TAIR:AT4G13630.1); similar to unnai	
JCVI_40406	2.029	moderately similar to (380)AT3G57260| Symbols: PR2, BG2, PR-2, BGL2 | BGL2 (PATHOGENESIS-RELATED PROTEIN 2); glucan 1	
EX020066	2.029	weakly similar to (121)AT4G36040| Symbols: | DNAJ heat shock N-terminal domain-containing protein (J11) | chr4:17049711-17050196	
EX043256	2.029	no similarity	
DY027910	2.029	no similarity	
JCVI_6955	2.029	moderately similar to (290)AT1G50900| Symbols: | similar to hypothetical protein [Vitis vinifera] (GB:CAN65357.1); similar to unnamed	
EV100505	2.029	weakly similar to (138)AT5G62760| Symbols: | nuclear protein ZAP-related | chr5:25223400-25226619 REVERSE [21477] 115 1079 107	
JCVI_19804	2.029	weakly similar to (187)AT3G51520| Symbols: | diacylglycerol acyltransferase family | chr3:19121718-19123497 FORWARD no original c	
CX188573	2.028	moderately similar to (397)AT5G58370| Symbols: | GTP binding | chr5:23610584-23612871 FORWARD [16807]	
JCVI_33592	2.028	very weakly similar to (80.9)AT5G46170| Symbols: | F-box family protein | chr5:18732865-18734052 REVERSE no original description	
JCVI_7172	2.028	highly similar to (617)AT1G70230| Symbols: | similar to unknown protein [Arabidopsis thaliana] (TAIR:AT1G01430.1); similar to Os09g	
EE408016	2.028	moderately similar to (322)AT3G58710| Symbols: ATWRKY69, WRKY69 | WRKY69 (WRKY DNA-binding protein 69); transcription fa	4.313
JCVI_34356	2.028	highly similar to (518)AT3G18290| Symbols: EMB2454 | EMB2454 (EMBRYO DEFECTIVE 2454); protein binding / zinc ion binding | c	
JCVI_18937	2.028	weakly similar to (158)AT3G10525| Symbols: | similar to SIM (SIAMESE) [Arabidopsis thaliana] (TAIR:AT5G04470.1) | chr3:3281581-	
ES911353	2.028	weakly similar to (191)AT2G43260| Symbols: | F-box family protein / S locus-related | chr2:17990821-17992166 REVERSE [21431]	
EV066336	2.027	moderately similar to (308)AT5G08270| Symbols: | similar to unknown protein [Arabidopsis thaliana] (TAIR:AT5G23200.1); similar to u	
EX137474	2.027	very weakly similar to (99.0)AT2G34040| Symbols: | apoptosis inhibitory 5 (API5) family protein | chr2:14385650-14389325 REVERSE [	-1.347
JCVI_1528	2.027	moderately similar to (231)AT3G14790| Symbols: RHM3 | RHM3 (RHAMNOSE BIOSYNTHESIS 3); catalytic | chr3:4964798-4966882	
AM395573	2.027	no similarity	
JCVI_22130	2.026	weakly similar to (174)AT1G09930| Symbols: ATOPT2 | ATOPT2 (oligopeptide transporter 2); oligopeptide transporter | chr1:3227492-3	
EX085528	2.026	moderately similar to (398)AT1G49580| Symbols: | calcium-dependent protein kinase, putative / CDPK, putative | chr1:18355279-183580	
EV051891	2.026	weakly similar to (105)AT1G11320| Symbols: | similar to unnamed protein product [Vitis vinifera] (GB:CAO60944.1) | chr1:3807241-380	
ES904551	2.026	moderately similar to (384)AT5G10770| Symbols: | chloroplast nucleoid DNA-binding protein, putative | chr5:3403332-3405332 REVERS	
EX074875	2.026	moderately similar to (434)AT1G34260| Symbols: | phosphatidylinositol-4-phosphate 5-kinase family protein | chr1:12485945-12491777 I	1.620
JCVI_37339	2.026	moderately similar to (288)AT2G12400| Symbols: | similar to unknown protein [Arabidopsis thaliana] (TAIR:AT2G25270.1); similar to h	
JCVI_28970	2.026	very weakly similar to (87.4)AT2G31560| Symbols: | similar to unknown protein [Arabidopsis thaliana] (TAIR:AT1G05870.1); similar to u	
JCVI_5205	2.026	no original description	
EV003998	2.025	weakly similar to (154)AT4G35730| Symbols: | similar to unknown protein [Arabidopsis thaliana] (TAIR:AT1G34220.2); similar to unnai	
JCVI_13273	2.025	moderately similar to (218)AT2G02860| Symbols: ATSUC3, SUC3, SUT2 | SUT2 (sucrose transporter 3); carbohydrate transmembrane tr	
CX194992	2.025	moderately similar to (254)AT3G50340| Symbols: | similar to unknown protein [Arabidopsis thaliana] (TAIR:AT5G67020.1); similar to u	
JCVI_32645	2.025	moderately similar to (338)AT5G19000| Symbols: ATBPM1 | ATBPM1 (BTB-POZ AND MATH DOMAIN 1); protein binding | chr5:634	
ES947700	2.025	moderately similar to (209)AT3G59990| Symbols: MAP2B | MAP2B (METHIONINE AMINOPEPTIDASE 2B); methionyl aminopeptida	
JCVI_27063	2.025	moderately similar to (450)AT4G29950| Symbols: | microtubule-associated protein | chr4:14658094-14660376 FORWARD no original de	
DN961514	2.025	moderately similar to (216)AT4G33500| Symbols: | protein phosphatase 2C-related / PP2C-related | chr4:16112838-16116246 REVERSE	
EE452942	2.025	weakly similar to (133)AT5G17530| Symbols: | phosphoglucosamine mutase family protein | chr5:5778170-5781865 FORWARD [20194]	
ES990164	2.025	moderately similar to (268)AT4G30560| Symbols: CNGC9, ATCNGC9 | ATCNGC9 (CYCLIC NUCLEOTIDE GATED CHANNEL 9); c	
JCVI_26984	2.024	weakly similar to (105)AT3G54380| Symbols: | SAC3/GANP family protein | chr3:20144963-20147730 REVERSE no original descriptio	-1.318
JCVI_4680	2.023	weakly similar to (130)AT2G30590| Symbols: WRKY21 | WRKY21 (WRKY DNA-binding protein 21); transcription factor | chr2:130409	
EX042218	2.023	moderately similar to (206)AT4G39140| Symbols: | protein binding / zinc ion binding | chr4:18229631-18231276 REVERSE [21811] 14 6	
EV068028	2.023	moderately similar to (211)AT4G27880| Symbols: | seven in absentia (SINA) family protein | chr4:13883629-13884933 FORWARD [214	
JCVI_248	2.023	moderately similar to (387)AT5G09230| Symbols: SRT2 | SRT2 | chr5:2871560-2873614 FORWARD no original description	
JCVI_24035	2.023	moderately similar to (263)AT4G26690| Symbols: MRH5, SHV3 | MRH5/SHV3 (morphogenesis of root hair 5); glycerophosphodiester ph	
JCVI_21922	2.023	moderately similar to (357)AT5G57800| Symbols: FLP1, YRE, CER3, WAX2 | CER3/FLP1/WAX2/YRE (ECERIFERUM 3); catalytic | c	
JCVI_27546	2.023	highly similar to (503)AT3G26410| Symbols: | methyltransferase/ nucleic acid binding | chr3:9670745-9672510 REVERSE no original de	
EX111156	2.022	moderately similar to (355)AT1G67980| Symbols: CCoAMT | CCoAMT (caffeoyl-CoA 3-O-methyltransferase) | chr1:25491902-2549259	
ES932722	2.022	moderately similar to (239)AT5G17670| Symbols: | hydrolase, acting on ester bonds | chr5:5821084-5822602 FORWARD [20143]	
JCVI_16936	2.022	moderately similar to (352)AT2G04550| Symbols: IBR5 | IBR5 (INDOLE-3-BUTYRIC ACID RESPONSE 5); protein tyrosine/serine/thre	
JCVI_9156	2.022	moderately similar to (372)AT1G51760| Symbols: JR3, IAR3 | IAR3 (IAA-ALANINE RESISTANT 3); metallopeptidase | chr1:19203231-	
JCVI_39959	2.021	moderately similar to (209)AT5G11960| Symbols: | similar to hypothetical protein [Vitis vinifera] (GB:CAN81798.1); contains InterPro d	
JCVI_13542	2.021	highly similar to (575)AT3G58560| Symbols: | endonuclease/exonuclease/phosphatase family protein | chr3:21661857-21664873 REVER	
EG021115	2.021	moderately similar to (232)AT1G68230| Symbols: | reticulon family protein (RTNLB14) | chr1:25575848-25576584 FORWARD [20440]	
JCVI_33663	2.021	moderately similar to (221)AT5G63070| Symbols: | 40S ribosomal protein S15, putative | chr5:25316404-25316886 REVERSEweakly sim	
EV051648	2.021	moderately similar to (236)AT5G38640| Symbols: | eukaryotic translation initiation factor 2B family protein / eIF-2B family protein | chr5	
JCVI_34728	2.021	very weakly similar to (91.7)AT4G33390| Symbols: | similar to unknown protein [Arabidopsis thaliana] (TAIR:AT2G26570.1); similar to u	
JCVI_33195	2.020	weakly similar to (119)AT5G56160| Symbols: | transporter | chr5:22749670-22752606 FORWARD no original description	-2.699
JCVI_42331	2.020	moderately similar to (290)AT1G08160| Symbols: | harpin-induced protein-related / HIN1-related / harpin-responsive protein-related | chr	
EX115454	2.020	moderately similar to (234)AT1G74700| Symbols: NUZ | NUZ | chr1:28069006-28070791 FORWARDweakly similar to (175)RNZN_WH	
JCVI_21455	2.020	moderately similar to (462)AT1G51560| Symbols: | FMN binding | chr1:19123568-19125527 REVERSE no original description	
JCVI_5879	2.020	moderately similar to (281)AT2G18410| Symbols: | similar to unknown [Populus trichocarpa] (GB:ABK94526.1) | chr2:7997850-799928	
JCVI_28170	2.020	moderately similar to (433)AT3G19640| Symbols: | magnesium transporter CorA-like family protein (MRS2-3) | chr3:6820975-6823110 F	
ES911968	2.020	weakly similar to (132)AT2G42670| Symbols: | similar to unknown protein [Arabidopsis thaliana] (TAIR:AT3G58670.2); similar to unkn	
JCVI_38001	2.020	moderately similar to (389)AT5G17020| Symbols: ATCRM1, ATXPO1, XPO1, XPO1A | XPO1A (exportin 1A); protein transporter | chr5	
EV050684	2.019	weakly similar to (144)AT4G18800| Symbols: AthSGBP, AtRab11B, AtRABA1d | AtRABA1d/AtRab11B/AthSGBP (Arabidopsis Rab GT	
EX088361	2.019	weakly similar to (153)AT1G70580| Symbols: GGT2, AOAT2 | AOAT2 (GLUTAMATE:GLYOXYLATE AMINOTRANSFERASE 2); al	
JCVI_11407	2.019	moderately similar to (261)AT1G21480| Symbols: | exostosin family protein | chr1:7519361-7521415 REVERSE no original description	
AM395145	2.019	moderately similar to (262)AT3G62360| Symbols: | carbohydrate binding | chr3:23083995-23091430 REVERSE [20346]	1.644
EV034862	2.019	weakly similar to (196)AT5G19000| Symbols: ATBPM1 | ATBPM1 (BTB-POZ AND MATH DOMAIN 1); protein binding | chr5:634256	
JCVI_235	2.019	moderately similar to (403)AT5G17920| Symbols: ATMETS, ATMS1, ATCIMS | ATCIMS (COBALAMIN-INDEPENDENT METHION	
JCVI_26099	2.019	moderately similar to (387)AT1G80640| Symbols: | protein kinase family protein | chr1:30316871-30318820 FORWARDweakly similar to	
JCVI_35463	2.019	moderately similar to (263)AT3G63130| Symbols: RANGAP1 | RANGAP1 (RAN GTPASE ACTIVATING PROTEIN 1); RAN GTPase a	
ES900267	2.019	moderately similar to (349)AT1G17580| Symbols: ATMYA1, MYA1 | MYA1 (ARABIDOPSIS MYOSIN); motor/ protein binding | chr1:6	
JCVI_4109	2.019	moderately similar to (363)AT1G45474| Symbols: LHCA5 | LHCA5 (Photosystem I light harvesting complex gene 5) | chr1:17181793-171	
JCVI_3781	2.018	very weakly similar to (91.7)AT4G29550| Symbols: | similar to unknown protein [Arabidopsis thaliana] (TAIR:AT2G20620.1); contains In	
EV012509	2.018	no similarity	
JCVI_27735	2.018	moderately similar to (295)AT4G00720| Symbols: | shaggy-related protein kinase theta / ASK-theta (ASK8) | chr4:294116-297002 REVE	
EX130787	2.018	weakly similar to (145)AT1G50460| Symbols: | hexokinase, putative | chr1:18697699-18701097 FORWARDweakly similar to (115)HXK	
JCVI_25540	2.018	no original description	
JCVI_39788	2.018	weakly similar to (157)AT5G05100| Symbols: | nucleic acid binding | chr5:1505462-1506941 REVERSE no original description	
JCVI_2999	2.018	moderately similar to (389)AT3G61430| Symbols: ATPIP1, PIP1, PIP1;1, PIP1A | PIP1A (PLASMA MEMBRANE INTRINSIC PROTEI	
JCVI_4875	2.018	highly similar to (576)AT5G04950| Symbols: | nicotianamine synthase, putative | chr5:1457877-1458839 REVERSEmoderately similar to	

EX091734	2.018	no similarity	
EV117232	2.018	weakly similar to (125)AT4G17080| Symbols: | MORN (Membrane Occupation and Recognition Nexus) repeat-containing protein /phosp	
JCVI_10436	2.018	moderately similar to (275)AT2G37770| Symbols: | aldo/keto reductase family protein | chr2:15841966-15843737 FORWARDweakly sim	
DN961238	2.018	weakly similar to (171)AT4G02350| Symbols: | exocyst complex subunit Sec15-like family protein | chr4:1038157-1040571 FORWARD [	
JCVI_30351	2.017	moderately similar to (227)AT1G69260| Symbols: | similar to unknown protein [Arabidopsis thaliana] (TAIR:AT1G13740.1); similar to u	
RC_AM395998	2.017	no similarity	
JCVI_17034	2.017	weakly similar to (110)AT5G15080| Symbols: | protein kinase, putative | chr5:4886417-4888558 FORWARD no original description	
JCVI_37039	2.017	no original description	
JCVI_26298	2.017	moderately similar to (315)AT4G04870| Symbols: CLS | CLS (CARDIOLIPIN SYNTHASE); cardiolipin synthase/ phosphatidyltransferas	
ES965932	2.017	no similarity	
JCVI_14448	2.017	moderately similar to (485)AT4G22720| Symbols: | glycoprotease M22 family protein | chr4:11937478-11938728 FORWARD no original	
JCVI_28454	2.016	moderately similar to (292)AT3G07525| Symbols: ATG10 | ATG10 (AUTOPHAGY 10); transporter | chr3:2399283-2400386 REVERSE	-1.827
JCVI_9729	2.016	no original description	
JCVI_3155	2.016	moderately similar to (341)AT2G43910| Symbols: | thiol methyltransferase, putative | chr2:18191672-18194027 REVERSE no original de	
EE548016	2.016	very weakly similar to (85.5)AT1G33140| Symbols: PGY2 | 60S ribosomal protein L9 (RPL90A/C) | chr1:12023340-12024482 FORWARD	
JCVI_141	2.016	highly similar to (777)AT2G25490| Symbols: FBL6, EBF1 | EBF1 (EIN3-BINDING F BOX PROTEIN 1); ubiquitin-protein ligase | chr2:1	-4.526
JCVI_26006	2.016	no original description	
JCVI_19488	2.016	moderately similar to (360)AT4G37880| Symbols: | protein binding / zinc ion binding | chr4:17810177-17811343 FORWARD no original	
EV194361	2.016	weakly similar to (166)AT3G02150| Symbols: TCP13, TFPD, PTF1 | PTF1 (PLASTID TRANSCRIPTION FACTOR 1); transcription fact	
JCVI_35929	2.016	moderately similar to (479)AT1G05570| Symbols: GSL06, ATGSL6, ATGSL06, GSL6, CALS1 | CALS1 (CALLOSE SYNTHASE 1); tran	
JCVI_2057	2.015	moderately similar to (250)AT5G47050| Symbols: | ATP binding / protein binding / shikimate kinase/ zinc ion binding | chr5:19123839-19	
JCVI_1229	2.015	weakly similar to (159)AT1G20696| Symbols: NFD3, NFD03, HMGB3 | HMGB3 (HIGH MOBILITY GROUP B 3) | chr1:7179815-71811	
JCVI_33782	2.015	highly similar to (543)AT5G66880| Symbols: SNRK2-3, SNRK2.3, SRK2I | SNRK2-3/SNRK2.3/SRK2I (SNF1-RELATED PROTEIN KI	
JCVI_39736	2.015	moderately similar to (292)AT1G71810| Symbols: | ABC1 family protein | chr1:27006264-27011626 REVERSE no original description	
EV153035	2.015	moderately similar to (206)AT3G07080| Symbols: | membrane protein | chr3:2241366-2242940 FORWARD [21484] 47 980 980	
EV219908	2.015	moderately similar to (334)AT1G62262| Symbols: SLAH4 | SLAH4 (SLAC1 HOMOLOGUE 4) | chr1:23003983-23005156 REVERSE [2	
CD812199	2.015	no similarity	
JCVI_18302	2.014	moderately similar to (215)AT4G13670| Symbols: PTAC5 | PTAC5 (PLASTID TRANSCRIPTIONALLY ACTIVE5); heat shock protein	
EV027945	2.014	no similarity	
EE456614	2.014	weakly similar to (192)AT3G01350| Symbols: | proton-dependent oligopeptide transport (POT) family protein | chr3:135031-137467 FOR	
CD825577	2.014	moderately similar to (238)AT1G62430| Symbols: ATCDS1 | ATCDS1 (CDP-diacylglycerol synthase 1); phosphatidate cytidylyltransferas	
EE420866	2.014	weakly similar to (188)AT1G55270| Symbols: | kelch repeat-containing F-box family protein | chr1:20622000-20623592 REVERSE [2014	
JCVI_35480	2.014	moderately similar to (369)AT2G44850| Symbols: | similar to unknown protein [Arabidopsis thaliana] (TAIR:AT2G45380.1); similar to h	
EG020428	2.014	moderately similar to (288)AT4G18630| Symbols: | similar to unknown protein [Arabidopsis thaliana] (TAIR:AT5G45850.1); similar to u	-3.603
CD823906	2.013	no similarity	
JCVI_36579	2.013	weakly similar to (153)AT5G66770| Symbols: | scarecrow transcription factor family protein | chr5:26677949-26679703 FORWARD no c	
JCVI_37818	2.013	no original description	
CD843690	2.013	weakly similar to (164)AT5G52960| Symbols: | similar to unnamed protein product [Vitis vinifera] (GB:CAO69341.1) | chr5:21494476-21	
EV203396	2.013	no similarity	
JCVI_5507	2.013	moderately similar to (288)AT3G10915| Symbols: | reticulon family protein | chr3:3416106-3417502 REVERSE no original description	
JCVI_9793	2.013	no original description	
EE557704	2.013	no similarity	
JCVI_8976	2.012	highly similar to (634)AT1G28350| Symbols: | ATP binding / aminoacyl-tRNA ligase | chr1:9944470-9949564 FORWARD no original de	
EE427618	2.012	moderately similar to (255)AT3G53690| Symbols: | zinc finger (C3HC4-type RING finger) family protein | chr3:19909975-19911022 REV	
EE418412	2.011	weakly similar to (158)AT5G06560| Symbols: | similar to unknown protein [Arabidopsis thaliana] (TAIR:AT3G11850.1); similar to unkno	
JCVI_3668	2.011	no original description	
ES983201	2.011	very weakly similar to (89.4)AT5G62930| Symbols: | GDSL-motif lipase/hydrolase family protein | chr5:25272138-25273583 FORWARD	
EX140352	2.011	weakly similar to (197)AT1G61370| Symbols: | S-locus lectin protein kinase family protein | chr1:22645761-22648812 REVERSEvery we	
JCVI_11387	2.011	weakly similar to (160)AT3G11600| Symbols: | similar to unknown protein [Arabidopsis thaliana] (TAIR:AT5G06270.1); similar to unnar	
JCVI_36134	2.011	moderately similar to (265)AT5G66190| Symbols: ATLFNR1 | ATLFNR1 (LEAF FNR 1); poly(U) binding | chr5:26468429-26469842 RE	
EX059689	2.011	moderately similar to (295)AT4G14580| Symbols: SnRK3.3, CIPK4 | CIPK4 (CBL-INTERACTING PROTEIN KINASE 4); kinase | chr4:	1.491
ES898916	2.010	moderately similar to (393)AT1G18460| Symbols: | lipase family protein | chr1:6352675-6355964 FORWARD [21405]	
JCVI_537	2.010	highly similar to (596)AT3G56940| Symbols: CRD1, CHL27, ACSF, AT103 | AT103 (DICARBOXYLATE DIIRON 1) | chr3:21087573-2	
EE506087	2.010	weakly similar to (195)AT3G01090| Symbols: SNRK1.1, AKIN10 | AKIN10 (ARABIDOPSIS SNF1 KINASE HOMOLOG 10) | chr3:3144	
EV181551	2.010	moderately similar to (456)AT5G23575| Symbols: | transmembrane protein, putative | chr5:7946566-7950044 FORWARD [21487] 38 750	
ES271441	2.010	weakly similar to (156)AT3G45060| Symbols: ATNRT2.6 | ATNRT2.6 (Arabidopsis thaliana high affinity nitrate transporter 2.6); nitrate t	
JCVI_37900	2.010	moderately similar to (486)AT2G43430| Symbols: GLY1, GLX2-1 | GLX2-1 (GLYOXALASE 2-1); hydroxyacylglutathione hydrolase | ch	
EV177748	2.010	moderately similar to (399)AT1G44100| Symbols: AAP5 | AAP5 (amino acid permease 5); amino acid transmembrane transporter | chr1:1	2.097
JCVI_20731	2.010	weakly similar to (111)AT3G15040| Symbols: | similar to unknown protein [Arabidopsis thaliana] (TAIR:AT4G21970.1); similar to hypot	
JCVI_10843	2.009	moderately similar to (322)AT3G14990| Symbols: | 4-methyl-5(b-hydroxyethyl)-thiazole monophosphate biosynthesis protein, putative | c	
DY014668	2.009	no similarity	
EX062560	2.009	weakly similar to (196)AT2G47330| Symbols: | DEAD/DEAH box helicase, putative | chr2:19436153-19438687 REVERSEweakly similar	
EE455223	2.009	no similarity	
JCVI_36596	2.009	moderately similar to (223)AT5G49740| Symbols: ATFRO7, FRO7 | ATFRO7/FRO7 (FERRIC REDUCTION OXIDASE 7); ferric-chelate	
JCVI_35495	2.009	highly similar to (866)AT4G09020| Symbols: ATISA3, ISA3 | ATISA3/ISA3 (ISOAMYLASE 3); alpha-amylase | chr4:5784096-5788836	
EE424332	2.008	very weakly similar to (92.0)AT3G51250| Symbols: | senescence/dehydration-associated protein-related | chr3:19039206-19041421 FORW	
JCVI_38588	2.008	no original description	
JCVI_18017	2.008	weakly similar to (188)AT1G06550| Symbols: | enoyl-CoA hydratase/isomerase family protein | chr1:2003833-2006563 REVERSE no ori	
ES940536	2.007	moderately similar to (262)AT5G64420| Symbols: | DNA polymerase V family | chr5:25773642-25778348 FORWARD [21391]	
JCVI_30074	2.007	moderately similar to (263)AT5G10440| Symbols: CYCD4;2 | CYCD4;2 (CYCLIN D4;2); cyclin-dependent protein kinase | chr5:3280612	
EX053743	2.007	weakly similar to (130)AT1G76350| Symbols: | RWP-RK domain-containing protein | chr1:28645037-28647711 FORWARD [21812]	
ES915998	2.007	weakly similar to (103)AT1G07140| Symbols: SIRANBP | SIRANBP (Ran-binding protein 1a); Ran GTPase binding | chr1:2192359-2193	
JCVI_5228	2.007	highly similar to (721)AT2G27600| Symbols: SKD1, VPS4 | SKD1/VPS4; ATP binding | chr2:11788303-11790807 FORWARDweakly sin	
EX021429	2.007	moderately similar to (298)AT3G04070| Symbols: ANAC047 | ANAC047 (Arabidopsis NAC domain containing protein 47) | chr3:106158	
JCVI_3213	2.007	moderately similar to (478)AT5G54960| Symbols: PDC2 | PDC2 (PYRUVATE DECARBOXYLASE-2); pyruvate decarboxylase | chr5:22	
JCVI_21980	2.007	highly similar to (661)AT5G03280| Symbols: EIN2, PIR2, CKR1, ERA3, ORE3, ORE2 | EIN2 (ETHYLENE INSENSITIVE 2); transporte	
JCVI_31849	2.007	highly similar to (672)AT2G28760| Symbols: UXS6 | UXS6 | chr2:12343546-12345719 REVERSEweakly similar to (106)GME1_ORYSA	
AM059544	2.007	no similarity	
EE429810	2.007	weakly similar to (103)AT5G53560| Symbols: B5 #2, ATB5-A | ATB5-A (Cytochrome b5 A) | chr5:21776854-21777579 FORWARDvery	
JCVI_20783	2.006	moderately similar to (201)AT5G67380| Symbols: ATCKA1, CKA1 | CKA1 (CASEIN KINASE ALPHA 1); kinase | chr5:26898382-2690	
DN965592	2.006	very weakly similar to (87.4)AT4G23180| Symbols: RLK4, CRK10 | CRK10 (CYSTEINE-RICH RLK10); kinase | chr4:12138182-121407	

JCVI_21432	2.006	moderately similar to (457)AT4G14605| Symbols: | mitochondrial transcription termination factor-related / mTERF-related | chr4:8378810	
EV192821	2.006	no similarity	
EX089117	2.006	moderately similar to (417)AT1G65020| Symbols: | similar to unnamed protein product [Vitis vinifera] (GB:CAO62149.1); contains Inter	
JCVI_1086	2.006	highly similar to (680)AT1G51680| Symbols: 4CL.1, AT4CL1, 4CL1 | 4CL1 (4-COUMARATE:COA LIGASE 1) | chr1:19162748-191651	
JCVI_35823	2.005	weakly similar to (111)AT3G56910| Symbols: PSRP5 | PSRP5 (PLASTID-SPECIFIC 50S RIBOSOMAL PROTEIN 5) | chr3:21080537-2	
DY027802	2.005	moderately similar to (208)AT1G20540| Symbols: | transducin family protein / WD-40 repeat family protein | chr1:7112642-7115181 FOR	
JCVI_1749	2.005	moderately similar to (347)AT3G15110| Symbols: | similar to unnamed protein product [Vitis vinifera] (GB:CAO39343.1) | chr3:5084401	
EV070544	2.005	weakly similar to (140)AT5G06160| Symbols: | splicing factor-related | chr5:1862624-1866299 REVERSE [21443]	
AI352871	2.004	very weakly similar to (97.4)AT2G34430| Symbols: LHCB1.4, LHB1B1 | LHB1B1 (Photosystem II light harvesting complex gene 1.4); chl	
JCVI_11667	2.004	moderately similar to (471)AT3G16910| Symbols: AAE7, ACN1 | AAE7/ACN1 (ACYL-ACTIVATING ENZYME 7); AMP binding / acet	-1.458
JCVI_37265	2.004	highly similar to (744)AT5G17490| Symbols: RGL3 | RGL3 (RGA-LIKE 3); transcription factor | chr5:5764318-5765889 REVERSEhighly	
JCVI_24182	2.004	moderately similar to (322)AT3G61130| Symbols: GAUT1, LGT1 | GAUT1/LGT1 (Galacturonosyltransferase 1); polygalacturonate 4-alp	
JCVI_9912	2.004	moderately similar to (382)AT1G70560| Symbols: | alliinase C-terminal domain-containing protein | chr1:26608557-26610982 FORWARI	
JCVI_35959	2.004	highly similar to (512)AT1G78060| Symbols: | glycosyl hydrolase family 3 protein | chr1:29354690-29357762 REVERSEweakly similar to	
JCVI_15305	2.004	moderately similar to (265)AT5G46250| Symbols: | RNA recognition motif (RRM)-containing protein | chr5:18772615-18774950 FORWA	-1.780
ES968910	2.003	no similarity	
EV214560	2.003	moderately similar to (219)AT3G01650| Symbols: RGLG1 | RGLG1 (RING DOMAIN LIGASE1); protein binding / zinc ion binding | chr3	
JCVI_39419	2.003	weakly similar to (135)AT1G22020| Symbols: SHM6 | SHM6 (serine hydroxymethyltransferase 6); glycine hydroxymethyltransferase | chr	
BG543879	2.003	weakly similar to (162)AT3G08650| Symbols: | metal transporter family protein | chr3:2624700-2627314 REVERSE [8791]	
EX064824	2.003	no similarity	
JCVI_13610	2.003	moderately similar to (226)AT5G14420| Symbols: RGLG2 | RGLG2 (RING DOMAIN LIGASE2) | chr5:4648358-4650566 REVERSE no	
JCVI_20866	2.003	moderately similar to (453)AT1G18040| Symbols: CDKD1;3, AT;CDCKD;3, CAK2AT | AT;CDCKD;3/CAK2AT/CDKD1;3 (CYCLIN-D	
EE429188	2.003	weakly similar to (162)AT1G22590| Symbols: AGL87 | AGL87 | chr1:7983500-7983991 FORWARD [20136] 14 580 619	
JCVI_2781	2.003	moderately similar to (367)AT1G30970| Symbols: SUF4 | SUF4 (SUPPRESSOR OF FRIGIDA4) | chr1:11040594-11043414 REVERSE n	
CD838691	2.003	moderately similar to (315)AT5G23930| Symbols: | mitochondrial transcription termination factor-related / mTERF-related | chr5:8074628	
EV080157	2.003	moderately similar to (260)AT4G22270| Symbols: | similar to unknown protein [Arabidopsis thaliana] (TAIR:AT4G03820.1); similar to u	
EX100473	2.003	moderately similar to (304)AT1G62640| Symbols: KAS III | KAS III (3-KETOACYL-ACYL CARRIER PROTEIN SYNTHASE III); 3-ox	
JCVI_1383	2.002	moderately similar to (463)AT5G55070| Symbols: | 2-oxoacid dehydrogenase family protein | chr5:22364863-22367635 FORWARD no o	-1.563
JCVI_8474	2.002	moderately similar to (389)AT1G74320| Symbols: | choline kinase, putative | chr1:27944853-27946564 FORWARD no original descriptio	-1.772
JCVI_17365	2.002	weakly similar to (121)AT1G67070| Symbols: DIN9 | DIN9 (DARK INDUCIBLE 9); mannose-6-phosphate isomerase | chr1:25045987-25	
JCVI_4659	2.002	highly similar to (917)AT5G62630| Symbols: HIPL2 | HIPL2 (HIPL2 PROTEIN PRECURSOR); catalytic | chr5:25160945-25163616 REV	
EV125682	2.002	no similarity	
EV012755	2.002	weakly similar to (106)AT3G27280| Symbols: ATPHB4 | ATPHB4 (PROHIBITIN 4) | chr3:10078141-10079288 FORWARD [21450]	
JCVI_13620	2.001	no original description	
JCVI_30139	2.001	moderately similar to (287)AT4G28590| Symbols: | similar to unknown protein [Arabidopsis thaliana] (TAIR:AT2G31840.1); similar to u	
JCVI_5482	2.001	moderately similar to (368)AT3G30841| Symbols: | 2,3-biphosphoglycerate-independent phosphoglycerate mutase-related / phosphoglyce	
JCVI_20640	2.001	moderately similar to (208)AT5G20190| Symbols: | binding | chr5:6814095-6815173 FORWARD no original description	
JCVI_32206	2.001	moderately similar to (492)AT1G36370| Symbols: SHM7 | SHM7 (serine hydroxymethyltransferase 7); glycine hydroxymethyltransferase	
EE552366	2.001	no similarity	
JCVI_10549	2.001	moderately similar to (461)AT4G22920| Symbols: ATNYE1, NYE1 | ATNYE1/NYE1 (NON-YELLOWING 1) | chr4:12016787-12017980	
CV545014	2.001	moderately similar to (225)AT3G14440| Symbols: ATNCED3, STO1, NCED3 | NCED3 (NINE-CIS-EPOXYCAROTENOID DIOXYGEN	
JCVI_4388	2.001	moderately similar to (206)AT4G34200| Symbols: EDA9 | EDA9 (embryo sac development arrest 9); NAD binding / amino acid binding /	
JCVI_5881	2.001	moderately similar to (287)AT5G05930| Symbols: | guanylyl cyclase-related (GC1) | chr5:1780271-1782533 REVERSE no original descri	
EE477104	2.001	weakly similar to (112)AT1G48770| Symbols: | similar to unknown protein [Arabidopsis thaliana] (TAIR:AT3G18295.1); similar to unnar	
JCVI_9441	2.000	moderately similar to (455)AT3G57260| Symbols: PR2, BG2, PR-2, BGL2 | BGL2 (PATHOGENESIS-RELATED PROTEIN 2); glucan 1	
JCVI_19359	2.000	highly similar to (730)AT4G16130| Symbols: ISA1, ATISA1, ARA1 | ARA1 (ARABINOSE KINASE); ATP binding / galactokinase | chr4	
JCVI_19519	2.000	moderately similar to (208)AT1G66620| Symbols: | seven in absentia (SINA) protein, putative | chr1:24856469-24857707 REVERSE no c	
JCVI_7964	2.000	weakly similar to (157)AT1G70900| Symbols: | similar to unknown protein [Arabidopsis thaliana] (TAIR:AT1G23110.3); similar to unkno	
CX192028	2.000	moderately similar to (228)AT1G79020| Symbols: | transcription factor-related | chr1:29732175-29734952 REVERSE [16807] 1 576 589	
CD826629	1.999	moderately similar to (282)AT5G14790| Symbols: | binding | chr5:4784061-4785511 FORWARD [13979]	
JCVI_1330	1.999	moderately similar to (291)AT4G37990| Symbols: ELI3, ELI3-2 | ELI3-2 (ELICITOR-ACTIVATED GENE 3) | chr4:17855958-17857382	
CV973880	1.999	no similarity	
JCVI_28100	1.999	no original description	
EE502254	1.998	moderately similar to (468)AT3G62120| Symbols: | tRNA synthetase class II (G, H, P and S) family protein | chr3:23012202-23014824 RI	
EX106905	1.998	moderately similar to (238)AT5G11250| Symbols: | disease resistance protein (TIR-NBS-LRR class), putative | chr5:3587979-3591961 RI	
JCVI_34946	1.998	no original description	
EE513258	1.998	no similarity	
CX193060	1.998	weakly similar to (118)AT3G13410| Symbols: | similar to unknown protein [Arabidopsis thaliana] (TAIR:AT1G55546.1); similar to hypot	
JCVI_28480	1.998	weakly similar to (197)AT2G36070| Symbols: ATTIM44-2 | ATTIM44-2 (Arabidopsis thaliana translocase inner membrane subunit 44-2)	
JCVI_12445	1.998	moderately similar to (409)AT5G25140| Symbols: CYP71B13 | CYP71B13 (cytochrome P450, family 71, subfamily B, polypeptide 13); o	
JCVI_38795	1.997	weakly similar to (161)AT1G64860| Symbols: SIG1, SIG2, SIGB, RPOD1, SIGA | SIGA (SIGMA FACTOR A); DNA binding / DNA-dire	
EX055333	1.997	no similarity	
JCVI_4119	1.997	moderately similar to (429)AT4G10790| Symbols: | UBX domain-containing protein | chr4:6640748-6643031 REVERSE no original descr	
JCVI_39108	1.997	moderately similar to (239)AT2G44680| Symbols: CKB4 | CKB4 (CASEIN KINASE II BETA SUBUNIT 4); protein kinase CK2 regulato	
JCVI_29802	1.997	highly similar to (603)AT4G26970| Symbols: | aconitate hydratase, cytoplasmic, putative / citrate hydro-lyase/aconitase, putative | chr4:13	
JCVI_14024	1.997	moderately similar to (380)AT3G11960| Symbols: | cleavage and polyadenylation specificity factor (CPSF) A subunit C-terminal domain-	
EV058984	1.997	weakly similar to (179)AT5G12840| Symbols: EMB2220, HAP2, ATHAP2A | HAP2A (EMBRYO DEFECTIVE 2220); transcription facto	
EG020633	1.996	weakly similar to (133)AT5G59400| Symbols: | similar to hypothetical protein [Vitis vinifera] (GB:CAN64889.1) | chr5:23974982-239767	
JCVI_7900	1.996	moderately similar to (482)AT1G22280| Symbols: | protein phosphatase 2C, putative / PP2C, putative | chr1:7874225-7875485 FORWAR	2.644
JCVI_5792	1.996	moderately similar to (476)AT4G38430| Symbols: ATROPGEF1, ROPGEF1 | ATROPGEF1/ROPGEF1 (KINASE PARTNER PROTEIN-	
JCVI_32010	1.996	nearly identical (1098)AT5G27030| Symbols: TPR3 | TPR3 (TOPLESS-RELATED 3) | chr5:9508916-9515266 REVERSE no original desc	
JCVI_22849	1.996	moderately similar to (331)AT5G56720| Symbols: | malate dehydrogenase, cytosolic, putative | chr5:22962763-22963944 FORWARDmod	
EV145804	1.996	moderately similar to (409)AT3G13530| Symbols: MAP3KE1, MAPKKK7 | MAPKKK7 (MAP3K EPSILON PROTEIN KINASE); kinase	
JCVI_2600	1.996	highly similar to (582)AT3G44680| Symbols: HDA09, HDA9 | HDA9 (histone deacetylase 9); histone deacetylase | chr3:16237756-162407	
JCVI_33390	1.995	weakly similar to (129)AT3G51310| Symbols: | vacuolar protein sorting-associated protein 35 family protein / VPS35 family protein | chr3	
CV544891	1.995	no similarity	
AM389446	1.995	very weakly similar to (99.4)AT2G37410| Symbols: TIM17, ATTIM17-2 | ATTIM17-2 (Arabidopsis thaliana translocase inner membrane s	
JCVI_4070	1.995	moderately similar to (391)AT3G55290| Symbols: | short-chain dehydrogenase/reductase (SDR) family protein | chr3:20513630-2051470	
AM062443	1.995	moderately similar to (383)AT4G05090| Symbols: | inositol monophosphatase family protein | chr4:2609242-2611625 FORWARDweakly	
EE564402	1.994	very weakly similar to (82.4)AT2G41040| Symbols: | methyltransferase-related | chr2:17128577-17130142 FORWARD [20153] 1 386 434	
EX126873	1.994	weakly similar to (116)AT2G26230| Symbols: | uricase / urate oxidase / nodulin 35, putative | chr2:11172034-11174046 FORWARD [218	

JCVI_7343	1.994	moderately similar to (452)AT1G76050| Symbols: | pseudouridine synthase family protein | chr1:28545752-28547720 FORWARD no orig	
JCVI_38286	1.994	moderately similar to (241)AT1G05320| Symbols: | similar to unknown protein [Arabidopsis thaliana] (TAIR:AT2G32240.1); similar to u	
JCVI_13241	1.994	moderately similar to (278)AT3G15140| Symbols: | exonuclease family protein | chr3:5099719-5101724 REVERSE no original descriptio	
JCVI_5933	1.994	moderately similar to (343)AT2G37660| Symbols: | binding / catalytic/ coenzyme binding | chr2:15802559-15804055 REVERSE no origin	
JCVI_27052	1.993	moderately similar to (495)AT2G26980| Symbols: SnRK3.17, CIPK3 | CIPK3 (CBL-INTERACTING PROTEIN KINASE 3); kinase | chr	
EV062993	1.993	moderately similar to (228)AT5G06150| Symbols: CYCB1;2, CYC1BAT | CYC1BAT (CYCLIN B 1;2); cyclin-dependent protein kinase r	
JCVI_26286	1.993	weakly similar to (162)AT4G12480| Symbols: pEARLI 1 | pEARLI 1; lipid binding | chr4:7406368-7406874 REVERSEweakly similar to (	
CD827444	1.993	moderately similar to (488)AT2G36390| Symbols: BE3, SBE2.1 | SBE2.1 (STARCH BRANCHING ENZYME 2.1); 1,4-alpha-glucan bran	
ES941723	1.993	no similarity	
AM391530	1.992	moderately similar to (320)AT1G56290| Symbols: | CwfJ-like family protein | chr1:21079604-21082273 FORWARD [20118]	
RC_AM394729	1.992	no similarity	
JCVI_22440	1.992	moderately similar to (291)AT2G39050| Symbols: | hydroxyproline-rich glycoprotein family protein | chr2:16310505-16312414 FORWAI	
JCVI_30424	1.992	moderately similar to (300)AT4G02880| Symbols: | similar to unknown protein [Arabidopsis thaliana] (TAIR:AT1G03290.1); similar to u	
JCVI_21718	1.992	moderately similar to (272)AT1G65060| Symbols: 4CL3 | 4CL3 (4-coumarate:CoA ligase 3); 4-coumarate-CoA ligase | chr1:24171590-24	
ES939619	1.992	no similarity	
JCVI_8121	1.992	moderately similar to (334)AT1G01230| Symbols: | ORMDL family protein | chr1:97620-99046 FORWARD no original description	
EV041715	1.992	no similarity	
JCVI_16524	1.992	moderately similar to (431)AT4G23740| Symbols: | leucine-rich repeat transmembrane protein kinase, putative | chr4:12367073-1236916	
JCVI_41158	1.992	moderately similar to (438)AT1G27120| Symbols: | galactosyltransferase family protein | chr1:9421376-9423897 FORWARD no original	-2.190
JCVI_4628	1.992	moderately similar to (414)AT3G03640| Symbols: GLUC | GLUC (Beta-glucosidase homolog); hydrolase, hydrolyzing O-glycosyl compou	2.324
EE484806	1.991	no similarity	
EV000633	1.991	weakly similar to (115)AT4G36250| Symbols: ALDH3F1 | ALDH3F1 (ALDEHYDE DEHYDROGENASE 3F1); 3-chloroallyl aldehyde de	
JCVI_10321	1.991	moderately similar to (436)AT2G01220| Symbols: | nucleotidyltransferase | chr2:123300-126212 FORWARD no original description	
EE515929	1.991	weakly similar to (114)AT1G48040| Symbols: | protein serine/threonine phosphatase | chr1:17723733-17725367 REVERSE [15713]	
EE432264	1.991	weakly similar to (135)AT5G48030| Symbols: GFA2 | GFA2 (GAMETOPHYTIC FACTOR 2); heat shock protein binding / unfolded prot	
JCVI_32039	1.991	no original description	-2.025
JCVI_31368	1.991	weakly similar to (144)AT3G19810| Symbols: | similar to unnamed protein product [Vitis vinifera] (GB:CAO64796.1); contains InterPro	-2.544
JCVI_32496	1.990	moderately similar to (364)AT1G80510| Symbols: | amino acid transporter family protein | chr1:30277992-30279461 FORWARD no orig	
JCVI_32978	1.990	moderately similar to (335)AT1G67660| Symbols: | DNA binding / magnesium ion binding / nuclease | chr1:25367815-25368732 FORWA	
DY023750	1.990	no similarity	
JCVI_11675	1.990	moderately similar to (450)AT5G44070| Symbols: ARA8, ATPCS1, PCS1, CAD1 | CAD1 (CADMIUM SENSITIVE 1) | chr5:17752103-1	
JCVI_348	1.990	moderately similar to (451)AT5G05340| Symbols: | peroxidase, putative | chr5:1579143-1580820 REVERSEmoderately similar to (468)F	
JCVI_5934	1.990	moderately similar to (231)AT2G36320| Symbols: | zinc finger (AN1-like) family protein | chr2:15236467-15236952 FORWARDweakly s	
JCVI_38861	1.990	moderately similar to (216)AT1G09760| Symbols: U2A' | U2A' (U2 small nuclear ribonucleoprotein A); protein binding | chr1:3159478-31	
EV108274	1.990	no similarity	
EE476779	1.990	moderately similar to (295)AT5G63830| Symbols: | zinc finger (HIT type) family protein | chr5:25560811-25562028 REVERSE [20157]	
EX031682	1.990	weakly similar to (129)AT1G22700| Symbols: | tetratricopeptide repeat (TPR)-containing protein | chr1:8028312-8029278 REVERSE [21	
EX124480	1.990	moderately similar to (265)AT5G40170| Symbols: | disease resistance family protein | chr5:16082407-16084785 REVERSEvery weakly si	
JCVI_33726	1.989	moderately similar to (367)AT2G31240| Symbols: | tetratricopeptide repeat (TPR)-containing protein | chr2:13324647-13326595 REVER	
JCVI_11443	1.989	moderately similar to (370)AT5G45410| Symbols: | similar to unknown protein [Arabidopsis thaliana] (TAIR:AT4G25030.2); similar to u	
JCVI_18285	1.989	moderately similar to (337)AT5G66070| Symbols: | zinc finger (C3HC4-type RING finger) family protein | chr5:26439149-26440259 FOI	
EE418765	1.989	moderately similar to (342)AT3G06540| Symbols: | GDP dissociation inhibitor family protein / Rab GTPase activator family protein | chr3	
DY024741	1.989	weakly similar to (190)AT1G25520| Symbols: | similar to unknown protein [Arabidopsis thaliana] (TAIR:AT1G68650.1); similar to unnar	
EV147921	1.989	moderately similar to (201)AT3G51120| Symbols: | zinc finger (CCCH-type) family protein | chr3:18997006-19002680 REVERSE [2148	
JCVI_33569	1.989	weakly similar to (172)AT3G61770| Symbols: | similar to unknown protein [Arabidopsis thaliana] (TAIR:AT1G67600.1); similar to unnar	
ES961375	1.988	moderately similar to (230)AT4G27680| Symbols: | MSP1 protein, putative / intramitochondrial sorting protein, putative | chr4:13821269-	
EX111924	1.988	moderately similar to (425)AT1G34380| Symbols: | 5'-3' exonuclease family protein | chr1:12552817-12554277 REVERSE [21827]	
JCVI_1438	1.988	highly similar to (552)AT4G20890| Symbols: TUB9 | TUB9 (tubulin beta-9 chain); structural molecule | chr4:11182229-11183851 FORW	
JCVI_39428	1.988	no original description	
DY027136	1.988	weakly similar to (150)AT3G17750| Symbols: | protein kinase family protein | chr3:6074234-6078434 FORWARD [18978]	1.874
JCVI_33597	1.988	moderately similar to (431)AT1G17020| Symbols: ATSRG1, SRG1 | SRG1 (SENESCENCE-RELATED GENE 1); oxidoreductase, acting	
ES922499	1.988	no similarity	
EX086261	1.988	no similarity	
EH430451	1.987	moderately similar to (349)AT4G31850| Symbols: PGR3 | PGR3 (PROTON GRADIENT REGULATION 3) | chr4:15403026-15406364 F	
JCVI_17280	1.987	weakly similar to (120)AT1G06515| Symbols: | similar to unknown protein [Arabidopsis thaliana] (TAIR:AT2G30942.1); similar to unkn	
EX094188	1.987	moderately similar to (230)AT1G03610| Symbols: | similar to unknown protein [Arabidopsis thaliana] (TAIR:AT4G03420.1); similar to u	2.025
JCVI_13677	1.987	moderately similar to (455)AT1G77180| Symbols: | chromatin protein family | chr1:29004685-29006526 REVERSE no original descriptio	
EE556441	1.987	no similarity	
JCVI_22872	1.987	highly similar to (558)AT3G55740| Symbols: ATPROT2, ProT2 | ProT2 (PROLINE TRANSPORTER 2) | chr3:20707552-20709136 FOR	
JCVI_15892	1.987	moderately similar to (294)AT5G59840| Symbols: | Ras-related GTP-binding family protein | chr5:24124676-24126275 REVERSEmodera	
JCVI_24053	1.987	no original description	
AT002133	1.986	no similarity	
EV143177	1.986	weakly similar to (135)AT5G64410| Symbols: ATOPT4 | ATOPT4 (oligopeptide transporter 4); oligopeptide transporter | chr5:25768147-	
DY018510	1.986	weakly similar to (159)AT5G48600| Symbols: ATCAP-C, SMC4, ATSMC3 | ATSMC3 (ARABIDOPSIS THALIANA STRUCTURAL M	
CV433797	1.986	weakly similar to (166)AT1G21450| Symbols: SCL1 | SCL1 (SCARECROW-LIKE 1); transcription factor | chr1:7509710-7511491 FORW	
JCVI_41771	1.986	highly similar to (592)AT3G54030| Symbols: | protein kinase family protein | chr3:20022140-20024468 FORWARDweakly similar to (13	
EE520916	1.986	no similarity	
EV054344	1.986	weakly similar to (182)AT1G22882| Symbols: | similar to unknown protein [Arabidopsis thaliana] (TAIR:AT1G71360.1); similar to unnar	
JCVI_20630	1.986	no original description	
JCVI_12121	1.985	moderately similar to (204)AT5G05987| Symbols: | prenylated rab acceptor (PRA1) family protein | chr5:1804882-1806560 FORWARD r	
EX061777	1.985	highly similar to (518)AT3G22420| Symbols: ZIK3, WNK2 | WNK2 (WITH NO K 2); kinase | chr3:7946659-7948965 FORWARDvery w	
JCVI_19762	1.985	highly similar to (714)AT2G43820| Symbols: GT, UGT74F2 | GT/UGT74F2 (UDP-GLUCOSYLTRANSFERASE 74F2); UDP-glucosyltra	
EX115614	1.985	moderately similar to (201)AT1G49350| Symbols: | pfkB-type carbohydrate kinase family protein | chr1:18268738-18270702 FORWARD	
EX056479	1.985	moderately similar to (303)AT1G75140| Symbols: | Identical to Uncharacterized membrane protein At1g75140 [Arabidopsis Thaliana] (G	
DY029624	1.984	moderately similar to (232)AT5G39590| Symbols: | similar to unnamed protein product [Vitis vinifera] (GB:CAO61019.1); contains Inter	
BG544867	1.984	very weakly similar to (94.4)AT5G02970| Symbols: | hydrolase, alpha/beta fold family protein | chr5:695389-697410 FORWARD [8791]	
EV171366	1.984	moderately similar to (399)AT3G47000| Symbols: | glycosyl hydrolase family 3 protein | chr3:17324796-17327524 REVERSE [21486] 10	
EX127459	1.984	moderately similar to (278)AT1G10720| Symbols: | BSD domain-containing protein | chr1:3562965-3564447 FORWARD [21831]	
EV116125	1.984	no similarity	
JCVI_8821	1.984	weakly similar to (135)AT5G55450| Symbols: | protease inhibitor/seed storage/lipid transfer protein (LTP) family protein | chr5:2248478	
EX091891	1.984	moderately similar to (323)AT5G52900| Symbols: | similar to unnamed protein product [Vitis vinifera] (GB:CAO49548.1) | chr5:2147011	

JCVI_31592	1.983	moderately similar to (270)AT1G15390| Symbols: PDF1A | PDF1A (PEPTIDE DEFORMYLASE 1A); peptide deformylase | chr1:529464	
CD826891	1.983	weakly similar to (166)AT1G12990| Symbols: | glycosyl transferase family 17 protein | chr1:4433971-4435550 FORWARD [13979]	
JCVI_25295	1.983	moderately similar to (219)AT1G76130| Symbols: ATAMY2, AMY2 | AMY2/ATAMY2 (ALPHA-AMYLASE-LIKE 2); alpha-amylase | c	
JCVI_15702	1.983	moderately similar to (471)AT5G39040| Symbols: ATTAP2 | ATTAP2 (Arabidopsis thaliana transporter associated with antigen processin	
EE464311	1.983	moderately similar to (239)AT5G12120| Symbols: | ubiquitin-associated (UBA)/TS-N domain-containing protein | chr5:3916230-3918089	
JCVI_16172	1.983	moderately similar to (424)AT3G46200| Symbols: ATNUDT9 | ATNUDT9 (Arabidopsis thaliana Nudix hydrolase homolog 9); hydrolase	
JCVI_36370	1.983	no original description	
EX110089	1.983	weakly similar to (171)AT4G11160| Symbols: | translation initiation factor IF-2, mitochondrial, putative | chr4:6803842-6806722 FORWA	
EX101975	1.983	weakly similar to (197)AT2G45200| Symbols: ATGOS12, GOS12 | GOS12 (GOLGI SNARE 12); SNARE binding | chr2:18644763-18646	
EE521881	1.982	no similarity	
JCVI_41192	1.982	highly similar to (548)AT5G48800| Symbols: | phototropic-responsive NPH3 family protein | chr5:19804107-19806229 FORWARDmode	
EX038025	1.982	moderately similar to (240)AT1G67950| Symbols: | RNA recognition motif (RRM)-containing protein | chr1:25482547-25483735 REVEF	
EV197431	1.982	no similarity	
JCVI_15260	1.982	no original description	
JCVI_20989	1.982	moderately similar to (343)AT4G31990| Symbols: AAT3, ATAAT1 | ASP5 (ASPARTATE AMINOTRANSFERASE 5) | chr4:15470882-1	-2.564
EV151695	1.982	weakly similar to (125)AT5G26667| Symbols: | uridylate kinase / uridine monophosphate kinase (PYR6) | chr5:9276662-92	
JCVI_27443	1.981	moderately similar to (360)AT2G23960| Symbols: | defense-related protein, putative | chr2:10203139-10204690 FORWARD no original d	
JCVI_32675	1.981	highly similar to (696)AT2G39890| Symbols: ATPROT1, ProT1 | ProT1 (PROLINE TRANSPORTER 1) | chr2:16663100-16665280 FOR	
JCVI_1021	1.981	moderately similar to (202)AT3G15690| Symbols: | biotin carboxyl carrier protein of acetyl-CoA carboxylase-related | chr3:5317115-531	
EV049322	1.981	no similarity	
JCVI_18786	1.980	moderately similar to (442)AT5G17760| Symbols: | AAA-type ATPase family protein | chr5:5861278-5862303 REVERSE no original des	
JCVI_1474	1.980	moderately similar to (436)AT3G25530| Symbols: GHBDH, ATGHBDH | ATGHBDH/GHBDH; phosphogluconate dehydrogenase (decar	
EE442658	1.980	weakly similar to (150)AT3G06960| Symbols: PDE320 | PDE320 (PIGMENT DEFECTIVE 320) | chr3:2195222-2196510 REVERSE [20	
JCVI_13392	1.980	highly similar to (789)AT5G41870| Symbols: | glycoside hydrolase family 28 protein / polygalacturonase (pectinase) family protein | chr5:	
RC_DT317682	1.980	no similarity	
JCVI_37832	1.979	weakly similar to (149)AT1G73930| Symbols: | similar to unnamed protein product [Vitis vinifera] (GB:CAO68016.1); similar to hypothe	
ES913281	1.979	moderately similar to (282)AT3G04140| Symbols: | ankyrin repeat family protein | chr3:1087070-1089113 FORWARD [21431]	
EX023486	1.979	moderately similar to (351)AT3G06450| Symbols: | anion exchange family protein | chr3:1976091-1979309 REVERSE [21809]	
JCVI_13084	1.979	moderately similar to (411)AT2G24200| Symbols: | cytosol aminopeptidase | chr2:10294097-10296530 REVERSEmoderately similar to (	
EX095882	1.979	moderately similar to (306)AT2G26040| Symbols: | Bet v I allergen family protein | chr2:11101910-11102482 REVERSE [21824]	
EV203648	1.979	no similarity	
JCVI_3239	1.979	moderately similar to (332)AT3G20600| Symbols: NDR1 | NDR1 (NON RACE-SPECIFIC DISEASE RESISTANCE 1); signal transducer	
DW999620	1.978	weakly similar to (113)AT3G22840| Symbols: ELIP, ELIP1 | ELIP1 (EARLY LIGHT-INDUCABLE PROTEIN); chlorophyll binding | chr	2.387
JCVI_5314	1.978	moderately similar to (231)AT3G25480| Symbols: | rhodanese-like domain-containing protein | chr3:9236828-9237835 REVERSE no orig	
EV208226	1.978	weakly similar to (107)AT1G53240| Symbols: | malate dehydrogenase (NAD), mitochondrial | chr1:19858634-19860470 REVERSEvery v	
JCVI_36489	1.978	moderately similar to (273)AT5G13310| Symbols: | similar to unknown protein [Arabidopsis thaliana] (TAIR:AT5G13970.1); similar to u	
JCVI_10106	1.978	very weakly similar to (90.9)AT1G54440| Symbols: | 3'-5' exonuclease/ nucleic acid binding | chr1:20326951-20331598 FORWARD no or	
JCVI_10697	1.978	no original description	
ES899171	1.977	no similarity	
ES901785	1.977	moderately similar to (492)AT3G53160| Symbols: UGT73C7 | UGT73C7 (UDP-GLUCOSYL TRANSFERASE 73C7); UDP-glycosyltrans	
ES953560	1.977	weakly similar to (172)AT4G24990| Symbols: ATGP4 | ATGP4 (Arabidopsis thaliana geranylgeranylated protein) | chr4:12849983-12851	
CD814345	1.976	weakly similar to (154)AT1G21380| Symbols: | VHS domain-containing protein / GAT domain-containing protein | chr1:7485795-748802	
JCVI_28350	1.976	moderately similar to (341)AT3G12977| Symbols: | DNA binding | chr3:4143839-4145867 FORWARDweakly similar to (192)NAC77_O	
JCVI_26783	1.976	moderately similar to (436)AT4G24690| Symbols: | ubiquitin-associated (UBA)/TS-N domain-containing protein / octicosapeptide/Phox/I	
EV156864	1.976	moderately similar to (207)AT1G48900| Symbols: | signal recognition particle 54 kDa protein 3 / SRP54 (SRP-54C) | chr1:18088699-180	
JCVI_1712	1.975	moderately similar to (313)AT3G26210| Symbols: CYP71B23 | CYP71B23 (cytochrome P450, family 71, subfamily B, polypeptide 23); o	
EX053782	1.975	moderately similar to (256)AT1G03310| Symbols: ATISA2, ISA2, DBE1, BE2 | ATISA2/BE2/DBE1/ISA2 (DEBRANCHING ENZYME	
JCVI_5372	1.975	moderately similar to (333)AT4G02520| Symbols: ATPM24.1, ATPM24, GST2, ATGSTF2 | ATGSTF2 (Arabidopsis thaliana Glutathione	
EV227376	1.975	moderately similar to (327)AT4G16430| Symbols: | basic helix-loop-helix (bHLH) family protein | chr4:9267622-9269025 FORWARD [2	
H74590	1.974	weakly similar to (112)AT5G08590| Symbols: ASK2, SNRK2-1, SNRK2.1, SRK2G | ASK2 (ARABIDOPSIS SERINE/THREONINE KIN	
JCVI_25421	1.974	moderately similar to (303)AT5G62000| Symbols: ARF1-BP, HSS, ARF2 | ARF2 (AUXIN RESPONSE FACTOR 2) | chr5:24928085-249	
JCVI_39828	1.974	very weakly similar to (85.1)AT4G01037| Symbols: | similar to EMB1692 (EMBRYO DEFECTIVE 1692) [Arabidopsis thaliana] (TAIR:A	
JCVI_16761	1.974	moderately similar to (244)AT3G29200| Symbols: ATCM1, CM1 | CM1 (chorismate mutase 1); chorismate mutase | chr3:11165819-1116	
JCVI_35632	1.973	weakly similar to (134)AT5G08100| Symbols: | L-asparaginase / L-asparagine amidohydrolase | chr5:2593243-2594587 REVERSEweakly	
JCVI_11682	1.973	very weakly similar to (93.2)AT3G45010| Symbols: SCPL48 | SCPL48 (serine carboxypeptidase-like 48); serine carboxypeptidase | chr3:1	
JCVI_29999	1.973	no original description	2.246
DY024659	1.973	moderately similar to (262)AT3G47670| Symbols: | pectinesterase inhibitor | chr3:17586004-17586834 REVERSEvery weakly similar to (	
CD837207	1.973	weakly similar to (176)AT1G27370| Symbols: | squamosa promoter-binding protein-like 10 (SPL10) | chr1:9505441-9506984 REVERSE	
EV079741	1.973	moderately similar to (365)AT1G04200| Symbols: | similar to hypothetical protein OsJ_003532 [Oryza sativa (japonica cultivar-group)] (C	
JCVI_25900	1.973	moderately similar to (339)AT5G49945| Symbols: | Identical to Uncharacterized protein At5g49945 precursor [Arabidopsis Thaliana] (GI	
EV108922	1.973	moderately similar to (307)AT1G10340| Symbols: | ankyrin repeat family protein | chr1:3390477-3392483 REVERSE [21478] 40 738 73	-1.777
EX108439	1.972	moderately similar to (299)AT3G13950| Symbols: | similar to unknown protein [Arabidopsis thaliana] (TAIR:AT4G13266.1); similar to A	
EE431212	1.972	moderately similar to (259)AT2G44940| Symbols: | AP2 domain-containing transcription factor TINY, putative | chr2:18544369-1854525	
EV137358	1.972	weakly similar to (139)AT2G24790| Symbols: COL3 | COL3 (CONSTANS-LIKE 3); protein binding / transcription factor/ zinc ion bindin	
JCVI_2056	1.972	moderately similar to (257)AT4G26550| Symbols: | similar to unknown protein [Arabidopsis thaliana] (TAIR:AT5G56020.1); similar to u	
EV058343	1.972	weakly similar to (115)AT3G59020| Symbols: | protein transporter | chr3:21821950-21828395 REVERSE [21442]	
JCVI_16003	1.971	moderately similar to (387)AT4G32180| Symbols: ATPANK2 | ATPANK2 (PANTOTHENATE KINASE 2) | chr4:15538346-15543721 R	
EV086324	1.971	very weakly similar to (85.9)AT4G34450| Symbols: | coatomer gamma-2 subunit, putative / gamma-2 coat protein, putative / gamma-2 CO	
ES266157	1.971	moderately similar to (286)AT3G14790| Symbols: RHM3 | RHM3 (RHAMNOSE BIOSYNTHESIS 3); catalytic | chr3:4964798-4966882	
JCVI_11643	1.971	highly similar to (731)AT5G12290| Symbols: DGS1 | similar to expressed protein [Oryza sativa (japonica cultivar-group)] (GB:ABF96236	
JCVI_41084	1.971	weakly similar to (153)AT1G67850| Symbols: | similar to unknown protein [Arabidopsis thaliana] (TAIR:AT1G13000.2); similar to unkn	
JCVI_989	1.971	moderately similar to (343)AT3G26060| Symbols: ATPRX Q | ATPRX Q; antioxidant/ peroxiredoxin | chr3:9526044-9527360 FORWARI	
EV108083	1.971	weakly similar to (154)AT2G36800| Symbols: UGT73C5, DOGT1 | DOGT1 (DON-GLUCOSYLTRANSFERASE); UDP-glycosyltransfer	
ES963411	1.971	very weakly similar to (84.0)AT4G17700| Symbols: | similar to unknown protein [Arabidopsis thaliana] (TAIR:AT1G23950.5) | chr4:9854	
DY029463	1.971	weakly similar to (191)AT4G00650| Symbols: FLA, FRI | FRI (FRIGIDA) | chr4:269026-270363 FORWARD [18978] 14 583 583	
JCVI_20617	1.970	very weakly similar to (90.9)AT3G49210| Symbols: | similar to unknown protein [Arabidopsis thaliana] (TAIR:AT3G49200.1); similar to c	
EV034588	1.970	weakly similar to (179)AT5G10650| Symbols: | zinc finger (C3HC4-type RING finger) family protein | chr5:3365238-3367264 REVERSE	3.359
JCVI_27918	1.970	moderately similar to (451)AT5G18520| Symbols: | similar to unknown protein [Arabidopsis thaliana] (TAIR:AT3G09570.1); similar to u	
EV092740	1.970	moderately similar to (229)AT2G45690| Symbols: SSE, PEX16, SSE1 | SSE1 (SHRUNKEN SEED 1) | chr2:18830539-18832675 REVER	
JCVI_41596	1.969	weakly similar to (155)AT1G32770| Symbols: ANAC012, SND1, NST3 | ANAC012/NST3/SND1 (ARABIDOPSIS NAC DOMAIN CON	
ES989764	1.969	moderately similar to (333)AT4G33210| Symbols: | F-box family protein (FBL15) | chr4:16015974-16020700 REVERSE [21425]	

EV226426	1.969	no similarity	2.086
JCVI_18488	1.969	moderately similar to (467)AT1G08920| Symbols: | sugar transporter, putative | chr1:2867449-2870193 FORWARDvery weakly similar to	
ES903746	1.969	weakly similar to (182)AT3G07570| Symbols: | membrane protein, putative | chr3:2418211-2420212 REVERSE [21432] 19 438 438	
EV025257	1.968	weakly similar to (107)AT1G12775| Symbols: | binding | chr1:4353904-4355838 FORWARD [21441] 1 707 722	
DY002793	1.968	moderately similar to (265)AT2G29260| Symbols: | tropinone reductase, putative / tropine dehydrogenase, putative | chr2:12589600-1259	
JCVI_41126	1.968	no original description	
ES983579	1.968	no similarity	1.322
EE421244	1.968	weakly similar to (185)AT2G14110| Symbols: | similar to unnamed protein product [Vitis vinifera] (GB:CAO43153.1); contains InterPro	
EE558221	1.968	very weakly similar to (96.3)AT3G03310| Symbols: | lecithin:cholesterol acyltransferase family protein / LACT family protein | chr3:7787	-2.579
EX036847	1.967	weakly similar to (199)AT3G29090| Symbols: | pectinesterase family protein | chr3:11075041-11076572 FORWARD [21811]	-2.055
EV036702	1.967	moderately similar to (204)AT1G20760| Symbols: | calcium-binding EF hand family protein | chr1:7209505-7214763 FORWARD [21441	
JCVI_24747	1.967	highly similar to (669)AT3G02050| Symbols: ATKUP3, ATKT4, KUP3 | KUP3 (K+ uptake permease 3); potassium ion transmembrane tr	
JCVI_2207	1.967	moderately similar to (325)AT3G62850| Symbols: | zinc finger protein-related | chr3:23248462-23250130 REVERSE no original descripti	
JCVI_41	1.967	highly similar to (526)AT1G50010| Symbols: TUA2 | TUA2 (tubulin alpha-2 chain) | chr1:18521405-18523397 FORWARDhighly similar	
EV217127	1.967	weakly similar to (139)AT2G39435| Symbols: | similar to unknown protein [Arabidopsis thaliana] (TAIR:AT3G53540.1); similar to unnar	
EV182136	1.967	no similarity	
JCVI_2535	1.967	moderately similar to (347)AT5G19990| Symbols: ATSUG1, RPT6A | RPT6A; ATPase | chr5:6752146-6754920 FORWARDvery weakly	
JCVI_23408	1.966	highly similar to (605)AT1G58080| Symbols: HISN1A, ATATP-PRT1 | ATATP-PRT1 (ATP PHOSPHORIBOSYL TRANSFERASE) | ch	
JCVI_26075	1.966	no original description	
JCVI_33755	1.966	moderately similar to (457)AT5G44370| Symbols: | transporter-related | chr5:17892374-17893672 REVERSE no original description	
EV164130	1.966	no similarity	
JCVI_21745	1.966	moderately similar to (213)AT5G40280| Symbols: ATFTB, WIG, ERA1 | ERA1 (ENHANCED RESPONSE TO ABA 1); protein farnesylt	
JCVI_13114	1.966	highly similar to (528)AT5G10240| Symbols: ASN3 | ASN3 (ASPARAGINE SYNTHETASE 3); asparagine synthase (glutamine-hydrolyz	1.422
CX188750	1.965	moderately similar to (343)AT3G03050| Symbols: KJK, ATCSLD3, CSLD3 | CSLD3 (CELLULOSE SYNTHASE-LIKE 3); cellulose synt	
JCVI_41753	1.965	moderately similar to (345)AT4G22120| Symbols: | early-responsive to dehydration protein-related / ERD protein-related | chr4:11715988	
JCVI_12196	1.965	weakly similar to (126)AT3G19520| Symbols: | similar to unknown protein [Arabidopsis thaliana] (TAIR:AT1G28500.1); contains InterPr	
JCVI_7882	1.965	moderately similar to (325)AT4G33010| Symbols: ATGLDP1 | ATGLDP1 (ARABIDOPSIS THALIANA GLYCINE DECARBOXYLASE	
JCVI_10267	1.965	highly similar to (739)AT4G35800| Symbols: RNA_POL_II_LSRNA_POL_II_LS, RNA_POL_II_LS, RPB1, NRPB1 | NRPB1 (RNA POI	
DY010661	1.965	weakly similar to (107)AT3G02290| Symbols: | zinc finger (C3HC4-type RING finger) family protein | chr3:459392-460235 FORWARD	
RC_CX194333	1.965	no similarity	
JCVI_34152	1.964	highly similar to (596)AT1G78000| Symbols: SEL1, SULTR1;2 | SULTR1;2 (SULFATE TRANSPORTER 1;2) | chr1:29334783-2933777	
JCVI_6319	1.964	moderately similar to (231)AT2G26590| Symbols: | adhesion regulating molecule family | chr2:11318260-11321795 REVERSE no origina	
EV088175	1.964	moderately similar to (255)AT3G14450| Symbols: CID9 | CID9 (CTC-Interacting Domain 9); RNA binding / protein binding | chr3:48497	
ES964091	1.964	no similarity	
ES953970	1.963	no similarity	
EV085329	1.963	weakly similar to (137)AT5G18410| Symbols: PIR, KLK, PIR121, SRA1, PIRP | KLK/PIR/PIR121/PIRP (KLUNKER, PIROGI) | chr5:60	
JCVI_16679	1.963	moderately similar to (434)AT5G01850| Symbols: | protein kinase, putative | chr5:332826-334177 FORWARDvery weakly similar to (95.	
EV110569	1.963	no similarity	
DT317696	1.963	moderately similar to (208)AT1G78780| Symbols: | pathogenesis-related family protein | chr1:29626340-29627324 REVERSEvery weakly	
ES910337	1.963	no similarity	
EV161338	1.963	moderately similar to (313)AT2G39450| Symbols: MTP11, ATMTP11 | ATMTP11/MTP11; cation transmembrane transporter/ manganes	
EX053157	1.963	no similarity	
JCVI_7205	1.962	moderately similar to (398)AT3G48750| Symbols: CDKA;1, CDC2AAT, CDK2, CDC2, CDC2A, CDKA1 | CDC2/CDC2A/CDC2AAT/CI	
EE458338	1.962	no similarity	
EV100127	1.962	weakly similar to (163)AT1G75900| Symbols: | family II extracellular lipase 3 (EXL3) | chr1:28502840-28504604 FORWARD [21477] 1(	
EE422485	1.962	weakly similar to (167)AT2G47110| Symbols: UBQ6 | UBQ6 (ubiquitin 6); protein binding | chr2:19351771-19352244 FORWARDweakly	
EX039589	1.962	weakly similar to (108)AT3G10050| Symbols: OMR1 | OMR1 (L-O-METHYLTHREONINE RESISTANT 1); L-threonine ammonia-lyase	
JCVI_35213	1.962	moderately similar to (216)AT2G28360| Symbols: | SIT4 phosphatase-associated family protein | chr2:12131681-12136956 REVERSE nc	
JCVI_17569	1.962	moderately similar to (274)AT1G12244| Symbols: | DNA binding / hydrolase, acting on ester bonds / nuclease/ nucleic acid binding / reco	
DY024965	1.962	moderately similar to (389)AT1G65620| Symbols: AS2 | AS2 (ASYMMETRIC LEAVES 2) | chr1:24403809-24404408 FORWARD [189	
EE520613	1.962	very weakly similar to (90.5)AT5G52240| Symbols: ATMP1, ATMAPR5, MSBP1 | MSBP1 (MEMBRANE STEROID BINDING PROTE	
EX089256	1.962	weakly similar to (165)AT2G26900| Symbols: | bile acid:sodium symporter family protein | chr2:11482234-11484948 REVERSE [21823]	-1.335
JCVI_18441	1.962	no original description	
JCVI_29438	1.962	weakly similar to (160)AT3G14680| Symbols: CYP72A14 | CYP72A14 (cytochrome P450, family 72, subfamily A, polypeptide 14); oxyg	
CD842383	1.961	moderately similar to (278)AT4G22920| Symbols: ATNYE1, NYE1 | ATNYE1/NYE1 (NON-YELLOWING 1) | chr4:12016787-1201798	
EE567629	1.961	very weakly similar to (80.1)AT3G10450| Symbols: SCPL7 | SCPL7; serine carboxypeptidase | chr3:3249775-3252325 FORWARD [2015	
JCVI_27973	1.961	weakly similar to (168)AT1G51700| Symbols: ADOF1 | ADOF1 (Arabidopsis dof zinc finger protein 1); DNA binding / transcription facto	
JCVI_152	1.961	moderately similar to (451)AT5G16710| Symbols: DHAR3 | DHAR3 (DEHYDROASCORBATE REDUCTASE 1); glutathione dehydroge	
AM390661	1.961	moderately similar to (208)AT4G35230| Symbols: | protein kinase family protein | chr4:16755330-16758046 REVERSE [20118]	
JCVI_17242	1.961	no original description	
EE533698	1.961	moderately similar to (364)AT4G21180| Symbols: | DNAJ heat shock N-terminal domain-containing protein / sec63 domain-containing pr	
JCVI_27694	1.961	no original description	-1.818
JCVI_13929	1.960	moderately similar to (372)AT4G14410| Symbols: | basic helix-loop-helix (bHLH) family protein | chr4:8300414-8301499 FORWARD nc	
JCVI_31191	1.960	weakly similar to (114)AT3G62290| Symbols: ATARFA1E | ATARFA1E (ADP-RIBOSYLATION FACTOR A1E); GTP binding / phosph	
CN728815	1.960	no similarity	
EE524397	1.960	moderately similar to (322)AT1G01290| Symbols: CNX3 | CNX3 (COFACTOR OF NITRATE REDUCTASE AND XANTHINE DEHYD	
ES897957	1.960	no similarity	
EE451264	1.960	weakly similar to (158)AT3G15890| Symbols: | protein kinase family protein | chr3:5374395-5376120 FORWARD [20194]	
JCVI_15956	1.959	moderately similar to (287)AT1G79680| Symbols: | wall-associated kinase, putative | chr1:29985081-29987642 REVERSEvery weakly sir	-1.383
JCVI_34487	1.959	highly similar to (650)AT5G06530| Symbols: | ABC transporter family protein | chr5:1990335-1994606 REVERSEweakly similar to (130	-1.378
JCVI_8456	1.959	weakly similar to (117)AT2G39705| Symbols: RTFL8, DVL11 | DVL11/RTFL8 (ROTUNDIFOLIA LIKE 8) | chr2:16564116-16564379 F	
JCVI_19264	1.959	moderately similar to (431)AT4G28300| Symbols: | hydroxyproline-rich glycoprotein family protein | chr4:14015420-14016829 FORWAI	
JCVI_1673	1.958	moderately similar to (411)AT3G13920| Symbols: RH4, TIF4A1, EIF4A1 | EIF4A1 (eukaryotic translation initiation factor 4A-1) | chr3:45	
JCVI_29115	1.958	highly similar to (594)AT3G23000| Symbols: SnRK3.10, PKS7, ATSRPK1, ATSR2, CIPK7 | CIPK7 (CBL-INTERACTING PROTEIN K	
AT000736	1.958	weakly similar to (129)AT5G19880| Symbols: | peroxidase, putative | chr5:6720580-6722413 REVERSEvery weakly similar to (80.1)PER	
EV100450	1.958	weakly similar to (171)AT5G10740| Symbols: | protein phosphatase 2C-related / PP2C-related | chr5:3393798-3395849 REVERSE [2147	
EV005150	1.958	moderately similar to (377)AT1G80070| Symbols: EMB158, EMB33, EMB177, EMB14, SUS2 | SUS2 (ABNORMAL SUSPENSOR 2) | c	
EE526916	1.958	weakly similar to (119)AT2G37380| Symbols: | similar to unknown protein [Arabidopsis thaliana] (TAIR:AT2G39370.1); similar to unnai	-1.588
JCVI_4620	1.958	moderately similar to (332)AT3G12800| Symbols: | short-chain dehydrogenase/reductase (SDR) family protein | chr3:4063470-4064764 F	
AM058165	1.958	weakly similar to (193)AT1G70505| Symbols: | similar to unknown protein [Arabidopsis thaliana] (TAIR:AT1G10660.3); similar to unkn	
EV111253	1.957	no similarity	1.635

JCVI_34669	1.957	moderately similar to (248)AT2G04240| Symbols: XERICO | XERICO; protein binding / zinc ion binding | chr2:1461813-1462301 REVE	
JCVI_14830	1.957	very weakly similar to (84.7)AT3G21110| Symbols: ATPURC, PUR7, PURC | PUR7 (PURIN 7); phosphoribosylaminoimidazolesuccinoca	
JCVI_21702	1.957	highly similar to (586)AT4G36530| Symbols: | hydrolase, alpha/beta fold family protein | chr4:17240123-17241773 REVERSE no origina	
JCVI_2246	1.957	weakly similar to (149)AT2G37480| Symbols: | similar to unknown protein [Arabidopsis thaliana] (TAIR:AT3G53670.1); similar to hypot	
JCVI_7865	1.957	no original description	
EX133223	1.956	moderately similar to (421)AT3G06490| Symbols: AtMYB108, BOS1, MYB108 | MYB108 (MYB DOMAIN PROTEIN 108); DNA bindi	
EE568731	1.956	no similarity	-2.822
JCVI_25381	1.956	moderately similar to (430)AT5G09220| Symbols: AAP2 | AAP2 (AMINO ACID PERMEASE 2); amino acid transmembrane transporter	
JCVI_29247	1.956	weakly similar to (195)AT2G47900| Symbols: AtTLP3 | AtTLP3 (TUBBY LIKE PROTEIN 3); phosphoric diester hydrolase/ transcription	
JCVI_37605	1.955	moderately similar to (351)AT1G73200| Symbols: | similar to unknown protein [Arabidopsis thaliana] (TAIR:AT1G17820.1); similar to u	
EE473477	1.955	weakly similar to (167)AT5G62300| Symbols: | 40S ribosomal protein S20 (RPS20C) | chr5:25038614-25039461 REVERSEweakly simila	
EX127158	1.955	moderately similar to (297)AT3G18850| Symbols: LPAT5 | LPAT5 | chr3:6499535-6500846 REVERSEvery weakly similar to (100)LPAT	1.280
JCVI_16736	1.955	moderately similar to (291)AT1G14810| Symbols: | semialdehyde dehydrogenase family protein | chr1:5102679-5104628 REVERSE no o	
JCVI_3032	1.955	moderately similar to (286)AT5G12170| Symbols: | similar to unknown protein [Arabidopsis thaliana] (TAIR:AT5G19380.1); similar to u	
CN730184	1.955	weakly similar to (171)AT1G71690| Symbols: | similar to unknown protein [Arabidopsis thaliana] (TAIR:AT1G33800.1); similar to unkno	
AM388072	1.954	moderately similar to (244)AT5G10940| Symbols: | transducin family protein / WD-40 repeat family protein | chr5:3448891-3454128 REV	
JCVI_27187	1.954	no original description	
JCVI_17994	1.954	highly similar to (512)AT2G43950| Symbols: OEP37 | OEP37 | chr2:18207906-18209720 REVERSE no original description	
JCVI_4423	1.954	moderately similar to (315)AT3G13340| Symbols: | WD-40 repeat family protein | chr3:4332377-4334610 FORWARD no original descrip	
JCVI_4185	1.954	highly similar to (608)AT3G48425| Symbols: | endonuclease/exonuclease/phosphatase family protein | chr3:17942895-17945478 FORWA	
EE410422	1.954	moderately similar to (252)AT3G47530| Symbols: | pentatricopeptide (PPR) repeat-containing protein | chr3:17528367-17530142 REVER	
JCVI_37802	1.954	highly similar to (619)AT1G69830| Symbols: ATAMY3, AMY3 | AMY3/ATAMY3 (ALPHA-AMYLASE-LIKE 3); alpha-amylase | chr1:	
EX135943	1.954	very weakly similar to (82.4)AT3G06760| Symbols: | Identical to Protein DEHYDRATION-INDUCED 19 homolog 4 (DI19-4) [Arabidop	2.675
JCVI_41163	1.954	weakly similar to (145)AT5G14050| Symbols: | transducin family protein / WD-40 repeat family protein | chr5:4533541-4535181 FORWA	
JCVI_1195	1.953	moderately similar to (336)AT3G61460| Symbols: BRH1 | BRH1 (BRASSINOSTEROID-RESPONSIVE RING-H2); protein binding / zinc	
EX115214	1.953	moderately similar to (280)AT1G47750| Symbols: PEX11A | PEX11A | chr1:17571828-17572574 REVERSE [21835]	-3.719
ES900502	1.953	moderately similar to (421)AT1G67530| Symbols: | armadillo/beta-catenin repeat family protein / U-box domain-containing family protein	
JCVI_42463	1.953	highly similar to (655)AT5G50390| Symbols: | pentatricopeptide (PPR) repeat-containing protein | chr5:20538015-20540206 REVERSE n	
EE447677	1.953	no similarity	
DY027422	1.953	moderately similar to (267)AT3G26840| Symbols: | esterase/lipase/thioesterase family protein | chr3:9894045-9897391 FORWARD [1897	
JCVI_6929	1.953	moderately similar to (207)AT3G15190| Symbols: | chloroplast 30S ribosomal protein S20, putative | chr3:5116223-5117419 FORWARD	
JCVI_35112	1.953	highly similar to (587)AT5G54630| Symbols: | zinc finger protein-related | chr5:22209833-22211486 REVERSE no original description	
CN830579	1.953	weakly similar to (139)AT4G20440| Symbols: SMB | SMB | chr4:11022854-11023627 REVERSE [15793]	
JCVI_15031	1.953	no original description	
JCVI_5180	1.953	moderately similar to (348)AT5G55710| Symbols: | similar to tic20 protein-related [Arabidopsis thaliana] (TAIR:AT2G47840.1); similar t	
JCVI_9310	1.952	moderately similar to (211)AT5G40690| Symbols: | similar to unknown protein [Arabidopsis thaliana] (TAIR:AT2G41730.1); similar to u	
JCVI_21962	1.952	highly similar to (643)AT2G17760| Symbols: | aspartyl protease family protein | chr2:7720570-7723351 FORWARDvery weakly similar t	1.710
JCVI_37993	1.952	no original description	
EX045165	1.952	moderately similar to (206)AT3G03470| Symbols: CYP89A9 | CYP89A9 (cytochrome P450, family 87, subfamily A, polypeptide 9); oxyg	
EV176382	1.952	weakly similar to (149)AT4G35450| Symbols: AFT, AKR2A, AKR2 | AKR2 (ANKYRIN REPEAT-CONTAINING PROTEIN 2); protein	
JCVI_1026	1.952	no original description	
CD821014	1.951	no similarity	
EE531027	1.951	weakly similar to (179)AT2G25580| Symbols: | binding | chr2:10895180-10897027 FORWARD [20175]	-3.092
EV026824	1.951	moderately similar to (353)AT5G67385| Symbols: | signal transducer | chr5:26901980-26904309 FORWARDweakly similar to (142)NPH	
JCVI_30020	1.951	moderately similar to (338)AT2G27090| Symbols: | similar to unknown protein [Arabidopsis thaliana] (TAIR:AT4G39790.1); similar to u	
EV151955	1.951	no similarity	
JCVI_18171	1.951	moderately similar to (419)AT1G77740| Symbols: | 1-phosphatidylinositol-4-phosphate 5-kinase, putative / PIP kinase, putative / PtdIns(4	
JCVI_39418	1.951	very weakly similar to (92.4)AT5G46795| Symbols: MSP2 | MSP2 (MICROSPORE-SPECIFIC PROMOTER 2) | chr5:19004282-1900539	
JCVI_23801	1.951	moderately similar to (394)AT1G06900| Symbols: | metalloendopeptidase | chr1:2115154-2120634 REVERSE no original description	
JCVI_31187	1.950	moderately similar to (436)AT5G08730| Symbols: | IBR domain-containing protein | chr5:2845825-2847416 REVERSE no original descri	
JCVI_34684	1.950	very weakly similar to (85.5)AT4G01840| Symbols: ATTPK5, KCO5, ATKCO5 | KCO5 (Ca2+ activated outward rectifying K+ channel 5)	
JCVI_2682	1.950	moderately similar to (239)AT4G01660| Symbols: ATATH10, ABC1At | ABC1At (ARABIDOPSIS THALIANA ABC TRANSPORTER 1	
JCVI_32367	1.950	no original description	
EV153477	1.950	weakly similar to (200)AT5G10790| Symbols: UBP22 | UBP22 (UBIQUITIN-SPECIFIC PROTEASE 22); ubiquitin-specific protease | chr	
EV003265	1.949	moderately similar to (234)AT3G60880| Symbols: | dihydrodipicolinate synthase 1 (DHDPS1) (DHDPS) (DHPS1) | chr3:22506061-22507	
JCVI_8410	1.949	moderately similar to (233)AT3G03950| Symbols: ECT1 | ECT1 | chr3:1021509-1023774 FORWARD no original description	
JCVI_24334	1.949	highly similar to (719)AT2G22330| Symbols: CYP79B3 | CYP79B3 (cytochrome P450, family 79, subfamily B, polypeptide 3); oxygen bi	
JCVI_17462	1.949	moderately similar to (286)AT3G23610| Symbols: | dual specificity protein phosphatase (DsPTP1) | chr3:8478287-8479648 FORWARDv	
JCVI_23291	1.949	moderately similar to (471)AT2G23980| Symbols: CNGC6, ATCNGC6 | ATCNGC6 (CYCLIC NUCLEOTIDE GATED CHANNEL 6); c	
CN727582	1.949	very weakly similar to (94.0)AT1G53240| Symbols: | malate dehydrogenase (NAD), mitochondrial | chr1:19858634-19860470 REVERSEv	-1.177
JCVI_14109	1.949	very weakly similar to (87.0)AT1G63206| Symbols: | similar to unknown protein [Arabidopsis thaliana] (TAIR:AT1G63190.1); contains In	
CD834689	1.949	weakly similar to (102)AT3G03330| Symbols: | short-chain dehydrogenase/reductase (SDR) family protein | chr3:783579-786155 REVER	
EV087740	1.949	no similarity	
EE472389	1.949	moderately similar to (335)AT1G77620| Symbols: | nucleoside-triphosphatase/ nucleotide binding | chr1:29172828-29177761 REVERSE	
EX081446	1.949	weakly similar to (106)AT5G52320| Symbols: CYP96A4 | CYP96A4 (cytochrome P450, family 96, subfamily A, polypeptide 4); oxygen b	
JCVI_21364	1.949	highly similar to (709)AT3G07690| Symbols: | glycerol-3-phosphate dehydrogenase (NAD+) | chr3:2457238-2459384 FORWARD no orig	
CX279636	1.949	moderately similar to (449)AT4G29830| Symbols: VIP3 | VIP3 (VERNALIZATION INDEPENDENCE 3); nucleotide binding | chr4:1459	
JCVI_14602	1.948	no original description	
EV092570	1.948	moderately similar to (347)AT5G39000| Symbols: | protein kinase family protein | chr5:15629090-15631711 FORWARD [21476] 45 899	
JCVI_16328	1.948	moderately similar to (385)AT1G73720| Symbols: | transducin family protein / WD-40 repeat family protein | chr1:27728720-27733383 F	
JCVI_26258	1.948	moderately similar to (309)AT2G34360| Symbols: | MATE efflux family protein | chr2:14514373-14517138 FORWARD no original descr	
JCVI_15969	1.948	moderately similar to (409)AT4G36790| Symbols: | transporter-related | chr4:17336365-17338309 FORWARD no original description	
EX079315	1.948	no similarity	
JCVI_7475	1.947	moderately similar to (453)AT5G23660| Symbols: MTN3 | MTN3 (ARABIDOPSIS HOMOLOG OF MEDICAGO TRUNCATULA MTN	
JCVI_1292	1.947	moderately similar to (377)AT5G19550| Symbols: AAT2, ASP2 | ASP2 (ASPARTATE AMINOTRANSFERASE 2) | chr5:6598203-66015	
JCVI_32303	1.947	highly similar to (513)AT5G04670| Symbols: | similar to nucleic acid binding [Arabidopsis thaliana] (TAIR:AT4G32620.1); similar to unk	
EV119790	1.947	moderately similar to (302)AT2G19160| Symbols: | similar to unknown protein [Arabidopsis thaliana] (TAIR:AT4G30060.1); similar to u	
JCVI_15434	1.947	moderately similar to (263)AT1G67325| Symbols: | binding | chr1:25213488-25216075 REVERSE no original description	
JCVI_2051	1.947	weakly similar to (146)AT3G07560| Symbols: APM2, PEX13 | APM2/PEX13 (ABERRANT PEROXISOME MORPHOLOGY 2); protein	
DY026204	1.947	moderately similar to (271)AT4G37590| Symbols: | phototropic-responsive NPH3 family protein | chr4:17663074-17665293 REVERSE [1	
ES899299	1.947	no similarity	2.076

JCVI_41298	1.947	no original description	
JCVI_9824	1.946	moderately similar to (491)AT5G42920| Symbols: | similar to unknown protein [Arabidopsis thaliana] (TAIR:AT1G45233.2); similar to h	
EV100917	1.946	no similarity	
EV080676	1.946	weakly similar to (114)AT1G65060| Symbols: 4CL3 | 4CL3 (4-coumarate:CoA ligase 3); 4-coumarate-CoA ligase | chr1:24171590-241751	
EE440241	1.946	weakly similar to (142)AT5G57190| Symbols: PSD2 | PSD2 (PHOSPHATIDYLSERINE DECARBOXYLASE 2); phosphatidylserine deca	
AM061634	1.945	very weakly similar to (95.1)AT1G77450| Symbols: ANAC032 | ANAC032 (Arabidopsis NAC domain containing protein 32); transcriptior	
EE568831	1.945	no similarity	
JCVI_11067	1.944	moderately similar to (440)AT2G35860| Symbols: FLA16 | FLA16 (FASCICLIN-LIKE ARABINOGALACTAN PROTEIN 16 PRECURS	
EX119239	1.944	moderately similar to (444)AT2G06040| Symbols: | similar to unknown protein [Arabidopsis thaliana] (TAIR:AT5G21900.1); similar to u	
JCVI_31897	1.944	weakly similar to (102)AT1G50020| Symbols: | similar to unnamed protein product [Vitis vinifera] (GB:CAO49863.1) | chr1:18523812-18	
JCVI_14596	1.944	moderately similar to (338)AT3G29030| Symbols: ATEXP5, ATHEXP ALPHA 1.4, ATEXPA5 | ATEXPA5 (ARABIDOPSIS THALIAN	
JCVI_17543	1.944	moderately similar to (333)AT2G33150| Symbols: PED1, KAT2 | KAT2/PED1 (PEROXISOME DEFECTIVE 1); acetyl-CoA C-acyltrans	
EV032062	1.944	no similarity	
EX037191	1.944	no similarity	
JCVI_7919	1.943	moderately similar to (236)AT3G05120| Symbols: ATGID1A, GID1A | ATGID1A/GID1A (GA INSENSITIVE DWARF1A); hydrolase | c	
JCVI_42378	1.943	weakly similar to (182)AT1G61990| Symbols: | mitochondrial transcription termination factor-related / mTERF-related | chr1:22915118-2	
AM389810	1.943	moderately similar to (340)AT1G62960| Symbols: ACS10 | ACS10 (ACC SYNTHASE 10); 1-aminocyclopropane-1-carboxylate synthase	
EV099578	1.943	moderately similar to (329)AT4G38180| Symbols: FRS5 | FRS5 (FAR1-RELATED SEQUENCE 5); zinc ion binding | chr4:17906696-179	-1.560
JCVI_6708	1.943	moderately similar to (388)AT1G50250| Symbols: FTSH1 | FTSH1 (FtsH protease 1); ATP-dependent peptidase/ ATPase/ metallopeptidas	
EX036279	1.943	no similarity	
RC_JCVI_24322	1.942	no original description	
JCVI_29638	1.942	weakly similar to (154)AT3G10480| Symbols: ANAC050 | ANAC050 | chr3:3264415-3266786 FORWARDvery weakly similar to (82.0)N	
EE462520	1.942	no similarity	
DN964168	1.942	moderately similar to (415)AT5G18490| Symbols: | similar to unknown protein [Arabidopsis thaliana] (TAIR:AT3G04350.1); similar to h	
JCVI_2953	1.942	moderately similar to (338)AT3G59490| Symbols: | similar to unnamed protein product [Vitis vinifera] (GB:CAO64819.1) | chr3:2199563	
JCVI_8977	1.942	weakly similar to (145)AT3G10760| Symbols: | myb family transcription factor | chr3:3369819-3370826 FORWARD no original descripti	-2.996
JCVI_37609	1.942	no original description	
JCVI_17911	1.942	moderately similar to (254)AT2G15530| Symbols: | zinc finger (C3HC4-type RING finger) family protein | chr2:6781232-6784150 FORW	
JCVI_23906	1.942	moderately similar to (233)AT1G64840| Symbols: | F-box family protein | chr1:24097673-24098827 FORWARD no original description	
CO750652	1.941	weakly similar to (152)AT5G10940| Symbols: | transducin family protein / WD-40 repeat family protein | chr5:3448891-3454128 REVER	
EV085714	1.941	no similarity	
JCVI_7939	1.941	weakly similar to (181)AT1G62960| Symbols: ACS10 | ACS10 (ACC SYNTHASE 10); 1-aminocyclopropane-1-carboxylate synthase | chr	
EE480231	1.941	weakly similar to (179)AT1G20760| Symbols: | calcium-binding EF hand family protein | chr1:7209505-7214763 FORWARD [20132]	
CO750499	1.941	weakly similar to (175)AT1G18360| Symbols: | hydrolase, alpha/beta fold family protein | chr1:6316989-6319197 REVERSE [16161]	
JCVI_2266	1.941	moderately similar to (304)AT1G58070| Symbols: | similar to hypothetical protein OsI_024693 [Oryza sativa (indica cultivar-group)] (GB	
JCVI_14322	1.941	moderately similar to (439)AT3G03990| Symbols: | esterase/lipase/thioesterase family protein | chr3:1033795-1034598 FORWARD no or	
JCVI_3139	1.940	moderately similar to (320)AT4G34131| Symbols: UGT73B3 | UGT73B3 (UDP-GLUCOSYL TRANSFERASE 73B3); UDP-glycosyltrans	-2.322
JCVI_19963	1.940	weakly similar to (130)AT3G18295| Symbols: | similar to unknown protein [Arabidopsis thaliana] (TAIR:AT1G48770.1); similar to unnar	
JCVI_24864	1.940	moderately similar to (291)AT1G20980| Symbols: FBR6, SPL1R2, ATSPL14, SPL14 | SPL14 (SQUAMOSA PROMOTER BINDING PR	
EE448414	1.939	weakly similar to (124)AT5G08139| Symbols: | zinc finger (C3HC4-type RING finger) family protein | chr5:2616488-2617618 FORWAR	
JCVI_5121	1.939	no original description	
JCVI_2674	1.939	highly similar to (639)AT5G62190| Symbols: PRH75 | PRH75 (plant RNA helicase 75); ATP-dependent helicase | chr5:24997768-250011	
EE483457	1.939	no similarity	
JCVI_6612	1.939	moderately similar to (257)AT3G62550| Symbols: | universal stress protein (USP) family protein | chr3:23147170-23148050 FORWARD	
EE542775	1.939	weakly similar to (114)AT2G43210| Symbols: | UBX domain-containing protein | chr2:17968019-17970637 FORWARD [20124] 1 294 3	
JCVI_31820	1.939	moderately similar to (306)AT1G11860| Symbols: | aminomethyltransferase, putative | chr1:4001800-4003244 FORWARDmoderately sin	
DW998405	1.939	no similarity	
CD832002	1.939	no similarity	
JCVI_4909	1.939	weakly similar to (197)AT5G59870| Symbols: HTA6 | HTA6; DNA binding | chr5:24132831-24133370 REVERSEweakly similar to (179	
ES940878	1.939	moderately similar to (232)AT5G56090| Symbols: COX15 | COX15 (CYTOCHROME C OXIDASE 15) | chr5:22731860-22733831 FORW	
JCVI_20481	1.939	moderately similar to (267)AT3G57050| Symbols: CBL | CBL (CYSTATHIONINE BETA-LYASE) | chr3:21122918-21125500 REVERSI	
JCVI_34909	1.938	moderately similar to (207)AT2G13290| Symbols: | glycosyl transferase family 17 protein | chr2:5519140-5520225 FORWARD no origina	
JCVI_7647	1.938	moderately similar to (230)AT5G35520| Symbols: | kinetochore protein-related | chr5:13718626-13720719 FORWARD no original descri	
ES989751	1.938	weakly similar to (165)AT4G17718| Symbols: | Encodes a defensin-like (DEFL) family protein. | chr4:9861609-9862008 REVERSE [214	
JCVI_17368	1.938	moderately similar to (306)AT2G37060| Symbols: | CCAAT-box binding transcription factor, putative | chr2:15583415-15584739 FORW	
JCVI_6407	1.938	moderately similar to (419)AT2G18030| Symbols: | peptide methionine sulfoxide reductase family protein | chr2:7847289-7848578 FORV	
EE567903	1.938	moderately similar to (284)AT1G76890| Symbols: AT-GT2, GT2 | GT2; transcription factor | chr1:28878105-28880097 REVERSE [2015	
JCVI_29439	1.938	no original description	
JCVI_14117	1.937	moderately similar to (218)AT2G46535| Symbols: | similar to unknown protein [Arabidopsis thaliana] (TAIR:AT3G61840.1) | chr2:19116	
JCVI_14784	1.937	very weakly similar to (92.4)AT1G21320| Symbols: | nucleic acid binding / nucleotide binding | chr1:7462822-7465188 REVERSE no orig	
JCVI_1828	1.937	moderately similar to (289)AT4G38040| Symbols: | exostosin family protein | chr4:17867495-17869125 FORWARD no original descriptio	
EV183791	1.937	no similarity	
JCVI_5126	1.937	highly similar to (526)AT1G12410| Symbols: CLPP2, NCLPP2, CLPR2 | CLPR2 (Clp protease proteolytic subunit 2); endopeptidase Clp |	
ES999191	1.937	moderately similar to (205)AT5G64813| Symbols: LIP1 | LIP1 (LIGHT INSENSITIVE PERIOD1); GTPase | chr5:25928062-25929851 F	
JCVI_38074	1.936	moderately similar to (370)AT5G62130| Symbols: | Per1-like protein-related | chr5:24967911-24970144 REVERSE no original descriptio	
EV057245	1.936	no similarity	
EE552516	1.936	no similarity	
EV125692	1.936	no similarity	-2.043
JCVI_11710	1.936	moderately similar to (356)AT1G11790| Symbols: ADT1 | ADT1 (AROGENATE DEHYDRATASE 1); prephenate dehydratase | chr1:398	
JCVI_126	1.936	moderately similar to (278)AT2G47320| Symbols: | peptidyl-prolyl cis-trans isomerase cyclophilin-type family protein | chr2:19434781-19	
CN735885	1.936	very weakly similar to (87.4)AT1G20225| Symbols: | similar to unknown protein [Arabidopsis thaliana] (TAIR:AT1G76020.1); similar to u	
JCVI_14785	1.936	weakly similar to (197)AT5G60790| Symbols: ATGCN1 | ATGCN1 (Arabidopsis thaliana general control non-repressible 1) | chr5:244709	
EE504692	1.935	moderately similar to (267)AT3G19260| Symbols: LAG1 HOMOLOG 2 | LAG1 HOMOLOG 2 (LONGEVITY ASSURANCE GENE1 HC	-4.018
JCVI_41816	1.935	moderately similar to (364)AT1G17110| Symbols: UBP15 | UBP15 (UBIQUITIN-SPECIFIC PROTEASE 15); ubiquitin-specific protease	
JCVI_33699	1.935	moderately similar to (214)AT2G23790| Symbols: | similar to unknown protein [Arabidopsis thaliana] (TAIR:AT4G36820.1); similar to u	
EE508770	1.935	no similarity	
JCVI_19947	1.935	weakly similar to (184)AT3G01140| Symbols: AtMYB106, MYB106 | MYB106 (myb domain protein 106); DNA binding / transcription f	
EE475265	1.934	no similarity	1.477
JCVI_33129	1.934	moderately similar to (223)AT2G36630| Symbols: | similar to unknown protein [Arabidopsis thaliana] (TAIR:AT2G25737.1); similar to u	
EE536932	1.933	very weakly similar to (85.5)AT3G17310| Symbols: | methyltransferase family protein | chr3:5909300-5912844 REVERSE [20161]	1.437
JCVI_41187	1.933	weakly similar to (126)AT5G10380| Symbols: | zinc finger (C3HC4-type RING finger) family protein | chr5:3267820-3268725 FORWAR	

JCVI_1880	1.933	moderately similar to (355)AT3G25230| Symbols: ROF1 | ROF1 (ROTAMASE FKBP 1) | chr3:9189494-9192412 FORWARDmoderately	
JCVI_25	1.933	moderately similar to (461)AT3G47470| Symbols: CAB4, LHCA4 | LHCA4 (Photosystem I light harvesting complex gene 4); chlorophyll	-1.671
JCVI_21598	1.933	highly similar to (588)AT1G13580| Symbols: LAG13 | LAG13 (LAG1 LONGEVITY ASSURANCE HOMOLOG 3) | chr1:4645003-4646	
EE470581	1.933	no similarity	
JCVI_37131	1.933	moderately similar to (236)AT3G46900| Symbols: COPT2 | COPT2 (Copper transporter 2); copper ion transmembrane transporter | chr3:1	
JCVI_30971	1.933	moderately similar to (223)AT5G60620| Symbols: | phospholipid/glycerol acyltransferase family protein | chr5:24384492-24386873 FOR	
EV096779	1.933	weakly similar to (186)AT5G03160| Symbols: | DNAJ heat shock N-terminal domain-containing protein | chr5:750285-752670 FORWAR	
EV035443	1.933	moderately similar to (206)AT4G16440| Symbols: | iron hydrogenase family protein | chr4:9269286-9271532 REVERSE [21441] 1 732 7	
JCVI_42395	1.933	moderately similar to (493)AT5G64020| Symbols: | similar to unknown protein [Arabidopsis thaliana] (TAIR:AT2G37720.1); similar to l	
JCVI_23190	1.933	weakly similar to (199)AT5G01530| Symbols: | chlorophyll A-B binding protein CP29 (LHCB4) | chr5:209083-210242 FORWARDweakl	
EV182537	1.933	weakly similar to (126)AT1G62430| Symbols: ATCDS1 | ATCDS1 (CDP-diacylglycerol synthase 1); phosphatidate cytidylyltransferase | c	
JCVI_26678	1.932	moderately similar to (301)AT5G17450| Symbols: | heavy-metal-associated domain-containing protein / copper chaperone (CCH)-related	
JCVI_16397	1.932	moderately similar to (491)AT4G32180| Symbols: ATPANK2 | ATPANK2 (PANTOTHENATE KINASE 2) | chr4:15538346-15543721 R	
JCVI_6948	1.932	moderately similar to (482)AT3G06580| Symbols: GALK, GAL1 | GAL1 (GALACTOSE KINASE 1); ATP binding / galactokinase | chr3:	
JCVI_31753	1.932	moderately similar to (210)AT3G48780| Symbols: | serine C-palmitoyltransferase, putative | chr3:18100331-18103261 FORWARD no ori	
JCVI_9679	1.932	moderately similar to (365)AT1G18490| Symbols: | similar to unknown protein [Arabidopsis thaliana] (TAIR:AT5G39890.1); similar to P	
JCVI_35734	1.932	highly similar to (546)AT3G57050| Symbols: CBL | CBL (CYSTATHIONINE BETA-LYASE) | chr3:21122918-21125500 REVERSE no	
JCVI_4636	1.932	highly similar to (585)AT4G30310| Symbols: | ribitol kinase, putative | chr4:14831919-14835098 FORWARD no original description	
JCVI_9881	1.932	moderately similar to (474)AT1G13980| Symbols: VAN7, EMB30, GN | GN (GNOM) | chr1:4789584-4794394 FORWARD no original de	
RC_ES978423	1.931	no similarity	
EX066229	1.931	very weakly similar to (99.0)AT5G44790| Symbols: HMA7, RAN1 | RAN1 (RESPONSIVE-TO-ANTAGONIST1); ATPase, coupled to tra	
ES918671	1.931	weakly similar to (121)AT3G24730| Symbols: | catalytic | chr3:9030159-9030901 REVERSE [15718]	
EE425434	1.931	weakly similar to (150)AT5G06550| Symbols: | Identical to F-box protein At5g06550 [Arabidopsis Thaliana] (GB:Q67XX3;GB:Q9FG15)	
AM396136	1.931	weakly similar to (177)AT4G23660| Symbols: ATPPT1 | ATPPT1 (ARABIDOPSIS THALIANA POLYPRENYLTRANSFERASE 1) | chr	-2.727
JCVI_25977	1.931	moderately similar to (357)AT3G28450| Symbols: | leucine-rich repeat transmembrane protein kinase, putative | chr3:10668596-1067041	
BQ704233	1.931	weakly similar to (123)AT5G13140| Symbols: | similar to unknown protein [Arabidopsis thaliana] (TAIR:AT3G26960.1); similar to unkn	
EV211102	1.931	no similarity	
JCVI_37132	1.931	no original description	
ES949537	1.930	moderately similar to (252)AT5G09580| Symbols: | similar to unknown protein [Arabidopsis thaliana] (TAIR:AT1G53345.1); similar to u	
JCVI_17584	1.930	moderately similar to (316)AT1G78915| Symbols: | binding | chr1:29672622-29675974 REVERSE no original description	
JCVI_30741	1.930	moderately similar to (427)AT4G16370| Symbols: OPT3, ATOPT3 | ATOPT3 (OLIGOPEPTIDE TRANSPORTER); oligopeptide transpo	
JCVI_10405	1.930	very weakly similar to (85.5)AT5G65870| Symbols: ATPSK5 | ATPSK5 (PHYTOSULFOKINE 5 PRECURSOR); growth factor | chr5:263	
EV188587	1.930	moderately similar to (203)AT5G61410| Symbols: EMB2728, RPE | RPE (EMBRYO DEFECTIVE 2728); ribulose-phosphate 3-epimeras	
EE567724	1.930	no similarity	
JCVI_3295	1.930	weakly similar to (185)AT3G22600| Symbols: | protease inhibitor/seed storage/lipid transfer protein (LTP) family protein | chr3:8006718-	
CN829677	1.930	moderately similar to (313)AT5G66420| Symbols: | similar to tm-1^GCR26 protein [Solanum lycopersicum] (GB:BAF75725.1); similar to	
EV099224	1.930	weakly similar to (156)AT5G46800| Symbols: BOU | BOU (A BOUT DE SOUFFLE); binding | chr5:19006006-19007037 REVERSE [21	
JCVI_8795	1.929	moderately similar to (256)AT4G28300| Symbols: | hydroxyproline-rich glycoprotein family protein | chr4:14015420-14016829 FORWA	
JCVI_39317	1.929	no original description	
EV184029	1.929	moderately similar to (245)AT5G05340| Symbols: | peroxidase, putative | chr5:1579143-1580820 REVERSEmoderately similar to (243)	
JCVI_16734	1.929	moderately similar to (284)AT3G56170| Symbols: CAN | CAN (CA-2+ DEPENDENT NUCLEASE); nuclease | chr3:20853593-20855298	
AT001877	1.929	no similarity	
EE568769	1.929	weakly similar to (101)AT4G08300| Symbols: | nodulin MtN21 family protein | chr4:5245021-5248150 FORWARD [20153] 46 693 693	
JCVI_5813	1.928	weakly similar to (164)AT4G38090| Symbols: | similar to unnamed protein product [Vitis vinifera] (GB:CAO22676.1); similar to hypothe	
JCVI_9790	1.928	moderately similar to (241)AT4G02725| Symbols: | similar to unnamed protein product [Vitis vinifera] (GB:CAO22536.1) | chr4:1206055	-1.669
JCVI_16287	1.928	highly similar to (512)AT5G10200| Symbols: | binding | chr5:3202379-3204368 FORWARD no original description	
CD829469	1.928	weakly similar to (153)AT5G17120| Symbols: | similar to unknown protein [Arabidopsis thaliana] (TAIR:AT5G17150.1); contains InterP	1.334
EV208446	1.928	weakly similar to (184)AT1G04790| Symbols: | zinc finger (C3HC4-type RING finger) family protein | chr1:1345468-1348142 FORWAR	
JCVI_7713	1.928	moderately similar to (348)AT1G68470| Symbols: | exostosin family protein | chr1:25680208-25681930 REVERSEweakly similar to (18	
JCVI_1368	1.928	moderately similar to (408)AT3G25780| Symbols: AOC3 | AOC3 (ALLENE OXIDE CYCLASE 3) | chr3:9410599-9411646 FORWARD	
EL591687	1.928	very weakly similar to (88.2)AT3G04650| Symbols: | oxidoreductase | chr3:1262023-1264349 FORWARD [20863] 1 257 273	
JCVI_224	1.928	highly similar to (717)AT5G63570| Symbols: GSA1 | GSA1 (GLUTAMATE-1-SEMIALDEHYDE-2,1-AMINOMUTASE); glutamate-1-s	
JCVI_18317	1.927	highly similar to (587)AT1G52980| Symbols: | GTP-binding family protein | chr1:19741161-19743869 FORWARD no original descriptio	
JCVI_13226	1.927	highly similar to (665)AT4G21760| Symbols: BGLU47 | BGLU47 (Beta-glucosidase 47); hydrolase, hydrolyzing O-glycosyl compounds |	
JCVI_21930	1.927	moderately similar to (283)AT1G75410| Symbols: BLH3 | BLH3 | chr1:28303756-28305551 REVERSE no original description	1.815
JCVI_18371	1.927	weakly similar to (154)AT1G64840| Symbols: | F-box family protein | chr1:24097673-24098827 FORWARD no original description	
ES949698	1.926	moderately similar to (226)AT3G63270| Symbols: | similar to unknown protein [Arabidopsis thaliana] (TAIR:AT3G55350.1); similar to u	
JCVI_15170	1.926	moderately similar to (251)AT5G19760| Symbols: | dicarboxylate/tricarboxylate carrier (DTC) | chr5:6679593-6681847 REVERSE no ori	
EV010020	1.926	very weakly similar to (85.1)AT2G42690| Symbols: | lipase, putative | chr2:17783433-17784759 REVERSE [21433]	
JCVI_23740	1.926	moderately similar to (332)AT5G04870| Symbols: AK1, ATCPK1, CPK1 | CPK1 (calcium-dependent protein kinase isoform AK1); calmo	-2.122
JCVI_27116	1.926	highly similar to (808)AT5G25060| Symbols: | RNA recognition motif (RRM)-containing protein | chr5:8634222-8639987 REVERSE no	
JCVI_24802	1.926	highly similar to (530)AT5G57800| Symbols: FLP1, YRE, CER3, WAX2 | CER3/FLP1/WAX2/YRE (ECERIFERUM 3); catalytic | chr5:2	
EE418044	1.926	moderately similar to (224)AT3G13080| Symbols: EST2, MRP3, ATMRP3 | ATMRP3 (Arabidopsis thaliana multidrug resistance-associa	
EV069201	1.925	moderately similar to (248)AT4G25840| Symbols: GPP1 | GPP1 (GLYCEROL-3-PHOSPHATASE 1); hydrolase | chr4:13139035-131407	
JCVI_10501	1.925	highly similar to (903)AT2G42790| Symbols: CSY3 | CSY3 (CITRATE SYNTHASE 3); citrate (SI)-synthase | chr2:17810209-17813068	
ES981578	1.925	weakly similar to (117)AT5G01470| Symbols: | similar to unnamed protein product [Vitis vinifera] (GB:CAO15817.1); contains domain S	-1.944
JCVI_1796	1.925	moderately similar to (450)AT3G04240| Symbols: SEC | SEC (SECRET AGENT); transferase, transferring glycosyl groups | chr3:111419	
EV148911	1.925	weakly similar to (155)AT2G41040| Symbols: | methyltransferase-related | chr2:17128577-17130142 FORWARD [21483]	
JCVI_39566	1.925	moderately similar to (314)AT1G74310| Symbols: HSP101, HOT1, ATHSP101 | ATHSP101 (HEAT SHOCK PROTEIN 101); ATP bindi	
EV211590	1.925	moderately similar to (340)AT1G03440| Symbols: | leucine-rich repeat family protein | chr1:852680-853873 FORWARDvery weakly simi	
EV121289	1.924	moderately similar to (436)AT2G32810| Symbols: BGAL9 | BGAL9 (BETA GALACTOSIDASE 9); beta-galactosidase | chr2:13926487-1	
CV546081	1.924	no similarity	
AM394302	1.924	moderately similar to (283)AT5G15550| Symbols: | transducin family protein / WD-40 repeat family protein | chr5:5059318-5062006 RE	
JCVI_18517	1.924	moderately similar to (374)AT1G24440| Symbols: | protein binding / zinc ion binding | chr1:8662328-8663765 FORWARD no original de	
DY000192	1.923	moderately similar to (285)AT5G46740| Symbols: UBP21 | UBP21 (UBIQUITIN-SPECIFIC PROTEASE 21); ubiquitin-specific protease	
JCVI_27216	1.923	moderately similar to (309)AT2G26150| Symbols: HSFA2, ATHSFA2 | ATHSFA2 (Arabidopsis thaliana heat shock transcription factor A	
CV432240	1.923	weakly similar to (119)AT1G61580| Symbols: RPL3B, ARP2 | ARP2/RPL3B (ARABIDOPSIS RIBOSOMAL PROTEIN 2); structural co	
CX280677	1.923	weakly similar to (169)AT4G21120| Symbols: CAT1 | AAT1 (CATIONIC AMINO ACID TRANSPORTER 1); cationic amino acid transm	
JCVI_15113	1.923	moderately similar to (214)AT3G26510| Symbols: | octicosapeptide/Phox/Bem1p (PB1) domain-containing protein | chr3:9713123-97137	
JCVI_2507	1.923	weakly similar to (151)AT4G37900| Symbols: | glycine-rich protein | chr4:17821731-17824439 REVERSE no original description	
EE462436	1.923	weakly similar to (194)AT2G14110| Symbols: | similar to unnamed protein product [Vitis vinifera] (GB:CAO43153.1); contains InterPro	

JCVI_3621	1.923	highly similar to (593)AT4G26530| Symbols: | fructose-bisphosphate aldolase, putative | chr4:13391573-13392944 FORWARDhighly sim	
JCVI_26838	1.923	moderately similar to (368)AT3G60260| Symbols: | phagocytosis and cell motility protein ELMO1-related | chr3:22285278-22287814 FO	
JCVI_5556	1.922	moderately similar to (208)AT5G46620| Symbols: | similar to unknown protein [Arabidopsis thaliana] (TAIR:AT4G17960.1); similar to u	
JCVI_27772	1.922	no original description	
JCVI_32569	1.922	moderately similar to (248)AT1G07640| Symbols: OBP2 | OBP2 (OBF BINDING PROTEIN 2); DNA binding | chr1:2354704-2356009 R	
EX138466	1.922	weakly similar to (102)AT5G62390| Symbols: ATBAG7 | ATBAG7 (ARABIDOPSIS THALIANA BCL-2-ASSOCIATED ATHANOGEN	
EX135893	1.922	moderately similar to (391)AT3G54220| Symbols: SGR1, SCR | SCR (SCARECROW); transcription factor | chr3:20081528-20083603 FO	1.456
EV027133	1.922	moderately similar to (249)AT3G42860| Symbols: | zinc knuckle (CCHC-type) family protein | chr3:14957446-14959085 REVERSE [214	
JCVI_5122	1.921	highly similar to (524)AT1G80780| Symbols: | CCR4-NOT transcription complex protein, putative | chr1:30363409-30364233 FORWAR	
EX035848	1.921	very weakly similar to (82.4)AT4G17560| Symbols: | ribosomal protein L19 family protein | chr4:9780356-9781765 FORWARD [21811]	
JCVI_146	1.921	moderately similar to (444)AT5G51550| Symbols: | phosphate-responsive 1 family protein | chr5:20957019-20958032 REVERSE no origi	
AM387990	1.921	no similarity	
JCVI_27768	1.921	no original description	
JCVI_29310	1.921	moderately similar to (213)AT3G03000| Symbols: | calmodulin, putative | chr3:677395-677892 FORWARDvery weakly similar to (93.2)C	
JCVI_722	1.921	highly similar to (622)AT5G04590| Symbols: SIR | SIR (sulfite reductase); sulfite reductase (ferredoxin) | chr5:1319405-1322299 FORWA	
ES981408	1.921	no similarity	
JCVI_1133	1.921	moderately similar to (326)AT5G36230| Symbols: | eIF4-gamma/eIF5/eIF2-epsilon domain-containing protein | chr5:14290749-14294003	-5.337
EV168947	1.921	moderately similar to (225)AT5G14460| Symbols: | pseudouridylate synthase TruB family protein | chr5:4660242-4662546 REVERSE [2	
JCVI_23444	1.920	moderately similar to (290)AT1G29160| Symbols: | Dof-type zinc finger domain-containing protein | chr1:10183783-10184310 REVERSI	
EE546467	1.920	no similarity	
EL588707	1.920	no similarity	
JCVI_269	1.920	moderately similar to (395)AT5G47650| Symbols: ATNUDT2 | ATNUDT2 (Arabidopsis thaliana Nudix hydrolase homolog 2); ADP-ribos	
EE557198	1.920	no similarity	
ES919471	1.920	weakly similar to (192)AT4G33040| Symbols: | glutaredoxin family protein | chr4:15940782-15941216 REVERSE [15718]	
EE531517	1.920	weakly similar to (165)AT1G14340| Symbols: | RNA recognition motif (RRM)-containing protein | chr1:4897704-4898771 FORWARD [2	
JCVI_19990	1.920	moderately similar to (233)AT2G24520| Symbols: AHA5 | AHA5 (ARABIDOPSIS H(+)-ATPASE 5); ATPase | chr2:10422602-10426810	
JCVI_30148	1.919	moderately similar to (240)AT3G58210| Symbols: | meprin and TRAF homology domain-containing protein / MATH domain-containing p	
JCVI_14323	1.919	moderately similar to (296)AT1G23200| Symbols: | pectinesterase family protein | chr1:8227225-8229389 FORWARD no original descrip	
JCVI_18312	1.919	no original description	
JCVI_28036	1.919	moderately similar to (232)AT1G68440| Symbols: | similar to unknown protein [Arabidopsis thaliana] (TAIR:AT1G25400.1); similar to u	
JCVI_11124	1.919	weakly similar to (138)AT4G17950| Symbols: | DNA-binding family protein | chr4:9967307-9969019 REVERSE no original description	
EE531664	1.919	weakly similar to (124)AT2G16405| Symbols: | transducin family protein / WD-40 repeat family protein | chr2:7112997-7115869 REVER	
EV215971	1.919	weakly similar to (182)AT1G70560| Symbols: | alliinase C-terminal domain-containing protein | chr1:26608557-26610982 FORWARD [2	
CD812804	1.919	moderately similar to (205)AT3G01970| Symbols: ATWRKY45, WRKY45 | WRKY45 (WRKY DNA-binding protein 45); transcription fa	
JCVI_34156	1.919	moderately similar to (292)AT1G51950| Symbols: IAA18 | IAA18 (indoleacetic acid-induced protein 18); transcription factor | chr1:19309	
JCVI_27820	1.918	moderately similar to (377)AT2G01350| Symbols: QPT | QPT (QUINOLINATE PHOSHORIBOSYLTRANSFERASE) | chr2:165331-167	1.080
JCVI_10996	1.918	highly similar to (557)AT3G14420| Symbols: | (S)-2-hydroxy-acid oxidase, peroxisomal, putative / glycolate oxidase, putative / short chai	
JCVI_37608	1.918	weakly similar to (144)AT5G42940| Symbols: | zinc finger (C3HC4-type RING finger) family protein | chr5:17233877-17236399 REVER	
JCVI_15081	1.918	highly similar to (685)AT3G48530| Symbols: KING1 | KING1 (SNF1-RELATED PROTEIN KINASE REGULATORY SUBUNIT GAMI	
JCVI_42437	1.918	moderately similar to (282)AT4G13350| Symbols: | human Rev interacting-like protein-related / hRIP protein-related | chr4:7770166-777	
JCVI_37191	1.918	weakly similar to (171)AT1G32660| Symbols: | F-box family protein | chr1:11811020-11812360 FORWARD no original description	
JCVI_15602	1.917	moderately similar to (347)AT3G56110| Symbols: | prenylated rab acceptor (PRA1) family protein | chr3:20833207-20833836 REVERSE	
EE560606	1.917	no similarity	
JCVI_10635	1.917	moderately similar to (373)AT4G32150| Symbols: VAMP711, ATVAMP711, VAMP7C | VAMP7C (VESICLE-ASSOCIATED MEMBR	
JCVI_26489	1.917	moderately similar to (477)AT1G20630| Symbols: CAT1 | CAT1 (CATALASE 1); catalase | chr1:7146802-7149599 FORWARDmoderate	
EV149017	1.917	no similarity	
EV004616	1.917	weakly similar to (150)AT5G03700| Symbols: | PAN domain-containing protein | chr5:965873-967321 REVERSE [21427]	
EV036610	1.917	no similarity	
EE429679	1.917	moderately similar to (322)AT5G16810| Symbols: | ATP binding / protein kinase | chr5:5526863-5529881 REVERSE [20136]	
EX085982	1.917	weakly similar to (122)AT2G47490| Symbols: | mitochondrial substrate carrier family protein | chr2:19494619-19496381 FORWARD [21	
JCVI_35040	1.917	weakly similar to (132)AT4G33467| Symbols: | unknown protein | chr4:16101694-16102087 REVERSE no original description	
EX129417	1.917	moderately similar to (349)AT3G60120| Symbols: | glycosyl hydrolase family 1 protein | chr3:22217322-22219927 FORWARDmoderatel	-1.948
EE440254	1.916	weakly similar to (169)AT2G14860| Symbols: | peroxisomal membrane protein 22 kDa, putative | chr2:6394902-6396645 REVERSE [201	
JCVI_20815	1.916	moderately similar to (333)AT5G22790| Symbols: RER1 | RER1 (RETICULATA-RELATED 1) | chr5:7599398-7601576 REVERSE no o	
JCVI_36958	1.916	no original description	
ES905135	1.915	moderately similar to (429)AT3G18190| Symbols: | chaperonin, putative | chr3:6232232-6233842 FORWARDweakly similar to (191)TC	
JCVI_3026	1.915	moderately similar to (347)AT3G12740| Symbols: ALIS1 | LEM3 (ligand-effect modulator 3) family protein / CDC50 family protein | chr3	
JCVI_25784	1.915	moderately similar to (343)AT1G19880| Symbols: | regulator of chromosome condensation (RCC1) family protein | chr1:6900639-690380	
EX090845	1.915	moderately similar to (375)AT3G11470| Symbols: | 4'-phosphopantetheinyl transferase family protein | chr3:3610267-3611794 REVERSE	
EV042890	1.915	weakly similar to (138)AT3G47850| Symbols: | similar to hypothetical protein [Vitis vinifera] (GB:CAN75379.1) | chr3:17665241-17666	
JCVI_14395	1.915	moderately similar to (384)AT5G19450| Symbols: CPK8, CDPK19 | CDPK19 (CALCIUM-DEPENDENT PROTEIN KINASE 19); calmo	
JCVI_6496	1.915	weakly similar to (114)AT2G19270| Symbols: | similar to unnamed protein product [Vitis vinifera] (GB:CAO69581.1) | chr2:8367431-83	
JCVI_7337	1.914	moderately similar to (444)AT5G11330| Symbols: | monooxygenase family protein | chr5:3617343-3618862 REVERSE no original descri	
JCVI_1273	1.914	moderately similar to (402)AT2G37990| Symbols: | ribosome biogenesis regulatory protein (RRS1) family protein | chr2:15907791-15910	
EV047932	1.914	moderately similar to (253)AT5G47840| Symbols: AMK2 | AMK2 (ADENOSINE MONOPHOSPHATE KINASE); adenylate kinase | chr	
JCVI_18342	1.914	highly similar to (964)AT1G53510| Symbols: ATMPK18 | ATMPK18 (ARABIDOPSIS THALIANA MAP KINASE 18); MAP kinase | ch	-1.022
EV177936	1.913	weakly similar to (161)AT4G16550| Symbols: | heat shock protein-related | chr4:9318464-9324276 REVERSE [21487] 142 919 919	
ES994446	1.913	weakly similar to (164)AT5G52390| Symbols: | photoassimilate-responsive protein, putative | chr5:21281507-21282399 REVERSE [2142	
EX111205	1.913	moderately similar to (358)AT1G13770| Symbols: | similar to unknown protein [Arabidopsis thaliana] (TAIR:AT3G45890.1); similar to u	
JCVI_29345	1.913	weakly similar to (130)AT3G03341| Symbols: | similar to Os01g0644200 [Oryza sativa (japonica cultivar-group)] (GB:NP_001043697.1)	-1.298
JCVI_28329	1.913	moderately similar to (204)AT4G37510| Symbols: | ribonuclease III family protein | chr4:17626253-17628848 REVERSE no original desc	
JCVI_27957	1.913	no original description	
JCVI_28750	1.913	no original description	
CN734193	1.913	weakly similar to (158)AT5G20840| Symbols: | phosphoinositide phosphatase family protein | chr5:7061640-7068574 REVERSE [15714]	
CD823099	1.912	weakly similar to (187)AT5G45720| Symbols: | ATP binding / DNA binding / DNA-directed DNA polymerase/ nucleoside-triphosphatase	
JCVI_31641	1.912	moderately similar to (251)AT1G53110| Symbols: | chorismate mutase | chr1:19794102-19795684 FORWARD no original description	
JCVI_11566	1.912	moderately similar to (220)AT5G67520| Symbols: | adenylylsulfate kinase, putative | chr5:26956568-26958068 FORWARDweakly simila	
EV130077	1.912	no similarity	
EV209425	1.911	weakly similar to (133)AT3G07880| Symbols: | Rho GDP-dissociation inhibitor family protein | chr3:2514181-2515550 FORWARD [214	
EV152911	1.911	moderately similar to (417)AT2G22125| Symbols: | binding | chr2:9413873-9421303 FORWARD [21484] 105 894 894	
CV973848	1.911	no similarity	

JCVI_7378	1.911	highly similar to (682)AT5G66120| Symbols: | 3-dehydroquinate synthase, putative | chr5:26448742-26450875 REVERSE no original des	-4.816
JCVI_13400	1.911	moderately similar to (350)AT3G02820| Symbols: | zinc knuckle (CCHC-type) family protein | chr3:611580-613301 FORWARD no origi	
JCVI_18749	1.911	highly similar to (723)AT2G18850| Symbols: | similar to SET domain-containing protein [Arabidopsis thaliana] (TAIR:AT3G07670.1); si	
JCVI_26107	1.911	moderately similar to (336)AT1G18720| Symbols: | similar to unknown protein [Arabidopsis thaliana] (TAIR:AT1G74440.1); similar to h	
EV089401	1.911	very weakly similar to (88.2)AT4G02610| Symbols: | tryptophan synthase, alpha subunit, putative | chr4:1147662-1149217 FORWARD [2	
DY005992	1.910	no similarity	
JCVI_24776	1.910	moderately similar to (218)AT5G43730| Symbols: | disease resistance protein (CC-NBS-LRR class), putative | chr5:17577494-17580040 I	
JCVI_32164	1.910	weakly similar to (199)AT5G57060| Symbols: | similar to unknown protein [Arabidopsis thaliana] (TAIR:AT4G26060.1); similar to unkn	-2.289
JCVI_18126	1.910	moderately similar to (233)AT4G32350| Symbols: | similar to unknown protein [Arabidopsis thaliana] (TAIR:AT1G79910.1); similar to u	
JCVI_8491	1.910	no original description	
RC_EX047982	1.909	no similarity	-1.612
JCVI_18265	1.909	moderately similar to (229)AT5G43140| Symbols: | peroxisomal membrane 22 kDa family protein | chr5:17338798-17340452 FORWARD	3.292
JCVI_22096	1.909	highly similar to (892)AT1G74800| Symbols: | galactosyltransferase family protein | chr1:28105882-28108654 REVERSE no original desc	
JCVI_11393	1.909	moderately similar to (494)AT3G21760| Symbols: | UDP-glucoronosyl/UDP-glucosyl transferase family protein | chr3:7667106-7668563	
JCVI_37183	1.909	weakly similar to (182)AT3G26000| Symbols: | F-box family protein | chr3:9508279-9509779 REVERSE no original description	
ES953055	1.909	no similarity	-1.353
JCVI_10294	1.909	moderately similar to (420)AT5G48300| Symbols: APS1, ADG1 | ADG1 (ADP GLUCOSE PYROPHOSPHORYLASE SMALL SUBUNIT	
JCVI_38244	1.909	no original description	
JCVI_11540	1.908	moderately similar to (347)AT1G70670| Symbols: | caleosin-related family protein | chr1:26648493-26649633 FORWARD no original des	
EV068464	1.908	weakly similar to (116)AT5G19473| Symbols: | similar to unknown protein [Arabidopsis thaliana] (TAIR:AT5G09960.1); similar to hypot	
JCVI_4951	1.908	highly similar to (559)AT4G19410| Symbols: | pectinacetylesterase, putative | chr4:10582199-10584777 REVERSE no original descriptio	
JCVI_34449	1.908	moderately similar to (223)AT1G19970| Symbols: | ER lumen protein retaining receptor family protein | chr1:6931185-6932588 REVERS	
DN961805	1.908	very weakly similar to (85.5)AT1G08450| Symbols: CRT3 | CRT3 (CALRETICULIN 3); calcium ion binding | chr1:2668005-2671797 REV	
JCVI_35602	1.908	weakly similar to (161)AT1G34360| Symbols: | translation initiation factor 3 (IF-3) family protein | chr1:12542964-12546003 FORWARD	-1.458
EV226763	1.907	weakly similar to (189)AT4G31410| Symbols: | similar to unknown protein [Arabidopsis thaliana] (TAIR:AT3G24740.2); similar to unkn	
JCVI_1610	1.907	moderately similar to (464)AT4G18710| Symbols: DWF12, UCU1, BIN2 | BIN2 (BRASSINOSTEROID-INSENSITIVE 2); kinase | chr4:	
JCVI_19857	1.907	highly similar to (720)AT2G38280| Symbols: ATAMPD, FAC1 | FAC1 (EMBRYONIC FACTOR1); AMP deaminase | chr2:16040845-16	
DY029554	1.907	no similarity	
JCVI_19103	1.907	moderately similar to (315)AT5G49360| Symbols: ATBXL1, BXL1 | BXL1 (BETA-XYLOSIDASE 1); hydrolase, hydrolyzing O-glycosyl	
JCVI_26590	1.907	moderately similar to (416)AT1G49710| Symbols: ATFUT12, FUCTB, FUCT2, FUT12 | FUT12 (fucosyltransferase 12); fucosyltransferas	
JCVI_17417	1.907	moderately similar to (216)AT2G21140| Symbols: ATPRP2 | ATPRP2 (PROLINE-RICH PROTEIN 2) | chr2:9067949-9069116 REVERS	
DY019211	1.907	moderately similar to (210)AT1G50380| Symbols: | prolyl oligopeptidase family protein | chr1:18666148-18669853 FORWARD [18966]	
JCVI_3283	1.907	moderately similar to (294)AT1G30230| Symbols: | elongation factor 1-beta / EF-1-beta | chr1:10639270-10640585 FORWARDmoderate	
EE392271	1.906	moderately similar to (242)AT3G26618| Symbols: ERF1-3 | ERF1-3 (EUKARYOTIC RELEASE FACTOR 1-3); translation release factor	
AM386634	1.906	weakly similar to (186)AT5G63870| Symbols: PP7 | PP7 (protein phosphatase 7); protein serine/threonine phosphatase | chr5:25578562-2	
EH419534	1.906	moderately similar to (330)AT2G40930| Symbols: ATUBP5, PDE323, UBP5 | UBP5 (UBIQUITIN-SPECIFIC PROTEASE 5); ubiquitin-	-1.564
JCVI_33347	1.906	moderately similar to (320)AT1G26120| Symbols: | esterase-related | chr1:9028643-9031389 REVERSE no original description	
JCVI_35908	1.906	no original description	
EX040649	1.906	no similarity	
JCVI_23790	1.906	very weakly similar to (82.8)AT1G14870| Symbols: | Identical to Uncharacterized protein At1g14870 [Arabidopsis Thaliana] (GB:Q9LQU	
EX036618	1.905	weakly similar to (112)AT4G30830| Symbols: | similar to unknown protein [Arabidopsis thaliana] (TAIR:AT2G24140.1); similar to unna	
JCVI_37364	1.905	highly similar to (689)AT5G18670| Symbols: BAM9, BMY3 | BMY3 (BETA-AMYLASE 9); beta-amylase | chr5:6226140-6228001 FOR	
JCVI_20883	1.905	moderately similar to (234)AT1G29400| Symbols: AML5 | AML5 (ARABIDOPSIS MEI2-LIKE PROTEIN 5); RNA binding | chr1:10290	
JCVI_13387	1.905	moderately similar to (349)AT1G73010| Symbols: | phosphoric monoester hydrolase | chr1:27468441-27469841 REVERSE no original de	
JCVI_35291	1.904	no original description	
EE417055	1.904	no similarity	
ES928318	1.904	no similarity	
EV109859	1.904	weakly similar to (127)AT2G15970| Symbols: WCOR413, WCOR413-LIKE, ATCOR413-PM1, FL3-5A3, COR413-PM1 | COR413-PM1	
JCVI_9361	1.904	moderately similar to (452)AT5G01250| Symbols: | alpha 1,4-glycosyltransferase family protein / glycosyltransferase sugar-binding DXD	
JCVI_6282	1.904	moderately similar to (432)AT2G39210| Symbols: | nodulin family protein | chr2:16373365-16375309 REVERSE no original description	
EX040600	1.904	weakly similar to (144)AT3G03790| Symbols: | ankyrin repeat family protein / regulator of chromosome condensation (RCC1) family prot	
DQ023594	1.903	moderately similar to (226)AT5G08280| Symbols: HEMC | HEMC (HYDROXYMETHYLBILANE SYNTHASE); hydroxymethylbilane s	-1.509
JCVI_30612	1.903	highly similar to (858)AT3G14630| Symbols: CYP72A9 | CYP72A9 (cytochrome P450, family 72, subfamily A, polypeptide 9); oxygen bi	
EV016336	1.903	no similarity	
AM059003	1.902	weakly similar to (182)AT1G12890| Symbols: | AP2 domain-containing transcription factor, putative | chr1:4391732-4392391 FORWARI	
CX194462	1.902	no similarity	
JCVI_7024	1.902	moderately similar to (329)AT1G65410| Symbols: TGD3, ATNAP11 | ATNAP11 (ARABIDOPSIS THALIANA NON-INTRINSIC ABC	
EE502262	1.902	moderately similar to (309)AT4G34670| Symbols: | 40S ribosomal protein S3A (RPS3aB) | chr4:16548729-16550227 FORWARDmodera	
EV220806	1.902	no similarity	
JCVI_3460	1.902	moderately similar to (240)AT5G17900| Symbols: | similar to microfibrillar-associated protein-related [Arabidopsis thaliana] (TAIR:AT4	
JCVI_22084	1.902	highly similar to (681)AT5G60360| Symbols: AALP | AALP (ARABIDOPSIS ALEURAIN-LIKE PROTEASE) | chr5:24297270-2429938	
JCVI_4455	1.902	moderately similar to (204)AT3G56440| Symbols: AtATG18d | AtATG18d (Arabidopsis thaliana homolog of yeast autophagy 18 (ATG18	
AM058621	1.901	no similarity	
JCVI_1612	1.901	highly similar to (637)AT5G54830| Symbols: | DOMON domain-containing protein / dopamine beta-monooxygenase N-terminal domain-	
JCVI_23024	1.901	no original description	1.379
EV086637	1.901	no similarity	
JCVI_11888	1.901	moderately similar to (455)AT5G18670| Symbols: BAM9, BMY3 | BMY3 (BETA-AMYLASE 9); beta-amylase | chr5:6226140-6228001	
JCVI_21528	1.900	moderately similar to (317)AT1G10570| Symbols: | Ulp1 protease family protein | chr1:3487640-3491103 FORWARD no original descrip	
JCVI_39505	1.900	weakly similar to (166)AT1G14250| Symbols: | nucleoside phosphatase family protein / GDA1/CD39 family protein | chr1:4868670-4871	
AM395446	1.900	weakly similar to (123)AT3G07050| Symbols: | GTP-binding family protein | chr3:2229608-2232285 REVERSE [20346] 26 556 556	
EX043605	1.900	moderately similar to (397)AT2G44280| Symbols: | similar to lactose permease-related [Arabidopsis thaliana] (TAIR:AT3G60070.1); sim	
JCVI_32791	1.900	moderately similar to (275)AT1G55480| Symbols: | binding / protein binding | chr1:20717488-20719017 FORWARD no original descripti	
JCVI_5604	1.900	moderately similar to (385)AT1G78590| Symbols: NADK3, ATNADK-3 | ATNADK-3/NADK3 (NAD(H) kinase 3); NAD+ kinase/ NADI	
JCVI_2916	1.900	weakly similar to (151)AT3G11510| Symbols: | 40S ribosomal protein S14 (RPS14B) | chr3:3623763-3624872 REVERSEweakly similar t	
EE451520	1.900	weakly similar to (133)AT1G20575| Symbols: | dolichyl-phosphate beta-D-mannosyltransferase, putative / dolichol-phosphate mannosyltr	-1.702
EV007808	1.900	no similarity	
RC_JCVI_8658	1.900	no original description	
JCVI_22047	1.899	weakly similar to (119)AT5G51120| Symbols: PABN1, ATPABN1 | ATPABN1/PABN1 (polyadenylate-binding protein 1); RNA binding /	
JCVI_22620	1.899	moderately similar to (451)AT2G14750| Symbols: AKN1, ATAKN1, APK | APK (APS KINASE); ATP binding / kinase/ transferase, trans	-1.551
EE562804	1.899	no similarity	1.768
ES961790	1.899	no similarity	

EV090829	1.899	no similarity	
JCVI_6863	1.899	weakly similar to (190)AT3G22490| Symbols: | late embryogenesis abundant protein, putative / LEA protein, putative | chr3:7969792-797	-2.619
EE546744	1.899	weakly similar to (174)AT3G12170| Symbols: | DNAJ heat shock N-terminal domain-containing protein | chr3:3881028-3882662 FORWA	
JCVI_18961	1.899	no original description	
AM395607	1.899	moderately similar to (230)AT5G19020| Symbols: | pentatricopeptide (PPR) repeat-containing protein | chr5:6352773-6357085 REVERSI	
EV012960	1.898	no similarity	
ES987162	1.898	moderately similar to (249)AT1G75660| Symbols: XRN3 | XRN3 (5'-3' exoribonuclease 3); 5'-3' exoribonuclease | chr1:28411950-284184	
JCVI_13085	1.898	moderately similar to (381)AT3G58830| Symbols: | haloacid dehalogenase (HAD) superfamily protein | chr3:21766149-21767614 REVEI	-3.120
JCVI_12462	1.898	moderately similar to (306)AT5G66730| Symbols: | zinc finger (C2H2 type) family protein | chr5:26659140-26661109 REVERSE no orig	
JCVI_4333	1.898	moderately similar to (330)AT4G27585| Symbols: | band 7 family protein | chr4:13766990-13769838 REVERSE no original description	-1.413
JCVI_7372	1.898	weakly similar to (196)AT1G21070| Symbols: | transporter-related | chr1:7376137-7377799 REVERSE no original description	
EV164207	1.897	no similarity	1.491
JCVI_17809	1.897	moderately similar to (342)AT4G32175| Symbols: | RNA binding | chr4:15535561-15537543 FORWARD no original description	
JCVI_37207	1.897	weakly similar to (144)AT5G19440| Symbols: | cinnamyl-alcohol dehydrogenase, putative (CAD) | chr5:6556495-6558125 FORWARDve	
JCVI_20880	1.897	weakly similar to (107)AT5G53200| Symbols: TRY | TRY (TRIPTYCHON); DNA binding / transcription factor | chr5:21600139-2160118	
JCVI_15091	1.897	moderately similar to (361)AT3G14360| Symbols: | lipase class 3 family protein | chr3:4791603-4793541 FORWARD no original descript	
ES966824	1.897	no similarity	
RC_EV012166	1.896	no similarity	
JCVI_19884	1.896	moderately similar to (479)AT2G22360| Symbols: | DNAJ heat shock family protein | chr2:9505242-9507539 FORWARDvery weakly sin	-2.266
EV179245	1.896	very weakly similar to (100)AT2G15290| Symbols: ATTIC21, TIC21, CIA5, PIC1 | ATTIC21/CIA5/PIC1/TIC21 (CHLOROPLAST IMP(	
JCVI_4273	1.895	moderately similar to (270)AT5G06960| Symbols: TGA5, OBF5 | OBF5 (OCS-ELEMENT BINDING FACTOR 5); DNA binding / transcı	1.431
JCVI_19291	1.895	moderately similar to (284)AT2G47800| Symbols: EST3, ATMRP4 | ATMRP4 (Arabidopsis thaliana multidrug resistance-associated prot	
CO729362	1.895	no similarity	
EV023652	1.895	moderately similar to (322)AT3G12760| Symbols: | similar to unknown protein [Arabidopsis thaliana] (TAIR:AT1G15860.1); similar to S	
JCVI_7091	1.895	moderately similar to (369)AT3G53710| Symbols: AGD6 | AGD6; DNA binding | chr3:19914708-19916397 REVERSE no original descrip	
JCVI_30858	1.894	moderately similar to (331)AT1G15130| Symbols: | hydroxyproline-rich glycoprotein family protein | chr1:5206212-5209843 REVERSE r	
JCVI_34025	1.894	moderately similar to (377)AT5G48240| Symbols: | similar to unnamed protein product [Vitis vinifera] (GB:CAO41129.1); contains Inter	
AM391859	1.894	highly similar to (517)AT5G18190| Symbols: | protein kinase family protein | chr5:6010217-6013726 REVERSE [20118]	
EV034881	1.894	weakly similar to (150)AT1G78110| Symbols: | similar to unknown protein [Arabidopsis thaliana] (TAIR:AT1G22230.1); similar to unnai	
EE514766	1.894	moderately similar to (206)AT3G09150| Symbols: GUN3, HY2 | HY2 (ELONGATED HYPOCOTYL 2); phytochromobilin:ferredoxin ox	
JCVI_9851	1.894	moderately similar to (223)AT2G37870| Symbols: | protease inhibitor/seed storage/lipid transfer protein (LTP) family protein | chr2:1586(	
JCVI_18820	1.894	moderately similar to (352)AT1G34180| Symbols: ANAC016 | ANAC016 (Arabidopsis NAC domain containing protein 16) | chr1:124487	
JCVI_188	1.893	moderately similar to (392)AT1G05190| Symbols: EMB2394 | EMB2394 (EMBRYO DEFECTIVE 2394); structural constituent of riboso	2.124
JCVI_37228	1.893	very weakly similar to (82.8)AT3G60130| Symbols: | glycosyl hydrolase family 1 protein / beta-glucosidase, putative (YLS1) | chr3:22221	
EE503113	1.893	moderately similar to (205)AT5G55310| Symbols: TOP1, TOP1alpha | TOP1alpha (TOPOISOMERASE I) | chr5:22447615-22451816 RE	-2.856
JCVI_7705	1.893	no original description	
EV133121	1.893	no similarity	
JCVI_6359	1.892	moderately similar to (354)AT3G27390| Symbols: | Identical to Uncharacterized membrane protein At3g27390 [Arabidopsis Thaliana] (G	
JCVI_12303	1.892	weakly similar to (153)AT3G19895| Symbols: | similar to unknown protein [Arabidopsis thaliana] (TAIR:AT3G12940.1); similar to unnai	
JCVI_9718	1.892	weakly similar to (118)AT5G66290| Symbols: | similar to hypothetical protein [Thellungiella halophila] (GB:ABB45846.1) | chr5:264951	
ES952938	1.892	no similarity	
ES903847	1.892	moderately similar to (498)AT4G33760| Symbols: | tRNA synthetase class II (D, K and N) family protein | chr4:16189287-16193262 REV	
EX098099	1.891	no similarity	
JCVI_377	1.891	moderately similar to (403)AT5G06860| Symbols: PGIP1 | PGIP1 (POLYGALACTURONASE INHIBITING PROTEIN 1); protein bindi	
EV057729	1.891	moderately similar to (230)AT1G76510| Symbols: | ARID/BRIGHT DNA-binding domain-containing protein | chr1:28713821-28717392	
EV177300	1.891	no similarity	
JCVI_13612	1.891	weakly similar to (174)AT3G17800| Symbols: | mRNA level of the MEB5.2 gene (At3g17800) remains unchanged after cutting the inflor	-1.231
JCVI_8726	1.891	moderately similar to (321)AT1G01190| Symbols: CYP78A8 | CYP78A8 (cytochrome P450, family 78, subfamily A, polypeptide 8); oxyg	
JCVI_24807	1.891	moderately similar to (443)AT4G33945| Symbols: | armadillo/beta-catenin repeat family protein | chr4:16268124-16270519 FORWARD r	
JCVI_27055	1.890	moderately similar to (276)AT3G17540| Symbols: | F-box family protein | chr3:6002789-6003979 FORWARD no original description	
JCVI_8475	1.890	moderately similar to (234)AT2G20930| Symbols: | similar to unknown [Populus trichocarpa] (GB:ABK93191.1); contains InterPro doma	
JCVI_16045	1.890	weakly similar to (169)AT5G47880| Symbols: ERF1, ERF1-1 | ERF1-1 (EUKARYOTIC RELEASE FACTOR 1-1); translation release fac	
JCVI_12446	1.890	moderately similar to (269)AT2G31230| Symbols: ATERF15 | ATERF15 (ETHYLENE-RESPONSIVE ELEMENT BINDING FACTOR 1	
EE464302	1.890	moderately similar to (215)AT4G38940| Symbols: | kelch repeat-containing F-box family protein | chr4:18152842-18153954 FORWARD	
JCVI_5891	1.890	moderately similar to (328)AT1G63970| Symbols: MECPS, ISPF | ISPF (Homolog of E. coli ispF (isoprenoids F)); 2-C-methyl-D-erythrit	
JCVI_6396	1.890	highly similar to (557)AT3G26410| Symbols: | methyltransferase/ nucleic acid binding | chr3:9670745-9672510 REVERSE no original de	
JCVI_15729	1.889	moderately similar to (315)AT5G02230| Symbols: | haloacid dehalogenase-like hydrolase family protein | chr5:449130-450505 FORWAR	
EV101942	1.889	no similarity	
CN730231	1.889	very weakly similar to (80.5)AT1G52160| Symbols: | metallo-beta-lactamase family protein | chr1:19424580-19428088 REVERSE [15718	
JCVI_5504	1.889	moderately similar to (263)AT5G07220| Symbols: ATBAG3 | ATBAG3 (ARABIDOPSIS THALIANA BCL-2-ASSOCIATED ATHANO(	
JCVI_2010	1.889	moderately similar to (333)AT1G24180| Symbols: IAR4 | IAR4 (IAA-conjugate-resistant 4); pyruvate dehydrogenase (acetyl-transferring)	
AM386090	1.889	no similarity	
JCVI_14612	1.889	moderately similar to (262)AT3G61790| Symbols: | seven in absentia (SINA) family protein | chr3:22882949-22884518 REVERSEvery w	
JCVI_20732	1.889	moderately similar to (286)AT5G08280| Symbols: HEMC | HEMC (HYDROXYMETHYLBILANE SYNTHASE); hydroxymethylbilane s	-2.140
EE414208	1.888	no similarity	
JCVI_15777	1.888	moderately similar to (472)AT3G57330| Symbols: ACA11 | ACA11 (AUTOINHIBITED CA2+-ATPASE 11); calcium-transporting ATPa	
EV146350	1.888	no similarity	
ES898173	1.888	no similarity	
CX270294	1.888	no similarity	
CN727611	1.888	weakly similar to (182)AT5G05380| Symbols: | prenylated rab acceptor (PRA1) family protein | chr5:1592215-1592868 FORWARD [157	
JCVI_16719	1.888	moderately similar to (226)AT4G21090| Symbols: | adrenodoxin-like ferredoxin 1 | chr4:11256674-11258280 REVERSE no original desc	
JCVI_6234	1.888	moderately similar to (331)AT5G66730| Symbols: | zinc finger (C2H2 type) family protein | chr5:26659140-26661109 REVERSE no orig	
JCVI_41875	1.888	weakly similar to (163)AT5G18970| Symbols: | AWPM-19-like membrane family protein | chr5:6333716-6334541 REVERSE no original	
ES898066	1.888	no similarity	
AT001707	1.888	no similarity	
EE459992	1.887	weakly similar to (138)AT4G23600| Symbols: JR2, CORI3 | CORI3 (CORONATINE INDUCED 1, JASMONIC ACID RESPONSIVE 2);	
EX037619	1.887	no similarity	
JCVI_33349	1.887	weakly similar to (167)AT2G18245| Symbols: | similar to unknown protein [Arabidopsis thaliana] (TAIR:AT3G19970.1); similar to unnai	
JCVI_34864	1.887	moderately similar to (317)AT3G03720| Symbols: CAT4 | CAT4 (CATIONIC AMINO ACID TRANSPORTER 4); cationic amino acid tra	
JCVI_14864	1.886	weakly similar to (166)AT1G04100| Symbols: IAA10 | IAA10 (indoleacetic acid-induced protein 10); transcription factor | chr1:1059808-1	
CN727467	1.886	no similarity	

JCVI_15958	1.886	moderately similar to (313)AT3G01440| Symbols: | oxygen evolving enhancer 3 (PsbQ) family protein | chr3:168485-169414 FORWARD	
JCVI_31087	1.886	highly similar to (833)AT5G41990| Symbols: ATWNK8, WNK8 | WNK8 (Arabidopsis WNK kinase 8); kinase | chr5:16812313-16814790	
JCVI_33310	1.885	weakly similar to (128)AT2G31470| Symbols: | F-box family protein | chr2:13414569-13415732 REVERSE no original description	
EV192915	1.885	weakly similar to (132)AT2G38000| Symbols: | chaperone protein dnaJ-related | chr2:15910393-15912354 REVERSE [21489] 105 814 81	1.872
JCVI_25942	1.885	weakly similar to (163)AT5G67390| Symbols: | similar to unknown protein [Arabidopsis thaliana] (TAIR:AT1G69360.1); similar to unnan	
EV039823	1.885	moderately similar to (364)AT1G66730| Symbols: | ATP dependent DNA ligase family protein | chr1:24888654-24895486 FORWARD [2	
JCVI_22814	1.885	moderately similar to (408)AT5G19450| Symbols: CPK8, CDPK19 | CDPK19 (CALCIUM-DEPENDENT PROTEIN KINASE 19); calmo	3.044
JCVI_15300	1.885	moderately similar to (399)AT2G19520| Symbols: ACG1, MSI4, NFC4, NFC04, ATMSI4, FVE | FVE | chr2:8463088-8466317 FORWAR	
EE466659	1.884	weakly similar to (137)AT5G47080| Symbols: CKB1 | CKB1 (casein kinase II beta chain 1); protein kinase CK2 regulator | chr5:1914245	
EV098304	1.884	no similarity	
EE484508	1.884	no similarity	-1.467
JCVI_30348	1.884	moderately similar to (492)AT1G28570| Symbols: | GDSL-motif lipase, putative | chr1:10041824-10044098 REVERSEweakly similar to (	
JCVI_29707	1.884	no original description	
EX028275	1.883	moderately similar to (429)AT5G01550| Symbols: | lectin protein kinase, putative | chr5:214516-216582 REVERSEweakly similar to (17	
EV047509	1.883	moderately similar to (213)AT4G18460| Symbols: | D-Tyr-tRNA(Tyr) deacylase family protein | chr4:10195774-10196690 REVERSE [2	
JCVI_18773	1.883	very weakly similar to (92.0)AT4G13990| Symbols: | exostosin family protein | chr4:8084390-8085955 FORWARD no original descriptior	
CV432851	1.883	weakly similar to (124)AT4G21700| Symbols: | similar to unknown protein [Arabidopsis thaliana] (TAIR:AT1G52780.1); similar to unnan	
EE558777	1.883	no similarity	
EH419546	1.883	no similarity	-1.362
EE533052	1.883	weakly similar to (120)AT3G58210| Symbols: | meprin and TRAF homology domain-containing protein / MATH domain-containing prote	
EX045518	1.883	moderately similar to (365)AT5G15470| Symbols: GAUT14 | GAUT14 (Galacturonosyltransferase 14); polygalacturonate 4-alpha-galactu	
JCVI_18668	1.883	moderately similar to (401)AT3G18830| Symbols: ATPLT5 | ATPLT5 (POLYOL TRANSPORTER 5); D-ribose transmembrane transport	
JCVI_21149	1.882	moderately similar to (402)AT5G64030| Symbols: | dehydration-responsive protein-related | chr5:25642191-25645483 FORWARD no ori	
ES929828	1.882	very weakly similar to (83.6)AT1G03290| Symbols: | similar to unknown protein [Arabidopsis thaliana] (TAIR:AT4G02880.1); similar to u	
JCVI_27282	1.882	weakly similar to (106)AT1G32070| Symbols: ATNSI | ATNSI (NUCLEAR SHUTTLE INTERACTING) | chr1:11534831-11536269 REV	
EV217091	1.882	no similarity	
EE470213	1.882	moderately similar to (207)AT3G26540| Symbols: | pentatricopeptide (PPR) repeat-containing protein | chr3:9745779-9747881 REVERSI	
EV147301	1.882	no similarity	
EE447305	1.882	no similarity	-2.087
JCVI_36476	1.882	highly similar to (525)AT1G78070| Symbols: | WD-40 repeat family protein | chr1:29360113-29363261 FORWARD no original descriptic	
EV152571	1.881	no similarity	
ES937933	1.881	moderately similar to (244)AT3G05670| Symbols: | PHD finger family protein | chr3:1653894-1657028 FORWARD [21390]	
CD840460	1.881	no similarity	
JCVI_3448	1.881	moderately similar to (428)AT5G19680| Symbols: | leucine-rich repeat family protein | chr5:6649665-6651566 FORWARD no original de	
CO749816	1.881	very weakly similar to (80.5)AT2G24280| Symbols: | serine carboxypeptidase S28 family protein | chr2:10341702-10344007 FORWARD [	
JCVI_36053	1.881	moderately similar to (391)AT5G57050| Symbols: ABI2 | ABI2 (ABA INSENSITIVE 2) | chr5:23104946-23106529 FORWARD no origir	
AM388862	1.880	very weakly similar to (85.5)AT3G04070| Symbols: ANAC047 | ANAC047 (Arabidopsis NAC domain containing protein 47) | chr3:10615	-1.710
JCVI_37829	1.880	moderately similar to (271)AT1G72450| Symbols: JAZ6, TIFY11B | JAZ6/TIFY11B (JASMONATE-ZIM-DOMAIN PROTEIN 6) | chr1:	
JCVI_8643	1.880	moderately similar to (258)AT3G18290| Symbols: EMB2454 | EMB2454 (EMBRYO DEFECTIVE 2454); protein binding / zinc ion bindi	1.271
BG543150	1.880	very weakly similar to (90.9)AT1G65290| Symbols: MTACP2 | MTACP2 (MITOCHONDRIAL ACYL CARRIER PROTEIN 2); acyl carri	
EX075777	1.880	moderately similar to (255)AT2G23390| Symbols: | similar to hypothetical protein OsI_015489 [Oryza sativa (indica cultivar-group)] (GB	
JCVI_1988	1.880	moderately similar to (297)AT1G29140| Symbols: | pollen Ole e 1 allergen and extensin family protein | chr1:10179015-10179797 FORW	-1.639
CV545128	1.880	weakly similar to (135)AT4G10380| Symbols: NIP5;1, NLM6, NLM8 | NIP5;1/NLM6/NLM8 (NOD26-like intrinsic protein 5;1); boron tr	
JCVI_26288	1.879	moderately similar to (406)AT1G25420| Symbols: | similar to unknown protein [Arabidopsis thaliana] (TAIR:AT1G34220.2); similar to u	
ES899742	1.879	no similarity	
JCVI_13260	1.879	moderately similar to (459)AT2G45790| Symbols: ATPMM | ATPMM; phosphomannomutase | chr2:18862950-18864827 FORWARD no	
JCVI_6439	1.879	moderately similar to (238)AT1G65020| Symbols: | similar to unnamed protein product [Vitis vinifera] (GB:CAO62149.1); contains Inter	-2.797
JCVI_5894	1.879	no original description	
JCVI_10360	1.879	weakly similar to (165)AT3G14200| Symbols: | DNAJ heat shock N-terminal domain-containing protein | chr3:4712888-4714368 REVER	
CD836635	1.878	no similarity	
JCVI_26046	1.878	no original description	
AM060585	1.878	weakly similar to (113)AT5G04990| Symbols: | sad1/unc-84 protein-related | chr5:1471699-1473771 REVERSE [17712]	
EV190421	1.878	moderately similar to (328)AT3G46610| Symbols: | pentatricopeptide (PPR) repeat-containing protein | chr3:17171209-17173206 REVER	
BQ791421	1.878	weakly similar to (129)AT3G60200| Symbols: | similar to unknown protein [Arabidopsis thaliana] (TAIR:AT2G44600.1); similar to unnan	
JCVI_24797	1.878	moderately similar to (211)AT3G09260| Symbols: PSR3.1, PYK10 | PYK10 (phosphate starvation-response 3.1); hydrolase, hydrolyzing (	
H07568	1.877	no similarity	
JCVI_17586	1.877	moderately similar to (227)AT1G34000| Symbols: OHP2 | OHP2 (ONE-HELIX PROTEIN 2) | chr1:12358131-12358882 REVERSE no or	
EV201815	1.877	moderately similar to (209)AT5G40520| Symbols: | similar to hypothetical protein [Vitis vinifera] (GB:CAN71839.1) | chr5:16248743-16	
CD843533	1.877	no similarity	
JCVI_29704	1.877	weakly similar to (117)AT5G15800| Symbols: AGL2, SEP1 | SEP1 (SEPALLATA1) | chr5:5151597-5153770 REVERSE no original descr	
JCVI_14897	1.877	moderately similar to (308)AT1G11755| Symbols: | transferase | chr1:3969987-3971488 REVERSE no original description	
EV126591	1.877	weakly similar to (191)AT4G11840| Symbols: PLDGAMMA3 | PLDGAMMA3 (phospholipase D gamma 3); phospholipase D | chr4:7122	
DY018697	1.877	weakly similar to (116)AT1G27930| Symbols: | similar to unknown protein [Arabidopsis thaliana] (TAIR:AT1G67330.1); similar to unkno	
JCVI_5764	1.876	moderately similar to (211)AT5G10140| Symbols: FLF, AGL25, FLC | FLC (FLOWERING LOCUS C) | chr5:3173878-3179340 REVERS	
JCVI_24887	1.876	weakly similar to (180)AT3G52560| Symbols: MMZ4, UEV1D, UEV1D-4 | MMZ4/UEV1D/UEV1D-4 (MMS ZWEI HOMOLOGE 4, UE	
JCVI_38477	1.876	highly similar to (689)AT3G55410| Symbols: | 2-oxoglutarate dehydrogenase E1 component, putative / oxoglutarate decarboxylase, putati	
JCVI_20434	1.876	weakly similar to (106)AT2G26865| Symbols: | Encodes a Plant thionin family protein | chr2:11462691-11462927 REVERSE no original	
JCVI_3220	1.876	moderately similar to (348)AT2G23070| Symbols: | casein kinase II alpha chain, putative | chr2:9831242-9833951 REVERSEmoderately s	
JCVI_24535	1.876	moderately similar to (318)AT3G15030| Symbols: MEE35, TCP4 | TCP4 (TCP FAMILY TRANSCRIPTION FACTOR 4); transcription f	
JCVI_12217	1.876	highly similar to (571)AT1G79440| Symbols: SSADH1, SSADH, ALDH5F1 | ALDH5F1 (SUCCINIC SEMIALDEHYDE DEHYDROGEN	
JCVI_41563	1.875	no original description	
JCVI_20205	1.875	moderately similar to (300)AT5G14960| Symbols: DEL2, E2L1, E2FD | DEL2/E2FD/E2L1 (DP-E2F-LIKE 2); DNA binding / transcriptio	
EV152704	1.875	moderately similar to (426)AT4G21530| Symbols: | nucleotide binding | chr4:11450755-11455874 FORWARD [21484] 53 865 865	-1.209
JCVI_38183	1.875	weakly similar to (176)AT5G50360| Symbols: | similar to unknown protein [Arabidopsis thaliana] (TAIR:AT5G63350.1); similar to unnan	
JCVI_2950	1.875	weakly similar to (112)AT1G20100| Symbols: | similar to unknown protein [Arabidopsis thaliana] (TAIR:AT1G75860.1) | chr1:6969400-(	
JCVI_1574	1.875	no original description	1.513
JCVI_42178	1.875	moderately similar to (268)AT1G69430| Symbols: | similar to unknown protein [Arabidopsis thaliana] (TAIR:AT1G26650.1); similar to u	
JCVI_27032	1.874	moderately similar to (386)AT1G52800| Symbols: | oxidoreductase, 2OG-Fe(II) oxygenase family protein | chr1:19667712-19669030 FOI	
EE565263	1.874	weakly similar to (138)AT5G16140| Symbols: | peptidyl-tRNA hydrolase family protein | chr5:5270311-5271520 REVERSE [20153] 21 3	
JCVI_21880	1.874	moderately similar to (263)AT1G60670| Symbols: | similar to unknown protein [Arabidopsis thaliana] (TAIR:AT1G10820.2); similar to u	
EV043361	1.874	weakly similar to (117)AT3G24580| Symbols: | F-box family protein | chr3:8969169-8970305 FORWARD [21442] 104 682 682	

EX028198	1.874	very weakly similar to (86.7)AT3G11280| Symbols: | myb family transcription factor | chr3:3533483-3534399 REVERSE [21810]	
EE434928	1.874	weakly similar to (174)AT1G76680| Symbols: OPR1 | OPR1 (12-oxophytodienoate reductase 1); 12-oxophytodienoate reductase | chr1:28	
JCVI_13567	1.874	moderately similar to (390)AT2G37130| Symbols: | peroxidase 21 (PER21) (P21) (PRXR5) | chr2:15605304-15606813 REVERSEweakly	
EE404711	1.874	no similarity	
EX035075	1.874	moderately similar to (219)AT2G29110| Symbols: GLR2.8, ATGLR2.8 | ATGLR2.8 (Arabidopsis thaliana glutamate receptor 2.8) | chr2:1	
JCVI_28406	1.874	weakly similar to (186)AT5G05220| Symbols: | similar to hypothetical protein [Vitis vinifera] (GB:CAN82940.1) | chr5:1550274-1550822	
JCVI_13727	1.874	highly similar to (656)AT1G57600| Symbols: | membrane bound O-acyl transferase (MBOAT) family protein | chr1:21334394-21338261	
JCVI_13851	1.873	moderately similar to (433)AT2G47250| Symbols: | RNA helicase, putative | chr2:19406993-19410051 REVERSE no original description	
CD818640	1.873	no similarity	
EV115318	1.873	moderately similar to (229)AT2G28650| Symbols: ATEXO70H8 | ATEXO70H8 (exocyst subunit EXO70 family protein H8); protein bind	
ES933778	1.873	moderately similar to (267)AT5G21140| Symbols: EMB1379 | EMB1379 (EMBRYO DEFECTIVE 1379) | chr5:7187419-7189524 REVE	
EV032648	1.873	weakly similar to (172)AT3G18295| Symbols: | similar to unknown protein [Arabidopsis thaliana] (TAIR:AT1G48770.1); similar to unnar	
EV136074	1.872	no similarity	
JCVI_5990	1.872	moderately similar to (298)AT1G50020| Symbols: | similar to unnamed protein product [Vitis vinifera] (GB:CAO49863.1) | chr1:1852381	
JCVI_25717	1.872	weakly similar to (133)AT1G50430| Symbols: PA, LE, ST7R, 7RED, DWF5 | DWF5 (DWARF 5) | chr1:18685843-18689223 REVERSE r	
JCVI_10028	1.872	highly similar to (968)AT4G19210| Symbols: ATRLI2 | ATRLI2 (Arabidopsis thaliana RNase L inhibitor protein 2) | chr4:10501917-1050	
JCVI_5229	1.872	moderately similar to (278)AT5G18420| Symbols: | similar to unknown [Picea sitchensis] (GB:ABK25419.1); contains domain PTHR159	
RC_ES266249	1.872	no similarity	
ES939591	1.872	no similarity	
JCVI_14129	1.871	moderately similar to (248)AT2G38110| Symbols: ATGPAT6, GPAT6 | ATGPAT6/GPAT6 (GLYCEROL-3-PHOSPHATE ACYLTRANS	
EV160950	1.871	moderately similar to (206)AT4G00040| Symbols: | chalcone and stilbene synthase family protein | chr4:14653-15897 FORWARDvery we	-3.000
JCVI_3348	1.871	weakly similar to (175)AT1G20696| Symbols: NFD3, NFD03, HMGB3 | HMGB3 (HIGH MOBILITY GROUP B 3) | chr1:7179815-7181	
EV010046	1.871	no similarity	
JCVI_14074	1.871	moderately similar to (327)AT4G32390| Symbols: | phosphate translocator-related | chr4:15636556-15637608 FORWARD no original des	
JCVI_41049	1.871	moderately similar to (211)AT1G67800| Symbols: | copine-related | chr1:25424692-25426900 REVERSE no original description	
ES902875	1.870	highly similar to (530)AT5G62600| Symbols: | transportin-SR-related | chr5:25140563-25149699 REVERSE [21432]	
EX028264	1.870	no similarity	
JCVI_39509	1.870	weakly similar to (138)AT3G53210| Symbols: | nodulin MtN21 family protein | chr3:19731160-19732742 FORWARD no original descrip	
JCVI_22729	1.870	weakly similar to (139)AT3G03440| Symbols: | armadillo/beta-catenin repeat family protein | chr3:815716-818575 FORWARD no origina	
JCVI_5260	1.870	highly similar to (511)AT3G27820| Symbols: ATMDAR4, MDAR4 | ATMDAR4/MDAR4 (MONODEHYDROASCORBATE REDUCTA	
EV144004	1.870	no similarity	1.600
AM058217	1.870	no similarity	
JCVI_38523	1.869	moderately similar to (472)AT1G18360| Symbols: | hydrolase, alpha/beta fold family protein | chr1:6316989-6319197 REVERSE no origi	
EE530590	1.869	moderately similar to (206)AT1G14560| Symbols: | mitochondrial substrate carrier family protein | chr1:4981295-4983077 FORWARD [2	
JCVI_26446	1.869	moderately similar to (207)AT3G03880| Symbols: | similar to unknown protein [Arabidopsis thaliana] (TAIR:AT1G55340.1); similar to u	-1.467
CD823006	1.869	no similarity	
JCVI_4700	1.869	weakly similar to (172)AT1G80460| Symbols: GLI1, NHO1 | NHO1 (NONHOST RESISTANCE TO P. S. PHASEOLICOLA 1); carbohyc	
JCVI_6864	1.869	highly similar to (619)AT2G24280| Symbols: | serine carboxypeptidase S28 family protein | chr2:10341702-10344007 FORWARD no orig	-1.268
JCVI_8269	1.869	weakly similar to (166)AT5G26170| Symbols: ATWRKY50, WRKY50 | WRKY50 (WRKY DNA-binding protein 50); transcription factor	
EV048906	1.869	very weakly similar to (83.6)AT3G09160| Symbols: | RNA recognition motif (RRM)-containing protein | chr3:2805784-2806917 REVERS	-1.693
EE520467	1.869	weakly similar to (154)AT1G43620| Symbols: | UDP-glucose:sterol glucosyltransferase, putative | chr1:16428094-16431940 REVERSE [2	
JCVI_6277	1.869	moderately similar to (467)AT2G38840| Symbols: | guanylate-binding family protein | chr2:16234407-16239193 FORWARD no original d	-2.537
JCVI_22421	1.869	moderately similar to (490)AT1G03740| Symbols: | protein kinase family protein | chr1:934055-936025 FORWARDmoderately similar to	
JCVI_11979	1.869	highly similar to (536)AT4G00660| Symbols: | DEAD/DEAH box helicase, putative | chr4:274638-277438 FORWARDweakly similar to (	1.250
JCVI_32395	1.868	moderately similar to (343)AT5G64360| Symbols: | DNAJ heat shock N-terminal domain-containing protein | chr5:25754434-25755828 R	
H07490	1.868	no similarity	
AM395895	1.868	weakly similar to (116)AT5G02230| Symbols: | haloacid dehalogenase-like hydrolase family protein | chr5:449130-450505 FORWARD [2	
EV155334	1.868	moderately similar to (300)AT5G44100| Symbols: CKL7 | CKL7 (Casein Kinase I-like 7); casein kinase I/ kinase | chr5:17766681-177695	
EX060606	1.868	weakly similar to (120)AT3G10525| Symbols: | similar to SIM (SIAMESE) [Arabidopsis thaliana] (TAIR:AT5G04470.1) | chr3:3281581-	
JCVI_36564	1.868	moderately similar to (290)AT1G26580| Symbols: | similar to myb family transcription factor / ELM2 domain-containing protein [Arabidc	
ES265148	1.868	moderately similar to (300)AT1G09770| Symbols: ATMYBCDC5, ATCDC5, CDC5 | ATCDC5 (ARABIDOPSIS THALIANA HOMOLO	2.313
JCVI_15736	1.868	moderately similar to (447)AT1G80600| Symbols: | acetylornithine aminotransferase, mitochondrial, putative / acetylornithine transamina	
EV056584	1.868	weakly similar to (189)AT3G13360| Symbols: WIP3 | WIP3 (WPP-DOMAIN INTERACTING PROTEIN 3) | chr3:4338479-4339989 REV	1.975
JCVI_1029	1.867	moderately similar to (225)AT3G54400| Symbols: | aspartyl protease family protein | chr3:20151269-20153577 REVERSE no original des	
JCVI_4170	1.867	moderately similar to (206)AT2G17710| Symbols: | similar to unnamed protein product [Vitis vinifera] (GB:CAO42932.1) | chr2:7700938	
AM388839	1.867	moderately similar to (331)AT1G02310| Symbols: | glycosyl hydrolase family protein 5 / cellulase family protein / (1-4)-beta-mannan endo	
CX281339	1.867	very weakly similar to (82.0)AT5G55125| Symbols: | similar to hypothetical protein OsI_035046 [Oryza sativa (indica cultivar-group)] (GI	
JCVI_36226	1.867	weakly similar to (187)AT3G59350| Symbols: | serine/threonine protein kinase, putative | chr3:21943907-21945860 FORWARDvery weal	
EE442102	1.867	very weakly similar to (82.0)AT4G32690| Symbols: GLB3 | GLB3 (2-on-2 hemoglobin like gene 3) | chr4:15765491-15766867 FORWARD	
JCVI_41737	1.867	no original description	
EV119559	1.867	very weakly similar to (95.5)AT5G17920| Symbols: ATMETS, ATMS1, ATCIMS | ATCIMS (COBALAMIN-INDEPENDENT METHION	1.441
EV073311	1.867	no similarity	
JCVI_8390	1.866	highly similar to (520)AT3G13682| Symbols: LDL2 | LDL2 (LSD1-LIKE2); amine oxidase | chr3:4479200-4481516 REVERSEvery weakl	
EV098696	1.866	moderately similar to (459)AT3G26020| Symbols: | serine/threonine protein phosphatase 2A (PP2A) regulatory subunit B', putative | chr3:	
JCVI_26778	1.866	weakly similar to (198)AT2G24570| Symbols: ATWRKY17, WRKY17 | WRKY17 (WRKY DNA-binding protein 17); transcription factor	
JCVI_17341	1.866	moderately similar to (398)AT3G01100| Symbols: ATHYP1, HYP1 | HYP1 (HYPOTHETICAL PROTEIN 1) | chr3:35817-38183 REVER	
JCVI_4270	1.866	moderately similar to (259)AT5G06430| Symbols: | thioredoxin-related | chr5:1963581-1964252 REVERSE no original description	
BQ704184	1.866	very weakly similar to (82.4)AT1G20340| Symbols: DRT112 | DRT112 (DNA-damage-repair/toleration protein 112); copper ion binding /	
EV017065	1.866	no similarity	
JCVI_20013	1.865	weakly similar to (164)AT5G45900| Symbols: ATAPG7, ATG7, APG7 | APG7 (AUTOPHAGY 7) | chr5:18632531-18635663 FORWARD	
L46452	1.865	very weakly similar to (99.0)AT5G57030| Symbols: LUT2 | LUT2 (LUTEIN DEFICIENT 2); lycopene epsilon cyclase | chr5:23094624-23	2.500
ES967943	1.865	moderately similar to (304)AT5G56790| Symbols: | protein kinase family protein | chr5:22985836-22988617 FORWARD [20153]	
JCVI_1123	1.865	moderately similar to (408)AT1G04680| Symbols: | pectate lyase family protein | chr1:1304051-1307779 REVERSEmoderately similar to	
JCVI_1124	1.865	moderately similar to (426)AT1G65930| Symbols: | isocitrate dehydrogenase, putative / NADP+ isocitrate dehydrogenase, putative | chr1:	
EE533980	1.865	weakly similar to (137)AT5G39500| Symbols: | pattern formation protein, putative | chr5:15832502-15837138 FORWARD [20150]	
EV190675	1.865	moderately similar to (219)AT5G10010| Symbols: | similar to unknown protein [Arabidopsis thaliana] (TAIR:AT5G64910.1); similar to 1	
JCVI_16886	1.864	moderately similar to (455)AT1G13195| Symbols: | zinc finger (C3HC4-type RING finger) family protein | chr1:4501775-4502852 REVE	1.795
JCVI_3396	1.864	highly similar to (593)AT5G41940| Symbols: | RabGAP/TBC domain-containing protein | chr5:16799267-16802679 FORWARD no origi	
EV204967	1.864	weakly similar to (192)AT1G79270| Symbols: ECT8 | ECT8 (evolutionarily conserved C-terminal region 8) | chr1:29821050-29823704 FC	
EX084772	1.864	moderately similar to (440)AT4G23180| Symbols: RLK4, CRK10 | CRK10 (CYSTEINE-RICH RLK10); kinase | chr4:12138182-1214079	
EL591513	1.864	weakly similar to (161)AT1G18520| Symbols: TET11 | TET11 (TETRASPANIN11) | chr1:6375044-6376171 FORWARD [20863]	

DN961759	1.864	weakly similar to (161)AT1G66880| Symbols: | serine/threonine protein kinase family protein | chr1:24950591-24959101 FORWARD [17	
JCVI_22810	1.864	very weakly similar to (86.3)AT5G09620| Symbols: | octicosapeptide/Phox/Bem1p (PB1) domain-containing protein | chr5:2983758-29853	
EV098804	1.864	moderately similar to (202)AT4G21700| Symbols: | similar to unknown protein [Arabidopsis thaliana] (TAIR:AT1G52780.1); similar to u	
DY030242	1.864	no similarity	
JCVI_25419	1.864	moderately similar to (437)AT1G65470| Symbols: NFB2, FAS1 | FAS1 (FASCIATA 1) | chr1:24323569-24327542 REVERSE no original	
EV219618	1.863	moderately similar to (248)AT4G00970| Symbols: | protein kinase family protein | chr4:418437-421694 FORWARD [21492] 50 737 737	
JCVI_28646	1.863	moderately similar to (435)AT1G04300| Symbols: | similar to meprin and TRAF homology domain-containing protein / MATH domain-co	
ES940303	1.863	weakly similar to (176)AT3G04590| Symbols: | DNA-binding family protein | chr3:1239251-1241609 REVERSE [21391]	
CX188578	1.863	moderately similar to (218)AT1G29790| Symbols: | similar to (ARABIDOPSIS THALIANA RAS ASSOCIATED WITH DIABETES PRO	
JCVI_13138	1.863	weakly similar to (185)AT4G08580| Symbols: | microfibrillar-associated protein-related | chr4:5462187-5463715 FORWARD no original	
JCVI_8130	1.863	highly similar to (534)AT1G66520| Symbols: PDE194 | PDE194 (PIGMENT DEFECTIVE 194); formyltetrahydrofolate deformylase/ hyd	
JCVI_27917	1.863	moderately similar to (269)AT5G67360| Symbols: ARA12 | ARA12; subtilase | chr5:26889418-26891691 REVERSE no original descriptio	
EV076295	1.862	weakly similar to (180)AT1G51790| Symbols: | kinase | chr1:19210527-19214243 REVERSE [21443]	
JCVI_31429	1.862	weakly similar to (164)AT3G62660| Symbols: GATL7 | GATL7 (Galacturonosyltransferase-like 7); polygalacturonate 4-alpha-galacturono	
JCVI_7482	1.862	moderately similar to (400)AT5G49555| Symbols: | amine oxidase-related | chr5:20124637-20127828 REVERSE no original description	
JCVI_1690	1.862	moderately similar to (258)AT4G25830| Symbols: | integral membrane family protein | chr4:13133736-13134799 FORWARD no original	
JCVI_41586	1.862	moderately similar to (273)AT5G47080| Symbols: CKB1 | CKB1 (casein kinase II beta chain 1); protein kinase CK2 regulator | chr5:1914	
CX272957	1.862	moderately similar to (258)AT4G24550| Symbols: | clathrin adaptor complexes medium subunit family protein | chr4:12675883-12678913	
JCVI_3671	1.862	moderately similar to (345)AT5G53970| Symbols: | aminotransferase, putative | chr5:21927902-21929820 FORWARD no original descrip	
EV114040	1.861	no similarity	
JCVI_40969	1.861	no original description	
JCVI_7402	1.861	weakly similar to (155)AT1G52720| Symbols: | similar to unknown protein [Arabidopsis thaliana] (TAIR:AT3G15630.1); similar to unnar	
JCVI_1070	1.861	highly similar to (527)AT5G63140| Symbols: ATPAP29, PAP29 | ATPAP29/PAP29 (purple acid phosphatase 29); acid phosphatase/ prote	
JCVI_2270	1.861	highly similar to (550)AT3G57290| Symbols: TIF3E1, ATEIF3E-1, INT-6, ATINT6, INT6, EIF3E | EIF3E (eukaryotic translation initiatio	
JCVI_7911	1.861	moderately similar to (355)AT3G51430| Symbols: YLS2 | YLS2 (yellow-leaf-specific gene 2); strictosidine synthase | chr3:19097527-1909	
RC_EE558426	1.861	no similarity	
JCVI_31888	1.861	weakly similar to (160)AT1G51060| Symbols: HTA10 | HTA10; DNA binding | chr1:18930616-18931111 FORWARDweakly similar to (	2.002
EG019853	1.861	no similarity	
JCVI_13916	1.860	moderately similar to (399)AT1G80210| Symbols: | similar to mov34 family protein [Arabidopsis thaliana] (TAIR:AT3G06820.2); similar	
JCVI_32837	1.860	moderately similar to (384)AT5G06580| Symbols: | FAD linked oxidase family protein | chr5:2011487-2016474 REVERSE no original de	
EE471943	1.860	moderately similar to (209)AT4G32980| Symbols: ATH1 | ATH1 (ARABIDOPSIS THALIANA HOMEOBOX GENE 1); transcription fac	
JCVI_22724	1.860	moderately similar to (384)AT2G43410| Symbols: FPA | FPA | chr2:18033474-18038066 REVERSE no original description	
ES941924	1.860	weakly similar to (135)AT3G57070| Symbols: | glutaredoxin family protein | chr3:21135153-21136406 FORWARD [21391]	
JCVI_29399	1.860	no original description	
EX118172	1.859	moderately similar to (204)AT4G02890| Symbols: UBQ14 | UBQ14 (ubiquitin 14) | chr4:1278747-1279664 REVERSEweakly similar to (	
ES931698	1.859	no similarity	
JCVI_32158	1.859	no original description	
EE515524	1.859	no similarity	
JCVI_41290	1.859	moderately similar to (275)AT3G53530| Symbols: | heavy-metal-associated domain-containing protein | chr3:19856177-19857561 FORW	
ES994527	1.858	very weakly similar to (89.7)AT1G24270| Symbols: | similar to unknown protein [Arabidopsis thaliana] (TAIR:AT5G13090.1); similar to u	-1.756
EE506307	1.858	moderately similar to (320)AT1G62260| Symbols: | pentatricopeptide (PPR) repeat-containing protein | chr1:23001491-23003461 REVER	
JCVI_21397	1.858	moderately similar to (390)AT2G32660| Symbols: | disease resistance family protein / LRR family protein | chr2:13860974-13862743 RE	
JCVI_208	1.858	moderately similar to (215)AT2G33380| Symbols: RD20 | RD20 (RESPONSIVE TO DESSICATION 20); calcium ion binding | chr2:1415	
JCVI_20668	1.858	weakly similar to (183)AT3G14420| Symbols: | (S)-2-hydroxy-acid oxidase, peroxisomal, putative / glycolate oxidase, putative / short cha	
EX088242	1.857	weakly similar to (128)AT1G55310| Symbols: ATSCL33, SCL33, SR33 | SR33 (SC35-like splicing factor 33); RNA binding | chr1:206343	
EV224125	1.857	very weakly similar to (92.0)AT1G11660| Symbols: | heat shock protein, putative | chr1:3921056-3924347 FORWARD [21493] 39 728 72	
JCVI_38810	1.857	weakly similar to (155)AT5G17680| Symbols: | disease resistance protein (TIR-NBS-LRR class), putative | chr5:5823001-5827155 FORW	
EX100716	1.857	highly similar to (518)AT5G10630| Symbols: | elongation factor 1-alpha, putative / EF-1-alpha, putative | chr5:3360562-3364415 FORWA	
RC_DY000029	1.857	no similarity	-1.713
EX104309	1.857	weakly similar to (175)AT1G05190| Symbols: EMB2394 | EMB2394 (EMBRYO DEFECTIVE 2394); structural constituent of ribosome |	
EE428698	1.857	weakly similar to (122)AT5G42650| Symbols: CYP74A, AOS | AOS (ALLENE OXIDE SYNTHASE); hydro-lyase/ oxygen binding | chr5	
JCVI_21775	1.857	moderately similar to (219)AT4G28990| Symbols: | RNA-binding protein-related | chr4:14291211-14293024 FORWARD no original desc	
JCVI_4762	1.856	moderately similar to (338)AT3G51870| Symbols: | mitochondrial substrate carrier family protein | chr3:19254955-19257588 FORWARD	1.518
ES264348	1.856	no similarity	
JCVI_28315	1.855	moderately similar to (418)AT1G08220| Symbols: | similar to unnamed protein product [Vitis vinifera] (GB:CAO16719.1); contains Inter	1.918
JCVI_10342	1.855	moderately similar to (383)AT3G09100| Symbols: | mRNA capping enzyme family protein | chr3:2788440-2792918 REVERSE no origina	
EV196747	1.855	very weakly similar to (92.4)AT1G08480| Symbols: | similar to unknown [Populus trichocarpa] (GB:ABK92625.1) | chr1:2684343-268539	
EX137970	1.855	moderately similar to (326)AT5G39610| Symbols: ANAC092, ATNAC6, ATNAC2 | ANAC092/ATNAC2/ATNAC6 (Arabidopsis NAC do	
JCVI_1277	1.855	moderately similar to (453)AT2G39780| Symbols: RNS2 | RNS2 (RIBONUCLEASE 2) | chr2:16598334-16600593 FORWARD no origina	
CD827840	1.854	weakly similar to (130)AT1G19200| Symbols: | senescence-associated protein-related | chr1:6625095-6625847 REVERSE [13979]	
JCVI_29443	1.854	highly similar to (511)AT3G17970| Symbols: ATTOC64-III | ATTOC64-III (ARABIDOPSIS THALIANA TRANSLOCON AT THE OUT	
JCVI_15875	1.854	moderately similar to (486)AT5G18630| Symbols: | lipase class 3 family protein | chr5:6202967-6205450 FORWARD no original descript	1.784
JCVI_36070	1.854	highly similar to (546)AT4G10710| Symbols: SPT16 | SPT16 (GLOBAL TRANSCRIPTION FACTOR C); metalloexopeptidase | chr4:660	
JCVI_23025	1.854	moderately similar to (340)AT1G23170| Symbols: | similar to unknown protein [Arabidopsis thaliana] (TAIR:AT1G70770.1); similar to h	
JCVI_26299	1.854	moderately similar to (289)AT4G09560| Symbols: | peptidase/ protein binding / zinc ion binding | chr4:6041649-6043678 REVERSE no o	-3.189
EX070185	1.854	no similarity	
EX040864	1.854	moderately similar to (223)AT1G11940| Symbols: | similar to unknown protein [Arabidopsis thaliana] (TAIR:AT1G62305.1); similar to u	
EE535007	1.853	no similarity	
CX279198	1.853	weakly similar to (119)AT4G38550| Symbols: | similar to unknown protein [Arabidopsis thaliana] (TAIR:AT2G20950.1); contains InterPr	
JCVI_18968	1.853	moderately similar to (452)AT5G51590| Symbols: | DNA-binding protein-related | chr5:20974089-20976155 REVERSE no original descr	-3.316
JCVI_2432	1.853	moderately similar to (430)AT4G39090| Symbols: RD19A, RD19 | RD19 (RESPONSIVE TO DEHYDRATION 19); cysteine-type peptida	
EE460230	1.853	weakly similar to (118)AT3G61800| Symbols: | similar to unnamed protein product [Vitis vinifera] (GB:CAO14384.1); contains InterPro	
CX266456	1.853	no similarity	
JCVI_11792	1.853	moderately similar to (261)AT5G24580| Symbols: | copper-binding family protein | chr5:8410397-8412090 REVERSE no original descrip	
JCVI_3605	1.853	moderately similar to (248)AT3G46940| Symbols: | deoxyuridine 5'-triphosphate nucleotidohydrolase family | chr3:17299352-17299852 F	
RC_ES937978	1.853	no similarity	
EX062695	1.853	no similarity	
JCVI_33523	1.852	no original description	
JCVI_31923	1.852	weakly similar to (137)AT2G34480| Symbols: | 60S ribosomal protein L18A (RPL18aB) | chr2:14539995-14541240 REVERSEweakly sir	
JCVI_35845	1.852	weakly similar to (142)AT3G12130| Symbols: | KH domain-containing protein / zinc finger (CCCH type) family protein | chr3:3864492-3	
JCVI_26656	1.852	very weakly similar to (85.5)AT3G46220| Symbols: | similar to unnamed protein product [Vitis vinifera] (GB:CAO21736.1) | chr3:169915	

JCVI_37794	1.851	weakly similar to (147)AT5G01830| Symbols: | armadillo/beta-catenin repeat family protein / U-box domain-containing protein | chr5:320	
JCVI_18228	1.851	moderately similar to (293)AT4G00752| Symbols: | UBX domain-containing protein | chr4:317771-320793 REVERSE no original descrip	
JCVI_8014	1.851	moderately similar to (214)AT2G33735| Symbols: | DNAJ heat shock N-terminal domain-containing protein | chr2:14275608-14276470 R	
JCVI_19056	1.851	weakly similar to (108)AT1G62310| Symbols: | transcription factor jumonji (jmjC) domain-containing protein | chr1:23039704-23042966	
CN731264	1.851	moderately similar to (261)AT2G26150| Symbols: HSFA2, ATHSFA2 | ATHSFA2 (Arabidopsis thaliana heat shock transcription factor A	
ES902427	1.851	moderately similar to (455)AT3G61270| Symbols: | similar to DTA2 (DOWNSTREAM TARGET OF AGL15 2) [Arabidopsis thaliana] (T	
JCVI_33330	1.851	highly similar to (822)AT1G65730| Symbols: YSL7 | YSL7 (YELLOW STRIPE LIKE 7); oligopeptide transporter | chr1:24446302-24449	
CX195283	1.851	weakly similar to (144)AT4G38010| Symbols: | pentatricopeptide (PPR) repeat-containing protein | chr4:17859576-17861255 REVERSE	
JCVI_2533	1.851	moderately similar to (273)AT3G63490| Symbols: | ribosomal protein L1 family protein | chr3:23455244-23456752 FORWARDmoderate	
EV136551	1.851	no similarity	
JCVI_7180	1.851	moderately similar to (407)AT1G48040| Symbols: | protein serine/threonine phosphatase | chr1:17723733-17725367 REVERSE no origin	
JCVI_32568	1.850	moderately similar to (409)AT1G12110| Symbols: CHL1-1, NRT1, B-1, ATNRT1, CHL1, NRT1.1 | NRT1.1 (NITRATE TRANSPORTER	
JCVI_18368	1.850	moderately similar to (227)AT3G06420| Symbols: ATG8H | ATG8H (AUTOPHAGY 8H); microtubule binding | chr3:1955225-1956280 F	
EE428599	1.850	no similarity	
JCVI_29110	1.850	moderately similar to (323)AT5G66010| Symbols: | RNA binding | chr5:26416838-26417979 FORWARD no original description	
JCVI_28125	1.850	moderately similar to (407)AT3G59480| Symbols: | pfkB-type carbohydrate kinase family protein | chr3:21994080-21995417 FORWARD	
JCVI_6919	1.850	moderately similar to (319)AT3G57020| Symbols: | strictosidine synthase family protein | chr3:21109494-21111282 REVERSE no origina	
ES984759	1.849	no similarity	
JCVI_16754	1.849	weakly similar to (167)AT3G53490| Symbols: | similar to unknown protein [Arabidopsis thaliana] (TAIR:AT5G02720.1); similar to Os03	
JCVI_17646	1.849	moderately similar to (243)AT4G23060| Symbols: IQD22 | IQD22 (IQ-domain 22); calmodulin binding | chr4:12087294-12090419 FORW	
JCVI_19247	1.849	weakly similar to (140)AT5G03030| Symbols: | DNAJ heat shock N-terminal domain-containing protein | chr5:708477-709511 REVERSE	
JCVI_8253	1.849	moderately similar to (245)AT4G35840| Symbols: | zinc finger (C3HC4-type RING finger) family protein | chr4:16981087-16982269 FO	
JCVI_18069	1.849	moderately similar to (291)AT3G10815| Symbols: | zinc finger (C3HC4-type RING finger) family protein | chr3:3385014-3385613 REVE	
EE530440	1.849	no similarity	
JCVI_5630	1.849	moderately similar to (421)AT3G02060| Symbols: | DEAD/DEAH box helicase, putative | chr3:354419-358326 FORWARD no original de	
JCVI_2495	1.848	moderately similar to (251)AT1G50320| Symbols: ATX, ATHX | ATHX (THIOREDOXIN X); thiol-disulfide exchange intermediate | chr	
JCVI_353	1.848	moderately similar to (292)AT1G71860| Symbols: ATPTP1, PTP1 | PTP1 (PROTEIN TYROSINE PHOSPHATASE 1) | chr1:27030528-2	
JCVI_3054	1.848	moderately similar to (399)AT5G66460| Symbols: | (1-4)-beta-mannan endohydrolase, putative | chr5:26556137-26558063 REVERSE no	
JCVI_39929	1.848	moderately similar to (329)AT1G08125| Symbols: | Expressed protein | chr1:2539745-2542227 REVERSE no original description	
EV049538	1.848	moderately similar to (342)AT2G02980| Symbols: | pentatricopeptide (PPR) repeat-containing protein | chr2:868467-870278 FORWARD	
ES906249	1.848	weakly similar to (191)AT4G00650| Symbols: FLA, FRI | FRI (FRIGIDA) | chr4:269026-270363 FORWARD [21429]	
JCVI_4639	1.847	highly similar to (546)AT3G14680| Symbols: CYP72A14 | CYP72A14 (cytochrome P450, family 72, subfamily A, polypeptide 14); oxyge	
JCVI_28844	1.847	moderately similar to (483)AT2G32700| Symbols: | WD-40 repeat family protein | chr2:13874312-13878921 FORWARD no original desc	
EV170956	1.847	moderately similar to (299)AT1G76130| Symbols: ATAMY2, AMY2 | AMY2/ATAMY2 (ALPHA-AMYLASE-LIKE 2); alpha-amylase | c	
EV112793	1.847	weakly similar to (112)AT1G50600| Symbols: SCL5 | SCL5; transcription factor | chr1:18741066-18743215 REVERSE [21479] 543 967 9	
EV061106	1.847	weakly similar to (115)AT1G30170| Symbols: | similar to unknown protein [Arabidopsis thaliana] (TAIR:AT1G30160.2); contains InterP	-4.019
JCVI_7733	1.847	highly similar to (503)AT5G51290| Symbols: | ceramide kinase-related | chr5:20861478-20865383 REVERSE no original description	
EV088649	1.847	moderately similar to (241)AT3G07390| Symbols: AIR12 | AIR12 (Auxin-Induced in Root cultures 12); extracellular matrix structural con	
JCVI_5479	1.847	moderately similar to (452)AT2G38110| Symbols: ATGPAT6, GPAT6 | ATGPAT6/GPAT6 (GLYCEROL-3-PHOSPHATE ACYLTRANS	
JCVI_10206	1.846	very weakly similar to (90.9)AT3G26730| Symbols: | zinc finger (C3HC4-type RING finger) family protein | chr3:9825007-9828507 FORV	
CD821291	1.846	no similarity	
JCVI_9148	1.846	no original description	
JCVI_22700	1.846	weakly similar to (196)AT5G59490| Symbols: | haloacid dehalogenase-like hydrolase family protein | chr5:24000148-24001645 REVERS	
JCVI_12363	1.846	highly similar to (894)AT1G30810| Symbols: | transcription factor jumonji (jmj) family protein / zinc finger (C5HC2 type) family protein	
EE479756	1.846	no similarity	
JCVI_9594	1.846	weakly similar to (164)AT3G29185| Symbols: | similar to hypothetical protein MtrDRAFT_AC147482g2v2 [Medicago truncatula] (GB:A	
JCVI_16035	1.845	no original description	
JCVI_13890	1.845	highly similar to (511)AT1G21440| Symbols: | mutase family protein | chr1:7502314-7504092 REVERSEmoderately similar to (325)CPF	
DY006660	1.845	weakly similar to (197)AT2G41705| Symbols: | camphor resistance CrcB family protein | chr2:17405161-17406988 FORWARD [18976]	
ES967361	1.845	no similarity	
JCVI_28195	1.845	moderately similar to (244)AT5G46860| Symbols: ATVAM3, SYP22, ATSYP22, SGR3, VAM3 | VAM3 (syntaxin 22); SNAP receptor | c	
H06421	1.845	no similarity	
DY025481	1.845	moderately similar to (224)AT5G16620| Symbols: TIC40, ATTIC40, PDE120 | PDE120 (PIGMENT DEFECTIVE EMBRYO) | chr5:545(	-1.515
JCVI_8945	1.845	highly similar to (576)AT1G31930| Symbols: XLG3 | XLG3 (EXTRA-LARGE GTP-BINDING PROTEIN 3); signal transducer | chr1:114	
EE527292	1.845	no similarity	
JCVI_26828	1.844	highly similar to (691)AT3G24040| Symbols: | glycosyltransferase family 14 protein / core-2/I-branching enzyme family protein | chr3:868	
JCVI_18110	1.844	moderately similar to (355)AT4G26120| Symbols: | ankyrin repeat family protein / BTB/POZ domain-containing protein | chr4:13236457-	
JCVI_10927	1.844	very weakly similar to (82.0)AT4G36440| Symbols: | similar to unnamed protein product [Vitis vinifera] (GB:CAO48295.1) | chr4:172072	
CD830341	1.844	moderately similar to (239)AT5G40240| Symbols: | nodulin MtN21 family protein | chr5:16099553-16101694 REVERSE [13980]	
DY026544	1.844	moderately similar to (213)AT5G06550| Symbols: | Identical to F-box protein At5g06550 [Arabidopsis Thaliana] (GB:Q67XX3;GB:Q9F(	
EV057320	1.844	weakly similar to (128)AT5G02580| Symbols: | similar to unknown protein [Arabidopsis thaliana] (TAIR:AT3G55240.1); similar to hypot	
AT002102	1.844	very weakly similar to (87.8)AT3G46560| Symbols: EMB2474, TIM9 | TIM9 (EMBRYO DEFECTIVE 2474); P-P-bond-hydrolysis-driven	-1.193
JCVI_33917	1.844	no original description	
EE434796	1.844	no similarity	
EX036128	1.844	moderately similar to (273)AT3G06240| Symbols: | F-box family protein | chr3:1887342-1888625 FORWARD [21811] 16 722 722	
JCVI_7209	1.843	weakly similar to (166)AT5G01650| Symbols: | macrophage migration inhibitory factor family protein / MIF family protein | chr5:242731-	
JCVI_11923	1.843	weakly similar to (188)AT1G61040| Symbols: VIP5 | VIP5 (VERNALIZATION INDEPENDENCE 5) | chr1:22487482-22489413 FORW	
JCVI_40577	1.843	highly similar to (503)AT1G78570| Symbols: RHM1, ROL1 | RHM1/ROL1 (RHAMNOSE BIOSYNTHESIS1); UDP-L-rhamnose synthas	
JCVI_31089	1.843	highly similar to (697)AT2G31170| Symbols: | tRNA synthetase class I (C) family protein | chr2:13289636-13291333 REVERSE no origi	
EE516325	1.843	no similarity	
JCVI_19878	1.843	no original description	
JCVI_26000	1.843	highly similar to (575)AT5G04140| Symbols: GLU1, GLS1, GLUS | GLS1/GLU1/GLUS (FERREDOXIN-DEPENDENT GLUTAMATE S	
CD836426	1.842	moderately similar to (258)AT1G80530| Symbols: | nodulin family protein | chr1:30283116-30285139 REVERSE [13981]	
EV151791	1.842	no similarity	
ES975765	1.842	no similarity	
EX047635	1.842	moderately similar to (230)AT1G51450| Symbols: | SPla/RYanodine receptor (SPRY) domain-containing protein | chr1:19078067-190798	
JCVI_4549	1.842	moderately similar to (317)AT2G39310| Symbols: | jacalin lectin family protein | chr2:16421340-16423401 REVERSE no original descrip	
RC_EV225491	1.842	no similarity	
JCVI_17467	1.842	no original description	
JCVI_7573	1.842	highly similar to (554)AT2G02040| Symbols: PTR2-B, NTR1, ATPTR2-B | ATPTR2-B (NITRATE TRANSPORTER 1); transporter | chr	
JCVI_2426	1.842	moderately similar to (345)AT4G00430| Symbols: PIP1;4, PIP1E, TMP-C | TMP-C (PLASMA MEMBRANE INTRINSIC PROTEIN 1;4	1.589

EE505193	1.841	very weakly similar to (97.4)AT4G34540| Symbols: | isoflavone reductase family protein | chr4:16500511-16501937 FORWARD [20139]	
JCVI_27897	1.841	very weakly similar to (94.0)AT5G53350| Symbols: CLPX | CLPX (Clp protease regulatory subunit X); ATPase | chr5:21661286-2166472	
JCVI_7190	1.841	moderately similar to (367)AT1G14740| Symbols: | similar to unknown protein [Arabidopsis thaliana] (TAIR:AT3G63500.2); similar to u	
EX094232	1.841	no similarity	
EX090230	1.841	weakly similar to (110)AT1G35460| Symbols: | basic helix-loop-helix (bHLH) family protein | chr1:13040070-13041885 FORWARD [21	
JCVI_35233	1.841	moderately similar to (288)AT1G45180| Symbols: | zinc finger (C3HC4-type RING finger) family protein | chr1:17101775-17104123 FO	-2.163
JCVI_35634	1.840	weakly similar to (197)AT1G55540| Symbols: EMB1011 | EMB1011 (EMBRYO DEFECTIVE 1011) | chr1:20738425-20741660 REVER	
JCVI_30444	1.840	very weakly similar to (87.4)AT4G19112| Symbols: CPuORF25 | CPuORF25 (Conserved peptide upstream open reading frame 25) | chr4:1	
JCVI_901	1.840	moderately similar to (452)AT5G53850| Symbols: | haloacid dehalogenase-like hydrolase family protein | chr5:21878843-21882043 REV	
DY018704	1.840	weakly similar to (138)AT1G06140| Symbols: | pentatricopeptide (PPR) repeat-containing protein | chr1:1864795-1866471 FORWARD [	-1.366
JCVI_4102	1.840	moderately similar to (349)AT4G16845| Symbols: VRN2 | VRN2 (REDUCED VERNALIZATION RESPONSE 2) | chr4:9477265-947974	
EX134586	1.840	moderately similar to (442)AT1G20480| Symbols: | 4-coumarate--CoA ligase family protein / 4-coumaroyl-CoA synthase family protein |	
JCVI_19918	1.840	highly similar to (573)AT3G45260| Symbols: | zinc finger (C2H2 type) family protein | chr3:16607835-16609535 REVERSE no original c	
JCVI_775	1.840	moderately similar to (424)AT5G08400| Symbols: | similar to unknown protein [Arabidopsis thaliana] (TAIR:AT4G29400.1); similar to u	
ES983206	1.840	weakly similar to (107)AT3G47930| Symbols: ATGLDH | ATGLDH (L-GALACTONO-1,4-LACTONE DEHYDROGENASE); FAD bi	
CV433045	1.839	no similarity	
JCVI_6204	1.839	moderately similar to (397)AT1G06290| Symbols: ATACX3, ACX3 | ACX3 (ACYL-COA OXIDASE 3); acyl-CoA oxidase | chr1:192242	
JCVI_29995	1.839	moderately similar to (403)AT1G31220| Symbols: | phosphoribosylglycinamide formyltransferase | chr1:11157045-11158389 FORWARD	
JCVI_15211	1.839	weakly similar to (142)AT1G12910| Symbols: ATAN11 | ATAN11 (ANTHOCYANIN11); nucleotide binding | chr1:4395112-4396152 RE	
EV123844	1.839	moderately similar to (359)AT5G23580| Symbols: ATCDPK9, CPK12, CDPK9 | CDPK9 (CALCIUM-DEPENDENT PROTEIN KINASE	
CX187593	1.839	no similarity	
EX037512	1.839	weakly similar to (160)AT1G79640| Symbols: | kinase | chr1:29971806-29975983 REVERSE [21811]	
JCVI_12410	1.838	weakly similar to (124)AT5G53650| Symbols: | similar to unnamed protein product [Vitis vinifera] (GB:CAO48588.1) | chr5:21808491-2	
EX126534	1.838	weakly similar to (118)AT3G55450| Symbols: | protein kinase, putative | chr3:20569106-20570940 FORWARD [21831]	
JCVI_27877	1.838	moderately similar to (424)AT3G26090| Symbols: ATRGS1, RGS1 | RGS1 (REGULATOR OF G-PROTEIN SIGNALING 1) | chr3:95341	
JCVI_1081	1.838	highly similar to (508)AT4G13430| Symbols: | aconitase family protein / aconitate hydratase family protein | chr4:7804190-7807785 REV	
DY015439	1.838	no similarity	
JCVI_11194	1.838	moderately similar to (488)AT4G04040| Symbols: MEE51 | MEE51 (maternal effect embryo arrest 51); diphosphate-fructose-6-phosphate	
EX126503	1.838	moderately similar to (292)AT2G41900| Symbols: | zinc finger (CCCH-type) family protein | chr2:17498430-17500580 FORWARD [218	
CD819397	1.838	weakly similar to (143)AT1G50710| Symbols: | similar to unnamed protein product [Vitis vinifera] (GB:CAO38953.1); contains domain F	
EV163185	1.838	moderately similar to (334)AT4G25880| Symbols: APUM6 | APUM6 (ARABIDOPSIS PUMILIO 6); RNA binding | chr4:13155527-1315	
JCVI_17249	1.838	moderately similar to (392)AT2G40980| Symbols: | ATP binding / protein kinase | chr2:17109168-17111521 FORWARD no original desc	
ES939084	1.837	weakly similar to (112)AT4G00660| Symbols: | DEAD/DEAH box helicase, putative | chr4:274638-277438 FORWARD [21390]	
JCVI_35253	1.837	highly similar to (540)AT4G26870| Symbols: | aspartyl-tRNA synthetase, putative / aspartate--tRNA ligase, putative | chr4:13505387-135	
EV088042	1.837	moderately similar to (447)AT2G39890| Symbols: ATPROT1, ProT1 | ProT1 (PROLINE TRANSPORTER 1) | chr2:16663100-16665280	
EV086031	1.836	no similarity	
ES945054	1.836	moderately similar to (241)AT5G27710| Symbols: | similar to unnamed protein product [Vitis vinifera] (GB:CAO17522.1) | chr5:9813073	
EV117286	1.836	moderately similar to (210)AT5G12850| Symbols: | zinc finger (CCCH-type) family protein | chr5:4057071-4059191 FORWARD [21479	
JCVI_38091	1.835	no original description	
JCVI_17359	1.835	moderately similar to (347)AT1G19450| Symbols: | integral membrane protein, putative / sugar transporter family protein | chr1:6731662-	
EV134094	1.835	no similarity	
EX111290	1.835	moderately similar to (325)AT5G55990| Symbols: ATCBL2, CBL2 | CBL2 (calcineurin B-like protein 2); calcium ion binding | chr5:2268	
JCVI_16250	1.835	highly similar to (526)AT5G24810| Symbols: | ABC1 family protein | chr5:8516905-8522619 REVERSE no original description	
JCVI_7548	1.835	moderately similar to (313)AT1G19180| Symbols: JAZ1, TIFY10A | JAZ1/TIFY10A (JASMONATE-ZIM-DOMAIN PROTEIN 1) | chr1:	1.933
JCVI_36512	1.834	moderately similar to (433)AT1G13640| Symbols: | phosphatidylinositol 3- and 4-kinase family protein | chr1:4677273-4679141 REVERS	
JCVI_35904	1.834	moderately similar to (261)AT1G60960| Symbols: IRT3 | IRT3 (Iron regulated transporter 3); cation transmembrane transporter/ metal ior	
EE540514	1.834	no similarity	
JCVI_36748	1.834	moderately similar to (353)AT3G13226| Symbols: | regulatory protein RecX family protein | chr3:4264449-4266293 REVERSE no origina	
EE549092	1.834	no similarity	
ES969060	1.834	no similarity	-1.550
ES936820	1.834	weakly similar to (102)AT1G74690| Symbols: IQD31 | IQD31 (IQ-domain 31); calmodulin binding | chr1:28065159-28067585 REVERSE	
EV012048	1.834	no similarity	
AM385552	1.834	moderately similar to (231)AT5G55530| Symbols: | C2 domain-containing protein | chr5:22511665-22512882 FORWARD [20118] 20 49	2.301
EV043299	1.834	moderately similar to (262)AT1G22490| Symbols: | basic helix-loop-helix (bHLH) family protein | chr1:7938437-7940478 REVERSE [21	
EE566815	1.833	no similarity	
JCVI_3987	1.833	moderately similar to (353)AT2G15290| Symbols: ATTIC21, TIC21, CIA5, PIC1 | ATTIC21/CIA5/PIC1/TIC21 (CHLOROPLAST IMPC	
JCVI_8242	1.833	moderately similar to (296)AT1G17860| Symbols: | trypsin and protease inhibitor family protein / Kunitz family protein | chr1:6149336-6	
CD825945	1.833	moderately similar to (364)AT5G50280| Symbols: EMB1006 | EMB1006 (EMBRYO DEFECTIVE 1006) | chr5:20476464-20478730 FOI	
EV058972	1.833	no similarity	
ES980499	1.833	moderately similar to (280)AT5G16830| Symbols: PEP12P, SYP21, ATSYP21, PEP12, ATPEP12 | SYP21 (syntaxin 21); SNAP receptor	
CV546883	1.833	weakly similar to (177)AT5G58950| Symbols: | protein kinase family protein | chr5:23818362-23820251 REVERSE [16551]	
JCVI_22879	1.832	very weakly similar to (83.6)AT2G44730| Symbols: | transcription factor | chr2:18444522-18445640 REVERSE no original description	1.801
RC_JCVI_35228	1.832	no original description	
JCVI_26809	1.832	moderately similar to (301)AT1G60850| Symbols: AAC42, ATRPAC42 | ATRPAC42 (Arabidopsis thaliana RNA polymerase I subunit 42	
DN963281	1.831	weakly similar to (177)AT3G45140| Symbols: ATLOX2, LOX2 | LOX2 (LIPOXYGENASE 2) | chr3:16536422-16540218 FORWARDwe	
JCVI_18571	1.831	moderately similar to (346)AT2G39220| Symbols: PLP6, PLA IIB | PLA IIB/PLP6 (Patatin-like protein 6); nutrient reservoir | chr2:16382	
EX112489	1.831	weakly similar to (194)AT3G28970| Symbols: AAR3 | AAR3 (ANTIAUXIN-RESISTANT 3) | chr3:10989207-10991019 REVERSE [218	
JCVI_27706	1.831	moderately similar to (465)AT1G32930| Symbols: | galactosyltransferase family protein | chr1:11931960-11934379 REVERSE no origina	
JCVI_1431	1.831	moderately similar to (413)AT5G23990| Symbols: ATFRO5, FRO5 | ATFRO5/FRO5 (FERRIC REDUCTION OXIDASE 5); ferric-chelat	
EX074863	1.831	moderately similar to (435)AT5G03260| Symbols: LAC11 | LAC11 (laccase 11); copper ion binding / oxidoreductase | chr5:777197-77933	
EX059522	1.831	moderately similar to (247)AT4G38225| Symbols: | similar to unnamed protein product [Vitis vinifera] (GB:CAO47538.1); contains doma	
JCVI_28933	1.830	moderately similar to (245)AT1G30640| Symbols: | protein kinase, putative | chr1:10861279-10864682 FORWARD no original descriptio	
JCVI_35385	1.830	moderately similar to (273)AT3G07565| Symbols: | DNA binding | chr3:2413829-2415878 FORWARD no original description	
JCVI_38479	1.830	moderately similar to (447)AT3G63410| Symbols: VTE3, APG1 | APG1 (ALBINO OR PALE GREEN MUTANT 1); methyltransferase | c	
JCVI_41539	1.830	weakly similar to (140)AT2G42570| Symbols: | similar to unknown protein [Arabidopsis thaliana] (TAIR:AT2G31110.2); similar to unkn	-3.147
EE417437	1.830	moderately similar to (213)AT2G19560| Symbols: | proteasome protein-related | chr2:8473006-8476151 REVERSE [20146] 1 560 578	
EV086074	1.829	weakly similar to (152)AT3G57050| Symbols: CBL | CBL (CYSTATHIONINE BETA-LYASE) | chr3:21122918-21125500 REVERSE [2	
ES921935	1.829	weakly similar to (153)AT5G48830| Symbols: | similar to unnamed protein product [Vitis vinifera] (GB:CAO46087.1) | chr5:19817495-1	
JCVI_8190	1.829	moderately similar to (322)AT1G65310| Symbols: ATXTH17 | ATXTH17 (XYLOGLUCAN ENDOTRANSGLUCOSYLASE/HYDROLA	
JCVI_13481	1.829	moderately similar to (296)AT5G66280| Symbols: GMD1 | GMD1 (GDP-D-MANNOSE 4,6-DEHYDRATASE 1); GDP-mannose 4,6-deh	
EV173184	1.829	weakly similar to (135)AT3G14680| Symbols: CYP72A14 | CYP72A14 (cytochrome P450, family 72, subfamily A, polypeptide 14); oxyg	

RC_CO749971	1.829	no similarity	
EE454844	1.829	weakly similar to (129)AT4G07390| Symbols: | PQ-loop repeat family protein / transmembrane family protein | chr4:4195747-4197314 FO	
EX103541	1.829	moderately similar to (398)AT3G07090| Symbols: | similar to unknown protein [Arabidopsis thaliana] (TAIR:AT5G25170.1); similar to u	
JCVI_26166	1.828	moderately similar to (281)AT5G27280| Symbols: | zinc finger (DNL type) family protein | chr5:9617551-9618372 FORWARD no origina	-1.986
DY001166	1.828	weakly similar to (155)AT5G67360| Symbols: ARA12 | ARA12; subtilase | chr5:26889418-26891691 REVERSE [18967]	1.976
ES941586	1.828	very weakly similar to (88.6)AT1G53190| Symbols: | zinc finger (C3HC4-type RING finger) family protein | chr1:19841740-19843671 FO	
CV545070	1.828	no similarity	-1.815
JCVI_429	1.828	moderately similar to (272)AT3G22970| Symbols: | similar to unknown protein [Arabidopsis thaliana] (TAIR:AT4G14620.1); similar to u	
JCVI_14247	1.828	moderately similar to (285)AT1G35460| Symbols: | basic helix-loop-helix (bHLH) family protein | chr1:13040070-13041885 FORWARD	
JCVI_4323	1.828	moderately similar to (311)AT1G71480| Symbols: | nuclear transport factor 2 (NTF2) family protein | chr1:26935741-26936748 FORWA	
JCVI_3580	1.828	moderately similar to (311)AT3G11660| Symbols: NHL1 | NHL1 (NDR1/HIN1-like 1) | chr3:3679037-3679666 REVERSE no original des	-1.911
DN963737	1.828	no similarity	
JCVI_6016	1.828	moderately similar to (212)AT4G21980| Symbols: ATG8A, APG8A | APG8A (autophagy 8A) | chr4:11655880-11656821 FORWARD no	
DY017400	1.828	weakly similar to (185)AT3G10490| Symbols: ANAC051, ANAC052 | ANAC051/ANAC052 (Arabidopsis NAC domain containing protei	
EX113549	1.828	moderately similar to (226)AT2G35910| Symbols: | zinc finger (C3HC4-type RING finger) family protein | chr2:15080304-15080957 REV	
JCVI_7756	1.828	moderately similar to (427)AT4G30960| Symbols: SIP3, SNRK3.14, CIPK6 | CIPK6 (CBL-INTERACTING PROTEIN KINASE 6); kinas	
JCVI_446	1.827	no original description	
JCVI_1519	1.827	moderately similar to (434)AT3G58610| Symbols: | ketol-acid reductoisomerase | chr3:21682538-21685616 FORWARDmoderately simila	
CX191743	1.827	no similarity	
EX142108	1.827	moderately similar to (403)AT5G47750| Symbols: | protein kinase, putative | chr5:19357173-19359090 REVERSEmoderately similar to (	
JCVI_16763	1.827	moderately similar to (206)AT1G63720| Symbols: | similar to hydroxyproline-rich glycoprotein family protein [Arabidopsis thaliana] (TA	
JCVI_13108	1.827	moderately similar to (391)AT1G32850| Symbols: | ubiquitin carboxyl-terminal hydrolase family protein | chr1:11902609-11906880 FOR	
EV227476	1.827	moderately similar to (363)AT4G28660| Symbols: PSB28 | PSB28 (PHOTOSYSTEM II REACTION CENTER PSB28 PROTEIN) | chr4:	
JCVI_20324	1.827	moderately similar to (415)AT5G02470| Symbols: DPA | DPA; transcription factor | chr5:542560-544421 REVERSE no original descriptio	
JCVI_34417	1.827	moderately similar to (414)AT4G16820| Symbols: | lipase class 3 family protein | chr4:9467582-9469135 FORWARD no original descript	
JCVI_32699	1.826	moderately similar to (224)AT5G62790| Symbols: PDE129, DXR | DXR (1-DEOXY-D-XYLULOSE 5-PHOSPHATE REDUCTOISOME	
ES977177	1.826	no similarity	
EE519520	1.826	moderately similar to (231)AT3G08550| Symbols: ELD1, ABI8, KOB1 | ABI8/ELD1/KOB1 (KOBITO) | chr3:2596519-2599521 FORWA	-2.054
JCVI_25653	1.826	moderately similar to (324)AT3G23920| Symbols: BMY7, TR-BAMY, BAM1 | BAM1/BMY7/TR-BAMY (BETA-AMYLASE 1); beta-ar	
JCVI_22346	1.826	moderately similar to (461)AT5G18520| Symbols: | similar to unknown protein [Arabidopsis thaliana] (TAIR:AT3G09570.1); similar to u	
JCVI_911	1.826	moderately similar to (266)AT5G66090| Symbols: | similar to unnamed protein product [Vitis vinifera] (GB:CAO23263.1); contains doma	
JCVI_30512	1.826	no original description	
JCVI_22222	1.826	moderately similar to (244)AT1G08980| Symbols: ATTOC64-I, AMI1, ATAMI1 | ATAMI1 (AMIDASE-LIKE PROTEIN 1); amidase | ch	
EV107068	1.826	very weakly similar to (80.9)AT1G79600| Symbols: | ABC1 family protein | chr1:29954998-29957409 REVERSE [21478]	
JCVI_34255	1.826	weakly similar to (197)AT3G10770| Symbols: | nucleic acid binding | chr3:3372466-3374031 REVERSE no original description	
JCVI_20479	1.826	moderately similar to (328)AT1G64970| Symbols: TMT1, VTE4, G-TMT | G-TMT (GAMMA-TOCOPHEROL METHYLTRANSFERAS	
JCVI_12115	1.825	moderately similar to (436)AT2G29400| Symbols: PP1-AT, TOPP1 | TOPP1 (TYPE ONE PROTEIN PHOSPHATASE 1); protein serine/	
ES268957	1.825	moderately similar to (288)AT5G16120| Symbols: | hydrolase, alpha/beta fold family protein | chr5:5265823-5267778 FORWARD [21032	
JCVI_1539	1.825	moderately similar to (441)AT1G11680| Symbols: EMB1738, CYP51A2, CYP51, CYP51G1 | CYP51G1 (CYTOCHROME P450 51); oxy	
JCVI_20471	1.825	moderately similar to (481)AT1G48270| Symbols: GCR1 | GCR1 (G-PROTEIN-COUPLED RECEPTOR 1) | chr1:17831982-17833882 R	
EV104184	1.825	moderately similar to (261)AT1G19710| Symbols: | glycosyl transferase family 1 protein | chr1:6814911-6816707 FORWARD [21477] 39	
EX025050	1.825	weakly similar to (180)AT5G53420| Symbols: | similar to unknown protein [Arabidopsis thaliana] (TAIR:AT4G27900.1); similar to unkno	1.594
EE457412	1.825	very weakly similar to (83.2)AT5G65870| Symbols: ATPSK5 | ATPSK5 (PHYTOSULFOKINE 5 PRECURSOR); growth factor | chr5:263	
H07718	1.825	no similarity	
EE551319	1.825	weakly similar to (127)AT1G67035| Symbols: | similar to unknown protein [Arabidopsis thaliana] (TAIR:AT5G38300.1); similar to hypot	
ES940965	1.824	moderately similar to (385)AT1G26900| Symbols: | pentatricopeptide (PPR) repeat-containing protein | chr1:9319743-9321461 REVERSI	
ES941526	1.824	moderately similar to (333)AT5G02910| Symbols: | F-box family protein | chr5:677118-678905 FORWARD [21391]	
EV226207	1.824	moderately similar to (235)AT1G67440| Symbols: EMB1688 | EMB1688 (EMBRYO DEFECTIVE 1688); GTP binding / GTPase | chr1:2	
JCVI_6077	1.824	moderately similar to (358)AT3G15000| Symbols: | Identical to Uncharacterized mitochondrial protein At3g15000 [Arabidopsis Thaliana]	
JCVI_8696	1.824	highly similar to (735)AT1G53210| Symbols: | sodium/calcium exchanger family protein / calcium-binding EF hand family protein | chr1:	
EV029867	1.824	moderately similar to (313)AT4G29860| Symbols: EMB2757, TAN | EMB2757/TAN (EMBRYO DEFECTIVE 2757); nucleotide binding	
EE533957	1.823	very weakly similar to (87.4)AT5G26731| Symbols: | unknown protein | chr5:9295679-9295978 FORWARD [20150] 14 485 485	
JCVI_5747	1.823	weakly similar to (110)AT4G35500| Symbols: | protein kinase family protein | chr4:16857480-16859412 FORWARD no original descripti	
JCVI_8105	1.823	moderately similar to (216)AT4G05470| Symbols: | F-box family protein (FBL21) | chr4:2763253-2767954 REVERSE no original descrip	
EE455773	1.823	moderately similar to (395)AT4G37640| Symbols: ACA2 | ACA2 (CALCIUM ATPASE 2); calmodulin binding | chr4:17683219-1768680	
JCVI_7365	1.823	moderately similar to (353)AT3G11800| Symbols: | similar to unknown protein [Arabidopsis thaliana] (TAIR:AT3G44150.1); similar to e	
JCVI_27693	1.823	weakly similar to (159)AT4G14815| Symbols: | protease inhibitor/seed storage/lipid transfer protein (LTP) family protein | chr4:8505117-	
JCVI_1995	1.823	weakly similar to (136)AT1G67856| Symbols: | protein binding / zinc ion binding | chr1:25446149-25446550 FORWARD no original desc	
JCVI_27037	1.823	moderately similar to (388)AT1G28200| Symbols: FIP1 | FIP1 (FH INTERACTING PROTEIN 1) | chr1:9850382-9852287 REVERSE no	
EV020373	1.822	weakly similar to (159)AT4G15940| Symbols: | fumarylacetoacetate hydrolase family protein | chr4:9038376-9040178 FORWARD [2144	
JCVI_2062	1.822	moderately similar to (414)AT3G33520| Symbols: ARP6, SUF3, ESD1, ATARP6 | ATARP6; structural constituent of cytoskeleton | chr3:	
JCVI_14706	1.822	moderately similar to (367)AT2G16370| Symbols: THY-1 | THY-1 (THYMIDYLATE SYNTHASE 1) | chr2:7089120-7091415 REVERSI	
JCVI_31528	1.822	very weakly similar to (88.2)AT1G15870| Symbols: | mitochondrial glycoprotein family protein / MAM33 family protein | chr1:5457147-5	
EX044264	1.822	very weakly similar to (81.6)AT4G04460| Symbols: | aspartyl protease family protein | chr4:2225230-2227744 FORWARD [21811]	
EX114040	1.822	moderately similar to (359)AT4G21410| Symbols: | protein kinase family protein | chr4:11402474-11405036 REVERSE [21827]	
JCVI_11975	1.822	moderately similar to (384)AT5G42030| Symbols: ABIL4 | ABIL4 (ABL INTERACTOR-LIKE PROTEIN 4) | chr5:16828746-16830315 I	
EV125986	1.822	weakly similar to (177)AT5G16150| Symbols: PGLCT, GLT1 | GLT1/PGLCT (GLUCOSE TRANSPORTER 1); carbohydrate transmemb	
JCVI_9249	1.821	moderately similar to (376)AT5G01590| Symbols: | similar to unnamed protein product [Vitis vinifera] (GB:CAO63840.1) | chr5:224248-	
JCVI_7971	1.821	highly similar to (760)AT4G31210| Symbols: | DNA topoisomerase family protein | chr4:15165286-15172540 FORWARD no original des	
JCVI_33010	1.821	weakly similar to (139)AT3G18295| Symbols: | similar to unknown protein [Arabidopsis thaliana] (TAIR:AT1G48770.1); similar to unnar	
JCVI_29719	1.821	weakly similar to (164)AT5G04160| Symbols: | phosphate translocator-related | chr5:1143034-1144778 REVERSE no original description	1.929
JCVI_10146	1.821	no original description	
EV191799	1.821	weakly similar to (167)AT1G14250| Symbols: | nucleoside phosphatase family protein / GDA1/CD39 family protein | chr1:4868670-4871	
AM056896	1.821	no similarity	
EE463008	1.820	weakly similar to (164)AT2G40980| Symbols: | ATP binding / protein kinase | chr2:17109168-17111521 FORWARD [20171]	
JCVI_11329	1.820	moderately similar to (393)AT1G47128| Symbols: RD21A, RD21 | RD21 (RESPONSIVE TO DEHYDRATION 21); cysteine-type peptida	
CX279118	1.820	weakly similar to (121)AT3G27670| Symbols: RST1 | RST1 (RESURRECTION1); binding | chr3:10246575-10254395 FORWARD [1681	
JCVI_33117	1.820	highly similar to (808)AT1G77130| Symbols: PGSIP2 | PGSIP2 (PLANT GLYCOGENIN-LIKE STARCH INITIATION PROTEIN 2); tra	
DY015647	1.820	weakly similar to (108)AT5G66760| Symbols: SDH1-1 | SDH1-1 (Succinate dehydrogenase 1-1) | chr5:26671002-26674450 FORWARDw	
EV179940	1.820	no similarity	
EV120117	1.819	weakly similar to (179)AT5G17330| Symbols: GAD1, GAD | GAD (Glutamate decarboxylase 1); calmodulin binding | chr5:5711143-5714	

JCVI_36811	1.819	moderately similar to (370)AT5G55950| Symbols: | transporter-related | chr5:22675637-22677479 REVERSE no original description	-1.443
JCVI_11160	1.819	moderately similar to (289)AT2G34280| Symbols: | S locus F-box-related / SLF-related | chr2:14477414-14478589 REVERSE no original	
JCVI_22459	1.819	moderately similar to (343)AT4G22670| Symbols: ATHIP1 | ATHIP1 (ARABIDOPSIS THALIANA HSP70-INTERACTING PROTEIN 1	
JCVI_39734	1.819	moderately similar to (282)AT1G22410| Symbols: | 2-dehydro-3-deoxyphosphoheptonate aldolase, putative / 3-deoxy-D-arabino-heptulos	
EV200593	1.819	weakly similar to (160)AT4G04850| Symbols: ATKEA3, KEA3 | KEA3 (K+ efflux antiporter 3); potassium:hydrogen antiporter | chr4:245	
JCVI_39882	1.819	weakly similar to (189)AT2G46870| Symbols: NGA1 | NGA1 (NGATHA1); transcription factor | chr2:19268382-19269314 FORWARD n	1.592
JCVI_18655	1.819	moderately similar to (208)AT1G21600| Symbols: PTAC6 | PTAC6 (PLASTID TRANSCRIPTIONALLY ACTIVE6) | chr1:7571503-757	
JCVI_2678	1.819	moderately similar to (287)AT5G67180| Symbols: | AP2 domain-containing transcription factor, putative | chr5:26819345-26821160 REV	
EX132502	1.819	moderately similar to (205)AT3G01970| Symbols: ATWRKY45, WRKY45 | WRKY45 (WRKY DNA-binding protein 45); transcription fa	
JCVI_992	1.818	moderately similar to (460)AT3G62600| Symbols: | DNAJ heat shock family protein | chr3:23162014-23164322 REVERSEweakly similar	-1.984
JCVI_22235	1.818	moderately similar to (456)AT5G57630| Symbols: SnRK3.4, CIPK21 | CIPK21 (CBL-INTERACTING PROTEIN KINASE 21); kinase | c	
EV110377	1.818	no similarity	
EV129459	1.818	weakly similar to (166)AT3G09630| Symbols: | 60S ribosomal protein L4/L1 (RPL4A) | chr3:2953818-2955449 FORWARD [21480]	
DW998133	1.818	weakly similar to (199)AT5G24290| Symbols: | integral membrane family protein | chr5:8263274-8265621 REVERSE [18977]	
JCVI_34510	1.818	no original description	
DY006708	1.818	moderately similar to (253)AT1G26100| Symbols: | cytochrome B561 family protein | chr1:9022703-9024068 REVERSE [18976]	
JCVI_19170	1.818	highly similar to (669)AT1G12380| Symbols: | similar to unknown protein [Arabidopsis thaliana] (TAIR:AT1G62870.1); similar to unnam	
EV151519	1.818	moderately similar to (327)AT2G16660| Symbols: | nodulin family protein | chr2:7226012-7228674 REVERSE [21483]	
EE443866	1.818	moderately similar to (249)AT1G27030| Symbols: | similar to unknown protein [Arabidopsis thaliana] (TAIR:AT1G27020.1); similar to u	
JCVI_30243	1.818	moderately similar to (213)AT3G49240| Symbols: EMB1796 | EMB1796 (EMBRYO DEFECTIVE 1796); binding | chr3:18267071-1826	
JCVI_14901	1.818	moderately similar to (335)AT5G46690| Symbols: BHLH071 | BHLH071 (BETA HLH PROTEIN 71); DNA binding / transcription factor	
EV105427	1.818	weakly similar to (119)AT5G53970| Symbols: | aminotransferase, putative | chr5:21927902-21929820 FORWARD [21478]	
JCVI_3546	1.817	moderately similar to (411)AT2G26070| Symbols: RTE1 | RTE1 (REVERSION-TO-ETHYLENE SENSITIVITY1) | chr2:11112819-1111	
EE557771	1.817	weakly similar to (112)AT5G66070| Symbols: | zinc finger (C3HC4-type RING finger) family protein | chr5:26439149-26440259 FORWA	
AM389689	1.817	weakly similar to (147)AT2G29730| Symbols: | UDP-glucoronosyl/UDP-glucosyl transferase family protein | chr2:12710729-12712132 F	
DY015794	1.817	no similarity	
H74963	1.817	very weakly similar to (92.8)AT3G43190| Symbols: SUS4 | SUS4; UDP-glycosyltransferase/ sucrose synthase/ transferase, transferring gly	
JCVI_16953	1.817	highly similar to (615)AT4G13050| Symbols: | acyl-(acyl carrier protein) thioesterase, putative / acyl-ACP thioesterase, putative / oleoyl-(	
JCVI_2248	1.816	moderately similar to (456)AT3G09300| Symbols: | oxysterol-binding family protein | chr3:2858074-2860468 FORWARD no original des	
EV111070	1.816	no similarity	
JCVI_17522	1.816	moderately similar to (243)AT3G11240| Symbols: | arginine-tRNA-protein transferase, putative / arginyltransferase, putative / arginyl-tRN	
JCVI_17843	1.816	very weakly similar to (93.2)AT1G78000| Symbols: SEL1, SULTR1;2 | SULTR1;2 (SULFATE TRANSPORTER 1;2) | chr1:29334783-293	
EV019254	1.816	no similarity	
EX131019	1.816	weakly similar to (127)AT4G11450| Symbols: | similar to unknown protein [Arabidopsis thaliana] (TAIR:AT1G63520.1); similar to hypot	
JCVI_25849	1.816	moderately similar to (231)AT5G15070| Symbols: | acid phosphatase/ oxidoreductase/ transition metal ion binding | chr5:4876901-488561	
ES914655	1.816	very weakly similar to (88.6)AT4G14965| Symbols: ATMAPR4 | ATMAPR4 (ARABIDOPSIS THALIANA MEMBRANE-ASSOCIATED	
JCVI_615	1.816	moderately similar to (350)AT2G20690| Symbols: | lumazine-binding family protein | chr2:8930423-8931702 FORWARD no original des	
EV051837	1.815	weakly similar to (124)AT4G19550| Symbols: | transcription activator/ transcription regulator/ zinc ion binding | chr4:10659424-1066110	
EE534619	1.815	no similarity	
JCVI_14351	1.815	moderately similar to (379)AT4G26650| Symbols: | RNA recognition motif (RRM)-containing protein | chr4:13445271-13447598 FORW	
EE479149	1.815	moderately similar to (240)AT5G43790| Symbols: | pentatricopeptide (PPR) repeat-containing protein | chr5:17609326-17610708 REVEF	
JCVI_36267	1.815	moderately similar to (434)AT2G17480| Symbols: ATMLO8, MLO8 | MLO8 (MILDEW RESISTANCE LOCUS O 8); calmodulin binding	
JCVI_9423	1.815	moderately similar to (469)AT3G05520| Symbols: | F-actin capping protein alpha subunit family protein | chr3:1598617-1601140 FORWA	
JCVI_38943	1.815	moderately similar to (499)AT3G21070| Symbols: ATNADK-1, NADK1 | NADK1 (NAD kinase 1); NAD+ kinase/ NADH kinase/ calmod	
CV432726	1.814	weakly similar to (114)AT3G05800| Symbols: | transcription factor | chr3:1727482-1728117 FORWARD [16490] 37 581 581	
L38210	1.814	no similarity	-1.707
JCVI_10936	1.814	moderately similar to (211)AT5G19930| Symbols: | integral membrane family protein | chr5:6737874-6739285 REVERSE no original des	
BG543402	1.814	no similarity	
EX089431	1.814	very weakly similar to (97.4)AT4G29530| Symbols: | 2,3-diketo-5-methylthio-1-phosphopentane phosphatase family | chr4:14496170-1449	
EV091988	1.814	moderately similar to (360)AT2G32530| Symbols: CSLB03, ATCSLB3, ATCSLB03 | ATCSLB03 (Cellulose synthase-like B3); transferas	
CD813923	1.814	weakly similar to (145)AT5G51380| Symbols: | F-box family protein | chr5:20893171-20895005 FORWARD [13977]	
JCVI_4221	1.814	highly similar to (625)AT1G80300| Symbols: ATNTT1 | ATNTT1; ATP:ADP antiporter | chr1:30196846-30199172 FORWARDhighly sir	
JCVI_38385	1.814	moderately similar to (468)AT5G48330| Symbols: | regulator of chromosome condensation (RCC1) family protein | chr5:19603215-19604	
BQ704241	1.813	moderately similar to (301)AT3G05580| Symbols: | serine/threonine protein phosphatase, putative | chr3:1618222-1619856 REVERSEmo	
JCVI_23964	1.813	moderately similar to (286)AT3G62240| Symbols: | zinc finger (C2H2 type) family protein | chr3:23044567-23047628 REVERSE no orig	
JCVI_41553	1.813	no original description	
CX191980	1.813	moderately similar to (237)AT4G25710| Symbols: | kelch repeat-containing F-box family protein | chr4:13098368-13099540 REVERSE [	
EV163063	1.813	no similarity	
ES916288	1.813	weakly similar to (150)AT5G23680| Symbols: | sterile alpha motif (SAM) domain-containing protein | chr5:7985576-7986463 REVERSE	-2.024
EV208237	1.813	no similarity	
RC_EV207814	1.813	no similarity	
EL590903	1.812	no similarity	
JCVI_32611	1.812	moderately similar to (283)AT5G37020| Symbols: ARF8 | ARF8 (AUXIN RESPONSE FACTOR 8) | chr5:14647381-14651146 FORWAR	
EE438913	1.812	no similarity	
JCVI_32566	1.812	highly similar to (712)AT1G30360| Symbols: ERD4 | ERD4 (EARLY-RESPONSIVE TO DEHYDRATION 4) | chr1:10715874-10718781	
JCVI_8662	1.812	weakly similar to (133)AT1G26270| Symbols: | phosphatidylinositol 3- and 4-kinase family protein | chr1:9089809-9091701 REVERSE n	
EX060534	1.812	weakly similar to (199)AT1G55915| Symbols: | zinc ion binding | chr1:20911328-20913094 FORWARD [21813]	1.560
JCVI_41498	1.812	no original description	
JCVI_1862	1.812	moderately similar to (409)AT2G14255| Symbols: | zinc ion binding | chr2:6044056-6047974 FORWARD no original description	
JCVI_2578	1.812	highly similar to (736)AT1G05055| Symbols: ATGTF2H2, GTF2H2 | ATGTF2H2/GTF2H2 (GENERAL TRANSCRIPTION FACTOR II	
JCVI_18419	1.812	highly similar to (683)AT2G16730| Symbols: BGAL13 | BGAL13 (beta-galactosidase 13); beta-galactosidase | chr2:7269068-7273187 RE	1.685
JCVI_11049	1.812	moderately similar to (320)AT1G73210| Symbols: | similar to unknown protein [Arabidopsis thaliana] (TAIR:AT1G17830.1); similar to u	
JCVI_33174	1.812	moderately similar to (449)AT3G63220| Symbols: | kelch repeat-containing F-box family protein | chr3:23368515-23369573 REVERSE n	
JCVI_9812	1.811	moderately similar to (238)AT5G18260| Symbols: | protein binding / zinc ion binding | chr5:6036228-6037297 REVERSE no original des	
EX133366	1.811	no similarity	1.386
EE565114	1.811	weakly similar to (142)AT3G55280| Symbols: | 60S ribosomal protein L23A (RPL23aB) | chr3:20511644-20512496 FORWARDweakly s	
JCVI_11916	1.811	moderately similar to (269)AT4G38940| Symbols: | kelch repeat-containing F-box family protein | chr4:18152842-18153954 FORWARD	
EV092412	1.811	no similarity	
JCVI_29400	1.810	nearly identical (1007)AT1G60140| Symbols: TPS10, ATTPS10 | ATTPS10 (TREHALOSE PHOSPHATE SYNTHASE); transferase, tran	
JCVI_30489	1.810	highly similar to (510)AT1G33800| Symbols: | similar to unknown protein [Arabidopsis thaliana] (TAIR:AT4G09990.1); similar to unkno	1.114
EE427453	1.810	weakly similar to (182)AT1G33110| Symbols: | MATE efflux family protein | chr1:12005064-12008020 FORWARD [20189]	1.969

JCVI_5999	1.810	moderately similar to (462)AT1G08410| Symbols: | GTP-binding family protein | chr1:2646304-2649103 FORWARD no original descript	
JCVI_42271	1.810	highly similar to (626)AT1G07510| Symbols: FTSH10 | FTSH10 (FtsH protease 10); ATPase | chr1:2305686-2309377 FORWARDmodera	
JCVI_8917	1.810	no original description	
JCVI_8263	1.810	moderately similar to (475)AT2G43000| Symbols: ANAC042 | ANAC042 (Arabidopsis NAC domain containing protein 42); transcription	
JCVI_3564	1.810	moderately similar to (458)AT2G39570| Symbols: | ACT domain-containing protein | chr2:16515041-16516819 FORWARD no original d	
EV056929	1.810	weakly similar to (125)AT3G06540| Symbols: | GDP dissociation inhibitor family protein / Rab GTPase activator family protein | chr3:20	
JCVI_462	1.810	moderately similar to (400)AT3G26780| Symbols: | catalytic | chr3:9849984-9851514 FORWARD no original description	
JCVI_13568	1.810	moderately similar to (415)AT1G12550| Symbols: | oxidoreductase family protein | chr1:4274647-4275829 FORWARD no original descri	
JCVI_24967	1.810	weakly similar to (112)AT2G43520| Symbols: ATTI2 | ATTI2 (ARABIDOPSIS THALIANA TRYPSIN INHIBITOR PROTEIN 2); trypsi	-2.117
JCVI_38778	1.810	moderately similar to (280)AT4G31430| Symbols: | similar to unnamed protein product [Vitis vinifera] (GB:CAO43934.1) | chr4:1524851	
JCVI_33971	1.809	moderately similar to (219)AT1G47210| Symbols: CYCA3;2 | CYCA3;2; cyclin-dependent protein kinase | chr1:17303476-17305024 FOR	
EE483539	1.809	no similarity	
JCVI_13777	1.809	moderately similar to (496)AT1G78580| Symbols: TPS1, ATTPS1 | ATTPS1 (TREHALOSE-6-PHOSPHATE SYNTHASE); transferase,	
EE455011	1.809	weakly similar to (185)AT3G25805| Symbols: | similar to unnamed protein product [Vitis vinifera] (GB:CAO42121.1) | chr3:9427591-942	
JCVI_16361	1.809	moderately similar to (399)AT2G33255| Symbols: | hydrolase | chr2:14105872-14107435 FORWARD no original description	
EX063129	1.809	moderately similar to (409)AT4G20170| Symbols: | similar to unknown protein [Arabidopsis thaliana] (TAIR:AT5G44670.1); similar to C	1.711
RC_EV080004	1.809	no similarity	-2.919
JCVI_39053	1.809	moderately similar to (444)AT4G11120| Symbols: | translation elongation factor Ts (EF-Ts), putative | chr4:6778062-6779930 FORWAR	
JCVI_8120	1.808	moderately similar to (326)AT5G02410| Symbols: | DIE2/ALG10 family | chr5:517317-519632 REVERSE no original description	
JCVI_5803	1.808	moderately similar to (283)AT1G03650| Symbols: | GCN5-related N-acetyltransferase (GNAT) family protein | chr1:910248-911102 REV	-1.348
EE420587	1.808	moderately similar to (355)AT1G17680| Symbols: | transcription factor-related | chr1:6076381-6082153 FORWARD [20149]	
ES933197	1.808	no similarity	
CX271362	1.808	very weakly similar to (80.1)AT2G13290| Symbols: | glycosyl transferase family 17 protein | chr2:5519140-5520225 FORWARD [16815]	-3.741
H07503	1.808	no similarity	
JCVI_35274	1.808	no original description	-2.990
CV546030	1.807	very weakly similar to (92.4)AT5G63130| Symbols: | octicosapeptide/Phox/Bem1p (PB1) domain-containing protein | chr5:25340416-253	-3.020
JCVI_39174	1.807	moderately similar to (222)AT4G02740| Symbols: | Identical to F-box protein At4g02740 [Arabidopsis Thaliana] (GB:Q0WRC9;GB:Q68	1.860
JCVI_10600	1.807	moderately similar to (419)AT5G16880| Symbols: | VHS domain-containing protein / GAT domain-containing protein | chr5:5549661-555	
EX108531	1.807	weakly similar to (181)AT3G02220| Symbols: | similar to hypothetical protein [Cleome spinosa] (GB:ABD96929.1); contains domain PT	
JCVI_12346	1.807	weakly similar to (152)AT1G23220| Symbols: | dynein light chain type 1 family protein | chr1:8242603-8244125 FORWARD no original	
JCVI_5852	1.807	moderately similar to (298)AT1G73760| Symbols: | zinc finger (C3HC4-type RING finger) family protein | chr1:27743027-27744822 RE	
JCVI_4202	1.807	moderately similar to (491)AT1G59580| Symbols: MPK2, ATMPK2 | ATMPK2 (MITOGEN-ACTIVATED PROTEIN KINASE HOMOI	
EV142925	1.807	no similarity	
EX125129	1.807	no similarity	
JCVI_21412	1.806	highly similar to (599)AT3G09840| Symbols: ATCDC48, CDC48A, CDC48 | CDC48 (CELL DIVISION CYCLE 48); ATPase | chr3:3019	
JCVI_24780	1.806	moderately similar to (376)AT1G76120| Symbols: | tRNA pseudouridine synthase family protein | chr1:28564118-28565188 REVERSE n	
EE411755	1.806	weakly similar to (128)AT1G71240| Symbols: | similar to unknown protein [Arabidopsis thaliana] (TAIR:AT1G48840.1); similar to Os01	
JCVI_16269	1.806	moderately similar to (463)AT3G26770| Symbols: | short-chain dehydrogenase/reductase (SDR) family protein | chr3:9846731-9848316 F	
EV053662	1.806	no similarity	
JCVI_16203	1.806	moderately similar to (448)AT1G27461| Symbols: | similar to unnamed protein product [Vitis vinifera] (GB:CAO61483.1) | chr1:9537935	
JCVI_3138	1.806	moderately similar to (391)AT4G26850| Symbols: VTC2 | VTC2 (VITAMIN C DEFECTIVE 2) | chr4:13499268-13501151 REVERSE no	
EV064322	1.805	no similarity	
EV221548	1.805	moderately similar to (407)AT4G34450| Symbols: | coatomer gamma-2 subunit, putative / gamma-2 coat protein, putative / gamma-2 CO	
EE408106	1.805	moderately similar to (238)AT4G27020| Symbols: | similar to unknown protein [Arabidopsis thaliana] (TAIR:AT1G70160.1); similar to u	
JCVI_34755	1.805	moderately similar to (350)AT5G45700| Symbols: | NLI interacting factor (NIF) family protein | chr5:18554605-18555423 FORWARD n	
JCVI_31701	1.805	moderately similar to (241)AT2G33150| Symbols: PED1, KAT2 | KAT2/PED1 (PEROXISOME DEFECTIVE 1); acetyl-CoA C-acyltrans	
ES966108	1.805	no similarity	
JCVI_34931	1.805	no original description	
JCVI_11079	1.805	highly similar to (575)AT2G32730| Symbols: | 26S proteasome regulatory subunit, putative | chr2:13887266-13892541 FORWARD no or	
JCVI_1091	1.804	moderately similar to (336)AT2G32260| Symbols: | cholinephosphate cytidylyltransferase, putative / phosphorylcholine transferase, putati	
JCVI_12141	1.804	highly similar to (510)AT4G27830| Symbols: | glycosyl hydrolase family 1 protein | chr4:13861800-13864495 REVERSEmoderately simi	2.790
JCVI_14195	1.804	moderately similar to (235)AT4G26690| Symbols: MRH5, SHV3 | MRH5/SHV3 (morphogenesis of root hair 5); glycerophosphodiester p	
ES952234	1.804	weakly similar to (102)AT2G37920| Symbols: EMB1513 | EMB1513 (EMBRYO DEFECTIVE 1513); copper ion transmembrane transpo	
JCVI_28639	1.804	highly similar to (676)AT3G21420| Symbols: | oxidoreductase, 2OG-Fe(II) oxygenase family protein | chr3:7541585-7543227 FORWAR	
EE432378	1.804	very weakly similar to (100)AT4G11655| Symbols: | transmembrane protein, putative | chr4:7038584-7039753 FORWARD [20151] 1 393	
AM061394	1.804	no similarity	
AT000610	1.804	no similarity	
DY008092	1.804	weakly similar to (108)AT4G18570| Symbols: | proline-rich family protein | chr4:10231450-10234545 FORWARD [18972] 1 494 519	
JCVI_14732	1.803	moderately similar to (226)AT1G76410| Symbols: ATL8 | ATL8; protein binding / zinc ion binding | chr1:28673809-28674366 FORWAR	
EX071872	1.803	moderately similar to (251)AT3G26750| Symbols: | similar to unnamed protein product [Vitis vinifera] (GB:CAO48317.1) | chr3:9841376	
JCVI_7604	1.803	weakly similar to (155)AT5G25540| Symbols: CID6 | CID6 (CTC-Interacting Domain 6); protein binding | chr5:8891773-8892365 REVE	
EE449499	1.803	no similarity	2.683
JCVI_30440	1.803	moderately similar to (420)AT4G04970| Symbols: ATGSL01, GSL01, ATGSL1 | ATGSL1 (GLUCAN SYNTHASE LIKE-1); 1,3-beta-glu	-1.806
EV151402	1.802	moderately similar to (284)AT4G32600| Symbols: | zinc finger (C3HC4-type RING finger) family protein | chr4:15724016-15725743 FO	
JCVI_3805	1.802	weakly similar to (187)AT3G03640| Symbols: GLUC | GLUC (Beta-glucosidase homolog); hydrolase, hydrolyzing O-glycosyl compounds	
JCVI_493	1.802	no original description	
EX021057	1.802	weakly similar to (160)AT1G06430| Symbols: FTSH8 | FTSH8 (FtsH protease 8); ATP-dependent peptidase/ ATPase/ metallopeptidase/ z	
JCVI_7518	1.802	no original description	
EX135976	1.801	weakly similar to (194)AT2G47320| Symbols: | peptidyl-prolyl cis-trans isomerase cyclophilin-type family protein | chr2:19434781-19435	
EV091356	1.801	weakly similar to (107)AT2G45660| Symbols: SOC1, AGL20 | AGL20 (AGAMOUS-LIKE 20); transcription factor | chr2:18814873-1881	
EX100138	1.801	no similarity	
JCVI_13144	1.801	moderately similar to (301)AT1G08450| Symbols: CRT3 | CRT3 (CALRETICULIN 3); calcium ion binding | chr1:2668005-2671797 REV	
JCVI_29780	1.801	weakly similar to (141)AT5G25590| Symbols: | similar to unknown protein [Arabidopsis thaliana] (TAIR:AT1G52320.3); similar to unkn	
EV041816	1.801	weakly similar to (127)AT1G53100| Symbols: | acetylglucosaminyltransferase | chr1:19790658-19792172 REVERSE [21442]	
EV106227	1.800	no similarity	
CD823848	1.800	weakly similar to (129)AT3G07560| Symbols: APM2, PEX13 | APM2/PEX13 (ABERRANT PEROXISOME MORPHOLOGY 2); protein	
JCVI_14501	1.800	weakly similar to (168)AT3G15760| Symbols: | similar to unknown protein [Arabidopsis thaliana] (TAIR:AT1G52565.1); similar to unna	
JCVI_288	1.800	moderately similar to (416)AT5G20720| Symbols: CPN10, CHCPN10, ATCPN21, CPN21, CPN20 | CPN20 (CHAPERONIN 20); calmod	
ES988053	1.800	weakly similar to (110)AT1G20010| Symbols: TUB5 | TUB5 (tubulin beta-5 chain) | chr1:6938024-6940472 REVERSEweakly similar to (	
JCVI_25609	1.800	highly similar to (526)AT3G05990| Symbols: | leucine-rich repeat family protein | chr3:1797122-1799738 REVERSE no original descripti	
JCVI_23876	1.800	no original description	

JCVI_4158	1.800	moderately similar to (307)AT5G67260| Symbols: CYCD3;2 | CYCD3;2 (CYCLIN D3;2); cyclin-dependent protein kinase | chr5:2685353	
JCVI_8071	1.800	moderately similar to (216)AT1G34270| Symbols: | exostosin family protein | chr1:12492549-12494492 REVERSE no original descriptio	
EV044992	1.800	weakly similar to (102)AT2G42030| Symbols: | zinc finger (C3HC4-type RING finger) family protein | chr2:17546147-17547424 REVER	
EV069575	1.800	no similarity	
JCVI_37945	1.800	moderately similar to (406)AT1G16490| Symbols: AtMYB58 | AtMYB58 (myb domain protein 58); DNA binding / transcription factor | c	-2.026
JCVI_35571	1.800	weakly similar to (164)AT1G23550| Symbols: SRO2 | SRO2 (SIMILAR TO RCD ONE 2); NAD+ ADP-ribosyltransferase | chr1:8350901-	
JCVI_388	1.800	moderately similar to (298)AT5G21430| Symbols: | DNAJ heat shock N-terminal domain-containing protein | chr5:7222297-7223403 FOI	
JCVI_27211	1.799	highly similar to (692)AT5G67360| Symbols: ARA12 | ARA12; subtilase | chr5:26889418-26891691 REVERSE no original description	
JCVI_41433	1.799	moderately similar to (419)AT4G25110| Symbols: ATMC2 | ATMC2 (METACASPASE 2); caspase | chr4:12887748-12889963 REVERS	
AM388435	1.799	moderately similar to (338)AT3G07890| Symbols: | RabGAP/TBC domain-containing protein | chr3:2516645-2518790 REVERSE [20118	
JCVI_28358	1.799	moderately similar to (322)AT3G61350| Symbols: SKIP4 | SKIP4 (SKP1 INTERACTING PARTNER 4) | chr3:22714178-22715349 FOR	
EE454545	1.799	moderately similar to (215)AT3G02250| Symbols: | similar to unknown protein [Arabidopsis thaliana] (TAIR:AT5G15740.1); similar to h	
EE559509	1.799	no similarity	
JCVI_37537	1.799	moderately similar to (284)AT1G24400| Symbols: AATL2, LHT2 | LHT2 (LYSINE HISTIDINE TRANSPORTER 2); amino acid transme	
EE558568	1.799	moderately similar to (332)AT4G26260| Symbols: MIOX4 | MIOX4 (MYO-INOSITOL OXYGENASE 4) | chr4:13297948-13300155 FOI	
EV124150	1.799	very weakly similar to (87.8)AT5G42650| Symbols: CYP74A, AOS | AOS (ALLENE OXIDE SYNTHASE); hydro-lyase/ oxygen binding |	
JCVI_37656	1.798	no original description	
JCVI_4206	1.798	no original description	
JCVI_37044	1.798	weakly similar to (176)AT3G47060| Symbols: FTSH7 | FTSH7 (FtsH protease 7); ATP-dependent peptidase/ ATPase/ metallopeptidase | c	
JCVI_27455	1.798	highly similar to (588)AT2G36800| Symbols: UGT73C5, DOGT1 | DOGT1 (DON-GLUCOSYLTRANSFERASE); UDP-glycosyltransfera	
DN960621	1.798	no similarity	
JCVI_27521	1.798	highly similar to (798)AT4G20830| Symbols: | FAD-binding domain-containing protein | chr4:11155497-11157119 FORWARD no origina	
JCVI_38227	1.798	moderately similar to (428)AT1G02880| Symbols: TPK1 | TPK1 (THIAMIN PYROPHOSPHOKINASE1) | chr1:643063-643921 REVERS	
JCVI_13630	1.798	moderately similar to (395)AT1G21760| Symbols: ATFBP7, FBP7 | F-box family protein | chr1:7649313-7651602 FORWARD no original	
JCVI_39825	1.798	weakly similar to (115)AT5G53050| Symbols: | hydrolase, alpha/beta fold family protein | chr5:21527760-21530658 REVERSE no origina	
EV227283	1.797	no similarity	2.923
EV092256	1.797	no similarity	
EX052765	1.797	no similarity	
JCVI_11692	1.797	weakly similar to (122)AT5G56170| Symbols: | similar to unknown protein [Arabidopsis thaliana] (TAIR:AT4G26466.1); similar to unna	
JCVI_33487	1.797	no original description	
EX125776	1.797	moderately similar to (435)AT5G40800| Symbols: | similar to unknown protein [Arabidopsis thaliana] (TAIR:AT3G27250.1); similar to u	
JCVI_19532	1.796	moderately similar to (403)AT1G65730| Symbols: YSL7 | YSL7 (YELLOW STRIPE LIKE 7); oligopeptide transporter | chr1:24446302-2	
JCVI_19983	1.796	moderately similar to (386)AT2G03800| Symbols: GEK1 | GEK1 (GEKO1) | chr2:1156779-1158692 FORWARD no original description	
JCVI_22705	1.796	weakly similar to (142)AT2G34430| Symbols: LHCB1.4, LHB1B1 | LHB1B1 (Photosystem II light harvesting complex gene 1.4); chlorop	
EE551766	1.796	moderately similar to (350)AT5G43920| Symbols: | transducin family protein / WD-40 repeat family protein | chr5:17690624-17692857 F	
EV001402	1.796	no similarity	
BQ705001	1.796	moderately similar to (248)AT4G11840| Symbols: PLDGAMMA3 | PLDGAMMA3 (phospholipase D gamma 3); phospholipase D | chr4:7	
EX132140	1.796	moderately similar to (484)AT5G13060| Symbols: | armadillo/beta-catenin repeat family protein / BTB/POZ domain-containing protein | c	
EV111940	1.795	very weakly similar to (96.3)AT3G57680| Symbols: | peptidase S41 family protein | chr3:21392031-21394606 FORWARD [21478] 1 452	
CX192473	1.795	weakly similar to (126)AT3G45600| Symbols: TET3 | TET3 (TETRASPANIN3) | chr3:16744958-16746839 REVERSE [16807]	
AM060041	1.795	weakly similar to (187)AT1G53210| Symbols: | sodium/calcium exchanger family protein / calcium-binding EF hand family protein | chr1	
JCVI_25020	1.795	moderately similar to (398)AT5G14880| Symbols: | potassium transporter, putative | chr5:4814247-4817670 FORWARDmoderately simil	
EV041638	1.794	moderately similar to (313)AT3G26220| Symbols: CYP71B3 | CYP71B3 (cytochrome P450, family 71, subfamily B, polypeptide 3); oxyg	
ES990289	1.794	moderately similar to (211)AT4G33210| Symbols: | F-box family protein (FBL15) | chr4:16015974-16020700 REVERSE [21425]	
JCVI_23340	1.794	moderately similar to (484)AT2G02220| Symbols: ATPSKR1, PSKR1 | ATPSKR1/PSKR1 (PHYTOSULFOKIN RECEPTOR 1); ATP bir	
AT000535	1.794	no similarity	
JCVI_3308	1.794	moderately similar to (446)AT3G45770| Symbols: | oxidoreductase, zinc-binding dehydrogenase family protein | chr3:16816738-1681875	
DY024036	1.793	moderately similar to (372)AT5G48840| Symbols: PANC | PANC (Arabidopsis homolog of bacterial panC); pantoate-beta-alanine ligase |	
EV100559	1.793	weakly similar to (165)AT2G14620| Symbols: | xyloglucan:xyloglucosyl transferase, putative / xyloglucan endotransglycosylase, putative	
EX096590	1.793	moderately similar to (204)AT1G68440| Symbols: | similar to unknown protein [Arabidopsis thaliana] (TAIR:AT1G25400.1); similar to u	-1.551
EV212594	1.793	moderately similar to (248)AT1G13030| Symbols: | sphere organelles protein-related | chr1:4444282-4447276 REVERSE [21491]	-1.650
EE420017	1.793	weakly similar to (144)AT2G45950| Symbols: ASK20 | ASK20 (ARABIDOPSIS SKP1-LIKE 20) | chr2:18911746-18914040 REVERSE [	
JCVI_26432	1.793	moderately similar to (220)AT4G22290| Symbols: | ubiquitin thiolesterase | chr4:11783211-11785742 REVERSE no original description	
JCVI_15645	1.793	moderately similar to (230)AT1G55300| Symbols: TAF7 | TAF7 (TBP-ASSOCIATED FACTOR 7); general RNA polymerase II transcript	
EV105926	1.793	weakly similar to (116)AT1G21400| Symbols: | 2-oxoisovalerate dehydrogenase, putative / 3-methyl-2-oxobutanoate dehydrogenase, puta	
EV042079	1.793	no similarity	
JCVI_30590	1.793	moderately similar to (327)AT2G16370| Symbols: THY-1 | THY-1 (THYMIDYLATE SYNTHASE 1) | chr2:7089120-7091415 REVERSI	
EV190862	1.793	moderately similar to (239)AT1G63740| Symbols: | disease resistance protein (TIR-NBS-LRR class), putative | chr1:23649188-23652470	
JCVI_26867	1.793	moderately similar to (218)AT5G54870| Symbols: | similar to unknown protein [Arabidopsis thaliana] (TAIR:AT1G70160.1); similar to u	2.028
JCVI_15926	1.793	moderately similar to (357)AT1G06110| Symbols: SKIP16 | SKIP16 (SKP1/ASK-INTERACTING PROTEIN 16); protein binding | chr1:1	
CX195612	1.793	very weakly similar to (84.7)AT5G62700| Symbols: ATGCP3, TUB3 | TUB3 (TUBULIN BETA-3); structural molecule | chr5:25201727-2	
JCVI_36279	1.792	moderately similar to (327)AT3G14790| Symbols: RHM3 | RHM3 (RHAMNOSE BIOSYNTHESIS 3); catalytic | chr3:4964798-4966882	
JCVI_1305	1.792	moderately similar to (272)AT3G43720| Symbols: | protease inhibitor/seed storage/lipid transfer protein (LTP) family protein | chr3:15620	
DY017571	1.792	moderately similar to (311)AT3G19950| Symbols: | zinc finger (C3HC4-type RING finger) family protein | chr3:6942859-6943845 FORW	
JCVI_602	1.792	no original description	
EL591991	1.792	no similarity	
ES264850	1.791	no similarity	
JCVI_9257	1.791	moderately similar to (207)AT3G62410| Symbols: CP12, CP12-2 | CP12-2 | chr3:23101982-23102377 FORWARD no original description	
JCVI_28763	1.791	weakly similar to (110)AT5G50460| Symbols: | protein transport protein SEC61 gamma subunit, putative | chr5:20569394-20569735 REV	-1.646
JCVI_17153	1.791	moderately similar to (216)AT1G18570| Symbols: AtMYB51, BW51A, BW51B, MYB51 | MYB51 (MYB DOMAIN PROTEIN 51); DNA	
ES908104	1.791	weakly similar to (192)AT2G18010| Symbols: | auxin-responsive family protein | chr2:7840984-7841322 FORWARDvery weakly similar	
CD835203	1.790	moderately similar to (332)AT1G16250| Symbols: | kelch repeat-containing F-box family protein | chr1:5557208-5558691 FORWARD [1	
JCVI_12891	1.790	weakly similar to (105)AT3G48660| Symbols: | similar to unknown protein [Arabidopsis thaliana] (TAIR:AT5G63500.1); similar to hypot	
CV546044	1.790	no similarity	
JCVI_921	1.790	moderately similar to (425)AT4G30530| Symbols: | defense-related protein, putative | chr4:14920611-14922292 FORWARD no original d	1.128
JCVI_20909	1.789	moderately similar to (216)AT4G33565| Symbols: | zinc finger (C3HC4-type RING finger) family protein | chr4:16137312-16137926 FOI	1.262
JCVI_14	1.789	moderately similar to (222)AT3G18780| Symbols: DER1, LSR2, ENL2, ACT2 | ACT2 (ACTIN 2); structural constituent of cytoskeleton |	
EV020973	1.789	moderately similar to (407)AT3G25100| Symbols: CDC45 | CDC45 (CELL DIVISION CYCLE 45) | chr3:9144299-9146089 FORWARD	1.760
JCVI_33303	1.789	weakly similar to (137)AT2G24100| Symbols: | similar to unknown protein [Arabidopsis thaliana] (TAIR:AT4G30780.1); similar to unna	
EX096964	1.789	moderately similar to (266)AT5G08240| Symbols: | similar to unknown protein [Arabidopsis thaliana] (TAIR:AT5G23160.1) | chr5:26513	
EE452455	1.789	moderately similar to (318)AT3G54170| Symbols: FIP37, ATFIP37 | ATFIP37 (ARABIDOPSIS THALIANA FKBP12 INTERACTING P	

JCVI_18347	1.789	moderately similar to (300)AT5G02530| Symbols: | RNA and export factor-binding protein, putative | chr5:564330-565774 REVERSE no	1.437
JCVI_36970	1.789	no original description	
JCVI_42616	1.789	highly similar to (585)AT5G51690| Symbols: ACS12 | ACS12 (1-Amino-cyclopropane-1-carboxylate synthase 12); 1-aminocyclopropane-	
JCVI_15871	1.789	moderately similar to (390)AT4G12590| Symbols: | similar to hypothetical protein [Vitis vinifera] (GB:CAN72892.1); contains InterPro d	
JCVI_12	1.789	moderately similar to (383)AT2G40100| Symbols: LHCB4.3 | LHCB4.3 (LIGHT HARVESTING COMPLEX PSII); chlorophyll binding |	
JCVI_13667	1.788	moderately similar to (249)AT5G24960| Symbols: CYP71A14 | CYP71A14 (cytochrome P450, family 71, subfamily A, polypeptide 14); c	
CX192663	1.788	no similarity	
EE530873	1.788	moderately similar to (202)AT5G01160| Symbols: | e-cadherin binding protein-related | chr5:54279-55726 FORWARD [20175]	
JCVI_37019	1.788	highly similar to (690)AT1G74400| Symbols: | pentatricopeptide (PPR) repeat-containing protein | chr1:27967614-27969002 FORWARD	
CN730073	1.788	moderately similar to (435)AT4G30360| Symbols: CNGC17, ATCNGC17 | ATCNGC17 (cyclic nucleotide gated channel 17); calmodulin	
JCVI_8889	1.788	weakly similar to (125)AT3G21070| Symbols: ATNADK-1, NADK1 | NADK1 (NAD kinase 1); NAD+ kinase/ NADH kinase/ calmodulin	
EH423274	1.788	moderately similar to (259)AT4G23530| Symbols: | similar to unknown protein [Arabidopsis thaliana] (TAIR:AT4G11300.1); similar to h	
JCVI_15141	1.788	moderately similar to (375)AT2G02730| Symbols: | similar to bZIP family transcription factor [Arabidopsis thaliana] (TAIR:AT1G27000.	
RC_EE569815	1.787	no similarity	
JCVI_4807	1.787	moderately similar to (325)AT3G57340| Symbols: | DNAJ heat shock N-terminal domain-containing protein | chr3:21230153-21231256 F	
JCVI_7673	1.787	moderately similar to (360)AT5G41980| Symbols: | similar to unknown protein [Arabidopsis thaliana] (TAIR:AT1G43722.1); similar to h	
JCVI_40539	1.787	moderately similar to (400)AT5G16300| Symbols: | similar to hypothetical protein OsI_003871 [Oryza sativa (indica cultivar-group)] (GB	
CV432464	1.787	weakly similar to (122)AT4G03550| Symbols: GSL05, ATGSL5, PMR4, GSL5, ATGSL05 | ATGSL05 (GLUCAN SYNTHASE-LIKE 5);	
ES952230	1.787	very weakly similar to (90.5)AT1G75290| Symbols: | isoflavone reductase, putative | chr1:28257551-28258834 FORWARDvery weakly si	
JCVI_1934	1.787	moderately similar to (226)AT2G36320| Symbols: | zinc finger (AN1-like) family protein | chr2:15236467-15236952 FORWARDweakly s	
EE423385	1.787	moderately similar to (209)AT1G69370| Symbols: CM-3, CM3 | CM3 (CHORISMATE MUTASE 3); chorismate mutase | chr1:26083761-	
JCVI_28162	1.787	moderately similar to (261)AT1G53035| Symbols: | similar to unknown protein [Arabidopsis thaliana] (TAIR:AT3G15358.1); similar to u	-2.923
JCVI_8129	1.787	moderately similar to (390)AT2G30670| Symbols: | tropinone reductase, putative / tropine dehydrogenase, putative | chr2:13076390-1307	
JCVI_1785	1.786	highly similar to (504)AT4G37870| Symbols: PCK1, PEPCK | PCK1/PEPCK (PHOSPHOENOLPYRUVATE CARBOXYKINASE 1); AT	
JCVI_38006	1.786	moderately similar to (348)AT3G19895| Symbols: | similar to unknown protein [Arabidopsis thaliana] (TAIR:AT3G12940.1); similar to u	
AM059731	1.786	moderately similar to (253)AT3G21760| Symbols: | UDP-glucoronosyl/UDP-glucosyl transferase family protein | chr3:7667106-7668563	
EX022980	1.786	very weakly similar to (98.6)AT3G62700| Symbols: ATMRP10 | ATMRP10 (Arabidopsis thaliana multidrug resistance-associated protein	
EX123243	1.786	moderately similar to (402)AT3G28670| Symbols: | oxidoreductase/ zinc ion binding | chr3:10745420-10748220 FORWARD [21830]	
JCVI_34361	1.786	weakly similar to (167)AT3G24350| Symbols: ATSYP32, SYP32 | SYP32 (syntaxin 32); SNAP receptor | chr3:8837740-8839409 FORWA	
JCVI_2516	1.786	moderately similar to (281)AT1G23205| Symbols: | invertase/pectin methylesterase inhibitor family protein | chr1:8234223-8234840 REV	
EX072352	1.786	no similarity	
EV110808	1.786	weakly similar to (128)AT5G47860| Symbols: | similar to unknown protein [Arabidopsis thaliana] (TAIR:AT3G43540.1); similar to unnar	
JCVI_29720	1.785	moderately similar to (249)AT5G17780| Symbols: | hydrolase, alpha/beta fold family protein | chr5:5867431-5868978 REVERSE no origi	
JCVI_7545	1.785	weakly similar to (194)AT5G23920| Symbols: | similar to unknown protein [Arabidopsis thaliana] (TAIR:AT5G52420.1); similar to unnar	
JCVI_22128	1.785	moderately similar to (383)AT4G35190| Symbols: | similar to unknown protein [Arabidopsis thaliana] (TAIR:AT2G37210.1); similar to u	
JCVI_26029	1.785	moderately similar to (301)AT4G15760| Symbols: | monooxygenase, putative (MO1) | chr4:8972783-8974229 REVERSE no original desc	
JCVI_1639	1.785	highly similar to (699)AT2G38080| Symbols: IRX12, LAC4 | IRX12/LAC4 (laccase 4); copper ion binding / oxidoreductase | chr2:159416	
JCVI_5648	1.785	moderately similar to (296)AT2G21110| Symbols: | disease resistance-responsive family protein | chr2:9057371-9057931 REVERSE no o	
EE415268	1.784	very weakly similar to (97.4)AT4G36440| Symbols: | similar to unnamed protein product [Vitis vinifera] (GB:CAO48295.1) | chr4:172072	
JCVI_15794	1.784	moderately similar to (205)AT5G05180| Symbols: | similar to unknown protein [Arabidopsis thaliana] (TAIR:AT3G10880.1); similar to u	
JCVI_7866	1.784	moderately similar to (375)AT1G19450| Symbols: | integral membrane protein, putative / sugar transporter family protein | chr1:6731662-	
JCVI_29905	1.784	weakly similar to (147)AT2G37980| Symbols: | similar to unknown protein [Arabidopsis thaliana] (TAIR:AT5G01100.1); similar to unkno	
JCVI_24937	1.784	weakly similar to (139)AT1G36370| Symbols: SHM7 | SHM7 (serine hydroxymethyltransferase 7); glycine hydroxymethyltransferase | chr	
JCVI_12540	1.784	moderately similar to (222)AT3G03750| Symbols: | SET domain-containing protein | chr3:939983-941518 FORWARD no original descrip	
JCVI_4256	1.784	weakly similar to (197)AT1G80440| Symbols: | kelch repeat-containing F-box family protein | chr1:30246655-30247719 FORWARD no c	
EX086464	1.784	moderately similar to (395)AT5G52570| Symbols: B2, CHY2, BETA-OHASE 2 | BETA-OHASE 2 (BETA-CAROTENE HYDROXYLAS	
EE533368	1.783	moderately similar to (391)AT1G17070| Symbols: | D111/G-patch domain-containing protein | chr1:5837646-5840195 FORWARD [2017	
JCVI_7555	1.783	highly similar to (509)AT1G32220| Symbols: | binding / catalytic/ coenzyme binding | chr1:11608018-11609571 FORWARD no original c	
CX194386	1.783	moderately similar to (409)AT5G40740| Symbols: | similar to Os02g0329300 [Oryza sativa (japonica cultivar-group)] (GB:NP_00104671	
JCVI_8457	1.783	moderately similar to (296)AT3G10770| Symbols: | nucleic acid binding | chr3:3372466-3374031 REVERSE no original description	
CX269416	1.783	weakly similar to (178)AT3G48800| Symbols: | sterile alpha motif (SAM) domain-containing protein | chr3:18106810-18107646 REVERS	
JCVI_7306	1.783	weakly similar to (179)AT4G10925| Symbols: | F-box family protein | chr4:6702983-6704279 REVERSE no original description	
EE451952	1.783	weakly similar to (165)AT1G11510| Symbols: | DNA-binding storekeeper protein-related | chr1:3871778-3872836 REVERSE [20194]	
EV218556	1.783	no similarity	
JCVI_17893	1.783	moderately similar to (206)AT5G16590| Symbols: | leucine-rich repeat transmembrane protein kinase, putative | chr5:5431865-5433924 F	
EV171085	1.783	no similarity	1.345
JCVI_1348	1.783	moderately similar to (343)AT5G58070| Symbols: | lipocalin, putative | chr5:23517738-23518382 REVERSE no original description	-1.578
JCVI_10554	1.782	no original description	
JCVI_8648	1.782	moderately similar to (277)AT1G10190| Symbols: | similar to unknown protein [Arabidopsis thaliana] (TAIR:AT4G15050.1); similar to h	
EE469321	1.782	very weakly similar to (80.9)AT5G01090| Symbols: | legume lectin family protein | chr5:33054-34115 FORWARD [20156]	
EX039879	1.782	moderately similar to (310)AT1G01520| Symbols: | myb family transcription factor | chr1:190596-192139 FORWARD [21811]	1.988
ES984651	1.782	weakly similar to (124)AT5G16760| Symbols: | inositol 1,3,4-trisphosphate 5/6-kinase | chr5:5509893-5510852 FORWARD [21389]	
JCVI_37770	1.782	moderately similar to (338)AT1G72500| Symbols: | inter-alpha-trypsin inhibitor heavy chain-related | chr1:27298998-27302218 REVERS	2.322
JCVI_18381	1.782	weakly similar to (111)AT1G07615| Symbols: | GTP binding | chr1:2342274-2344197 REVERSE no original description	
ES942120	1.781	weakly similar to (140)AT3G20290| Symbols: | calcium-binding EF hand family protein | chr3:7075063-7078661 REVERSE [21391]	
EV112633	1.781	no similarity	
JCVI_33441	1.781	moderately similar to (251)AT5G60850| Symbols: OBP4 | OBP4 (OBF BINDING PROTEIN 4); DNA binding / transcription factor | chr5	-1.964
JCVI_36998	1.781	moderately similar to (491)AT5G58830| Symbols: | subtilase family protein | chr5:23773199-23775826 FORWARD no original descriptio	
EV089082	1.781	moderately similar to (334)AT4G22790| Symbols: | MATE efflux family protein | chr4:11975164-11976639 REVERSE [21444]	
JCVI_2741	1.781	moderately similar to (381)AT3G04870| Symbols: PDE181, ZDS | ZDS (ZETA-CAROTENE DESATURASE); carotene 7,8-desaturase | c	
JCVI_1781	1.780	moderately similar to (459)AT4G24820| Symbols: | 26S proteasome regulatory subunit, putative (RPN7) | chr4:12790481-12792609 REV	
ES903408	1.780	moderately similar to (350)AT4G27060| Symbols: CN, SPR2, TOR1 | TOR1 (TORTIFOLIA 1) | chr4:13581587-13585076 REVERSE [21	
JCVI_13004	1.779	moderately similar to (257)AT1G11440| Symbols: | similar to glycine-rich protein [Arabidopsis thaliana] (TAIR:AT3G29075.1); similar to	
JCVI_17623	1.779	weakly similar to (134)AT2G17130| Symbols: IDH2 | IDH2 (ISOCITRATE DEHYDROGENASE SUBUNIT 2); isocitrate dehydrogenase	
DW999106	1.779	weakly similar to (119)AT4G29090| Symbols: | reverse transcriptase, putative / RNA-dependent DNA polymerase, putative | chr4:143335	
JCVI_2668	1.779	moderately similar to (419)AT2G30970| Symbols: ASP1 | ASP1 (ASPARTATE AMINOTRANSFERASE 1) | chr2:13186089-13188763 F	
JCVI_10903	1.779	moderately similar to (391)AT3G13360| Symbols: WIP3 | WIP3 (WPP-DOMAIN INTERACTING PROTEIN 3) | chr3:4338479-4339989	
JCVI_1770	1.779	moderately similar to (301)AT1G54030| Symbols: | GDSL-motif lipase, putative | chr1:20171353-20173144 FORWARD no original descr	
EV033790	1.779	moderately similar to (251)AT2G03670| Symbols: CDC48B | CDC48B; ATPase | chr2:1117592-1120358 FORWARDweakly similar to (1	
JCVI_13164	1.779	moderately similar to (452)AT3G52990| Symbols: | pyruvate kinase, putative | chr3:19660314-19663215 FORWARDweakly similar to (1	
JCVI_11838	1.779	moderately similar to (240)AT4G00600| Symbols: | tetrahydrofolate dehydrogenase/cyclohydrolase, putative | chr4:255320-256610 REVE	

JCVI_6671	1.778	moderately similar to (244)AT2G35790| Symbols: | similar to unnamed protein product [Vitis vinifera] (GB:CAO68985.1) | chr2:1504781	
JCVI_14057	1.778	no original description	-2.152
EE447857	1.778	weakly similar to (177)AT1G73940| Symbols: | similar to unknown protein [Arabidopsis thaliana] (TAIR:AT5G49410.1); similar to unkno	
AM388641	1.778	moderately similar to (284)AT1G03080| Symbols: | kinase interacting family protein | chr1:731794-737332 REVERSE [20118]	
JCVI_28863	1.778	moderately similar to (296)AT3G04560| Symbols: | similar to unnamed protein product [Vitis vinifera] (GB:CAO65029.1) | chr3:1227512	
JCVI_37279	1.777	moderately similar to (271)AT5G01510| Symbols: | similar to unknown protein [Arabidopsis thaliana] (TAIR:AT3G45890.1); similar to u	
JCVI_18774	1.777	moderately similar to (266)AT2G46620| Symbols: | AAA-type ATPase family protein | chr2:19146141-19147616 REVERSE no original d	
EV115814	1.777	no similarity	
JCVI_13364	1.777	moderately similar to (306)AT2G45060| Symbols: | similar to unknown protein [Arabidopsis thaliana] (TAIR:AT4G26410.1); similar to u	
JCVI_1157	1.777	moderately similar to (311)AT3G63080| Symbols: MEE42, ATGPX5 | ATGPX5 (GLUTATHIONE PEROXIDASE 5); glutathione peroxi	
JCVI_26790	1.776	moderately similar to (364)AT4G06634| Symbols: | zinc finger (C2H2 type) family protein | chr4:3764496-3766174 REVERSE no origina	2.099
ES937696	1.776	moderately similar to (232)AT3G52780| Symbols: ATPAP20, PAP20 | ATPAP20/PAP20; protein serine/threonine phosphatase | chr3:195	
JCVI_20440	1.776	moderately similar to (281)AT3G15090| Symbols: | oxidoreductase, zinc-binding dehydrogenase family protein | chr3:5076854-5078877 F	
EX093168	1.776	moderately similar to (217)AT5G65205| Symbols: | short-chain dehydrogenase/reductase (SDR) family protein | chr5:26068152-2606924	
JCVI_8256	1.776	moderately similar to (278)AT1G05270| Symbols: | TraB family protein | chr1:1531805-1534304 REVERSE no original description	
EE519056	1.776	no similarity	
EX122427	1.776	no similarity	
ES969469	1.776	weakly similar to (121)AT2G01275| Symbols: | zinc finger (C3HC4-type RING finger) family protein | chr2:142609-143808 REVERSE [	
JCVI_6259	1.775	moderately similar to (441)AT2G44210| Symbols: | similar to unknown protein [Arabidopsis thaliana] (TAIR:AT1G55360.1); similar to u	
ES991856	1.775	moderately similar to (242)AT5G10900| Symbols: | calcineurin-like phosphoesterase family protein | chr5:3436414-3439222 REVERSE [	
EX078810	1.775	no similarity	-1.975
EV086924	1.775	no similarity	
JCVI_28482	1.775	moderately similar to (433)AT1G49780| Symbols: | U-box domain-containing protein | chr1:18432692-18433957 REVERSE no original d	
JCVI_10966	1.775	moderately similar to (202)AT1G08680| Symbols: AGD14, ZIGA4 | ZIGA4 (ARF GAP-LIKE ZINC FINGER-CONTAINING PROTEIN	1.654
JCVI_8233	1.775	moderately similar to (210)AT4G33780| Symbols: | similar to unknown protein [Arabidopsis thaliana] (TAIR:AT1G69935.1); similar to u	
JCVI_20214	1.775	weakly similar to (199)AT3G12500| Symbols: PR3, PR-3, CHI-B, B-CHI, ATHCHIB | ATHCHIB (BASIC CHITINASE); chitinase | chr3:	
JCVI_31952	1.775	no original description	
JCVI_42297	1.775	moderately similar to (258)AT1G19840| Symbols: | auxin-responsive family protein | chr1:6872785-6873246 REVERSE no original descr	
JCVI_3754	1.774	moderately similar to (308)AT5G47110| Symbols: | lil3 protein, putative | chr5:19151445-19152465 REVERSE no original description	-4.396
JCVI_28211	1.774	moderately similar to (357)AT3G50620| Symbols: | nodulation protein-related | chr3:18795974-18797728 REVERSE no original descripti	
CV546649	1.774	very weakly similar to (87.8)AT1G66340| Symbols: EIN1, ETR, ETR1 | ETR1 (ETHYLENE RESPONSE 1); two-component response reg	
EE437362	1.774	weakly similar to (191)AT1G24310| Symbols: | similar to unnamed protein product [Vitis vinifera] (GB:CAO66197.1); contains domain P	
JCVI_17115	1.774	moderately similar to (470)AT4G33420| Symbols: | peroxidase, putative | chr4:16084859-16086108 FORWARDmoderately similar to (2	
DY012708	1.773	moderately similar to (388)AT1G61380| Symbols: | S-locus protein kinase, putative | chr1:22649942-22653066 REVERSEweakly similar	
JCVI_24706	1.773	weakly similar to (120)AT3G12800| Symbols: | short-chain dehydrogenase/reductase (SDR) family protein | chr3:4063470-4064764 REV	
ES988121	1.773	no similarity	
JCVI_2161	1.773	moderately similar to (335)AT4G37470| Symbols: | hydrolase, alpha/beta fold family protein | chr4:17617039-17618357 REVERSE no or	
JCVI_27083	1.773	moderately similar to (341)AT3G50440| Symbols: | hydrolase | chr3:18728373-18729416 REVERSEweakly similar to (153)PIR7B_ORY	
EV099977	1.773	moderately similar to (231)AT4G15410| Symbols: PUX5 | UBX domain-containing protein | chr4:8814868-8816596 FORWARD [21477]	1.378
JCVI_9196	1.773	highly similar to (731)AT5G26600| Symbols: | catalytic/ pyridoxal phosphate binding | chr5:9377458-9378885 FORWARD no original de	
CX187547	1.773	moderately similar to (346)AT3G59470| Symbols: | far-red impaired responsive family protein / FAR1 family protein | chr3:21990076-219	
JCVI_6236	1.772	moderately similar to (256)AT1G54850| Symbols: | similar to unknown protein [Arabidopsis thaliana] (TAIR:AT1G54840.1); similar to h	
EV126846	1.772	weakly similar to (121)AT3G48800| Symbols: | sterile alpha motif (SAM) domain-containing protein | chr3:18106810-18107646 REVERS	
JCVI_29167	1.772	moderately similar to (206)AT3G56220| Symbols: | transcription regulator | chr3:20869920-20871281 FORWARD no original description	1.605
EX057806	1.772	moderately similar to (299)AT1G24180| Symbols: IAR4 | IAR4 (IAA-conjugate-resistant 4); pyruvate dehydrogenase (acetyl-transferring)	
JCVI_942	1.772	moderately similar to (474)AT5G67370| Symbols: | similar to unknown protein [Arabidopsis thaliana] (TAIR:AT5G11840.1); similar to h	
JCVI_13187	1.771	moderately similar to (292)AT1G26110| Symbols: | similar to unknown protein [Arabidopsis thaliana] (TAIR:AT5G45330.1); similar to h	
AT000877	1.771	no similarity	
JCVI_2022	1.771	weakly similar to (195)AT3G54620| Symbols: ATBZIP25, BZO2H4 | ATBZIP25/BZO2H4 (ARABIDOPSIS THALIANA BASIC LEUCI	
JCVI_34977	1.771	highly similar to (578)AT5G09430| Symbols: | hydrolase | chr5:2932163-2933363 FORWARD no original description	
JCVI_13404	1.771	highly similar to (852)AT4G00710| Symbols: | protein kinase family protein | chr4:290807-293096 FORWARDweakly similar to (120)CF	
JCVI_41376	1.771	moderately similar to (366)AT1G07390| Symbols: | protein binding | chr1:2269891-2274651 FORWARDweakly similar to (109)PSKR_D	
JCVI_16257	1.771	moderately similar to (207)AT3G48120| Symbols: | similar to unnamed protein product [Vitis vinifera] (GB:CAO64408.1) | chr3:1778312	
JCVI_3826	1.771	no original description	
JCVI_4084	1.771	weakly similar to (197)AT2G37410| Symbols: TIM17, ATTIM17-2 | ATTIM17-2 (Arabidopsis thaliana translocase inner membrane subur	
ES935268	1.771	weakly similar to (119)AT4G34680| Symbols: | GATA transcription factor 3, putative (GATA-3) | chr4:16553705-16554615 FORWARD	
JCVI_36850	1.771	moderately similar to (387)AT4G26940| Symbols: | galactosyltransferase family protein | chr4:13530229-13532393 REVERSE no origina	
EV199230	1.771	moderately similar to (248)AT4G18880| Symbols: HSFA4A, AT-HSFA4A | AT-HSFA4A (Arabidopsis thaliana heat shock transcription fa	
EV219887	1.771	no similarity	
EX065729	1.770	weakly similar to (187)AT5G60660| Symbols: PIP2F, PIP2;4 | PIP2;4/PIP2F (plasma membrane intrinsic protein 2;4); water channel | chr5	
EX029777	1.770	moderately similar to (447)AT3G26430| Symbols: | GDSL-motif lipase/hydrolase family protein | chr3:9675656-9677126 FORWARDmod	
ES943297	1.770	very weakly similar to (93.2)AT2G28900| Symbols: ATOEP16-L, ATOEP16-1, OEP16 | OEP16 (OUTER ENVELOPE PROTEIN 16); P-P	-1.809
EE439823	1.770	weakly similar to (180)AT2G16780| Symbols: MSI02, NFC02, NFC2, MSI2 | MSI2 (NUCLEOSOME/CHROMATIN ASSEMBLY FACT	
JCVI_22248	1.769	weakly similar to (104)AT5G06520| Symbols: | SWAP (Suppressor-of-White-APricot)/surp domain-containing protein | chr5:1987251-198	
JCVI_31168	1.769	highly similar to (754)AT5G15630| Symbols: COBL4, IRX6 | COBL4/IRX6 (COBRA-LIKE4) | chr5:5084845-5086548 FORWARDhighly	
CN725800	1.769	weakly similar to (126)AT1G16770| Symbols: | similar to unnamed protein product [Vitis vinifera] (GB:CAO41707.1) | chr1:5738128-573	
RC_EV087250	1.769	no similarity	
JCVI_15780	1.769	weakly similar to (200)AT1G62120| Symbols: | mitochondrial transcription termination factor-related / mTERF-related | chr1:22963991-2	
JCVI_3441	1.769	moderately similar to (273)AT4G17940| Symbols: | binding | chr4:9965799-9966790 FORWARD no original description	
JCVI_16797	1.769	moderately similar to (291)AT3G47480| Symbols: | calcium-binding EF hand family protein | chr3:17507339-17507890 REVERSE no ori	
JCVI_37881	1.769	no original description	
EX128203	1.769	no similarity	
JCVI_29869	1.769	moderately similar to (349)AT4G34135| Symbols: UGT73B2 | UGT73B2; UDP-glycosyltransferase | chr4:16346013-16347020 REVERSE	
ES997406	1.768	no similarity	
JCVI_32607	1.768	moderately similar to (390)AT1G25540| Symbols: PFT1 | PFT1 (PHYTOCHROME AND FLOWERING TIME 1) | chr1:8969379-897328	
JCVI_17846	1.768	moderately similar to (263)AT3G18440| Symbols: ATALMT9 | ATALMT9 (ALUMINUM-ACTIVATED MALATE TRANSPORTER 9);	
JCVI_1687	1.768	moderately similar to (486)AT1G44970| Symbols: | peroxidase, putative | chr1:17004677-17006003 FORWARDmoderately similar to (2	-1.438
EV210903	1.768	moderately similar to (210)AT5G15260| Symbols: | structural constituent of ribosome | chr5:4953649-4954353 REVERSE [21491] 1 520	
CD811808	1.768	no similarity	
EE479177	1.768	no similarity	
JCVI_38465	1.768	highly similar to (621)AT3G04460| Symbols: PEX12, ATPEX12, APM4 | APM4/ATPEX12/PEX12 (PEROXIN-12); actin binding | chr3:	

JCVI_36462	1.768	moderately similar to (334)AT5G40910| Symbols: | disease resistance protein (TIR-NBS-LRR class), putative | chr5:16412735-16416357	
JCVI_3266	1.768	moderately similar to (340)AT4G28050| Symbols: TET7 | TET7 (TETRASPANIN7) | chr4:13942572-13943627 REVERSE no original de	
JCVI_22582	1.768	moderately similar to (379)AT4G10340| Symbols: LHCB5 | LHCB5 (LIGHT HARVESTING COMPLEX OF PHOTOSYSTEM II 5); chl	
JCVI_6978	1.768	moderately similar to (229)AT1G73760| Symbols: | zinc finger (C3HC4-type RING finger) family protein | chr1:27743027-27744822 REV	
EE419453	1.767	weakly similar to (144)AT1G75050| Symbols: | similar to ATLP-3 (Arabidopsis thaumatin-like protein 3) [Arabidopsis thaliana] (TAIR:A	
JCVI_6511	1.767	moderately similar to (209)AT2G19520| Symbols: ACG1, MSI4, NFC4, NFC04, ATMSI4, FVE | FVE | chr2:8463088-8466317 FORWAR	
EX128765	1.767	moderately similar to (243)AT5G43420| Symbols: | zinc finger (C3HC4-type RING finger) family protein | chr5:17469018-17470145 FO	2.010
EE455422	1.767	no similarity	
JCVI_706	1.767	moderately similar to (255)AT3G48070| Symbols: | protein binding / zinc ion binding | chr3:17761863-17763282 FORWARD no original	
EX045510	1.767	no similarity	
EE566228	1.767	no similarity	
DY019674	1.767	no similarity	
EE448759	1.767	moderately similar to (308)AT5G60910| Symbols: FUL, AGL8 | AGL8 (AGAMOUS-LIKE 8) | chr5:24519962-24522160 REVERSEmode	
EV125297	1.766	weakly similar to (150)AT3G21175| Symbols: TIFY2B, ZML1 | ZML1 (ZIM-LIKE 1) | chr3:7422838-7423771 FORWARD [21479]	
JCVI_28814	1.766	weakly similar to (190)AT5G25510| Symbols: | serine/threonine protein phosphatase 2A (PP2A) regulatory subunit B', putative | chr5:888	
JCVI_2948	1.766	moderately similar to (252)AT2G20370| Symbols: KAM1, MUR3 | KAM1/MUR3 (MURUS 3); catalytic/ transferase, transferring glycosy	
EX021056	1.765	weakly similar to (128)AT2G01460| Symbols: | phosphoribulokinase/uridine kinase family protein | chr2:206135-210974 FORWARD [21	
JCVI_9750	1.765	moderately similar to (359)AT3G10230| Symbols: LYC | LYC (LYCOPENE CYCLASE) | chr3:3164345-3165454 REVERSEmoderately s	
JCVI_21592	1.765	no original description	
ES952389	1.765	very weakly similar to (89.7)AT1G77470| Symbols: | replication factor C 36 kDA, putative | chr1:29117088-29119217 REVERSE [21423]	
JCVI_26581	1.765	moderately similar to (419)AT1G48310| Symbols: | SNF2 domain-containing protein / helicase domain-containing protein | chr1:1785228	
EE446718	1.765	weakly similar to (173)AT4G01290| Symbols: | similar to unnamed protein product [Vitis vinifera] (GB:CAO39638.1) | chr4:538213-543	
EV119344	1.765	moderately similar to (327)AT5G13110| Symbols: G6PD2 | G6PD2 (GLUCOSE-6-PHOSPHATE DEHYDROGENASE 2); glucose-6-pho	
JCVI_40109	1.765	moderately similar to (389)AT3G48720| Symbols: | transferase family protein | chr3:18057512-18060280 FORWARDweakly similar to (	
JCVI_31847	1.765	weakly similar to (181)AT1G15320| Symbols: | similar to unnamed protein product [Vitis vinifera] (GB:CAO62706.1) | chr1:5272463-527	
JCVI_9526	1.765	moderately similar to (284)AT3G10815| Symbols: | zinc finger (C3HC4-type RING finger) family protein | chr3:3385014-3385613 REVE	
JCVI_2086	1.764	moderately similar to (212)AT2G36530| Symbols: LOS2 | LOS2 (Low expression of osmotically responsive genes 1); phosphopyruvate hy	
JCVI_17106	1.764	weakly similar to (196)AT5G60680| Symbols: | similar to unknown protein [Arabidopsis thaliana] (TAIR:AT3G45210.1); similar to unna	
JCVI_5670	1.764	moderately similar to (353)AT5G19320| Symbols: RANGAP2 | RANGAP2 (RAN GTPASE ACTIVATING PROTEIN 2); RAN GTPase a	
JCVI_35977	1.764	moderately similar to (424)AT1G74850| Symbols: PTAC2 | PTAC2 (PLASTID TRANSCRIPTIONALLY ACTIVE2) | chr1:28122898-28	
EE471250	1.764	no similarity	
CD835541	1.764	moderately similar to (221)AT3G21690| Symbols: | MATE efflux family protein | chr3:7638757-7641868 FORWARD [13981]	
EE530658	1.764	no similarity	
EX096631	1.764	weakly similar to (107)AT5G27710| Symbols: | similar to unnamed protein product [Vitis vinifera] (GB:CAO17522.1) | chr5:9813073-981	
EX046835	1.764	moderately similar to (269)AT3G52500| Symbols: | aspartyl protease family protein | chr3:19476622-19478031 REVERSE [21812]	
JCVI_14081	1.764	moderately similar to (320)AT1G10640| Symbols: | polygalacturonase, putative / pectinase, putative | chr1:3515479-3516932 REVERSEv	
JCVI_14511	1.763	no original description	
EV185340	1.763	no similarity	
EX103904	1.763	moderately similar to (414)AT3G17970| Symbols: ATTOC64-III | ATTOC64-III (ARABIDOPSIS THALIANA TRANSLOCON AT THE	
EV043533	1.763	no similarity	
JCVI_39883	1.763	moderately similar to (339)AT3G19770| Symbols: ATVPS9A | ATVPS9A (ARABIDOPSIS THALIANA VACUOLAR PROTEIN SORTI	
EE411584	1.763	moderately similar to (386)AT5G60410| Symbols: ATSIZ1, SIZ1 | ATSIZ1/SIZ1 | chr5:24312452-24318018 FORWARD [20140]	
JCVI_2164	1.763	moderately similar to (295)AT5G22840| Symbols: | protein kinase family protein | chr5:7631106-7633106 REVERSE no original descript	
EV090980	1.762	moderately similar to (382)AT4G24280| Symbols: CPHSC70-1 | CPHSC70-1 (chloroplast heat shock protein 70-1); ATP binding / unfolde	
JCVI_8765	1.762	moderately similar to (318)AT5G22950| Symbols: VPS24.1 | VPS24.1 | chr5:7681383-7682723 FORWARD no original description	
JCVI_6477	1.762	moderately similar to (258)AT3G13570| Symbols: SCL30a | SCL30a (SC35-like splicing factor 30a); RNA binding | chr3:4429571-443160	1.100
JCVI_33221	1.762	moderately similar to (332)AT2G40460| Symbols: | proton-dependent oligopeptide transport (POT) family protein | chr2:16904201-16908	
EX030096	1.762	moderately similar to (317)AT2G45320| Symbols: | binding / catalytic | chr2:18691256-18693181 REVERSE [21810]	
JCVI_10381	1.762	highly similar to (609)AT2G36390| Symbols: BE3, SBE2.1 | SBE2.1 (STARCH BRANCHING ENZYME 2.1); 1,4-alpha-glucan branchin	2.059
EV060801	1.762	no similarity	
JCVI_6984	1.762	highly similar to (547)AT1G21100| Symbols: | O-methyltransferase, putative | chr1:7386980-7388307 REVERSEmoderately similar to (2	
JCVI_36872	1.762	weakly similar to (113)AT5G01200| Symbols: | myb family transcription factor | chr5:77115-78293 FORWARD no original description	
JCVI_41466	1.762	no original description	
JCVI_11095	1.762	moderately similar to (427)AT1G53240| Symbols: | malate dehydrogenase (NAD), mitochondrial | chr1:19858634-19860470 REVERSEm	
JCVI_24523	1.762	moderately similar to (409)AT1G15500| Symbols: ATNTT2 | ATNTT2; ATP:ADP antiporter | chr1:5326421-5328683 FORWARDmodera	
EV031830	1.762	very weakly similar to (92.8)AT2G28520| Symbols: VHA-A1 | VHA-A1 (VACUOLAR PROTON ATPASE A 1); ATPase | chr2:12217103	
RC_EV000942	1.761	no similarity	
EG021311	1.761	weakly similar to (175)AT5G65670| Symbols: IAA9 | IAA9 (indoleacetic acid-induced protein 9); transcription factor | chr5:26271689-262	
CX267046	1.761	weakly similar to (115)AT5G56890| Symbols: | protein kinase family protein | chr5:23028027-23032785 REVERSE [16816]	
JCVI_20394	1.761	very weakly similar to (82.0)AT4G01895| Symbols: | systemic acquired resistance (SAR) regulator protein NIMIN-1-related | chr4:819957	
EV205170	1.760	no similarity	
EV174247	1.760	weakly similar to (189)AT4G36840| Symbols: | kelch repeat-containing protein | chr4:17352169-17352885 FORWARD [21486] 75 891 8	
EV060654	1.760	moderately similar to (315)AT3G26950| Symbols: | binding | chr3:9942439-9944648 REVERSE [21442]	
JCVI_502	1.760	moderately similar to (431)AT5G22480| Symbols: | zinc finger (ZPR1-type) family protein | chr5:7451646-7456171 REVERSE no origina	-3.336
JCVI_42190	1.760	moderately similar to (237)AT2G33880| Symbols: HB-3, STIP, WOX9 | WOX9 (STIMPY); transcription factor | chr2:14348718-1435067	
JCVI_16216	1.760	moderately similar to (311)AT2G26350| Symbols: PEX10 | PEX10 (PEROXIN 10); protein binding / zinc ion binding | chr2:11224845-11	
EH414396	1.759	weakly similar to (110)AT3G08690| Symbols: UBC11 | UBC11 (ubiquitin-conjugating enzyme 11); ubiquitin-protein ligase | chr3:264149	
JCVI_9836	1.759	highly similar to (828)AT3G01120| Symbols: CGS, ATCYS1, CGS1, MTO1 | MTO1 (METHIONINE OVERACCUMULATION 1) | chr3	
JCVI_7121	1.759	moderately similar to (350)AT2G02010| Symbols: GAD4 | GAD4 (GLUTAMATE DECARBOXYLASE 4); calmodulin binding | chr2:474	
BQ704205	1.759	no similarity	
JCVI_31404	1.759	weakly similar to (132)AT5G41350| Symbols: | zinc finger (C3HC4-type RING finger) family protein | chr5:16559254-16560320 REVER	
JCVI_6180	1.759	weakly similar to (189)AT5G64850| Symbols: | similar to unknown protein [Arabidopsis thaliana] (TAIR:AT5G09960.1); similar to 80C0	
JCVI_13253	1.759	highly similar to (551)AT4G36220| Symbols: CYP84A1, FAH1 | FAH1 (FERULATE-5-HYDROXYLASE 1); ferulate 5-hydroxylase | chr	
JCVI_38713	1.759	highly similar to (504)AT3G62590| Symbols: | lipase class 3 family protein | chr3:23158949-23161145 REVERSE no original description	
JCVI_33474	1.759	moderately similar to (431)AT5G67160| Symbols: | transferase family protein | chr5:26814926-26816230 REVERSEvery weakly similar t	
ES911778	1.758	moderately similar to (371)AT1G61560| Symbols: ATMLO6, MLO6 | MLO6 (MILDEW RESISTANCE LOCUS O 6); calmodulin binding	
EE443458	1.758	weakly similar to (150)AT2G36810| Symbols: | binding | chr2:15432818-15443960 REVERSE [20160]	
ES963559	1.758	no similarity	-1.428
JCVI_35288	1.758	moderately similar to (298)AT5G14950| Symbols: GMII, ATGMII | ATGMII/GMII (GOLGI ALPHA-MANNOSIDASE II); alpha-manno	-1.337
ES997889	1.758	moderately similar to (300)AT5G23230| Symbols: NIC2 | NIC2 (NICOTINAMIDASE 2); catalytic/ nicotinamidase | chr5:7826008-78266	
EE533353	1.758	moderately similar to (329)AT3G11920| Symbols: | glutaredoxin-related | chr3:3772317-3774893 FORWARD [20175]	

CN727324	1.758	no similarity	
JCVI_5159	1.758	moderately similar to (352)AT2G01540| Symbols: | C2 domain-containing protein | chr2:242296-243232 REVERSE no original descriptic	
EX042240	1.757	weakly similar to (170)AT1G22960| Symbols: | pentatricopeptide (PPR) repeat-containing protein | chr1:8128075-8130231 REVERSE [2	
EV182507	1.757	weakly similar to (110)AT3G57480| Symbols: | zinc finger (C2H2 type, AN1-like) family protein | chr3:21289060-21290096 REVERSE [	
JCVI_22190	1.757	weakly similar to (193)AT2G35680| Symbols: | dual specificity protein phosphatase family protein | chr2:15004083-15005669 REVERSE	
JCVI_7854	1.757	highly similar to (519)AT4G39970| Symbols: | haloacid dehalogenase-like hydrolase family protein | chr4:18536672-18538423 REVERSE	
EE467457	1.757	no similarity	
EV107992	1.757	no similarity	
EV080043	1.757	no similarity	
EX037570	1.757	moderately similar to (262)AT1G29960| Symbols: AGL64 | AGL64; peptidase | chr1:10494797-10495835 FORWARD [21811]	
AM395241	1.757	weakly similar to (142)AT3G61150| Symbols: HDG1 | HDG1 (HOMEODOMAIN GLABROUS1); DNA binding / transcription factor | ch	
EE429944	1.757	moderately similar to (291)AT1G14870| Symbols: | Identical to Uncharacterized protein At1g14870 [Arabidopsis Thaliana] (GB:Q9LQU	
JCVI_28625	1.757	moderately similar to (473)AT5G56890| Symbols: | protein kinase family protein | chr5:23028027-23032785 REVERSEweakly similar to	
JCVI_30844	1.757	moderately similar to (264)AT5G15710| Symbols: | F-box family protein | chr5:5122794-5124140 FORWARD no original description	1.252
DW997300	1.756	no similarity	
EX092360	1.756	moderately similar to (265)AT4G00840| Symbols: | zinc ion binding | chr4:355483-357105 REVERSE [21823]	
JCVI_2287	1.756	moderately similar to (432)AT3G12120| Symbols: FAD2 | FAD2 (FATTY ACID DESATURASE 2) | chr3:3860598-3861749 REVERSEm	
EV104127	1.756	no similarity	
RC_AM057102	1.756	no similarity	
JCVI_39424	1.756	moderately similar to (260)AT3G25680| Symbols: | similar to unknown protein [Arabidopsis thaliana] (TAIR:AT5G23890.1); similar to u	
JCVI_31470	1.756	moderately similar to (316)AT5G26030| Symbols: FC1 | FC1 (FERROCHELATASE 1); ferrochelatase | chr5:9096678-9098755 FORWAI	
JCVI_38534	1.756	weakly similar to (181)AT5G26760| Symbols: | similar to hypothetical protein OsI_017683 [Oryza sativa (indica cultivar-group)] (GB:EA	
JCVI_14033	1.755	moderately similar to (270)AT5G04170| Symbols: | calcium-binding EF hand family protein | chr5:1145580-1147520 FORWARD no orig	
EV172917	1.755	moderately similar to (240)AT3G25480| Symbols: | rhodanese-like domain-containing protein | chr3:9236828-9237835 REVERSE [2148	
JCVI_34250	1.755	highly similar to (547)AT4G35870| Symbols: | similar to early-responsive to dehydration protein-related / ERD protein-related [Arabidops	
JCVI_32902	1.755	weakly similar to (110)AT4G17695| Symbols: KAN3 | KAN3 (KANADI 3); DNA binding / transcription factor | chr4:9848147-9850711 R	
JCVI_29448	1.755	no original description	
JCVI_1800	1.755	highly similar to (570)AT3G13110| Symbols: SAT-1, SAT-A, SAT3, AtSerat2;2 | AtSerat2;2 (SERINE ACETYLTRANSFERASE 1); seri	-7.224
JCVI_40694	1.754	moderately similar to (439)AT2G16090| Symbols: | zinc finger protein-related | chr2:6994449-6996904 REVERSE no original description	
JCVI_3490	1.754	moderately similar to (365)AT3G07700| Symbols: | ABC1 family protein | chr3:2459702-2463247 REVERSE no original description	
JCVI_11096	1.754	moderately similar to (417)AT3G59480| Symbols: | pfkB-type carbohydrate kinase family protein | chr3:21994080-21995417 FORWARD	
JCVI_16503	1.754	weakly similar to (124)AT5G08139| Symbols: | zinc finger (C3HC4-type RING finger) family protein | chr5:2616488-2617618 FORWAR	
JCVI_37962	1.754	no original description	
JCVI_28718	1.754	no original description	
ES899837	1.754	no similarity	
DY011947	1.754	no similarity	
JCVI_37524	1.754	very weakly similar to (81.3)AT1G06190| Symbols: | ATP binding / ATPase, coupled to transmembrane movement of ions, phosphorylativ	
JCVI_561	1.753	highly similar to (619)AT1G78000| Symbols: SEL1, SULTR1;2 | SULTR1;2 (SULFATE TRANSPORTER 1;2) | chr1:29334783-2933777	
JCVI_1044	1.753	highly similar to (857)AT5G02500| Symbols: HSP70-1, AT-HSC70-1, HSC70, HSC70-1 | HSC70-1 (heat shock cognate 70 kDa protein 1)	
DN961461	1.753	no similarity	
ES914379	1.753	no similarity	-3.141
JCVI_25371	1.753	moderately similar to (479)AT3G57150| Symbols: ATNAP57, CBF5, ATCBF5, NAP57 | NAP57 (ARABIDOPSIS THALIANA HOMOL(	
JCVI_4521	1.753	moderately similar to (234)AT5G47640| Symbols: | CCAAT-box binding transcription factor subunit B (NF-YB) (HAP3) (AHAP3) famil	
DY005886	1.753	moderately similar to (293)AT2G16480| Symbols: | SWIB complex BAF60b domain-containing protein / plus-3 domain-containing protei	-1.350
EE520142	1.753	moderately similar to (350)AT3G05670| Symbols: | PHD finger family protein | chr3:1653894-1657028 FORWARD [20185]	
CO749493	1.753	moderately similar to (269)AT2G27110| Symbols: FRS3 | FRS3 (FAR1-RELATED SEQUENCE 3); zinc ion binding | chr2:11584047-115	
EE480519	1.753	weakly similar to (166)AT2G30140| Symbols: | UDP-glucoronosyl/UDP-glucosyl transferase family protein | chr2:12879277-12880768 F(	
JCVI_5446	1.752	moderately similar to (341)AT3G58970| Symbols: | magnesium transporter CorA-like family protein | chr3:21800636-21802140 REVERS	
JCVI_42345	1.752	weakly similar to (166)AT4G34960| Symbols: | peptidyl-prolyl cis-trans isomerase, putative / cyclophilin, putative / rotamase, putative | ch	
EV191037	1.752	moderately similar to (221)AT5G55550| Symbols: | RNA recognition motif (RRM)-containing protein | chr5:22519361-22520884 REVEF	
JCVI_4800	1.752	moderately similar to (328)AT3G04080| Symbols: ATAPY1 | ATAPY1 (APYRASE 1); calmodulin binding | chr3:1068075-1070924 REV	
H07791	1.752	no similarity	
EV024105	1.752	moderately similar to (269)AT4G22720| Symbols: | glycoprotease M22 family protein | chr4:11937478-11938728 FORWARD [21441]	
AM059577	1.752	no similarity	
AM057181	1.752	no similarity	1.983
JCVI_33116	1.752	weakly similar to (164)AT4G00305| Symbols: | zinc finger (C3HC4-type RING finger) family protein | chr4:131550-131930 FORWARD	
EE566829	1.752	very weakly similar to (98.6)AT3G43740| Symbols: | leucine-rich repeat family protein | chr3:15655114-15656433 FORWARD [20153] 7	
ES942688	1.752	moderately similar to (385)AT3G57630| Symbols: | exostosin family protein | chr3:21350520-21354057 REVERSE [21391]	
JCVI_28417	1.751	moderately similar to (372)AT4G26980| Symbols: | similar to unnamed protein product [Vitis vinifera] (GB:CAO38952.1); contains doma	
JCVI_9833	1.751	moderately similar to (447)AT2G21430| Symbols: | cysteine proteinase A494, putative / thiol protease, putative | chr2:9179044-9180381 I	
JCVI_13803	1.750	moderately similar to (363)AT4G01650| Symbols: | similar to unknown protein [Arabidopsis thaliana] (TAIR:AT5G08720.1); similar to u	
JCVI_34007	1.750	moderately similar to (439)AT5G38830| Symbols: | tRNA synthetase class I (C) family protein | chr5:15562994-15565324 REVERSE no	
JCVI_38349	1.750	moderately similar to (311)AT5G63290| Symbols: | coproporphyrinogen oxidase-related | chr5:25386575-25388120 REVERSE no origina	
EG020427	1.750	moderately similar to (209)AT2G38090| Symbols: | myb family transcription factor | chr2:15952356-15953853 FORWARD [20440]	
JCVI_10004	1.750	moderately similar to (309)AT5G63990| Symbols: | 3'(2'),5'-bisphosphate nucleotidase, putative / inositol polyphosphate 1-phosphatase, p	
JCVI_2498	1.750	moderately similar to (298)AT1G63690| Symbols: | protease-associated (PA) domain-containing protein | chr1:23622153-23625745 FOR	
RC_JCVI_35798	1.750	no original description	
JCVI_21428	1.750	moderately similar to (244)AT5G13010| Symbols: EMB3011 | EMB3011 (EMBRYO DEFECTIVE 3011); RNA helicase | chr5:4122750-	
JCVI_29920	1.749	moderately similar to (318)AT4G35460| Symbols: TRB1, ATNTRB, NTRB, NTR1 | NTR1 (NADPH-dependent thioredoxin reductase 1)	
EV119743	1.749	weakly similar to (157)AT1G79670| Symbols: WAKL22, RFO1 | RFO1 (RESISTANCE TO FUSARIUM OXYSPORUM 1) | chr1:299817	
JCVI_34260	1.749	weakly similar to (106)AT5G16160| Symbols: | similar to hypothetical protein [Cleome spinosa] (GB:ABD96920.1) | chr5:5275816-5276	
RC_ES968928	1.749	no similarity	
JCVI_39701	1.749	no original description	
JCVI_24826	1.749	no original description	1.777
JCVI_21205	1.749	very weakly similar to (95.9)AT5G64980| Symbols: | transcription regulator | chr5:25978173-25980226 REVERSE no original description	
JCVI_8467	1.749	moderately similar to (270)AT4G16850| Symbols: | similar to unknown protein [Arabidopsis thaliana] (TAIR:AT1G31130.1); similar to h	
EX092863	1.749	moderately similar to (216)AT4G25740| Symbols: | 40S ribosomal protein S10 (RPS10A) | chr4:13107497-13108760 REVERSEweakly si	
CN729447	1.749	moderately similar to (318)AT5G11480| Symbols: | GTP binding | chr5:3669351-3671472 REVERSE [15725]	
JCVI_13984	1.748	moderately similar to (444)AT2G01600| Symbols: | epsin N-terminal homology (ENTH) domain-containing protein | chr2:268974-272355	
DY011510	1.748	weakly similar to (200)AT1G11480| Symbols: | eukaryotic translation initiation factor-related | chr1:3864368-3866707 REVERSE [18980	
JCVI_35515	1.748	no original description	

JCVI_40528	1.748	very weakly similar to (80.9)AT3G19910| Symbols: | zinc finger (C3HC4-type RING finger) family protein | chr3:6926503-6929330 FORW	
EV064965	1.748	no similarity	
JCVI_16826	1.747	moderately similar to (256)AT5G52870| Symbols: | similar to unknown protein [Arabidopsis thaliana] (TAIR:AT1G64080.1); similar to u	-1.716
JCVI_2255	1.747	moderately similar to (495)AT5G47500| Symbols: | pectinesterase family protein | chr5:19288489-19290072 REVERSEweakly similar to	
JCVI_30212	1.747	moderately similar to (389)AT1G66830| Symbols: | leucine-rich repeat transmembrane protein kinase, putative | chr1:24934363-24936497	
JCVI_20553	1.747	moderately similar to (215)AT5G54250| Symbols: CNGC4, HLM1, DND2, ATCNGC4 | ATCNGC4 (DEFENSE, NO DEATH 2); calmod	
EE418936	1.747	moderately similar to (410)AT1G22620| Symbols: ATSAC1 | ATSAC1 (SUPPRESSOR OF ACTIN 1); phosphoinositide 5-phosphatase | c	
CD814731	1.746	moderately similar to (325)AT5G57300| Symbols: | UbiE/COQ5 methyltransferase family protein | chr5:23225931-23227837 REVERSE |	
JCVI_13105	1.746	moderately similar to (415)AT4G34230| Symbols: ATCAD5, CAD-5, CAD5 | CAD5 (CINNAMYL ALCOHOL DEHYDROGENASE 5) |	
EE475200	1.746	very weakly similar to (88.2)AT1G05500| Symbols: SYTE, ATSYTE, NTMC2TYPE2.1, NTMC2T2.1 | ATSYTE/NTMC2T2.1/NTMC2T	
AM061937	1.746	no similarity	
ES935156	1.746	no similarity	2.564
JCVI_33589	1.745	very weakly similar to (97.8)AT2G21660| Symbols: GR-RBP7, GRP7, CCR2, ATGRP7 | ATGRP7 (COLD, CIRCADIAN RHYTHM, ANI	
EV226406	1.745	no similarity	
EE478467	1.745	weakly similar to (189)AT3G07565| Symbols: | DNA binding | chr3:2413829-2415878 FORWARD [20132]	1.516
JCVI_42624	1.745	no original description	
JCVI_31599	1.745	moderately similar to (255)AT2G21070| Symbols: FIO1 | FIO1 (FIONA1) | chr2:9048021-9049687 FORWARD no original description	
JCVI_7064	1.745	highly similar to (596)AT5G11670| Symbols: ATNADP-ME2 | ATNADP-ME2 (NADP-MALIC ENZYME 2); malate dehydrogenase (oxa	
JCVI_8585	1.745	moderately similar to (393)AT3G16910| Symbols: AAE7, ACN1 | AAE7/ACN1 (ACYL-ACTIVATING ENZYME 7); AMP binding / acet	
JCVI_12464	1.745	moderately similar to (353)AT4G17950| Symbols: | DNA-binding family protein | chr4:9967307-9969019 REVERSE no original descripti	
EX093326	1.745	no similarity	
EE562418	1.744	no similarity	
EV153369	1.744	weakly similar to (170)AT5G07820| Symbols: | similar to chromosome scaffold protein-related [Arabidopsis thaliana] (TAIR:AT5G6126(	
EX060852	1.744	moderately similar to (301)AT2G37290| Symbols: | RabGAP/TBC domain-containing protein | chr2:15664014-15668414 REVERSE [218	-1.396
EV035374	1.744	no similarity	
JCVI_3082	1.744	no original description	
JCVI_3636	1.744	weakly similar to (137)AT1G77510| Symbols: ATPDIL1-2 | ATPDIL1-2 (PDI-LIKE 1-2); protein disulfide isomerase | chr1:29131636-29	
EV112650	1.744	no similarity	
EE522000	1.743	weakly similar to (184)AT2G38480| Symbols: | integral membrane protein, putative | chr2:16118038-16118772 REVERSE [20185]	
AM389423	1.743	moderately similar to (258)AT5G18610| Symbols: | protein kinase family protein | chr5:6192738-6195373 FORWARD [20118]	
JCVI_16343	1.743	moderately similar to (343)AT1G51630| Symbols: | similar to unknown protein [Arabidopsis thaliana] (TAIR:AT3G21190.1); similar to u	
AM388683	1.743	moderately similar to (271)AT1G19190| Symbols: | hydrolase | chr1:6623867-6624823 FORWARDvery weakly similar to (83.2)GID1_OI	
JCVI_35007	1.743	moderately similar to (384)AT5G47620| Symbols: | heterogeneous nuclear ribonucleoprotein, putative / hnRNP, putative | chr5:19319774	
JCVI_36373	1.743	very weakly similar to (89.0)AT3G58680| Symbols: MBF1B, ATMBF1B | ATMBF1B/MBF1B (MULTIPROTEIN BRIDGING FACTOR	
JCVI_20168	1.743	moderately similar to (207)AT4G36840| Symbols: | kelch repeat-containing protein | chr4:17352169-17352885 FORWARD no original de	
JCVI_22638	1.743	moderately similar to (219)AT3G04450| Symbols: | transcription factor | chr3:1184308-1186270 FORWARD no original description	
ES909459	1.742	moderately similar to (247)AT5G23340| Symbols: | protein binding | chr5:7856317-7857986 FORWARD [21431]	
DY013360	1.742	weakly similar to (140)AT1G43722| Symbols: | similar to unknown protein [Arabidopsis thaliana] (TAIR:AT5G28730.1); similar to hypot	
JCVI_40006	1.742	no original description	
JCVI_13866	1.742	moderately similar to (370)AT3G44880| Symbols: LLS1, PAO, ACD1 | ACD1 (ACCELERATED CELL DEATH 1) | chr3:16394843-1639	
JCVI_15977	1.742	weakly similar to (178)AT1G09370| Symbols: | enzyme inhibitor/ pectinesterase | chr1:3024903-3025427 FORWARD no original descript	
CD826166	1.742	weakly similar to (193)AT3G13222| Symbols: GIP1 | GIP1 (GBF-INTERACTING PROTEIN 1) | chr3:4251294-4254213 REVERSE [139	
EV176709	1.742	moderately similar to (247)AT2G35100| Symbols: ARAD1 | ARAD1 (ARABINAN DEFICIENT 1); catalytic/ transferase, transferring gly	
JCVI_4147	1.742	moderately similar to (265)AT5G39360| Symbols: EDL2 | EDL2 (EID1-LIKE 2) | chr5:15770541-15771290 REVERSE no original descrip	
EE484983	1.741	moderately similar to (207)AT2G37710| Symbols: RLK | RLK (RECEPTOR LECTIN KINASE); kinase | chr2:15822012-15824039 REVE	
EV086780	1.741	weakly similar to (181)AT1G18480| Symbols: | calcineurin-like phosphoesterase family protein | chr1:6361632-6362807 FORWARD [214	
JCVI_5133	1.741	moderately similar to (401)AT3G18215| Symbols: | similar to unknown protein [Arabidopsis thaliana] (TAIR:AT5G24600.1); similar to u	
JCVI_9163	1.741	moderately similar to (283)AT5G14120| Symbols: | nodulin family protein | chr5:4556310-4558449 FORWARD no original description	
EE423142	1.741	no similarity	
JCVI_857	1.741	moderately similar to (240)AT4G03520| Symbols: ATHM2 | ATHM2 (Arabidopsis thioredoxin M-type 2); thiol-disulfide exchange interm	
CD827309	1.741	weakly similar to (166)AT2G21590| Symbols: APL4 | APL4 (large subunit of AGP 4); glucose-1-phosphate adenylyltransferase | chr2:924	
JCVI_35389	1.741	no original description	
JCVI_15559	1.741	weakly similar to (135)AT5G49220| Symbols: | similar to unknown protein [Arabidopsis thaliana] (TAIR:AT2G01260.1); similar to unnar	
JCVI_25826	1.741	weakly similar to (124)AT1G34290| Symbols: | leucine-rich repeat family protein | chr1:12497978-12498778 FORWARD no original desc	
JCVI_40086	1.741	highly similar to (633)AT1G09960| Symbols: ATSUT4, SUC4, SUT4 | SUT4 (SUCROSE TRANSPORTER 4); carbohydrate transmembra	
JCVI_3825	1.741	weakly similar to (105)AT5G08050| Symbols: | similar to unnamed protein product [Vitis vinifera] (GB:CAO62462.1); similar to unknow	
EX015942	1.740	moderately similar to (213)AT3G55990| Symbols: | Encodes ESK1 (Eskimo1). A member of a large gene family of DUF231 domain prot	
EV062069	1.740	moderately similar to (371)AT1G65810| Symbols: | tRNA-splicing endonuclease positive effector-related | chr1:24480706-24484391 REV	
JCVI_28156	1.740	moderately similar to (488)AT4G25360| Symbols: | similar to YLS7 (yellow-leaf-specific gene 7) [Arabidopsis thaliana] (TAIR:AT5G516	
AM060990	1.740	moderately similar to (245)AT2G25930| Symbols: PYK20, ELF3 | ELF3 (EARLY FLOWERING 3) | chr2:11066537-11070256 FORWAR	
JCVI_17719	1.740	weakly similar to (107)AT4G19540| Symbols: | similar to HCF101 (HIGH-CHLOROPHYLL-FLUORESCENCE 101), ATP binding [Aral	
EE534833	1.740	weakly similar to (153)AT2G45010| Symbols: | similar to unknown protein [Arabidopsis thaliana] (TAIR:AT5G51400.1); similar to unkno	
EX114202	1.740	moderately similar to (342)AT5G01540| Symbols: | lectin protein kinase, putative | chr5:211284-213332 REVERSEweakly similar to (118	
AM056728	1.740	moderately similar to (280)AT4G24190| Symbols: SHD | SHD (SHEPHERD); ATP binding | chr4:12551912-12555861 REVERSEmodera	
JCVI_30299	1.740	moderately similar to (280)AT1G01120| Symbols: KCS1 | KCS1 (3-KETOACYL-COA SYNTHASE 1); acyltransferase | chr1:57392-5897	
EX117802	1.740	weakly similar to (112)AT3G44020| Symbols: | thylakoid lumenal P17.1 protein | chr3:15812369-15813548 FORWARD [21828] 1 791 81	
AM389560	1.740	no similarity	
JCVI_17835	1.740	weakly similar to (142)AT1G23010| Symbols: LPR1 | LPR1 (LOW PHOSPHATE ROOT1); copper ion binding / oxidoreductase, acting or	
ES267316	1.740	no similarity	
JCVI_13699	1.740	moderately similar to (239)AT4G20780| Symbols: | calcium-binding protein, putative | chr4:11133320-11133895 REVERSEweakly simila	
JCVI_2685	1.740	moderately similar to (434)AT3G62060| Symbols: | pectinacetylesterase family protein | chr3:22991098-22993709 FORWARD no origina	
JCVI_27658	1.739	moderately similar to (239)AT4G04210| Symbols: PUX4 | PUX4 (PLANT UBX DOMAIN-CONTAINING PROTEIN 4) | chr4:2030389-2	
CD821356	1.739	no similarity	
EE560808	1.739	no similarity	
JCVI_1023	1.739	highly similar to (508)AT3G27300| Symbols: G6PD5 | G6PD5 (GLUCOSE-6-PHOSPHATE DEHYDROGENASE 5) | chr3:10084555-100	
EE559323	1.739	no similarity	
JCVI_9726	1.739	moderately similar to (342)AT2G38660| Symbols: | tetrahydrofolate dehydrogenase/cyclohydrolase, putative | chr2:16173470-16175272 F	
JCVI_7550	1.739	highly similar to (502)AT5G58290| Symbols: RPT3 | RPT3 (root phototropism 3); ATPase | chr5:23586381-23588342 FORWARDmodera	
EE484256	1.739	no similarity	-1.696
EX049655	1.739	no similarity	
EL588204	1.738	weakly similar to (151)AT5G25350| Symbols: EBF2 | EBF2 (EIN3-BINDING F BOX PROTEIN 2) | chr5:8794845-8796885 REVERSE [1	

ES920146	1.738	moderately similar to (327)AT5G57300| Symbols: | UbiE/COQ5 methyltransferase family protein | chr5:23225931-23227837 REVERSE |	
JCVI_30940	1.738	no original description	-1.449
ES912842	1.738	very weakly similar to (86.3)AT5G59370| Symbols: ACT4 | ACT4 (ACTIN 4) | chr5:23967335-23968812 FORWARDvery weakly similar	-2.194
EX113721	1.738	no similarity	
DY003375	1.738	no similarity	
JCVI_33911	1.738	moderately similar to (423)AT1G04120| Symbols: MRP5, ATMRP5 | ATMRP5 (Arabidopsis thaliana multidrug resistance-associated pro	
JCVI_356	1.737	highly similar to (514)AT1G17470| Symbols: ATDRG, ATDRG1 | ATDRG1 (ARABIDOPSIS THALIANA DEVELOPMENTALLY REG	
ES912996	1.737	moderately similar to (224)AT2G45290| Symbols: | transketolase, putative | chr2:18679811-18682663 FORWARDmoderately similar to (	
JCVI_37159	1.737	weakly similar to (117)AT3G04470| Symbols: | similar to ankyrin repeat family protein [Arabidopsis thaliana] (TAIR:AT1G04780.1); sim	-1.194
JCVI_27839	1.737	no original description	1.274
JCVI_41220	1.737	no original description	
ES981481	1.737	weakly similar to (142)AT1G17720| Symbols: | ATB BETA (Arabidopsis thaliana serine/threonine protein phosphatase 2A 55 kDa regula	
EE478017	1.737	moderately similar to (215)AT3G61860| Symbols: RSP31, ATRSP31 | ATRSP31 (ARGININE/SERINE-RICH SPLICING FACTOR 31);	
JCVI_26151	1.736	moderately similar to (215)AT2G45940| Symbols: | similar to unknown protein [Arabidopsis thaliana] (TAIR:AT4G16080.1); contains In	
JCVI_38743	1.736	moderately similar to (237)AT1G67840| Symbols: | ATP-binding region, ATPase-like domain-containing protein | chr1:25437819-254405	
EV071983	1.736	weakly similar to (125)AT5G65990| Symbols: | amino acid transporter family protein | chr5:26412181-26413547 FORWARD [21443] 1 5	
EL589299	1.736	moderately similar to (254)AT3G29670| Symbols: | transferase family protein | chr3:11530431-11531786 FORWARD [20863]	
JCVI_35676	1.736	moderately similar to (252)AT4G27470| Symbols: | zinc finger (C3HC4-type RING finger) family protein | chr4:13735582-13736313 FO	
ES987693	1.736	very weakly similar to (96.3)AT1G65270| Symbols: | similar to hypothetical protein [Vitis vinifera] (GB:CAN71035.1) | chr1:24245518-24	-2.525
JCVI_2194	1.736	moderately similar to (419)AT1G03900| Symbols: ATNAP4 | ATNAP4 (ARABIDOPSIS THALIANA NON-INTRINSIC ABC PROTEIN	
EV110632	1.736	no similarity	
JCVI_24168	1.736	very weakly similar to (96.3)AT1G25055| Symbols: | Identical to F-box/Kelch-repeat protein At1g24795/At1g24885/At1g25056/At1g2514	
JCVI_2098	1.736	moderately similar to (367)AT5G05170| Symbols: CESA3, IXR1, ATCESA3, ATH-B, CEV1 | CESA3 (CELLULOSE SYNTHASE 3); cel	
AM388498	1.735	weakly similar to (133)AT1G19400| Symbols: | similar to unknown protein [Arabidopsis thaliana] (TAIR:AT1G75180.3); similar to unkn	
JCVI_34439	1.735	moderately similar to (343)AT3G53830| Symbols: | regulator of chromosome condensation (RCC1) family protein / UVB-resistance prote	
JCVI_5620	1.735	very weakly similar to (91.7)AT4G38710| Symbols: | glycine-rich protein | chr4:18078135-18079996 REVERSE no original description	
EE450006	1.735	moderately similar to (210)AT1G48790| Symbols: | mov34 family protein | chr1:18047593-18051095 REVERSE [20170]	
JCVI_19273	1.735	moderately similar to (234)AT1G27390| Symbols: TOM20-2 | TOM20-2 (TRANSLOCASE OUTER MEMBRANE 20-2) | chr1:9513456-	
JCVI_6150	1.735	weakly similar to (182)AT1G32130| Symbols: | similar to IWS1 C-terminus family protein [Arabidopsis thaliana] (TAIR:AT4G19000.1);	
AT001694	1.735	weakly similar to (190)AT5G46700| Symbols: TET1, TRN2 | TET1/TRN2 (TETRASPANIN2) | chr5:18968262-18969666 FORWARD [1	
JCVI_24796	1.735	moderately similar to (310)AT1G21570| Symbols: | zinc finger (CCCH-type) family protein | chr1:7557811-7560272 REVERSE no origin	
JCVI_41727	1.735	moderately similar to (421)AT1G54115| Symbols: | cation exchanger, putative | chr1:20205786-20207720 FORWARD no original descrip	
JCVI_11175	1.735	moderately similar to (369)AT3G02260| Symbols: DOC1, TIR3, UMB1, LPR1, ASA1, CRM1, BIG | BIG (DARK OVER-EXPRESSION	
JCVI_1750	1.735	weakly similar to (165)AT5G64080| Symbols: | protease inhibitor/seed storage/lipid transfer protein (LTP) family protein | chr5:25662701	-1.207
JCVI_39684	1.735	moderately similar to (239)AT4G24590| Symbols: | similar to unknown protein [Arabidopsis thaliana] (TAIR:AT5G49710.3); similar to u	
JCVI_16241	1.735	highly similar to (845)AT1G21680| Symbols: | similar to unknown protein [Arabidopsis thaliana] (TAIR:AT1G21670.1); similar to hypot	
JCVI_6144	1.735	moderately similar to (278)AT5G54680| Symbols: ILR3 | ILR3 (IAA-LEUCINE RESISTANT3); DNA binding / transcription factor | chr5	
JCVI_42303	1.734	weakly similar to (112)AT3G21180| Symbols: ATACA9, ACA9 | ACA9 (autoinhibited Ca2+ -ATPase 9); calcium-transporting ATPase/ c	
JCVI_34653	1.734	weakly similar to (120)AT1G47210| Symbols: CYCA3;2 | CYCA3;2; cyclin-dependent protein kinase | chr1:17303476-17305024 FORWA	
JCVI_16880	1.734	weakly similar to (181)AT3G15030| Symbols: MEE35, TCP4 | TCP4 (TCP FAMILY TRANSCRIPTION FACTOR 4); transcription facto	
AM387783	1.733	very weakly similar to (95.5)AT1G24360| Symbols: | 3-oxoacyl-(acyl-carrier protein) reductase, chloroplast / 3-ketoacyl-acyl carrier protei	
EE524629	1.733	moderately similar to (228)AT1G74120| Symbols: | mitochondrial transcription termination factor-related / mTERF-related | chr1:278755	
JCVI_2250	1.733	moderately similar to (405)AT2G29330| Symbols: TRI | TRI (TROPINONE REDUCTASE); oxidoreductase | chr2:12601681-12603273 F	
JCVI_1682	1.733	moderately similar to (352)AT5G08680| Symbols: | ATP synthase beta chain, mitochondrial, putative | chr5:2821993-2824684 FORWAR	
JCVI_41012	1.733	weakly similar to (174)AT5G35690| Symbols: | similar to zinc ion binding [Arabidopsis thaliana] (TAIR:AT1G55915.1); similar to unnan	
DY028331	1.733	moderately similar to (281)AT5G49555| Symbols: | amine oxidase-related | chr5:20124637-20127828 REVERSE [18978]	-1.742
DY006360	1.733	no similarity	
JCVI_32889	1.733	moderately similar to (438)AT5G23575| Symbols: | transmembrane protein, putative | chr5:7946566-7950044 FORWARD no original des	
ES943365	1.733	moderately similar to (219)AT2G03710| Symbols: AGL3, SEP4 | SEP4 (SEPALLATA4); DNA binding | chr2:1129619-1131239 FORWA	
JCVI_20390	1.733	moderately similar to (315)AT3G54480| Symbols: SKP5, SKIP5 | SKIP5 (SKP1-INTERACTING PARTNER 5) | chr3:20183496-2018430	
EV206025	1.733	no similarity	
JCVI_12880	1.732	moderately similar to (475)AT2G32070| Symbols: | CCR4-NOT transcription complex protein, putative | chr2:13647906-13648733 REVE	
JCVI_7959	1.732	highly similar to (573)AT3G63250| Symbols: HMT-2, ATHMT-2, HMT2 | ATHMT-2/HMT-2/HMT2 (HOMOCYSTEINE METHYLTRA	
JCVI_39253	1.732	moderately similar to (483)AT5G64320| Symbols: | pentatricopeptide (PPR) repeat-containing protein | chr5:25740473-25742665 REVER	
JCVI_5668	1.732	moderately similar to (351)AT1G80090| Symbols: | CBS domain-containing protein | chr1:30134997-30136748 FORWARD no original d	
ES992032	1.732	weakly similar to (138)AT5G03030| Symbols: | DNAJ heat shock N-terminal domain-containing protein | chr5:708477-709511 REVERSE	
JCVI_32731	1.732	moderately similar to (396)AT5G46060| Symbols: | similar to unknown protein [Arabidopsis thaliana] (TAIR:AT5G24600.1); similar to u	
JCVI_8714	1.732	moderately similar to (310)AT1G73380| Symbols: | similar to unnamed protein product [Vitis vinifera] (GB:CAO62045.1); contains Inter	
ES904760	1.731	no similarity	
ES904376	1.731	moderately similar to (233)AT3G58000| Symbols: | VQ motif-containing protein | chr3:21485927-21486454 FORWARD [21432] 19 717	
CX193479	1.731	weakly similar to (107)AT3G13050| Symbols: | transporter-related | chr3:4176873-4178875 FORWARD [16807]	
EX077007	1.731	moderately similar to (217)AT4G36380| Symbols: ROT3 | ROT3 (ROTUNDIFOLIA 3); oxygen binding / steroid hydroxylase | chr4:1718	
JCVI_29098	1.731	moderately similar to (398)AT5G48030| Symbols: GFA2 | GFA2 (GAMETOPHYTIC FACTOR 2); heat shock protein binding / unfolded	
EV159733	1.731	weakly similar to (109)AT2G38370| Symbols: | similar to unknown protein [Arabidopsis thaliana] (TAIR:AT3G51720.1); similar to unna	
JCVI_28526	1.731	moderately similar to (238)AT2G04050| Symbols: | MATE efflux family protein | chr2:1337383-1339267 REVERSE no original descripti	
EX051964	1.731	no similarity	
JCVI_12034	1.730	weakly similar to (145)AT1G18730| Symbols: | similar to unknown [Populus trichocarpa] (GB:ABK95263.1) | chr1:6460617-6461456 FO	
EE533748	1.730	moderately similar to (263)AT4G25800| Symbols: | calmodulin-binding protein | chr4:13125233-13127774 FORWARD [20175]	
ES266268	1.730	weakly similar to (134)AT1G23170| Symbols: | similar to unknown protein [Arabidopsis thaliana] (TAIR:AT1G70770.1); similar to hypot	
JCVI_23160	1.730	highly similar to (537)AT3G14410| Symbols: | transporter-related | chr3:4815989-4817859 REVERSE no original description	
EV167741	1.729	moderately similar to (311)AT4G10060| Symbols: | catalytic | chr4:6289352-6295254 FORWARD [21486] 115 775 775	
EV088608	1.729	no similarity	
EL593016	1.729	moderately similar to (313)AT1G77170| Symbols: | pentatricopeptide (PPR) repeat-containing protein | chr1:29003027-29004430 REVER	
EV012106	1.729	no similarity	
JCVI_29501	1.729	moderately similar to (333)AT1G07380| Symbols: | ceramidase family protein | chr1:2264827-2268304 REVERSE no original description	
EX017555	1.729	moderately similar to (377)AT5G08610| Symbols: | DEAD box RNA helicase (RH26) | chr5:2790342-2794060 FORWARDweakly simila	
JCVI_30799	1.729	weakly similar to (118)AT4G11960| Symbols: PGRL1B | PGRL1B (PGR5-LIKE B) | chr4:7175334-7177703 REVERSE no original descr	
JCVI_1137	1.729	moderately similar to (452)AT1G08450| Symbols: CRT3 | CRT3 (CALRETICULIN 3); calcium ion binding | chr1:2668005-2671797 REV	
DY017191	1.729	very weakly similar to (92.0)AT3G47680| Symbols: | DNA binding | chr3:17588468-17589376 REVERSE [18975] 1 639 669	
JCVI_7307	1.729	weakly similar to (176)AT1G16790| Symbols: | ribosomal protein-related | chr1:5744631-5745065 FORWARD no original description	
JCVI_41472	1.728	moderately similar to (391)AT3G54650| Symbols: | F-box family protein (FBL17) | chr3:20236982-20239860 REVERSE no original desc	

EV215154	1.728	moderately similar to (223)AT5G03200| Symbols: | zinc finger (C3HC4-type RING finger) family protein | chr5:760449-761666 REVERS	
ES931666	1.727	no similarity	
JCVI_3945	1.727	moderately similar to (374)AT4G14430| Symbols: | enoyl-CoA hydratase/isomerase family protein | chr4:8304906-8305628 REVERSE no	
EE451996	1.727	no similarity	
JCVI_6904	1.727	moderately similar to (434)AT1G76110| Symbols: | high mobility group (HMG1/2) family protein / ARID/BRIGHT DNA-binding domain	
JCVI_8940	1.727	moderately similar to (346)AT4G11740| Symbols: SAY1 | SAY1 | chr4:7071949-7075250 FORWARD no original description	
EE530651	1.727	no similarity	-1.702
JCVI_5991	1.727	moderately similar to (205)AT5G41620| Symbols: | Identical to Uncharacterized protein At5g41620 [Arabidopsis Thaliana] (GB:Q66GQ2	
JCVI_32954	1.726	weakly similar to (192)AT1G58160| Symbols: | jacalin lectin family protein | chr1:21537422-21538105 FORWARD no original descriptio	
JCVI_6507	1.726	moderately similar to (259)AT5G08770| Symbols: | similar to unnamed protein product [Vitis vinifera] (GB:CAO71661.1); similar to hyp	
JCVI_32114	1.726	no original description	
EE564855	1.726	no similarity	
JCVI_32510	1.726	moderately similar to (276)AT3G15010| Symbols: | RNA recognition motif (RRM)-containing protein | chr3:5052851-5054065 FORWAR	
JCVI_27906	1.726	moderately similar to (391)AT2G32040| Symbols: | integral membrane transporter family protein | chr2:13642193-13644669 FORWARD	
CN725895	1.726	very weakly similar to (85.9)DHLE_RAPSA [15715]	
JCVI_13615	1.726	no original description	
EE468985	1.726	weakly similar to (125)AT1G08315| Symbols: | armadillo/beta-catenin repeat family protein | chr1:2620497-2621474 REVERSE [20156]	-1.785
AM386147	1.726	no similarity	
EV056382	1.726	weakly similar to (167)AT5G59050| Symbols: | unknown protein | chr5:23859075-23859745 REVERSE [21442] 1 607 624	
ES901713	1.725	moderately similar to (390)AT5G45970| Symbols: ROP7, ATRAC2, ARAC2, ATROP7 | ARAC2 (RHO-RELATED PROTEIN FROM PL	
JCVI_36202	1.725	moderately similar to (434)AT1G63170| Symbols: | zinc finger (C3HC4-type RING finger) family protein | chr1:23429239-23430738 FOF	
ES913539	1.725	no similarity	
ES906343	1.725	moderately similar to (432)AT5G55700| Symbols: BMY6, BAM4 | BAM4/BMY6 (BETA-AMYLASE 4); beta-amylase | chr5:22569099-2	
EV109739	1.725	very weakly similar to (84.0)AT1G22550| Symbols: | proton-dependent oligopeptide transport (POT) family protein | chr1:7966597-79685	
EV194351	1.725	moderately similar to (305)AT1G54130| Symbols: AT-RSH3, RSH3 | RSH3 (RELA/SPOT HOMOLOG 3); catalytic | chr1:20214845-2021	
JCVI_38552	1.725	no original description	
EV007482	1.725	very weakly similar to (81.6)AT1G22570| Symbols: | proton-dependent oligopeptide transport (POT) family protein | chr1:7976609-79785	
JCVI_18329	1.724	moderately similar to (324)AT5G42220| Symbols: | ubiquitin family protein | chr5:16890190-16894683 FORWARD no original descriptic	
EX088469	1.724	moderately similar to (245)AT5G54130| Symbols: | calcium-binding EF hand family protein | chr5:21980126-21982067 FORWARD [218	
JCVI_31201	1.724	very weakly similar to (81.6)AT2G39550| Symbols: PGGT-I, GGB, ATGGT-IB | ATGGT-IB (GERANYLGERANYLTRANSFERASE-I E	
JCVI_34990	1.724	moderately similar to (294)AT3G22970| Symbols: | similar to unknown protein [Arabidopsis thaliana] (TAIR:AT4G14620.1); similar to u	
EV001722	1.724	no similarity	
JCVI_6145	1.724	moderately similar to (454)AT5G48230| Symbols: EMB1276, ACAT2 | ACAT2/EMB1276 (ACETOACETYL-COA THIOLASE 2); acety	
JCVI_905	1.724	moderately similar to (471)AT4G14930| Symbols: | acid phosphatase survival protein SurE, putative | chr4:8538827-8541201 FORWARD	
DY005478	1.724	moderately similar to (255)AT1G76120| Symbols: | tRNA pseudouridine synthase family protein | chr1:28564118-28565188 REVERSE [	
JCVI_29742	1.724	moderately similar to (293)AT5G65170| Symbols: | VQ motif-containing protein | chr5:26058444-26059532 FORWARD no original desc	-3.163
EE455098	1.724	moderately similar to (211)AT2G24830| Symbols: | zinc finger (CCCH-type) family protein / D111/G-patch domain-containing protein | c	
JCVI_9575	1.723	weakly similar to (101)AT3G27240| Symbols: | cytochrome c1, putative | chr3:10057381-10059607 REVERSE no original description	
JCVI_9817	1.723	weakly similar to (187)AT3G19910| Symbols: | zinc finger (C3HC4-type RING finger) family protein | chr3:6926503-6929330 FORWAR	
JCVI_5869	1.723	moderately similar to (343)AT3G01910| Symbols: AT-SO, ATSO, SOX | SOX (SULFITE OXIDASE) | chr3:314926-317060 REVERSEw	
JCVI_14840	1.723	moderately similar to (431)AT5G22780| Symbols: | adaptin family protein | chr5:7590103-7597831 REVERSE no original description	
EV028698	1.723	weakly similar to (167)AT3G54930| Symbols: | serine/threonine protein phosphatase 2A (PP2A) regulatory subunit B', putative | chr3:203	
RC_EX126842	1.723	no similarity	
JCVI_14506	1.723	highly similar to (586)AT2G23030| Symbols: SNRK2-9, SNRK2.9 | SNRK2-9/SNRK2.9 (SNF1-RELATED PROTEIN KINASE 2.9); kina	
CD826154	1.723	moderately similar to (218)AT3G24040| Symbols: | glycosyltransferase family 14 protein / core-2/I-branching enzyme family protein | chr	
JCVI_13002	1.723	weakly similar to (150)AT2G44090| Symbols: | similar to unknown protein [Arabidopsis thaliana] (TAIR:AT3G59910.1); similar to hypot	
EE458817	1.723	weakly similar to (145)AT3G19950| Symbols: | zinc finger (C3HC4-type RING finger) family protein | chr3:6942859-6943845 FORWAR	
JCVI_32564	1.723	weakly similar to (179)AT2G38090| Symbols: | myb family transcription factor | chr2:15952356-15953853 FORWARD no original descri	1.407
EE443270	1.723	very weakly similar to (96.3)AT3G13930| Symbols: | dihydrolipoamide S-acetyltransferase, putative | chr3:4596247-4600150 FORWARD	
JCVI_15867	1.723	weakly similar to (130)AT2G38360| Symbols: | prenylated rab acceptor (PRA1) family protein | chr2:16076918-16077580 REVERSE no	
JCVI_12802	1.723	moderately similar to (309)AT5G51890| Symbols: | peroxidase | chr5:21108389-21109561 REVERSEweakly similar to (101)PERX_NIC	1.604
JCVI_34473	1.722	moderately similar to (222)AT5G35400| Symbols: | tRNA pseudouridine synthase family protein | chr5:13616646-13619470 REVERSE n	
EE446014	1.722	weakly similar to (126)AT3G06760| Symbols: | Identical to Protein DEHYDRATION-INDUCED 19 homolog 4 (DI19-4) [Arabidopsis T	1.133
ES928164	1.722	no similarity	
EV158810	1.722	weakly similar to (139)AT5G06460| Symbols: ATUBA2 | ATUBA2 (Arabidopsis thaliana ubiquitin activating enzyme 2); ubiquitin activat	
ES913707	1.722	moderately similar to (415)AT4G31920| Symbols: ARR10 | ARR10 (ARABIDOPSIS RESPONSE REGULATOR 10); transcription factor	
JCVI_22253	1.722	moderately similar to (335)AT2G43590| Symbols: | chitinase, putative | chr2:18088669-18089826 REVERSEmoderately similar to (358)	
JCVI_19068	1.722	moderately similar to (319)AT3G02760| Symbols: | histidyl-tRNA synthetase, putative / histidine--tRNA ligase, putative | chr3:597595-59	
ES942457	1.722	no similarity	
EV075582	1.721	no similarity	
JCVI_41128	1.721	no original description	
JCVI_7960	1.721	moderately similar to (416)AT1G05380| Symbols: | similar to PHD finger transcription factor, putative [Arabidopsis thaliana] (TAIR:AT4	
JCVI_36377	1.721	highly similar to (611)AT3G60120| Symbols: | glycosyl hydrolase family 1 protein | chr3:22217322-22219927 FORWARDmoderately sim	-1.744
JCVI_3660	1.721	moderately similar to (421)AT4G17510| Symbols: UCH3 | UCH3; ubiquitin thiolesterase | chr4:9767127-9768661 REVERSE no original	
EE457879	1.721	weakly similar to (178)AT3G09010| Symbols: | protein kinase family protein | chr3:2750290-2752091 FORWARD [20179]	
JCVI_25648	1.721	moderately similar to (353)AT5G04460| Symbols: | protein binding / zinc ion binding | chr5:1260010-1263696 FORWARD no original de	-1.767
JCVI_36666	1.721	moderately similar to (394)AT4G37640| Symbols: ACA2 | ACA2 (CALCIUM ATPASE 2); calmodulin binding | chr4:17683219-1768680	
JCVI_11315	1.721	moderately similar to (422)AT5G54170| Symbols: | similar to CP5 [Arabidopsis thaliana] (TAIR:AT1G64720.1); similar to putative nodu	
JCVI_26690	1.721	moderately similar to (309)AT1G48580| Symbols: | similar to unnamed protein product [Vitis vinifera] (GB:CAO67053.1) | chr1:1796207	
EV105902	1.720	moderately similar to (205)AT5G07920| Symbols: ATDGK1 | DGK1 (DIACYLGLYCEROL KINASE 1, DIACYLGLYCEROL KINASE1	
JCVI_19817	1.720	no original description	
EV040719	1.720	very weakly similar to (97.8)AT1G16500| Symbols: | similar to unknown protein [Arabidopsis thaliana] (TAIR:AT1G79160.1); similar to u	
JCVI_37391	1.720	highly similar to (527)AT2G19670| Symbols: | protein arginine N-methyltransferase, putative | chr2:8506360-8508196 REVERSE no orig	
JCVI_27765	1.720	moderately similar to (313)AT3G09760| Symbols: | zinc finger (C3HC4-type RING finger) family protein | chr3:2992980-2995456 REVE	
JCVI_27929	1.720	moderately similar to (371)AT3G17660| Symbols: AGD15 | AGD15 (ARF-GAP DOMAIN 15); DNA binding | chr3:6037723-6039098 FO	2.284
JCVI_11117	1.720	moderately similar to (307)AT5G24460| Symbols: | hydrolase | chr5:8354818-8355720 FORWARD no original description	
EX135595	1.720	no similarity	
ES958403	1.720	no similarity	
EV171255	1.719	weakly similar to (125)AT2G22250| Symbols: ATAAT, AAT, MEE17 | AAT/ATAAT/MEE17 (maternal effect embryo arrest 17); asparta	
JCVI_23095	1.719	highly similar to (505)AT4G04020| Symbols: FIB | FIB (FIBRILLIN); structural molecule | chr4:1932159-1933544 FORWARDmoderatel	
EV066944	1.719	moderately similar to (382)AT1G60560| Symbols: | SWIM zinc finger family protein | chr1:22312456-22314942 REVERSE [21443]	

EX131670	1.719	moderately similar to (457)AT2G25010| Symbols: | similar to unknown protein [Arabidopsis thaliana] (TAIR:AT1G17930.1); similar to u	
JCVI_11815	1.719	moderately similar to (312)AT4G16710| Symbols: | glycosyltransferase family protein 28 | chr4:9398840-9399640 FORWARD no origina	
JCVI_2172	1.719	moderately similar to (379)AT3G14680| Symbols: CYP72A14 | CYP72A14 (cytochrome P450, family 72, subfamily A, polypeptide 14); c	
EX141949	1.719	moderately similar to (296)AT5G20350| Symbols: TIP1 | TIP1 (TIP GROWTH DEFECTIVE 1) | chr5:6876774-6881104 FORWARD [21	
AM389039	1.718	moderately similar to (272)AT5G08200| Symbols: | peptidoglycan-binding LysM domain-containing protein | chr5:2638386-2640509 FOI	
EV089399	1.718	no similarity	
ES940156	1.718	weakly similar to (113)AT3G18620| Symbols: | zinc finger (DHHC type) family protein | chr3:6408855-6410722 FORWARD [21391]	
JCVI_31468	1.718	moderately similar to (469)AT5G67480| Symbols: BT4 | BT4 (BTB AND TAZ DOMAIN PROTEIN 4); protein binding | chr5:26948281-	
JCVI_15118	1.718	no original description	
JCVI_4360	1.718	moderately similar to (266)AT4G27070| Symbols: TSB2 | TSB2 (TRYPTOPHAN SYNTHASE BETA-SUBUNIT); tryptophan synthase |	
RC_H74357	1.718	no similarity	
EX098626	1.717	moderately similar to (290)AT5G53050| Symbols: | hydrolase, alpha/beta fold family protein | chr5:21527760-21530658 REVERSE [218	
EX065546	1.717	very weakly similar to (80.5)ERG11_BRANA [21815]	
CD831313	1.717	weakly similar to (175)AT3G52740| Symbols: | similar to unknown protein [Arabidopsis thaliana] (TAIR:AT3G44450.1); similar to unnar	
EE533696	1.717	no similarity	
JCVI_6915	1.716	moderately similar to (339)AT1G76790| Symbols: | O-methyltransferase family 2 protein | chr1:28827249-28828524 REVERSEweakly si	
JCVI_34502	1.716	weakly similar to (199)AT5G61340| Symbols: | similar to unknown protein [Arabidopsis thaliana] (TAIR:AT1G26650.1); similar to hypot	
CD837234	1.716	moderately similar to (270)AT5G63640| Symbols: | VHS domain-containing protein / GAT domain-containing protein | chr5:25496161-25	
JCVI_4525	1.716	moderately similar to (464)AT5G41970| Symbols: | similar to unknown protein [Arabidopsis thaliana] (TAIR:AT3G49320.1); similar to u	
EX115162	1.715	moderately similar to (387)AT3G15800| Symbols: | glycosyl hydrolase family 17 protein | chr3:5345058-5346995 FORWARDweakly sim	
EV147308	1.715	moderately similar to (235)AT3G18490| Symbols: | aspartyl protease family protein | chr3:6349096-6350598 REVERSE [21482]	
EE535876	1.715	moderately similar to (229)AT3G52030| Symbols: | F-box family protein / WD-40 repeat family protein | chr3:19312850-19315205 FORV	
JCVI_14320	1.715	moderately similar to (344)AT3G18940| Symbols: | clast3-related | chr3:6527087-6529056 REVERSE no original description	
JCVI_40332	1.715	weakly similar to (179)AT4G01130| Symbols: | acetylesterase, putative | chr4:485868-488007 FORWARDvery weakly similar to (80.5)ES	
EV195116	1.715	moderately similar to (224)AT5G26860| Symbols: LON_ARA_ARA | LON_ARA_ARA (Lon protease homolog gene); ATP binding / ATI	
JCVI_33063	1.715	moderately similar to (289)AT1G69690| Symbols: | TCP family transcription factor, putative | chr1:26220112-26221089 FORWARD no c	
EV210184	1.715	moderately similar to (253)AT2G41900| Symbols: | zinc finger (CCCH-type) family protein | chr2:17498430-17500580 FORWARD [214	
EV152610	1.715	no similarity	
EE473893	1.715	weakly similar to (172)AT1G27360| Symbols: | squamosa promoter-binding protein-like 11 (SPL11) | chr1:9502126-9503702 FORWARD	
JCVI_20161	1.714	no original description	
EE561009	1.714	weakly similar to (165)AT1G14600| Symbols: | DNA binding / transcription factor | chr1:5001180-5003365 REVERSE [20153] 44 628 64	
DY025783	1.714	moderately similar to (321)AT5G16370| Symbols: | AMP-binding protein, putative | chr5:5356826-5358484 REVERSEweakly similar to (	
JCVI_39226	1.714	highly similar to (647)AT1G55680| Symbols: | WD-40 repeat family protein | chr1:20808654-20810960 REVERSE no original descriptio	
EX039908	1.714	moderately similar to (267)AT1G22770| Symbols: FB, GI | GI (GIGANTEA); binding | chr1:8062387-8067436 FORWARDweakly similar	-1.773
ES907950	1.714	weakly similar to (177)AT2G16700| Symbols: ADF5 | ADF5 (ACTIN DEPOLYMERIZING FACTOR 5); actin binding | chr2:7251799-72	
EX025604	1.713	weakly similar to (155)AT3G24515| Symbols: UBC37 | UBC37 (UBIQUITIN-CONJUGATING ENZYME 37); ubiquitin-protein ligase | c	
CX195618	1.713	weakly similar to (140)AT3G62840| Symbols: | similar to small nuclear ribonucleoprotein D2, putative / snRNP core protein D2, putative	
JCVI_20841	1.713	highly similar to (758)AT5G54860| Symbols: | integral membrane transporter family protein | chr5:22301947-22304251 FORWARD no o	
JCVI_3093	1.713	moderately similar to (261)AT5G14120| Symbols: | nodulin family protein | chr5:4556310-4558449 FORWARD no original description	
JCVI_2013	1.712	highly similar to (629)AT5G45020| Symbols: | similar to unknown protein [Arabidopsis thaliana] (TAIR:AT4G19880.1); similar to Intrac	-1.723
JCVI_27923	1.712	moderately similar to (263)AT5G66160| Symbols: ATRMR1, JR700 | JR700 (Arabidopsis thaliana receptor homology region transmembra	
JCVI_14772	1.712	moderately similar to (228)AT5G67310| Symbols: CYP81G1 | CYP81G1 (cytochrome P450, family 81, subfamily G, polypeptide 1); oxyg	2.415
JCVI_25747	1.712	moderately similar to (453)AT5G08670| Symbols: | ATP synthase beta chain 1, mitochondrial | chr5:2818396-2821150 REVERSEmodera	
AM390008	1.712	no similarity	
JCVI_10825	1.712	moderately similar to (251)AT1G30320| Symbols: | remorin family protein | chr1:10680330-10682834 FORWARD no original descriptior	
JCVI_2320	1.712	moderately similar to (370)AT1G07450| Symbols: | tropinone reductase, putative / tropine dehydrogenase, putative | chr1:2288035-22892	
DY003547	1.712	no similarity	
JCVI_17375	1.712	no original description	
EX088937	1.712	weakly similar to (110)AT5G26210| Symbols: | PHD finger family protein | chr5:9158569-9160224 REVERSE [21823]	
JCVI_24397	1.711	highly similar to (686)AT3G58060| Symbols: | cation efflux family protein / metal tolerance protein, putative (MTPc3) | chr3:21508755-2	
JCVI_20812	1.711	moderately similar to (255)AT1G74890| Symbols: ARR15 | ARR15 (RESPONSE REGULATOR 15); transcription regulator | chr1:281352	
JCVI_2374	1.711	moderately similar to (233)AT2G02060| Symbols: | transcription factor | chr2:495690-497608 FORWARD no original description	
JCVI_17920	1.711	highly similar to (630)AT2G40950| Symbols: BZIP17 | BZIP17; DNA binding / transcription activator/ transcription factor | chr2:1709490	
JCVI_23178	1.711	very weakly similar to (97.1)AT2G17033| Symbols: | pentatricopeptide (PPR) repeat-containing protein | chr2:7408287-7410289 FORWAI	
RC_EX036945	1.711	no similarity	1.710
JCVI_25862	1.710	highly similar to (525)AT5G61000| Symbols: | replication protein, putative | chr5:24566908-24569867 REVERSE no original description	
JCVI_5722	1.710	moderately similar to (407)AT1G60710| Symbols: ATB2 | ATB2; oxidoreductase | chr1:22358738-22360292 REVERSEmoderately simila	
JCVI_16009	1.710	moderately similar to (278)AT2G21370| Symbols: | xylulose kinase, putative | chr2:9144662-9146653 REVERSE no original description	
JCVI_17927	1.710	moderately similar to (372)AT1G35420| Symbols: | dienelactone hydrolase family protein | chr1:13026298-13027441 FORWARD no orig	
EL591243	1.710	very weakly similar to (89.0)AT2G35040| Symbols: | AICARFT/IMPCHase bienzyme family protein | chr2:14772426-14775348 REVERS	-1.397
JCVI_33694	1.710	weakly similar to (120)AT2G26830| Symbols: EMB1187 | EMB1187 (EMBRYO DEFECTIVE 1187); kinase/ phosphotransferase, alcoho	
JCVI_7622	1.710	moderately similar to (212)AT3G55960| Symbols: | NLI interacting factor (NIF) family protein | chr3:20771776-20773871 REVERSE no	
EE462560	1.710	moderately similar to (369)AT5G43380| Symbols: TOPP6 | TOPP6 (Type one serine/threonine protein phosphatase 6); protein serine/thre	
EV148865	1.710	no similarity	
EE465948	1.710	no similarity	
JCVI_2597	1.709	weakly similar to (184)AT1G19740| Symbols: | ATP-dependent protease La (LON) domain-containing protein | chr1:6824387-6825223 F	
EE418030	1.709	very weakly similar to (96.3)AT4G19450| Symbols: | nodulin-related | chr4:10606549-10609229 FORWARD [20146] 24 577 577	
EX091324	1.709	no similarity	-1.451
JCVI_23913	1.709	moderately similar to (326)AT3G59830| Symbols: | ankyrin protein kinase, putative | chr3:22113983-22116300 REVERSE no original des	-1.254
JCVI_21210	1.709	moderately similar to (492)AT2G40690| Symbols: SFD1, GLY1 | GLY1 (SUPPRESSOR OF FATTY ACID DESATURASE DEFICIENC	
EV103759	1.709	weakly similar to (189)AT1G07440| Symbols: | tropinone reductase, putative / tropine dehydrogenase, putative | chr1:2286697-2287662 R	
JCVI_26384	1.709	moderately similar to (370)AT1G47530| Symbols: | ripening-responsive protein, putative | chr1:17454164-17456550 FORWARD no origi	
EE530887	1.709	moderately similar to (278)AT1G17790| Symbols: | DNA-binding bromodomain-containing protein | chr1:6125525-6127269 REVERSE [	
AI352839	1.709	weakly similar to (135)AT4G18100| Symbols: | 60S ribosomal protein L32 (RPL32A) | chr4:10035727-10036487 REVERSE [1285] 9 314	
EE558433	1.709	no similarity	1.808
JCVI_1267	1.708	moderately similar to (445)AT1G17290| Symbols: ALAAT1 | ALAAT1 (ALANINE AMINOTRANSFERAS); alanine transaminase | chr1:	
JCVI_27852	1.708	no original description	
JCVI_4858	1.708	highly similar to (638)AT1G04140| Symbols: | transducin family protein / WD-40 repeat family protein | chr1:1075991-1080320 REVERS	
JCVI_22357	1.708	moderately similar to (419)AT1G30740| Symbols: | FAD-binding domain-containing protein | chr1:10903011-10904612 FORWARD no o	
JCVI_36318	1.708	moderately similar to (405)AT1G11130| Symbols: SCM, SRF9, SUB | SUB (STRUBBELIG); protein binding | chr1:3723135-3727178 FO	
JCVI_34397	1.708	moderately similar to (233)AT4G30600| Symbols: | signal recognition particle receptor alpha subunit family protein | chr4:14938148-1494	

ES987215	1.707	weakly similar to (194)AT1G73180| Symbols: | eukaryotic translation initiation factor-related | chr1:27521996-27524675 FORWARD [21	
DW999192	1.707	no similarity	
EE433462	1.707	very weakly similar to (94.7)AT2G19790| Symbols: | clathrin adaptor complex small chain family protein | chr2:8534384-8535477 FORW.	
EX132946	1.707	weakly similar to (185)AT2G20110| Symbols: | tesmin/TSO1-like CXC domain-containing protein | chr2:8691577-8693951 FORWARD [	-2.257
JCVI_41524	1.707	moderately similar to (371)AT1G80070| Symbols: EMB158, EMB33, EMB177, EMB14, SUS2 | SUS2 (ABNORMAL SUSPENSOR 2) | c	
JCVI_39732	1.707	moderately similar to (393)AT5G17420| Symbols: CESA7, ATCESA7, MUR10, IRX3 | IRX3 (IRREGULAR XYLEM 3, MURUS 10); ce	
EE465921	1.707	very weakly similar to (91.7)AT2G23140| Symbols: | binding / ubiquitin-protein ligase | chr2:9852776-9855842 REVERSE [20188]	
DN962947	1.706	weakly similar to (142)AT1G32900| Symbols: | starch synthase, putative | chr1:11920562-11923486 REVERSEweakly similar to (117)S	
JCVI_37940	1.706	moderately similar to (314)AT4G39920| Symbols: TFC C, POR | POR (PORCINO); binding | chr4:18515876-18516913 FORWARD no o	
ES991997	1.706	weakly similar to (171)AT4G22320| Symbols: | similar to unknown protein [Arabidopsis thaliana] (TAIR:AT5G55210.1); similar to unna	
L46416	1.706	weakly similar to (145)AT1G29520| Symbols: | AWPM-19-like membrane family protein | chr1:10323721-10324511 FORWARD [132]	
ES911468	1.706	moderately similar to (454)AT3G15354| Symbols: SPA3 | SPA3 (SPA1-RELATED 3); signal transducer | chr3:5169334-5172487 REVER	
EE449986	1.706	weakly similar to (129)AT2G29420| Symbols: GST25, ATGSTU7 | ATGSTU7 (GLUTATHIONE S-TRANSFERASE 25); glutathione tra	
EX024132	1.705	moderately similar to (283)AT3G13790| Symbols: ATCWINV1, ATBFRUCT1 | ATBFRUCT1/ATCWINV1 (ARABIDOPSIS THALIAN.	-1.984
JCVI_23227	1.705	moderately similar to (336)AT1G17100| Symbols: | SOUL heme-binding family protein | chr1:5844759-5845532 FORWARD no original	-1.366
EX043962	1.705	moderately similar to (448)AT1G74750| Symbols: | pentatricopeptide (PPR) repeat-containing protein | chr1:28090461-28093028 FORW.	
EV159642	1.705	very weakly similar to (95.9)AT4G17620| Symbols: | glycine-rich protein | chr4:9822018-9824872 FORWARD [21484] 54 811 811	
JCVI_28403	1.705	weakly similar to (102)AT3G11890| Symbols: | similar to unknown protein [Arabidopsis thaliana] (TAIR:AT3G11860.1); similar to unna	
CN730287	1.705	weakly similar to (147)AT1G74680| Symbols: | exostosin family protein | chr1:28063189-28064645 FORWARD [15718]	
ES942411	1.705	no similarity	
ES966868	1.705	no similarity	
JCVI_36849	1.704	moderately similar to (246)AT1G79820| Symbols: SGB1 | SGB1; carbohydrate transmembrane transporter/ sugar:hydrogen ion symporter	
JCVI_17796	1.704	no original description	
ES941812	1.704	weakly similar to (196)AT4G27660| Symbols: | similar to RIN13 (RPM1 INTERACTING PROTEIN 13) [Arabidopsis thaliana] (TAIR:A	
JCVI_23707	1.703	highly similar to (633)AT5G03450| Symbols: | zinc finger (C3HC4-type RING finger) family protein | chr5:858953-862237 REVERSE nc	
EE442214	1.703	no similarity	
ES936287	1.703	weakly similar to (184)AT5G51590| Symbols: | DNA-binding protein-related | chr5:20974089-20976155 REVERSE [16815]	
EV008304	1.703	weakly similar to (185)AT5G05100| Symbols: | nucleic acid binding | chr5:1505462-1506941 REVERSE [21427]	
CN727851	1.703	moderately similar to (223)AT2G27490| Symbols: ATCOAE | ATCOAE; ATP binding / dephospho-CoA kinase | chr2:11755165-1175608	
CX194281	1.703	moderately similar to (300)AT1G79020| Symbols: | transcription factor-related | chr1:29732175-29734952 REVERSE [16807]	2.975
EE513961	1.703	weakly similar to (168)AT4G20390| Symbols: | integral membrane family protein | chr4:11007079-11007880 FORWARD [15713]	1.913
DW999372	1.703	weakly similar to (181)AT2G30700| Symbols: | similar to unknown protein [Arabidopsis thaliana] (TAIR:AT1G61900.1); similar to hypo	
JCVI_20605	1.703	weakly similar to (149)AT5G42020| Symbols: BIP2, BIP | BIP (LUMINAL BINDING PROTEIN); ATP binding | chr5:16824925-168277	
JCVI_30980	1.703	no original description	
CD828760	1.702	moderately similar to (318)AT1G07380| Symbols: | ceramidase family protein | chr1:2264827-2268304 REVERSE [13979]	
AM060266	1.702	no similarity	
JCVI_20090	1.702	moderately similar to (409)AT1G29820| Symbols: | similar to unknown protein [Arabidopsis thaliana] (TAIR:AT1G29830.2); similar to u	1.262
JCVI_37314	1.702	moderately similar to (336)AT3G55510| Symbols: | similar to unknown protein [Arabidopsis thaliana] (TAIR:AT2G18220.1); similar to u	
JCVI_34261	1.702	highly similar to (654)AT2G29730| Symbols: | UDP-glucoronosyl/UDP-glucosyl transferase family protein | chr2:12710729-12712132 FC	
JCVI_1064	1.702	weakly similar to (160)AT5G55730| Symbols: FLA1 | FLA1 | chr5:22575601-22577618 REVERSE no original description	
EV002470	1.702	no similarity	
JCVI_21808	1.702	moderately similar to (202)AT4G34460| Symbols: ELK4, AGB1 | AGB1 (GTP BINDING PROTEIN BETA 1) | chr4:16477397-1647927	-1.887
DY028164	1.702	moderately similar to (255)AT2G16900| Symbols: | similar to unknown protein [Arabidopsis thaliana] (TAIR:AT4G35110.2); similar to u	
JCVI_14527	1.701	moderately similar to (500)AT5G67330| Symbols: NRAMP4, ATNRAMP4 | ATNRAMP4 (Arabidopsis natural resistance-associated mac	
CD823136	1.701	weakly similar to (160)AT2G20310| Symbols: RIN13 | RIN13 (RPM1 INTERACTING PROTEIN 13) | chr2:8768409-8770164 REVERSI	
JCVI_16474	1.701	no original description	
JCVI_7584	1.701	moderately similar to (488)AT1G03110| Symbols: | transducin family protein / WD-40 repeat family protein | chr1:749359-751796 FORW	
EX063449	1.701	moderately similar to (274)AT1G22620| Symbols: ATSAC1 | ATSAC1 (SUPPRESSOR OF ACTIN 1); phosphoinositide 5-phosphatase | c	
JCVI_23845	1.701	weakly similar to (184)AT5G53030| Symbols: | similar to unknown protein [Arabidopsis thaliana] (TAIR:AT4G27810.1); similar to hypo	
DY018691	1.701	weakly similar to (180)AT4G14455| Symbols: ATBS14B, ATBET12, BET12 | ATBET12; SNAP receptor/ protein transporter | chr4:8310	
JCVI_546	1.701	moderately similar to (332)AT1G55920| Symbols: SAT5, SAT1, AtSerat2;1 | AtSerat2;1 (SERINE ACETYLTRANSFERASE 1) | chr1:20	-1.932
JCVI_4187	1.700	moderately similar to (234)AT3G21700| Symbols: | GTP binding | chr3:7644588-7645961 FORWARD no original description	
CO750586	1.700	very weakly similar to (85.1)AT1G60160| Symbols: | potassium transporter family protein | chr1:22191995-22195060 REVERSE [16161]	
EV019739	1.700	no similarity	
ES988871	1.700	weakly similar to (157)AT5G48300| Symbols: APS1, ADG1 | ADG1 (ADP GLUCOSE PYROPHOSPHORYLASE SMALL SUBUNIT 1);	
JCVI_24688	1.700	moderately similar to (407)AT4G36090| Symbols: | oxidoreductase, 2OG-Fe(II) oxygenase family protein | chr4:17078379-17080673 RE	
JCVI_17617	1.700	moderately similar to (223)AT2G18680| Symbols: | similar to unknown protein [Arabidopsis thaliana] (TAIR:AT2G18690.1); similar to h	
CX190781	1.700	moderately similar to (379)AT1G79380| Symbols: | copine-related | chr1:29865705-29867916 FORWARD [16807]	
EL589250	1.700	very weakly similar to (89.4)AT1G53020| Symbols: UBC26, PFU3 | PFU3/UBC26 (UBIQUITIN-CONJUGATING ENZYME 26); ubiquit	
EV198826	1.700	weakly similar to (120)AT2G14750| Symbols: AKN1, ATAKN1, APK | APK (APS KINASE); ATP binding / kinase/ transferase, transfer	
JCVI_26059	1.699	highly similar to (577)AT5G41460| Symbols: | fringe-related protein | chr5:16606952-16609425 REVERSE no original description	
JCVI_29971	1.699	highly similar to (520)AT2G45910| Symbols: | protein kinase family protein / U-box domain-containing protein | chr2:18901593-1890528	
JCVI_24420	1.699	moderately similar to (243)AT4G34120| Symbols: LEJ1 | LEJ1 (LOSS OF THE TIMING OF ET AND JA BIOSYNTHESIS 1) | chr4:1634	
EX137998	1.699	no similarity	
CD826359	1.699	moderately similar to (298)AT4G00700| Symbols: | C2 domain-containing protein | chr4:286260-289369 FORWARD [13979]	
EV151458	1.699	moderately similar to (258)AT5G08710| Symbols: | regulator of chromosome condensation (RCC1) family protein / UVB-resistance prote	
EE419923	1.699	moderately similar to (428)AT4G31140| Symbols: | glycosyl hydrolase family 17 protein | chr4:15141587-15143194 FORWARDweakly s	
ES900687	1.699	moderately similar to (305)AT5G67450| Symbols: AZF1 | AZF1 (ARABIDOPSIS ZINC-FINGER PROTEIN 1); nucleic acid binding / tra	
JCVI_33538	1.698	moderately similar to (341)AT5G05240| Symbols: | similar to unknown protein [Arabidopsis thaliana] (TAIR:AT2G40630.1); similar to h	
ES980475	1.698	no similarity	
JCVI_24598	1.698	highly similar to (835)AT3G61280| Symbols: | similar to unknown protein [Arabidopsis thaliana] (TAIR:AT3G61290.1); similar to unnam	
EE424660	1.698	very weakly similar to (89.7)AT1G08510| Symbols: FATB | FATB (FATTY ACYL-ACP THIOESTERASES B); acyl carrier/ acyl-ACP thi	
DY009544	1.697	weakly similar to (105)AT1G30860| Symbols: | protein binding / zinc ion binding | chr1:10986677-10989227 REVERSE [18969] 15 316 3	
JCVI_19051	1.697	highly similar to (626)AT4G13700| Symbols: ATPAP23, PAP23 | ATPAP23/PAP23 (purple acid phosphatase 23); acid phosphatase/ prote	
JCVI_10528	1.697	moderately similar to (252)AT2G04280| Symbols: | similar to unknown protein [Arabidopsis thaliana] (TAIR:AT4G12700.1); similar to C	
EV135340	1.697	no similarity	-2.288
ES941194	1.697	weakly similar to (194)AT3G59150| Symbols: | F-box family protein | chr3:21883706-21885399 FORWARD [21391]	
CN729799	1.697	weakly similar to (149)AT4G16660| Symbols: | heat shock protein 70, putative / HSP70, putative | chr4:9377247-9381254 FORWARD [1	
JCVI_14436	1.697	moderately similar to (320)AT4G34230| Symbols: ATCAD5, CAD-5, CAD5 | CAD5 (CINNAMYL ALCOHOL DEHYDROGENASE 5) |	-1.665
JCVI_254	1.697	moderately similar to (313)AT5G48180| Symbols: | kelch repeat-containing protein | chr5:19558509-19559584 REVERSE no original des	1.998
EV226734	1.696	no similarity	

JCVI_3007	1.696	moderately similar to (427)AT1G09430| Symbols: ACLA-3 | ACLA-3 (ATP-citrate lyase A-3) | chr1:3042137-3044980 FORWARD no ori	-1.971
JCVI_40160	1.696	moderately similar to (221)AT5G26330| Symbols: | plastocyanin-like domain-containing protein / mavicyanin, putative | chr5:9241617-92	
EE546449	1.696	moderately similar to (298)AT5G63570| Symbols: GSA1 | GSA1 (GLUTAMATE-1-SEMIALDEHYDE-2,1-AMINOMUTASE); glutamate	
JCVI_24148	1.695	highly similar to (534)AT5G46800| Symbols: BOU | BOU (A BOUT DE SOUFFLE); binding | chr5:19006006-19007037 REVERSEvery v	1.281
JCVI_12749	1.695	moderately similar to (471)AT4G03110| Symbols: | RNA-binding protein, putative | chr4:1376710-1379275 REVERSE no original descrip	
EV019639	1.695	moderately similar to (469)AT3G09920| Symbols: PIP5K9 | PIP5K9 (PHOSPHATIDYL INOSITOL MONOPHOSPHATE 5 KINASE); 1	
JCVI_37862	1.695	moderately similar to (481)AT5G61060| Symbols: HDA5, HDA05 | HDA05 (HISTONE DEACETYLASE5); histone deacetylase | chr5:24	
EV223801	1.695	very weakly similar to (92.0)AT1G10720| Symbols: | BSD domain-containing protein | chr1:3562965-3564447 FORWARD [21493]	
CD827893	1.695	moderately similar to (242)AT5G07630| Symbols: | nuclear division RFT family protein | chr5:2412033-2414524 FORWARD [13979]	
JCVI_18022	1.695	highly similar to (597)AT1G60990| Symbols: | similar to aminomethyltransferase, putative [Arabidopsis thaliana] (TAIR:AT1G11860.1);	
JCVI_16792	1.695	moderately similar to (436)AT3G13790| Symbols: ATCWINV1, ATBFRUCT1 | ATBFRUCT1/ATCWINV1 (ARABIDOPSIS THALIAN	
EV067003	1.695	moderately similar to (391)AT5G60230| Symbols: ATSEN2, SEN2 | SEN2 (SPLICING ENDONUCLEASE 2); tRNA-intron endonuclease	
JCVI_37948	1.695	moderately similar to (226)AT5G09260| Symbols: VPS20.2 | VPS20.2 | chr5:2876798-2878356 FORWARD no original description	
JCVI_25797	1.695	moderately similar to (219)AT4G27860| Symbols: | integral membrane family protein | chr4:13873814-13876246 FORWARD no original	
JCVI_37231	1.695	moderately similar to (262)AT4G32440| Symbols: | agenet domain-containing protein | chr4:15657301-15659360 FORWARD no original	
AM386881	1.695	very weakly similar to (97.1)AT5G08790| Symbols: ANAC081, ATAF2 | ATAF2 (Arabidopsis NAC domain containing protein 81) | chr5:2	
JCVI_25873	1.694	moderately similar to (381)AT1G01120| Symbols: KCS1 | KCS1 (3-KETOACYL-COA SYNTHASE 1); acyltransferase | chr1:57392-5897	
ES937762	1.694	no similarity	
AT000658	1.694	no similarity	
JCVI_10157	1.694	moderately similar to (325)AT5G41940| Symbols: | RabGAP/TBC domain-containing protein | chr5:16799267-16802679 FORWARD no	
JCVI_7077	1.694	highly similar to (586)AT3G02570| Symbols: MEE31 | MEE31 (maternal effect embryo arrest 31); mannose-6-phosphate isomerase | chr3	
EV072499	1.694	moderately similar to (399)AT1G19940| Symbols: ATGH9B5 | ATGH9B5 (ARABIDOPSIS THALIANA GLYCOSYL HYDROLASE 9B	1.640
JCVI_8684	1.694	weakly similar to (193)AT1G64510| Symbols: | ribosomal protein S6 family protein | chr1:23958656-23959868 REVERSEweakly similar	2.237
EV198732	1.694	very weakly similar to (97.8)AT1G72280| Symbols: AERO1 | AERO1 (ARABIDOPSIS ENDOPLASMIC RETICULUM OXIDOREDUCT	
EX037670	1.693	moderately similar to (288)AT1G44350| Symbols: ILL6 | ILL6 (IAA-leucine resistant (ILR)-like gene 6); metallopeptidase | chr1:1683718	
EX064135	1.693	moderately similar to (355)AT5G44090| Symbols: | calcium-binding EF hand family protein, putative / protein phosphatase 2A 62 kDa B"	
EE532149	1.693	moderately similar to (233)AT1G15430| Symbols: | similar to zinc ion binding [Arabidopsis thaliana] (TAIR:AT1G80220.1); similar to O	
EE532304	1.693	very weakly similar to (88.2)AT3G07565| Symbols: | DNA binding | chr3:2413829-2415878 FORWARD [20175]	
EX070129	1.693	no similarity	
JCVI_29087	1.692	weakly similar to (118)AT5G66440| Symbols: | similar to unknown protein [Arabidopsis thaliana] (TAIR:AT4G34560.1); similar to hypot	
RC_ES948251	1.692	no similarity	
EE467545	1.692	moderately similar to (353)AT1G78320| Symbols: ATGSTU23 | ATGSTU23 (Arabidopsis thaliana Glutathione S-transferase (class tau) 2	
JCVI_36537	1.692	moderately similar to (380)AT1G11660| Symbols: | heat shock protein, putative | chr1:3921056-3924347 FORWARD no original descript	
JCVI_2546	1.692	moderately similar to (466)AT1G19580| Symbols: GAMMA CA1 | GAMMA CA1 (GAMMA CARBONIC ANHYDRASE 1); carbonate d	
JCVI_13459	1.692	no original description	
EV149332	1.692	no similarity	
CX195231	1.691	no similarity	
JCVI_25141	1.691	moderately similar to (249)AT3G02580| Symbols: DWF7, BUL1 | STE1 (STEROL 1); C-5 sterol desaturase | chr3:547055-548622 FORW	
DW999457	1.691	no similarity	
JCVI_18085	1.690	moderately similar to (360)AT2G32700| Symbols: | WD-40 repeat family protein | chr2:13874312-13878921 FORWARD no original desc	
JCVI_32777	1.690	moderately similar to (326)AT3G20770| Symbols: EIN3 | EIN3 (ETHYLENE-INSENSITIVE3); transcription factor | chr3:7260708-72625	
JCVI_10976	1.690	moderately similar to (426)AT4G21520| Symbols: | transducin family protein / WD-40 repeat family protein | chr4:11447526-11450389 R	
JCVI_5532	1.690	moderately similar to (328)AT2G24270| Symbols: ALDH11A3 | ALDH11A3 (Aldehyde dehydrogenase 11A3); 3-chloroallyl aldehyde deh	
JCVI_11492	1.690	moderately similar to (419)AT3G48200| Symbols: | similar to hypothetical protein OsI_020499 [Oryza sativa (indica cultivar-group)] (GB	-1.354
JCVI_34711	1.690	moderately similar to (223)AT2G45550| Symbols: CYP76C4 | CYP76C4 (cytochrome P450, family 76, subfamily C, polypeptide 4); oxyg	
JCVI_11178	1.689	moderately similar to (271)AT2G17450| Symbols: RHA3A | RHA3A (RING-H2 finger A3A); protein binding / zinc ion binding | chr2:758	
JCVI_31062	1.689	moderately similar to (461)AT3G58620| Symbols: TTL4 | TTL4 (TETRATRICOPETIDE-REPEAT THIOREDOXIN-LIKE 4); binding | c	
CX266514	1.689	no similarity	
JCVI_24574	1.689	no original description	
JCVI_26901	1.689	moderately similar to (334)AT5G19150| Symbols: | carbohydrate kinase family | chr5:6426282-6428404 REVERSE no original descriptio	
JCVI_12612	1.689	moderately similar to (204)AT4G37400| Symbols: CYP81F3 | CYP81F3 (cytochrome P450, family 81, subfamily F, polypeptide 3); oxyge	
EE484328	1.688	weakly similar to (141)AT1G29400| Symbols: AML5 | AML5 (ARABIDOPSIS MEI2-LIKE PROTEIN 5); RNA binding | chr1:10290379-	
JCVI_7030	1.688	moderately similar to (340)AT3G19390| Symbols: | cysteine proteinase, putative / thiol protease, putative | chr3:6723030-6724774 FORW	
EE420010	1.688	moderately similar to (304)AT2G23980| Symbols: CNGC6, ATCNGC6 | ATCNGC6 (CYCLIC NUCLEOTIDE GATED CHANNEL 6); c	
JCVI_8541	1.688	moderately similar to (337)AT2G34355| Symbols: | nodulin-related | chr2:14503455-14505390 FORWARD no original description	
JCVI_41631	1.688	moderately similar to (362)AT1G74860| Symbols: | similar to unknown protein [Arabidopsis thaliana] (TAIR:AT1G19010.1); similar to h	1.254
EE475633	1.688	weakly similar to (140)AT1G73700| Symbols: | MATE efflux family protein | chr1:27721215-27723291 REVERSE [20134] 1 522 535	-3.725
DN962534	1.688	no similarity	
JCVI_277	1.688	moderately similar to (402)AT4G34960| Symbols: | peptidyl-prolyl cis-trans isomerase, putative / cyclophilin, putative / rotamase, putative	
JCVI_36574	1.687	weakly similar to (177)AT1G50450| Symbols: | binding / catalytic | chr1:18691570-18694016 REVERSE no original description	
EV176857	1.687	weakly similar to (147)AT1G22640| Symbols: ATMYB3, MYB3 | MYB3 (myb domain protein 3); DNA binding / transcription factor | chr	
JCVI_29392	1.687	weakly similar to (193)AT4G38940| Symbols: | kelch repeat-containing F-box family protein | chr4:18152842-18153954 FORWARD no o	
EE440247	1.687	weakly similar to (186)AT1G48550| Symbols: | vacuolar protein sorting-associated protein 26 family protein / VPS26 family protein | chr1	
JCVI_41519	1.687	very weakly similar to (81.3)AT5G65840| Symbols: | similar to antioxidant/ oxidoreductase [Arabidopsis thaliana] (TAIR:AT2G37240.1);	
CV546557	1.687	very weakly similar to (99.8)AT1G07310| Symbols: | C2 domain-containing protein | chr1:2247774-2248832 REVERSE [16551]	
JCVI_30339	1.687	moderately similar to (437)AT3G46780| Symbols: PTAC16 | PTAC16 (PLASTID TRANSCRIPTIONALLY ACTIVE18); binding / cataly	
EL588094	1.687	no similarity	
JCVI_8963	1.687	no original description	
ES958727	1.687	weakly similar to (120)AT3G14290| Symbols: PAE2 | PAE2 (20S proteasome alpha subunit E2); peptidase | chr3:4764371-4766388 FORV	
EE517895	1.687	moderately similar to (283)AT1G78550| Symbols: | oxidoreductase, 2OG-Fe(II) oxygenase family protein | chr1:29549924-29551253 REV	
JCVI_9815	1.686	moderately similar to (286)AT1G76950| Symbols: PRAF1 | PRAF1; Ran GTPase binding / chromatin binding / zinc ion binding | chr1:289	
JCVI_16863	1.686	moderately similar to (385)AT2G45290| Symbols: | transketolase, putative | chr2:18679811-18682663 FORWARDmoderately similar to (	
JCVI_18161	1.686	weakly similar to (127)AT5G58040| Symbols: ATFIP1[V] | ATFIP1[V] (ARABIDOPSIS HOMOLOG OF YEAST FIP1 [V]); RNA bindir	
JCVI_30	1.686	moderately similar to (364)AT2G02930| Symbols: GST16, ATGSTF3 | ATGSTF3 (GLUTATHIONE S-TRANSFERASE 16); glutathione	
CD820495	1.686	weakly similar to (152)AT5G15260| Symbols: | structural constituent of ribosome | chr5:4953649-4954353 REVERSE [13978]	
JCVI_26885	1.686	moderately similar to (431)AT3G13380| Symbols: BRL3 | BRL3 (BRI1-LIKE 3); protein binding / protein kinase | chr3:4347247-4350741	
EX069636	1.686	moderately similar to (300)AT1G59840| Symbols: | similar to unnamed protein product [Vitis vinifera] (GB:CAO40980.1) | chr1:2203023	
ES981622	1.686	moderately similar to (374)AT4G29730| Symbols: MSI5, NFC5 | NFC5 (NUCLEOSOME/CHROMATIN ASSEMBLY FACTOR GROUF	
CX189862	1.686	weakly similar to (162)AT1G06630| Symbols: | F-box family protein | chr1:2028070-2029441 FORWARD [16807]	
EE421696	1.685	moderately similar to (316)AT4G33410| Symbols: | signal peptide peptidase family protein | chr4:16081643-16083122 FORWARD [2014	
JCVI_16753	1.685	moderately similar to (286)AT5G23230| Symbols: NIC2 | NIC2 (NICOTINAMIDASE 2); catalytic/ nicotinamidase | chr5:7826008-78266	

JCVI_24152	1.685	highly similar to (620)AT3G54360| Symbols: | protein binding / zinc ion binding | chr3:20139548-20142559 REVERSE no original descri	
EV108519	1.685	moderately similar to (278)AT1G10340| Symbols: | ankyrin repeat family protein | chr1:3390477-3392483 REVERSE [21478]	
JCVI_39650	1.685	moderately similar to (246)AT2G39140| Symbols: | pseudouridine synthase family protein | chr2:16337316-16340231 FORWARD no orig	-1.429
JCVI_39312	1.685	moderately similar to (394)AT3G06950| Symbols: | tRNA pseudouridine synthase family protein | chr3:2192875-2194260 FORWARD no	-2.703
EE418046	1.685	moderately similar to (288)AT5G17490| Symbols: RGL3 | RGL3 (RGA-LIKE 3); transcription factor | chr5:5764318-5765889 REVERSE	
JCVI_30419	1.685	highly similar to (859)AT2G38110| Symbols: ATGPAT6, GPAT6 | ATGPAT6/GPAT6 (GLYCEROL-3-PHOSPHATE ACYLTRANSFER	-2.262
EL591658	1.685	no similarity	
EX126908	1.685	weakly similar to (198)AT4G32160| Symbols: | phox (PX) domain-containing protein | chr4:15529062-15532898 FORWARD [21831]	
ES946666	1.685	moderately similar to (213)AT5G03970| Symbols: | F-box family protein | chr5:1071720-1072940 REVERSE [21393]	
JCVI_29213	1.685	moderately similar to (408)AT1G05180| Symbols: AXR1 | AXR1 (AUXIN RESISTANT 1); small protein activating enzyme | chr1:14985	
JCVI_10957	1.685	no original description	
JCVI_36213	1.684	moderately similar to (258)AT5G25390| Symbols: SHN2 | SHN2 (SHINE2); DNA binding / transcription factor | chr5:8820640-8821744 F	
JCVI_19593	1.684	highly similar to (566)AT3G43300| Symbols: ATMIN7 | ATMIN7 (ARABIDOPSIS THALIANA HOPM INTERACTOR 7); guanyl-nucle	
ES933571	1.684	moderately similar to (221)AT2G41130| Symbols: | basic helix-loop-helix (bHLH) family protein | chr2:17150438-17151764 FORWARD	
JCVI_569	1.684	moderately similar to (450)AT1G73500| Symbols: ATMKK9 | ATMKK9 (Arabidopsis thaliana MAP kinase kinase 9); kinase | chr1:27643	
JCVI_16977	1.683	moderately similar to (438)AT5G05860| Symbols: UGT76C2 | UGT76C2 (UDP-glucosyl transferase 76C2); UDP-glycosyltransferase/ tra	
JCVI_38746	1.683	no original description	
JCVI_32471	1.683	moderately similar to (369)AT1G73740| Symbols: | glycosyl transferase family 28 protein | chr1:27738112-27739669 FORWARD no orig	
ES984105	1.683	weakly similar to (200)AT3G04380| Symbols: SUVR4 | SUVR4 | chr3:1161608-1164545 FORWARD [21389]	
EX120466	1.683	moderately similar to (283)AT5G04380| Symbols: | S-adenosyl-L-methionine:carboxyl methyltransferase family protein | chr5:1234884-1	
EV153799	1.683	moderately similar to (319)AT4G16070| Symbols: | lipase class 3 family protein | chr4:9096820-9100608 REVERSE [21484]	
JCVI_18415	1.683	moderately similar to (356)AT5G13210| Symbols: | similar to unknown protein [Arabidopsis thaliana] (TAIR:AT3G24780.1); similar to u	
JCVI_15853	1.682	moderately similar to (283)AT5G67580| Symbols: TRB2, ATTRB2 | ATTRB2/TRB2 (TELOMERE REPEAT BINDING FACTOR 2); DN	
CV545491	1.682	moderately similar to (244)AT1G72270| Symbols: | similar to binding [Arabidopsis thaliana] (TAIR:AT4G27010.1); similar to unnamed p	
JCVI_2113	1.682	weakly similar to (180)AT5G20190| Symbols: | binding | chr5:6814095-6815173 FORWARD no original description	
JCVI_18182	1.682	no original description	
JCVI_1360	1.682	moderately similar to (442)AT1G60770| Symbols: | pentatricopeptide (PPR) repeat-containing protein | chr1:22370624-22372313 REVER	
JCVI_3766	1.682	moderately similar to (310)AT3G13410| Symbols: | similar to unknown protein [Arabidopsis thaliana] (TAIR:AT1G55546.1); similar to h	
JCVI_18281	1.682	moderately similar to (369)AT1G49240| Symbols: ACT8 | ACT8 (ACTIN 8); structural constituent of cytoskeleton | chr1:18220207-1822	
EV112435	1.682	weakly similar to (153)AT3G50830| Symbols: ATCOR413-PM2 | COR413-PM2 (cold regulated 413 plasma membrane 2) | chr3:1890509	
JCVI_22635	1.681	highly similar to (642)AT4G09980| Symbols: EMB1691 | EMB1691 (EMBRYO DEFECTIVE 1691) | chr4:6249108-6252027 REVERSE	
EV096451	1.681	no similarity	
JCVI_610	1.681	moderately similar to (381)AT2G30860| Symbols: GLUTTR, ATGSTF7, ATGSTF9 | ATGSTF9 (Arabidopsis thaliana Glutathione S-trans	
JCVI_1909	1.681	moderately similar to (328)AT2G28000| Symbols: CH-CPN60A, SLP, CPN60A | CPN60A (chloroplast / 60 kDa chaperonin alpha subunit	
JCVI_37277	1.681	moderately similar to (329)AT5G52510| Symbols: | scarecrow-like transcription factor 8 (SCL8) | chr5:21324422-21326344 FORWARD	
EV070221	1.681	weakly similar to (159)AT1G71800| Symbols: | cleavage stimulation factor, putative | chr1:27003268-27005512 FORWARD [21443] 16 6	
EE477458	1.681	weakly similar to (155)AT1G27370| Symbols: | squamosa promoter-binding protein-like 10 (SPL10) | chr1:9505441-9506984 REVERSE	
EE556743	1.681	weakly similar to (164)AT3G18524| Symbols: ATMSH2, MSH2 | MSH2 (MUTS HOMOLOG 2); ATP binding / damaged DNA binding |	
JCVI_34890	1.681	moderately similar to (299)AT3G57020| Symbols: | strictosidine synthase family protein | chr3:21109494-21111282 REVERSE no origina	
ES267768	1.680	moderately similar to (338)AT2G44450| Symbols: | glycosyl hydrolase family 1 protein | chr2:18348042-18350820 FORWARDweakly sir	
H07347	1.680	no similarity	
JCVI_11600	1.680	moderately similar to (251)AT3G10700| Symbols: | GHMP kinase family protein | chr3:3346794-3350868 REVERSE no original descript	
JCVI_13772	1.679	moderately similar to (307)AT2G44060| Symbols: | late embryogenesis abundant family protein / LEA family protein | chr2:18233998-18	
JCVI_17902	1.679	moderately similar to (488)AT5G37150| Symbols: | tRNA-splicing endonuclease positive effector-related | chr5:14718560-14721792 FOR	
EE522887	1.679	moderately similar to (212)AT2G20930| Symbols: | similar to unknown [Populus trichocarpa] (GB:ABK93191.1); contains InterPro doma	
EE431625	1.679	no similarity	1.855
DN960461	1.679	weakly similar to (200)AT4G24520| Symbols: AR1, ATR1 | ATR1 (ARABIDOPSIS P450 REDUCTASE 1) | chr4:12663075-12667076 R	
JCVI_20713	1.679	moderately similar to (461)AT3G16530| Symbols: | legume lectin family protein | chr3:5624592-5625422 REVERSEvery weakly similar t	
JCVI_10301	1.679	moderately similar to (389)AT1G30400| Symbols: EST1, ATMRP1 | ATMRP1 (Arabidopsis thaliana multidrug resistance-associated prot	
EX131740	1.679	weakly similar to (137)AT5G52450| Symbols: | MATE efflux protein-related | chr5:21306268-21308975 REVERSE [21833] 1 673 692	
JCVI_5884	1.679	no original description	
EV032219	1.679	no similarity	
EV029018	1.679	moderately similar to (409)AT1G53050| Symbols: | protein kinase family protein | chr1:19776242-19779199 FORWARD [21441]	
EX056126	1.678	moderately similar to (379)AT4G32750| Symbols: | similar to unnamed protein product [Vitis vinifera] (GB:CAO44059.1) | chr4:1579642	
JCVI_33238	1.678	highly similar to (649)AT3G17800| Symbols: | mRNA level of the MEB5.2 gene (At3g17800) remains unchanged after cutting the inflore	
JCVI_5910	1.677	weakly similar to (192)AT3G45830| Symbols: | similar to unknown protein [Arabidopsis thaliana] (TAIR:AT1G02290.1); similar to unna	
EX131663	1.677	no similarity	
JCVI_9453	1.677	moderately similar to (204)AT3G02310| Symbols: AGL4, SEP2 | SEP2 (SEPALLATA2); DNA binding / transcription factor | chr3:464561	-3.501
EV124029	1.677	moderately similar to (369)AT4G21390| Symbols: B120 | B120; protein kinase/ sugar binding | chr4:11394469-11397485 REVERSEweak	
JCVI_947	1.677	no original description	
EX027565	1.677	moderately similar to (382)AT2G25580| Symbols: | binding | chr2:10895180-10897027 FORWARD [21810]	2.078
EV170770	1.677	moderately similar to (278)AT2G45340| Symbols: | leucine-rich repeat transmembrane protein kinase, putative | chr2:18698813-1870154	
JCVI_7870	1.677	highly similar to (531)AT2G35680| Symbols: | dual specificity protein phosphatase family protein | chr2:15004083-15005669 REVERSE	
JCVI_25076	1.677	weakly similar to (105)AT1G69490| Symbols: ANAC029, ATNAP, NAP | NAP (NAC-LIKE, ACTIVATED BY AP3/PI); transcription fac	
JCVI_15745	1.677	highly similar to (632)AT3G48000| Symbols: ALDH2, ALDH2B4 | ALDH2B4 (ALDEHYDE DEHYDROGENASE 2); 3-chloroallyl aldeh	
DY015590	1.676	very weakly similar to (87.8)AT5G59350| Symbols: | similar to unnamed protein product [Vitis vinifera] (GB:CAO21684.1) | chr5:239583	
JCVI_38474	1.676	very weakly similar to (92.4)AT3G17820| Symbols: ATGSKB6 | ATGSKB6 (Arabidopsis thaliana glutamine synthase clone KB6); glutama	
ES913163	1.676	moderately similar to (272)AT4G38340| Symbols: | RWP-RK domain-containing protein | chr4:17954704-17957822 FORWARD [21430]	
EE503284	1.676	very weakly similar to (95.5)AT1G75820| Symbols: FAS3, FLO5, CLV1 | CLV1 (CLAVATA 1); ATP binding / kinase/ protein serine/threo	
JCVI_3817	1.676	moderately similar to (460)AT1G19600| Symbols: | pfkB-type carbohydrate kinase family protein | chr1:6779076-6780889 FORWARD n	
JCVI_36284	1.676	weakly similar to (124)AT3G30460| Symbols: | zinc finger (C3HC4-type RING finger) family protein | chr3:12106912-12107355 FORWA	
JCVI_35818	1.676	no original description	
JCVI_2200	1.675	weakly similar to (173)AT3G13390| Symbols: SKS11 | SKS11 (SKU5 Similar 11); copper ion binding / oxidoreductase | chr3:4351408-43	2.000
JCVI_11232	1.675	moderately similar to (239)AT1G48400| Symbols: | F-box family protein | chr1:17885804-17887523 REVERSE no original description	
EX045267	1.675	moderately similar to (261)AT1G71410| Symbols: | protein kinase family protein | chr1:26916732-26921177 REVERSE [21811]	
ES923561	1.675	no similarity	
JCVI_13240	1.675	moderately similar to (400)AT2G43090| Symbols: | aconitase C-terminal domain-containing protein | chr2:17926034-17926789 FORWAR	
JCVI_42008	1.675	weakly similar to (186)AT5G04040| Symbols: SDP1 | SDP1 (SUGAR-DEPENDENT1); triacylglycerol lipase | chr5:1090345-1093002 FO	
JCVI_15597	1.675	highly similar to (658)AT2G41620| Symbols: | nucleoporin interacting component family protein | chr2:17357497-17362075 REVERSE n	
EE435246	1.675	very weakly similar to (96.3)AT1G67850| Symbols: | similar to unknown protein [Arabidopsis thaliana] (TAIR:AT1G13000.2); similar to	
JCVI_13541	1.675	moderately similar to (448)AT1G33270| Symbols: | patatin-related | chr1:12068803-12070137 REVERSE no original description	-1.496

JCVI_3119	1.675	moderately similar to (229)AT3G12130| Symbols: | KH domain-containing protein / zinc finger (CCCH type) family protein | chr3:38644(	
JCVI_15036	1.674	moderately similar to (259)AT4G30640| Symbols: | F-box family protein (FBL19) | chr4:14952676-14953688 FORWARD no original des	
EX049461	1.674	moderately similar to (320)AT2G03200| Symbols: | aspartyl protease family protein | chr2:966503-967888 REVERSE [21812] 32 686 68(	-1.670
JCVI_10034	1.674	highly similar to (773)AT4G34710| Symbols: SPE2, ADC2 | ADC2 (ARGININE DECARBOXYLASE 2) | chr4:16560320-16562455 REV	
JCVI_41127	1.674	no original description	
JCVI_35574	1.674	moderately similar to (420)AT3G11560| Symbols: | similar to unknown protein [Arabidopsis thaliana] (TAIR:AT5G06220.1); similar to h	
JCVI_32309	1.674	moderately similar to (268)AT5G22840| Symbols: | protein kinase family protein | chr5:7631106-7633106 REVERSE no original descript	
EV211204	1.674	moderately similar to (416)AT4G18240| Symbols: ATSS4, SSIV | ATSS4/SSIV (STARCH SYNTHASE 4); transferase, transferring glyco	
JCVI_10042	1.674	highly similar to (738)AT5G28350| Symbols: | similar to unknown protein [Arabidopsis thaliana] (TAIR:AT3G61480.1); similar to unnam	
EV111807	1.674	no similarity	1.718
JCVI_18164	1.674	moderately similar to (205)AT1G72360| Symbols: | ethylene-responsive element-binding protein, putative | chr1:27245566-27246439 FOI	
JCVI_12902	1.674	highly similar to (662)AT5G16890| Symbols: | exostosin family protein | chr5:5551667-5554744 FORWARD no original description	
EV198384	1.674	weakly similar to (198)AT2G29510| Symbols: | similar to unknown protein [Arabidopsis thaliana] (TAIR:AT5G59020.1); similar to unnai	
JCVI_20575	1.674	moderately similar to (353)AT2G25840| Symbols: OVA4 | OVA4 (OVULE ABORTION 4); ATP binding / aminoacyl-tRNA ligase | chr2:	1.408
JCVI_32056	1.674	weakly similar to (194)AT2G44940| Symbols: | AP2 domain-containing transcription factor TINY, putative | chr2:18544369-18545256 FC	3.458
JCVI_14812	1.674	weakly similar to (142)AT1G67400| Symbols: | similar to phagocytosis and cell motility protein ELMO1-related [Arabidopsis thaliana] (T	
EX118324	1.674	moderately similar to (479)AT3G16260| Symbols: | catalytic | chr3:5509403-5513124 FORWARD [21828] 18 863 863	
JCVI_23276	1.674	no original description	
JCVI_22006	1.673	highly similar to (539)AT3G03960| Symbols: | chaperonin, putative | chr3:1024439-1027611 FORWARDvery weakly similar to (87.4)TC	
JCVI_18142	1.673	moderately similar to (434)AT5G64170| Symbols: | dentin sialophosphoprotein-related | chr5:25690130-25693160 REVERSE no original	
JCVI_14806	1.673	moderately similar to (448)AT5G08400| Symbols: | similar to unknown protein [Arabidopsis thaliana] (TAIR:AT4G29400.1); similar to u	-2.364
JCVI_8271	1.673	no original description	
JCVI_64	1.673	moderately similar to (285)AT5G57900| Symbols: SKIP1 | SKIP1 (SKP1 INTERACTING PARTNER 1) | chr5:23467142-23468141 REV	-2.780
JCVI_29673	1.673	moderately similar to (469)AT3G56630| Symbols: CYP94D2 | CYP94D2 (cytochrome P450, family 94, subfamily D, polypeptide 2); oxyg	
EE482479	1.673	moderately similar to (283)AT1G20560| Symbols: | AMP-dependent synthetase and ligase family protein | chr1:7119917-7121353 REVEI	
EV163130	1.673	no similarity	
JCVI_18682	1.673	no original description	
AM389563	1.673	moderately similar to (380)AT3G05990| Symbols: | leucine-rich repeat family protein | chr3:1797122-1799738 REVERSE [20118]	
JCVI_24733	1.673	no original description	
EV216908	1.673	no similarity	
EX098969	1.672	moderately similar to (452)AT1G14190| Symbols: | glucose-methanol-choline (GMC) oxidoreductase family protein | chr1:4852797-4854	
JCVI_19312	1.672	moderately similar to (431)AT3G07870| Symbols: | F-box family protein | chr3:2510877-2512130 FORWARD no original description	
JCVI_8028	1.672	moderately similar to (478)AT1G09850| Symbols: XBCP3 | XBCP3 (XYLEM BARK CYSTEINE PEPTIDASE 3); cysteine-type peptidas	
DY028415	1.672	moderately similar to (234)AT3G52180| Symbols: ATPTPKIS1, DSP4, SEX4 | ATPTPKIS1/DSP4/SEX4 (STARCH-EXCESS 4); protein	
JCVI_28094	1.672	moderately similar to (266)AT2G44410| Symbols: | protein binding / zinc ion binding | chr2:18335949-18337190 FORWARD no original	
EE533226	1.672	moderately similar to (269)AT2G35160| Symbols: SUVH5 | SUVH5 (SU(VAR)3-9 HOMOLOG 5) | chr2:14830641-14833025 FORWARI	
JCVI_5767	1.671	highly similar to (690)AT1G76680| Symbols: OPR1 | OPR1 (12-oxophytodienoate reductase 1); 12-oxophytodienoate reductase | chr1:287	
JCVI_29216	1.671	moderately similar to (324)AT5G02460| Symbols: | Dof-type zinc finger domain-containing protein | chr5:539547-541056 REVERSEwea	
JCVI_17245	1.671	moderately similar to (273)AT5G50100| Symbols: | similar to PBng143 [Vigna radiata] (GB:BAB82450.1); contains InterPro domain Thi	
AM057391	1.670	weakly similar to (138)AT5G20270| Symbols: HHP1 | HHP1 (HEPTAHELICAL TRANSMEMBRANE PROTEIN1) | chr5:6841027-6842	
ES902797	1.670	moderately similar to (352)AT3G22830| Symbols: HSFA6B, AT-HSFA6B | AT-HSFA6B (Arabidopsis thaliana heat shock transcription fa	
JCVI_2861	1.670	moderately similar to (218)AT5G08560| Symbols: | transducin family protein / WD-40 repeat family protein | chr5:2771105-2773828 REV	-2.995
JCVI_31989	1.670	moderately similar to (416)AT3G24430| Symbols: HCF101 | HCF101 (HIGH-CHLOROPHYLL-FLUORESCENCE 101); ATP binding | c	
ES951850	1.670	no similarity	
EV200816	1.670	moderately similar to (400)AT1G65450| Symbols: | transferase family protein | chr1:24321698-24322558 FORWARDvery weakly similar	
JCVI_21257	1.670	no original description	
CN736572	1.670	weakly similar to (181)AT2G01590| Symbols: | similar to unnamed protein product [Vitis vinifera] (GB:CAO42320.1) | chr2:266674-2673	1.632
JCVI_8241	1.670	highly similar to (705)AT5G08170| Symbols: EMB1873, ATAIH | ATAIH/EMB1873 (AGMATINE IMINOHYDROLASE); agmatine dei	
JCVI_9056	1.670	no original description	
EV147554	1.670	moderately similar to (243)AT1G05790| Symbols: | lipase class 3 family protein | chr1:1733995-1737364 FORWARD [21482]	
JCVI_15331	1.670	moderately similar to (342)AT1G63120| Symbols: ATRBL2 | ATRBL2 (ARABIDOPSIS THALIANA RHOMBOID-LIKE 2); serine-type	
JCVI_29040	1.670	moderately similar to (227)AT5G47480| Symbols: | similar to unknown protein [Arabidopsis thaliana] (TAIR:AT5G47490.1); similar to h	
JCVI_24479	1.670	weakly similar to (171)AT5G58300| Symbols: | leucine-rich repeat transmembrane protein kinase, putative | chr5:23590047-23592097 FO	
EX137659	1.669	moderately similar to (444)AT1G70530| Symbols: | protein kinase family protein | chr1:26592413-26595042 REVERSEweakly similar to	
JCVI_4982	1.669	moderately similar to (288)AT5G59290| Symbols: ATUXS3, UXS3 | UXS3 (UDP-GLUCURONIC ACID DECARBOXYLASE) | chr5:23(	
JCVI_8270	1.669	moderately similar to (393)AT5G10070| Symbols: | RNase L inhibitor protein-related | chr5:3148685-3150325 REVERSE no original des	
EX093076	1.668	moderately similar to (309)AT4G24590| Symbols: | similar to unknown protein [Arabidopsis thaliana] (TAIR:AT5G49710.3); similar to u	
ES968040	1.668	no similarity	
JCVI_4169	1.668	moderately similar to (224)AT1G27950| Symbols: | lipid transfer protein-related | chr1:9740727-9741978 FORWARD no original descript	
EX036111	1.668	no similarity	-1.987
JCVI_29071	1.668	no original description	
EX141566	1.668	weakly similar to (158)AT5G03880| Symbols: | similar to unknown protein [Arabidopsis thaliana] (TAIR:AT4G10000.2); similar to unkn	
ES952048	1.668	moderately similar to (288)AT1G67320| Symbols: | DNA primase, large subunit family | chr1:25209183-25212629 REVERSE [21423]	-1.455
EV175881	1.668	no similarity	
JCVI_9019	1.668	moderately similar to (361)AT4G31870| Symbols: ATGPX7 | ATGPX7 (GLUTATHIONE PEROXIDASE 7); glutathione peroxidase | chr	
JCVI_11593	1.668	weakly similar to (108)AT5G03460| Symbols: | similar to unknown [Populus trichocarpa] (GB:ABK93498.1) | chr5:864390-865508 FOR\	
JCVI_17981	1.668	moderately similar to (473)AT4G29900| Symbols: ATACA10, ACA10 | ACA10 (autoinhibited Ca2+ -ATPase 10); calcium-transporting A	
JCVI_33873	1.667	moderately similar to (305)AT2G23890| Symbols: | 5' nucleotidase family protein | chr2:10177760-10180702 FORWARD no original des	
JCVI_500	1.667	moderately similar to (335)AT3G20370| Symbols: | meprin and TRAF homology domain-containing protein / MATH domain-containing p	
JCVI_33852	1.667	weakly similar to (109)AT4G00420| Symbols: | double-stranded RNA-binding domain (DsRBD)-containing protein | chr4:181518-182408	
EG019910	1.667	weakly similar to (171)AT1G51500| Symbols: D3, ABCG12, WBC12, CER5 | CER5 (ECERIFERUM 5); ATPase, coupled to transmembr	
JCVI_17195	1.666	moderately similar to (429)AT5G14420| Symbols: RGLG2 | RGLG2 (RING DOMAIN LIGASE2) | chr5:4648358-4650566 REVERSE no	
JCVI_39583	1.666	moderately similar to (251)AT4G27990| Symbols: | YGGT family protein | chr4:13923997-13924653 REVERSE no original description	
JCVI_8825	1.666	moderately similar to (222)AT3G51370| Symbols: | protein phosphatase 2C, putative / PP2C, putative | chr3:19081366-19082954 FORW/	
JCVI_12280	1.666	moderately similar to (262)AT5G55050| Symbols: | GDSL-motif lipase/hydrolase family protein | chr5:22354971-22356967 FORWARD r	
EE462556	1.666	moderately similar to (218)AT4G37030| Symbols: | similar to unknown protein [Arabidopsis thaliana] (TAIR:AT4G12680.1); similar to u	
EX135836	1.666	moderately similar to (333)AT3G05650| Symbols: | disease resistance family protein | chr3:1645890-1648496 REVERSEvery weakly simi	
JCVI_11143	1.666	moderately similar to (209)AT2G41680| Symbols: | thioredoxin reductase, putative / NADPH-dependent thioredoxin reductase, putative |	
JCVI_9949	1.665	no original description	
CD836374	1.665	weakly similar to (156)AT1G63630| Symbols: | pentatricopeptide (PPR) repeat-containing protein | chr1:23590961-23591883 FORWARD	-2.191
JCVI_29654	1.665	highly similar to (588)AT3G43240| Symbols: | ARID/BRIGHT DNA-binding domain-containing protein | chr3:15221020-15225428 REV	

JCVI_5775	1.665	moderately similar to (284)AT1G01090| Symbols: PDH-E1 ALPHA | PDH-E1 ALPHA (PYRUVATE DEHYDROGENASE E1 ALPHA);	
ES265465	1.665	no similarity	
JCVI_21439	1.665	moderately similar to (333)AT5G51680| Symbols: | hydroxyproline-rich glycoprotein family protein | chr5:21014817-21015981 FORWAI	1.451
JCVI_6837	1.665	no original description	
JCVI_2872	1.665	moderately similar to (376)AT3G20370| Symbols: | meprin and TRAF homology domain-containing protein / MATH domain-containing p	
EE544434	1.665	weakly similar to (194)AT1G75030| Symbols: ATLP-3 | ATLP-3 (Arabidopsis thaumatin-like protein 3) | chr1:28178079-28178916 FORW	
JCVI_24183	1.665	moderately similar to (408)AT3G54150| Symbols: | embryo-abundant protein-related | chr3:20061780-20063720 REVERSE no original de	
JCVI_35678	1.665	moderately similar to (262)AT3G53180| Symbols: | glutamate-ammonia ligase | chr3:19718046-19722166 FORWARD no original descrip	
JCVI_8206	1.665	moderately similar to (237)AT2G12480| Symbols: SCPL43 | SCPL43; serine carboxypeptidase | chr2:5076877-5079309 REVERSEweakly	
JCVI_34129	1.665	weakly similar to (165)AT5G38435| Symbols: SPH8 | SPH8 (S-PROTEIN HOMOLOGUE 8) | chr5:15405310-15405708 FORWARD no o	
EE557568	1.664	no similarity	
JCVI_11767	1.664	moderately similar to (210)AT4G32400| Symbols: SHS1 | SHS1 (SODIUM HYPERSENSITIVE 1); binding / transporter | chr4:15638692-	
EG019157	1.664	moderately similar to (270)AT1G58230| Symbols: | WD-40 repeat family protein / beige-related | chr1:21577371-21582530 FORWARD [	
ES966823	1.664	no similarity	
DN191694	1.664	moderately similar to (202)AT2G03510| Symbols: | band 7 family protein | chr2:1066714-1068931 FORWARD [12405]	
EV041542	1.664	weakly similar to (183)AT3G43590| Symbols: | zinc knuckle (CCHC-type) family protein | chr3:15520741-15523243 FORWARD [21442	
EV020694	1.664	moderately similar to (226)AT5G42720| Symbols: | glycosyl hydrolase family 17 protein | chr5:17147763-17149991 FORWARDvery wea	
JCVI_3976	1.663	weakly similar to (191)AT1G59600| Symbols: ZCW7 | ZCW7 | chr1:21893592-21895575 REVERSE no original description	-1.861
CX267276	1.663	moderately similar to (236)AT4G25640| Symbols: | MATE efflux family protein | chr4:13076962-13078974 REVERSE [16816]	
EX075033	1.663	moderately similar to (437)AT2G13610| Symbols: | ABC transporter family protein | chr2:5680909-5682858 REVERSE [21817] 26 799 7	
CD821035	1.663	no similarity	
EV213948	1.663	moderately similar to (303)AT1G61140| Symbols: EDA16 | EDA16 (embryo sac development arrest 16) | chr1:22538703-22543421 REVE	2.052
AM387211	1.663	weakly similar to (107)AT2G32800| Symbols: AP4.3A | AP4.3A; ATP binding / protein kinase | chr2:13923555-13926110 FORWARD [2	
CN729991	1.663	weakly similar to (199)AT5G25070| Symbols: | similar to unnamed protein product [Vitis vinifera] (GB:CAO66326.1) | chr5:8641264-864	
JCVI_16638	1.663	moderately similar to (329)AT1G78600| Symbols: | zinc finger (B-box type) family protein | chr1:29572263-29573555 FORWARD no ori	
ES913343	1.663	moderately similar to (306)AT5G14420| Symbols: RGLG2 | RGLG2 (RING DOMAIN LIGASE2) | chr5:4648358-4650566 REVERSE [21	
CN731527	1.663	weakly similar to (185)AT4G34340| Symbols: TAF8 | TAF8 (TBP-ASSOCIATED FACTOR 8); DNA binding | chr4:16426775-16427836	
EV167294	1.663	weakly similar to (152)AT5G23080| Symbols: TGH | TGH (TOUGH); RNA binding | chr5:7743229-7748892 REVERSE [21486] 250 131	
JCVI_29992	1.663	moderately similar to (322)AT1G06130| Symbols: GLX2-4 | GLX2-4 (GLYOXALASE 2-4); hydroxyacylglutathione hydrolase | chr1:1858	
JCVI_27021	1.663	moderately similar to (394)AT3G48380| Symbols: | Identical to Probable Ufm1-specific protease [Arabidopsis Thaliana] (GB:Q9STL8;G	
ES906790	1.663	moderately similar to (280)AT5G24470| Symbols: PRR5, APRR5 | APRR5 (PSEUDO-RESPONSE REGULATOR 5); transcription regula	
EV046900	1.663	moderately similar to (271)AT4G03030| Symbols: | kelch repeat-containing F-box family protein | chr4:1335942-1337270 REVERSE [214	
JCVI_3109	1.662	moderately similar to (364)AT1G12350| Symbols: ATCOAB | ATCOAB (4-PHOSPHO-PANTO-THENOYLCYSTEINE SYNTHETASE)	
RC_DN964000	1.662	no similarity	
DY019448	1.662	weakly similar to (143)AT1G54320| Symbols: | LEM3 (ligand-effect modulator 3) family protein / CDC50 family protein | chr1:20279399	-2.946
JCVI_18772	1.662	moderately similar to (499)AT5G11650| Symbols: | hydrolase, alpha/beta fold family protein | chr5:3745070-3746817 FORWARD no orig	
JCVI_9629	1.662	moderately similar to (303)AT1G09070| Symbols: SRC2, (AT)SRC2 | (AT)SRC2/SRC2 (SOYBEAN GENE REGULATED BY COLD-2)	
EV214014	1.662	weakly similar to (150)AT1G47830| Symbols: | clathrin coat assembly protein, putative | chr1:17615786-17617224 REVERSEweakly sim	
AM394377	1.662	no similarity	
JCVI_29828	1.662	moderately similar to (410)AT2G26800| Symbols: | hydroxymethylglutaryl-CoA lyase, putative / 3-hydroxy-3-methylglutarate-CoA lyase,	
JCVI_25251	1.662	weakly similar to (130)AT5G11270| Symbols: OCP3 | OCP3 (OVEREXPRESSOR OF CATIONIC PEROXIDASE 3) | chr5:3595558-359	
JCVI_24531	1.661	moderately similar to (288)AT5G57270| Symbols: | similar to unknown protein [Arabidopsis thaliana] (TAIR:AT4G25870.1); similar to u	
CN736259	1.661	no similarity	
EV026794	1.661	moderately similar to (203)AT3G47850| Symbols: | similar to hypothetical protein [Vitis vinifera] (GB:CAN75379.1) | chr3:17665241-17	
EX138448	1.661	moderately similar to (336)AT3G18130| Symbols: | guanine nucleotide-binding family protein / activated protein kinase C receptor (RACl	
CD812264	1.660	no similarity	
JCVI_8302	1.660	no original description	
EX087549	1.660	moderately similar to (314)AT1G12000| Symbols: | pyrophosphate--fructose-6-phosphate 1-phosphotransferase beta subunit, putative / py	
JCVI_33764	1.660	moderately similar to (413)AT5G26680| Symbols: | endonuclease, putative | chr5:9311885-9315461 REVERSEmoderately similar to (37	-1.471
CX193824	1.660	weakly similar to (172)AT1G51580| Symbols: | KH domain-containing protein | chr1:19129280-19131770 FORWARD [16807]	
JCVI_12933	1.660	moderately similar to (382)AT1G32520| Symbols: | similar to unnamed protein product [Vitis vinifera] (GB:CAO63428.1) | chr1:1175840	
BG544831	1.660	weakly similar to (108)AT5G35320| Symbols: | similar to hypothetical protein [Vitis vinifera] (GB:CAN60849.1) | chr5:13539123-135403	
JCVI_25640	1.660	highly similar to (510)AT2G38620| Symbols: CDKB1;2 | CDKB1;2 (cyclin-dependent kinase B1;2); kinase | chr2:16159629-16160944 FC	
JCVI_37143	1.660	moderately similar to (247)AT4G20720| Symbols: | dentin sialophosphoprotein-related | chr4:11105420-11107852 FORWARD no origina	
JCVI_17588	1.659	moderately similar to (465)AT4G28490| Symbols: RLK5, HAE, HAESA | HAESA (RECEPTOR-LIKE PROTEIN KINASE 5); ATP bindi	-1.565
DY004627	1.659	no similarity	
EE476212	1.659	moderately similar to (353)AT1G10410| Symbols: | similar to CW14 [Arabidopsis thaliana] (TAIR:AT1G59650.1); similar to expressed p	
DW999915	1.659	very weakly similar to (97.4)AT3G20650| Symbols: | mRNA capping enzyme family protein | chr3:7221174-7223945 REVERSEvery weal	
CD827994	1.659	moderately similar to (226)AT2G35100| Symbols: ARAD1 | ARAD1 (ARABINAN DEFICIENT 1); catalytic/ transferase, transferring gly	
EV087170	1.659	moderately similar to (217)AT2G28880| Symbols: EMB1997 | EMB1997 (EMBRYO DEFECTIVE 1997); anthranilate synthase/ catalytic	
JCVI_28890	1.659	moderately similar to (367)AT1G13990| Symbols: | similar to unnamed protein product [Vitis vinifera] (GB:CAO68469.1) | chr1:4794807	
JCVI_33608	1.658	highly similar to (728)AT3G11710| Symbols: | lysyl-tRNA synthetase, putative / lysine--tRNA ligase, putative | chr3:3702365-3705619 RI	
EV013172	1.658	no similarity	
JCVI_20433	1.658	moderately similar to (266)AT5G22640| Symbols: EMB1211 | EMB1211 (EMBRYO DEFECTIVE 1211) | chr5:7529426-7533644 FORW	
ES938910	1.658	moderately similar to (352)AT5G22030| Symbols: | ubiquitin-specific protease 8, putative (UBP8) | chr5:7290158-7293597 REVERSE [2	
EX027929	1.658	moderately similar to (253)AT3G50440| Symbols: | hydrolase | chr3:18728373-18729416 REVERSEweakly similar to (104)PIR7A_ORY	-4.415
JCVI_28722	1.658	no original description	
JCVI_10826	1.658	highly similar to (527)AT1G21670| Symbols: | similar to unknown protein [Arabidopsis thaliana] (TAIR:AT1G21680.1); similar to hypotl	
JCVI_36690	1.658	weakly similar to (199)AT1G27060| Symbols: | regulator of chromosome condensation (RCC1) family protein | chr1:9395010-9396660 F(	
L38178	1.657	very weakly similar to (89.4)AT1G14510| Symbols: | PHD finger family protein | chr1:4962166-4964149 REVERSE [132]	
EE440642	1.657	weakly similar to (183)AT5G10030| Symbols: OBF4, TGA4 | TGA4 (TGACG MOTIF-BINDING FACTOR 4); DNA binding / calmodulin	
JCVI_14875	1.657	very weakly similar to (82.8)AT5G61865| Symbols: | similar to hypothetical protein [Vitis vinifera] (GB:CAN73072.1) | chr5:24864621-24	
CX281082	1.657	weakly similar to (134)AT3G13780| Symbols: | similar to SMAD/FHA [Medicago truncatula] (GB:ABN05826.1); contains InterPro doma	
JCVI_38113	1.657	weakly similar to (125)AT3G56010| Symbols: | similar to hypothetical protein [Vitis vinifera] (GB:CAN79918.1); similar to unnamed pro	
JCVI_2590	1.657	moderately similar to (268)AT4G24330| Symbols: | similar to unknown protein [Arabidopsis thaliana] (TAIR:AT5G49945.1); similar to h	
JCVI_8621	1.657	moderately similar to (401)AT4G24610| Symbols: | similar to unknown protein [Arabidopsis thaliana] (TAIR:AT5G65440.2); similar to u	
EX088642	1.656	very weakly similar to (83.6)AT3G12130| Symbols: | KH domain-containing protein / zinc finger (CCCH type) family protein | chr3:38644	
ES986984	1.656	no similarity	
JCVI_25364	1.656	highly similar to (784)AT1G16970| Symbols: KU70, ATKU70 | ATKU70/KU70 (ARABIDOPSIS THALIANA KU70 HOMOLOG); doub	
EE458948	1.656	no similarity	2.447
ES952366	1.656	no similarity	

JCVI_1279	1.656	moderately similar to (210)AT1G78700| Symbols: | brassinosteroid signalling positive regulator-related | chr1:29604747-29606432 FORW	
JCVI_29113	1.655	weakly similar to (135)AT3G44750| Symbols: HDT1, HD2A, HDA3, ATHD2A | HD2A (HISTONE DEACETYLASE 2A); nucleic acid bi	
EV222084	1.655	moderately similar to (238)AT2G36830| Symbols: TIP1;1, GAMMA-TIP1, GAMMA-TIP | GAMMA-TIP (Tonoplast intrinsic protein (TI	
JCVI_28453	1.655	moderately similar to (211)AT3G08010| Symbols: ATAB2 | ATAB2; RNA binding | chr3:2556052-2557432 FORWARD no original descr	
DY017032	1.655	moderately similar to (288)AT5G41920| Symbols: | scarecrow transcription factor family protein | chr5:16797210-16798427 FORWARD	
JCVI_2395	1.655	highly similar to (684)AT3G06170| Symbols: | TMS membrane family protein / tumour differentially expressed (TDE) family protein | chi	
EH429199	1.655	moderately similar to (304)AT2G21860| Symbols: | violaxanthin de-epoxidase-related | chr2:9325413-9327070 REVERSE [20767]	
JCVI_18516	1.654	no original description	
JCVI_3128	1.654	moderately similar to (310)AT4G15630| Symbols: | integral membrane family protein | chr4:8917525-8918681 FORWARD no original de	
EV084861	1.654	no similarity	
JCVI_5008	1.654	weakly similar to (196)AT5G42520| Symbols: BPC6, BBR/BPC6, ATBPC6 | ATBPC6/BBR/BPC6/BPC6 (BASIC PENTACYSTEINE 6)	
EX089125	1.654	moderately similar to (209)AT1G15970| Symbols: | methyladenine glycosylase family protein | chr1:5486538-5488488 REVERSE [21823	
JCVI_22456	1.654	weakly similar to (178)AT2G38540| Symbols: LTP1, ATLTP1, LP1 | LP1 (nonspecific lipid transfer protein 1) | chr2:16137496-16137971	
JCVI_17568	1.654	moderately similar to (301)AT1G69010| Symbols: BIM2 | BIM2 (BES1-INTERACTING MYC-LIKE PROTEIN 2); DNA binding / transc	
JCVI_684	1.653	moderately similar to (418)AT5G54190| Symbols: PORA | PORA (Protochlorophyllide reductase A); oxidoreductase/ protochlorophyllide	
EE423652	1.653	moderately similar to (307)AT2G38610| Symbols: | KH domain-containing protein | chr2:16154630-16156716 REVERSE [20158]	
ES999509	1.653	no similarity	
JCVI_23271	1.653	weakly similar to (178)AT1G19740| Symbols: | ATP-dependent protease La (LON) domain-containing protein | chr1:6824387-6825223 F	
EE445597	1.653	moderately similar to (241)AT3G61650| Symbols: TUBG1 | TUBG1 (GAMMA-TUBULIN); structural molecule | chr3:22823576-228259	
JCVI_34907	1.653	weakly similar to (122)AT5G19080| Symbols: | zinc finger (C3HC4-type RING finger) family protein | chr5:6378402-6380289 FORWAR	
JCVI_29712	1.652	very weakly similar to (99.4)AT1G76954| Symbols: | Encodes a defensin-like (DEFL) family protein. | chr1:28922760-28923431 REVERS	
JCVI_30594	1.652	highly similar to (520)AT1G67930| Symbols: | Golgi transport complex protein-related | chr1:25477881-25480995 REVERSE no original	
EE459110	1.652	weakly similar to (115)AT1G27360| Symbols: | squamosa promoter-binding protein-like 11 (SPL11) | chr1:9502126-9503702 FORWARD	
EX132242	1.652	weakly similar to (159)AT2G32150| Symbols: | haloacid dehalogenase-like hydrolase family protein | chr2:13666172-13667608 FORWAR	
RC_CD831722	1.652	no similarity	
DY023714	1.652	very weakly similar to (83.6)AT5G13240| Symbols: | transcription regulator | chr5:4225264-4227155 REVERSE [18974] 1 569 582	
EE445190	1.652	moderately similar to (203)AT3G11550| Symbols: | integral membrane family protein | chr3:3638268-3639058 FORWARD [20201]	
JCVI_16265	1.652	moderately similar to (308)AT5G60580| Symbols: | zinc finger (C3HC4-type RING finger) family protein | chr5:24371524-24373932 FOI	
JCVI_28110	1.652	no original description	
JCVI_14075	1.652	moderately similar to (236)AT5G02770| Symbols: | similar to unnamed protein product [Vitis vinifera] (GB:CAO18065.1) | chr5:628099-	
JCVI_37333	1.651	moderately similar to (227)AT4G25230| Symbols: RIN2 | RIN2 (RPM1 INTERACTING PROTEIN 2); protein binding / zinc ion binding	
JCVI_9191	1.651	moderately similar to (330)AT4G13180| Symbols: | short-chain dehydrogenase/reductase (SDR) family protein | chr4:7657369-7658160 F	
JCVI_42544	1.651	moderately similar to (213)AT3G23740| Symbols: | similar to unknown protein [Arabidopsis thaliana] (TAIR:AT4G14120.1); similar to h	
EE560445	1.651	weakly similar to (129)AT1G67570| Symbols: | similar to unknown protein [Arabidopsis thaliana] (TAIR:AT1G50630.1); similar to unnai	
JCVI_39079	1.651	weakly similar to (192)AT4G09650| Symbols: | ATP synthase delta chain, chloroplast, putative / H(+)-transporting two-sector ATPase, de	
EE454098	1.651	moderately similar to (247)AT1G68070| Symbols: | zinc finger (C3HC4-type RING finger) family protein | chr1:25519075-25520430 RE	
JCVI_33646	1.651	moderately similar to (256)AT5G51150| Symbols: | similar to unknown protein [Arabidopsis thaliana] (TAIR:AT1G34630.1); similar to u	
JCVI_34621	1.651	very weakly similar to (90.1)AT5G60730| Symbols: | anion-transporting ATPase family protein | chr5:24440064-24442578 FORWARD nc	
EL588469	1.651	moderately similar to (208)AT2G42470| Symbols: | meprin and TRAF homology domain-containing protein / MATH domain-containing p	
EV193291	1.650	weakly similar to (176)AT2G26690| Symbols: | nitrate transporter (NTP2) | chr2:11354425-11357994 REVERSE [21489] 30 736 736	
JCVI_16749	1.650	moderately similar to (417)AT5G36250| Symbols: | protein phosphatase 2C, putative / PP2C, putative | chr5:14299820-14301606 FORW	
JCVI_39440	1.650	moderately similar to (281)AT4G16100| Symbols: | similar to unknown protein [Arabidopsis thaliana] (TAIR:AT5G49220.1); similar to u	
EH425498	1.650	no similarity	
JCVI_35765	1.650	moderately similar to (309)AT1G67340| Symbols: | zinc finger (MYND type) family protein / F-box family protein | chr1:25233986-2523	
JCVI_7976	1.650	highly similar to (659)AT4G30790| Symbols: | similar to protein transport protein-related [Arabidopsis thaliana] (TAIR:AT4G27595.1); s	
JCVI_32982	1.650	moderately similar to (329)AT3G18520| Symbols: HDA15 | HDA15 (HISTONE DEACETYLASE 15); histone deacetylase | chr3:6361611	
EV110650	1.650	no similarity	
DY023408	1.650	no similarity	
EV130275	1.650	no similarity	
EE413170	1.650	moderately similar to (223)AT1G12030| Symbols: | similar to unknown protein [Arabidopsis thaliana] (TAIR:AT1G62420.1); similar to P	
JCVI_24750	1.650	no original description	
AM060092	1.649	moderately similar to (234)AT1G20880| Symbols: | RNA recognition motif (RRM)-containing protein | chr1:7262868-7264823 REVERSI	
EE504551	1.649	moderately similar to (206)AT5G63060| Symbols: | transporter | chr5:25312308-25313940 REVERSE [20198]	
EX045106	1.649	weakly similar to (196)AT4G39780| Symbols: | AP2 domain-containing transcription factor, putative | chr4:18458210-18459028 REVERS	1.419
JCVI_31569	1.649	moderately similar to (249)AT5G49460| Symbols: ACLB-2 | ACLB-2 (ATP-citrate lyase B-2) | chr5:20072274-20075421 FORWARD no	
CD823382	1.649	moderately similar to (336)AT4G17380| Symbols: MSH4 | MSH4 (MUTS-LIKE PROTEIN 4); ATP binding / damaged DNA binding | chi	
ES987314	1.649	moderately similar to (310)AT5G44050| Symbols: | MATE efflux family protein | chr5:17739711-17743436 FORWARD [21425]	
JCVI_16094	1.649	moderately similar to (298)AT3G55605| Symbols: | mitochondrial glycoprotein family protein / MAM33 family protein | chr3:20633647-2	
EX129843	1.649	weakly similar to (135)AT4G11220| Symbols: BTI2 | BTI2 (VIRB2-INTERACTING PROTEIN 2) | chr4:6838172-6839574 REVERSE [2	
JCVI_8604	1.649	moderately similar to (411)AT2G16280| Symbols: | very-long-chain fatty acid condensing enzyme, putative | chr2:7058268-7059806 FOR	
JCVI_27067	1.649	moderately similar to (234)AT1G23465| Symbols: | signal peptidase-related | chr1:8330044-8330927 FORWARD no original description	-1.435
JCVI_18675	1.648	moderately similar to (263)AT1G15950| Symbols: IRX4, ATCCR1, CCR1 | CCR1 (CINNAMOYL COA REDUCTASE 1) | chr1:5478849	
JCVI_15904	1.648	moderately similar to (399)AT5G40760| Symbols: G6PD6 | G6PD6 (GLUCOSE-6-PHOSPHATE DEHYDROGENASE 6); glucose-6-pho	
JCVI_28633	1.648	moderately similar to (222)AT5G07640| Symbols: | zinc finger (C3HC4-type RING finger) family protein | chr5:2414848-2415877 FORW	
JCVI_25863	1.648	highly similar to (614)AT3G14940| Symbols: ATPPC3 | ATPPC3 (PHOSPHOENOLPYRUVATE CARBOXYLASE 3); phosphoenolpyru	
DY030108	1.648	weakly similar to (159)AT1G12820| Symbols: AFB3 | AFB3 (AUXIN SIGNALING F-BOX 3); auxin binding / ubiquitin-protein ligase | ch	
EV108230	1.648	weakly similar to (130)AT1G62370| Symbols: | zinc finger (C3HC4-type RING finger) family protein | chr1:23076240-23076854 FORWA	
JCVI_12381	1.648	moderately similar to (245)AT3G46490| Symbols: | oxidoreductase, acting on paired donors, with incorporation or reduction of molecular	
JCVI_11726	1.648	highly similar to (898)AT1G30360| Symbols: ERD4 | ERD4 (EARLY-RESPONSIVE TO DEHYDRATION 4) | chr1:10715874-10718781	
EX115059	1.648	weakly similar to (139)AT2G47990| Symbols: EDA13, EDA19, SWA1 | SWA1 (SLOW WALKER1); nucleotide binding | chr2:19644080-	
JCVI_4253	1.648	moderately similar to (349)AT3G28970| Symbols: AAR3 | AAR3 (ANTIAUXIN-RESISTANT 3) | chr3:10989207-10991019 REVERSE n	
EG020213	1.648	no similarity	
JCVI_10494	1.648	moderately similar to (423)AT5G52450| Symbols: | MATE efflux protein-related | chr5:21306268-21308975 REVERSE no original descr	
BQ791057	1.647	very weakly similar to (81.3)AT4G14090| Symbols: | UDP-glucoronosyl/UDP-glucosyl transferase family protein | chr4:8122429-8123799	
CX280611	1.647	moderately similar to (338)AT1G66510| Symbols: | AAR2 protein family | chr1:24816544-24819467 FORWARD [16818]	
JCVI_6517	1.647	moderately similar to (268)AT5G07690| Symbols: ATMYB29, PMG2, MYB29 | MYB29 (myb domain protein 29); DNA binding / transc	
ES938975	1.647	moderately similar to (242)AT1G67970| Symbols: HSFA8, AT-HSFA8 | AT-HSFA8 (Arabidopsis thaliana heat shock transcription factor	
EV209059	1.647	very weakly similar to (80.9)AT5G40810| Symbols: | cytochrome c1, putative | chr5:16357428-16359555 FORWARD [21491]	
JCVI_38901	1.646	very weakly similar to (95.5)AT5G27660| Symbols: | serine-type peptidase/ trypsin | chr5:9789902-9792238 REVERSE no original descrip	
DY028457	1.646	weakly similar to (104)AT5G12310| Symbols: | zinc finger (C3HC4-type RING finger) family protein | chr5:3980486-3982094 REVERSE	
EV113394	1.646	very weakly similar to (81.3)AT4G08395| Symbols: | unknown protein | chr4:5319631-5320709 FORWARD [21479]	

ES940807	1.646	weakly similar to (156)AT3G15660| Symbols: | glutaredoxin family protein | chr3:5308141-5309390 REVERSE [21391]	
EX017576	1.646	moderately similar to (362)AT1G27470| Symbols: | transducin-related / WD-40 repeat protein-related | chr1:9540355-9544205 REVERSE	
JCVI_9951	1.646	moderately similar to (255)AT3G60140| Symbols: SRG2, DIN2 | DIN2 (DARK INDUCIBLE 2); hydrolase, hydrolyzing O-glycosyl compo	
JCVI_28071	1.646	no original description	
CD822443	1.645	moderately similar to (337)AT5G06050| Symbols: | dehydration-responsive protein-related | chr5:1820197-1823573 FORWARD [13979]	
JCVI_23161	1.645	highly similar to (585)AT1G66150| Symbols: TMK1 | TMK1 (TRANSMEMBRANE KINASE 1) | chr1:24635166-24638078 FORWARD	
EV150861	1.645	no similarity	
RC_ES979435	1.645	no similarity	
EV140348	1.645	no similarity	
EX092055	1.645	weakly similar to (107)AT3G28720| Symbols: | similar to unknown protein [Arabidopsis thaliana] (TAIR:AT5G58100.1); similar to hypot	
ES936284	1.645	no similarity	
JCVI_6475	1.644	weakly similar to (125)AT5G15510| Symbols: | similar to unknown protein [Arabidopsis thaliana] (TAIR:AT3G01015.1); similar to unna	
EL590443	1.644	no similarity	
JCVI_42481	1.644	no original description	
JCVI_7557	1.643	weakly similar to (154)AT2G28340| Symbols: | zinc finger (GATA type) family protein | chr2:12110749-12113249 FORWARD no origin	
JCVI_36037	1.643	moderately similar to (360)AT1G14680| Symbols: | similar to structural molecule [Arabidopsis thaliana] (TAIR:AT4G09060.1); similar to	
EX108375	1.643	moderately similar to (425)AT5G07010| Symbols: | sulfotransferase family protein | chr5:2174961-2176040 REVERSE [21827]	
JCVI_21882	1.643	moderately similar to (357)AT3G14067| Symbols: | subtilase family protein | chr3:4658428-4660761 REVERSE no original description	
EX096783	1.643	no similarity	-1.365
JCVI_3563	1.643	moderately similar to (372)AT3G17800| Symbols: | mRNA level of the MEB5.2 gene (At3g17800) remains unchanged after cutting the in	
ES948651	1.643	weakly similar to (114)AT3G22430| Symbols: | similar to hypothetical protein [Vitis vinifera] (GB:CAN69769.1) | chr3:7953462-7954490	1.410
JCVI_21035	1.642	weakly similar to (114)AT1G20090| Symbols: ARAC4, ROP2, ATROP2, ATRAC4 | ARAC4/ATRAC4/ATROP2/ROP2 (RHO-RELATED	
JCVI_2282	1.642	moderately similar to (303)AT4G20020| Symbols: | similar to unknown protein [Arabidopsis thaliana] (TAIR:AT5G44780.1); similar to C	
JCVI_12001	1.642	moderately similar to (259)AT2G35940| Symbols: EDA29, BLH1 | BLH1 (embryo sac development arrest 29) | chr2:15096250-15098778	
EV029456	1.642	weakly similar to (115)AT5G15080| Symbols: | protein kinase, putative | chr5:4886417-4888558 FORWARD [21441]	
EV002272	1.642	no similarity	
EE445772	1.642	no similarity	
ES984520	1.642	moderately similar to (272)AT5G19070| Symbols: | similar to unknown protein [Arabidopsis thaliana] (TAIR:AT1G03260.1); similar to u	
EV226272	1.642	no similarity	
CD835296	1.642	moderately similar to (283)AT3G20930| Symbols: | RNA recognition motif (RRM)-containing protein | chr3:7331745-7333755 FORWAR	-1.939
JCVI_13574	1.642	weakly similar to (156)AT3G44200| Symbols: ATNEK6, NEK6, IBO1 | ATNEK6/IBO1/NEK6 (NIMA (NEVER IN MITOSIS, GENE A)-	
EE534687	1.642	moderately similar to (229)AT1G77220| Symbols: | similar to unknown protein [Arabidopsis thaliana] (TAIR:AT4G38360.2); similar to u	
JCVI_22371	1.642	weakly similar to (119)AT1G07570| Symbols: APK1, APK1A | APK1A (Arabidopsis protein kinase 1A); kinase | chr1:2331366-2333207 I	
EX119769	1.642	weakly similar to (183)AT4G40080| Symbols: | epsin N-terminal homology (ENTH) domain-containing protein / clathrin assembly protein	
JCVI_29644	1.641	moderately similar to (233)AT1G76730| Symbols: | 5-formyltetrahydrofolate cyclo-ligase family protein | chr1:28807927-28809597 REV	
JCVI_40984	1.641	moderately similar to (285)AT2G43500| Symbols: | RWP-RK domain-containing protein | chr2:18069792-18073527 FORWARD no origi	
ES963657	1.641	no similarity	
EE423230	1.641	moderately similar to (211)AT1G69800| Symbols: | CBS domain-containing protein | chr1:26278079-26279988 REVERSE [20146] 1 587	
JCVI_38933	1.640	moderately similar to (282)AT1G01360| Symbols: | similar to unknown protein [Arabidopsis thaliana] (TAIR:AT4G01026.1); similar to h	
JCVI_4279	1.640	moderately similar to (247)AT1G47960| Symbols: ATC/VIF1, C/VIF1 | C/VIF1 (CELL WALL / VACUOLAR INHIBITOR OF FRUCTO	
JCVI_16353	1.640	moderately similar to (385)AT4G34660| Symbols: | SH3 domain-containing protein 2 (SH3P2) | chr4:16545600-16548299 REVERSE no	
EH417509	1.640	moderately similar to (375)AT3G45420| Symbols: | lectin protein kinase family protein | chr3:16668248-16670251 REVERSEweakly simi	
CX193806	1.640	no similarity	
CX270435	1.640	no similarity	
JCVI_31920	1.640	moderately similar to (234)AT4G21150| Symbols: | ribophorin II (RPN2) family protein | chr4:11278656-11283609 FORWARD no origin	
ES923256	1.639	weakly similar to (118)AT2G31290| Symbols: | similar to unknown protein [Arabidopsis thaliana] (TAIR:AT3G63090.1); similar to hypot	-2.271
EV041241	1.639	moderately similar to (301)AT3G10572| Symbols: | 3-phosphoinositide-dependent protein kinase-1, putative | chr3:3304351-3306311 FOI	
EE543685	1.639	moderately similar to (214)AT4G11330| Symbols: ATMPK5 | ATMPK5 (MAP KINASE 5); MAP kinase/ kinase | chr4:6892840-6893840	
DT469117	1.639	weakly similar to (200)AT5G66910| Symbols: | disease resistance protein (CC-NBS-LRR class), putative | chr5:26735564-26738359 REV	
JCVI_30499	1.639	moderately similar to (366)AT2G17040| Symbols: ANAC036 | ANAC036 (Arabidopsis NAC domain containing protein 36); transcription	
ES978072	1.638	no similarity	
JCVI_38570	1.638	moderately similar to (354)AT3G13620| Symbols: | amino acid permease family protein | chr3:4450911-4452563 REVERSE no original d	
JCVI_35521	1.638	moderately similar to (305)AT3G09270| Symbols: ATGSTU8 | ATGSTU8 (Arabidopsis thaliana Glutathione S-transferase (class tau) 8); g	
CD825058	1.638	weakly similar to (150)AT1G67840| Symbols: | ATP-binding region, ATPase-like domain-containing protein | chr1:25437819-25440502 F	
EX131753	1.638	moderately similar to (382)AT2G22420| Symbols: | peroxidase 17 (PER17) (P17) | chr2:9520421-9521564 FORWARDweakly similar to (	
EX086382	1.638	moderately similar to (249)AT1G30690| Symbols: | SEC14 cytosolic factor family protein / phosphoglyceride transfer family protein | chr	
JCVI_36710	1.638	moderately similar to (251)AT3G15940| Symbols: | glycosyl transferase family 1 protein | chr3:5393638-5396193 REVERSE no original d	
JCVI_7842	1.638	weakly similar to (121)AT2G14900| Symbols: | gibberellin-regulated family protein | chr2:6411292-6412125 FORWARD no original desc	
EV207263	1.638	moderately similar to (201)AT3G18295| Symbols: | similar to unknown protein [Arabidopsis thaliana] (TAIR:AT1G48770.1); similar to u	
ES900129	1.637	moderately similar to (245)AT5G40240| Symbols: | nodulin MtN21 family protein | chr5:16099553-16101694 REVERSE [21428]	
EE546974	1.637	moderately similar to (215)AT5G48580| Symbols: FKBP15-2 | FKBP15-2 (FK506-binding protein 15 kD-2); FK506 binding / peptidyl-pro	
EV023357	1.637	weakly similar to (145)AT1G07980| Symbols: | histone-like transcription factor (CBF/NF-Y) family protein | chr1:2473520-2474907 REV	
EX042521	1.637	weakly similar to (180)AT3G29090| Symbols: | pectinesterase family protein | chr3:11075041-11076572 FORWARD [21811]	
CD832633	1.637	weakly similar to (133)AT2G31370| Symbols: | bZIP transcription factor (POSF21) | chr2:13386525-13388114 FORWARD [13980]	1.621
JCVI_22122	1.637	moderately similar to (239)AT3G58350| Symbols: | meprin and TRAF homology domain-containing protein / MATH domain-containing p	
CD822728	1.637	moderately similar to (206)AT5G66170| Symbols: | similar to unknown protein [Arabidopsis thaliana] (TAIR:AT2G17850.1); similar to u	
JCVI_28426	1.637	weakly similar to (158)AT4G36730| Symbols: GBF1 | GBF1 (G-box binding factor 1); transcription factor | chr4:17309854-17311756 REV	
JCVI_34494	1.637	weakly similar to (155)AT4G00660| Symbols: | DEAD/DEAH box helicase, putative | chr4:274638-277438 FORWARD no original descri	
JCVI_31016	1.637	highly similar to (647)AT1G72090| Symbols: | radical SAM domain-containing protein / TRAM domain-containing protein | chr1:2712727	
EV037367	1.637	no similarity	
JCVI_10000	1.637	highly similar to (817)AT4G05020| Symbols: NDB2 | NDB2 (NAD(P)H DEHYDROGENASE B2); disulfide oxidoreductase | chr4:257275	
JCVI_18922	1.637	moderately similar to (403)AT5G63790| Symbols: ANAC102 | ANAC102 (Arabidopsis NAC domain containing protein 102); transcription	
JCVI_1594	1.637	moderately similar to (366)AT5G54750| Symbols: | transport protein particle (TRAPP) component Bet3, putative | chr5:22259306-222607	
EE530214	1.637	weakly similar to (159)AT3G07050| Symbols: | GTP-binding family protein | chr3:2229608-2232285 REVERSE [16815]	
EV132187	1.636	no similarity	
ES911005	1.636	moderately similar to (477)AT2G37710| Symbols: RLK | RLK (RECEPTOR LECTIN KINASE); kinase | chr2:15822012-15824039 REVE	
EV020465	1.636	no similarity	
CD813571	1.636	moderately similar to (274)AT3G62260| Symbols: | protein phosphatase 2C, putative / PP2C, putative | chr3:23049491-23051366 REVER	
JCVI_8389	1.636	highly similar to (511)AT1G18500| Symbols: MAML-4, IPMS1 | IPMS1/MAML-4 (METHYLTHIOALKYLMALATE SYNTHASE-LIKE	
CN828699	1.636	moderately similar to (205)AT4G30600| Symbols: | signal recognition particle receptor alpha subunit family protein | chr4:14938148-1494	
JCVI_20215	1.636	moderately similar to (416)AT4G31780| Symbols: MGDA, MGD1 | MGD1 (MONOGALACTOSYLDIACYLGLYCEROL SYNTHASE 1	

EE558124	1.635	no similarity	
JCVI_6526	1.635	highly similar to (644)AT1G56340| Symbols: CRT1 | CRT1 (CALRETICULIN 1); calcium ion binding | chr1:21093687-21096295 REVEI	
JCVI_25159	1.635	no original description	
JCVI_28695	1.635	highly similar to (587)AT1G15170| Symbols: | MATE efflux family protein | chr1:5220685-5222751 FORWARD no original description	
JCVI_32581	1.635	moderately similar to (295)AT5G11790| Symbols: | Ndr family protein | chr5:3799683-3802497 FORWARDweakly similar to (197)SF21	2.304
EE560938	1.635	no similarity	
JCVI_22951	1.635	very weakly similar to (90.5)AT5G50850| Symbols: MAB1 | MAB1 (MACCI-BOU); pyruvate dehydrogenase (acetyl-transferring) | chr5:2(	
JCVI_19866	1.635	moderately similar to (221)AT1G29670| Symbols: | GDSL-motif lipase/hydrolase family protein | chr1:10375829-10377703 FORWARD r	
JCVI_14824	1.634	moderately similar to (395)AT3G11280| Symbols: | myb family transcription factor | chr3:3533483-3534399 REVERSE no original descri	
JCVI_15691	1.634	moderately similar to (259)AT5G01260| Symbols: | glycoside hydrolase starch-binding domain-containing protein | chr5:105366-106487 I	
JCVI_3755	1.634	moderately similar to (224)AT1G52280| Symbols: AtRABG3d | AtRABG3d (Arabidopsis Rab GTPase homolog G3d); GTP binding | chr1	
CN728205	1.634	no similarity	
CV650422	1.634	weakly similar to (109)AT4G40030| Symbols: | histone H3.2 | chr4:18555834-18556411 REVERSEweakly similar to (109)H33_VITVI [	
EV145899	1.634	no similarity	
EE566004	1.634	no similarity	
JCVI_19612	1.634	moderately similar to (334)AT1G79090| Symbols: | similar to unknown protein [Arabidopsis thaliana] (TAIR:AT3G22270.1); similar to u	
EE444208	1.634	weakly similar to (166)AT5G13160| Symbols: PBS1 | PBS1 (AVRPPHB SUSCEPTIBLE 1); kinase | chr5:4176857-4179685 FORWARD	
JCVI_6554	1.633	moderately similar to (221)AT1G55810| Symbols: | uracil phosphoribosyltransferase, putative / UMP pyrophosphorylase, putative / UPRT	
EV101751	1.633	no similarity	
JCVI_9234	1.633	weakly similar to (178)AT2G44360| Symbols: | similar to unnamed protein product [Vitis vinifera] (GB:CAO67186.1); contains InterPro (	
ES964955	1.633	no similarity	
JCVI_12406	1.633	no original description	
RC_AM056987	1.633	no similarity	
JCVI_6796	1.633	moderately similar to (378)AT1G48380| Symbols: HYP7, RHL1 | RHL1 (ROOT HAIRLESS 1) | chr1:17881305-17882871 REVERSE no	
EV129458	1.633	weakly similar to (140)AT4G40030| Symbols: | histone H3.2 | chr4:18555834-18556411 REVERSEweakly similar to (136)H33_VITVI [	2.513
EV027655	1.632	moderately similar to (314)AT3G19553| Symbols: | amino acid permease family protein | chr3:6790994-6792513 REVERSE [21441] 1 64	
ES913937	1.632	moderately similar to (243)AT5G13460| Symbols: IQD11 | IQD11 (IQ-domain 11); calmodulin binding | chr5:4316326-4318250 FORWAI	-2.673
AM389775	1.632	weakly similar to (147)AT2G26990| Symbols: ATCSN2, COP12, FUS12 | FUS12 (FUSCA 12) | chr2:11526762-11529490 REVERSE [20	
EX096168	1.632	moderately similar to (305)AT1G15530| Symbols: | receptor lectin kinase, putative | chr1:5339956-5341926 REVERSE [21824]	
JCVI_10315	1.631	moderately similar to (358)AT1G25520| Symbols: | similar to unknown protein [Arabidopsis thaliana] (TAIR:AT1G68650.1); similar to u	1.419
JCVI_23368	1.631	moderately similar to (234)AT4G10300| Symbols: | similar to unknown protein [Arabidopsis thaliana] (TAIR:AT3G04300.1); similar to u	
JCVI_1534	1.631	moderately similar to (400)AT3G24120| Symbols: | myb family transcription factor | chr3:8705932-8708155 REVERSE no original descri	
JCVI_4235	1.631	highly similar to (615)AT1G01120| Symbols: KCS1 | KCS1 (3-KETOACYL-COA SYNTHASE 1); acyltransferase | chr1:57392-58978 RE	
ES919008	1.631	weakly similar to (102)AT4G17670| Symbols: | senescence-associated protein-related | chr4:9833961-9834676 REVERSE [15718]	
EX089495	1.631	moderately similar to (257)AT4G08500| Symbols: ATMEKK1, MAPKKK8, MEKK1 | MEKK1 (MYTOGEN ACTIVATED PROTEIN K	
JCVI_28689	1.630	weakly similar to (161)AT3G56590| Symbols: | hydroxyproline-rich glycoprotein family protein | chr3:20976084-20978763 FORWARD r	
EX100576	1.630	moderately similar to (474)AT4G24560| Symbols: UBP16 | UBP16 (UBIQUITIN-SPECIFIC PROTEASE 16); ubiquitin-specific protease	
JCVI_10978	1.630	weakly similar to (113)AT4G30010| Symbols: | similar to unknown [Populus trichocarpa x Populus deltoides] (GB:ABK96249.1) | chr4:14	
JCVI_29305	1.630	moderately similar to (270)AT4G10610| Symbols: CID12, ATRBP37, RBP37 | RBP37 (RNA-BINDING PROTEIN 37); RNA binding | ch	
JCVI_14992	1.630	highly similar to (687)AT3G08760| Symbols: ATSIK | ATSIK; kinase | chr3:2658135-2659990 REVERSEweakly similar to (142)NORK_	
JCVI_26085	1.630	moderately similar to (399)AT1G79380| Symbols: | copine-related | chr1:29865705-29867916 FORWARD no original description	2.091
JCVI_4735	1.630	moderately similar to (365)AT2G44510| Symbols: | p21Cip1-binding protein-related | chr2:18384487-18386106 FORWARD no original d	
EV192577	1.629	moderately similar to (207)AT5G11580| Symbols: | UVB-resistance protein-related / regulator of chromosome condensation (RCC1) fami	
RC_ES969060	1.629	no similarity	
EE424627	1.629	weakly similar to (157)AT5G11500| Symbols: | similar to unknown [Brassica juncea] (GB:ABX10747.1); contains InterPro domain Prote	
JCVI_10917	1.629	moderately similar to (328)AT1G04130| Symbols: | tetratricopeptide repeat (TPR)-containing protein | chr1:1073464-1075373 FORWARI	
CV546447	1.629	very weakly similar to (87.8)AT4G31200| Symbols: | SWAP (Suppressor-of-White-APricot)/surp domain-containing protein | chr4:151623	
H07460	1.629	no similarity	
JCVI_41111	1.628	no original description	-1.785
JCVI_756	1.628	moderately similar to (316)AT5G40850| Symbols: UPM1 | UPM1 (UROPHORPHYRIN METHYLASE 1); uroporphyrin-III C-methyltran	
JCVI_15629	1.628	moderately similar to (239)AT1G51130| Symbols: | similar to unknown protein [Arabidopsis thaliana] (TAIR:AT3G20760.1); similar to u	
JCVI_9991	1.628	moderately similar to (358)AT4G33950| Symbols: OST1, SNRK2-6, SRK2E, SNRK2.6, P44 | OST1/P44/SNRK2-6/SRK2E (OPEN STOM	
JCVI_22652	1.628	moderately similar to (339)AT1G57590| Symbols: | carboxylesterase | chr1:21331123-21333372 REVERSE no original description	
ES978087	1.628	no similarity	
JCVI_14492	1.628	moderately similar to (224)AT3G59090| Symbols: | similar to TOM1 (TOBAMOVIRUS MULTIPLICATION 1) [Arabidopsis thaliana] (T	
AM387573	1.628	weakly similar to (143)AT4G15280| Symbols: | UDP-glucoronosyl/UDP-glucosyl transferase family protein | chr4:8719182-8720618 FOR	
CX195092	1.627	weakly similar to (159)AT4G13800| Symbols: | permease-related | chr4:8002125-8003854 REVERSE [16807]	
EE411134	1.627	moderately similar to (367)AT1G75660| Symbols: XRN3 | XRN3 (5'-3' exoribonuclease 3); 5'-3' exoribonuclease | chr1:28411950-284184	
JCVI_8072	1.627	moderately similar to (360)AT5G63110| Symbols: AXE1, ATHDA6, RTS1, RPD3B, SIL1, HDA6 | HDA6 (HISTONE DEACETYLASE 6	
JCVI_14859	1.627	moderately similar to (373)AT1G29800| Symbols: | zinc ion binding | chr1:10432720-10435030 FORWARD no original description	
AM061665	1.627	weakly similar to (198)AT4G18040| Symbols: EIF4E, LSP1, CUM1, AT.EIF4E1 | EIF4E (EUKARYOTIC TRANSLATION INITATION	
JCVI_17999	1.627	weakly similar to (154)AT2G46510| Symbols: | basic helix-loop-helix (bHLH) family protein | chr2:19098259-19099959 REVERSE no or	
JCVI_307	1.627	moderately similar to (305)AT2G40010| Symbols: | 60S acidic ribosomal protein P0 (RPP0A) | chr2:16715656-16717526 REVERSEmod	-1.331
EE485315	1.627	very weakly similar to (100)AT4G26020| Symbols: | similar to ATGRIP/GRIP, protein binding [Arabidopsis thaliana] (TAIR:AT5G66030	
EE518688	1.626	very weakly similar to (89.4)AT5G42080| Symbols: ADL1A, AG68, DRP1A | ADL1 (ARABIDOPSIS DYNAMIN-LIKE PROTEIN); GTF	
ES936870	1.626	moderately similar to (431)AT4G25230| Symbols: RIN2 | RIN2 (RPM1 INTERACTING PROTEIN 2); protein binding / zinc ion binding	-2.793
JCVI_19879	1.626	no original description	
EV087116	1.626	no similarity	-1.194
AM385157	1.626	no similarity	-1.466
JCVI_5407	1.626	moderately similar to (321)AT2G15320| Symbols: | leucine-rich repeat family protein | chr2:6673609-6674757 REVERSE no original des	
JCVI_6132	1.626	moderately similar to (261)AT2G36145| Symbols: | similar to unnamed protein product [Vitis vinifera] (GB:CAO15876.1); similar to hyp	-4.479
EV085902	1.626	very weakly similar to (82.4)AT4G26570| Symbols: CBL3 | ATCBL3 (CALCINEURIN B-LIKE 3) | chr4:13408614-13410004 REVERSE	
EV052313	1.626	no similarity	
JCVI_19653	1.626	moderately similar to (221)AT4G32520| Symbols: SHM3 | SHM3 (SERINE HYDROXYMETHYLTRANSFERASE 3) | chr4:15689648-1	
EV120240	1.625	moderately similar to (374)AT4G27600| Symbols: | pfkB-type carbohydrate kinase family protein | chr4:13782759-13785011 REVERSE [	
JCVI_7915	1.625	moderately similar to (286)AT4G24770| Symbols: ATRBP31, CP31, ATRBP33, RBP31 | RBP31 (31-KDA RNA BINDING PROTEIN); I	2.302
JCVI_22926	1.625	moderately similar to (482)AT2G45310| Symbols: GAE4 | GAE4 (UDP-D-GLUCURONATE 4-EPIMERASE 4); catalytic | chr2:1868972	
EV013082	1.625	weakly similar to (127)AT3G13340| Symbols: | WD-40 repeat family protein | chr3:4332377-4334610 FORWARD [21451]	
JCVI_18176	1.625	moderately similar to (348)AT5G48560| Symbols: | basic helix-loop-helix (bHLH) family protein | chr5:19701386-19704097 FORWARD	
EE481389	1.625	weakly similar to (198)AT2G40935| Symbols: | similar to unknown protein [Arabidopsis thaliana] (TAIR:AT3G18470.1); similar to hypot	
JCVI_36056	1.625	moderately similar to (253)AT4G04340| Symbols: | early-responsive to dehydration protein-related / ERD protein-related | chr4:2123233-2	-3.184

JCVI_31342	1.625	moderately similar to (285)AT5G49720| Symbols: DEC, KOR, RSW2, OR16PEP, IRX2, KOR1, AtGH9A1 | AtGH9A1 (ARABIDOPSIS T	
ES983192	1.625	no similarity	
CD819233	1.624	moderately similar to (248)AT1G08700| Symbols: | presenilin family protein | chr1:2769888-2771351 REVERSE [13978]	
EV162063	1.624	weakly similar to (134)AT2G31670| Symbols: | similar to unknown protein [Arabidopsis thaliana] (TAIR:AT1G51360.1); similar to unkno	
JCVI_41786	1.624	moderately similar to (202)AT1G52420| Symbols: | glycosyl transferase family 1 protein | chr1:19532336-19534704 FORWARD no origi	
JCVI_3203	1.624	moderately similar to (301)AT4G17020| Symbols: | transcription factor-related | chr4:9578171-9581101 REVERSE no original descriptio	
JCVI_39336	1.623	weakly similar to (194)AT5G15260| Symbols: | structural constituent of ribosome | chr5:4953649-4954353 REVERSE no original descrip	
JCVI_29538	1.623	moderately similar to (201)AT1G17640| Symbols: | RNA recognition motif (RRM)-containing protein | chr1:6067387-6069091 REVERSI	
JCVI_35190	1.623	moderately similar to (374)AT2G19570| Symbols: AT-CDA1, DESZ, CDA1 | CDA1 (CYTIDINE DEAMINASE 1) | chr2:8477680-84785	
EE550315	1.623	no similarity	
EX141732	1.623	moderately similar to (223)AT5G01990| Symbols: | auxin efflux carrier family protein | chr5:377370-379597 REVERSE [21834]	
EE553527	1.623	no similarity	
JCVI_26280	1.623	no original description	
ES939676	1.622	moderately similar to (362)AT1G80260| Symbols: EMB1427 | EMB1427 (EMBRYO DEFECTIVE 1427); tubulin binding | chr1:3018081	-3.158
JCVI_35966	1.622	moderately similar to (295)AT3G09050| Symbols: | similar to unknown [Populus trichocarpa x Populus deltoides] (GB:ABK96465.1) | ch	
JCVI_18789	1.622	highly similar to (629)AT2G28890| Symbols: PLL4 | PLL4 (POLTERGEIST LIKE 4); protein serine/threonine phosphatase | chr2:124128	1.764
JCVI_7393	1.622	no original description	
JCVI_27039	1.622	highly similar to (590)AT1G21880| Symbols: LYM1 | LYM1 (LYSM DOMAIN GPI-ANCHORED PROTEIN 1 PRECURSOR) | chr1:768	
RC_AM396015	1.622	no similarity	-1.194
JCVI_11224	1.622	highly similar to (653)AT1G74210| Symbols: | glycerophosphoryl diester phosphodiesterase family protein | chr1:27914057-27916446 FC	
EV158732	1.622	moderately similar to (295)AT2G05230| Symbols: | DNAJ heat shock N-terminal domain-containing protein | chr2:1899815-1901935 REV	
JCVI_7124	1.622	moderately similar to (249)AT4G03510| Symbols: RMA1 | RMA1 (Ring finger protein with Membrane Anchor 1); protein binding / ubiqu	
EE513340	1.622	weakly similar to (115)AT3G23960| Symbols: | F-box family protein | chr3:8657743-8658951 FORWARD [15713]	1.472
JCVI_9162	1.622	highly similar to (574)AT3G55450| Symbols: | protein kinase, putative | chr3:20569106-20570940 FORWARDmoderately similar to (241	
EV136303	1.621	moderately similar to (234)AT1G69780| Symbols: ATHB13 | ATHB13; DNA binding / transcription factor | chr1:26262829-26264128 FO	
EX049784	1.621	weakly similar to (196)AT1G30320| Symbols: | remorin family protein | chr1:10680330-10682834 FORWARD [21812]	
JCVI_15548	1.621	no original description	
CD821605	1.621	no similarity	
EE548690	1.621	weakly similar to (135)AT3G10940| Symbols: | protein phosphatase-related | chr3:3422264-3423399 REVERSE [20184] 40 450 450	
EX136822	1.621	moderately similar to (401)AT5G57140| Symbols: ATPAP28, PAP28 | ATPAP28/PAP28 (purple acid phosphatase 28); acid phosphatase/	
JCVI_9983	1.621	weakly similar to (179)AT5G12140| Symbols: ATCYS1 | ATCYS1 (A. THALIANA CYSTATIN-1); cysteine protease inhibitor | chr5:392	
JCVI_3075	1.621	moderately similar to (292)AT1G52140| Symbols: | similar to unknown protein [Arabidopsis thaliana] (TAIR:AT3G16330.1); similar to h	2.688
JCVI_15218	1.620	moderately similar to (332)AT3G10420| Symbols: | sporulation protein-related | chr3:3239312-3241576 FORWARD no original descriptio	
EV020039	1.620	weakly similar to (171)AT1G28070| Symbols: | protein binding | chr1:9783069-9783851 FORWARD [21441] 16 552 552	
DY018446	1.620	no similarity	
JCVI_13569	1.620	moderately similar to (233)AT4G25080| Symbols: CHLM | CHLM (MAGNESIUM-PROTOPORPHYRIN IX METHYLTRANSFERASE	
DY015889	1.620	moderately similar to (292)AT1G77010| Symbols: | pentatricopeptide (PPR) repeat-containing protein | chr1:28947604-28949691 FORW/	
JCVI_20861	1.620	weakly similar to (160)AT5G11630| Symbols: | similar to transcription factor [Arabidopsis thaliana] (TAIR:AT4G17310.2); similar to unl	1.697
EV144207	1.620	weakly similar to (101)AT2G23950| Symbols: | leucine-rich repeat family protein / protein kinase family protein | chr2:10194284-1019704	
JCVI_39048	1.620	no original description	
EE402103	1.619	weakly similar to (197)AT4G09560| Symbols: | peptidase/ protein binding / zinc ion binding | chr4:6041649-6043678 REVERSE [20197]	
EV208549	1.619	no similarity	
EX026005	1.619	moderately similar to (260)AT5G24800| Symbols: ATBZIP9, BZO2H2 | ATBZIP9/BZO2H2 (BASIC LEUCINE ZIPPER O2 HOMOLOG	
JCVI_31204	1.619	moderately similar to (424)AT1G22370| Symbols: ATUGT85A5 | ATUGT85A5 (UDP-GLUCOSYL TRANSFERASE 85A5); glucuronosy	-2.013
EV085798	1.619	no similarity	3.947
JCVI_16777	1.619	moderately similar to (226)AT1G28190| Symbols: | similar to unknown protein [Arabidopsis thaliana] (TAIR:AT5G12340.1); similar to u	
JCVI_19034	1.619	highly similar to (517)AT2G02860| Symbols: ATSUC3, SUC3, SUT2 | SUT2 (sucrose transporter 3); carbohydrate transmembrane transpo	
ES979789	1.619	no similarity	
JCVI_25278	1.619	moderately similar to (475)AT4G13980| Symbols: HSFA5, AT-HSFA5 | AT-HSFA5 (Arabidopsis thaliana heat shock transcription factor /	
JCVI_2021	1.619	no original description	
JCVI_27950	1.618	moderately similar to (306)AT2G45550| Symbols: CYP76C4 | CYP76C4 (cytochrome P450, family 76, subfamily C, polypeptide 4); oxyg	
JCVI_25952	1.618	highly similar to (503)AT5G56680| Symbols: EMB2755, SYNC1 | SYNC1 (EMBRYO DEFECTIVE 2755); ATP binding / aminoacyl-tRN	-1.349
JCVI_31193	1.618	moderately similar to (277)AT1G21065| Symbols: | similar to unnamed protein product [Vitis vinifera] (GB:CAO66320.1); contains Inter	
EX126631	1.618	very weakly similar to (89.7)AT2G35500| Symbols: | shikimate kinase-related | chr2:14921117-14922988 FORWARD [21831] 1 458 477	1.835
EV157605	1.618	moderately similar to (250)AT2G23200| Symbols: | protein kinase family protein | chr2:9886431-9888935 FORWARD [21484]	
EX134440	1.618	no similarity	1.529
EE505344	1.618	weakly similar to (112)AT5G47560| Symbols: ATTDT, ATSDAT | ATSDAT/ATTDT (TONOPLAST DICARBOXYLATE TRANSPORT	
CD837137	1.618	moderately similar to (348)AT4G24880| Symbols: | similar to unnamed protein product [Vitis vinifera] (GB:CAO68295.1); contains doma	
AM060123	1.618	no similarity	
JCVI_38356	1.618	moderately similar to (217)AT1G48860| Symbols: | 3-phosphoshikimate 1-carboxyvinyltransferase, putative / 5-enolpyruvylshikimate-3-p	1.603
JCVI_144	1.618	moderately similar to (365)AT2G20920| Symbols: | similar to unnamed protein product [Vitis vinifera] (GB:CAO47410.1) | chr2:9005809	1.823
JCVI_22536	1.617	moderately similar to (420)AT1G43910| Symbols: | AAA-type ATPase family protein | chr1:16658490-16660068 REVERSE no original d	
JCVI_3734	1.617	moderately similar to (404)AT5G27380| Symbols: GSH2, GSHB | GSH2/GSHB (GLUTATHIONE SYNTHETASE 2); glutathione synthas	
ES906904	1.617	moderately similar to (457)AT1G33540| Symbols: SCPL18 | SCPL18 (serine carboxypeptidase-like 18); serine carboxypeptidase | chr1:12	
EX040871	1.617	weakly similar to (121)AT4G36090| Symbols: | oxidoreductase, 2OG-Fe(II) oxygenase family protein | chr4:17078379-17080673 REVER	
JCVI_27201	1.617	weakly similar to (176)AT3G13570| Symbols: SCL30a | SCL30a (SC35-like splicing factor 30a); RNA binding | chr3:4429571-4431609 R	
JCVI_15903	1.617	moderately similar to (342)AT2G43590| Symbols: | chitinase, putative | chr2:18088669-18089826 REVERSEmoderately similar to (333)(	
JCVI_42639	1.617	moderately similar to (289)AT1G24267| Symbols: | similar to unknown protein [Arabidopsis thaliana] (TAIR:AT1G24265.2); similar to u	-2.014
EV092689	1.616	no similarity	
JCVI_20952	1.616	very weakly similar to (81.6)AT3G18290| Symbols: EMB2454 | EMB2454 (EMBRYO DEFECTIVE 2454); protein binding / zinc ion bind	
JCVI_11672	1.616	weakly similar to (161)AT1G16610| Symbols: SR45 | SR45 (arginine/serine-rich 45); RNA binding | chr1:5675918-5678679 REVERSE nc	
EE450406	1.616	no similarity	1.564
JCVI_3391	1.616	moderately similar to (393)AT5G44410| Symbols: | FAD-binding domain-containing protein | chr5:17908473-17910080 REVERSE no ori	
JCVI_7718	1.616	highly similar to (540)AT5G64120| Symbols: | peroxidase, putative | chr5:25676777-25678172 REVERSEmoderately similar to (384)PEI	
JCVI_8309	1.616	no original description	1.345
JCVI_24417	1.616	moderately similar to (258)AT3G45100| Symbols: SETH2 | SETH2; transferase, transferring glycosyl groups | chr3:16515633-16517843 F	
EX042196	1.616	moderately similar to (282)AT3G04820| Symbols: | RNA binding / pseudouridylate synthase/ tRNA-pseudouridine synthase | chr3:132145	
DY008444	1.616	weakly similar to (139)AT1G22490| Symbols: | basic helix-loop-helix (bHLH) family protein | chr1:7938437-7940478 REVERSE [18972]	
CD815931	1.615	moderately similar to (292)AT1G04570| Symbols: | integral membrane transporter family protein | chr1:1246858-1248599 REVERSE [13	
ES922562	1.615	no similarity	
JCVI_4157	1.615	no original description	

EV072989	1.615	moderately similar to (258)AT1G63700| Symbols: MAPKKK4, YDA | YDA (YODA); kinase | chr1:23628871-23632694 REVERSE [214	
DY022533	1.615	weakly similar to (133)AT1G11510| Symbols: | DNA-binding storekeeper protein-related | chr1:3871778-3872836 REVERSE [18979]	
JCVI_29205	1.615	moderately similar to (412)AT2G04305| Symbols: | magnesium transporter CorA-like protein-related | chr2:1501676-1503445 REVERSE	
JCVI_16546	1.615	moderately similar to (398)AT1G69780| Symbols: ATHB13 | ATHB13; DNA binding / transcription factor | chr1:26262829-26264128 FO	
EE532194	1.615	moderately similar to (233)AT4G12570| Symbols: UPL5 | UPL5 (UBIQUITIN PROTEIN LIGASE 5); ubiquitin-protein ligase | chr4:7445	
DY027697	1.615	weakly similar to (167)AT5G60850| Symbols: OBP4 | OBP4 (OBF BINDING PROTEIN 4); DNA binding / transcription factor | chr5:244	
JCVI_18847	1.615	moderately similar to (299)AT4G33625| Symbols: | similar to hypothetical protein MtrDRAFT_AC148971g6v2 [Medicago truncatula] (G	
ES913345	1.615	no similarity	
EE556451	1.615	no similarity	-1.504
AM391525	1.615	moderately similar to (314)AT5G16210| Symbols: | HEAT repeat-containing protein | chr5:5291002-5297782 REVERSE [20118]	
RC_EE565820	1.615	no similarity	
JCVI_39660	1.615	moderately similar to (428)AT1G28570| Symbols: | GDSL-motif lipase, putative | chr1:10041824-10044098 REVERSEweakly similar to (	
ES922471	1.614	moderately similar to (228)AT1G18480| Symbols: | calcineurin-like phosphoesterase family protein | chr1:6361632-6362807 FORWARD	
EE413200	1.614	weakly similar to (186)AT1G34120| Symbols: AT5P1, ATIP5PI, IP5PI | IP5PI (INOSITOL POLYPHOSPHATE 5-PHOSPHATASE I); ir	
EV004929	1.614	moderately similar to (216)AT3G63220| Symbols: | kelch repeat-containing F-box family protein | chr3:23368515-23369573 REVERSE [	
JCVI_1415	1.614	moderately similar to (255)AT5G21090| Symbols: | leucine-rich repeat protein, putative | chr5:7164760-7166906 FORWARDvery weakly	
EG020786	1.614	weakly similar to (112)AT3G12080| Symbols: EMB2738 | EMB2738 (EMBRYO DEFECTIVE 2738) | chr3:3847857-3851683 FORWAR	
JCVI_16654	1.613	moderately similar to (317)AT4G38550| Symbols: | similar to unknown protein [Arabidopsis thaliana] (TAIR:AT2G20950.1); contains In	-1.616
JCVI_15510	1.613	highly similar to (796)AT2G32700| Symbols: | WD-40 repeat family protein | chr2:13874312-13878921 FORWARD no original descriptic	
JCVI_15358	1.613	weakly similar to (177)AT3G13130| Symbols: | similar to hypothetical protein [Vitis vinifera] (GB:CAN76025.1) | chr3:4223015-422362	
JCVI_37963	1.613	weakly similar to (135)AT2G33410| Symbols: | heterogeneous nuclear ribonucleoprotein, putative / hnRNP, putative | chr2:14163161-141	
EE470645	1.613	no similarity	
JCVI_18098	1.613	moderately similar to (370)AT5G60220| Symbols: TET4 | TET4 (TETRASPANIN4) | chr5:24266183-24267291 FORWARD no original d	
RC_ES950660	1.613	no similarity	
JCVI_20266	1.613	no original description	
EE566040	1.613	weakly similar to (144)AT3G56220| Symbols: | transcription regulator | chr3:20869920-20871281 FORWARD [20153] 21 741 768	
ES929183	1.613	weakly similar to (126)AT3G07080| Symbols: | membrane protein | chr3:2241366-2242940 FORWARD [20185]	-1.329
ES913245	1.613	highly similar to (528)AT5G19730| Symbols: | pectinesterase family protein | chr5:6670564-6673204 FORWARDweakly similar to (155)	
BG544279	1.613	weakly similar to (159)AT4G09650| Symbols: | ATP synthase delta chain, chloroplast, putative / H(+)-transporting two-sector ATPase, de	
JCVI_12030	1.613	moderately similar to (313)AT2G36885| Symbols: | similar to unnamed protein product [Vitis vinifera] (GB:CAO63025.1) | chr2:1548933	
JCVI_5206	1.612	moderately similar to (261)AT2G28660| Symbols: | copper-binding family protein | chr2:12302496-12304021 FORWARD no original des	
JCVI_5421	1.612	moderately similar to (310)AT5G54540| Symbols: | similar to unknown protein [Arabidopsis thaliana] (TAIR:AT4G25170.1); similar to u	
JCVI_35025	1.612	highly similar to (625)AT3G50820| Symbols: PSBO2, PSBO-2 | PSBO-2/PSBO2 (PHOTOSYSTEM II SUBUNIT O-2); oxygen evolving/	-1.947
EE560645	1.612	no similarity	
JCVI_28840	1.612	moderately similar to (390)AT5G56750| Symbols: | Ndr family protein | chr5:22975212-22977832 FORWARDmoderately similar to (29	
ES945698	1.611	moderately similar to (208)AT3G18600| Symbols: | DEAD/DEAH box helicase, putative | chr3:6399730-6403013 REVERSE [21393]	
JCVI_19568	1.611	weakly similar to (162)AT3G06450| Symbols: | anion exchange family protein | chr3:1976091-1979309 REVERSE no original description	
EE561234	1.611	no similarity	
JCVI_9173	1.611	moderately similar to (271)AT1G22220| Symbols: | F-box family protein | chr1:7846683-7847627 FORWARD no original description	
JCVI_24876	1.611	moderately similar to (394)AT3G48150| Symbols: CDC23, APC8 | APC8 (anaphase-promoting complex/cyclosome 8); binding | chr3:177	
JCVI_9539	1.611	moderately similar to (333)AT2G28840| Symbols: | ankyrin repeat family protein | chr2:12385749-12387551 FORWARD no original desc	
EV087077	1.611	no similarity	
JCVI_22863	1.611	moderately similar to (346)AT4G25100| Symbols: FSD1 | FSD1 (FE SUPEROXIDE DISMUTASE 1); iron superoxide dismutase | chr4:12	
ES957754	1.611	no similarity	
RC_EE553259	1.611	no similarity	
BG543200	1.610	no similarity	
EV059671	1.610	moderately similar to (461)AT4G22580| Symbols: | exostosin family protein | chr4:11889393-11890700 REVERSEweakly similar to (14	
JCVI_39824	1.610	moderately similar to (432)AT2G18915| Symbols: ADO2, LKP2 | LKP2 (LOV KELCH PROTEIN 2); ubiquitin-protein ligase | chr2:8201	
EV000762	1.610	weakly similar to (155)AT5G58040| Symbols: ATFIP1[V] | ATFIP1[V] (ARABIDOPSIS HOMOLOG OF YEAST FIP1 [V]); RNA bindir	3.362
EE445265	1.610	weakly similar to (154)AT5G52870| Symbols: | similar to unknown protein [Arabidopsis thaliana] (TAIR:AT1G64080.1); similar to unna	
H07541	1.610	no similarity	
JCVI_22298	1.610	no original description	-1.778
EE470519	1.609	moderately similar to (468)AT1G08420| Symbols: | kelch repeat-containing protein / serine/threonine phosphoesterase family protein | chr	
JCVI_6875	1.609	weakly similar to (127)AT3G10070| Symbols: TAF12, TAFII58 | TAF12/TAFII58 (TBP-ASSOCIATED FACTOR 12); DNA binding / tra	-1.021
JCVI_34558	1.609	moderately similar to (371)AT5G25180| Symbols: CYP71B14 | CYP71B14 (cytochrome P450, family 71, subfamily B, polypeptide 14); o	
DN964562	1.609	weakly similar to (147)AT1G74910| Symbols: | ADP-glucose pyrophosphorylase family protein | chr1:28139431-28142117 REVERSE [1	
JCVI_13821	1.609	moderately similar to (235)AT4G31550| Symbols: ATWRKY11, WRKY11 | WRKY11 (WRKY DNA-binding protein 11); transcription fa	
EE557844	1.609	no similarity	
EV052214	1.608	moderately similar to (221)AT5G60740| Symbols: | ABC transporter family protein | chr5:24443298-24447495 REVERSE [21442]	
JCVI_24150	1.608	very weakly similar to (94.7)AT1G51745| Symbols: | Identical to Uncharacterized protein At1g51745 [Arabidopsis Thaliana] (GB:P59278	
EV099306	1.608	weakly similar to (191)AT2G02180| Symbols: TOM3 | TOM3 (tobamovirus multiplication protein 3) | chr2:560975-562960 FORWARD [	
EE550357	1.608	moderately similar to (383)AT5G24360| Symbols: | protein kinase family protein / Ire1 homolog-1 (IRE1-1) | chr5:8316630-8319830 FOR	-3.720
JCVI_18433	1.608	moderately similar to (258)AT2G32920| Symbols: ATPDIL2-3 | ATPDIL2-3 (PDI-LIKE 2-3); protein disulfide isomerase | chr2:1396957	2.055
ES906980	1.608	moderately similar to (481)AT1G31070| Symbols: | UDP-N-acetylglucosamine pyrophosphorylase-related | chr1:11084932-11088342 FO	
JCVI_623	1.608	moderately similar to (468)AT2G39010| Symbols: PIP2E, PIP2;6 | PIP2;6/PIP2E (plasma membrane intrinsic protein 2;6); water channel |	
JCVI_10623	1.608	moderately similar to (336)AT3G09740| Symbols: ATSYP71, SYP71 | SYP71 (SYNTAXIN OF PLANTS 71) | chr3:2989620-2991359 FC	
AM062379	1.607	weakly similar to (123)AT5G03455| Symbols: ARATH;CDC25, ACR2, CDC25 | CDC25 | chr5:862591-863948 FORWARD [17712]	
EV113298	1.607	moderately similar to (367)AT2G30740| Symbols: | serine/threonine protein kinase, putative | chr2:13103476-13105362 FORWARDweak	
JCVI_29384	1.607	highly similar to (736)AT1G22940| Symbols: TH-1, TH1 | TH1 (THIAMINE REQUIRING 1); catalytic/ phosphomethylpyrimidine kinase	
JCVI_26197	1.607	weakly similar to (164)AT4G20300| Symbols: | similar to unknown protein [Arabidopsis thaliana] (TAIR:AT1G55340.1); similar to unkno	
JCVI_23907	1.607	very weakly similar to (84.0)AT4G26080| Symbols: ABI1 | ABI1 (ABA INSENSITIVE 1); calcium ion binding / protein serine/threonine p	-1.467
JCVI_4282	1.607	moderately similar to (416)AT5G07440| Symbols: GDH2 | GDH2 (GLUTAMATE DEHYDROGENASE 2) | chr5:2356154-2357547 FOR	
DN192274	1.607	moderately similar to (277)AT4G27710| Symbols: CYP709B3 | CYP709B3 (cytochrome P450, family 709, subfamily B, polypeptide 3); o	
JCVI_35220	1.607	no original description	
EE443421	1.607	weakly similar to (190)AT1G07570| Symbols: APK1, APK1A | APK1A (Arabidopsis protein kinase 1A); kinase | chr1:2331366-2333207 I	
CD826650	1.607	moderately similar to (317)AT4G32730| Symbols: PC-MYB1, ATMYB3R1, MYB3R-1, ATMYB3R-1 | PC-MYB1 (myb domain protein 3	1.668
DY024454	1.607	moderately similar to (212)AT2G46160| Symbols: | zinc finger (C3HC4-type RING finger) family protein | chr2:18970182-18970826 FOI	
ES998686	1.607	weakly similar to (123)AT4G26960| Symbols: | similar to unknown protein [Arabidopsis thaliana] (TAIR:AT5G54970.1) | chr4:13538929	
JCVI_5214	1.606	moderately similar to (378)AT2G36830| Symbols: TIP1;1, GAMMA-TIP1, GAMMA-TIP | GAMMA-TIP (Tonoplast intrinsic protein (TI	
ES949746	1.606	no similarity	
JCVI_2690	1.606	moderately similar to (359)AT3G09840| Symbols: ATCDC48, CDC48A, CDC48 | CDC48 (CELL DIVISION CYCLE 48); ATPase | chr3:	

ES264362	1.606	no similarity	
CN727016	1.606	weakly similar to (107)AT2G44510| Symbols: | p21Cip1-binding protein-related | chr2:18384487-18386106 FORWARD [15722]	
JCVI_31370	1.606	moderately similar to (444)AT3G19740| Symbols: | ATPase | chr3:6855950-6862936 REVERSEweakly similar to (148)CDC48_CAPAN	
EV102041	1.606	no similarity	
JCVI_18039	1.606	highly similar to (518)AT5G20950| Symbols: | glycosyl hydrolase family 3 protein | chr5:7107611-7110777 REVERSE no original descrip	
ES905497	1.605	moderately similar to (345)AT5G16370| Symbols: | AMP-binding protein, putative | chr5:5356826-5358484 REVERSEweakly similar to (	-1.763
JCVI_27916	1.605	weakly similar to (190)AT3G05480| Symbols: | cell cycle checkpoint control protein family | chr3:1585389-1588244 FORWARD no origi	
EV216069	1.605	weakly similar to (121)AT3G18260| Symbols: | reticulon family protein (RTNLB9) | chr3:6260334-6261510 REVERSE [21491]	
JCVI_10958	1.605	moderately similar to (286)AT2G37450| Symbols: | nodulin MtN21 family protein | chr2:15729907-15731930 REVERSE no original desc	
DN964528	1.605	weakly similar to (114)AT2G29310| Symbols: | tropinone reductase, putative / tropine dehydrogenase, putative | chr2:12597145-12598337	
JCVI_8336	1.604	moderately similar to (370)AT4G29130| Symbols: GIN2, HXK1, ATHXK1 | ATHXK1 (GLUCOSE INSENSITIVE 2); ATP binding / hex	
EV198800	1.604	moderately similar to (309)AT5G39000| Symbols: | protein kinase family protein | chr5:15629090-15631711 FORWARDvery weakly sim	
JCVI_31992	1.604	moderately similar to (219)AT1G65070| Symbols: | DNA mismatch repair MutS family protein | chr1:24176795-24179907 REVERSE no	
JCVI_21839	1.604	moderately similar to (379)AT2G45270| Symbols: | glycoprotease M22 family protein | chr2:18673657-18676406 FORWARD no original	
EV203310	1.604	moderately similar to (386)AT5G67360| Symbols: ARA12 | ARA12; subtilase | chr5:26889418-26891691 REVERSE [21490]	
EV103072	1.604	weakly similar to (167)AT2G24120| Symbols: PDE319, SCA3 | PDE319/SCA3 (SCABRA 3); DNA binding / DNA-directed RNA polymer	
JCVI_18411	1.604	moderately similar to (290)AT1G03140| Symbols: | splicing factor Prp18 family protein | chr1:754471-756223 REVERSE no original desc	
JCVI_38137	1.604	no original description	
JCVI_36741	1.604	moderately similar to (304)AT1G08750| Symbols: | GPI-anchor transamidase, putative | chr1:2801286-2804395 FORWARDvery weakly s	
JCVI_29692	1.604	moderately similar to (348)AT1G05680| Symbols: | UDP-glucoronosyl/UDP-glucosyl transferase family protein | chr1:1703195-1704638	
JCVI_30437	1.604	no original description	
JCVI_14047	1.603	moderately similar to (296)AT3G47520| Symbols: MDH | MDH (MALATE DEHYDROGENASE); malate dehydrogenase | chr3:1752464	
JCVI_15872	1.603	moderately similar to (316)AT3G49680| Symbols: ATBCAT-3, BCAT3 | ATBCAT-3/BCAT3 (BRANCHED-CHAIN AMINOTRANSFEI	
EV014415	1.603	no similarity	-1.572
JCVI_27268	1.603	moderately similar to (325)AT2G24260| Symbols: | basic helix-loop-helix (bHLH) family protein | chr2:10326726-10329257 REVERSE r	
JCVI_42494	1.603	no original description	
JCVI_40326	1.602	no original description	
JCVI_12131	1.602	no original description	
JCVI_5409	1.602	weakly similar to (178)AT5G11260| Symbols: TED 5, HY5 | HY5 (ELONGATED HYPOCOTYL 5); DNA binding / transcription factor |	
EX068173	1.602	moderately similar to (233)AT1G26230| Symbols: | chaperonin, putative | chr1:9072375-9075259 REVERSEweakly similar to (190)RUB	
JCVI_28154	1.602	weakly similar to (145)AT5G46070| Symbols: | GTP binding / GTPase | chr5:18700695-18705624 FORWARD no original description	
EV225132	1.602	moderately similar to (376)AT2G39200| Symbols: ATMLO12, MLO12 | MLO12 (MILDEW RESISTANCE LOCUS O 12); calmodulin bi	
EV102803	1.602	moderately similar to (226)AT4G12310| Symbols: CYP706A5 | CYP706A5 (cytochrome P450, family 706, subfamily A, polypeptide 5); c	
JCVI_17753	1.602	moderately similar to (276)AT4G02440| Symbols: EID1 | EID1 (EMPFINDLICHER IM DUNKELROTEN LICHT 1) | chr4:1072555-107	
EE462415	1.602	weakly similar to (110)AT1G62250| Symbols: | similar to unnamed protein product [Vitis vinifera] (GB:CAO21221.1) | chr1:22999280-2	
EE517946	1.602	no similarity	
ES933015	1.602	no similarity	
JCVI_41527	1.601	moderately similar to (244)AT5G48545| Symbols: | histidine triad family protein / HIT family protein | chr5:19693454-19695171 FORWA	
JCVI_12062	1.601	moderately similar to (253)AT4G28140| Symbols: | AP2 domain-containing transcription factor, putative | chr4:13974911-13975789 REV	
JCVI_745	1.601	moderately similar to (459)AT5G04590| Symbols: SIR | SIR (sulfite reductase); sulfite reductase (ferredoxin) | chr5:1319405-1322299 FO	
JCVI_2991	1.601	moderately similar to (447)AT2G13360| Symbols: AGT1, AGT | AGT (ALANINE:GLYOXYLATE AMINOTRANSFERASE) | chr2:5546	
JCVI_29614	1.601	moderately similar to (262)AT4G00231| Symbols: MEE50 | MEE50 (maternal effect embryo arrest 50); binding | chr4:97528-99050 FORV	
JCVI_22015	1.600	moderately similar to (405)AT3G14205| Symbols: | phosphoinositide phosphatase family protein | chr3:4716015-4720531 REVERSE no	
JCVI_18019	1.600	weakly similar to (155)AT5G44420| Symbols: PDF1.2a, LCR77, PDF1.2 | PDF1.2 (Low-molecular-weight cysteine-rich 77) | chr5:179244	
JCVI_30922	1.600	moderately similar to (437)AT3G20330| Symbols: | aspartate carabmoyltransferase, chloroplast / aspartate transcarbamylase / ATCase (P	
JCVI_13479	1.600	moderately similar to (450)AT2G31880| Symbols: | leucine-rich repeat transmembrane protein kinase, putative | chr2:13561997-13563922	
EE568131	1.600	no similarity	
JCVI_3300	1.600	moderately similar to (204)AT1G74730| Symbols: | similar to unknown [Populus trichocarpa x Populus deltoides] (GB:ABK96654.1); co	
DW999878	1.600	moderately similar to (333)AT4G28540| Symbols: CKL6, PAPK1 | CKL6/PAPK1 (Casein Kinase I-like 6); casein kinase I/ kinase | chr4:1	
EV159729	1.600	moderately similar to (280)AT2G44210| Symbols: | similar to unknown protein [Arabidopsis thaliana] (TAIR:AT1G55360.1); similar to u	
EV039906	1.600	moderately similar to (357)AT1G03920| Symbols: | protein kinase, putative | chr1:1001472-1004239 FORWARD [21442]	
JCVI_32263	1.600	weakly similar to (154)AT3G19000| Symbols: | oxidoreductase, 2OG-Fe(II) oxygenase family protein | chr3:6554010-6554993 REVERSE	
JCVI_37980	1.600	very weakly similar to (89.4)AT4G15770| Symbols: | 60S ribosome subunit biogenesis protein, putative | chr4:8978334-8978624 FORWA	
EV042778	1.600	weakly similar to (197)AT4G13690| Symbols: | similar to hypothetical protein MtrDRAFT_AC161864g11v2 [Medicago truncatula] (GB:	
JCVI_36615	1.600	moderately similar to (265)AT1G60460| Symbols: | similar to hypothetical protein OsI_023542 [Oryza sativa (indica cultivar-group)] (GB	
JCVI_210	1.599	moderately similar to (385)AT4G03280| Symbols: PGR1, PETC | PETC (PHOTOSYNTHETIC ELECTRON TRANSFER C) | chr4:1440	
EX021616	1.599	weakly similar to (200)AT3G02990| Symbols: HSFA1E, ATHSFA1E | ATHSFA1E (Arabidopsis thaliana heat shock transcription factor A	
EE429793	1.599	moderately similar to (294)AT4G12020| Symbols: ATWRKY19, MAPKKK11, WRKY19 | WRKY19 (WRKY DNA-binding protein 19) |	
ES958176	1.599	weakly similar to (159)AT1G75670| Symbols: | DNA-directed RNA polymerase/ RNA binding | chr1:28419039-28420166 REVERSE [21	
JCVI_12276	1.599	moderately similar to (325)AT2G37970| Symbols: SOUL-1 | SOUL-1; binding | chr2:15898105-15898782 FORWARD no original descrip	
JCVI_39335	1.599	moderately similar to (251)AT1G80350| Symbols: AAA1, LUE1, FRA2, ATKTN1, KTN1, FRC2, BOT1, FTR, ERH3 | ERH3 (ECTOPIC	1.474
CX190231	1.599	moderately similar to (447)AT3G02260| Symbols: DOC1, TIR3, UMB1, LPR1, ASA1, CRM1, BIG | BIG (DARK OVER-EXPRESSION	
JCVI_5590	1.598	weakly similar to (182)AT5G14670| Symbols: ATARFA1B | ATARFA1B (ADP-RIBOSYLATION FACTOR A1B); GTP binding / phospl	
EX018727	1.598	moderately similar to (285)AT4G35470| Symbols: | leucine-rich repeat family protein | chr4:16846536-16848453 FORWARD [21809]	
L38155	1.598	very weakly similar to (84.0)AT5G26360| Symbols: | chaperonin, putative | chr5:9255564-9258894 REVERSE [132]	
RC_DT317725	1.598	no similarity	
DY029556	1.598	moderately similar to (208)AT5G61120| Symbols: | zinc ion binding | chr5:24599712-24602256 REVERSE [18978]	
JCVI_2046	1.598	moderately similar to (369)AT1G19920| Symbols: ASA1, APS2 | APS2 (ATP SULFURYLASE PRECURSOR) | chr1:6914826-6916648 R	
JCVI_36076	1.598	moderately similar to (371)AT4G32640| Symbols: | sec23/sec24 transport protein-related | chr4:15742666-15750550 FORWARD no origi	
EV001540	1.597	weakly similar to (141)AT2G36910| Symbols: PGP1, ATMDR1 | ATPGP1 (ARABIDOPSIS THALIANA P GLYCOPROTEIN1); calmod	
JCVI_2910	1.597	moderately similar to (365)AT1G01800| Symbols: | short-chain dehydrogenase/reductase (SDR) family protein | chr1:293595-294888 FO	
EV193562	1.597	moderately similar to (252)AT4G13730| Symbols: | RabGAP/TBC domain-containing protein | chr4:7970295-7973897 FORWARD [2148	
RC_JCVI_37016	1.597	no original description	
EV048300	1.597	no similarity	
JCVI_24761	1.596	moderately similar to (347)AT5G26920| Symbols: | calmodulin binding | chr5:9475863-9478451 FORWARD no original description	
AM058214	1.596	moderately similar to (241)AT3G15300| Symbols: | VQ motif-containing protein | chr3:5147558-5148217 REVERSE [17712]	
CD836489	1.596	moderately similar to (268)AT5G51260| Symbols: | acid phosphatase, putative | chr5:20849448-20850692 REVERSEweakly similar to (1	
EV012816	1.596	weakly similar to (103)AT5G20660| Symbols: | 24 kDa vacuolar protein, putative | chr5:6986404-6990949 FORWARD [21450]	
EE528532	1.596	weakly similar to (102)AT4G25880| Symbols: APUM6 | APUM6 (ARABIDOPSIS PUMILIO 6); RNA binding | chr4:13155527-13159079	
JCVI_27000	1.596	moderately similar to (293)AT1G52155| Symbols: | similar to unnamed protein product [Vitis vinifera] (GB:CAO45853.1) | chr1:1942197	
CX272490	1.595	moderately similar to (336)AT3G49320| Symbols: | similar to unknown protein [Arabidopsis thaliana] (TAIR:AT5G41970.1); similar to u	

JCVI_18754	1.595	moderately similar to (498)AT1G18660| Symbols: | zinc finger (C3HC4-type RING finger) family protein | chr1:6421425-6425557 FORW	
CD830864	1.595	no similarity	
CV545555	1.595	weakly similar to (143)AT3G52060| Symbols: | similar to unknown protein [Arabidopsis thaliana] (TAIR:AT5G22070.1); similar to unnar	
EX045680	1.595	weakly similar to (120)AT2G30360| Symbols: SIP4, SNRK3.22, PKS5, CIPK11 | CIPK11 (SOS3-INTERACTING PROTEIN 4); kinase |	
JCVI_4988	1.595	moderately similar to (372)AT4G12320| Symbols: CYP706A6 | CYP706A6 (cytochrome P450, family 706, subfamily A, polypeptide 6); c	
JCVI_3450	1.595	moderately similar to (201)AT2G30980| Symbols: | shaggy-related protein kinase delta / ASK-delta / ASK-dzeta (ASK4) | chr2:13189427-	
EV124048	1.594	weakly similar to (128)AT4G35160| Symbols: | O-methyltransferase family 2 protein | chr4:16730994-16732813 REVERSE [21479] 1 60	
JCVI_1357	1.594	moderately similar to (238)AT5G59890| Symbols: ADF4 | ADF4 (ACTIN DEPOLYMERIZING FACTOR 4); actin binding | chr5:241403	
JCVI_17502	1.594	weakly similar to (133)AT3G06600| Symbols: | unknown protein | chr3:2059564-2060464 REVERSE no original description	
JCVI_40005	1.594	moderately similar to (292)AT4G13640| Symbols: UNE16 | UNE16 (unfertilized embryo sac 16) | chr4:7936860-7938493 REVERSE no o	
CD841236	1.594	weakly similar to (124)AT2G33220| Symbols: | similar to MEE4 (maternal effect embryo arrest 4) [Arabidopsis thaliana] (TAIR:AT1G04	
JCVI_3550	1.594	no original description	
JCVI_6004	1.594	moderately similar to (418)AT1G65220| Symbols: | eIF4-gamma/eIF5/eIF2-epsilon domain-containing protein | chr1:24229867-24232462	
JCVI_133	1.594	weakly similar to (107)AT2G30620| Symbols: | histone H1.2 | chr2:13052437-13053344 FORWARDvery weakly similar to (92.4)H1_PEA	
JCVI_3068	1.594	weakly similar to (196)AT3G27670| Symbols: RST1 | RST1 (RESURRECTION1); binding | chr3:10246575-10254395 FORWARD no ori	
JCVI_3992	1.593	moderately similar to (306)AT5G55950| Symbols: | transporter-related | chr5:22675637-22677479 REVERSE no original description	
JCVI_8591	1.593	moderately similar to (231)AT5G26730| Symbols: | similar to unknown protein [Arabidopsis thaliana] (TAIR:AT5G16920.1); similar to h	
EV111295	1.593	no similarity	
EV120642	1.593	weakly similar to (192)AT2G01600| Symbols: | epsin N-terminal homology (ENTH) domain-containing protein | chr2:268974-272355 FO	
ES930287	1.593	no similarity	
JCVI_8892	1.593	moderately similar to (261)AT2G15970| Symbols: WCOR413, WCOR413-LIKE, ATCOR413-PM1, FL3-5A3, COR413-PM1 | COR413-F	
RC_EE556705	1.593	no similarity	
JCVI_22701	1.593	moderately similar to (473)AT3G17630| Symbols: CHX19, ATCHX19 | ATCHX19 (CATION/H+ EXCHANGER 19); monovalent cation:	
JCVI_2585	1.592	moderately similar to (318)AT3G10640| Symbols: VPS60.1 | VPS60.1 | chr3:3323506-3324489 REVERSE no original description	
EE531784	1.592	moderately similar to (266)AT1G58470| Symbols: RBP1, XF41, ATRBP1 | ATRBP1 (ARABIDOPSIS THALIANA RNA-BINDING PRC	
EV029640	1.592	moderately similar to (256)AT5G18480| Symbols: PGSIP6 | PGSIP6 (PLANT GLYCOGENIN-LIKE STARCH INITIATION PROTEIN 6	
JCVI_8050	1.592	weakly similar to (140)AT1G76400| Symbols: | ribophorin I family protein | chr1:28663607-28666566 REVERSE no original description	
JCVI_4111	1.592	weakly similar to (187)AT3G01820| Symbols: | adenylate kinase family protein | chr3:293988-295364 REVERSEweakly similar to (104)I	
JCVI_2738	1.592	moderately similar to (394)AT3G05290| Symbols: | mitochondrial substrate carrier family protein | chr3:1506135-1507620 REVERSE no	-1.671
RC_EE567608	1.592	no similarity	-1.424
JCVI_38367	1.592	moderately similar to (467)AT5G14100| Symbols: ATNAP14 | ATNAP14 (Non-intrinsic ABC protein 14) | chr5:4549708-4551634 REVE	
JCVI_20132	1.592	highly similar to (573)AT3G19540| Symbols: | similar to unknown protein [Arabidopsis thaliana] (TAIR:AT1G49840.1); similar to unnam	
RC_EE559383	1.591	no similarity	
EV007064	1.591	weakly similar to (187)AT4G02530| Symbols: | chloroplast thylakoid lumen protein | chr4:1112335-1114005 REVERSE [21427]	
AM395501	1.591	no similarity	
JCVI_2906	1.591	moderately similar to (313)AT1G09760| Symbols: U2A' | U2A' (U2 small nuclear ribonucleoprotein A); protein binding | chr1:3159478-31	
L33574	1.591	no similarity	
JCVI_1454	1.590	moderately similar to (474)AT1G53240| Symbols: | malate dehydrogenase (NAD), mitochondrial | chr1:19858634-19860470 REVERSEm	
EX019070	1.590	weakly similar to (194)AT2G14740| Symbols: | vacuolar sorting receptor, putative | chr2:6315977-6319385 FORWARDweakly similar to	
JCVI_20096	1.590	moderately similar to (380)AT3G20820| Symbols: | leucine-rich repeat family protein | chr3:7280936-7282033 FORWARDweakly similar	
JCVI_19582	1.590	nearly identical (1167)AT5G65110| Symbols: ATACX2, ACX2 | ACX2 (ACYL-COA OXIDASE 2); acyl-CoA oxidase | chr5:26027286-26	
JCVI_33933	1.590	weakly similar to (156)AT3G61800| Symbols: | similar to unnamed protein product [Vitis vinifera] (GB:CAO14384.1); contains InterPro	
JCVI_10984	1.590	weakly similar to (138)AT2G43510| Symbols: ATTI1 | ATTI1 (ARABIDOPSIS THALIANA TRYPSIN INHIBITOR PROTEIN 1) | chr2:	
JCVI_30864	1.590	weakly similar to (156)AT5G55890| Symbols: | similar to unknown protein [Arabidopsis thaliana] (TAIR:AT5G55880.1); contains InterPr	
JCVI_14144	1.590	weakly similar to (102)AT3G25480| Symbols: | rhodanese-like domain-containing protein | chr3:9236828-9237835 REVERSE no original	
JCVI_9478	1.590	no original description	
JCVI_9581	1.590	weakly similar to (162)AT5G17710| Symbols: EMB1241 | EMB1241 (EMBRYO DEFECTIVE 1241); adenyl-nucleotide exchange factor/	
JCVI_25073	1.590	very weakly similar to (84.0)AT3G58140| Symbols: | phenylalanyl-tRNA synthetase class IIc family protein | chr3:21540965-21543363 RI	
EV007927	1.590	no similarity	
JCVI_18951	1.589	moderately similar to (312)AT3G14680| Symbols: CYP72A14 | CYP72A14 (cytochrome P450, family 72, subfamily A, polypeptide 14); c	
EX135068	1.589	moderately similar to (292)AT4G24340| Symbols: | phosphorylase family protein | chr4:12607485-12609158 FORWARDvery weakly sim	
EE484501	1.589	no similarity	
EE564839	1.589	very weakly similar to (81.3)AT1G07010| Symbols: | calcineurin-like phosphoesterase family protein | chr1:2152948-2154967 FORWARD	
JCVI_24464	1.589	moderately similar to (280)AT1G27060| Symbols: | regulator of chromosome condensation (RCC1) family protein | chr1:9395010-939666	
JCVI_13704	1.589	highly similar to (945)AT1G19715| Symbols: | jacalin lectin family protein | chr1:6816925-6819235 REVERSE no original description	-1.997
CX267260	1.589	weakly similar to (191)AT3G10490| Symbols: ANAC051, ANAC052 | ANAC051/ANAC052 (Arabidopsis NAC domain containing protein	
ES943556	1.589	moderately similar to (293)AT3G49310| Symbols: | similar to unknown protein [Arabidopsis thaliana] (TAIR:AT1G64650.2); similar to u	-1.379
EV036118	1.589	no similarity	
CD832485	1.588	weakly similar to (196)AT5G61690| Symbols: ATATH15 | ATATH15 (ABC2 homolog 15); ATPase, coupled to transmembrane movemer	
JCVI_11355	1.588	moderately similar to (233)AT5G62640| Symbols: ELF5 | ELF5 (EARLY FLOWERING 5) | chr5:25166810-25169577 REVERSE no orig	
EE536108	1.588	moderately similar to (201)AT4G34640| Symbols: ERG9, SQS1 | SQS1 (SQUALENE SYNTHASE 1); farnesyl-diphosphate farnesyltransf	
EE450748	1.588	moderately similar to (227)AT3G16270| Symbols: | binding | chr3:5513707-5516546 FORWARD [20170]	-1.869
JCVI_39010	1.588	no original description	
EE534429	1.588	moderately similar to (343)AT1G07180| Symbols: ATNDI1, NDA1 | ATNDI1/NDA1 (ALTERNATIVE NAD(P)H DEHYDROGENASE	
JCVI_34476	1.588	no original description	
EV067930	1.588	no similarity	
JCVI_41973	1.588	moderately similar to (252)AT1G15920| Symbols: | CCR4-NOT transcription complex protein, putative | chr1:5469956-5470816 FORWA	
JCVI_16721	1.588	highly similar to (722)AT5G04420| Symbols: | kelch repeat-containing protein | chr5:1246868-1249456 REVERSEvery weakly similar to	
JCVI_7766	1.587	moderately similar to (311)AT5G46290| Symbols: KAS I | KAS I (3-KETOACYL-ACYL CARRIER PROTEIN SYNTHASE I); fatty-acic	
EX029827	1.587	moderately similar to (224)AT5G14940| Symbols: | proton-dependent oligopeptide transport (POT) family protein | chr5:4831751-483431	
RC_EX051456	1.587	no similarity	
EE560503	1.587	no similarity	
RC_ES972789	1.587	no similarity	
JCVI_15455	1.586	highly similar to (530)AT5G15740| Symbols: | similar to unknown protein [Arabidopsis thaliana] (TAIR:AT3G02250.1); similar to hypotl	
JCVI_37289	1.586	no original description	
JCVI_586	1.586	moderately similar to (442)AT3G27430| Symbols: PBB1 | PBB1 (20S proteasome beta subunit B 1); peptidase | chr3:10154147-10156289	
JCVI_5646	1.586	highly similar to (665)AT3G22990| Symbols: | binding | chr3:8164022-8166152 FORWARD no original description	
AM385928	1.586	weakly similar to (103)AT5G26600| Symbols: | catalytic/ pyridoxal phosphate binding | chr5:9377458-9378885 FORWARD [20118]	
JCVI_26968	1.586	moderately similar to (366)AT3G44190| Symbols: | pyridine nucleotide-disulphide oxidoreductase family protein | chr3:15912991-159143	
JCVI_30508	1.586	moderately similar to (218)AT4G23890| Symbols: | similar to unnamed protein product [Vitis vinifera] (GB:CAO69542.1); similar to hyp	
EV075965	1.586	weakly similar to (140)AT3G13910| Symbols: | similar to unknown protein [Arabidopsis thaliana] (TAIR:AT2G19460.1); similar to unkno	

JCVI_32715	1.585	weakly similar to (149)AT2G46800| Symbols: ATMTP1, MTP1, ZAT1, ZAT | ATMTP1/MTP1/ZAT1 (ZINC TRANSPORTER OF ARA	
JCVI_17801	1.585	moderately similar to (224)AT1G08780| Symbols: ABI3 | ABI3 (ABI3-INTERACTING PROTEIN 3); unfolded protein binding | chr1:280	
JCVI_11558	1.585	moderately similar to (368)AT1G08980| Symbols: ATTOC64-I, AMI1, ATAMI1 | ATAMI1 (AMIDASE-LIKE PROTEIN 1); amidase | ch	
JCVI_4838	1.585	moderately similar to (423)AT5G56950| Symbols: NAP1;3, NFA3 | NAP1;3/NFA3 (NUCLEOSOME ASSEMBLY PROTEIN1;3); DNA b	
JCVI_8706	1.585	moderately similar to (372)AT1G67190| Symbols: | F-box family protein | chr1:25136717-25137976 FORWARD no original description	
EX065315	1.585	weakly similar to (176)AT5G62910| Symbols: | protein binding / zinc ion binding | chr5:25268056-25269241 FORWARD [21815]	
JCVI_16	1.585	highly similar to (508)AT4G05320| Symbols: UBQ10 | UBQ10 (POLYUBIQUITIN 10) | chr4:2718557-2719951 FORWARDweakly simil	
JCVI_32523	1.585	highly similar to (507)AT3G51160| Symbols: MUR_1, GMD2, MUR1 | MUR1 (MURUS 1) | chr3:19018211-19019332 REVERSE no orig	
ES908955	1.585	moderately similar to (398)AT3G20170| Symbols: | armadillo/beta-catenin repeat family protein | chr3:7041786-7043213 FORWARD [21	
JCVI_4824	1.585	moderately similar to (285)AT4G33580| Symbols: | carbonic anhydrase family protein / carbonate dehydratase family protein | chr4:1613	
EE446631	1.585	very weakly similar to (80.9)AT5G42310| Symbols: | pentatricopeptide (PPR) repeat-containing protein | chr5:16933088-16935466 FORW	
JCVI_7833	1.585	no original description	
JCVI_31366	1.584	moderately similar to (246)AT1G22450| Symbols: ATCOX6B2, COX6B | COX6B (CYTOCHROME C OXIDASE 6B); cytochrome-c oxi	
JCVI_34732	1.584	no original description	
JCVI_26835	1.584	moderately similar to (298)AT1G34220| Symbols: | similar to unknown protein [Arabidopsis thaliana] (TAIR:AT4G35730.1); similar to h	
JCVI_39829	1.584	weakly similar to (140)AT3G15115| Symbols: | similar to unknown protein [Arabidopsis thaliana] (TAIR:AT1G53180.1) | chr3:5086226-5	
AM395420	1.584	moderately similar to (212)AT5G60980| Symbols: | nuclear transport factor 2 (NTF2) family protein / RNA recognition motif (RRM)-con	-2.847
EV143510	1.584	no similarity	
CN736659	1.584	moderately similar to (242)AT2G43710| Symbols: FAB2, SSI2 | SSI2 (fatty acid biosynthesis 2); acyl-[acyl-carrier-protein] desaturase | ch	
EH424698	1.584	moderately similar to (344)AT1G10930| Symbols: ATSGS1, RECQL4A, ATRECQ4A | ATRECQ4A/ATSGS1/RECQL4A; ATP-depender	
JCVI_19395	1.584	weakly similar to (134)AT2G39960| Symbols: | microsomal signal peptidase 25 kDa subunit, putative (SPC25) | chr2:16688744-16690569	
EX094983	1.584	moderately similar to (345)AT4G30510| Symbols: AtATG18b | AtATG18b (Arabidopsis thaliana homolog of yeast autophagy 18 (ATG18	
EE471247	1.584	weakly similar to (103)AT4G26000| Symbols: PEP | PEP (PEPPER); nucleic acid binding | chr4:13197289-13199548 FORWARD [20163	
JCVI_7000	1.584	weakly similar to (196)AT5G60730| Symbols: | anion-transporting ATPase family protein | chr5:24440064-24442578 FORWARD no orig	
EH430410	1.583	weakly similar to (156)AT5G20730| Symbols: MSG1, IAA21, ARF7, TIR5, BIP, IAA23, IAA25, NPH4 | NPH4 (NON-PHOTOTROPHIC	
EX018572	1.583	moderately similar to (330)AT5G46340| Symbols: | O-acetyltransferase-related | chr5:18813588-18816952 REVERSE [21809]	
AY460110	1.583	weakly similar to (135)AT4G39260| Symbols: GR-RBP8, ATGRP8, CCR1 | ATGRP8/GR-RBP8 (COLD, CIRCADIAN RHYTHM, AND	
JCVI_26521	1.583	moderately similar to (282)AT3G53180| Symbols: | glutamate-ammonia ligase | chr3:19718046-19722166 FORWARD no original descrip	
EV202019	1.583	moderately similar to (303)AT4G18130| Symbols: PHYE | PHYE (PHYTOCHROME DEFECTIVE E); G-protein coupled photoreceptor/	-1.789
JCVI_22626	1.583	weakly similar to (135)AT4G31550| Symbols: ATWRKY11, WRKY11 | WRKY11 (WRKY DNA-binding protein 11); transcription factor	1.426
JCVI_781	1.583	moderately similar to (481)AT5G13440| Symbols: | ubiquinol-cytochrome C reductase iron-sulfur subunit, mitochondrial, putative / Riesk	
EV092795	1.583	weakly similar to (179)AT5G64040| Symbols: PSAN | PSAN (photosystem I reaction center subunit PSI-N); calmodulin binding | chr5:256	
EX096499	1.583	no similarity	
RC_ES945133	1.583	no similarity	
JCVI_3363	1.583	moderately similar to (358)AT5G01750| Symbols: | Identical to Uncharacterized protein At5g01750 [Arabidopsis Thaliana] (GB:Q9LZX1	
JCVI_15240	1.582	weakly similar to (105)AT1G04850| Symbols: | ubiquitin-associated (UBA)/TS-N domain-containing protein | chr1:1365310-1368705 RE	
ES996011	1.582	very weakly similar to (82.8)AT4G00560| Symbols: | methionine adenosyltransferase regulatory beta subunit-related | chr4:241135-242267	
EE471752	1.582	weakly similar to (169)AT1G62680| Symbols: | binding | chr1:23211912-23213558 REVERSEvery weakly similar to (86.3)RF1_ORYSA	
JCVI_32011	1.582	no original description	
EE432853	1.582	weakly similar to (141)AT3G15534| Symbols: | similar to unknown protein [Arabidopsis thaliana] (TAIR:AT1G52855.1); similar to unnar	
EV000942	1.582	no similarity	
EE568168	1.582	no similarity	
JCVI_40318	1.582	no original description	
EL590195	1.582	no similarity	
JCVI_20139	1.581	highly similar to (573)AT5G08260| Symbols: SCPL35 | SCPL35 (serine carboxypeptidase-like 35); serine carboxypeptidase | chr5:265723	
JCVI_4995	1.581	weakly similar to (160)AT3G25805| Symbols: | similar to unnamed protein product [Vitis vinifera] (GB:CAO42121.1) | chr3:9427591-942	
H07390	1.581	no similarity	
JCVI_39338	1.581	moderately similar to (399)AT1G61040| Symbols: VIP5 | VIP5 (VERNALIZATION INDEPENDENCE 5) | chr1:22487482-22489413 FO	2.209
EE470178	1.581	no similarity	-2.138
DT317692	1.581	no similarity	
ES981110	1.581	no similarity	
CD828569	1.580	moderately similar to (312)AT2G16050| Symbols: | DC1 domain-containing protein | chr2:6987532-6989021 FORWARD [13979]	
JCVI_2385	1.580	moderately similar to (308)AT1G47420| Symbols: | Identical to Uncharacterized protein At1g47420, mitochondrial precursor [Arabidopsi	-2.037
JCVI_3245	1.580	weakly similar to (184)AT4G25050| Symbols: ACP4 | ACP4 (ACYL CARRIER PROTEIN 4) | chr4:12870188-12871034 FORWARDwea	
JCVI_6941	1.580	moderately similar to (494)AT5G28530| Symbols: FRS10 | FRS10 (FAR1-RELATED SEQUENCE 10); zinc ion binding | chr5:10525082-	
JCVI_36937	1.580	moderately similar to (333)AT2G31890| Symbols: | similar to hypothetical protein [Vitis vinifera] (GB:CAN78554.1); similar to unnamed	
JCVI_10189	1.579	moderately similar to (406)AT1G29400| Symbols: AML5 | AML5 (ARABIDOPSIS MEI2-LIKE PROTEIN 5); RNA binding | chr1:10290	
JCVI_34053	1.579	highly similar to (506)AT2G40190| Symbols: | glycosyl transferase family 1 protein | chr2:16792310-16794335 FORWARD no original de	
CD826708	1.579	weakly similar to (103)AT5G43860| Symbols: ATCLH2 | ATCLH2 (Chlorophyll-chlorophyllido hydrolase 2) | chr5:17647719-17649411 F	
EE445575	1.579	moderately similar to (208)AT3G18500| Symbols: | similar to endonuclease/exonuclease/phosphatase family protein [Arabidopsis thaliana	
JCVI_24289	1.579	weakly similar to (117)AT2G02950| Symbols: PKS1 | PKS1 (PHYTOCHROME KINASE SUBSTRATE 1) | chr2:855148-856467 REVER	
JCVI_3413	1.579	moderately similar to (290)AT3G22840| Symbols: ELIP, ELIP1 | ELIP1 (EARLY LIGHT-INDUCABLE PROTEIN); chlorophyll binding	
EX016523	1.579	no similarity	-1.666
JCVI_15117	1.579	moderately similar to (409)AT2G03780| Symbols: | translin family protein | chr2:1152713-1154282 REVERSE no original description	
EE406691	1.579	weakly similar to (140)AT4G38940| Symbols: | kelch repeat-containing F-box family protein | chr4:18152842-18153954 FORWARD [16	
ES953672	1.579	very weakly similar to (94.4)AT2G20190| Symbols: ATCLASP, CLASP | ATCLASP/CLASP; binding | chr2:8718943-8725894 REVERSE	
ES984890	1.579	no similarity	
JCVI_13595	1.579	moderately similar to (207)AT1G03040| Symbols: | basic helix-loop-helix (bHLH) family protein | chr1:704279-706457 REVERSE no ori	
EE420122	1.578	moderately similar to (225)AT3G18580| Symbols: | single-strand-binding family protein | chr3:6396946-6398074 REVERSE [20149]	
EV089440	1.578	moderately similar to (206)AT5G61820| Symbols: | similar to MtN19-like protein [Pisum sativum] (GB:AAU14999.2); contains InterPro	
EE560866	1.578	no similarity	
JCVI_20607	1.578	weakly similar to (169)AT3G51270| Symbols: | ATP binding / protein serine/threonine kinase | chr3:19044830-19047751 FORWARD no	
JCVI_10769	1.578	moderately similar to (238)AT5G40190| Symbols: | calmodulin-binding protein | chr5:16086526-16087077 REVERSE no original descrip	-3.580
ES902341	1.578	highly similar to (539)AT5G19010| Symbols: MPK16 | MPK16 (mitogen-activated protein kinase 16); MAP kinase | chr5:6345098-634767	
EE505319	1.578	very weakly similar to (96.3)AT1G52580| Symbols: | rhomboid family protein | chr1:19591306-19592627 FORWARD [20139]	
ES928428	1.578	weakly similar to (126)AT2G33845| Symbols: | DNA-binding protein-related | chr2:14324857-14325788 FORWARD [15713] 15 595 595	
EV066632	1.578	no similarity	
JCVI_10287	1.578	moderately similar to (206)AT4G14870| Symbols: | P-P-bond-hydrolysis-driven protein transmembrane transporter | chr4:8517243-85177	
JCVI_13672	1.578	moderately similar to (430)AT5G08420| Symbols: | RNA binding | chr5:2713556-2716065 FORWARD no original description	
EV110321	1.578	no similarity	
EX048824	1.578	weakly similar to (101)AT5G10240| Symbols: ASN3 | ASN3 (ASPARAGINE SYNTHETASE 3); asparagine synthase (glutamine-hydroly	

JCVI_19522	1.577	moderately similar to (283)AT3G52610| Symbols: | similar to hypothetical protein [Vitis vinifera] (GB:CAN71367.1); contains domain C	
JCVI_16976	1.577	no original description	
JCVI_3880	1.577	moderately similar to (290)AT1G17860| Symbols: | trypsin and protease inhibitor family protein / Kunitz family protein | chr1:6149336-6	
JCVI_34067	1.577	moderately similar to (360)AT2G18915| Symbols: ADO2, LKP2 | LKP2 (LOV KELCH PROTEIN 2); ubiquitin-protein ligase | chr2:8201	
AM390031	1.577	weakly similar to (185)AT4G08460| Symbols: | protein binding / zinc ion binding | chr4:5377375-5378199 REVERSE [20118]	
JCVI_19480	1.577	moderately similar to (377)AT1G75660| Symbols: XRN3 | XRN3 (5'-3' exoribonuclease 3); 5'-3' exoribonuclease | chr1:28411950-284118	
EX043390	1.577	weakly similar to (154)AT3G17010| Symbols: | transcriptional factor B3 family protein | chr3:5800466-5802309 FORWARD [21811]	
CN737434	1.577	no similarity	
JCVI_19699	1.577	no original description	
EE472860	1.577	no similarity	
JCVI_9221	1.576	moderately similar to (280)AT1G75330| Symbols: OTC | OTC (ORNITHINE CARBAMOYLTRANSFERASE); amino acid binding / carb	
EX125192	1.576	weakly similar to (187)AT4G23240| Symbols: | protein kinase family protein | chr4:12160512-12161964 REVERSE [21830]	
EL591431	1.576	very weakly similar to (99.8)AT1G30240| Symbols: | binding | chr1:10641225-10645822 REVERSE [20863] 16 203 203	
EE529639	1.576	weakly similar to (179)AT2G32960| Symbols: | tyrosine specific protein phosphatase family protein | chr2:13995053-13997797 FORWAR	
JCVI_40708	1.575	moderately similar to (450)AT5G12980| Symbols: | rcd1-like cell differentiation protein, putative | chr5:4105565-4108142 REVERSE no	
JCVI_11941	1.575	moderately similar to (248)AT1G71030| Symbols: ATMYBL2 | ATMYBL2 (Arabidopsis myb-like 2); DNA binding / transcription factor	
EX127810	1.575	very weakly similar to (97.4)AT1G29951| Symbols: CPuORF35 | CPuORF35 (Conserved peptide upstream open reading frame 35) | chr1:1	
JCVI_2389	1.575	highly similar to (565)AT3G63000| Symbols: NPL41 | NPL41 (NPL4-LIKE PROTEIN 1) | chr3:23294811-23296332 FORWARDmoderat	
JCVI_33656	1.575	moderately similar to (382)AT1G78240| Symbols: QUA2, TSD2 | TSD2 (TUMOROUS SHOOT DEVELOPMENT 2); methyltransferase |	
EV048404	1.575	weakly similar to (152)AT4G36840| Symbols: | kelch repeat-containing protein | chr4:17352169-17352885 FORWARD [21442] 1 590 62	
JCVI_7039	1.575	moderately similar to (375)AT1G10940| Symbols: ASK1, SNRK2-4, SNRK2.4, SRK2A | ASK1 (ARABIDOPSIS SERINE/THREONINE	-1.827
JCVI_3963	1.575	weakly similar to (180)AT4G25230| Symbols: RIN2 | RIN2 (RPM1 INTERACTING PROTEIN 2); protein binding / zinc ion binding | chr	
EV183969	1.575	moderately similar to (202)AT1G67170| Symbols: | similar to unknown protein [Arabidopsis thaliana] (TAIR:AT3G14750.1); similar to u	
JCVI_42011	1.574	highly similar to (587)AT2G23450| Symbols: | protein kinase family protein | chr2:9996006-9998324 REVERSEmoderately similar to (2	
JCVI_30660	1.574	moderately similar to (453)AT3G22170| Symbols: FHY3 | FHY3 (FAR-RED ELONGATED HYPOCOTYLS 3) | chr3:7822366-7825421	
JCVI_30829	1.574	weakly similar to (200)AT5G11430| Symbols: | transcription elongation factor-related | chr5:3648470-3652257 FORWARD no original de	
EX043405	1.574	no similarity	
JCVI_4045	1.574	moderately similar to (333)AT4G21680| Symbols: | proton-dependent oligopeptide transport (POT) family protein | chr4:11517552-11519	
EX119807	1.574	moderately similar to (464)AT1G52520| Symbols: FRS6 | FRS6 (FAR1-related sequence 6); zinc ion binding | chr1:19569602-19571917 F	
JCVI_17260	1.574	moderately similar to (469)AT5G03290| Symbols: | isocitrate dehydrogenase, putative / NAD+ isocitrate dehydrogenase, putative | chr5:7	-2.836
JCVI_23091	1.574	weakly similar to (138)AT5G45400| Symbols: | replication protein, putative | chr5:18416217-18418871 FORWARD no original descriptio	
EV184047	1.574	weakly similar to (181)AT1G32700| Symbols: | zinc-binding family protein | chr1:11827966-11829424 FORWARD [21487]	
JCVI_21492	1.574	very weakly similar to (89.0)AT3G55890| Symbols: | yippee family protein | chr3:20751887-20752847 FORWARD no original description	
JCVI_7807	1.574	moderately similar to (299)AT5G25560| Symbols: | zinc finger (C3HC4-type RING finger) family protein | chr5:8899489-8901456 FORW	
JCVI_40081	1.574	no original description	
EE502449	1.574	no similarity	
JCVI_12383	1.574	highly similar to (810)AT3G12580| Symbols: HSP70 | HSP70 (heat shock protein 70); ATP binding | chr3:3991494-3993696 REVERSEhi	
EV226862	1.574	very weakly similar to (85.5)FAD3E_BRANA [21493]	
JCVI_28284	1.574	moderately similar to (431)AT1G11330| Symbols: | S-locus lectin protein kinase family protein | chr1:3810372-3813416 FORWARDweak	
EX055899	1.574	moderately similar to (239)AT4G37670| Symbols: | GCN5-related N-acetyltransferase (GNAT) family protein / amino acid kinase family	
JCVI_40132	1.573	moderately similar to (380)AT5G35200| Symbols: | epsin N-terminal homology (ENTH) domain-containing protein | chr5:13479693-1348	
CD826046	1.573	weakly similar to (131)AT3G06550| Symbols: | similar to O-acetyltransferase family protein [Arabidopsis thaliana] (TAIR:AT2G34410.2)	
EE472897	1.573	weakly similar to (102)AT2G37460| Symbols: | nodulin MtN21 family protein | chr2:15733745-15736088 REVERSE [20163]	
JCVI_199	1.573	moderately similar to (467)AT2G18730| Symbols: | diacylglycerol kinase, putative | chr2:8126058-8128771 FORWARD no original descr	
JCVI_31419	1.573	moderately similar to (377)AT4G03420| Symbols: | similar to unknown protein [Arabidopsis thaliana] (TAIR:AT1G03610.1); similar to u	
JCVI_4435	1.573	moderately similar to (292)AT1G06240| Symbols: | oxidoreductase/ transition metal ion binding | chr1:1911165-1912874 FORWARD no	
EX038860	1.573	no similarity	
EE558066	1.572	very weakly similar to (84.0)AT1G74430| Symbols: AtMYB95, AtMYBCP66, MYB95 | MYB95 (myb domain protein 95); DNA binding /	
EV123139	1.572	moderately similar to (407)AT5G13990| Symbols: ATEXO70C2 | ATEXO70C2 (exocyst subunit EXO70 family protein C2); protein bind	
EV108096	1.572	very weakly similar to (86.3)AT5G11170| Symbols: | DEAD/DEAH box helicase, putative (RH15) | chr5:3553335-3556647 FORWARD [	
EV084338	1.572	no similarity	
JCVI_13702	1.572	moderately similar to (399)AT2G43945| Symbols: | similar to unknown protein [Arabidopsis thaliana] (TAIR:AT3G59870.1); similar to h	
JCVI_8488	1.571	moderately similar to (394)AT3G14690| Symbols: CYP72A15 | CYP72A15 (cytochrome P450, family 72, subfamily A, polypeptide 15); c	
CD843747	1.571	weakly similar to (120)AT5G14670| Symbols: ATARFA1B | ATARFA1B (ADP-RIBOSYLATION FACTOR A1B); GTP binding / phospl	
JCVI_24302	1.571	moderately similar to (452)AT3G11080| Symbols: | disease resistance family protein | chr3:3470487-3473318 FORWARDweakly similar	
EV079325	1.570	moderately similar to (259)AT5G20885| Symbols: | zinc finger (C3HC4-type RING finger) family protein | chr5:7084135-7084665 REVE	
EE439231	1.570	no similarity	
DN961279	1.570	no similarity	
EX135764	1.570	moderately similar to (421)AT4G36710| Symbols: | scarecrow transcription factor family protein | chr4:17306064-17307524 FORWARD	
EV204320	1.570	moderately similar to (426)AT2G40460| Symbols: | proton-dependent oligopeptide transport (POT) family protein | chr2:16904201-16908	
EX044767	1.570	no similarity	
EE549692	1.570	no similarity	
JCVI_19813	1.570	moderately similar to (260)AT5G62270| Symbols: | similar to mucin-related [Arabidopsis thaliana] (TAIR:AT2G02880.1); similar to unna	
ES900439	1.570	moderately similar to (334)AT1G25540| Symbols: PFT1 | PFT1 (PHYTOCHROME AND FLOWERING TIME 1) | chr1:8969379-897328	
JCVI_11185	1.570	highly similar to (558)AT4G20830| Symbols: | FAD-binding domain-containing protein | chr4:11155497-11157119 FORWARD no origina	
EV108212	1.570	no similarity	
JCVI_35265	1.570	very weakly similar to (99.0)AT4G03175| Symbols: | protein kinase family protein | chr4:1402187-1402864 REVERSE no original descrip	
JCVI_6549	1.569	moderately similar to (457)AT1G55620| Symbols: ATCLC-F, CLC-F | CLC-F (CHLORIDE CHANNEL F); voltage-gated chloride channe	
EV012008	1.569	no similarity	
JCVI_13389	1.569	moderately similar to (401)AT3G12760| Symbols: | similar to unknown protein [Arabidopsis thaliana] (TAIR:AT1G15860.1); similar to S	
JCVI_28272	1.569	moderately similar to (250)AT3G47990| Symbols: | zinc finger (C3HC4-type RING finger) family protein | chr3:17724352-17727036 RE	
ES902498	1.569	weakly similar to (189)AT1G54220| Symbols: | dihydrolipoamide S-acetyltransferase, putative | chr1:20250127-20253875 REVERSE [21	
AM387356	1.569	weakly similar to (153)AT3G13680| Symbols: | F-box family protein | chr3:4477541-4478728 REVERSE [20118] 1 553 568	
AM395056	1.569	weakly similar to (106)AT2G32120| Symbols: HSP70T-2 | HSP70T-2; ATP binding | chr2:13658797-13660488 REVERSE [20346]	
EE426249	1.568	weakly similar to (135)AT5G66130| Symbols: RAD17, ATRAD17 | ATRAD17 (RADIATION SENSITIVE) | chr5:26451462-26454317 F	
AM391388	1.568	moderately similar to (312)AT3G50560| Symbols: | short-chain dehydrogenase/reductase (SDR) family protein | chr3:18772228-1877481	
JCVI_8498	1.568	moderately similar to (237)AT2G44310| Symbols: | calcium-binding EF hand family protein | chr2:18316361-18316789 FORWARD no o	
JCVI_24506	1.568	no original description	
EH415981	1.568	moderately similar to (337)AT1G76430| Symbols: | phosphate transporter family protein | chr1:28684009-28686768 REVERSE [20767]	
JCVI_13971	1.568	moderately similar to (312)AT3G27020| Symbols: YSL6 | YSL6 (YELLOW STRIPE LIKE 6); oligopeptide transporter | chr3:9962860-99	
JCVI_30377	1.568	moderately similar to (482)AT3G03060| Symbols: | ATPase | chr3:692195-695431 FORWARD no original description	

JCVI_40423	1.568	moderately similar to (417)AT1G32080| Symbols: | membrane protein, putative | chr1:11537552-11539736 REVERSE no original descrip	
ES914330	1.567	moderately similar to (260)AT5G10020| Symbols: | leucine-rich repeat transmembrane protein kinase, putative | chr5:3133515-3136950 F	
JCVI_7757	1.567	weakly similar to (188)AT5G60790| Symbols: ATGCN1 | ATGCN1 (Arabidopsis thaliana general control non-repressible 1) | chr5:244709	
JCVI_26038	1.567	no original description	
EE514048	1.567	no similarity	
EV132731	1.567	very weakly similar to (98.2)AT5G46180| Symbols: delta-OAT | delta-OAT (ornithine- delta-aminotransferase); ornithine-oxo-acid transam	
JCVI_13492	1.567	highly similar to (648)AT2G18170| Symbols: MPK7, ATMPK7 | ATMPK7 (MAP KINASE 7); MAP kinase/ kinase | chr2:7915260-79164	
JCVI_7934	1.567	weakly similar to (182)AT3G25870| Symbols: | similar to unknown protein [Arabidopsis thaliana] (TAIR:AT1G13360.1); similar to unkn	
JCVI_32432	1.567	moderately similar to (301)AT4G23180| Symbols: RLK4, CRK10 | CRK10 (CYSTEINE-RICH RLK10); kinase | chr4:12138182-1214079	
CD821185	1.567	moderately similar to (409)AT2G37980| Symbols: | similar to unknown protein [Arabidopsis thaliana] (TAIR:AT5G01100.1); similar to u	
EV151665	1.567	no similarity	
AM390091	1.566	moderately similar to (349)AT4G04180| Symbols: | AAA-type ATPase family protein | chr4:2020469-2023671 FORWARD [20118]	
JCVI_12853	1.566	weakly similar to (197)AT5G05960| Symbols: | protease inhibitor/seed storage/lipid transfer protein (LTP) family protein | chr5:1790257-	1.657
JCVI_1309	1.566	no original description	
JCVI_42182	1.566	no original description	
JCVI_10070	1.566	moderately similar to (326)AT4G35730| Symbols: | similar to unknown protein [Arabidopsis thaliana] (TAIR:AT1G34220.2); similar to u	
JCVI_327	1.566	moderately similar to (281)AT1G19350| Symbols: BZR2, BES1 | BES1 (BRI1-EMS-SUPPRESSOR 1) | chr1:6688832-6690156 FORWAI	
EV135235	1.566	no similarity	
JCVI_26123	1.566	highly similar to (605)AT1G08510| Symbols: FATB | FATB (FATTY ACYL-ACP THIOESTERASES B); acyl carrier/ acyl-ACP thioester	
EE415749	1.565	no similarity	
ES912635	1.565	no similarity	
JCVI_36696	1.565	moderately similar to (411)AT4G27600| Symbols: | pfkB-type carbohydrate kinase family protein | chr4:13782759-13785011 REVERSE r	
JCVI_32021	1.565	highly similar to (526)AT1G04680| Symbols: | pectate lyase family protein | chr1:1304051-1307779 REVERSEmoderately similar to (34(	
JCVI_14238	1.565	moderately similar to (273)AT5G19980| Symbols: | integral membrane family protein | chr5:6749909-6750934 REVERSE no original des	
JCVI_22258	1.565	moderately similar to (447)AT2G35630| Symbols: GEM1, MOR1 | MOR1 (MICROTUBULE ORGANIZATION 1) | chr2:14973907-1498	
JCVI_6372	1.565	weakly similar to (197)AT3G62650| Symbols: | binding | chr3:23179507-23179965 FORWARD no original description	
JCVI_6622	1.564	weakly similar to (122)AT1G04530| Symbols: | binding | chr1:1234455-1235894 REVERSE no original description	
EV195546	1.564	weakly similar to (136)AT1G70280| Symbols: | NHL repeat-containing protein | chr1:26469749-26472134 REVERSE [21490]	
JCVI_19605	1.564	moderately similar to (444)AT4G35335| Symbols: | nucleotide-sugar transporter family protein | chr4:16807291-16809809 FORWARD no	
AM391335	1.563	moderately similar to (265)AT2G35940| Symbols: EDA29, BLH1 | BLH1 (embryo sac development arrest 29) | chr2:15096250-15098778	
JCVI_9700	1.563	moderately similar to (388)AT4G09010| Symbols: APX4 | APX4 (ASCORBATE PEROXIDASE 4); peroxidase | chr4:5777499-5779335 I	
ES909790	1.563	weakly similar to (131)AT4G33240| Symbols: | similar to phosphatidylinositol-4-phosphate 5-kinase family protein [Arabidopsis thaliana]	
JCVI_28108	1.563	moderately similar to (380)AT2G39840| Symbols: TOPP4 | TOPP4 (Type one serine/threonine protein phosphatase 4); protein serine/thre	
JCVI_36764	1.563	moderately similar to (432)AT3G23940| Symbols: | dehydratase family | chr3:8648787-8652330 FORWARD no original description	
AT000462	1.563	no similarity	
JCVI_17213	1.562	very weakly similar to (92.8)AT1G43770| Symbols: | PHD finger family protein | chr1:16550949-16552697 FORWARD no original descri	
EE427826	1.562	weakly similar to (194)AT4G28310| Symbols: | similar to unknown protein [Arabidopsis thaliana] (TAIR:AT1G52270.1); similar to unnar	1.168
JCVI_28692	1.562	weakly similar to (181)AT5G51720| Symbols: | similar to Os07g0467200 [Oryza sativa (japonica cultivar-group)] (GB:NP_001059590.1)	
JCVI_32472	1.562	moderately similar to (362)AT2G30575| Symbols: GAUT5, LGT5 | GAUT5/LGT5 (Galacturonosyltransferase 5); polygalacturonate 4-alp	
JCVI_35024	1.562	moderately similar to (226)AT1G72290| Symbols: | trypsin and protease inhibitor family protein / Kunitz family protein | chr1:27219514-2	
JCVI_4856	1.562	moderately similar to (222)AT2G20330| Symbols: | transducin family protein / WD-40 repeat family protein | chr2:8779969-8782599 RE	
JCVI_23334	1.562	highly similar to (507)AT1G77610| Symbols: | glucose-6-phosphate/phosphate translocator-related | chr1:29170383-29172380 FORWARI	
ES919574	1.562	weakly similar to (198)AT5G60930| Symbols: | chromosome-associated kinesin, putative | chr5:24532624-24539737 REVERSE [15718]	
JCVI_10906	1.562	moderately similar to (415)AT5G22000| Symbols: RHF2A, CIC7E11 | CIC7E11; protein binding / zinc ion binding | chr5:7277439-727955	
JCVI_25030	1.562	moderately similar to (318)AT5G19900| Symbols: | PRLI-interacting factor, putative | chr5:6728563-6730047 REVERSE no original desc	
ES907807	1.562	highly similar to (513)AT1G12040| Symbols: LRX1 | LRX1 (LEUCINE-RICH REPEAT/EXTENSIN 1); protein binding / structural const	
ES266710	1.562	no similarity	
EV177598	1.562	weakly similar to (177)AT2G48030| Symbols: | endonuclease/exonuclease/phosphatase family protein | chr2:19654904-19657058 REVEF	
JCVI_26612	1.562	moderately similar to (421)AT1G61740| Symbols: | similar to unknown protein [Arabidopsis thaliana] (TAIR:AT1G11540.1); similar to u	2.009
JCVI_1686	1.562	moderately similar to (441)AT4G16690| Symbols: | esterase/lipase/thioesterase family protein | chr4:9392427-9393446 REVERSEweakly	
JCVI_22510	1.561	moderately similar to (404)AT4G26450| Symbols: WIP1 | WIP1 (WPP-DOMAIN INTERACTING PROTEIN 1); protein heterodimerizati	-2.327
EV178306	1.561	weakly similar to (114)AT4G38260| Symbols: | similar to unknown protein [Arabidopsis thaliana] (TAIR:AT1G20740.1); similar to unnar	
JCVI_18080	1.561	weakly similar to (154)AT1G10140| Symbols: | similar to unknown protein [Arabidopsis thaliana] (TAIR:AT1G58420.1); contains InterPı	1.587
JCVI_6799	1.561	weakly similar to (140)AT1G77122| Symbols: | similar to unknown protein [Arabidopsis thaliana] (TAIR:AT1G69210.1); similar to unnar	
EE414831	1.561	moderately similar to (204)AT2G25740| Symbols: | ATP-dependent protease La (LON) domain-containing protein | chr2:10987253-1099(	
JCVI_12595	1.561	moderately similar to (417)AT5G12850| Symbols: | zinc finger (CCCH-type) family protein | chr5:4057071-4059191 FORWARD no origi	
EV172122	1.561	weakly similar to (145)AT1G14870| Symbols: | Identical to Uncharacterized protein At1g14870 [Arabidopsis Thaliana] (GB:Q9LQU4); s	
JCVI_15154	1.561	moderately similar to (298)AT4G35260| Symbols: IDH1 | IDH1 (ISOCITRATE DEHYDROGENASE 1); isocitrate dehydrogenase (NAD-	
JCVI_36996	1.561	moderately similar to (211)AT3G09940| Symbols: MDHAR, ATMDAR3 | ATMDAR3/MDHAR (MONODEHYDROASCORBATE RED	
JCVI_14822	1.561	moderately similar to (371)AT3G26085| Symbols: | CAAX amino terminal protease family protein | chr3:9532079-9533634 FORWARD r	
JCVI_18408	1.560	moderately similar to (334)AT2G01600| Symbols: | epsin N-terminal homology (ENTH) domain-containing protein | chr2:268974-272355	
EX141856	1.560	moderately similar to (215)AT5G44260| Symbols: | zinc finger (CCCH-type) family protein | chr5:17847201-17848346 REVERSE [2183	
EX139582	1.560	very weakly similar to (81.6)AT2G45980| Symbols: | similar to unknown protein [Arabidopsis thaliana] (TAIR:AT4G00355.2); similar to u	
JCVI_32255	1.560	highly similar to (540)AT5G16300| Symbols: | similar to hypothetical protein OsI_003871 [Oryza sativa (indica cultivar-group)] (GB:EAY	
JCVI_1069	1.560	highly similar to (545)AT5G03240| Symbols: UBQ3 | UBQ3 (POLYUBIQUITIN 3); protein binding | chr5:771975-772895 REVERSEwea	
JCVI_5300	1.560	moderately similar to (326)AT4G34200| Symbols: EDA9 | EDA9 (embryo sac development arrest 9); NAD binding / amino acid binding /	
JCVI_7614	1.560	weakly similar to (200)AT4G02620| Symbols: | (VACUOLAR ATPASE SUBUNIT F); hydrogen ion transporting ATP synthase, rotationa	
JCVI_25586	1.559	no original description	
EE483632	1.559	weakly similar to (124)AT5G65740| Symbols: | protein binding / zinc ion binding | chr5:26318928-26320118 REVERSE [20174]	
EE471695	1.559	no similarity	
JCVI_25044	1.559	weakly similar to (157)AT5G20910| Symbols: | zinc finger (C3HC4-type RING finger) family protein | chr5:7092665-7094312 REVERSE	
JCVI_37774	1.559	weakly similar to (174)AT3G62080| Symbols: | SNF7 family protein | chr3:22997166-22999492 FORWARD no original description	
JCVI_42014	1.559	no original description	
EE563777	1.559	weakly similar to (194)AT1G14360| Symbols: ATUTR3, UTR3 | ATUTR3/UTR3 (UDP-GALACTOSE TRANSPORTER 3); pyrimidine r	
JCVI_3351	1.559	moderately similar to (306)AT4G37010| Symbols: | caltractin, putative / centrin, putative | chr4:17444309-17445374 FORWARDweakly s	1.527
RC_JCVI_23276	1.559	no original description	
EV100129	1.559	weakly similar to (116)AT2G13360| Symbols: AGT1, AGT | AGT (ALANINE:GLYOXYLATE AMINOTRANSFERASE) | chr2:5546499	-2.616
JCVI_8217	1.559	highly similar to (503)AT4G27585| Symbols: | band 7 family protein | chr4:13766990-13769838 REVERSE no original description	
JCVI_18491	1.559	very weakly similar to (88.2)AT2G02870| Symbols: | kelch repeat-containing F-box family protein | chr2:838377-839780 FORWARD no c	-3.012
JCVI_2476	1.558	moderately similar to (290)AT3G06035| Symbols: | Identical to Uncharacterized GPI-anchored protein At3g06035 precursor [Arabidopsis	
ES933030	1.558	no similarity	

EV010283	1.558	no similarity	
EV034305	1.558	no similarity	
EE477451	1.558	no similarity	
JCVI_14939	1.558	highly similar to (595)AT5G01230| Symbols: | FtsJ-like methyltransferase family protein | chr5:92788-95379 REVERSE no original descr	
JCVI_28387	1.558	weakly similar to (153)AT5G46780| Symbols: | VQ motif-containing protein | chr5:18996463-18997176 FORWARD no original descripti	
CX192389	1.557	no similarity	
JCVI_24906	1.557	moderately similar to (330)AT2G25760| Symbols: | protein kinase family protein | chr2:10992196-10995730 REVERSE no original descri	
EV062839	1.557	no similarity	
ES967594	1.557	no similarity	
JCVI_14593	1.557	moderately similar to (288)AT1G34350| Symbols: | similar to unnamed protein product [Vitis vinifera] (GB:CAO46981.1); contains doma	
JCVI_2222	1.557	very weakly similar to (81.3)AT3G15400| Symbols: ATA20 | ATA20 (Arabidopsis thaliana anther 20) | chr3:5202021-5202935 FORWARI	
CX194095	1.557	no similarity	
EE433349	1.557	very weakly similar to (86.7)AT3G24770| Symbols: CLE41 | CLE41 (CLAVATA3/ESR-RELATED 41); receptor binding | chr3:9046786-9	
RC_H07713	1.557	no similarity	
JCVI_31968	1.557	moderately similar to (476)AT4G34450| Symbols: | coatomer gamma-2 subunit, putative / gamma-2 coat protein, putative / gamma-2 COF	
ES941095	1.557	weakly similar to (133)AT1G67340| Symbols: | zinc finger (MYND type) family protein / F-box family protein | chr1:25233986-25235285	
ES976945	1.557	very weakly similar to (95.1)AT4G17730| Symbols: ATSYP23, SYP23 | SYP23 (syntaxin 23) | chr4:9865364-9866730 FORWARD [20129	
EE557214	1.557	no similarity	
EE427345	1.557	no similarity	
EV018907	1.557	very weakly similar to (86.7)AT5G45410| Symbols: | similar to unknown protein [Arabidopsis thaliana] (TAIR:AT4G25030.2); similar to u	
EE433698	1.557	moderately similar to (214)AT2G38940| Symbols: PHT1;4, ATPT2 | ATPT2 (PHOSPHATE TRANSPORTER 2); carbohydrate transmem	
EV169990	1.556	weakly similar to (193)AT1G80780| Symbols: | CCR4-NOT transcription complex protein, putative | chr1:30363409-30364233 FORWAR	
JCVI_42310	1.556	moderately similar to (273)AT2G17370| Symbols: HMGR2, HMG2 | HMG2 (3-HYDROXY-3-METHYLGLUTARYL-COA REDUCTAS	
JCVI_3855	1.556	moderately similar to (389)AT4G25650| Symbols: ACD1-LIKE | ACD1-LIKE; electron carrier | chr4:13081030-13083162 REVERSE no c	
ES902663	1.556	no similarity	
JCVI_4538	1.556	weakly similar to (182)AT5G60600| Symbols: ISPG, CSB3, CLB4, GcpE | GcpE (CHLOROPLAST BIOGENESIS 4) | chr5:24376673-243	
CO749810	1.556	no similarity	
JCVI_38560	1.556	moderately similar to (312)AT5G11980| Symbols: | conserved oligomeric Golgi complex component-related / COG complex component-r	
CD828545	1.555	moderately similar to (211)AT3G19490| Symbols: NHD1, ATNHD1 | ATNHD1 (Arabidopsis thaliana Na/H antiporter 1); sodium:hydroge	
JCVI_3223	1.555	highly similar to (543)AT4G12290| Symbols: | copper amine oxidase, putative | chr4:7304954-7306968 FORWARDmoderately similar to	
EV057708	1.555	weakly similar to (131)AT1G16870| Symbols: | mitochondrial 28S ribosomal protein S29-related | chr1:5770957-5773150 REVERSE [214	
JCVI_24436	1.555	highly similar to (738)AT1G26830| Symbols: CUL3A, ATCUL3A, ATCUL3, CUL3 | ATCUL3/ATCUL3A/CUL3/CUL3A (Cullin 3A); pr	
EV091377	1.555	weakly similar to (129)AT5G55710| Symbols: | similar to tic20 protein-related [Arabidopsis thaliana] (TAIR:AT2G47840.1); similar to ur	
JCVI_8533	1.555	highly similar to (504)AT1G22570| Symbols: | proton-dependent oligopeptide transport (POT) family protein | chr1:7976609-7978562 RE	
JCVI_12542	1.554	moderately similar to (315)AT5G42810| Symbols: ATIPK1 | ATIPK1 (Inositol-pentakisphosphate 2-kinase 1); inositol pentakisphosphate	
JCVI_33630	1.554	moderately similar to (426)AT5G56660| Symbols: ILL2 | ILL2 (IAA-leucine resistant (ILR)-like gene 2); metallopeptidase | chr5:2295050	
JCVI_8552	1.554	moderately similar to (348)AT1G20225| Symbols: | similar to unknown protein [Arabidopsis thaliana] (TAIR:AT1G76020.1); similar to u	-1.921
JCVI_20490	1.554	highly similar to (523)AT5G09400| Symbols: KUP7 | KUP7 (K+ uptake permease 7); potassium ion transmembrane transporter | chr5:291	
EX070154	1.554	weakly similar to (169)AT1G27360| Symbols: | squamosa promoter-binding protein-like 11 (SPL11) | chr1:9502126-9503702 FORWARD	
EV080703	1.554	moderately similar to (203)AT5G20080| Symbols: | NADH-cytochrome b5 reductase, putative | chr5:6782710-6786362 FORWARD [214	
JCVI_37281	1.554	highly similar to (538)AT4G11270| Symbols: | transducin family protein / WD-40 repeat family protein | chr4:6854457-6859486 FORWA	
JCVI_5683	1.554	moderately similar to (330)AT5G06360| Symbols: | ribosomal protein S8e family protein | chr5:1944836-1946513 FORWARD no origina	
EE411496	1.554	moderately similar to (272)AT3G04590| Symbols: | DNA-binding family protein | chr3:1239251-1241609 REVERSE [20140]	
EV009148	1.553	moderately similar to (204)AT1G16710| Symbols: HAC12 | HAC12 (HISTONE ACETYLTRANSFERASE OF THE CBP FAMILY 12); H	
EV038168	1.553	moderately similar to (310)AT4G11540| Symbols: | DC1 domain-containing protein | chr4:6990972-6992549 REVERSE [21441]	
EV097621	1.553	moderately similar to (211)AT1G19980| Symbols: | cytomatrix protein-related | chr1:6933153-6934634 FORWARD [21476]	
JCVI_11042	1.553	moderately similar to (217)AT5G06550| Symbols: | Identical to F-box protein At5g06550 [Arabidopsis Thaliana] (GB:Q67XX3;GB:Q9F(	-2.087
EV136082	1.553	no similarity	-1.055
JCVI_41276	1.553	weakly similar to (102)AT4G26190| Symbols: | similar to unknown protein [Arabidopsis thaliana] (TAIR:AT2G36550.1); similar to unnar	
DY009587	1.552	weakly similar to (153)AT5G19000| Symbols: ATBPM1 | ATBPM1 (BTB-POZ AND MATH DOMAIN 1); protein binding | chr5:634256	
JCVI_30007	1.552	weakly similar to (155)AT3G13670| Symbols: | protein kinase family protein | chr3:4469441-4473241 FORWARD no original description	
JCVI_9783	1.552	highly similar to (633)AT5G04360| Symbols: ATPU1, ATLDA | ATLDA/ATPU1 (PULLULANASE 1); alpha-amylase/ limit dextrinase | c	
EX083463	1.552	weakly similar to (156)AT2G29900| Symbols: | presenilin family protein | chr2:12756914-12758107 FORWARD [21819] 1 279 297	
JCVI_12563	1.552	no original description	
CD827790	1.552	weakly similar to (140)AT1G09270| Symbols: | importin alpha-1 subunit, putative (IMPA4) | chr1:2995164-2997835 FORWARDweakly s	
JCVI_15633	1.552	moderately similar to (491)AT1G21480| Symbols: | exostosin family protein | chr1:7519361-7521415 REVERSE no original description	
JCVI_3317	1.552	moderately similar to (363)AT5G45680| Symbols: | FK506-binding protein 1 (FKBP13) | chr5:18548121-18549355 FORWARDvery weal	
JCVI_31510	1.552	moderately similar to (280)AT1G66130| Symbols: | oxidoreductase N-terminal domain-containing protein | chr1:24618703-24620405 FOI	
JCVI_36443	1.551	moderately similar to (370)AT5G45110| Symbols: NPR3 | NPR3 (NPR1-LIKE PROTEIN 3); protein binding | chr5:18246546-18248561 F	
JCVI_6377	1.551	highly similar to (619)AT3G10150| Symbols: ATPAP16, PAP16 | ATPAP16/PAP16 (purple acid phosphatase 16); acid phosphatase/ prote	
CO749711	1.551	weakly similar to (162)AT2G33340| Symbols: | transducin family protein / WD-40 repeat family protein | chr2:14133779-14138076 REVI	
EV201316	1.551	moderately similar to (468)AT4G00900| Symbols: ATECA2, ECA2 | ECA2 (CALCIUM-TRANSPORTING ATPASE 2, ENDOPLASMIC	
JCVI_16624	1.551	moderately similar to (459)AT5G38520| Symbols: | hydrolase, alpha/beta fold family protein | chr5:15438835-15440464 FORWARD no c	
JCVI_4190	1.551	moderately similar to (412)AT3G62660| Symbols: GATL7 | GATL7 (Galacturonosyltransferase-like 7); polygalacturonate 4-alpha-galactu	
JCVI_18851	1.551	highly similar to (854)AT3G26170| Symbols: CYP71B19 | CYP71B19 (cytochrome P450, family 71, subfamily B, polypeptide 19); oxyge	
JCVI_1078	1.551	highly similar to (681)AT4G37870| Symbols: PCK1, PEPCK | PCK1/PEPCK (PHOSPHOENOLPYRUVATE CARBOXYKINASE 1); AT	
JCVI_30042	1.551	weakly similar to (111)AT2G43820| Symbols: GT, UGT74F2 | GT/UGT74F2 (UDP-GLUCOSYLTRANSFERASE 74F2); UDP-glucosyltr	
JCVI_5402	1.550	highly similar to (721)AT5G08170| Symbols: EMB1873, ATAIH | ATAIH/EMB1873 (AGMATINE IMINOHYDROLASE); agmatine dei	
JCVI_29680	1.550	moderately similar to (222)AT2G32530| Symbols: CSLB03, ATCSLB3, ATCSLB03 | ATCSLB03 (Cellulose synthase-like B3); transferas	
JCVI_33085	1.550	moderately similar to (317)AT3G14790| Symbols: RHM3 | RHM3 (RHAMNOSE BIOSYNTHESIS 3); catalytic | chr3:4964798-4966882	
JCVI_20554	1.550	weakly similar to (154)AT5G18280| Symbols: APY2, ATAPY2 | ATAPY2 (APYRASE 2) | chr5:6050801-6054025 REVERSE no original	
JCVI_31955	1.549	weakly similar to (101)AT3G44620| Symbols: | protein tyrosine phosphatase | chr3:16204845-16206409 FORWARD no original descripti	
JCVI_34300	1.549	moderately similar to (249)AT2G43260| Symbols: | F-box family protein / S locus-related | chr2:17990821-17992166 REVERSE no origir	
JCVI_40773	1.549	weakly similar to (149)AT5G35560| Symbols: | DENN (AEX-3) domain-containing protein | chr5:13759688-13764694 REVERSE no orig	1.052
JCVI_10548	1.549	moderately similar to (473)AT4G20430| Symbols: | subtilase family protein | chr4:11017667-11021116 REVERSE no original description	
EX021564	1.549	very weakly similar to (92.8)AT3G59990| Symbols: MAP2B | MAP2B (METHIONINE AMINOPEPTIDASE 2B); methionyl aminopeptid	
EE478899	1.549	no similarity	
EV186665	1.549	moderately similar to (285)AT2G22770| Symbols: NAI1 | NAI1; DNA binding / transcription factor | chr2:9691938-9693401 FORWARD	
JCVI_6589	1.549	weakly similar to (108)AT5G03495| Symbols: | nucleotide binding | chr5:873803-875024 REVERSE no original description	
JCVI_40114	1.549	moderately similar to (270)AT1G28580| Symbols: | GDSL-motif lipase, putative | chr1:10044589-10045860 REVERSEvery weakly simila	-1.286
EG021036	1.549	weakly similar to (194)AT3G01610| Symbols: EMB1354, CDC48C | CDC48C (EMBRYO DEFECTIVE 1354); ATPase | chr3:231794-23	

JCVI_23523	1.549	moderately similar to (229)AT3G24190| Symbols: | ABC1 family protein | chr3:8743326-8747710 FORWARD no original description	
JCVI_32752	1.549	moderately similar to (238)AT5G27280| Symbols: | zinc finger (DNL type) family protein | chr5:9617551-9618372 FORWARD no origina	
JCVI_8628	1.549	moderately similar to (415)AT3G53900| Symbols: | uracil phosphoribosyltransferase, putative / UMP pyrophosphorylase, putative / UPRT	
JCVI_8674	1.548	no original description	
CD843775	1.548	no similarity	1.280
CD843531	1.548	no similarity	
JCVI_16767	1.548	moderately similar to (493)AT2G39260| Symbols: | RNA binding | chr2:16399366-16406666 REVERSE no original description	
AM388983	1.548	weakly similar to (195)AT2G43400| Symbols: ETFQO | ETFQO (ELECTRON-TRANSFER FLAVOPROTEIN:UBIQUINONE OXIDORE	
JCVI_27003	1.548	weakly similar to (165)AT1G54530| Symbols: | calcium-binding EF hand family protein | chr1:20370172-20370555 FORWARD no origin	-1.304
BQ704171	1.548	weakly similar to (154)AT5G65670| Symbols: IAA9 | IAA9 (indoleacetic acid-induced protein 9); transcription factor | chr5:26271689-262	1.657
CV546905	1.547	no similarity	
JCVI_19205	1.547	weakly similar to (164)AT1G49710| Symbols: ATFUT12, FUCTB, FUCT2, FUT12 | FUT12 (fucosyltransferase 12); fucosyltransferase/ tr	
JCVI_20611	1.547	weakly similar to (161)AT1G55810| Symbols: | uracil phosphoribosyltransferase, putative / UMP pyrophosphorylase, putative / UPRTase,	
BQ790852	1.547	weakly similar to (125)AT4G17940| Symbols: | binding | chr4:9965799-9966790 FORWARD [8791]	
EV073144	1.547	moderately similar to (280)AT1G21010| Symbols: | similar to unknown protein [Arabidopsis thaliana] (TAIR:AT1G76600.1); similar to u	
EE534067	1.547	very weakly similar to (92.0)AT1G69610| Symbols: | structural constituent of ribosome | chr1:26190617-26193012 FORWARD [20150]	
EE485839	1.547	moderately similar to (209)AT2G39760| Symbols: ATBPM3 | ATBPM3; protein binding | chr2:16590291-16591893 FORWARD [20165]	
AM388325	1.547	weakly similar to (184)AT2G17110| Symbols: | similar to unknown protein [Arabidopsis thaliana] (TAIR:AT4G35240.1); similar to unnan	
JCVI_38961	1.547	moderately similar to (236)AT3G14560| Symbols: | unknown protein | chr3:4889375-4889839 FORWARD no original description	
JCVI_31622	1.547	moderately similar to (430)AT4G11380| Symbols: | beta-adaptin, putative | chr4:6920603-6925439 FORWARD no original description	
JCVI_11086	1.547	weakly similar to (129)AT5G14420| Symbols: RGLG2 | RGLG2 (RING DOMAIN LIGASE2) | chr5:4648358-4650566 REVERSE no orig	
EX042314	1.546	moderately similar to (214)AT5G11040| Symbols: | similar to hypothetical protein OsI_015984 [Oryza sativa (indica cultivar-group)] (GB	
JCVI_32590	1.546	moderately similar to (300)AT4G04450| Symbols: ATWRKY42, WRKY42 | WRKY42 (WRKY DNA-binding protein 42); transcription fa	
JCVI_9911	1.546	moderately similar to (345)AT1G44170| Symbols: ALDH4, ALDH3H1 | ALDH3H1 (ALDEHYDE DEHYDROGENASE 4) | chr1:167990	
JCVI_16121	1.546	moderately similar to (403)AT4G27750| Symbols: ISI1 | ISI1 (IMPAIRED SUCROSE INDUCTION 1); binding | chr4:13841714-1384350	-1.760
JCVI_39920	1.546	weakly similar to (137)AT5G44070| Symbols: ARA8, ATPCS1, PCS1, CAD1 | CAD1 (CADMIUM SENSITIVE 1) | chr5:17752103-1775	
H74463	1.546	no similarity	
JCVI_37766	1.546	no original description	
JCVI_24752	1.546	weakly similar to (151)AT1G68450| Symbols: | VQ motif-containing protein | chr1:25665384-25665842 REVERSE no original descriptio	
EV106902	1.546	weakly similar to (127)AT1G50020| Symbols: | similar to unnamed protein product [Vitis vinifera] (GB:CAO49863.1) | chr1:18523812-18	
EE450166	1.546	moderately similar to (283)AT1G04350| Symbols: | 2-oxoglutarate-dependent dioxygenase, putative | chr1:1165295-1166537 FORWARD	
JCVI_19481	1.546	moderately similar to (335)AT1G50510| Symbols: | indigoidine synthase A family protein | chr1:18720246-18722728 FORWARD no orig	
CX270955	1.546	no similarity	
JCVI_33339	1.545	moderately similar to (264)AT1G42970| Symbols: GAPB | GAPB (GLYCERALDEHYDE-3-PHOSPHATE DEHYDROGENASE B SUBU	
JCVI_1188	1.545	moderately similar to (459)AT3G48880| Symbols: | F-box family protein | chr3:18138858-18139993 FORWARD no original description	
ES945557	1.545	weakly similar to (191)AT1G57790| Symbols: | F-box family protein | chr1:21408243-21409301 REVERSE [21393]	
RC_L46475	1.545	no similarity	
JCVI_96	1.545	highly similar to (531)AT3G47340| Symbols: DIN6, AT-ASN1, ASN1 | ASN1 (DARK INDUCIBLE 6) | chr3:17449430-17452028 REVE	
JCVI_33434	1.545	highly similar to (535)AT1G11630| Symbols: | pentatricopeptide (PPR) repeat-containing protein | chr1:3913168-3914385 REVERSEwea	
EV065447	1.545	no similarity	-2.540
DY002509	1.544	very weakly similar to (91.3)AT1G11780| Symbols: | oxidoreductase, 2OG-Fe(II) oxygenase family protein | chr1:3977613-3979176 REV	1.581
EX100251	1.544	moderately similar to (471)AT5G47560| Symbols: ATTDT, ATSDAT | ATSDAT/ATTDT (TONOPLAST DICARBOXYLATE TRANSP	
JCVI_26368	1.544	moderately similar to (469)AT5G24530| Symbols: | oxidoreductase, 2OG-Fe(II) oxygenase family protein | chr5:8378967-8383157 FORW	
JCVI_10771	1.544	moderately similar to (451)AT5G45390| Symbols: NCLPP3, NCLPP4, CLPP4 | CLPP4 (Clp protease proteolytic subunit 4); endopeptidas	
JCVI_16012	1.544	moderately similar to (440)AT1G54340| Symbols: ICDH | ICDH; isocitrate dehydrogenase (NADP+) | chr1:20287187-20290173 FORWA	-2.009
JCVI_28533	1.544	moderately similar to (290)AT2G38025| Symbols: | similar to OTU-like cysteine protease family protein [Arabidopsis thaliana] (TAIR:AT	
EV008235	1.544	moderately similar to (267)AT1G77530| Symbols: | O-methyltransferase family 2 protein | chr1:29140931-29142317 FORWARDweakly s	
JCVI_1244	1.544	moderately similar to (215)AT1G09780| Symbols: | 2,3-biphosphoglycerate-independent phosphoglycerate mutase, putative / phosphoglyc	
EV121117	1.544	no similarity	
JCVI_9497	1.544	moderately similar to (432)AT1G51740| Symbols: ATUFE1, ATSYP81, SYP81 | SYP81 (SYNTAXIN 81); protein binding | chr1:1919273	
CD843668	1.544	no similarity	
JCVI_24312	1.544	moderately similar to (417)AT5G22400| Symbols: | rac GTPase activating protein, putative | chr5:7423053-7425338 REVERSE no origina	-1.445
EE475700	1.544	weakly similar to (142)AT2G27900| Symbols: | similar to predicted protein [Physcomitrella patens subsp. patens] (GB:EDQ50820.1); sim	
JCVI_21371	1.544	weakly similar to (147)AT5G17920| Symbols: ATMETS, ATMS1, ATCIMS | ATCIMS (COBALAMIN-INDEPENDENT METHIONINE	
JCVI_25112	1.543	moderately similar to (458)AT2G18960| Symbols: PMA, OST2, AHA1 | AHA1 (ARABIDOPSIS H+ ATPASE 1); ATPase | chr2:8228940	
EE420063	1.543	moderately similar to (247)AT5G11310| Symbols: | pentatricopeptide (PPR) repeat-containing protein | chr5:3606491-3608410 FORWAR	
JCVI_27284	1.543	moderately similar to (320)AT4G26000| Symbols: PEP | PEP (PEPPER); nucleic acid binding | chr4:13197289-13199548 FORWARD no	
EV056710	1.543	moderately similar to (270)AT3G05790| Symbols: | Lon protease, putative | chr3:1720160-1725188 REVERSEmoderately similar to (206	
EX091951	1.543	moderately similar to (285)AT4G24100| Symbols: | protein kinase family protein | chr4:12515233-12519346 FORWARD [21823]	
AM392094	1.543	moderately similar to (372)AT3G11960| Symbols: | cleavage and polyadenylation specificity factor (CPSF) A subunit C-terminal domain-	
JCVI_9831	1.543	highly similar to (799)AT4G37270| Symbols: HMA1 | HMA1 (Heavy metal ATPase 1); copper-exporting ATPase | chr4:17541981-175463	
JCVI_25455	1.543	highly similar to (709)AT3G06080| Symbols: | similar to unknown protein [Arabidopsis thaliana] (TAIR:AT5G19160.1); similar to unnam	
JCVI_4128	1.543	moderately similar to (273)AT4G09800| Symbols: RPS18C | RPS18C (S18 RIBOSOMAL PROTEIN); structural constituent of ribosome |	-1.225
EV182056	1.543	moderately similar to (348)AT5G55180| Symbols: | glycosyl hydrolase family 17 protein | chr5:22406060-22407776 FORWARDweakly s	
JCVI_16234	1.543	moderately similar to (471)AT1G80670| Symbols: | transducin family protein / WD-40 repeat family protein | chr1:30325701-30328435 R	-1.749
CD813650	1.542	moderately similar to (387)AT1G79820| Symbols: SGB1 | SGB1; carbohydrate transmembrane transporter/ sugar:hydrogen ion symporter	
JCVI_26231	1.542	moderately similar to (444)AT5G55990| Symbols: ATCBL2, CBL2 | CBL2 (calcineurin B-like protein 2); calcium ion binding | chr5:22689	
JCVI_26406	1.542	highly similar to (529)AT3G50530| Symbols: CRK | CRK (CDPK-RELATED KINASE); calcium ion binding / calcium-dependent protein	
JCVI_23239	1.542	weakly similar to (129)AT1G61790| Symbols: | OST3/OST6 family protein | chr1:22818055-22819095 FORWARD no original descriptio	
JCVI_24566	1.542	moderately similar to (276)AT3G08040| Symbols: MAN1, FRD3 | FRD3 (FERRIC REDUCTASE DEFECTIVE 3); antiporter | chr3:25665	
JCVI_7637	1.542	weakly similar to (147)AT3G25950| Symbols: | similar to DNA-binding storekeeper protein-related [Arabidopsis thaliana] (TAIR:AT5G14	-1.491
EV138589	1.542	weakly similar to (158)AT3G48000| Symbols: ALDH2, ALDH2B4 | ALDH2B4 (ALDEHYDE DEHYDROGENASE 2); 3-chloroallyl alde	
DY016446	1.541	weakly similar to (184)AT3G59350| Symbols: | serine/threonine protein kinase, putative | chr3:21943907-21945860 FORWARD [18975]	
JCVI_25737	1.541	moderately similar to (365)AT2G17650| Symbols: | AMP-dependent synthetase and ligase family protein | chr2:7678123-7680018 FORW	
JCVI_29455	1.541	moderately similar to (386)AT1G50920| Symbols: | GTP-binding protein-related | chr1:18874223-18876238 FORWARD no original descr	
JCVI_11993	1.541	weakly similar to (117)AT1G73930| Symbols: | similar to unnamed protein product [Vitis vinifera] (GB:CAO68016.1); similar to hypothe	-1.812
ES966585	1.541	no similarity	
JCVI_34501	1.540	weakly similar to (150)AT1G15120| Symbols: | ubiquinol-cytochrome C reductase complex 7.8 kDa protein, putative / mitochondrial hing	-2.390
JCVI_3487	1.540	highly similar to (832)AT2G46950| Symbols: CYP709B2 | CYP709B2 (cytochrome P450, family 709, subfamily B, polypeptide 2); oxyge	
ES968222	1.540	no similarity	
ES909852	1.540	very weakly similar to (98.2)AT2G43550| Symbols: | trypsin inhibitor, putative | chr2:18080340-18080728 FORWARD [21430] 1 491 509	

JCVI_7496	1.540	moderately similar to (220)AT3G06190| Symbols: ATBPM2 | ATBPM2; protein binding | chr3:1874583-1876581 REVERSE no original d	-2.232
EH418894	1.540	no similarity	
JCVI_14461	1.540	moderately similar to (218)AT1G73760| Symbols: | zinc finger (C3HC4-type RING finger) family protein | chr1:27743027-27744822 REV	
EX128451	1.540	moderately similar to (211)AT1G64840| Symbols: | F-box family protein | chr1:24097673-24098827 FORWARD [21832]	
EV061744	1.540	no similarity	
EV131611	1.540	no similarity	
EX132209	1.540	moderately similar to (226)AT4G39940| Symbols: AKN2 | AKN2 (APS-KINASE 2); ATP binding / kinase/ transferase, transferring phosp	
EX047663	1.539	weakly similar to (129)AT5G23575| Symbols: | transmembrane protein, putative | chr5:7946566-7950044 FORWARD [21812]	
JCVI_8	1.539	highly similar to (523)AT2G45960| Symbols: TMP-A, ATHH2, PIP1;2, PIP1B | PIP1B (plasma membrane intrinsic protein 1;2) | chr2:189	
JCVI_30937	1.539	weakly similar to (137)AT5G39080| Symbols: | transferase family protein | chr5:15658909-15660300 FORWARD no original description	
JCVI_2315	1.539	moderately similar to (263)AT3G09300| Symbols: | oxysterol-binding family protein | chr3:2858074-2860468 FORWARD no original des	-1.630
JCVI_24859	1.539	highly similar to (574)AT1G11870| Symbols: OVA7, SRS, AtSRS | AtSRS (OVULE ABORTION 7); serine-tRNA ligase | chr1:4003894-4	
DT317716	1.539	no similarity	
ES967429	1.539	no similarity	
JCVI_42326	1.539	moderately similar to (279)AT3G15358| Symbols: | similar to unknown protein [Arabidopsis thaliana] (TAIR:AT1G53035.1); similar to u	
JCVI_26923	1.539	moderately similar to (465)AT4G18750| Symbols: | pentatricopeptide (PPR) repeat-containing protein | chr4:10304861-10307476 FORWA	
JCVI_7251	1.538	highly similar to (523)AT2G46370| Symbols: JAR, FIN219, JAR1 | JAR1 (JASMONATE RESISTANT 1) | chr2:19041652-19043442 FOR	
JCVI_14595	1.538	moderately similar to (471)AT5G65165| Symbols: SDH2-3 | SDH2-3 (Succinate dehydrogenase 2-3) | chr5:26051741-26053148 REVERS	
JCVI_18107	1.538	moderately similar to (415)AT2G34640| Symbols: PTAC12 | PTAC12 (PLASTID TRANSCRIPTIONALLY ACTIVE12) | chr2:14589140	1.524
EX088798	1.538	no similarity	
JCVI_26066	1.538	weakly similar to (102)AT5G18360| Symbols: | disease resistance protein (TIR-NBS-LRR class), putative | chr5:6080051-6083029 REVE	
JCVI_5930	1.538	moderately similar to (387)AT4G01310| Symbols: | ribosomal protein L5 family protein | chr4:544166-545480 REVERSEmoderately simi	
JCVI_6573	1.538	highly similar to (602)AT5G19290| Symbols: | esterase/lipase/thioesterase family protein | chr5:6494115-6495107 FORWARD no origina	
JCVI_33229	1.537	moderately similar to (389)AT3G57570| Symbols: | binding | chr3:21323575-21331082 FORWARD no original description	
JCVI_41845	1.537	no original description	
JCVI_27295	1.537	weakly similar to (157)AT3G44280| Symbols: | similar to unknown protein [Arabidopsis thaliana] (TAIR:AT5G22280.1); similar to unnar	
EX061058	1.537	moderately similar to (238)AT4G00450| Symbols: CRP | CRP (CRYPTIC PRECOCIOUS) | chr4:203471-210666 FORWARD [21813]	
JCVI_5596	1.537	moderately similar to (271)AT5G61210| Symbols: ATSNAP33, SNP33, ATSNAP33B, SNAP33 | SNAP33 (synaptosomal-associated prote	
EX085383	1.537	moderately similar to (374)AT4G23180| Symbols: RLK4, CRK10 | CRK10 (CYSTEINE-RICH RLK10); kinase | chr4:12138182-1214079	
JCVI_8738	1.537	moderately similar to (295)AT5G16270| Symbols: ATRAD21.3, SYN4 | ATRAD21.3/SYN4 (ARABIDOPSIS HOMOLOG OF RAD21 3)	
JCVI_30034	1.537	weakly similar to (116)AT4G17570| Symbols: | zinc finger (GATA type) family protein | chr4:9784342-9786987 REVERSE no original de	
CD843683	1.537	moderately similar to (237)AT5G16130| Symbols: | 40S ribosomal protein S7 (RPS7C) | chr5:5268987-5269915 FORWARDmoderately s	
EV169095	1.537	no similarity	
JCVI_5627	1.537	moderately similar to (343)AT2G15430| Symbols: RPB35.5A, RBP36A | RBP36A (RNA polymerase II 36 kDa polypeptide A); DNA bind	
ES949517	1.537	no similarity	
JCVI_24964	1.536	moderately similar to (239)AT3G27930| Symbols: | similar to unnamed protein product [Vitis vinifera] (GB:CAO14767.1) | chr3:1037083	1.190
JCVI_39951	1.536	weakly similar to (124)AT4G16380| Symbols: | metal ion binding | chr4:9254659-9255977 FORWARD no original description	
EX101493	1.536	moderately similar to (352)AT2G01600| Symbols: | epsin N-terminal homology (ENTH) domain-containing protein | chr2:268974-272355	
JCVI_31058	1.536	weakly similar to (181)AT2G24590| Symbols: | splicing factor, putative | chr2:10456916-10457939 FORWARD no original description	
JCVI_11290	1.536	moderately similar to (260)AT2G03220| Symbols: MUR2, ATFUT1, ATFT1, FT1 | FT1 (FUCOSYLTRANSFERASE 1); fucosyltransfera	
JCVI_9683	1.536	weakly similar to (139)AT1G31812| Symbols: ACBP | ACBP (ACYL-COA-BINDING PROTEIN); acyl-CoA binding | chr1:11411113-114	
JCVI_25928	1.536	weakly similar to (120)AT2G30350| Symbols: | endo/excinuclease amino terminal domain-containing protein | chr2:12941314-12942819 I	
CD814247	1.536	weakly similar to (106)AT1G51460| Symbols: | ABC transporter family protein | chr1:19080800-19085003 REVERSE [13977]	1.131
JCVI_907	1.536	moderately similar to (265)AT3G11400| Symbols: ATEIF3G1, EIF3G1 | EIF3G1 (eukaryotic translation initiation factor 3G1); RNA bindi	
EV130342	1.535	weakly similar to (171)AT4G23880| Symbols: | unknown protein | chr4:12415799-12416398 REVERSE [21481]	
EX085756	1.535	moderately similar to (407)AT4G12830| Symbols: | hydrolase, alpha/beta fold family protein | chr4:7531186-7533324 FORWARD [21823	
JCVI_17258	1.535	moderately similar to (486)AT5G58870| Symbols: FTSH9 | FTSH9 (FtsH protease 9); ATP-dependent peptidase/ ATPase/ metallopeptidas	
EV210685	1.535	moderately similar to (258)AT4G19500| Symbols: | disease resistance protein (TIR-NBS-LRR class), putative | chr4:10625798-10630150	
JCVI_14007	1.535	moderately similar to (407)AT1G79080| Symbols: | pentatricopeptide (PPR) repeat-containing protein | chr1:29751995-29753725 REVER	
JCVI_9573	1.535	highly similar to (823)AT5G41770| Symbols: | crooked neck protein, putative / cell cycle protein, putative | chr5:16735249-16738164 FOI	
JCVI_18833	1.535	highly similar to (841)AT5G64580| Symbols: | AAA-type ATPase family protein | chr5:25834617-25838691 REVERSEweakly similar to	
JCVI_7270	1.535	moderately similar to (238)AT1G58290| Symbols: HEMA1 | HEMA1; glutamyl-tRNA reductase | chr1:21627693-21629716 REVERSEmo	1.780
JCVI_22961	1.535	moderately similar to (311)AT5G16370| Symbols: | AMP-binding protein, putative | chr5:5356826-5358484 REVERSEweakly similar to (	
DY024944	1.535	weakly similar to (101)AT3G01050| Symbols: MUB1 | MUB1 (MEMBRANE-ANCHORED UBIQUITIN-FOLD PROTEIN 1 PRECURSO	
DT317661	1.534	no similarity	
EX015735	1.534	no similarity	
EV041892	1.534	no similarity	
EV212430	1.534	weakly similar to (131)AT2G44710| Symbols: | RNA recognition motif (RRM)-containing protein | chr2:18439977-18443704 FORWARD	
EE553742	1.534	no similarity	
JCVI_6998	1.534	moderately similar to (243)AT2G45740| Symbols: PEX11D | PEX11D | chr2:18846939-18848176 FORWARD no original description	
EX100072	1.534	moderately similar to (250)AT2G24640| Symbols: | ubiquitin carboxyl-terminal hydrolase family protein / zinc finger (MYND type) famil	
EX067021	1.534	weakly similar to (197)AT1G53190| Symbols: | zinc finger (C3HC4-type RING finger) family protein | chr1:19841740-19843671 FORWA	
JCVI_25733	1.534	moderately similar to (289)AT5G20680| Symbols: | similar to unknown protein [Arabidopsis thaliana] (TAIR:AT5G64020.1); similar to C	
EE513550	1.534	weakly similar to (166)AT1G25460| Symbols: | oxidoreductase family protein | chr1:8942798-8944231 FORWARD [15713]	
JCVI_38036	1.534	moderately similar to (348)AT4G22300| Symbols: SOBER1 | SOBER1 (SUPPRESSOR OF AVRBST-ELICITED RESISTANCE 1); carbo	
JCVI_41164	1.534	moderately similar to (271)AT4G39370| Symbols: UBP27 | UBP27 (UBIQUITIN-SPECIFIC PROTEASE 27); ubiquitin-specific protease	
JCVI_33411	1.534	moderately similar to (379)AT2G03550| Symbols: | hydrolase | chr2:1077077-1078015 FORWARDweakly similar to (115)GID1_ORYSA	-2.068
EX053269	1.534	weakly similar to (169)AT5G61020| Symbols: ECT3 | ECT3 (evolutionary conserved C-terminal 3) | chr5:24574711-24577006 REVERSE	
EX066580	1.534	weakly similar to (151)AT2G42460| Symbols: | meprin and TRAF homology domain-containing protein / MATH domain-containing prote	
JCVI_9964	1.533	moderately similar to (368)AT1G69870| Symbols: | proton-dependent oligopeptide transport (POT) family protein | chr1:26319871-26323	
JCVI_13215	1.533	moderately similar to (360)AT4G17890| Symbols: AGD8 | AGD8 (ARF-GAP DOMAIN 8); DNA binding | chr4:9937134-9939001 FORW	
JCVI_15363	1.533	weakly similar to (157)AT4G25210| Symbols: | transcription regulator | chr4:12918456-12919562 FORWARD no original description	
EV201295	1.533	moderately similar to (438)AT4G02750| Symbols: | pentatricopeptide (PPR) repeat-containing protein | chr4:1221116-1223461 REVERSI	
EH414504	1.533	weakly similar to (152)AT1G18580| Symbols: GAUT11 | GAUT11 (Galacturonosyltransferase 11); polygalacturonate 4-alpha-galacturono	
EX040463	1.533	no similarity	
JCVI_30507	1.533	moderately similar to (316)AT1G03000| Symbols: PEX6 | PEX6 (PEROXIN6); ATPase | chr1:688057-692453 REVERSE no original desc	
CX281075	1.533	moderately similar to (281)AT1G78290| Symbols: | serine/threonine protein kinase, putative | chr1:29462350-29463802 REVERSEmoder	
JCVI_40690	1.532	no original description	
JCVI_31639	1.532	no original description	
JCVI_16791	1.532	nearly identical (1007)AT4G32250| Symbols: | protein kinase family protein | chr4:15570291-15572534 REVERSE no original description	-1.802
JCVI_38220	1.532	weakly similar to (191)AT1G69800| Symbols: | CBS domain-containing protein | chr1:26278079-26279988 REVERSE no original descrip	-1.979

JCVI_24911	1.532	no original description	
JCVI_22615	1.532	weakly similar to (135)AT5G58040| Symbols: ATFIP1[V] | ATFIP1[V] (ARABIDOPSIS HOMOLOG OF YEAST FIP1 [V]); RNA bindi	
EV000599	1.532	no similarity	
JCVI_7174	1.532	moderately similar to (481)AT4G34890| Symbols: ATXDH1 | ATXDH1 (XANTHINE DEHYDROGENASE 1); xanthine dehydrogenase |	
EX056817	1.532	moderately similar to (270)AT5G14950| Symbols: GMII, ATGMII | ATGMII/GMII (GOLGI ALPHA-MANNOSIDASE II); alpha-manno	
JCVI_4177	1.531	highly similar to (568)AT1G75900| Symbols: | family II extracellular lipase 3 (EXL3) | chr1:28502840-28504604 FORWARDmoderately	
JCVI_2523	1.531	highly similar to (532)AT3G03520| Symbols: | phosphoesterase family protein | chr3:837979-840518 REVERSE no original description	
JCVI_23614	1.531	moderately similar to (360)AT4G39960| Symbols: | DNAJ heat shock family protein | chr4:18534188-18536314 FORWARD no original d	
ES954103	1.531	no similarity	
JCVI_20335	1.531	moderately similar to (331)AT5G54250| Symbols: CNGC4, HLM1, DND2, ATCNGC4 | ATCNGC4 (DEFENSE, NO DEATH 2); calmodu	
JCVI_23903	1.531	moderately similar to (247)AT4G02890| Symbols: UBQ14 | UBQ14 (ubiquitin 14) | chr4:1278747-1279664 REVERSEweakly similar to (	
EX123440	1.531	no similarity	
EE473992	1.531	moderately similar to (219)AT1G24070| Symbols: CSLA10, ATCSLA10 | ATCSLA10 (Cellulose synthase-like A10); transferase, transfer	
AM391549	1.531	weakly similar to (120)AT5G57880| Symbols: | similar to unnamed protein product [Vitis vinifera] (GB:CAO66652.1) | chr5:23461203-23	
JCVI_34170	1.531	moderately similar to (282)AT3G28100| Symbols: | nodulin MtN21 family protein | chr3:10457388-10462050 FORWARD no original des	
ES955420	1.531	moderately similar to (277)AT3G02660| Symbols: EMB2768 | EMB2768 (EMBRYO DEFECTIVE 2768); ATP binding / aminoacyl-tRNA	
EV080390	1.530	moderately similar to (260)AT3G28740| Symbols: CYP81D1 | cytochrome P450 family protein | chr3:10790001-10791789 REVERSEvery	
ES921669	1.530	weakly similar to (177)AT1G61980| Symbols: | mitochondrial transcription termination factor-related / mTERF-related | chr1:22911827-2	
EV094356	1.530	moderately similar to (351)AT4G01010| Symbols: CNGC13, ATCNGC13 | ATCNGC13 (cyclic nucleotide gated channel 13); calmodulin	1.476
EE568801	1.530	no similarity	
JCVI_31931	1.530	moderately similar to (213)AT2G38420| Symbols: | pentatricopeptide (PPR) repeat-containing protein | chr2:16098198-16099532 FORW	
JCVI_6275	1.530	highly similar to (566)AT2G39410| Symbols: | hydrolase, alpha/beta fold family protein | chr2:16462745-16465295 FORWARD no origin	
JCVI_24505	1.530	moderately similar to (355)AT5G26667| Symbols: | uridylate kinase / uridine monophosphate kinase / UMP kinase (PYR6) | chr5:927666	-1.345
EX099013	1.530	moderately similar to (322)AT3G56410| Symbols: | similar to unknown protein [Arabidopsis thaliana] (TAIR:AT5G05190.1); similar to u	
JCVI_3100	1.530	moderately similar to (412)AT4G18040| Symbols: EIF4E, LSP1, CUM1, AT.EIF4E1 | EIF4E (EUKARYOTIC TRANSLATION INITATI	
EE464760	1.530	weakly similar to (160)AT5G67220| Symbols: | nitrogen regulation family protein | chr5:26837502-26839152 REVERSE [20171]	
JCVI_22860	1.530	no original description	
EV148082	1.530	no similarity	
JCVI_28117	1.529	moderately similar to (258)AT1G60960| Symbols: IRT3 | IRT3 (Iron regulated transporter 3); cation transmembrane transporter/ metal ion	
EE543306	1.529	no similarity	
CD813400	1.529	moderately similar to (279)AT3G02600| Symbols: LPP3, ATLPP3 | ATLPP3/LPP3 (LIPID PHOSPHATE PHOSPHATASE 3) | chr3:552	
JCVI_24431	1.529	moderately similar to (264)AT1G79150| Symbols: | binding | chr1:29777609-29782767 REVERSE no original description	
JCVI_17685	1.529	moderately similar to (319)AT1G18400| Symbols: BEE1 | BEE1 (BR ENHANCED EXPRESSION 1); transcription factor | chr1:6331457-	-2.220
JCVI_35517	1.529	moderately similar to (269)AT3G59630| Symbols: | diphthamide synthesis DPH2 family protein | chr3:22036332-22038753 REVERSE no	
JCVI_33829	1.529	moderately similar to (450)AT5G67500| Symbols: | porin, putative | chr5:26952449-26954349 FORWARDmoderately similar to (263)VI	
CX268781	1.529	moderately similar to (275)AT1G80360| Symbols: | aminotransferase class I and II family protein | chr1:30213628-30215535 REVERSE [	
EX110626	1.529	moderately similar to (216)AT2G20410| Symbols: | activating signal cointegrator-related | chr2:8809642-8811275 FORWARD [21827]	
JCVI_10190	1.529	moderately similar to (337)AT2G03430| Symbols: | ankyrin repeat family protein | chr2:1036189-1037533 REVERSE no original descript	
JCVI_95	1.529	moderately similar to (483)AT3G03990| Symbols: | esterase/lipase/thioesterase family protein | chr3:1033795-1034598 FORWARD no or	
EV013271	1.528	no similarity	
JCVI_7572	1.528	moderately similar to (351)AT5G08100| Symbols: | L-asparaginase / L-asparagine amidohydrolase | chr5:2593243-2594587 REVERSEmo	
ES967347	1.528	no similarity	
JCVI_14489	1.528	weakly similar to (147)AT2G33610| Symbols: CHB2, ATSWI3B | ATSWI3B (Arabidopsis thaliana switching protein 3B); DNA binding |	
JCVI_2941	1.528	moderately similar to (219)AT3G02950| Symbols: | similar to unknown protein [Arabidopsis thaliana] (TAIR:AT5G16790.1); similar to u	
EV215634	1.528	moderately similar to (237)AT1G27960| Symbols: ECT9 | ECT9 (evolutionarily conserved C-terminal region 9) | chr1:9742346-9745635 F	
JCVI_40519	1.528	no original description	
JCVI_24084	1.528	moderately similar to (473)AT5G07680| Symbols: ANAC079, ATNAC4, ANAC080 | ANAC079/ANAC080/ATNAC4 (Arabidopsis NAC	
EV158162	1.528	moderately similar to (206)AT3G26920| Symbols: | F-box family protein | chr3:9922478-9925128 FORWARD [21484]	
ES960083	1.528	moderately similar to (365)AT3G17365| Symbols: | catalytic | chr3:5947150-5948772 REVERSE [21423] 18 725 725	
EE483833	1.528	weakly similar to (181)AT5G48670| Symbols: FEM111, AGL80 | AGL80/FEM111 (AGAMOUS-LIKE80); DNA binding / transcription fa	
EX103444	1.527	moderately similar to (303)AT5G09470| Symbols: | mitochondrial substrate carrier family protein | chr5:2949242-2950514 REVERSE [21	
EH425591	1.527	moderately similar to (231)AT4G25360| Symbols: | similar to YLS7 (yellow-leaf-specific gene 7) [Arabidopsis thaliana] (TAIR:AT5G516	
JCVI_20454	1.527	moderately similar to (411)AT4G35450| Symbols: AFT, AKR2A, AKR2 | AKR2 (ANKYRIN REPEAT-CONTAINING PROTEIN 2); pro	
EV206937	1.527	no similarity	-3.443
EV088049	1.527	weakly similar to (152)AT4G24265| Symbols: | unknown protein | chr4:12580750-12581172 REVERSE [21444]	
EE465780	1.527	weakly similar to (147)AT4G05590| Symbols: | similar to unknown protein [Arabidopsis thaliana] (TAIR:AT4G22310.1); similar to unkn	
CD828379	1.527	moderately similar to (275)AT5G55960| Symbols: | similar to unnamed protein product [Vitis vinifera] (GB:CAO45175.1); contains Inter	
JCVI_29103	1.527	highly similar to (652)AT4G28540| Symbols: CKL6, PAPK1 | CKL6/PAPK1 (Casein Kinase I-like 6); casein kinase I/ kinase | chr4:14107	
ES949285	1.527	no similarity	-2.572
JCVI_41248	1.526	highly similar to (545)AT2G20010| Symbols: | similar to unknown protein [Arabidopsis thaliana] (TAIR:AT2G25800.1); similar to unnam	
JCVI_32707	1.526	moderately similar to (401)AT5G58330| Symbols: | malate dehydrogenase (NADP), chloroplast, putative | chr5:23597236-23599513 REV	
EE447607	1.526	very weakly similar to (95.1)AT1G79380| Symbols: | copine-related | chr1:29865705-29867916 FORWARD [20125]	
ES992476	1.526	very weakly similar to (90.1)AT2G36530| Symbols: LOS2 | LOS2 (Low expression of osmotically responsive genes 1); phosphopyruvate hy	
EE547679	1.526	no similarity	
EE562010	1.526	no similarity	-1.123
JCVI_17710	1.526	highly similar to (577)AT2G26280| Symbols: CID7 | CID7; ATP binding / damaged DNA binding | chr2:11195221-11197657 REVERSE n	
JCVI_23841	1.526	moderately similar to (329)AT4G12900| Symbols: | gamma interferon responsive lysosomal thiol reductase family protein / GILT family p	
JCVI_22966	1.526	weakly similar to (103)AT1G27430| Symbols: | GYF domain-containing protein | chr1:9521032-9526915 REVERSE no original descriptio	
JCVI_11272	1.526	highly similar to (514)AT2G46070| Symbols: ATMPK12 | ATMPK12 (Arabidopsis thaliana MAP kinase 12); MAP kinase/ kinase | chr2:1	
JCVI_34799	1.526	highly similar to (647)AT5G49880| Symbols: | mitotic checkpoint family protein | chr5:20299531-20305001 FORWARD no original descr	
AM388500	1.526	moderately similar to (260)AT5G48470| Symbols: | similar to unnamed protein product [Vitis vinifera] (GB:CAO17042.1) | chr5:1965903	
JCVI_13674	1.526	moderately similar to (337)AT1G34260| Symbols: | phosphatidylinositol-4-phosphate 5-kinase family protein | chr1:12485945-12491777 F	
EV105152	1.526	no similarity	
EL589549	1.525	moderately similar to (283)AT1G03620| Symbols: | phagocytosis and cell motility protein ELMO1-related | chr1:904318-906012 REVERS	
JCVI_10032	1.525	moderately similar to (271)AT5G28590| Symbols: | DNA-binding protein-related | chr5:10579450-10581563 REVERSE no original descr	
JCVI_19062	1.525	moderately similar to (483)AT1G67300| Symbols: | hexose transporter, putative | chr1:25197495-25200414 REVERSEvery weakly simila	
JCVI_7464	1.525	moderately similar to (314)AT1G01020| Symbols: ARV1 | ARV1 | chr1:7315-8666 REVERSE no original description	
JCVI_15138	1.525	highly similar to (593)AT5G63860| Symbols: UVR8 | UVR8 (UVB-RESISTANCE 8) | chr5:25572047-25575813 REVERSE no original d	
JCVI_10291	1.525	moderately similar to (403)AT5G63010| Symbols: | WD-40 repeat family protein | chr5:25298965-25300193 FORWARD no original desc	
JCVI_30252	1.525	highly similar to (745)AT3G17470| Symbols: | RelA/SpoT domain-containing protein / calcium-binding EF-hand family protein | chr3:597	-1.922
EV189045	1.525	moderately similar to (253)AT1G62120| Symbols: | mitochondrial transcription termination factor-related / mTERF-related | chr1:229639	

JCVI_8775	1.525	moderately similar to (394)AT1G26940| Symbols: | peptidyl-prolyl cis-trans isomerase cyclophilin-type family protein | chr1:9343180-934	
EE442478	1.525	no similarity	
JCVI_26613	1.525	no original description	
JCVI_24068	1.524	highly similar to (602)AT4G26080| Symbols: ABI1 | ABI1 (ABA INSENSITIVE 1); calcium ion binding / protein serine/threonine phosph	
RC_AI352714	1.524	no similarity	
JCVI_21108	1.524	highly similar to (582)AT4G38220| Symbols: | aminoacylase, putative / N-acyl-L-amino-acid amidohydrolase, putative | chr4:17925245-1	
AM058003	1.524	weakly similar to (154)AT1G48400| Symbols: | F-box family protein | chr1:17885804-17887523 REVERSE [17712]	
JCVI_5825	1.524	moderately similar to (249)AT1G48320| Symbols: | thioesterase family protein | chr1:17858692-17859245 REVERSE no original descript	
JCVI_17022	1.524	highly similar to (689)AT4G37910| Symbols: MTHSC70-1 | MTHSC70-1 (mitochondrial heat shock protein 70-1); ATP binding / unfolde	
CO729375	1.524	weakly similar to (120)AT2G04070| Symbols: | transporter | chr2:1353944-1355787 REVERSE [12919] 32 347 347	
EE537039	1.524	no similarity	
JCVI_1769	1.524	moderately similar to (378)AT3G52380| Symbols: PDE322, CP33 | CP33 (PIGMENT DEFECTIVE 322); RNA binding | chr3:19432597-1	
ES964894	1.524	weakly similar to (133)AT1G26830| Symbols: CUL3A, ATCUL3A, ATCUL3, CUL3 | ATCUL3/ATCUL3A/CUL3/CUL3A (Cullin 3A); p	
EV174135	1.524	very weakly similar to (80.1)AT4G24590| Symbols: | similar to unknown protein [Arabidopsis thaliana] (TAIR:AT5G49710.3); similar to u	
RC_ES964533	1.524	no similarity	
JCVI_40768	1.523	highly similar to (548)AT5G63940| Symbols: | protein kinase family protein | chr5:25605480-25608455 FORWARDweakly similar to (18	
EH415074	1.523	moderately similar to (322)AT5G22760| Symbols: | PHD finger family protein | chr5:7571638-7577665 FORWARD [20767]	
CO749752	1.523	no similarity	
EE520794	1.523	very weakly similar to (95.1)AT5G42330| Symbols: | similar to unnamed protein product [Vitis vinifera] (GB:CAO62026.1) | chr5:169439	
EV058466	1.523	very weakly similar to (94.7)AT4G14110| Symbols: CSN8, FUS7, EMB143, COP9 | COP9 (CONSTITUTIVE PHOTOMORPHOGENIC 9	
JCVI_4237	1.523	moderately similar to (337)AT3G60300| Symbols: | RWD domain-containing protein | chr3:22296346-22298357 FORWARD no original d	1.277
EV213527	1.523	very weakly similar to (86.7)AT5G50360| Symbols: | similar to unknown protein [Arabidopsis thaliana] (TAIR:AT5G63350.1); similar to u	
JCVI_23010	1.523	moderately similar to (218)AT2G36530| Symbols: LOS2 | LOS2 (Low expression of osmotically responsive genes 1); phosphopyruvate hy	
ES978589	1.522	no similarity	-2.202
EE484184	1.522	weakly similar to (179)AT1G23950| Symbols: | similar to unknown protein [Arabidopsis thaliana] (TAIR:AT1G23970.2); contains InterPr	
RC_AM387571	1.522	no similarity	
JCVI_32926	1.522	moderately similar to (225)AT1G12530| Symbols: | similar to unknown protein [Arabidopsis thaliana] (TAIR:AT1G56420.1); similar to C	
DY023971	1.522	moderately similar to (289)AT5G16640| Symbols: | pentatricopeptide (PPR) repeat-containing protein | chr5:5461034-5462548 FORWAR	-3.128
JCVI_11653	1.522	moderately similar to (268)AT1G55900| Symbols: EMB1860, TIM50 | TIM50 (EMBRYO DEFECTIVE 1860) | chr1:20906828-20909085	
JCVI_5061	1.522	moderately similar to (496)AT5G27830| Symbols: | similar to hypothetical protein [Vitis vinifera] (GB:CAN74239.1) | chr5:9861356-986	
RC_H07279	1.522	no similarity	
EV212837	1.522	moderately similar to (208)AT1G12900| Symbols: GAPA-2 | GAPA-2 | chr1:4392632-4393848 REVERSEmoderately similar to (208)G3F	
EV015571	1.522	moderately similar to (381)AT1G07910| Symbols: ATRNL, RNL | ATRNL/RNL (ARABIDOPSIS THALIANA RNA LIGASE); 2',3'-cycli	
JCVI_34974	1.521	weakly similar to (123)AT2G35470| Symbols: | similar to unknown [Populus trichocarpa] (GB:ABK94143.1) | chr2:14916093-14916799 I	
JCVI_855	1.521	moderately similar to (210)AT3G56090| Symbols: ATFER3 | ATFER3 (FERRITIN 3); ferric iron binding | chr3:20825329-20826963 REV	
JCVI_9112	1.521	moderately similar to (317)AT1G69010| Symbols: BIM2 | BIM2 (BES1-INTERACTING MYC-LIKE PROTEIN 2); DNA binding / transc	
JCVI_32930	1.521	moderately similar to (293)AT1G19480| Symbols: | HhH-GPD base excision DNA repair family protein | chr1:6744511-6745644 FORWA	
EE531940	1.521	moderately similar to (377)AT2G21390| Symbols: | coatomer protein complex, subunit alpha, putative | chr2:9159508-9163657 FORWAR	
EX133313	1.521	moderately similar to (225)AT2G28370| Symbols: | Identical to UPF0497 membrane protein At2g28370 [Arabidopsis Thaliana] (GB:Q9S	
EX033939	1.521	no similarity	
EE463340	1.521	moderately similar to (301)AT4G19660| Symbols: NPR4 | NPR4 (NPR1-LIKE PROTEIN 4); protein binding | chr4:10696276-10698253 F	
JCVI_38453	1.521	moderately similar to (347)AT5G27220| Symbols: | protein transport protein-related | chr5:9578760-9582755 FORWARD no original desc	
EX057560	1.520	weakly similar to (151)AT1G51170| Symbols: | protein kinase family protein | chr1:18957293-18958507 REVERSE [21813]	-1.628
JCVI_35673	1.520	highly similar to (511)AT1G64400| Symbols: | long-chain-fatty-acid--CoA ligase, putative / long-chain acyl-CoA synthetase, putative | chr	
CX271810	1.520	moderately similar to (282)AT2G36200| Symbols: | kinesin motor protein-related | chr2:15186818-15192268 REVERSEweakly similar to	-1.255
JCVI_206	1.520	highly similar to (884)AT1G77760| Symbols: GNR1, NR1, NIA1 | NIA1 (NITRATE REDUCTASE 1) | chr1:29240899-29244261 REVER	1.621
JCVI_11856	1.520	highly similar to (577)AT3G43920| Symbols: DCL3 | DCL3 (DICER-LIKE 3); RNA binding / ribonuclease III | chr3:15764535-15771817	
JCVI_35639	1.520	moderately similar to (398)AT1G14610| Symbols: VALRS, TWN2 | TWN2 (TWIN 2); ATP binding / aminoacyl-tRNA ligase | chr1:50084	
CN730979	1.520	weakly similar to (122)AT2G27950| Symbols: | similar to protein binding / zinc ion binding [Arabidopsis thaliana] (TAIR:AT5G04460.1);	
ES930324	1.520	weakly similar to (108)AT2G27040| Symbols: OCP11, AGO4 | AGO4 (ARGONAUTE 4); nucleic acid binding | chr2:11543873-11548581	
JCVI_38784	1.520	very weakly similar to (89.4)AT1G78920| Symbols: AVPL1, AVP2 | AVP2 (ARABIDOPSIS VACUOLAR H+-PYROPHOSPHATASE 2)	
JCVI_38445	1.519	weakly similar to (160)AT3G23870| Symbols: | permease-related | chr3:8620260-8621762 FORWARD no original description	
JCVI_14166	1.519	highly similar to (504)AT1G59950| Symbols: | aldo/keto reductase, putative | chr1:22071698-22074253 REVERSEmoderately similar to (	
JCVI_9705	1.519	moderately similar to (386)AT5G21010| Symbols: ATBPM5 | ATBPM5 (BTB-POZ AND MATH DOMAIN 5); protein binding | chr5:713	
EV111360	1.519	no similarity	
JCVI_40225	1.519	moderately similar to (278)AT4G26450| Symbols: WIP1 | WIP1 (WPP-DOMAIN INTERACTING PROTEIN 1); protein heterodimerizati	
EE563515	1.519	no similarity	
CD841599	1.519	no similarity	
ES996506	1.519	no similarity	
EE420750	1.519	weakly similar to (145)AT5G53280| Symbols: PDV1 | PDV1 (PLASTID DIVISION1) | chr5:21624812-21626306 FORWARD [20149]	
JCVI_29980	1.519	weakly similar to (199)AT4G19670| Symbols: | zinc finger (C3HC4-type RING finger) family protein | chr4:10699393-10701352 REVER	
JCVI_34405	1.518	weakly similar to (109)AT2G39700| Symbols: ATEXPA4, ATHEXP ALPHA 1.6, ATEXPA4 | ATEXPA4 (ARABIDOPSIS THALIANA EX	
EV006381	1.518	moderately similar to (362)AT5G62480| Symbols: GST14, GST14B, ATGSTU9 | ATGSTU9 (GLUTATHIONE S-TRANSFERASE TAU	
JCVI_9992	1.518	moderately similar to (430)AT4G13430| Symbols: | aconitase family protein / aconitate hydratase family protein | chr4:7804190-7807785	
JCVI_41612	1.518	no original description	
JCVI_41601	1.518	highly similar to (505)AT5G49760| Symbols: | leucine-rich repeat family protein / protein kinase family protein | chr5:20233905-2023827	
JCVI_42315	1.518	highly similar to (525)AT2G35155| Symbols: | catalytic | chr2:14826288-14828409 REVERSE no original description	
EV012854	1.518	no similarity	
JCVI_2020	1.517	moderately similar to (308)AT3G11580| Symbols: | DNA-binding protein, putative | chr3:3650585-3651277 REVERSE no original descrip	
JCVI_35813	1.517	moderately similar to (418)AT3G19740| Symbols: | ATPase | chr3:6855950-6862936 REVERSE no original description	
EV011943	1.517	no similarity	
JCVI_24487	1.517	moderately similar to (249)AT2G33480| Symbols: ANAC041 | ANAC041 (Arabidopsis NAC domain containing protein 41) | chr2:141883	
CX268682	1.517	weakly similar to (109)AT5G66720| Symbols: | 5-azacytidine resistance protein -related | chr5:26656241-26657771 REVERSE [16816]	
JCVI_37503	1.517	moderately similar to (384)AT3G53800| Symbols: | armadillo/beta-catenin repeat family protein | chr3:19941920-19943697 FORWARD r	
EE449633	1.517	no similarity	-3.864
JCVI_29220	1.517	moderately similar to (226)AT1G32750| Symbols: TAF1, HAF1, HAC13, GTD1, HAF01 | HAF01 (HISTONE ACETYLTRANSFERASE	
JCVI_27898	1.517	moderately similar to (280)AT1G56720| Symbols: | protein kinase family protein | chr1:21267295-21269224 REVERSE no original descri	
JCVI_34551	1.516	moderately similar to (476)AT3G47960| Symbols: | proton-dependent oligopeptide transport (POT) family protein | chr3:17709111-17711	
JCVI_35360	1.516	moderately similar to (377)AT2G28680| Symbols: | cupin family protein | chr2:12310195-12311824 REVERSE no original description	
EE503482	1.516	moderately similar to (281)AT5G58140| Symbols: NPL1, PHOT2 | PHOT2 (NON PHOTOTROPIC HYPOCOTYL 1-LIKE); kinase | chr5	
JCVI_15311	1.516	moderately similar to (463)AT3G43270| Symbols: | pectinesterase family protein | chr3:15233390-15236112 REVERSEmoderately simila	

JCVI_38452	1.516	weakly similar to (108)AT4G08850| Symbols: | leucine-rich repeat family protein / protein kinase family protein | chr4:5637464-5640493	
AM394309	1.516	weakly similar to (140)AT1G04250| Symbols: IAA17, AXR3 | AXR3 (AUXIN RESISTANT 3); transcription factor | chr1:1136381-11383	
EV121392	1.516	moderately similar to (363)AT5G08660| Symbols: | similar to unknown protein [Arabidopsis thaliana] (TAIR:AT1G34320.1); similar to u	
JCVI_4247	1.516	moderately similar to (346)AT1G02910| Symbols: LPA1 | LPA1 (LOW PSII ACCUMULATION1); binding | chr1:655749-658125 REVEI	
DN961343	1.516	no similarity	
JCVI_35031	1.516	moderately similar to (469)AT1G07910| Symbols: ATRNL, RNL | ATRNL/RNL (ARABIDOPSIS THALIANA RNA LIGASE); 2',3'-cycli	
JCVI_24477	1.516	weakly similar to (168)AT4G03080| Symbols: | kelch repeat-containing serine/threonine phosphoesterase family protein | chr4:1359935-1:	
JCVI_17991	1.516	moderately similar to (371)AT3G63460| Symbols: | WD-40 repeat family protein | chr3:23441984-23448216 REVERSE no original descr	
JCVI_874	1.515	moderately similar to (490)AT1G30630| Symbols: | coatomer protein epsilon subunit family protein / COPE family protein | chr1:1085852	
JCVI_34351	1.515	no original description	
EE441783	1.515	weakly similar to (172)AT1G68980| Symbols: | pentatricopeptide (PPR) repeat-containing protein | chr1:25936686-25938545 FORWARD	
JCVI_19967	1.515	highly similar to (639)AT4G12250| Symbols: GAE5 | GAE5 (UDP-D-GLUCURONATE 4-EPIMERASE 5); catalytic | chr4:7289532-729(	
EE407427	1.515	no similarity	
JCVI_15070	1.515	no original description	
JCVI_18768	1.515	no original description	
CD837498	1.515	moderately similar to (397)AT3G11910| Symbols: | ubiquitin-specific protease, putative | chr3:3761764-3770296 REVERSE [13981]	
JCVI_7417	1.515	weakly similar to (122)AT4G08395| Symbols: | unknown protein | chr4:5319631-5320709 FORWARD no original description	
RC_EE564107	1.515	no similarity	
EE471385	1.515	moderately similar to (284)AT3G26115| Symbols: | catalytic/ pyridoxal phosphate binding | chr3:9543538-9545483 FORWARD [20163]	
ES898084	1.515	weakly similar to (117)AT1G80300| Symbols: ATNTT1 | ATNTT1; ATP:ADP antiporter | chr1:30196846-30199172 FORWARD [21405]	
CD825339	1.515	moderately similar to (384)AT1G69830| Symbols: ATAMY3, AMY3 | AMY3/ATAMY3 (ALPHA-AMYLASE-LIKE 3); alpha-amylase | c	
AM389400	1.515	moderately similar to (254)AT1G76510| Symbols: | ARID/BRIGHT DNA-binding domain-containing protein | chr1:28713821-28717392	
EV044657	1.515	weakly similar to (150)AT1G07200| Symbols: | ATP-dependent Clp protease ClpB protein-related | chr1:2209032-2212315 REVERSE [2	
ES990837	1.515	no similarity	
EV137114	1.514	no similarity	
ES902653	1.514	moderately similar to (278)AT4G12080| Symbols: | DNA-binding family protein | chr4:7239460-7241240 FORWARD [21432] 27 894 89	
JCVI_17033	1.514	moderately similar to (367)AT1G73090| Symbols: | similar to unnamed protein product [Vitis vinifera] (GB:CAO23197.1) | chr1:2749192	
AM058551	1.514	no similarity	
JCVI_7366	1.514	moderately similar to (392)AT3G12170| Symbols: | DNAJ heat shock N-terminal domain-containing protein | chr3:3881028-3882662 FOI	-1.524
JCVI_13169	1.514	highly similar to (610)AT1G06520| Symbols: ATGPAT1, GPAT1 | ATGPAT1/GPAT1 (GLYCEROL-3-PHOSPHATE ACYLTRANSFEF	
EE534751	1.514	very weakly similar to (91.3)AT1G22310| Symbols: MBD8 | MBD8 (methyl-CpG-binding domain 8) | chr1:7881703-7883633 REVERSE [	
JCVI_20462	1.514	weakly similar to (114)AT1G12680| Symbols: PEPKR2 | PEPKR2 (PHOSPHOENOLPYRUVATE CARBOXYLASE-RELATED KINAS	
JCVI_14223	1.514	no original description	-1.871
ES966702	1.514	weakly similar to (135)AT5G42540| Symbols: XRN2 | XRN2 (EXORIBONUCLEASE 2); 5'-3' exonuclease/ nucleic acid binding | chr5:17	
JCVI_6540	1.514	weakly similar to (139)AT2G47590| Symbols: PHR2 | PHR2 (PHOTOLYASE/BLUE-LIGHT RECEPTOR 2) | chr2:19528958-19530802	
JCVI_41180	1.514	weakly similar to (159)AT4G29100| Symbols: | ethylene-responsive family protein | chr4:14341146-14344581 FORWARD no original de	
JCVI_795	1.513	moderately similar to (233)AT2G39330| Symbols: | jacalin lectin family protein | chr2:16426865-16428651 REVERSE no original descrip	
EV106903	1.513	moderately similar to (201)AT1G47128| Symbols: RD21A, RD21 | RD21 (RESPONSIVE TO DEHYDRATION 21); cysteine-type peptid	-2.397
JCVI_7519	1.513	moderately similar to (348)AT5G13720| Symbols: | structural constituent of ribosome | chr5:4427963-4429032 FORWARD no original de	
JCVI_5135	1.513	moderately similar to (284)AT3G13062| Symbols: | similar to unknown protein [Arabidopsis thaliana] (TAIR:AT1G55960.1); similar to u	
JCVI_36863	1.513	no original description	
JCVI_20641	1.513	moderately similar to (421)AT1G06780| Symbols: GAUT6 | GAUT6 (Galacturonosyltransferase 6); polygalacturonate 4-alpha-galacturono	
EE478690	1.513	weakly similar to (117)AT5G05365| Symbols: | metal ion binding | chr5:1590126-1590673 FORWARD [20132] 1 375 411	
L38157	1.513	weakly similar to (165)AT1G19270| Symbols: | ubiquitin interaction motif-containing protein / LIM domain-containing protein | chr1:666	
JCVI_32576	1.513	moderately similar to (451)AT4G01700| Symbols: | chitinase, putative | chr4:732413-733487 REVERSEmoderately similar to (313)CHI2	
EX130668	1.513	weakly similar to (142)AT1G64200| Symbols: VHA-E3 | VHA-E3 (VACUOLAR H+-ATPASE SUBUNIT E ISOFORM 3); hydrogen ion	
CX281009	1.513	moderately similar to (227)AT4G25910| Symbols: ATCNFU3, NFU3 | NFU3 (NFU domain protein 3) | chr4:13164137-13165103 FORWA	
JCVI_39020	1.513	moderately similar to (333)AT1G06240| Symbols: | oxidoreductase/ transition metal ion binding | chr1:1911165-1912874 FORWARD no	
JCVI_13602	1.512	moderately similar to (217)AT5G23420| Symbols: HMGB6 | HMGB6 (High mobility group B 6); transcription factor | chr5:7888715-7890	
EV202094	1.512	weakly similar to (144)AT3G01870| Symbols: | similar to unknown protein [Arabidopsis thaliana] (TAIR:AT2G44260.1); similar to unkn	
JCVI_41693	1.512	highly similar to (501)AT4G28570| Symbols: | alcohol oxidase-related | chr4:14119554-14121929 FORWARD no original description	
ES946915	1.512	weakly similar to (147)AT1G03750| Symbols: SWI2, SNF2, CHR9 | CHR9/SNF2/SWI2 (chromatin remodeling 9); helicase | chr1:937919-	
EH421119	1.512	moderately similar to (279)AT4G18210| Symbols: ATPUP10 | ATPUP10 (Arabidopsis thaliana purine permease 10); purine transmembra	
JCVI_20505	1.512	moderately similar to (374)AT5G54770| Symbols: TZ, THI1 | THI1 (THIAZOLE REQUIRING) | chr5:22263860-22265117 FORWARDm	
EV210650	1.512	weakly similar to (200)AT3G06980| Symbols: | DEAD/DEAH box helicase, putative | chr3:2201537-2204668 FORWARD [21491] 52 74	
ES911229	1.511	moderately similar to (309)AT5G48120| Symbols: | binding | chr5:19525436-19532000 REVERSE [21430]	
EV130373	1.511	no similarity	1.970
EX135524	1.511	moderately similar to (234)AT5G28050| Symbols: | cytidine/deoxycytidylate deaminase family protein | chr5:10044213-10045488 REVEI	
JCVI_6785	1.511	weakly similar to (160)AT1G10270| Symbols: GRP23 | GRP23 (GLUTAMINE-RICH PROTEIN23); binding | chr1:3363537-3366278 FO	
EH419638	1.511	no similarity	
EE500964	1.511	no similarity	
JCVI_29788	1.511	moderately similar to (330)AT2G14750| Symbols: AKN1, ATAKN1, APK | APK (APS KINASE); ATP binding / kinase/ transferase, trans	
JCVI_29475	1.511	highly similar to (660)AT5G42720| Symbols: | glycosyl hydrolase family 17 protein | chr5:17147763-17149991 FORWARDmoderately si	
EE555248	1.511	moderately similar to (285)AT5G51370| Symbols: | F-box family protein | chr5:20890009-20891418 FORWARD [20184]	
JCVI_23188	1.510	moderately similar to (299)AT1G33330| Symbols: | peptide chain release factor, putative | chr1:12084948-12086218 FORWARD no origi	
JCVI_36682	1.510	highly similar to (672)AT5G04040| Symbols: SDP1 | SDP1 (SUGAR-DEPENDENT1); triacylglycerol lipase | chr5:1090345-1093002 FOI	
JCVI_27219	1.510	moderately similar to (416)AT4G39955| Symbols: | hydrolase, alpha/beta fold family protein | chr4:18530567-18532082 FORWARD no c	
JCVI_40020	1.510	moderately similar to (376)AT1G26840| Symbols: ORC6, ATORC6 | ATORC6/ORC6 (Origin recognition complex protein 6); DNA bindi	
JCVI_7097	1.510	weakly similar to (188)AT5G10460| Symbols: | haloacid dehalogenase-like hydrolase family protein | chr5:3287823-3289845 FORWARD	
EV023701	1.510	moderately similar to (454)AT1G36280| Symbols: | adenylosuccinate lyase, putative / adenylosuccinase, putative | chr1:13641812-136441	
EV219946	1.510	no similarity	
EV129494	1.510	very weakly similar to (87.4)AT1G77490| Symbols: TAPX | TAPX; L-ascorbate peroxidase | chr1:29122582-29124940 FORWARD [2148(	
JCVI_11118	1.510	moderately similar to (328)AT5G40740| Symbols: | similar to Os02g0329300 [Oryza sativa (japonica cultivar-group)] (GB:NP_00104671	
JCVI_6950	1.510	moderately similar to (203)AT1G76810| Symbols: | eukaryotic translation initiation factor 2 family protein / eIF-2 family protein | chr1:28	
JCVI_30915	1.509	no original description	
JCVI_6258	1.509	highly similar to (502)AT1G53990| Symbols: GLIP3 | GLIP3 (GDSL-motif lipase 3); carboxylesterase/ lipase | chr1:20154684-20156420 I	
EX074554	1.509	moderately similar to (266)AT3G26340| Symbols: | 20S proteasome beta subunit E, putative | chr3:9651837-9653809 REVERSEmoderate	-2.780
JCVI_5475	1.509	moderately similar to (372)AT5G19150| Symbols: | carbohydrate kinase family | chr5:6426282-6428404 REVERSE no original descriptio	
JCVI_24672	1.509	nearly identical (1038)AT5G02250| Symbols: EMB2730, RNR1 | EMB2730/RNR1 (EMBRYO DEFECTIVE 2730); RNA binding / ribonu	-1.191
JCVI_18643	1.509	no original description	
JCVI_11402	1.509	moderately similar to (411)AT5G03760| Symbols: CSLA09, ATCSLA9, CSLA9, RAT4, ATCSLA09 | ATCSLA09 (RESISTANT TO AGI	

JCVI_14680	1.509	weakly similar to (200)AT3G57610| Symbols: ATPURA | ATPURA; adenylosuccinate synthase | chr3:21345496-21347580 REVERSEwea	
CN732026	1.509	no similarity	
JCVI_29499	1.509	moderately similar to (357)AT5G09390| Symbols: | CD2-binding protein-related | chr5:2913592-2915804 FORWARD no original descrip	
JCVI_6762	1.509	moderately similar to (223)AT5G20260| Symbols: | exostosin family protein | chr5:6836808-6837889 REVERSE no original description	
JCVI_29320	1.508	no original description	
JCVI_32125	1.508	moderately similar to (273)AT5G61930| Symbols: APO3 | APO3 (ACCUMULATION OF PHOTOSYSTEM ONE 3) | chr5:24883456-248	
JCVI_14493	1.508	moderately similar to (314)AT4G01883| Symbols: | similar to unknown protein [Arabidopsis thaliana] (TAIR:AT1G02475.1); similar to u	
JCVI_11021	1.508	weakly similar to (157)AT1G30515| Symbols: | unknown protein | chr1:10809414-10809848 REVERSE no original description	
EV210023	1.508	no similarity	
JCVI_15627	1.507	weakly similar to (155)AT2G38820| Symbols: | similar to unknown protein [Arabidopsis thaliana] (TAIR:AT3G22970.1); similar to unna	
JCVI_15473	1.507	moderately similar to (304)AT3G57470| Symbols: | peptidase M16 family protein / insulinase family protein | chr3:21280405-21286377 R	
EX113764	1.507	moderately similar to (239)AT4G23260| Symbols: | protein kinase | chr4:12167538-12170065 REVERSE [21827]	-1.321
JCVI_13992	1.507	no original description	
EL590550	1.507	weakly similar to (147)AT5G07950| Symbols: | similar to unnamed protein product [Vitis vinifera] (GB:CAO64456.1) | chr5:2540531-254	
JCVI_39455	1.507	moderately similar to (356)AT5G14760| Symbols: AO | AO (L-ASPARTATE OXIDASE); L-aspartate oxidase | chr5:4769136-4772015 F	
JCVI_17121	1.507	highly similar to (695)AT5G42250| Symbols: | alcohol dehydrogenase, putative | chr5:16911315-16914678 FORWARDmoderately simila	
DY022255	1.507	moderately similar to (210)AT5G20480| Symbols: EFR | EFR (EF-TU RECEPTOR); ATP binding / kinase/ protein serine/threonine kinase	
EX040878	1.507	very weakly similar to (94.0)AT5G13000| Symbols: GSL12, ATGSL12 | ATGSL12 (GLUCAN SYNTHASE-LIKE 12); 1,3-beta-glucan syn	
JCVI_24623	1.507	no original description	
EV108467	1.507	weakly similar to (108)AT3G16150| Symbols: | L-asparaginase, putative / L-asparagine amidohydrolase, putative | chr3:5471800-5473039	
EV046053	1.506	weakly similar to (107)AT4G38940| Symbols: | kelch repeat-containing F-box family protein | chr4:18152842-18153954 FORWARD [214	
EV109922	1.506	weakly similar to (179)AT3G20720| Symbols: | similar to hypothetical protein OsI_016901 [Oryza sativa (indica cultivar-group)] (GB:EA	
JCVI_37526	1.506	no original description	
EE503085	1.505	moderately similar to (272)AT2G38460| Symbols: | iron transporter-related | chr2:16110681-16113008 FORWARD [20129]	
JCVI_10262	1.505	moderately similar to (291)AT1G21010| Symbols: | similar to unknown protein [Arabidopsis thaliana] (TAIR:AT1G76600.1); similar to u	
EE455876	1.505	weakly similar to (167)AT4G38730| Symbols: | similar to unknown protein [Arabidopsis thaliana] (TAIR:AT2G21120.1); similar to unna	
JCVI_27637	1.505	moderately similar to (301)AT5G59540| Symbols: | oxidoreductase, 2OG-Fe(II) oxygenase family protein | chr5:24013841-24014802 RE	
JCVI_31339	1.505	highly similar to (754)AT2G15090| Symbols: | fatty acid elongase, putative | chr2:6549418-6550863 FORWARD no original description	
RC_ES968910	1.505	no similarity	
JCVI_2810	1.505	moderately similar to (431)AT1G80030| Symbols: | DNAJ heat shock protein, putative | chr1:30110291-30113766 REVERSEweakly simi	
JCVI_16407	1.505	moderately similar to (462)AT1G49760| Symbols: PAB8 | PAB8 (POLY(A) BINDING PROTEIN 8); RNA binding / translation initiation	
JCVI_22105	1.505	moderately similar to (459)AT4G24260| Symbols: ATGH9A3, KOR3 | ATGH9A3/KOR3 (ARABIDOPSIS THALIANA GLYCOSYL HY	-1.931
EX136350	1.504	weakly similar to (192)AT4G10450| Symbols: | 60S ribosomal protein L9 (RPL90D) | chr4:6463197-6464454 REVERSEweakly similar to	
JCVI_22853	1.504	moderately similar to (215)AT5G53290| Symbols: CRF3 | CRF3 (CYTOKININ RESPONSE FACTOR 3); DNA binding / transcription fac	
EV158224	1.504	weakly similar to (124)AT1G19170| Symbols: | glycoside hydrolase family 28 protein / polygalacturonase (pectinase) family protein | chr1	
JCVI_1226	1.504	moderately similar to (266)AT1G76020| Symbols: | similar to unknown protein [Arabidopsis thaliana] (TAIR:AT1G20225.1); similar to u	
JCVI_26495	1.504	highly similar to (691)AT1G22540| Symbols: | proton-dependent oligopeptide transport (POT) family protein | chr1:7964191-7966211 FO	
JCVI_13299	1.504	highly similar to (552)AT5G24760| Symbols: | alcohol dehydrogenase, putative | chr5:8495038-8497093 REVERSEmoderately similar to	
JCVI_38978	1.504	moderately similar to (231)AT3G63310| Symbols: | glutamate binding / | chr3:23398913-23399857 REVERSE no original description	
JCVI_2618	1.504	moderately similar to (360)AT4G17050| Symbols: | transcription factor | chr4:9589681-9592432 FORWARD no original description	
EE435702	1.504	moderately similar to (243)AT5G03430| Symbols: | phosphoadenosine phosphosulfate (PAPS) reductase family protein | chr5:849236-852	
EE566383	1.504	no similarity	
CV434111	1.503	no similarity	
RC_EE558440	1.503	no similarity	
EH428121	1.503	moderately similar to (296)AT5G66790| Symbols: | protein kinase family protein | chr5:26682407-26684613 FORWARDweakly similar to	
EE516526	1.503	no similarity	
JCVI_35545	1.503	moderately similar to (203)AT5G06560| Symbols: | similar to unknown protein [Arabidopsis thaliana] (TAIR:AT3G11850.1); similar to u	
JCVI_19379	1.503	moderately similar to (269)AT3G06340| Symbols: | DNAJ heat shock N-terminal domain-containing protein | chr3:1920613-1922634 RE	
JCVI_37031	1.503	moderately similar to (226)AT1G17140| Symbols: | tropomyosin-related | chr1:5856733-5857854 REVERSE no original description	
EV001401	1.502	no similarity	
JCVI_41965	1.502	highly similar to (590)AT5G46190| Symbols: | KH domain-containing protein | chr5:18740662-18743061 REVERSE no original descripti	
JCVI_8363	1.502	moderately similar to (451)AT3G63270| Symbols: | similar to unknown protein [Arabidopsis thaliana] (TAIR:AT3G55350.1); similar to u	
JCVI_10947	1.502	moderately similar to (348)AT3G27220| Symbols: | kelch repeat-containing protein | chr3:10052979-10054831 REVERSE no original des	
EX109665	1.502	no similarity	
AT000497	1.502	no similarity	
DY025805	1.502	moderately similar to (333)AT3G48270| Symbols: CYP71A26 | CYP71A26 (cytochrome P450, family 71, subfamily A, polypeptide 26); c	
EX063265	1.502	no similarity	
JCVI_27570	1.502	highly similar to (780)AT3G55260| Symbols: HEXO1, ATHEX2 | ATHEX2/HEXO1 (BETA-HEXOSAMINIDASE 1); beta-N-acetylhexo	
JCVI_6702	1.502	moderately similar to (367)AT3G15000| Symbols: | Identical to Uncharacterized mitochondrial protein At3g15000 [Arabidopsis Thaliana]	
JCVI_7226	1.501	moderately similar to (355)AT1G13080| Symbols: CYP71B2 | CYP71B2 (CYTOCHROME P450 71B2); oxygen binding | chr1:4459491-4	
AM395415	1.501	moderately similar to (220)AT1G62800| Symbols: ASP4 | ASP4 (ASPARTATE AMINOTRANSFERASE 4); catalytic/ pyridoxal phospha	
JCVI_4767	1.501	moderately similar to (332)AT3G10260| Symbols: | reticulon family protein | chr3:3171418-3172573 REVERSE no original description	-1.212
DY001426	1.501	no similarity	
EV110827	1.501	no similarity	
JCVI_15426	1.501	moderately similar to (214)AT3G12660| Symbols: FLA14 | FLA14 (FASCICLIN-LIKE ARABINOGALACTAN PROTEIN 14 PRECURS	-1.232
AI352869	1.501	moderately similar to (233)AT3G47580| Symbols: | leucine-rich repeat transmembrane protein kinase, putative | chr3:17543672-17546795	
JCVI_6493	1.501	moderately similar to (306)AT1G33490| Symbols: | similar to unknown protein [Arabidopsis thaliana] (TAIR:AT4G10140.1); similar to u	
JCVI_16527	1.501	highly similar to (536)AT1G21080| Symbols: | DNAJ heat shock N-terminal domain-containing protein | chr1:7378811-7382264 REVERS	
JCVI_29880	1.500	moderately similar to (450)AT3G15990| Symbols: SULTR3;4 | SULTR3;4; sulfate transmembrane transporter | chr3:5427087-5430685 FO	-1.649
JCVI_19977	1.500	moderately similar to (246)AT3G17800| Symbols: | mRNA level of the MEB5.2 gene (At3g17800) remains unchanged after cutting the in	
JCVI_20589	1.500	no original description	-1.281
JCVI_10569	1.500	weakly similar to (143)AT5G03460| Symbols: | similar to unknown [Populus trichocarpa] (GB:ABK93498.1) | chr5:864390-865508 FOR	
JCVI_26194	1.500	weakly similar to (125)AT3G21175| Symbols: TIFY2B, ZML1 | ZML1 (ZIM-LIKE 1) | chr3:7422838-7423771 FORWARD no original de	
EE446602	1.500	weakly similar to (123)AT4G36730| Symbols: GBF1 | GBF1 (G-box binding factor 1); transcription factor | chr4:17309854-17311756 RE	
JCVI_18662	1.500	moderately similar to (403)AT5G55390| Symbols: EDM2 | EDM2; transcription factor | chr5:22465378-22471640 REVERSE no original	
JCVI_36363	1.500	weakly similar to (105)AT2G30440| Symbols: | chloroplast thylakoidal processing peptidase | chr2:12980321-12982104 FORWARD no o	-4.110
CD829521	1.500	moderately similar to (201)AT3G48310| Symbols: CYP71A22 | CYP71A22 (cytochrome P450, family 71, subfamily A, polypeptide 22); c	-1.288
JCVI_37384	1.500	moderately similar to (426)AT3G54920| Symbols: PMR6 | PMR6 (POWDERY MILDEW RESISTANT 6); lyase/ pectate lyase | chr3:203	
DY014593	1.500	moderately similar to (303)AT5G06050| Symbols: | dehydration-responsive protein-related | chr5:1820197-1823573 FORWARD [18966]	
JCVI_22919	1.500	moderately similar to (489)AT4G30870| Symbols: ATMUS81 | ATMUS81; endonuclease/ nucleic acid binding | chr4:15028592-15032467	
JCVI_26002	1.500	moderately similar to (409)AT3G49800| Symbols: | BSD domain-containing protein | chr3:18482942-18484661 REVERSE no original des	

AM057378	1.499	moderately similar to (338)AT3G60320| Symbols: | DNA binding | chr3:22303048-22306203 REVERSE [17712]	
ES905653	1.499	very weakly similar to (82.0)AT5G63370| Symbols: | protein kinase family protein | chr5:25402180-25403616 REVERSE [21429]	
EX043550	1.499	no similarity	
EX027936	1.499	moderately similar to (378)AT5G15080| Symbols: | protein kinase, putative | chr5:4886417-4888558 FORWARDweakly similar to (106)I	
EV213225	1.499	no similarity	
RC_EE392370	1.499	no similarity	
JCVI_10155	1.499	moderately similar to (254)AT5G35160| Symbols: | endomembrane protein 70, putative | chr5:13432175-13434151 FORWARD no origin	
AM391748	1.499	moderately similar to (414)AT3G60120| Symbols: | glycosyl hydrolase family 1 protein | chr3:22217322-22219927 FORWARDmoderatel	
JCVI_112	1.499	moderately similar to (410)AT1G04410| Symbols: | malate dehydrogenase, cytosolic, putative | chr1:1189417-1191266 REVERSEmodera	
DY014950	1.499	no similarity	
ES915455	1.499	moderately similar to (288)AT2G22790| Symbols: | similar to unknown protein [Arabidopsis thaliana] (TAIR:AT5G67020.1); similar to u	-1.512
EV171154	1.498	no similarity	
JCVI_3897	1.498	moderately similar to (311)AT5G53330| Symbols: | similar to hypothetical protein [Vitis vinifera] (GB:CAN66995.1); contains InterPro d	-2.388
EV042059	1.498	moderately similar to (388)AT1G49340| Symbols: ATPI4K ALPHA | ATPI4K ALPHA (Arabidopsis thaliana phosphatidylinositol 4-kinas	
AM056768	1.498	weakly similar to (122)AT3G11290| Symbols: | similar to unknown protein [Arabidopsis thaliana] (TAIR:AT2G19220.1); similar to unnar	
EX093564	1.498	moderately similar to (426)AT2G45550| Symbols: CYP76C4 | CYP76C4 (cytochrome P450, family 76, subfamily C, polypeptide 4); oxyg	
CV432283	1.498	no similarity	
JCVI_14149	1.498	moderately similar to (347)AT1G62305| Symbols: | similar to unknown protein [Arabidopsis thaliana] (TAIR:AT1G11940.1); similar to u	1.499
JCVI_35028	1.497	highly similar to (545)AT2G17790| Symbols: | similar to unknown protein [Arabidopsis thaliana] (TAIR:AT3G51310.1); similar to bindin	
EE535296	1.497	weakly similar to (140)AT1G22070| Symbols: TGA3 | TGA3 (TGA1a-related gene 3); DNA binding / calmodulin binding / transcription fa	-2.430
JCVI_31623	1.497	moderately similar to (280)AT3G61820| Symbols: | aspartyl protease family protein | chr3:22891049-22892500 REVERSE no original des	
JCVI_14035	1.497	moderately similar to (334)AT2G19470| Symbols: CKL5 | CKL5 (Casein Kinase I-like 5); casein kinase I/ kinase | chr2:8440933-8443377	
BG543653	1.497	no similarity	
JCVI_2350	1.497	moderately similar to (342)AT4G12060| Symbols: | Clp amino terminal domain-containing protein | chr4:7228263-7229892 REVERSE nc	
JCVI_40260	1.497	weakly similar to (151)AT4G12970| Symbols: | similar to unnamed protein product [Vitis vinifera] (GB:CAO17947.1) | chr4:7586241-758	
DY028929	1.497	moderately similar to (242)AT2G32940| Symbols: AGO6 | AGO6 (ARGONAUTE 6); nucleic acid binding | chr2:13979295-13983933 RE	
JCVI_10938	1.497	moderately similar to (266)AT2G17840| Symbols: ERD7 | ERD7 (EARLY-RESPONSIVE TO DEHYDRATION 7) | chr2:7763005-77648	
JCVI_18425	1.497	moderately similar to (366)AT4G38150| Symbols: | pentatricopeptide (PPR) repeat-containing protein | chr4:17901205-17902113 REVER	
JCVI_34661	1.497	weakly similar to (103)AT1G73550| Symbols: | Encodes a Protease inhibitor/seed storage/LTP family protein | chr1:27651685-27652197	
JCVI_20286	1.497	no original description	-1.800
JCVI_24927	1.497	moderately similar to (305)AT5G39050| Symbols: | transferase family protein | chr5:15651825-15652871 FORWARD no original descript	-1.174
JCVI_12370	1.496	very weakly similar to (81.3)AT5G65870| Symbols: ATPSK5 | ATPSK5 (PHYTOSULFOKINE 5 PRECURSOR); growth factor | chr5:263	
JCVI_23395	1.496	highly similar to (544)AT3G10540| Symbols: | 3-phosphoinositide-dependent protein kinase, putative | chr3:3289921-3292434 FORWAR	
JCVI_6135	1.496	moderately similar to (378)AT2G35620| Symbols: | leucine-rich repeat transmembrane protein kinase, putative | chr2:14968266-1497171	
JCVI_35580	1.496	moderately similar to (496)AT4G36710| Symbols: | scarecrow transcription factor family protein | chr4:17306064-17307524 FORWARD	
JCVI_35401	1.496	weakly similar to (107)AT1G22710| Symbols: SUT1, ATSUC2, SUC2 | SUC2 (SUCROSE-PROTON SYMPORTER 2); carbohydrate trar	
JCVI_39513	1.496	weakly similar to (114)AT5G13320| Symbols: GDG1, WIN3, PBS3 | PBS3 (AVRPPHB SUSCEPTIBLE 3) | chr5:4268905-4270899 FOR	
JCVI_8521	1.496	moderately similar to (406)AT1G72010| Symbols: | TCP family transcription factor, putative | chr1:27111506-27112633 FORWARD no c	
EV194331	1.496	weakly similar to (191)AT1G26150| Symbols: | protein kinase | chr1:9039777-9042860 REVERSEvery weakly similar to (100)PSKR_DA	
ES966584	1.495	very weakly similar to (90.1)AT2G37980| Symbols: | similar to unknown protein [Arabidopsis thaliana] (TAIR:AT5G01100.1); similar to u	
EL589573	1.495	no similarity	
H07518	1.495	no similarity	
JCVI_5199	1.495	weakly similar to (113)AT3G02830| Symbols: ZFN1 | ZFN1 (ZINC FINGER PROTEIN 1); nucleic acid binding | chr3:614082-615923 FC	
EX132929	1.495	moderately similar to (213)AT3G51660| Symbols: | macrophage migration inhibitory factor family protein / MIF family protein | chr3:191	
JCVI_13993	1.495	weakly similar to (199)AT5G64730| Symbols: | transducin family protein / WD-40 repeat family protein | chr5:25890372-25892247 FOR	
JCVI_5559	1.495	moderately similar to (251)AT1G10720| Symbols: | BSD domain-containing protein | chr1:3562965-3564447 FORWARD no original desc	
JCVI_41755	1.495	highly similar to (618)AT1G11170| Symbols: | similar to unknown protein [Arabidopsis thaliana] (TAIR:AT1G61240.2); similar to unkno	
JCVI_36831	1.495	highly similar to (735)AT1G31230| Symbols: AK-HSDH I, AK-HSDH | AK-HSDH/AK-HSDH I (ASPARTATE KINASE-HOMOSERINE	
JCVI_3870	1.495	moderately similar to (224)AT3G05000| Symbols: | transport protein particle (TRAPP) component Bet3 family protein | chr3:1387450-13	
JCVI_24347	1.495	moderately similar to (240)AT1G16470| Symbols: PAB1 | PAB1 (PROTEASOME SUBUNIT PAB1); peptidase | chr1:5623116-5625433	
EV070626	1.495	weakly similar to (127)AT5G19830| Symbols: | aminoacyl-tRNA hydrolase | chr5:6703385-6705127 FORWARD [21443]	
JCVI_15038	1.495	moderately similar to (202)AT4G32600| Symbols: | zinc finger (C3HC4-type RING finger) family protein | chr4:15724016-15725743 FOI	
JCVI_15714	1.494	highly similar to (524)AT5G49720| Symbols: DEC, KOR, RSW2, OR16PEP, IRX2, KOR1, AtGH9A1 | AtGH9A1 (ARABIDOPSIS THAI	
JCVI_12798	1.494	moderately similar to (300)AT1G72050| Symbols: | zinc finger (C2H2 type) family protein | chr1:27119470-27120890 FORWARD no ori	
EX063606	1.494	no similarity	
EV099982	1.494	moderately similar to (233)AT3G05640| Symbols: | protein phosphatase 2C, putative / PP2C, putative | chr3:1640616-1642233 REVERSI	
JCVI_10186	1.494	moderately similar to (339)AT4G35335| Symbols: | nucleotide-sugar transporter family protein | chr4:16807291-16809809 FORWARD nc	
JCVI_33550	1.494	no original description	1.058
JCVI_1913	1.494	moderately similar to (375)AT1G21840| Symbols: UREF | UREF (UREASE ACCESSORY PROTEIN F); nickel ion binding | chr1:766684	
JCVI_32298	1.494	weakly similar to (194)AT4G38970| Symbols: | fructose-bisphosphate aldolase, putative | chr4:18163763-18165653 REVERSEweakly sin	
EV020611	1.494	no similarity	
JCVI_33291	1.494	moderately similar to (233)AT4G39840| Symbols: | similar to unnamed protein product [Vitis vinifera] (GB:CAO21162.1); similar to unn	
JCVI_29851	1.494	moderately similar to (371)AT1G30490| Symbols: ATHB9 | PHV (PHAVOLUTA); DNA binding / transcription factor | chr1:10796310-1(	-1.393
EX135587	1.494	moderately similar to (233)AT5G13860| Symbols: ELC-LIKE | ATELC-LIKE/ELC-LIKE; small conjugating protein ligase | chr5:4473214	2.965
JCVI_4499	1.493	moderately similar to (369)AT4G35630| Symbols: PSAT | PSAT (phosphoserine aminotransferase); phosphoserine transaminase | chr4:16	
CA991496	1.493	weakly similar to (187)AT3G54120| Symbols: | reticulon family protein (RTNLB12) | chr3:20052247-20053111 REVERSE [12405]	
EE457196	1.493	no similarity	
CN735168	1.493	no similarity	
EE512499	1.493	moderately similar to (227)AT4G20380| Symbols: LSD1 | LSD1 (LESION SIMULATING DISEASE) | chr4:11005023-11006449 FORWA	1.654
DY007266	1.493	moderately similar to (320)AT3G18260| Symbols: | reticulon family protein (RTNLB9) | chr3:6260334-6261510 REVERSE [18976]	
EV194211	1.493	weakly similar to (147)AT1G80900| Symbols: | magnesium transporter CorA-like family protein (MGT1) (MRS2) | chr1:30403181-30404	
JCVI_291	1.493	moderately similar to (276)AT4G11650| Symbols: ATOSM34 | ATOSM34 (OSMOTIN 34) | chr4:7025121-7026107 REVERSEmoderatel	
JCVI_31161	1.493	weakly similar to (176)AT1G78230| Symbols: | protein binding | chr1:29435119-29437409 FORWARD no original description	
JCVI_7087	1.492	moderately similar to (210)AT5G48220| Symbols: | indole-3-glycerol phosphate synthase, putative | chr5:19567665-19569272 FORWARI	
DY002229	1.492	no similarity	
JCVI_17206	1.492	moderately similar to (313)AT3G57480| Symbols: | zinc finger (C2H2 type, AN1-like) family protein | chr3:21289060-21290096 REVER!	
JCVI_20975	1.492	moderately similar to (286)AT4G16060| Symbols: | similar to unnamed protein product [Vitis vinifera] (GB:CAO44835.1) | chr4:9094436	
EV114070	1.492	no similarity	
CN733163	1.491	moderately similar to (226)AT3G51270| Symbols: | ATP binding / protein serine/threonine kinase | chr3:19044830-19047751 FORWARD	
EV203397	1.491	moderately similar to (364)AT5G67360| Symbols: ARA12 | ARA12; subtilase | chr5:26889418-26891691 REVERSE [21490] 39 792 792	
JCVI_20982	1.491	no original description	

JCVI_34681	1.491	moderately similar to (292)AT4G12570| Symbols: UPL5 | UPL5 (UBIQUITIN PROTEIN LIGASE 5); ubiquitin-protein ligase | chr4:7445	
EX040176	1.491	weakly similar to (121)AT5G38220| Symbols: | similar to unknown protein [Arabidopsis thaliana] (TAIR:AT1G66900.1); similar to hypot	-1.249
JCVI_34945	1.491	moderately similar to (312)AT5G06220| Symbols: | similar to unknown protein [Arabidopsis thaliana] (TAIR:AT3G11560.3); similar to u	
JCVI_9517	1.491	moderately similar to (462)AT1G60200| Symbols: | splicing factor PWI domain-containing protein / RNA recognition motif (RRM)-conta	
JCVI_2502	1.491	weakly similar to (188)AT5G24810| Symbols: | ABC1 family protein | chr5:8516905-8522619 REVERSE no original description	
DY018384	1.491	weakly similar to (183)AT1G67570| Symbols: | similar to unknown protein [Arabidopsis thaliana] (TAIR:AT1G50630.1); similar to unnar	
EE453769	1.491	moderately similar to (225)AT2G36305| Symbols: | prenyl-dependent CAAX protease | chr2:15221686-15223528 FORWARD [20178]	
EE423819	1.491	no similarity	
JCVI_41831	1.491	no original description	
EV208292	1.491	no similarity	
EE555566	1.491	no similarity	
EX089193	1.490	weakly similar to (139)AT5G56440| Symbols: | F-box family protein | chr5:22872968-22874343 REVERSE [21823]	
RC_EE561843	1.490	no similarity	
EE449342	1.490	no similarity	
EV123240	1.490	very weakly similar to (100)AT5G67230| Symbols: | glycosyl transferase family 43 protein | chr5:26839732-26841407 FORWARD [2147	
JCVI_4281	1.490	moderately similar to (343)AT1G14740| Symbols: | similar to unknown protein [Arabidopsis thaliana] (TAIR:AT3G63500.2); similar to u	2.085
CX189756	1.490	moderately similar to (315)AT5G06160| Symbols: | splicing factor-related | chr5:1862624-1866299 REVERSE [16807]	
EE453034	1.489	no similarity	
JCVI_11753	1.489	moderately similar to (321)AT1G53210| Symbols: | sodium/calcium exchanger family protein / calcium-binding EF hand family protein | c	
ES966835	1.489	moderately similar to (244)AT4G32640| Symbols: | sec23/sec24 transport protein-related | chr4:15742666-15750550 FORWARD [20153]	
DY025153	1.489	moderately similar to (337)AT2G34980| Symbols: SETH1 | SETH1; transferase | chr2:14756046-14756957 FORWARD [18971]	
RC_ES979278	1.489	no similarity	
JCVI_35919	1.489	moderately similar to (323)AT1G44350| Symbols: ILL6 | ILL6 (IAA-leucine resistant (ILR)-like gene 6); metallopeptidase | chr1:1683718	
ES991347	1.489	very weakly similar to (85.9)AT1G78700| Symbols: | brassinosteroid signalling positive regulator-related | chr1:29604747-29606432 FORV	
EX092835	1.489	moderately similar to (259)AT5G11170| Symbols: | DEAD/DEAH box helicase, putative (RH15) | chr5:3553335-3556647 FORWARDver	1.714
JCVI_340	1.488	moderately similar to (372)AT2G35980| Symbols: NHL10, YLS9 | YLS9 (YELLOW-LEAF-SPECIFIC GENE 9) | chr2:15117714-151183	
JCVI_3923	1.488	moderately similar to (387)AT4G34180| Symbols: | cyclase family protein | chr4:16370064-16371387 REVERSE no original description	
JCVI_27935	1.488	moderately similar to (500)AT1G21450| Symbols: SCL1 | SCL1 (SCARECROW-LIKE 1); transcription factor | chr1:7509710-7511491 FC	
EV026769	1.488	moderately similar to (238)AT5G55730| Symbols: FLA1 | FLA1 | chr5:22575601-22577618 REVERSE [21441]	
ES953032	1.488	no similarity	
AM391561	1.488	no similarity	
EV226767	1.488	moderately similar to (222)AT1G19800| Symbols: TGD1 | TGD1 (TRIGALACTOSYLDIACYLGLYCEROL 1) | chr1:6846803-6847945	
JCVI_7734	1.488	moderately similar to (361)AT5G58090| Symbols: | glycosyl hydrolase family 17 protein | chr5:23522782-23524419 REVERSEweakly sir	
EE422801	1.488	moderately similar to (245)AT5G46760| Symbols: | basic helix-loop-helix (bHLH) family protein | chr5:18991458-18993236 FORWARD	-1.836
JCVI_32040	1.488	weakly similar to (128)AT3G16370| Symbols: | GDSL-motif lipase/hydrolase family protein | chr3:5556934-5558357 FORWARD no orig	
JCVI_4740	1.488	moderately similar to (275)AT3G23770| Symbols: | glycosyl hydrolase family 17 protein | chr3:8565563-8567203 FORWARD no original	
JCVI_16341	1.488	moderately similar to (452)AT2G44200| Symbols: | similar to unknown protein [Arabidopsis thaliana] (TAIR:AT2G44195.1); similar to h	
JCVI_5738	1.487	moderately similar to (337)AT3G27320| Symbols: | hydrolase | chr3:10091544-10093628 FORWARD no original description	
ES959175	1.487	moderately similar to (380)AT3G48500| Symbols: PDE312, PTAC10 | PDE312/PTAC10 (PIGMENT DEFECTIVE 312); RNA binding | c	
JCVI_14303	1.487	moderately similar to (448)AT1G06430| Symbols: FTSH8 | FTSH8 (FtsH protease 8); ATP-dependent peptidase/ ATPase/ metallopeptidas	
JCVI_31357	1.487	highly similar to (650)AT5G09660| Symbols: PMDH2 | PMDH2 (PEROXISOMAL NAD-MALATE DEHYDROGENASE 2) | chr5:29936	
JCVI_29244	1.487	highly similar to (588)AT1G19710| Symbols: | glycosyl transferase family 1 protein | chr1:6814911-6816707 FORWARD no original desc	
JCVI_2442	1.487	moderately similar to (297)AT5G26210| Symbols: | PHD finger family protein | chr5:9158569-9160224 REVERSE no original description	
JCVI_22605	1.487	weakly similar to (151)AT1G49990| Symbols: | Identical to F-box protein At1g49990 [Arabidopsis Thaliana] (GB:Q9LPM2); similar to F-	
JCVI_13485	1.486	moderately similar to (381)AT5G04720| Symbols: ADR1-L2 | ADR1-L2 (ADR1-LIKE 2); ATP binding / nucleoside-triphosphatase/ nucle	
EE531178	1.486	weakly similar to (112)AT3G07770| Symbols: | ATP binding | chr3:2479617-2483976 FORWARD [20175]	
RC_AT002097	1.486	no similarity	
JCVI_8688	1.486	no original description	
EX105137	1.486	weakly similar to (128)AT1G75770| Symbols: | unknown protein | chr1:28453683-28454632 FORWARD [21826]	
JCVI_9291	1.486	highly similar to (535)AT1G64660| Symbols: ATMGL | ATMGL; catalytic/ methionine gamma-lyase | chr1:24032640-24034200 FORWA	-2.777
ES914288	1.486	no similarity	
EE555669	1.486	no similarity	
JCVI_11527	1.486	very weakly similar to (92.8)AT1G07175| Symbols: | similar to unknown protein [Arabidopsis thaliana] (TAIR:AT2G29995.1) | chr1:2202	
JCVI_3978	1.486	moderately similar to (424)AT3G03600| Symbols: RPS2 | RPS2 (RIBOSOMAL PROTEIN S2); structural constituent of ribosome | chr3:8	
EX094196	1.486	no similarity	
DY007082	1.486	weakly similar to (156)AT4G38800| Symbols: ATMTN1 | ATMTN1; catalytic/ methylthioadenosine nucleosidase | chr4:18113349-18114	
JCVI_24013	1.486	moderately similar to (330)AT1G25480| Symbols: | similar to unknown protein [Arabidopsis thaliana] (TAIR:AT2G17470.1); similar to u	
JCVI_40186	1.486	weakly similar to (176)AT3G10050| Symbols: OMR1 | OMR1 (L-O-METHYLTHREONINE RESISTANT 1); L-threonine ammonia-lyase	
JCVI_11887	1.485	no original description	
JCVI_41409	1.485	weakly similar to (166)AT1G68570| Symbols: | proton-dependent oligopeptide transport (POT) family protein | chr1:25750474-25753773	
JCVI_14944	1.485	moderately similar to (241)AT5G46250| Symbols: | RNA recognition motif (RRM)-containing protein | chr5:18772615-18774950 FORW/	
JCVI_30906	1.485	weakly similar to (173)AT4G21610| Symbols: LOL2 | LOL2 (LSD ONE LIKE 2); transcription factor | chr4:11489081-11490312 FORWA	
EE562674	1.485	no similarity	
EV015674	1.485	moderately similar to (380)AT5G06940| Symbols: | leucine-rich repeat family protein | chr5:2148079-2150772 REVERSEweakly similar t	
JCVI_39025	1.485	moderately similar to (447)AT4G17430| Symbols: | similar to hypothetical protein 31.t00055 [Brassica oleracea] (GB:ABD65093.1) | chr4	
EE460615	1.485	no similarity	
EE404022	1.485	no similarity	
JCVI_394	1.485	moderately similar to (297)AT5G07020| Symbols: | proline-rich family protein | chr5:2180670-2182285 REVERSE no original description	
JCVI_16227	1.485	weakly similar to (196)AT2G33990| Symbols: IQD9 | IQD9 (IQ-domain 9); calmodulin binding | chr2:14367581-14368709 REVERSE no	
JCVI_23661	1.485	weakly similar to (140)AT3G62860| Symbols: | esterase/lipase/thioesterase family protein | chr3:23250552-23253118 REVERSE no origi	
JCVI_15880	1.484	moderately similar to (234)AT3G56290| Symbols: | similar to hypothetical protein [Vitis vinifera] (GB:CAN75527.1) | chr3:20889722-20	
JCVI_28388	1.484	moderately similar to (373)AT3G62240| Symbols: | zinc finger (C2H2 type) family protein | chr3:23044567-23047628 REVERSE no orig	
JCVI_26016	1.484	moderately similar to (306)AT2G39795| Symbols: | mitochondrial glycoprotein family protein / MAM33 family protein | chr2:16604104-1	
EV098831	1.484	weakly similar to (149)AT1G52230| Symbols: PSAH2, PSAH-2, PSI-H | PSAH-2/PSAH2/PSI-H (PHOTOSYSTEM I SUBUNIT H-2) | ch	
EE569744	1.484	no similarity	
JCVI_1613	1.483	weakly similar to (160)AT1G61730| Symbols: | DNA-binding storekeeper protein-related | chr1:22797112-22798242 REVERSE no origin	
JCVI_39041	1.483	very weakly similar to (94.7)AT5G13090| Symbols: | similar to unknown protein [Arabidopsis thaliana] (TAIR:AT1G24270.1); similar to u	
JCVI_36116	1.483	moderately similar to (382)AT5G09330| Symbols: ANAC082 | ANAC082 (Arabidopsis NAC domain containing protein 82) | chr5:289262	2.192
ES264648	1.483	no similarity	
JCVI_28081	1.483	no original description	
EE566920	1.483	no similarity	

JCVI_8049	1.483	moderately similar to (342)AT5G64670| Symbols: | ribosomal protein L15 family protein | chr5:25869761-25871106 REVERSE no origin	
EV221069	1.482	weakly similar to (195)AT3G05200| Symbols: ATL6 | ATL6 (Arabidopsis T?xicos en Levadura 6); protein binding / zinc ion binding | chr3	1.740
JCVI_10121	1.482	no original description	
JCVI_15695	1.482	highly similar to (888)AT5G08720| Symbols: | similar to unknown protein [Arabidopsis thaliana] (TAIR:AT4G01650.2); similar to unkno	
JCVI_28334	1.482	moderately similar to (454)AT1G30890| Symbols: | integral membrane HRF1 family protein | chr1:10994871-10995980 FORWARD no o	1.187
EX090952	1.482	no similarity	
JCVI_19691	1.482	moderately similar to (478)AT5G21222| Symbols: | protein kinase family protein | chr5:7209425-7213703 FORWARDweakly similar to (	
JCVI_16857	1.482	no original description	-1.367
JCVI_17687	1.482	moderately similar to (227)AT3G62410| Symbols: CP12, CP12-2 | CP12-2 | chr3:23101982-23102377 FORWARD no original description	-1.626
JCVI_9009	1.482	weakly similar to (142)AT5G59970| Symbols: | histone H4 | chr5:24163578-24163889 REVERSEweakly similar to (142)H4_PEA no orig	
EL588827	1.482	no similarity	
EL592413	1.482	weakly similar to (140)AT1G09710| Symbols: | DNA binding | chr1:3141121-3144234 FORWARD [20863]	
JCVI_29389	1.482	moderately similar to (282)AT1G23580| Symbols: | similar to unknown protein [Arabidopsis thaliana] (TAIR:AT1G23600.1); similar to u	
ES269621	1.482	moderately similar to (253)AT5G40870| Symbols: ATUK/UPRT1 | ATUK/UPRT1 (URIDINE KINASE/URACIL PHOSPHORIBOSYLT	
EX124732	1.481	moderately similar to (304)AT1G61560| Symbols: ATMLO6, MLO6 | MLO6 (MILDEW RESISTANCE LOCUS O 6); calmodulin binding	
JCVI_14594	1.481	moderately similar to (352)AT1G01430| Symbols: | similar to unknown protein [Arabidopsis thaliana] (TAIR:AT4G01080.1); similar to u	
EE550165	1.481	no similarity	
JCVI_19198	1.481	moderately similar to (233)AT4G29520| Symbols: | similar to unnamed protein product [Vitis vinifera] (GB:CAO65389.1); contains Inter	
AM389860	1.481	moderately similar to (265)AT2G43500| Symbols: | RWP-RK domain-containing protein | chr2:18069792-18073527 FORWARD [20118]	
ES911146	1.481	weakly similar to (151)AT1G20160| Symbols: ATSBT5.2 | ATSBT5.2; subtilase | chr1:6990843-6993728 REVERSE [21430] 25 769 769	
CB686317	1.481	no similarity	
JCVI_23537	1.481	moderately similar to (284)AT4G36400| Symbols: | FAD linked oxidase family protein | chr4:17197268-17200475 FORWARD no origina	
ES967164	1.481	no similarity	
RC_EV012775	1.481	no similarity	
EV183049	1.481	weakly similar to (127)AT1G64780| Symbols: ATAMT1;2 | ATAMT1;2 (AMMONIUM TRANSPORTER 1;2); ammonium transmembra	
JCVI_3494	1.480	moderately similar to (236)AT5G24314| Symbols: PDE225, PTAC7 | PDE225/PTAC7 (PIGMENT DEFECTIVE 225) | chr5:8277753-827	
JCVI_8347	1.480	moderately similar to (254)AT1G80040| Symbols: | similar to unknown protein [Arabidopsis thaliana] (TAIR:AT5G32440.1); similar to h	
JCVI_41855	1.480	moderately similar to (306)AT2G31350| Symbols: GLX2-5 | GLX2-5 (GLYOXALASE 2-5); hydroxyacylglutathione hydrolase/ iron ion b	
JCVI_3762	1.480	moderately similar to (400)AT2G14260| Symbols: PIP | PIP (proline iminopeptidase); prolyl aminopeptidase | chr2:6048523-6050557 REV	
EE413055	1.480	moderately similar to (278)AT1G77250| Symbols: | PHD finger family protein | chr1:29025338-29027832 REVERSE [20145]	
JCVI_26840	1.480	highly similar to (707)AT3G16200| Symbols: | similar to unnamed protein product [Vitis vinifera] (GB:CAO45864.1) | chr3:5491625-549	
EV057215	1.480	moderately similar to (293)AT3G28760| Symbols: | similar to unnamed protein product [Vitis vinifera] (GB:CAO14940.1); contains Inter	
EE546865	1.480	moderately similar to (206)AT5G67360| Symbols: ARA12 | ARA12; subtilase | chr5:26889418-26891691 REVERSE [20128] 41 365 365	-1.671
JCVI_6889	1.480	moderately similar to (233)AT4G31840| Symbols: | plastocyanin-like domain-containing protein | chr4:15401804-15402432 FORWARDv	
JCVI_26902	1.479	moderately similar to (266)AT3G51520| Symbols: | diacylglycerol acyltransferase family | chr3:19121718-19123497 FORWARD no origi	
EE547171	1.479	weakly similar to (133)AT3G14600| Symbols: | 60S ribosomal protein L18A (RPL18aC) | chr3:4910780-4911940 FORWARDweakly sim	
EE547794	1.479	moderately similar to (313)AT2G20810| Symbols: GAUT10, LGT4 | GAUT10/LGT4 (Galacturonosyltransferase 10); polygalacturonate 4-	
JCVI_5510	1.479	highly similar to (502)AT5G37510| Symbols: EMB1467 | EMB1467 (EMBRYO DEFECTIVE 1467); NADH dehydrogenase | chr5:14914	
EE444296	1.479	weakly similar to (175)AT1G45145| Symbols: ATH5, LIV1, ATTRX5 | ATTRX5 (thioredoxin H-type 5); thiol-disulfide exchange interme	
EV151302	1.478	no similarity	
EV184768	1.478	moderately similar to (230)AT3G07760| Symbols: | similar to unnamed protein product [Vitis vinifera] (GB:CAO41197.1); contains Inter	
EX041591	1.478	weakly similar to (185)AT1G04970| Symbols: | lipid-binding serum glycoprotein family protein | chr1:1411923-1413430 FORWARD [21	
JCVI_3517	1.478	highly similar to (578)AT5G63980| Symbols: ATSAL1, HOS2, FRY1, SAL1 | SAL1 (FIERY1); 3'(2'),5'-bisphosphate nucleotidase/ inositc	
EE518153	1.478	moderately similar to (290)AT5G20660| Symbols: | 24 kDa vacuolar protein, putative | chr5:6986404-6990949 FORWARD [20185]	
EE565800	1.477	no similarity	
JCVI_37263	1.477	highly similar to (675)AT5G57580| Symbols: | calmodulin-binding protein | chr5:23332220-23334909 REVERSE no original description	
JCVI_1709	1.477	moderately similar to (404)AT5G61210| Symbols: ATSNAP33, SNP33, ATSNAP33B, SNAP33 | SNAP33 (synaptosomal-associated prote	
JCVI_27233	1.477	moderately similar to (491)AT2G41020| Symbols: | WW domain-containing protein | chr2:17124503-17127979 FORWARD no original de	
JCVI_24327	1.477	highly similar to (957)AT1G54570| Symbols: | esterase/lipase/thioesterase family protein | chr1:20384316-20388620 REVERSE no origin	
JCVI_1790	1.477	moderately similar to (436)AT4G24890| Symbols: ATPAP24, PAP24 | ATPAP24/PAP24 (purple acid phosphatase 24); acid phosphatase/	1.870
JCVI_37080	1.477	weakly similar to (174)AT5G01960| Symbols: | zinc finger (C3HC4-type RING finger) family protein | chr5:370808-372772 FORWARD	
JCVI_26035	1.477	weakly similar to (199)AT1G11400| Symbols: PYM | PYM (PARTNER OF Y14-MAGO) | chr1:3838777-3839978 FORWARD no origina	
JCVI_17645	1.477	moderately similar to (478)AT1G32230| Symbols: CEO, ATP8, CEO1, RCD1 | RCD1 (RADICAL-INDUCED CELL DEATH1) | chr1:116	1.392
JCVI_3343	1.477	moderately similar to (234)AT2G04520| Symbols: | eukaryotic translation initiation factor 1A, putative / eIF-1A, putative / eIF-4C, putativ	
CD828412	1.477	moderately similar to (253)AT5G39450| Symbols: | F-box family protein | chr5:15803273-15805105 FORWARD [13979]	
CV546508	1.477	no similarity	
JCVI_1164	1.477	weakly similar to (182)AT3G15400| Symbols: ATA20 | ATA20 (Arabidopsis thaliana anther 20) | chr3:5202021-5202935 FORWARD no	
EE519322	1.476	no similarity	
JCVI_29801	1.476	no original description	
ES937982	1.476	moderately similar to (341)AT4G33090| Symbols: APM1, ATAPM1 | APM1 (AMINOPEPTIDASE M1) | chr4:15965918-15970421 REV	
DY020536	1.476	no similarity	2.746
JCVI_19682	1.476	moderately similar to (333)AT1G80560| Symbols: | 3-isopropylmalate dehydrogenase, chloroplast, putative | chr1:30292725-30295018 FC	
JCVI_23383	1.476	moderately similar to (209)AT5G19000| Symbols: ATBPM1 | ATBPM1 (BTB-POZ AND MATH DOMAIN 1); protein binding | chr5:634	
EE459650	1.476	weakly similar to (114)AT4G02350| Symbols: | exocyst complex subunit Sec15-like family protein | chr4:1038157-1040571 FORWARD [	
CN827569	1.476	moderately similar to (375)AT5G23720| Symbols: PHS1 | PHS1 (PROPYZAMIDE-HYPERSENSITIVE 1); protein tyrosine/serine/threon	1.551
JCVI_19143	1.475	no original description	
JCVI_41346	1.475	moderately similar to (276)AT1G48580| Symbols: | similar to unnamed protein product [Vitis vinifera] (GB:CAO67053.1) | chr1:1796207	
JCVI_10768	1.475	highly similar to (701)AT1G12330| Symbols: | similar to unknown protein [Arabidopsis thaliana] (TAIR:AT5G12900.1); similar to unnam	
JCVI_11377	1.475	no original description	
JCVI_14135	1.475	moderately similar to (465)AT5G26570| Symbols: ATGWD3, OK1, PWD | PWD (PHOSPHOGLUCAN WATER DIKINASE) | chr5:9261	
JCVI_20149	1.475	very weakly similar to (98.6)AT3G56400| Symbols: ATWRKY70, WRKY70 | WRKY70 (WRKY DNA-binding protein 70); transcription f	
EX101290	1.475	moderately similar to (446)AT3G58510| Symbols: | DEAD box RNA helicase, putative (RH11) | chr3:21651585-21654441 FORWARDm	
EE445077	1.475	no similarity	
ES269414	1.475	moderately similar to (243)AT1G28030| Symbols: | oxidoreductase, 2OG-Fe(II) oxygenase family protein | chr1:9771780-9773332 FORW	
ES912717	1.475	weakly similar to (118)AT1G21360| Symbols: GLTP2 | GLTP2 (GLYCOLIPID TRANSFER PROTEIN 2); glycolipid binding / glycolipid	
CV432327	1.475	no similarity	-1.861
AM385490	1.475	weakly similar to (197)AT5G42310| Symbols: | pentatricopeptide (PPR) repeat-containing protein | chr5:16933088-16935466 FORWARD	
JCVI_42059	1.475	no original description	
JCVI_11956	1.475	weakly similar to (183)AT1G26665| Symbols: | similar to RNA polymerase II mediator complex protein-related [Arabidopsis thaliana] (T	
ES919983	1.475	no similarity	
JCVI_15266	1.474	moderately similar to (386)AT2G27810| Symbols: | xanthine/uracil permease family protein | chr2:11859415-11862488 FORWARD no or	

EE402061	1.474	very weakly similar to (85.9)AT2G45220| Symbols: | pectinesterase family protein | chr2:18651355-18653468 REVERSE [20197] 1 250 4	
EE559834	1.474	no similarity	
EV131882	1.474	weakly similar to (116)AT5G19290| Symbols: | esterase/lipase/thioesterase family protein | chr5:6494115-6495107 FORWARD [21481]	
CD834526	1.474	weakly similar to (143)AT1G17665| Symbols: | similar to CA-responsive protein [Brassica oleracea] (GB:ABB83615.1) | chr1:6072591-6	
EE545378	1.474	moderately similar to (269)AT5G25760| Symbols: UBC21, PEX4 | PEX4 (PEROXIN4); ubiquitin-protein ligase | chr5:8967986-8969176	
EX078119	1.473	no similarity	
JCVI_14233	1.473	highly similar to (774)AT1G47670| Symbols: | amino acid transporter family protein | chr1:17539274-17541926 REVERSE no original de	
JCVI_5656	1.473	moderately similar to (483)AT5G61790| Symbols: | calnexin 1 (CNX1) | chr5:24844620-24846868 REVERSEmoderately similar to (413	
JCVI_27802	1.473	highly similar to (791)AT3G04260| Symbols: PTAC3 | PTAC3 (PLASTID TRANSCRIPTIONALLY ACTIVE3); DNA binding | chr3:112	
ES906144	1.473	moderately similar to (273)AT4G28610| Symbols: ATPHR1, PHR1 | PHR1 (PHOSPHATE STARVATION RESPONSE 1); transcription	
EV002384	1.473	moderately similar to (204)AT2G29070| Symbols: | ubiquitin fusion degradation UFD1 family protein | chr2:12495138-12496412 FORW	
JCVI_27645	1.473	no original description	
JCVI_24267	1.473	weakly similar to (115)AT1G43690| Symbols: | ubiquitin interaction motif-containing protein | chr1:16480959-16485029 FORWARD no	-1.042
EV166927	1.473	no similarity	
JCVI_34396	1.473	weakly similar to (167)AT5G04540| Symbols: | inositol or phosphatidylinositol phosphatase/ phosphoric monoester hydrolase/ protein tyr	
JCVI_32478	1.473	moderately similar to (206)AT3G03670| Symbols: | peroxidase, putative | chr3:901992-903356 REVERSEvery weakly similar to (89.7)PE	
ES901896	1.473	moderately similar to (282)AT3G23180| Symbols: | similar to lesion inducing protein-related [Arabidopsis thaliana] (TAIR:AT3G23190.1	
EE521217	1.473	no similarity	-1.578
CD834709	1.473	moderately similar to (207)AT3G22680| Symbols: | Identical to Uncharacterized protein At3g22680 [Arabidopsis Thaliana] (GB:Q9LUJ3	
RC_EL591641	1.473	no similarity	
JCVI_35844	1.472	weakly similar to (189)AT3G02990| Symbols: HSFA1E, ATHSFA1E | ATHSFA1E (Arabidopsis thaliana heat shock transcription factor A	
EX108444	1.472	weakly similar to (169)AT3G56290| Symbols: | similar to hypothetical protein [Vitis vinifera] (GB:CAN75527.1) | chr3:20889722-20890	
AM385174	1.472	weakly similar to (198)AT1G14370| Symbols: APK2A | APK2A (PROTEIN KINASE 2A); kinase | chr1:4915854-4917954 FORWARD [2	
JCVI_38989	1.472	moderately similar to (214)AT3G29130| Symbols: | similar to unknown [Populus trichocarpa] (GB:ABK93868.1); contains domain PTHR	
CX191033	1.472	very weakly similar to (98.6)AT3G13910| Symbols: | similar to unknown protein [Arabidopsis thaliana] (TAIR:AT2G19460.1); similar to	
EV096398	1.472	very weakly similar to (96.7)AT1G60995| Symbols: | similar to S3 self-incompatibility locus-linked pollen 3.15 protein [Petunia integrifoli	
JCVI_7167	1.471	moderately similar to (250)AT2G06925| Symbols: ATSPLA2-ALPHA, PLA2-ALPHA | ATSPLA2-ALPHA/PLA2-ALPHA (PHOSPHOLI	
JCVI_18190	1.471	moderately similar to (238)AT5G11950| Symbols: | Encodes a protein of unknown function. It has been crystallized and shown to be stru	
EV155440	1.471	very weakly similar to (87.0)AT2G16430| Symbols: PAP10, ATPAP10 | ATPAP10/PAP10; acid phosphatase/ protein serine/threonine pho	-2.802
ES966295	1.471	no similarity	
CV650911	1.471	very weakly similar to (99.8)AT3G18790| Symbols: | similar to hypothetical protein [Vitis vinifera] (GB:CAN60270.1); contains InterPro	
CD827392	1.471	moderately similar to (301)AT1G34270| Symbols: | exostosin family protein | chr1:12492549-12494492 REVERSE [13979]	
JCVI_7075	1.471	no original description	
JCVI_28503	1.471	moderately similar to (374)AT1G09060| Symbols: | transcription factor jumonji (jmjC) domain-containing protein | chr1:2921238-292525	
JCVI_39738	1.471	weakly similar to (189)AT5G65380| Symbols: | ripening-responsive protein, putative | chr5:26140467-26143578 REVERSE no original de	
JCVI_2396	1.470	highly similar to (549)AT5G46170| Symbols: | F-box family protein | chr5:18732865-18734052 REVERSE no original description	
JCVI_9746	1.470	moderately similar to (244)AT1G68190| Symbols: | zinc finger (B-box type) family protein | chr1:25563047-25564667 FORWARD no ori	-1.751
EE547845	1.470	moderately similar to (308)AT3G17830| Symbols: | DNAJ heat shock family protein | chr3:6101874-6104509 FORWARDvery weakly sin	
CX281629	1.470	no similarity	
JCVI_18671	1.470	moderately similar to (293)AT1G51540| Symbols: | similar to kelch repeat-containing protein [Arabidopsis thaliana] (TAIR:AT3G27220.	
JCVI_459	1.470	moderately similar to (446)AT2G32150| Symbols: | haloacid dehalogenase-like hydrolase family protein | chr2:13666172-13667608 FOR	
JCVI_16910	1.469	no original description	
JCVI_40786	1.469	no original description	-2.011
JCVI_39101	1.469	moderately similar to (235)AT4G32870| Symbols: | similar to unknown protein [Arabidopsis thaliana] (TAIR:AT2G25770.2); similar to u	
EX122023	1.469	no similarity	
JCVI_24449	1.469	highly similar to (564)AT2G22250| Symbols: ATAAT, AAT, MEE17 | AAT/ATAAT/MEE17 (maternal effect embryo arrest 17); aspartat	
EV204752	1.469	weakly similar to (160)AT5G10170| Symbols: | inositol-3-phosphate synthase, putative / myo-inositol-1-phosphate synthase, putative / MI	
JCVI_7931	1.469	moderately similar to (439)AT4G39480| Symbols: CYP96A9 | CYP96A9 (cytochrome P450, family 96, subfamily A, polypeptide 9); oxyg	
AM060610	1.469	moderately similar to (280)AT1G78815| Symbols: | similar to unknown protein [Arabidopsis thaliana] (TAIR:AT1G16910.1); similar to h	
EE440015	1.469	weakly similar to (113)AT5G11970| Symbols: | similar to unknown protein [Arabidopsis thaliana] (TAIR:AT2G19460.1); similar to unnai	
DY020183	1.469	weakly similar to (146)AT3G54650| Symbols: | F-box family protein (FBL17) | chr3:20236982-20239860 REVERSE [18974]	
EV058678	1.469	no similarity	
EV202349	1.468	weakly similar to (144)AT3G03790| Symbols: | ankyrin repeat family protein / regulator of chromosome condensation (RCC1) family pro	
H07323	1.468	no similarity	
JCVI_41431	1.468	no original description	
EV152608	1.468	moderately similar to (271)AT4G38130| Symbols: HDA19, ATHD1, HDA1, RPD3A | HD1 (HISTONE DEACETYLASE19) | chr4:17896	
JCVI_10642	1.468	moderately similar to (244)AT5G47370| Symbols: HAT2 | HAT2; transcription factor | chr5:19233709-19234874 REVERSE no original d	2.231
JCVI_2851	1.468	weakly similar to (172)AT5G64080| Symbols: | protease inhibitor/seed storage/lipid transfer protein (LTP) family protein | chr5:25662701	
JCVI_25929	1.468	weakly similar to (168)AT5G23610| Symbols: | similar to SWI1 (SWITCH1), phospholipase C [Arabidopsis thaliana] (TAIR:AT5G51330	
EE551684	1.468	no similarity	
EX021190	1.468	weakly similar to (156)AT1G14230| Symbols: | nucleoside phosphatase family protein / GDA1/CD39 family protein | chr1:4861492-4863	
JCVI_24037	1.467	highly similar to (524)AT5G16280| Symbols: | binding | chr5:5323380-5331348 REVERSE no original description	
JCVI_3537	1.467	weakly similar to (145)AT1G53645| Symbols: | hydroxyproline-rich glycoprotein family protein | chr1:20030102-20032255 REVERSE nc	
JCVI_28984	1.467	moderately similar to (381)AT2G21410| Symbols: VHA-A2 | VHA-A2 (VACUOLAR PROTON ATPASE A2); ATPase | chr2:9169783-91	
JCVI_5426	1.467	weakly similar to (178)AT5G59910| Symbols: HTB4 | HTB4; DNA binding | chr5:24144432-24144884 FORWARDweakly similar to (17	-1.513
DY026351	1.467	very weakly similar to (87.0)AT4G10810| Symbols: | similar to unknown protein [Arabidopsis thaliana] (TAIR:AT4G24026.1) | chr4:6645	
JCVI_3961	1.467	highly similar to (715)AT1G68010| Symbols: HPR | HPR (HYDROXYPYRUVATE REDUCTASE); glycerate dehydrogenase/ poly(U) bi	
ES986162	1.467	no similarity	
RC_AM386303	1.467	no similarity	
EX126353	1.466	moderately similar to (488)AT2G41830| Symbols: | cyclin-related | chr2:17457509-17463531 REVERSE [21831]	
EV221280	1.466	weakly similar to (157)AT1G27760| Symbols: | interferon-related developmental regulator family protein / IFRD protein family | chr1:966	
JCVI_25210	1.466	weakly similar to (198)AT5G62260| Symbols: | DNA binding | chr5:25026557-25028574 FORWARD no original description	
JCVI_39365	1.466	moderately similar to (391)AT5G27150| Symbols: ATNHX, AT-NHX1, ATNHX1, NHX1 | NHX1 (NA+/H+ EXCHANGER); sodium ion	
JCVI_25186	1.466	moderately similar to (205)AT1G51950| Symbols: IAA18 | IAA18 (indoleacetic acid-induced protein 18); transcription factor | chr1:19309	
CD821642	1.465	moderately similar to (220)AT3G17130| Symbols: | invertase/pectin methylesterase inhibitor family protein | chr3:5844501-5845052 REV	
JCVI_1817	1.465	moderately similar to (438)AT2G06050| Symbols: OPR3 | OPR3 (OPDA-REDUCTASE 3) | chr2:2359237-2361968 REVERSE no origina	
JCVI_30232	1.465	highly similar to (695)AT1G25280| Symbols: AtTLP10 | AtTLP10 (TUBBY LIKE PROTEIN 10) | chr1:8865663-8866595 FORWARD nc	
EE417439	1.465	moderately similar to (266)AT2G42620| Symbols: ORE9, MAX2 | MAX2 (MORE AXILLARY BRANCHES 2); ubiquitin-protein ligase |	
AM389692	1.465	weakly similar to (155)AT1G80850| Symbols: | methyladenine glycosylase family protein | chr1:30390499-30392164 REVERSE [20118]	
JCVI_28032	1.465	moderately similar to (400)AT4G09160| Symbols: | SEC14 cytosolic factor family protein / phosphoglyceride transfer family protein | chr	
AM396149	1.465	moderately similar to (219)AT3G14900| Symbols: | similar to unnamed protein product [Vitis vinifera] (GB:CAO39402.1); contains doma	

JCVI_15799	1.465	moderately similar to (364)AT2G32860| Symbols: | glycosyl hydrolase family 1 protein | chr2:13947310-13950673 FORWARDweakly sin	
EE433447	1.465	weakly similar to (132)AT4G18370| Symbols: DEG5, DEGP5, HHOA | DEG5/DEGP5/HHOA (DEGP PROTEASE 5); serine-type peptida	
EV128692	1.464	no similarity	
RC_ES268830	1.464	no similarity	
EE561800	1.464	no similarity	
JCVI_33223	1.464	no original description	
EX140350	1.464	no similarity	
JCVI_30017	1.464	no original description	
EX040816	1.464	very weakly similar to (85.1)AT5G24580| Symbols: | copper-binding family protein | chr5:8410397-8412090 REVERSE [21811]	
JCVI_17309	1.463	weakly similar to (174)AT3G23280| Symbols: | zinc finger (C3HC4-type RING finger) family protein / ankyrin repeat family protein | chr3	
JCVI_22216	1.463	very weakly similar to (90.5)AT1G64220| Symbols: TOM7-2 | TOM7-2 (TRANSLOCASE OF OUTER MEMBRANE 7 KDA SUBUNIT 2	
JCVI_18286	1.463	weakly similar to (107)AT3G57390| Symbols: AGL18 | AGL18 (AGAMOUS-LIKE 18); transcription factor | chr3:21244887-21246712 FO	
JCVI_32497	1.463	moderately similar to (207)AT1G12060| Symbols: ATBAG5 | ATBAG5 (ARABIDOPSIS THALIANA BCL-2-ASSOCIATED ATHANOG	
ES940620	1.463	no similarity	
EE438508	1.463	very weakly similar to (89.7)AT5G39720| Symbols: AIG2L | AIG2L (AVIRULENCE INDUCED GENE 2 LIKE PROTEIN) | chr5:159169	-1.481
JCVI_40472	1.463	moderately similar to (293)AT5G42560| Symbols: | abscisic acid-responsive HVA22 family protein | chr5:17032931-17034197 FORWAR	
JCVI_14495	1.463	moderately similar to (456)AT3G01090| Symbols: SNRK1.1, AKIN10 | AKIN10 (ARABIDOPSIS SNF1 KINASE HOMOLOG 10) | chr3:	
JCVI_32213	1.463	weakly similar to (199)AT5G64550| Symbols: | loricrin-related | chr5:25819020-25820924 REVERSE no original description	
EE475292	1.463	weakly similar to (146)AT4G02840| Symbols: | small nuclear ribonucleoprotein D1, putative / snRNP core protein D1, putative / Sm prote	
JCVI_15839	1.463	moderately similar to (365)AT4G14950| Symbols: | similar to unknown protein [Arabidopsis thaliana] (TAIR:AT1G05360.1); similar to u	
ES269456	1.462	no similarity	
EX125779	1.462	moderately similar to (201)AT1G75280| Symbols: | isoflavone reductase, putative | chr1:28255691-28257016 FORWARDweakly similar t	
JCVI_12681	1.462	moderately similar to (277)AT5G26280| Symbols: | meprin and TRAF homology domain-containing protein / MATH domain-containing p	
JCVI_39468	1.462	moderately similar to (276)AT4G36820| Symbols: | transcription factor | chr4:17346893-17347998 FORWARD no original description	
JCVI_11852	1.462	moderately similar to (335)AT1G80360| Symbols: | aminotransferase class I and II family protein | chr1:30213628-30215535 REVERSE n	
JCVI_38615	1.462	no original description	
JCVI_4650	1.462	moderately similar to (459)AT4G31500| Symbols: SUR2, RNT1, RED1, ATR4, CYP83B1 | CYP83B1 (CYTOCHROME P450 MONOOX	1.072
CD834983	1.462	weakly similar to (166)AT3G61900| Symbols: | auxin-responsive family protein | chr3:22936788-22937354 FORWARD [13981] 14 697 6	
JCVI_13584	1.462	weakly similar to (164)AT1G31175| Symbols: | similar to unnamed protein product [Vitis vinifera] (GB:CAO41995.1) | chr1:11140530-1	
AM391914	1.462	no similarity	
JCVI_13994	1.461	moderately similar to (349)AT1G01090| Symbols: PDH-E1 ALPHA | PDH-E1 ALPHA (PYRUVATE DEHYDROGENASE E1 ALPHA);	
JCVI_28372	1.461	highly similar to (528)AT5G63610| Symbols: CDKE;1, HEN3 | HEN3 (HUA ENHANCER 3); kinase | chr5:25480871-25482283 REVERS	
JCVI_4165	1.461	weakly similar to (198)AT4G16100| Symbols: | similar to unknown protein [Arabidopsis thaliana] (TAIR:AT5G49220.1); similar to unnar	
JCVI_24097	1.461	moderately similar to (348)AT2G36070| Symbols: ATTIM44-2 | ATTIM44-2 (Arabidopsis thaliana translocase inner membrane subunit 4	
JCVI_42584	1.461	no original description	
JCVI_670	1.461	moderately similar to (468)AT5G43860| Symbols: ATCLH2 | ATCLH2 (Chlorophyll-chlorophyllido hydrolase 2) | chr5:17647719-176494	
JCVI_16597	1.461	moderately similar to (391)AT5G45510| Symbols: | leucine-rich repeat family protein | chr5:18462025-18466179 FORWARD no original	
JCVI_40762	1.461	moderately similar to (224)AT4G39540| Symbols: | shikimate kinase family protein | chr4:18378555-18380247 FORWARD no original de	-3.223
EV197938	1.461	weakly similar to (141)AT1G72610| Symbols: GLP1 | GLP1 (GERMIN-LIKE PROTEIN 1); manganese ion binding / metal ion binding / n	
EE448262	1.461	weakly similar to (124)AT5G08650| Symbols: | GTP-binding protein LepA, putative | chr5:2806534-2813221 REVERSE [20172]	
JCVI_12755	1.460	highly similar to (568)AT1G17210| Symbols: | zinc ion binding | chr1:5880495-5884650 REVERSE no original description	
JCVI_3891	1.460	moderately similar to (360)AT1G61870| Symbols: PPR336 | PPR336 (PENTATRICOPEPTIDE REPEAT 336) | chr1:22868991-2287021	
JCVI_13453	1.460	moderately similar to (263)AT1G53510| Symbols: ATMPK18 | ATMPK18 (ARABIDOPSIS THALIANA MAP KINASE 18); MAP kinas	
CN730955	1.460	very weakly similar to (99.8)AT2G17972| Symbols: | similar to unnamed protein product [Vitis vinifera] (GB:CAO48225.1) | chr2:782870	
CV545235	1.460	very weakly similar to (94.7)AT3G11250| Symbols: | 60S acidic ribosomal protein P0 (RPP0C) | chr3:3521459-3522832 FORWARDvery	
JCVI_11825	1.460	weakly similar to (196)AT1G07180| Symbols: ATNDI1, NDA1 | ATNDI1/NDA1 (ALTERNATIVE NAD(P)H DEHYDROGENASE 1); N	
JCVI_20653	1.460	highly similar to (514)AT3G03780| Symbols: AtMS2 | AtMS2 (Arabidopsis thaliana methionine synthase 2) | chr3:957609-960747 FORW	
ES944054	1.459	no similarity	
EX058390	1.459	moderately similar to (243)AT1G31500| Symbols: | endonuclease/exonuclease/phosphatase family protein | chr1:11273802-11276515 RE	
JCVI_9538	1.459	weakly similar to (140)AT1G68140| Symbols: | similar to protein binding / zinc ion binding [Arabidopsis thaliana] (TAIR:AT4G08460.1);	
EX135194	1.459	moderately similar to (203)AT1G54010| Symbols: | myrosinase-associated protein, putative | chr1:20162522-20164415 REVERSE [2183	
JCVI_17608	1.459	moderately similar to (299)AT5G52980| Symbols: | similar to unnamed protein product [Vitis vinifera] (GB:CAO63390.1) | chr5:2149955	-1.772
CN730643	1.459	moderately similar to (266)AT3G29390| Symbols: RIK | RIK (RS2-INTERACTING KH PROTEIN) | chr3:11290912-11295761 FORWAI	1.263
JCVI_38630	1.459	moderately similar to (341)AT3G43240| Symbols: | ARID/BRIGHT DNA-binding domain-containing protein | chr3:15221020-15225428	
JCVI_18150	1.459	no original description	
EE538741	1.459	weakly similar to (107)AT1G11440| Symbols: | similar to glycine-rich protein [Arabidopsis thaliana] (TAIR:AT3G29075.1); similar to hy	
JCVI_39166	1.459	no original description	
JCVI_28858	1.458	moderately similar to (372)AT4G13330| Symbols: | similar to unnamed protein product [Vitis vinifera] (GB:CAO71472.1); contains doma	
JCVI_23810	1.458	weakly similar to (173)AT5G04250| Symbols: | OTU-like cysteine protease family protein | chr5:1176398-1178493 FORWARD no origin	
ES942683	1.458	no similarity	
EV215023	1.458	weakly similar to (200)AT2G45500| Symbols: | similar to ATPase [Arabidopsis thaliana] (TAIR:AT3G27120.1); similar to Tobacco mosa	
AM060462	1.458	weakly similar to (171)AT1G75150| Symbols: | similar to unnamed protein product [Vitis vinifera] (GB:CAO61179.1) | chr1:28208370-2	
DN962631	1.458	weakly similar to (163)AT1G80840| Symbols: ATWRKY40, WRKY40 | WRKY40 (WRKY DNA-binding protein 40); transcription factor	
EV107636	1.458	very weakly similar to (95.9)AT3G10230| Symbols: LYC | LYC (LYCOPENE CYCLASE) | chr3:3164345-3165454 REVERSEvery weakly	
EE457309	1.458	weakly similar to (162)AT5G18800| Symbols: | NADH-ubiquinone oxidoreductase 19 kDa subunit (NDUFA8) family protein | chr5:6267	
EX082684	1.458	moderately similar to (257)AT4G29510| Symbols: ATPRMT11, PRMT11 | ATPRMT11/PRMT11 (ARABIDOPSIS ARGININE METHY	
JCVI_39384	1.458	weakly similar to (131)AT1G15890| Symbols: | disease resistance protein (CC-NBS-LRR class), putative | chr1:5461400-5463955 FORW	
JCVI_41734	1.457	weakly similar to (140)AT3G18690| Symbols: MKS1 | MKS1 (MAP KINASE SUBSTRATE 1) | chr3:6429761-6430429 REVERSE no or	
EE534109	1.457	weakly similar to (165)AT5G11070| Symbols: | similar to unknown protein [Arabidopsis thaliana] (TAIR:AT5G35090.1) | chr5:3516441-	
EX115382	1.457	weakly similar to (144)AT5G53340| Symbols: | galactosyltransferase family protein | chr5:21658271-21660421 REVERSE [21835]	
EX086157	1.457	moderately similar to (345)AT1G61210| Symbols: | WD-40 repeat family protein / katanin p80 subunit, putative | chr1:22568450-2257522	
JCVI_38306	1.457	weakly similar to (147)AT2G24360| Symbols: | serine/threonine/tyrosine kinase, putative | chr2:10371822-10373155 REVERSE no origin	
DY018995	1.457	weakly similar to (134)AT3G09180| Symbols: | similar to unnamed protein product [Vitis vinifera] (GB:CAO45433.1); contains domain F	
JCVI_4183	1.457	moderately similar to (387)AT2G41760| Symbols: | similar to unnamed protein product [Vitis vinifera] (GB:CAO23603.1); contains doma	
JCVI_8758	1.457	moderately similar to (358)AT1G77280| Symbols: | protein kinase family protein | chr1:29036362-29040776 REVERSEvery weakly simil	-4.574
JCVI_8218	1.457	moderately similar to (359)AT4G15960| Symbols: | epoxide hydrolase, putative | chr4:9045777-9047213 REVERSE no original descriptic	
ES911470	1.457	no similarity	
AM056852	1.457	no similarity	
JCVI_33996	1.457	highly similar to (550)AT1G77630| Symbols: | peptidoglycan-binding LysM domain-containing protein | chr1:29178620-29180281 FORW	
EX056793	1.457	moderately similar to (206)AT1G60690| Symbols: | aldo/keto reductase family protein | chr1:22353557-22355333 REVERSEweakly simil	
JCVI_18397	1.457	moderately similar to (429)AT4G00700| Symbols: | C2 domain-containing protein | chr4:286260-289369 FORWARD no original descripti	

CD813531	1.457	weakly similar to (158)AT1G06870| Symbols: | signal peptidase, putative | chr1:2108831-2110641 FORWARD [13977]	
ES964499	1.456	no similarity	
JCVI_24697	1.456	highly similar to (576)AT4G10180| Symbols: FUS2, DET1 | DET1 (DE-ETIOLATED 1); catalytic | chr4:6346539-6349142 FORWARD n	1.461
AM395006	1.456	no similarity	
EV215181	1.456	moderately similar to (306)AT5G10490| Symbols: MSL2 | MSL2 (MSCS-LIKE 2) | chr5:3300377-3303837 REVERSE [21491] 30 576 74	
EV029026	1.456	moderately similar to (228)AT3G60150| Symbols: | similar to unknown protein [Arabidopsis thaliana] (TAIR:AT2G44525.1); similar to u	
EV153501	1.456	moderately similar to (352)AT5G01400| Symbols: ESP4 | ESP4 (ENHANCED SILENCING PHENOTYPE 4); binding | chr5:162802-171	-1.153
JCVI_25579	1.456	moderately similar to (410)AT1G18370| Symbols: HIK | HIK (HINKEL); microtubule motor | chr1:6319725-6323813 REVERSE no origi	
EX099834	1.455	moderately similar to (258)AT2G20680| Symbols: | glycosyl hydrolase family 5 protein / cellulase family protein | chr2:8928105-8930147	
JCVI_3666	1.455	moderately similar to (310)AT1G16180| Symbols: | TMS membrane family protein / tumour differentially expressed (TDE) family protein	
ES900791	1.455	moderately similar to (449)AT1G19630| Symbols: CYP722A1 | CYP722A1 (cytochrome P450, family 722, subfamily A, polypeptide 1); c	
JCVI_28620	1.455	weakly similar to (166)AT2G43810| Symbols: | small nuclear ribonucleoprotein F, putative / U6 snRNA-associated Sm-like protein, putati	
EX048204	1.455	weakly similar to (122)AT2G18328| Symbols: | DNA binding | chr2:7971560-7971793 FORWARD [21812]	
JCVI_36734	1.455	weakly similar to (185)AT5G46170| Symbols: | F-box family protein | chr5:18732865-18734052 REVERSE no original description	1.311
JCVI_41807	1.455	weakly similar to (169)AT1G10470| Symbols: MEE7, ATRR1, IBC7, ARR4 | ARR4 (RESPONSE REGULATOR 4); transcription regulat	1.535
JCVI_2308	1.455	weakly similar to (197)AT1G18080| Symbols: ATARCA | ATARCA (Arabidopsis thaliana Homolog of the Tobacco ArcA); nucleotide bin	
CD819976	1.455	moderately similar to (260)AT3G11950| Symbols: | homogentisate farnesyltransferase/ homogentisate geranylgeranyltransferase/ homoge	
JCVI_7176	1.455	moderately similar to (322)AT1G54650| Symbols: | similar to unknown protein [Arabidopsis thaliana] (TAIR:AT2G26200.1); similar to u	
ES967692	1.455	no similarity	
JCVI_19122	1.455	moderately similar to (352)AT4G16660| Symbols: | heat shock protein 70, putative / HSP70, putative | chr4:9377247-9381254 FORWARI	
EV089704	1.455	weakly similar to (127)AT5G18880| Symbols: | glucose transmembrane transporter | chr5:6300537-6301424 REVERSE [21444]	
JCVI_16419	1.455	moderately similar to (350)AT5G66900| Symbols: | disease resistance protein (CC-NBS-LRR class), putative | chr5:26732157-26734983 I	
JCVI_5143	1.455	no original description	
EX126322	1.455	moderately similar to (245)AT5G48680| Symbols: | sterile alpha motif (SAM) domain-containing protein | chr5:19761951-19763279 REV	
JCVI_8325	1.455	moderately similar to (260)AT5G28540| Symbols: BIP1 | BIP1; ATP binding | chr5:10540669-10543278 REVERSEmoderately similar to	
EV061005	1.455	no similarity	
EV020696	1.455	moderately similar to (243)AT3G15390| Symbols: SDE5 | SDE5 (SILENCING DEFECTIVE 5) | chr3:5196696-5199538 REVERSE [214	
JCVI_6912	1.455	no original description	
JCVI_19385	1.454	no original description	
DY013446	1.454	weakly similar to (110)AT5G66450| Symbols: | phosphatidic acid phosphatase-related / PAP2-related | chr5:26552204-26553177 FORWA	
EX128240	1.454	weakly similar to (152)AT5G57520| Symbols: ZFP2 | ZFP2 (ZINC FINGER PROTEIN 2); nucleic acid binding / transcription factor/ zinc	3.292
JCVI_4817	1.454	no original description	
CD826639	1.454	moderately similar to (220)AT4G31340| Symbols: | myosin heavy chain-related | chr4:15205668-15208901 FORWARD [13979]	
EV086178	1.454	no similarity	
JCVI_25465	1.454	moderately similar to (237)AT5G19070| Symbols: | similar to unknown protein [Arabidopsis thaliana] (TAIR:AT1G03260.1); similar to u	
EX097480	1.454	moderately similar to (386)AT1G05180| Symbols: AXR1 | AXR1 (AUXIN RESISTANT 1); small protein activating enzyme | chr1:14985	
JCVI_7819	1.454	moderately similar to (307)AT5G64240| Symbols: ATMC3 | ATMC3 (METACASPASE 3); caspase | chr5:25713062-25714475 FORWAI	
RC_EE561897	1.454	no similarity	
JCVI_12429	1.454	weakly similar to (189)AT2G19270| Symbols: | similar to unnamed protein product [Vitis vinifera] (GB:CAO69581.1) | chr2:8367431-83	-1.365
EV092752	1.454	weakly similar to (189)AT1G29450| Symbols: | auxin-responsive protein, putative | chr1:10305967-10306392 REVERSE [21476] 47 621	
JCVI_34826	1.454	moderately similar to (229)AT4G14147| Symbols: ARPC4 | ARPC4 (actin-related protein C4) | chr4:8154074-8156241 REVERSE no orig	
JCVI_15341	1.453	moderately similar to (348)AT5G15870| Symbols: | glycosyl hydrolase family 81 protein | chr5:5182644-5184881 REVERSE no original	
JCVI_29426	1.453	highly similar to (628)AT2G18790| Symbols: HY3, OOP1, PHYB | PHYB (PHYTOCHROME B); G-protein coupled photoreceptor/ signa	
JCVI_10240	1.453	moderately similar to (467)AT1G08620| Symbols: | transcription factor jumonji (jmj) family protein / zinc finger (C5HC2 type) family pro	
JCVI_328	1.453	highly similar to (510)AT1G71695| Symbols: | peroxidase 12 (PER12) (P12) (PRXR6) | chr1:26968021-26970219 FORWARDmoderately	2.212
EX127670	1.453	moderately similar to (221)AT5G03540| Symbols: ATEXO70A1 | ATEXO70A1 (EXOCYST SUBUNIT EXO70 FAMILY PROTEIN A1)	
JCVI_12747	1.453	highly similar to (547)AT4G26900| Symbols: HISHF, HISN4, AT-HF | AT-HF (ARABIDOPSIS THALIANA HISF PROTEIN) | chr4:135	
JCVI_29221	1.452	no original description	
EE509306	1.452	no similarity	
JCVI_31095	1.452	moderately similar to (410)AT5G15450| Symbols: APG6, CLPB3, CLPB-P | APG6/CLPB-P/CLPB3 (ALBINO AND PALE GREEN 6); A	
EE568472	1.452	no similarity	
JCVI_16608	1.452	weakly similar to (191)AT5G62990| Symbols: EMB1692 | EMB1692 (EMBRYO DEFECTIVE 1692) | chr5:25295054-25296538 FORWA	
JCVI_31738	1.451	no original description	
JCVI_26110	1.451	moderately similar to (337)AT1G61870| Symbols: PPR336 | PPR336 (PENTATRICOPEPTIDE REPEAT 336) | chr1:22868991-2287021	
DY024912	1.451	weakly similar to (163)AT1G77400| Symbols: | similar to hydroxyproline-rich glycoprotein family protein [Arabidopsis thaliana] (TAIR:A	
JCVI_7009	1.451	moderately similar to (208)AT3G16140| Symbols: PSAH-1 | PSAH-1 (photosystem I subunit H-1) | chr3:5468676-5469421 REVERSEmo	
JCVI_16440	1.451	moderately similar to (394)AT4G26270| Symbols: | phosphofructokinase family protein | chr4:13301103-13304039 REVERSE no origina	
EV217349	1.451	weakly similar to (167)AT1G14240| Symbols: | nucleoside phosphatase family protein / GDA1/CD39 family protein | chr1:4865154-4867	
JCVI_26410	1.450	moderately similar to (395)AT4G19160| Symbols: | binding | chr4:10477532-10479958 FORWARD no original description	
CV545468	1.450	weakly similar to (126)AT4G17730| Symbols: ATSYP23, SYP23 | SYP23 (syntaxin 23) | chr4:9865364-9866730 FORWARD [16551]	
EX118764	1.450	weakly similar to (125)AT5G64150| Symbols: | methylase family protein | chr5:25686223-25687957 REVERSE [21828]	
EV135311	1.450	no similarity	
JCVI_14571	1.450	moderately similar to (465)AT2G14835| Symbols: | zinc finger (C3HC4-type RING finger) family protein | chr2:6374320-6377692 FORW	
JCVI_20111	1.450	very weakly similar to (100)AT5G41685| Symbols: | mitochondrial import receptor subunit TOM7 / translocase of outer membrane 7 kDa	
JCVI_6732	1.450	moderately similar to (337)AT1G14010| Symbols: | emp24/gp25L/p24 family protein | chr1:4800382-4801787 REVERSE no original desc	
JCVI_32580	1.450	moderately similar to (442)AT4G24220| Symbols: AWI31, VEP1 | VEP1 (VEIN PATTERNING 1); binding / catalytic | chr4:12565229-1	-1.865
EE530852	1.450	no similarity	
AM057167	1.450	moderately similar to (269)AT1G33990| Symbols: | hydrolase, alpha/beta fold family protein | chr1:12355889-12357874 FORWARD [17	
JCVI_38732	1.450	weakly similar to (191)AT1G68100| Symbols: IAR1 | IAR1 (IAA-ALANINE RESISTANT 1); metal ion transmembrane transporter | chr1	
EX127401	1.450	moderately similar to (404)AT2G34960| Symbols: CAT5 | CAT5 (CATIONIC AMINO ACID TRANSPORTER 5); cationic amino acid tr	
JCVI_21355	1.450	weakly similar to (125)AT2G33150| Symbols: PED1, KAT2 | KAT2/PED1 (PEROXISOME DEFECTIVE 1); acetyl-CoA C-acyltransfera	
EX140431	1.450	no similarity	
DY000857	1.450	weakly similar to (107)AT5G48490| Symbols: | protease inhibitor/seed storage/lipid transfer protein (LTP) family protein | chr5:1966515	
JCVI_29886	1.449	weakly similar to (172)AT1G15880| Symbols: ATGOS11, GOS11 | GOS11 (GOLGI SNARE 11); SNARE binding | chr1:5458712-546008	
JCVI_21280	1.449	moderately similar to (351)AT1G68470| Symbols: | exostosin family protein | chr1:25680208-25681930 REVERSEweakly similar to (13	
JCVI_20378	1.449	moderately similar to (378)AT4G20910| Symbols: CRM2, HEN1 | HEN1 (HUA ENHANCER 1) | chr4:11186275-11190586 REVERSE n	
JCVI_41819	1.449	moderately similar to (446)AT5G22480| Symbols: | zinc finger (ZPR1-type) family protein | chr5:7451646-7456171 REVERSE no origina	
JCVI_17731	1.449	moderately similar to (210)AT5G40190| Symbols: | calmodulin-binding protein | chr5:16086526-16087077 REVERSE no original descrip	
ES904326	1.448	moderately similar to (457)AT2G36670| Symbols: | aspartyl protease family protein | chr2:15372028-15375095 REVERSEvery weakly si	
JCVI_10292	1.448	moderately similar to (238)AT1G70830| Symbols: MLP28 | MLP28 (MLP-LIKE PROTEIN 28) | chr1:26713865-26715057 REVERSE no	
CD825166	1.448	weakly similar to (179)AT5G19280| Symbols: RAG1, KAPP | KAPP (Kinase-associated protein phosphatase); protein serine/threonine ph	

CD829687	1.448	no similarity	
JCVI_39995	1.448	moderately similar to (268)AT1G06660| Symbols: | similar to unknown protein [Arabidopsis thaliana] (TAIR:AT2G30820.1); similar to u	
JCVI_10277	1.448	moderately similar to (302)AT5G47580| Symbols: | similar to unknown protein [Arabidopsis thaliana] (TAIR:AT4G17250.1); similar to h	
EV214117	1.448	moderately similar to (223)AT2G47460| Symbols: MYB12, ATMYB12 | ATMYB12/MYB12 (MYB DOMAIN PROTEIN 12); DNA bind	
RC_EX130747	1.448	no similarity	-1.838
EV091707	1.448	weakly similar to (175)AT2G40400| Symbols: | similar to unknown protein [Arabidopsis thaliana] (TAIR:AT3G56140.1); similar to hypot	
JCVI_36387	1.448	no original description	
AM395183	1.448	very weakly similar to (99.0)AT3G10390| Symbols: FLD | FLD (FLOWERING LOCUS D); amine oxidase | chr3:3229298-3231824 FORW	
JCVI_11808	1.447	highly similar to (511)AT2G28470| Symbols: BGAL8 | BGAL8 (BETA-GALACTOSIDASE 8); beta-galactosidase | chr2:12176124-12180	
EV159431	1.447	no similarity	
JCVI_28185	1.447	no original description	
EX122309	1.447	moderately similar to (266)AT5G56360| Symbols: | calmodulin-binding protein | chr5:22840812-22845176 REVERSE [21830]	
JCVI_29601	1.447	highly similar to (796)AT1G26850| Symbols: | dehydration-responsive family protein | chr1:9301133-9303419 REVERSE no original desc	
JCVI_7119	1.447	very weakly similar to (87.0)AT5G49940| Symbols: ATCNFU2, NFU2 | NFU2 (NFU domain protein 2) | chr5:20332690-20334293 FORW	
JCVI_25063	1.447	very weakly similar to (97.4)AT1G74250| Symbols: | DNAJ heat shock N-terminal domain-containing protein | chr1:27923989-27926075 I	
JCVI_9125	1.447	weakly similar to (181)AT1G53210| Symbols: | sodium/calcium exchanger family protein / calcium-binding EF hand family protein | chr1:	
JCVI_40378	1.447	moderately similar to (297)AT4G24450| Symbols: PWD, GWD3, ATGWD2 | ATGWD2/GWD3/PWD (PHOSPHOGLUCAN, WATER D	
JCVI_18100	1.447	moderately similar to (345)AT1G04710| Symbols: | acetyl-CoA C-acyltransferase, putative / 3-ketoacyl-CoA thiolase, putative | chr1:1321	
EV214083	1.447	no similarity	-1.170
JCVI_27162	1.447	moderately similar to (399)AT3G60260| Symbols: | phagocytosis and cell motility protein ELMO1-related | chr3:22285278-22287814 FOI	
JCVI_18545	1.447	weakly similar to (120)AT1G33500| Symbols: | similar to kinase interacting family protein [Arabidopsis thaliana] (TAIR:AT3G22790.1);	
EE558727	1.447	no similarity	
EV047324	1.447	moderately similar to (384)AT1G01960| Symbols: EDA10 | EDA10 (embryo sac development arrest 10); guanyl-nucleotide exchange facto	
JCVI_1510	1.447	weakly similar to (146)AT5G11070| Symbols: | similar to unknown protein [Arabidopsis thaliana] (TAIR:AT5G35090.1) | chr5:3516441-3	
JCVI_5899	1.446	moderately similar to (350)AT2G21170| Symbols: TIM | TIM (TRIOSEPHOSPHATE ISOMERASE) | chr2:9078128-9080187 REVERSE	
JCVI_27452	1.446	moderately similar to (329)AT1G02850| Symbols: | glycosyl hydrolase family 1 protein | chr1:630569-633085 FORWARDweakly similar	
DY026662	1.446	weakly similar to (142)AT4G10400| Symbols: | F-box family protein | chr4:6446331-6447711 REVERSE [18978]	
EX105200	1.446	very weakly similar to (93.2)AT1G15130| Symbols: | hydroxyproline-rich glycoprotein family protein | chr1:5206212-5209843 REVERSE	1.283
EX045099	1.446	weakly similar to (130)AT1G27000| Symbols: | bZIP family transcription factor | chr1:9374055-9376409 FORWARD [21811]	
JCVI_16768	1.446	highly similar to (704)AT3G56640| Symbols: | exocyst complex subunit Sec15-like family protein | chr3:20992946-20995315 FORWARD	
EE532480	1.446	moderately similar to (290)AT1G22650| Symbols: | beta-fructofuranosidase, putative / invertase, putative / saccharase, putative / beta-fruc	
JCVI_21013	1.446	highly similar to (550)AT5G40030| Symbols: | protein kinase, putative | chr5:16043455-16045511 FORWARDmoderately similar to (419	
JCVI_25203	1.446	highly similar to (544)AT4G11800| Symbols: | protein serine/threonine phosphatase | chr4:7093660-7098513 REVERSE no original descr	
EE426817	1.446	no similarity	
EX133140	1.446	weakly similar to (114)AT5G43490| Symbols: | similar to unnamed protein product [Vitis vinifera] (GB:CAO43512.1) | chr5:17485699-17	
JCVI_22767	1.445	moderately similar to (370)AT1G05810| Symbols: ARA, Ara-1, AtRab11D, AtRABA5e | ARA/Ara-1/AtRABA5e/AtRab11D (Arabidopsis	
DY014203	1.445	weakly similar to (158)AT5G59980| Symbols: | RNase P subunit p30 family protein | chr5:24164433-24166618 FORWARD [18966]	
JCVI_40602	1.445	no original description	
EV093374	1.445	weakly similar to (167)AT3G09010| Symbols: | protein kinase family protein | chr3:2750290-2752091 FORWARD [21476] 44 888 888	
EX085872	1.445	weakly similar to (135)AT3G08880| Symbols: | similar to unknown protein [Arabidopsis thaliana] (TAIR:AT5G01570.1); similar to unkno	
EX090309	1.445	weakly similar to (198)AT1G61260| Symbols: | similar to unknown protein [Arabidopsis thaliana] (TAIR:AT1G11220.1); similar to unkno	1.410
JCVI_24250	1.445	moderately similar to (312)AT3G07400| Symbols: | lipase class 3 family protein | chr3:2367462-2372244 FORWARD no original descript	
JCVI_30769	1.445	moderately similar to (262)AT5G67250| Symbols: VFB4, SKIP2 | SKIP2 (SKP1 INTERACTING PARTNER 2); ubiquitin-protein ligase |	
JCVI_34362	1.445	highly similar to (511)AT5G13000| Symbols: GSL12, ATGSL12 | ATGSL12 (GLUCAN SYNTHASE-LIKE 12); 1,3-beta-glucan synthase	
JCVI_6815	1.445	weakly similar to (179)AT4G21980| Symbols: ATG8A, APG8A | APG8A (autophagy 8A) | chr4:11655880-11656821 FORWARD no orig	
JCVI_40703	1.445	moderately similar to (300)AT3G24090| Symbols: | transaminase | chr3:8697845-8700978 REVERSE no original description	-2.120
CD825118	1.445	moderately similar to (260)AT3G48420| Symbols: | haloacid dehalogenase-like hydrolase family protein | chr3:17940728-17942536 FORV	
RC_T18365	1.445	no similarity	
JCVI_7279	1.445	moderately similar to (412)AT2G46225| Symbols: ABIL1 | ABIL1 (ABI-1-LIKE 1) | chr2:18989186-18991147 FORWARDmoderately sin	3.444
EX068024	1.445	no similarity	
JCVI_2866	1.445	moderately similar to (327)AT5G05990| Symbols: | mitochondrial glycoprotein family protein / MAM33 family protein | chr5:1806912-18	
JCVI_35068	1.444	moderately similar to (468)AT1G67750| Symbols: | pectate lyase family protein | chr1:25405323-25406828 FORWARDmoderately simila	
EE520014	1.444	moderately similar to (317)AT1G71696| Symbols: SOL1 | SOL1 (suppressor of LLP1 1); carboxypeptidase A | chr1:26970658-26974026 I	
EV018919	1.444	moderately similar to (362)AT1G79020| Symbols: | transcription factor-related | chr1:29732175-29734952 REVERSE [21441]	
JCVI_11111	1.444	moderately similar to (291)AT2G43950| Symbols: OEP37 | OEP37 | chr2:18207906-18209720 REVERSE no original description	
ES936709	1.444	moderately similar to (377)AT2G36200| Symbols: | kinesin motor protein-related | chr2:15186818-15192268 REVERSEmoderately simila	
EV012388	1.444	no similarity	
ES955258	1.444	no similarity	
JCVI_23407	1.443	no original description	
EV098190	1.443	very weakly similar to (100)AT1G53250| Symbols: | endonuclease | chr1:19861136-19862824 FORWARD [21477]	
DY025198	1.443	no similarity	
JCVI_30745	1.443	weakly similar to (183)AT5G61060| Symbols: HDA5, HDA05 | HDA05 (HISTONE DEACETYLASE5); histone deacetylase | chr5:24584	
JCVI_3061	1.443	weakly similar to (182)AT4G37090| Symbols: | similar to unnamed protein product [Vitis vinifera] (GB:CAO63256.1) | chr4:17477332-17	
JCVI_21410	1.443	no original description	
JCVI_8855	1.443	moderately similar to (401)AT5G21150| Symbols: | PAZ domain-containing protein / piwi domain-containing protein | chr5:7193475-7198	
EE421903	1.443	moderately similar to (250)AT3G54660| Symbols: EMB2360, ATGR2 | GR (GLUTATHIONE REDUCTASE); glutathione-disulfide reduc	
EV134295	1.443	moderately similar to (334)AT5G17860| Symbols: CAX7 | CAX7 (CALCIUM EXCHANGER 7); calcium:sodium antiporter/ cation:cation	
JCVI_27441	1.443	very weakly similar to (100)AT4G02550| Symbols: | similar to unknown protein [Arabidopsis thaliana] (TAIR:AT4G02210.1); similar to u	
JCVI_17536	1.443	highly similar to (667)AT5G22760| Symbols: | PHD finger family protein | chr5:7571638-7577665 FORWARD no original description	
JCVI_7489	1.442	moderately similar to (321)AT4G14305| Symbols: | similar to PMP22 (peroxisomal membrane protein 22) [Arabidopsis thaliana] (TAIR:A	
JCVI_37274	1.442	weakly similar to (108)AT5G60400| Symbols: | unknown protein | chr5:24308611-24308980 REVERSE no original description	
EV211119	1.442	moderately similar to (342)AT5G25880| Symbols: ATNADP-ME3 | ATNADP-ME3 (NADP-MALIC ENZYME 3); malate dehydrogenase	
JCVI_17909	1.442	no original description	
EL591103	1.442	moderately similar to (471)AT2G47630| Symbols: | esterase/lipase/thioesterase family protein | chr2:19541707-19543981 FORWARD [20	
JCVI_677	1.442	highly similar to (592)AT5G20620| Symbols: UBQ4 | UBQ4 (ubiquitin 4); protein binding | chr5:6973317-6974465 REVERSEweakly sim	
JCVI_689	1.442	moderately similar to (353)AT5G15490| Symbols: | UDP-glucose 6-dehydrogenase, putative | chr5:5027875-5029317 REVERSEmoderate	
JCVI_201	1.442	weakly similar to (191)AT1G55840| Symbols: | SEC14 cytosolic factor (SEC14) / phosphoglyceride transfer protein | chr1:20877609-2087	
EX057920	1.441	moderately similar to (219)AT3G18850| Symbols: LPAT5 | LPAT5 | chr3:6499535-6500846 REVERSE [21813]	1.750
JCVI_25026	1.441	weakly similar to (189)AT4G30935| Symbols: ATWRKY32, WRKY32 | WRKY32 (WRKY DNA-binding protein 32); transcription factor	
EH415937	1.441	weakly similar to (130)AT3G18390| Symbols: EMB1865 | EMB1865 (EMBRYO DEFECTIVE 1865) | chr3:6313578-6317590 FORWAR	
EV120139	1.441	moderately similar to (306)AT1G80680| Symbols: SAR3, MOS3, PRE | MOS3/PRE/SAR3 (SUPPRESSOR OF AUXIN RESISTANCE3);	

JCVI_34185	1.441	moderately similar to (204)AT1G45110| Symbols: | tetrapyrrole methylase family protein | chr1:17055012-17056986 FORWARD no origi	
ES955628	1.441	moderately similar to (263)AT2G28160| Symbols: FIT1, ATBHLH029, FRU, BHLH029 | ATBHLH029/BHLH029/FIT1/FRU (FE-DEFIC	
ES981436	1.441	weakly similar to (157)AT3G62850| Symbols: | zinc finger protein-related | chr3:23248462-23250130 REVERSE [21388]	1.366
JCVI_39170	1.441	very weakly similar to (92.0)AT2G28480| Symbols: | similar to group II intron splicing factor CRS1-related [Arabidopsis thaliana] (TAIR:	-1.564
EV133358	1.440	very weakly similar to (91.7)AT2G47050| Symbols: | invertase/pectin methylesterase inhibitor family protein | chr2:19338997-19339647 R	
H07417	1.440	weakly similar to (113)AT1G16700| Symbols: ATMLO14 | ATMLO14 (ARABIDOPSIS THALIANA MILDEW RESISTANCE LOCUS (	
EV227181	1.440	no similarity	
EV093133	1.440	no similarity	
ES898521	1.440	no similarity	
DY021885	1.440	no similarity	
JCVI_37104	1.439	very weakly similar to (84.7)AT5G55930| Symbols: ATOPT1 | ATOPT1 (oligopeptide transporter 1); oligopeptide transporter | chr5:22670	
JCVI_22929	1.439	very weakly similar to (95.9)AT1G77350| Symbols: | similar to unnamed protein product [Vitis vinifera] (GB:CAO47891.1) | chr1:290753	
EX073402	1.439	no similarity	
AM389522	1.439	moderately similar to (405)AT4G33650| Symbols: ADL2 | ADL2 (ARABIDOPSIS DYNAMIN-LIKE 2); GTP binding / GTPase | chr4:16	
JCVI_36960	1.439	moderately similar to (422)AT1G48450| Symbols: | similar to unknown protein [Arabidopsis thaliana] (TAIR:AT3G17800.1); similar to u	
JCVI_668	1.439	moderately similar to (286)AT3G07300| Symbols: | eukaryotic translation initiation factor 2B family protein / eIF-2B family protein | chr3	
JCVI_37634	1.439	weakly similar to (139)AT2G46510| Symbols: | basic helix-loop-helix (bHLH) family protein | chr2:19098259-19099959 REVERSE no or	
JCVI_34282	1.438	weakly similar to (127)AT3G47680| Symbols: | DNA binding | chr3:17588468-17589376 REVERSE no original description	
JCVI_24470	1.438	no original description	
EE535302	1.438	no similarity	
EX023071	1.438	weakly similar to (140)AT3G46960| Symbols: | ATP-dependent helicase | chr3:17301989-17309052 REVERSE [21809]	
JCVI_719	1.438	moderately similar to (415)AT4G39840| Symbols: | similar to unnamed protein product [Vitis vinifera] (GB:CAO21162.1); similar to unn	
JCVI_8356	1.438	moderately similar to (270)AT5G03490| Symbols: | UDP-glucoronosyl/UDP-glucosyl transferase family protein | chr5:871549-872946 FC	
JCVI_42279	1.437	highly similar to (586)AT5G27150| Symbols: ATNHX, AT-NHX1, ATNHX1, NHX1 | NHX1 (NA+/H+ EXCHANGER); sodium ion trans	
ES902719	1.437	moderately similar to (370)AT3G45880| Symbols: | similar to transcription factor jumonji (jmjC) domain-containing protein [Arabidopsis	
EV026600	1.437	no similarity	
CD817124	1.437	moderately similar to (240)AT5G09320| Symbols: | vacuolar sorting protein 9 domain-containing protein / VPS9 domain-containing prote	
JCVI_16171	1.437	moderately similar to (408)AT4G14680| Symbols: APS3 | APS3 (ATP sulfurylase 2); sulfate adenylyltransferase (ATP) | chr4:8413438-84	
EV072180	1.437	no similarity	
AM386264	1.437	no similarity	
JCVI_4149	1.436	moderately similar to (225)AT3G02910| Symbols: | Identical to UPF0131 protein At3g02910 [Arabidopsis Thaliana] (GB:Q9M8T3); simi	
JCVI_14034	1.436	moderately similar to (354)AT5G37540| Symbols: | aspartyl protease family protein | chr5:14930092-14931420 FORWARD no original de	
EV155450	1.436	moderately similar to (275)AT5G54290| Symbols: | cytochrome c biogenesis protein family | chr5:22068323-22071149 FORWARD [214	
JCVI_5157	1.436	moderately similar to (306)AT3G51390| Symbols: | zinc finger (DHHC type) family protein | chr3:19086697-19088945 FORWARD no or	
EE544890	1.436	moderately similar to (235)AT5G11380| Symbols: DXPS3 | DXPS3 (1-DEOXY-D-XYLULOSE 5-PHOSPHATE SYNTHASE 3) | chr5:36	
JCVI_41757	1.436	moderately similar to (291)AT1G14010| Symbols: | emp24/gp25L/p24 family protein | chr1:4800382-4801787 REVERSE no original desc	
JCVI_35729	1.436	highly similar to (651)AT1G56500| Symbols: | haloacid dehalogenase-like hydrolase family protein | chr1:21163440-21170757 FORWAR	
JCVI_280	1.436	moderately similar to (367)AT1G31330| Symbols: PSAF | PSAF (photosystem I subunit F) | chr1:11214992-11215920 REVERSEmoderat	
EV091800	1.436	weakly similar to (166)AT3G59210| Symbols: | F-box family protein | chr3:21900919-21902530 FORWARD [21476] 131 1045 1045	
EE471932	1.435	no similarity	
JCVI_11448	1.435	no original description	
JCVI_5489	1.435	weakly similar to (134)AT1G68550| Symbols: | AP2 domain-containing transcription factor, putative | chr1:25729473-25730447 REVERS	
EV016615	1.435	no similarity	
CD819037	1.435	weakly similar to (154)AT5G25770| Symbols: | similar to unnamed protein product [Vitis vinifera] (GB:CAO44054.1); contains domain F	
JCVI_9702	1.435	moderately similar to (437)AT1G51380| Symbols: | eukaryotic translation initiation factor 4A, putative / eIF-4A, putative | chr1:19051628	
EV027215	1.435	moderately similar to (488)AT1G11720| Symbols: ATSS3 | ATSS3 (STARCH SYNTHASE 3); starch synthase/ transferase, transferring gl	
EE466893	1.435	moderately similar to (375)AT4G16660| Symbols: | heat shock protein 70, putative / HSP70, putative | chr4:9377247-9381254 FORWARI	
EE558988	1.435	weakly similar to (155)AT3G57630| Symbols: | exostosin family protein | chr3:21350520-21354057 REVERSE [20153] 67 759 759	
EE444842	1.435	moderately similar to (355)AT4G34030| Symbols: MCCB | MCCB (3-METHYLCROTONYL-COA CARBOXYLASE); biotin carboxylas	
EE519246	1.435	weakly similar to (194)AT1G12990| Symbols: | glycosyl transferase family 17 protein | chr1:4433971-4435550 FORWARD [20185]	
JCVI_4344	1.435	highly similar to (519)AT1G72340| Symbols: | eukaryotic translation initiation factor 2B family protein / eIF-2B family protein | chr1:272	1.722
EG021206	1.435	no similarity	
JCVI_32914	1.434	moderately similar to (219)AT5G63480| Symbols: | similar to unknown [Populus trichocarpa] (GB:ABK95190.1) | chr5:25434543-25435(	
JCVI_37778	1.434	highly similar to (570)AT4G04350| Symbols: EMB2369 | EMB2369 (EMBRYO DEFECTIVE 2369); ATP binding / aminoacyl-tRNA liga	
JCVI_4508	1.434	moderately similar to (271)AT5G17840| Symbols: | chaperone protein dnaJ-related | chr5:5895881-5897057 REVERSE no original descri	
JCVI_35167	1.434	moderately similar to (226)AT1G68790| Symbols: LINC3 | LINC3 (LITTLE NUCLEI3) | chr1:25838595-25842820 REVERSE no origina	
ES954631	1.434	no similarity	
JCVI_30786	1.434	no original description	
JCVI_30609	1.434	weakly similar to (108)AT2G19240| Symbols: | RabGAP/TBC domain-containing protein | chr2:8356130-8359644 REVERSE no original	
JCVI_17305	1.434	no original description	
DN964921	1.434	weakly similar to (136)AT3G12320| Symbols: | similar to unknown protein [Arabidopsis thaliana] (TAIR:AT5G06980.1); similar to hypot	
EE473785	1.434	very weakly similar to (99.0)AT5G59000| Symbols: | zinc finger (C3HC4-type RING finger) family protein | chr5:23835994-23837244 FO	
CD820683	1.434	moderately similar to (262)AT1G17940| Symbols: | similar to unknown protein [Arabidopsis thaliana] (TAIR:AT1G73390.2); similar to u	
JCVI_27047	1.434	weakly similar to (144)AT2G20340| Symbols: | tyrosine decarboxylase, putative | chr2:8786885-8789571 FORWARDweakly similar to (1	
EE441356	1.434	weakly similar to (178)AT1G32760| Symbols: | glutaredoxin family protein | chr1:11858231-11859175 FORWARD [20167]	-3.345
JCVI_8511	1.433	highly similar to (569)AT5G67360| Symbols: ARA12 | ARA12; subtilase | chr5:26889418-26891691 REVERSE no original description	1.438
JCVI_738	1.433	weakly similar to (122)AT4G03520| Symbols: ATHM2 | ATHM2 (Arabidopsis thioredoxin M-type 2); thiol-disulfide exchange intermedia	-1.861
JCVI_2306	1.433	moderately similar to (373)AT5G22800| Symbols: EMB1030 | EMB1030 (EMBRYO DEFECTIVE 1030); ATP binding / alanine-tRNA li	
JCVI_28024	1.433	moderately similar to (415)AT5G09330| Symbols: ANAC082 | ANAC082 (Arabidopsis NAC domain containing protein 82) | chr5:289262	
ES930197	1.433	moderately similar to (216)AT4G05120| Symbols: ENT3, FUR1 | ENT3/FUR1 (FUDR RESISTANT 1); nucleoside transmembrane transp	
JCVI_38929	1.433	moderately similar to (455)AT2G37330| Symbols: ALS3 | ALS3 (ALUMINUM SENSITIVE 3) | chr2:15676269-15677251 FORWARD no	
JCVI_1420	1.433	weakly similar to (193)AT2G37160| Symbols: | transducin family protein / WD-40 repeat family protein | chr2:15616126-15619608 FORV	
JCVI_22307	1.433	moderately similar to (409)AT3G48160| Symbols: E2L3, E2FE, DEL1 | DEL1 (DP-E2F-like 1); transcription factor | chr3:17794626-1779	
JCVI_13185	1.433	highly similar to (560)AT4G04040| Symbols: MEE51 | MEE51 (maternal effect embryo arrest 51); diphosphate-fructose-6-phosphate 1-ph	
JCVI_628	1.432	moderately similar to (253)AT5G25490| Symbols: | zinc finger (Ran-binding) family protein | chr5:8876642-8877342 FORWARD no orig	-3.261
EV180633	1.432	weakly similar to (112)AT1G19610| Symbols: LCR78, PDF1.4 | LCR78/PDF1.4 (Low-molecular-weight cysteine-rich 78) | chr1:6781665-	-1.108
ES943481	1.432	weakly similar to (105)AT5G36120| Symbols: | YGGT family protein | chr5:14215909-14216433 REVERSE [21392]	
JCVI_2232	1.432	very weakly similar to (92.8)AT3G12390| Symbols: | nascent polypeptide associated complex alpha chain protein, putative / alpha-NAC, p	
JCVI_5846	1.432	moderately similar to (380)AT1G31860| Symbols: HISN2, AT-IE | AT-IE (Arabidopsis thaliana bifunctional HisI-HisE protein) | chr1:114	
JCVI_36234	1.432	moderately similar to (296)AT4G38120| Symbols: | similar to unnamed protein product [Vitis vinifera] (GB:CAO62868.1) | chr4:1789378	
EE420753	1.432	weakly similar to (177)AT4G16420| Symbols: PRZ1, ADA2B | ADA2B (PROPORZ1); DNA binding / transcription factor | chr4:9262828	

JCVI_21189	1.432	very weakly similar to (82.8)AT3G02190| Symbols: | 60S ribosomal protein L39 (RPL39B) | chr3:406012-406349 REVERSE no original d	
EV176357	1.432	very weakly similar to (92.8)AT3G25520| Symbols: ATL5, PGY3 | ATL5 (A. THALIANA RIBOSOMAL PROTEIN L5); structural constit	
EV129473	1.431	no similarity	
EE415421	1.431	moderately similar to (347)AT5G41330| Symbols: | potassium channel tetramerisation domain-containing protein | chr5:16553680-165550	1.283
JCVI_12617	1.431	no original description	
JCVI_40619	1.431	moderately similar to (363)AT5G48370| Symbols: | thioesterase family protein | chr5:19615892-19617738 REVERSE no original descript	
JCVI_41435	1.431	moderately similar to (248)AT3G05420| Symbols: ACBP4 | ACBP4 (ACYL-COA BINDING PROTEIN 4); acyl-CoA binding | chr3:15618	
JCVI_34676	1.431	moderately similar to (352)AT2G40060| Symbols: | protein binding / protein transporter/ structural molecule | chr2:16733642-16735079 F	
ES968897	1.431	no similarity	
EE401539	1.431	no similarity	
JCVI_20019	1.431	very weakly similar to (92.8)AT5G08040| Symbols: TOM5 | TOM5 (MITOCHONDRIAL IMPORT RECEPTOR SUBUNIT TOM5 HOM	
EV132289	1.431	no similarity	-1.425
EE411855	1.431	very weakly similar to (100)AT5G66450| Symbols: | phosphatidic acid phosphatase-related / PAP2-related | chr5:26552204-26553177 FO	
JCVI_8366	1.430	weakly similar to (167)AT3G22530| Symbols: | similar to unknown protein [Arabidopsis thaliana] (TAIR:AT4G14830.1); similar to unnar	
JCVI_31400	1.430	moderately similar to (301)AT1G56330| Symbols: ATSARA1B, ATSAR1, SAR1 | SAR1 (SECRETION-ASSOCIATED RAS); GTP bindi	
JCVI_455	1.430	highly similar to (507)AT3G29810| Symbols: COBL2 | COBL2 (COBRA-LIKE PROTEIN 2 PRECURSOR) | chr3:11730691-11732637 F	
BQ791430	1.430	weakly similar to (152)AT4G23850| Symbols: | long-chain-fatty-acid--CoA ligase / long-chain acyl-CoA synthetase | chr4:12403730-1240	
EX052363	1.430	moderately similar to (379)AT1G53020| Symbols: UBC26, PFU3 | PFU3/UBC26 (UBIQUITIN-CONJUGATING ENZYME 26); ubiquitin	
JCVI_787	1.430	moderately similar to (356)AT1G36390| Symbols: | co-chaperone grpE family protein | chr1:13703023-13704736 REVERSE no original d	
JCVI_41704	1.429	no original description	-5.418
EV195955	1.429	weakly similar to (187)AT5G03940| Symbols: 54CP, CPSRP54, SRP54CP, FFC | FFC (FIFTY-FOUR CHLOROPLAST HOMOLOGUE);	
EV087518	1.429	no similarity	
ES993339	1.429	moderately similar to (222)AT1G16040| Symbols: | similar to unknown [Populus trichocarpa] (GB:ABK92967.1); contains InterPro doma	
EE455077	1.429	moderately similar to (232)AT1G19010| Symbols: | similar to unknown protein [Arabidopsis thaliana] (TAIR:AT1G74860.1); similar to h	
JCVI_1217	1.429	weakly similar to (110)AT2G40475| Symbols: | unknown protein | chr2:16914300-16914881 REVERSE no original description	
JCVI_27585	1.429	weakly similar to (193)AT2G01150| Symbols: RHA2B | RHA2B (RING-H2 FINGER PROTEIN 2B); protein binding / zinc ion binding | c	
JCVI_11668	1.429	weakly similar to (123)AT4G23885| Symbols: | similar to unknown protein [Arabidopsis thaliana] (TAIR:AT5G24165.1); similar to unnar	
ES942733	1.429	no similarity	
JCVI_40707	1.429	weakly similar to (147)AT3G25882| Symbols: NIMIN-2 | NIMIN-2 (NIM1-INTERACTING 2) | chr3:9471758-9472126 REVERSE no ori	
ES941285	1.429	weakly similar to (112)AT3G15110| Symbols: | similar to unnamed protein product [Vitis vinifera] (GB:CAO39343.1) | chr3:5084401-508	
EX132417	1.429	moderately similar to (380)AT3G28450| Symbols: | leucine-rich repeat transmembrane protein kinase, putative | chr3:10668596-10670413	1.565
ES946645	1.429	very weakly similar to (98.6)AT1G16010| Symbols: | magnesium transporter CorA-like family protein (MRS2-1) | chr1:5495456-5497076	
JCVI_11885	1.428	no original description	4.097
JCVI_28399	1.428	moderately similar to (273)AT2G35330| Symbols: | zinc finger (C3HC4-type RING finger) protein-related | chr2:14876258-14878752 FO	
JCVI_4505	1.428	moderately similar to (202)AT4G31840| Symbols: | plastocyanin-like domain-containing protein | chr4:15401804-15402432 FORWARDv	
RC_EV011336	1.428	no similarity	
JCVI_42292	1.428	no original description	
EE569427	1.428	weakly similar to (181)AT4G12960| Symbols: | gamma interferon responsive lysosomal thiol reductase family protein / GILT family prote	
JCVI_2998	1.428	moderately similar to (293)AT5G58000| Symbols: | CPL4 (C-TERMINAL DOMAIN PHOSPHATASE-LIKE 4) | chr5:23494642-234967	
EV220187	1.428	moderately similar to (404)AT3G27530| Symbols: GC6 | GC6 (GOLGIN CANDIDATE 6); binding / protein transporter | chr3:10195015-1	
EX084431	1.428	no similarity	
EE524516	1.428	moderately similar to (250)AT5G13740| Symbols: ZIF1 | ZIF1 (ZINC INDUCED FACILITATOR 1); carbohydrate transmembrane transp	
EV009435	1.428	moderately similar to (278)AT1G03780| Symbols: | targeting protein-related | chr1:948063-951695 REVERSE [21427]	
EX049287	1.427	no similarity	
JCVI_31142	1.427	moderately similar to (363)AT3G55120| Symbols: TT5, A11, CFI | A11/CFI/TT5 (TRANSPARENT TESTA 5); chalcone isomerase | chr3	
JCVI_5834	1.427	moderately similar to (251)AT5G05950| Symbols: MEE60 | MEE60 (maternal effect embryo arrest 60) | chr5:1788815-1789342 FORWAI	
EV202659	1.427	very weakly similar to (85.9)AT2G17640| Symbols: SAT-106, AtSerat3;1 | AtSerat3;1 (SERINE ACETYLTRANSFERASE-106); acetyltra	
EE464699	1.427	moderately similar to (223)AT2G45280| Symbols: RAD51C, ATRAD51C | ATRAD51C (Arabidopsis thaliana Ras Associated with Diabet	
JCVI_17018	1.427	weakly similar to (118)AT4G35890| Symbols: | La domain-containing protein | chr4:16997436-17000413 FORWARD no original descript	
JCVI_14931	1.427	moderately similar to (379)AT4G01660| Symbols: ATATH10, ABC1At | ABC1At (ARABIDOPSIS THALIANA ABC TRANSPORTER 1	
EE474095	1.427	no similarity	
JCVI_16403	1.427	weakly similar to (191)AT1G08510| Symbols: FATB | FATB (FATTY ACYL-ACP THIOESTERASES B); acyl carrier/ acyl-ACP thioeste	
AM395618	1.427	weakly similar to (157)AT4G15410| Symbols: PUX5 | UBX domain-containing protein | chr4:8814868-8816596 FORWARD [20346]	
JCVI_29820	1.426	moderately similar to (275)AT4G24520| Symbols: AR1, ATR1 | ATR1 (ARABIDOPSIS P450 REDUCTASE 1) | chr4:12663075-1266707	
JCVI_10188	1.426	moderately similar to (209)AT3G07510| Symbols: | similar to unknown protein [Arabidopsis thaliana] (TAIR:AT2G01580.1); similar to h	
JCVI_7462	1.426	moderately similar to (287)AT5G02280| Symbols: | synbindin, putative | chr5:469375-470127 FORWARD no original description	-2.063
JCVI_22779	1.426	weakly similar to (192)AT3G61180| Symbols: | zinc finger (C3HC4-type RING finger) family protein | chr3:22656655-22658265 FORWA	
EV058627	1.426	no similarity	
CD825661	1.426	weakly similar to (198)AT5G19670| Symbols: | exostosin family protein | chr5:6647027-6649360 FORWARD [13979]	
JCVI_42381	1.426	no original description	
JCVI_32419	1.425	moderately similar to (476)AT1G73950| Symbols: | zinc finger (C3HC4-type RING finger) family protein | chr1:27803787-27808137 REV	
JCVI_31964	1.425	moderately similar to (335)AT2G22480| Symbols: | phosphofructokinase family protein | chr2:9552750-9555494 FORWARD no original	
JCVI_4583	1.425	weakly similar to (160)AT5G19550| Symbols: AAT2, ASP2 | ASP2 (ASPARTATE AMINOTRANSFERASE 2) | chr5:6598203-6601599 I	
JCVI_14799	1.425	moderately similar to (217)AT5G09760| Symbols: | pectinesterase family protein | chr5:3032447-3034365 FORWARD no original descrip	
EV112979	1.425	moderately similar to (310)AT4G24830| Symbols: | arginosuccinate synthase family | chr4:12793095-12795867 REVERSE [21479] 45 90	
EV091684	1.425	moderately similar to (236)AT4G19810| Symbols: | glycosyl hydrolase family 18 protein | chr4:10764161-10765763 REVERSE [21476] 1	-1.438
EV174170	1.425	no similarity	
EV013735	1.424	no similarity	
JCVI_6979	1.424	highly similar to (691)AT3G08590| Symbols: | 2,3-biphosphoglycerate-independent phosphoglycerate mutase, putative / phosphoglyceron	
JCVI_24200	1.424	moderately similar to (345)AT3G11760| Symbols: | similar to unknown protein [Arabidopsis thaliana] (TAIR:AT5G04860.1); similar to u	
JCVI_26238	1.424	moderately similar to (297)AT5G22860| Symbols: | serine carboxypeptidase S28 family protein | chr5:7639910-7642948 REVERSE no or	
CD824539	1.424	moderately similar to (201)AT5G54680| Symbols: ILR3 | ILR3 (IAA-LEUCINE RESISTANT3); DNA binding / transcription factor | chr5	
JCVI_36585	1.424	moderately similar to (273)AT4G14330| Symbols: | phragmoplast-associated kinesin-related protein 2 (PAKRP2) | chr4:8244224-8247282	
JCVI_33807	1.424	very weakly similar to (90.5)AT3G23810| Symbols: SAHH2 | SAHH2 (S-ADENOSYL-L-HOMOCYSTEINE (SAH) HYDROLASE 2); ade	
EX122544	1.424	moderately similar to (345)AT2G46915| Symbols: | similar to sodium:dicarboxylate symporter [Arabidopsis thaliana] (TAIR:AT3G19340	
EV192931	1.424	weakly similar to (143)AT3G59430| Symbols: | similar to hypothetical protein [Vitis vinifera] (GB:CAN70075.1) | chr3:21975461-219765	
EV142944	1.424	no similarity	
EV226510	1.424	no similarity	
JCVI_27305	1.424	very weakly similar to (82.8)AT5G08535| Symbols: | D111/G-patch domain-containing protein | chr5:2762456-2763321 FORWARD no or	
JCVI_14703	1.424	highly similar to (732)AT2G15695| Symbols: | similar to unknown protein [Arabidopsis thaliana] (TAIR:AT5G44250.1); similar to unnam	
AM387213	1.424	weakly similar to (106)AT2G36780| Symbols: | UDP-glucoronosyl/UDP-glucosyl transferase family protein | chr2:15424697-15426187 R	

RC_AT000610	1.424	no similarity	
JCVI_8159	1.424	highly similar to (779)AT2G01190| Symbols: | octicosapeptide/Phox/Bem1p (PB1) domain-containing protein | chr2:115022-117295 FOR	
JCVI_30279	1.424	highly similar to (625)AT2G41770| Symbols: | similar to unknown protein [Arabidopsis thaliana] (TAIR:AT3G57420.1); similar to unnam	
EV160119	1.423	weakly similar to (152)AT5G45330| Symbols: | similar to unknown protein [Arabidopsis thaliana] (TAIR:AT1G26110.1); similar to hypot	
EX090406	1.423	weakly similar to (122)AT5G52550| Symbols: | similar to unknown protein [Arabidopsis thaliana] (TAIR:AT4G25670.1); similar to hypot	
EV047490	1.423	no similarity	
JCVI_15133	1.423	weakly similar to (188)AT2G16740| Symbols: UBC29 | UBC29 (UBIQUITIN-CONJUGATING ENZYME 29); ubiquitin-protein ligase | c	
JCVI_3209	1.423	moderately similar to (274)AT5G16110| Symbols: | similar to unknown protein [Arabidopsis thaliana] (TAIR:AT3G02555.1); similar to h	1.367
JCVI_20011	1.423	no original description	
EV209417	1.423	very weakly similar to (89.7)AT1G70580| Symbols: GGT2, AOAT2 | AOAT2 (GLUTAMATE:GLYOXYLATE AMINOTRANSFERASE	
EE474206	1.422	no similarity	
CN737571	1.422	moderately similar to (341)AT1G12140| Symbols: | flavin-containing monooxygenase family protein / FMO family protein | chr1:4121384	
EG021132	1.422	weakly similar to (103)AT3G61960| Symbols: | protein kinase family protein | chr3:22952941-22955971 REVERSE [20440] 16 353 353	
EV011970	1.422	no similarity	
EL589472	1.422	weakly similar to (124)AT5G15190| Symbols: | unknown protein | chr5:4933151-4933501 REVERSE [20863]	
CX280144	1.422	no similarity	
EV009160	1.422	moderately similar to (441)AT3G51130| Symbols: | Identical to UPF0183 protein At3g51130 [Arabidopsis Thaliana] (GB:Q9SD33;GB:Q	
JCVI_28256	1.422	moderately similar to (269)AT3G20040| Symbols: ATHXK4 | ATHXK4; ATP binding / hexokinase | chr3:6995323-6998070 FORWARDv	
JCVI_26979	1.422	moderately similar to (343)AT1G09940| Symbols: HEMA2 | HEMA2; glutamyl-tRNA reductase | chr1:3237226-3239264 REVERSEmode	
JCVI_10844	1.422	weakly similar to (176)AT5G64870| Symbols: | similar to unknown protein [Arabidopsis thaliana] (TAIR:AT5G25250.1); similar to unkno	
ES957290	1.421	weakly similar to (169)AT1G28070| Symbols: | protein binding | chr1:9783069-9783851 FORWARD [21423]	
EV103308	1.421	no similarity	
EE462238	1.421	weakly similar to (152)AT1G29290| Symbols: | similar to hypothetical protein [Vitis vinifera] (GB:CAN69942.1) | chr1:10245041-102453	
EX130165	1.421	no similarity	
EE480364	1.421	no similarity	
JCVI_40173	1.421	moderately similar to (305)AT4G20940| Symbols: | leucine-rich repeat family protein | chr4:11202739-11206049 FORWARD no original	
JCVI_3096	1.421	weakly similar to (102)AT5G60550| Symbols: GRIK2 | GRIK2 (GEMINIVIRUS REP INTERACTING KINASE 2); kinase | chr5:2435730	
JCVI_10374	1.421	weakly similar to (200)AT3G32940| Symbols: | RNA binding | chr3:13494802-13497343 REVERSE no original description	
JCVI_12089	1.421	no original description	0.987
JCVI_16876	1.421	weakly similar to (150)AT2G27580| Symbols: | zinc finger (AN1-like) family protein | chr2:11783717-11784208 REVERSE no original de	
ES901710	1.421	weakly similar to (184)AT5G40670| Symbols: | PQ-loop repeat family protein / transmembrane family protein | chr5:16303196-16304819	
JCVI_29370	1.421	moderately similar to (383)AT2G42810| Symbols: PAPP5, PP5 | PAPP5/PP5 (PROTEIN PHOSPHATASE 5); phosphoprotein phosphatas	
EX068222	1.421	moderately similar to (375)AT5G06850| Symbols: | C2 domain-containing protein | chr5:2127201-2129210 REVERSE [21816]	
EX037943	1.420	very weakly similar to (98.2)AT1G10650| Symbols: | protein binding / zinc ion binding | chr1:3524123-3525061 REVERSE [21811]	
JCVI_3319	1.420	moderately similar to (289)AT5G23240| Symbols: | DNAJ heat shock N-terminal domain-containing protein | chr5:7826860-7828537 REV	
CX267333	1.420	moderately similar to (233)AT3G07550| Symbols: | F-box family protein (FBL12) | chr3:2409952-2411139 FORWARD [16816] 1 593 60	
ES905538	1.420	no similarity	
ES910530	1.420	weakly similar to (177)AT5G43100| Symbols: | aspartyl protease family protein | chr5:17316492-17319946 FORWARD [21430]	
JCVI_2549	1.420	moderately similar to (398)AT3G59920| Symbols: ATGDI2 | ATGDI2 (RAB GDP DISSOCIATION INHIBITOR 2); RAB GDP-dissociat	
JCVI_11437	1.419	moderately similar to (370)AT2G21520| Symbols: | similar to SEC14 cytosolic factor, putative / phosphoglyceride transfer protein, putativ	
EV209380	1.419	weakly similar to (120)AT1G48760| Symbols: DELTA-ADR | DELTA-ADR (DELTA-ADAPTIN); clathrin binding | chr1:18040320-1804	
JCVI_20160	1.419	moderately similar to (431)AT3G12250| Symbols: BZIP45, TGA6 | TGA6 (TGA1a-related gene 6) | chr3:3906643-3908590 FORWARDm	
JCVI_15666	1.419	no original description	
EX037652	1.419	moderately similar to (341)AT5G53060| Symbols: | KH domain-containing protein | chr5:21532581-21535423 FORWARD [21811]	
RC_EV012764	1.419	no similarity	
EV066648	1.419	weakly similar to (151)AT1G69830| Symbols: ATAMY3, AMY3 | AMY3/ATAMY3 (ALPHA-AMYLASE-LIKE 3); alpha-amylase | chr1	
EV108600	1.419	no similarity	
JCVI_317	1.419	moderately similar to (279)AT2G43090| Symbols: | aconitase C-terminal domain-containing protein | chr2:17926034-17926789 FORWAR	
JCVI_13893	1.419	weakly similar to (160)AT5G51440| Symbols: | 23.5 kDa mitochondrial small heat shock protein (HSP23.5-M) | chr5:20908468-2090923	3.667
EV012214	1.419	no similarity	
EV093279	1.419	moderately similar to (221)AT5G45680| Symbols: | FK506-binding protein 1 (FKBP13) | chr5:18548121-18549355 FORWARDvery weal	
EX098073	1.419	moderately similar to (298)AT1G66760| Symbols: | MATE efflux family protein | chr1:24905773-24907876 FORWARD [21824]	
ES993612	1.418	moderately similar to (241)AT1G16930| Symbols: | F-box family protein | chr1:5789980-5791527 FORWARD [21427]	
EV100456	1.418	no similarity	
EX126629	1.418	moderately similar to (287)AT5G25265| Symbols: | similar to unknown protein [Arabidopsis thaliana] (TAIR:AT2G25260.1); similar to u	
JCVI_12718	1.418	no original description	
EE403063	1.418	moderately similar to (320)AT5G24600| Symbols: | similar to unknown protein [Arabidopsis thaliana] (TAIR:AT3G18215.1); similar to u	
EE529675	1.418	moderately similar to (367)AT3G20440| Symbols: EMB2729, BE1 | BE1/EMB2729 (BRANCHING ENZYME 1); alpha-amylase | chr3:71	
JCVI_11721	1.418	moderately similar to (230)AT2G21560| Symbols: | similar to unknown protein [Arabidopsis thaliana] (TAIR:AT4G39190.1); similar to h	
JCVI_11209	1.418	no original description	
JCVI_7495	1.418	moderately similar to (372)AT4G38640| Symbols: | choline transporter-related | chr4:18059878-18062085 REVERSE no original descript	
JCVI_38617	1.418	no original description	
CV545809	1.418	weakly similar to (124)AT1G74710| Symbols: ICS1, EDS16, SID2 | ICS1 (ISOCHORISMATE SYNTHASEI); isochorismate synthase | ch	
CX193979	1.418	no similarity	
EX025044	1.418	very weakly similar to (100)AT5G03330| Symbols: | OTU-like cysteine protease family protein | chr5:807727-809607 FORWARD [2181(	
EV199635	1.418	moderately similar to (275)AT3G12780| Symbols: PGK1 | PGK1 (PHOSPHOGLYCERATE KINASE 1); phosphoglycerate kinase | chr3:4	
JCVI_19391	1.417	weakly similar to (109)AT5G54970| Symbols: | similar to unknown protein [Arabidopsis thaliana] (TAIR:AT4G26960.1) | chr5:22330497	
JCVI_9926	1.417	moderately similar to (293)AT1G07410| Symbols: AtRABA2b | AtRABA2b (Arabidopsis Rab GTPase homolog A2b); GTP binding | chr1	-1.178
JCVI_9371	1.417	moderately similar to (464)AT3G61760| Symbols: | dynamin-like protein B (DL1B) | chr3:22871521-22875067 REVERSE no original des	-1.419
JCVI_7268	1.417	moderately similar to (276)AT5G56180| Symbols: ATARP8 | ATARP8 (ACTIN-RELATED PROTEIN 8); structural constituent of cytosk	
JCVI_31119	1.417	moderately similar to (363)AT4G09750| Symbols: | short-chain dehydrogenase/reductase (SDR) family protein | chr4:6146767-6148695 F	
JCVI_39229	1.417	moderately similar to (356)AT1G26670| Symbols: VTI12, ATVTI12, VTI1B | ATVTI12/VTI12/VTI1B (VESICAL TRANSPORT V-SNA	
JCVI_2332	1.417	moderately similar to (452)AT1G47500| Symbols: ATRBP47C' | ATRBP47C' (RNA-BINDING PROTEIN 47C'); RNA binding | chr1:174	
ES918792	1.417	moderately similar to (428)AT4G30060| Symbols: | similar to unknown protein [Arabidopsis thaliana] (TAIR:AT2G19160.1); similar to u	
AM388303	1.417	moderately similar to (277)AT5G55560| Symbols: | protein kinase family protein | chr5:22523703-22524983 REVERSE [20118] 32 520 5	
ES907832	1.417	no similarity	
EV095067	1.416	no similarity	
ES995282	1.416	weakly similar to (162)AT1G11720| Symbols: ATSS3 | ATSS3 (STARCH SYNTHASE 3); starch synthase/ transferase, transferring glyco:	
RC_JCVI_31431	1.416	no original description	
CN728753	1.416	moderately similar to (307)AT4G01935| Symbols: | similar to unnamed protein product [Vitis vinifera] (GB:CAO40149.1) | chr4:841102-	
JCVI_23096	1.416	moderately similar to (341)AT1G73360| Symbols: HDG11 | HDG11 (HOMEODOMAIN GLABROUS11); DNA binding / transcription fa	1.341

EX111970	1.416	moderately similar to (276)AT5G28490| Symbols: LSH1 | LSH1 (LIGHT-DEPENDENT SHORT HYPOCOTYLS 1) | chr5:10454545-104	
JCVI_38075	1.416	moderately similar to (214)AT3G19800| Symbols: | similar to unnamed protein product [Vitis vinifera] (GB:CAO15360.1) | chr3:6876159	
EV095777	1.416	moderately similar to (201)AT2G39950| Symbols: | similar to unnamed protein product [Vitis vinifera] (GB:CAO69028.1) | chr2:1668383	
CD835536	1.416	no similarity	
JCVI_16964	1.416	moderately similar to (291)AT3G07880| Symbols: | Rho GDP-dissociation inhibitor family protein | chr3:2514181-2515550 FORWARD n	
JCVI_9467	1.416	moderately similar to (213)AT3G10110| Symbols: MEE67 | MEE67 (maternal effect embryo arrest 67); P-P-bond-hydrolysis-driven protei	
EE466848	1.416	no similarity	
EV157138	1.415	weakly similar to (142)AT5G42300| Symbols: UBL5 | UBL5 (UBIQUITIN-LIKE PROTEIN 5) | chr5:16930839-16931060 REVERSE [21	
EX088841	1.415	weakly similar to (160)AT1G68980| Symbols: | pentatricopeptide (PPR) repeat-containing protein | chr1:25936686-25938545 FORWARD	
JCVI_40556	1.415	no original description	
CN727465	1.415	weakly similar to (108)AT3G21610| Symbols: | similar to unknown protein [Arabidopsis thaliana] (TAIR:AT1G67600.1); similar to unnan	
JCVI_10444	1.415	moderately similar to (374)AT4G24800| Symbols: | MA3 domain-containing protein | chr4:12782473-12784912 FORWARD no original d	
EV123956	1.415	no similarity	
JCVI_36893	1.414	no original description	
JCVI_15391	1.414	moderately similar to (333)AT1G15170| Symbols: | MATE efflux family protein | chr1:5220685-5222751 FORWARD no original descrip	
JCVI_34283	1.414	moderately similar to (253)AT4G34430| Symbols: ATSWI3D, CHB3 | CHB3 (Arabidopsis thaliana switch 3D); DNA binding / transcriptio	-2.600
JCVI_24996	1.414	no original description	
JCVI_12496	1.414	weakly similar to (179)AT4G21470| Symbols: ATFMN/FHY | ATFMN/FHY (RIBOFLAVIN KINASE/FMN HYDROLASE); FMN adeny	
ES905950	1.414	moderately similar to (263)AT5G59710| Symbols: ATVIP2, VIP2 | VIP2 (VIRE2 INTERACTING PROTEIN2); transcription regulator | c	
JCVI_32894	1.414	moderately similar to (372)AT5G21326| Symbols: | protein kinase family protein / NAF domain-containing protein | chr5:7218084-72217	
AM386913	1.414	weakly similar to (103)AT4G19970| Symbols: | similar to unknown protein [Arabidopsis thaliana] (TAIR:AT5G44820.1); similar to unnan	
JCVI_6331	1.414	moderately similar to (352)AT3G50960| Symbols: PLP3A | PLP3A (PHOSDUCIN-LIKE PROTEIN 3 HOMOLOG) | chr3:18949872-189	
RC_EV106497	1.413	no similarity	
DY024942	1.413	moderately similar to (422)AT1G09390| Symbols: | GDSL-motif lipase/hydrolase family protein | chr1:3031266-3033417 FORWARDwea	
DY023448	1.413	weakly similar to (101)AT3G05680| Symbols: EMB2016 | EMB2016 (EMBRYO DEFECTIVE 2016) | chr3:1660808-1671052 REVERSE	
JCVI_25276	1.413	weakly similar to (162)AT1G75660| Symbols: XRN3 | XRN3 (5'-3' exoribonuclease 3); 5'-3' exoribonuclease | chr1:28411950-28418486 F	
L37635	1.413	moderately similar to (231)AT1G27440| Symbols: GUT2 | GUT2; catalytic | chr1:9529252-9531200 REVERSE [132]	
AM387205	1.413	very weakly similar to (100)AT1G24350| Symbols: | similar to unknown protein [Arabidopsis thaliana] (TAIR:AT1G67600.1); similar to u	
AM395340	1.413	no similarity	
JCVI_40152	1.413	moderately similar to (429)AT5G19520| Symbols: | mechanosensitive ion channel domain-containing protein / MS ion channel domain-co	
ES903061	1.413	weakly similar to (162)AT1G65590| Symbols: HEXO3, ATHEX1 | ATHEX1/HEXO3 (BETA-HEXOSAMINIDASE 3); beta-N-acetylhex	
ES900102	1.413	moderately similar to (352)AT1G48660| Symbols: | auxin-responsive GH3 family protein | chr1:17999616-18001640 REVERSEmoderate	
JCVI_24364	1.413	no original description	
ES944995	1.413	moderately similar to (231)AT1G12770| Symbols: EMB1586 | EMB1586 (EMBRYO DEFECTIVE 1586) | chr1:4351886-4353541 FORW	-4.403
DY003360	1.412	very weakly similar to (88.6)AT3G11930| Symbols: | universal stress protein (USP) family protein | chr3:3776377-3777399 FORWARD [1	
JCVI_7739	1.412	no original description	
JCVI_8761	1.412	moderately similar to (372)AT4G36940| Symbols: | nicotinate phosphoribosyltransferase | chr4:17416935-17419880 FORWARD no origi	
JCVI_26952	1.412	moderately similar to (435)AT1G08350| Symbols: | endomembrane protein 70 family protein | chr1:2632967-2635602 REVERSE no origi	
EX031747	1.412	moderately similar to (240)AT4G22770| Symbols: | DNA-binding family protein | chr4:11963890-11965450 REVERSE [21810]	
ES906027	1.412	moderately similar to (421)AT2G19410| Symbols: | protein kinase family protein | chr2:8411983-8416094 REVERSE [21429] 14 878 878	
JCVI_844	1.412	moderately similar to (439)AT3G53130| Symbols: CYP97C1, LUT1 | LUT1 (LUTEIN DEFICIENT 1); oxygen binding | chr3:19703790-1	
ES937461	1.412	no similarity	
EX032073	1.412	weakly similar to (102)AT1G78800| Symbols: | glycosyl transferase family 1 protein | chr1:29630752-29632834 REVERSE [21810]	
JCVI_13145	1.412	weakly similar to (168)AT3G53570| Symbols: AME2, AFC1 | AFC1 (ARABIDOPSIS FUS3-COMPLEMENTING GENE 1); kinase | chr3	
JCVI_13862	1.411	highly similar to (676)AT5G11580| Symbols: | UVB-resistance protein-related / regulator of chromosome condensation (RCC1) family pr	
JCVI_27158	1.411	moderately similar to (265)AT4G39910| Symbols: UBP3, ATUBP3 | ATUBP3 (UBIQUITIN-SPECIFIC PROTEASE 3); ubiquitin-specifi	1.608
H74506	1.411	no similarity	
ES955590	1.411	weakly similar to (125)AT5G03780| Symbols: TRFL10 | TRFL10 (TRF-LIKE 10); DNA binding | chr5:999265-1000946 REVERSE [2142	
JCVI_1443	1.411	moderately similar to (351)AT1G72030| Symbols: | GCN5-related N-acetyltransferase (GNAT) family protein | chr1:27114549-27115487	
JCVI_22557	1.411	weakly similar to (189)AT3G24450| Symbols: | copper-binding family protein | chr3:8880293-8881618 REVERSE no original description	
JCVI_34289	1.411	highly similar to (523)AT4G25240| Symbols: SKS1 | SKS1 (SKU5 SIMILAR 1); copper ion binding | chr4:12930549-12933573 FORWAR	
EV109949	1.411	no similarity	
ES904070	1.410	moderately similar to (292)AT1G32200| Symbols: ACT1, ATS1 | ATS1 (ACYLTRANSFERASE 1) | chr1:11602203-11604981 REVERSE	
EE473605	1.410	weakly similar to (155)AT4G02620| Symbols: | (VACUOLAR ATPASE SUBUNIT F); hydrogen ion transporting ATP synthase, rotationa	
JCVI_24811	1.410	no original description	
JCVI_39322	1.410	moderately similar to (272)AT1G52190| Symbols: | proton-dependent oligopeptide transport (POT) family protein | chr1:19438340-19442	
EV209740	1.410	moderately similar to (303)AT1G21070| Symbols: | transporter-related | chr1:7376137-7377799 REVERSE [21491]	
CD834960	1.410	moderately similar to (346)AT5G53440| Symbols: | similar to unknown protein [Arabidopsis thaliana] (TAIR:AT1G56660.1); similar to h	0.969
JCVI_24787	1.410	moderately similar to (357)AT2G27350| Symbols: | OTU-like cysteine protease family protein | chr2:11706858-11709441 REVERSE no c	
JCVI_14961	1.410	moderately similar to (232)AT1G10960| Symbols: ATFD1 | ATFD1 (FERREDOXIN 1); 2 iron, 2 sulfur cluster binding / electron carrier/ i	
EE563762	1.410	weakly similar to (101)AT1G53830| Symbols: ATPME2 | ATPME2 (Arabidopsis thaliana pectin methylesterase 2) | chr1:20102230-20104	
JCVI_24154	1.410	moderately similar to (457)AT3G49390| Symbols: CID10 | CID10; RNA binding | chr3:18325886-18328643 REVERSE no original descrip	
JCVI_23352	1.409	moderately similar to (330)AT2G37390| Symbols: | heavy-metal-associated domain-containing protein | chr2:15701379-15702540 FORW	
EE412973	1.409	no similarity	
EL592921	1.409	weakly similar to (147)AT1G53320| Symbols: AtTLP7 | AtTLP7 (TUBBY LIKE PROTEIN 7); phosphoric diester hydrolase/ transcription	
EV179818	1.409	moderately similar to (270)AT1G69680| Symbols: | similar to unknown [Populus trichocarpa] (GB:ABK93471.1); contains InterPro doma	
EE464584	1.409	moderately similar to (238)AT1G24440| Symbols: | protein binding / zinc ion binding | chr1:8662328-8663765 FORWARD [20171] 31 74	
JCVI_1568	1.409	moderately similar to (385)AT5G15650| Symbols: RGP2 | RGP2 (REVERSIBLY GLYCOSYLATED POLYPEPTIDE 2); alpha-1,4-gluca	
ES991644	1.409	moderately similar to (214)AT2G02740| Symbols: ATWHY3, PTAC11 | ATWHY3/PTAC11 (A. THALIANA WHIRLY 3) | chr2:769388-	
CX190294	1.409	no similarity	
JCVI_34586	1.408	moderately similar to (477)AT3G26840| Symbols: | esterase/lipase/thioesterase family protein | chr3:9894045-9897391 FORWARD no or	
EX138092	1.408	weakly similar to (183)AT5G52960| Symbols: | similar to unnamed protein product [Vitis vinifera] (GB:CAO69341.1) | chr5:21494476-2	
AM387287	1.408	no similarity	
EV117438	1.408	weakly similar to (174)AT1G79280| Symbols: NUA | NUA (NUCLEAR PORE ANCHOR) | chr1:29824069-29837702 REVERSE [21479]	
JCVI_34675	1.408	moderately similar to (300)AT1G21990| Symbols: | F-box family protein | chr1:7740519-7742095 REVERSE no original description	
JCVI_27348	1.408	no original description	
CN733761	1.408	no similarity	
EV217243	1.408	no similarity	
EV098981	1.408	moderately similar to (362)AT3G21330| Symbols: | basic helix-loop-helix (bHLH) family protein | chr3:7507726-7508847 FORWARD [2	
EX087303	1.408	very weakly similar to (97.1)AT2G15000| Symbols: | similar to unknown protein [Arabidopsis thaliana] (TAIR:AT4G34265.2); similar to u	
EE459104	1.408	no similarity	1.433

JCVI_20906	1.408	moderately similar to (374)AT2G32860| Symbols: | glycosyl hydrolase family 1 protein | chr2:13947310-13950673 FORWARDweakly sir	
JCVI_35110	1.408	moderately similar to (381)AT5G60410| Symbols: ATSIZ1, SIZ1 | ATSIZ1/SIZ1 | chr5:24312452-24318018 FORWARD no original descr	
EX040744	1.407	weakly similar to (144)AT2G26280| Symbols: CID7 | CID7; ATP binding / damaged DNA binding | chr2:11195221-11197657 REVERSE	
EV157472	1.407	moderately similar to (228)AT4G22290| Symbols: | ubiquitin thiolesterase | chr4:11783211-11785742 REVERSE [21484]	
AM389288	1.407	weakly similar to (179)AT5G11250| Symbols: | disease resistance protein (TIR-NBS-LRR class), putative | chr5:3587979-3591961 REVE	
JCVI_36993	1.407	no original description	
JCVI_4513	1.407	moderately similar to (351)AT3G08740| Symbols: | elongation factor P (EF-P) family protein | chr3:2654794-2656160 REVERSE no orig	
EX069369	1.407	no similarity	
JCVI_20242	1.407	highly similar to (686)AT5G39970| Symbols: | catalytic | chr5:16015383-16017979 FORWARD no original description	
JCVI_31237	1.407	weakly similar to (172)AT2G40080| Symbols: ELF4 | ELF4 (EARLY FLOWERING 4) | chr2:16741623-16741958 REVERSE no original	
EV193501	1.407	moderately similar to (342)AT3G56630| Symbols: CYP94D2 | CYP94D2 (cytochrome P450, family 94, subfamily D, polypeptide 2); oxyg	
ES918017	1.407	moderately similar to (279)AT1G67780| Symbols: | similar to unknown protein [Arabidopsis thaliana] (TAIR:AT1G67270.1); similar to u	
JCVI_20025	1.407	no original description	
EV110700	1.407	no similarity	
JCVI_2554	1.407	moderately similar to (333)AT1G58030| Symbols: CAT2 | CAT2 (CATIONIC AMINO ACID TRANSPORTER 2); amino acid transmeml	
JCVI_8980	1.407	moderately similar to (280)AT3G23820| Symbols: GAE6 | GAE6 (UDP-D-GLUCURONATE 4-EPIMERASE 6); catalytic | chr3:8603652-	
JCVI_20391	1.406	moderately similar to (295)AT3G02280| Symbols: | flavodoxin family protein | chr3:453653-457666 FORWARD no original description	
JCVI_15603	1.406	moderately similar to (367)AT1G20510| Symbols: OPCL1 | OPCL1 (OPC-8:0 COA LIGASE1); 4-coumarate-CoA ligase | chr1:7103929-7	2.022
AM386889	1.406	no similarity	
EV179110	1.406	weakly similar to (148)AT1G27752| Symbols: | similar to unnamed protein product [Vitis vinifera] (GB:CAO61467.1); contains InterPro	
JCVI_21468	1.406	no original description	
EE534222	1.406	no similarity	
JCVI_8495	1.406	weakly similar to (185)AT2G39910| Symbols: | binding | chr2:16667682-16669964 FORWARD no original description	
JCVI_21642	1.406	highly similar to (588)AT4G33520| Symbols: HMA6, PAA1 | PAA1 (metal-transporting P-type ATPase 1) | chr4:16118996-16125852 FO	
JCVI_20966	1.406	highly similar to (519)AT4G27080| Symbols: ATPDIL5-4 | ATPDIL5-4 (PDI-LIKE 5-4) | chr4:13589162-13593341 FORWARD no origir	
JCVI_38710	1.406	moderately similar to (446)AT2G28390| Symbols: | SAND family protein | chr2:12146913-12150452 REVERSE no original description	
JCVI_12695	1.406	no original description	
ES992424	1.405	no similarity	
EV142479	1.405	moderately similar to (236)AT4G35750| Symbols: | Rho-GTPase-activating protein-related | chr4:16940870-16941679 REVERSE [21482	
CX273025	1.405	weakly similar to (160)AT1G24050| Symbols: | similar to unknown protein [Arabidopsis thaliana] (TAIR:AT1G70220.1); similar to unkn	
EE475239	1.405	very weakly similar to (100)AT4G18570| Symbols: | proline-rich family protein | chr4:10231450-10234545 FORWARD [20134]	1.419
JCVI_6417	1.405	moderately similar to (318)AT1G05170| Symbols: | galactosyltransferase family protein | chr1:1491459-1493930 REVERSE no original d	
EE532039	1.405	weakly similar to (161)AT3G54930| Symbols: | serine/threonine protein phosphatase 2A (PP2A) regulatory subunit B', putative | chr3:203	
JCVI_22519	1.405	weakly similar to (160)AT1G71840| Symbols: | transducin family protein / WD-40 repeat family protein | chr1:27026086-27028042 FORV	0.486
JCVI_40308	1.405	no original description	-3.046
JCVI_15477	1.405	weakly similar to (191)AT2G46690| Symbols: | auxin-responsive family protein | chr2:19187974-19188339 FORWARD no original descri	
JCVI_18166	1.405	weakly similar to (120)AT5G32440| Symbols: | similar to unknown [Populus trichocarpa] (GB:ABK93674.1); contains InterPro domain U	
JCVI_36230	1.405	no original description	
JCVI_11465	1.404	weakly similar to (144)AT3G10525| Symbols: | similar to SIM (SIAMESE) [Arabidopsis thaliana] (TAIR:AT5G04470.1) | chr3:3281581-	1.567
EV073328	1.404	no similarity	
JCVI_41253	1.404	weakly similar to (158)AT5G58380| Symbols: SIP1, SNRK3.8, PKS2, CIPK10 | CIPK10 (CBL-INTERACTING PROTEIN KINASE 10);	
EV215693	1.404	weakly similar to (197)AT3G23080| Symbols: | similar to unknown protein [Arabidopsis thaliana] (TAIR:AT4G14500.1); similar to unnar	-1.515
JCVI_37775	1.404	weakly similar to (133)AT5G08520| Symbols: | myb family transcription factor | chr5:2755471-2757742 REVERSE no original descriptio	
JCVI_14855	1.404	weakly similar to (139)AT1G12830| Symbols: | unknown protein | chr1:4374410-4375051 REVERSE no original description	
EX072833	1.404	moderately similar to (421)AT1G80350| Symbols: AAA1, LUE1, FRA2, ATKTN1, KTN1, FRC2, BOT1, FTR, ERH3 | ERH3 (ECTOPIC	
JCVI_8470	1.403	highly similar to (603)AT2G34660| Symbols: EST4, MRP2, ATMRP2 | ATMRP2 (MULTIDRUG RESISTANCE-ASSOCIATED PROTE	
JCVI_6791	1.403	moderately similar to (392)AT3G17611| Symbols: | rhomboid family protein / zinc finger protein-related | chr3:6025658-6026179 FORW	
EX127658	1.403	moderately similar to (254)AT3G01380| Symbols: | phosphatidylinositolglycan class N (PIG-N) family protein | chr3:144295-149835 REV	
JCVI_10918	1.403	highly similar to (569)AT3G16810| Symbols: APUM24 | APUM24 (ARABIDOPSIS PUMILIO 24); RNA binding | chr3:5723442-572754	
JCVI_34943	1.403	moderately similar to (435)AT3G14910| Symbols: | similar to unnamed protein product [Vitis vinifera] (GB:CAO39401.1); contains doma	
ES941304	1.403	weakly similar to (106)AT3G03420| Symbols: | Ku70-binding family protein | chr3:812534-813651 FORWARD [21391]	-1.689
JCVI_13583	1.403	moderately similar to (461)AT5G65110| Symbols: ATACX2, ACX2 | ACX2 (ACYL-COA OXIDASE 2); acyl-CoA oxidase | chr5:260272	
JCVI_11442	1.403	highly similar to (585)AT5G48230| Symbols: EMB1276, ACAT2 | ACAT2/EMB1276 (ACETOACETYL-COA THIOLASE 2); acetyl-Co	
EV151794	1.403	no similarity	
EE538336	1.403	no similarity	
JCVI_21388	1.403	highly similar to (555)AT3G17850| Symbols: | protein kinase, putative | chr3:6109860-6116251 REVERSEweakly similar to (139)KPK1_	
EV043384	1.403	moderately similar to (325)AT3G05480| Symbols: | cell cycle checkpoint control protein family | chr3:1585389-1588244 FORWARD [21	
JCVI_14851	1.403	highly similar to (539)AT5G22100| Symbols: | RNA cyclase family protein | chr5:7329018-7330721 FORWARD no original description	
JCVI_32733	1.402	moderately similar to (446)AT3G48160| Symbols: E2L3, E2FE, DEL1 | DEL1 (DP-E2F-like 1); transcription factor | chr3:17794626-1779	
EE434960	1.402	no similarity	
JCVI_4248	1.402	moderately similar to (497)AT5G17290| Symbols: ATG5, APG5 | APG5/ATG5 (AUTOPHAGY 5); transporter | chr5:5687162-5689823 F	
JCVI_1966	1.401	weakly similar to (141)AT4G17486| Symbols: | Identical to UPF0326 protein At4g17486 [Arabidopsis Thaliana] (GB:Q93VG8;GB:O235	
EV081908	1.401	very weakly similar to (86.7)AT1G43130| Symbols: LCV2 | LCV2 (LIKE COV 2) | chr1:16231101-16233598 REVERSE [21444]	-1.100
JCVI_28531	1.401	no original description	
JCVI_10317	1.401	weakly similar to (170)AT2G24150| Symbols: HHP3 | HHP3 (heptahelical protein 3); receptor | chr2:10272712-10274425 REVERSE no c	
JCVI_20442	1.401	moderately similar to (423)AT3G11890| Symbols: | similar to unknown protein [Arabidopsis thaliana] (TAIR:AT3G11860.1); similar to u	
ES964191	1.401	no similarity	
JCVI_13113	1.401	moderately similar to (426)AT3G06490| Symbols: AtMYB108, BOS1, MYB108 | MYB108 (MYB DOMAIN PROTEIN 108); DNA bindi	
EE542986	1.401	moderately similar to (287)AT2G31600| Symbols: | similar to unknown protein [Arabidopsis thaliana] (TAIR:AT3G53860.1); similar to u	
JCVI_34601	1.401	moderately similar to (244)AT5G14070| Symbols: ROXY2 | ROXY2; thiol-disulfide exchange intermediate | chr5:4541917-4542339 FOR	
JCVI_34272	1.401	highly similar to (884)AT2G44450| Symbols: | glycosyl hydrolase family 1 protein | chr2:18348042-18350820 FORWARDmoderately sim	
DY010548	1.401	weakly similar to (109)AT4G01070| Symbols: GT72B1 | GT72B1; UDP-glucosyltransferase/ UDP-glycosyltransferase/ transferase, transf	
AM061936	1.401	weakly similar to (149)AT5G09880| Symbols: | RNA recognition motif (RRM)-containing protein | chr5:3081647-3085180 REVERSE [1	
RC_EE502279	1.401	no similarity	
EX119173	1.400	moderately similar to (370)AT2G28690| Symbols: | similar to unknown protein [Arabidopsis thaliana] (TAIR:AT5G59760.1); similar to u	
JCVI_18220	1.400	nearly identical (1654)AT2G05710| Symbols: | aconitate hydratase, cytoplasmic, putative / citrate hydro-lyase/aconitase, putative | chr2:21	
JCVI_30414	1.400	moderately similar to (254)AT1G79975| Symbols: | similar to Os01g0593600 [Oryza sativa (japonica cultivar-group)] (GB:NP_00104346	
JCVI_1258	1.400	moderately similar to (365)AT3G27240| Symbols: | cytochrome c1, putative | chr3:10057381-10059607 REVERSEmoderately similar to (	
JCVI_18822	1.400	weakly similar to (142)AT4G00430| Symbols: PIP1;4, PIP1E, TMP-C | TMP-C (PLASMA MEMBRANE INTRINSIC PROTEIN 1;4); wa	
JCVI_36923	1.400	moderately similar to (367)AT2G45690| Symbols: SSE, PEX16, SSE1 | SSE1 (SHRUNKEN SEED 1) | chr2:18830539-18832675 REVER	
JCVI_5827	1.399	weakly similar to (150)AT4G34190| Symbols: SEP1 | SEP1 (STRESS ENHANCED PROTEIN 1) | chr4:16372610-16373515 REVERSE r	

JCVI_6452	1.399	weakly similar to (192)AT3G04920| Symbols: | 40S ribosomal protein S24 (RPS24A) | chr3:1360995-1362071 FORWARD no original de	
JCVI_39007	1.399	moderately similar to (358)AT1G24480| Symbols: | methyltransferase | chr1:8676428-8677147 REVERSE no original description	
EE462294	1.399	weakly similar to (169)AT1G75580| Symbols: | auxin-responsive protein, putative | chr1:28381191-28381517 FORWARD [15722]	
JCVI_31813	1.399	moderately similar to (210)AT2G21045| Symbols: | similar to unknown protein [Arabidopsis thaliana] (TAIR:AT5G66170.2); similar to p	
EX135590	1.399	no similarity	
JCVI_24114	1.399	no original description	
AM394610	1.399	no similarity	
EV223406	1.399	moderately similar to (361)AT2G47410| Symbols: | nucleotide binding | chr2:19456203-19464061 FORWARD [21493]	
JCVI_21464	1.399	moderately similar to (382)AT1G61210| Symbols: | WD-40 repeat family protein / katanin p80 subunit, putative | chr1:22568450-2257522	
EV110741	1.398	no similarity	
EV071267	1.398	very weakly similar to (97.8)AT2G43640| Symbols: | signal recognition particle 14 kDa family protein / SRP14 family protein | chr2:18104	
EV090569	1.398	weakly similar to (109)AT5G43330| Symbols: | malate dehydrogenase, cytosolic, putative | chr5:17407780-17409677 FORWARDweakly	
EX131577	1.398	no similarity	
EV105943	1.398	no similarity	
EE458919	1.398	weakly similar to (186)AT5G44390| Symbols: | FAD-binding domain-containing protein | chr5:17899556-17902133 REVERSE [20152] 1	
EV022258	1.398	weakly similar to (115)AT3G51390| Symbols: | zinc finger (DHHC type) family protein | chr3:19086697-19088945 FORWARD [21441]	
JCVI_14421	1.398	highly similar to (595)AT4G02500| Symbols: ATXT2 | ATXT2; UDP-xylosyltransferase/ transferase/ transferase, transferring glycosyl gro	
EE549722	1.398	weakly similar to (112)AT5G60390| Symbols: | elongation factor 1-alpha / EF-1-alpha | chr5:24306452-24307901 FORWARDweakly sim	
RC_EE534313	1.398	no similarity	
JCVI_11523	1.398	no original description	
EV132667	1.398	weakly similar to (118)AT5G15770| Symbols: ATGNA1 | ATGNA1 (ARABIDOPSIS THALIANA GLUCOSE-6-PHOSPHATE ACETYL	
EX053498	1.398	moderately similar to (273)AT2G14530| Symbols: | similar to unknown protein [Arabidopsis thaliana] (TAIR:AT5G64470.2); similar to h	
EV174410	1.398	no similarity	-1.377
EV159595	1.398	no similarity	
JCVI_11733	1.398	moderately similar to (267)AT5G50200| Symbols: ATNRT3.1, NRT3.1, WR3 | WR3 (WOUND-RESPONSIVE 3); nitrate transmembrane	-1.266
EV135378	1.397	weakly similar to (102)AT5G55100| Symbols: | SWAP (Suppressor-of-White-APricot)/surp domain-containing protein | chr5:22378627-2	
JCVI_13410	1.397	weakly similar to (139)AT5G25270| Symbols: | similar to ubiquitin family protein [Arabidopsis thaliana] (TAIR:AT5G11080.1); similar to	
JCVI_27262	1.397	highly similar to (608)AT3G02690| Symbols: | integral membrane family protein | chr3:579634-581455 FORWARD no original descriptio	
EH419222	1.397	very weakly similar to (87.4)AT4G12300| Symbols: CYP706A4 | CYP706A4 (cytochrome P450, family 706, subfamily A, polypeptide 4);	
JCVI_8016	1.396	moderately similar to (313)AT2G21940| Symbols: | shikimate kinase, putative | chr2:9358186-9359961 FORWARD no original descriptio	
JCVI_19584	1.396	moderately similar to (333)AT1G80770| Symbols: PDE318 | PDE318 (PIGMENT DEFECTIVE 318); GTP binding | chr1:30360158-3036	
JCVI_26739	1.396	weakly similar to (157)AT5G08335| Symbols: ATSTE14B | ATSTE14B (PRENYLCYSTEINE ALPHA-CARBOXYL METHYLTRANSF	
JCVI_20412	1.396	moderately similar to (269)AT5G66160| Symbols: ATRMR1, JR700 | JR700 (Arabidopsis thaliana receptor homology region transmembra	
JCVI_15059	1.396	weakly similar to (199)AT3G27880| Symbols: | similar to unknown protein [Arabidopsis thaliana] (TAIR:AT1G23710.1); similar to hypot	
JCVI_19074	1.396	weakly similar to (159)AT1G54210| Symbols: APG12, ATG12a | ATG12a (AUTOPHAGY 12); protein binding | chr1:20244945-2024568	
EV170748	1.396	moderately similar to (391)AT2G39830| Symbols: | zinc ion binding | chr2:16627028-16630736 REVERSE [21486] 100 966 966	
JCVI_37703	1.396	moderately similar to (409)AT4G23050| Symbols: | protein kinase, putative | chr4:12080123-12083719 FORWARDvery weakly similar to	
EE484610	1.396	moderately similar to (221)AT2G20810| Symbols: GAUT10, LGT4 | GAUT10/LGT4 (Galacturonosyltransferase 10); polygalacturonate 4-	
JCVI_26949	1.396	weakly similar to (175)AT3G60820| Symbols: PBF1 | PBF1 (20S proteasome beta subunit F1); peptidase | chr3:22483013-22484784 REV	
JCVI_12007	1.396	weakly similar to (126)AT3G13280| Symbols: | similar to nucleic acid binding / zinc ion binding [Arabidopsis thaliana] (TAIR:AT1G0135	
ES946111	1.396	moderately similar to (247)AT1G54140| Symbols: TAF9, TAFII21 | TAFII21 (TATA BINDING PROTEIN ASSOCIATED FACTOR 21K	
JCVI_39692	1.396	very weakly similar to (99.4)AT5G52750| Symbols: | heavy-metal-associated domain-containing protein | chr5:21401360-21402021 FORW	
JCVI_17589	1.396	highly similar to (586)AT2G28110| Symbols: IRX7, FRA8 | FRA8 (FRAGILE FIBER8); transferase | chr2:11985101-11986718 REVERSI	
JCVI_4086	1.396	moderately similar to (401)AT1G49670| Symbols: | ARP protein (REF) | chr1:18385259-18389689 REVERSEvery weakly similar to (90.1	
ES264210	1.395	no similarity	
JCVI_24260	1.395	no original description	
CD818792	1.395	no similarity	1.442
EE480886	1.395	moderately similar to (304)AT4G29820| Symbols: CFIM-25, ATCFIM-25 | ATCFIM-25/CFIM-25 (ARABIDOPSIS HOMOLOG OF CFIM	
JCVI_30099	1.395	weakly similar to (110)AT5G38670| Symbols: | F-box family protein | chr5:15493016-15494101 FORWARD no original description	-1.246
JCVI_18728	1.395	moderately similar to (278)AT3G12350| Symbols: | F-box family protein | chr3:3931633-3933111 FORWARD no original description	
JCVI_29541	1.395	no original description	
JCVI_18102	1.395	moderately similar to (497)AT1G07040| Symbols: | similar to unknown protein [Arabidopsis thaliana] (TAIR:AT1G27030.1); similar to u	
JCVI_16029	1.394	weakly similar to (127)AT4G17520| Symbols: | nuclear RNA-binding protein, putative | chr4:9771509-9773326 FORWARD no original d	
EX112545	1.394	weakly similar to (188)AT1G13580| Symbols: LAG13 | LAG13 (LAG1 LONGEVITY ASSURANCE HOMOLOG 3) | chr1:4645003-4646	
EV217392	1.394	weakly similar to (118)AT1G55990| Symbols: | glycine-rich protein | chr1:20946152-20946616 REVERSE [21492]	
JCVI_12606	1.394	moderately similar to (438)AT5G63780| Symbols: SHA1 | SHA1 (SHOOT APICAL MERISTEM ARREST 1); protein binding / zinc ion b	
ES909342	1.394	moderately similar to (322)AT5G39990| Symbols: | glycosyltransferase family 14 protein / core-2/I-branching enzyme family protein | chr	
CD831644	1.394	moderately similar to (333)AT4G22270| Symbols: | similar to unknown protein [Arabidopsis thaliana] (TAIR:AT4G03820.1); similar to u	
EV088366	1.394	moderately similar to (413)AT5G60700| Symbols: | glycosyltransferase family protein 2 | chr5:24419555-24421955 REVERSE [21444]	
ES946441	1.394	weakly similar to (119)AT1G69210| Symbols: | similar to unknown protein [Arabidopsis thaliana] (TAIR:AT1G77122.1); similar to unnar	
CA991555	1.394	moderately similar to (233)AT3G28760| Symbols: | similar to unnamed protein product [Vitis vinifera] (GB:CAO14940.1); contains Inter	
EV171065	1.393	moderately similar to (286)AT3G22890| Symbols: APS1 | APS1 (ATP sulfurylase 3) | chr3:8112844-8114741 FORWARD [21486]	
JCVI_13933	1.393	moderately similar to (437)AT3G01060| Symbols: | similar to unnamed protein product [Vitis vinifera] (GB:CAO15045.1); similar to unk	
JCVI_29323	1.393	moderately similar to (228)AT5G16840| Symbols: | RNA recognition motif (RRM)-containing protein | chr5:5536045-5538029 FORWAR	
EV135006	1.393	weakly similar to (118)AT1G28350| Symbols: | ATP binding / aminoacyl-tRNA ligase | chr1:9944470-9949564 FORWARD [21481] 1 45	
EE565013	1.393	no similarity	
JCVI_5305	1.393	moderately similar to (336)AT1G14685| Symbols: BBR/BPC2, ATBPC2, BPC2 | BPC2 (BASIC PENTACYSTEINE 2) | chr1:5043081-50	
JCVI_38583	1.393	moderately similar to (431)AT3G21230| Symbols: 4CL5 | 4CL5 (4-COUMARATE:COA LIGASE 5); 4-coumarate-CoA ligase | chr3:7448	
EE512033	1.393	very weakly similar to (85.9)AT2G34720| Symbols: | CCAAT-binding transcription factor (CBF-B/NF-YA) family protein | chr2:1465709	
AM385557	1.393	moderately similar to (235)AT1G49670| Symbols: | ARP protein (REF) | chr1:18385259-18389689 REVERSE [20118] 1 537 568	
JCVI_36427	1.392	highly similar to (639)AT2G39760| Symbols: ATBPM3 | ATBPM3; protein binding | chr2:16590291-16591893 FORWARD no original de	
JCVI_18346	1.392	very weakly similar to (83.2)AT1G60870| Symbols: MEE9 | MEE9 (maternal effect embryo arrest 9) | chr1:22413377-22413892 FORWAR	
JCVI_32095	1.392	moderately similar to (292)AT5G44040| Symbols: | similar to unknown protein [Arabidopsis thaliana] (TAIR:AT1G04030.1); similar to u	
JCVI_15518	1.392	moderately similar to (281)AT4G28980| Symbols: CDKF;1, CAK1AT | CAK1AT/CDKF;1 (CDK-ACTIVATING KINASE 1AT); cyclin-c	
JCVI_1970	1.392	moderately similar to (441)AT5G39320| Symbols: | UDP-glucose 6-dehydrogenase, putative | chr5:15760482-15761924 FORWARDmode	
ES945704	1.392	moderately similar to (362)AT3G45830| Symbols: | similar to unknown protein [Arabidopsis thaliana] (TAIR:AT1G02290.1); similar to u	
JCVI_38865	1.392	moderately similar to (278)AT5G14790| Symbols: | binding | chr5:4784061-4785511 FORWARD no original description	
EV142390	1.392	no similarity	
JCVI_741	1.392	moderately similar to (442)AT2G43000| Symbols: ANAC042 | ANAC042 (Arabidopsis NAC domain containing protein 42); transcription	
JCVI_15295	1.391	moderately similar to (360)AT4G26650| Symbols: | RNA recognition motif (RRM)-containing protein | chr4:13445271-13447598 FORW	
EX131456	1.391	weakly similar to (120)AT4G36040| Symbols: | DNAJ heat shock N-terminal domain-containing protein (J11) | chr4:17049711-17050196	

EV052005	1.391	no similarity	
EX094324	1.391	moderately similar to (294)AT1G12000| Symbols: | pyrophosphate--fructose-6-phosphate 1-phosphotransferase beta subunit, putative / py	1.758
JCVI_13857	1.391	weakly similar to (161)AT5G49220| Symbols: | similar to unknown protein [Arabidopsis thaliana] (TAIR:AT2G01260.1); similar to unnar	
JCVI_28336	1.391	highly similar to (626)AT3G63070| Symbols: | PWWP domain-containing protein | chr3:23313642-23320550 FORWARD no original desc	
JCVI_41019	1.390	no original description	
JCVI_39264	1.390	moderately similar to (344)AT5G18590| Symbols: | kelch repeat-containing protein | chr5:6178518-6182188 REVERSE no original descri	
JCVI_2508	1.390	weakly similar to (138)AT3G60750| Symbols: | transketolase, putative | chr3:22464979-22467799 FORWARDweakly similar to (131)TK	
DY019898	1.390	no similarity	
JCVI_17495	1.390	highly similar to (521)AT2G35940| Symbols: EDA29, BLH1 | BLH1 (embryo sac development arrest 29) | chr2:15096250-15098778 REV	
EX103668	1.390	moderately similar to (224)AT3G50920| Symbols: | phosphatidic acid phosphatase-related / PAP2-related | chr3:18933384-18934361 REV	
JCVI_11601	1.390	no original description	
JCVI_21431	1.390	highly similar to (536)AT3G15730| Symbols: PLD, PLDALPHA1 | PLDALPHA1 (PHOSPHOLIPASE D ALPHA 1); phospholipase D | ch	
EV132535	1.390	no similarity	
EE556724	1.390	very weakly similar to (92.0)AT1G61590| Symbols: | protein kinase, putative | chr1:22727356-22729687 REVERSE [20153] 30 755 778	
L47851	1.390	weakly similar to (142)AT4G34200| Symbols: EDA9 | EDA9 (embryo sac development arrest 9); NAD binding / amino acid binding / bind	
JCVI_22631	1.389	weakly similar to (104)AT1G74950| Symbols: JAZ2, TIFY10B | JAZ2/TIFY10B (JASMONATE-ZIM-DOMAIN PROTEIN 2) | chr1:281	
JCVI_3467	1.389	moderately similar to (410)AT5G03240| Symbols: UBQ3 | UBQ3 (POLYUBIQUITIN 3); protein binding | chr5:771975-772895 REVERS	
ES986822	1.389	no similarity	
EE515436	1.389	moderately similar to (215)AT1G65420| Symbols: | Identical to Ycf20-like protein [Arabidopsis Thaliana] (GB:O80813); similar to unkno	
JCVI_6840	1.389	moderately similar to (381)AT3G04290| Symbols: ATLTL1, LTL1 | ATLTL1/LTL1 (LI-TOLERANT LIPASE 1); carboxylesterase | chr3:	
JCVI_39525	1.389	moderately similar to (454)AT2G29370| Symbols: | tropinone reductase, putative / tropine dehydrogenase, putative | chr2:12613136-1261	
JCVI_12931	1.389	moderately similar to (448)AT2G28790| Symbols: | osmotin-like protein, putative | chr2:12361741-12362490 REVERSEweakly similar to	
EV184556	1.389	weakly similar to (187)AT4G23290| Symbols: | protein kinase family protein | chr4:12177920-12180820 REVERSE [21487] 39 795 795	
DW997803	1.389	moderately similar to (210)AT5G21140| Symbols: EMB1379 | EMB1379 (EMBRYO DEFECTIVE 1379) | chr5:7187419-7189524 REVE	
EV112896	1.389	moderately similar to (296)AT3G11570| Symbols: | similar to unknown protein [Arabidopsis thaliana] (TAIR:AT5G06230.1); similar to h	
JCVI_7018	1.389	highly similar to (642)AT2G44100| Symbols: AT-GDI1 | ATGDI1 (Arabidopsis thaliana guanosine diphosphate dissociation inhibitor 1) |	
JCVI_28312	1.389	no original description	-1.385
BG543396	1.389	no similarity	-1.125
CX190826	1.388	no similarity	
JCVI_32826	1.388	moderately similar to (480)AT1G08410| Symbols: | GTP-binding family protein | chr1:2646304-2649103 FORWARD no original descript	
JCVI_39158	1.388	moderately similar to (244)AT1G54140| Symbols: TAF9, TAFII21 | TAFII21 (TATA BINDING PROTEIN ASSOCIATED FACTOR 21K	
JCVI_38046	1.388	highly similar to (564)AT2G03760| Symbols: RAR047, ST | ST (steroid sulfotransferase); sulfotransferase | chr2:1149472-1150452 REVE	
EV131042	1.388	weakly similar to (150)AT5G45900| Symbols: ATAPG7, ATG7, APG7 | APG7 (AUTOPHAGY 7) | chr5:18632531-18635663 FORWARI	
JCVI_27068	1.388	moderately similar to (250)AT4G20780| Symbols: | calcium-binding protein, putative | chr4:11133320-11133895 REVERSEweakly simila	
JCVI_19697	1.388	weakly similar to (136)AT3G10810| Symbols: | zinc finger (C3HC4-type RING finger) family protein | chr3:3381853-3384232 REVERSE	
EV215728	1.388	no similarity	
JCVI_40310	1.388	moderately similar to (387)AT2G02080| Symbols: ATIDD4 | ATIDD4 (ARABIDOPSIS THALIANA INDETERMINATE(ID)-DOMAIN	
JCVI_20919	1.388	no original description	-1.223
JCVI_3695	1.388	weakly similar to (166)AT3G56240| Symbols: CCH | CCH (COPPER CHAPERONE) | chr3:20874439-20875381 REVERSE no original d	
JCVI_5132	1.388	moderately similar to (268)AT3G04400| Symbols: EMB2171 | EMB2171 (EMBRYO DEFECTIVE 2171); structural constituent of riboso	
EX040325	1.388	moderately similar to (269)AT5G06410| Symbols: | DNAJ heat shock N-terminal domain-containing protein | chr5:1959720-1961133 REV	
JCVI_37211	1.388	no original description	
JCVI_1421	1.388	moderately similar to (412)AT3G23820| Symbols: GAE6 | GAE6 (UDP-D-GLUCURONATE 4-EPIMERASE 6); catalytic | chr3:8603652	
EV102811	1.388	weakly similar to (131)AT1G12420| Symbols: ACR8 | ACR8 (ACT Domain Repeat 8) | chr1:4226671-4228915 REVERSE [21477]	
JCVI_25630	1.387	weakly similar to (196)AT1G60770| Symbols: | pentatricopeptide (PPR) repeat-containing protein | chr1:22370624-22372313 REVERSE	
EV104032	1.387	weakly similar to (159)AT3G61480| Symbols: | similar to unknown protein [Arabidopsis thaliana] (TAIR:AT5G28350.2); similar to unkno	
JCVI_21077	1.387	moderately similar to (440)AT5G04720| Symbols: ADR1-L2 | ADR1-L2 (ADR1-LIKE 2); ATP binding / nucleoside-triphosphatase/ nucle	1.369
EE440983	1.387	moderately similar to (239)AT1G02700| Symbols: | similar to unknown protein [Arabidopsis thaliana] (TAIR:AT4G02140.1); similar to h	
JCVI_19489	1.387	moderately similar to (402)AT4G16143| Symbols: | importin alpha-2, putative (IMPA-2) | chr4:9134736-9137147 REVERSEmoderately s	
EE533713	1.387	no similarity	
JCVI_19935	1.387	very weakly similar to (85.1)AT3G51330| Symbols: | aspartyl protease family protein | chr3:19064459-19067131 REVERSE no original de	
H07239	1.386	no similarity	
JCVI_38318	1.386	moderately similar to (228)AT3G13062| Symbols: | similar to unknown protein [Arabidopsis thaliana] (TAIR:AT1G55960.1); similar to u	
JCVI_2761	1.386	moderately similar to (379)AT1G32160| Symbols: | similar to unknown protein [Arabidopsis thaliana] (TAIR:AT3G17800.1); similar to u	
JCVI_41777	1.386	moderately similar to (268)AT3G27260| Symbols: GTE8 | GTE8 (GLOBAL TRANSCRIPTION FACTOR GROUP E8); DNA binding | ch	
JCVI_17945	1.386	moderately similar to (221)AT2G30250| Symbols: ATWRKY25, WRKY25 | WRKY25 (WRKY DNA-binding protein 25); transcription fa	
JCVI_18401	1.386	moderately similar to (210)AT4G18810| Symbols: | binding / catalytic/ transcription repressor | chr4:10322633-10325746 REVERSE no o	
AM387983	1.386	no similarity	
JCVI_41648	1.386	no original description	
ES946550	1.385	no similarity	
BG543324	1.385	weakly similar to (106)AT4G34450| Symbols: | coatomer gamma-2 subunit, putative / gamma-2 coat protein, putative / gamma-2 COP, pu	
JCVI_34873	1.385	moderately similar to (468)AT2G22475| Symbols: GEM | GEM | chr2:9548603-9551858 FORWARD no original description	
JCVI_33356	1.385	moderately similar to (319)AT4G29060| Symbols: EMB2726 | EMB2726 (EMBRYO DEFECTIVE 2726); translation elongation factor | c	
JCVI_34054	1.385	no original description	
JCVI_9867	1.385	moderately similar to (494)AT3G57290| Symbols: TIF3E1, ATEIF3E-1, INT-6, ATINT6, INT6, EIF3E | EIF3E (eukaryotic translation ini	
DY005623	1.385	no similarity	
EV101039	1.385	no similarity	
JCVI_40531	1.385	weakly similar to (143)AT5G06500| Symbols: AGL96 | AGL96; DNA binding / transcription factor | chr5:1982445-1983173 FORWARD r	
EE557837	1.385	no similarity	
JCVI_13779	1.385	moderately similar to (435)AT1G66150| Symbols: TMK1 | TMK1 (TRANSMEMBRANE KINASE 1) | chr1:24635166-24638078 FORW	
CX266948	1.384	weakly similar to (149)AT2G45900| Symbols: | similar to unknown protein [Arabidopsis thaliana] (TAIR:AT3G61380.1); similar to {, rel	1.053
JCVI_39739	1.384	nearly identical (1170)AT1G05150| Symbols: | calcium-binding EF hand family protein | chr1:1484279-1486705 REVERSE no original de	
JCVI_1921	1.384	moderately similar to (285)AT1G26630| Symbols: FBR12 | FBR12 (FUMONISIN B1-RESISTANT12) | chr1:9205955-9207000 FORWAI	
EV222443	1.384	no similarity	
EX092629	1.384	moderately similar to (405)AT1G01320| Symbols: | tetratricopeptide repeat (TPR)-containing protein | chr1:121582-130099 REVERSE [2	
JCVI_18243	1.384	moderately similar to (286)AT1G50420| Symbols: SCL-3, SCL3 | SCL3 (SCARECROW-LIKE 3); transcription factor | chr1:18681845-18	
JCVI_29237	1.384	weakly similar to (188)AT1G79090| Symbols: | similar to unknown protein [Arabidopsis thaliana] (TAIR:AT3G22270.1); similar to unnar	
EX126943	1.384	moderately similar to (338)AT5G15310| Symbols: AtMYB16, AtMIXTA | AtMIXTA/AtMYB16 (myb domain protein 16) | chr5:4974897	
JCVI_42299	1.384	very weakly similar to (82.4)AT2G17787| Symbols: | similar to unknown protein [Arabidopsis thaliana] (TAIR:AT4G35940.1); similar to l	
JCVI_7468	1.383	weakly similar to (172)AT1G71950| Symbols: | identical protein binding / subtilase | chr1:27084115-27085235 REVERSE no original des	
JCVI_42611	1.383	highly similar to (506)AT1G53040| Symbols: | hydrolase, acting on carbon-nitrogen (but not peptide) bonds, in linear amides | chr1:19768	

JCVI_35201	1.383	very weakly similar to (93.2)AT4G36820| Symbols: | transcription factor | chr4:17346893-17347998 FORWARD no original description	
JCVI_4564	1.383	moderately similar to (370)AT4G02640| Symbols: ATBZIP10, BZO2H1 | BZO2H1 (ARABIDOPSIS THALIANA BASIC LEUCINE ZIPI	
ES943003	1.383	moderately similar to (284)AT3G06550| Symbols: | similar to O-acetyltransferase family protein [Arabidopsis thaliana] (TAIR:AT2G344	
JCVI_17292	1.383	moderately similar to (318)AT4G03020| Symbols: | transducin family protein / WD-40 repeat family protein | chr4:1331704-1334472 REV	
EG021112	1.383	weakly similar to (140)AT3G55880| Symbols: | similar to unknown protein [Arabidopsis thaliana] (TAIR:AT2G40095.1); similar to unnar	
JCVI_9521	1.383	moderately similar to (399)AT2G38010| Symbols: | ceramidase family protein | chr2:15913940-15916945 FORWARD no original descrip	
JCVI_1846	1.382	highly similar to (538)AT2G36360| Symbols: | kelch repeat-containing protein | chr2:15250540-15254602 REVERSEvery weakly similar	
JCVI_26461	1.382	weakly similar to (181)AT3G49490| Symbols: | unknown protein | chr3:18355911-18359633 REVERSE no original description	
JCVI_20104	1.382	moderately similar to (299)AT2G16530| Symbols: | 3-oxo-5-alpha-steroid 4-dehydrogenase family protein / steroid 5-alpha-reductase fam	
EX029534	1.382	weakly similar to (133)AT1G24267| Symbols: | similar to unknown protein [Arabidopsis thaliana] (TAIR:AT1G24265.2); similar to unkn	
CD825087	1.382	weakly similar to (152)AT3G54270| Symbols: | sucrose-phosphatase 3 (SPP3) | chr3:20098397-20100713 REVERSE [13979] 14 570 570	
JCVI_7298	1.382	weakly similar to (164)AT1G03290| Symbols: | similar to unknown protein [Arabidopsis thaliana] (TAIR:AT4G02880.1); similar to unnar	
AT001754	1.382	very weakly similar to (98.6)AT5G04800| Symbols: | 40S ribosomal protein S17 (RPS17D) | chr5:1389218-1389643 FORWARD [1846]	
EX081696	1.382	no similarity	
JCVI_42328	1.382	no original description	
JCVI_6187	1.382	moderately similar to (346)AT3G23920| Symbols: BMY7, TR-BAMY, BAM1 | BAM1/BMY7/TR-BAMY (BETA-AMYLASE 1); beta-ar	
JCVI_730	1.382	moderately similar to (387)AT1G10200| Symbols: WLIM1 | WLIM1; transcription factor | chr1:3346679-3347765 REVERSEmoderately s	
JCVI_29957	1.382	highly similar to (777)AT1G66880| Symbols: | serine/threonine protein kinase family protein | chr1:24950591-24959101 FORWARDmod	
JCVI_28809	1.382	moderately similar to (369)AT5G37580| Symbols: | protein binding | chr5:14944633-14946796 REVERSE no original description	
JCVI_20265	1.382	highly similar to (865)AT3G58750| Symbols: CSY2 | CSY2 (CITRATE SYNTHASE 2); citrate (SI)-synthase | chr3:21735541-21738435 I	
CD843675	1.381	very weakly similar to (88.2)AT2G43945| Symbols: | similar to unknown protein [Arabidopsis thaliana] (TAIR:AT3G59870.1); similar to I	
EV087609	1.381	weakly similar to (176)AT1G49975| Symbols: | similar to hypothetical protein [Vitis vinifera] (GB:CAN66219.1) | chr1:18508513-18509(	2.517
JCVI_16789	1.381	highly similar to (702)AT1G74210| Symbols: | glycerophosphoryl diester phosphodiesterase family protein | chr1:27914057-27916446 FC	
JCVI_12537	1.381	moderately similar to (432)AT1G08200| Symbols: AXS2 | AXS2 (UDP-D-APIOSE/UDP-D-XYLOSE SYNTHASE 2) | chr1:2574256-257	
JCVI_10473	1.381	moderately similar to (216)AT3G10800| Symbols: BZIP28 | BZIP28; DNA binding / transcription factor | chr3:3379331-3381435 FORWA	
JCVI_28187	1.381	no original description	-1.498
JCVI_34180	1.381	moderately similar to (478)AT5G54390| Symbols: ATAHL, AHL | AHL (HAL2-LIKE); 3'(2'),5'-bisphosphate nucleotidase/ inositol or pho	
ES980817	1.380	moderately similar to (229)AT2G42360| Symbols: | zinc finger (C3HC4-type RING finger) family protein | chr2:17647985-17648695 FOI	
EE525715	1.380	weakly similar to (159)AT2G31840| Symbols: | similar to unknown protein [Arabidopsis thaliana] (TAIR:AT4G28590.1); similar to hypot	
EV004240	1.380	moderately similar to (297)AT4G25910| Symbols: ATCNFU3, NFU3 | NFU3 (NFU domain protein 3) | chr4:13164137-13165103 FORWA	
JCVI_18690	1.380	moderately similar to (391)AT1G11190| Symbols: ENDO1, BFN1 | BFN1 (BIFUNCTIONAL NUCLEASE I); nucleic acid binding | chr1:3	
JCVI_4706	1.380	moderately similar to (433)AT5G64670| Symbols: | ribosomal protein L15 family protein | chr5:25869761-25871106 REVERSE no origin	1.262
JCVI_39078	1.380	weakly similar to (167)AT4G21610| Symbols: LOL2 | LOL2 (LSD ONE LIKE 2); transcription factor | chr4:11489081-11490312 FORWA	
EX137160	1.380	no similarity	
EX015542	1.380	no similarity	
JCVI_14819	1.380	moderately similar to (273)AT3G18750| Symbols: ZIK5, WNK6 | WNK6 (Arabidopsis WNK kinase 6); kinase | chr3:6454313-6456836 R	
JCVI_28931	1.380	moderately similar to (436)ATCG00340| Symbols: PSAB | Encodes the D1 subunit of photosystem I and II reaction centers. | chrC:37375	
EV032553	1.380	moderately similar to (386)AT1G20575| Symbols: | dolichyl-phosphate beta-D-mannosyltransferase, putative / dolichol-phosphate manno	
JCVI_31584	1.380	moderately similar to (201)AT4G15417| Symbols: ATRTL1 | ribonuclease III family protein | chr4:8821759-8822991 FORWARD no orig	
JCVI_28242	1.380	no original description	-1.607
EL591262	1.379	moderately similar to (308)AT5G64560| Symbols: | magnesium transporter CorA-like family protein (MRS2-2) | chr5:25824389-2582662	
EX118767	1.379	weakly similar to (132)AT1G04870| Symbols: | protein arginine N-methyltransferase family protein | chr1:1373484-1375597 REVERSE [	
JCVI_31440	1.379	no original description	
EV036045	1.379	moderately similar to (207)AT3G22990| Symbols: | binding | chr3:8164022-8166152 FORWARD [21441]	-1.636
JCVI_32500	1.379	moderately similar to (315)AT1G19920| Symbols: ASA1, APS2 | APS2 (ATP SULFURYLASE PRECURSOR) | chr1:6914826-6916648 R	
JCVI_15418	1.379	moderately similar to (272)AT1G03350| Symbols: | BSD domain-containing protein | chr1:822834-824246 REVERSE no original descript	
JCVI_33899	1.379	no original description	
JCVI_33812	1.379	moderately similar to (380)AT5G07870| Symbols: | transferase family protein | chr5:2513865-2515352 FORWARD no original descriptio	1.179
JCVI_15085	1.379	moderately similar to (269)AT2G17975| Symbols: | zinc finger (Ran-binding) family protein | chr2:7829320-7831052 REVERSE no origi	1.464
EE567814	1.379	no similarity	
JCVI_21162	1.379	weakly similar to (114)AT5G48040| Symbols: | similar to unknown protein [Arabidopsis thaliana] (TAIR:AT3G58520.1); similar to unkn	
ES960029	1.378	no similarity	1.606
JCVI_13518	1.378	moderately similar to (366)AT3G17810| Symbols: | dihydroorotate dehydrogenase family protein / dihydroorotate oxidase family protein |	
ES903146	1.378	weakly similar to (116)AT2G47020| Symbols: | peptide chain release factor, putative | chr2:19327160-19329349 REVERSE [21432] 1 85	
JCVI_10350	1.378	moderately similar to (288)AT3G09050| Symbols: | similar to unknown [Populus trichocarpa x Populus deltoides] (GB:ABK96465.1) | ch	1.680
JCVI_22940	1.378	moderately similar to (367)AT1G28310| Symbols: | Dof-type zinc finger domain-containing protein | chr1:9912521-9913672 REVERSEw	-1.274
JCVI_16966	1.378	moderately similar to (289)AT3G54750| Symbols: | similar to unnamed protein product [Vitis vinifera] (GB:CAO48944.1); contains doma	
JCVI_7215	1.378	highly similar to (513)AT2G25910| Symbols: | 3'-5' exonuclease domain-containing protein / K homology domain-containing protein / KH	
RC_EE558049	1.378	no similarity	
ES935313	1.378	moderately similar to (383)AT3G24090| Symbols: | transaminase | chr3:8697845-8700978 REVERSE [16816]	
EE472139	1.377	moderately similar to (352)AT2G35540| Symbols: | DNAJ heat shock N-terminal domain-containing protein | chr2:14934237-14936009 F	
JCVI_294	1.377	moderately similar to (422)AT1G67060| Symbols: | similar to unnamed protein product [Vitis vinifera] (GB:CAO49338.1) | chr1:2504039	-1.855
JCVI_20789	1.377	very weakly similar to (91.7)AT5G41685| Symbols: | mitochondrial import receptor subunit TOM7 / translocase of outer membrane 7 kDa	
JCVI_35316	1.377	moderately similar to (340)AT3G20270| Symbols: | lipid-binding serum glycoprotein family protein | chr3:7068885-7070872 FORWARD	
ES904711	1.377	no similarity	
JCVI_7013	1.377	weakly similar to (177)AT1G48400| Symbols: | F-box family protein | chr1:17885804-17887523 REVERSE no original description	
ES937632	1.377	no similarity	
H07174	1.377	no similarity	
CN736874	1.376	moderately similar to (206)AT1G72210| Symbols: | basic helix-loop-helix (bHLH) family protein (bHLH096) | chr1:27183728-27185930	
JCVI_40476	1.376	moderately similar to (259)AT4G31080| Symbols: | similar to unknown protein [Arabidopsis thaliana] (TAIR:AT2G24330.1); similar to u	
EE560396	1.376	no similarity	
EV070967	1.376	no similarity	
JCVI_24794	1.376	moderately similar to (332)AT3G05320| Symbols: | similar to unnamed protein product [Vitis vinifera] (GB:CAO44440.1) | chr3:1513564	
EE557738	1.376	no similarity	
EX067786	1.376	weakly similar to (164)AT4G35790| Symbols: PLDDELTA, ATPLDDELTA | ATPLDDELTA (Arabidopsis thaliana phospholipase D delt	
JCVI_40948	1.376	moderately similar to (218)AT2G19530| Symbols: | similar to unknown protein [Arabidopsis thaliana] (TAIR:AT1G55160.1); similar to u	
DY027303	1.376	weakly similar to (114)AT3G62450| Symbols: | similar to hypothetical protein MtrDRAFT_AC140551g61v2 [Medicago truncatula] (GB:	
DY019557	1.376	weakly similar to (173)AT2G32840| Symbols: | proline-rich family protein | chr2:13940505-13941913 REVERSE [18966] 18 603 603	
JCVI_32727	1.376	moderately similar to (468)AT4G35380| Symbols: | guanine nucleotide exchange family protein | chr4:16819888-16825965 FORWARD n	
JCVI_24915	1.376	no original description	
EX123200	1.376	moderately similar to (477)AT5G04360| Symbols: ATPU1, ATLDA | ATLDA/ATPU1 (PULLULANASE 1); alpha-amylase/ limit dextrina	

EX083238	1.376	weakly similar to (172)AT1G63650| Symbols: EGL1, ATMYC-2, EGL3 | EGL3 (ENHANCER OF GLABRA3); DNA binding | chr1:2360	
EX139023	1.376	no similarity	
JCVI_35788	1.376	weakly similar to (111)AT5G18120| Symbols: ATAPRL7 | ATAPRL7 (APR-LIKE 7) | chr5:5991387-5993698 FORWARD no original de	
JCVI_19093	1.375	weakly similar to (159)AT2G25710| Symbols: HCS1 | HCS1 (HOLOCARBOXYLASE SYNTHASE); catalytic | chr2:10959797-10962139	1.602
JCVI_22939	1.375	very weakly similar to (80.1)AT2G30950| Symbols: FTSH2, VAR2 | VAR2 (VARIEGATED 2); ATP-dependent peptidase/ ATPase/ metal	
JCVI_23679	1.375	moderately similar to (207)AT1G58080| Symbols: HISN1A, ATATP-PRT1 | ATATP-PRT1 (ATP PHOSPHORIBOSYL TRANSFERASE	
CD832209	1.375	moderately similar to (293)AT5G47750| Symbols: | protein kinase, putative | chr5:19357173-19359090 REVERSEmoderately similar to (	
CX269557	1.375	weakly similar to (112)AT3G09730| Symbols: | similar to unnamed protein product [Vitis vinifera] (GB:CAO18124.1) | chr3:2984023-298	
EE505133	1.375	no similarity	
JCVI_9494	1.374	weakly similar to (177)AT3G52740| Symbols: | similar to unknown protein [Arabidopsis thaliana] (TAIR:AT3G44450.1); similar to unna	
JCVI_33445	1.374	no original description	
EV108960	1.374	no similarity	
JCVI_13927	1.374	moderately similar to (423)AT4G15520| Symbols: | tRNA/rRNA methyltransferase (SpoU) family protein | chr4:8862827-8864364 FORW	
JCVI_3651	1.374	weakly similar to (155)AT2G27330| Symbols: | RNA recognition motif (RRM)-containing protein | chr2:11702428-11703641 REVERSE	
ES952618	1.374	weakly similar to (101)AT2G45200| Symbols: ATGOS12, GOS12 | GOS12 (GOLGI SNARE 12); SNARE binding | chr2:18644763-18646	
JCVI_11456	1.374	weakly similar to (176)AT2G37250| Symbols: ADK, ATPADK1 | ADK/ATPADK1 (ADENOSINE KINASE); adenylate kinase/ nucleotid	
DY024374	1.374	weakly similar to (146)AT3G03305| Symbols: | calcineurin-like phosphoesterase family protein | chr3:775511-778278 REVERSE [18971]	
JCVI_22931	1.374	weakly similar to (155)AT3G08020| Symbols: | protein binding / zinc ion binding | chr3:2557759-2561540 REVERSE no original descript	
JCVI_35104	1.374	weakly similar to (177)AT1G72210| Symbols: | basic helix-loop-helix (bHLH) family protein (bHLH096) | chr1:27183728-27185930 FOR	
EE511184	1.374	moderately similar to (373)AT5G46920| Symbols: | intron maturase, type II family protein | chr5:19070895-19073102 FORWARD [1571	
JCVI_17680	1.374	moderately similar to (251)AT2G35190| Symbols: ATNPSN11, NSPN11, NPSN11 | NPSN11 (NOVEL PLANT SNARE 11); protein trans	-2.680
RC_CX192077	1.373	no similarity	
EE566379	1.373	moderately similar to (238)AT5G65560| Symbols: | pentatricopeptide (PPR) repeat-containing protein | chr5:26218238-26220985 REVER	
EV155393	1.373	moderately similar to (205)AT1G32120| Symbols: | similar to unknown protein [Arabidopsis thaliana] (TAIR:AT4G16050.1); similar to u	
EE518187	1.373	no similarity	
EV123899	1.373	moderately similar to (211)AT3G62600| Symbols: | DNAJ heat shock family protein | chr3:23162014-23164322 REVERSE [21479] 26 75	1.661
JCVI_16476	1.373	moderately similar to (396)AT4G35600| Symbols: CONNEXIN 32 | CONNEXIN 32; kinase | chr4:16896453-16898719 FORWARDweak	1.374
JCVI_40169	1.373	highly similar to (595)AT4G15530| Symbols: PPDK | PPDK (PYRUVATE ORTHOPHOSPHATE DIKINASE) | chr4:8864826-8870746 F	
EX039922	1.373	no similarity	
JCVI_33031	1.373	moderately similar to (297)AT3G28690| Symbols: | protein kinase, putative | chr3:10756718-10758731 FORWARD no original descriptio	
EV205119	1.373	no similarity	
EL589686	1.373	very weakly similar to (86.7)AT2G14288| Symbols: | similar to kelch repeat-containing F-box family protein [Arabidopsis thaliana] (TAIR	
JCVI_41446	1.373	weakly similar to (190)AT5G15740| Symbols: | similar to unknown protein [Arabidopsis thaliana] (TAIR:AT3G02250.1); similar to hypot	
JCVI_16539	1.373	highly similar to (686)AT5G65700| Symbols: BAM1 | BAM1 (big apical meristem 1); ATP binding / kinase/ protein serine/threonine kinas	
JCVI_21448	1.372	moderately similar to (338)AT5G48250| Symbols: | zinc finger (B-box type) family protein | chr5:19578945-19580339 REVERSE no orig	
JCVI_24946	1.372	highly similar to (513)AT3G56370| Symbols: | leucine-rich repeat transmembrane protein kinase, putative | chr3:20910382-20913369 RE	
ES913878	1.372	no similarity	
JCVI_36848	1.372	moderately similar to (250)AT2G37150| Symbols: | zinc finger (C3HC4-type RING finger) family protein | chr2:15610827-15613067 RE	2.761
JCVI_17890	1.372	moderately similar to (225)AT4G27830| Symbols: | glycosyl hydrolase family 1 protein | chr4:13861800-13864495 REVERSE no original	
JCVI_26523	1.372	moderately similar to (474)AT2G28680| Symbols: | cupin family protein | chr2:12310195-12311824 REVERSE no original description	
JCVI_10339	1.372	weakly similar to (185)AT4G21390| Symbols: B120 | B120; protein kinase/ sugar binding | chr4:11394469-11397485 REVERSE no origin	
JCVI_34856	1.372	very weakly similar to (82.0)AT5G67350| Symbols: | unknown protein | chr5:26885747-26886655 REVERSE no original description	
JCVI_2060	1.372	moderately similar to (221)AT1G27300| Symbols: | similar to unnamed protein product [Vitis vinifera] (GB:CAO66149.1) | chr1:9483311	
JCVI_37557	1.372	weakly similar to (135)AT1G22090| Symbols: EMB2204 | EMB2204 (EMBRYO DEFECTIVE 2204) | chr1:7795710-7797241 FORWAR	
EE480264	1.372	moderately similar to (244)AT5G55840| Symbols: | pentatricopeptide (PPR) repeat-containing protein | chr5:22615624-22619725 FORW	
RC_ES964857	1.371	no similarity	-1.543
JCVI_32595	1.371	moderately similar to (266)AT4G32140| Symbols: | similar to membrane protein [Arabidopsis thaliana] (TAIR:AT3G07080.1); similar to	
JCVI_16447	1.371	weakly similar to (105)AT4G29550| Symbols: | similar to unknown protein [Arabidopsis thaliana] (TAIR:AT2G20620.1); contains InterP	
EV032692	1.371	very weakly similar to (92.4)AT1G17690| Symbols: | similar to unnamed protein product [Vitis vinifera] (GB:CAO64063.1); contains Inte	
JCVI_26672	1.371	moderately similar to (307)AT3G20550| Symbols: DDL | DDL (DAWDLE) | chr3:7174701-7177606 REVERSE no original description	
JCVI_21789	1.371	moderately similar to (206)AT2G44200| Symbols: | similar to unknown protein [Arabidopsis thaliana] (TAIR:AT2G44195.1); similar to h	-1.454
AM385381	1.370	weakly similar to (136)AT2G23450| Symbols: | protein kinase family protein | chr2:9996006-9998324 REVERSE [20118]	
JCVI_34443	1.370	moderately similar to (353)AT1G07110| Symbols: ATF2KP, FKFBP, F2KP | F2KP (FRUCTOSE-2,6-BISPHOSPHATASE); fructose-2,6-	
JCVI_9001	1.370	moderately similar to (321)AT5G52840| Symbols: | NADH-ubiquinone oxidoreductase-related | chr5:21430944-21432020 FORWARD nc	
JCVI_39575	1.370	very weakly similar to (85.1)AT1G10000| Symbols: | nucleic acid binding / ribonuclease H | chr1:3263879-3264790 REVERSE no original	
JCVI_29143	1.370	moderately similar to (341)AT3G04740| Symbols: MED14, SWP | SWP (STRUWWELPETER) | chr3:1294043-1300561 FORWARD no c	
JCVI_27501	1.370	moderately similar to (250)AT1G13120| Symbols: EMB1745 | EMB1745 (EMBRYO DEFECTIVE 1745) | chr1:4469332-4472773 REVE	
EX131964	1.370	highly similar to (542)AT4G30100| Symbols: | tRNA-splicing endonuclease positive effector-related | chr4:14714197-14719341 FORWA	
EV003564	1.370	no similarity	
EV152022	1.370	no similarity	
JCVI_18533	1.370	highly similar to (552)AT3G63500| Symbols: | similar to unknown protein [Arabidopsis thaliana] (TAIR:AT1G14740.1); similar to unnam	
JCVI_17949	1.370	weakly similar to (192)AT3G50690| Symbols: | leucine-rich repeat family protein | chr3:18846252-18848078 REVERSEweakly similar to	
JCVI_12526	1.370	moderately similar to (273)AT3G62310| Symbols: | RNA helicase, putative | chr3:23068491-23071536 REVERSE no original description	
EV208376	1.370	no similarity	
BQ705072	1.369	weakly similar to (124)AT4G00585| Symbols: | similar to hypothetical protein MtrDRAFT_AC148817g12v2 [Medicago truncatula] (GB:	
JCVI_17768	1.369	moderately similar to (206)AT2G43500| Symbols: | RWP-RK domain-containing protein | chr2:18069792-18073527 FORWARD no origi	
JCVI_40178	1.369	moderately similar to (321)AT5G24120| Symbols: SIG5, SIGE | SIGE (RNA polymerase sigma subunit E); DNA binding / DNA-directed	
JCVI_34518	1.369	highly similar to (665)AT3G14690| Symbols: CYP72A15 | CYP72A15 (cytochrome P450, family 72, subfamily A, polypeptide 15); oxyge	
EV087790	1.369	weakly similar to (105)AT2G26540| Symbols: HEMD | HEMD; uroporphyrinogen-III synthase | chr2:11294744-11297302 REVERSE [21	
JCVI_33618	1.369	moderately similar to (457)AT4G35160| Symbols: | O-methyltransferase family 2 protein | chr4:16730994-16732813 REVERSEweakly si	
JCVI_37758	1.369	moderately similar to (405)AT3G12010| Symbols: | similar to Colon cancer-associated protein Mic1-like containing protein, expressed [O	-1.474
EE459238	1.369	moderately similar to (266)AT4G04320| Symbols: | malonyl-CoA decarboxylase family protein | chr4:2113563-2116523 FORWARD [20	
JCVI_9978	1.369	moderately similar to (226)AT3G18030| Symbols: HAL3A, HAL3, ATHAL3, ATHAL3A | ATHAL3A (Arabidopsis thaliana Hal3-like pro	
EV146186	1.369	no similarity	
AM395004	1.369	weakly similar to (124)AT1G70840| Symbols: MLP31 | MLP31 (MLP-LIKE PROTEIN 31) | chr1:26716832-26717676 REVERSE [2034	
JCVI_2273	1.369	weakly similar to (159)AT5G03740| Symbols: HDT3, HD2C | HD2C (HISTONE DEACETYLASE 2C); nucleic acid binding / zinc ion bin	
EX059435	1.369	very weakly similar to (86.3)AT5G18780| Symbols: | F-box family protein | chr5:6264612-6266099 FORWARD [21813]	
EV152290	1.368	no similarity	-1.717
EE480911	1.368	weakly similar to (180)AT5G01940| Symbols: | eukaryotic translation initiation factor 2B family protein / eIF-2B family protein | chr5:363	
H07446	1.368	no similarity	1.751
JCVI_23131	1.368	weakly similar to (174)AT2G17220| Symbols: | protein kinase, putative | chr2:7494948-7496850 REVERSE no original description	

JCVI_11899	1.368	weakly similar to (165)AT5G52220| Symbols: | similar to unnamed protein product [Vitis vinifera] (GB:CAO15518.1); similar to hypothe	-2.103
EV030136	1.368	weakly similar to (125)AT1G74510| Symbols: | kelch repeat-containing F-box family protein | chr1:28009726-28011081 FORWARD [214	-1.508
EV022199	1.368	moderately similar to (252)AT2G31270| Symbols: ATCDT1A, CDT1A, CDT1 | ATCDT1A/CDT1/CDT1A (ARABIDOPSIS HOMOLOG	
EE530972	1.368	no similarity	3.652
EV096191	1.368	no similarity	-1.685
EE438830	1.368	no similarity	
JCVI_27029	1.368	no original description	
JCVI_39498	1.368	moderately similar to (330)AT1G05580| Symbols: CHX23, ATCHX23 | ATCHX23 (CATION/H+ EXCHANGER 23); monovalent cation:	
CX195240	1.368	moderately similar to (421)AT2G16920| Symbols: UBC23, PFU2 | PFU2/UBC23 (UBIQUITIN-CONJUGATING ENZYME 23); ubiquiti	
JCVI_40607	1.367	weakly similar to (156)AT1G63090| Symbols: ATPP2-A11 | ATPP2-A11 (Phloem protein 2-A11); carbohydrate binding | chr1:23394948-	
CV433143	1.367	moderately similar to (223)AT4G08500| Symbols: ATMEKK1, MAPKKK8, MEKK1 | MEKK1 (MYTOGEN ACTIVATED PROTEIN K	
JCVI_381	1.367	moderately similar to (317)AT4G08460| Symbols: | protein binding / zinc ion binding | chr4:5377375-5378199 REVERSE no original desc	
EV226525	1.367	no similarity	
EV163383	1.366	moderately similar to (238)AT2G26600| Symbols: | glycosyl hydrolase family 17 protein | chr2:11324292-11325293 FORWARDweakly s	
JCVI_8350	1.366	weakly similar to (195)AT3G05350| Symbols: | aminopeptidase | chr3:1527109-1530001 REVERSE no original description	
JCVI_21758	1.366	moderately similar to (456)AT5G19380| Symbols: | similar to unknown protein [Arabidopsis thaliana] (TAIR:AT5G12170.2); similar to u	
JCVI_37969	1.366	moderately similar to (285)AT2G20635| Symbols: | similar to spindle checkpoint protein-related [Arabidopsis thaliana] (TAIR:AT2G3356	
JCVI_34641	1.366	weakly similar to (171)AT1G22410| Symbols: | 2-dehydro-3-deoxyphosphoheptonate aldolase, putative / 3-deoxy-D-arabino-heptulosonat	
EL589940	1.366	weakly similar to (108)AT1G14800| Symbols: | similar to F-box family protein [Arabidopsis thaliana] (TAIR:AT2G35280.1); similar to hy	
JCVI_11491	1.366	moderately similar to (250)AT1G15220| Symbols: ATCCMH | ATCCMH | chr1:5240466-5241112 REVERSE no original description	
AT002111	1.365	weakly similar to (160)AT1G12200| Symbols: | flavin-containing monooxygenase family protein / FMO family protein | chr1:4137625-41	
JCVI_14856	1.365	moderately similar to (382)AT5G07460| Symbols: PMSR2 | PMSR2 (PEPTIDEMETHIONINE SULFOXIDE REDUCTASE 2); peptide-m	
JCVI_6440	1.365	weakly similar to (157)AT4G00270| Symbols: | DNA-binding storekeeper protein-related | chr4:117477-118468 REVERSE no original de	
CD828119	1.365	no similarity	
EV153540	1.365	weakly similar to (106)AT1G68020| Symbols: TPS6, ATTPS6 | ATTPS6 (Arabidopsis thaliana trehalose phosphatase/synthase 6); trehalos	
EX090880	1.365	weakly similar to (159)AT1G51690| Symbols: ATB ALPHA | ATB ALPHA (protein phosphatase 2A 55 kDa regulatory subunit B alpha is	
JCVI_19132	1.365	moderately similar to (263)AT2G38290| Symbols: ATAMT2 | ATAMT2 (AMMONIUM TRANSPORTER 2); ammonium transmembrane	
EX118783	1.365	moderately similar to (313)AT4G16850| Symbols: | similar to unknown protein [Arabidopsis thaliana] (TAIR:AT1G31130.1); similar to h	
JCVI_23755	1.365	weakly similar to (145)AT5G38660| Symbols: APE1 | APE1 (ACCLIMATION OF PHOTOSYNTHESIS TO ENVIRONMENT) | chr5:15	
CD833447	1.365	no similarity	
JCVI_13940	1.364	moderately similar to (295)AT4G27080| Symbols: ATPDIL5-4 | ATPDIL5-4 (PDI-LIKE 5-4) | chr4:13589162-13593341 FORWARD no c	
CD820185	1.364	weakly similar to (186)AT3G55890| Symbols: | yippee family protein | chr3:20751887-20752847 FORWARDweakly similar to (154)YIP	
JCVI_11044	1.364	weakly similar to (141)AT5G36120| Symbols: | YGGT family protein | chr5:14215909-14216433 REVERSE no original description	
JCVI_23270	1.364	moderately similar to (413)AT2G05590| Symbols: | similar to unknown protein [Arabidopsis thaliana] (TAIR:AT4G39870.1); similar to u	
EH429329	1.364	no similarity	
EX040095	1.364	moderately similar to (241)AT1G75010| Symbols: | GTP binding / GTPase | chr1:28168655-28173602 REVERSE [21811]	
AM390780	1.364	moderately similar to (347)AT1G62680| Symbols: | binding | chr1:23211912-23213558 REVERSEweakly similar to (159)RF1_ORYSA [	
JCVI_40351	1.363	moderately similar to (223)AT5G41580| Symbols: | zinc ion binding | chr5:16643791-16647887 REVERSE no original description	
BG544852	1.363	no similarity	
JCVI_5321	1.363	moderately similar to (390)AT3G21865| Symbols: PEX22 | PEX22 (PEROXIN 22); protein binding | chr3:7701315-7703225 REVERSE n	
ES982884	1.363	weakly similar to (122)AT4G16390| Symbols: | chloroplastic RNA-binding protein P67, putative | chr4:9258050-9260116 FORWARD [2	
JCVI_13909	1.363	moderately similar to (276)AT3G09740| Symbols: ATSYP71, SYP71 | SYP71 (SYNTAXIN OF PLANTS 71) | chr3:2989620-2991359 FC	
JCVI_19163	1.363	no original description	
JCVI_38826	1.363	moderately similar to (205)AT2G48130| Symbols: | protease inhibitor/seed storage/lipid transfer protein (LTP) family protein | chr2:19692	
ES900913	1.362	weakly similar to (196)AT3G49200| Symbols: | similar to unknown protein [Arabidopsis thaliana] (TAIR:AT3G49210.1); similar to conde	
JCVI_35973	1.362	moderately similar to (325)AT5G54520| Symbols: | WD-40 repeat family protein | chr5:22164007-22166315 REVERSE no original descr	
JCVI_16582	1.362	moderately similar to (230)AT5G58310| Symbols: | hydrolase, alpha/beta fold family protein | chr5:23592353-23593233 REVERSE no or	
JCVI_7719	1.362	highly similar to (548)AT1G09420| Symbols: G6PD4 | G6PD4 (GLUCOSE-6-PHOSPHATE DEHYDROGENASE 4); glucose-6-phosphat	
JCVI_33385	1.362	moderately similar to (265)AT4G16580| Symbols: | catalytic | chr4:9341174-9342577 REVERSE no original description	
JCVI_32207	1.362	no original description	
JCVI_7802	1.362	moderately similar to (214)AT5G61130| Symbols: | glycosyl hydrolase family protein 17 | chr5:24604664-24606595 REVERSEvery weak	
JCVI_22030	1.362	weakly similar to (170)AT1G55830| Symbols: | similar to unnamed protein product [Vitis vinifera] (GB:CAO45172.1); contains InterPro	
EX114916	1.362	moderately similar to (232)AT3G62970| Symbols: | protein binding / zinc ion binding | chr3:23281611-23283673 FORWARD [21835]	
JCVI_11382	1.362	moderately similar to (226)AT2G41475| Symbols: | similar to embryo-specific protein-related [Arabidopsis thaliana] (TAIR:AT5G62200.	
JCVI_25036	1.362	moderately similar to (280)AT1G49980| Symbols: | similar to REV1 (Reversionless 1), damaged DNA binding / magnesium ion binding /	
EV051095	1.362	moderately similar to (245)AT5G22460| Symbols: | esterase/lipase/thioesterase family protein | chr5:7443662-7445272 REVERSE [21442	
JCVI_3909	1.362	highly similar to (815)AT3G15730| Symbols: PLD, PLDALPHA1 | PLDALPHA1 (PHOSPHOLIPASE D ALPHA 1); phospholipase D | ch	
CV546037	1.362	no similarity	
EX047134	1.361	moderately similar to (332)AT5G51950| Symbols: | glucose-methanol-choline (GMC) oxidoreductase family protein | chr5:21123319-211	
JCVI_36529	1.361	highly similar to (669)AT2G32480| Symbols: | membrane-associated zinc metalloprotease, putative | chr2:13795756-13797099 REVERSE	
JCVI_33369	1.361	moderately similar to (293)AT5G08750| Symbols: | zinc finger (C3HC4-type RING finger) family protein | chr5:2852913-2854344 FORW	
EV096453	1.361	weakly similar to (125)AT5G01220| Symbols: SQD2 | SQD2 (SULFOQUINOVOSYLDIACYLGLYCEROL 2); UDP-sulfoquinovose:DAC	-1.595
EV045594	1.361	moderately similar to (295)AT1G80870| Symbols: | protein kinase family protein | chr1:30397025-30399103 FORWARDvery weakly sim	
EV051426	1.361	no similarity	
EE526248	1.361	no similarity	
JCVI_5062	1.360	moderately similar to (267)AT5G08290| Symbols: YLS8 | YLS8 (yellow-leaf-specific gene 8); catalytic | chr5:2666044-2666937 FORWA	-1.265
JCVI_4676	1.360	weakly similar to (123)AT5G50110| Symbols: | methyltransferase-related | chr5:20391428-20393294 FORWARD no original description	
JCVI_6130	1.360	no original description	2.047
JCVI_7925	1.360	weakly similar to (166)AT1G03170| Symbols: | similar to unknown protein [Arabidopsis thaliana] (TAIR:AT4G02810.1); similar to unna	
EV223084	1.360	weakly similar to (108)AT3G28430| Symbols: | similar to unnamed protein product [Vitis vinifera] (GB:CAO69571.1); similar to Os04g0	
JCVI_13196	1.360	highly similar to (632)AT4G01070| Symbols: GT72B1 | GT72B1; UDP-glucosyltransferase/ UDP-glycosyltransferase/ transferase, transfe	
JCVI_41987	1.360	no original description	
JCVI_39714	1.360	no original description	
JCVI_1837	1.360	moderately similar to (392)AT1G62740| Symbols: | stress-inducible protein, putative | chr1:23234691-23237045 FORWARDmoderately s	
JCVI_41134	1.359	moderately similar to (448)AT1G05120| Symbols: | SNF2 domain-containing protein / helicase domain-containing protein / RING finger c	
EX101325	1.359	weakly similar to (106)AT5G59840| Symbols: | Ras-related GTP-binding family protein | chr5:24124676-24126275 REVERSEweakly sim	
ES900916	1.359	no similarity	
ES955671	1.359	no similarity	
EX067380	1.359	no similarity	
EX124800	1.359	weakly similar to (185)AT2G42975| Symbols: | similar to unnamed protein product [Vitis vinifera] (GB:CAO69996.1) | chr2:17881075-17	
JCVI_11635	1.359	weakly similar to (149)AT3G10860| Symbols: | ubiquinol-cytochrome C reductase complex ubiquinone-binding protein, putative / ubiquir	

JCVI_40889	1.358	weakly similar to (176)AT3G60860| Symbols: | guanine nucleotide exchange family protein | chr3:22495779-22502485 FORWARD no or	
JCVI_30370	1.358	highly similar to (549)AT2G02170| Symbols: | remorin family protein | chr2:556594-558609 REVERSE no original description	
JCVI_42341	1.358	moderately similar to (441)AT3G07540| Symbols: | formin homology 2 domain-containing protein / FH2 domain-containing protein | chr3	-2.737
CD828398	1.358	moderately similar to (361)AT3G53960| Symbols: | proton-dependent oligopeptide transport (POT) family protein | chr3:19989284-19991	
JCVI_26549	1.358	no original description	
JCVI_7796	1.358	moderately similar to (239)AT2G29020| Symbols: | Rab5-interacting family protein | chr2:12476946-12478505 FORWARD no original de	
JCVI_27238	1.358	very weakly similar to (92.4)AT3G26000| Symbols: | F-box family protein | chr3:9508279-9509779 REVERSE no original description	
JCVI_14440	1.358	highly similar to (874)AT2G45220| Symbols: | pectinesterase family protein | chr2:18651355-18653468 REVERSEhighly similar to (625)	
JCVI_23295	1.358	weakly similar to (112)AT5G13710| Symbols: CPH, SMT1 | SMT1 (STEROL METHYLTRANSFERASE 1) | chr5:4424051-4426869 REV	
JCVI_12455	1.358	weakly similar to (130)AT4G31880| Symbols: | binding | chr4:15419441-15423945 REVERSE no original description	
JCVI_10181	1.358	no original description	
JCVI_38424	1.357	no original description	
JCVI_11719	1.357	no original description	
EV060128	1.357	no similarity	
JCVI_29433	1.357	moderately similar to (478)AT5G02820| Symbols: BIN5, RHL2 | RHL2 (ROOT HAIRLESS 2); ATP binding / DNA binding / DNA topois	
EV110047	1.357	no similarity	
DY028174	1.357	no similarity	
JCVI_31736	1.357	moderately similar to (275)AT2G02040| Symbols: PTR2-B, NTR1, ATPTR2-B | ATPTR2-B (NITRATE TRANSPORTER 1); transporter	
EV055720	1.357	very weakly similar to (95.9)AT3G58030| Symbols: | zinc finger (C3HC4-type RING finger) family protein | chr3:21496504-21497814 FO	
EE425447	1.357	moderately similar to (286)AT5G11640| Symbols: | similar to unnamed protein product [Vitis vinifera] (GB:CAO44011.1); contains Inter	
JCVI_8740	1.356	highly similar to (538)AT4G04770| Symbols: LAF6, ATNAP1, ATABC1 | ATABC1 (ARABIDOPSIS THALIANA NUCLEOSOME ASS	
JCVI_8117	1.356	moderately similar to (362)AT3G55030| Symbols: PGPS2 | PGPS2 (phosphatidylglycerolphosphate synthase 2); CDP-alcohol phosphatidy	
AM395693	1.356	weakly similar to (122)AT1G32150| Symbols: | bZIP transcription factor family protein | chr1:11566002-11568297 FORWARD [20346]	
EE539675	1.356	weakly similar to (179)AT3G53470| Symbols: | similar to unnamed protein product [Vitis vinifera] (GB:CAO18045.1) | chr3:19833791-19	
JCVI_39247	1.356	no original description	
JCVI_40891	1.356	moderately similar to (347)AT5G41800| Symbols: | amino acid transporter family protein | chr5:16751070-16753116 FORWARD no orig	
EV152269	1.355	no similarity	
CD812150	1.355	no similarity	-1.936
EE470819	1.355	moderately similar to (377)AT3G13772| Symbols: | endomembrane protein 70, putative | chr3:4521719-4524401 REVERSE [20163]	
JCVI_15898	1.355	moderately similar to (372)AT4G24940| Symbols: AT-SAE1-1, ATSAE1A, SAE1A | SAE1A (SUMO-ACTIVATING ENZYME 1A); SUI	
EV174719	1.355	no similarity	
JCVI_234	1.355	no original description	
JCVI_16212	1.355	weakly similar to (147)AT3G04930| Symbols: | transcription regulator | chr3:1363035-1364405 FORWARD no original description	
EV134065	1.355	no similarity	
JCVI_10481	1.355	moderately similar to (340)AT1G29820| Symbols: | similar to unknown protein [Arabidopsis thaliana] (TAIR:AT1G29830.2); similar to u	
JCVI_1009	1.354	moderately similar to (385)AT2G33470| Symbols: GLTP1 | GLTP1 (GLYCOLIPID TRANSFER PROTEIN 1); glycolipid binding / glycol	
EX063204	1.354	moderately similar to (368)AT1G07650| Symbols: | leucine-rich repeat transmembrane protein kinase, putative | chr1:2359814-2366420 R	
JCVI_26285	1.354	weakly similar to (110)AT1G24360| Symbols: | 3-oxoacyl-(acyl-carrier protein) reductase, chloroplast / 3-ketoacyl-acyl carrier protein red	
JCVI_27487	1.354	moderately similar to (284)AT4G00755| Symbols: | F-box family protein | chr4:325294-326787 FORWARD no original description	-2.000
EE526521	1.354	no similarity	
ES987411	1.354	no similarity	
EX111142	1.354	moderately similar to (219)AT1G68840| Symbols: RAP2.8, RAV2 | RAV2 (REGULATOR OF THE ATPASE OF THE VACUOLAR ME	
EE527736	1.354	moderately similar to (239)AT4G02500| Symbols: ATXT2 | ATXT2; UDP-xylosyltransferase/ transferase/ transferase, transferring glycos	
EV190831	1.354	weakly similar to (182)AT1G77490| Symbols: TAPX | TAPX; L-ascorbate peroxidase | chr1:29122582-29124940 FORWARDweakly simi	1.464
JCVI_27880	1.354	weakly similar to (198)AT2G47880| Symbols: | glutaredoxin family protein | chr2:19612194-19612502 FORWARD no original descriptio	
JCVI_41011	1.353	weakly similar to (169)AT2G33585| Symbols: | similar to unnamed protein product [Vitis vinifera] (GB:CAO66293.1) | chr2:14230479-14	
JCVI_4452	1.353	moderately similar to (364)AT1G72330| Symbols: ALAAT2 | ALAAT2 (ALANINE AMINOTRANSFERASE 2) | chr1:27237299-2723985	
CX270194	1.353	moderately similar to (250)AT3G11940| Symbols: AML1, ATRPS5A | ATRPS5A (RIBOSOMAL PROTEIN 5A); structural constituent of	-1.640
JCVI_32417	1.353	moderately similar to (367)AT3G63290| Symbols: | similar to unknown protein [Arabidopsis thaliana] (TAIR:AT4G13400.1); similar to u	
AM391363	1.353	moderately similar to (333)AT2G32730| Symbols: | 26S proteasome regulatory subunit, putative | chr2:13887266-13892541 FORWARD [	
JCVI_40489	1.353	no original description	
EE472198	1.353	weakly similar to (128)AT4G05390| Symbols: ATRFNR1 | ATRFNR1 (ROOT FNR 1); oxidoreductase | chr4:2738836-2740480 REVERS	
JCVI_32708	1.352	no original description	-1.673
ES976891	1.352	no similarity	
EL590014	1.352	no similarity	
ES969277	1.352	no similarity	
ES910915	1.352	moderately similar to (347)AT3G29680| Symbols: | transferase family protein | chr3:11533928-11535283 REVERSE [21430]	
EX126541	1.352	highly similar to (520)AT1G66830| Symbols: | leucine-rich repeat transmembrane protein kinase, putative | chr1:24934363-24936497 REV	
JCVI_38864	1.352	moderately similar to (429)AT3G52970| Symbols: CYP76G1 | CYP76G1 (cytochrome P450, family 76, subfamily G, polypeptide 1); oxyg	1.278
EX117702	1.352	no similarity	2.348
EV077920	1.352	no similarity	1.734
JCVI_35406	1.352	moderately similar to (293)AT3G26750| Symbols: | similar to unnamed protein product [Vitis vinifera] (GB:CAO48317.1) | chr3:9841376	
EE418890	1.352	no similarity	2.645
EG019750	1.351	no similarity	
JCVI_34972	1.351	weakly similar to (120)AT1G14450| Symbols: | similar to unknown protein [Arabidopsis thaliana] (TAIR:AT2G02510.1); similar to unkno	
JCVI_28136	1.351	moderately similar to (246)AT2G40935| Symbols: | similar to unknown protein [Arabidopsis thaliana] (TAIR:AT3G18470.1); similar to h	
EE508257	1.351	very weakly similar to (95.9)AT2G15000| Symbols: | similar to unknown protein [Arabidopsis thaliana] (TAIR:AT4G34265.2); similar to u	
JCVI_24332	1.351	no original description	
JCVI_18497	1.351	moderately similar to (253)AT4G33690| Symbols: | similar to hypothetical protein [Vitis vinifera] (GB:CAN61243.1) | chr4:16175201-16	
ES975701	1.351	no similarity	
JCVI_20404	1.351	moderately similar to (249)AT2G43180| Symbols: | catalytic | chr2:17960909-17962851 REVERSE no original description	
JCVI_9624	1.351	weakly similar to (182)AT4G12230| Symbols: | esterase/lipase/thioesterase family protein | chr4:7284639-7287342 FORWARD no origina	
JCVI_442	1.350	moderately similar to (273)AT4G02530| Symbols: | chloroplast thylakoid lumen protein | chr4:1112335-1114005 REVERSE no original de	
JCVI_34742	1.350	moderately similar to (359)AT2G37980| Symbols: | similar to unknown protein [Arabidopsis thaliana] (TAIR:AT5G01100.1); similar to u	
JCVI_18569	1.350	moderately similar to (322)AT4G31460| Symbols: | ribosomal protein L28 family protein | chr4:15259779-15260853 REVERSE no origin	
JCVI_19811	1.350	weakly similar to (106)AT1G54440| Symbols: | 3'-5' exonuclease/ nucleic acid binding | chr1:20326951-20331598 FORWARD no origina	
EH423475	1.350	moderately similar to (425)AT1G73430| Symbols: | sec34-like family protein | chr1:27607757-27614490 FORWARD [20767]	
JCVI_35570	1.350	moderately similar to (293)AT5G09640| Symbols: SCPL19, SNG2 | SNG2 (SINAPOYLGLUCOSE ACCUMULATOR 2); serine carboxyp	
JCVI_21424	1.350	weakly similar to (125)AT1G80660| Symbols: AHA9 | AHA9 (Arabidopsis H(+)-ATPase 9); hydrogen-exporting ATPase, phosphorylative	
JCVI_27995	1.350	highly similar to (555)AT1G61900| Symbols: | Identical to Uncharacterized GPI-anchored protein At1g61900 precursor [Arabidopsis Tha	
JCVI_25706	1.350	moderately similar to (268)AT2G43500| Symbols: | RWP-RK domain-containing protein | chr2:18069792-18073527 FORWARD no origi	

JCVI_6738	1.350	highly similar to (559)AT4G28320| Symbols: | glycosyl hydrolase family 5 protein / cellulase family protein | chr4:14018299-14019978 R	
ES938220	1.350	moderately similar to (320)AT5G08415| Symbols: | lipoic acid synthase family protein | chr5:2710984-2713102 REVERSE [21390]	
EE532644	1.350	no similarity	
JCVI_9386	1.350	moderately similar to (256)AT5G15770| Symbols: ATGNA1 | ATGNA1 (ARABIDOPSIS THALIANA GLUCOSE-6-PHOSPHATE ACE	
JCVI_2405	1.350	highly similar to (801)ATCG00480| Symbols: ATPB | chloroplast-encoded gene for beta subunit of ATP synthase | chrC:52660-54156 RE	
JCVI_17241	1.350	weakly similar to (141)AT5G13810| Symbols: | glutaredoxin family protein | chr5:4455772-4456596 FORWARD no original description	
JCVI_24585	1.350	weakly similar to (153)AT2G47700| Symbols: | zinc finger (C3HC4-type RING finger) family protein | chr2:19559576-19561421 REVER	
EV134109	1.349	no similarity	
JCVI_19622	1.349	highly similar to (572)AT4G28370| Symbols: | zinc ion binding | chr4:14035022-14039128 FORWARD no original description	-1.501
JCVI_22698	1.349	moderately similar to (436)AT5G55560| Symbols: | protein kinase family protein | chr5:22523703-22524983 REVERSEvery weakly simil	
EL586998	1.349	no similarity	
EE531563	1.349	moderately similar to (347)AT1G52540| Symbols: | protein kinase, putative | chr1:19573967-19575553 REVERSEweakly similar to (151	
JCVI_23770	1.349	very weakly similar to (83.2)AT5G16850| Symbols: ATTERT | ATTERT (TELOMERASE REVERSE TRANSCRIPTASE); telomeric tem	1.664
EV155473	1.349	weakly similar to (143)AT3G50660| Symbols: CYP90B1, CLM, SNP2, DWF4 | DWF4 (DWARF 4) | chr3:18825243-18828149 REVERSI	
JCVI_13865	1.349	moderately similar to (335)AT2G34730| Symbols: | myosin heavy chain-related | chr2:14661690-14664522 FORWARD no original descri	
JCVI_8149	1.349	moderately similar to (443)AT5G15860| Symbols: ATPCME | ATPCME (PRENYLCYSTEINE METHYLESTERASE) | chr5:5179397-51	
JCVI_39052	1.349	highly similar to (644)AT3G52940| Symbols: HYD2, ELL1, FK | FK (FACKEL) | chr3:19642008-19644090 REVERSE no original descrip	
EV160627	1.348	moderately similar to (358)AT3G26840| Symbols: | esterase/lipase/thioesterase family protein | chr3:9894045-9897391 FORWARD [214	
EX092794	1.348	no similarity	
AM390320	1.348	moderately similar to (265)AT4G38550| Symbols: | similar to unknown protein [Arabidopsis thaliana] (TAIR:AT2G20950.1); contains In	
JCVI_37545	1.348	moderately similar to (239)AT5G08200| Symbols: | peptidoglycan-binding LysM domain-containing protein | chr5:2638386-2640509 FO	
JCVI_35418	1.348	no original description	
EV037964	1.348	moderately similar to (211)AT3G60240| Symbols: CUM2, EIF4G | EIF4G (EUKARYOTIC TRANSLATION INITIATION FACTOR 4G	
AM391991	1.348	moderately similar to (337)AT5G07270| Symbols: | ankyrin repeat family protein | chr5:2280822-2283385 FORWARD [20118]	1.577
JCVI_227	1.348	moderately similar to (333)AT2G38310| Symbols: | similar to unknown protein [Arabidopsis thaliana] (TAIR:AT5G05440.1); similar to u	
EV149711	1.348	no similarity	
EV008467	1.348	no similarity	
EE555851	1.347	very weakly similar to (99.0)AT2G38430| Symbols: | similar to unknown protein [Arabidopsis thaliana] (TAIR:AT3G54310.1); similar to u	
JCVI_39540	1.347	moderately similar to (254)AT2G32760| Symbols: | similar to unnamed protein product [Vitis vinifera] (GB:CAO50091.1); contains doma	
JCVI_12518	1.347	moderately similar to (249)AT5G53160| Symbols: | similar to unknown protein [Arabidopsis thaliana] (TAIR:AT1G01360.1); similar to u	
JCVI_14762	1.347	moderately similar to (456)AT4G20830| Symbols: | FAD-binding domain-containing protein | chr4:11155497-11157119 FORWARD no o	
JCVI_19569	1.347	moderately similar to (325)AT1G10840| Symbols: TIF3H1 | TIF3H1 (EUKARYOTIC TRANSLATION INITIATION FACTOR 3 SUBU	
EV079931	1.347	no similarity	
JCVI_37920	1.347	moderately similar to (409)AT1G72300| Symbols: | leucine-rich repeat transmembrane protein kinase, putative | chr1:27221341-2722462	
EE544205	1.347	weakly similar to (142)AT5G65810| Symbols: | similar to unknown protein [Arabidopsis thaliana] (TAIR:AT3G49720.1); similar to unkn	
JCVI_656	1.346	moderately similar to (364)AT2G29450| Symbols: ATGSTU1, AT103-1A, ATGSTU5 | ATGSTU5 (Arabidopsis thaliana Glutathione S-tra	
JCVI_1992	1.346	highly similar to (608)AT3G63140| Symbols: | mRNA-binding protein, putative | chr3:23337981-23339595 REVERSE no original descrip	
JCVI_35239	1.346	weakly similar to (160)AT5G59960| Symbols: | similar to unnamed protein product [Vitis vinifera] (GB:CAO21698.1) | chr5:24159578-24	
JCVI_6662	1.346	highly similar to (669)AT5G66680| Symbols: DGL1 | DGL1 (defective glycosylation 1); dolichyl-diphosphooligosaccharide-protein glycot	
BQ704998	1.346	weakly similar to (154)AT3G08960| Symbols: | protein transporter | chr3:2730410-2736767 REVERSE [11009] 245 684 701	
CV432949	1.346	no similarity	
JCVI_15595	1.346	weakly similar to (185)AT5G66730| Symbols: | zinc finger (C2H2 type) family protein | chr5:26659140-26661109 REVERSE no original	1.241
JCVI_6865	1.345	weakly similar to (179)AT3G25120| Symbols: | mitochondrial import inner membrane translocase subunit Tim17/Tim22/Tim23 family pr	
JCVI_12167	1.345	moderately similar to (461)AT5G10300| Symbols: | hydrolase, alpha/beta fold family protein | chr5:3239685-3240725 FORWARDmodera	
JCVI_2789	1.345	very weakly similar to (99.4)AT5G13360| Symbols: | auxin-responsive GH3 family protein | chr5:4283808-4286123 FORWARDweakly si	1.547
ES902594	1.345	moderately similar to (432)AT3G08800| Symbols: | binding | chr3:2671154-2674916 FORWARD [21428]	
JCVI_13124	1.345	weakly similar to (197)AT4G24590| Symbols: | similar to unknown protein [Arabidopsis thaliana] (TAIR:AT5G49710.3); similar to unna	
JCVI_14107	1.345	moderately similar to (257)AT5G51120| Symbols: PABN1, ATPABN1 | ATPABN1/PABN1 (polyadenylate-binding protein 1); RNA bind	
AM057330	1.345	moderately similar to (369)AT2G17020| Symbols: | F-box family protein (FBL10) | chr2:7403641-7405869 REVERSE [17712]	
EX087572	1.345	moderately similar to (296)AT5G21970| Symbols: | similar to unknown protein [Arabidopsis thaliana] (TAIR:AT4G01037.1); similar to u	1.411
EV081280	1.345	no similarity	
JCVI_15018	1.345	weakly similar to (109)AT4G29440| Symbols: | similar to unknown protein [Arabidopsis thaliana] (TAIR:AT2G19710.1); similar to unna	
JCVI_361	1.345	highly similar to (509)AT1G68620| Symbols: | hydrolase | chr1:25769681-25770691 FORWARDweakly similar to (119)GID1_ORYSA r	
JCVI_7160	1.345	weakly similar to (103)AT4G37900| Symbols: | glycine-rich protein | chr4:17821731-17824439 REVERSE no original description	
JCVI_38524	1.345	moderately similar to (311)AT5G64360| Symbols: | DNAJ heat shock N-terminal domain-containing protein | chr5:25754434-25755828 R	
EV013466	1.344	weakly similar to (105)AT1G22250| Symbols: | similar to unknown protein [Arabidopsis thaliana] (TAIR:AT1G78170.1); similar to unna	
CD830308	1.344	no similarity	
JCVI_34227	1.344	very weakly similar to (92.8)AT3G56250| Symbols: | unknown protein | chr3:20877099-20878819 FORWARD no original description	
EX063127	1.344	moderately similar to (363)AT4G16390| Symbols: | chloroplastic RNA-binding protein P67, putative | chr4:9258050-9260116 FORWARI	-4.446
EV058576	1.344	moderately similar to (274)AT4G22390| Symbols: | F-box family protein-related | chr4:11813769-11815086 REVERSE [21442] 1 702 71	
JCVI_32993	1.344	weakly similar to (167)AT4G35940| Symbols: | similar to unknown protein [Arabidopsis thaliana] (TAIR:AT2G17787.1); similar to simil	
JCVI_41605	1.344	moderately similar to (426)AT3G19340| Symbols: | sodium:dicarboxylate symporter | chr3:6701393-6704077 REVERSE no original desc	
ES266112	1.343	weakly similar to (183)AT5G04250| Symbols: | OTU-like cysteine protease family protein | chr5:1176398-1178493 FORWARD [21031]	
ES922841	1.343	weakly similar to (143)AT3G51650| Symbols: | similar to unknown protein [Arabidopsis thaliana] (TAIR:AT3G51640.1); similar to unna	
JCVI_497	1.343	moderately similar to (402)AT1G09640| Symbols: | elongation factor 1B-gamma, putative / eEF-1B gamma, putative | chr1:3120164-3122	
ES937522	1.343	no similarity	
JCVI_11706	1.343	moderately similar to (270)AT2G26990| Symbols: ATCSN2, COP12, FUS12 | FUS12 (FUSCA 12) | chr2:11526762-11529490 REVERSE	
JCVI_21239	1.343	moderately similar to (316)AT1G17890| Symbols: GER2 | GER2; catalytic | chr1:6154471-6155589 REVERSEmoderately similar to (275	
EV133626	1.343	very weakly similar to (99.4)AT4G17310| Symbols: | transcription factor | chr4:9685441-9686860 REVERSE [21481] 1 577 740	-1.490
EE430607	1.343	weakly similar to (143)AT1G29290| Symbols: | similar to hypothetical protein [Vitis vinifera] (GB:CAN69942.1) | chr1:10245041-102453	
EV176716	1.343	moderately similar to (327)AT1G61380| Symbols: | S-locus protein kinase, putative | chr1:22649942-22653066 REVERSEvery weakly sin	
EV130085	1.343	weakly similar to (144)AT3G61160| Symbols: | shaggy-related protein kinase beta / ASK-beta (ASK2) | chr3:22647184-22649568 FORW	
JCVI_26255	1.342	highly similar to (647)AT4G02330| Symbols: ATPMEPCRB | ATPMEPCRB; pectinesterase | chr4:1032479-1034928 FORWARDmodera	
DY016382	1.342	no similarity	
EV155694	1.342	moderately similar to (461)AT5G05170| Symbols: CESA3, IXR1, ATCESA3, ATH-B, CEV1 | CESA3 (CELLULOSE SYNTHASE 3); cel	
JCVI_28476	1.342	weakly similar to (196)AT1G21100| Symbols: | O-methyltransferase, putative | chr1:7386980-7388307 REVERSEweakly similar to (108)	
JCVI_37343	1.342	moderately similar to (208)AT1G14800| Symbols: | similar to F-box family protein [Arabidopsis thaliana] (TAIR:AT2G35280.1); similar	
CX265808	1.342	weakly similar to (191)AT1G19860| Symbols: | zinc finger (CCCH-type) family protein | chr1:6891645-6894391 REVERSE [16816]	-1.677
EX100282	1.342	moderately similar to (290)AT3G61460| Symbols: BRH1 | BRH1 (BRASSINOSTEROID-RESPONSIVE RING-H2); protein binding / zin	
JCVI_27691	1.342	moderately similar to (218)AT5G12310| Symbols: | zinc finger (C3HC4-type RING finger) family protein | chr5:3980486-3982094 REVE	
JCVI_35738	1.341	very weakly similar to (89.4)AT5G11520| Symbols: YLS4, ASP3 | ASP3 (ASPARTATE AMINOTRANSFERASE 3) | chr5:3685258-3687	1.497

EE531012	1.341	weakly similar to (149)AT1G06720| Symbols: | similar to unknown protein [Arabidopsis thaliana] (TAIR:AT1G42440.1); similar to hypot	
JCVI_16076	1.341	weakly similar to (196)AT1G04930| Symbols: | hydroxyproline-rich glycoprotein family protein | chr1:1396545-1398987 REVERSE no or	
JCVI_25746	1.341	moderately similar to (296)AT1G53790| Symbols: | F-box family protein | chr1:20083226-20085062 REVERSE no original description	
EV100950	1.341	moderately similar to (213)AT4G23180| Symbols: RLK4, CRK10 | CRK10 (CYSTEINE-RICH RLK10); kinase | chr4:12138182-1214079	
JCVI_12181	1.341	moderately similar to (432)AT3G03220| Symbols: EXP13, ATEXP13, ATHEXP ALPHA 1.22, ATEXPA13 | ATEXPA13 (ARABIDOPSI	
CX281146	1.341	moderately similar to (201)AT1G50180| Symbols: | disease resistance protein (CC-NBS-LRR class), putative | chr1:18587903-18590804 I	
EX043301	1.341	moderately similar to (236)AT4G26850| Symbols: VTC2 | VTC2 (VITAMIN C DEFECTIVE 2) | chr4:13499268-13501151 REVERSE [2	-1.307
JCVI_34101	1.341	highly similar to (510)AT1G73650| Symbols: | oxidoreductase, acting on the CH-CH group of donors | chr1:27692081-27693267 REVER	-1.374
ES953011	1.341	weakly similar to (101)AT3G57200| Symbols: | similar to ABI8/ELD1/KOB1 (KOBITO) [Arabidopsis thaliana] (TAIR:AT3G08550.1); si	
JCVI_26010	1.341	moderately similar to (485)AT1G47670| Symbols: | amino acid transporter family protein | chr1:17539274-17541926 REVERSE no origin	
JCVI_37571	1.341	very weakly similar to (86.3)AT4G30930| Symbols: NFD1 | NFD1 (NUCLEAR FUSION DEFECTIVE 1); structural constituent of riboson	
JCVI_2548	1.340	moderately similar to (353)AT5G51110| Symbols: | similar to dehydratase family [Arabidopsis thaliana] (TAIR:AT1G29810.1); similar to	
BQ705076	1.340	very weakly similar to (84.7)AT3G23710| Symbols: | chloroplast inner membrane import protein Tic22, putative | chr3:8534068-8536155 I	
EV176491	1.340	weakly similar to (120)AT2G13770| Symbols: | similar to ribosomal protein-like [Oryza sativa (japonica cultivar-group)] (GB:BAD37986.	
EH414829	1.340	very weakly similar to (82.4)AT3G53750| Symbols: ACT3 | ACT3 (ACTIN 3); structural constituent of cytoskeleton | chr3:19926902-1992	
JCVI_547	1.340	very weakly similar to (95.5)AT1G78080| Symbols: RAP2.4 | RAP2.4 (related to AP2 4); DNA binding / transcription factor | chr1:293696	
JCVI_13923	1.340	weakly similar to (163)AT1G55520| Symbols: ATTBP2 | TBP2 (TATA binding protein 2); DNA binding / RNA polymerase II transcriptio	-1.505
JCVI_12413	1.340	moderately similar to (291)AT4G35450| Symbols: AFT, AKR2A, AKR2 | AKR2 (ANKYRIN REPEAT-CONTAINING PROTEIN 2); pro	
JCVI_36876	1.340	moderately similar to (289)AT1G51100| Symbols: | similar to unnamed protein product [Vitis vinifera] (GB:CAO39038.1) | chr1:1893801	
JCVI_18684	1.340	weakly similar to (167)AT5G11010| Symbols: | pre-mRNA cleavage complex-related | chr5:3484177-3486068 FORWARD no original de	-1.819
JCVI_858	1.339	moderately similar to (385)AT4G37790| Symbols: HAT22 | HAT22 (homeobox-leucine zipper protein 22); transcription factor | chr4:1776	
JCVI_42272	1.339	highly similar to (706)AT3G11540| Symbols: SPY | SPY (SPINDLY) | chr3:3632848-3637553 FORWARDhighly similar to (620)SPY_PI	
JCVI_20588	1.339	weakly similar to (115)AT1G47820| Symbols: | similar to unknown protein [Arabidopsis thaliana] (TAIR:AT1G47813.1); similar to unnar	
JCVI_8179	1.339	moderately similar to (453)AT4G04020| Symbols: FIB | FIB (FIBRILLIN); structural molecule | chr4:1932159-1933544 FORWARDhighly	
JCVI_1633	1.339	moderately similar to (431)AT2G01450| Symbols: ATMPK17 | ATMPK17 (Arabidopsis thaliana MAP kinase 17); MAP kinase | chr2:199	
ES938961	1.339	moderately similar to (212)AT4G27585| Symbols: | band 7 family protein | chr4:13766990-13769838 REVERSE [21390]	
EE551980	1.339	no similarity	
JCVI_2925	1.339	no original description	
EE401811	1.339	no similarity	
EX093229	1.339	weakly similar to (103)AT1G10410| Symbols: | similar to CW14 [Arabidopsis thaliana] (TAIR:AT1G59650.1); similar to expressed prote	
RC_EX016086	1.339	no similarity	
JCVI_12350	1.338	weakly similar to (106)AT1G52342| Symbols: | unknown protein | chr1:19496109-19496372 REVERSE no original description	
JCVI_6181	1.338	weakly similar to (159)AT5G02610| Symbols: | 60S ribosomal protein L35 (RPL35D) | chr5:587609-588545 FORWARDvery weakly simi	1.213
JCVI_39178	1.338	moderately similar to (330)AT1G10460| Symbols: GLP7 | GLP7 (GERMIN-LIKE PROTEIN 7); manganese ion binding / metal ion bindin	
EE448025	1.338	very weakly similar to (91.3)AT3G05580| Symbols: | serine/threonine protein phosphatase, putative | chr3:1618222-1619856 REVERSEve	
JCVI_11144	1.338	no original description	
EX021572	1.338	moderately similar to (260)AT2G30520| Symbols: RPT2 | RPT2 (ROOT PHOTOTROPISM 2) | chr2:13009997-13012230 REVERSE [21	-1.523
JCVI_29505	1.338	highly similar to (639)AT1G47270| Symbols: AtTLP6 | AtTLP6 (TUBBY LIKE PROTEIN 6); phosphoric diester hydrolase/ transcription	
JCVI_27800	1.338	moderately similar to (313)AT3G58660| Symbols: | 60S ribosomal protein-related | chr3:21712551-21713891 FORWARD no original des	
CD825635	1.338	weakly similar to (106)AT2G03810| Symbols: | 18S pre-ribosomal assembly protein gar2-related | chr2:1162700-1164283 FORWARD [1	
CX194779	1.338	weakly similar to (120)AT2G37270| Symbols: ATRPS5B | ATRPS5B (RIBOSOMAL PROTEIN 5B); structural constituent of ribosome |	
ES985189	1.337	moderately similar to (238)AT1G50950| Symbols: | thioredoxin-related | chr1:18884133-18887711 FORWARD [21389]	
EV151460	1.337	very weakly similar to (90.9)AT4G21810| Symbols: DER2.1 | DER2.1 (DERLIN-2.1) | chr4:11575357-11577015 REVERSE [21483]	
RC_EE556607	1.337	no similarity	
ES968028	1.337	no similarity	
JCVI_3369	1.337	moderately similar to (405)AT1G07310| Symbols: | C2 domain-containing protein | chr1:2247774-2248832 REVERSE no original descrip	
JCVI_17313	1.337	weakly similar to (125)AT3G07770| Symbols: | ATP binding | chr3:2479617-2483976 FORWARDvery weakly similar to (87.0)HSP83_IP	
JCVI_13182	1.337	moderately similar to (327)AT2G15690| Symbols: | pentatricopeptide (PPR) repeat-containing protein | chr2:6838937-6840676 REVERSI	
JCVI_16917	1.337	highly similar to (950)AT1G22410| Symbols: | 2-dehydro-3-deoxyphosphoheptonate aldolase, putative / 3-deoxy-D-arabino-heptulosonate	
EV201291	1.337	moderately similar to (221)AT1G20760| Symbols: | calcium-binding EF hand family protein | chr1:7209505-7214763 FORWARD [21490	
CD830947	1.336	no similarity	
JCVI_4400	1.336	moderately similar to (338)AT5G40200| Symbols: DEGP9 | DEGP9 (DEGP PROTEASE 9); serine-type peptidase/ trypsin | chr5:1608763	
JCVI_25221	1.336	no original description	
JCVI_27054	1.336	moderately similar to (312)AT3G45040| Symbols: | phosphatidate cytidylyltransferase family protein | chr3:16483786-16486687 REVERS	
JCVI_41233	1.336	very weakly similar to (97.4)AT5G57220| Symbols: CYP81F2 | CYP81F2 (cytochrome P450, family 81, subfamily F, polypeptide 2); oxyg	
JCVI_28541	1.336	highly similar to (554)AT5G05820| Symbols: | phosphate translocator-related | chr5:1752107-1753858 REVERSEvery weakly similar to (	
ES269654	1.336	weakly similar to (171)AT3G59480| Symbols: | pfkB-type carbohydrate kinase family protein | chr3:21994080-21995417 FORWARDwea	
JCVI_35458	1.336	highly similar to (690)AT3G03770| Symbols: | leucine-rich repeat transmembrane protein kinase, putative | chr3:945310-948443 REVERS	
JCVI_5047	1.336	moderately similar to (384)AT4G33400| Symbols: | dem protein-related / defective embryo and meristems protein-related | chr4:16078192	
JCVI_27026	1.336	no original description	
ES943768	1.336	moderately similar to (204)AT4G16090| Symbols: | similar to unknown protein [Arabidopsis thaliana] (TAIR:AT2G45930.1) | chr4:91030	
JCVI_337	1.336	highly similar to (607)AT4G13940| Symbols: EMB1395, MEE58, SAHH, SAHH1, HOG1 | HOG1 (HOMOLOGY-DEPENDENT GENE S	
JCVI_10458	1.336	moderately similar to (419)AT3G24350| Symbols: ATSYP32, SYP32 | SYP32 (syntaxin 32); SNAP receptor | chr3:8837740-8839409 FOF	-1.436
JCVI_33051	1.336	weakly similar to (158)AT2G24100| Symbols: | similar to unknown protein [Arabidopsis thaliana] (TAIR:AT4G30780.1); similar to unnar	
JCVI_42513	1.336	no original description	
DY030220	1.335	weakly similar to (157)AT2G05590| Symbols: | similar to unknown protein [Arabidopsis thaliana] (TAIR:AT4G39870.1); similar to unnar	
JCVI_16533	1.335	moderately similar to (338)AT1G80630| Symbols: | leucine-rich repeat family protein | chr1:30313771-30315507 REVERSE no original d	
JCVI_23204	1.335	moderately similar to (309)AT5G56030| Symbols: ERD8, HSP81-2 | HSP81-2 (EARLY-RESPONSIVE TO DEHYDRATION 8); ATP bir	
JCVI_36163	1.335	no original description	
JCVI_25308	1.335	highly similar to (586)AT1G12330| Symbols: | similar to unknown protein [Arabidopsis thaliana] (TAIR:AT5G12900.1); similar to unnam	
DN964000	1.335	no similarity	
L47861	1.335	no similarity	
EE441355	1.335	moderately similar to (355)AT3G08680| Symbols: | leucine-rich repeat transmembrane protein kinase, putative | chr3:2638597-2640596 F	
JCVI_12988	1.335	no original description	
EE559157	1.335	no similarity	
EE459714	1.335	moderately similar to (365)AT5G48120| Symbols: | binding | chr5:19525436-19532000 REVERSE [20152]	
ES931148	1.334	weakly similar to (124)AT1G70040| Symbols: | similar to unknown protein [Arabidopsis thaliana] (TAIR:AT1G70120.1); contains InterPr	
EE532196	1.334	weakly similar to (112)AT4G32090| Symbols: | galactosyltransferase | chr4:15509996-15510826 REVERSE [20175]	
JCVI_40100	1.334	weakly similar to (177)AT5G49760| Symbols: | leucine-rich repeat family protein / protein kinase family protein | chr5:20233905-2023827	-2.968
JCVI_35186	1.334	very weakly similar to (91.3)AT4G10810| Symbols: | similar to unknown protein [Arabidopsis thaliana] (TAIR:AT4G24026.1) | chr4:6645	
AM396057	1.334	no similarity	

JCVI_39644	1.334	moderately similar to (325)AT5G02590| Symbols: | chloroplast lumen common family protein | chr5:583115-584095 FORWARD no origi	
EV068289	1.333	no similarity	
JCVI_41726	1.333	no original description	
CD823837	1.333	moderately similar to (229)AT5G45100| Symbols: | protein binding / zinc ion binding | chr5:18236165-18236968 FORWARD [13979]	
JCVI_2310	1.333	moderately similar to (227)AT5G07720| Symbols: | galactosyl transferase GMA12/MNN10 family protein | chr5:2455758-2457131 FORV	-1.491
JCVI_39646	1.332	moderately similar to (485)AT1G18140| Symbols: LAC1 | LAC1 (Laccase 1); copper ion binding / oxidoreductase | chr1:6238979-624138	
JCVI_22614	1.332	very weakly similar to (85.1)AT2G47490| Symbols: | mitochondrial substrate carrier family protein | chr2:19494619-19496381 FORWARI	
JCVI_29473	1.332	weakly similar to (106)AT1G66980| Symbols: | protein kinase family protein / glycerophosphoryl diester phosphodiesterase family protein	
DY018904	1.332	weakly similar to (148)AT3G45890| Symbols: | similar to unknown protein [Arabidopsis thaliana] (TAIR:AT1G13770.1); similar to unnar	
JCVI_35810	1.332	moderately similar to (221)AT1G76980| Symbols: | similar to EMB2170 (EMBRYO DEFECTIVE 2170) [Arabidopsis thaliana] (TAIR:A	
JCVI_571	1.332	moderately similar to (362)AT2G25110| Symbols: SDF2 | SDF2 (STROMAL CELL-DERIVED FACTOR 2-LIKE PROTEIN PRECURSC	
JCVI_7081	1.332	moderately similar to (207)AT3G03070| Symbols: | NADH-ubiquinone oxidoreductase-related | chr3:696600-698076 REVERSE no origir	
JCVI_13581	1.332	highly similar to (867)AT2G35720| Symbols: | DNAJ heat shock N-terminal domain-containing protein | chr2:15023962-15026945 FORW	
ES941763	1.331	moderately similar to (239)AT1G14840| Symbols: ATMAP70-4 | ATMAP70-4 (microtubule-associated proteins 70-4); microtubule bindir	
JCVI_39140	1.331	weakly similar to (163)AT5G59960| Symbols: | similar to unnamed protein product [Vitis vinifera] (GB:CAO21698.1) | chr5:24159578-24	
DY028897	1.331	moderately similar to (308)AT3G13180| Symbols: | NOL1/NOP2/sun family protein / antitermination NusB domain-containing protein | c	
JCVI_15744	1.331	highly similar to (518)AT5G21160| Symbols: | La domain-containing protein / proline-rich family protein | chr5:7199194-7203882 REVEI	
JCVI_2363	1.331	weakly similar to (113)AT4G23910| Symbols: | similar to unknown protein [Arabidopsis thaliana] (TAIR:AT4G10970.4); similar to unkno	
ES963497	1.331	no similarity	
JCVI_7256	1.331	highly similar to (687)AT2G25730| Symbols: | binding / heme binding | chr2:10963829-10979807 REVERSE no original description	-1.313
JCVI_243	1.331	moderately similar to (426)AT1G19540| Symbols: | isoflavone reductase, putative | chr1:6765704-6767238 FORWARDmoderately simila	
DY019261	1.330	moderately similar to (221)AT3G19180| Symbols: ARC6H | ARC6H | chr3:6632816-6636553 FORWARD [18966]	
JCVI_20451	1.330	no original description	1.622
ES901899	1.330	no similarity	
JCVI_4537	1.330	no original description	
JCVI_17440	1.330	moderately similar to (320)AT1G09660| Symbols: | KH domain-containing quaking protein, putative | chr1:3128207-3130793 REVERSE	
JCVI_4997	1.330	moderately similar to (407)AT5G25630| Symbols: | pentatricopeptide (PPR) repeat-containing protein | chr5:8947429-8949427 FORWAR	
EX037246	1.330	weakly similar to (129)AT5G45100| Symbols: | protein binding / zinc ion binding | chr5:18236165-18236968 FORWARD [21811]	
JCVI_31426	1.329	highly similar to (572)AT2G40360| Symbols: | transducin family protein / WD-40 repeat family protein | chr2:16860201-16863713 REVE	
AM059008	1.329	weakly similar to (123)AT5G08560| Symbols: | transducin family protein / WD-40 repeat family protein | chr5:2771105-2773828 REVER	
JCVI_4801	1.329	highly similar to (527)AT3G61130| Symbols: GAUT1, LGT1 | GAUT1/LGT1 (Galacturonosyltransferase 1); polygalacturonate 4-alpha-ga	
JCVI_16511	1.329	very weakly similar to (87.0)AT5G05180| Symbols: | similar to unknown protein [Arabidopsis thaliana] (TAIR:AT3G10880.1); similar to u	
CD834887	1.329	no similarity	
ES939726	1.329	weakly similar to (161)AT2G30110| Symbols: MOS5, ATUBA1 | ATUBA1 (ARABIDOPSIS THALIANA UBIQUITIN-ACTIVATING E	
DY021507	1.329	weakly similar to (200)AT5G27430| Symbols: | signal peptidase subunit family protein | chr5:9687473-9689188 FORWARDvery weakly s	
JCVI_40070	1.329	no original description	
CX194329	1.329	moderately similar to (218)AT3G09030| Symbols: | potassium channel tetramerisation domain-containing protein | chr3:2754846-2756228	
EE546822	1.328	very weakly similar to (100)AT1G65840| Symbols: ATPAO4 | ATPAO4 (POLYAMINE OXIDASE 4); amine oxidase | chr1:24493836-24	
EV171221	1.328	no similarity	-1.441
JCVI_32357	1.328	moderately similar to (224)AT4G29440| Symbols: | similar to unknown protein [Arabidopsis thaliana] (TAIR:AT2G19710.1); similar to u	
JCVI_12523	1.328	moderately similar to (226)AT2G16630| Symbols: | proline-rich family protein | chr2:7216368-7217954 FORWARD no original descriptio	
EE484489	1.328	weakly similar to (160)AT3G56760| Symbols: | calcium-dependent protein kinase, putative / CDPK, putative | chr3:21031640-21034735 F	
EV035594	1.328	moderately similar to (229)AT1G75760| Symbols: | ER lumen protein retaining receptor family protein | chr1:28450601-28452082 REVE	
JCVI_32415	1.328	moderately similar to (479)AT3G33530| Symbols: | transducin family protein / WD-40 repeat family protein | chr3:14096340-14104435 F	
JCVI_28644	1.328	moderately similar to (262)AT4G25240| Symbols: SKS1 | SKS1 (SKU5 SIMILAR 1); copper ion binding | chr4:12930549-12933573 FOR	
JCVI_20330	1.328	no original description	
JCVI_12660	1.328	moderately similar to (445)AT1G78780| Symbols: | pathogenesis-related family protein | chr1:29626340-29627324 REVERSEmoderately	
EV131630	1.327	weakly similar to (140)AT5G23060| Symbols: CAS | similar to unknown protein [Arabidopsis thaliana] (TAIR:AT3G59780.1); similar to e	
JCVI_33009	1.327	moderately similar to (256)AT1G67290| Symbols: | glyoxal oxidase-related | chr1:25194831-25196823 REVERSE no original description	
EX091904	1.327	no similarity	
EE481304	1.327	no similarity	
EV219225	1.327	moderately similar to (266)AT5G49760| Symbols: | leucine-rich repeat family protein / protein kinase family protein | chr5:20233905-202	
JCVI_19305	1.327	moderately similar to (477)AT5G37310| Symbols: | transporter | chr5:14790066-14793323 REVERSE no original description	
JCVI_27958	1.327	moderately similar to (311)AT1G07890| Symbols: MEE6, CS1, APX1 | APX1 (ASCORBATE PEROXIDASE 1) | chr1:2438002-2439432	
CX192315	1.327	moderately similar to (282)AT1G59540| Symbols: ZCF125 | ZCF125 | chr1:21878243-21883047 FORWARD [16807]	
JCVI_17201	1.327	moderately similar to (310)AT3G11410| Symbols: ATPP2CA, AHG3 | AHG3/ATPP2CA (ARABIDOPSIS THALIANA PROTEIN PHOS	
EV029503	1.327	no similarity	
EV136490	1.327	no similarity	
JCVI_9843	1.327	moderately similar to (220)AT3G50910| Symbols: | similar to unknown protein [Arabidopsis thaliana] (TAIR:AT5G66480.1); similar to h	-1.481
EG020667	1.327	no similarity	
ES903412	1.326	highly similar to (504)AT1G25570| Symbols: | leucine-rich repeat protein-related | chr1:8992170-8995417 REVERSE [21432]	
EV111764	1.326	no similarity	1.719
JCVI_37580	1.326	moderately similar to (426)AT1G53500| Symbols: RHM2, MUM4 | MUM4 (MUCILAGE-MODIFIED 4); catalytic | chr1:19970825-1997	
JCVI_14750	1.326	moderately similar to (315)AT1G13600| Symbols: ATBZIP58 | ATBZIP58 (ARABIDOPSIS THALIANA BASIC LEUCINE-ZIPPER 58)	
EX042542	1.326	moderately similar to (256)AT1G02040| Symbols: | zinc finger (C2H2 type) family protein | chr1:358104-359078 REVERSE [21811]	
JCVI_7677	1.326	highly similar to (562)AT3G08730| Symbols: ATPK6, ATPK1, ATS6K1, PK6, PK1, S6K1 | ATPK1 (P70 RIBOSOMAL S6 KINASE); kir	
DY005737	1.326	weakly similar to (123)AT2G31970| Symbols: ATRAD50, RAD50 | RAD50; ATP binding / nuclease/ zinc ion binding | chr2:13607734-13	
EV087550	1.326	very weakly similar to (89.7)AT1G44110| Symbols: CYCA1;1 | CYCA1;1 (CYCLIN A1;1); cyclin-dependent protein kinase regulator | chr	
EV150940	1.325	no similarity	
EV197701	1.325	moderately similar to (430)AT5G46390| Symbols: | peptidase S41 family protein | chr5:18833839-18836375 FORWARD [21490] 39 757	
JCVI_5342	1.325	moderately similar to (395)AT4G21810| Symbols: DER2.1 | DER2.1 (DERLIN-2.1) | chr4:11575357-11577015 REVERSEmoderately sim	
DY000590	1.325	weakly similar to (121)AT4G39080| Symbols: VHA-A3 | VHA-A3 (VACUOLAR PROTON ATPASE A3); ATPase | chr4:18209507-1821	
JCVI_2199	1.325	moderately similar to (246)AT5G25280| Symbols: | serine-rich protein-related | chr5:8773885-8774547 FORWARD no original descriptio	
JCVI_35005	1.325	weakly similar to (108)AT4G23630| Symbols: BTI1 | BTI1 (VIRB2-INTERACTING PROTEIN 1) | chr4:12318080-12319584 FORWARI	
JCVI_31177	1.325	moderately similar to (255)AT5G07120| Symbols: | phox (PX) domain-containing protein | chr5:2207066-2209356 REVERSE no original	
EV131791	1.325	very weakly similar to (82.0)AT1G25520| Symbols: | similar to unknown protein [Arabidopsis thaliana] (TAIR:AT1G68650.1); similar to u	
EE566120	1.325	no similarity	
JCVI_25568	1.325	no original description	
JCVI_12459	1.325	moderately similar to (374)AT1G75680| Symbols: ATGH9B7 | ATGH9B7 (ARABIDOPSIS THALIANA GLYCOSYL HYDROLASE 9B	
ES912837	1.325	no similarity	
JCVI_23279	1.325	moderately similar to (234)AT3G28710| Symbols: | H+-transporting two-sector ATPase, putative | chr3:10774381-10776831 REVERSEm	

RC_JCVI_41910	1.324	no original description	-2.628
EV192032	1.324	moderately similar to (338)AT1G31350| Symbols: | F-box family protein | chr1:11221500-11222687 REVERSE [21489] 31 744 744	
EV069828	1.324	moderately similar to (258)AT1G69870| Symbols: | proton-dependent oligopeptide transport (POT) family protein | chr1:26319871-26323	
JCVI_10539	1.324	weakly similar to (106)AT5G01980| Symbols: | zinc finger (C3HC4-type RING finger) family protein | chr5:375539-377020 FORWARD	
EX024004	1.324	no similarity	
JCVI_27483	1.324	no original description	-1.868
EX034457	1.324	moderately similar to (371)AT1G67630| Symbols: POLA2 | POLA2; alpha DNA polymerase | chr1:25348236-25351950 REVERSE [2181	
JCVI_2791	1.324	moderately similar to (412)AT5G49360| Symbols: ATBXL1, BXL1 | BXL1 (BETA-XYLOSIDASE 1); hydrolase, hydrolyzing O-glycosyl	
JCVI_21820	1.324	weakly similar to (138)AT3G48890| Symbols: ATMAPR3, MSBP2, ATMP2 | ATMP2 (MEMBRANE STEROID BINDING PROTEIN 2)	
JCVI_41088	1.324	weakly similar to (159)AT1G74680| Symbols: | exostosin family protein | chr1:28063189-28064645 FORWARD no original description	
DY000489	1.323	moderately similar to (223)AT3G20640| Symbols: | ethylene-responsive protein -related | chr3:7210660-7213205 REVERSE [18967]	
EV025526	1.323	very weakly similar to (82.8)AT5G16140| Symbols: | peptidyl-tRNA hydrolase family protein | chr5:5270311-5271520 REVERSE [21441]	-1.296
EV050348	1.323	weakly similar to (107)AT4G30630| Symbols: | similar to unknown protein [Arabidopsis thaliana] (TAIR:AT5G57910.1); similar to unnam	
EX020717	1.323	moderately similar to (232)AT1G46480| Symbols: | homeobox-leucine zipper transcription factor family protein | chr1:17239343-1724039	
JCVI_21258	1.323	moderately similar to (351)AT4G31340| Symbols: | myosin heavy chain-related | chr4:15205668-15208901 FORWARD no original descri	
JCVI_40404	1.322	moderately similar to (427)AT5G20610| Symbols: | similar to unknown protein [Arabidopsis thaliana] (TAIR:AT5G26160.1); similar to h	
JCVI_10708	1.322	moderately similar to (406)AT2G04650| Symbols: | ADP-glucose pyrophosphorylase family protein | chr2:1621983-1624483 REVERSE n	
EV072964	1.322	moderately similar to (223)AT5G07670| Symbols: | F-box family protein | chr5:2430422-2432066 FORWARD [21443]	
JCVI_24537	1.322	weakly similar to (158)AT3G51090| Symbols: | similar to unknown protein [Arabidopsis thaliana] (TAIR:AT2G16460.1); similar to unnam	
CV523235	1.322	no similarity	-1.354
DW998059	1.322	very weakly similar to (95.5)AT3G30460| Symbols: | zinc finger (C3HC4-type RING finger) family protein | chr3:12106912-12107355 FO	
JCVI_39710	1.322	moderately similar to (360)AT3G56970| Symbols: BHLH038, ORG2 | BHLH038/ORG2 (OBP3-RESPONSIVE GENE 2); DNA binding /	
EV030709	1.321	no similarity	
JCVI_16623	1.321	moderately similar to (274)AT4G10320| Symbols: | isoleucyl-tRNA synthetase, putative / isoleucine--tRNA ligase, putative | chr4:639752	
JCVI_19609	1.321	moderately similar to (270)AT4G02130| Symbols: GATL6, LGT10 | GATL6/LGT10; transferase, transferring glycosyl groups / transferase	
ES899087	1.321	no similarity	
EV141560	1.321	moderately similar to (224)AT2G43410| Symbols: FPA | FPA | chr2:18033474-18038066 REVERSE [21482]	
EH419245	1.321	weakly similar to (105)AT5G17523| Symbols: | Similar to Maltose excess protein 1 | chr5:5776246-5777233 REVERSE [20767]	2.239
JCVI_25859	1.321	weakly similar to (150)AT5G60580| Symbols: | zinc finger (C3HC4-type RING finger) family protein | chr5:24371524-24373932 FORWA	
JCVI_41416	1.320	highly similar to (733)AT2G35110| Symbols: GRL, NAP1, NAPP | GRL/NAP1/NAPP (NCK-ASSOCIATED PROTEIN) | chr2:14803049	
JCVI_22986	1.320	moderately similar to (339)AT3G02740| Symbols: | aspartyl protease family protein | chr3:590568-593096 FORWARD no original descrip	
JCVI_23138	1.320	no original description	
JCVI_2421	1.320	weakly similar to (175)AT3G17020| Symbols: | universal stress protein (USP) family protein | chr3:5802734-5804069 REVERSE no origi	
JCVI_8661	1.320	weakly similar to (149)AT2G31670| Symbols: | similar to unknown protein [Arabidopsis thaliana] (TAIR:AT1G51360.1); similar to unkno	
JCVI_41530	1.320	moderately similar to (233)AT4G33220| Symbols: | pectinesterase family protein | chr4:16024449-16026134 FORWARDweakly similar to	
JCVI_4704	1.320	moderately similar to (409)AT3G29810| Symbols: COBL2 | COBL2 (COBRA-LIKE PROTEIN 2 PRECURSOR) | chr3:11730691-117326	
EV012023	1.320	no similarity	
JCVI_32032	1.320	highly similar to (607)AT4G34280| Symbols: | transducin family protein / WD-40 repeat family protein | chr4:16407088-16410648 FORW	
JCVI_37011	1.320	no original description	
BQ704855	1.320	no similarity	
JCVI_37978	1.319	moderately similar to (221)AT3G16760| Symbols: | tetratricopeptide repeat (TPR)-containing protein | chr3:5703219-5705086 FORWARD	
EV205444	1.319	very weakly similar to (85.1)AT3G51270| Symbols: | ATP binding / protein serine/threonine kinase | chr3:19044830-19047751 FORWARD	
JCVI_40253	1.319	moderately similar to (319)AT1G26150| Symbols: | protein kinase | chr1:9039777-9042860 REVERSEweakly similar to (168)PSKR_DA	
JCVI_17865	1.319	highly similar to (633)AT1G06620| Symbols: | 2-oxoglutarate-dependent dioxygenase, putative | chr1:2025617-2027093 FORWARDmod	
JCVI_16292	1.319	no original description	
JCVI_33835	1.319	moderately similar to (233)AT4G04570| Symbols: | protein kinase family protein | chr4:2290043-2292253 FORWARD no original descrip	
EX040295	1.319	moderately similar to (237)AT2G27820| Symbols: ADT3, PD1 | PD1 (PREPHENATE DEHYDRATASE 1); arogenate dehydratase/ preph	
EV023656	1.319	moderately similar to (229)AT5G52890| Symbols: | AT hook motif-containing protein | chr5:21462266-21463423 REVERSE [21441]	
ES940888	1.319	moderately similar to (408)AT2G33760| Symbols: | pentatricopeptide (PPR) repeat-containing protein | chr2:14282876-14284627 FORWA	
JCVI_25843	1.319	highly similar to (613)AT5G63140| Symbols: ATPAP29, PAP29 | ATPAP29/PAP29 (purple acid phosphatase 29); acid phosphatase/ prote	
JCVI_36365	1.318	moderately similar to (344)AT3G03440| Symbols: | armadillo/beta-catenin repeat family protein | chr3:815716-818575 FORWARD no ori	
JCVI_27572	1.318	moderately similar to (320)AT4G28980| Symbols: CDKF;1, CAK1AT | CAK1AT/CDKF;1 (CDK-ACTIVATING KINASE 1AT); cyclin-c	
JCVI_6081	1.318	no original description	
JCVI_15265	1.318	moderately similar to (342)AT2G42250| Symbols: CYP712A1 | CYP712A1 (cytochrome P450, family 712, subfamily A, polypeptide 1); c	
JCVI_5469	1.318	weakly similar to (121)AT1G07985| Symbols: | Expressed protein | chr1:2475505-2475939 FORWARD no original description	
JCVI_33106	1.318	moderately similar to (268)AT3G08710| Symbols: ATH9 | ATH9 (thioredoxin H-type 9) | chr3:2645596-2646310 FORWARDweakly simi	
ES967841	1.318	no similarity	
JCVI_3074	1.318	highly similar to (523)AT5G13440| Symbols: | ubiquinol-cytochrome C reductase iron-sulfur subunit, mitochondrial, putative / Rieske iron	
JCVI_30806	1.318	moderately similar to (313)AT2G22790| Symbols: | similar to unknown protein [Arabidopsis thaliana] (TAIR:AT5G67020.1); similar to u	
EE546439	1.318	no similarity	
JCVI_40667	1.318	moderately similar to (353)AT5G12340| Symbols: | similar to unknown protein [Arabidopsis thaliana] (TAIR:AT1G28190.1); similar to u	
EV205016	1.317	weakly similar to (133)AT4G00780| Symbols: | meprin and TRAF homology domain-containing protein / MATH domain-containing prote	
JCVI_14803	1.317	moderately similar to (361)AT5G67230| Symbols: | glycosyl transferase family 43 protein | chr5:26839732-26841407 FORWARD no orig	
JCVI_26802	1.317	moderately similar to (320)AT1G08220| Symbols: | similar to unnamed protein product [Vitis vinifera] (GB:CAO16719.1); contains Inter	
EV045346	1.317	weakly similar to (199)AT5G27220| Symbols: | protein transport protein-related | chr5:9578760-9582755 FORWARD [21442]	
JCVI_3151	1.317	moderately similar to (402)AT3G08030| Symbols: | similar to unknown protein [Arabidopsis thaliana] (TAIR:AT2G41800.1); similar to u	
JCVI_4853	1.316	no original description	
AI352875	1.316	weakly similar to (182)AT2G20190| Symbols: ATCLASP, CLASP | ATCLASP/CLASP; binding | chr2:8718943-8725894 REVERSE [128	
JCVI_38586	1.316	highly similar to (918)AT4G19120| Symbols: ERD3 | ERD3 (EARLY-RESPONSIVE TO DEHYDRATION 3) | chr4:10460676-10463045	
JCVI_12209	1.316	moderately similar to (448)AT2G04850| Symbols: | auxin-responsive protein-related | chr2:1704295-1705605 FORWARD no original des	
JCVI_6328	1.316	weakly similar to (192)AT5G14370| Symbols: | similar to CIL [Arabidopsis thaliana] (TAIR:AT4G25990.1); similar to unnamed protein p	
JCVI_16264	1.316	moderately similar to (397)AT3G15160| Symbols: | binding | chr3:5104589-5108286 FORWARD no original description	
JCVI_538	1.316	moderately similar to (461)AT4G30270| Symbols: MERI-5, SEN4, MERI5B | MERI5B (MERISTEM-5); hydrolase, acting on glycosyl bo	
JCVI_39587	1.316	very weakly similar to (87.4)AT1G32660| Symbols: | F-box family protein | chr1:11811020-11812360 FORWARD no original description	
JCVI_3944	1.315	moderately similar to (394)AT1G17190| Symbols: ATGSTU26 | ATGSTU26 (Arabidopsis thaliana Glutathione S-transferase (class tau) 26	
JCVI_9667	1.315	moderately similar to (230)AT2G45170| Symbols: ATG8E, AtATG8e | AtATG8e (AUTOPHAGY 8E); microtubule binding | chr2:186316	
ES927039	1.315	no similarity	
EE401111	1.315	very weakly similar to (90.5)AT1G51270| Symbols: | structural molecule | chr1:19011677-19014079 FORWARD [20197]	
JCVI_28767	1.315	moderately similar to (315)AT5G56140| Symbols: | KH domain-containing protein | chr5:22742688-22745158 FORWARD no original des	
EV146737	1.315	weakly similar to (186)AT5G67180| Symbols: | AP2 domain-containing transcription factor, putative | chr5:26819345-26821160 REVERS	
JCVI_15614	1.315	highly similar to (961)AT1G13210| Symbols: ACA.L | ACA.L (AUTOINHIBITED CA2+/ATPASE II); ATPase, coupled to transmembran	

JCVI_5639	1.315	moderately similar to (407)AT5G20280| Symbols: ATSPS1F | ATSPS1F (SUCROSE PHOSPHATE SYNTHASE 1F); sucrose-phosphate s	
JCVI_25031	1.315	no original description	
JCVI_2304	1.315	moderately similar to (273)AT4G14730| Symbols: | transmembrane protein-related | chr4:8448602-8449996 FORWARD no original descr	
JCVI_15430	1.315	highly similar to (551)AT1G12760| Symbols: | protein binding / zinc ion binding | chr1:4348939-4350510 FORWARD no original descrip	
JCVI_3399	1.315	moderately similar to (314)AT1G73030| Symbols: VPS46.2 | VPS46.2 | chr1:27477599-27478509 FORWARD no original description	
DW999118	1.315	weakly similar to (147)AT1G09840| Symbols: | shaggy-related protein kinase kappa / ASK-kappa (ASK10) | chr1:3196116-3199526 REV	
JCVI_575	1.315	moderately similar to (329)AT3G61560| Symbols: | reticulon family protein (RTNLB6) | chr3:22788893-22790146 FORWARD no origina	
JCVI_8755	1.315	moderately similar to (263)AT5G18680| Symbols: AtTLP11 | AtTLP11 (TUBBY LIKE PROTEIN 11); phosphoric diester hydrolase/ trans	
EV127026	1.314	weakly similar to (102)AT4G31410| Symbols: | similar to unknown protein [Arabidopsis thaliana] (TAIR:AT3G24740.2); similar to unkno	
JCVI_25005	1.314	moderately similar to (277)AT1G15880| Symbols: ATGOS11, GOS11 | GOS11 (GOLGI SNARE 11); SNARE binding | chr1:5458712-546	
JCVI_21613	1.314	moderately similar to (215)AT4G26690| Symbols: MRH5, SHV3 | MRH5/SHV3 (morphogenesis of root hair 5); glycerophosphodiester ph	1.986
EV216944	1.314	moderately similar to (250)AT2G39435| Symbols: | similar to unknown protein [Arabidopsis thaliana] (TAIR:AT3G53540.1); similar to u	
JCVI_32994	1.314	moderately similar to (324)AT5G59530| Symbols: | 2-oxoglutarate-dependent dioxygenase, putative | chr5:24011660-24012941 REVERS	
JCVI_36323	1.314	highly similar to (502)AT2G46915| Symbols: | similar to sodium:dicarboxylate symporter [Arabidopsis thaliana] (TAIR:AT3G19340.1); s	
EX035806	1.314	no similarity	
EV004185	1.314	moderately similar to (357)AT4G27585| Symbols: | band 7 family protein | chr4:13766990-13769838 REVERSE [21427]	
EV072159	1.314	very weakly similar to (90.9)AT2G22440| Symbols: | similar to reverse transcriptase, putative / RNA-dependent DNA polymerase, putative	
CV545062	1.314	very weakly similar to (93.6)AT5G32440| Symbols: | similar to unknown [Populus trichocarpa] (GB:ABK93674.1); contains InterPro dom	1.616
JCVI_39306	1.314	no original description	
JCVI_24211	1.313	moderately similar to (296)AT1G76400| Symbols: | ribophorin I family protein | chr1:28663607-28666566 REVERSE no original descript	
EV017412	1.313	moderately similar to (345)AT2G39940| Symbols: COI1 | COI1 (CORONATINE INSENSITIVE 1); ubiquitin-protein ligase | chr2:166799	
JCVI_37217	1.313	very weakly similar to (83.2)AT4G34270| Symbols: | TIP41-like family protein | chr4:16404142-16406157 REVERSE no original descript	
JCVI_3839	1.313	moderately similar to (451)AT4G09670| Symbols: | oxidoreductase family protein | chr4:6107379-6109046 REVERSE no original descrip	
JCVI_4781	1.313	moderately similar to (309)AT4G19170| Symbols: NCED4 | NCED4 (NINE-CIS-EPOXYCAROTENOID DIOXYGENASE 4) | chr4:1048	
JCVI_18984	1.313	moderately similar to (498)AT2G28470| Symbols: BGAL8 | BGAL8 (BETA-GALACTOSIDASE 8); beta-galactosidase | chr2:12176124-1	
EE522760	1.313	weakly similar to (161)AT5G64813| Symbols: LIP1 | LIP1 (LIGHT INSENSITIVE PERIOD1); GTPase | chr5:25928062-25929851 FORW	
EV146181	1.313	moderately similar to (223)AT1G14460| Symbols: | DNA polymerase-related | chr1:4948957-4952745 REVERSE [21482]	
EE566693	1.313	weakly similar to (135)AT1G18470| Symbols: | zinc finger (C3HC4-type RING finger) family protein | chr1:6356965-6360050 REVERSE	
EV181348	1.313	moderately similar to (345)AT1G09970| Symbols: | leucine-rich repeat transmembrane protein kinase, putative | chr1:3252410-3255430 F	
JCVI_32614	1.312	moderately similar to (367)AT1G66860| Symbols: | hydrolase | chr1:24945903-24948015 FORWARD no original description	
EV205516	1.312	weakly similar to (158)AT1G08620| Symbols: | transcription factor jumonji (jmj) family protein / zinc finger (C5HC2 type) family protein	
JCVI_14089	1.312	moderately similar to (290)AT5G61865| Symbols: | similar to hypothetical protein [Vitis vinifera] (GB:CAN73072.1) | chr5:24864621-248	
EV049373	1.312	moderately similar to (233)AT3G03750| Symbols: | SET domain-containing protein | chr3:939983-941518 FORWARD [21442]	
EX136031	1.312	moderately similar to (479)AT5G16910| Symbols: CSLD2, ATCSLD2 | ATCSLD2 (Cellulose synthase-like D2); cellulose synthase/ transf	1.859
JCVI_15606	1.312	moderately similar to (311)AT4G16490| Symbols: | armadillo/beta-catenin repeat family protein | chr4:9294747-9295553 REVERSE no o	
JCVI_20152	1.311	highly similar to (560)AT5G49660| Symbols: | leucine-rich repeat transmembrane protein kinase, putative | chr5:20178627-20181760 REV	
H07732	1.311	no similarity	
EX098474	1.311	no similarity	
RC_EE561759	1.311	no similarity	
JCVI_23879	1.311	highly similar to (525)AT1G30330| Symbols: ARF6 | ARF6 (AUXIN RESPONSE FACTOR 6) | chr1:10686107-10690018 REVERSE no	
EE438124	1.311	moderately similar to (359)AT5G09760| Symbols: | pectinesterase family protein | chr5:3032447-3034365 FORWARD [20181]	
JCVI_12639	1.311	highly similar to (514)AT1G56050| Symbols: | GTP-binding protein-related | chr1:20967458-20969846 FORWARD no original descriptio	
EX120505	1.311	weakly similar to (125)AT3G15115| Symbols: | similar to unknown protein [Arabidopsis thaliana] (TAIR:AT1G53180.1) | chr3:5086226-5	
RC_EE568004	1.310	no similarity	
JCVI_13985	1.310	weakly similar to (192)AT2G24150| Symbols: HHP3 | HHP3 (heptahelical protein 3); receptor | chr2:10272712-10274425 REVERSE no c	
JCVI_18551	1.310	no original description	1.534
JCVI_9973	1.310	moderately similar to (290)AT2G25450| Symbols: | 2-oxoglutarate-dependent dioxygenase, putative | chr2:10837364-10838641 REVERS	
JCVI_324	1.310	moderately similar to (447)AT3G01280| Symbols: | porin, putative | chr3:85761-87619 FORWARDmoderately similar to (370)VDAC1_S	
JCVI_27948	1.310	moderately similar to (474)AT1G17270| Symbols: | similar to unknown protein [Arabidopsis thaliana] (TAIR:AT5G50420.1); similar to C	
EV026415	1.310	moderately similar to (407)AT2G42580| Symbols: TTL3 | TTL3 (TETRATRICOPETIDE-REPEAT THIOREDOXIN-LIKE 3); protein bi	-1.702
JCVI_28817	1.310	very weakly similar to (89.4)AT2G43745| Symbols: | similar to unknown protein [Arabidopsis thaliana] (TAIR:AT2G43740.2); similar to u	
CX272532	1.310	no similarity	
JCVI_13309	1.309	highly similar to (836)AT5G27380| Symbols: GSH2, GSHB | GSH2/GSHB (GLUTATHIONE SYNTHETASE 2); glutathione synthase | cl	
JCVI_16668	1.309	weakly similar to (111)AT3G15357| Symbols: | unknown protein | chr3:5187171-5187602 FORWARD no original description	
EE440776	1.309	weakly similar to (108)AT4G36970| Symbols: | remorin family protein | chr4:17429821-17431454 REVERSE [20167]	
EX089262	1.309	no similarity	
JCVI_16534	1.309	moderately similar to (423)AT5G60910| Symbols: FUL, AGL8 | AGL8 (AGAMOUS-LIKE 8) | chr5:24519962-24522160 REVERSEmode	
JCVI_14637	1.309	moderately similar to (441)AT1G59520| Symbols: CW7 | CW7 | chr1:21865254-21869681 FORWARD no original description	1.493
JCVI_23936	1.308	moderately similar to (383)AT5G04920| Symbols: | vacuolar protein sorting 36 family protein / VPS36 family protein | chr5:1439173-144	
EX125506	1.308	moderately similar to (237)AT5G28050| Symbols: | cytidine/deoxycytidylate deaminase family protein | chr5:10044213-10045488 REVEI	
CX192067	1.308	weakly similar to (120)AT5G23750| Symbols: | remorin family protein | chr5:8010007-8011456 REVERSEvery weakly similar to (80.1)R	
DY026716	1.308	very weakly similar to (88.2)AT3G17970| Symbols: ATTOC64-III | ATTOC64-III (ARABIDOPSIS THALIANA TRANSLOCON AT THE	
EV225495	1.308	moderately similar to (387)AT4G32910| Symbols: | similar to nucleoporin [Lotus japonicus] (GB:BAF45348.1); similar to putative nucleo	
JCVI_31096	1.308	highly similar to (832)AT5G04550| Symbols: | similar to unknown protein [Arabidopsis thaliana] (TAIR:AT3G23160.1); similar to unnam	
JCVI_16960	1.308	moderately similar to (295)AT4G30240| Symbols: | protein binding | chr4:14808365-14809751 REVERSE no original description	
EX130509	1.308	moderately similar to (272)AT5G65450| Symbols: | ubiquitin carboxyl-terminal hydrolase family protein / zinc finger (MYND type) famil	
EV142213	1.308	no similarity	
EV040791	1.308	weakly similar to (192)AT1G77330| Symbols: | 1-aminocyclopropane-1-carboxylate oxidase, putative / ACC oxidase, putative | chr1:2906	
ES921804	1.307	moderately similar to (432)AT1G67550| Symbols: URE | URE (UREASE); urease | chr1:25316505-25320574 FORWARDweakly similar t	
L38203	1.307	no similarity	
JCVI_3526	1.307	moderately similar to (248)AT1G14620| Symbols: DECOY | DECOY (endoxyloglucan transferase A2) | chr1:5014943-5016496 REVERS	
EX135696	1.307	no similarity	
EV174723	1.307	very weakly similar to (98.6)AT3G43810| Symbols: CAM7 | CAM7 (CALMODULIN 7); calcium ion binding | chr3:15675606-15677342 F	
CV433706	1.307	no similarity	
EE501908	1.306	no similarity	
RC_ES969201	1.306	no similarity	
JCVI_35871	1.306	weakly similar to (196)AT2G45670| Symbols: | calcineurin B subunit-related | chr2:18824154-18825456 REVERSE no original descriptio	
JCVI_8317	1.306	weakly similar to (152)AT1G04140| Symbols: | transducin family protein / WD-40 repeat family protein | chr1:1075991-1080320 REVER	
JCVI_1858	1.306	highly similar to (530)AT5G42080| Symbols: ADL1A, AG68, DRP1A | ADL1 (ARABIDOPSIS DYNAMIN-LIKE PROTEIN); GTP bindi	
JCVI_28626	1.306	highly similar to (664)AT3G43920| Symbols: DCL3 | DCL3 (DICER-LIKE 3); RNA binding / ribonuclease III | chr3:15764535-15771817	
JCVI_814	1.306	highly similar to (602)AT1G15950| Symbols: IRX4, ATCCR1, CCR1 | CCR1 (CINNAMOYL COA REDUCTASE 1) | chr1:5478849-548	

JCVI_11134	1.306	moderately similar to (415)AT1G62290| Symbols: | aspartyl protease family protein | chr1:23013772-23016346 REVERSEmoderately sim	
EE518109	1.306	weakly similar to (124)AT2G14720| Symbols: VSR-2 | VSR-2 (Vacuolar sorting receptor 2); calcium ion binding | chr2:6307960-6311238	
JCVI_28766	1.306	moderately similar to (273)AT4G30060| Symbols: | similar to unknown protein [Arabidopsis thaliana] (TAIR:AT2G19160.1); similar to u	
BQ704574	1.306	no similarity	
EV211907	1.306	moderately similar to (368)AT5G47020| Symbols: | glycine-rich protein | chr5:19099232-19107027 FORWARD [21491] 32 753 753	-1.221
JCVI_1811	1.306	moderately similar to (302)AT3G12030| Symbols: | similar to unknown protein [Arabidopsis thaliana] (TAIR:AT5G06660.1); similar to fi	
ES922654	1.306	moderately similar to (369)AT2G34770| Symbols: FAH1 | FAH1 (FATTY ACID HYDROXYLASE 1); catalytic | chr2:14673855-1467514	
EV080799	1.306	moderately similar to (236)AT3G59830| Symbols: | ankyrin protein kinase, putative | chr3:22113983-22116300 REVERSE [21444]	
AM061688	1.306	moderately similar to (254)AT3G24315| Symbols: ATSEC20 | ATSEC20 | chr3:8820606-8822452 REVERSE [17712]	
EV205980	1.305	no similarity	
JCVI_29047	1.305	no original description	-1.908
JCVI_8590	1.305	moderately similar to (327)AT5G19150| Symbols: | carbohydrate kinase family | chr5:6426282-6428404 REVERSE no original descriptio	
EV195119	1.305	moderately similar to (408)AT3G06720| Symbols: ATKAP ALPHA, AT-IMP | AT-IMP (Arabidopsis thaliana importin alpha); protein trai	
JCVI_22528	1.305	moderately similar to (421)AT1G44100| Symbols: AAP5 | AAP5 (amino acid permease 5); amino acid transmembrane transporter | chr1:1	
EX041844	1.305	no similarity	
JCVI_1303	1.304	moderately similar to (434)AT1G02280| Symbols: ATTOC33, PPI1, TOC33 | TOC33 (PLASTID PROTEIN IMPORT 1) | chr1:448665-45	
ES901991	1.304	moderately similar to (277)AT2G20960| Symbols: pEARLI4 | pEARLI4 | chr2:9014170-9016692 FORWARD [21428]	
JCVI_18737	1.304	no original description	
JCVI_37661	1.304	moderately similar to (256)AT1G06040| Symbols: STO | STO (SALT TOLERANCE); transcription factor/ zinc ion binding | chr1:182866	
JCVI_9178	1.304	moderately similar to (266)AT5G16380| Symbols: | similar to unknown protein [Arabidopsis thaliana] (TAIR:AT3G07470.1); similar to u	-1.439
EV003535	1.304	very weakly similar to (99.4)AT4G19870| Symbols: | kelch repeat-containing F-box family protein | chr4:10783064-10784266 REVERSE |	
JCVI_34917	1.303	moderately similar to (458)AT2G39940| Symbols: COI1 | COI1 (CORONATINE INSENSITIVE 1); ubiquitin-protein ligase | chr2:16679	
JCVI_38691	1.303	moderately similar to (308)AT1G58220| Symbols: | myb family transcription factor | chr1:21562053-21566334 REVERSE no original des	
JCVI_29020	1.302	moderately similar to (480)AT3G26380| Symbols: | glycosyl hydrolase family protein 27 / alpha-galactosidase family protein / melibiase f	
EE441804	1.302	no similarity	
JCVI_41997	1.302	weakly similar to (115)AT1G79380| Symbols: | copine-related | chr1:29865705-29867916 FORWARD no original description	
EE479654	1.302	no similarity	
JCVI_5023	1.302	moderately similar to (301)AT1G71780| Symbols: | similar to unnamed protein product [Vitis vinifera] (GB:CAO67543.1) | chr1:2699906	
DR697862	1.302	weakly similar to (145)AT1G79280| Symbols: NUA | NUA (NUCLEAR PORE ANCHOR) | chr1:29824069-29837702 REVERSE [17621]	
EV078595	1.302	no similarity	
JCVI_13329	1.302	moderately similar to (450)AT2G37760| Symbols: | aldo/keto reductase family protein | chr2:15839073-15840756 FORWARDweakly sim	
EV043999	1.302	no similarity	
JCVI_27523	1.301	moderately similar to (262)AT1G79700| Symbols: | ovule development protein, putative | chr1:29995333-29998551 REVERSEweakly sim	
JCVI_34263	1.301	no original description	
EE450908	1.301	moderately similar to (215)AT2G02590| Symbols: | similar to unnamed protein product [Vitis vinifera] (GB:CAO41025.1); contains Inter	
JCVI_41823	1.301	moderately similar to (399)AT4G01220| Symbols: | similar to RGXT1 (RHAMNOGALACTURONAN XYLOSYLTRANSFERASE 1), U	
JCVI_4443	1.301	moderately similar to (343)AT4G24805| Symbols: | methyltransferase | chr4:12785325-12786068 FORWARD no original description	
JCVI_12585	1.301	weakly similar to (183)AT5G19570| Symbols: | similar to hypothetical protein OsI_018061 [Oryza sativa (indica cultivar-group)] (GB:EA	
JCVI_36968	1.301	weakly similar to (129)AT3G18310| Symbols: | similar to hypothetical protein [Vitis vinifera] (GB:CAN64638.1) | chr3:6284529-628715	
JCVI_36915	1.301	highly similar to (553)AT1G55680| Symbols: | WD-40 repeat family protein | chr1:20808654-20810960 REVERSE no original descriptior	
EE480768	1.301	weakly similar to (135)AT1G14710| Symbols: | hydroxyproline-rich glycoprotein family protein | chr1:5062163-5064692 REVERSE [201	-1.351
JCVI_35800	1.301	very weakly similar to (89.7)AT3G26490| Symbols: | phototropic-responsive NPH3 family protein | chr3:9705379-9707398 FORWARD no	
EX089301	1.301	no similarity	
EE479671	1.301	weakly similar to (133)AT3G09310| Symbols: | Identical to UPF0161 protein At3g09310 [Arabidopsis Thaliana] (GB:Q9SR32); similar to	
EE556866	1.301	no similarity	
JCVI_32634	1.300	moderately similar to (455)AT2G35100| Symbols: ARAD1 | ARAD1 (ARABINAN DEFICIENT 1); catalytic/ transferase, transferring gly	-1.267
EV051730	1.300	no similarity	
EX016086	1.300	no similarity	
EE564543	1.300	no similarity	
EV125358	1.300	moderately similar to (207)AT5G65800| Symbols: CIN5, ETO2, ACS5 | ACS5 (ACC SYNTHASE 5); 1-aminocyclopropane-1-carboxylate	
EV104051	1.300	no similarity	
EV091052	1.300	weakly similar to (142)AT2G37660| Symbols: | binding / catalytic/ coenzyme binding | chr2:15802559-15804055 REVERSE [21476] 154	
EV171028	1.300	very weakly similar to (98.6)AT1G68560| Symbols: ATXYL1, XYL1 | ATXYL1/XYL1 (ALPHA-XYLOSIDASE 1); alpha-N-arabinofuran	
EV021454	1.299	no similarity	
EV176066	1.299	very weakly similar to (99.4)AT1G02500| Symbols: SAM-1, MAT1, SAM1 | SAM1 (S-adenosylmethionine synthetase 1); methionine aden	
EX052677	1.299	weakly similar to (149)AT4G04650| Symbols: | similar to unknown protein [Arabidopsis thaliana] (TAIR:AT1G43730.1); similar to putati	
JCVI_29004	1.299	moderately similar to (265)AT2G33670| Symbols: ATMLO5, MLO5 | MLO5 (MILDEW RESISTANCE LOCUS O 5); calmodulin binding	
EV173489	1.299	no similarity	
JCVI_10514	1.298	weakly similar to (147)AT1G13830| Symbols: | beta-1,3-glucanase-related | chr1:4739996-4740923 REVERSE no original description	
ES897894	1.298	weakly similar to (133)AT1G67370| Symbols: ASY1 | ASY1 (ASYNAPTIC 1); DNA binding | chr1:25243010-25247376 REVERSE [214	
JCVI_21360	1.298	moderately similar to (238)AT3G55030| Symbols: PGPS2 | PGPS2 (phosphatidylglycerolphosphate synthase 2); CDP-alcohol phosphatidy	
EV135083	1.297	weakly similar to (137)AT1G53750| Symbols: RPT1A | RPT1A (regulatory particle triple-A 1A); ATPase | chr1:20069589-20071992 REV	
JCVI_102	1.297	moderately similar to (332)AT2G38170| Symbols: ATCAX1, RCI4, CAX1 | CAX1 (CATION EXCHANGER 1); calcium:hydrogen antipo	-1.256
CX269524	1.297	weakly similar to (189)AT1G03830| Symbols: | guanylate-binding family protein | chr1:962127-966621 REVERSE [16816]	
JCVI_3067	1.297	moderately similar to (300)AT3G56680| Symbols: | nucleic acid binding | chr3:21002516-21004425 FORWARD no original description	-1.529
EX094647	1.297	moderately similar to (237)AT3G57400| Symbols: | similar to unknown protein [Arabidopsis thaliana] (TAIR:AT5G52500.1); similar to u	
JCVI_639	1.297	moderately similar to (379)AT4G32150| Symbols: VAMP711, ATVAMP711, VAMP7C | VAMP7C (VESICLE-ASSOCIATED MEMBR.	
EV015635	1.296	weakly similar to (137)AT5G64880| Symbols: | unknown protein | chr5:25949579-25951100 FORWARD [21440]	
JCVI_23364	1.296	moderately similar to (202)AT5G42000| Symbols: | ORMDL family protein | chr5:16816683-16817909 FORWARD no original descriptic	
JCVI_30121	1.296	moderately similar to (324)AT3G17900| Symbols: | similar to unnamed protein product [Vitis vinifera] (GB:CAO70781.1); similar to unn	
JCVI_2088	1.296	moderately similar to (332)AT1G71780| Symbols: | similar to unnamed protein product [Vitis vinifera] (GB:CAO67543.1) | chr1:2699906	
JCVI_39084	1.296	weakly similar to (105)AT3G11650| Symbols: NHL2 | NHL2 (NDR1/HIN1-like 2) | chr3:3676270-3676992 REVERSE no original descrip	
EV168965	1.296	no similarity	
JCVI_34568	1.296	moderately similar to (356)AT1G36990| Symbols: | similar to unknown protein [Arabidopsis thaliana] (TAIR:AT4G08510.1); similar to u	
EV172073	1.296	no similarity	
EE456776	1.295	no similarity	
JCVI_9400	1.295	weakly similar to (193)AT3G54680| Symbols: | proteophosphoglycan-related | chr3:20255546-20256667 FORWARD no original descripti	
DY023757	1.295	weakly similar to (118)AT3G32904| Symbols: | similar to unknown protein [Arabidopsis thaliana] (TAIR:AT1G43720.1) | chr3:13455151	
EX111100	1.295	moderately similar to (214)AT4G31750| Symbols: | protein phosphatase 2C, putative / PP2C, putative | chr4:15364663-15367213 REVER	
L38196	1.295	no similarity	
JCVI_30916	1.295	highly similar to (517)AT1G05890| Symbols: | zinc finger protein-related | chr1:1779630-1784165 FORWARD no original description	

JCVI_326	1.295	moderately similar to (266)AT4G36220| Symbols: CYP84A1, FAH1 | FAH1 (FERULATE-5-HYDROXYLASE 1); ferulate 5-hydroxylase	
JCVI_33882	1.295	moderately similar to (399)AT5G40480| Symbols: EMB3012 | EMB3012 (EMBRYO DEFECTIVE 3012) | chr5:16230589-16241210 FOR	
ES939052	1.295	moderately similar to (256)AT2G19260| Symbols: | ELM2 domain-containing protein / PHD finger family protein | chr2:8364413-836701	
EE561849	1.295	no similarity	
JCVI_26875	1.295	moderately similar to (439)AT3G66658| Symbols: ALDH22a1 | ALDH22a1 (ALDEHYDE DEHYDROGENASE 22A1); 3-chloroallyl alde	
JCVI_20921	1.294	moderately similar to (216)AT3G55040| Symbols: | In2-1 protein, putative | chr3:20409695-20411282 REVERSEweakly similar to (106)	
JCVI_90	1.294	moderately similar to (415)AT2G43590| Symbols: | chitinase, putative | chr2:18088669-18089826 REVERSEmoderately similar to (412)	
EV125456	1.294	weakly similar to (150)AT5G64430| Symbols: | octicosapeptide/Phox/Bem1p (PB1) domain-containing protein | chr5:25779766-2578130	
JCVI_27544	1.294	no original description	
EX130338	1.294	very weakly similar to (92.4)AT5G07980| Symbols: | dentin sialophosphoprotein-related | chr5:2549433-2554670 REVERSE [21832]	
EX037465	1.294	weakly similar to (123)AT3G49360| Symbols: | glucosamine/galactosamine-6-phosphate isomerase family protein | chr3:18314174-18315	
JCVI_39755	1.294	moderately similar to (350)AT1G02145| Symbols: | similar to mannosyltransferase, putative [Arabidopsis thaliana] (TAIR:AT5G14850.2)	
DY003408	1.294	moderately similar to (361)AT2G28880| Symbols: EMB1997 | EMB1997 (EMBRYO DEFECTIVE 1997); anthranilate synthase/ catalytic	
JCVI_35874	1.294	weakly similar to (112)AT3G23810| Symbols: SAHH2 | SAHH2 (S-ADENOSYL-L-HOMOCYSTEINE (SAH) HYDROLASE 2); adenosy	
CX271853	1.294	no similarity	
JCVI_39996	1.293	highly similar to (815)AT5G20490| Symbols: ATXIK, XIK | XIK (Myosin-like protein XIK); motor/ protein binding | chr5:6927066-6936	
JCVI_42453	1.293	weakly similar to (154)AT2G01755| Symbols: | similar to unnamed protein product [Vitis vinifera] (GB:CAO42367.1) | chr2:332248-332	
EE464285	1.293	no similarity	
JCVI_7504	1.293	moderately similar to (329)AT3G57610| Symbols: ATPURA | ATPURA; adenylosuccinate synthase | chr3:21345496-21347580 REVERS	
EV021042	1.293	moderately similar to (441)AT2G25760| Symbols: | protein kinase family protein | chr2:10992196-10995730 REVERSE [21441]	
JCVI_31078	1.293	weakly similar to (134)AT3G53500| Symbols: RSZ32 | RSZ32; nucleic acid binding | chr3:19845535-19847485 REVERSE no original des	1.559
JCVI_25602	1.293	moderately similar to (314)AT1G05180| Symbols: AXR1 | AXR1 (AUXIN RESISTANT 1); small protein activating enzyme | chr1:14985	
EE475798	1.293	weakly similar to (197)AT2G39900| Symbols: | LIM domain-containing protein | chr2:16666214-16667345 FORWARDweakly similar to	
JCVI_42317	1.293	moderately similar to (313)AT2G40750| Symbols: ATWRKY54, WRKY54 | WRKY54 (WRKY DNA-binding protein 54); transcription fa	
JCVI_28831	1.293	no original description	
JCVI_5071	1.293	highly similar to (727)AT5G04130| Symbols: | DNA topoisomerase, ATP-hydrolyzing, putative / DNA topoisomerase II, putative / DNA g	
JCVI_10934	1.293	highly similar to (535)AT1G11680| Symbols: EMB1738, CYP51A2, CYP51, CYP51G1 | CYP51G1 (CYTOCHROME P450 51); oxygen	
EV089495	1.293	moderately similar to (266)AT4G35560| Symbols: | similar to transducin family protein / WD-40 repeat family protein [Arabidopsis thalia	
JCVI_22738	1.292	highly similar to (630)AT2G05790| Symbols: | glycosyl hydrolase family 17 protein | chr2:2199447-2201290 FORWARDmoderately simi	
EE447560	1.292	no similarity	
CD829743	1.292	weakly similar to (196)AT5G57550| Symbols: XTR3 | XTR3 (XYLOGLUCAN ENDOTRANSGLYCOSYLASE 3); hydrolase, acting on g	
JCVI_13189	1.292	moderately similar to (458)AT1G51980| Symbols: | mitochondrial processing peptidase alpha subunit, putative | chr1:19327598-1933044	
EE447421	1.292	no similarity	1.598
JCVI_18353	1.292	moderately similar to (406)AT3G53130| Symbols: CYP97C1, LUT1 | LUT1 (LUTEIN DEFICIENT 1); oxygen binding | chr3:19703790-1	
CD824857	1.292	moderately similar to (293)AT1G11310| Symbols: ATMLO2, PMR2, MLO2 | MLO2 (MILDEW RESISTANCE LOCUS O 2) | chr1:3801	
EV192308	1.292	moderately similar to (259)AT3G60240| Symbols: CUM2, EIF4G | EIF4G (EUKARYOTIC TRANSLATION INITIATION FACTOR 4G)	
JCVI_8379	1.292	no original description	
JCVI_13462	1.291	moderately similar to (234)AT5G01400| Symbols: ESP4 | ESP4 (ENHANCED SILENCING PHENOTYPE 4); binding | chr5:162802-171	
EE541131	1.291	moderately similar to (309)AT5G27395| Symbols: | P-P-bond-hydrolysis-driven protein transmembrane transporter | chr5:9671848-96740	
JCVI_20737	1.291	weakly similar to (153)AT2G17760| Symbols: | aspartyl protease family protein | chr2:7720570-7723351 FORWARD no original descript	
JCVI_2730	1.291	moderately similar to (369)AT5G07460| Symbols: PMSR2 | PMSR2 (PEPTIDEMETHIONINE SULFOXIDE REDUCTASE 2); peptide-m	
JCVI_20482	1.291	moderately similar to (204)AT5G59870| Symbols: HTA6 | HTA6; DNA binding | chr5:24132831-24133370 REVERSEweakly similar to (	
JCVI_39431	1.291	weakly similar to (123)AT3G58130| Symbols: | N-acetylglucosaminyl-phosphatidylinositol de-N-acetylase-related | chr3:21538923-21540	
ES902454	1.291	weakly similar to (140)AT5G53486| Symbols: | unknown protein | chr5:21739048-21739755 FORWARD [21428] 1 523 588	1.167
JCVI_18784	1.291	moderately similar to (246)AT1G20823| Symbols: | zinc finger (C3HC4-type RING finger) family protein | chr1:7238869-7239462 FORW	
EV135130	1.291	no similarity	
JCVI_17898	1.291	weakly similar to (122)AT1G14800| Symbols: | similar to F-box family protein [Arabidopsis thaliana] (TAIR:AT2G35280.1); similar to hy	
H74799	1.290	no similarity	
EE559285	1.290	no similarity	
EV203368	1.290	very weakly similar to (81.3)AT1G14850| Symbols: NUP155 | NUP155 (Nucleoporin 155) | chr1:5116916-5123254 REVERSE [21490] 40	
JCVI_325	1.290	highly similar to (788)AT1G02080| Symbols: | transcriptional regulator-related | chr1:373694-386682 FORWARD no original description	
JCVI_20636	1.290	moderately similar to (291)AT5G20190| Symbols: | binding | chr5:6814095-6815173 FORWARD no original description	
EE469173	1.290	weakly similar to (188)AT2G01870| Symbols: | similar to unnamed protein product [Vitis vinifera] (GB:CAO21902.1) | chr2:389845-3903	
DY029691	1.290	weakly similar to (106)AT2G29310| Symbols: | tropinone reductase, putative / tropine dehydrogenase, putative | chr2:12597145-1259833	
EX092363	1.290	moderately similar to (394)AT1G08610| Symbols: | pentatricopeptide (PPR) repeat-containing protein | chr1:2733791-2735470 REVERS	
EX093457	1.290	weakly similar to (158)AT5G04160| Symbols: | phosphate translocator-related | chr5:1143034-1144778 REVERSE [21823]	
EE484552	1.289	no similarity	
CD835918	1.289	weakly similar to (196)AT3G08040| Symbols: MAN1, FRD3 | FRD3 (FERRIC REDUCTASE DEFECTIVE 3); antiporter | chr3:2566599-	
EV133287	1.288	weakly similar to (152)AT2G46210| Symbols: | delta-8 sphingolipid desaturase, putative | chr2:18984615-18985964 FORWARD [21481]	
JCVI_11136	1.288	moderately similar to (257)AT4G01850| Symbols: SAM-2, MAT2 | MAT2/SAM-2 (S-adenosylmethionine synthetase 2) | chr4:796298-79	
JCVI_34414	1.288	moderately similar to (364)AT4G18030| Symbols: | dehydration-responsive family protein | chr4:10012862-10015279 REVERSE no origi	
EV214620	1.288	moderately similar to (296)AT3G54030| Symbols: | protein kinase family protein | chr3:20022140-20024468 FORWARD [21491] 62 725	
EV110672	1.288	no similarity	
JCVI_14692	1.288	moderately similar to (343)AT3G52470| Symbols: | harpin-induced family protein / HIN1 family protein / harpin-responsive family protei	-2.469
EE420397	1.288	weakly similar to (150)AT1G67680| Symbols: | 7S RNA binding | chr1:25369625-25372127 REVERSE [20149]	
EV188499	1.288	moderately similar to (386)AT3G46610| Symbols: | pentatricopeptide (PPR) repeat-containing protein | chr3:17171209-17173206 REVER	
EX126020	1.288	weakly similar to (117)AT1G47720| Symbols: OSB1 | OSB1 (ORGANELLAR SINGLE-STRANDED); single-stranded DNA binding | chr	
JCVI_11905	1.287	moderately similar to (219)AT5G08060| Symbols: | similar to unknown [Populus trichocarpa] (GB:ABK93975.1) | chr5:2580589-258098	
JCVI_35147	1.287	no original description	
JCVI_30259	1.287	no original description	
EE504270	1.287	no similarity	
JCVI_10476	1.287	moderately similar to (255)AT1G64060| Symbols: ATRBOHF, RBOHAP108, RBOHF, ATRBOH F | ATRBOH F (RESPIRATORY BUR	
ES931230	1.287	weakly similar to (150)AT3G18860| Symbols: | transducin family protein / WD-40 repeat family protein | chr3:6501780-6508358 FORWA	
EV050881	1.287	moderately similar to (222)AT5G17690| Symbols: LHP1, TFL2 | TFL2 (TERMINAL FLOWER 2) | chr5:5827506-5829539 REVERSE [2	
ES945600	1.287	no similarity	
JCVI_12224	1.287	weakly similar to (152)AT1G32270| Symbols: SYP24, ATSYP24 | ATSYP24 (syntaxin 24); protein binding | chr1:11642573-11644942 FC	
JCVI_16079	1.287	highly similar to (651)AT5G64570| Symbols: ATBXL4, XYL4 | XYL4 (beta-xylosidase 4); hydrolase, hydrolyzing O-glycosyl compounds	
JCVI_34385	1.286	moderately similar to (457)AT3G16760| Symbols: | tetratricopeptide repeat (TPR)-containing protein | chr3:5703219-5705086 FORWAR	
JCVI_20200	1.286	weakly similar to (114)AT1G07020| Symbols: | similar to unnamed protein product [Vitis vinifera] (GB:CAO21378.1) | chr1:2155318-215	-1.767
JCVI_29495	1.286	moderately similar to (360)AT1G18660| Symbols: | zinc finger (C3HC4-type RING finger) family protein | chr1:6421425-6425557 FORW	-1.279
JCVI_28325	1.286	moderately similar to (466)AT3G56040| Symbols: | similar to hypothetical protein OsJ_018104 [Oryza sativa (japonica cultivar-group)] ((	

JCVI_1161	1.286	moderately similar to (413)AT4G11570| Symbols: | haloacid dehalogenase-like hydrolase family protein | chr4:7004737-7005858 FORWA	
RC_EE474158	1.286	no similarity	
JCVI_6900	1.286	moderately similar to (378)AT3G07320| Symbols: | glycosyl hydrolase family 17 protein | chr3:2332330-2333931 REVERSEweakly simil	
BG544859	1.286	no similarity	1.071
JCVI_34370	1.286	moderately similar to (323)AT5G38360| Symbols: | catalytic | chr5:15349519-15350786 REVERSE no original description	
JCVI_22475	1.286	moderately similar to (203)AT5G59960| Symbols: | similar to unnamed protein product [Vitis vinifera] (GB:CAO21698.1) | chr5:2415957	
JCVI_3665	1.286	moderately similar to (439)AT1G66580| Symbols: | 60S ribosomal protein L10 (RPL10C) | chr1:24842871-24844102 FORWARDmodera	
EX063658	1.285	weakly similar to (110)AT1G25260| Symbols: | acidic ribosomal protein P0-related | chr1:8854150-8855753 REVERSE [21814]	
EV052452	1.285	very weakly similar to (88.6)AT5G16453| Symbols: | Encodes a defensin-like (DEFL) family protein. | chr5:5375546-5375922 REVERSE	
EX133733	1.285	no similarity	
EE545557	1.285	no similarity	
EE448577	1.285	no similarity	
L46469	1.285	weakly similar to (159)AT3G53780| Symbols: | rhomboid family protein | chr3:19935707-19937626 REVERSE [132]	
CD824937	1.284	weakly similar to (163)AT5G09680| Symbols: | cytochrome b5 domain-containing protein | chr5:2999363-3000186 REVERSE [13979] 1	
BG544222	1.284	weakly similar to (187)AT1G15670| Symbols: | kelch repeat-containing F-box family protein | chr1:5390114-5391193 FORWARD [8791]	
JCVI_6071	1.284	moderately similar to (475)AT3G11960| Symbols: | cleavage and polyadenylation specificity factor (CPSF) A subunit C-terminal domain-	1.262
JCVI_16619	1.284	highly similar to (548)AT5G51070| Symbols: CLPD, ERD1 | ERD1 (EARLY RESPONSIVE TO DEHYDRATION 1); ATP binding / ATI	
RC_AM387594	1.284	no similarity	
JCVI_511	1.284	moderately similar to (490)AT1G27930| Symbols: | similar to unknown protein [Arabidopsis thaliana] (TAIR:AT1G67330.1); similar to u	
ES898670	1.284	no similarity	
JCVI_4491	1.283	moderately similar to (385)AT3G45740| Symbols: | hydrolase family protein / HAD-superfamily protein | chr3:16801815-16804265 FORV	
JCVI_3350	1.283	moderately similar to (384)AT4G16210| Symbols: | enoyl-CoA hydratase/isomerase family protein | chr4:9176879-9177993 REVERSEve	
JCVI_15845	1.283	weakly similar to (117)AT5G27620| Symbols: CYCH;1 | CYCH;1 (CYCLIN H;1); cyclin-dependent protein kinase/ protein binding / prote	
EV123228	1.283	no similarity	
ES931347	1.283	no similarity	
JCVI_22183	1.283	no original description	
EX101161	1.283	moderately similar to (242)AT1G69880| Symbols: ATH8 | ATH8 (thioredoxin H-type 8); thiol-disulfide exchange intermediate | chr1:2632	
ES934064	1.282	no similarity	
EE440012	1.282	weakly similar to (127)AT3G16620| Symbols: ATTOC120 | ATTOC120 (Arabidopsis thaliana translocon outer complex protein 120); GT	
JCVI_9673	1.282	weakly similar to (162)AT5G59970| Symbols: | histone H4 | chr5:24163578-24163889 REVERSEweakly similar to (162)H4_PEA no orig	
JCVI_5793	1.282	moderately similar to (212)AT1G74230| Symbols: GR-RBP5 | GR-RBP5 (glycine-rich RNA-binding protein 5); RNA binding | chr1:27919	
JCVI_939	1.282	moderately similar to (474)AT3G06490| Symbols: AtMYB108, BOS1, MYB108 | MYB108 (MYB DOMAIN PROTEIN 108); DNA bindi	
JCVI_35645	1.282	highly similar to (650)AT3G46220| Symbols: | similar to unnamed protein product [Vitis vinifera] (GB:CAO21736.1) | chr3:16991592-16	
EV211944	1.282	no similarity	1.247
EV104385	1.282	no similarity	
JCVI_11525	1.282	moderately similar to (347)AT1G30110| Symbols: | diadenosine 5',5'''-P1,P4-tetraphosphate hydrolase, putative | chr1:10582684-1058380	
JCVI_31909	1.282	no original description	
JCVI_34286	1.281	moderately similar to (215)AT5G06050| Symbols: | dehydration-responsive protein-related | chr5:1820197-1823573 FORWARD no origir	
EE548751	1.281	weakly similar to (196)AT3G26510| Symbols: | octicosapeptide/Phox/Bem1p (PB1) domain-containing protein | chr3:9713123-9713779 F	
EH424303	1.281	moderately similar to (218)AT4G08500| Symbols: ATMEKK1, MAPKKK8, MEKK1 | MEKK1 (MYTOGEN ACTIVATED PROTEIN K	
JCVI_11276	1.281	moderately similar to (325)AT5G24590| Symbols: ANAC091, TIP | ANAC091/TIP (TCV-INTERACTING PROTEIN); transcription coac	
JCVI_12509	1.280	weakly similar to (160)AT2G37480| Symbols: | similar to unknown protein [Arabidopsis thaliana] (TAIR:AT3G53670.1); similar to hypot	
EE434865	1.280	moderately similar to (222)AT1G19010| Symbols: | similar to unknown protein [Arabidopsis thaliana] (TAIR:AT1G74860.1); similar to h	
JCVI_15971	1.280	very weakly similar to (86.7)AT3G01160| Symbols: | similar to unnamed protein product [Vitis vinifera] (GB:CAO23260.1); contains dom	
EX102752	1.280	very weakly similar to (100)AT3G58630| Symbols: | transcription factor | chr3:21694905-21696748 REVERSE [21826]	
RC_EX093160	1.280	no similarity	
EV203243	1.280	weakly similar to (140)AT3G52140| Symbols: | tetratricopeptide repeat (TPR)-containing protein | chr3:19344210-19352273 FORWARD	
EE569302	1.280	no similarity	
JCVI_13972	1.280	moderately similar to (357)AT4G00110| Symbols: GAE3 | GAE3 (UDP-D-GLUCURONATE 4-EPIMERASE 3); catalytic | chr4:38702-39	
EV017945	1.280	very weakly similar to (87.4)AT1G30880| Symbols: | unknown protein | chr1:10993360-10994037 REVERSE [21440]	
JCVI_23222	1.279	very weakly similar to (86.7)AT5G26260| Symbols: | meprin and TRAF homology domain-containing protein / MATH domain-containing	
EE547307	1.279	weakly similar to (134)AT3G56240| Symbols: CCH | CCH (COPPER CHAPERONE) | chr3:20874439-20875381 REVERSE [20128] 45 6	
JCVI_37432	1.279	very weakly similar to (89.0)AT5G24150| Symbols: SQP1 | SQP1 (Squalene monooxygenase 1) | chr5:8172673-8175398 REVERSEweakly	
EV021956	1.279	no similarity	
DY010625	1.279	no similarity	
ES269383	1.279	moderately similar to (401)AT1G11770| Symbols: | electron carrier | chr1:3975678-3977288 FORWARD [21032]	
JCVI_4369	1.279	weakly similar to (161)AT1G62790| Symbols: | protease inhibitor/seed storage/lipid transfer protein (LTP) family protein | chr1:23256034	
JCVI_12662	1.279	highly similar to (984)AT2G20330| Symbols: | transducin family protein / WD-40 repeat family protein | chr2:8779969-8782599 REVERS	
EV110397	1.279	no similarity	
EE531761	1.279	weakly similar to (184)AT5G08180| Symbols: | ribosomal protein L7Ae/L30e/S12e/Gadd45 family protein | chr5:2631844-2633375 REVI	
CX194014	1.279	weakly similar to (122)AT5G63550| Symbols: | GTP binding / RNA binding | chr5:25462031-25465160 FORWARD [16807]	
JCVI_37791	1.278	no original description	
JCVI_22150	1.278	moderately similar to (347)AT5G10550| Symbols: GTE2 | GTE2 (GLOBAL TRANSCRIPTION FACTOR GROUP E 2); DNA binding | c	
EX131128	1.278	highly similar to (601)AT3G55410| Symbols: | 2-oxoglutarate dehydrogenase E1 component, putative / oxoglutarate decarboxylase, putati	
EV183960	1.278	weakly similar to (191)AT3G49140| Symbols: | binding | chr3:18223172-18228833 REVERSE [21487] 39 779 779	
JCVI_543	1.278	weakly similar to (123)AT5G47190| Symbols: | ribosomal protein L19 family protein | chr5:19181659-19183291 REVERSE no original de	
JCVI_2360	1.278	highly similar to (521)AT2G44770| Symbols: | phagocytosis and cell motility protein ELMO1-related | chr2:18464892-18466961 FORWA	
EX102057	1.278	very weakly similar to (91.3)AT4G25240| Symbols: SKS1 | SKS1 (SKU5 SIMILAR 1); copper ion binding | chr4:12930549-12933573 FOF	
EX093771	1.278	no similarity	
ES905903	1.278	moderately similar to (349)AT3G16060| Symbols: | kinesin motor family protein | chr3:5447509-5451202 FORWARD [21429]	
JCVI_42090	1.278	moderately similar to (274)AT2G23320| Symbols: WRKY15 | WRKY15 (WRKY DNA-binding protein 15); transcription factor | chr2:993	-2.129
JCVI_1074	1.278	moderately similar to (442)AT1G13730| Symbols: | nuclear transport factor 2 (NTF2) family protein / RNA recognition motif (RRM)-con	
EV222617	1.277	weakly similar to (170)AT3G13790| Symbols: ATCWINV1, ATBFRUCT1 | ATBFRUCT1/ATCWINV1 (ARABIDOPSIS THALIANA CI	
JCVI_38559	1.277	very weakly similar to (95.9)AT2G31305| Symbols: | similar to Protein phosphatase inhibitor [Medicago truncatula] (GB:ABN09808.1); co	
EX037501	1.277	no similarity	
JCVI_29126	1.277	highly similar to (521)AT1G78510| Symbols: SPS1 | SPS1 (SOLANESYL DIPHOSPHATE SYNTHASE 1) | chr1:29540303-29541935 RI	
JCVI_42428	1.277	moderately similar to (224)AT3G17440| Symbols: ATNPSN13, NPSN13 | NPSN13 (novel plant SNARE 13) | chr3:5970159-5972296 REV	
EV129168	1.277	no similarity	
AM395845	1.277	weakly similar to (120)AT4G22100| Symbols: | glycosyl hydrolase family 1 protein | chr4:11707382-11709944 REVERSE [20346]	
JCVI_15227	1.276	moderately similar to (438)AT1G78100| Symbols: | F-box family protein | chr1:29392800-29393804 FORWARD no original description	
EH417831	1.276	moderately similar to (206)AT3G19290| Symbols: AREB2, ABF4 | ABF4 (ABRE BINDING FACTOR 4); DNA binding / transcription act	

JCVI_7985	1.276	no original description	
JCVI_5707	1.276	highly similar to (576)AT2G36390| Symbols: BE3, SBE2.1 | SBE2.1 (STARCH BRANCHING ENZYME 2.1); 1,4-alpha-glucan branching	
JCVI_1680	1.276	moderately similar to (226)AT4G13930| Symbols: SHM4 | SHM4 (SERINE HYDROXYMETHYLTRANSFERASE 4); glycine hydroxym	
JCVI_34768	1.276	moderately similar to (362)AT1G01930| Symbols: | zinc finger protein-related | chr1:320041-322809 REVERSE no original description	
ES956116	1.276	no similarity	
JCVI_10812	1.276	moderately similar to (236)AT5G17840| Symbols: | chaperone protein dnaJ-related | chr5:5895881-5897057 REVERSE no original descri	
EX040739	1.276	no similarity	-1.498
JCVI_14638	1.276	no original description	
JCVI_28693	1.275	moderately similar to (318)AT3G27250| Symbols: | similar to unknown protein [Arabidopsis thaliana] (TAIR:AT5G40800.1); similar to u	1.464
JCVI_8622	1.275	moderately similar to (360)AT5G44450| Symbols: | similar to hypothetical protein OsI_013284 [Oryza sativa (indica cultivar-group)] (GB	
ES990255	1.275	no similarity	
EE402180	1.275	very weakly similar to (89.4)AT4G36420| Symbols: | ribosomal protein L12 family protein | chr4:17203721-17204260 REVERSE [20197]	
RC_ES264884	1.275	no similarity	
JCVI_2228	1.275	moderately similar to (275)AT1G61150| Symbols: | similar to unknown protein [Arabidopsis thaliana] (TAIR:AT4G09300.1); similar to u	
ES969062	1.275	no similarity	
EX095316	1.275	moderately similar to (292)AT3G04930| Symbols: | transcription regulator | chr3:1363035-1364405 FORWARD [21824] 1 764 778	
EV128855	1.275	no similarity	
JCVI_16521	1.275	moderately similar to (212)AT3G51600| Symbols: LTP5 | LTP5 (LIPID TRANSFER PROTEIN 5); lipid transporter | chr3:19149640-1915	
JCVI_38456	1.275	no original description	
JCVI_19059	1.275	moderately similar to (348)AT1G27590| Symbols: | similar to phosphatidylinositol 3- and 4-kinase family protein [Arabidopsis thaliana] ('	
EX036925	1.275	no similarity	
JCVI_9524	1.275	highly similar to (660)AT3G30775| Symbols: AT-POX, ATPOX, ATPDH, PRO1, PRODH, ERD5 | ERD5 (EARLY RESPONSIVE TO D	
JCVI_10472	1.274	highly similar to (724)AT2G43240| Symbols: | similar to ATUTR6/UTR6 (UDP-GALACTOSE TRANSPORTER 6), nucleotide-sugar tra	
EX117804	1.274	moderately similar to (302)AT5G55520| Symbols: | similar to unknown protein [Arabidopsis thaliana] (TAIR:AT4G26660.1); similar to u	
JCVI_38205	1.274	no original description	
JCVI_19616	1.273	weakly similar to (177)AT5G24300| Symbols: SSI, ATSS1 | ATSS1/SSI (STARCH SYNTHASE I); transferase, transferring glycosyl grou	
JCVI_11274	1.273	very weakly similar to (97.1)AT4G10810| Symbols: | similar to unknown protein [Arabidopsis thaliana] (TAIR:AT4G24026.1) | chr4:6645	
DY003709	1.273	weakly similar to (193)AT1G18340| Symbols: | basal transcription factor complex subunit-related | chr1:6311605-6313727 REVERSE [18	
JCVI_11005	1.273	moderately similar to (286)AT5G55550| Symbols: | RNA recognition motif (RRM)-containing protein | chr5:22519361-22520884 REVEF	1.560
JCVI_22202	1.273	weakly similar to (107)AT1G06760| Symbols: | histone H1, putative | chr1:2076686-2077615 REVERSEvery weakly similar to (92.4)H1_	
JCVI_27	1.273	moderately similar to (370)AT3G17860| Symbols: JAZ3, JAI3, TIFY6B | JAI3/JAZ3/TIFY6B (JASMONATE-ZIM-DOMAIN PROTEIN	
JCVI_6687	1.272	no original description	
EE424048	1.272	weakly similar to (146)AT5G42520| Symbols: BPC6, BBR/BPC6, ATBPC6 | ATBPC6/BBR/BPC6/BPC6 (BASIC PENTACYSTEINE 6)	
JCVI_7005	1.272	no original description	
JCVI_15452	1.272	weakly similar to (166)AT1G52200| Symbols: | similar to unknown protein [Arabidopsis thaliana] (TAIR:AT3G18470.1); similar to unna	-1.209
EE440318	1.272	moderately similar to (213)AT3G06330| Symbols: | zinc finger (C3HC4-type RING finger) family protein | chr3:1917340-1919336 FORW	
JCVI_42184	1.272	moderately similar to (478)AT5G44100| Symbols: CKL7 | CKL7 (Casein Kinase I-like 7); casein kinase I/ kinase | chr5:17766681-177695	
EV216887	1.272	moderately similar to (228)AT3G05840| Symbols: ATSK12 | ATSK12 (Arabidopsis thaliana SHAGGY-like kinase 12); protein kinase | chr	1.723
JCVI_23179	1.272	weakly similar to (120)AT4G10890| Symbols: | similar to unknown protein [Arabidopsis thaliana] (TAIR:AT1G43722.1); similar to unna	
EE460327	1.272	very weakly similar to (91.7)AT1G64255| Symbols: | SWIM zinc finger family protein | chr1:23848617-23850869 FORWARD [20152]	1.320
ES922981	1.272	moderately similar to (377)AT4G25270| Symbols: | pentatricopeptide (PPR) repeat-containing protein | chr4:12937263-12938846 REVEF	
ES983349	1.272	moderately similar to (317)AT3G03800| Symbols: ATSYP131, SYP131 | SYP131 (syntaxin 131); SNAP receptor | chr3:969321-971467 F	
JCVI_7978	1.272	highly similar to (525)AT2G39750| Symbols: | dehydration-responsive family protein | chr2:16586064-16589359 REVERSE no original d	
JCVI_16564	1.272	moderately similar to (255)AT1G15350| Symbols: | similar to unknown protein [Arabidopsis thaliana] (TAIR:AT3G15770.2); similar to u	
EE418095	1.271	weakly similar to (154)AT1G56423| Symbols: | similar to unnamed protein product [Vitis vinifera] (GB:CAO17431.1) | chr1:21132740-2	
EE568886	1.271	no similarity	
ES962647	1.271	no similarity	
EX086791	1.271	no similarity	
EX083949	1.271	no similarity	
JCVI_26909	1.270	moderately similar to (327)AT1G71410| Symbols: | protein kinase family protein | chr1:26916732-26921177 REVERSE no original descri	
JCVI_19066	1.270	moderately similar to (381)AT4G17070| Symbols: | peptidyl-prolyl cis-trans isomerase | chr4:9595542-9598157 REVERSE no original de	
CB686426	1.270	weakly similar to (187)AT2G36970| Symbols: | UDP-glucoronosyl/UDP-glucosyl transferase family protein | chr2:15536129-15537791 F	
CD821558	1.270	weakly similar to (200)AT2G45950| Symbols: ASK20 | ASK20 (ARABIDOPSIS SKP1-LIKE 20) | chr2:18911746-18914040 REVERSE [	1.338
JCVI_5018	1.270	moderately similar to (352)AT4G38090| Symbols: | similar to unnamed protein product [Vitis vinifera] (GB:CAO22676.1); similar to hyp	
JCVI_1361	1.270	moderately similar to (440)AT1G19140| Symbols: | similar to unnamed protein product [Vitis vinifera] (GB:CAO65215.1); contains Inter	
JCVI_22486	1.270	moderately similar to (384)AT4G38260| Symbols: | similar to unknown protein [Arabidopsis thaliana] (TAIR:AT1G20740.1); similar to u	
ES912167	1.270	weakly similar to (199)AT5G61730| Symbols: ATATH11 | ATATH11 (ABC2 homolog 11); ATPase, coupled to transmembrane movemer	1.385
EV206863	1.270	moderately similar to (207)AT4G37520| Symbols: | peroxidase 50 (PER50) (P50) (PRXR2) | chr4:17631698-17633054 FORWARDvery v	
JCVI_16049	1.269	moderately similar to (442)AT1G49710| Symbols: ATFUT12, FUCTB, FUCT2, FUT12 | FUT12 (fucosyltransferase 12); fucosyltransferas	
EX108870	1.269	very weakly similar to (85.9)AT1G69620| Symbols: RPL34 | RPL34 (RIBOSOMAL PROTEIN L34); structural constituent of ribosome | cl	
EE514629	1.269	no similarity	
EE522465	1.269	moderately similar to (353)AT5G10790| Symbols: UBP22 | UBP22 (UBIQUITIN-SPECIFIC PROTEASE 22); ubiquitin-specific protease	
EV012860	1.269	no similarity	1.611
JCVI_3626	1.269	moderately similar to (361)AT4G25420| Symbols: ATGA20OX1, GA20OX1, AT2301, GA5 | GA5 (GA REQUIRING 5); gibberellin 20-o	
JCVI_39639	1.269	moderately similar to (408)AT5G04885| Symbols: | glycosyl hydrolase family 3 protein | chr5:1423370-1426629 FORWARD no original c	
ES922266	1.269	very weakly similar to (90.1)AT5G45570| Symbols: | Ulp1 protease family protein | chr5:18489528-18492801 FORWARD [15718]	
AM388754	1.268	moderately similar to (207)AT4G35890| Symbols: | La domain-containing protein | chr4:16997436-17000413 FORWARD [20118]	
CX278794	1.268	very weakly similar to (91.3)AT2G47710| Symbols: | universal stress protein (USP) family protein | chr2:19562115-19563026 REVERSE |	
CN729250	1.268	weakly similar to (192)AT2G32680| Symbols: | disease resistance family protein | chr2:13867019-13869691 REVERSE [15725]	-1.247
JCVI_9124	1.268	weakly similar to (176)AT3G19290| Symbols: AREB2, ABF4 | ABF4 (ABRE BINDING FACTOR 4); DNA binding / transcription activat	
JCVI_23574	1.268	weakly similar to (129)AT2G47230| Symbols: | agenet domain-containing protein | chr2:19394196-19397081 FORWARD no original desc	
EX120790	1.267	moderately similar to (332)AT4G18800| Symbols: AthSGBP, AtRab11B, AtRABA1d | AtRABA1d/AtRab11B/AthSGBP (Arabidopsis Ral	
EE424112	1.267	weakly similar to (127)AT4G16710| Symbols: | glycosyltransferase family protein 28 | chr4:9398840-9399640 FORWARD [20158]	
EE468765	1.267	weakly similar to (167)AT2G40095| Symbols: | similar to unknown protein [Arabidopsis thaliana] (TAIR:AT3G55880.2); similar to unna	
JCVI_182	1.267	moderately similar to (439)AT4G05050| Symbols: UBQ11 | UBQ11 (UBIQUITIN 11) | chr4:2588269-2588958 REVERSEweakly similar	
EV105085	1.267	no similarity	
EV157782	1.267	no similarity	
JCVI_37681	1.267	weakly similar to (134)AT2G42975| Symbols: | similar to unnamed protein product [Vitis vinifera] (GB:CAO69996.1) | chr2:17881075-1	
JCVI_3070	1.267	moderately similar to (348)AT2G31440| Symbols: | protein binding | chr2:13406386-13408048 REVERSE no original description	-1.438
EV143001	1.266	no similarity	
JCVI_8629	1.266	moderately similar to (310)AT1G01780| Symbols: | LIM domain-containing protein | chr1:282919-284082 FORWARDweakly similar to (	

ES942671	1.266	moderately similar to (243)AT3G03030| Symbols: | F-box family protein | chr3:682259-683857 FORWARD [21391]	1.866
DN963009	1.266	weakly similar to (122)AT5G18640| Symbols: | lipase class 3 family protein | chr5:6213249-6215496 FORWARD [17359]	
ES937769	1.266	weakly similar to (179)AT3G62200| Symbols: | similar to EDA32 (embryo sac development arrest 32) [Arabidopsis thaliana] (TAIR:AT3(	
EE461347	1.266	moderately similar to (240)AT3G05740| Symbols: RECQI1 | RECQI1 (Arabidopsis RecQ helicase l1); ATP-dependent helicase | chr3:169	
EE443179	1.265	moderately similar to (275)AT5G60950| Symbols: COBL5 | COBL5 (COBRA-LIKE PROTEIN 5 PRECURSOR) | chr5:24544383-245452	
EH423228	1.265	moderately similar to (296)AT1G29720| Symbols: | protein kinase family protein | chr1:10393880-10395067 REVERSEvery weakly simil	
JCVI_26819	1.265	moderately similar to (385)AT5G23450| Symbols: ATLCBK1 | ATLCBK1 (A. THALIANA LONG-CHAIN BASE (LCB) KINASE 1); dia	1.647
JCVI_37109	1.265	moderately similar to (301)AT5G25220| Symbols: KNAT3 | KNAT3 (KNOTTED1-LIKE HOMEOBOX GENE 3) | chr5:8736211-873809	
JCVI_33572	1.265	weakly similar to (127)AT1G32560| Symbols: | late embryogenesis abundant group 1 domain-containing protein / LEA group 1 domain-co	
DY017480	1.265	no similarity	
EE464960	1.265	moderately similar to (287)AT1G71800| Symbols: | cleavage stimulation factor, putative | chr1:27003268-27005512 FORWARD [20171]	
EE568494	1.265	no similarity	1.359
EE454164	1.265	moderately similar to (219)AT3G48560| Symbols: ALS, AHAS, TZP5, IMR1, CSR1 | CSR1 (CHLORSULFURON/IMIDAZOLINONE RI	
JCVI_39624	1.264	moderately similar to (229)AT4G26640| Symbols: ATWRKY20, WRKY20 | WRKY20 (WRKY DNA-binding protein 20); transcription fa	
DY017774	1.264	no similarity	
JCVI_10238	1.264	highly similar to (542)AT5G48160| Symbols: | tropomyosin-related | chr5:19545245-19547046 REVERSE no original description	
EX032983	1.264	weakly similar to (160)AT1G69520| Symbols: | methyltransferase-related | chr1:26131951-26133265 FORWARD [21810]	
EE418539	1.264	no similarity	
AM395510	1.264	very weakly similar to (98.6)AT2G45540| Symbols: | WD-40 repeat family protein / beige-related | chr2:18764955-18779303 REVERSE [1	
RC_AT000563	1.264	no similarity	
EE534540	1.264	no similarity	
EX080276	1.264	weakly similar to (135)AT1G52930| Symbols: | brix domain-containing protein | chr1:19714929-19716771 FORWARD [21818]	
JCVI_42042	1.263	no original description	
JCVI_7591	1.263	no original description	
EE552236	1.263	no similarity	
JCVI_9159	1.263	weakly similar to (139)AT1G54560| Symbols: ATXIE, PCR1, XIE | XIE (Myosin-like protein XIE); motor/ protein binding | chr1:2037531	
JCVI_11533	1.263	weakly similar to (183)AT3G06170| Symbols: | TMS membrane family protein / tumour differentially expressed (TDE) family protein | ch	
JCVI_8815	1.263	moderately similar to (251)AT1G67070| Symbols: DIN9 | DIN9 (DARK INDUCIBLE 9); mannose-6-phosphate isomerase | chr1:2504598	
JCVI_42322	1.262	weakly similar to (134)AT3G29390| Symbols: RIK | RIK (RS2-INTERACTING KH PROTEIN) | chr3:11290912-11295761 FORWARD r	
JCVI_21802	1.262	no original description	
CD819238	1.262	no similarity	
ES910064	1.262	moderately similar to (222)AT5G65140| Symbols: | trehalose-6-phosphate phosphatase, putative | chr5:26037637-26039303 REVERSE [2	
JCVI_2881	1.262	moderately similar to (315)AT4G04640| Symbols: ATPC1 | ATPC1 (ATP synthase gamma chain 1) | chr4:2350759-2351880 REVERSEm	
EV100927	1.261	weakly similar to (134)AT1G02750| Symbols: | zinc ion binding | chr1:602673-604134 FORWARD [21477] 79 762 762	
L37650	1.261	no similarity	
ES913719	1.261	moderately similar to (227)AT4G04450| Symbols: ATWRKY42, WRKY42 | WRKY42 (WRKY DNA-binding protein 42); transcription fa	
JCVI_18038	1.261	highly similar to (573)AT2G39120| Symbols: | similar to unknown protein [Arabidopsis thaliana] (TAIR:AT3G58520.1); similar to unnam	
DY010503	1.261	moderately similar to (388)AT1G09190| Symbols: | pentatricopeptide (PPR) repeat-containing protein | chr1:2966265-2967719 REVERSI	
EX087671	1.261	weakly similar to (182)AT3G54970| Symbols: | catalytic | chr3:20379550-20381047 REVERSE [21823]	
JCVI_5234	1.261	weakly similar to (191)AT5G45380| Symbols: | sodium:solute symporter family protein | chr5:18408564-18412923 FORWARD no origina	
JCVI_16254	1.260	weakly similar to (196)AT1G70190| Symbols: | ribosomal protein L12 family protein | chr1:26434279-26434905 FORWARD no original (	
JCVI_30601	1.260	moderately similar to (363)AT5G08680| Symbols: | ATP synthase beta chain, mitochondrial, putative | chr5:2821993-2824684 FORWAR	
EV044228	1.260	weakly similar to (137)AT3G08780| Symbols: | similar to unnamed protein product [Vitis vinifera] (GB:CAO48785.1) | chr3:2666520-26(	-1.181
ES267860	1.260	weakly similar to (173)AT4G15980| Symbols: | pectinesterase family protein | chr4:9057478-9059995 REVERSEvery weakly similar to (9	
JCVI_933	1.260	weakly similar to (125)AT3G60280| Symbols: UCC3 | UCC3 (UCLACYANIN 3); copper ion binding | chr3:22290842-22291608 REVERS	
EV086506	1.260	no similarity	
EE556142	1.260	moderately similar to (314)AT4G00240| Symbols: PLDBETA2 | PLDBETA2 (phospholipase D beta 2) | chr4:106380-110718 REVERSEv	
JCVI_8434	1.260	moderately similar to (236)AT5G61490| Symbols: | similar to unknown protein [Arabidopsis thaliana] (TAIR:AT4G25170.1); similar to u	
JCVI_40037	1.260	moderately similar to (451)AT4G35880| Symbols: | aspartyl protease family protein | chr4:16993342-16995724 FORWARD no original de	
JCVI_4837	1.260	weakly similar to (155)AT3G52230| Symbols: | similar to unknown [Populus trichocarpa] (GB:ABK93315.1) | chr3:19382303-19383375 I	
DY010700	1.260	moderately similar to (281)AT1G16270| Symbols: | protein kinase family protein | chr1:5563884-5568139 FORWARD [18980]	
EV200962	1.259	moderately similar to (243)AT4G24680| Symbols: | similar to unnamed protein product [Vitis vinifera] (GB:CAO64289.1) | chr4:1273343	
JCVI_37040	1.259	no original description	
EV102882	1.259	no similarity	
JCVI_3801	1.259	weakly similar to (156)AT2G41840| Symbols: | 40S ribosomal protein S2 (RPS2C) | chr2:17467094-17468476 REVERSE no original desc	
JCVI_11552	1.259	moderately similar to (366)AT3G54270| Symbols: | sucrose-phosphatase 3 (SPP3) | chr3:20098397-20100713 REVERSE no original desc	
JCVI_22203	1.259	moderately similar to (465)AT2G37540| Symbols: | short-chain dehydrogenase/reductase (SDR) family protein | chr2:15758773-1576089	
JCVI_18830	1.259	moderately similar to (270)AT4G26490| Symbols: | similar to unknown protein [Arabidopsis thaliana] (TAIR:AT5G56050.1); similar to h	-1.199
EE516133	1.259	moderately similar to (367)AT5G49020| Symbols: | protein arginine N-methyltransferase family protein | chr5:19888567-19891909 FORV	
JCVI_16363	1.258	moderately similar to (236)AT1G12200| Symbols: | flavin-containing monooxygenase family protein / FMO family protein | chr1:4137625	
RC_L38165	1.258	no similarity	
BQ704223	1.258	very weakly similar to (97.1)AT1G32150| Symbols: | bZIP transcription factor family protein | chr1:11566002-11568297 FORWARD [11(	
JCVI_34819	1.258	moderately similar to (211)AT5G57260| Symbols: CYP71B10 | CYP71B10 (cytochrome P450, family 71, subfamily B, polypeptide 10); o	
JCVI_20488	1.258	moderately similar to (299)AT5G24690| Symbols: | similar to RER1 (RETICULATA-RELATED 1) [Arabidopsis thaliana] (TAIR:AT5G2	
EE409861	1.258	no similarity	
H07485	1.258	no similarity	
JCVI_7940	1.258	moderately similar to (253)AT4G22756| Symbols: ATSMO1-2, ATSMO1, SMO1, SMO1-2 | SMO1-2 (STEROL C4-METHYL OXIDASE	
JCVI_16653	1.258	moderately similar to (264)AT4G17490| Symbols: ERF-6-6, ATERF6 | ATERF6 (ETHYLENE RESPONSIVE ELEMENT BINDING FAC	
CO750351	1.258	weakly similar to (154)AT1G27595| Symbols: | similar to ESP4 (ENHANCED SILENCING PHENOTYPE 4), binding [Arabidopsis thalia	
JCVI_19763	1.258	no original description	
JCVI_27654	1.258	moderately similar to (496)AT5G55930| Symbols: ATOPT1 | ATOPT1 (oligopeptide transporter 1); oligopeptide transporter | chr5:226702	
JCVI_10348	1.258	moderately similar to (313)AT1G71692| Symbols: XAL1, AGL12 | AGL12 (AGAMOUS-LIKE 12); transcription factor | chr1:26956565-2	
JCVI_10794	1.257	moderately similar to (322)AT3G17770| Symbols: | dihydroxyacetone kinase family protein | chr3:6081979-6085963 REVERSE no origin	
JCVI_24674	1.257	highly similar to (622)AT3G13300| Symbols: VCS | VCS (VARICOSE); nucleotide binding | chr3:4304092-4309956 FORWARD no origi	
JCVI_5211	1.257	weakly similar to (122)AT1G75580| Symbols: | auxin-responsive protein, putative | chr1:28381191-28381517 FORWARD no original des	
EV153355	1.257	moderately similar to (209)AT3G24490| Symbols: | transcription factor | chr3:8911029-8912030 FORWARD [21484] 63 997 997	
EV057842	1.257	moderately similar to (300)AT5G50170| Symbols: | C2 domain-containing protein / GRAM domain-containing protein | chr5:20438376-2(	
AJ270276	1.257	weakly similar to (112)AT1G67170| Symbols: | similar to unknown protein [Arabidopsis thaliana] (TAIR:AT3G14750.1); similar to unnar	
JCVI_24571	1.257	moderately similar to (334)AT1G52570| Symbols: PLDALPHA2 | PLDALPHA2 (PHOSPHLIPASE D ALPHA 2); phospholipase D | chr1	
AT000827	1.257	no similarity	1.907
EV148416	1.256	no similarity	

EV142101	1.256	no similarity	
JCVI_27668	1.256	highly similar to (519)AT3G10050| Symbols: OMR1 | OMR1 (L-O-METHYLTHREONINE RESISTANT 1); L-threonine ammonia-lyase	
JCVI_18527	1.256	highly similar to (826)AT1G69850| Symbols: NTL1, ATNRT1:2 | ATNRT1:2 (NITRATE TRANSPORTER 1:2); calcium ion binding / tra	
ES957641	1.256	very weakly similar to (83.6)AT5G10890| Symbols: | myosin heavy chain-related | chr5:3434031-3435068 REVERSE [21423]	-1.669
JCVI_7654	1.256	moderately similar to (228)AT2G18040| Symbols: PIN1AT | PIN1AT (parvulin 1At) | chr2:7849428-7850619 FORWARDweakly similar t	
CN830809	1.256	weakly similar to (166)AT1G64380| Symbols: | AP2 domain-containing transcription factor, putative | chr1:23894644-23895651 REVERS	
JCVI_36450	1.256	very weakly similar to (89.0)AT1G17220| Symbols: FUG1 | FUG1 (FU-GAERI1); translation initiation factor | chr1:5885376-5890158 FOI	
EE526729	1.255	moderately similar to (232)AT4G19003| Symbols: | similar to unnamed protein product [Vitis vinifera] (GB:CAO63315.1); contains Inter	
JCVI_16995	1.255	moderately similar to (340)AT1G73220| Symbols: ATOCT1 | ATOCT1 (ARABIDOPSIS THALIANA ORGANIC CATION/CARNITINE	2.964
CX187754	1.255	moderately similar to (228)AT4G14110| Symbols: CSN8, FUS7, EMB143, COP9 | COP9 (CONSTITUTIVE PHOTOMORPHOGENIC 9)	
CX193712	1.255	weakly similar to (167)AT4G28740| Symbols: | similar to LPA1 (LOW PSII ACCUMULATION1), binding [Arabidopsis thaliana] (TAIR	
CA991741	1.255	very weakly similar to (80.1)AT5G24580| Symbols: | copper-binding family protein | chr5:8410397-8412090 REVERSE [12405]	
DY028347	1.255	weakly similar to (154)AT4G37740| Symbols: AtGRF2 | AtGRF2 (GROWTHREGULATING FACTOR 2) | chr4:17725527-17727603 RE	
JCVI_21893	1.255	no original description	
EV051761	1.255	weakly similar to (101)AT5G41690| Symbols: | RNA binding | chr5:16687354-16691417 REVERSE [21442]	
EE484214	1.255	no similarity	
JCVI_6271	1.255	moderately similar to (239)AT4G17520| Symbols: | nuclear RNA-binding protein, putative | chr4:9771509-9773326 FORWARD no origir	
EX088122	1.255	moderately similar to (342)AT4G28050| Symbols: TET7 | TET7 (TETRASPANIN7) | chr4:13942572-13943627 REVERSE [21823]	
JCVI_17288	1.254	moderately similar to (245)AT4G27000| Symbols: ATRBP45C | ATRBP45C; RNA binding | chr4:13554989-13557769 REVERSE no orig	
JCVI_12601	1.254	very weakly similar to (99.0)AT5G14800| Symbols: AT-P5R, AT-P5C1, EMB2772, P5CR | P5CR (PYRROLINE-5- CARBOXYLATE (P5	
EV074217	1.254	moderately similar to (311)AT1G53900| Symbols: | GTP binding / translation initiation factor | chr1:20131302-20134502 FORWARD [21	
DW997539	1.254	no similarity	
JCVI_18718	1.254	moderately similar to (416)AT2G43080| Symbols: AT-P4H-1 | AT-P4H-1 (A. THALIANA P4H ISOFORM 1); oxidoreductase, acting on p	
JCVI_28528	1.254	moderately similar to (295)AT2G19640| Symbols: ASHR2 | ASHR2 (ASH1-RELATED PROTEIN 2) | chr2:8498483-8499679 FORWARI	
CD837644	1.253	moderately similar to (300)AT1G04120| Symbols: MRP5, ATMRP5 | ATMRP5 (Arabidopsis thaliana multidrug resistance-associated prot	
JCVI_41980	1.253	moderately similar to (333)AT2G46500| Symbols: | phosphatidylinositol 3- and 4-kinase family protein / ubiquitin family protein | chr2:19	
JCVI_14735	1.253	no original description	
JCVI_13892	1.253	moderately similar to (228)AT5G03730| Symbols: SIS1, CTR1 | CTR1 (CONSTITUTIVE TRIPLE RESPONSE 1); kinase/ protein serine/	
EE418226	1.253	weakly similar to (103)AT3G58180| Symbols: | PBS lyase HEAT-like repeat-containing protein | chr3:21555166-21556958 FORWARDve	
JCVI_18799	1.253	moderately similar to (384)AT1G54180| Symbols: ATBRXL3, BRX-LIKE3 | ATBRXL3/BRX-LIKE3 (BREVIS RADIX-LIKE 3) | chr1:2	
JCVI_36002	1.253	moderately similar to (238)AT3G13680| Symbols: | F-box family protein | chr3:4477541-4478728 REVERSE no original description	
JCVI_39258	1.253	moderately similar to (500)AT3G59520| Symbols: | rhomboid family protein | chr3:22003031-22003840 FORWARD no original descripti	
JCVI_33115	1.253	moderately similar to (401)AT2G22450| Symbols: | riboflavin biosynthesis protein, putative | chr2:9537734-9539771 FORWARD no origi	
EE530804	1.253	weakly similar to (115)AT1G72710| Symbols: CKL2 | CKL2; casein kinase I/ kinase | chr1:27376215-27379840 FORWARD [20175]	
JCVI_34640	1.253	weakly similar to (160)AT2G20320| Symbols: | DENN (AEX-3) domain-containing protein | chr2:8774187-8779217 FORWARD no origi	
JCVI_9394	1.253	highly similar to (518)AT3G19130| Symbols: ATRBP47B | ATRBP47B (RNA-BINDING PROTEIN 47B); RNA binding | chr3:6611404-(	
EV193090	1.253	no similarity	
JCVI_33023	1.253	no original description	
JCVI_42295	1.253	no original description	-0.948
JCVI_12380	1.253	weakly similar to (135)AT1G36380| Symbols: | electron carrier/ ubiquinol-cytochrome-c reductase | chr1:13702129-13702558 FORWARI	-1.866
JCVI_25052	1.252	weakly similar to (199)AT1G06360| Symbols: | fatty acid desaturase family protein | chr1:1939172-1940416 REVERSE no original descri	
EE568814	1.252	no similarity	
JCVI_12201	1.252	very weakly similar to (88.6)AT5G22950| Symbols: VPS24.1 | VPS24.1 | chr5:7681383-7682723 FORWARD no original description	
EE560406	1.252	no similarity	1.086
CN726928	1.252	very weakly similar to (81.3)AT5G55050| Symbols: | GDSL-motif lipase/hydrolase family protein | chr5:22354971-22356967 FORWARD	
ES907905	1.252	weakly similar to (105)AT1G15950| Symbols: IRX4, ATCCR1, CCR1 | CCR1 (CINNAMOYL COA REDUCTASE 1) | chr1:5478849-548	
JCVI_9242	1.252	moderately similar to (343)AT5G09590| Symbols: HSC70-5, mtHSC70-2 | mtHSC70-2 (HEAT SHOCK PROTEIN 70); ATP binding / unf	-1.531
EV126082	1.252	very weakly similar to (84.3)AT1G53310| Symbols: ATPPC1 | ATPPC1 (PHOSPHOENOLPYRUVATE CARBOXYLASE 1); phosphoeno	
EV022216	1.252	moderately similar to (249)AT1G36310| Symbols: | methyltransferase | chr1:13670856-13672146 FORWARD [21441]	1.531
JCVI_38870	1.251	very weakly similar to (80.9)AT1G09840| Symbols: | shaggy-related protein kinase kappa / ASK-kappa (ASK10) | chr1:3196116-3199526	
JCVI_1588	1.251	moderately similar to (406)AT4G27585| Symbols: | band 7 family protein | chr4:13766990-13769838 REVERSE no original description	
EX123229	1.251	moderately similar to (247)AT4G18630| Symbols: | similar to unknown protein [Arabidopsis thaliana] (TAIR:AT5G45850.1); similar to u	
JCVI_3543	1.251	moderately similar to (313)AT2G17380| Symbols: AP19 | AP19 (associated protein 19); ATP binding / protein binding / protein kinase | ch	
JCVI_12245	1.251	moderately similar to (469)AT2G18510| Symbols: EMB2444 | EMB2444 (EMBRYO DEFECTIVE 2444); RNA binding | chr2:8038636-8	
EX043453	1.251	very weakly similar to (97.4)AT2G26280| Symbols: CID7 | CID7; ATP binding / damaged DNA binding | chr2:11195221-11197657 REVE	
ES939899	1.251	no similarity	
JCVI_3519	1.251	weakly similar to (152)AT2G42610| Symbols: | similar to unknown protein [Arabidopsis thaliana] (TAIR:AT1G07090.1); similar to hypot	
JCVI_17413	1.250	weakly similar to (161)AT3G58840| Symbols: | similar to myosin heavy chain-related [Arabidopsis thaliana] (TAIR:AT1G06530.1); simil	
JCVI_13706	1.250	moderately similar to (359)AT1G69030| Symbols: | similar to BSD domain-containing protein [Arabidopsis thaliana] (TAIR:AT1G26300.	
CD816266	1.250	weakly similar to (187)AT2G46260| Symbols: | BTB/POZ domain-containing protein | chr2:19003184-19005536 FORWARD [13977]	
JCVI_3656	1.250	moderately similar to (264)AT3G62250| Symbols: UBQ5 | UBQ5 (UBIQUITIN 5); protein binding | chr3:23048113-23048586 FORWARI	
JCVI_23506	1.250	moderately similar to (379)AT5G58450| Symbols: | binding | chr5:23644052-23649297 FORWARD no original description	
EE450070	1.250	no similarity	
JCVI_31116	1.250	weakly similar to (188)AT5G67620| Symbols: | similar to unknown protein [Arabidopsis thaliana] (TAIR:AT5G62900.1); similar to unnar	
JCVI_38892	1.250	moderately similar to (202)AT2G17990| Symbols: | similar to kinectin-related [Arabidopsis thaliana] (TAIR:AT5G66250.3); similar to kir	
JCVI_5524	1.250	moderately similar to (365)AT3G15060| Symbols: AtRABA1g | AtRABA1g (Arabidopsis Rab GTPase homolog A1g); GTP binding | chr3	
ES969372	1.249	no similarity	
EV193460	1.249	weakly similar to (131)AT3G59380| Symbols: PLP, ATFTA, PFT/PGGT-IALPHA, FTA | FTA (FARNESYLTRANSFERASE A); protein	
ES927331	1.249	weakly similar to (196)AT1G72210| Symbols: | basic helix-loop-helix (bHLH) family protein (bHLH096) | chr1:27183728-27185930 FOR	
JCVI_26088	1.249	moderately similar to (354)AT5G47840| Symbols: AMK2 | AMK2 (ADENOSINE MONOPHOSPHATE KINASE); adenylate kinase | chr5	
JCVI_13946	1.249	weakly similar to (192)AT3G22530| Symbols: | similar to unknown protein [Arabidopsis thaliana] (TAIR:AT4G14830.1); similar to unnar	
EX023581	1.249	moderately similar to (395)AT5G24010| Symbols: | protein kinase family protein | chr5:8113913-8116387 FORWARD [21809] 15 745 74	
JCVI_10604	1.249	highly similar to (825)AT4G12910| Symbols: SCPL20 | SCPL20 (serine carboxypeptidase-like 20); serine carboxypeptidase | chr4:755061	
JCVI_26981	1.249	moderately similar to (291)AT5G10750| Symbols: | similar to unknown protein [Arabidopsis thaliana] (TAIR:AT5G24990.1); similar to h	
JCVI_15327	1.249	moderately similar to (233)AT5G02770| Symbols: | similar to unnamed protein product [Vitis vinifera] (GB:CAO18065.1) | chr5:628099-	
JCVI_24814	1.249	highly similar to (688)AT2G31270| Symbols: ATCDT1A, CDT1A, CDT1 | ATCDT1A/CDT1/CDT1A (ARABIDOPSIS HOMOLOG OF Y	
JCVI_3329	1.249	weakly similar to (200)AT5G64260| Symbols: | phosphate-responsive protein, putative | chr5:25721206-25722123 FORWARD no origina	
JCVI_10351	1.249	highly similar to (548)AT5G11560| Symbols: | catalytic | chr5:3709735-3713995 REVERSE no original description	-1.571
DY002543	1.249	weakly similar to (164)AT2G43940| Symbols: | thiol methyltransferase, putative | chr2:18203167-18204740 REVERSE [18968] 1 628 642	
ES916858	1.249	moderately similar to (293)AT1G28110| Symbols: SCPL45 | SCPL45; serine carboxypeptidase | chr1:9804140-9806819 REVERSEweakly	
EE528292	1.248	moderately similar to (215)AT2G46780| Symbols: | RNA recognition motif (RRM)-containing protein | chr2:19236586-19238017 FORWA	

JCVI_26606	1.248	moderately similar to (211)AT3G17600| Symbols: IAA31 | IAA31 (indoleacetic acid-induced protein 31); transcription factor | chr3:60202	
EV031500	1.248	very weakly similar to (99.0)AT1G73130| Symbols: | similar to unknown protein [Arabidopsis thaliana] (TAIR:AT1G17780.2); similar to I	
JCVI_27353	1.248	moderately similar to (264)AT3G46890| Symbols: | similar to MEE60 (maternal effect embryo arrest 60) [Arabidopsis thaliana] (TAIR:AT	
JCVI_36148	1.248	moderately similar to (255)AT5G51150| Symbols: | similar to unknown protein [Arabidopsis thaliana] (TAIR:AT1G34630.1); similar to u	
CX190718	1.248	weakly similar to (132)AT1G25370| Symbols: | similar to unknown protein [Arabidopsis thaliana] (TAIR:AT1G68340.1); similar to unnar	-1.513
JCVI_6300	1.248	no original description	
EX056312	1.248	weakly similar to (196)AT4G32770| Symbols: ATSDX1, VTE1 | VTE1 (VITAMIN E DEFICIENT 1) | chr4:15804986-15807795 FORWA	
JCVI_7574	1.247	moderately similar to (235)AT3G54540| Symbols: ATGCN4 | ATGCN4 (Arabidopsis thaliana general control non-repressible 4) | chr3:20	
EV206370	1.247	no similarity	
JCVI_39653	1.247	weakly similar to (177)AT5G01980| Symbols: | zinc finger (C3HC4-type RING finger) family protein | chr5:375539-377020 FORWARD	
JCVI_25090	1.247	very weakly similar to (86.7)AT3G57050| Symbols: CBL | CBL (CYSTATHIONINE BETA-LYASE) | chr3:21122918-21125500 REVERS	
JCVI_37704	1.247	weakly similar to (113)AT5G46690| Symbols: BHLH071 | BHLH071 (BETA HLH PROTEIN 71); DNA binding / transcription factor | chr	
JCVI_30362	1.247	weakly similar to (109)AT3G14200| Symbols: | DNAJ heat shock N-terminal domain-containing protein | chr3:4712888-4714368 REVER	
JCVI_38375	1.247	no original description	
EV072021	1.247	weakly similar to (140)AT5G03040| Symbols: IQD2 | IQD2 (IQ-domain 2); calmodulin binding | chr5:710378-712405 REVERSE [21443]	-1.364
JCVI_16501	1.247	highly similar to (580)AT5G14520| Symbols: | pescadillo-related | chr5:4680792-4684333 REVERSE no original description	
DY011178	1.247	moderately similar to (415)AT1G07970| Symbols: | similar to hypothetical protein [Vitis vinifera] (GB:CAN63581.1); similar to unnamed	
JCVI_9256	1.247	moderately similar to (249)AT1G68590| Symbols: | plastid-specific 30S ribosomal protein 3, putative / PSRP-3, putative | chr1:25761256-	
EX114657	1.247	weakly similar to (143)AT4G08500| Symbols: ATMEKK1, MAPKKK8, MEKK1 | MEKK1 (MYTOGEN ACTIVATED PROTEIN KINA	-1.148
JCVI_31133	1.246	no original description	
JCVI_38939	1.246	weakly similar to (141)AT3G60966| Symbols: | protein binding / zinc ion binding | chr3:22563693-22564112 FORWARD no original desc	
JCVI_7382	1.246	moderately similar to (313)AT3G55610| Symbols: P5CS2 | P5CS2 (DELTA 1-PYRROLINE-5-CARBOXYLATE SYNTHASE 2); catalyti	
JCVI_27442	1.246	moderately similar to (362)AT1G49650| Symbols: | cell death associated protein-related | chr1:18381031-18382155 REVERSEvery weakl	
JCVI_22218	1.246	weakly similar to (172)AT4G10040| Symbols: CYTC-2 | CYTC-2 (CYTOCHROME C-2); electron carrier | chr4:6277080-6278278 FORW	
EV134100	1.245	no similarity	
JCVI_35364	1.245	no original description	
JCVI_24223	1.245	weakly similar to (132)AT3G24130| Symbols: | pectinesterase family protein | chr3:8711670-8713368 REVERSE no original description	
JCVI_11926	1.245	highly similar to (666)AT2G26600| Symbols: | glycosyl hydrolase family 17 protein | chr2:11324292-11325293 FORWARDmoderately si	
EV192657	1.245	no similarity	
JCVI_8832	1.245	moderately similar to (435)AT4G39520| Symbols: | GTP-binding protein, putative | chr4:18371323-18373994 REVERSE no original desc	
JCVI_515	1.245	moderately similar to (348)AT4G01310| Symbols: | ribosomal protein L5 family protein | chr4:544166-545480 REVERSEmoderately simi	-1.442
EE407830	1.245	moderately similar to (249)AT1G09880| Symbols: | lyase | chr1:3208830-3211655 REVERSE [16821] 1 441 477	
JCVI_5926	1.245	moderately similar to (426)AT2G41290| Symbols: | strictosidine synthase family protein | chr2:17217677-17219982 REVERSEweakly sin	
JCVI_25505	1.245	no original description	
JCVI_20108	1.245	highly similar to (563)AT5G14080| Symbols: | pentatricopeptide (PPR) repeat-containing protein | chr5:4543267-4545258 REVERSEvery	
EX102400	1.245	moderately similar to (259)AT5G51140| Symbols: | pseudouridine synthase family protein | chr5:20801503-20804019 REVERSE [21826]	
EX055567	1.245	very weakly similar to (94.4)AT1G70150| Symbols: | zinc finger (MYND type) family protein | chr1:26420561-26422880 FORWARD [21	-1.258
JCVI_23623	1.244	highly similar to (675)AT3G60120| Symbols: | glycosyl hydrolase family 1 protein | chr3:22217322-22219927 FORWARDmoderately sim	
RC_CD813881	1.244	no similarity	
JCVI_40394	1.244	moderately similar to (321)AT3G16350| Symbols: | myb family transcription factor | chr3:5547834-5549403 FORWARD no original desc	
JCVI_17345	1.244	no original description	
EX103378	1.244	weakly similar to (137)AT3G08950| Symbols: | electron transport SCO1/SenC family protein | chr3:2727290-2729294 FORWARD [2182	
JCVI_1845	1.244	weakly similar to (121)AT4G24190| Symbols: SHD | SHD (SHEPHERD); ATP binding | chr4:12551912-12555861 REVERSEvery weakly	-1.880
JCVI_21096	1.244	moderately similar to (205)AT5G62000| Symbols: ARF1-BP, HSS, ARF2 | ARF2 (AUXIN RESPONSE FACTOR 2) | chr5:24928085-249	
JCVI_40311	1.244	moderately similar to (444)AT5G52560| Symbols: ATUSP | ATUSP (ARABIDOPSIS THALIANA UDP-SUGAR PYROPHOSPHORYLA	
ES948611	1.243	weakly similar to (159)AT1G18630| Symbols: GR-RBP6 | GR-RBP6 (glycine-rich RNA-binding protein 6); RNA binding | chr1:6415218-6	
JCVI_15157	1.243	highly similar to (996)AT5G62890| Symbols: | permease, putative | chr5:25260949-25263974 FORWARD no original description	
EE449415	1.243	no similarity	
CD824954	1.243	moderately similar to (209)AT3G09730| Symbols: | similar to unnamed protein product [Vitis vinifera] (GB:CAO18124.1) | chr3:2984023	
EV004707	1.243	weakly similar to (137)AT5G09390| Symbols: | CD2-binding protein-related | chr5:2913592-2915804 FORWARD [21427]	
EV040720	1.243	moderately similar to (379)AT4G20270| Symbols: BAM3 | BAM3 (big apical meristem 3); ATP binding / protein serine/threonine kinase |	
JCVI_26040	1.243	weakly similar to (142)AT4G09690| Symbols: | DC1 domain-containing protein | chr4:6124894-6126117 FORWARD no original descript	
JCVI_38114	1.243	moderately similar to (483)AT1G66130| Symbols: | oxidoreductase N-terminal domain-containing protein | chr1:24618703-24620405 FOI	
EV112756	1.243	weakly similar to (184)AT2G41660| Symbols: MIZ1 | MIZ1 (MIZU-KUSSEI 1) | chr2:17375023-17375916 FORWARD [21479] 45 881 8	
DY019003	1.243	weakly similar to (198)AT4G02100| Symbols: | DNAJ heat shock N-terminal domain-containing protein | chr4:930228-932049 FORWAR	
EV085910	1.242	weakly similar to (122)AT1G67570| Symbols: | similar to unknown protein [Arabidopsis thaliana] (TAIR:AT1G50630.1); similar to unnar	
ES930162	1.242	weakly similar to (122)AT4G15110| Symbols: CYP97B3 | CYP97B3 (cytochrome P450, family 97, subfamily B, polypeptide 3); oxygen b	
ES967567	1.242	no similarity	
EV080634	1.242	very weakly similar to (80.5)AT2G21660| Symbols: GR-RBP7, GRP7, CCR2, ATGRP7 | ATGRP7 (COLD, CIRCADIAN RHYTHM, ANI	
EV090632	1.242	weakly similar to (136)AT1G07310| Symbols: | C2 domain-containing protein | chr1:2247774-2248832 REVERSE [21444]	
EV212706	1.242	no similarity	-2.181
JCVI_9879	1.242	moderately similar to (244)AT3G53110| Symbols: LOS4 | LOS4 (Low expression of osmotically responsive genes 1); ATP-dependent heli	
DN960925	1.242	no similarity	
AM395631	1.242	weakly similar to (118)AT4G14365| Symbols: | zinc finger (C3HC4-type RING finger) family protein / ankyrin repeat family protein | chr	
EV060964	1.241	no similarity	
JCVI_7863	1.241	moderately similar to (352)AT5G17440| Symbols: | LUC7 N_terminus domain-containing protein | chr5:5749851-5753417 FORWARD no	
JCVI_37098	1.241	moderately similar to (364)AT1G67560| Symbols: | lipoxygenase family protein | chr1:25323589-25327780 FORWARDmoderately simila	
JCVI_8657	1.241	weakly similar to (166)AT4G00585| Symbols: | similar to hypothetical protein MtrDRAFT_AC148817g12v2 [Medicago truncatula] (GB:	
JCVI_28061	1.241	weakly similar to (199)AT1G11020| Symbols: | zinc finger (C3HC4-type RING finger) family protein | chr1:3676968-3678350 FORWAR	
JCVI_30926	1.241	no original description	
EV026663	1.240	moderately similar to (277)AT3G08910| Symbols: | DNAJ heat shock protein, putative | chr3:2710408-2711817 REVERSEvery weakly si	
EV199291	1.240	weakly similar to (187)AT1G70760| Symbols: | inorganic carbon transport protein-related | chr1:26690929-26691863 FORWARD [21490	
EE439854	1.240	moderately similar to (228)AT2G34357| Symbols: | binding | chr2:14506315-14512634 FORWARD [20167]	
DY000796	1.240	very weakly similar to (92.4)AT5G06160| Symbols: | splicing factor-related | chr5:1862624-1866299 REVERSE [18967] 1 257 279	-1.172
CV432227	1.240	moderately similar to (204)AT4G34670| Symbols: | 40S ribosomal protein S3A (RPS3aB) | chr4:16548729-16550227 FORWARDweakly	
EV102945	1.240	weakly similar to (112)AT2G29910| Symbols: | F-box family protein | chr2:12758900-12759907 REVERSE [21477]	
EX091835	1.240	moderately similar to (207)AT5G19960| Symbols: | RNA recognition motif (RRM)-containing protein | chr5:6744031-6746135 FORWAR	
JCVI_12508	1.240	weakly similar to (118)AT3G03920| Symbols: | Gar1 RNA-binding region family protein | chr3:1009130-1010386 REVERSE no original	-1.337
JCVI_12711	1.240	moderately similar to (254)AT1G11700| Symbols: | similar to unknown protein [Arabidopsis thaliana] (TAIR:AT1G61930.1); similar to u	
JCVI_79	1.240	no original description	
EX063380	1.240	no similarity	

EV209484	1.240	no similarity	
JCVI_16621	1.240	weakly similar to (103)AT2G16360| Symbols: | 40S ribosomal protein S25 (RPS25A) | chr2:7083795-7084448 REVERSE no original desc	
JCVI_9607	1.240	moderately similar to (469)AT2G26890| Symbols: KAM2, GRV2 | GRV2 (KATAMARI2); binding / heat shock protein binding | chr2:114	
JCVI_1592	1.240	moderately similar to (243)AT5G56030| Symbols: ERD8, HSP81-2 | HSP81-2 (EARLY-RESPONSIVE TO DEHYDRATION 8); ATP bin	
EE508763	1.240	moderately similar to (229)AT4G29890| Symbols: | choline monooxygenase, putative (CMO-like) | chr4:14608874-14610911 FORWARD	
EE563718	1.240	weakly similar to (106)AT2G27040| Symbols: OCP11, AGO4 | AGO4 (ARGONAUTE 4); nucleic acid binding | chr2:11543873-11548581	
DY022027	1.239	moderately similar to (432)AT3G49060| Symbols: | protein kinase family protein / U-box domain-containing protein | chr3:18198371-182	1.309
JCVI_1353	1.239	moderately similar to (437)AT1G54020| Symbols: | myrosinase-associated protein, putative | chr1:20165473-20166591 REVERSE no orig	
EX088015	1.239	no similarity	
CD843498	1.239	weakly similar to (140)AT5G42890| Symbols: | sterol carrier protein 2 (SCP-2) family protein | chr5:17211686-17213138 REVERSE [13	
JCVI_8355	1.239	highly similar to (915)AT5G53450| Symbols: ORG1 | ORG1 (OBP3-RESPONSIVE GENE 1); kinase | chr5:21706670-21709468 FORWA	
JCVI_14689	1.239	moderately similar to (329)AT3G11200| Symbols: | PHD finger family protein | chr3:3508393-3510201 REVERSE no original description	
JCVI_33728	1.239	no original description	
CD832695	1.239	weakly similar to (180)AT5G45920| Symbols: | carboxylesterase | chr5:18640049-18641904 FORWARD [13980]	
EE541107	1.239	weakly similar to (169)AT5G42980| Symbols: ATH3, ATTRXH3, TRXH3, ATTRX3 | ATTRX3 (thioredoxin H-type 3); thiol-disulfide ex	
JCVI_12962	1.239	moderately similar to (374)AT1G15130| Symbols: | hydroxyproline-rich glycoprotein family protein | chr1:5206212-5209843 REVERSE	
JCVI_13322	1.238	highly similar to (507)AT3G16480| Symbols: MPPALPHA | MPPALPHA (mitochondrial processing peptidase alpha subunit); metalloend	
EX135395	1.238	moderately similar to (280)AT5G64810| Symbols: ATWRKY51, WRKY51 | WRKY51 (WRKY DNA-binding protein 51); transcription fa	
RC_H75007	1.238	no similarity	
EE509837	1.238	no similarity	
JCVI_28616	1.238	no original description	
AM390987	1.238	moderately similar to (313)AT1G05060| Symbols: | similar to hypothetical protein [Vitis vinifera] (GB:CAN75913.1) | chr1:1451254-145	
EV040985	1.238	moderately similar to (347)AT5G18650| Symbols: | zinc finger (C3HC4-type RING finger) family protein | chr5:6218262-6220376 FORW	
RC_AM385895	1.238	no similarity	
JCVI_3876	1.238	weakly similar to (166)AT4G39900| Symbols: | similar to unnamed protein product [Vitis vinifera] (GB:CAO67041.1) | chr4:18508563-1	
EV040080	1.237	moderately similar to (375)AT2G17265| Symbols: HSK | HSK (HOMOSERINE KINASE); homoserine kinase | chr2:7515688-7516800 F	-1.674
EV054282	1.237	weakly similar to (101)AT2G45740| Symbols: PEX11D | PEX11D | chr2:18846939-18848176 FORWARD [21442]	
JCVI_13249	1.237	moderately similar to (392)AT3G60600| Symbols: VAP, (AT)VAP, VAP27, VAP27-1 | VAP27-1 (VAMP/SYNAPTOBREVIN-ASSOCIA	
EE563091	1.237	no similarity	
CD828157	1.237	weakly similar to (194)AT1G07370| Symbols: PCNA1 | PCNA1 (PROLIFERATING CELLULAR NUCLEAR ANTIGEN); DNA binding	1.200
RC_EV010782	1.237	no similarity	
DN961284	1.236	moderately similar to (271)AT2G43590| Symbols: | chitinase, putative | chr2:18088669-18089826 REVERSEmoderately similar to (263)	
JCVI_6480	1.236	no original description	
AM390432	1.236	very weakly similar to (80.1)AT3G63400| Symbols: | peptidyl-prolyl cis-trans isomerase cyclophilin-type family protein | chr3:23423424-2	
ES933064	1.236	moderately similar to (282)AT1G21730| Symbols: | kinesin-related protein (MKRP1) | chr1:7630354-7636236 FORWARDvery weakly si	
JCVI_33285	1.236	weakly similar to (157)AT3G56240| Symbols: CCH | CCH (COPPER CHAPERONE) | chr3:20874439-20875381 REVERSE no original d	
EX062957	1.236	weakly similar to (111)AT1G17040| Symbols: | transcription factor-related | chr1:5825102-5828049 FORWARD [21814]	
EV066208	1.236	very weakly similar to (87.4)7SB1_SOYBN [21443] 1 663 676	
JCVI_22943	1.236	no original description	
JCVI_21766	1.235	moderately similar to (241)AT5G64420| Symbols: | DNA polymerase V family | chr5:25773642-25778348 FORWARD no original descri	
RC_JCVI_42129	1.235	no original description	
JCVI_20359	1.235	moderately similar to (394)AT1G05490| Symbols: CHR31 | CHR31 (chromatin remodeling 31); ATP binding / DNA binding / helicase/ nu	
EV176680	1.235	moderately similar to (231)AT1G61380| Symbols: | S-locus protein kinase, putative | chr1:22649942-22653066 REVERSE [21487] 129 1	
EL588260	1.235	moderately similar to (297)AT2G12400| Symbols: | similar to unknown protein [Arabidopsis thaliana] (TAIR:AT2G25270.1); similar to h	
JCVI_35704	1.235	very weakly similar to (94.4)AT3G48180| Symbols: | similar to unknown protein [Arabidopsis thaliana] (TAIR:AT1G19020.1); similar to	
JCVI_759	1.235	moderately similar to (469)AT2G27200| Symbols: | GTP-binding family protein | chr2:11632527-11635037 FORWARD no original descr	
AM394536	1.235	no similarity	
JCVI_17217	1.234	highly similar to (772)AT1G45160| Symbols: | similar to protein kinase, putative [Arabidopsis thaliana] (TAIR:AT3G17850.1); similar to	
EV062128	1.234	moderately similar to (265)AT1G27410| Symbols: | DNA cross-link repair protein-related | chr1:9517213-9518730 FORWARD [21443]	
EL586777	1.234	no similarity	
EX083458	1.234	weakly similar to (124)AT1G76890| Symbols: AT-GT2, GT2 | GT2; transcription factor | chr1:28878105-28880097 REVERSE [21819] 1	
JCVI_37244	1.234	no original description	
EE471622	1.234	weakly similar to (120)AT5G13750| Symbols: ZIFL1 | ZIFL1 (ZINC INDUCED FACILITATOR-LIKE 1); tetracycline:hydrogen antiporte	
EE563784	1.234	no similarity	
JCVI_13045	1.234	weakly similar to (116)AT3G29035| Symbols: ANAC059, ATNAC3 | ANAC059/ATNAC3 (Arabidopsis NAC domain containing protein	-1.647
JCVI_35290	1.234	moderately similar to (201)AT4G05410| Symbols: | transducin family protein / WD-40 repeat family protein | chr4:2743226-2745518 RE	
JCVI_34716	1.234	moderately similar to (407)AT1G78690| Symbols: | phospholipid/glycerol acyltransferase family protein | chr1:29601895-29603302 FOR	-1.761
EV012808	1.234	no similarity	
CX195603	1.233	very weakly similar to (91.7)AT3G20270| Symbols: | lipid-binding serum glycoprotein family protein | chr3:7068885-7070872 FORWARD	
JCVI_23622	1.233	very weakly similar to (91.3)AT5G02520| Symbols: | unknown protein | chr5:562791-563838 FORWARD no original description	
EV226333	1.233	no similarity	
EV124681	1.233	moderately similar to (349)AT5G10720| Symbols: CKI2, AHK5 | AHK5 (CYTOKININ INDEPENDENT 2) | chr5:3386836-3390542 FOR	
EV107326	1.233	no similarity	
AM390381	1.233	moderately similar to (204)AT4G38440| Symbols: | similar to hypothetical protein [Vitis vinifera] (GB:CAN83259.1); similar to hypothet	
JCVI_17435	1.233	moderately similar to (315)AT5G66510| Symbols: GAMMA CA3 | GAMMA CA3 (GAMMA CARBONIC ANHYDRASE 3) | chr5:26567	
JCVI_35736	1.233	moderately similar to (350)AT5G02590| Symbols: | chloroplast lumen common family protein | chr5:583115-584095 FORWARD no origi	-1.604
JCVI_16206	1.233	highly similar to (553)AT1G21120| Symbols: | O-methyltransferase, putative | chr1:7395320-7396629 REVERSEmoderately similar to (2	
EV022454	1.233	no similarity	
EV042322	1.232	very weakly similar to (82.4)AT2G39100| Symbols: | zinc finger (C3HC4-type RING finger) family protein | chr2:16324540-16325947 FO	
JCVI_11127	1.232	moderately similar to (304)AT5G22020| Symbols: | strictosidine synthase family protein | chr5:7287881-7289360 REVERSE no original	
JCVI_41862	1.232	moderately similar to (492)AT3G09410| Symbols: | pectinacetylesterase family protein | chr3:2895015-2897372 REVERSE no original de	
JCVI_23441	1.232	moderately similar to (265)AT2G37330| Symbols: ALS3 | ALS3 (ALUMINUM SENSITIVE 3) | chr2:15676269-15677251 FORWARD no	
ES906722	1.232	moderately similar to (294)AT4G35880| Symbols: | aspartyl protease family protein | chr4:16993342-16995724 FORWARD [21429]	-2.447
ES944737	1.232	weakly similar to (172)AT1G33290| Symbols: | sporulation protein-related | chr1:12074105-12075969 FORWARD [21392]	
EV171919	1.232	moderately similar to (407)AT1G59890| Symbols: | paired amphipathic helix repeat-containing protein | chr1:22047991-22054335 FORW	
EX136729	1.232	weakly similar to (116)AT5G18630| Symbols: | lipase class 3 family protein | chr5:6202967-6205450 FORWARD [21833] 25 659 659	
JCVI_9327	1.232	weakly similar to (181)AT1G03050| Symbols: | epsin N-terminal homology (ENTH) domain-containing protein / clathrin assembly protei	
EV194204	1.232	moderately similar to (333)AT3G29400| Symbols: ATEXO70E1 | ATEXO70E1 (exocyst subunit EXO70 family protein E1); protein bindi	
JCVI_3759	1.232	moderately similar to (287)AT4G14220| Symbols: | zinc finger (C3HC4-type RING finger) family protein | chr4:8196007-8198235 FORW	
CX191289	1.232	no similarity	
CV545693	1.232	weakly similar to (105)AT4G00570| Symbols: | malate oxidoreductase, putative | chr4:242817-246522 REVERSEvery weakly similar to (	

JCVI_31726	1.232	moderately similar to (345)AT1G69360| Symbols: | similar to unknown protein [Arabidopsis thaliana] (TAIR:AT1G26620.1); similar to h	
JCVI_13960	1.232	moderately similar to (420)AT3G23530| Symbols: | cyclopropane fatty acid synthase, putative / CPA-FA synthase, putative | chr3:843747	
EE518139	1.231	weakly similar to (195)AT2G20800| Symbols: NDB4 | NDB4 (NAD(P)H DEHYDROGENASE B4); NADH dehydrogenase | chr2:896033	
DY016861	1.231	moderately similar to (356)AT5G24330| Symbols: ATXR6 | ATXR6 (Arabidopsis thaliana Trithorax- related protein 6); DNA binding | ch	
JCVI_9736	1.231	moderately similar to (202)AT1G24320| Symbols: | alpha-glucosidase, putative | chr1:8626426-8630924 REVERSE no original descriptio	
JCVI_27548	1.231	very weakly similar to (94.7)AT1G67325| Symbols: | binding | chr1:25213488-25216075 REVERSE no original description	
JCVI_9789	1.231	weakly similar to (194)AT1G56420| Symbols: | similar to unknown protein [Arabidopsis thaliana] (TAIR:AT1G12530.1); similar to unnar	
JCVI_14758	1.231	moderately similar to (312)AT2G23390| Symbols: | similar to hypothetical protein OsI_015489 [Oryza sativa (indica cultivar-group)] (GB	
EV149536	1.231	very weakly similar to (87.4)AT2G31980| Symbols: | cysteine proteinase inhibitor-related | chr2:13616323-13616847 REVERSE [21483]	
EH423345	1.231	weakly similar to (177)AT3G54670| Symbols: SMC1, ATSMC1, TTN8 | TTN8 (TITAN8); ATP binding | chr3:20251406-20254679 FOR	
JCVI_11842	1.230	moderately similar to (215)AT5G20280| Symbols: ATSPS1F | ATSPS1F (SUCROSE PHOSPHATE SYNTHASE 1F); sucrose-phosphate s	
DY006601	1.230	moderately similar to (241)AT1G32360| Symbols: | zinc finger (CCCH-type) family protein | chr1:11673305-11675142 FORWARD [189	
JCVI_17974	1.230	no original description	
JCVI_39064	1.230	highly similar to (526)AT2G18100| Symbols: | similar to unknown protein [Arabidopsis thaliana] (TAIR:AT4G36210.1); similar to unkno	
EV131514	1.230	no similarity	
JCVI_33169	1.230	moderately similar to (485)AT3G02590| Symbols: | delta 7-sterol-C5-desaturase, putative | chr3:549347-550560 FORWARDmoderately s	
JCVI_8064	1.230	moderately similar to (251)AT4G10170| Symbols: | synaptobrevin-related family protein | chr4:6344583-6345347 FORWARD no original	
JCVI_2855	1.230	weakly similar to (179)AT1G13830| Symbols: | beta-1,3-glucanase-related | chr1:4739996-4740923 REVERSE no original description	
ES904449	1.230	moderately similar to (374)AT3G24190| Symbols: | ABC1 family protein | chr3:8743326-8747710 FORWARD [21432]	
EH429807	1.230	moderately similar to (251)AT1G71400| Symbols: | disease resistance family protein / LRR family protein | chr1:26913567-26916110 FO	
EE453894	1.230	moderately similar to (355)AT4G32040| Symbols: KNAT5 | KNAT5 (KNOTTED1-LIKE HOMEOBOX GENE 5); transcription factor | c	
JCVI_30948	1.229	no original description	-1.378
JCVI_26859	1.229	moderately similar to (426)AT1G48310| Symbols: | SNF2 domain-containing protein / helicase domain-containing protein | chr1:1785228	
AT000655	1.229	no similarity	
JCVI_6834	1.229	moderately similar to (404)AT2G01690| Symbols: | binding | chr2:309143-313498 REVERSE no original description	1.148
JCVI_12932	1.229	moderately similar to (309)AT4G36610| Symbols: | hydrolase, alpha/beta fold family protein | chr4:17265551-17267280 REVERSE no or	
JCVI_38278	1.229	moderately similar to (217)AT1G23950| Symbols: | similar to unknown protein [Arabidopsis thaliana] (TAIR:AT1G23970.2); contains In	
JCVI_30642	1.229	moderately similar to (232)AT1G43722| Symbols: | similar to unknown protein [Arabidopsis thaliana] (TAIR:AT5G28730.1); similar to h	
JCVI_23247	1.228	no original description	
JCVI_16682	1.228	moderately similar to (257)AT4G39520| Symbols: | GTP-binding protein, putative | chr4:18371323-18373994 REVERSE no original desc	
DY019556	1.228	moderately similar to (227)AT1G31230| Symbols: AK-HSDH I, AK-HSDH | AK-HSDH/AK-HSDH I (ASPARTATE KINASE-HOMOSE	
JCVI_25625	1.228	no original description	
EV178108	1.228	moderately similar to (243)AT1G22870| Symbols: | protein kinase family protein | chr1:8089490-8094162 FORWARD [21487] 99 923 92	
EV108040	1.228	no similarity	
EX021662	1.228	moderately similar to (317)AT2G35050| Symbols: | protein kinase family protein | chr2:14776787-14781875 FORWARD [21809]	
EE409212	1.228	weakly similar to (185)AT2G15560| Symbols: | similar to unknown protein [Arabidopsis thaliana] (TAIR:AT3G62200.1); similar to EDA	
JCVI_19557	1.228	moderately similar to (296)AT4G38620| Symbols: ATMYB4, MYB4 | MYB4 (myb domain protein 4); transcription factor | chr4:1805386	
ES992054	1.228	no similarity	
JCVI_26012	1.227	moderately similar to (375)AT4G11120| Symbols: | translation elongation factor Ts (EF-Ts), putative | chr4:6778062-6779930 FORWAR	
JCVI_952	1.227	moderately similar to (242)AT4G10480| Symbols: | nascent polypeptide associated complex alpha chain protein, putative / alpha-NAC, pu	
JCVI_38379	1.227	no original description	
JCVI_3473	1.227	moderately similar to (413)AT5G18110| Symbols: NCBP | NCBP (NOVEL CAP-BINDING PROTEIN); RNA binding / translation initiati	
EE548701	1.227	no similarity	
EV170725	1.227	no similarity	
JCVI_27419	1.227	moderately similar to (356)AT5G39080| Symbols: | transferase family protein | chr5:15658909-15660300 FORWARD no original descrip	
EX061796	1.227	moderately similar to (340)AT5G18830| Symbols: SPL7 | SPL7 (SQUAMOSA PROMOTER BINDING PROTEIN-LIKE 7); DNA binding	
JCVI_31577	1.227	moderately similar to (449)AT1G55270| Symbols: | kelch repeat-containing F-box family protein | chr1:20622000-20623592 REVERSE n	
JCVI_36085	1.227	moderately similar to (317)AT2G35650| Symbols: CSLA07, ATCSLA7, CSLA7, ATCSLA07 | ATCSLA07 (CELLULOSE SYNTHASE L	
JCVI_2095	1.227	weakly similar to (174)AT2G37270| Symbols: ATRPS5B | ATRPS5B (RIBOSOMAL PROTEIN 5B); structural constituent of ribosome |	
JCVI_4413	1.227	weakly similar to (140)AT2G38230| Symbols: ATPDX1.1 | ATPDX1.1 (PYRIDOXINE BIOSYNTHESIS 1.1); protein heterodimerization	
EV202565	1.227	moderately similar to (427)AT3G14920| Symbols: | similar to unknown protein [Arabidopsis thaliana] (TAIR:AT5G05480.1); similar to P	
JCVI_33079	1.226	weakly similar to (166)AT1G48210| Symbols: | serine/threonine protein kinase, putative | chr1:17803220-17805467 FORWARD no origin	1.337
EV153083	1.226	moderately similar to (384)AT3G57220| Symbols: | UDP-GlcNAc:dolichol phosphate N-acetylglucosamine-1-phosphate transferase, puta	
ES907464	1.226	moderately similar to (351)AT1G54710| Symbols: AtATG18h | AtATG18h (Arabidopsis thaliana homolog of yeast autophagy 18 (ATG18	
JCVI_4955	1.226	highly similar to (608)AT4G11310| Symbols: | cysteine proteinase, putative | chr4:6883590-6885314 FORWARDmoderately similar to (3	
JCVI_25250	1.226	moderately similar to (381)AT3G17840| Symbols: RLK902 | RLK902 (receptor-like kinase 902); ATP binding / kinase/ protein serine/thre	
JCVI_12269	1.226	weakly similar to (174)AT3G05640| Symbols: | protein phosphatase 2C, putative / PP2C, putative | chr3:1640616-1642233 REVERSE no	
JCVI_24610	1.226	weakly similar to (182)AT1G16710| Symbols: HAC12 | HAC12 (HISTONE ACETYLTRANSFERASE OF THE CBP FAMILY 12); H3/H	
JCVI_27869	1.226	moderately similar to (281)AT1G04680| Symbols: | pectate lyase family protein | chr1:1304051-1307779 REVERSEmoderately similar to	
ES904092	1.226	moderately similar to (414)AT4G03390| Symbols: SRF3 | SRF3 (STRUBBELIG-RECEPTOR FAMILY 3); ATP binding / kinase/ protein	
EV030726	1.226	no similarity	
JCVI_15671	1.226	moderately similar to (312)AT1G73100| Symbols: SUVH3 | SUVH3 (SU(VAR)3-9 HOMOLOG 3) | chr1:27495631-27497640 FORWAR	
JCVI_27253	1.226	moderately similar to (296)AT3G49800| Symbols: | BSD domain-containing protein | chr3:18482942-18484661 REVERSE no original de	
JCVI_2272	1.226	moderately similar to (310)AT1G44920| Symbols: | similar to unnamed protein product [Vitis vinifera] (GB:CAO61509.1) | chr1:1698566	
JCVI_14372	1.226	weakly similar to (151)AT2G03505| Symbols: | glycosyl hydrolase family protein 17 | chr2:1064314-1065091 FORWARD no original des	1.456
JCVI_26556	1.226	moderately similar to (317)AT4G27690| Symbols: | vacuolar protein sorting-associated protein 26, putative / VPS26, putative | chr4:1382	
JCVI_18481	1.225	moderately similar to (209)AT1G31350| Symbols: | F-box family protein | chr1:11221500-11222687 REVERSE no original description	
EX122323	1.225	moderately similar to (246)AT4G29040| Symbols: RPT2A | RPT2A (REGULATORY PARTICLE AAA-ATPASE 2A); ATPase | chr4:143	
JCVI_31223	1.225	no original description	-1.426
EE565181	1.225	weakly similar to (135)AT5G16630| Symbols: | DNA repair protein Rad4 family | chr5:5455328-5459464 FORWARD [20153] 40 720 72	
JCVI_14018	1.225	moderately similar to (279)AT4G32680| Symbols: | similar to unnamed protein product [Vitis vinifera] (GB:CAO44139.1) | chr4:1576346	
ES967436	1.225	no similarity	
JCVI_37554	1.225	moderately similar to (280)AT5G48720| Symbols: | similar to unknown protein [Arabidopsis thaliana] (TAIR:AT2G01990.1); similar to u	
EX060341	1.225	no similarity	
JCVI_5745	1.225	moderately similar to (395)AT4G12070| Symbols: | protein binding | chr4:7231935-7234266 FORWARD no original description	
EX090673	1.225	moderately similar to (275)AT3G17440| Symbols: ATNPSN13, NPSN13 | NPSN13 (novel plant SNARE 13) | chr3:5970159-5972296 RE	
JCVI_22726	1.225	moderately similar to (357)AT3G55640| Symbols: | mitochondrial substrate carrier family protein | chr3:20651027-20653390 FORWARD	
JCVI_34324	1.225	highly similar to (530)AT3G53180| Symbols: | glutamate-ammonia ligase | chr3:19718046-19722166 FORWARD no original description	
EV011579	1.224	no similarity	
JCVI_36456	1.224	moderately similar to (420)AT2G02240| Symbols: MEE66 | MEE66 (maternal effect embryo arrest 66) | chr2:592358-593601 REVERSE	
EX063063	1.224	no similarity	

EE535335	1.224	no similarity	
EV122966	1.223	no similarity	3.030
JCVI_5534	1.223	highly similar to (563)AT3G57020| Symbols: | strictosidine synthase family protein | chr3:21109494-21111282 REVERSEweakly similar	
EX035993	1.223	moderately similar to (365)AT1G23800| Symbols: ALDH2B, ALDH2B7 | ALDH2B7 (Aldehyde dehydrogenase 2B7); 3-chloroallyl aldeh	
EE461889	1.223	no similarity	
EV130725	1.223	no similarity	
EX089990	1.223	moderately similar to (327)AT5G40880| Symbols: | WD-40 repeat family protein / zfwd3 protein (ZFWD3) | chr5:16396709-16398433 F	
CD824834	1.223	moderately similar to (212)AT3G01100| Symbols: ATHYP1, HYP1 | HYP1 (HYPOTHETICAL PROTEIN 1) | chr3:35817-38183 REVER	
EV093392	1.223	no similarity	
RC_EX064916	1.222	no similarity	
JCVI_4857	1.222	highly similar to (514)AT3G25430| Symbols: | CAF1 family ribonuclease | chr3:9222441-9224937 FORWARD no original description	
JCVI_26865	1.222	moderately similar to (396)AT5G15400| Symbols: | U-box domain-containing protein | chr5:4997767-5002910 REVERSE no original des	
ES947539	1.222	no similarity	
JCVI_2249	1.222	moderately similar to (446)AT2G26250| Symbols: FDH | FDH (FIDDLEHEAD); acyltransferase | chr2:11177877-11180137 REVERSE no	
JCVI_21894	1.222	no original description	
JCVI_34411	1.222	weakly similar to (136)AT4G00270| Symbols: | DNA-binding storekeeper protein-related | chr4:117477-118468 REVERSE no original de	-1.235
JCVI_4904	1.222	weakly similar to (120)AT5G11170| Symbols: | DEAD/DEAH box helicase, putative (RH15) | chr5:3553335-3556647 FORWARD no orig	-1.614
EX138590	1.222	weakly similar to (103)AT1G21280| Symbols: | similar to hypothetical protein [Vitis vinifera] (GB:CAN81001.1); contains InterPro doma	
JCVI_41822	1.221	no original description	
JCVI_25723	1.221	highly similar to (561)AT4G22010| Symbols: SKS4 | SKS4 (SKU5 Similar 4); copper ion binding / oxidoreductase | chr4:11663441-11666	
ES950574	1.221	no similarity	
EX114214	1.221	moderately similar to (368)AT5G51140| Symbols: | pseudouridine synthase family protein | chr5:20801503-20804019 REVERSE [21827]	
EE512167	1.221	very weakly similar to (88.2)AT5G07950| Symbols: | similar to unnamed protein product [Vitis vinifera] (GB:CAO64456.1) | chr5:254053	
JCVI_4588	1.221	moderately similar to (403)AT1G55360| Symbols: | similar to unknown protein [Arabidopsis thaliana] (TAIR:AT5G56530.1); similar to u	-1.927
JCVI_548	1.220	moderately similar to (466)AT5G42790| Symbols: PAF1 | PAF1 (proteasome alpha subunit F1); peptidase | chr5:17176498-17178203 REV	
JCVI_40495	1.220	moderately similar to (466)AT5G20350| Symbols: TIP1 | TIP1 (TIP GROWTH DEFECTIVE 1) | chr5:6876774-6881104 FORWARD no	
EV067622	1.220	no similarity	1.415
JCVI_27362	1.220	moderately similar to (249)AT5G19350| Symbols: | RNA-binding protein 45 (RBP45), putative | chr5:6518980-6521297 FORWARD no c	
JCVI_876	1.220	moderately similar to (385)AT1G29670| Symbols: | GDSL-motif lipase/hydrolase family protein | chr1:10375829-10377703 FORWARDw	
JCVI_2323	1.220	no original description	
EV060142	1.220	no similarity	
EV159611	1.220	moderately similar to (257)AT1G23330| Symbols: | similar to unknown protein [Arabidopsis thaliana] (TAIR:AT1G10740.1); similar to C	
JCVI_26667	1.220	weakly similar to (177)AT2G40790| Symbols: | thioredoxin family protein | chr2:17030187-17030993 REVERSEvery weakly similar to (8	
JCVI_9707	1.220	highly similar to (550)AT1G18080| Symbols: ATARCA | ATARCA (Arabidopsis thaliana Homolog of the Tobacco ArcA); nucleotide bind	
DY029608	1.220	moderately similar to (245)AT5G39450| Symbols: | F-box family protein | chr5:15803273-15805105 FORWARD [18978]	
JCVI_13292	1.219	moderately similar to (345)AT4G26400| Symbols: | zinc finger (C3HC4-type RING finger) family protein | chr4:13344962-13346032 REV	
JCVI_1631	1.219	moderately similar to (370)AT3G52990| Symbols: | pyruvate kinase, putative | chr3:19660314-19663215 FORWARDweakly similar to (1	
JCVI_34066	1.219	no original description	
RC_ES967813	1.219	no similarity	
AT000606	1.219	very weakly similar to (90.1)AT3G61790| Symbols: | seven in absentia (SINA) family protein | chr3:22882949-22884518 REVERSE [1392	
JCVI_38683	1.219	moderately similar to (338)AT4G12570| Symbols: UPL5 | UPL5 (UBIQUITIN PROTEIN LIGASE 5); ubiquitin-protein ligase | chr4:7445	
JCVI_29487	1.219	moderately similar to (394)AT3G62940| Symbols: | OTU-like cysteine protease family protein | chr3:23274081-23275220 REVERSE no c	
JCVI_5850	1.219	moderately similar to (257)AT1G07450| Symbols: | tropinone reductase, putative / tropine dehydrogenase, putative | chr1:2288035-22892	
EV061312	1.218	no similarity	
EX038101	1.218	no similarity	
JCVI_24932	1.218	moderately similar to (468)AT2G23760| Symbols: SAW2, BLH4 | BLH4 (SAWTOOTH 2); DNA binding | chr2:10115031-10119816 REV	
EV216839	1.217	no similarity	
JCVI_3608	1.217	weakly similar to (189)AT5G60340| Symbols: | maoC-like dehydratase domain-containing protein | chr5:24291105-24293117 REVERSE	
EE555326	1.217	weakly similar to (173)AT3G61410| Symbols: | similar to protein kinase family protein / U-box domain-containing protein [Arabidopsis th	1.631
EV116790	1.217	moderately similar to (304)AT5G13320| Symbols: GDG1, WIN3, PBS3 | PBS3 (AVRPPHB SUSCEPTIBLE 3) | chr5:4268905-4270899 F	
JCVI_797	1.217	moderately similar to (401)AT2G24790| Symbols: COL3 | COL3 (CONSTANS-LIKE 3); protein binding / transcription factor/ zinc ion bi	
JCVI_32704	1.217	moderately similar to (201)AT3G15190| Symbols: | chloroplast 30S ribosomal protein S20, putative | chr3:5116223-5117419 FORWARD	
AM395429	1.217	weakly similar to (176)AT1G27752| Symbols: | similar to unnamed protein product [Vitis vinifera] (GB:CAO61467.1); contains InterPro	
JCVI_32909	1.217	moderately similar to (302)AT2G45330| Symbols: EMB1067 | EMB1067 (EMBRYO DEFECTIVE 1067); tRNA 2'-phosphotransferase | c	
RC_ES966207	1.217	no similarity	1.740
EE558721	1.217	no similarity	
JCVI_21524	1.216	moderately similar to (363)AT4G00440| Symbols: | similar to unknown protein [Arabidopsis thaliana] (TAIR:AT2G45900.1); similar to u	
CD833454	1.216	no similarity	
EE431301	1.216	very weakly similar to (85.1)AT1G80555| Symbols: | 3-isopropylmalate dehydrogenase/ oxidoreductase, acting on the CH-OH group of do	
EX086423	1.216	weakly similar to (117)AT5G43750| Symbols: | similar to unnamed protein product [Vitis vinifera] (GB:CAO71280.1) | chr5:17597327-17	
AM387581	1.216	no similarity	
JCVI_9138	1.216	very weakly similar to (99.0)AT1G49790| Symbols: | F-box family protein-related | chr1:18439805-18440736 REVERSE no original descr	
EE459432	1.216	moderately similar to (219)AT3G26430| Symbols: | GDSL-motif lipase/hydrolase family protein | chr3:9675656-9677126 FORWARDwea	
RC_EE566795	1.216	no similarity	
ES993867	1.216	very weakly similar to (84.3)AT5G50310| Symbols: | kelch repeat-containing protein | chr5:20491788-20496507 FORWARD [21427]	
AM392044	1.216	moderately similar to (408)AT5G47040| Symbols: | Lon protease homolog 1, mitochondrial (LON) | chr5:19110583-19115905 REVERSE	-1.789
JCVI_8907	1.216	moderately similar to (236)AT3G09630| Symbols: | 60S ribosomal protein L4/L1 (RPL4A) | chr3:2953818-2955449 FORWARD no origin	
EV071876	1.216	moderately similar to (292)AT5G29000| Symbols: | myb family transcription factor | chr5:11023017-11024233 REVERSE [21443]	
EX091409	1.215	weakly similar to (115)AT4G24590| Symbols: | similar to unknown protein [Arabidopsis thaliana] (TAIR:AT5G49710.3); similar to unna	
JCVI_6578	1.215	very weakly similar to (99.4)AT2G31290| Symbols: | similar to unknown protein [Arabidopsis thaliana] (TAIR:AT3G63090.1); similar to l	
JCVI_798	1.215	moderately similar to (297)AT3G44840| Symbols: | S-adenosyl-L-methionine:carboxyl methyltransferase family protein | chr3:16383484-	
JCVI_21617	1.215	highly similar to (713)AT5G07920| Symbols: ATDGK1 | DGK1 (DIACYLGLYCEROL KINASE 1, DIACYLGLYCEROL KINASE1); di	
JCVI_23318	1.215	no original description	
EX093764	1.215	weakly similar to (103)AT5G18790| Symbols: | ribosomal protein L33 family protein | chr5:6266326-6266502 REVERSE [21823] 1 592 6	
JCVI_8515	1.215	highly similar to (776)AT1G59870| Symbols: PEN3, PDR8 | PDR8/PEN3 (PLEIOTROPIC DRUG RESISTANCE8); ATPase, coupled to	
EX094640	1.215	moderately similar to (320)AT2G43400| Symbols: ETFQO | ETFQO (ELECTRON-TRANSFER FLAVOPROTEIN:UBIQUINONE OXID	
EV026034	1.215	moderately similar to (369)AT5G07300| Symbols: BON2 | BON2 (BONZAI 2) | chr5:2299997-2303041 FORWARD [21441] 15 776 776	
JCVI_37569	1.214	no original description	
JCVI_14888	1.214	moderately similar to (406)AT4G02350| Symbols: | exocyst complex subunit Sec15-like family protein | chr4:1038157-1040571 FORWA	
CD822722	1.214	moderately similar to (221)AT5G57060| Symbols: | similar to unknown protein [Arabidopsis thaliana] (TAIR:AT4G26060.1); similar to u	
JCVI_4204	1.214	moderately similar to (439)AT3G03480| Symbols: CHAT | CHAT (ACETYL COA:(Z)-3-HEXEN-1-OL ACETYLTRANSFERASE); acet	

JCVI_15664	1.214	moderately similar to (275)AT1G47240| Symbols: ATNRAMP2, NRAMP2 | NRAMP2 (NRAMP metal ion transporter 2); metal ion trans	
JCVI_28230	1.214	weakly similar to (134)AT2G47440| Symbols: | DNAJ heat shock N-terminal domain-containing protein | chr2:19476982-19478730 FOR	
JCVI_9761	1.214	moderately similar to (204)AT5G14910| Symbols: | heavy-metal-associated domain-containing protein | chr5:4823818-4825199 FORWAI	
JCVI_32163	1.214	weakly similar to (157)AT1G22990| Symbols: | heavy-metal-associated domain-containing protein / copper chaperone (CCH)-related | chr	
JCVI_29953	1.214	moderately similar to (293)AT3G09770| Symbols: | zinc finger (C3HC4-type RING finger) family protein | chr3:2996634-2997840 REVE	
DY026256	1.214	no similarity	
JCVI_34420	1.214	moderately similar to (218)AT4G23190| Symbols: AT-RLK3, CRK11 | CRK11 (CYSTEINE-RICH RLK11); kinase | chr4:12141208-1214	
JCVI_9096	1.214	moderately similar to (311)AT3G01980| Symbols: | short-chain dehydrogenase/reductase (SDR) family protein | chr3:327754-328840 RE	
JCVI_31692	1.214	weakly similar to (181)AT5G07940| Symbols: | similar to dentin sialophosphoprotein-related [Arabidopsis thaliana] (TAIR:AT5G07970.1	
JCVI_12535	1.214	moderately similar to (321)AT2G17975| Symbols: | zinc finger (Ran-binding) family protein | chr2:7829320-7831052 REVERSE no origi	
ES978200	1.214	no similarity	-2.300
JCVI_36291	1.213	no original description	
JCVI_15407	1.213	moderately similar to (273)AT3G28860| Symbols: ATMDR11, ATMDR1, PGP19, MDR11, MDR1, ATPGP19 | ATMDR11/ATPGP19/M	
EX054805	1.213	moderately similar to (386)AT2G18890| Symbols: | protein kinase family protein | chr2:8191109-8192755 FORWARDweakly similar to (	-1.562
JCVI_22797	1.213	highly similar to (800)AT4G34520| Symbols: FAE1 | FAE1 (FATTY ACID ELONGATION1); acyltransferase | chr4:16494210-16495730	
EX086534	1.213	no similarity	
JCVI_19794	1.213	no original description	
EX040942	1.213	weakly similar to (183)AT4G28630| Symbols: ATATM1, ATM1 | ATM1 (Arabidopsis thaliana ABC transported of the mitochondria 1); A	
JCVI_24686	1.213	no original description	
ES967883	1.213	no similarity	
EE472115	1.213	no similarity	
JCVI_23791	1.212	no original description	
JCVI_321	1.212	moderately similar to (273)AT4G23310| Symbols: | receptor-like protein kinase, putative | chr4:12185747-12188773 FORWARD no origi	
JCVI_42346	1.212	highly similar to (546)AT4G21705| Symbols: | pentatricopeptide (PPR) repeat-containing protein | chr4:11533269-11534829 REVERSE r	
DY007220	1.212	no similarity	
JCVI_24193	1.212	weakly similar to (157)AT1G55530| Symbols: | zinc finger (C3HC4-type RING finger) family protein | chr1:20733138-20734193 REVER	
JCVI_15367	1.212	moderately similar to (364)AT1G61670| Symbols: | similar to unknown protein [Arabidopsis thaliana] (TAIR:AT1G10980.1); similar to h	
JCVI_40656	1.212	moderately similar to (309)AT5G24450| Symbols: | similar to unknown protein [Arabidopsis thaliana] (TAIR:AT3G49410.1); similar to p	
AM391436	1.212	no similarity	
EX127082	1.212	no similarity	
CD816166	1.212	no similarity	
JCVI_26275	1.212	no original description	
JCVI_40751	1.212	weakly similar to (120)AT4G37000| Symbols: ATRCCR, ACD2 | ACD2 (ACCELERATED CELL DEATH 2) | chr4:17442621-17443756	
JCVI_6910	1.211	moderately similar to (267)AT1G32080| Symbols: | membrane protein, putative | chr1:11537552-11539736 REVERSE no original descrip	
JCVI_35952	1.211	weakly similar to (150)AT3G16530| Symbols: | legume lectin family protein | chr3:5624592-5625422 REVERSE no original description	
JCVI_16051	1.211	no original description	
JCVI_24099	1.211	moderately similar to (335)AT1G47270| Symbols: AtTLP6 | AtTLP6 (TUBBY LIKE PROTEIN 6); phosphoric diester hydrolase/ transcrip	
JCVI_18680	1.211	moderately similar to (234)AT1G67840| Symbols: | ATP-binding region, ATPase-like domain-containing protein | chr1:25437819-254405	
JCVI_16588	1.211	moderately similar to (434)AT1G79930| Symbols: HSP91 | HSP91 (Heat shock protein 91) | chr1:30068817-30071960 REVERSE no origi	
EV003991	1.211	weakly similar to (153)AT2G05600| Symbols: | similar to F-box family protein [Arabidopsis thaliana] (TAIR:AT2G02030.1); similar to pr	
JCVI_28730	1.211	moderately similar to (315)AT4G37740| Symbols: AtGRF2 | AtGRF2 (GROWTHREGULATING FACTOR 2) | chr4:17725527-17727603	
ES941402	1.211	moderately similar to (223)AT3G17650| Symbols: PDE321, YSL5 | YSL5 (YELLOW STRIPE LIKE 5); oligopeptide transporter | chr3:60	
JCVI_6642	1.211	moderately similar to (233)AT1G65820| Symbols: | microsomal glutathione s-transferase, putative | chr1:24488876-24490345 FORWARI	
ES271898	1.211	moderately similar to (206)AT5G09730| Symbols: ATBXL3, XYL3, BXL3 | BXL3 (BETA-XYLOSIDASE 3); hydrolase, hydrolyzing O-g	
JCVI_34211	1.211	no original description	
EV120200	1.211	moderately similar to (341)AT1G02420| Symbols: | pentatricopeptide (PPR) repeat-containing protein | chr1:493683-495158 FORWARD	1.873
EX117764	1.211	very weakly similar to (85.9)AT1G70590| Symbols: | F-box family protein | chr1:26622066-26623822 FORWARD [21828]	
EV002274	1.210	moderately similar to (213)AT1G04870| Symbols: | protein arginine N-methyltransferase family protein | chr1:1373484-1375597 REVER	
EV002916	1.210	no similarity	
JCVI_15995	1.210	moderately similar to (333)AT1G03950| Symbols: VPS2.3 | VPS2.3 | chr1:1011387-1013211 REVERSE no original description	
EX073614	1.210	moderately similar to (271)AT1G27180| Symbols: | disease resistance protein (TIR-NBS-LRR class), putative | chr1:9439846-9445805 FC	
JCVI_33296	1.210	very weakly similar to (99.8)AT1G19025| Symbols: | DNA cross-link repair protein-related | chr1:6568911-6570791 FORWARD no origin	
EV201480	1.210	weakly similar to (144)AT4G01935| Symbols: | similar to unnamed protein product [Vitis vinifera] (GB:CAO40149.1) | chr4:841102-842	1.928
JCVI_29406	1.209	moderately similar to (353)AT4G26640| Symbols: ATWRKY20, WRKY20 | WRKY20 (WRKY DNA-binding protein 20); transcription fa	
JCVI_36991	1.209	very weakly similar to (93.6)AT3G43590| Symbols: | zinc knuckle (CCHC-type) family protein | chr3:15520741-15523243 FORWARD no	
AM394234	1.209	weakly similar to (200)AT1G62350| Symbols: | similar to pentatricopeptide (PPR) repeat-containing protein [Arabidopsis thaliana] (TAIR	
JCVI_36516	1.209	weakly similar to (162)AT2G29990| Symbols: NDA2 | NDA2 (ALTERNATIVE NAD(P)H DEHYDROGENASE 2); NADH dehydrogena	
EV056738	1.209	moderately similar to (231)AT4G32670| Symbols: | protein binding / zinc ion binding | chr4:15759532-15762852 REVERSE [21442]	
EV197143	1.209	no similarity	
ES901853	1.209	moderately similar to (305)AT5G27610| Symbols: | DNA binding / transcription factor | chr5:9764855-9769709 FORWARD [21428] 1 72	
EV078235	1.209	moderately similar to (414)AT1G65580| Symbols: FRA3 | FRA3 (FRAGILE FIBER3); inositol or phosphatidylinositol phosphatase | chr1:	-1.300
JCVI_32063	1.209	no original description	
EV205861	1.209	moderately similar to (222)AT2G39730| Symbols: RCA | RCA (RUBISCO ACTIVASE) | chr2:16578252-16580423 REVERSEweakly sin	
JCVI_29522	1.208	weakly similar to (191)AT4G17270| Symbols: | Mo25 family protein | chr4:9676419-9678581 FORWARD no original description	
JCVI_15382	1.208	moderately similar to (355)AT3G22950| Symbols: ATARFC1 | ATARFC1 (ADP-ribosylation factor C1); GTP binding | chr3:8136371-813	1.604
JCVI_18044	1.208	moderately similar to (215)AT1G71440| Symbols: TFC E, PFI | PFI (PFIFFERLING); protein binding | chr1:26924933-26928035 REVER	
EE428615	1.208	moderately similar to (258)AT3G08800| Symbols: | binding | chr3:2671154-2674916 FORWARD [20180]	
EX093883	1.208	very weakly similar to (100)AT1G80290| Symbols: | glycosyltransferase family protein 47 | chr1:30193312-30194466 FORWARD [2182	
JCVI_25902	1.207	moderately similar to (422)AT1G28640| Symbols: | GDSL-motif lipase, putative | chr1:10067549-10069095 REVERSEweakly similar to (	
AM389145	1.207	moderately similar to (258)AT4G19160| Symbols: | binding | chr4:10477532-10479958 FORWARD [20118]	
ES980663	1.207	weakly similar to (183)AT1G30120| Symbols: PDH-E1 BETA | PDH-E1 BETA (PYRUVATE DEHYDROGENASE E1 BETA); pyruvate	
EX093957	1.207	no similarity	
JCVI_6519	1.207	weakly similar to (119)AT3G20310| Symbols: ATERF-7, ATERF7, ERF7 | ATERF-7/ATERF7/ERF7 (ETHYLINE RESPONSE FACTOR	
JCVI_32385	1.207	highly similar to (522)AT5G60690| Symbols: IFL, IFL1, REV | REV (REVOLUTA); DNA binding / lipid binding / transcription factor | ch	
EV109508	1.207	moderately similar to (306)AT2G37970| Symbols: SOUL-1 | SOUL-1; binding | chr2:15898105-15898782 FORWARD [21478]	
EE478285	1.207	no similarity	
EV183510	1.207	very weakly similar to (96.7)AT1G64710| Symbols: | alcohol dehydrogenase, putative | chr1:24048499-24049976 FORWARD [21487] 1 3	
EV010117	1.206	no similarity	
JCVI_25617	1.206	no original description	
JCVI_3248	1.206	highly similar to (612)AT5G27740| Symbols: EMB2775 | EMB2775 (EMBRYO DEFECTIVE 2775); DNA binding / nucleoside-triphosph	
CO749843	1.206	no similarity	

JCVI_21052	1.206	moderately similar to (463)AT3G02920| Symbols: | replication protein-related | chr3:651851-654196 REVERSE no original description	
JCVI_120	1.206	highly similar to (510)AT4G37470| Symbols: | hydrolase, alpha/beta fold family protein | chr4:17617039-17618357 REVERSE no origina	
JCVI_39847	1.206	no original description	
EE433418	1.205	moderately similar to (272)AT2G20750| Symbols: EXPB1, ATHEXP BETA 1.5, ATEXPB1 | ATEXPB1 (ARABIDOPSIS THALIANA E	
JCVI_4657	1.205	moderately similar to (233)AT1G17280| Symbols: UBC34 | UBC34 (UBIQUITIN-CONJUGATING ENZYME 34) | chr1:5917139-591915	
EH428282	1.205	moderately similar to (387)AT5G25610| Symbols: RD22 | RD22 (RESPONSIVE TO DESSICATION 22) | chr5:8914501-8916687 REVE	
JCVI_13998	1.205	weakly similar to (115)AT5G22090| Symbols: | similar to unnamed protein product [Vitis vinifera] (GB:CAO67470.1) | chr5:7315006-731	
DY022441	1.205	moderately similar to (296)AT1G30400| Symbols: EST1, ATMRP1 | ATMRP1 (Arabidopsis thaliana multidrug resistance-associated prot	
JCVI_16784	1.205	weakly similar to (146)AT2G02760| Symbols: UBC2, ATUBC2 | ATUBC2 (UBIQUITING-CONJUGATING ENZYME 2); ubiquitin-prot	
JCVI_40914	1.205	highly similar to (666)AT1G80510| Symbols: | amino acid transporter family protein | chr1:30277992-30279461 FORWARD no original d	
JCVI_37513	1.205	highly similar to (754)AT1G61380| Symbols: | S-locus protein kinase, putative | chr1:22649942-22653066 REVERSEmoderately similar t	
JCVI_3152	1.205	weakly similar to (123)AT3G07880| Symbols: | Rho GDP-dissociation inhibitor family protein | chr3:2514181-2515550 FORWARD no or	-1.437
EE567187	1.204	weakly similar to (168)AT5G19450| Symbols: CPK8, CDPK19 | CDPK19 (CALCIUM-DEPENDENT PROTEIN KINASE 19); calmoduli	
EX039084	1.204	moderately similar to (361)AT1G65800| Symbols: ARK2 | ARK2 (Arabidopsis Receptor Kinase 2); kinase | chr1:24476829-24480186 FO	
CX193458	1.204	moderately similar to (355)AT3G51650| Symbols: | similar to unknown protein [Arabidopsis thaliana] (TAIR:AT3G51640.1); similar to u	
EX092731	1.204	moderately similar to (214)AT4G17420| Symbols: | similar to unknown protein [Arabidopsis thaliana] (TAIR:AT5G47420.1); similar to h	
JCVI_14800	1.204	moderately similar to (307)AT5G23550| Symbols: | similar to unknown protein [Arabidopsis thaliana] (TAIR:AT5G24170.1); similar to u	
JCVI_778	1.204	highly similar to (978)AT1G20620| Symbols: SEN2, CAT3 | CAT3 (CATALASE 3); catalase | chr1:7143132-7146183 FORWARDhighly	
EH422975	1.204	weakly similar to (126)AT3G14420| Symbols: | (S)-2-hydroxy-acid oxidase, peroxisomal, putative / glycolate oxidase, putative / short cha	
JCVI_28627	1.204	moderately similar to (208)AT1G27360| Symbols: | squamosa promoter-binding protein-like 11 (SPL11) | chr1:9502126-9503702 FORW	
JCVI_27172	1.204	moderately similar to (387)AT1G21210| Symbols: WAK4 | WAK4 (WALL ASSOCIATED KINASE 4); protein serine/threonine kinase | c	
EV214257	1.204	no similarity	
ES991587	1.204	moderately similar to (280)AT1G74160| Symbols: | similar to unknown protein [Arabidopsis thaliana] (TAIR:AT1G18620.2); similar to u	
JCVI_25027	1.204	highly similar to (812)AT5G49980| Symbols: AFB5 | AFB5 (AUXIN F-BOX PROTEIN 5); ubiquitin-protein ligase | chr5:20351646-2035	
JCVI_36391	1.204	no original description	
EX126640	1.204	no similarity	
JCVI_32359	1.203	highly similar to (666)AT1G15530| Symbols: | receptor lectin kinase, putative | chr1:5339956-5341926 REVERSEweakly similar to (116)	
JCVI_17728	1.203	moderately similar to (347)AT1G19940| Symbols: ATGH9B5 | ATGH9B5 (ARABIDOPSIS THALIANA GLYCOSYL HYDROLASE 9B	
JCVI_10960	1.203	moderately similar to (415)AT4G32830| Symbols: ATAUR1 | ATAUR1 (ATAURORA1); histone serine kinase(H3-S10 specific) / kinase/	
EV060550	1.203	moderately similar to (321)AT5G01950| Symbols: | ATP binding / kinase/ protein serine/threonine kinase | chr5:365037-369529 REVERS	
ES950503	1.203	no similarity	
JCVI_20786	1.203	highly similar to (616)AT5G53540| Symbols: | MSP1 protein, putative / intramitochondrial sorting protein, putative | chr5:21766787-2176	-2.072
AM062598	1.202	no similarity	
JCVI_6928	1.202	moderately similar to (349)AT1G17745| Symbols: PGDH | PGDH (3-PHOSPHOGLYCERATE DEHYDROGENASE); phosphoglycerate	
ES911550	1.202	weakly similar to (114)AT5G06280| Symbols: | similar to hypothetical protein [Vitis vinifera] (GB:CAN67410.1) | chr5:1918380-191885(	
EE483771	1.202	weakly similar to (147)AT4G21710| Symbols: EMB1989, RPB2, NRPB2 | NRPB2 (EMBRYO DEFECTIVE 1989); DNA binding | chr4:1	
RC_EE433163	1.202	no similarity	
CD839829	1.202	no similarity	
JCVI_10993	1.202	moderately similar to (212)AT1G21370| Symbols: | similar to unnamed protein product [Vitis vinifera] (GB:CAO60871.1) | chr1:7484239	
ES901679	1.202	no similarity	
ES983927	1.201	weakly similar to (178)AT3G06060| Symbols: | short-chain dehydrogenase/reductase (SDR) family protein | chr3:1828302-1830166 REV	
JCVI_29169	1.201	moderately similar to (334)AT5G03490| Symbols: | UDP-glucoronosyl/UDP-glucosyl transferase family protein | chr5:871549-872946 FC	
JCVI_11238	1.201	highly similar to (851)AT2G46720| Symbols: HIC | HIC (HIGH CARBON DIOXIDE); acyltransferase | chr2:19205891-19207291 FORW	
EE530671	1.201	weakly similar to (156)AT1G77600| Symbols: | binding | chr1:29157784-29167856 REVERSE [20175]	
JCVI_23258	1.201	weakly similar to (135)AT3G62450| Symbols: | similar to hypothetical protein MtrDRAFT_AC140551g61v2 [Medicago truncatula] (GB:	
RC_EX051211	1.200	no similarity	
DY020328	1.200	very weakly similar to (98.6)AT2G16730| Symbols: BGAL13 | BGAL13 (beta-galactosidase 13); beta-galactosidase | chr2:7269068-727318	
JCVI_32578	1.200	moderately similar to (223)AT1G24510| Symbols: | T-complex protein 1 epsilon subunit, putative / TCP-1-epsilon, putative / chaperonin,	
EV006559	1.200	weakly similar to (114)AT1G14800| Symbols: | similar to F-box family protein [Arabidopsis thaliana] (TAIR:AT2G35280.1); similar to hy	-1.433
CV433503	1.200	no similarity	
CV432588	1.200	moderately similar to (215)AT5G20350| Symbols: TIP1 | TIP1 (TIP GROWTH DEFECTIVE 1) | chr5:6876774-6881104 FORWARD [16	
EV019185	1.200	no similarity	1.524
CD827525	1.200	moderately similar to (456)AT2G40460| Symbols: | proton-dependent oligopeptide transport (POT) family protein | chr2:16904201-16908	
ES949525	1.200	weakly similar to (183)AT3G06510| Symbols: SFR2 | SFR2 (SENSITIVE TO FREEZING 2) | chr3:2016456-2019539 FORWARD [21393	
EV166141	1.200	no similarity	
JCVI_6039	1.199	highly similar to (890)AT3G04910| Symbols: ZIK4, WNK1 | WNK1 (WITH NO LYSINE (K) 1) | chr3:1356067-1358063 FORWARDver	
CD827416	1.199	weakly similar to (179)AT3G10480| Symbols: ANAC050 | ANAC050 | chr3:3264415-3266786 FORWARDweakly similar to (113)NAC7	
JCVI_39901	1.199	moderately similar to (409)AT3G57880| Symbols: | C2 domain-containing protein | chr3:21442175-21444496 REVERSE no original desc	
EX094919	1.199	weakly similar to (115)AT4G05010| Symbols: | F-box family protein | chr4:2567472-2568160 FORWARD [21824]	
EV064613	1.199	very weakly similar to (89.4)AT1G48400| Symbols: | F-box family protein | chr1:17885804-17887523 REVERSE [21443]	
JCVI_4806	1.199	very weakly similar to (99.0)AT1G53110| Symbols: | chorismate mutase | chr1:19794102-19795684 FORWARD no original description	
JCVI_36552	1.199	no original description	
EE507830	1.198	weakly similar to (131)AT3G55730| Symbols: AtMYB109, MYB109 | MYB109 (myb domain protein 109); DNA binding / transcription f	
JCVI_12107	1.198	highly similar to (665)AT3G13670| Symbols: | protein kinase family protein | chr3:4469441-4473241 FORWARD no original description	
JCVI_7228	1.198	moderately similar to (274)AT2G15480| Symbols: UGT73B5 | UGT73B5 (UDP-GLUCOSYL TRANSFERASE 73B5); UDP-glycosyltrans	
JCVI_1867	1.198	moderately similar to (429)AT1G22410| Symbols: | 2-dehydro-3-deoxyphosphoheptonate aldolase, putative / 3-deoxy-D-arabino-heptulos	
EX098992	1.198	moderately similar to (258)AT4G39720| Symbols: | VQ motif-containing protein | chr4:18429986-18430858 REVERSE [21825]	
JCVI_17563	1.198	weakly similar to (164)AT1G64810| Symbols: APO1 | APO1 (ACCUMULATION OF PHOTOSYSTEM ONE 1) | chr1:24090473-240919	
JCVI_8887	1.198	moderately similar to (251)AT5G29000| Symbols: | myb family transcription factor | chr5:11023017-11024233 REVERSE no original des	
JCVI_12267	1.198	moderately similar to (450)AT5G15570| Symbols: | similar to DNA binding [Arabidopsis thaliana] (TAIR:AT3G02160.1); similar to hypo	
JCVI_4062	1.197	moderately similar to (328)AT1G21650| Symbols: | ATP binding | chr1:7592880-7600108 REVERSE no original description	
JCVI_8313	1.197	moderately similar to (320)AT2G03760| Symbols: RAR047, ST | ST (steroid sulfotransferase); sulfotransferase | chr2:1149472-1150452 R	
JCVI_3409	1.197	very weakly similar to (94.0)AT1G11905| Symbols: | similar to unknown protein [Arabidopsis thaliana] (TAIR:AT5G42570.1); similar to u	
EV194056	1.197	weakly similar to (156)AT2G17480| Symbols: ATMLO8, MLO8 | MLO8 (MILDEW RESISTANCE LOCUS O 8); calmodulin binding | ch	
JCVI_21114	1.197	weakly similar to (109)AT5G14330| Symbols: | unknown protein | chr5:4619950-4620554 REVERSE no original description	
EE560964	1.196	no similarity	
JCVI_42435	1.196	weakly similar to (169)AT1G12420| Symbols: ACR8 | ACR8 (ACT Domain Repeat 8) | chr1:4226671-4228915 REVERSE no original des	
EV002319	1.196	weakly similar to (109)AT4G37110| Symbols: | protein binding / zinc ion binding | chr4:17484337-17486191 REVERSE [21427]	
JCVI_3949	1.196	moderately similar to (238)AT5G02960| Symbols: | 40S ribosomal protein S23 (RPS23B) | chr5:693278-694394 REVERSEmoderately sir	
JCVI_129	1.196	moderately similar to (423)AT2G30860| Symbols: GLUTTR, ATGSTF7, ATGSTF9 | ATGSTF9 (Arabidopsis thaliana Glutathione S-trans	
JCVI_32135	1.196	highly similar to (660)AT3G63010| Symbols: ATGID1B, GID1B | ATGID1B/GID1B (GA INSENSITIVE DWARF1B); hydrolase | chr3:2	

JCVI_6078	1.196	weakly similar to (112)AT1G14060| Symbols: | similar to unknown protein [Arabidopsis thaliana] (TAIR:AT5G02210.1); similar to unna	
EV219040	1.196	moderately similar to (310)AT5G42970| Symbols: FUS4, EMB134, COP14, CSN4, FUS8, COP8 | COP8 (CONSTITUTIVE PHOTOMOF	-1.733
JCVI_9872	1.195	moderately similar to (348)AT4G01600| Symbols: | GRAM domain-containing protein / ABA-responsive protein-related | chr4:693210-69	
JCVI_36592	1.195	weakly similar to (139)AT4G28320| Symbols: | glycosyl hydrolase family 5 protein / cellulase family protein | chr4:14018299-14019978 F	
EH418190	1.195	moderately similar to (291)AT4G19500| Symbols: | disease resistance protein (TIR-NBS-LRR class), putative | chr4:10625798-10630150	
L46433	1.195	no similarity	
JCVI_40368	1.195	moderately similar to (360)AT1G52920| Symbols: GCR2, GPCR | GCR2/GPCR (G PROTEIN COUPLED RECEPTOR); abscisic acid bin	
EX020913	1.195	moderately similar to (248)AT2G13840| Symbols: | PHP domain-containing protein | chr2:5795698-5798139 REVERSE [21809] 20 477 4	
JCVI_13977	1.194	no original description	
JCVI_34062	1.194	moderately similar to (384)AT4G01440| Symbols: | nodulin MtN21 family protein | chr4:596531-598512 FORWARD no original descript	
JCVI_7046	1.194	moderately similar to (329)AT1G76020| Symbols: | similar to unknown protein [Arabidopsis thaliana] (TAIR:AT1G20225.1); similar to u	
JCVI_20741	1.194	weakly similar to (105)AT5G56670| Symbols: | 40S ribosomal protein S30 (RPS30C) | chr5:22952639-22953207 REVERSE no original d	
JCVI_10127	1.194	moderately similar to (240)AT3G48120| Symbols: | similar to unnamed protein product [Vitis vinifera] (GB:CAO64408.1) | chr3:1778312	
JCVI_23675	1.194	moderately similar to (465)AT4G34830| Symbols: | binding | chr4:16599981-16605999 REVERSEvery weakly similar to (100)RF1_ORY	
EV110007	1.194	very weakly similar to (95.1)AT2G19570| Symbols: AT-CDA1, DESZ, CDA1 | CDA1 (CYTIDINE DEAMINASE 1) | chr2:8477680-8478	
JCVI_29276	1.193	weakly similar to (172)AT3G21890| Symbols: | zinc finger (B-box type) family protein | chr3:7709305-7709670 REVERSE no original de	
JCVI_28199	1.193	weakly similar to (174)AT1G17620| Symbols: | similar to unknown protein [Arabidopsis thaliana] (TAIR:AT5G11890.1); similar to unkn	
JCVI_17963	1.193	no original description	
JCVI_17673	1.193	moderately similar to (281)AT2G38090| Symbols: | myb family transcription factor | chr2:15952356-15953853 FORWARD no original de	
ES969044	1.193	no similarity	
JCVI_36869	1.193	no original description	
JCVI_35876	1.192	moderately similar to (365)AT1G09750| Symbols: | chloroplast nucleoid DNA-binding protein-related | chr1:3157543-3158962 FORWAR	
JCVI_41947	1.192	no original description	
JCVI_18359	1.192	moderately similar to (459)AT3G49250| Symbols: | similar to ATP binding [Arabidopsis thaliana] (TAIR:AT5G24280.1); similar to unna	
EV158945	1.192	moderately similar to (271)AT3G28700| Symbols: | similar to Os01g0588800 [Oryza sativa (japonica cultivar-group)] (GB:NP_00104343	
EX099045	1.192	moderately similar to (295)AT2G19130| Symbols: | S-locus lectin protein kinase family protein | chr2:8300871-8303357 FORWARDmod	
JCVI_12942	1.192	weakly similar to (126)AT2G46540| Symbols: | similar to unknown [Populus trichocarpa] (GB:ABK93335.1) | chr2:19117759-19118753	-1.081
ES982195	1.192	no similarity	
EE447735	1.192	moderately similar to (309)AT2G22890| Symbols: | small conjugating protein ligase | chr2:9749580-9750419 REVERSE [20125]	1.720
JCVI_24247	1.192	very weakly similar to (85.5)AT1G06850| Symbols: ATBZIP52 | ATBZIP52 (ARABIDOPSIS THALIANA BASIC LEUCINE ZIPPER 52	
JCVI_8888	1.191	highly similar to (524)AT5G54780| Symbols: | RAB GTPase activator | chr5:22265922-22268918 REVERSE no original description	
JCVI_25920	1.191	moderately similar to (404)AT1G61660| Symbols: | basic helix-loop-helix (bHLH) family protein | chr1:22758268-22759836 REVERSE n	
JCVI_37744	1.191	moderately similar to (254)AT5G22950| Symbols: VPS24.1 | VPS24.1 | chr5:7681383-7682723 FORWARD no original description	
JCVI_31976	1.191	moderately similar to (286)AT3G48580| Symbols: | xyloglucan:xyloglucosyl transferase, putative / xyloglucan endotransglycosylase, puta	
CV546864	1.191	no similarity	
EE471272	1.191	weakly similar to (135)AT5G27220| Symbols: | protein transport protein-related | chr5:9578760-9582755 FORWARD [20163]	
EX138916	1.191	no similarity	
JCVI_19032	1.191	moderately similar to (486)AT1G24440| Symbols: | protein binding / zinc ion binding | chr1:8662328-8663765 FORWARD no original de	
JCVI_26440	1.191	nearly identical (1004)AT2G15620| Symbols: NIR, ATHNIR, NIR1 | NIR1 (NITRITE REDUCTASE); ferredoxin-nitrate reductase | chr2:6	
EV009255	1.191	weakly similar to (169)AT3G54120| Symbols: | reticulon family protein (RTNLB12) | chr3:20052247-20053111 REVERSE [21427]	
EV080327	1.191	weakly similar to (198)AT3G23610| Symbols: | dual specificity protein phosphatase (DsPTP1) | chr3:8478287-8479648 FORWARD [214	
EV031154	1.191	no similarity	
JCVI_11553	1.191	moderately similar to (253)AT2G31530| Symbols: EMB2289, SCY2 | EMB2289/SCY2 (EMBRYO DEFECTIVE 2289); P-P-bond-hydrol	
ES957182	1.191	weakly similar to (148)AT4G18100| Symbols: | 60S ribosomal protein L32 (RPL32A) | chr4:10035727-10036487 REVERSE [21423]	
RC_EV014664	1.190	no similarity	
JCVI_22356	1.190	moderately similar to (376)AT5G05140| Symbols: | transcription elongation factor-related | chr5:1520354-1522298 FORWARD no origin	
ES935396	1.190	moderately similar to (347)AT4G26860| Symbols: | alanine racemase family protein | chr4:13503297-13504699 REVERSE [16816]	
JCVI_39303	1.190	moderately similar to (302)AT3G58510| Symbols: | DEAD box RNA helicase, putative (RH11) | chr3:21651585-21654441 FORWARD n	
EV201379	1.190	weakly similar to (181)AT3G19340| Symbols: | sodium:dicarboxylate symporter | chr3:6701393-6704077 REVERSE [21490]	
CD833393	1.190	moderately similar to (315)AT4G30520| Symbols: | leucine-rich repeat family protein / protein kinase family protein | chr4:14908199-149	
JCVI_5874	1.189	moderately similar to (295)AT4G00100| Symbols: RPS13, PFL2, ATRPS13A | ATRPS13A (RIBOSOMAL PROTEIN S13A); structural c	
JCVI_34218	1.189	weakly similar to (168)AT3G55560| Symbols: AGF2 | AGF2 (AT-HOOK PROTEIN OF GA FEEDBACK 2) | chr3:20615883-20616815 R	
JCVI_41265	1.189	weakly similar to (164)AT3G13440| Symbols: | similar to unnamed protein product [Vitis vinifera] (GB:CAO41305.1); contains InterPro	1.240
JCVI_4386	1.189	moderately similar to (219)AT3G22680| Symbols: | Identical to Uncharacterized protein At3g22680 [Arabidopsis Thaliana] (GB:Q9LUJ3	
JCVI_32157	1.189	no original description	
ES940209	1.188	moderately similar to (408)AT3G03380| Symbols: DEGP7 | DEGP7 (DEGP PROTEASE 7); serine-type peptidase/ trypsin | chr3:799727-8	
JCVI_29114	1.188	highly similar to (604)AT4G08980| Symbols: | F-box family protein (FBW2) | chr4:5758990-5760105 FORWARD no original description	
JCVI_13636	1.188	weakly similar to (114)AT5G60690| Symbols: IFL, IFL1, REV | REV (REVOLUTA); DNA binding / lipid binding / transcription factor | c	
EV145973	1.188	no similarity	
EX104256	1.188	weakly similar to (113)AT3G11230| Symbols: | yippee family protein | chr3:3516689-3518199 FORWARDvery weakly similar to (88.6)Y	
CX273108	1.188	moderately similar to (205)AT1G51060| Symbols: HTA10 | HTA10; DNA binding | chr1:18930616-18931111 FORWARDweakly similar	
JCVI_912	1.188	no original description	
JCVI_2231	1.188	moderately similar to (446)AT1G29310| Symbols: | protein transport protein sec61, putative | chr1:10252648-10254512 FORWARD no o	
JCVI_14499	1.187	highly similar to (961)AT1G01600| Symbols: CYP86A4 | CYP86A4 (cytochrome P450, family 86, subfamily A, polypeptide 4); oxygen bi	
JCVI_33442	1.187	weakly similar to (193)AT4G24810| Symbols: | ABC1 family protein | chr4:12786801-12789608 REVERSE no original description	
JCVI_826	1.187	no original description	
JCVI_19245	1.187	weakly similar to (200)AT2G01400| Symbols: | unknown protein | chr2:174314-175079 FORWARD no original description	
JCVI_13147	1.187	weakly similar to (119)AT3G60220| Symbols: ATL4 | ATL4 (Arabidopsis T?xicos en Levadura 4); protein binding / zinc ion binding | chr3	
EX119809	1.187	no similarity	
EV090479	1.186	weakly similar to (114)AT3G23510| Symbols: | cyclopropane fatty acid synthase, putative / CPA-FA synthase, putative | chr3:8428078-84	
CX280929	1.186	weakly similar to (189)AT3G14075| Symbols: | lipase class 3 family protein | chr3:4663826-4666345 REVERSE [16818]	
ES966619	1.186	no similarity	
EV020438	1.185	moderately similar to (388)AT4G14340| Symbols: CKL11, CKI1 | CKI1 (CASEIN KINASE I); casein kinase I/ kinase | chr4:8248528-825	
JCVI_39241	1.185	weakly similar to (191)AT4G27520| Symbols: | plastocyanin-like domain-containing protein | chr4:13750674-13751825 REVERSE no ori	
JCVI_41469	1.185	moderately similar to (437)AT4G11220| Symbols: BTI2 | BTI2 (VIRB2-INTERACTING PROTEIN 2) | chr4:6838172-6839574 REVERS	
JCVI_16651	1.185	moderately similar to (290)AT5G63060| Symbols: | transporter | chr5:25312308-25313940 REVERSE no original description	
JCVI_770	1.185	moderately similar to (440)AT5G14800| Symbols: AT-P5R, AT-P5C1, EMB2772, P5CR | P5CR (PYRROLINE-5- CARBOXYLATE (P5	1.746
JCVI_39344	1.185	moderately similar to (226)AT1G09020| Symbols: ATSNF4, SNF4 | SNF4 (Sucrose NonFermenting 4) | chr1:2900152-2904215 REVERSI	
DY007003	1.184	weakly similar to (132)AT5G18800| Symbols: | NADH-ubiquinone oxidoreductase 19 kDa subunit (NDUFA8) family protein | chr5:62673	
EV087994	1.184	no similarity	
JCVI_42161	1.184	highly similar to (751)AT3G61580| Symbols: | delta-8 sphingolipid desaturase (SLD1) | chr3:22797228-22798577 FORWARD no original	

JCVI_8200	1.184	highly similar to (530)AT3G12100| Symbols: | cation efflux family protein / metal tolerance protein, putative | chr3:3855111-3857276 RE	
JCVI_2453	1.184	moderately similar to (266)AT3G47120| Symbols: | RNA recognition motif (RRM)-containing protein | chr3:17362319-17363754 REVER	
EV160125	1.184	moderately similar to (366)AT4G36890| Symbols: IRX14 | IRX14 (IRREGULAR XYLEM 14); transferase, transferring glycosyl groups /	
ES940700	1.184	no similarity	
JCVI_40066	1.184	weakly similar to (142)AT5G05210| Symbols: | nucleolar matrix protein-related | chr5:1548399-1549535 FORWARD no original descript	
CD830547	1.184	weakly similar to (162)AT3G46450| Symbols: | SEC14 cytosolic factor family protein / phosphoglyceride transfer family protein | chr3:17	
JCVI_11845	1.184	moderately similar to (219)AT3G12140| Symbols: | emsy N terminus domain-containing protein / ENT domain-containing protein | chr3:3	
JCVI_31227	1.184	highly similar to (634)AT1G54490| Symbols: EIN5, XRN4, AIN1 | AIN1 (ACC INSENSITIVE 1); 5'-3' exonuclease/ nucleic acid binding	
CX190451	1.184	weakly similar to (166)AT1G11880| Symbols: | similar to unnamed protein product [Vitis vinifera] (GB:CAO22037.1); contains InterPro	
EX089393	1.184	weakly similar to (176)AT5G19410| Symbols: | ABC transporter family protein | chr5:6545239-6547113 REVERSE [21823]	
JCVI_6185	1.184	moderately similar to (283)AT5G15120| Symbols: | similar to unknown protein [Arabidopsis thaliana] (TAIR:AT5G39890.1); similar to u	
JCVI_7027	1.183	moderately similar to (350)AT5G23940| Symbols: EMB3009 | EMB3009 (EMBRYO DEFECTIVE 3009); transferase | chr5:8076619-807	
JCVI_6330	1.183	moderately similar to (297)AT1G74970| Symbols: TWN3, RPS9 | RPS9 (RIBOSOMAL PROTEIN S9); structural constituent of ribosome	
DY026553	1.183	moderately similar to (209)AT1G49180| Symbols: | protein kinase family protein | chr1:18188508-18191112 REVERSE [18978]	
EE412847	1.183	moderately similar to (205)AT1G18100| Symbols: E12A11 | E12A11; phosphatidylethanolamine binding | chr1:6228042-6230103 REVER	
EE451861	1.183	weakly similar to (115)AT4G33770| Symbols: | inositol 1,3,4-trisphosphate 5/6-kinase family protein | chr4:16193591-16195240 REVERS	
EV218546	1.183	moderately similar to (240)AT1G34130| Symbols: STT3B | STT3B (STAUROSPORIN AND TEMPERATURE SENSITIVE 3-LIKE B);	
EE505308	1.183	weakly similar to (127)AT4G11480| Symbols: | protein kinase family protein | chr4:6971403-6973794 FORWARD [20139]	
EE535273	1.183	weakly similar to (165)AT5G35700| Symbols: FIM2 | FIM2 (FIMBRIN-LIKE PROTEIN 2); actin binding | chr5:13890063-13893662 REV	
JCVI_16028	1.183	highly similar to (519)AT3G26020| Symbols: | serine/threonine protein phosphatase 2A (PP2A) regulatory subunit B', putative | chr3:9515	
EE531115	1.183	moderately similar to (202)AT1G72650| Symbols: TRFL6 | TRFL6 (TRF-LIKE 6); DNA binding / transcription factor | chr1:27353915-27	
JCVI_24970	1.183	very weakly similar to (100)AT5G42890| Symbols: | sterol carrier protein 2 (SCP-2) family protein | chr5:17211686-17213138 REVERSE	
JCVI_39914	1.183	nearly identical (1211)AT3G12810| Symbols: SRCAP, CHR13, PIE1 | PIE1 (PHOTOPERIOD-INDEPENDENT EARLY FLOWERING 1)	
ES984423	1.182	no similarity	
EX087826	1.182	moderately similar to (342)AT2G04530| Symbols: CPZ | CPZ | chr2:1576805-1578608 FORWARDmoderately similar to (211)RNZN_W	
EE559078	1.182	no similarity	
JCVI_15868	1.182	moderately similar to (246)AT5G56150| Symbols: UBC30 | UBC30 (UBIQUITIN-CONJUGATING ENZYME 30); ubiquitin-protein ligas	
JCVI_4359	1.182	moderately similar to (227)AT4G15530| Symbols: PPDK | PPDK (PYRUVATE ORTHOPHOSPHATE DIKINASE) | chr4:8864826-8870	
JCVI_7731	1.182	highly similar to (517)AT4G30310| Symbols: | ribitol kinase, putative | chr4:14831919-14835098 FORWARD no original description	
JCVI_338	1.181	moderately similar to (224)AT3G02470| Symbols: SAMDC | SAMDC (S-ADENOSYLMETHIONINE DECARBOXYLASE) | chr3:51023	-1.958
EX098701	1.181	moderately similar to (370)AT3G01380| Symbols: | phosphatidylinositolglycan class N (PIG-N) family protein | chr3:144295-149835 REV	
JCVI_25104	1.181	no original description	
ES934966	1.181	moderately similar to (323)AT1G26940| Symbols: | peptidyl-prolyl cis-trans isomerase cyclophilin-type family protein | chr1:9343180-934	
JCVI_31957	1.181	weakly similar to (175)AT1G76760| Symbols: ATY1 | ATY1 (Arabidopsis thioredoxin y1); thiol-disulfide exchange intermediate | chr1:28	
JCVI_27849	1.181	moderately similar to (233)AT2G23450| Symbols: | protein kinase family protein | chr2:9996006-9998324 REVERSEvery weakly similar	
EV156621	1.181	moderately similar to (417)AT3G62980| Symbols: TIR1 | TIR1 (TRANSPORT INHIBITOR RESPONSE 1); ubiquitin-protein ligase | chr3	
EX088625	1.181	no similarity	
EE463219	1.181	moderately similar to (296)AT3G24310| Symbols: AtMYB71, MYB305 | MYB305 (myb domain protein 305); DNA binding / transcriptio	
CV433421	1.181	no similarity	
EE523625	1.180	weakly similar to (138)AT5G05240| Symbols: | similar to unknown protein [Arabidopsis thaliana] (TAIR:AT2G40630.1); similar to hypot	
RC_EH416351	1.180	no similarity	
JCVI_1451	1.180	moderately similar to (472)AT5G18650| Symbols: | zinc finger (C3HC4-type RING finger) family protein | chr5:6218262-6220376 FORW	-1.334
EE546677	1.180	weakly similar to (178)AT1G55490| Symbols: LEN1, CPN60B | CPN60B (CHAPERONIN 60 BETA); ATP binding / protein binding / un	
JCVI_2468	1.180	moderately similar to (206)AT5G01790| Symbols: | unknown protein | chr5:305054-305680 REVERSE no original description	
ES968162	1.180	no similarity	-1.552
JCVI_24071	1.180	highly similar to (649)AT5G06150| Symbols: CYCB1;2, CYC1BAT | CYC1BAT (CYCLIN B 1;2); cyclin-dependent protein kinase regula	
EV136964	1.180	weakly similar to (123)AT5G11770| Symbols: | NADH-ubiquinone oxidoreductase 20 kDa subunit, mitochondrial | chr5:3791149-379293	
ES945640	1.180	no similarity	1.940
JCVI_34378	1.179	moderately similar to (398)AT5G09970| Symbols: CYP78A7 | CYP78A7 (cytochrome P450, family 78, subfamily A, polypeptide 7); oxyg	
JCVI_22162	1.179	highly similar to (589)AT3G25210| Symbols: | pentatricopeptide (PPR) repeat-containing protein | chr3:9181585-9182724 FORWARDwe	
CN730574	1.179	no similarity	
JCVI_7984	1.179	moderately similar to (246)AT2G38480| Symbols: | integral membrane protein, putative | chr2:16118038-16118772 REVERSE no origina	
AM396071	1.178	no similarity	
EX121034	1.178	weakly similar to (109)AT3G61180| Symbols: | zinc finger (C3HC4-type RING finger) family protein | chr3:22656655-22658265 FORWA	
EV108405	1.178	weakly similar to (141)AT5G03430| Symbols: | phosphoadenosine phosphosulfate (PAPS) reductase family protein | chr5:849236-852866	
JCVI_42288	1.178	moderately similar to (289)AT5G62410| Symbols: TTN3, ATCAP-E1, ATSMC4, SMC2 | SMC2 (STRUCTURAL MAINTENANCE OF C	
JCVI_10945	1.178	moderately similar to (285)AT3G50920| Symbols: | phosphatidic acid phosphatase-related / PAP2-related | chr3:18933384-18934361 REV	
EX085832	1.178	weakly similar to (164)AT4G24740| Symbols: AME1, AFC2 | AFC2 (ARABIDOPSIS FUS3-COMPLEMENTING GENE 1); kinase | chr4	-3.001
EX051063	1.177	no similarity	
JCVI_4430	1.177	moderately similar to (284)AT1G75510| Symbols: | transcription initiation factor IIF beta subunit (TFIIF-beta) family protein | chr1:2835(	
JCVI_1688	1.177	moderately similar to (386)AT5G55610| Symbols: | similar to unknown [Populus trichocarpa] (GB:ABK94457.1) | chr5:22543207-22545(	
JCVI_23741	1.177	highly similar to (529)AT5G38990| Symbols: | protein kinase family protein | chr5:15626054-15628696 FORWARDmoderately similar to	
JCVI_24138	1.177	no original description	
JCVI_13769	1.177	moderately similar to (276)AT1G71900| Symbols: | similar to permease-related [Arabidopsis thaliana] (TAIR:AT1G34470.1); similar to h	
JCVI_5851	1.177	highly similar to (524)AT5G57015| Symbols: CKL12 | CKL12 (Casein Kinase I-like 12); casein kinase I/ kinase | chr5:23088734-2309180	
JCVI_26294	1.177	no original description	-1.100
DN965558	1.177	no similarity	
ES906448	1.176	weakly similar to (168)AT4G08850| Symbols: | leucine-rich repeat family protein / protein kinase family protein | chr4:5637464-5640493	
EV141108	1.176	no similarity	
AM061214	1.176	moderately similar to (278)AT3G02060| Symbols: | DEAD/DEAH box helicase, putative | chr3:354419-358326 FORWARD [17712]	
JCVI_25759	1.176	very weakly similar to (95.9)AT1G56220| Symbols: | dormancy/auxin associated family protein | chr1:21047079-21048046 FORWARD n	
JCVI_23177	1.175	highly similar to (957)AT4G29210| Symbols: GGT3, GGT4 | GGT3/GGT4 (GAMMA-GLUTAMYL TRANSPEPTIDASE 3); gamma-glut	
JCVI_26041	1.175	moderately similar to (385)AT1G54115| Symbols: | cation exchanger, putative | chr1:20205786-20207720 FORWARD no original descrip	-1.363
JCVI_5614	1.175	moderately similar to (221)AT4G34720| Symbols: VHA-C1, ATVHA-C1, AVA-P1 | AVA-P1 (vacuolar H+-pumping ATPase 16 kDa prot	
JCVI_7181	1.175	moderately similar to (202)AT3G05020| Symbols: ACP, ACP1 | ACP1 (ACYL CARRIER PROTEIN 1) | chr3:1391869-1392884 REVERS	
JCVI_34281	1.175	no original description	
JCVI_32923	1.174	no original description	
EX085716	1.174	moderately similar to (262)AT1G35510| Symbols: | similar to unknown protein [Arabidopsis thaliana] (TAIR:AT2G01480.1); similar to u	
JCVI_2408	1.174	moderately similar to (207)AT2G01970| Symbols: | endomembrane protein 70, putative | chr2:452196-454818 REVERSE no original desc	-1.724
JCVI_7634	1.174	moderately similar to (246)AT3G13000| Symbols: | transcription factor | chr3:4158221-4160825 REVERSE no original description	1.034
JCVI_2081	1.174	moderately similar to (459)AT3G57610| Symbols: ATPURA | ATPURA; adenylosuccinate synthase | chr3:21345496-21347580 REVERSI	

EV041722	1.174	moderately similar to (263)AT2G40935| Symbols: | similar to unknown protein [Arabidopsis thaliana] (TAIR:AT3G18470.1); similar to h	
ES958257	1.174	no similarity	
JCVI_5147	1.174	moderately similar to (335)AT3G08010| Symbols: ATAB2 | ATAB2; RNA binding | chr3:2556052-2557432 FORWARD no original descr	
JCVI_32863	1.174	no original description	
JCVI_42633	1.174	moderately similar to (238)AT5G22090| Symbols: | similar to unnamed protein product [Vitis vinifera] (GB:CAO67470.1) | chr5:7315006	
JCVI_16320	1.173	moderately similar to (294)AT1G20780| Symbols: | armadillo/beta-catenin repeat protein-related / U-box domain-containing protein | chr1	
JCVI_14963	1.173	no original description	
JCVI_8406	1.173	moderately similar to (411)AT4G26870| Symbols: | aspartyl-tRNA synthetase, putative / aspartate--tRNA ligase, putative | chr4:13505387	
EE521368	1.173	no similarity	
JCVI_12467	1.173	very weakly similar to (91.7)AT1G19000| Symbols: | myb family transcription factor | chr1:6561326-6562675 REVERSE no original descr	
JCVI_7771	1.173	highly similar to (624)AT1G20950| Symbols: | pyrophosphate--fructose-6-phosphate 1-phosphotransferase-related / pyrophosphate-depen	
EV215259	1.172	weakly similar to (178)AT5G23110| Symbols: | zinc finger (C3HC4-type RING finger) family protein | chr5:7758310-7775512 FORWAR	-1.240
JCVI_22107	1.172	moderately similar to (317)AT5G59780| Symbols: AtMYB59, MYB59 | MYB59 (myb domain protein 59); DNA binding / transcription fa	
JCVI_5212	1.172	moderately similar to (264)AT1G31160| Symbols: | zinc-binding protein, putative / protein kinase C inhibitor, putative | chr1:11122861-11	
JCVI_27785	1.172	very weakly similar to (90.1)AT1G31010| Symbols: OSB4 | OSB4 (ORGANELLAR SINGLE-STRANDED DNA BINDING PROTEIN 4);	
DY028030	1.172	weakly similar to (185)AT3G26920| Symbols: | F-box family protein | chr3:9922478-9925128 FORWARD [18978]	
JCVI_23135	1.172	moderately similar to (452)AT5G04620| Symbols: ATBIOF | ATBIOF; transaminase | chr5:1328005-1329935 FORWARD no original des	
JCVI_16392	1.172	moderately similar to (442)AT5G58020| Symbols: | similar to predicted protein [Physcomitrella patens subsp. patens] (GB:EDQ76028.1);	
ES899946	1.172	weakly similar to (103)AT3G25870| Symbols: | similar to unknown protein [Arabidopsis thaliana] (TAIR:AT1G13360.1); similar to unkn	
JCVI_41561	1.171	moderately similar to (480)AT2G22840| Symbols: AtGRF1 | AtGRF1 (GROWTH-REGULATING FACTOR 1) | chr2:9735921-9738221 F	-1.285
EV219551	1.171	very weakly similar to (91.3)AT1G79630| Symbols: | protein phosphatase 2C family protein / PP2C family protein | chr1:29967824-29969	
EE450220	1.171	no similarity	
JCVI_5844	1.171	moderately similar to (211)AT5G65360| Symbols: | histone H3 | chr5:26137325-26137735 REVERSEmoderately similar to (209)H32_W	
JCVI_29039	1.171	no original description	
JCVI_16345	1.171	weakly similar to (156)AT2G32100| Symbols: ATOFP16, OFP16 | ATOFP16/OFP16 (Arabidopsis thaliana ovate family protein 16) | chr2	
EV042408	1.171	weakly similar to (111)AT5G10890| Symbols: | myosin heavy chain-related | chr5:3434031-3435068 REVERSE [21442]	
EV156557	1.171	moderately similar to (427)AT2G40540| Symbols: ATKT2, SHY3, KUP2, ATKUP2, TRK2, KT2 | KT2 (POTASSIUM TRANSPORTER	
EE392323	1.171	no similarity	
CD821274	1.170	moderately similar to (217)AT2G38670| Symbols: PECT1 | PECT1 (PHOSPHORYLETHANOLAMINE CYTIDYLYLTRANSFERASE 1	
JCVI_25353	1.170	no original description	
JCVI_4395	1.170	moderately similar to (314)AT3G47380| Symbols: | invertase/pectin methylesterase inhibitor family protein | chr3:17468780-17469388 FC	
JCVI_8066	1.170	moderately similar to (353)AT5G13240| Symbols: | transcription regulator | chr5:4225264-4227155 REVERSE no original description	
EV158333	1.170	moderately similar to (205)AT1G55670| Symbols: PSAG | PSAG | chr1:20806540-20807022 REVERSEweakly similar to (156)PSAG_SP	
EL588261	1.170	moderately similar to (301)AT4G24040| Symbols: ATTRE1, TRE1 | ATTRE1/TRE1 (TREHALASE 1); alpha,alpha-trehalase/ trehalase | c	
ES978206	1.170	no similarity	
JCVI_3252	1.170	moderately similar to (313)AT3G19390| Symbols: | cysteine proteinase, putative / thiol protease, putative | chr3:6723030-6724774 FORW	
JCVI_15306	1.170	moderately similar to (312)AT3G21650| Symbols: | serine/threonine protein phosphatase 2A (PP2A) regulatory subunit B', putative | chr3:	-1.479
JCVI_29031	1.170	weakly similar to (127)AT3G56290| Symbols: | similar to hypothetical protein [Vitis vinifera] (GB:CAN75527.1) | chr3:20889722-20890	
JCVI_22336	1.169	weakly similar to (127)AT1G53850| Symbols: PAE1 | PAE1 (20S proteasome alpha subunit E1) | chr1:20107799-20109460 REVERSEwe	
EE415527	1.169	weakly similar to (169)AT3G59845| Symbols: | NADP-dependent oxidoreductase, putative | chr3:22117398-22118384 REVERSE [20141	
JCVI_38413	1.169	no original description	
EX054175	1.169	no similarity	1.586
AM056590	1.169	weakly similar to (178)AT5G39970| Symbols: | catalytic | chr5:16015383-16017979 FORWARD [17712] 1 459 502	
JCVI_25828	1.169	moderately similar to (461)AT4G12720| Symbols: NUDT7, GFG1, AtNUDT7 | AtNUDT7 (ARABIDOPSIS THALIANA NUDIX HYDRO	
JCVI_32301	1.169	moderately similar to (455)AT5G58270| Symbols: ATATM3, ATM3, STA1 | STA1 (STARIK 1); ATPase, coupled to transmembrane mov	
EV073327	1.169	weakly similar to (145)AT2G31390| Symbols: | pfkB-type carbohydrate kinase family protein | chr2:13390712-13393193 REVERSEweak	
JCVI_23251	1.169	highly similar to (577)AT2G18915| Symbols: ADO2, LKP2 | LKP2 (LOV KELCH PROTEIN 2); ubiquitin-protein ligase | chr2:8201874-8	
EV079655	1.168	no similarity	
JCVI_23996	1.168	weakly similar to (105)AT3G08810| Symbols: | kelch repeat-containing F-box family protein | chr3:2675637-2676668 REVERSE no origi	-2.359
EV008363	1.168	moderately similar to (267)AT4G37170| Symbols: | pentatricopeptide (PPR) repeat-containing protein | chr4:17498574-17500649 REVER	
EE531010	1.167	no similarity	
JCVI_1079	1.167	moderately similar to (290)AT5G07020| Symbols: | proline-rich family protein | chr5:2180670-2182285 REVERSE no original description	
JCVI_3592	1.167	no original description	
DY015875	1.167	no similarity	
EE551967	1.167	no similarity	
JCVI_20169	1.167	weakly similar to (121)AT3G23740| Symbols: | similar to unknown protein [Arabidopsis thaliana] (TAIR:AT4G14120.1); similar to hypot	
EL592134	1.167	weakly similar to (132)AT1G25055| Symbols: | Identical to F-box/Kelch-repeat protein At1g24795/At1g24885/At1g25056/At1g25143/At	
BP999926	1.167	weakly similar to (152)AT1G60500| Symbols: | dynamin family protein | chr1:22295247-22297487 FORWARD [19755]	
JCVI_24901	1.167	weakly similar to (149)AT3G54620| Symbols: ATBZIP25, BZO2H4 | ATBZIP25/BZO2H4 (ARABIDOPSIS THALIANA BASIC LEUCI	1.423
ES928982	1.167	no similarity	
JCVI_21454	1.167	moderately similar to (342)AT1G27385| Symbols: | similar to unnamed protein product [Vitis vinifera] (GB:CAO66165.1); contains Inter	
JCVI_35575	1.166	very weakly similar to (100)AT1G20910| Symbols: | ARID/BRIGHT DNA-binding domain-containing protein | chr1:7277198-7280624 R	
JCVI_17683	1.166	moderately similar to (498)AT4G20800| Symbols: | FAD-binding domain-containing protein | chr4:11139667-11141253 FORWARD no o	
JCVI_17773	1.166	moderately similar to (219)AT5G59950| Symbols: | RNA and export factor-binding protein, putative | chr5:24157461-24158636 FORWAI	
ES948708	1.166	weakly similar to (146)AT1G55690| Symbols: | SEC14 cytosolic factor family protein / phosphoglyceride transfer family protein | chr1:20	
JCVI_19351	1.166	moderately similar to (201)AT1G27170| Symbols: | ATP binding / protein binding / transmembrane receptor | chr1:9434705-9439206 FOI	
JCVI_35577	1.166	no original description	
EE531508	1.166	weakly similar to (152)AT4G24520| Symbols: AR1, ATR1 | ATR1 (ARABIDOPSIS P450 REDUCTASE 1) | chr4:12663075-12667076 RI	-2.857
EE423194	1.166	weakly similar to (144)AT4G02750| Symbols: | pentatricopeptide (PPR) repeat-containing protein | chr4:1221116-1223461 REVERSE [2(	
JCVI_19329	1.166	moderately similar to (238)AT1G02860| Symbols: NLA | NLA (NITROGEN LIMITATION ADAPTATION); protein binding / zinc ion bi	
JCVI_9010	1.166	moderately similar to (454)AT3G52140| Symbols: | tetratricopeptide repeat (TPR)-containing protein | chr3:19344210-19352273 FORWA	1.234
EE449511	1.166	weakly similar to (189)AT3G06580| Symbols: GALK, GAL1 | GAL1 (GALACTOSE KINASE 1); ATP binding / galactokinase | chr3:2049	
EV170898	1.166	very weakly similar to (89.4)AT3G61430| Symbols: ATPIP1, PIP1, PIP1;1, PIP1A | PIP1A (PLASMA MEMBRANE INTRINSIC PROTE	
ES964763	1.166	no similarity	1.441
JCVI_29217	1.166	very weakly similar to (96.3)AT3G47070| Symbols: | similar to unknown [Populus trichocarpa] (GB:ABK95428.1) | chr3:17348190-17348	
JCVI_6390	1.165	moderately similar to (205)AT2G34310| Symbols: | similar to unknown protein [Arabidopsis thaliana] (TAIR:AT1G29530.1) | chr2:14485	
JCVI_5781	1.165	moderately similar to (228)AT1G03140| Symbols: | splicing factor Prp18 family protein | chr1:754471-756223 REVERSE no original desc	
JCVI_25329	1.165	highly similar to (540)AT5G05340| Symbols: | peroxidase, putative | chr5:1579143-1580820 REVERSEhighly similar to (515)PERP7_BI	
JCVI_11702	1.165	highly similar to (523)AT4G33150| Symbols: LKR/SDH, LKR, SDH | LKR (SACCHAROPINE DEHYDROGENASE) | chr4:15985482-15	
EV147961	1.165	very weakly similar to (83.6)AT2G15730| Symbols: | similar to unknown protein [Arabidopsis thaliana] (TAIR:AT4G34420.1); similar to u	
DY015564	1.165	no similarity	

EV149067	1.165	no similarity	-3.439
EX112843	1.164	weakly similar to (136)AT3G27350| Symbols: | similar to unknown protein [Arabidopsis thaliana] (TAIR:AT5G40700.1); similar to unna	
JCVI_6653	1.164	weakly similar to (191)AT2G23670| Symbols: YCF37 | YCF37 (Arabidopsis homolog of Synechocystis YCF37) | chr2:10070387-1007089	
EE522820	1.164	no similarity	
JCVI_41740	1.164	weakly similar to (130)AT2G18328| Symbols: | DNA binding | chr2:7971560-7971793 FORWARD no original description	
ES266497	1.164	weakly similar to (155)AT2G30370| Symbols: | allergen-related | chr2:12947654-12949244 REVERSE [21032]	
JCVI_4645	1.164	moderately similar to (392)AT2G38710| Symbols: | AMMECR1 family | chr2:16191595-16193842 REVERSE no original description	-1.697
JCVI_20934	1.164	weakly similar to (171)AT4G25340| Symbols: | immunophilin-related / FKBP-type peptidyl-prolyl cis-trans isomerase-related | chr4:1295	
EV181486	1.164	moderately similar to (327)AT4G23990| Symbols: CSLG3, ATCSLG3 | ATCSLG3 (Cellulose synthase-like G3); transferase/ transferase, t	
JCVI_32732	1.163	weakly similar to (105)AT2G29020| Symbols: | Rab5-interacting family protein | chr2:12476946-12478505 FORWARD no original descri	
JCVI_40128	1.163	weakly similar to (179)AT3G47780| Symbols: ATATH6 | ATATH6 (ABC2 homolog 6); ATPase, coupled to transmembrane movement of	
JCVI_8188	1.163	no original description	
JCVI_34207	1.163	weakly similar to (187)AT5G39760| Symbols: ATHB23 | ATHB23 (ARABIDOPSIS THALIANA HOMEOBOX PROTEIN 23); DNA bin	
JCVI_11918	1.163	weakly similar to (183)AT4G00650| Symbols: FLA, FRI | FRI (FRIGIDA) | chr4:269026-270363 FORWARD no original description	
JCVI_11836	1.163	moderately similar to (402)AT4G16440| Symbols: | iron hydrogenase family protein | chr4:9269286-9271532 REVERSE no original desc	
JCVI_16375	1.163	highly similar to (630)AT1G08460| Symbols: HDA8, HDA08 | HDA08 (histone deacetylase 8); histone deacetylase | chr1:2672524-26744	
EE556000	1.163	no similarity	
EE476206	1.163	no similarity	
JCVI_7454	1.162	moderately similar to (225)AT3G25930| Symbols: | universal stress protein (USP) family protein | chr3:9494269-9495108 REVERSE no	
ES941952	1.162	weakly similar to (172)AT1G14330| Symbols: | kelch repeat-containing F-box family protein | chr1:4890370-4891695 REVERSE [21391]	
EE560063	1.162	no similarity	
EE408221	1.162	no similarity	
JCVI_32672	1.162	moderately similar to (479)AT3G01180| Symbols: ATSS2 | ATSS2 (STARCH SYNTHASE 2); transferase, transferring glycosyl groups |	
JCVI_5593	1.162	moderately similar to (297)AT5G18460| Symbols: | similar to unknown protein [Arabidopsis thaliana] (TAIR:AT1G23340.1); similar to u	
EE489665	1.162	moderately similar to (250)AT1G67570| Symbols: | similar to unknown protein [Arabidopsis thaliana] (TAIR:AT1G50630.1); similar to u	
JCVI_5904	1.162	moderately similar to (318)AT2G20860| Symbols: LIP1 | LIP1 (LIPOIC ACID SYNTHASE 1); lipoic acid synthase | chr2:8986717-89880	
CD817459	1.162	no similarity	
JCVI_38584	1.162	moderately similar to (387)AT2G47000| Symbols: PGP4, MDR4 | MDR4/PGP4 (P-GLYCOPROTEIN4); ATPase, coupled to transmembr	
JCVI_20007	1.162	moderately similar to (406)AT3G49720| Symbols: | Identical to Uncharacterized protein At3g49720 [Arabidopsis Thaliana] (GB:Q9M2Y	
JCVI_5841	1.161	moderately similar to (213)AT5G51440| Symbols: | 23.5 kDa mitochondrial small heat shock protein (HSP23.5-M) | chr5:20908468-2090	
JCVI_39441	1.161	moderately similar to (363)AT2G17620| Symbols: CYCB2;1 | CYCB2;1 (CYCLIN B2;1); cyclin-dependent protein kinase regulator | chr2	
CB617707	1.161	no similarity	
JCVI_18162	1.161	highly similar to (604)AT5G66810| Symbols: | similar to unknown protein [Arabidopsis thaliana] (TAIR:AT1G61150.6); similar to unkno	
JCVI_33525	1.161	highly similar to (624)AT5G54390| Symbols: ATAHL, AHL | AHL (HAL2-LIKE); 3'(2'),5'-bisphosphate nucleotidase/ inositol or phospha	
EX099259	1.161	moderately similar to (310)AT1G04180| Symbols: | flavin-containing monooxygenase family protein / FMO family protein | chr1:110462	
CX280791	1.161	no similarity	
DN964664	1.161	very weakly similar to (98.2)AT1G07890| Symbols: MEE6, CS1, APX1 | APX1 (ASCORBATE PEROXIDASE 1) | chr1:2438002-243943	-1.403
EV153664	1.161	weakly similar to (183)AT3G14860| Symbols: | NHL repeat-containing protein | chr3:4998598-5000901 REVERSE [21484]	
JCVI_17179	1.161	highly similar to (508)AT2G03090| Symbols: EXP15, ATEXP15, ATHEXP ALPHA 1.3, ATEXPA15 | ATEXPA15 (ARABIDOPSIS TH	
JCVI_8344	1.161	moderately similar to (307)AT3G54030| Symbols: | protein kinase family protein | chr3:20022140-20024468 FORWARD no original desc	
JCVI_11391	1.160	moderately similar to (404)AT2G44040| Symbols: | dihydrodipicolinate reductase family protein | chr2:18229061-18231074 REVERSE n	
JCVI_35383	1.160	no original description	
JCVI_9504	1.160	weakly similar to (157)AT4G33250| Symbols: TIF3K1, ATTIF3K1, EIF3K | EIF3K (eukaryotic translation initiation factor 3K) | chr4:160	
JCVI_15093	1.160	weakly similar to (116)AT2G40000| Symbols: HSPRO2, ATHSPRO2 | similar to unknown protein [Arabidopsis thaliana] (TAIR:AT3G55	
JCVI_19720	1.160	no original description	
EV218621	1.160	no similarity	
AM385650	1.160	moderately similar to (231)AT5G02310| Symbols: PRT6 | PRT6 (PROTEOLYSIS 6); ubiquitin-protein ligase | chr5:474277-482550 FOR	
JCVI_36435	1.160	no original description	
CX280840	1.160	no similarity	
AM388890	1.160	weakly similar to (110)AT5G42870| Symbols: | lipin family protein | chr5:17202947-17206909 REVERSE [20118]	
JCVI_5251	1.160	moderately similar to (319)AT4G27960| Symbols: UBC9 | UBC9 (UBIQUITIN CONJUGATING ENZYME 9); ubiquitin-protein ligase |	
ES991947	1.160	moderately similar to (308)AT5G04940| Symbols: SUVH1 | SUVH1 (SU(VAR)3-9 HOMOLOG 1) | chr5:1454617-1456629 REVERSE [2	
JCVI_311	1.160	highly similar to (604)AT3G12500| Symbols: PR3, PR-3, CHI-B, B-CHI, ATHCHIB | ATHCHIB (BASIC CHITINASE); chitinase | chr3:	
JCVI_20990	1.159	weakly similar to (129)AT2G37975| Symbols: | similar to unknown protein [Arabidopsis thaliana] (TAIR:AT3G54085.1); similar to hypo	
ES955205	1.159	no similarity	
EV191641	1.159	very weakly similar to (83.2)AT4G32760| Symbols: | protein transporter | chr4:15799381-15803837 FORWARD [21489] 28 734 748	
EX087211	1.159	moderately similar to (310)AT3G50890| Symbols: ATHB28 | ATHB28 (ARABIDOPSIS THALIANA HOMEOBOX PROTEIN 28); DNA	
JCVI_29204	1.159	weakly similar to (160)AT3G10185| Symbols: | Encodes a Gibberellin-regulated GASA/GAST/Snakin family protein | chr3:3145584-314	-1.052
EE457772	1.159	no similarity	
JCVI_19801	1.158	highly similar to (555)AT4G28710| Symbols: ATXIH, XIH | XIH (Myosin-like protein XIH) | chr4:14172286-14181777 FORWARD no o	
JCVI_22694	1.158	moderately similar to (390)AT3G02360| Symbols: | 6-phosphogluconate dehydrogenase family protein | chr3:482505-483965 FORWARD	
JCVI_11401	1.158	highly similar to (537)AT1G51965| Symbols: | pentatricopeptide (PPR) repeat-containing protein | chr1:19315747-19317814 REVERSEw	
JCVI_35221	1.158	weakly similar to (114)AT2G03680| Symbols: SKU6, SPR1 | SPR1 (SPIRAL1) | chr2:1121395-1121847 FORWARD no original descripti	
EE567049	1.158	no similarity	
JCVI_13724	1.158	weakly similar to (157)AT4G20070| Symbols: ATAAH | ATAAH (ARABIDOPSIS THALIANA ALLANTOATE AMIDOHYDROLASE)	
JCVI_8860	1.157	weakly similar to (118)AT1G16810| Symbols: | similar to CGI-144-like protein [Solanum lycopersicum] (GB:CAC81814.1); similar to Os	
JCVI_13579	1.157	no original description	
EV212414	1.157	moderately similar to (251)AT3G62750| Symbols: | hydrolase, hydrolyzing O-glycosyl compounds | chr3:23225351-23227876 FORWAR	
JCVI_5163	1.157	moderately similar to (213)AT1G55670| Symbols: PSAG | PSAG | chr1:20806540-20807022 REVERSEweakly similar to (192)PSAG_SP	
JCVI_26131	1.157	moderately similar to (284)AT5G06430| Symbols: | thioredoxin-related | chr5:1963581-1964252 REVERSE no original description	
EV088164	1.157	no similarity	
JCVI_41522	1.157	no original description	-1.230
EV088449	1.156	no similarity	
EV177060	1.156	moderately similar to (241)AT2G27060| Symbols: | ATP binding / protein serine/threonine kinase | chr2:11558366-11561655 FORWARD	
JCVI_17220	1.156	moderately similar to (306)AT4G38580| Symbols: ATFP6 | ATFP6 (FARNESYLATED PROTEIN 6); metal ion binding | chr4:18034590-	
ES942963	1.156	no similarity	
EV028600	1.156	moderately similar to (436)AT1G75200| Symbols: | flavodoxin family protein / radical SAM domain-containing protein | chr1:28224510-2	
EE456806	1.156	no similarity	-1.284
JCVI_5144	1.156	moderately similar to (372)AT2G29460| Symbols: GST22, ATGSTU4 | ATGSTU4 (GLUTATHIONE S-TRANSFERASE 22); glutathione	
EE476451	1.156	moderately similar to (347)AT5G65900| Symbols: | DEAD/DEAH box helicase, putative | chr5:26375554-26378470 FORWARDweakly s	
JCVI_18510	1.156	no original description	

JCVI_38425	1.155	no original description	
JCVI_11577	1.155	moderately similar to (280)AT2G29450| Symbols: ATGSTU1, AT103-1A, ATGSTU5 | ATGSTU5 (Arabidopsis thaliana Glutathione S-tra	
CO750497	1.155	weakly similar to (184)AT5G47490| Symbols: | similar to unknown protein [Arabidopsis thaliana] (TAIR:AT5G47480.1); similar to hypot	
JCVI_39864	1.155	no original description	
JCVI_27471	1.155	moderately similar to (362)AT3G15980| Symbols: | coatomer protein complex, subunit beta 2 (beta prime), putative | chr3:5412021-5418	
JCVI_929	1.155	weakly similar to (196)AT1G65720| Symbols: | similar to hypothetical protein [Vitis vinifera] (GB:CAN83711.1); similar to unnamed pro	
EE408644	1.154	moderately similar to (232)AT1G75040| Symbols: PR-5, PR5 | PR5 (PATHOGENESIS-RELATED GENE 5) | chr1:28181415-28182392 I	
EX027678	1.154	moderately similar to (223)AT5G59540| Symbols: | oxidoreductase, 2OG-Fe(II) oxygenase family protein | chr5:24013841-24014802 RE	
ES940316	1.154	no similarity	
EV077392	1.154	weakly similar to (129)AT3G10620| Symbols: | diadenosine 5',5'''-P1,P4-tetraphosphate hydrolase, putative | chr3:3320268-3321570 FOR	
EV136796	1.154	very weakly similar to (98.6)AT3G07680| Symbols: | emp24/gp25L/p24 family protein | chr3:2455633-2456658 FORWARD [21481] 1 36	-1.287
JCVI_25866	1.154	moderately similar to (298)AT4G24390| Symbols: | F-box family protein (FBX14) | chr4:12613919-12615976 REVERSE no original desc	
JCVI_15901	1.153	moderately similar to (243)AT5G54680| Symbols: ILR3 | ILR3 (IAA-LEUCINE RESISTANT3); DNA binding / transcription factor | chr5	
EV176396	1.153	weakly similar to (150)AT2G39200| Symbols: ATMLO12, MLO12 | MLO12 (MILDEW RESISTANCE LOCUS O 12); calmodulin bindin	
JCVI_23620	1.153	no original description	
EV138048	1.153	no similarity	
JCVI_28745	1.153	weakly similar to (187)AT1G13020| Symbols: | eukaryotic translation initiation factor, putative (EIF4B5) | chr1:4440925-4443518 REVE	
JCVI_40396	1.153	highly similar to (654)AT1G53050| Symbols: | protein kinase family protein | chr1:19776242-19779199 FORWARDweakly similar to (11	
JCVI_36124	1.153	moderately similar to (496)AT2G32640| Symbols: | similar to unnamed protein product [Vitis vinifera] (GB:CAO24043.1); contains doma	
JCVI_35516	1.152	weakly similar to (119)AT5G40980| Symbols: | similar to unknown protein [Arabidopsis thaliana] (TAIR:AT3G27027.1); similar to unna	
JCVI_21124	1.152	moderately similar to (354)AT4G35160| Symbols: | O-methyltransferase family 2 protein | chr4:16730994-16732813 REVERSE no origin	
AM394827	1.152	very weakly similar to (89.7)AT5G53340| Symbols: | galactosyltransferase family protein | chr5:21658271-21660421 REVERSE [20346]	
JCVI_31476	1.152	very weakly similar to (98.6)AT3G20370| Symbols: | meprin and TRAF homology domain-containing protein / MATH domain-containing	
JCVI_1409	1.152	moderately similar to (430)AT3G51950| Symbols: | zinc finger (CCCH-type) family protein / RNA recognition motif (RRM)-containing p	
JCVI_33789	1.152	moderately similar to (424)AT1G03350| Symbols: | BSD domain-containing protein | chr1:822834-824246 REVERSE no original descript	
JCVI_5570	1.152	moderately similar to (223)AT5G54855| Symbols: | pollen Ole e 1 allergen and extensin family protein | chr5:22300128-22301477 FORW	1.732
EX063872	1.152	weakly similar to (103)AT5G62660| Symbols: | F-box family protein | chr5:25173552-25174691 REVERSE [21814]	1.406
EV134863	1.152	no similarity	
JCVI_20377	1.152	no original description	
EV227040	1.152	no similarity	
EX063012	1.152	no similarity	
JCVI_21084	1.152	no original description	
JCVI_10488	1.152	no original description	
EV147842	1.151	weakly similar to (187)AT5G45020| Symbols: | similar to unknown protein [Arabidopsis thaliana] (TAIR:AT4G19880.1); similar to Intrac	
JCVI_11350	1.151	moderately similar to (373)AT1G71870| Symbols: | MATE efflux family protein | chr1:27036118-27038557 REVERSE no original descri	-1.123
EV113998	1.151	weakly similar to (192)AT4G28600| Symbols: NPGR2 | NPGR2 (NO POLLEN GERMINATION RELATED 2); calmodulin binding | chr4	
CX268635	1.151	very weakly similar to (82.8)AT2G20370| Symbols: KAM1, MUR3 | KAM1/MUR3 (MURUS 3); catalytic/ transferase, transferring glycos	
JCVI_6315	1.151	moderately similar to (267)AT2G01970| Symbols: | endomembrane protein 70, putative | chr2:452196-454818 REVERSE no original desc	
JCVI_32577	1.151	no original description	
JCVI_12815	1.151	weakly similar to (124)AT1G12845| Symbols: | similar to hypothetical protein MtrDRAFT_AC149131g9v2 [Medicago truncatula] (GB:A	
EE531000	1.150	weakly similar to (147)AT5G13600| Symbols: | phototropic-responsive NPH3 family protein | chr5:4380435-4382500 FORWARD [2017	
JCVI_19574	1.150	weakly similar to (185)AT4G03060| Symbols: AOP2 | AOP2 (ALKENYL HYDROXALKYL PRODUCING 2); oxidoreductase, acting on	-1.211
JCVI_31011	1.150	no original description	
JCVI_32524	1.150	weakly similar to (156)AT3G47080| Symbols: | binding | chr3:17349400-17351114 REVERSE no original description	
EE440931	1.150	moderately similar to (282)AT3G16830| Symbols: TPR2 | TPR2 (TOPLESS-RELATED 2) | chr3:5731715-5737537 FORWARD [20167]	
EX092216	1.150	weakly similar to (157)AT3G05220| Symbols: | heavy-metal-associated domain-containing protein | chr3:1489741-1491177 FORWARD [	
JCVI_7697	1.150	moderately similar to (248)AT3G12350| Symbols: | F-box family protein | chr3:3931633-3933111 FORWARD no original description	
EX055250	1.149	moderately similar to (202)AT4G10310| Symbols: ATHKT1, HKT1 | HKT1 (HIGH-AFFINITY K+ TRANSPORTER 1); sodium ion trans	
JCVI_38155	1.149	weakly similar to (145)AT1G04945| Symbols: | similar to hypothetical protein [Vitis vinifera] (GB:CAN60314.1); contains InterPro doma	-1.492
JCVI_37273	1.149	moderately similar to (273)AT1G70740| Symbols: | protein kinase family protein | chr1:26677509-26679349 REVERSE no original descri	
EV031610	1.149	no similarity	
JCVI_40141	1.149	moderately similar to (230)AT5G49890| Symbols: ATCLC-C, CLC-C | CLC-C (chloride channel C); anion channel/ voltage-gated chloride	
EE568115	1.149	no similarity	
JCVI_6457	1.149	moderately similar to (284)AT1G18210| Symbols: | calcium-binding protein, putative | chr1:6268266-6268778 REVERSEweakly similar t	2.289
JCVI_35613	1.148	moderately similar to (268)AT1G20640| Symbols: | RWP-RK domain-containing protein | chr1:7155191-7157976 FORWARD no original	
JCVI_1365	1.148	moderately similar to (427)AT1G75180| Symbols: | similar to unknown protein [Arabidopsis thaliana] (TAIR:AT1G19400.1); similar to u	
JCVI_20323	1.148	moderately similar to (425)AT1G72040| Symbols: | deoxynucleoside kinase family | chr1:27115821-27117910 REVERSE no original des	
DY020450	1.148	no similarity	
JCVI_13955	1.148	weakly similar to (110)AT1G25570| Symbols: | leucine-rich repeat protein-related | chr1:8992170-8995417 REVERSE no original descrip	
JCVI_41010	1.148	moderately similar to (265)AT1G19790| Symbols: SRS7 | SRS7 (SHI-RELATED SEQUENCE 7) | chr1:6838391-6839822 REVERSE no	
ES903078	1.147	very weakly similar to (86.7)AT3G09830| Symbols: | protein kinase, putative | chr3:3017204-3018701 FORWARD [21432]	
EE402513	1.147	no similarity	
JCVI_39328	1.147	moderately similar to (241)AT1G52330| Symbols: | similar to unknown protein [Arabidopsis thaliana] (TAIR:AT4G13270.1); similar to h	
EX058496	1.147	very weakly similar to (95.1)AT3G18370| Symbols: SYTF, ATSYTF, NTMC2TYPE3, NTMC2T3 | ATSYTF/NTMC2T3/NTMC2TYPE3/	
EE484152	1.147	no similarity	
EE503331	1.147	weakly similar to (147)AT3G62740| Symbols: | glycosyl hydrolase family 1 protein | chr3:23222392-23224864 FORWARD [20129]	-1.430
CX267346	1.147	moderately similar to (251)AT2G41070| Symbols: ATBZIP12, DPBF4, EEL | EEL (ENHANCED EM LEVEL); DNA binding / transcripti	
JCVI_25260	1.147	moderately similar to (207)AT1G17520| Symbols: | DNA-binding protein, putative | chr1:6024952-6027217 REVERSE no original descri	
JCVI_5414	1.146	weakly similar to (161)AT2G27290| Symbols: | similar to unnamed protein product [Vitis vinifera] (GB:CAO48682.1); contains InterPro	
EX015757	1.146	no similarity	
JCVI_3225	1.146	weakly similar to (124)AT5G61020| Symbols: ECT3 | ECT3 (evolutionary conserved C-terminal 3) | chr5:24574711-24577006 REVERSE	
JCVI_4152	1.146	moderately similar to (258)AT3G53970| Symbols: | proteasome inhibitor-related | chr3:19996186-19998110 FORWARD no original desc	
JCVI_17517	1.146	moderately similar to (325)AT5G08160| Symbols: ATPK3 | ATPK3 (Arabidopsis thaliana serine/threonine protein kinase 3); kinase | chr5	
JCVI_4338	1.146	moderately similar to (413)AT3G07680| Symbols: | emp24/gp25L/p24 family protein | chr3:2455633-2456658 FORWARD no original de	
EE541070	1.146	no similarity	
JCVI_29586	1.146	moderately similar to (281)AT5G19910| Symbols: | SOH1 family protein | chr5:6731486-6732823 REVERSE no original description	
JCVI_12024	1.146	moderately similar to (217)AT3G12580| Symbols: HSP70 | HSP70 (heat shock protein 70); ATP binding | chr3:3991494-3993696 REVER	
EV012915	1.146	no similarity	
JCVI_2915	1.146	moderately similar to (377)AT2G21330| Symbols: | fructose-bisphosphate aldolase, putative | chr2:9135497-9137233 REVERSEmoderate	
EE464431	1.146	moderately similar to (246)AT3G03720| Symbols: CAT4 | CAT4 (CATIONIC AMINO ACID TRANSPORTER 4); cationic amino acid tr	
EE443395	1.146	no similarity	

JCVI_38380	1.145	very weakly similar to (83.2)AT5G06110| Symbols: | DNAJ heat shock N-terminal domain-containing protein / cell division protein-relatec	1.275
EE559228	1.145	no similarity	
EV192116	1.145	weakly similar to (106)AT5G19130| Symbols: | GPI transamidase component family protein / Gaa1-like family protein | chr5:6416130-64	
EX061339	1.145	moderately similar to (223)AT5G10770| Symbols: | chloroplast nucleoid DNA-binding protein, putative | chr5:3403332-3405332 REVERS	
EV157107	1.145	weakly similar to (124)AT1G65120| Symbols: | ubiquitin carboxyl-terminal hydrolase-related | chr1:24195384-24199570 REVERSE [214	
JCVI_19675	1.145	weakly similar to (131)AT5G53340| Symbols: | galactosyltransferase family protein | chr5:21658271-21660421 REVERSE no original des	
JCVI_1106	1.145	moderately similar to (281)AT5G65860| Symbols: | ankyrin repeat family protein | chr5:26364932-26365912 REVERSE no original descr	
JCVI_42148	1.145	moderately similar to (318)AT3G49590| Symbols: | similar to unknown protein [Arabidopsis thaliana] (TAIR:AT3G18770.1); similar to u	
ES977600	1.145	no similarity	
JCVI_23063	1.145	moderately similar to (404)AT5G47670| Symbols: | CCAAT-box binding transcription factor family protein / leafy cotyledon 1-related (L	
JCVI_1933	1.145	highly similar to (606)AT1G23090| Symbols: SULTR3;3, AST91 | AST91 (SULFATE TRANSPORTER 91); sulfate transmembrane trans	-2.055
JCVI_24207	1.144	highly similar to (621)AT5G41970| Symbols: | similar to unknown protein [Arabidopsis thaliana] (TAIR:AT3G49320.1); similar to unnam	
JCVI_8443	1.144	moderately similar to (464)AT3G52610| Symbols: | similar to hypothetical protein [Vitis vinifera] (GB:CAN71367.1); contains domain C	
EV041837	1.144	weakly similar to (144)AT5G17400| Symbols: ER-ANT1 | ADP, ATP carrier protein, mitochondrial, putative / ADP/ATP translocase, puta	
JCVI_38521	1.144	no original description	
EE545858	1.144	very weakly similar to (93.6)AT2G06000| Symbols: | pentatricopeptide (PPR) repeat-containing protein | chr2:2327997-2329607 REVERS	
JCVI_31070	1.144	moderately similar to (254)AT4G20110| Symbols: | vacuolar sorting receptor, putative | chr4:10875577-10878555 FORWARDweakly sim	
DV643335	1.144	no similarity	
EV114910	1.143	weakly similar to (188)AT5G25930| Symbols: | leucine-rich repeat family protein / protein kinase family protein | chr5:9050883-9053981	1.281
JCVI_4674	1.143	moderately similar to (384)AT1G76160| Symbols: SKS5 | SKS5 (SKU5 Similar 5); copper ion binding / oxidoreductase | chr1:28583105-2	
JCVI_10076	1.143	highly similar to (546)AT1G05850| Symbols: ERH2, ELP1, CTL1, ELP, HOT2, POM1 | POM1 (POM-POM1); chitinase | chr1:1766832-1	
EE433626	1.143	weakly similar to (118)AT5G12320| Symbols: | ankyrin repeat family protein | chr5:3982763-3983900 FORWARD [15720]	-1.683
CX194238	1.143	moderately similar to (344)AT1G08960| Symbols: ATCAX11, CAX11 | CAX11 (cation exchanger 11); cation:cation antiporter | chr1:2879	
JCVI_10215	1.143	highly similar to (535)AT1G04920| Symbols: ATSPS3F | ATSPS3F (sucrose phosphate synthase 3F); sucrose-phosphate synthase/ transfe	
EX089858	1.142	weakly similar to (115)AT1G25370| Symbols: | similar to unknown protein [Arabidopsis thaliana] (TAIR:AT1G68340.1); similar to unna	
DY022531	1.142	moderately similar to (226)AT5G07730| Symbols: | similar to unknown protein [Arabidopsis thaliana] (TAIR:AT5G61360.1) | chr5:24575	
RC_EX067752	1.142	no similarity	
EV040723	1.142	no similarity	
JCVI_9390	1.142	moderately similar to (489)AT5G26940| Symbols: | exonuclease family protein | chr5:9481432-9482650 FORWARD no original descripti	
JCVI_12221	1.142	highly similar to (511)AT4G25900| Symbols: | aldose 1-epimerase family protein | chr4:13161496-13163406 FORWARD no original desc	
ES902503	1.142	very weakly similar to (95.5)AT2G24220| Symbols: ATPUP5 | ATPUP5 (Arabidopsis thaliana purine permease 5); purine transmembrane t	-1.360
JCVI_5937	1.142	weakly similar to (180)AT2G44360| Symbols: | similar to unnamed protein product [Vitis vinifera] (GB:CAO67186.1); contains InterPro	
ES966402	1.142	very weakly similar to (81.3)AT5G46740| Symbols: UBP21 | UBP21 (UBIQUITIN-SPECIFIC PROTEASE 21); ubiquitin-specific protease	
DY028781	1.142	moderately similar to (269)AT5G16610| Symbols: | similar to unnamed protein product [Vitis vinifera] (GB:CAO44747.1) | chr5:5445071	
JCVI_32148	1.141	moderately similar to (280)AT4G14340| Symbols: CKL11, CKI1 | CKI1 (CASEIN KINASE I); casein kinase I/ kinase | chr4:8248528-825	
JCVI_8526	1.141	moderately similar to (394)AT1G08930| Symbols: ERD6 | ERD6 (EARLY RESPONSE TO DEHYDRATION 6); carbohydrate transmeml	
EV192567	1.141	weakly similar to (155)AT2G44430| Symbols: | DNA-binding bromodomain-containing protein | chr2:18340679-18343241 FORWARD [2	
JCVI_17402	1.141	moderately similar to (428)AT5G22030| Symbols: | ubiquitin-specific protease 8, putative (UBP8) | chr5:7290158-7293597 REVERSE no	1.129
CB686250	1.141	no similarity	
JCVI_18821	1.141	highly similar to (694)AT3G01300| Symbols: | protein kinase, putative | chr3:90824-93342 REVERSEmoderately similar to (211)NORK_	
EV085430	1.141	no similarity	
JCVI_15524	1.141	highly similar to (631)AT3G45140| Symbols: ATLOX2, LOX2 | LOX2 (LIPOXYGENASE 2) | chr3:16536422-16540218 FORWARDmoc	
EE457254	1.140	weakly similar to (135)AT4G01510| Symbols: ARV2 | ARV2 | chr4:642755-644190 FORWARD [20179]	
ES927658	1.140	no similarity	
JCVI_2790	1.140	moderately similar to (343)AT1G73655| Symbols: | immunophilin / FKBP-type peptidyl-prolyl cis-trans isomerase family protein | chr1:2	
JCVI_31259	1.140	weakly similar to (173)AT4G00270| Symbols: | DNA-binding storekeeper protein-related | chr4:117477-118468 REVERSE no original de	
EX074328	1.140	moderately similar to (366)AT4G32810| Symbols: MAX4, CCD8 | CCD8 (CAROTENOID CLEAVAGE DIOXYGENASE 8) | chr4:15828	
CD829593	1.140	moderately similar to (249)AT5G47670| Symbols: | CCAAT-box binding transcription factor family protein / leafy cotyledon 1-related (L	
JCVI_11581	1.140	weakly similar to (103)AT2G15960| Symbols: | unknown protein | chr2:6954435-6954668 FORWARD no original description	
JCVI_6625	1.140	moderately similar to (304)AT4G18170| Symbols: ATWRKY28, WRKY28 | WRKY28 (WRKY DNA-binding protein 28); transcription fa	
JCVI_31025	1.140	no original description	
JCVI_4097	1.140	moderately similar to (428)AT5G64930| Symbols: HYS1, CPR5 | CPR5 (CONSTITUTIVE EXPRESSION OF PR GENES 5) | chr5:25963	
JCVI_20015	1.140	moderately similar to (390)AT1G10010| Symbols: AAP8 | AAP8 (amino acid permease 8); amino acid transmembrane transporter | chr1:3	
JCVI_6813	1.139	moderately similar to (425)AT1G36730| Symbols: | eukaryotic translation initiation factor 5, putative / eIF-5, putative | chr1:13899918-13	
RC_ES966140	1.139	no similarity	
ES936614	1.139	weakly similar to (108)AT1G11820| Symbols: | hydrolase, hydrolyzing O-glycosyl compounds | chr1:3991258-3993326 REVERSE [1681	
EE513100	1.139	no similarity	
AM392019	1.139	moderately similar to (271)AT3G60850| Symbols: | similar to unnamed protein product [Vitis vinifera] (GB:CAO24486.1) | chr3:2249302	
EV043944	1.138	moderately similar to (249)AT5G14940| Symbols: | proton-dependent oligopeptide transport (POT) family protein | chr5:4831751-483431	
JCVI_8886	1.138	no original description	
EV146043	1.138	moderately similar to (337)AT3G05380| Symbols: | similar to DNA binding / transcription factor [Arabidopsis thaliana] (TAIR:AT5G276	
JCVI_4166	1.138	moderately similar to (204)AT4G32590| Symbols: | ferredoxin-related | chr4:15721423-15722787 FORWARD no original description	
JCVI_809	1.138	moderately similar to (258)AT3G29635| Symbols: | transferase family protein | chr3:11468330-11469706 REVERSE no original descripti	
JCVI_6454	1.138	very weakly similar to (85.5)AT5G33393| Symbols: | unknown protein | chr5:12673660-12675818 REVERSE no original description	
ES967766	1.138	no similarity	
JCVI_36669	1.138	moderately similar to (261)AT2G28540| Symbols: | nucleic acid binding / nucleotide binding / protein binding / zinc ion binding | chr2:12	
JCVI_16107	1.138	weakly similar to (157)AT5G22630| Symbols: ADT5 | ADT5 (AROGENATE DEHYDRATASE 5); arogenate dehydratase/ prephenate de	
JCVI_12516	1.137	moderately similar to (233)AT1G68550| Symbols: | AP2 domain-containing transcription factor, putative | chr1:25729473-25730447 REV	
JCVI_19525	1.137	moderately similar to (313)AT5G61770| Symbols: PPAN | PPAN (PETER PAN-LIKE PROTEIN) | chr5:24833114-24834721 FORWARD	
EV030317	1.137	moderately similar to (266)AT1G67720| Symbols: | leucine-rich repeat family protein / protein kinase family protein | chr1:25390157-253	
JCVI_12653	1.137	weakly similar to (160)AT5G60370| Symbols: | similar to unknown protein [Arabidopsis thaliana] (TAIR:AT3G57110.1); similar to hypot	
EV035510	1.137	no similarity	
EX127162	1.137	weakly similar to (112)AT5G01010| Symbols: | similar to unnamed protein product [Vitis vinifera] (GB:CAO45344.1); contains InterPro	
DY000440	1.137	weakly similar to (166)AT3G10770| Symbols: | nucleic acid binding | chr3:3372466-3374031 REVERSE [18967]	
EV184037	1.137	no similarity	
EE454902	1.137	no similarity	
JCVI_31907	1.136	no original description	-2.995
EE443593	1.136	weakly similar to (146)AT3G20920| Symbols: | translocation protein-related | chr3:7328769-7330704 REVERSE [20160]	
CD827092	1.136	weakly similar to (181)AT3G11890| Symbols: | similar to unknown protein [Arabidopsis thaliana] (TAIR:AT3G11860.1); similar to unna	
JCVI_28554	1.136	moderately similar to (452)AT3G49220| Symbols: | pectinesterase family protein | chr3:18260825-18264632 FORWARDmoderately simil	
EV159630	1.136	no similarity	

JCVI_9451	1.136	moderately similar to (276)AT2G41475| Symbols: | similar to embryo-specific protein-related [Arabidopsis thaliana] (TAIR:AT5G62200.	
JCVI_31362	1.136	highly similar to (692)AT5G16715| Symbols: EMB2247 | EMB2247 (EMBRYO DEFECTIVE 2247); ATP binding / aminoacyl-tRNA liga	
JCVI_1960	1.136	moderately similar to (398)AT5G05080| Symbols: UBC22 | UBC22 (UBIQUITIN-CONJUGATING ENZYME 22) | chr5:1498728-14999	
EX088827	1.136	moderately similar to (332)AT4G11920| Symbols: | WD-40 repeat family protein | chr4:7160612-7163251 REVERSE [21823]	
JCVI_20182	1.136	moderately similar to (258)AT5G62470| Symbols: mybcov1, AtMYB96, MYB96 | MYB96 (myb domain protein 96); DNA binding / trans	
ES968540	1.135	weakly similar to (158)AT5G18500| Symbols: | protein kinase family protein | chr5:6139265-6141285 FORWARD [20153]	1.684
EV089013	1.135	no similarity	
JCVI_15923	1.135	weakly similar to (161)AT5G52300| Symbols: RD29B, LTI65 | LTI65/RD29B (RESPONSIVE TO DESSICATION 29B) | chr5:21254431-	
JCVI_19454	1.135	weakly similar to (144)AT4G38900| Symbols: | bZIP protein | chr4:18139558-18141514 REVERSE no original description	1.512
JCVI_8044	1.135	highly similar to (754)AT5G05010| Symbols: | clathrin adaptor complexes medium subunit-related | chr5:1477138-1479873 FORWARDh	
JCVI_34683	1.135	moderately similar to (246)AT2G34750| Symbols: | RNA polymerase I specific transcription initiation factor RRN3 family protein | chr2:1	-1.402
EV159700	1.134	weakly similar to (131)AT5G62960| Symbols: | similar to unknown protein [Arabidopsis thaliana] (TAIR:AT1G10660.3); similar to unkno	
JCVI_17592	1.134	moderately similar to (340)AT2G14960| Symbols: GH3.1 | GH3.1 | chr2:6458741-6460752 REVERSEmoderately similar to (263)GH38_(	
EV010004	1.134	no similarity	
RC_JCVI_42530	1.134	no original description	
JCVI_23183	1.134	weakly similar to (102)AT1G63000| Symbols: UER1, NRS/ER | NRS/ER (NUCLEOTIDE-RHAMNOSE SYNTHASE/EPIMERASE-RED	
EV077815	1.134	moderately similar to (231)AT3G26310| Symbols: CYP71B35 | CYP71B35 (cytochrome P450, family 71, subfamily B, polypeptide 35); o	
JCVI_19863	1.134	weakly similar to (129)AT3G57340| Symbols: | DNAJ heat shock N-terminal domain-containing protein | chr3:21230153-21231256 FORW	
ES912347	1.134	moderately similar to (318)AT1G50520| Symbols: CYP705A27 | CYP705A27 (cytochrome P450, family 705, subfamily A, polypeptide 27	
EG019252	1.133	no similarity	
JCVI_25130	1.133	no original description	
DN964373	1.133	weakly similar to (185)AT4G34100| Symbols: | similar to protein binding / zinc ion binding [Arabidopsis thaliana] (TAIR:AT4G32670.1);	
JCVI_7667	1.132	weakly similar to (147)AT4G32030| Symbols: | unknown protein | chr4:15490909-15493013 FORWARD no original description	
EV131677	1.132	no similarity	
EX083439	1.132	no similarity	
CB686073	1.132	no similarity	
ES913537	1.132	weakly similar to (133)AT1G62660| Symbols: | beta-fructosidase (BFRUCT3) / beta-fructofuranosidase / invertase, vacuolar | chr1:23203	
JCVI_33941	1.132	moderately similar to (478)AT4G30720| Symbols: | oxidoreductase | chr4:14972218-14975467 REVERSE no original description	
JCVI_40668	1.132	no original description	
ES897684	1.132	no similarity	
JCVI_18483	1.132	weakly similar to (139)AT3G27830| Symbols: RPL12, RPL12-A | RPL12-A (RIBOSOMAL PROTEIN L12-A); structural constituent of ri	
JCVI_2107	1.132	moderately similar to (474)AT5G65205| Symbols: | short-chain dehydrogenase/reductase (SDR) family protein | chr5:26068152-2606924	
EE558854	1.131	no similarity	
JCVI_28184	1.131	weakly similar to (125)AT3G17440| Symbols: ATNPSN13, NPSN13 | NPSN13 (novel plant SNARE 13) | chr3:5970159-5972296 REVER	
EV205882	1.131	no similarity	
JCVI_18674	1.131	moderately similar to (485)AT3G14840| Symbols: | leucine-rich repeat family protein / protein kinase family protein | chr3:4988278-4993	
EE557342	1.131	no similarity	
JCVI_19685	1.131	weakly similar to (191)AT5G39990| Symbols: | glycosyltransferase family 14 protein / core-2/I-branching enzyme family protein | chr5:16	
ES266078	1.130	moderately similar to (218)AT1G05960| Symbols: | binding | chr1:1808477-1815059 REVERSE [21031]	
JCVI_30777	1.130	highly similar to (837)AT1G78900| Symbols: VHA-A | VHA-A; ATP binding / hydrogen ion transporting ATP synthase, rotational mechar	
EV111413	1.130	no similarity	
AM058518	1.130	moderately similar to (383)AT2G36350| Symbols: | protein kinase, putative | chr2:15245982-15248943 FORWARDmoderately similar to	-1.999
EX108780	1.130	weakly similar to (103)AT1G20880| Symbols: | RNA recognition motif (RRM)-containing protein | chr1:7262868-7264823 REVERSE [2	
JCVI_27343	1.130	moderately similar to (270)AT5G37020| Symbols: ARF8 | ARF8 (AUXIN RESPONSE FACTOR 8) | chr5:14647381-14651146 FORWAR	
EV057308	1.129	weakly similar to (159)AT3G06130| Symbols: | heavy-metal-associated domain-containing protein | chr3:1853054-1854679 REVERSE [2	
EE532379	1.129	moderately similar to (234)AT4G26900| Symbols: HISHF, HISN4, AT-HF | AT-HF (ARABIDOPSIS THALIANA HISF PROTEIN) | chr4	
JCVI_26453	1.129	weakly similar to (174)AT1G68960| Symbols: | similar to unknown protein [Arabidopsis thaliana] (TAIR:AT5G03390.1); contains InterPr	
AM058681	1.128	no similarity	
EE509485	1.128	weakly similar to (182)AT2G23450| Symbols: | protein kinase family protein | chr2:9996006-9998324 REVERSE [15718]	
EE547859	1.128	moderately similar to (478)AT2G30950| Symbols: FTSH2, VAR2 | VAR2 (VARIEGATED 2); ATP-dependent peptidase/ ATPase/ metallo	
RC_ES997598	1.128	no similarity	
EE553941	1.127	no similarity	
DY003680	1.127	no similarity	
JCVI_31722	1.127	moderately similar to (488)AT3G55730| Symbols: AtMYB109, MYB109 | MYB109 (myb domain protein 109); DNA binding / transcripti	
CN731695	1.127	no similarity	
ES942036	1.127	moderately similar to (314)AT2G04865| Symbols: | similar to unknown protein [Arabidopsis thaliana] (TAIR:AT2G25010.1); similar to u	
EV065356	1.127	no similarity	
JCVI_20474	1.126	moderately similar to (223)AT1G27190| Symbols: | leucine-rich repeat transmembrane protein kinase, putative | chr1:9446910-9448715 R	1.639
JCVI_1025	1.126	moderately similar to (308)AT5G57040| Symbols: | lactoylglutathione lyase family protein / glyoxalase I family protein | chr5:23101261-2	
JCVI_41851	1.126	moderately similar to (402)AT1G73190| Symbols: ALPHA-TIP, TIP3;1 | ALPHA-TIP/TIP3;1 (ALPHA-TONOPLAST INTRINSIC PROT	
JCVI_1045	1.126	moderately similar to (302)AT5G16250| Symbols: | similar to unknown protein [Arabidopsis thaliana] (TAIR:AT3G02640.1); similar to u	
JCVI_12015	1.126	weakly similar to (180)AT4G13230| Symbols: | late embryogenesis abundant domain-containing protein / LEA domain-containing protein	
EV224011	1.126	weakly similar to (167)AT5G60600| Symbols: ISPG, CSB3, CLB4, GcpE | GcpE (CHLOROPLAST BIOGENESIS 4) | chr5:24376673-243	
JCVI_31856	1.126	weakly similar to (197)AT1G23150| Symbols: | similar to unknown protein [Arabidopsis thaliana] (TAIR:AT1G70780.1); similar to unnar	
JCVI_37185	1.126	moderately similar to (307)AT2G21710| Symbols: EMB2219 | EMB2219 (EMBRYO DEFECTIVE 2219) | chr2:9277966-9280387 FORW	
JCVI_3569	1.126	moderately similar to (481)AT5G41060| Symbols: | zinc finger (DHHC type) family protein | chr5:16453177-16454768 FORWARD no or	
JCVI_7526	1.126	moderately similar to (442)AT1G52400| Symbols: BGL1 | BGL1 (BETA-GLUCOSIDASE HOMOLOG 1); hydrolase, hydrolyzing O-glyc	
JCVI_40449	1.125	moderately similar to (438)AT1G17460| Symbols: TRFL3 | TRFL3 (TRF-LIKE 3); DNA binding / transcription factor | chr1:5999512-600	
ES947863	1.125	weakly similar to (152)AT5G26830| Symbols: | threonyl-tRNA synthetase / threonine--tRNA ligase (THRRS) | chr5:9437354-9441571 FC	
JCVI_28678	1.125	nearly identical (1113)AT3G59020| Symbols: | protein transporter | chr3:21821950-21828395 REVERSE no original description	
EV041242	1.125	weakly similar to (104)AT1G07410| Symbols: AtRABA2b | AtRABA2b (Arabidopsis Rab GTPase homolog A2b); GTP binding | chr1:227	
EE564549	1.125	no similarity	
JCVI_42389	1.125	moderately similar to (233)AT2G24200| Symbols: | cytosol aminopeptidase | chr2:10294097-10296530 REVERSEweakly similar to (184	
DY025423	1.125	moderately similar to (236)AT4G17810| Symbols: | nucleic acid binding / transcription factor/ zinc ion binding | chr4:9906931-9907545 F	
AM387297	1.125	no similarity	
EE440987	1.125	moderately similar to (342)AT1G71070| Symbols: | glycosyltransferase family 14 protein / core-2/I-branching enzyme family protein | chr	
JCVI_18455	1.125	highly similar to (716)AT1G09440| Symbols: | protein kinase family protein | chr1:3045515-3047395 REVERSEmoderately similar to (23	
JCVI_39345	1.125	highly similar to (566)AT3G14120| Symbols: | similar to hypothetical protein OsJ_033267 [Oryza sativa (japonica cultivar-group)] (GB:E	
EX092678	1.125	moderately similar to (244)AT2G33750| Symbols: ATPUP2 | ATPUP2 (Arabidopsis thaliana purine permease 2); purine transmembrane tr	
JCVI_2128	1.125	moderately similar to (246)AT5G48580| Symbols: FKBP15-2 | FKBP15-2 (FK506-binding protein 15 kD-2); FK506 binding / peptidyl-pro	
JCVI_9722	1.124	highly similar to (690)AT4G19880| Symbols: | similar to unknown protein [Arabidopsis thaliana] (TAIR:AT5G45020.1); similar to Intrac	

JCVI_4854	1.124	highly similar to (694)AT5G05170| Symbols: CESA3, IXR1, ATCESA3, ATH-B, CEV1 | CESA3 (CELLULOSE SYNTHASE 3); cellulos	
EV051281	1.124	moderately similar to (404)AT4G03550| Symbols: GSL05, ATGSL5, PMR4, GSL5, ATGSL05 | ATGSL05 (GLUCAN SYNTHASE-LIKE	
JCVI_12361	1.124	moderately similar to (424)AT4G24670| Symbols: | alliinase family protein | chr4:12727950-12730704 REVERSE no original description	
JCVI_24203	1.124	moderately similar to (350)AT4G39550| Symbols: | kelch repeat-containing F-box family protein | chr4:18380675-18381853 REVERSE n	
EV104183	1.124	moderately similar to (404)AT1G78580| Symbols: TPS1, ATTPS1 | ATTPS1 (TREHALOSE-6-PHOSPHATE SYNTHASE); transferase, t	
CV432635	1.124	weakly similar to (134)AT5G09870| Symbols: CESA5 | CESA5 (CELLULOSE SYNTHASE 5); transferase, transferring glycosyl groups |	
EX064845	1.123	weakly similar to (112)AT2G31500| Symbols: CPK24 | CPK24 (calcium-dependent protein kinase 24); calmodulin-dependent protein kina	
RC_ES963559	1.123	no similarity	
ES962131	1.123	no similarity	
EH414215	1.123	very weakly similar to (84.0)AT5G42030| Symbols: ABIL4 | ABIL4 (ABL INTERACTOR-LIKE PROTEIN 4) | chr5:16828746-16830315	
ES912981	1.123	moderately similar to (204)AT4G34430| Symbols: ATSWI3D, CHB3 | CHB3 (Arabidopsis thaliana switch 3D); DNA binding / transcriptio	
JCVI_15578	1.123	moderately similar to (365)AT1G53000| Symbols: | cytidylyltransferase family | chr1:19748998-19750801 REVERSE no original descript	
EG020774	1.123	weakly similar to (194)AT2G02230| Symbols: ATPP2-B1 | ATPP2-B1 (Phloem protein 2-B1); carbohydrate binding | chr2:590392-591594	
ES905471	1.123	moderately similar to (213)AT1G32240| Symbols: KAN2 | KAN2 (KANADI 2); DNA binding / transcription factor | chr1:11625862-1163	
JCVI_2047	1.122	weakly similar to (104)AT1G60870| Symbols: MEE9 | MEE9 (maternal effect embryo arrest 9) | chr1:22413377-22413892 FORWARD no	
JCVI_28082	1.122	moderately similar to (481)AT5G24380| Symbols: ATYSL2, YSL2 | YSL2 (YELLOW STRIPE LIKE 2); oligopeptide transporter | chr5:83	
JCVI_38922	1.122	weakly similar to (130)AT5G11460| Symbols: | senescence-associated protein-related | chr5:3657065-3658389 REVERSE no original des	
JCVI_31846	1.122	no original description	
ES946059	1.122	moderately similar to (234)AT2G41520| Symbols: | DNAJ heat shock N-terminal domain-containing protein | chr2:17324740-17329783 F	-1.939
JCVI_2751	1.122	moderately similar to (246)AT1G68490| Symbols: | similar to unknown protein [Arabidopsis thaliana] (TAIR:AT1G13390.2); similar to u	-1.570
EE554705	1.122	no similarity	
EE521994	1.122	weakly similar to (156)AT5G18660| Symbols: PCB2, DVR | DVR (PALE-GREEN AND CHLOROPHYLL B REDUCED 2); 3,8-divinyl p	
JCVI_9245	1.122	moderately similar to (469)AT3G13410| Symbols: | similar to unknown protein [Arabidopsis thaliana] (TAIR:AT1G55546.1); similar to h	1.680
EV168047	1.122	no similarity	
JCVI_19255	1.121	highly similar to (724)AT5G23580| Symbols: ATCDPK9, CPK12, CDPK9 | CDPK9 (CALCIUM-DEPENDENT PROTEIN KINASE 9); c	
EX138385	1.121	no similarity	
JCVI_7749	1.121	moderately similar to (316)AT5G13780| Symbols: | GCN5-related N-acetyltransferase, putative | chr5:4447613-4448606 REVERSE no or	
JCVI_37627	1.121	no original description	-1.317
EV009274	1.120	no similarity	
JCVI_21019	1.120	weakly similar to (159)AT1G11650| Symbols: ATRBP45B | ATRBP45B; RNA binding | chr1:3914895-3917301 FORWARD no original c	
JCVI_27771	1.120	no original description	
EE478101	1.120	no similarity	
JCVI_5555	1.119	moderately similar to (351)AT5G65950| Symbols: | binding | chr5:26397516-26402186 FORWARD no original description	
JCVI_39397	1.119	highly similar to (720)AT2G44180| Symbols: MAP2A | MAP2A (METHIONINE AMINOPEPTIDASE 2A); methionyl aminopeptidase | c	
JCVI_3912	1.119	no original description	
JCVI_18212	1.119	moderately similar to (255)AT1G52410| Symbols: TSA1 | TSA1 (TSK-ASSOCIATING PROTEIN 1) | chr1:19524431-19529030 FORWA	
AM394457	1.119	no similarity	
JCVI_17585	1.119	highly similar to (548)AT4G16660| Symbols: | heat shock protein 70, putative / HSP70, putative | chr4:9377247-9381254 FORWARDwea	1.538
JCVI_27408	1.119	weakly similar to (161)AT4G39300| Symbols: | similar to unknown protein [Arabidopsis thaliana] (TAIR:AT3G13674.1); similar to unkno	
JCVI_27336	1.119	moderately similar to (486)AT1G78570| Symbols: RHM1, ROL1 | RHM1/ROL1 (RHAMNOSE BIOSYNTHESIS1); UDP-L-rhamnose sy	
JCVI_33998	1.118	no original description	
JCVI_35970	1.118	moderately similar to (355)AT5G11300| Symbols: CYC2BAT, CYCA2;2, CYC3B | CYC3B (MITOTIC-LIKE CYCLIN 3B FROM ARAB	
EE482899	1.118	very weakly similar to (80.9)AT2G24220| Symbols: ATPUP5 | ATPUP5 (Arabidopsis thaliana purine permease 5); purine transmembrane t	
EV001893	1.118	weakly similar to (158)AT3G03790| Symbols: | ankyrin repeat family protein / regulator of chromosome condensation (RCC1) family prot	
EE519328	1.118	moderately similar to (238)AT4G30570| Symbols: | GDP-mannose pyrophosphorylase, putative | chr4:14930712-14931957 REVERSE [2	
JCVI_26279	1.118	moderately similar to (427)AT1G13780| Symbols: | Identical to F-box/FBD/LRR-repeat protein At1g13780 [Arabidopsis Thaliana] (GB:C	
CA991987	1.118	very weakly similar to (90.9)AT1G51880| Symbols: | leucine-rich repeat protein kinase, putative | chr1:19273862-19277737 REVERSE [1	
JCVI_6593	1.118	moderately similar to (276)AT3G50685| Symbols: | similar to unnamed protein product [Vitis vinifera] (GB:CAO22868.1) | chr3:1884525	
EV024664	1.118	moderately similar to (271)AT5G60820| Symbols: | zinc finger (C3HC4-type RING finger) family protein | chr5:24486862-24488121 FOI	
JCVI_35766	1.118	moderately similar to (259)AT1G26761| Symbols: | similar to unnamed protein product [Vitis vinifera] (GB:CAO68448.1); contains doma	
EE424718	1.118	weakly similar to (131)AT4G35785| Symbols: | transformer serine/arginine-rich ribonucleoprotein, putative | chr4:16953989-16955131 RI	
DY023978	1.118	moderately similar to (220)AT3G27010| Symbols: PCF1, AT-TCP20 | AT-TCP20 (ARABIDOPSIS THALIANA TEOSINTE BRANCHEI	1.735
EV028461	1.117	moderately similar to (217)AT3G62770| Symbols: AtATG18a | AtATG18a (Arabidopsis thaliana homolog of yeast autophagy 18 (ATG18)	
EX131869	1.117	no similarity	
EE561370	1.117	moderately similar to (256)AT3G51160| Symbols: MUR_1, GMD2, MUR1 | MUR1 (MURUS 1) | chr3:19018211-19019332 REVERSE [2	
JCVI_20257	1.117	moderately similar to (326)AT1G32260| Symbols: | similar to unknown protein [Arabidopsis thaliana] (TAIR:AT2G35480.1); similar to u	
JCVI_17343	1.117	moderately similar to (275)AT1G60190| Symbols: | armadillo/beta-catenin repeat family protein / U-box domain-containing protein | chr1	
EV161793	1.116	weakly similar to (143)AT5G63020| Symbols: | disease resistance protein (CC-NBS-LRR class), putative | chr5:25300478-25303228 REV	
JCVI_38483	1.116	moderately similar to (369)AT3G21480| Symbols: | similar to BRCT domain-containing protein [Arabidopsis thaliana] (TAIR:AT4G0313	
EX115272	1.116	no similarity	
EV100925	1.116	no similarity	
ES902368	1.116	moderately similar to (272)AT4G19160| Symbols: | binding | chr4:10477532-10479958 FORWARD [21428]	
JCVI_11857	1.116	moderately similar to (464)AT1G62750| Symbols: ATSCO1, ATSCO1/CPEF-G, SCO1 | ATSCO1/ATSCO1/CPEF-G/SCO1 (SNOWY CO	
EV068703	1.116	no similarity	
JCVI_21569	1.116	weakly similar to (103)AT4G20095| Symbols: | similar to unknown protein [Arabidopsis thaliana] (TAIR:AT4G29550.1); contains InterPr	1.374
JCVI_20435	1.116	moderately similar to (369)AT4G28706| Symbols: | pfkB-type carbohydrate kinase family protein | chr4:14167811-14170625 FORWARD	
DY023734	1.116	moderately similar to (299)AT1G14440| Symbols: ATHB31 | ATHB31 (ARABIDOPSIS THALIANA HOMEOBOX PROTEIN 31); trans	
JCVI_2811	1.116	weakly similar to (171)AT4G17670| Symbols: | senescence-associated protein-related | chr4:9833961-9834676 REVERSE no original desc	
EV148852	1.115	no similarity	
EX117943	1.115	moderately similar to (329)AT1G58110| Symbols: | bZIP family transcription factor | chr1:21519584-21521039 REVERSE [21828]	
ES998511	1.115	weakly similar to (184)AT2G35540| Symbols: | DNAJ heat shock N-terminal domain-containing protein | chr2:14934237-14936009 FORV	
JCVI_11747	1.115	weakly similar to (192)AT4G32400| Symbols: SHS1 | SHS1 (SODIUM HYPERSENSITIVE 1); binding / transporter | chr4:15638692-156	
JCVI_17304	1.115	highly similar to (778)AT1G05840| Symbols: | aspartyl protease family protein | chr1:1762842-1766149 REVERSEvery weakly similar to	
JCVI_30105	1.115	no original description	
EV212910	1.115	moderately similar to (409)AT1G73430| Symbols: | sec34-like family protein | chr1:27607757-27614490 FORWARD [21491]	
JCVI_34648	1.115	moderately similar to (375)AT5G65687| Symbols: | transporter-related | chr5:26287264-26290013 REVERSE no original description	
JCVI_19047	1.115	moderately similar to (223)AT1G47510| Symbols: | endonuclease/exonuclease/phosphatase family protein | chr1:17438431-17440736 RE	
JCVI_33529	1.114	moderately similar to (306)AT5G26860| Symbols: LON_ARA_ARA | LON_ARA_ARA (Lon protease homolog gene); ATP binding / ATI	
EV079305	1.114	weakly similar to (129)AT3G26700| Symbols: | protein kinase family protein | chr3:9811906-9813593 FORWARD [21443]	
EE523574	1.114	no similarity	
EV140615	1.114	moderately similar to (454)AT2G31320| Symbols: | poly (ADP-ribose) polymerase, putative / NAD(+) ADP-ribosyltransferase, putative /	

JCVI_9703	1.113	moderately similar to (355)AT3G51850| Symbols: CPK13 | CPK13 (calcium-dependent protein kinase 13); calmodulin-dependent protein	
JCVI_28455	1.113	no original description	
EV144831	1.113	no similarity	
CO749567	1.113	no similarity	
ES968621	1.113	no similarity	
EE532937	1.112	no similarity	
CD836625	1.112	moderately similar to (224)AT5G19600| Symbols: SULTR3;5 | SULTR3;5; sulfate transmembrane transporter | chr5:6613115-6616893 RI	
JCVI_3375	1.112	moderately similar to (421)AT5G47330| Symbols: | palmitoyl protein thioesterase family protein | chr5:19224397-19225938 FORWARD	
JCVI_6342	1.112	weakly similar to (194)AT1G19380| Symbols: | similar to unknown protein [Arabidopsis thaliana] (TAIR:AT5G65650.1); similar to unkno	
JCVI_37835	1.112	weakly similar to (181)AT3G13670| Symbols: | protein kinase family protein | chr3:4469441-4473241 FORWARD no original description	
JCVI_21824	1.112	no original description	
EX045913	1.112	no similarity	-1.493
JCVI_41025	1.112	moderately similar to (220)AT5G62260| Symbols: | DNA binding | chr5:25026557-25028574 FORWARD no original description	
EE560455	1.112	no similarity	
BG543219	1.112	moderately similar to (265)AT3G07090| Symbols: | similar to unknown protein [Arabidopsis thaliana] (TAIR:AT5G25170.1); similar to u	
JCVI_15756	1.112	moderately similar to (500)AT3G29320| Symbols: | glucan phosphorylase, putative | chr3:11254108-11258824 FORWARDmoderately sir	
JCVI_35198	1.112	no original description	
EG020723	1.112	no similarity	
JCVI_783	1.111	moderately similar to (285)AT4G10030| Symbols: | hydrolase, alpha/beta fold family protein | chr4:6270487-6273095 REVERSE no origi	
EE418602	1.111	weakly similar to (163)AT2G30700| Symbols: | similar to unknown protein [Arabidopsis thaliana] (TAIR:AT1G61900.1); similar to hypot	
JCVI_19249	1.111	moderately similar to (367)AT3G59180| Symbols: | similar to F-box family protein [Arabidopsis thaliana] (TAIR:AT3G59210.1); similar	
JCVI_40637	1.111	weakly similar to (170)AT1G53240| Symbols: | malate dehydrogenase (NAD), mitochondrial | chr1:19858634-19860470 REVERSEweakl	
JCVI_7348	1.111	weakly similar to (185)AT1G09910| Symbols: | lyase | chr1:3220153-3224453 REVERSE no original description	
EE432213	1.111	weakly similar to (170)AT2G37770| Symbols: | aldo/keto reductase family protein | chr2:15841966-15843737 FORWARDvery weakly sin	
EE538308	1.111	weakly similar to (128)AT4G21192| Symbols: | similar to unnamed protein product [Vitis vinifera] (GB:CAO46886.1); contains InterPro	
JCVI_15703	1.111	moderately similar to (246)AT5G15760| Symbols: | plastid-specific 30S ribosomal protein 3, putative / PSRP-3, putative | chr5:5143103-5	
JCVI_6943	1.110	weakly similar to (177)AT1G23860| Symbols: RSZP21, SRZ21, SRZ-21 | SRZ-21 | chr1:8428080-8429153 REVERSE no original descript	
JCVI_20421	1.110	moderately similar to (273)AT5G51910| Symbols: | TCP family transcription factor, putative | chr5:21111930-21112811 REVERSE no or	
ES967267	1.110	very weakly similar to (89.0)AT1G64220| Symbols: TOM7-2 | TOM7-2 (TRANSLOCASE OF OUTER MEMBRANE 7 KDA SUBUNIT 2	
JCVI_33596	1.110	weakly similar to (180)AT4G22320| Symbols: | similar to unknown protein [Arabidopsis thaliana] (TAIR:AT5G55210.1); similar to unnar	
EE451176	1.110	moderately similar to (420)AT3G12150| Symbols: | similar to hypothetical protein [Vitis vinifera] (GB:CAN81264.1); contains domain P	
JCVI_5687	1.110	highly similar to (842)AT5G60920| Symbols: COB | COB (COBRA) | chr5:24528692-24531158 REVERSEhighly similar to (658)COBL1	
EV109726	1.110	very weakly similar to (95.1)AT2G19570| Symbols: AT-CDA1, DESZ, CDA1 | CDA1 (CYTIDINE DEAMINASE 1) | chr2:8477680-8478	1.152
JCVI_9093	1.110	moderately similar to (224)AT3G24610| Symbols: | kelch repeat-containing F-box family protein | chr3:8975373-8978109 REVERSE no c	
JCVI_9120	1.109	moderately similar to (251)AT1G31730| Symbols: | epsilon-adaptin, putative | chr1:11359888-11363897 FORWARD no original descripti	
JCVI_39070	1.109	no original description	-1.577
CO750244	1.109	no similarity	
EV066486	1.109	moderately similar to (407)AT3G50940| Symbols: | AAA-type ATPase family protein | chr3:18945067-18946509 FORWARD [21443]	
EV028136	1.109	moderately similar to (278)AT5G15260| Symbols: | structural constituent of ribosome | chr5:4953649-4954353 REVERSE [21441]	
EV192328	1.109	moderately similar to (290)AT2G02070| Symbols: ATIDD5 | ATIDD5 (ARABIDOPSIS THALIANA INDETERMINATE(ID)-DOMAIN	
JCVI_24589	1.109	moderately similar to (306)AT5G04820| Symbols: ATOFP13, OFP13 | ATOFP13/OFP13 (Arabidopsis thaliana ovate family protein 13) | c	1.425
EV158802	1.109	weakly similar to (149)AT2G26730| Symbols: | leucine-rich repeat transmembrane protein kinase, putative | chr2:11395699-11398364 FO	
EE465832	1.108	moderately similar to (307)AT3G60740| Symbols: EMB133, TFC D, CHO, TTN1 | TTN1 (TITAN 1) | chr3:22458360-22464300 REVERS	
JCVI_22841	1.108	moderately similar to (373)AT4G21540| Symbols: | diacylglycerol kinase | chr4:11460715-11462368 FORWARD no original description	
JCVI_28739	1.108	moderately similar to (268)AT2G15220| Symbols: | secretory protein, putative | chr2:6615771-6616448 FORWARD no original descriptic	
JCVI_25466	1.108	highly similar to (675)AT1G08230| Symbols: | amino acid transporter family protein | chr1:2583712-2586697 REVERSE no original desc	1.957
EV096343	1.108	weakly similar to (119)AT2G37770| Symbols: | aldo/keto reductase family protein | chr2:15841966-15843737 FORWARD [21476]	1.967
EX132622	1.108	moderately similar to (377)AT3G10250| Symbols: | similar to unknown protein [Arabidopsis thaliana] (TAIR:AT5G04090.2); similar to u	
JCVI_23858	1.108	weakly similar to (149)AT1G30845| Symbols: | similar to unnamed protein product [Vitis vinifera] (GB:CAO61037.1); contains domain M	
JCVI_32328	1.108	moderately similar to (302)AT3G55030| Symbols: PGPS2 | PGPS2 (phosphatidylglycerolphosphate synthase 2); CDP-alcohol phosphatidy	
JCVI_38785	1.108	no original description	1.181
JCVI_33324	1.108	weakly similar to (156)AT1G53510| Symbols: ATMPK18 | ATMPK18 (ARABIDOPSIS THALIANA MAP KINASE 18); MAP kinase | cl	
JCVI_13831	1.107	moderately similar to (308)AT1G23740| Symbols: | oxidoreductase, zinc-binding dehydrogenase family protein | chr1:8398234-8399645 F	
EX057878	1.107	moderately similar to (360)AT5G26742| Symbols: EMB1138 | EMB1138 (EMBRYO DEFECTIVE 1138); ATP binding / ATP-dependent	
JCVI_2090	1.107	moderately similar to (308)AT1G67360| Symbols: | rubber elongation factor (REF) family protein | chr1:25240735-25241576 REVERSEv	
EV176702	1.107	moderately similar to (265)AT1G61380| Symbols: | S-locus protein kinase, putative | chr1:22649942-22653066 REVERSE [21487] 128 1(	
JCVI_14823	1.106	highly similar to (618)AT4G34890| Symbols: ATXDH1 | ATXDH1 (XANTHINE DEHYDROGENASE 1); xanthine dehydrogenase | chr4	
CX272027	1.106	moderately similar to (202)AT5G07270| Symbols: | ankyrin repeat family protein | chr5:2280822-2283385 FORWARD [16815]	
EE525458	1.106	moderately similar to (258)AT2G40480| Symbols: | similar to unknown protein [Arabidopsis thaliana] (TAIR:AT3G56270.1); similar to u	
JCVI_5081	1.106	moderately similar to (266)AT3G56490| Symbols: | zinc-binding protein, putative / protein kinase C inhibitor, putative | chr3:20952511-2(	
AM059057	1.106	moderately similar to (291)AT2G36960| Symbols: TKI1 | TKI1 (TSL-KINASE INTERACTING PROTEIN 1); DNA binding / transcriptic	
EE414457	1.106	weakly similar to (139)AT1G71940| Symbols: | similar to unknown protein [Arabidopsis thaliana] (TAIR:AT4G09580.1); similar to unnar	
EV193321	1.105	weakly similar to (134)AT2G41330| Symbols: | glutaredoxin family protein | chr2:17234145-17235353 FORWARD [21489] 30 720 720	
JCVI_33680	1.105	no original description	
JCVI_39619	1.105	moderately similar to (214)AT4G17570| Symbols: | zinc finger (GATA type) family protein | chr4:9784342-9786987 REVERSE no origin	
EE442656	1.105	moderately similar to (275)AT2G42450| Symbols: | lipase class 3 family protein | chr2:17679503-17681932 REVERSE [20164]	
JCVI_19372	1.105	moderately similar to (272)AT3G55970| Symbols: | oxidoreductase, 2OG-Fe(II) oxygenase family protein | chr3:20777949-20780243 RE\	-1.193
CX189502	1.105	no similarity	
JCVI_5675	1.105	moderately similar to (318)AT1G24360| Symbols: | 3-oxoacyl-(acyl-carrier protein) reductase, chloroplast / 3-ketoacyl-acyl carrier proteir	
JCVI_6999	1.105	moderately similar to (429)AT1G70160| Symbols: | similar to unknown protein [Arabidopsis thaliana] (TAIR:AT5G54870.1); similar to u	
JCVI_39854	1.104	moderately similar to (379)AT5G44635| Symbols: | minichromosome maintenance family protein / MCM family protein | chr5:18023658-	
EV015221	1.104	no similarity	
JCVI_20311	1.104	weakly similar to (157)AT4G23250| Symbols: EMB1290 | EMB1290 (EMBRYO DEFECTIVE 1290); kinase | chr4:12162014-12167036 I	
JCVI_13070	1.104	no original description	
JCVI_29510	1.104	moderately similar to (437)AT1G28350| Symbols: | ATP binding / aminoacyl-tRNA ligase | chr1:9944470-9949564 FORWARD no origin	
JCVI_25917	1.104	weakly similar to (199)AT3G43590| Symbols: | zinc knuckle (CCHC-type) family protein | chr3:15520741-15523243 FORWARD no orig	
EX141344	1.104	no similarity	
EE529458	1.103	weakly similar to (104)AT5G58470| Symbols: TAF15b | TAF15b (TBP-ASSOCIATED FACTOR 15b); binding | chr5:23655792-2365808	
ES998935	1.103	moderately similar to (232)AT1G61210| Symbols: | WD-40 repeat family protein / katanin p80 subunit, putative | chr1:22568450-2257522	
JCVI_30494	1.103	no original description	
ES264476	1.103	no similarity	

EE471375	1.103	weakly similar to (112)AT3G50990| Symbols: | peroxidase | chr3:18954136-18955586 FORWARDvery weakly similar to (81.6)PERP7_E	
EX119916	1.103	moderately similar to (285)AT2G43770| Symbols: | transducin family protein / WD-40 repeat family protein | chr2:18141349-18142380 R	
ES931219	1.103	moderately similar to (298)AT3G23280| Symbols: | zinc finger (C3HC4-type RING finger) family protein / ankyrin repeat family protein |	
JCVI_34495	1.103	moderately similar to (462)AT1G14850| Symbols: NUP155 | NUP155 (Nucleoporin 155) | chr1:5116916-5123254 REVERSE no original	
JCVI_18953	1.102	moderately similar to (254)AT4G29360| Symbols: | glycosyl hydrolase family 17 protein | chr4:14451568-14453693 REVERSEweakly sir	
JCVI_17504	1.102	moderately similar to (223)AT2G43590| Symbols: | chitinase, putative | chr2:18088669-18089826 REVERSEmoderately similar to (228)	
JCVI_19290	1.102	no original description	
ES904191	1.102	moderately similar to (346)AT5G04940| Symbols: SUVH1 | SUVH1 (SU(VAR)3-9 HOMOLOG 1) | chr5:1454617-1456629 REVERSE [2	
JCVI_16406	1.102	moderately similar to (384)AT4G34570| Symbols: THY-2 | THY-2 (THYMIDYLATE SYNTHASE 2) | chr4:16511134-16514115 REVEF	
JCVI_38337	1.102	moderately similar to (353)AT1G26850| Symbols: | dehydration-responsive family protein | chr1:9301133-9303419 REVERSE no origina	
RC_EX122808	1.102	no similarity	-2.071
JCVI_33851	1.102	moderately similar to (265)AT1G77680| Symbols: | ribonuclease II family protein | chr1:29197082-29200857 REVERSE no original desc	
EV201739	1.102	no similarity	
EV089744	1.102	weakly similar to (176)AT4G03820| Symbols: | similar to unknown protein [Arabidopsis thaliana] (TAIR:AT4G22270.1); similar to unnar	
ES264273	1.102	moderately similar to (280)AT5G24940| Symbols: | protein phosphatase 2C, putative / PP2C, putative | chr5:8591410-8593604 REVERSI	
JCVI_40679	1.102	very weakly similar to (91.7)AT5G63370| Symbols: | protein kinase family protein | chr5:25402180-25403616 REVERSE no original desc	
EX063406	1.101	no similarity	
EV214476	1.101	weakly similar to (176)AT5G39600| Symbols: | similar to unknown [Populus trichocarpa] (GB:ABK93996.1); similar to unnamed protein	
JCVI_8763	1.101	no original description	
CV545274	1.101	weakly similar to (133)AT3G20570| Symbols: | plastocyanin-like domain-containing protein | chr3:7186760-7187459 REVERSE [16551]	
JCVI_18328	1.101	highly similar to (672)AT1G30820| Symbols: | CTP synthase, putative / UTP--ammonia ligase, putative | chr1:10945232-10948806 REVE	
JCVI_25291	1.101	moderately similar to (404)AT5G67140| Symbols: | F-box family protein | chr5:26811235-26812439 REVERSE no original description	
ES947274	1.101	weakly similar to (170)AT3G19470| Symbols: | F-box family protein | chr3:6749772-6750920 FORWARD [21393]	
RC_AT000963	1.101	no similarity	
EV129673	1.101	weakly similar to (117)AT3G62070| Symbols: | similar to unknown protein [Arabidopsis thaliana] (TAIR:AT2G46940.1); similar to unnar	
JCVI_35698	1.100	very weakly similar to (80.9)AT4G30960| Symbols: SIP3, SNRK3.14, CIPK6 | CIPK6 (CBL-INTERACTING PROTEIN KINASE 6); kina	
EV076567	1.100	weakly similar to (162)AT4G16690| Symbols: | esterase/lipase/thioesterase family protein | chr4:9392427-9393446 REVERSE [21443]	-1.395
AI352723	1.100	weakly similar to (168)AT5G64850| Symbols: | similar to unknown protein [Arabidopsis thaliana] (TAIR:AT5G09960.1); similar to 80C0	
JCVI_40785	1.100	moderately similar to (451)AT4G24740| Symbols: AME1, AFC2 | AFC2 (ARABIDOPSIS FUS3-COMPLEMENTING GENE 1); kinase |	
JCVI_1598	1.099	moderately similar to (479)AT4G20130| Symbols: PTAC14 | PTAC14 (PLASTID TRANSCRIPTIONALLY ACTIVE14) | chr4:10878924	
JCVI_28551	1.099	no original description	
EV129875	1.099	weakly similar to (147)AT4G04210| Symbols: PUX4 | PUX4 (PLANT UBX DOMAIN-CONTAINING PROTEIN 4) | chr4:2030389-2031	
EV149387	1.099	no similarity	
EE564160	1.099	no similarity	
JCVI_21521	1.099	moderately similar to (357)AT4G17650| Symbols: | aromatic-rich family protein | chr4:9827762-9829456 FORWARD no original descrip	
ES959055	1.099	weakly similar to (148)AT4G27020| Symbols: | similar to unknown protein [Arabidopsis thaliana] (TAIR:AT1G70160.1); similar to unkn	
EE473382	1.099	no similarity	
ES930171	1.098	no similarity	
EV179031	1.098	weakly similar to (200)AT5G39110| Symbols: | germin-like protein, putative | chr5:15675030-15675812 REVERSEweakly similar to (10	
EV106492	1.098	weakly similar to (144)AT3G07470| Symbols: | similar to unknown protein [Arabidopsis thaliana] (TAIR:AT3G07460.1); similar to unnar	
EV135273	1.098	moderately similar to (251)AT4G28680| Symbols: | tyrosine decarboxylase, putative | chr4:14155254-14158552 FORWARDweakly simil	
JCVI_341	1.098	highly similar to (741)AT5G26180| Symbols: | NOL1/NOP2/sun family protein | chr5:9149256-9152598 FORWARD no original descripti	
AM396131	1.098	no similarity	
JCVI_5617	1.098	weakly similar to (147)AT5G67070| Symbols: RALFL34 | RALFL34 (RALF-LIKE 34) | chr5:26785346-26785735 FORWARD no origina	
ES932868	1.098	moderately similar to (207)AT3G61670| Symbols: | similar to unknown protein [Arabidopsis thaliana] (TAIR:AT2G46380.1); similar to u	
EX105810	1.098	no similarity	
JCVI_25289	1.098	moderately similar to (435)AT3G01140| Symbols: AtMYB106, MYB106 | MYB106 (myb domain protein 106); DNA binding / transcripti	
JCVI_13396	1.098	moderately similar to (283)AT5G43320| Symbols: CKL8 | CKL8 (Casein Kinase I-like 8); casein kinase I/ kinase | chr5:17403271-174061	
JCVI_8081	1.097	moderately similar to (278)AT5G18380| Symbols: | 40S ribosomal protein S16 (RPS16C) | chr5:6090130-6090695 REVERSEmoderately	
JCVI_18167	1.097	moderately similar to (245)AT1G05300| Symbols: ZIP5 | ZIP5 (ZINC TRANSPORTER 5 PRECURSOR); cation transmembrane transpor	
EE440720	1.097	weakly similar to (116)AT2G39110| Symbols: | protein kinase, putative | chr2:16326848-16328646 FORWARD [20167]	
JCVI_13212	1.097	moderately similar to (288)AT1G67680| Symbols: | 7S RNA binding | chr1:25369625-25372127 REVERSE no original description	
JCVI_8884	1.097	highly similar to (586)AT4G30210| Symbols: AR2, ATR2 | ATR2 (ARABIDOPSIS P450 REDUCTASE 2) | chr4:14796906-14800584 FO	
JCVI_34098	1.097	no original description	
JCVI_37099	1.097	no original description	
EV103062	1.097	moderately similar to (239)AT3G13360| Symbols: WIP3 | WIP3 (WPP-DOMAIN INTERACTING PROTEIN 3) | chr3:4338479-4339989	
EE455720	1.097	weakly similar to (118)AT4G36420| Symbols: | ribosomal protein L12 family protein | chr4:17203721-17204260 REVERSE [20178]	
ES266017	1.097	weakly similar to (123)AT1G53290| Symbols: | galactosyltransferase family protein | chr1:19875021-19876919 FORWARD [21031]	
AM395470	1.097	weakly similar to (186)AT5G10690| Symbols: | pentatricopeptide (PPR) repeat-containing protein / CBS domain-containing protein | chr5	
JCVI_26763	1.097	moderately similar to (261)AT4G22360| Symbols: | SWIB complex BAF60b domain-containing protein | chr4:11807823-11809672 FORV	
JCVI_4337	1.097	weakly similar to (127)AT5G09920| Symbols: ATRPB15.9, RPB15.9.9, RPB15.9 | RPB15.9 (RNA polymerase II 15.9) | chr5:3096277-30	-1.270
AM389512	1.096	weakly similar to (171)AT3G24580| Symbols: | F-box family protein | chr3:8969169-8970305 FORWARD [20118] 1 774 787	
JCVI_5335	1.096	moderately similar to (375)AT3G11800| Symbols: | similar to unknown protein [Arabidopsis thaliana] (TAIR:AT3G44150.1); similar to e	
JCVI_21054	1.096	highly similar to (939)AT1G12360| Symbols: KEU | KEU (KEULE); protein transporter | chr1:4201170-4206142 FORWARDhighly simil	
EX063432	1.096	weakly similar to (157)AT4G21530| Symbols: | nucleotide binding | chr4:11450755-11455874 FORWARD [21814]	
JCVI_29481	1.096	moderately similar to (293)AT4G31390| Symbols: | ABC1 family protein | chr4:15233132-15236770 FORWARD no original description	
JCVI_11782	1.096	moderately similar to (402)AT5G24800| Symbols: ATBZIP9, BZO2H2 | ATBZIP9/BZO2H2 (BASIC LEUCINE ZIPPER O2 HOMOLOG	
JCVI_18350	1.096	highly similar to (554)AT2G35780| Symbols: SCPL26 | SCPL26 (serine carboxypeptidase-like 26); serine carboxypeptidase | chr2:150448	
EE444258	1.095	weakly similar to (150)AT1G72060| Symbols: | serine-type endopeptidase inhibitor | chr1:27122156-27122505 FORWARD [20160] 1 43	
JCVI_35492	1.095	moderately similar to (473)AT4G05440| Symbols: EDA35 | EDA35 (embryo sac development arrest 35) | chr4:2757271-2758114 FORWA	
JCVI_36750	1.095	weakly similar to (199)AT2G19110| Symbols: HMA4 | HMA4 (Heavy metal ATPase 4); cadmium-transporting ATPase | chr2:8286560-82	
ES925428	1.095	no similarity	
EV136748	1.095	very weakly similar to (85.1)AT3G05480| Symbols: | cell cycle checkpoint control protein family | chr3:1585389-1588244 FORWARD [21	
JCVI_16735	1.095	highly similar to (501)AT5G60750| Symbols: | CAAX amino terminal protease family protein | chr5:24448298-24450425 FORWARD no	
JCVI_19280	1.095	moderately similar to (349)AT5G50860| Symbols: | protein kinase family protein | chr5:20711004-20714209 REVERSE no original descri	
JCVI_6516	1.094	weakly similar to (175)AT3G12260| Symbols: | complex 1 family protein / LVR family protein | chr3:3909259-3910344 REVERSE no ori	-2.050
EV018505	1.094	no similarity	
JCVI_33016	1.094	highly similar to (531)AT4G23240| Symbols: | protein kinase family protein | chr4:12160512-12161964 REVERSEweakly similar to (200	
JCVI_1788	1.094	moderately similar to (410)AT1G43130| Symbols: LCV2 | LCV2 (LIKE COV 2) | chr1:16231101-16233598 REVERSE no original descrip	
EE418086	1.094	no similarity	
EV101138	1.094	no similarity	

JCVI_24841	1.094	moderately similar to (389)AT4G19700| Symbols: | protein binding / zinc ion binding | chr4:10713643-10714645 REVERSE no original d	
JCVI_10336	1.093	weakly similar to (144)AT5G58710| Symbols: ROC7 | ROC7 (rotamase CyP 7); peptidyl-prolyl cis-trans isomerase | chr5:23735066-23736	
JCVI_9556	1.093	highly similar to (784)AT1G55350| Symbols: EMB1275, ATDEK1, DEK1 | DEK1 (DEFECTIVE KERNEL 1); calcium-dependent cystein	
JCVI_11243	1.093	moderately similar to (212)AT3G07700| Symbols: | ABC1 family protein | chr3:2459702-2463247 REVERSE no original description	
JCVI_15244	1.093	moderately similar to (391)AT3G24430| Symbols: HCF101 | HCF101 (HIGH-CHLOROPHYLL-FLUORESCENCE 101); ATP binding | c	
EV104873	1.093	no similarity	
JCVI_39569	1.093	very weakly similar to (88.2)AT1G34640| Symbols: | similar to Os07g0622900 [Oryza sativa (japonica cultivar-group)] (GB:NP_0010603	
EX036927	1.093	moderately similar to (227)AT1G29370| Symbols: | kinase-related | chr1:10278066-10283010 REVERSE [21811] 1 436 453	
CD837276	1.093	weakly similar to (137)AT3G61700| Symbols: | similar to unknown protein [Arabidopsis thaliana] (TAIR:AT2G46420.1); similar to unnar	
JCVI_14102	1.093	moderately similar to (290)AT5G67030| Symbols: LOS6, NPQ2, ATABA1, ZEP, IBS3, ATZEP, ABA1 | ABA1 (ABA DEFICIENT 1); ze	
JCVI_42497	1.093	no original description	
CD813729	1.093	moderately similar to (329)AT4G19185| Symbols: | integral membrane family protein | chr4:10489212-10491499 REVERSE [13977]	
JCVI_9143	1.093	moderately similar to (363)AT5G55700| Symbols: BMY6, BAM4 | BAM4/BMY6 (BETA-AMYLASE 4); beta-amylase | chr5:22569099-2	
JCVI_19327	1.093	moderately similar to (490)AT3G57240| Symbols: BG3 | BG3 (BETA-1,3-GLUCANASE 3); hydrolase, hydrolyzing O-glycosyl compound	
EX105451	1.093	no similarity	
EV019132	1.093	moderately similar to (202)AT5G13880| Symbols: | similar to unknown protein [Arabidopsis thaliana] (TAIR:AT5G47920.1); similar to h	
AM387087	1.092	moderately similar to (310)AT4G23900| Symbols: | nucleoside diphosphate kinase 4 (NDK4) | chr4:12424515-12426328 FORWARDmod	
EE568825	1.092	no similarity	
ES914667	1.092	no similarity	
CD817058	1.092	very weakly similar to (85.9)AT1G54540| Symbols: | similar to harpin-induced protein-related / HIN1-related / harpin-responsive protein-r	
DY021097	1.092	moderately similar to (452)AT5G38730| Symbols: | pentatricopeptide (PPR) repeat-containing protein | chr5:15528131-15529921 FORW	
EE521247	1.092	very weakly similar to (95.9)AT5G27430| Symbols: | signal peptidase subunit family protein | chr5:9687473-9689188 FORWARD [20185]	
DY030611	1.092	moderately similar to (256)AT5G66550| Symbols: | Maf family protein | chr5:26576978-26578388 REVERSE [18973]	
JCVI_42275	1.092	moderately similar to (396)AT1G53510| Symbols: ATMPK18 | ATMPK18 (ARABIDOPSIS THALIANA MAP KINASE 18); MAP kinas	
JCVI_8583	1.091	moderately similar to (497)AT5G03800| Symbols: EMB1899, EMB166, EMB175 | EMB175 (EMBRYO DEFECTIVE 175); catalytic | ch	
JCVI_10137	1.091	weakly similar to (162)AT5G59970| Symbols: | histone H4 | chr5:24163578-24163889 REVERSEweakly similar to (162)H4_PEA no orig	
JCVI_35135	1.091	moderately similar to (301)AT5G54980| Symbols: | integral membrane family protein | chr5:22333025-22333786 FORWARD no original	
JCVI_1601	1.091	moderately similar to (475)AT1G48450| Symbols: | similar to unknown protein [Arabidopsis thaliana] (TAIR:AT3G17800.1); similar to u	
JCVI_41896	1.091	moderately similar to (272)AT4G38150| Symbols: | pentatricopeptide (PPR) repeat-containing protein | chr4:17901205-17902113 REVER	
JCVI_36132	1.090	moderately similar to (441)AT2G14720| Symbols: VSR-2 | VSR-2 (Vacuolar sorting receptor 2); calcium ion binding | chr2:6307960-6311	
EV142649	1.090	moderately similar to (238)AT3G28610| Symbols: | ATP binding / ATPase | chr3:10726227-10727651 FORWARD [21482]	
JCVI_36463	1.090	weakly similar to (176)AT1G20610| Symbols: CYCB2;3 | CYCB2;3 (CYCLIN B2;3); cyclin-dependent protein kinase regulator | chr1:713	
JCVI_5854	1.090	moderately similar to (295)AT5G16390| Symbols: CAC1A, BCCP, BCCP1, CAC1-A, BCCP-1, CAC1 | BCCP/BCCP-1/BCCP1/CAC1-A/	
CN734920	1.090	no similarity	-1.444
EV011577	1.090	no similarity	
JCVI_6611	1.089	moderately similar to (433)AT3G12740| Symbols: ALIS1 | LEM3 (ligand-effect modulator 3) family protein / CDC50 family protein | chr3	
CX192962	1.089	weakly similar to (114)AT3G14080| Symbols: | small nuclear ribonucleoprotein, putative / snRNP, putative / Sm protein, putative | chr3:4	
CA991482	1.089	weakly similar to (108)AT5G20700| Symbols: | senescence-associated protein-related | chr5:7006180-7007005 REVERSE [12405]	
JCVI_31618	1.089	no original description	
JCVI_33205	1.088	moderately similar to (286)AT5G65650| Symbols: | similar to unknown protein [Arabidopsis thaliana] (TAIR:AT4G36660.1); similar to u	
JCVI_8032	1.088	moderately similar to (224)AT3G47370| Symbols: | 40S ribosomal protein S20 (RPS20B) | chr3:17464656-17465422 REVERSEweakly si	
AM394969	1.088	weakly similar to (182)AT2G17410| Symbols: | ARID/BRIGHT DNA-binding domain-containing protein | chr2:7566951-7570556 FORW	
EV209236	1.088	no similarity	
JCVI_18711	1.088	weakly similar to (196)AT4G26640| Symbols: ATWRKY20, WRKY20 | WRKY20 (WRKY DNA-binding protein 20); transcription factor	
JCVI_38971	1.088	highly similar to (643)AT2G13790| Symbols: ATSERK4 | ATSERK4 (SOMATIC EMBRYOGENESIS RECEPTOR-LIKE KINASE 4); p	
EE559412	1.088	moderately similar to (296)AT5G10930| Symbols: SnRK3.24, CIPK5 | CIPK5 (CBL-INTERACTING PROTEIN KINASE 5); kinase | chr	
JCVI_16305	1.088	moderately similar to (305)AT2G31230| Symbols: ATERF15 | ATERF15 (ETHYLENE-RESPONSIVE ELEMENT BINDING FACTOR 1	
JCVI_34531	1.088	moderately similar to (238)AT5G27710| Symbols: | similar to unnamed protein product [Vitis vinifera] (GB:CAO17522.1) | chr5:9813073	
JCVI_35759	1.087	moderately similar to (479)AT3G11820| Symbols: AT-SYR1, ATSYP121, SYR1, ATSYR1, PEN1, SYP121 | SYP121 (syntaxin 121); SN	
ES947742	1.087	no similarity	
JCVI_5261	1.087	moderately similar to (424)AT4G25700| Symbols: B1, CHY1, BETA-OHASE 1 | BETA-OHASE 1 (BETA-HYDROXYLASE 1) | chr4:13	
JCVI_1768	1.087	moderately similar to (280)AT3G17390| Symbols: SAMS3, MAT4, MTO3 | MTO3 (S-adenosylmethionine synthase 3); methionine adeno	
JCVI_13210	1.086	moderately similar to (345)AT5G13860| Symbols: ELC-LIKE | ATELC-LIKE/ELC-LIKE; small conjugating protein ligase | chr5:4473214	
EV218058	1.086	no similarity	
JCVI_7639	1.086	moderately similar to (290)AT3G52750| Symbols: FTSZ2-2 | FTSZ2-2 (FtsZ2-2); structural molecule | chr3:19560819-19563413 REVERS	
JCVI_522	1.086	weakly similar to (121)AT2G19830| Symbols: SNF7.2 | SNF7.2 | chr2:8565183-8566471 REVERSE no original description	
JCVI_31535	1.086	very weakly similar to (82.0)AT5G61230| Symbols: | ankyrin repeat family protein | chr5:24645480-24646004 FORWARD no original des	
JCVI_37146	1.085	weakly similar to (146)AT1G18190| Symbols: GC2 | GC2 (GOLGIN CANDIDATE 2) | chr1:6257956-6261318 REVERSE no original des	-1.171
JCVI_828	1.085	no original description	
EE473850	1.085	weakly similar to (168)AT4G38060| Symbols: | similar to unknown protein [Arabidopsis thaliana] (TAIR:AT5G65480.1); similar to unkn	
JCVI_25262	1.085	moderately similar to (280)AT1G55530| Symbols: | zinc finger (C3HC4-type RING finger) family protein | chr1:20733138-20734193 RE	
JCVI_20725	1.085	moderately similar to (350)AT2G43160| Symbols: | epsin N-terminal homology (ENTH) domain-containing protein | chr2:17955961-1795	
EV172190	1.085	weakly similar to (124)AT5G02480| Symbols: | similar to SLT1 (SODIUM- AND LITHIUM-TOLERANT 1) [Arabidopsis thaliana] (TAI	
EV013061	1.085	no similarity	
EG020859	1.085	moderately similar to (216)AT3G62910| Symbols: APG3 | APG3 (ALBINO AND PALE GREEN); translation release factor | chr3:232686	
EV142023	1.085	no similarity	
JCVI_6414	1.085	moderately similar to (370)AT4G10360| Symbols: | similar to unknown protein [Arabidopsis thaliana] (TAIR:AT1G31300.2); similar to u	
JCVI_16225	1.085	moderately similar to (299)AT3G13670| Symbols: | protein kinase family protein | chr3:4469441-4473241 FORWARD no original descrip	
JCVI_3975	1.085	moderately similar to (254)AT5G20650| Symbols: COPT5 | COPT5 (copper transporter 5); copper ion transmembrane transporter | chr5:6	
JCVI_41688	1.084	moderately similar to (382)AT4G18950| Symbols: | ankyrin protein kinase, putative | chr4:10375696-10378140 FORWARD no original d	
JCVI_18366	1.084	moderately similar to (482)AT1G67850| Symbols: | similar to unknown protein [Arabidopsis thaliana] (TAIR:AT1G13000.2); similar to u	
EE437249	1.084	moderately similar to (261)AT3G18440| Symbols: ATALMT9 | ATALMT9 (ALUMINUM-ACTIVATED MALATE TRANSPORTER 9);	
DY012631	1.084	moderately similar to (229)AT3G47080| Symbols: | binding | chr3:17349400-17351114 REVERSE [18975]	
JCVI_11370	1.084	highly similar to (618)AT1G76380| Symbols: | DNA-binding bromodomain-containing protein | chr1:28656310-28659430 REVERSE no	
JCVI_1328	1.084	moderately similar to (318)AT1G31910| Symbols: | GHMP kinase family protein | chr1:11459030-11461629 FORWARD no original desc	
JCVI_28069	1.083	weakly similar to (171)AT3G18524| Symbols: ATMSH2, MSH2 | MSH2 (MUTS HOMOLOG 2); ATP binding / damaged DNA binding |	
JCVI_5770	1.083	moderately similar to (316)AT1G35470| Symbols: | SPla/RYanodine receptor (SPRY) domain-containing protein | chr1:13051614-130549	
EE481897	1.083	weakly similar to (154)AT4G25850| Symbols: | oxysterol-binding family protein | chr4:13143868-13146663 FORWARD [20154]	
AM395507	1.083	no similarity	
JCVI_1922	1.083	moderately similar to (496)AT1G20270| Symbols: | oxidoreductase, 2OG-Fe(II) oxygenase family protein | chr1:7021373-7022913 REVE	
JCVI_19194	1.083	moderately similar to (286)AT1G25520| Symbols: | similar to unknown protein [Arabidopsis thaliana] (TAIR:AT1G68650.1); similar to u	

JCVI_27900	1.082	weakly similar to (165)AT5G53340| Symbols: | galactosyltransferase family protein | chr5:21658271-21660421 REVERSE no original des	
JCVI_108	1.082	moderately similar to (407)AT5G27520| Symbols: | mitochondrial substrate carrier family protein | chr5:9714667-9716247 REVERSE no	
JCVI_11947	1.082	weakly similar to (119)AT3G57120| Symbols: | protein kinase family protein | chr3:21150078-21151448 FORWARD no original descripti	
EE421897	1.082	no similarity	
JCVI_19560	1.082	no original description	
EX123017	1.081	no similarity	
EV155598	1.081	moderately similar to (310)AT3G21690| Symbols: | MATE efflux family protein | chr3:7638757-7641868 FORWARD [21484] 57 775 77	1.158
EE458112	1.081	moderately similar to (230)AT2G22980| Symbols: SCPL13 | SCPL13 | chr2:9786109-9790168 FORWARDweakly similar to (123)CBP1_	
EE548218	1.081	moderately similar to (266)AT4G32551| Symbols: RON2, LUG | LUG (LEUNIG) | chr4:15707869-15713365 FORWARD [20120] 45 736	
EX092364	1.081	moderately similar to (283)AT5G61420| Symbols: ATMYB28, PMG1, HAG1, MYB28 | MYB28 (MYB DOMAIN PROTEIN 28); DNA b	
JCVI_13008	1.081	moderately similar to (248)AT1G29240| Symbols: | similar to unknown protein [Arabidopsis thaliana] (TAIR:AT2G34170.1); similar to u	
JCVI_41658	1.081	highly similar to (701)AT3G22780| Symbols: TSO1 | TSO1 (CHINESE FOR 'UGLY'); transcription factor | chr3:8048934-8052065 FORW	
DY029219	1.081	weakly similar to (137)AT3G19650| Symbols: | cyclin-related | chr3:6823549-6825180 FORWARD [18978]	
EX044090	1.081	no similarity	
ES909645	1.080	weakly similar to (117)AT2G47710| Symbols: | universal stress protein (USP) family protein | chr2:19562115-19563026 REVERSE [2143	
EV087794	1.080	weakly similar to (153)AT5G44390| Symbols: | FAD-binding domain-containing protein | chr5:17899556-17902133 REVERSE [21444]	
JCVI_1315	1.080	moderately similar to (315)AT4G27960| Symbols: UBC9 | UBC9 (UBIQUITIN CONJUGATING ENZYME 9); ubiquitin-protein ligase | c	
JCVI_4186	1.080	moderately similar to (252)AT3G47640| Symbols: | basic helix-loop-helix (bHLH) family protein | chr3:17579020-17580086 FORWARD	
EE567617	1.079	no similarity	
JCVI_32891	1.079	moderately similar to (270)AT1G21380| Symbols: | VHS domain-containing protein / GAT domain-containing protein | chr1:7485795-748	
JCVI_32025	1.079	moderately similar to (263)AT5G15540| Symbols: EMB2773 | EMB2773 (EMBRYO DEFECTIVE 2773); binding / protein binding / zinc	
EV206346	1.079	very weakly similar to (86.3)AT5G08720| Symbols: | similar to unknown protein [Arabidopsis thaliana] (TAIR:AT4G01650.2); similar to u	
JCVI_41949	1.079	no original description	
CD833507	1.079	moderately similar to (245)AT3G02065| Symbols: | DEAD/DEAH box helicase family protein | chr3:359143-360741 FORWARD [13980]	-1.332
AM060597	1.078	no similarity	
JCVI_12072	1.078	highly similar to (613)AT3G27060| Symbols: TSO2 | TSO2 (TSO MEANING 'UGLY' IN CHINESE); ribonucleoside-diphosphate reducta	
JCVI_13607	1.078	highly similar to (502)AT2G38610| Symbols: | KH domain-containing protein | chr2:16154630-16156716 REVERSE no original descripti	
JCVI_12438	1.078	moderately similar to (463)AT2G26780| Symbols: | binding | chr2:11417203-11430676 FORWARD no original description	
ES975529	1.077	no similarity	-1.610
JCVI_36286	1.077	no original description	
DY026415	1.076	moderately similar to (283)AT1G18040| Symbols: CDKD1;3, AT;CDCKD;3, CAK2AT | AT;CDCKD;3/CAK2AT/CDKD1;3 (CYCLIN-D	
EV211189	1.076	weakly similar to (109)AT4G00780| Symbols: | meprin and TRAF homology domain-containing protein / MATH domain-containing prote	
JCVI_29965	1.076	moderately similar to (476)AT4G15415| Symbols: ATB' GAMMA | ATB' GAMMA; poly(U) binding / protein phosphatase type 2A regula	
RC_ES966048	1.076	no similarity	
EE531205	1.076	weakly similar to (123)AT3G50660| Symbols: CYP90B1, CLM, SNP2, DWF4 | DWF4 (DWARF 4) | chr3:18825243-18828149 REVERSI	
JCVI_41175	1.076	weakly similar to (173)AT5G06870| Symbols: PGIP2 | PGIP2 (POLYGALACTURONASE INHIBITING PROTEIN 2); protein binding | c	-1.280
EE517807	1.075	no similarity	
EL586787	1.075	no similarity	
JCVI_31269	1.075	moderately similar to (253)AT3G51080| Symbols: | zinc finger (GATA type) family protein | chr3:18984619-18985648 FORWARD no or	-1.343
EE546203	1.075	moderately similar to (414)AT5G09670| Symbols: | loricrin-related | chr5:2996110-2997750 REVERSE [20128] 37 797 797	
JCVI_2520	1.075	moderately similar to (373)AT1G18060| Symbols: | similar to unnamed protein product [Vitis vinifera] (GB:CAO68142.1) | chr1:6212058	
JCVI_13063	1.074	moderately similar to (297)AT2G36950| Symbols: | heavy-metal-associated domain-containing protein | chr2:15522295-15523833 FORW	
EE442502	1.074	no similarity	2.077
RC_EX116514	1.074	no similarity	
EX092309	1.074	moderately similar to (357)AT3G18210| Symbols: | oxidoreductase, 2OG-Fe(II) oxygenase family protein | chr3:6238270-6240402 REVE	
JCVI_34533	1.074	moderately similar to (229)AT1G27190| Symbols: | leucine-rich repeat transmembrane protein kinase, putative | chr1:9446910-9448715 R	
JCVI_28530	1.074	weakly similar to (164)AT5G66640| Symbols: | LIM domain-containing protein-related | chr5:26617044-26618991 FORWARD no origina	-1.368
EE555103	1.074	very weakly similar to (100)AT1G76040| Symbols: CPK29 | CPK29 (calcium-dependent protein kinase 29); calmodulin-dependent protein	
JCVI_6718	1.073	moderately similar to (241)AT4G13530| Symbols: | similar to unknown protein [Arabidopsis thaliana] (TAIR:AT4G10080.1); similar to u	
ES966979	1.073	no similarity	
JCVI_32482	1.073	moderately similar to (397)AT1G56080| Symbols: | similar to unknown protein [Arabidopsis thaliana] (TAIR:AT1G16520.1); similar to u	
JCVI_657	1.073	moderately similar to (367)AT1G64090| Symbols: | reticulon family protein (RTNLB3) | chr1:23793058-23794332 FORWARD no origina	
JCVI_9918	1.073	weakly similar to (120)AT3G47833| Symbols: | similar to unknown protein [Arabidopsis thaliana] (TAIR:AT5G62575.1); similar to unnar	
CX281814	1.072	weakly similar to (106)AT4G28300| Symbols: | hydroxyproline-rich glycoprotein family protein | chr4:14015420-14016829 FORWARD [	
ES989063	1.072	moderately similar to (208)AT1G77230| Symbols: | tetratricopeptide repeat (TPR)-containing protein | chr1:29021023-29022767 FORWA	
AM390245	1.072	weakly similar to (179)AT1G02860| Symbols: NLA | NLA (NITROGEN LIMITATION ADAPTATION); protein binding / zinc ion bindir	
EV061001	1.072	moderately similar to (245)AT3G05040| Symbols: HST1, HST | HST (HASTY) | chr3:1401485-1408101 REVERSE [21442] 1 586 599	
EV045212	1.072	weakly similar to (138)AT5G20280| Symbols: ATSPS1F | ATSPS1F (SUCROSE PHOSPHATE SYNTHASE 1F); sucrose-phosphate syntl	1.181
ES944523	1.072	no similarity	
JCVI_3907	1.072	moderately similar to (353)AT2G38650| Symbols: GAUT7, LGT7 | GAUT7/LGT7 (GALACTURONOSYLTRANSFERASE 7); polygalac	
JCVI_6082	1.072	moderately similar to (278)AT1G01470| Symbols: LSR3, LEA14 | LEA14 (LATE EMBRYOGENESIS ABUNDANT 14) | chr1:172295-17	
JCVI_16583	1.072	weakly similar to (128)AT3G04720| Symbols: HEL, PR-4, PR4 | PR4 (PATHOGENESIS-RELATED 4) | chr3:1285697-1286537 REVERS	
JCVI_34536	1.071	no original description	
ES992736	1.071	weakly similar to (167)AT4G16490| Symbols: | armadillo/beta-catenin repeat family protein | chr4:9294747-9295553 REVERSE [21426]	
BG732267	1.071	moderately similar to (256)AT5G28510| Symbols: | glycosyl hydrolase family 1 protein | chr5:10481045-10484026 REVERSEvery weakly	
RC_ES969249	1.071	no similarity	
EE472376	1.071	moderately similar to (339)AT5G45940| Symbols: ATNUDT11 | ATNUDT11 (Arabidopsis thaliana Nudix hydrolase homolog 11) | chr5:1	
JCVI_16799	1.071	moderately similar to (203)AT5G02060| Symbols: | integral membrane protein, putative | chr5:404905-405771 FORWARD no original de	
JCVI_28190	1.071	moderately similar to (316)AT4G33350| Symbols: | chloroplast inner membrane import protein Tic22, putative | chr4:16064642-1606671	
JCVI_6348	1.071	moderately similar to (303)AT2G30350| Symbols: | endo/excinuclease amino terminal domain-containing protein | chr2:12941314-129428	
CV545368	1.070	no similarity	
JCVI_10362	1.070	highly similar to (795)AT5G67360| Symbols: ARA12 | ARA12; subtilase | chr5:26889418-26891691 REVERSE no original description	
AM392235	1.070	no similarity	
EE436231	1.070	very weakly similar to (83.6)AT3G59280| Symbols: TXR1 | TXR1 (THAXTOMIN A RESISTANT 1) | chr3:21920243-21921496 REVERS	
JCVI_396	1.070	moderately similar to (238)AT1G68310| Symbols: | Identical to UPF0195 protein At1g68310 [Arabidopsis Thaliana] (GB:Q9C9G6); simi	
RC_EV052313	1.070	no similarity	
JCVI_10364	1.070	moderately similar to (369)AT3G25690| Symbols: CHUP1 | CHUP1 (CHLOROPLAST UNUSUAL POSITIONING 1) | chr3:9355298-935	
EV143316	1.070	weakly similar to (141)AT4G24380| Symbols: | similar to unknown protein [Arabidopsis thaliana] (TAIR:AT5G65400.1); similar to unkn	
JCVI_4911	1.070	moderately similar to (236)AT3G27390| Symbols: | Identical to Uncharacterized membrane protein At3g27390 [Arabidopsis Thaliana] (G	
CD826523	1.070	very weakly similar to (85.5)AT2G29930| Symbols: | F-box family protein | chr2:12763534-12765164 REVERSE [13979]	
JCVI_27837	1.070	weakly similar to (139)AT1G45230| Symbols: | defective chloroplasts and leaves protein-related / DCL protein-related | chr1:17172314-17	

JCVI_10108	1.069	moderately similar to (403)AT5G11980| Symbols: | conserved oligomeric Golgi complex component-related / COG complex component-r	
JCVI_23464	1.069	moderately similar to (223)AT1G14180| Symbols: | unknown protein | chr1:4847972-4848801 FORWARD no original description	
RC_CV432080	1.069	no similarity	
JCVI_25327	1.069	moderately similar to (445)AT5G10030| Symbols: OBF4, TGA4 | TGA4 (TGACG MOTIF-BINDING FACTOR 4); DNA binding / calmo	1.704
EV172468	1.069	weakly similar to (131)AT3G03170| Symbols: | similar to hypothetical protein [Vitis vinifera] (GB:CAN71065.1); similar to unnamed pro	
JCVI_14446	1.069	no original description	
JCVI_25632	1.069	moderately similar to (393)AT2G20900| Symbols: | diacylglycerol kinase, putative | chr2:8996493-8999879 REVERSEmoderately similar	
JCVI_11787	1.068	weakly similar to (182)AT5G67070| Symbols: RALFL34 | RALFL34 (RALF-LIKE 34) | chr5:26785346-26785735 FORWARD no original	
EV040797	1.068	moderately similar to (312)AT5G39680| Symbols: EMB2744 | EMB2744 (EMBRYO DEFECTIVE 2744) | chr5:15901464-15903596 REV	
JCVI_7214	1.068	moderately similar to (312)AT3G57910| Symbols: | D111/G-patch domain-containing protein | chr3:21453748-21454945 FORWARD no	
JCVI_19436	1.068	weakly similar to (197)AT3G57560| Symbols: | aspartate/glutamate/uridylate kinase family protein | chr3:21322141-21323184 REVERSE	
JCVI_28883	1.068	weakly similar to (161)AT1G09970| Symbols: | leucine-rich repeat transmembrane protein kinase, putative | chr1:3252410-3255430 FORV	
JCVI_40847	1.068	highly similar to (597)AT3G16785| Symbols: PLDZ1, PLDZETA1, PLD ZETA 1, PLDP1 | PLDP1 (PHOSPHOLIPASE D ZETA1); phos	
JCVI_26154	1.068	moderately similar to (296)AT4G36540| Symbols: BEE2 | BEE2 (BR ENHANCED EXPRESSION 2); DNA binding / transcription factor	
JCVI_8666	1.068	no original description	
CD835552	1.068	moderately similar to (205)AT3G62260| Symbols: | protein phosphatase 2C, putative / PP2C, putative | chr3:23049491-23051366 REVER	
EV158972	1.068	no similarity	
EX071696	1.067	weakly similar to (109)AT4G31390| Symbols: | ABC1 family protein | chr4:15233132-15236770 FORWARD [21816]	
JCVI_13962	1.067	highly similar to (561)AT3G51770| Symbols: ETO1 | ETO1 (ETHYLENE OVERPRODUCER 1) | chr3:19211307-19214974 REVERSE n	
JCVI_27811	1.067	no original description	
ES913453	1.067	moderately similar to (324)AT1G74590| Symbols: ATGSTU10 | ATGSTU10 (Arabidopsis thaliana Glutathione S-transferase (class tau) 1	
JCVI_28741	1.067	weakly similar to (111)AT4G31670| Symbols: | ubiquitin carboxyl-terminal hydrolase family protein / zinc finger (MYND type) family pr	
EH427830	1.066	moderately similar to (349)AT1G11310| Symbols: ATMLO2, PMR2, MLO2 | MLO2 (MILDEW RESISTANCE LOCUS O 2) | chr1:3801	
JCVI_20113	1.066	very weakly similar to (95.5)AT3G15353| Symbols: MT3 | MT3 (METALLOTHIONEIN 3) | chr3:5180832-5181332 REVERSE no origina	
JCVI_14947	1.066	weakly similar to (160)AT1G76310| Symbols: CYCB2;4 | CYCB2;4 (CYCLIN B2;4); cyclin-dependent protein kinase regulator | chr1:286	
JCVI_15716	1.066	moderately similar to (299)AT4G14950| Symbols: | similar to unknown protein [Arabidopsis thaliana] (TAIR:AT1G05360.1); similar to u	
CX192271	1.065	weakly similar to (124)AT1G14450| Symbols: | similar to unknown protein [Arabidopsis thaliana] (TAIR:AT2G02510.1); similar to unkn	
EE538302	1.065	moderately similar to (260)AT3G21690| Symbols: | MATE efflux family protein | chr3:7638757-7641868 FORWARD [20161]	
JCVI_28645	1.065	moderately similar to (372)AT1G62300| Symbols: WRKY6 | WRKY6 (WRKY DNA-binding protein 6); transcription factor | chr1:230205	
JCVI_32887	1.065	nearly identical (1227)AT5G04930| Symbols: ALA1 | ALA1 (AMINOPHOSPHOLIPID ATPASE1); ATPase, coupled to transmembrane m	
EE505231	1.065	no similarity	
JCVI_10193	1.065	moderately similar to (252)AT5G64480| Symbols: | similar to hypothetical protein MtrDRAFT_AC148396g23v2 [Medicago truncatula] (	
EV153310	1.065	moderately similar to (317)AT4G28350| Symbols: | lectin protein kinase family protein | chr4:14026583-14028628 FORWARD [21484]	
CX273003	1.065	moderately similar to (239)AT5G18750| Symbols: | DNAJ heat shock N-terminal domain-containing protein | chr5:6255179-6257833 FOF	
DY017708	1.065	weakly similar to (126)AT2G47240| Symbols: | long-chain-fatty-acid--CoA ligase family protein / long-chain acyl-CoA synthetase family	
CN728844	1.064	no similarity	
JCVI_31391	1.064	no original description	
EG019285	1.064	no similarity	
JCVI_30605	1.064	highly similar to (778)AT5G10290| Symbols: | leucine-rich repeat family protein / protein kinase family protein | chr5:3235463-3238172 I	
JCVI_41264	1.064	highly similar to (564)AT3G20530| Symbols: | protein kinase family protein | chr3:7166324-7167812 FORWARDmoderately similar to (	
JCVI_15309	1.064	weakly similar to (134)AT2G20330| Symbols: | transducin family protein / WD-40 repeat family protein | chr2:8779969-8782599 REVER	
ES978370	1.063	very weakly similar to (98.6)AT4G12230| Symbols: | esterase/lipase/thioesterase family protein | chr4:7284639-7287342 FORWARD [214	
EE436672	1.063	weakly similar to (113)AT1G71865| Symbols: | similar to unnamed protein product [Vitis vinifera] (GB:CAO64617.1) | chr1:27033779-2	
EV053883	1.063	weakly similar to (191)AT4G38620| Symbols: ATMYB4, MYB4 | MYB4 (myb domain protein 4); transcription factor | chr4:18053860-18	
EX035156	1.063	no similarity	
JCVI_36201	1.063	no original description	-1.636
JCVI_9641	1.063	moderately similar to (395)AT2G12190| Symbols: | cytochrome P450, putative | chr2:4898889-4900427 REVERSEmoderately similar to (	
EE443710	1.062	no similarity	-1.312
EE550056	1.062	no similarity	
JCVI_30593	1.062	moderately similar to (212)AT2G38800| Symbols: | calmodulin-binding protein-related | chr2:16224077-16225915 FORWARD no origina	
BQ704469	1.062	no similarity	
JCVI_39057	1.062	highly similar to (893)AT2G42600| Symbols: ATPPC2 | ATPPC2 (PHOSPHOENOLPYRUVATE CARBOXYLASE 2); phosphoenolpyru	
EV201303	1.062	moderately similar to (248)AT5G58330| Symbols: | malate dehydrogenase (NADP), chloroplast, putative | chr5:23597236-23599513 REV	
EV087684	1.062	weakly similar to (132)AT5G20720| Symbols: CPN10, CHCPN10, ATCPN21, CPN21, CPN20 | CPN20 (CHAPERONIN 20); calmodulin	
JCVI_40143	1.062	moderately similar to (309)AT5G62820| Symbols: | integral membrane protein, putative | chr5:25241054-25242124 REVERSE no origina	
JCVI_38394	1.062	highly similar to (544)AT3G56330| Symbols: | N2,N2-dimethylguanosine tRNA methyltransferase family protein | chr3:20901229-20902	
JCVI_15650	1.061	weakly similar to (149)AT3G60740| Symbols: EMB133, TFC D, CHO, TTN1 | TTN1 (TITAN 1) | chr3:22458360-22464300 REVERSE n	
JCVI_36276	1.061	moderately similar to (418)AT1G79530| Symbols: GAPCP-1 | GAPCP-1; glyceraldehyde-3-phosphate dehydrogenase | chr1:29921125-299	
JCVI_1962	1.061	weakly similar to (120)AT1G56330| Symbols: ATSARA1B, ATSAR1, SAR1 | SAR1 (SECRETION-ASSOCIATED RAS); GTP binding |	
EX060408	1.061	weakly similar to (157)AT1G52290| Symbols: | protein kinase family protein | chr1:19473920-19476031 REVERSE [21813]	
EX087749	1.061	moderately similar to (384)AT5G45820| Symbols: PKS18, SnRK3.6, CIPK20 | CIPK20 (CBL-INTERACTING PROTEIN KINASE 20); k	
JCVI_8183	1.061	moderately similar to (351)AT3G14920| Symbols: | similar to unknown protein [Arabidopsis thaliana] (TAIR:AT5G05480.1); similar to P	-3.263
JCVI_6869	1.061	highly similar to (802)AT5G08260| Symbols: SCPL35 | SCPL35 (serine carboxypeptidase-like 35); serine carboxypeptidase | chr5:265723	
JCVI_18124	1.061	no original description	
CO750485	1.061	very weakly similar to (92.4)AT1G24267| Symbols: | similar to unknown protein [Arabidopsis thaliana] (TAIR:AT1G24265.2); similar to u	
JCVI_10185	1.060	no original description	1.670
CD813037	1.060	no similarity	
ES978365	1.060	no similarity	
JCVI_4589	1.060	moderately similar to (280)AT1G69870| Symbols: | proton-dependent oligopeptide transport (POT) family protein | chr1:26319871-26323	
JCVI_34810	1.060	moderately similar to (306)AT1G72050| Symbols: | zinc finger (C2H2 type) family protein | chr1:27119470-27120890 FORWARD no ori	
JCVI_29401	1.059	moderately similar to (234)AT4G03260| Symbols: | leucine-rich repeat family protein | chr4:1429092-1431431 FORWARD no original de	-1.164
EV125974	1.059	moderately similar to (320)AT4G00710| Symbols: | protein kinase family protein | chr4:290807-293096 FORWARD [21479] 44 791 791	
EE448199	1.059	no similarity	
JCVI_15653	1.059	moderately similar to (477)AT2G46470| Symbols: OXA1L | OXA1L (INNER MEMBRANE PROTEIN OXA1-LIKE) | chr2:19079731-19	
EX044782	1.059	no similarity	
JCVI_4965	1.058	no original description	
DY027977	1.058	weakly similar to (195)AT1G15910| Symbols: | XH/XS domain-containing protein / XS zinc finger domain-containing protein | chr1:5465	
EX020367	1.058	no similarity	
JCVI_31706	1.058	moderately similar to (399)AT5G24650| Symbols: | mitochondrial import inner membrane translocase subunit Tim17/Tim22/Tim23 famil	1.221
JCVI_21140	1.058	moderately similar to (207)AT5G18310| Symbols: | similar to unknown protein [Arabidopsis thaliana] (TAIR:AT5G48500.1); similar to u	
DY014301	1.058	very weakly similar to (91.7)AT5G18660| Symbols: PCB2, DVR | DVR (PALE-GREEN AND CHLOROPHYLL B REDUCED 2); 3,8-div	

JCVI_22583	1.058	no original description	
JCVI_21914	1.058	no original description	-1.161
EV012734	1.058	no similarity	
JCVI_17653	1.057	moderately similar to (364)AT2G39700| Symbols: ATEXP4, ATHEXP ALPHA 1.6, ATEXPA4 | ATEXPA4 (ARABIDOPSIS THALIAN	
JCVI_2259	1.057	moderately similar to (327)AT5G33320| Symbols: PPT, ARAPPT, CUE1 | CUE1 (CAB UNDEREXPRESSED 1); antiporter/ triose-phosp	-1.051
RC_ES899358	1.057	no similarity	
EE402999	1.057	moderately similar to (207)AT1G19860| Symbols: | zinc finger (CCCH-type) family protein | chr1:6891645-6894391 REVERSE [16820]	
JCVI_31901	1.057	moderately similar to (452)AT3G01400| Symbols: | armadillo/beta-catenin repeat family protein | chr3:151927-152994 FORWARDweakly	
AM390711	1.057	moderately similar to (260)AT1G09970| Symbols: | leucine-rich repeat transmembrane protein kinase, putative | chr1:3252410-3255430 F	
JCVI_20143	1.057	moderately similar to (207)AT2G35610| Symbols: | similar to unknown protein [Arabidopsis thaliana] (TAIR:AT1G70630.1); similar to u	
EX046574	1.057	moderately similar to (262)AT4G36630| Symbols: EMB2754 | EMB2754 (EMBRYO DEFECTIVE 2754); binding / small GTPase regulat	
CX190796	1.056	weakly similar to (108)AT4G27680| Symbols: | MSP1 protein, putative / intramitochondrial sorting protein, putative | chr4:13821269-138	
JCVI_11863	1.056	highly similar to (527)AT4G36440| Symbols: | similar to unnamed protein product [Vitis vinifera] (GB:CAO48295.1) | chr4:17207216-17	
EE532680	1.056	moderately similar to (251)AT2G47160| Symbols: BOR1 | BOR1 (REQUIRES HIGH BORON 1) | chr2:19364810-19367857 REVERSE [	
ES924045	1.056	weakly similar to (119)AT2G30970| Symbols: ASP1 | ASP1 (ASPARTATE AMINOTRANSFERASE 1) | chr2:13186089-13188763 FORV	
ES913478	1.056	no similarity	
JCVI_32935	1.056	moderately similar to (383)AT2G38750| Symbols: ANNAT4 | ANNAT4 (ANNEXIN ARABIDOPSIS 4); calcium ion binding / calcium-de	
JCVI_8804	1.056	weakly similar to (109)AT5G59970| Symbols: | histone H4 | chr5:24163578-24163889 REVERSEweakly similar to (109)H4_PEA no orig	-1.057
EX126730	1.055	very weakly similar to (96.7)AT2G04350| Symbols: | long-chain-fatty-acid--CoA ligase family protein / long-chain acyl-CoA synthetase fa	
EV159713	1.055	weakly similar to (153)AT4G17620| Symbols: | glycine-rich protein | chr4:9822018-9824872 FORWARD [21484]	
JCVI_40446	1.054	moderately similar to (467)AT3G22180| Symbols: | zinc finger (DHHC type) family protein | chr3:7827197-7830515 FORWARD no origi	
EV139945	1.054	no similarity	1.280
JCVI_18321	1.054	moderately similar to (275)AT3G17130| Symbols: | invertase/pectin methylesterase inhibitor family protein | chr3:5844501-5845052 REV	1.877
CX188179	1.054	moderately similar to (222)AT3G21320| Symbols: | similar to ELF3 (EARLY FLOWERING 3) [Arabidopsis thaliana] (TAIR:AT2G2593	
JCVI_19710	1.054	moderately similar to (314)AT3G45140| Symbols: ATLOX2, LOX2 | LOX2 (LIPOXYGENASE 2) | chr3:16536422-16540218 FORWARI	
EV062556	1.054	no similarity	
AM062044	1.054	moderately similar to (295)AT4G27710| Symbols: CYP709B3 | CYP709B3 (cytochrome P450, family 709, subfamily B, polypeptide 3); o	
JCVI_34719	1.053	no original description	
JCVI_12950	1.053	moderately similar to (323)AT5G01200| Symbols: | myb family transcription factor | chr5:77115-78293 FORWARD no original descriptic	
JCVI_2124	1.053	moderately similar to (212)AT2G23760| Symbols: SAW2, BLH4 | BLH4 (SAWTOOTH 2); DNA binding | chr2:10115031-10119816 REV	
EE549006	1.053	moderately similar to (451)AT3G07960| Symbols: | phosphatidylinositol-4-phosphate 5-kinase family protein | chr3:2535280-2538096 FO	
ES969254	1.053	no similarity	
EE530259	1.053	moderately similar to (286)AT2G40410| Symbols: | Ca(2+)-dependent nuclease, putative | chr2:16880587-16882293 FORWARD [16815]	
JCVI_26289	1.052	moderately similar to (338)AT1G07220| Symbols: | similar to unknown protein [Arabidopsis thaliana] (TAIR:AT5G23850.1); similar to h	
JCVI_25437	1.052	nearly identical (1081)AT1G62020| Symbols: | coatomer protein complex, subunit alpha, putative | chr1:22923479-22927393 FORWARD	
JCVI_5247	1.052	moderately similar to (243)AT2G28900| Symbols: ATOEP16-L, ATOEP16-1, OEP16 | OEP16 (OUTER ENVELOPE PROTEIN 16); P-P	
EX072254	1.052	moderately similar to (447)AT3G58520| Symbols: | similar to unknown protein [Arabidopsis thaliana] (TAIR:AT5G48040.1); similar to u	
JCVI_33901	1.052	weakly similar to (195)AT1G76410| Symbols: ATL8 | ATL8; protein binding / zinc ion binding | chr1:28673809-28674366 FORWARDve	
ES968892	1.051	weakly similar to (181)AT1G64210| Symbols: | leucine-rich repeat transmembrane protein kinase, putative | chr1:23834696-23836526 FO	
EV115177	1.051	moderately similar to (213)AT3G11570| Symbols: | similar to unknown protein [Arabidopsis thaliana] (TAIR:AT5G06230.1); similar to h	
EE515915	1.051	no similarity	
JCVI_11550	1.051	moderately similar to (360)AT1G33990| Symbols: | hydrolase, alpha/beta fold family protein | chr1:12355889-12357874 FORWARDvery	
EV127594	1.051	very weakly similar to (85.1)AT1G17750| Symbols: | leucine-rich repeat transmembrane protein kinase, putative | chr1:6106649-6110001 I	
ES900323	1.051	moderately similar to (255)AT1G76360| Symbols: | protein kinase, putative | chr1:28648136-28651377 REVERSEvery weakly similar to (	
EV110472	1.051	no similarity	
EV069615	1.050	no similarity	
ES918166	1.050	very weakly similar to (92.8)AT2G29140| Symbols: APUM3 | APUM3 (ARABIDOPSIS PUMILIO 3); RNA binding | chr2:12538469-1254	
EV075752	1.050	no similarity	
JCVI_41438	1.050	moderately similar to (358)AT4G19900| Symbols: | glycosyl transferase-related | chr4:10786958-10791443 REVERSE no original descrip	
JCVI_19634	1.050	highly similar to (557)AT3G16630| Symbols: ATKINESIN-13A, KINESIN-13A | ATKINESIN-13A/KINESIN-13A; microtubule motor | c	
JCVI_20187	1.050	moderately similar to (464)AT5G18760| Symbols: | zinc finger (C3HC4-type RING finger) family protein | chr5:6258510-6260830 REVE	
DY010126	1.050	no similarity	
EV153507	1.050	moderately similar to (415)AT3G04810| Symbols: ATNEK2 | ATNEK2; kinase | chr3:1318102-1321107 FORWARD [21484]	
EX030491	1.050	moderately similar to (206)AT1G29690| Symbols: CAD1 | CAD1 (CONSTITUTIVELY ACTIVATED CELL DEATH 1) | chr1:10379296	
JCVI_27676	1.050	moderately similar to (244)AT1G49600| Symbols: ATRBP47A | ATRBP47A (ARABIDOPSIS THALIANA RNA-BINDING PROTEIN 4	
DY024874	1.050	moderately similar to (251)AT1G03820| Symbols: | similar to E6-4 [Gossypium hirsutum] (GB:AAY43793.1); similar to E6-3 [Gossypiur	
JCVI_209	1.050	moderately similar to (366)AT3G56090| Symbols: ATFER3 | ATFER3 (FERRITIN 3); ferric iron binding | chr3:20825329-20826963 REV	
JCVI_5245	1.049	moderately similar to (276)AT5G01460| Symbols: | LMBR1 integral membrane family protein | chr5:186822-190007 FORWARD no orig	
ES980194	1.049	moderately similar to (282)AT5G20320| Symbols: DCL4 | DCL4 (DICER-LIKE 4); RNA binding / ribonuclease III | chr5:6859573-68690	
JCVI_20525	1.049	nearly identical (1173)AT5G02500| Symbols: HSP70-1, AT-HSC70-1, HSC70, HSC70-1 | HSC70-1 (heat shock cognate 70 kDa protein 1)	
JCVI_7319	1.049	highly similar to (717)AT4G19110| Symbols: | protein kinase, putative | chr4:10454781-10457043 REVERSEmoderately similar to (222)	
JCVI_27137	1.049	moderately similar to (330)AT5G18520| Symbols: | similar to unknown protein [Arabidopsis thaliana] (TAIR:AT3G09570.1); similar to u	
JCVI_19897	1.049	moderately similar to (276)AT3G54750| Symbols: | similar to unnamed protein product [Vitis vinifera] (GB:CAO48944.1); contains doma	
EL590643	1.049	moderately similar to (282)AT2G05160| Symbols: | zinc finger (CCCH-type) family protein / RNA recognition motif (RRM)-containing p	
JCVI_37841	1.049	no original description	
JCVI_26632	1.049	moderately similar to (269)AT1G72470| Symbols: ATEXO70D1 | ATEXO70D1 (exocyst subunit EXO70 family protein D1); protein bind	
CD827011	1.048	moderately similar to (333)AT4G16144| Symbols: | similar to mov34 family protein [Arabidopsis thaliana] (TAIR:AT1G48790.1); similar	
JCVI_4180	1.047	moderately similar to (383)AT1G03630| Symbols: PORC, POR C | POR C (PROTOCHLOROPHYLLIDE OXIDOREDUCTASE); oxidor	
EV067384	1.047	no similarity	
CD815959	1.047	weakly similar to (161)AT1G53550| Symbols: | F-box family protein | chr1:19987079-19988305 FORWARD [13977]	
JCVI_559	1.046	moderately similar to (485)AT3G56190| Symbols: ASNAP, ALPHA-SNAP2 | ALPHA-SNAP2 (ALPHA-SOLUBLE NSF ATTACHMENT	
EX085687	1.046	moderately similar to (325)AT5G36890| Symbols: | glycosyl hydrolase family 1 protein | chr5:14558757-14563320 REVERSEweakly sim	
AM388361	1.046	no similarity	
EV144184	1.046	no similarity	
EV058267	1.046	no similarity	-1.298
ES969192	1.046	no similarity	-1.205
EE442587	1.046	no similarity	
JCVI_8686	1.046	moderately similar to (257)AT1G13195| Symbols: | zinc finger (C3HC4-type RING finger) family protein | chr1:4501775-4502852 REVE	
EV186777	1.046	moderately similar to (259)AT2G24520| Symbols: AHA5 | AHA5 (ARABIDOPSIS H(+)-ATPASE 5); ATPase | chr2:10422602-10426810	
DT317658	1.046	no similarity	
RC_H07461	1.045	no similarity	

JCVI_5288	1.045	moderately similar to (422)AT3G60450| Symbols: | similar to unknown protein [Arabidopsis thaliana] (TAIR:AT3G60440.1); similar to u	
JCVI_39545	1.045	very weakly similar to (95.5)AT5G40460| Symbols: | similar to unknown protein [Arabidopsis thaliana] (TAIR:AT3G27630.1); similar to l	
EV108352	1.045	no similarity	
EE471110	1.045	no similarity	
EV131892	1.044	no similarity	
JCVI_30658	1.044	moderately similar to (277)AT5G50110| Symbols: | methyltransferase-related | chr5:20391428-20393294 FORWARD no original descript	
EX030395	1.044	highly similar to (511)AT2G27210| Symbols: | kelch repeat-containing serine/threonine phosphoesterase family protein | chr2:11637266-1	
JCVI_17655	1.044	moderately similar to (452)AT2G02040| Symbols: PTR2-B, NTR1, ATPTR2-B | ATPTR2-B (NITRATE TRANSPORTER 1); transporter	
EE469326	1.044	moderately similar to (243)AT1G22200| Symbols: | similar to unknown protein [Arabidopsis thaliana] (TAIR:AT1G36050.1); similar to u	
JCVI_4811	1.044	no original description	-1.032
JCVI_40999	1.044	no original description	
ES940539	1.043	weakly similar to (159)AT4G11860| Symbols: | similar to unknown protein [Arabidopsis thaliana] (TAIR:AT4G22960.1); similar to unna	-1.587
JCVI_13060	1.043	very weakly similar to (97.4)AT5G58375| Symbols: | similar to unknown protein [Arabidopsis thaliana] (TAIR:AT5G14602.1); similar to u	
AM393889	1.043	weakly similar to (144)AT3G52250| Symbols: | DNA binding / transcription factor | chr3:19387607-19394078 FORWARD [20346]	
JCVI_35195	1.043	moderately similar to (461)AT4G04890| Symbols: PDF2 | PDF2 (PROTODERMAL FACTOR2); DNA binding / transcription factor | chr4	
JCVI_20129	1.043	moderately similar to (434)AT3G16740| Symbols: | F-box family protein | chr3:5699482-5700657 FORWARD no original description	
JCVI_14100	1.043	moderately similar to (340)AT5G07580| Symbols: | DNA binding / transcription factor | chr5:2399526-2400350 FORWARDweakly simila	
EE521915	1.042	weakly similar to (141)AT3G10860| Symbols: | ubiquinol-cytochrome C reductase complex ubiquinone-binding protein, putative / ubiquin	
JCVI_26883	1.042	moderately similar to (275)AT3G60590| Symbols: | similar to unknown protein [Arabidopsis thaliana] (TAIR:AT1G48460.1); similar to u	
JCVI_35795	1.042	no original description	
AM390312	1.042	no similarity	
JCVI_15092	1.042	no original description	
JCVI_34534	1.042	moderately similar to (400)AT1G28380| Symbols: NSL1 | NSL1 (NECROTIC SPOTTED LESIONS 1) | chr1:9963682-9966046 FORWAI	
DY030586	1.042	moderately similar to (327)AT4G28500| Symbols: ANAC073 | ANAC073 (Arabidopsis NAC domain containing protein 73); transcription	
JCVI_21376	1.041	moderately similar to (274)AT3G47700| Symbols: MAG2 | MAG2 (MAIGO2) | chr3:17594758-17597563 REVERSE no original descripti	
JCVI_18688	1.041	moderately similar to (318)AT5G10730| Symbols: | binding / catalytic/ coenzyme binding | chr5:3390823-3392948 REVERSE no original	
JCVI_36000	1.041	highly similar to (575)AT1G15910| Symbols: | XH/XS domain-containing protein / XS zinc finger domain-containing protein | chr1:54659	
AM389056	1.041	no similarity	
ES911488	1.041	moderately similar to (327)AT4G19140| Symbols: | similar to unnamed protein product [Vitis vinifera] (GB:CAO66715.1) | chr4:1046984	
JCVI_23589	1.041	moderately similar to (366)AT1G71500| Symbols: | Rieske (2Fe-2S) domain-containing protein | chr1:26939746-26940993 FORWARD n	
EX030449	1.040	no similarity	
JCVI_4028	1.040	moderately similar to (239)AT4G31720| Symbols: TAF10, STG1, TAFII15 | TAFII15 (SALT TOLERANCE DURING GERMINATION 1	
EV087808	1.040	no similarity	
JCVI_15718	1.040	moderately similar to (347)AT3G10200| Symbols: | dehydration-responsive protein-related | chr3:3157623-3160021 FORWARD no origin	
JCVI_28298	1.040	moderately similar to (220)AT2G20635| Symbols: | similar to spindle checkpoint protein-related [Arabidopsis thaliana] (TAIR:AT2G3356	
CV546885	1.040	no similarity	
EV028472	1.040	weakly similar to (115)AT3G60260| Symbols: | phagocytosis and cell motility protein ELMO1-related | chr3:22285278-22287814 FORWA	-1.531
EE531020	1.039	weakly similar to (149)AT3G23070| Symbols: | similar to unknown protein [Arabidopsis thaliana] (TAIR:AT4G14510.1); similar to hypot	
JCVI_39539	1.039	moderately similar to (424)AT5G27540| Symbols: EMB2473 | EMB2473 (EMBRYO DEFECTIVE 2473); GTP binding | chr5:9722819-97	
ES968382	1.039	no similarity	
ES911311	1.039	moderately similar to (233)AT5G11250| Symbols: | disease resistance protein (TIR-NBS-LRR class), putative | chr5:3587979-3591961 RI	
EX130819	1.039	moderately similar to (272)AT5G32440| Symbols: | similar to unknown [Populus trichocarpa] (GB:ABK93674.1); contains InterPro doma	
EV222327	1.039	no similarity	
EV209169	1.039	no similarity	
EE558643	1.039	no similarity	
JCVI_29845	1.039	weakly similar to (107)AT3G17620| Symbols: | F-box family protein | chr3:6026504-6027700 REVERSE no original description	
EX100455	1.038	moderately similar to (311)AT5G05310| Symbols: | similar to unnamed protein product [Vitis vinifera] (GB:CAO41480.1); contains Inter	
EE559677	1.038	weakly similar to (159)AT2G39460| Symbols: RPL23A, ATRPL23A | ATRPL23A (RIBOSOMAL PROTEIN L23A); RNA binding / struc	
JCVI_21156	1.038	weakly similar to (154)AT4G38910| Symbols: BPC5, BBR/BPC5, ATBPC5 | ATBPC5/BBR/BPC5/BPC5 (BASIC PENTACYSTEINE 5)	
JCVI_23818	1.038	no original description	
JCVI_27013	1.038	weakly similar to (118)AT3G12600| Symbols: ATNUDT16 | ATNUDT16 (Arabidopsis thaliana Nudix hydrolase homolog 16) | chr3:4004	
JCVI_20898	1.038	very weakly similar to (95.5)AT3G53800| Symbols: | armadillo/beta-catenin repeat family protein | chr3:19941920-19943697 FORWARD	
H74571	1.038	no similarity	
EX131870	1.038	no similarity	
JCVI_21460	1.038	moderately similar to (325)AT5G51250| Symbols: | kelch repeat-containing F-box family protein | chr5:20847969-20849075 FORWARD	
EX096842	1.038	weakly similar to (170)AT5G01750| Symbols: | Identical to Uncharacterized protein At5g01750 [Arabidopsis Thaliana] (GB:Q9LZX1;GE	
JCVI_7022	1.037	moderately similar to (243)AT5G12020| Symbols: HSP17.6II | HSP17.6II (17.6 KDA CLASS II HEAT SHOCK PROTEIN) | chr5:388241	
EV089310	1.037	no similarity	
JCVI_23722	1.037	moderately similar to (298)AT5G04830| Symbols: | similar to unknown [Populus trichocarpa x Populus deltoides] (GB:ABK96633.1); con	1.366
JCVI_25903	1.037	moderately similar to (415)AT5G51340| Symbols: | binding | chr5:20880820-20884397 FORWARD no original description	
JCVI_2817	1.037	moderately similar to (244)AT2G38800| Symbols: | calmodulin-binding protein-related | chr2:16224077-16225915 FORWARD no origina	
JCVI_16599	1.037	moderately similar to (420)AT2G22300| Symbols: | ethylene-responsive calmodulin-binding protein, putative (SR1) | chr2:9478679-94835	
JCVI_13306	1.037	moderately similar to (453)AT3G22110| Symbols: PAC1 | PAC1 (20S proteasome alpha subunit C1); peptidase | chr3:7792826-7793578 R	
JCVI_6393	1.036	moderately similar to (350)AT3G12170| Symbols: | DNAJ heat shock N-terminal domain-containing protein | chr3:3881028-3882662 FOF	
JCVI_14234	1.036	moderately similar to (338)AT1G80380| Symbols: | phosphoribulokinase/uridine kinase-related | chr1:30222787-30224676 FORWARD n	
JCVI_27946	1.036	moderately similar to (391)AT5G52210| Symbols: ATARLB1, ATGB1 | ATARLB1/ATGB1 (ARABIDOPSIS THALIANA GTP-BINDIN	
JCVI_2361	1.036	moderately similar to (247)AT5G57040| Symbols: | lactoylglutathione lyase family protein / glyoxalase I family protein | chr5:23101261-2	
JCVI_31752	1.036	weakly similar to (137)AT3G44300| Symbols: NIT2 | NIT2 (NITRILASE 2) | chr3:15994338-15996159 FORWARDvery weakly similar to	
ES902478	1.035	weakly similar to (122)AT3G27050| Symbols: | similar to unnamed protein product [Vitis vinifera] (GB:CAO49017.1) | chr3:9979523-998	
JCVI_35772	1.035	no original description	
JCVI_39975	1.035	moderately similar to (457)AT4G25080| Symbols: CHLM | CHLM (MAGNESIUM-PROTOPORPHYRIN IX METHYLTRANSFERASE	
JCVI_5984	1.035	weakly similar to (177)AT1G11430| Symbols: | plastid developmental protein DAG, putative | chr1:3847273-3848938 FORWARDweakly	
JCVI_693	1.034	moderately similar to (234)AT5G27395| Symbols: | P-P-bond-hydrolysis-driven protein transmembrane transporter | chr5:9671848-96740	
DY020485	1.034	no similarity	
EX043115	1.034	moderately similar to (323)AT2G16750| Symbols: | protein kinase family protein | chr2:7278868-7281528 FORWARDweakly similar to (	-1.928
EV026423	1.034	moderately similar to (245)AT2G15490| Symbols: UGT73B4 | UGT73B4; UDP-glycosyltransferase/ transferase, transferring glycosyl grou	
JCVI_1342	1.033	moderately similar to (223)AT4G08520| Symbols: | clathrin adaptor complex small chain family protein | chr4:5417884-5420292 FORWA	
EV194077	1.033	weakly similar to (166)AT5G40810| Symbols: | cytochrome c1, putative | chr5:16357428-16359555 FORWARDweakly similar to (158)C	
JCVI_5376	1.033	moderately similar to (315)AT1G69410| Symbols: | eukaryotic translation initiation factor 5A, putative / eIF-5A, putative | chr1:26092964	
CD836329	1.033	no similarity	
EE448815	1.033	moderately similar to (228)AT1G15750| Symbols: WSIP1, TPL | TPL/WSIP1 (WUS-INTERACTING PROTEIN 1) | chr1:5415081-54203	1.246

JCVI_41481	1.033	no original description	
AM059823	1.033	no similarity	
EE483256	1.032	moderately similar to (218)AT5G63180| Symbols: | pectate lyase family protein | chr5:25358332-25360339 REVERSEvery weakly similar	
JCVI_13963	1.032	weakly similar to (147)AT1G65720| Symbols: | similar to hypothetical protein [Vitis vinifera] (GB:CAN83711.1); similar to unnamed pro	
JCVI_32226	1.032	weakly similar to (154)AT1G06290| Symbols: ATACX3, ACX3 | ACX3 (ACYL-COA OXIDASE 3); acyl-CoA oxidase | chr1:1922422-19	
JCVI_17686	1.032	highly similar to (503)AT1G07360| Symbols: | zinc finger (CCCH-type) family protein / RNA recognition motif (RRM)-containing protein	
JCVI_12076	1.032	moderately similar to (414)AT3G05050| Symbols: | protein kinase family protein | chr3:1408795-1411200 REVERSEweakly similar to (1	
JCVI_14935	1.031	moderately similar to (302)AT4G28660| Symbols: PSB28 | PSB28 (PHOTOSYSTEM II REACTION CENTER PSB28 PROTEIN) | chr4:	
JCVI_3302	1.031	moderately similar to (289)AT2G18290| Symbols: | anaphase-promoting complex, subunit 10 family / APC10 family | chr2:7955604-7957	
ES952643	1.031	weakly similar to (135)AT5G41020| Symbols: | myb family transcription factor | chr5:16443111-16444877 REVERSE [21423]	
JCVI_17464	1.031	highly similar to (729)AT1G03190| Symbols: ATXPD, UVH6 | ATXPD/UVH6 (ULTRAVIOLET HYPERSENSITIVE 6); ATP binding /	
JCVI_17578	1.031	weakly similar to (106)AT3G09390| Symbols: ATMT-K, ATMT-1, MT2, MT2A | MT2A (METALLOTHIONEIN 2A) | chr3:2889743-28	
JCVI_29952	1.031	moderately similar to (410)AT2G31450| Symbols: | endonuclease-related | chr2:13408395-13411211 REVERSE no original description	
JCVI_18801	1.031	weakly similar to (131)AT5G43970| Symbols: TOM9-2, ATTOM22-V, TOM22-V | TOM22-V (TRANSLOCASE OUTER MITOCHOND	
DY001271	1.031	moderately similar to (258)AT3G25800| Symbols: PR 65, PR65, PDF1 | PDF1 (65 KDA REGULATORY SUBUNIT OF PROTEIN PHOS	
JCVI_26944	1.031	moderately similar to (318)AT4G14190| Symbols: | binding | chr4:8183589-8185175 REVERSE no original description	
EV130420	1.030	no similarity	
JCVI_6030	1.030	moderately similar to (277)AT4G13830| Symbols: J20 | J20 (DNAJ-LIKE 20); heat shock protein binding | chr4:8011514-8012573 FORW	
EX132454	1.030	moderately similar to (281)AT2G47260| Symbols: ATWRKY23, WRKY23 | WRKY23 (WRKY DNA-binding protein 23); transcription fa	
EV004444	1.030	no similarity	
ES913100	1.030	weakly similar to (191)AT1G32470| Symbols: | glycine cleavage system H protein, mitochondrial, putative | chr1:11739459-11740226 RE	
JCVI_31026	1.030	weakly similar to (135)AT3G44750| Symbols: HDT1, HD2A, HDA3, ATHD2A | HD2A (HISTONE DEACETYLASE 2A); nucleic acid bi	
EV061516	1.030	no similarity	
EE543914	1.030	no similarity	
JCVI_16217	1.030	moderately similar to (288)AT3G28730| Symbols: SSRP1, NFD, ATHMG | ATHMG (HIGH MOBILITY GROUP); transcription factor | c	
JCVI_34881	1.030	moderately similar to (457)AT2G16880| Symbols: | pentatricopeptide (PPR) repeat-containing protein | chr2:7319344-7321575 REVERSI	
EV102027	1.029	very weakly similar to (100)AT2G23000| Symbols: SCPL10 | SCPL10 (serine carboxypeptidase-like 10); serine carboxypeptidase | chr2:97	
JCVI_28245	1.029	no original description	
JCVI_26550	1.029	moderately similar to (303)AT1G34630| Symbols: | similar to unknown protein [Arabidopsis thaliana] (TAIR:AT5G51150.1); similar to C	
JCVI_29163	1.029	moderately similar to (289)AT5G46760| Symbols: | basic helix-loop-helix (bHLH) family protein | chr5:18991458-18993236 FORWARD	
CV434058	1.029	very weakly similar to (98.6)AT4G25320| Symbols: | DNA-binding protein-related | chr4:12954498-12956352 FORWARD [16490]	
JCVI_3394	1.029	moderately similar to (376)AT5G10860| Symbols: | CBS domain-containing protein | chr5:3429174-3430143 REVERSE no original descr	
EV158484	1.029	moderately similar to (292)AT5G57240| Symbols: | oxysterol-binding family protein | chr5:23210675-23212529 FORWARD [21484]	
CX281030	1.028	weakly similar to (123)AT1G26850| Symbols: | dehydration-responsive family protein | chr1:9301133-9303419 REVERSE [16818]	
EV066033	1.028	no similarity	
JCVI_8100	1.028	moderately similar to (287)AT1G50900| Symbols: | similar to hypothetical protein [Vitis vinifera] (GB:CAN65357.1); similar to unnamed	
JCVI_25355	1.028	no original description	
JCVI_36404	1.027	highly similar to (558)AT4G30560| Symbols: CNGC9, ATCNGC9 | ATCNGC9 (CYCLIC NUCLEOTIDE GATED CHANNEL 9); calmo	
JCVI_15889	1.027	moderately similar to (306)AT1G65690| Symbols: | harpin-induced protein-related / HIN1-related / harpin-responsive protein-related | chr	1.393
EX098402	1.027	moderately similar to (480)AT5G02030| Symbols: HB-6, RPL, PNY, BLR, BLH9, VAN, LSN | LSN (LARSON); DNA binding / transcrip	
JCVI_40273	1.027	weakly similar to (199)AT2G40880| Symbols: FL3-27 | FL3-27; cysteine protease inhibitor | chr2:17064541-17065008 FORWARDweakly	
ES919140	1.027	weakly similar to (148)AT2G46090| Symbols: | diacylglycerol kinase family protein | chr2:18957992-18960152 FORWARD [15718]	
ES964123	1.027	no similarity	
EH429480	1.027	weakly similar to (125)AT1G76700| Symbols: | DNAJ heat shock N-terminal domain-containing protein | chr1:28785513-28787916 REVI	
JCVI_6539	1.027	weakly similar to (126)AT4G10080| Symbols: | similar to unknown protein [Arabidopsis thaliana] (TAIR:AT4G13530.1); similar to unna	-1.071
JCVI_7236	1.026	moderately similar to (466)AT2G35780| Symbols: SCPL26 | SCPL26 (serine carboxypeptidase-like 26); serine carboxypeptidase | chr2:15	
ES942982	1.026	moderately similar to (299)AT2G42070| Symbols: ATNUDT23 | ATNUDT23 (Arabidopsis thaliana Nudix hydrolase homolog 23); hydrol	
EV124463	1.025	moderately similar to (258)AT1G67730| Symbols: YBR159 | YBR159; ketoreductase/ oxidoreductase | chr1:25395339-25397028 FORWA	
JCVI_9442	1.025	moderately similar to (257)AT2G14610| Symbols: PR-1, PR1 | PR1 (PATHOGENESIS-RELATED GENE 1) | chr2:6249026-6249511 RE	
ES992902	1.025	weakly similar to (190)AT2G04650| Symbols: | ADP-glucose pyrophosphorylase family protein | chr2:1621983-1624483 REVERSE [214	
JCVI_22246	1.024	moderately similar to (375)AT1G76810| Symbols: | eukaryotic translation initiation factor 2 family protein / eIF-2 family protein | chr1:28	
JCVI_3959	1.024	moderately similar to (224)AT1G74910| Symbols: | ADP-glucose pyrophosphorylase family protein | chr1:28139431-28142117 REVERSI	
JCVI_31066	1.024	moderately similar to (287)AT3G55470| Symbols: | C2 domain-containing protein | chr3:20575334-20577070 FORWARDweakly similar	
CV432387	1.024	weakly similar to (142)AT1G55490| Symbols: LEN1, CPN60B | CPN60B (CHAPERONIN 60 BETA); ATP binding / protein binding / un	1.090
JCVI_34645	1.023	no original description	
JCVI_40010	1.023	no original description	
EE550081	1.023	weakly similar to (145)AT1G28540| Symbols: | similar to Os06g0524500 [Oryza sativa (japonica cultivar-group)] (GB:NP_001057759.1)	
EX118276	1.023	highly similar to (540)AT5G13640| Symbols: ATPDAT | ATPDAT (Arabidopsis thaliana phospholipid:diacylglycerol acyltransferase); pho	
EV009150	1.023	weakly similar to (154)AT1G19580| Symbols: GAMMA CA1 | GAMMA CA1 (GAMMA CARBONIC ANHYDRASE 1); carbonate dehy	
JCVI_11154	1.023	weakly similar to (108)AT5G03500| Symbols: | transcription coactivator | chr5:876672-877659 REVERSE no original description	
EV013609	1.023	weakly similar to (144)AT2G32990| Symbols: ATGH9B8 | ATGH9B8 (ARABIDOPSIS THALIANA GLYCOSYL HYDROLASE 9B8); h	
EV090391	1.023	moderately similar to (301)AT3G02980| Symbols: | GCN5-related N-acetyltransferase (GNAT) family protein | chr3:670904-672222 REV	
EX108062	1.022	no similarity	
JCVI_1555	1.022	weakly similar to (193)AT3G32930| Symbols: | similar to unnamed protein product [Vitis vinifera] (GB:CAO22035.1) | chr3:13493308-13	
DN962837	1.022	moderately similar to (362)AT3G03305| Symbols: | calcineurin-like phosphoesterase family protein | chr3:775511-778278 REVERSE [17	
JCVI_24616	1.022	weakly similar to (194)AT5G14890| Symbols: | NHL repeat-containing protein | chr5:4818059-4821537 FORWARD no original descriptio	
JCVI_7286	1.022	moderately similar to (390)AT4G08790| Symbols: | nitrilase, putative | chr4:5608486-5611115 REVERSE no original description	
EV214579	1.022	weakly similar to (114)AT2G24090| Symbols: | ribosomal protein L35 family protein | chr2:10249118-10250496 FORWARDvery weakly	
JCVI_34216	1.021	moderately similar to (248)AT2G35700| Symbols: | AP2 domain-containing transcription factor, putative | chr2:15012284-15012868 FOR	
JCVI_10302	1.021	moderately similar to (368)AT1G33030| Symbols: | O-methyltransferase family 2 protein | chr1:11964865-11966192 REVERSEmoderate	
JCVI_11875	1.021	no original description	-1.359
JCVI_5013	1.021	highly similar to (717)AT1G17110| Symbols: UBP15 | UBP15 (UBIQUITIN-SPECIFIC PROTEASE 15); ubiquitin-specific protease | chr	
JCVI_7401	1.021	moderately similar to (286)AT5G13190| Symbols: | similar to unknown [Populus trichocarpa] (GB:ABK93196.1); contains InterPro doma	-1.474
EV117919	1.021	moderately similar to (317)AT4G19960| Symbols: | potassium ion transmembrane transporter | chr4:10813817-10817007 FORWARDmod	
JCVI_352	1.021	moderately similar to (315)AT3G57340| Symbols: | DNAJ heat shock N-terminal domain-containing protein | chr3:21230153-21231256 F	
JCVI_10687	1.021	no original description	
JCVI_17654	1.020	moderately similar to (388)AT5G19140| Symbols: | auxin/aluminum-responsive protein, putative | chr5:6423400-6425787 FORWARDwe	
RC_ES968271	1.020	no similarity	
JCVI_6641	1.020	very weakly similar to (88.2)AT1G21900| Symbols: | emp24/gp25L/p24 family protein | chr1:7691154-7692316 REVERSE no original des	
JCVI_2740	1.020	no original description	
JCVI_38950	1.020	moderately similar to (329)AT2G23580| Symbols: | hydrolase, alpha/beta fold family protein | chr2:10040440-10041390 REVERSEweakl	

JCVI_6700	1.020	moderately similar to (278)AT4G37510| Symbols: | ribonuclease III family protein | chr4:17626253-17628848 REVERSE no original desc	
AM394404	1.020	no similarity	
JCVI_6989	1.020	moderately similar to (270)AT2G26140| Symbols: FTSH4 | FTSH4 (FtsH protease 4); ATP-dependent peptidase/ ATPase/ metallopeptidas	
EE556160	1.019	moderately similar to (289)AT5G06600| Symbols: UBP12 | UBP12 (UBIQUITIN-SPECIFIC PROTEASE 12) | chr5:2020683-2027835 RI	
JCVI_27557	1.019	moderately similar to (336)AT3G30390| Symbols: | amino acid transporter family protein | chr3:11979591-11981306 REVERSE no origin	
EE502670	1.019	no similarity	
JCVI_17409	1.018	weakly similar to (152)AT1G25380| Symbols: | mitochondrial substrate carrier family protein | chr1:8903713-8905805 FORWARD no ori	
JCVI_17199	1.018	no original description	
JCVI_8768	1.018	weakly similar to (179)AT3G19910| Symbols: | zinc finger (C3HC4-type RING finger) family protein | chr3:6926503-6929330 FORWAR	
JCVI_21187	1.018	weakly similar to (148)AT1G22050| Symbols: MUB6 | MUB6 (MEMBRANE-ANCHORED UBIQUITIN-FOLD PROTEIN 6 PRECURS	
JCVI_416	1.018	moderately similar to (307)AT1G69840| Symbols: | band 7 family protein | chr1:26297595-26298813 REVERSE no original description	
JCVI_4018	1.018	weakly similar to (124)AT1G19000| Symbols: | myb family transcription factor | chr1:6561326-6562675 REVERSE no original descriptio	
CX266050	1.017	no similarity	
JCVI_38818	1.017	weakly similar to (163)AT3G12250| Symbols: BZIP45, TGA6 | TGA6 (TGA1a-related gene 6) | chr3:3906643-3908590 FORWARDweakl	
JCVI_39926	1.017	no original description	
JCVI_26589	1.017	moderately similar to (295)AT5G58660| Symbols: | oxidoreductase, 2OG-Fe(II) oxygenase family protein | chr5:23718735-23721028 FOI	
JCVI_39989	1.016	moderately similar to (387)AT1G27740| Symbols: | basic helix-loop-helix (bHLH) family protein | chr1:9654740-9655793 FORWARD nc	
EX099379	1.016	moderately similar to (208)AT3G11964| Symbols: | S1 RNA-binding domain-containing protein | chr3:3793963-3806632 REVERSE [218	
JCVI_37397	1.016	moderately similar to (379)AT3G49060| Symbols: | protein kinase family protein / U-box domain-containing protein | chr3:18198371-182	
EE568128	1.016	no similarity	
JCVI_4122	1.016	moderately similar to (327)AT4G12590| Symbols: | similar to hypothetical protein [Vitis vinifera] (GB:CAN72892.1); contains InterPro d	
EE567763	1.016	very weakly similar to (85.9)AT1G10640| Symbols: | polygalacturonase, putative / pectinase, putative | chr1:3515479-3516932 REVERSE	
EE527722	1.016	moderately similar to (327)AT4G38180| Symbols: FRS5 | FRS5 (FAR1-RELATED SEQUENCE 5); zinc ion binding | chr4:17906696-179	
JCVI_38872	1.016	moderately similar to (350)AT3G54250| Symbols: | mevalonate diphosphate decarboxylase, putative | chr3:20093446-20095666 REVERS	
JCVI_1698	1.015	weakly similar to (174)AT1G11480| Symbols: | eukaryotic translation initiation factor-related | chr1:3864368-3866707 REVERSE no orig	-1.730
EV057774	1.015	no similarity	
AM057995	1.014	moderately similar to (335)AT5G02260| Symbols: EXP9, ATEXP9, ATHEXP ALPHA 1.10, ATEXPA9 | ATEXPA9 (ARABIDOPSIS TH	
JCVI_17353	1.014	moderately similar to (445)AT4G39680| Symbols: | SAP domain-containing protein | chr4:18414598-18416932 REVERSE no original des	
DY003396	1.014	no similarity	
EV003450	1.014	no similarity	
EV137142	1.013	no similarity	
EX103196	1.013	weakly similar to (169)AT2G24220| Symbols: ATPUP5 | ATPUP5 (Arabidopsis thaliana purine permease 5); purine transmembrane transp	
RC_H07158	1.013	no similarity	
JCVI_36	1.013	moderately similar to (370)AT1G61520| Symbols: LHCA3 | LHCA3 (Photosystem I light harvesting complex gene 3) | chr1:22704158-227	
EV089714	1.013	no similarity	
JCVI_10761	1.013	moderately similar to (309)AT2G03690| Symbols: | coenzyme Q biosynthesis Coq4 family protein / ubiquinone biosynthesis Coq4 family	-1.165
JCVI_12900	1.013	highly similar to (606)AT5G51430| Symbols: | conserved oligomeric Golgi complex component-related / COG complex component-relate	
EX072990	1.013	moderately similar to (483)AT2G15730| Symbols: | similar to unknown protein [Arabidopsis thaliana] (TAIR:AT4G34420.1); similar to u	
JCVI_21527	1.012	very weakly similar to (88.6)AT1G24145| Symbols: | unknown protein | chr1:8541029-8541605 FORWARD no original description	
JCVI_24662	1.012	weakly similar to (104)AT4G36920| Symbols: FLO2, FL1, AP2 | AP2 (APETALA 2); transcription factor | chr4:17400995-17403137 FOR	
EV098998	1.012	no similarity	
EV084116	1.012	no similarity	
JCVI_37155	1.012	moderately similar to (233)AT3G04970| Symbols: | zinc finger (DHHC type) family protein | chr3:1376246-1378016 FORWARD no origi	
EX028317	1.012	weakly similar to (141)AT5G44190| Symbols: ATGLK2, GPRI2, GLK2 | GLK2 (GOLDEN2-LIKE 2); DNA binding / transcription factor	
EX090803	1.012	moderately similar to (297)AT3G47820| Symbols: | armadillo/beta-catenin repeat family protein / U-box domain-containing protein | chr3	1.121
EV042060	1.011	moderately similar to (352)AT2G34780| Symbols: EMB1611, MEE22 | EMB1611/MEE22 (EMBRYO DEFECTIVE 1611); binding | chr2	
JCVI_27528	1.011	weakly similar to (109)AT1G30620| Symbols: HSR8, MUR4, UXE1 | HSR8/MUR4/UXE1 (MURUS 4) | chr1:10855478-10857952 FORW	
JCVI_19815	1.011	no original description	
JCVI_21503	1.011	moderately similar to (311)AT5G46840| Symbols: | RNA recognition motif (RRM)-containing protein | chr5:19023289-19024836 FORW.	
JCVI_11942	1.011	no original description	
EE452728	1.011	weakly similar to (155)AT5G05670| Symbols: | signal recognition particle binding | chr5:1695917-1697535 REVERSE [20194]	
JCVI_6505	1.011	weakly similar to (176)AT5G22880| Symbols: H2B, HTB2 | H2B/HTB2 (HISTONE H2B); DNA binding | chr5:7652133-7652570 REVEI	
JCVI_39348	1.011	moderately similar to (409)AT2G43000| Symbols: ANAC042 | ANAC042 (Arabidopsis NAC domain containing protein 42); transcription	
AM395669	1.011	moderately similar to (252)AT4G21065| Symbols: | binding | chr4:11246386-11247774 FORWARD [20346]	
EV186947	1.011	weakly similar to (193)AT1G06430| Symbols: FTSH8 | FTSH8 (FtsH protease 8); ATP-dependent peptidase/ ATPase/ metallopeptidase/ z	
JCVI_22781	1.011	moderately similar to (207)AT3G48460| Symbols: | GDSL-motif lipase/hydrolase family protein | chr3:17960481-17962067 FORWARD r	
JCVI_18719	1.010	highly similar to (749)AT2G16430| Symbols: PAP10, ATPAP10 | ATPAP10/PAP10; acid phosphatase/ protein serine/threonine phosphata	
JCVI_3566	1.010	moderately similar to (276)AT5G46720| Symbols: | similar to unknown protein [Arabidopsis thaliana] (TAIR:AT3G02910.1); similar to C	
EV218573	1.010	no similarity	-2.275
EE421261	1.010	weakly similar to (147)AT2G34050| Symbols: | similar to unnamed protein product [Vitis vinifera] (GB:CAO66363.1); contains InterPro	
EL590288	1.010	no similarity	
ES935709	1.009	weakly similar to (155)AT1G16830| Symbols: | pentatricopeptide (PPR) repeat-containing protein | chr1:5758870-5762612 FORWARD [	
EX138019	1.009	weakly similar to (104)AT1G68490| Symbols: | similar to unknown protein [Arabidopsis thaliana] (TAIR:AT1G13390.2); similar to unkno	
EE462981	1.009	no similarity	
DY025592	1.009	moderately similar to (298)AT1G72560| Symbols: PSD | PSD (PAUSED) | chr1:27328688-27333129 REVERSE [18971] 16 797 797	
JCVI_5070	1.008	moderately similar to (376)AT3G62600| Symbols: | DNAJ heat shock family protein | chr3:23162014-23164322 REVERSEvery weakly si	
EE478212	1.008	weakly similar to (130)AT4G38800| Symbols: ATMTN1 | ATMTN1; catalytic/ methylthioadenosine nucleosidase | chr4:18113349-18114	
CX194358	1.008	moderately similar to (420)AT5G65440| Symbols: | similar to unknown protein [Arabidopsis thaliana] (TAIR:AT4G24610.1); similar to u	
AM061512	1.008	no similarity	
JCVI_30398	1.008	weakly similar to (171)AT4G01840| Symbols: ATTPK5, KCO5, ATKCO5 | KCO5 (Ca2+ activated outward rectifying K+ channel 5); out	
EE419729	1.007	weakly similar to (123)AT1G08980| Symbols: ATTOC64-I, AMI1, ATAMI1 | ATAMI1 (AMIDASE-LIKE PROTEIN 1); amidase | chr1:2	
JCVI_26538	1.007	moderately similar to (428)AT1G18250| Symbols: ATLP-1 | ATLP-1 (Arabidopsis thaumatin-like protein 1) | chr1:6277017-6277998 REV	
EV033230	1.007	moderately similar to (203)AT1G20860| Symbols: | phosphate transporter family protein | chr1:7253996-7258666 REVERSE [21441]	
JCVI_15411	1.007	moderately similar to (315)AT2G32170| Symbols: | similar to unknown protein [Arabidopsis thaliana] (TAIR:AT2G32160.2); similar to u	
RC_ES899171	1.006	no similarity	
EX132693	1.006	very weakly similar to (93.6)AT1G77850| Symbols: ARF17 | ARF17 (AUXIN RESPONSE FACTOR 17); transcription factor | chr1:29277	
JCVI_25578	1.006	highly similar to (658)AT2G24640| Symbols: | ubiquitin carboxyl-terminal hydrolase family protein / zinc finger (MYND type) family pro	
EE473239	1.005	weakly similar to (130)AT5G24540| Symbols: | glycosyl hydrolase family 1 protein | chr5:8384879-8388030 REVERSE [20163]	
EX128399	1.004	highly similar to (502)AT1G31070| Symbols: | UDP-N-acetylglucosamine pyrophosphorylase-related | chr1:11084932-11088342 FORWA	
AM395461	1.004	moderately similar to (257)AT4G24840| Symbols: | similar to unnamed protein product [Vitis vinifera] (GB:CAO68282.1); similar to hyp	
EX054895	1.004	moderately similar to (293)AT3G05970| Symbols: LACS6 | LACS6 (LONG-CHAIN ACYL-COA SYNTHETASE 6) | chr3:1786516-1791	

EV048763	1.004	moderately similar to (249)AT5G58190| Symbols: ECT10 | ECT10 | chr5:23563660-23566589 FORWARD [21442]	
JCVI_9758	1.004	moderately similar to (206)AT3G44020| Symbols: | thylakoid lumenal P17.1 protein | chr3:15812369-15813548 FORWARD no original d	
JCVI_36912	1.003	moderately similar to (347)AT5G47150| Symbols: | YDG/SRA domain-containing protein | chr5:19168034-19169020 FORWARD no orig	
EV150424	1.003	weakly similar to (114)AT1G47240| Symbols: ATNRAMP2, NRAMP2 | NRAMP2 (NRAMP metal ion transporter 2); metal ion transmem	-2.104
EE531986	1.003	very weakly similar to (98.6)AT1G32660| Symbols: | F-box family protein | chr1:11811020-11812360 FORWARD [20175]	2.015
JCVI_26014	1.003	highly similar to (558)AT5G59540| Symbols: | oxidoreductase, 2OG-Fe(II) oxygenase family protein | chr5:24013841-24014802 REVERS	
EV180013	1.003	moderately similar to (230)AT5G56460| Symbols: | protein kinase, putative | chr5:22882735-22885092 FORWARD [21487]	
JCVI_12592	1.002	moderately similar to (258)AT2G40060| Symbols: | protein binding / protein transporter/ structural molecule | chr2:16733642-16735079 F	1.096
JCVI_3791	1.002	weakly similar to (196)AT5G26210| Symbols: | PHD finger family protein | chr5:9158569-9160224 REVERSE no original description	
EV104189	1.002	very weakly similar to (82.8)AT5G47970| Symbols: | nitrogen regulation family protein | chr5:19440646-19442811 FORWARD [21477]	
JCVI_5788	1.002	moderately similar to (290)AT5G14000| Symbols: ANAC084 | ANAC084 (Arabidopsis NAC domain containing protein 84); transcription	
EX018778	1.002	weakly similar to (154)AT4G02390| Symbols: APP | APP (ARABIDOPSIS POLY(ADP-RIBOSE) POLYMERASE); NAD+ ADP-ribosylt	1.193
ES913136	1.002	weakly similar to (132)AT1G10690| Symbols: | similar to unknown protein [Arabidopsis thaliana] (TAIR:AT1G60783.1); similar to hypot	
EV003666	1.002	very weakly similar to (93.6)AT3G07020| Symbols: | UDP-glucose:sterol glucosyltransferase (UGT80A2) | chr3:2217847-2221596 REVE	1.121
AM387829	1.001	weakly similar to (125)AT1G48420| Symbols: D-CDES | D-CDES (D-CYSTEINE DESULFHYDRASE); 1-aminocyclopropane-1-carboxy	-1.129
ES993030	1.001	moderately similar to (245)AT1G27410| Symbols: | DNA cross-link repair protein-related | chr1:9517213-9518730 FORWARD [21427]	
EX087437	1.001	moderately similar to (216)AT1G62120| Symbols: | mitochondrial transcription termination factor-related / mTERF-related | chr1:2296399	
DY008207	1.001	no similarity	-1.197
JCVI_32130	1.001	moderately similar to (412)AT5G18470| Symbols: | curculin-like (mannose-binding) lectin family protein | chr5:6127954-6129195 FORW	
EE531579	1.001	moderately similar to (272)AT1G15780| Symbols: | protein binding / transcription cofactor | chr1:5430441-5435916 REVERSE [20175]	
JCVI_4367	1.001	moderately similar to (495)AT2G39750| Symbols: | dehydration-responsive family protein | chr2:16586064-16589359 REVERSE no origi	
JCVI_22005	1.001	weakly similar to (148)AT4G30993| Symbols: | similar to hypothetical protein [Vitis vinifera] (GB:CAN67945.1); contains domain Metall	
EV225869	1.000	very weakly similar to (86.3)AT1G47128| Symbols: RD21A, RD21 | RD21 (RESPONSIVE TO DEHYDRATION 21); cysteine-type peptic	
EV046536	1.000	moderately similar to (273)AT1G33390| Symbols: | helicase domain-containing protein | chr1:12099718-12104088 REVERSE [21442]	
EV196161	1.000	moderately similar to (255)AT5G03555| Symbols: | permease, cytosine/purines, uracil, thiamine, allantoin family protein | chr5:898353-90	
JCVI_3871	0.999	highly similar to (716)AT4G17140| Symbols: | pleckstrin homology (PH) domain-containing protein | chr4:9613629-9636629 REVERSE r	
JCVI_21243	0.998	weakly similar to (171)AT2G29540| Symbols: ATRPAC14, ATRPC14 | ATRPC14 (Arabidopsis thaliana RNA polymerase I(A) and III(C)	
EV091967	0.997	weakly similar to (162)AT4G12010| Symbols: | disease resistance protein (TIR-NBS-LRR class), putative | chr4:7197319-7201387 REVE	1.215
EX133190	0.997	moderately similar to (305)AT1G07570| Symbols: APK1, APK1A | APK1A (Arabidopsis protein kinase 1A); kinase | chr1:2331366-23332	
EX106290	0.997	moderately similar to (266)AT4G37330| Symbols: CYP81D4 | CYP81D4 (cytochrome P450, family 81, subfamily D, polypeptide 4); oxyg	
EV223461	0.996	moderately similar to (271)AT3G14840| Symbols: | leucine-rich repeat family protein / protein kinase family protein | chr3:4988278-4993	
JCVI_36777	0.995	no original description	
EV198229	0.995	weakly similar to (187)AT1G78820| Symbols: | curculin-like (mannose-binding) lectin family protein / PAN domain-containing protein | c	
EE559076	0.992	no similarity	
ES929301	0.992	no similarity	
CD815952	0.990	no similarity	
BQ790896	0.990	no similarity	
JCVI_36942	0.987	very weakly similar to (84.0)AT5G54170| Symbols: | similar to CP5 [Arabidopsis thaliana] (TAIR:AT1G64720.1); similar to putative nodu	
JCVI_13430	0.983	moderately similar to (380)AT3G09210| Symbols: PTAC13 | PTAC13 (PLASTID TRANSCRIPTIONALLY ACTIVE13); transcription el	
EG020891	0.983	no similarity	
EV203044	0.983	weakly similar to (190)AT1G02150| Symbols: | pentatricopeptide (PPR) repeat-containing protein | chr1:408779-410433 FORWARD [21	
EX120853	0.982	no similarity	
JCVI_10798	0.981	moderately similar to (475)AT5G42090| Symbols: | similar to unknown protein [Arabidopsis thaliana] (TAIR:AT3G09570.1); similar to u	
JCVI_36203	0.979	moderately similar to (249)AT4G31570| Symbols: | similar to myosin-related [Arabidopsis thaliana] (TAIR:AT1G24460.1); similar to hyp	
JCVI_21648	0.973	moderately similar to (259)AT1G76010| Symbols: | nucleic acid binding | chr1:28532166-28534149 REVERSE no original description	
EX057755	0.966	weakly similar to (173)AT4G34610| Symbols: BLH6 | BLH6 (BELL1-LIKE HOMEODOMAIN 5) | chr4:16530551-16532503 REVERSE	
JCVI_16415	0.966	moderately similar to (206)AT2G25970| Symbols: | KH domain-containing protein | chr2:11078922-11082682 REVERSE no original desc	
ES992852	0.966	no similarity	
DY025113	0.962	no similarity	
EV206491	0.960	weakly similar to (120)AT1G29350| Symbols: | similar to kinase-related [Arabidopsis thaliana] (TAIR:AT1G29370.1); similar to unnamed	
JCVI_23060	0.960	moderately similar to (391)AT1G06690| Symbols: | aldo/keto reductase family protein | chr1:2049741-2052038 REVERSE no original des	
EV142835	0.957	no similarity	
EE472267	0.954	no similarity	
JCVI_26932	0.950	moderately similar to (222)AT4G28920| Symbols: | similar to unknown protein [Arabidopsis thaliana] (TAIR:AT4G29550.1); contains Int	
JCVI_5688	0.944	weakly similar to (189)AT5G14610| Symbols: | ATP binding / ATP-dependent helicase | chr5:4711274-4714716 FORWARDweakly simil	
AM389721	0.930	no similarity	
JCVI_39985	0.930	moderately similar to (244)AT1G15210| Symbols: PDR7, ATPDR7 | ATPDR7/PDR7 (PLEIOTROPIC DRUG RESISTANCE 7); ATPase	
EV100574	0.924	moderately similar to (412)AT2G46720| Symbols: HIC | HIC (HIGH CARBON DIOXIDE); acyltransferase | chr2:19205891-19207291 FO	
JCVI_24132	0.920	no original description	
JCVI_41429	0.920	moderately similar to (269)AT4G17540| Symbols: | similar to hypothetical protein OsI_033497 [Oryza sativa (indica cultivar-group)] (GB	
EV020283	0.908	moderately similar to (425)AT3G50660| Symbols: CYP90B1, CLM, SNP2, DWF4 | DWF4 (DWARF 4) | chr3:18825243-18828149 REVE	
JCVI_11481	0.899	no original description	
JCVI_22064	0.882	moderately similar to (287)AT1G09760| Symbols: U2A' | U2A' (U2 small nuclear ribonucleoprotein A); protein binding | chr1:3159478-31	
BQ704677	0.881	weakly similar to (183)AT1G56140| Symbols: | leucine-rich repeat family protein / protein kinase family protein | chr1:21005373-2101139	
JCVI_15406	0.878	moderately similar to (344)AT1G30220| Symbols: ATINT2 | ATINT2 (INOSITOL TRANSPORTER 2); carbohydrate transmembrane tran	
DY003258	0.875	no similarity	
EE557281	0.861	no similarity	
JCVI_4038	0.860	moderately similar to (220)AT5G19250| Symbols: | Identical to Uncharacterized GPI-anchored protein At5g19250 precursor [Arabidopsis	
JCVI_5153	0.858	moderately similar to (340)AT2G24765| Symbols: ARF3, ARL1, ATARL1 | ARF3/ARL1/ATARL1 (ADP-RIBOSYLATION FACTOR 3)	
CX269955	0.825	moderately similar to (274)AT5G27710| Symbols: | similar to unnamed protein product [Vitis vinifera] (GB:CAO17522.1) | chr5:9813073	
JCVI_39963	0.815	moderately similar to (365)AT1G79430| Symbols: WDY, APL | APL (ALTERED PHLOEM DEVELOPMENT); transcription factor | chr1	
EV173758	0.802	no similarity	
JCVI_9247	0.801	highly similar to (800)AT5G10690| Symbols: | pentatricopeptide (PPR) repeat-containing protein / CBS domain-containing protein | chr5:	
JCVI_15463	0.800	very weakly similar to (86.7)TRA1_MAIZE no original description	
JCVI_4629	0.797	no original description	
BQ704326	0.781	moderately similar to (238)AT5G19690| Symbols: STT3A | STT3A (STAUROSPORIN AND TEMPERATURE SENSITIVE 3-LIKE A);	
EV179139	0.779	moderately similar to (236)AT2G20120| Symbols: COV1 | COV1 (CONTINUOUS VASCULAR RING) | chr2:8694649-8696402 REVER	
JCVI_13353	0.770	weakly similar to (186)AT2G18520| Symbols: | pentatricopeptide (PPR) repeat-containing protein | chr2:8041118-8042374 REVERSE no	
JCVI_26525	0.762	moderately similar to (339)AT1G07010| Symbols: | calcineurin-like phosphoesterase family protein | chr1:2152948-2154967 FORWARD	
JCVI_29564	0.755	moderately similar to (227)AT1G02816| Symbols: | similar to unknown protein [Arabidopsis thaliana] (TAIR:AT4G02370.1); similar to u	
JCVI_33007	0.745	no original description	

EX029032	0.728	weakly similar to (181)AT2G37678| Symbols: PAT3, FRY1, FHY1 | FHY1 (FAR-RED ELONGATED HYPOCOTYL 1) | chr2:15808767-	
JCVI_16762	0.725	moderately similar to (348)AT3G13080| Symbols: EST2, MRP3, ATMRP3 | ATMRP3 (Arabidopsis thaliana multidrug resistance-associat	
JCVI_11192	0.700	moderately similar to (384)AT5G18420| Symbols: | similar to unknown [Picea sitchensis] (GB:ABK25419.1); contains domain PTHR159	
JCVI_1299	0.691	moderately similar to (297)AT2G29300| Symbols: | tropinone reductase, putative / tropine dehydrogenase, putative | chr2:12595291-1259	
JCVI_1216	0.690	weakly similar to (119)AT5G57370| Symbols: | similar to unnamed protein product [Vitis vinifera] (GB:CAO43225.1); contains InterPro	
JCVI_18549	0.680	weakly similar to (183)AT3G04120| Symbols: GAPC-1, GAPC | GAPC (GLYCERALDEHYDE-3-PHOSPHATE DEHYDROGENASE C	
CD832512	0.680	no similarity	
JCVI_18644	0.662	moderately similar to (483)AT5G59730| Symbols: ATEXO70H7 | ATEXO70H7 (EXOCYST SUBUNIT EXO70 FAMILY PROTEIN H7)	
JCVI_15441	0.660	moderately similar to (360)AT2G32990| Symbols: ATGH9B8 | ATGH9B8 (ARABIDOPSIS THALIANA GLYCOSYL HYDROLASE 9B	
CX272425	0.624	no similarity	
JCVI_35436	0.613	very weakly similar to (87.4)AT1G53190| Symbols: | zinc finger (C3HC4-type RING finger) family protein | chr1:19841740-19843671 FO	
JCVI_24012	0.608	weakly similar to (187)AT3G54080| Symbols: | sugar binding | chr3:20036375-20037427 FORWARD no original description	
H07750	0.605	no similarity	
EE465688	0.565	no similarity	
EV074673	0.560	moderately similar to (280)AT5G47870| Symbols: | similar to unknown protein [Arabidopsis thaliana] (TAIR:AT1G71310.2); similar to u	
EE434657	0.549	moderately similar to (218)AT1G09870| Symbols: | histidine acid phosphatase family protein | chr1:3205819-3208446 FORWARD [1572	
JCVI_16657	0.549	highly similar to (508)AT5G10770| Symbols: | chloroplast nucleoid DNA-binding protein, putative | chr5:3403332-3405332 REVERSE no	
EX089740	0.548	moderately similar to (323)AT1G60430| Symbols: ARPC3 | ARPC3 (actin-related protein C3) | chr1:22268462-22269347 REVERSE [218	
JCVI_23267	0.527	moderately similar to (351)AT3G20240| Symbols: | mitochondrial substrate carrier family protein | chr3:7057198-7058722 FORWARDwe	
JCVI_32181	0.513	moderately similar to (272)AT5G15390| Symbols: | tRNA/rRNA methyltransferase (SpoU) family protein | chr5:4995336-4997258 FORW	
JCVI_14119	0.505	moderately similar to (318)AT4G22360| Symbols: | SWIB complex BAF60b domain-containing protein | chr4:11807823-11809672 FORW	
JCVI_25016	0.496	highly similar to (594)AT3G47780| Symbols: ATATH6 | ATATH6 (ABC2 homolog 6); ATPase, coupled to transmembrane movement of	1.849
EE408388	0.490	moderately similar to (216)AT3G50910| Symbols: | similar to unknown protein [Arabidopsis thaliana] (TAIR:AT5G66480.1); similar to h	
JCVI_270	0.480	moderately similar to (390)AT4G11010| Symbols: NDPK3 | NDPK3 (NUCLEOSIDE DIPHOSPHATE KINASE 3); ATP binding / nucleo	
AT000563	0.397	no similarity	
JCVI_2949	0.391	moderately similar to (220)AT5G40930| Symbols: TOM20, TOM20-4 | TOM20-4 (TRANSLOCASE OF OUTER MEMBRANE 20-4) | ch	
EV128660	0.355	no similarity	
JCVI_17875	0.310	moderately similar to (411)AT3G07180| Symbols: | GPI transamidase component PIG-S-related | chr3:2282461-2285481 REVERSE no o	
EV224641	0.295	no similarity	
JCVI_34843	0.271	weakly similar to (115)AT5G17890| Symbols: | LIM domain-containing protein / disease resistance protein-related | chr5:5917017-59231	
JCVI_41963	0.247	no original description	
RC_ES955671	-0.033	no similarity	
