# Supplementary material for: Influence of Light and Temperature on Gene Expression Leading to Accumulation of Specific Flavonol Glycosides and Hydroxycinnamic Acid Derivatives in Kale (Brassica oleracea var. sabellica)
Source: Front Plant Sci. 2016 Mar 30;7:326. doi: 10.3389/fpls.2016.00326 (PMC4812050; doi:10.3389/fpls.2016.00326)
Supplement: Supplementary file 3 [file Table3.PDF]

Identifier	log2-fold LT induced	Description	log2-fold LL-HL
JCVI_11085	6.816	highly similar to (613)AT2G15020| Symbols: | similar to unknown protein [Arabidopsis thaliana] (TAIR:AT5G64190.1); similar to hypo	5.216
EV198679	6.596	weakly similar to (176)AT2G15020| Symbols: | similar to unknown protein [Arabidopsis thaliana] (TAIR:AT5G64190.1); similar to hypo	5.412
EX041860	6.331	weakly similar to (138)AT1G02040| Symbols: | zinc finger (C2H2 type) family protein | chr1:358104-359078 REVERSE [21811]	3.434
ES967782	6.217	moderately similar to (249)AT5G38700| Symbols: | similar to unknown protein [Arabidopsis thaliana] (TAIR:AT4G02170.1); similar to u	4.874
EV103808	6.217	no similarity	
JCVI_18444	5.864	no original description	
JCVI_4881	5.609	very weakly similar to (87.0)AT5G15110| Symbols: | pectate lyase family protein | chr5:4895969-4897685 FORWARD no original descri	5.199
JCVI_20042	5.546	moderately similar to (349)AT5G45340| Symbols: CYP707A3 | CYP707A3 (cytochrome P450, family 707, subfamily A, polypeptide 3);	4.064
JCVI_22385	5.534	moderately similar to (303)AT2G41640| Symbols: | similar to unknown protein [Arabidopsis thaliana] (TAIR:AT3G57380.1); similar to g	4.714
JCVI_20804	5.442	weakly similar to (113)AT3G19580| Symbols: AZF2 | AZF2 (ARABIDOPSIS ZINC-FINGER PROTEIN 2) | chr3:6803299-6804120 RE	3.844
JCVI_13878	5.419	moderately similar to (466)AT5G45340| Symbols: CYP707A3 | CYP707A3 (cytochrome P450, family 707, subfamily A, polypeptide 3);	3.174
JCVI_36344	5.395	weakly similar to (121)AT4G14450| Symbols: | Identical to Uncharacterized protein At4g14450, chloroplast precursor [Arabidopsis Thal	3.725
JCVI_10510	5.290	moderately similar to (305)AT1G64110| Symbols: | AAA-type ATPase family protein | chr1:23800550-23804918 REVERSE no original	3.744
AM395641	5.289	very weakly similar to (82.8)AT4G27657| Symbols: | similar to unknown protein [Arabidopsis thaliana] (TAIR:AT4G27652.1) | chr4:138	5.590
JCVI_12679	5.272	no original description	4.464
JCVI_143	5.238	moderately similar to (431)AT2G40000| Symbols: HSPRO2, ATHSPRO2 | similar to unknown protein [Arabidopsis thaliana] (TAIR:AT3	2.782
JCVI_26301	5.208	moderately similar to (240)AT1G19210| Symbols: | AP2 domain-containing transcription factor, putative | chr1:6626964-6627521 REVE	5.982
JCVI_4569	5.183	weakly similar to (110)AT3G17520| Symbols: | late embryogenesis abundant domain-containing protein / LEA domain-containing protei	3.196
JCVI_3404	5.155	moderately similar to (384)AT3G50060| Symbols: MYB77 | MYB77; DNA binding / transcription factor | chr3:18569129-18570034 REV	2.935
JCVI_25760	5.132	moderately similar to (475)AT4G24570| Symbols: | mitochondrial substrate carrier family protein | chr4:12686556-12687497 FORWARI	2.684
JCVI_20638	5.118	weakly similar to (145)AT2G20835| Symbols: | similar to unknown protein [Arabidopsis thaliana] (TAIR:AT3G15534.1); similar to unna	3.442
JCVI_669	5.114	moderately similar to (315)AT4G17490| Symbols: ERF-6-6, ATERF6 | ATERF6 (ETHYLENE RESPONSIVE ELEMENT BINDING FA	3.990
EV158484	5.103	moderately similar to (292)AT5G57240| Symbols: | oxysterol-binding family protein | chr5:23210675-23212529 FORWARD [21484]	3.118
JCVI_30169	5.089	very weakly similar to (100)AT2G40000| Symbols: HSPRO2, ATHSPRO2 | similar to unknown protein [Arabidopsis thaliana] (TAIR:AT	
JCVI_20373	5.057	moderately similar to (255)AT1G80390| Symbols: IAA15 | IAA15 (indoleacetic acid-induced protein 15); transcription factor | chr1:3022	2.348
JCVI_8461	5.048	moderately similar to (376)AT1G32450| Symbols: | proton-dependent oligopeptide transport (POT) family protein | chr1:11715317-1171	2.603
EE422870	5.045	no similarity	3.710
AT000615	5.012	no similarity	2.612
DW998935	5.010	weakly similar to (165)AT1G21910| Symbols: | AP2 domain-containing transcription factor family protein | chr1:7696644-7697336 FOR	
EV227530	5.005	no similarity	4.019
EX138359	5.003	highly similar to (534)AT1G50090| Symbols: | aminotransferase class IV family protein | chr1:18558309-18560462 REVERSE [21833]	3.901
EX131720	4.993	no similarity	4.803
EV182486	4.962	very weakly similar to (100)AT5G57560| Symbols: XTH22, TCH4 | TCH4 (TOUCH 4); hydrolase, acting on glycosyl bonds / xyloglucar	3.371
JCVI_17081	4.943	weakly similar to (108)AT5G17350| Symbols: | similar to unknown protein [Arabidopsis thaliana] (TAIR:AT3G03280.1); similar to unkn	3.982
JCVI_30677	4.939	moderately similar to (417)AT1G64110| Symbols: | AAA-type ATPase family protein | chr1:23800550-23804918 REVERSE no original	3.455
EX126027	4.871	moderately similar to (478)AT1G68570| Symbols: | proton-dependent oligopeptide transport (POT) family protein | chr1:25750474-2575	2.996
EV096109	4.866	very weakly similar to (80.1)AT5G14740| Symbols: CA18, BETA CA2, CA2 | CA2 (BETA CARBONIC ANHYDRASE 2) | chr5:475826	5.304
EV103471	4.838	no similarity	4.871
JCVI_23438	4.834	no original description	1.839
EV218551	4.824	moderately similar to (332)AT2G40140| Symbols: CZF1, ZFAR1 | CZF1/ZFAR1 | chr2:16779615-16781408 FORWARD [21492] 53 73	
JCVI_9883	4.779	weakly similar to (193)AT5G66780| Symbols: | similar to unknown [Ammopiptanthus mongolicus] (GB:AAW33981.1) | chr5:26680853-	3.058
JCVI_22325	4.777	weakly similar to (196)AT3G19680| Symbols: | similar to unknown protein [Arabidopsis thaliana] (TAIR:AT1G50040.1); similar to unna	3.553
JCVI_37336	4.761	weakly similar to (146)AT4G08950| Symbols: | phosphate-responsive protein, putative (EXO) | chr4:5740375-5741319 FORWARD no c	4.147
ES985248	4.747	weakly similar to (157)AT3G51910| Symbols: HSFA7A, AT-HSFA7A | AT-HSFA7A (Arabidopsis thaliana heat shock transcription facto	3.870
ES271272	4.700	no similarity	
EE476768	4.699	moderately similar to (254)AT5G17350| Symbols: | similar to unknown protein [Arabidopsis thaliana] (TAIR:AT3G03280.1); similar to u	3.685
H07351	4.673	very weakly similar to (85.9)AT5G17350| Symbols: | similar to unknown protein [Arabidopsis thaliana] (TAIR:AT3G03280.1); similar to	4.062
AM386021	4.669	no similarity	2.981
JCVI_21671	4.635	moderately similar to (271)AT3G18710| Symbols: | U-box domain-containing protein | chr3:6434240-6435487 REVERSE no original de	3.085
JCVI_20277	4.630	no original description	4.476
H74770	4.621	very weakly similar to (87.8)AT3G19680| Symbols: | similar to unknown protein [Arabidopsis thaliana] (TAIR:AT1G50040.1); similar to	3.331
AM394357	4.615	weakly similar to (110)AT2G40000| Symbols: HSPRO2, ATHSPRO2 | similar to unknown protein [Arabidopsis thaliana] (TAIR:AT3G5	2.561
AM389155	4.608	moderately similar to (261)AT1G14540| Symbols: | anionic peroxidase, putative | chr1:4974228-4975595 REVERSEweakly similar to (	3.151
EE549718	4.589	no similarity	2.903
JCVI_3712	4.581	weakly similar to (141)AT1G75390| Symbols: ATBZIP44 | ATBZIP44 (ARABIDOPSIS THALIANA BASIC LEUCINE-ZIPPER 44) | ch	
EE556375	4.581	no similarity	3.293
JCVI_27911	4.581	moderately similar to (416)AT2G36800| Symbols: UGT73C5, DOGT1 | DOGT1 (DON-GLUCOSYLTRANSFERASE); UDP-glycosyltra	3.111
JCVI_31122	4.579	weakly similar to (144)AT3G24750| Symbols: | unknown protein | chr3:9036742-9037571 FORWARD no original description	4.071
JCVI_14769	4.579	weakly similar to (171)AT5G66650| Symbols: | similar to unknown protein [Arabidopsis thaliana] (TAIR:AT2G23790.1); similar to unna	
JCVI_24381	4.559	no original description	2.784
JCVI_5468	4.530	moderately similar to (296)AT5G67300| Symbols: ATMYBR1, ATMYB44, MYBR1 | ATMYB44/ATMYBR1/MYBR1 (MYB DOMAIN	2.184
ES988910	4.530	very weakly similar to (86.3)AT3G12880| Symbols: | invertase/pectin methylesterase inhibitor family protein | chr3:4095570-4096109 FC	
JCVI_19002	4.527	weakly similar to (124)AT4G02075| Symbols: PIT1 | PIT1 (PITCHOUN 1); protein binding / zinc ion binding | chr4:913555-916414 REV	3.138
JCVI_38038	4.507	weakly similar to (136)AT5G66650| Symbols: | similar to unknown protein [Arabidopsis thaliana] (TAIR:AT2G23790.1); similar to unna	2.925
JCVI_10857	4.500	moderately similar to (353)AT1G02700| Symbols: | similar to unknown protein [Arabidopsis thaliana] (TAIR:AT4G02140.1); similar to h	
EV108573	4.486	no similarity	3.612
JCVI_1699	4.484	weakly similar to (148)AT5G06760| Symbols: | late embryogenesis abundant group 1 domain-containing protein / LEA group 1 domain-c	
EE477048	4.472	weakly similar to (139)AT3G18710| Symbols: | U-box domain-containing protein | chr3:6434240-6435487 REVERSE [20157] 1 623 65	3.504
JCVI_1995	4.459	weakly similar to (136)AT1G67856| Symbols: | protein binding / zinc ion binding | chr1:25446149-25446550 FORWARD no original de	3.130
JCVI_12350	4.454	weakly similar to (106)AT1G52342| Symbols: | unknown protein | chr1:19496109-19496372 REVERSE no original description	2.748
DT317662	4.453	very weakly similar to (80.1)AT3G19030| Symbols: | similar to unknown protein [Arabidopsis thaliana] (TAIR:AT1G49500.1) | chr3:656	4.774
EE481897	4.451	weakly similar to (154)AT4G25850| Symbols: | oxysterol-binding family protein | chr4:13143868-13146663 FORWARD [20154]	2.804
RC_ES968676	4.448	no similarity	3.655
JCVI_26223	4.427	moderately similar to (300)AT5G51990| Symbols: CBF4, DREB1D | CBF4/DREB1D (C- REPEAT-BINDING FACTOR 4); DNA bindir	2.459
JCVI_28808	4.410	weakly similar to (106)AT3G19030| Symbols: | similar to unknown protein [Arabidopsis thaliana] (TAIR:AT1G49500.1) | chr3:6564123	4.596
EV061913	4.406	no similarity	
EX071969	4.334	moderately similar to (281)AT5G07475| Symbols: | plastocyanin-like domain-containing protein | chr5:2364828-2365537 REVERSEvery	
JCVI_38563	4.329	weakly similar to (138)AT3G19580| Symbols: AZF2 | AZF2 (ARABIDOPSIS ZINC-FINGER PROTEIN 2) | chr3:6803299-6804120 RE	2.852
EV108083	4.313	weakly similar to (154)AT2G36800| Symbols: UGT73C5, DOGT1 | DOGT1 (DON-GLUCOSYLTRANSFERASE); UDP-glycosyltransfe	3.033
EX125037	4.297	weakly similar to (126)AT4G17615| Symbols: ATCBL1, SCABP5 | CBL1 (CALCINEURIN B-LIKE PROTEIN 1); calcium ion binding |	3.202
JCVI_1711	4.279	weakly similar to (185)AT3G55980| Symbols: | zinc finger (CCCH-type) family protein | chr3:20787836-20789578 FORWARD no origi	2.607

JCVI_17114	4.279	moderately similar to (344)AT1G18300| Symbols: ATNUDT4 | ATNUDT4 (Arabidopsis thaliana Nudix hydrolase homolog 4); hydrolase	3.644
JCVI_11504	4.266	moderately similar to (361)AT1G18300| Symbols: ATNUDT4 | ATNUDT4 (Arabidopsis thaliana Nudix hydrolase homolog 4); hydrolase	3.623
EX132042	4.252	weakly similar to (172)AT5G17350| Symbols: | similar to unknown protein [Arabidopsis thaliana] (TAIR:AT3G03280.1); similar to unkn	4.537
JCVI_8551	4.222	moderately similar to (225)AT5G42380| Symbols: CML39, CML37 | CML37/CML39; calcium ion binding | chr5:16959986-16960543 R	4.057
JCVI_24649	4.215	moderately similar to (204)AT4G25490| Symbols: DREB1B, CBF1 | CBF1 (C-REPEAT/DRE BINDING FACTOR 1); DNA binding / tra	4.664
JCVI_23374	4.206	no original description	3.015
JCVI_24275	4.203	highly similar to (581)AT5G42760| Symbols: | similar to unknown [Populus trichocarpa] (GB:ABK95091.1); contains InterPro domain C	3.310
JCVI_25058	4.181	weakly similar to (197)AT5G59820| Symbols: ZAT12, RHL41 | RHL41 (RESPONSIVE TO HIGH LIGHT 41); nucleic acid binding / tra	3.388
JCVI_25314	4.169	weakly similar to (160)AT4G15810| Symbols: | chloroplast outer membrane protein, putative | chr4:8989175-8992604 REVERSE no orig	2.692
EV189264	4.137	moderately similar to (215)AT4G15810| Symbols: | chloroplast outer membrane protein, putative | chr4:8989175-8992604 REVERSE [2	2.071
EX132070	4.124	moderately similar to (232)AT4G25810| Symbols: XTH23, XTR6 | XTR6 (XYLOGLUCAN ENDOTRANSGLYCOSYLASE 6); hydrola	
ES903612	4.113	no similarity	3.433
EX042542	4.110	moderately similar to (256)AT1G02040| Symbols: | zinc finger (C2H2 type) family protein | chr1:358104-359078 REVERSE [21811]	
JCVI_15634	4.109	moderately similar to (419)AT4G17615| Symbols: ATCBL1, SCABP5 | CBL1 (CALCINEURIN B-LIKE PROTEIN 1); calcium ion bindi	2.218
JCVI_28290	4.104	weakly similar to (119)AT1G02820| Symbols: | late embryogenesis abundant 3 family protein / LEA3 family protein | chr1:623933-6243	3.404
JCVI_27796	4.104	weakly similar to (107)AT4G27657| Symbols: | similar to unknown protein [Arabidopsis thaliana] (TAIR:AT4G27652.1) | chr4:1381313	4.153
JCVI_29276	4.097	weakly similar to (172)AT3G21890| Symbols: | zinc finger (B-box type) family protein | chr3:7709305-7709670 REVERSE no original d	3.349
JCVI_32745	4.083	moderately similar to (427)AT4G21200| Symbols: ATGA2OX8 | ATGA2OX8 (GIBBERELLIN 2-OXIDASE 8); gibberellin 2-beta-dioxy	2.895
JCVI_29480	4.079	no original description	
EE419156	4.052	moderately similar to (215)AT1G64110| Symbols: | AAA-type ATPase family protein | chr1:23800550-23804918 REVERSE [20146]	3.430
JCVI_40407	4.045	highly similar to (523)AT5G57560| Symbols: XTH22, TCH4 | TCH4 (TOUCH 4); hydrolase, acting on glycosyl bonds / xyloglucan:xylo	
EV207068	4.036	no similarity	3.035
JCVI_26042	4.032	moderately similar to (338)AT5G66520| Symbols: | pentatricopeptide (PPR) repeat-containing protein | chr5:26569105-26570967 FORW	
JCVI_21687	4.032	moderately similar to (311)AT4G01470| Symbols: GAMMA-TIP3, TIP1;3 | GAMMA-TIP3/TIP1;3 (tonoplast intrinsic protein 1;3); water	3.464
JCVI_13720	4.020	no original description	2.367
EV028754	4.013	no similarity	
JCVI_7693	4.009	moderately similar to (225)AT5G54490| Symbols: PBP1 | PBP1 (PINOID-BINDING PROTEIN 1); calcium ion binding | chr5:22138684-	3.374
CD830269	4.006	moderately similar to (323)AT4G17490| Symbols: ERF-6-6, ATERF6 | ATERF6 (ETHYLENE RESPONSIVE ELEMENT BINDING FA	3.408
JCVI_34980	3.999	no original description	3.387
JCVI_40963	3.991	weakly similar to (156)AT5G57240| Symbols: | oxysterol-binding family protein | chr5:23210675-23212529 FORWARD no original des	2.974
JCVI_33365	3.991	no original description	
JCVI_26705	3.989	highly similar to (633)AT2G38470| Symbols: ATWRKY33, WRKY33 | WRKY33 (WRKY DNA-binding protein 33); transcription facto	2.146
JCVI_14519	3.982	highly similar to (871)AT5G45340| Symbols: CYP707A3 | CYP707A3 (cytochrome P450, family 707, subfamily A, polypeptide 3); oxyg	3.631
JCVI_38370	3.975	moderately similar to (416)AT4G29780| Symbols: | similar to unknown protein [Arabidopsis thaliana] (TAIR:AT5G12010.1); similar to u	2.720
JCVI_20498	3.971	no original description	
ES966758	3.953	no similarity	3.192
JCVI_15880	3.947	moderately similar to (234)AT3G56290| Symbols: | similar to hypothetical protein [Vitis vinifera] (GB:CAN75527.1) | chr3:20889722-20	2.979
JCVI_31716	3.941	weakly similar to (105)AT5G41130| Symbols: | catalytic | chr5:16476942-16480469 REVERSE no original description	
EV217148	3.936	no similarity	
JCVI_15796	3.933	moderately similar to (311)AT1G65480| Symbols: FT | FT (FLOWERING LOCUS T) | chr1:24335173-24337352 FORWARDweakly sin	4.445
JCVI_33149	3.930	highly similar to (659)AT5G15500| Symbols: | ankyrin repeat family protein | chr5:5031794-5033446 REVERSE no original description	
JCVI_30459	3.916	no original description	3.324
JCVI_4715	3.911	moderately similar to (329)AT5G64660| Symbols: | U-box domain-containing protein | chr5:25859345-25860607 REVERSE no original	3.394
EV177185	3.892	no similarity	1.662
ES979866	3.891	no similarity	
EX130622	3.888	moderately similar to (210)AT2G01300| Symbols: | similar to unknown protein [Arabidopsis thaliana] (TAIR:AT1G15010.1); similar to u	3.126
JCVI_7161	3.882	moderately similar to (337)AT3G50060| Symbols: MYB77 | MYB77; DNA binding / transcription factor | chr3:18569129-18570034 REV	3.255
EE409984	3.881	no similarity	3.202
EE440983	3.873	moderately similar to (239)AT1G02700| Symbols: | similar to unknown protein [Arabidopsis thaliana] (TAIR:AT4G02140.1); similar to h	
EL591180	3.872	moderately similar to (210)AT1G70780| Symbols: | similar to unknown protein [Arabidopsis thaliana] (TAIR:AT1G23150.1); similar to u	
CX271990	3.869	moderately similar to (206)AT5G64660| Symbols: | U-box domain-containing protein | chr5:25859345-25860607 REVERSE [16815]	3.423
ES902870	3.865	no similarity	
JCVI_12059	3.850	moderately similar to (328)AT1G23710| Symbols: | similar to unknown protein [Arabidopsis thaliana] (TAIR:AT1G70420.1); similar to u	2.520
JCVI_12579	3.839	weakly similar to (182)AT4G27360| Symbols: | dynein light chain, putative | chr4:13694038-13694523 FORWARD no original descripti	3.362
JCVI_12357	3.835	no original description	2.786
JCVI_21944	3.821	no original description	
DN961610	3.819	moderately similar to (278)AT3G55980| Symbols: | zinc finger (CCCH-type) family protein | chr3:20787836-20789578 FORWARD [17	1.987
CN727255	3.818	no similarity	
JCVI_40666	3.816	moderately similar to (299)AT1G72200| Symbols: | zinc finger (C3HC4-type RING finger) family protein | chr1:27173597-27174811 RE	
JCVI_32644	3.812	moderately similar to (345)AT3G28340| Symbols: GATL10 | GATL10 (Galacturonosyltransferase-like 10); polygalacturonate 4-alpha-gal	3.522
JCVI_32406	3.802	moderately similar to (274)AT3G24460| Symbols: | TMS membrane family protein / tumour differentially expressed (TDE) family protei	2.499
EV165884	3.782	weakly similar to (126)AT4G38170| Symbols: FRS9 | FRS9 (FAR1-related sequence 9); zinc ion binding | chr4:17904607-17906433 FOR	
EV202611	3.781	moderately similar to (238)AT2G36750| Symbols: UGT72C1 | UGT72C1 (UDP-GLUCOSYL TRANSFERASE 72C1); UDP-glycosyltran	5.300
ES963726	3.773	no similarity	3.024
EE433603	3.758	weakly similar to (107)AT1G44830| Symbols: | AP2 domain-containing transcription factor TINY, putative | chr1:16936232-16936867 F	4.143
JCVI_2735	3.753	weakly similar to (136)AT4G09600| Symbols: GASA3 | GASA3 (GAST1 PROTEIN HOMOLOG 3) | chr4:6073011-6073513 REVERSE	3.113
JCVI_1908	3.742	moderately similar to (395)AT3G55980| Symbols: | zinc finger (CCCH-type) family protein | chr3:20787836-20789578 FORWARD no o	2.591
JCVI_17029	3.741	weakly similar to (111)AT4G27350| Symbols: | similar to unknown protein [Arabidopsis thaliana] (TAIR:AT5G54240.1); similar to unna	
JCVI_9598	3.736	highly similar to (962)AT3G15880| Symbols: WSIP2, TPR4 | TPR4/WSIP2 (TOPLESS-RELATED 4) | chr3:5364460-5371875 REVERS	
JCVI_16210	3.736	moderately similar to (386)AT3G55010| Symbols: ATPURM, PUR5 | ATPURM/PUR5; phosphoribosylformylglycinamidine cyclo-ligase	
JCVI_11822	3.733	weakly similar to (179)AT5G24260| Symbols: | prolyl oligopeptidase family protein | chr5:8234868-8237813 REVERSE no original desc	
JCVI_15991	3.732	weakly similar to (172)AT1G28370| Symbols: ERF11, ATERF11 | ATERF11/ERF11 (ERF domain protein 11); DNA binding / transcript	
AM060617	3.730	moderately similar to (415)AT3G24020| Symbols: | disease resistance-responsive family protein | chr3:8678834-8679565 FORWARD [1	2.654
JCVI_3116	3.727	moderately similar to (321)AT5G01640| Symbols: | prenylated rab acceptor (PRA1) family protein | chr5:241439-242110 REVERSE no	2.369
EX122776	3.726	no similarity	3.104
DN237910	3.725	no similarity	
JCVI_41332	3.722	no original description	
JCVI_22598	3.713	moderately similar to (280)AT1G30860| Symbols: | protein binding / zinc ion binding | chr1:10986677-10989227 REVERSE no original	
EV114051	3.704	no similarity	
JCVI_36270	3.702	moderately similar to (300)AT2G38470| Symbols: ATWRKY33, WRKY33 | WRKY33 (WRKY DNA-binding protein 33); transcription	2.147
JCVI_15747	3.696	weakly similar to (148)AT4G27280| Symbols: | calcium-binding EF hand family protein | chr4:13663776-13664168 REVERSE no origin	3.053
EX125877	3.694	weakly similar to (130)AT5G21930| Symbols: PAA2, HMA8 | HMA8/PAA2 (P-TYPE ATPASE OF ARABIDOPSIS 2); ATPase, couple	2.808
JCVI_40327	3.679	moderately similar to (227)AT1G64340| Symbols: | similar to unknown protein [Arabidopsis thaliana] (TAIR:AT5G41810.1); similar to	2.725

JCVI_31757	3.677	no original description	2.764
JCVI_40353	3.676	weakly similar to (128)AT1G01060| Symbols: LHY1, LHY | LHY (LATE ELONGATED HYPOCOTYL) | chr1:33992-37061 REVERSI	2.044
BQ704574	3.673	no similarity	
JCVI_40374	3.671	no original description	2.492
EE471788	3.670	very weakly similar to (99.4)AT5G21930| Symbols: PAA2, HMA8 | HMA8/PAA2 (P-TYPE ATPASE OF ARABIDOPSIS 2); ATPase, cc	3.055
JCVI_29031	3.667	weakly similar to (127)AT3G56290| Symbols: | similar to hypothetical protein [Vitis vinifera] (GB:CAN75527.1) | chr3:20889722-2089(	2.655
JCVI_39808	3.662	highly similar to (623)AT1G24530| Symbols: | transducin family protein / WD-40 repeat family protein | chr1:8693274-8694530 FORW/	2.388
JCVI_37841	3.652	no original description	2.779
ES911751	3.648	weakly similar to (186)AT1G70230| Symbols: | similar to unknown protein [Arabidopsis thaliana] (TAIR:AT1G01430.1); similar to Os0	2.352
CX194101	3.638	no similarity	
AM394346	3.630	moderately similar to (306)AT3G28340| Symbols: GATL10 | GATL10 (Galacturonosyltransferase-like 10); polygalacturonate 4-alpha-gal	3.352
JCVI_37625	3.618	moderately similar to (381)AT1G60470| Symbols: ATGOLS4 | ATGOLS4 (ARABIDOPSIS THALIANA GALACTINOL SYNTHASE 4	
JCVI_37355	3.614	moderately similar to (313)AT1G64065| Symbols: | similar to unknown protein [Arabidopsis thaliana] (TAIR:AT2G44000.1); similar to	
EE509254	3.598	moderately similar to (289)AT2G30900| Symbols: | similar to unknown protein [Arabidopsis thaliana] (TAIR:AT2G42570.1); similar to	
JCVI_19043	3.591	moderately similar to (421)AT2G38470| Symbols: ATWRKY33, WRKY33 | WRKY33 (WRKY DNA-binding protein 33); transcription	2.218
JCVI_32996	3.589	no original description	1.543
EV175794	3.571	very weakly similar to (83.6)AT1G55960| Symbols: | similar to unknown protein [Arabidopsis thaliana] (TAIR:AT3G13062.2); similar to	2.603
JCVI_34901	3.569	no original description	1.943
RC_EE566317	3.568	no similarity	
EV116513	3.564	weakly similar to (150)AT1G19770| Symbols: ATPUP14 | ATPUP14 (Arabidopsis thaliana purine permease 14); purine transmembrane t	
EE417832	3.562	no similarity	
JCVI_33185	3.560	weakly similar to (145)AT5G62520| Symbols: SRO5 | SRO5 (SIMILAR TO RCD ONE 5); NAD+ ADP-ribosyltransferase | chr5:251152	2.781
JCVI_18362	3.559	weakly similar to (192)AT4G15550| Symbols: IAGLU | IAGLU (INDOLE-3-ACETATE BETA-D-GLUCOSYLTRANSFERASE); UDP-	
JCVI_35037	3.555	no original description	2.291
JCVI_20824	3.551	weakly similar to (120)AT4G28460| Symbols: | unknown protein | chr4:14066090-14066308 FORWARD no original description	2.846
JCVI_28244	3.541	moderately similar to (244)AT5G37770| Symbols: CML24, TCH2 | TCH2 (TOUCH 2); calcium ion binding | chr5:15016305-15016790 F	3.011
AT000719	3.540	no similarity	1.956
JCVI_6560	3.533	moderately similar to (316)AT3G21380| Symbols: | similar to MBP1 (MYROSINASE-BINDING PROTEIN 1) [Arabidopsis thaliana] (T	4.678
EE413000	3.519	no similarity	1.796
ES929348	3.519	weakly similar to (170)AT2G36610| Symbols: ATHB22 | ATHB22 (ARABIDOPSIS THALIANA HOMEOBOX PROTEIN 22); DNA bi	
ES968676	3.518	no similarity	3.282
JCVI_16840	3.516	weakly similar to (163)AT1G27100| Symbols: | similar to unknown protein [Arabidopsis thaliana] (TAIR:AT1G69900.1); similar to hypo	2.060
EE559467	3.516	no similarity	
JCVI_20274	3.501	weakly similar to (182)AT2G30020| Symbols: | protein phosphatase 2C, putative / PP2C, putative | chr2:12821514-12822981 FORWAR	3.466
ES907288	3.493	moderately similar to (262)AT2G39180| Symbols: | protein kinase family protein | chr2:16351356-16353686 REVERSEweakly similar to	3.666
JCVI_32474	3.488	moderately similar to (221)AT3G19595| Symbols: | phosphoprotein phosphatase | chr3:6808591-6809514 REVERSE no original descripti	3.843
EE481297	3.486	very weakly similar to (86.7)AT4G28460| Symbols: | unknown protein | chr4:14066090-14066308 FORWARD [20154] 1 595 631	2.814
JCVI_14554	3.483	moderately similar to (319)AT1G19770| Symbols: ATPUP14 | ATPUP14 (Arabidopsis thaliana purine permease 14); purine transmembra	
JCVI_16974	3.483	very weakly similar to (84.7)AT3G19680| Symbols: | similar to unknown protein [Arabidopsis thaliana] (TAIR:AT1G50040.1); similar to	3.490
JCVI_4245	3.479	moderately similar to (369)AT5G13170| Symbols: | nodulin MtN3 family protein | chr5:4181334-4183174 REVERSE no original descrip	2.792
DY003886	3.479	weakly similar to (140)AT3G63350| Symbols: HSFA7B, AT-HSFA7B | AT-HSFA7B (Arabidopsis thaliana heat shock transcription facto	3.126
JCVI_25396	3.477	weakly similar to (113)AT4G23870| Symbols: | similar to unknown protein [Arabidopsis thaliana] (TAIR:AT4G11020.1) | chr4:1241441	3.862
JCVI_734	3.472	highly similar to (652)AT1G67830| Symbols: ATFXG1 | ATFXG1 (ALPHA-FUCOSIDASE 1); alpha-L-fucosidase/ carboxylesterase | ch	
JCVI_21093	3.471	moderately similar to (204)AT3G27540| Symbols: | glycosyl transferase family 17 protein | chr3:10206726-10208125 FORWARD no ori	1.996
EV066486	3.466	moderately similar to (407)AT3G50940| Symbols: | AAA-type ATPase family protein | chr3:18945067-18946509 FORWARD [21443]	
BG543167	3.460	no similarity	
EE450166	3.458	moderately similar to (283)AT1G04350| Symbols: | 2-oxoglutarate-dependent dioxygenase, putative | chr1:1165295-1166537 FORWARI	2.324
JCVI_34258	3.458	weakly similar to (117)AT1G73965| Symbols: CLE13 | CLE13 (CLAVATA3/ESR-RELATED 13); receptor binding | chr1:27819483-278	2.032
JCVI_38371	3.457	moderately similar to (228)AT1G74930| Symbols: ORA47 | ORA47; DNA binding / transcription factor | chr1:28147900-28148487 FOR	4.050
JCVI_36119	3.455	weakly similar to (115)AT3G13062| Symbols: | similar to unknown protein [Arabidopsis thaliana] (TAIR:AT1G55960.1); similar to unna	2.214
EV058378	3.454	moderately similar to (322)AT4G26200| Symbols: ACS7 | ACS7 (1-Amino-cyclopropane-1-carboxylate synthase 7); 1-aminocyclopropan	2.538
JCVI_41856	3.454	moderately similar to (224)AT1G74930| Symbols: ORA47 | ORA47; DNA binding / transcription factor | chr1:28147900-28148487 FOR	4.314
EV015453	3.450	very weakly similar to (92.4)AT4G25990| Symbols: CIL | CIL | chr4:13191946-13193552 REVERSE [21440]	
EV118150	3.450	moderately similar to (274)AT2G40000| Symbols: HSPRO2, ATHSPRO2 | similar to unknown protein [Arabidopsis thaliana] (TAIR:AT3	3.009
JCVI_19330	3.446	weakly similar to (107)AT1G32920| Symbols: | similar to unknown protein [Arabidopsis thaliana] (TAIR:AT1G32928.1) | chr1:1192888	3.865
JCVI_13423	3.445	highly similar to (545)AT4G21320| Symbols: HSA32 | HSA32 (HEAT-STRESS-ASSOCIATED 32); catalytic | chr4:11340502-11341742	
EV130725	3.444	no similarity	2.937
JCVI_32123	3.438	weakly similar to (103)AT2G23810| Symbols: TET8 | TET8 (TETRASPANIN8) | chr2:10142939-10144432 REVERSE no original descr	
EL590902	3.437	weakly similar to (146)AT3G61110| Symbols: ARS27A | ARS27A (ARABIDOPSIS RIBOSOMAL PROTEIN S27); structural constituen	
JCVI_1343	3.436	moderately similar to (468)AT5G22250| Symbols: | CCR4-NOT transcription complex protein, putative | chr5:7365608-7366444 REVEF	2.381
JCVI_22268	3.435	moderately similar to (213)AT3G19580| Symbols: AZF2 | AZF2 (ARABIDOPSIS ZINC-FINGER PROTEIN 2) | chr3:6803299-6804120	
JCVI_26936	3.431	moderately similar to (243)AT3G21150| Symbols: | zinc finger (B-box type) family protein | chr3:7412719-7413396 REVERSE no origi	2.350
JCVI_1210	3.428	moderately similar to (295)AT2G47770| Symbols: | benzodiazepine receptor-related | chr2:19575771-19576361 FORWARD no original	
JCVI_1953	3.425	moderately similar to (234)AT4G25200| Symbols: ATHSP23.6-MITO | ATHSP23.6-MITO (MITOCHONDRION-LOCALIZED SMALL	3.536
EV209060	3.418	weakly similar to (151)AT4G15810| Symbols: | chloroplast outer membrane protein, putative | chr4:8989175-8992604 REVERSE [2149	1.901
AM058651	3.417	moderately similar to (301)AT4G30810| Symbols: SCPL29 | SCPL29 (serine carboxypeptidase-like 29); serine carboxypeptidase | chr4:1	
EX121530	3.416	no similarity	
JCVI_21020	3.413	weakly similar to (141)AT2G40000| Symbols: HSPRO2, ATHSPRO2 | similar to unknown protein [Arabidopsis thaliana] (TAIR:AT3G5	3.003
EX015383	3.413	no similarity	1.808
JCVI_20770	3.399	weakly similar to (145)AT2G20835| Symbols: | similar to unknown protein [Arabidopsis thaliana] (TAIR:AT3G15534.1); similar to unna	3.082
JCVI_1347	3.399	moderately similar to (346)AT3G52400| Symbols: ATSYP122, SYP122 | SYP122 (syntaxin 122); SNAP receptor | chr3:19436813-19438	
JCVI_34633	3.391	weakly similar to (122)AT3G05936| Symbols: | similar to unnamed protein product [Vitis vinifera] (GB:CAO17316.1) | chr3:1774068-17	
JCVI_18790	3.390	moderately similar to (342)AT3G14680| Symbols: CYP72A14 | CYP72A14 (cytochrome P450, family 72, subfamily A, polypeptide 14);	
JCVI_36253	3.388	moderately similar to (437)AT4G11280| Symbols: | ACS6 (1-AMINOCYCLOPROPANE-1-CARBOXYLIC ACID (ACC) SYNTHASE	2.579
JCVI_16841	3.387	weakly similar to (181)AT4G14805| Symbols: | protease inhibitor/seed storage/lipid transfer protein (LTP)-related | chr4:8502369-85031	
JCVI_28384	3.382	moderately similar to (336)AT4G11660| Symbols: HSFB2B, AT-HSFB2B | AT-HSFB2B (Arabidopsis thaliana heat shock transcription f	2.027
EE549228	3.377	very weakly similar to (91.3)AT3G50060| Symbols: MYB77 | MYB77; DNA binding / transcription factor | chr3:18569129-18570034 RE	
EE567489	3.373	no similarity	
JCVI_40345	3.369	moderately similar to (259)AT5G47230| Symbols: ATERF-5, ATERF5, ERF5 | ERF5 (ETHYLENE RESPONSIVE ELEMENT BINDIN	1.837
EE439539	3.366	weakly similar to (109)AT4G01960| Symbols: | similar to unknown protein [Arabidopsis thaliana] (TAIR:AT1G02380.1); similar to unna	2.107
EV213527	3.365	very weakly similar to (86.7)AT5G50360| Symbols: | similar to unknown protein [Arabidopsis thaliana] (TAIR:AT5G63350.1); similar to	
EX042807	3.365	no similarity	2.909
EX108444	3.362	weakly similar to (169)AT3G56290| Symbols: | similar to hypothetical protein [Vitis vinifera] (GB:CAN75527.1) | chr3:20889722-2089(	2.446

EX036618	3.359	weakly similar to (112)AT4G30830| Symbols: | similar to unknown protein [Arabidopsis thaliana] (TAIR:AT2G24140.1); similar to unna	
JCVI_36421	3.359	no original description	2.642
EV160261	3.354	weakly similar to (107)AT1G32920| Symbols: | similar to unknown protein [Arabidopsis thaliana] (TAIR:AT1G32928.1) | chr1:1192888	3.694
EV142990	3.346	weakly similar to (119)AT2G40000| Symbols: HSPRO2, ATHSPRO2 | similar to unknown protein [Arabidopsis thaliana] (TAIR:AT3G5	3.002
JCVI_15939	3.344	highly similar to (608)AT5G14700| Symbols: | cinnamoyl-CoA reductase-related | chr5:4740505-4743330 REVERSEvery weakly simila	2.563
EV120770	3.341	weakly similar to (195)AT3G44260| Symbols: | CCR4-NOT transcription complex protein, putative | chr3:15963200-15964042 REVERS	2.206
JCVI_11759	3.339	weakly similar to (125)AT4G21020| Symbols: | late embryogenesis abundant domain-containing protein / LEA domain-containing protei	
JCVI_231	3.337	highly similar to (515)AT4G25810| Symbols: XTH23, XTR6 | XTR6 (XYLOGLUCAN ENDOTRANSGLYCOSYLASE 6); hydrolase, a	
JCVI_3132	3.337	moderately similar to (321)AT2G27080| Symbols: | harpin-induced protein-related / HIN1-related / harpin-responsive protein-related | ch	2.473
ES938263	3.335	no similarity	
EV227409	3.331	moderately similar to (385)AT1G32450| Symbols: | proton-dependent oligopeptide transport (POT) family protein | chr1:11715317-1171	2.820
EV115961	3.329	weakly similar to (164)AT5G13490| Symbols: AAC2 | AAC2 (ADP/ATP CARRIER 2); binding | chr5:4336037-4337382 FORWARDwe	
JCVI_32987	3.329	moderately similar to (204)AT5G62520| Symbols: SRO5 | SRO5 (SIMILAR TO RCD ONE 5); NAD+ ADP-ribosyltransferase | chr5:251	
JCVI_28478	3.329	weakly similar to (135)AT3G19680| Symbols: | similar to unknown protein [Arabidopsis thaliana] (TAIR:AT1G50040.1); similar to unna	2.581
JCVI_31731	3.321	weakly similar to (196)AT4G02920| Symbols: | similar to unknown protein [Arabidopsis thaliana] (TAIR:AT1G03340.1); similar to hypo	
EE524509	3.320	moderately similar to (215)AT2G19710| Symbols: | similar to unknown protein [Arabidopsis thaliana] (TAIR:AT4G29440.1); similar to	
JCVI_42207	3.317	no original description	3.087
JCVI_35716	3.316	moderately similar to (408)AT4G36030| Symbols: | armadillo/beta-catenin repeat family protein | chr4:17045087-17047099 REVERSE r	2.075
JCVI_35706	3.316	moderately similar to (309)AT5G15100| Symbols: PIN8 | PIN8 (PIN-FORMED 8); auxin:hydrogen symporter/ transporter | chr5:4892162	1.770
JCVI_21991	3.311	no original description	
CD825949	3.310	weakly similar to (172)AT3G28030| Symbols: UVR1, UVH3 | UVH3 (ULTRAVIOLET HYPERSENSITIVE 3); nuclease | chr3:1042555	3.080
EX102092	3.306	moderately similar to (314)AT4G29780| Symbols: | similar to unknown protein [Arabidopsis thaliana] (TAIR:AT5G12010.1); similar to	2.567
JCVI_9483	3.305	moderately similar to (338)AT3G20810| Symbols: | transcription factor jumonji (jmjC) domain-containing protein | chr3:7275820-727818	
EV109707	3.304	no similarity	
EX040796	3.303	weakly similar to (198)AT3G22050| Symbols: | receptor-like protein kinase-related | chr3:7764138-7765150 FORWARD [21811] 20 521	
ES902008	3.302	weakly similar to (144)AT2G40080| Symbols: ELF4 | ELF4 (EARLY FLOWERING 4) | chr2:16741623-16741958 REVERSE [21428] 1	2.464
EV137114	3.296	no similarity	
EV173184	3.292	weakly similar to (135)AT3G14680| Symbols: CYP72A14 | CYP72A14 (cytochrome P450, family 72, subfamily A, polypeptide 14); oxy	2.231
EE508763	3.292	moderately similar to (229)AT4G29890| Symbols: | choline monooxygenase, putative (CMO-like) | chr4:14608874-14610911 FORWAR	
EX113607	3.288	weakly similar to (177)AT2G40080| Symbols: ELF4 | ELF4 (EARLY FLOWERING 4) | chr2:16741623-16741958 REVERSE [21827] 5	2.615
ES969123	3.288	no similarity	3.043
EV190074	3.280	moderately similar to (249)AT2G41640| Symbols: | similar to unknown protein [Arabidopsis thaliana] (TAIR:AT3G57380.1); similar to	3.628
CV433416	3.259	very weakly similar to (97.8)AT3G02410| Symbols: | similar to ATPCME (PRENYLCYSTEINE METHYLESTERASE), prenylcysteine	2.947
EV147360	3.254	no similarity	
JCVI_20339	3.249	very weakly similar to (89.4)AT1G65510| Symbols: | similar to unknown protein [Arabidopsis thaliana] (TAIR:AT1G65490.1) | chr1:243	
JCVI_35682	3.248	no original description	
EV100456	3.243	no similarity	
JCVI_36013	3.243	very weakly similar to (88.6)AT1G51990| Symbols: | O-methyltransferase family 2 protein | chr1:19334618-19336336 FORWARDvery w	
JCVI_5107	3.243	weakly similar to (141)AT3G47430| Symbols: PEX11B | PEX11B | chr3:17491783-17492677 FORWARD no original description	3.514
CV432238	3.241	weakly similar to (109)AT3G46620| Symbols: | zinc finger (C3HC4-type RING finger) family protein | chr3:17189822-17191009 REVE	2.684
EV191785	3.240	weakly similar to (131)AT4G01090| Symbols: | extra-large G-protein-related | chr4:470834-473248 REVERSE [21489]	
JCVI_40884	3.239	weakly similar to (146)AT4G29780| Symbols: | similar to unknown protein [Arabidopsis thaliana] (TAIR:AT5G12010.1); similar to unna	3.113
JCVI_3719	3.237	weakly similar to (164)AT3G29000| Symbols: | calcium-binding EF hand family protein | chr3:11007028-11007612 FORWARD no orig	3.731
ES965872	3.235	no similarity	
JCVI_20887	3.235	no original description	2.828
EV109361	3.230	no similarity	
JCVI_40125	3.229	weakly similar to (156)AT2G11810| Symbols: MGD3, ATMGD3, MGDC | MGDC (monogalactosyldiacylglycerol synthase type C) | chr	
JCVI_39508	3.223	no original description	
EE408753	3.219	weakly similar to (179)AT3G03620| Symbols: | MATE efflux family protein | chr3:873911-876259 REVERSE [16817] 1 642 671	2.834
L46575	3.215	no similarity	
JCVI_37858	3.215	no original description	
JCVI_6313	3.205	very weakly similar to (97.4)AT3G23170| Symbols: | similar to unknown protein [Arabidopsis thaliana] (TAIR:AT4G14450.1) | chr3:826	1.780
EV102814	3.199	weakly similar to (196)AT3G12320| Symbols: | similar to unknown protein [Arabidopsis thaliana] (TAIR:AT5G06980.1); similar to hypo	1.962
JCVI_15314	3.198	moderately similar to (387)AT2G31380| Symbols: STH | STH (salt tolerance homologue); transcription factor/ zinc ion binding | chr2:133	2.666
EV128635	3.196	no similarity	2.637
JCVI_4571	3.195	moderately similar to (266)AT5G59820| Symbols: ZAT12, RHL41 | RHL41 (RESPONSIVE TO HIGH LIGHT 41); nucleic acid binding	
JCVI_28902	3.194	moderately similar to (271)AT2G40140| Symbols: CZF1, ZFAR1 | CZF1/ZFAR1 | chr2:16779615-16781408 FORWARD no original des	
JCVI_925	3.194	weakly similar to (140)AT3G15670| Symbols: | late embryogenesis abundant protein, putative / LEA protein, putative | chr3:5310148-53	
JCVI_39089	3.193	moderately similar to (216)AT2G01300| Symbols: | similar to unknown protein [Arabidopsis thaliana] (TAIR:AT1G15010.1); similar to	2.522
DY011298	3.190	no similarity	
JCVI_22191	3.189	weakly similar to (194)AT4G01090| Symbols: | extra-large G-protein-related | chr4:470834-473248 REVERSE no original description	
EX136337	3.189	weakly similar to (145)AT5G18580| Symbols: TON2, EMB40, FS1, GDO, FASS | FASS (FASS 1) | chr5:6175156-6178216 FORWARD	
AM393934	3.180	no similarity	3.187
JCVI_15179	3.178	highly similar to (517)AT1G01540| Symbols: | protein kinase family protein | chr1:195980-198383 FORWARDmoderately similar to (2	2.029
JCVI_14336	3.175	weakly similar to (125)AT2G42560| Symbols: | late embryogenesis abundant domain-containing protein / LEA domain-containing protei	3.980
JCVI_22226	3.175	moderately similar to (303)AT4G35240| Symbols: | similar to unknown protein [Arabidopsis thaliana] (TAIR:AT2G17110.1); similar to	1.773
EV224869	3.171	very weakly similar to (96.3)AT1G74930| Symbols: ORA47 | ORA47; DNA binding / transcription factor | chr1:28147900-28148487 FOF	5.241
JCVI_25673	3.169	moderately similar to (376)AT3G60420| Symbols: | similar to unknown protein [Arabidopsis thaliana] (TAIR:AT3G60450.1); similar to	
DW997843	3.167	no similarity	
AT000502	3.161	no similarity	
JCVI_14546	3.158	moderately similar to (310)AT3G46620| Symbols: | zinc finger (C3HC4-type RING finger) family protein | chr3:17189822-17191009 RE	2.150
JCVI_151	3.157	moderately similar to (259)AT5G04340| Symbols: CZF2, ZAT6, C2H2 | C2H2 (ZINC FINGER OF ARABIDOPSIS THALIANA 6); nuc	2.878
JCVI_2085	3.152	moderately similar to (258)AT3G22840| Symbols: ELIP, ELIP1 | ELIP1 (EARLY LIGHT-INDUCABLE PROTEIN); chlorophyll binding	
EE568085	3.151	no similarity	
JCVI_14921	3.149	moderately similar to (303)AT5G64430| Symbols: | octicosapeptide/Phox/Bem1p (PB1) domain-containing protein | chr5:25779766-2578	2.067
ES993537	3.147	moderately similar to (306)AT1G01540| Symbols: | protein kinase family protein | chr1:195980-198383 FORWARDweakly similar to (1	1.749
JCVI_38161	3.146	no original description	
JCVI_26910	3.141	no original description	
JCVI_16211	3.141	moderately similar to (250)AT1G12020| Symbols: | similar to unknown protein [Arabidopsis thaliana] (TAIR:AT1G62422.1); similar to	
EE559860	3.138	no similarity	
EX109901	3.137	moderately similar to (231)AT1G06160| Symbols: | ethylene-responsive factor, putative | chr1:1883045-1883779 FORWARDvery weakl	
EV197730	3.133	no similarity	2.750
EE507700	3.130	no similarity	

JCVI_11394	3.129	moderately similar to (421)AT1G70170| Symbols: MMP | MMP (MATRIX METALLOPROTEINASE); metalloendopeptidase | chr1:264	
JCVI_36976	3.127	moderately similar to (362)AT3G55840| Symbols: | similar to unknown protein [Arabidopsis thaliana] (TAIR:AT2G40000.1); similar to u	1.964
EX134698	3.126	weakly similar to (179)AT4G30830| Symbols: | similar to unknown protein [Arabidopsis thaliana] (TAIR:AT2G24140.1); similar to unna	
EV140755	3.119	moderately similar to (203)AT3G14950| Symbols: TTL2 | TTL2 (TETRATRICOPETIDE-REPEAT THIOREDOXIN-LIKE 2); binding |	3.229
DN965490	3.116	no similarity	
JCVI_4934	3.114	no original description	
EE406707	3.112	no similarity	2.152
JCVI_3622	3.110	moderately similar to (306)AT3G19380| Symbols: | U-box domain-containing protein | chr3:6714608-6715873 REVERSEweakly similar	2.699
EE559806	3.098	no similarity	1.604
JCVI_29566	3.097	weakly similar to (174)AT1G18265| Symbols: | contains InterPro domain Protein of unknown function DUF593 (InterPro:IPR007656) | c	3.135
EV125502	3.096	moderately similar to (298)AT4G33920| Symbols: | protein phosphatase 2C family protein / PP2C family protein | chr4:16260881-16262	2.125
JCVI_15246	3.094	moderately similar to (300)AT3G57530| Symbols: ATCPK32, CDPK32, CPK32 | CPK32 (CALCIUM-DEPENDENT PROTEIN KINAS	
JCVI_33840	3.092	no original description	
JCVI_40911	3.091	moderately similar to (446)AT1G13110| Symbols: CYP71B7 | CYP71B7 (cytochrome P450, family 71, subfamily B, polypeptide 7); oxy	
JCVI_23644	3.089	weakly similar to (120)AT4G29780| Symbols: | similar to unknown protein [Arabidopsis thaliana] (TAIR:AT5G12010.1); similar to unna	2.928
RC_EE564068	3.083	no similarity	
EV103862	3.081	moderately similar to (310)AT1G70170| Symbols: MMP | MMP (MATRIX METALLOPROTEINASE); metalloendopeptidase | chr1:264	
JCVI_9015	3.079	weakly similar to (174)AT5G47320| Symbols: RPS19 | RPS19 (40S ribosomal protein S19); RNA binding | chr5:19221028-19222178 FO	2.229
JCVI_5733	3.078	moderately similar to (204)AT1G21910| Symbols: | AP2 domain-containing transcription factor family protein | chr1:7696644-7697336 F	
JCVI_9589	3.076	moderately similar to (421)AT1G27100| Symbols: | similar to unknown protein [Arabidopsis thaliana] (TAIR:AT1G69900.1); similar to l	1.967
CN728830	3.074	no similarity	
DY010300	3.071	weakly similar to (131)AT5G10695| Symbols: | similar to unknown protein [Arabidopsis thaliana] (TAIR:AT5G57123.1); similar to unkn	
JCVI_8881	3.070	no original description	
EX084336	3.070	moderately similar to (343)AT2G42900| Symbols: | similar to unnamed protein product [Vitis vinifera] (GB:CAO70018.1); contains Inter	
DW997751	3.069	very weakly similar to (98.6)AT3G61640| Symbols: ATAGP20, AGP20 | AGP20 (ARABINOGALACTAN PROTEIN 20) | chr3:2282125	1.787
JCVI_34219	3.068	no original description	2.340
JCVI_16259	3.068	moderately similar to (491)AT4G02300| Symbols: | pectinesterase family protein | chr4:1009366-1013034 REVERSEmoderately similar	
JCVI_6893	3.067	weakly similar to (151)AT3G50800| Symbols: | similar to unknown protein [Arabidopsis thaliana] (TAIR:AT5G66580.1); similar to unna	
JCVI_28622	3.062	highly similar to (546)AT3G28340| Symbols: GATL10 | GATL10 (Galacturonosyltransferase-like 10); polygalacturonate 4-alpha-galactu	3.103
JCVI_4850	3.062	weakly similar to (197)AT3G12320| Symbols: | similar to unknown protein [Arabidopsis thaliana] (TAIR:AT5G06980.1); similar to hypo	
EE539186	3.061	moderately similar to (211)AT1G71000| Symbols: | DNAJ heat shock N-terminal domain-containing protein | chr1:26772998-26773773 I	1.740
JCVI_8403	3.060	moderately similar to (268)AT5G05410| Symbols: DREB2, DREB2A | DREB2A (DRE-BINDING PROTEIN 2A); DNA binding / transc	
JCVI_16557	3.059	moderately similar to (273)AT3G50060| Symbols: MYB77 | MYB77; DNA binding / transcription factor | chr3:18569129-18570034 REV	
EV107830	3.057	no similarity	
JCVI_11277	3.057	moderately similar to (379)AT3G23250| Symbols: MYB15, AtY19, AtMYB15 | AtMYB15/AtY19/MYB15 (myb domain protein 15); DN	
JCVI_9832	3.056	highly similar to (582)AT1G64500| Symbols: | glutaredoxin family protein | chr1:23956933-23958039 FORWARD no original descriptic	2.103
JCVI_26540	3.054	moderately similar to (490)AT5G52300| Symbols: RD29B, LTI65 | LTI65/RD29B (RESPONSIVE TO DESSICATION 29B) | chr5:2125	3.339
AM058038	3.053	weakly similar to (106)AT3G30580| Symbols: | similar to unknown protein [Arabidopsis thaliana] (TAIR:AT5G38790.1) | chr3:1217024	2.671
EV175274	3.050	no similarity	
EV175339	3.046	no similarity	2.469
JCVI_9836	3.046	highly similar to (828)AT3G01120| Symbols: CGS, ATCYS1, CGS1, MTO1 | MTO1 (METHIONINE OVERACCUMULATION 1) | chr	
BQ704951	3.046	no similarity	1.831
JCVI_34138	3.045	moderately similar to (311)AT3G07250| Symbols: | nuclear transport factor 2 (NTF2) family protein / RNA recognition motif (RRM)-co	
ES966108	3.044	no similarity	
ES968570	3.041	no similarity	
EV111322	3.040	no similarity	
EV111066	3.038	no similarity	
EV153307	3.036	weakly similar to (101)AT3G26100| Symbols: | regulator of chromosome condensation (RCC1) family protein | chr3:9539695-9541386 F	
JCVI_22827	3.035	highly similar to (518)AT1G21790| Symbols: | similar to unnamed protein product [Vitis vinifera] (GB:CAO61872.1); contains InterPro	
AM394323	3.035	no similarity	3.039
EE545244	3.030	no similarity	
JCVI_8272	3.030	moderately similar to (256)AT1G05560| Symbols: UGT75B1, UGT1 | UGT1 (UDP-glucosyl transferase 75B1); UDP-glycosyltransferase	2.528
BQ790750	3.029	weakly similar to (181)AT4G10250| Symbols: ATHSP22.0 | ATHSP22.0 (Arabidopsis thaliana heat shock protein 22.0) | chr4:6370533-6	
EX122617	3.028	moderately similar to (347)AT5G22380| Symbols: ANAC090 | ANAC090 (Arabidopsis NAC domain containing protein 90); transcriptio	
ES969090	3.028	no similarity	
CX278676	3.026	no similarity	
JCVI_894	3.024	moderately similar to (423)AT3G21380| Symbols: | similar to MBP1 (MYROSINASE-BINDING PROTEIN 1) [Arabidopsis thaliana] (T	3.447
JCVI_28463	3.021	moderately similar to (257)AT3G09020| Symbols: | alpha 1,4-glycosyltransferase family protein / glycosyltransferase sugar-binding DXD	
JCVI_41678	3.020	highly similar to (507)AT3G09450| Symbols: | similar to unknown protein [Arabidopsis thaliana] (TAIR:AT2G28780.1); similar to unna	3.940
JCVI_14049	3.019	moderately similar to (226)AT3G26980| Symbols: MUB4 | MUB4 (MEMBRANE-ANCHORED UBIQUITIN-FOLD PROTEIN 4 PREC	
JCVI_4734	3.014	moderately similar to (397)AT1G06260| Symbols: | cysteine proteinase, putative | chr1:1916448-1917584 FORWARDweakly similar to (	2.013
JCVI_42470	3.010	weakly similar to (171)AT3G28200| Symbols: | peroxidase, putative | chr3:10519319-10520269 FORWARD no original description	
JCVI_2152	3.008	very weakly similar to (100)AT5G22650| Symbols: HDT02, HDT2, ATHD2B, HDA4, HD2, HD2B | HD2B (HISTONE DEACETYLAS	1.877
JCVI_2702	3.006	no original description	
JCVI_4875	3.004	highly similar to (576)AT5G04950| Symbols: | nicotianamine synthase, putative | chr5:1457877-1458839 REVERSEmoderately similar t	
JCVI_4490	2.999	moderately similar to (320)AT4G40010| Symbols: SNRK2-7, SNRK2.7, SRK2F | SNRK2-7/SNRK2.7/SRK2F (SNF1-RELATED PROT	
EE524030	2.997	very weakly similar to (82.0)AT3G46620| Symbols: | zinc finger (C3HC4-type RING finger) family protein | chr3:17189822-17191009 R	2.810
JCVI_14678	2.990	moderately similar to (346)AT4G24660| Symbols: ATHB22, MEE68 | ATHB22/MEE68 (ARABIDOPSIS THALIANA HOMEOBOX PR	2.963
JCVI_7022	2.988	moderately similar to (243)AT5G12020| Symbols: HSP17.6II | HSP17.6II (17.6 KDA CLASS II HEAT SHOCK PROTEIN) | chr5:38824	
JCVI_13680	2.988	moderately similar to (207)AT1G09520| Symbols: | similar to PHD finger family protein [Arabidopsis thaliana] (TAIR:AT3G17460.1); s	
JCVI_38283	2.986	moderately similar to (231)AT4G01090| Symbols: | extra-large G-protein-related | chr4:470834-473248 REVERSE no original descriptio	
JCVI_15052	2.983	moderately similar to (230)AT3G21300| Symbols: | RNA methyltransferase family protein | chr3:7493660-7496263 REVERSE no origin	1.927
JCVI_36240	2.982	moderately similar to (355)AT3G60420| Symbols: | similar to unknown protein [Arabidopsis thaliana] (TAIR:AT3G60450.1); similar to u	
JCVI_16530	2.978	highly similar to (636)AT5G14700| Symbols: | cinnamoyl-CoA reductase-related | chr5:4740505-4743330 REVERSEvery weakly similar	3.718
JCVI_3413	2.975	moderately similar to (290)AT3G22840| Symbols: ELIP, ELIP1 | ELIP1 (EARLY LIGHT-INDUCABLE PROTEIN); chlorophyll binding	
JCVI_2015	2.972	moderately similar to (300)AT3G21710| Symbols: | unknown protein | chr3:7648387-7649540 FORWARD no original description	
JCVI_11681	2.968	weakly similar to (182)AT5G23730| Symbols: | nucleotide binding | chr5:8005289-8006395 FORWARD no original description	2.705
JCVI_16668	2.965	weakly similar to (111)AT3G15357| Symbols: | unknown protein | chr3:5187171-5187602 FORWARD no original description	
JCVI_31954	2.964	weakly similar to (124)AT1G26320| Symbols: | NADP-dependent oxidoreductase, putative | chr1:9105227-9107016 FORWARD no orig	
JCVI_25854	2.964	no original description	
JCVI_16510	2.964	weakly similar to (180)AT5G04340| Symbols: CZF2, ZAT6, C2H2 | C2H2 (ZINC FINGER OF ARABIDOPSIS THALIANA 6); nucleic	2.840
CD831059	2.964	no similarity	1.480
RC_EV111589	2.963	no similarity	2.477

JCVI_23775	2.963	highly similar to (641)AT3G24460| Symbols: | TMS membrane family protein / tumour differentially expressed (TDE) family protein | ch	
EV151697	2.961	very weakly similar to (93.2)AT5G22020| Symbols: | strictosidine synthase family protein | chr5:7287881-7289360 REVERSE [21483]	
EV177509	2.961	weakly similar to (185)AT1G23130| Symbols: | Bet v I allergen family protein | chr1:8200423-8200986 FORWARD [21487]	
JCVI_35980	2.960	moderately similar to (489)AT1G28230| Symbols: ATPUP1, PUP1 | PUP1 (PURINE PERMEASE 1); purine transmembrane transporter |	
EX120951	2.958	weakly similar to (113)AT1G43910| Symbols: | AAA-type ATPase family protein | chr1:16658490-16660068 REVERSE [21829] 49 475	
JCVI_40355	2.956	moderately similar to (250)AT5G46830| Symbols: | basic helix-loop-helix (bHLH) family protein | chr5:19019946-19021481 FORWARI	2.014
JCVI_32708	2.948	no original description	
JCVI_32703	2.948	moderately similar to (350)AT1G29340| Symbols: PUB17 | PUB17 (PLANT U-BOX17); ubiquitin-protein ligase | chr1:10264398-10266	1.987
JCVI_1521	2.947	moderately similar to (246)AT3G17520| Symbols: | late embryogenesis abundant domain-containing protein / LEA domain-containing pr	
EX083488	2.945	no similarity	
JCVI_19446	2.943	weakly similar to (161)AT1G69890| Symbols: | similar to unknown protein [Arabidopsis thaliana] (TAIR:AT1G27100.1); similar to unk	1.721
JCVI_24715	2.942	weakly similar to (189)AT2G39980| Symbols: | transferase family protein | chr2:16695515-16696963 REVERSE no original description	2.870
JCVI_29935	2.941	moderately similar to (314)AT4G38960| Symbols: | zinc finger (B-box type) family protein | chr4:18162294-18163039 FORWARD no o	1.957
JCVI_34936	2.940	moderately similar to (360)AT1G78160| Symbols: APUM7 | APUM7 (ARABIDOPSIS PUMILIO 7); RNA binding | chr1:29412793-2941	
EE566364	2.939	no similarity	
RC_EE557564	2.938	no similarity	
EV108354	2.931	weakly similar to (141)AT2G36780| Symbols: | UDP-glucoronosyl/UDP-glucosyl transferase family protein | chr2:15424697-15426187 I	
EV078290	2.928	no similarity	1.275
DY009544	2.923	weakly similar to (105)AT1G30860| Symbols: | protein binding / zinc ion binding | chr1:10986677-10989227 REVERSE [18969] 15 316	1.882
JCVI_40879	2.923	very weakly similar to (83.6)AT5G46910| Symbols: | transcription factor jumonji (jmj) family protein | chr5:19065007-19068107 FORW/	
RC_EE567343	2.916	no similarity	
JCVI_21121	2.914	no original description	4.155
EV136565	2.912	no similarity	2.792
ES966278	2.911	no similarity	2.952
ES902248	2.909	moderately similar to (286)AT1G28230| Symbols: ATPUP1, PUP1 | PUP1 (PURINE PERMEASE 1); purine transmembrane transporter |	
EE505148	2.908	no similarity	
JCVI_4252	2.906	moderately similar to (446)AT3G11320| Symbols: | organic anion transmembrane transporter | chr3:3547023-3548545 REVERSE no orig	
CB686059	2.906	no similarity	2.080
JCVI_10884	2.902	moderately similar to (467)AT1G07040| Symbols: | similar to unknown protein [Arabidopsis thaliana] (TAIR:AT1G27030.1); similar to u	
EX092288	2.900	weakly similar to (179)AT1G69840| Symbols: | band 7 family protein | chr1:26297595-26298813 REVERSE [21823]	2.726
DW999620	2.896	weakly similar to (113)AT3G22840| Symbols: ELIP, ELIP1 | ELIP1 (EARLY LIGHT-INDUCABLE PROTEIN); chlorophyll binding | ch	
EX041857	2.896	weakly similar to (193)AT3G11510| Symbols: | 40S ribosomal protein S14 (RPS14B) | chr3:3623763-3624872 REVERSEweakly similar	
JCVI_32755	2.894	moderately similar to (259)AT4G01360| Symbols: | similar to BPS1 (BYPASS 1) [Arabidopsis thaliana] (TAIR:AT1G01550.2); similar t	2.619
EV126970	2.892	no similarity	
JCVI_12599	2.888	moderately similar to (252)AT5G13170| Symbols: | nodulin MtN3 family protein | chr5:4181334-4183174 REVERSE no original descrip	2.880
JCVI_31606	2.887	weakly similar to (151)AT2G26770| Symbols: | plectin-related | chr2:11411335-11414821 REVERSE no original description	2.278
EV149994	2.887	no similarity	
ES957422	2.886	no similarity	
JCVI_8830	2.883	moderately similar to (352)AT4G33920| Symbols: | protein phosphatase 2C family protein / PP2C family protein | chr4:16260881-16262	
JCVI_37351	2.881	moderately similar to (229)AT2G19710| Symbols: | similar to unknown protein [Arabidopsis thaliana] (TAIR:AT4G29440.1); similar to u	
JCVI_13330	2.880	highly similar to (693)AT1G69790| Symbols: | protein kinase, putative | chr1:26270501-26272481 FORWARDmoderately similar to (21	
JCVI_24437	2.878	moderately similar to (296)AT4G33920| Symbols: | protein phosphatase 2C family protein / PP2C family protein | chr4:16260881-16262	2.305
EX064199	2.875	no similarity	2.075
EV130300	2.874	no similarity	
JCVI_4685	2.872	moderately similar to (277)AT5G11160| Symbols: APT5 | APT5 (ADENINE PHOSPHORIBOSYLTRANSFERASE 5); adenine phospho	2.652
EG020213	2.867	no similarity	
EX065455	2.866	no similarity	
CD811973	2.860	no similarity	
JCVI_6977	2.858	moderately similar to (333)AT5G43700| Symbols: IAA4, ATAUX2-11 | ATAUX2-11 (indoleacetic acid-induced protein 4); transcription	2.621
CD828717	2.857	no similarity	
EE563641	2.851	no similarity	
RC_JCVI_37047	2.849	no original description	
JCVI_39461	2.847	moderately similar to (230)AT4G29890| Symbols: | choline monooxygenase, putative (CMO-like) | chr4:14608874-14610911 FORWAR	
EV120033	2.847	moderately similar to (287)AT1G65310| Symbols: ATXTH17 | ATXTH17 (XYLOGLUCAN ENDOTRANSGLUCOSYLASE/HYDROL	3.176
JCVI_13959	2.842	weakly similar to (113)AT5G39670| Symbols: | calcium-binding EF hand family protein | chr5:15900498-15901112 FORWARD no orig	1.870
EV096758	2.839	weakly similar to (181)AT1G69523| Symbols: | UbiE/COQ5 methyltransferase family protein | chr1:26133490-26134820 FORWARD [2	
JCVI_10583	2.838	moderately similar to (278)AT3G50770| Symbols: | calmodulin-related protein, putative | chr3:18884968-18885585 FORWARDvery wea	
H74834	2.833	no similarity	2.780
CD835542	2.831	weakly similar to (180)AT3G62720| Symbols: ATXT1 | ATXT1; transferase/ transferase, transferring glycosyl groups | chr3:23212302-23	1.756
ES965824	2.827	no similarity	
JCVI_33886	2.819	weakly similar to (147)AT3G05490| Symbols: RALFL22 | RALFL22 (RALF-LIKE 22) | chr3:1591387-1591746 FORWARD no original	3.350
EV111641	2.817	no similarity	
JCVI_30402	2.817	highly similar to (629)AT3G19270| Symbols: CYP707A4 | CYP707A4 (cytochrome P450, family 707, subfamily A, polypeptide 4); oxyg	2.564
EV175334	2.816	no similarity	2.899
JCVI_14789	2.816	moderately similar to (326)AT2G40140| Symbols: CZF1, ZFAR1 | CZF1/ZFAR1 | chr2:16779615-16781408 FORWARD no original des	
EE531628	2.813	no similarity	2.455
JCVI_25976	2.813	moderately similar to (271)AT4G33720| Symbols: | pathogenesis-related protein, putative | chr4:16182816-16183307 FORWARDmodera	
JCVI_37942	2.813	highly similar to (725)AT4G24550| Symbols: | clathrin adaptor complexes medium subunit family protein | chr4:12675883-12678913 FO	
JCVI_11454	2.810	moderately similar to (247)AT2G11810| Symbols: MGD3, ATMGD3, MGDC | MGDC (monogalactosyldiacylglycerol synthase type C) |	
RC_ES929155	2.806	no similarity	
JCVI_38257	2.806	no original description	
JCVI_20204	2.803	weakly similar to (163)AT1G13710| Symbols: CYP78A5 | CYP78A5 (cytochrome P450, family 78, subfamily A, polypeptide 5); oxygen	1.712
RC_EH424674	2.802	no similarity	
RC_EE556375	2.801	no similarity	2.866
EV103993	2.801	moderately similar to (333)AT1G70170| Symbols: MMP | MMP (MATRIX METALLOPROTEINASE); metalloendopeptidase | chr1:264	
JCVI_36313	2.800	moderately similar to (228)AT3G02840| Symbols: | immediate-early fungal elicitor family protein | chr3:618487-619626 FORWARD no	
EV217098	2.799	very weakly similar to (100)AT1G50910| Symbols: | similar to unnamed protein product [Vitis vinifera] (GB:CAO68056.1) | chr1:18870	
EX076183	2.799	no similarity	2.021
JCVI_40617	2.797	very weakly similar to (94.4)AT1G70170| Symbols: MMP | MMP (MATRIX METALLOPROTEINASE); metalloendopeptidase | chr1:26	
RC_EE560638	2.796	no similarity	
EV112555	2.794	very weakly similar to (98.6)AT2G46790| Symbols: PRR9, TL1, APRR9 | APRR9 (PSEUDO-RESPONSE REGULATOR 9); transcriptio	
JCVI_2166	2.793	moderately similar to (375)AT2G40000| Symbols: HSPRO2, ATHSPRO2 | similar to unknown protein [Arabidopsis thaliana] (TAIR:AT3	2.136
EV205882	2.791	no similarity	

JCVI_27852	2.790	no original description	
EX097178	2.789	moderately similar to (236)AT2G40000| Symbols: HSPRO2, ATHSPRO2 | similar to unknown protein [Arabidopsis thaliana] (TAIR:AT3	2.170
AM386327	2.788	moderately similar to (224)AT4G37320| Symbols: CYP81D5 | CYP81D5 (cytochrome P450, family 81, subfamily D, polypeptide 5); oxy	
JCVI_7932	2.783	very weakly similar to (87.0)AT5G64310| Symbols: ATAGP1, AGP1 | AGP1 (ARABINOGALACTAN-PROTEIN 1) | chr5:25739244-257	2.884
EV128384	2.783	no similarity	2.259
JCVI_7475	2.782	moderately similar to (453)AT5G23660| Symbols: MTN3 | MTN3 (ARABIDOPSIS HOMOLOG OF MEDICAGO TRUNCATULA MTN	
JCVI_7746	2.782	moderately similar to (205)AT5G13100| Symbols: | oxidoreductase | chr5:4156792-4158238 FORWARD no original description	2.240
EV218325	2.781	weakly similar to (142)AT2G40140| Symbols: CZF1, ZFAR1 | CZF1/ZFAR1 | chr2:16779615-16781408 FORWARD [21492]	
JCVI_30149	2.779	no original description	3.540
JCVI_17491	2.779	highly similar to (581)AT1G30370| Symbols: | lipase class 3 family protein | chr1:10719151-10720740 REVERSE no original descriptio	
EE558512	2.777	no similarity	
JCVI_21233	2.776	no original description	
JCVI_32502	2.774	moderately similar to (386)AT5G56080| Symbols: | nicotianamine synthase, putative | chr5:22728628-22729590 REVERSEweakly simil	2.380
EX039472	2.773	weakly similar to (147)AT5G05490| Symbols: SYN1, DIF1 | DIF1/SYN1 | chr5:1624713-1629145 FORWARD [21811] 20 300 300	
EV112617	2.772	no similarity	
CV546572	2.770	no similarity	
JCVI_6116	2.769	moderately similar to (271)AT1G76600| Symbols: | similar to unknown protein [Arabidopsis thaliana] (TAIR:AT1G21010.1); similar to	1.973
EV193118	2.768	weakly similar to (147)AT4G18210| Symbols: ATPUP10 | ATPUP10 (Arabidopsis thaliana purine permease 10); purine transmembrane t	
JCVI_30704	2.764	very weakly similar to (83.6)AT3G62130| Symbols: | epimerase-related | chr3:23015969-23017333 FORWARD no original description	
JCVI_4382	2.764	moderately similar to (220)AT1G54050| Symbols: | 17.4 kDa class III heat shock protein (HSP17.4-CIII) | chr1:20183226-20183790 RE	1.949
AM060704	2.763	no similarity	
EX028970	2.763	weakly similar to (108)AT3G21400| Symbols: | similar to unknown [Populus trichocarpa] (GB:ABK93428.1) | chr3:7534830-7536299 R	
JCVI_22427	2.762	moderately similar to (257)AT1G59590| Symbols: ZCF37 | ZCF37 | chr1:21891525-21892160 FORWARD no original description	
JCVI_7689	2.762	moderately similar to (400)AT3G24550| Symbols: ATPERK1 | ATPERK1 (PROLINE EXTENSIN-LIKE RECEPTOR KINASE 1); ATP	1.558
JCVI_13339	2.761	moderately similar to (437)AT2G29090| Symbols: CYP707A2 | CYP707A2 (cytochrome P450, family 707, subfamily A, polypeptide 2);	1.876
JCVI_18124	2.761	no original description	1.874
JCVI_1097	2.761	moderately similar to (439)AT3G46600| Symbols: | scarecrow transcription factor family protein | chr3:17169037-17170784 FORWARD	1.632
JCVI_18406	2.759	moderately similar to (444)AT3G49810| Symbols: | U-box domain-containing protein | chr3:18485921-18487267 REVERSE no original	
JCVI_32775	2.757	moderately similar to (377)AT4G36670| Symbols: | mannitol transporter, putative | chr4:17287684-17289487 REVERSEweakly similar t	
EV093388	2.749	no similarity	
JCVI_30401	2.746	no original description	
JCVI_28693	2.746	moderately similar to (318)AT3G27250| Symbols: | similar to unknown protein [Arabidopsis thaliana] (TAIR:AT5G40800.1); similar to	
EX131452	2.742	weakly similar to (181)AT5G59870| Symbols: HTA6 | HTA6; DNA binding | chr5:24132831-24133370 REVERSEweakly similar to (15	2.256
EV131203	2.738	no similarity	3.184
JCVI_32596	2.733	moderately similar to (316)AT5G46910| Symbols: | transcription factor jumonji (jmj) family protein | chr5:19065007-19068107 FORWA	
EX140135	2.732	weakly similar to (102)AT1G19770| Symbols: ATPUP14 | ATPUP14 (Arabidopsis thaliana purine permease 14); purine transmembrane t	
JCVI_32120	2.732	very weakly similar to (86.7)AT5G08150| Symbols: | unknown protein | chr5:2622165-2622599 REVERSE no original description	
EV156297	2.731	moderately similar to (229)AT3G19380| Symbols: | U-box domain-containing protein | chr3:6714608-6715873 REVERSE [21484] 70 77	2.334
EV128921	2.729	moderately similar to (221)AT1G77380| Symbols: AAP3 | AAP3 (amino acid permease 3); amino acid transmembrane transporter | chr1:2	1.843
CX281139	2.728	no similarity	2.322
JCVI_38799	2.727	moderately similar to (219)AT5G01880| Symbols: | zinc finger (C3HC4-type RING finger) family protein | chr5:339014-339493 FORW	
EE559501	2.727	no similarity	
JCVI_26071	2.726	moderately similar to (273)AT2G40140| Symbols: CZF1, ZFAR1 | CZF1/ZFAR1 | chr2:16779615-16781408 FORWARD no original des	
JCVI_30046	2.726	moderately similar to (322)AT1G70170| Symbols: MMP | MMP (MATRIX METALLOPROTEINASE); metalloendopeptidase | chr1:264	
JCVI_34039	2.724	weakly similar to (138)AT2G46790| Symbols: PRR9, TL1, APRR9 | APRR9 (PSEUDO-RESPONSE REGULATOR 9); transcription reg	1.432
JCVI_21646	2.723	weakly similar to (115)AT3G22880| Symbols: DMC1, ARLIM15, ATDMC1 | ATDMC1 (RECA-LIKE GENE); ATP binding / DNA-dep	
JCVI_40704	2.722	very weakly similar to (100)AT5G46295| Symbols: | unknown protein | chr5:18797131-18797346 REVERSE no original description	2.586
JCVI_20968	2.719	moderately similar to (235)AT3G49810| Symbols: | U-box domain-containing protein | chr3:18485921-18487267 REVERSEvery weakly	
JCVI_26106	2.716	moderately similar to (366)AT1G29340| Symbols: PUB17 | PUB17 (PLANT U-BOX17); ubiquitin-protein ligase | chr1:10264398-10266	1.661
JCVI_8440	2.715	no original description	2.640
JCVI_31045	2.715	moderately similar to (260)AT5G11090| Symbols: | serine-rich protein-related | chr5:3524797-3525450 FORWARD no original descripti	
JCVI_20	2.714	moderately similar to (293)AT1G27730| Symbols: ZAT10, STZ | STZ (SALT TOLERANCE ZINC FINGER); nucleic acid binding / tran	2.788
JCVI_21446	2.713	no original description	
JCVI_21265	2.710	moderately similar to (251)AT1G21550| Symbols: | calcium-binding protein, putative | chr1:7553306-7553773 REVERSEvery weakly si	
EE448760	2.706	no similarity	2.618
EV107639	2.705	no similarity	
JCVI_31237	2.698	weakly similar to (172)AT2G40080| Symbols: ELF4 | ELF4 (EARLY FLOWERING 4) | chr2:16741623-16741958 REVERSE no origina	
EV199204	2.695	very weakly similar to (85.9)AT2G34720| Symbols: | CCAAT-binding transcription factor (CBF-B/NF-YA) family protein | chr2:146570	
AT000768	2.695	no similarity	
EX137378	2.691	moderately similar to (466)AT1G61360| Symbols: | S-locus lectin protein kinase family protein | chr1:22641532-22644639 REVERSEwe	
JCVI_14397	2.690	moderately similar to (237)AT1G01250| Symbols: | AP2 domain-containing transcription factor, putative | chr1:104731-105309 REVERS	2.247
AM393924	2.689	no similarity	3.027
EX105137	2.688	weakly similar to (128)AT1G75770| Symbols: | unknown protein | chr1:28453683-28454632 FORWARD [21826]	
JCVI_802	2.687	moderately similar to (272)AT3G24500| Symbols: MBF1C, ATMBF1C | ATMBF1C/MBF1C (MULTIPROTEIN BRIDGING FACTOR	1.699
EE425481	2.686	moderately similar to (298)AT3G06145| Symbols: | similar to unnamed protein product [Vitis vinifera] (GB:CAO71096.1) | chr3:186012	3.572
ES957953	2.685	no similarity	2.594
ES945101	2.685	weakly similar to (114)AT1G09840| Symbols: | shaggy-related protein kinase kappa / ASK-kappa (ASK10) | chr1:3196116-3199526 RE	2.892
EX095727	2.684	weakly similar to (111)AT2G40000| Symbols: HSPRO2, ATHSPRO2 | similar to unknown protein [Arabidopsis thaliana] (TAIR:AT3G5	2.357
ES911468	2.683	moderately similar to (454)AT3G15354| Symbols: SPA3 | SPA3 (SPA1-RELATED 3); signal transducer | chr3:5169334-5172487 REVE	1.843
JCVI_4818	2.683	moderately similar to (238)AT3G16720| Symbols: ATL2 | ATL2 (Arabidopsis T?xicos en Levadura 2); protein binding / zinc ion binding	
EX130490	2.682	no similarity	2.328
EV191125	2.680	weakly similar to (186)AT4G08450| Symbols: | disease resistance protein (TIR-NBS-LRR class), putative | chr4:5365607-5371098 FOR	
JCVI_11330	2.680	weakly similar to (135)AT4G18880| Symbols: HSFA4A, AT-HSFA4A | AT-HSFA4A (Arabidopsis thaliana heat shock transcription facto	1.746
JCVI_21968	2.677	no original description	
EX028275	2.675	moderately similar to (429)AT5G01550| Symbols: | lectin protein kinase, putative | chr5:214516-216582 REVERSEweakly similar to (1	
EE447525	2.674	no similarity	
JCVI_9907	2.674	moderately similar to (286)AT1G76650| Symbols: CML38 | CML38 | chr1:28771803-28772336 REVERSEvery weakly similar to (82.4)(	3.144
JCVI_13591	2.671	weakly similar to (184)AT2G30020| Symbols: | protein phosphatase 2C, putative / PP2C, putative | chr2:12821514-12822981 FORWAR	2.145
JCVI_39390	2.671	moderately similar to (315)AT3G12700| Symbols: | aspartyl protease family protein | chr3:4037143-4039050 FORWARD no original des	
EV090704	2.670	weakly similar to (147)AT1G72890| Symbols: | disease resistance protein (TIR-NBS class), putative | chr1:27433608-27435378 FORWA	
EE551050	2.666	weakly similar to (112)AT5G18370| Symbols: | disease resistance protein (TIR-NBS-LRR class), putative | chr5:6085038-6088928 REV	
EV160381	2.665	moderately similar to (228)AT2G39980| Symbols: | transferase family protein | chr2:16695515-16696963 REVERSE [21484] 64 800 800	2.967
EE424279	2.664	moderately similar to (339)AT1G11050| Symbols: | protein kinase family protein | chr1:3681892-3683769 FORWARDweakly similar to	

CD828081	2.662	no similarity	
EE550074	2.657	no similarity	
JCVI_10466	2.653	weakly similar to (142)AT1G76900| Symbols: AtTLP1 | AtTLP1 (TUBBY LIKE PROTEIN 1); phosphoric diester hydrolase/ transcriptio	
JCVI_18326	2.652	moderately similar to (384)AT4G01360| Symbols: | similar to BPS1 (BYPASS 1) [Arabidopsis thaliana] (TAIR:AT1G01550.2); similar t	
JCVI_4558	2.646	moderately similar to (209)AT1G71000| Symbols: | DNAJ heat shock N-terminal domain-containing protein | chr1:26772998-26773773	
EV226676	2.646	very weakly similar to (91.3)AT3G16510| Symbols: | C2 domain-containing protein | chr3:5617118-5618200 REVERSE [21493] 1 637 7	
JCVI_22143	2.646	no original description	
EE474581	2.645	weakly similar to (118)AT3G12320| Symbols: | similar to unknown protein [Arabidopsis thaliana] (TAIR:AT5G06980.1); similar to hyp	1.447
JCVI_35317	2.644	no original description	
JCVI_37131	2.644	moderately similar to (236)AT3G46900| Symbols: COPT2 | COPT2 (Copper transporter 2); copper ion transmembrane transporter | chr3:	
EE546160	2.643	moderately similar to (286)AT2G40000| Symbols: HSPRO2, ATHSPRO2 | similar to unknown protein [Arabidopsis thaliana] (TAIR:AT3	1.857
JCVI_12489	2.642	weakly similar to (154)AT3G23930| Symbols: | similar to unknown protein [Arabidopsis thaliana] (TAIR:AT4G13540.1); similar to hyp	
JCVI_24827	2.641	highly similar to (686)AT4G12010| Symbols: | disease resistance protein (TIR-NBS-LRR class), putative | chr4:7197319-7201387 REVE	
ES962263	2.639	no similarity	
JCVI_40401	2.638	no original description	
JCVI_10133	2.637	weakly similar to (167)AT5G57180| Symbols: CIA2 | CIA2 (CHLOROPLAST IMPORT APPARATUS 2) | chr5:23185619-23186749 FC	2.034
EX132818	2.637	moderately similar to (221)AT5G66070| Symbols: | zinc finger (C3HC4-type RING finger) family protein | chr5:26439149-26440259 FC	
JCVI_29717	2.633	moderately similar to (305)AT4G00050| Symbols: UNE10 | UNE10 (unfertilized embryo sac 10); DNA binding / transcription factor | chr	2.463
RC_EX130490	2.632	no similarity	2.512
JCVI_35	2.632	moderately similar to (318)AT1G27730| Symbols: ZAT10, STZ | STZ (SALT TOLERANCE ZINC FINGER); nucleic acid binding / tran	2.825
JCVI_14587	2.631	highly similar to (590)AT3G22880| Symbols: DMC1, ARLIM15, ATDMC1 | ATDMC1 (RECA-LIKE GENE); ATP binding / DNA-depe	
ES987173	2.631	no similarity	
CV546797	2.629	no similarity	
CD842657	2.629	very weakly similar to (80.1)AT2G22470| Symbols: ATAGP2, AGP2 | AGP2 (ARABINOGALACTAN-PROTEIN 2) | chr2:9545480-954	
CX192125	2.623	weakly similar to (179)AT2G39800| Symbols: ATP5CS, P5CS1 | P5CS1 (DELTA1-PYRROLINE-5-CARBOXYLATE SYNTHASE 1) |	
JCVI_7050	2.620	moderately similar to (467)AT5G44730| Symbols: | haloacid dehalogenase-like hydrolase family protein | chr5:18062815-18063746 REV	2.380
EV133520	2.620	moderately similar to (308)AT3G54800| Symbols: | pleckstrin homology (PH) domain-containing protein / lipid-binding START domain-	
JCVI_34508	2.619	moderately similar to (221)AT4G19450| Symbols: | nodulin-related | chr4:10606549-10609229 FORWARD no original description	2.169
EV129366	2.615	weakly similar to (119)AT4G12970| Symbols: | similar to unnamed protein product [Vitis vinifera] (GB:CAO17947.1) | chr4:7586241-75	1.752
EX037889	2.614	moderately similar to (289)AT4G15320| Symbols: CSLB06, ATCSLB6, ATCSLB06 | ATCSLB06 (Cellulose synthase-like B6); transfera	
AM387181	2.613	no similarity	
JCVI_4467	2.613	very weakly similar to (82.0)AT5G14970| Symbols: | amine oxidase/ copper ion binding / quinone binding | chr5:4847371-4848763 FOR	
JCVI_32381	2.610	moderately similar to (229)AT3G08040| Symbols: MAN1, FRD3 | FRD3 (FERRIC REDUCTASE DEFECTIVE 3); antiporter | chr3:256	
JCVI_13251	2.609	weakly similar to (134)AT5G53590| Symbols: | auxin-responsive family protein | chr5:21789333-21789761 FORWARD no original desc	
JCVI_22207	2.604	no original description	
JCVI_1821	2.602	very weakly similar to (98.2)AT1G14180| Symbols: | unknown protein | chr1:4847972-4848801 FORWARD no original description	1.720
JCVI_31508	2.601	moderately similar to (235)AT5G43400| Symbols: | similar to unknown protein [Arabidopsis thaliana] (TAIR:AT5G13210.1); similar to	
JCVI_16182	2.600	moderately similar to (300)AT1G67590| Symbols: | remorin family protein | chr1:25336918-25338135 REVERSE no original description	
JCVI_20074	2.600	weakly similar to (171)AT1G72430| Symbols: | auxin-responsive protein-related | chr1:27268812-27269171 REVERSE no original descr	
JCVI_6244	2.598	highly similar to (742)AT2G35710| Symbols: | glycogenin glucosyltransferase (glycogenin)-related | chr2:15018004-15019330 REVERS	
EV066504	2.597	weakly similar to (101)AT5G15950| Symbols: | adenosylmethionine decarboxylase family protein | chr5:5206709-5207797 FORWARDv	
CV544507	2.596	weakly similar to (114)AT2G40000| Symbols: HSPRO2, ATHSPRO2 | similar to unknown protein [Arabidopsis thaliana] (TAIR:AT3G5	1.848
JCVI_10442	2.596	moderately similar to (486)AT5G62520| Symbols: SRO5 | SRO5 (SIMILAR TO RCD ONE 5); NAD+ ADP-ribosyltransferase | chr5:251	2.103
JCVI_21126	2.594	moderately similar to (216)AT1G04040| Symbols: | acid phosphatase class B family protein | chr1:1042563-1043818 REVERSE no origi	3.431
AT000526	2.592	no similarity	
EV124143	2.588	very weakly similar to (89.4)AT3G16720| Symbols: ATL2 | ATL2 (Arabidopsis T?xicos en Levadura 2); protein binding / zinc ion binding	
JCVI_616	2.582	moderately similar to (429)AT5G59550| Symbols: | zinc finger (C3HC4-type RING finger) family protein | chr5:24015648-24016871 RE	2.271
JCVI_33076	2.578	weakly similar to (117)AT1G05690| Symbols: BT3 | BT3 (BTB and TAZ domain protein 3); protein binding / transcription regulator | chr	
JCVI_30803	2.577	moderately similar to (318)AT3G52060| Symbols: | similar to unknown protein [Arabidopsis thaliana] (TAIR:AT5G22070.1); similar to	2.017
EE460220	2.574	weakly similar to (125)AT5G10930| Symbols: SnRK3.24, CIPK5 | CIPK5 (CBL-INTERACTING PROTEIN KINASE 5); kinase | chr5:3	
JCVI_30647	2.573	no original description	2.365
JCVI_12701	2.573	moderately similar to (268)AT2G21320| Symbols: | zinc finger (B-box type) family protein | chr2:9133583-9134733 FORWARD no orig	
BG543660	2.572	weakly similar to (122)AT2G01190| Symbols: | octicosapeptide/Phox/Bem1p (PB1) domain-containing protein | chr2:115022-117295 FC	
ES897719	2.571	no similarity	
JCVI_5043	2.570	moderately similar to (261)AT2G41010| Symbols: ATCAMBP25 | ATCAMBP25 (ARABIDOPSIS THALIANA CALMODULIN (CAM)	
EV224419	2.570	no similarity	
JCVI_41139	2.567	no original description	1.472
JCVI_4958	2.567	no original description	1.365
JCVI_30564	2.565	no original description	
JCVI_1306	2.565	highly similar to (546)AT1G09130| Symbols: | ATP-dependent Clp protease proteolytic subunit, putative | chr1:2940065-2942219 REVE	
EX016630	2.565	weakly similar to (131)AT1G06170| Symbols: | basic helix-loop-helix (bHLH) family protein | chr1:1885145-1886563 REVERSE [2180	2.722
RC_AM056987	2.564	no similarity	
EE562117	2.562	no similarity	
EV150359	2.561	no similarity	
JCVI_39405	2.559	moderately similar to (236)AT3G62090| Symbols: PIF6, PIL2 | PIL2 (PHYTOCHROME INTERACTING FACTOR 3-LIKE 2) | chr3:23	
JCVI_35156	2.558	moderately similar to (327)AT5G22250| Symbols: | CCR4-NOT transcription complex protein, putative | chr5:7365608-7366444 REVEF	1.716
JCVI_16109	2.558	weakly similar to (113)AT1G76900| Symbols: AtTLP1 | AtTLP1 (TUBBY LIKE PROTEIN 1); phosphoric diester hydrolase/ transcriptio	
JCVI_16715	2.558	moderately similar to (205)AT5G15110| Symbols: | pectate lyase family protein | chr5:4895969-4897685 FORWARDvery weakly simila	
EH430231	2.558	no similarity	1.697
AM390828	2.557	no similarity	2.294
JCVI_27435	2.555	no original description	
JCVI_42224	2.555	no original description	2.595
JCVI_22974	2.554	weakly similar to (118)AT3G02840| Symbols: | immediate-early fungal elicitor family protein | chr3:618487-619626 FORWARD no orig	2.423
JCVI_14413	2.550	no original description	4.969
JCVI_41635	2.549	no original description	
EV121602	2.548	weakly similar to (102)AT1G67590| Symbols: | remorin family protein | chr1:25336918-25338135 REVERSE [21479]	1.685
JCVI_24850	2.546	no original description	
JCVI_28178	2.546	very weakly similar to (99.0)AT5G26760| Symbols: | similar to hypothetical protein OsI_017683 [Oryza sativa (indica cultivar-group)] (C	
JCVI_29711	2.545	weakly similar to (155)AT1G75770| Symbols: | unknown protein | chr1:28453683-28454632 FORWARD no original description	
EE441912	2.545	no similarity	
EE538236	2.545	moderately similar to (214)AT5G47590| Symbols: | heat shock protein-related | chr5:19315171-19316325 REVERSE [20161] 14 665 66	
JCVI_9144	2.544	very weakly similar to (100)AT3G23170| Symbols: | similar to unknown protein [Arabidopsis thaliana] (TAIR:AT4G14450.1) | chr3:826	
DY004157	2.543	no similarity	2.964

JCVI_12056	2.542	weakly similar to (107)AT5G66985| Symbols: | unknown protein | chr5:26760638-26760889 FORWARD no original description	
JCVI_34623	2.541	no original description	
EE450348	2.539	weakly similar to (105)AT5G17300| Symbols: | myb family transcription factor | chr5:5690437-5692437 REVERSE [20170] 1 428 455	2.213
ES908712	2.537	moderately similar to (269)AT1G21326| Symbols: | VQ motif-containing protein | chr1:7468990-7469709 REVERSE [21430]	
EE502099	2.533	no similarity	
EX096129	2.532	no similarity	1.972
JCVI_30427	2.530	moderately similar to (234)AT1G18740| Symbols: | similar to unknown protein [Arabidopsis thaliana] (TAIR:AT1G74450.1); similar to	
EE445350	2.530	no similarity	2.993
JCVI_16916	2.529	moderately similar to (270)AT1G73540| Symbols: ATNUDT21 | ATNUDT21 (Arabidopsis thaliana Nudix hydrolase homolog 21); hydro	
JCVI_26735	2.529	moderately similar to (358)AT1G05560| Symbols: UGT75B1, UGT1 | UGT1 (UDP-glucosyl transferase 75B1); UDP-glycosyltransferase	1.870
ES912597	2.527	no similarity	
JCVI_38066	2.527	moderately similar to (232)AT5G59550| Symbols: | zinc finger (C3HC4-type RING finger) family protein | chr5:24015648-24016871 RE	2.329
JCVI_468	2.524	highly similar to (721)AT1G70740| Symbols: | protein kinase family protein | chr1:26677509-26679349 REVERSEmoderately similar to	1.668
JCVI_31741	2.523	moderately similar to (249)AT3G24520| Symbols: HSFC1, AT-HSFC1 | AT-HSFC1 (Arabidopsis thaliana heat shock transcription factor	2.365
EE423578	2.522	moderately similar to (201)AT5G38150| Symbols: PMI15 | PMI15 (plastid movement impaired 15) | chr5:15240346-15242165 REVERSI	2.036
RC_CV433289	2.520	no similarity	1.211
JCVI_14130	2.518	moderately similar to (359)AT5G49330| Symbols: AtMYB111 | AtMYB111 (myb domain protein 111); DNA binding / transcription facto	
EE559412	2.517	moderately similar to (296)AT5G10930| Symbols: SnRK3.24, CIPK5 | CIPK5 (CBL-INTERACTING PROTEIN KINASE 5); kinase | chi	
ES961878	2.516	weakly similar to (147)AT4G26430| Symbols: CSN6B | CSN6B (COP9 SIGNALOSOME SUBUNIT 6B) | chr4:13355237-13357334 FOI	
JCVI_9781	2.516	weakly similar to (154)AT1G29640| Symbols: | similar to unknown protein [Arabidopsis thaliana] (TAIR:AT2G34340.1); similar to unkı	
JCVI_3294	2.513	moderately similar to (209)AT3G21300| Symbols: | RNA methyltransferase family protein | chr3:7493660-7496263 REVERSE no origin	1.679
ES905653	2.513	very weakly similar to (82.0)AT5G63370| Symbols: | protein kinase family protein | chr5:25402180-25403616 REVERSE [21429]	
JCVI_31056	2.511	weakly similar to (126)AT4G05010| Symbols: | F-box family protein | chr4:2567472-2568160 FORWARD no original description	2.742
JCVI_6659	2.510	moderately similar to (444)AT2G23810| Symbols: TET8 | TET8 (TETRASPANIN8) | chr2:10142939-10144432 REVERSE no original d	
JCVI_23576	2.509	moderately similar to (232)AT3G16720| Symbols: ATL2 | ATL2 (Arabidopsis T?xicos en Levadura 2); protein binding / zinc ion binding	1.344
JCVI_12897	2.508	no original description	2.415
EV213568	2.506	moderately similar to (248)AT2G15300| Symbols: | leucine-rich repeat transmembrane protein kinase, putative | chr2:6656712-6659092 I	1.914
JCVI_16980	2.506	moderately similar to (376)AT1G12500| Symbols: | phosphate translocator-related | chr1:4263540-4264955 REVERSE no original descri	
JCVI_29716	2.504	weakly similar to (105)AT5G17230| Symbols: PSY | PSY (PHYTOENE SYNTHASE) | chr5:5659841-5662089 REVERSEvery weakly s	1.447
JCVI_34766	2.502	moderately similar to (288)AT4G37260| Symbols: MYB73, AtMYB73 | AtMYB73/MYB73 (myb domain protein 73); DNA binding / tra	
CD821801	2.501	weakly similar to (101)AT4G34600| Symbols: | similar to unknown protein [Arabidopsis thaliana] (TAIR:AT2G16385.1) | chr4:1652928	
JCVI_23539	2.501	moderately similar to (291)AT1G66840| Symbols: | Identical to Protein PLASTID MOVEMENT IMPAIRED 2 (PMI2) [Arabidopsis Tha	2.029
JCVI_34618	2.501	moderately similar to (371)AT5G66210| Symbols: CPK28 | CPK28 (calcium-dependent protein kinase 28) | chr5:26474371-26476660 RE	1.782
EX039879	2.500	moderately similar to (310)AT1G01520| Symbols: | myb family transcription factor | chr1:190596-192139 FORWARD [21811]	
JCVI_30847	2.499	weakly similar to (109)AT2G25735| Symbols: | unknown protein | chr2:10982423-10982782 REVERSE no original description	
JCVI_42505	2.498	moderately similar to (300)AT1G25400| Symbols: | similar to unknown protein [Arabidopsis thaliana] (TAIR:AT1G68440.1); similar to	
EV132889	2.497	no similarity	1.853
EE557380	2.496	no similarity	2.008
EV082737	2.496	no similarity	2.548
RC_H74690	2.495	no similarity	2.567
JCVI_3653	2.492	moderately similar to (473)AT1G26480| Symbols: GF14 IOTA, GRF12 | GRF12 (GENERAL REGULATORY FACTOR 12); protein pho	3.259
EV066279	2.491	no similarity	
JCVI_5377	2.488	moderately similar to (240)AT2G04680| Symbols: | DC1 domain-containing protein | chr2:1640185-1642158 FORWARD no original des	
DY029300	2.488	no similarity	
JCVI_22830	2.487	weakly similar to (168)AT2G34340| Symbols: | similar to unknown protein [Arabidopsis thaliana] (TAIR:AT1G29640.1); similar to unkı	
EV124854	2.485	moderately similar to (414)AT4G23190| Symbols: AT-RLK3, CRK11 | CRK11 (CYSTEINE-RICH RLK11); kinase | chr4:12141208-121	
JCVI_40185	2.485	weakly similar to (194)AT1G74890| Symbols: ARR15 | ARR15 (RESPONSE REGULATOR 15); transcription regulator | chr1:28135251	
JCVI_36250	2.483	very weakly similar to (92.8)AT3G61640| Symbols: ATAGP20, AGP20 | AGP20 (ARABINOGALACTAN PROTEIN 20) | chr3:2282125	
JCVI_21619	2.478	no original description	2.283
EV132187	2.478	no similarity	
EE462195	2.478	very weakly similar to (100)AT3G24255| Symbols: | similar to unknown protein [Arabidopsis thaliana] (TAIR:AT3G23910.1); similar to	
EE543569	2.475	weakly similar to (191)AT1G73066| Symbols: | protein binding | chr1:27485446-27487242 FORWARDweakly similar to (120)RPK1_II	
JCVI_21877	2.475	weakly similar to (102)AT1G32928| Symbols: | similar to unknown protein [Arabidopsis thaliana] (TAIR:AT1G32920.1) | chr1:1193131	2.337
JCVI_5269	2.474	weakly similar to (168)AT1G14200| Symbols: | zinc finger (C3HC4-type RING finger) family protein | chr1:4854527-4855066 REVERS	2.142
EE554654	2.471	weakly similar to (185)AT3G11020| Symbols: DREB2, DREB2B | DREB2B (DRE-binding protein 2B); DNA binding / transcription acti	
EV198526	2.467	no similarity	
JCVI_23772	2.466	weakly similar to (141)AT5G42660| Symbols: | similar to unknown protein [Arabidopsis thaliana] (TAIR:AT2G02910.1); similar to unna	
EV214080	2.463	moderately similar to (237)AT3G15115| Symbols: | similar to unknown protein [Arabidopsis thaliana] (TAIR:AT1G53180.1) | chr3:5086	
JCVI_37519	2.463	weakly similar to (129)AT1G26590| Symbols: | zinc finger (C2H2 type) family protein | chr1:9189611-9190696 FORWARD no original	
JCVI_19864	2.462	moderately similar to (402)AT1G24140| Symbols: | matrixin family protein | chr1:8536120-8537274 REVERSEweakly similar to (185)M	
JCVI_19926	2.457	no original description	
JCVI_23713	2.455	highly similar to (593)AT1G13710| Symbols: CYP78A5 | CYP78A5 (cytochrome P450, family 78, subfamily A, polypeptide 5); oxygen l	
EV137277	2.454	no similarity	1.457
JCVI_37887	2.452	weakly similar to (188)AT4G21895| Symbols: | similar to AT hook motif-containing protein [Arabidopsis thaliana] (TAIR:AT5G52890.1	
EV103398	2.451	no similarity	
H74690	2.449	no similarity	2.436
JCVI_4015	2.448	weakly similar to (159)AT4G02740| Symbols: | Identical to F-box protein At4g02740 [Arabidopsis Thaliana] (GB:Q0WRC9;GB:Q683B	
JCVI_11272	2.447	highly similar to (514)AT2G46070| Symbols: ATMPK12 | ATMPK12 (Arabidopsis thaliana MAP kinase 12); MAP kinase/ kinase | chr2:	1.769
ES900133	2.443	weakly similar to (167)AT5G45960| Symbols: | GDSL-motif lipase/hydrolase family protein | chr5:18654401-18657546 REVERSEvery	2.195
JCVI_12498	2.443	very weakly similar to (94.7)AT2G16005| Symbols: | MD-2-related lipid recognition domain-containing protein / ML domain-containing	
RC_EE566066	2.437	no similarity	3.353
DN961108	2.437	moderately similar to (265)AT3G26220| Symbols: CYP71B3 | CYP71B3 (cytochrome P450, family 71, subfamily B, polypeptide 3); oxy	
CD837899	2.436	no similarity	
EV170912	2.436	weakly similar to (191)AT5G25900| Symbols: CYP701A3, GA3 | GA3 (GA REQUIRING 3); oxygen binding | chr5:9036076-9038281 F(	
JCVI_20622	2.436	no original description	2.048
EV091268	2.435	moderately similar to (214)AT5G39530| Symbols: | similar to unknown protein [Arabidopsis thaliana] (TAIR:AT5G39520.1); similar to	
JCVI_19317	2.434	weakly similar to (189)AT4G30880| Symbols: | protease inhibitor/seed storage/lipid transfer protein (LTP) family protein | chr4:1503523	
EE547032	2.434	weakly similar to (107)AT5G55620| Symbols: | similar to unknown protein [Arabidopsis thaliana] (TAIR:AT3G09950.1); similar to unna	1.583
EX089219	2.433	weakly similar to (110)AT5G40230| Symbols: | nodulin-related | chr5:16097042-16098963 REVERSE [21823]	
ES969208	2.433	weakly similar to (140)AT1G15890| Symbols: | disease resistance protein (CC-NBS-LRR class), putative | chr1:5461400-5463955 FORV	
JCVI_35549	2.431	no original description	
JCVI_28155	2.431	weakly similar to (177)AT4G24700| Symbols: | unknown protein | chr4:12744817-12745248 REVERSE no original description	1.491
JCVI_37808	2.431	weakly similar to (111)AT1G33990| Symbols: | hydrolase, alpha/beta fold family protein | chr1:12355889-12357874 FORWARD no orig	3.250

JCVI_28194	2.430	highly similar to (590)AT5G39080| Symbols: | transferase family protein | chr5:15658909-15660300 FORWARD no original description	2.848
EV095717	2.429	no similarity	
EV120020	2.429	no similarity	
EX135350	2.427	weakly similar to (146)AT3G61850| Symbols: DAG1 | DAG1 (DOF AFFECTING GERMINATION 1) | chr3:22906470-22908125 FORV	2.109
EV150514	2.423	moderately similar to (269)AT4G14080| Symbols: MEE48 | MEE48 (maternal effect embryo arrest 48); hydrolase, hydrolyzing O-glycosy	
EV167695	2.423	weakly similar to (181)AT5G05190| Symbols: | Identical to Uncharacterized protein At5g05190 (Y-1) [Arabidopsis Thaliana] (GB:Q9FF	
CX268655	2.420	weakly similar to (102)AT5G17790| Symbols: VAR3 | VAR3 (VARIEGATED 3); binding | chr5:5869812-5872673 REVERSE [16816] 1	
JCVI_12229	2.419	moderately similar to (328)AT3G21870| Symbols: CYCP2;1 | CYCP2;1 (cyclin p2;1); cyclin-dependent protein kinase | chr3:7703934-77	2.436
EV132939	2.418	no similarity	
EV165235	2.418	weakly similar to (155)AT1G24530| Symbols: | transducin family protein / WD-40 repeat family protein | chr1:8693274-8694530 FORW	2.110
EX123750	2.415	very weakly similar to (99.4)AT5G54300| Symbols: | similar to unknown protein [Arabidopsis thaliana] (TAIR:AT1G61260.1); similar to	
EE564839	2.415	very weakly similar to (81.3)AT1G07010| Symbols: | calcineurin-like phosphoesterase family protein | chr1:2152948-2154967 FORWAR	
EV116241	2.414	moderately similar to (283)AT5G39670| Symbols: | calcium-binding EF hand family protein | chr5:15900498-15901112 FORWARD [21	
DY026840	2.414	no similarity	1.784
JCVI_22811	2.413	moderately similar to (202)AT4G14690| Symbols: ELIP2 | ELIP2 (EARLY LIGHT-INDUCIBLE PROTEIN 2); chlorophyll binding | chr	2.354
EE561389	2.413	moderately similar to (299)AT2G27210| Symbols: | kelch repeat-containing serine/threonine phosphoesterase family protein | chr2:11637	
JCVI_13374	2.413	moderately similar to (303)AT1G70260| Symbols: | nodulin MtN21 family protein | chr1:26460730-26463001 REVERSE no original des	2.750
EV090686	2.413	weakly similar to (176)AT3G24420| Symbols: | hydrolase, alpha/beta fold family protein | chr3:8863118-8864890 REVERSE [21476] 10	2.488
EX042931	2.413	moderately similar to (333)AT4G38960| Symbols: | zinc finger (B-box type) family protein | chr4:18162294-18163039 FORWARD [218	
JCVI_4284	2.411	no original description	
EV130781	2.409	no similarity	
JCVI_7784	2.409	moderately similar to (235)AT4G37260| Symbols: MYB73, AtMYB73 | AtMYB73/MYB73 (myb domain protein 73); DNA binding / tra	
CD825311	2.405	weakly similar to (145)AT3G24255| Symbols: | similar to unknown protein [Arabidopsis thaliana] (TAIR:AT3G23910.1); similar to unna	
JCVI_24635	2.403	moderately similar to (280)AT2G38160| Symbols: | similar to unknown protein [Arabidopsis thaliana] (TAIR:AT2G40070.1); similar to	2.245
JCVI_18765	2.401	moderately similar to (290)AT1G07615| Symbols: | GTP binding | chr1:2342274-2344197 REVERSE no original description	
CX194043	2.399	moderately similar to (337)AT3G55060| Symbols: | similar to unknown protein [Arabidopsis thaliana] (TAIR:AT2G39300.1); similar to	
JCVI_32817	2.393	moderately similar to (396)AT4G01950| Symbols: ATGPAT3, GPAT3 | ATGPAT3/GPAT3 (GLYCEROL-3-PHOSPHATE ACYLTRAN	
JCVI_3997	2.393	highly similar to (504)AT5G15900| Symbols: | similar to unknown protein [Arabidopsis thaliana] (TAIR:AT5G15890.1); similar to unnar	
EE450982	2.391	moderately similar to (317)AT1G75100| Symbols: JAC1 | JAC1 (J-DOMAIN PROTEIN REQUIRED FOR CHLOROPLAST ACCUMUI	
JCVI_17257	2.390	moderately similar to (318)AT5G15300| Symbols: | pentatricopeptide (PPR) repeat-containing protein | chr5:4968387-4970033 REVERS	
JCVI_33958	2.388	moderately similar to (317)AT4G32860| Symbols: | similar to unknown [Populus trichocarpa x Populus deltoides] (GB:ABK96753.1) | cl	
EV177936	2.387	weakly similar to (161)AT4G16550| Symbols: | heat shock protein-related | chr4:9318464-9324276 REVERSE [21487] 142 919 919	
EE423815	2.386	weakly similar to (140)AT4G19400| Symbols: | actin binding | chr4:10581048-10581920 REVERSE [20158]	2.031
JCVI_31295	2.385	weakly similar to (136)AT5G44005| Symbols: | unknown protein | chr5:17722436-17722720 REVERSE no original description	
JCVI_38427	2.383	no original description	
EE404663	2.382	no similarity	1.340
JCVI_29410	2.379	moderately similar to (338)AT2G26190| Symbols: | calmodulin-binding family protein | chr2:11154979-11157160 REVERSE no original	
JCVI_28898	2.378	no original description	
JCVI_13323	2.378	moderately similar to (271)AT5G06690| Symbols: | (THIOREDOXIN-LIKE 5); thiol-disulfide exchange intermediate | chr5:2060853-20	2.279
JCVI_2658	2.378	weakly similar to (134)AT3G56880| Symbols: | VQ motif-containing protein | chr3:21071023-21071760 FORWARD no original descrip	
JCVI_514	2.376	moderately similar to (383)AT3G15354| Symbols: SPA3 | SPA3 (SPA1-RELATED 3); signal transducer | chr3:5169334-5172487 REVEI	1.740
RC_AM387464	2.375	no similarity	
JCVI_39744	2.375	weakly similar to (199)AT1G31740| Symbols: BGAL15 | BGAL15 (beta-galactosidase 15); beta-galactosidase | chr1:11365266-11369889	
EX135021	2.372	weakly similar to (109)AT2G46400| Symbols: ATWRKY46, WRKY46 | WRKY46 (WRKY DNA-binding protein 46); transcription facto	
JCVI_34717	2.370	moderately similar to (459)AT4G31020| Symbols: | similar to unknown protein [Arabidopsis thaliana] (TAIR:AT2G24320.1); similar to	
JCVI_28085	2.364	moderately similar to (207)AT4G21200| Symbols: ATGA2OX8 | ATGA2OX8 (GIBBERELLIN 2-OXIDASE 8); gibberellin 2-beta-dioxy	2.360
EV145867	2.364	no similarity	
JCVI_36064	2.363	moderately similar to (320)AT2G17040| Symbols: ANAC036 | ANAC036 (Arabidopsis NAC domain containing protein 36); transcriptio	
EX119365	2.363	moderately similar to (361)AT3G55580| Symbols: | regulator of chromosome condensation (RCC1) family protein | chr3:20623745-2062	
EV173310	2.363	no similarity	1.440
EE513584	2.362	no similarity	2.422
EX125631	2.359	no similarity	
JCVI_24165	2.356	moderately similar to (305)AT1G07010| Symbols: | calcineurin-like phosphoesterase family protein | chr1:2152948-2154967 FORWARD	1.403
JCVI_41748	2.354	no original description	
AM057705	2.354	weakly similar to (119)AT2G43470| Symbols: | similar to unknown protein [Arabidopsis thaliana] (TAIR:AT3G03320.1); similar to unna	
EV124770	2.354	weakly similar to (169)AT4G23190| Symbols: AT-RLK3, CRK11 | CRK11 (CYSTEINE-RICH RLK11); kinase | chr4:12141208-121437	
JCVI_34254	2.352	very weakly similar to (84.3)AT3G15357| Symbols: | unknown protein | chr3:5187171-5187602 FORWARD no original description	
JCVI_22252	2.350	moderately similar to (406)AT3G19970| Symbols: | similar to unknown protein [Arabidopsis thaliana] (TAIR:AT2G18245.1); similar to	
EE404244	2.349	moderately similar to (236)AT3G22410| Symbols: | similar to unknown protein [Arabidopsis thaliana] (TAIR:AT1G05370.1); similar to	
EV225869	2.348	very weakly similar to (86.3)AT1G47128| Symbols: RD21A, RD21 | RD21 (RESPONSIVE TO DEHYDRATION 21); cysteine-type pept	
JCVI_22813	2.348	no original description	
JCVI_15274	2.348	weakly similar to (145)AT1G72390| Symbols: | similar to unnamed protein product [Vitis vinifera] (GB:CAO45587.1) | chr1:27249117-2	
JCVI_18512	2.347	weakly similar to (187)AT1G52250| Symbols: | dynein light chain type 1 family protein | chr1:19462513-19462904 REVERSE no origin	
EL589385	2.347	moderately similar to (346)AT5G46910| Symbols: | transcription factor jumonji (jmj) family protein | chr5:19065007-19068107 FORWA	
EE555489	2.346	no similarity	
EX062936	2.343	weakly similar to (121)AT4G23160| Symbols: | protein kinase family protein | chr4:12129496-12134097 FORWARD [21814] 15 594 59	
JCVI_35452	2.342	no original description	3.296
JCVI_27061	2.341	very weakly similar to (86.3)AT4G34150| Symbols: | C2 domain-containing protein | chr4:16355039-16356959 FORWARD no original d	
EV098668	2.341	no similarity	
EG019920	2.339	very weakly similar to (82.8)AT2G34720| Symbols: | CCAAT-binding transcription factor (CBF-B/NF-YA) family protein | chr2:146570	1.953
DY001046	2.337	moderately similar to (269)AT3G06145| Symbols: | similar to unnamed protein product [Vitis vinifera] (GB:CAO71096.1) | chr3:186012	3.615
JCVI_10088	2.337	no original description	
ES952471	2.336	no similarity	2.133
RC_JCVI_20887	2.335	no original description	
JCVI_27603	2.333	moderately similar to (309)AT4G38960| Symbols: | zinc finger (B-box type) family protein | chr4:18162294-18163039 FORWARD no o	1.394
EE568852	2.331	very weakly similar to (83.6)AT1G44160| Symbols: | DNAJ chaperone C-terminal domain-containing protein | chr1:16797472-16798716	
JCVI_17306	2.331	moderately similar to (250)AT5G13770| Symbols: | pentatricopeptide (PPR) repeat-containing protein | chr5:4445464-4447293 FORWA	1.737
ES902908	2.330	moderately similar to (244)AT2G35380| Symbols: | peroxidase 20 (PER20) (P20) | chr2:14900188-14901037 FORWARDweakly similar	
JCVI_39496	2.330	weakly similar to (127)AT3G56290| Symbols: | similar to hypothetical protein [Vitis vinifera] (GB:CAN75527.1) | chr3:20889722-20890	3.442
JCVI_19361	2.325	moderately similar to (399)AT3G28910| Symbols: ATMYB30, MYB30 | MYB30 (myb domain protein 30); DNA binding / transcription	
EE568032	2.324	weakly similar to (112)AT5G42240| Symbols: SCPL42 | SCPL42 (serine carboxypeptidase-like 42); serine carboxypeptidase | chr5:16905	
AT001877	2.324	no similarity	1.805
CD817870	2.324	moderately similar to (248)AT5G42630| Symbols: KAN4, ATS | ATS/KAN4 (ABERRANT TESTA SHAPE) | chr5:17091808-17092975	

JCVI_6789	2.324	no original description	
JCVI_34189	2.322	moderately similar to (246)AT5G50530| Symbols: | CBS domain-containing protein / octicosapeptide/Phox/Bemp1 (PB1) domain-contai	
JCVI_27468	2.322	moderately similar to (324)AT5G15890| Symbols: | similar to unknown protein [Arabidopsis thaliana] (TAIR:AT5G15900.1); similar to	
DY015590	2.322	very weakly similar to (87.8)AT5G59350| Symbols: | similar to unnamed protein product [Vitis vinifera] (GB:CAO21684.1) | chr5:23958	
JCVI_12037	2.322	moderately similar to (353)AT3G16510| Symbols: | C2 domain-containing protein | chr3:5617118-5618200 REVERSE no original descri	
EV197575	2.319	moderately similar to (285)AT3G13030| Symbols: | hAT dimerisation domain-containing protein | chr3:4169682-4171424 REVERSE [21	1.704
EV110835	2.317	no similarity	
DY008941	2.317	no similarity	
JCVI_38912	2.316	moderately similar to (422)AT3G15354| Symbols: SPA3 | SPA3 (SPA1-RELATED 3); signal transducer | chr3:5169334-5172487 REVEI	1.814
EE455707	2.315	weakly similar to (139)AT5G55890| Symbols: | similar to unknown protein [Arabidopsis thaliana] (TAIR:AT5G55880.1); contains InterI	
JCVI_11064	2.314	very weakly similar to (89.4)AT5G22460| Symbols: | esterase/lipase/thioesterase family protein | chr5:7443662-7445272 REVERSE no o	
EV137742	2.314	no similarity	1.888
JCVI_29720	2.313	moderately similar to (249)AT5G17780| Symbols: | hydrolase, alpha/beta fold family protein | chr5:5867431-5868978 REVERSE no orig	1.349
JCVI_1823	2.312	moderately similar to (269)AT3G14200| Symbols: | DNAJ heat shock N-terminal domain-containing protein | chr3:4712888-4714368 RE	1.874
JCVI_15084	2.310	moderately similar to (212)AT3G16720| Symbols: ATL2 | ATL2 (Arabidopsis T?xicos en Levadura 2); protein binding / zinc ion binding	
EV182475	2.310	weakly similar to (176)AT2G18240| Symbols: | RER1 protein, putative | chr2:7942548-7943546 FORWARD [21487]	
EE475842	2.309	weakly similar to (157)AT1G75100| Symbols: JAC1 | JAC1 (J-DOMAIN PROTEIN REQUIRED FOR CHLOROPLAST ACCUMULAT	1.556
EX115234	2.309	moderately similar to (251)AT3G60420| Symbols: | similar to unknown protein [Arabidopsis thaliana] (TAIR:AT3G60450.1); similar to	
JCVI_26208	2.307	moderately similar to (322)AT5G61380| Symbols: APRR1, PRR1, TOC1 | TOC1 (TIMING OF CAB EXPRESSION 1); transcription reg	2.179
EV182967	2.305	no similarity	
JCVI_16649	2.305	weakly similar to (113)AT5G40810| Symbols: | cytochrome c1, putative | chr5:16357428-16359555 FORWARDweakly similar to (111)	
DY025805	2.304	moderately similar to (333)AT3G48270| Symbols: CYP71A26 | CYP71A26 (cytochrome P450, family 71, subfamily A, polypeptide 26);	
JCVI_18755	2.304	weakly similar to (113)AT3G14595| Symbols: | similar to unknown protein [Arabidopsis thaliana] (TAIR:AT1G17080.1); similar to unkr	
JCVI_39284	2.304	weakly similar to (125)AT5G15430| Symbols: | calmodulin-binding protein-related | chr5:5010183-5011619 FORWARD no original des	
JCVI_7087	2.302	moderately similar to (210)AT5G48220| Symbols: | indole-3-glycerol phosphate synthase, putative | chr5:19567665-19569272 FORWAI	1.450
ES999466	2.302	moderately similar to (225)AT3G14080| Symbols: | small nuclear ribonucleoprotein, putative / snRNP, putative / Sm protein, putative | cl	1.552
EV226406	2.301	no similarity	
JCVI_29048	2.301	no original description	
JCVI_42194	2.300	no original description	
EV020693	2.298	weakly similar to (109)AT2G45760| Symbols: BAL, BAP2 | BAP2 (BON ASSOCIATION PROTEIN 2) | chr2:18854199-18854822 REV	
JCVI_26518	2.297	moderately similar to (340)AT2G47650| Symbols: UXS4 | UXS4 (UDP-XYLOSE SYNTHASE 4); catalytic | chr2:19545821-19548434 F	
EV108938	2.297	very weakly similar to (88.6)CYSA_CUCSA [21478]	
JCVI_34125	2.297	moderately similar to (499)AT2G30020| Symbols: | protein phosphatase 2C, putative / PP2C, putative | chr2:12821514-12822981 FORW	
DW999792	2.296	no similarity	1.934
EV091965	2.296	weakly similar to (167)AT3G59400| Symbols: GUN4 | GUN4 (Genomes uncoupled 4) | chr3:21959858-21960655 REVERSE [21476]	2.432
ES957665	2.294	no similarity	
DY020656	2.293	moderately similar to (214)AT3G24520| Symbols: HSFC1, AT-HSFC1 | AT-HSFC1 (Arabidopsis thaliana heat shock transcription factor	
JCVI_10560	2.293	moderately similar to (210)AT5G06530| Symbols: | ABC transporter family protein | chr5:1990335-1994606 REVERSE no original desc	1.761
JCVI_1578	2.292	very weakly similar to (87.4)AT3G59940| Symbols: | kelch repeat-containing F-box family protein | chr3:22153986-22155242 FORWAR	
JCVI_33192	2.291	weakly similar to (132)AT1G28370| Symbols: ERF11, ATERF11 | ATERF11/ERF11 (ERF domain protein 11); DNA binding / transcript	
JCVI_21367	2.289	moderately similar to (299)AT2G32030| Symbols: | GCN5-related N-acetyltransferase (GNAT) family protein | chr2:13639752-1364031	
JCVI_4747	2.289	moderately similar to (298)AT3G19184| Symbols: | DNA binding | chr3:6637561-6639041 FORWARD no original description	
JCVI_17360	2.289	moderately similar to (459)AT3G51860| Symbols: ATHCX1, CAX1-LIKE, ATCAX3, CAX3 | CAX3 (cation exchanger 3); cation:cation	
CV433836	2.289	no similarity	
EV142995	2.288	no similarity	
JCVI_16772	2.287	moderately similar to (254)AT1G70420| Symbols: | similar to unknown protein [Arabidopsis thaliana] (TAIR:AT1G23710.1); similar to	2.136
EE482280	2.285	no similarity	
JCVI_12276	2.284	moderately similar to (325)AT2G37970| Symbols: SOUL-1 | SOUL-1; binding | chr2:15898105-15898782 FORWARD no original descri	
CB686367	2.283	no similarity	
ES967756	2.282	very weakly similar to (87.4)AT5G58570| Symbols: | unknown protein | chr5:23690096-23690356 REVERSE [20153] 1 505 518	
EV135776	2.279	no similarity	
JCVI_25479	2.278	highly similar to (582)AT4G22100| Symbols: | glycosyl hydrolase family 1 protein | chr4:11707382-11709944 REVERSEmoderately sim	
EV057520	2.273	no similarity	
JCVI_36889	2.273	moderately similar to (282)AT4G36550| Symbols: | binding / ubiquitin-protein ligase | chr4:17245403-17247721 REVERSEvery weakly	
JCVI_875	2.273	weakly similar to (166)AT1G28370| Symbols: ERF11, ATERF11 | ATERF11/ERF11 (ERF domain protein 11); DNA binding / transcript	
ES271655	2.272	moderately similar to (328)AT1G31740| Symbols: BGAL15 | BGAL15 (beta-galactosidase 15); beta-galactosidase | chr1:11365266-1136	
JCVI_9499	2.272	weakly similar to (139)AT5G27770| Symbols: | 60S ribosomal protein L22 (RPL22C) | chr5:9836170-9837117 FORWARD no original c	
JCVI_18505	2.271	moderately similar to (487)AT4G33050| Symbols: EDA39 | EDA39 (embryo sac development arrest 39) | chr4:15945238-15946739 REV	
JCVI_37060	2.270	highly similar to (558)AT1G42550| Symbols: PMI1 | PMI1 (PLASTID MOVEMENT IMPAIRED1) | chr1:15979976-15982174 FORWA	
JCVI_32916	2.270	moderately similar to (263)AT5G27420| Symbols: | zinc finger (C3HC4-type RING finger) family protein | chr5:9684122-9685228 FOR	1.732
JCVI_31517	2.266	highly similar to (706)AT1G28130| Symbols: GH3.17 | GH3.17 | chr1:9826366-9827870 FORWARDmoderately similar to (439)GH311	2.565
JCVI_41437	2.264	moderately similar to (203)AT5G27420| Symbols: | zinc finger (C3HC4-type RING finger) family protein | chr5:9684122-9685228 FOR	1.540
EV202472	2.263	moderately similar to (322)AT3G14920| Symbols: | similar to unknown protein [Arabidopsis thaliana] (TAIR:AT5G05480.1); similar to	
JCVI_23112	2.262	weakly similar to (113)AT3G24600| Symbols: | similar to unknown protein [Arabidopsis thaliana] (TAIR:AT4G35170.1); similar to unna	
JCVI_487	2.262	highly similar to (586)AT1G18740| Symbols: | similar to unknown protein [Arabidopsis thaliana] (TAIR:AT1G74450.1); similar to unnai	1.651
JCVI_35336	2.259	weakly similar to (151)AT5G48657| Symbols: | defense protein-related | chr5:19751346-19752742 REVERSE no original description	
ES919907	2.252	moderately similar to (280)AT1G17145| Symbols: | protein binding / zinc ion binding | chr1:5860344-5862234 REVERSE [15718]	1.453
JCVI_35793	2.252	highly similar to (829)AT2G36190| Symbols: ATCWINV4 | ATCWINV4 (ARABIDOPSIS THALIANA CELL WALL INVERTASE 4);	
EX056114	2.251	weakly similar to (192)AT3G02380| Symbols: COL2 | COL2 (CONSTANS-LIKE 2); transcription factor/ zinc ion binding | chr3:487445-	2.443
JCVI_42329	2.251	weakly similar to (138)AT1G80760| Symbols: NIP6;1, NLM7, NIP6 | NIP6;1 (NOD26-like intrinsic protein 6;1); water channel | chr1:30	
JCVI_10712	2.250	moderately similar to (315)AT5G24120| Symbols: SIG5, SIGE | SIGE (RNA polymerase sigma subunit E); DNA binding / DNA-directed	
JCVI_13791	2.249	moderately similar to (358)AT5G62140| Symbols: | similar to unknown [Populus trichocarpa] (GB:ABK94834.1) | chr5:24971789-24972	2.108
JCVI_21574	2.249	weakly similar to (139)AT1G58520| Symbols: RXW8 | RXW8 | chr1:21737208-21741830 FORWARD no original description	
ES953055	2.246	no similarity	
ES949089	2.244	weakly similar to (152)AT3G13020| Symbols: | hAT dimerisation domain-containing protein | chr3:4167002-4168924 REVERSE [21393	1.796
EV141934	2.244	no similarity	
JCVI_42114	2.242	no original description	2.305
EV226520	2.242	weakly similar to (143)AT5G54600| Symbols: | 50S ribosomal protein L24, chloroplast (CL24) | chr5:22200272-22201629 FORWARD	
JCVI_8092	2.241	weakly similar to (173)AT5G55620| Symbols: | similar to unknown protein [Arabidopsis thaliana] (TAIR:AT3G09950.1); similar to unna	1.452
ES906146	2.240	moderately similar to (362)AT2G17080| Symbols: | similar to unknown protein [Arabidopsis thaliana] (TAIR:AT2G17070.1); similar to	2.608
JCVI_37786	2.239	no original description	
JCVI_26525	2.239	moderately similar to (339)AT1G07010| Symbols: | calcineurin-like phosphoesterase family protein | chr1:2152948-2154967 FORWARI	
JCVI_16615	2.238	moderately similar to (454)AT4G12400| Symbols: | stress-inducible protein, putative | chr4:7338863-7341236 REVERSEmoderately sim	2.201

ES966375	2.238	no similarity	
JCVI_5109	2.237	moderately similar to (385)AT5G15850| Symbols: COL1 | COL1 (CONSTANS-LIKE 1); transcription factor/ zinc ion binding | chr5:517(	
AM391388	2.237	moderately similar to (312)AT3G50560| Symbols: | short-chain dehydrogenase/reductase (SDR) family protein | chr3:18772228-1877481	2.082
EE556723	2.237	no similarity	
JCVI_6722	2.235	weakly similar to (135)AT5G65300| Symbols: | unknown protein | chr5:26112492-26112944 REVERSE no original description	1.914
EX027155	2.234	moderately similar to (293)AT1G14480| Symbols: | protein binding | chr1:4956399-4957883 FORWARD [21810]	
JCVI_20559	2.234	no original description	
JCVI_18985	2.232	moderately similar to (330)AT1G77380| Symbols: AAP3 | AAP3 (amino acid permease 3); amino acid transmembrane transporter | chr1:2	
JCVI_8434	2.231	moderately similar to (236)AT5G61490| Symbols: | similar to unknown protein [Arabidopsis thaliana] (TAIR:AT4G25170.1); similar to u	1.238
JCVI_16435	2.230	moderately similar to (354)AT1G70090| Symbols: GATL9, LGT8 | GATL9/LGT8 (Galacturonosyltransferase-like 9) | chr1:26404590-26	1.963
JCVI_30712	2.229	weakly similar to (167)AT4G15248| Symbols: | DNA binding / zinc ion binding | chr4:8708881-8709234 FORWARD no original descrip	1.808
JCVI_2425	2.227	moderately similar to (339)AT2G41860| Symbols: CPK14 | CPK14 (calcium-dependent protein kinase 14); calmodulin-dependent protein	
JCVI_9329	2.226	moderately similar to (317)AT1G70090| Symbols: GATL9, LGT8 | GATL9/LGT8 (Galacturonosyltransferase-like 9) | chr1:26404590-26	
JCVI_35269	2.226	no original description	
JCVI_11814	2.226	no original description	
JCVI_20419	2.225	very weakly similar to (84.0)AT3G17609| Symbols: HYH | HYH (HY5-HOMOLOG) | chr3:6023977-6024591 FORWARD no original de	1.625
RC_ES957953	2.225	no similarity	2.906
CN727151	2.224	no similarity	1.222
RC_EE557217	2.223	no similarity	
JCVI_38669	2.222	moderately similar to (302)AT1G32640| Symbols: RD22BP1, JAI1, JIN1, MYC2, ZBF1, ATMYC2 | ATMYC2 (JASMONATE INSENS	
ES956130	2.222	no similarity	
ES266382	2.221	moderately similar to (255)AT3G60680| Symbols: | similar to unknown protein [Arabidopsis thaliana] (TAIR:AT2G45260.1); similar to u	
EX095680	2.219	moderately similar to (275)AT3G02840| Symbols: | immediate-early fungal elicitor family protein | chr3:618487-619626 FORWARD [21	
JCVI_35880	2.219	highly similar to (697)AT1G23030| Symbols: | armadillo/beta-catenin repeat family protein / U-box domain-containing protein | chr1:815	
JCVI_10244	2.219	very weakly similar to (90.1)AT1G07985| Symbols: | Expressed protein | chr1:2475505-2475939 FORWARD no original description	
ES936687	2.218	no similarity	
JCVI_22426	2.217	very weakly similar to (96.7)AT4G29280| Symbols: LCR22 | LCR22 (Low-molecular-weight cysteine-rich 22) | chr4:14427907-14428271	2.528
EE459037	2.217	very weakly similar to (88.2)AT1G12800| Symbols: | S1 RNA-binding domain-containing protein | chr1:4361776-4365187 REVERSE [2(	
JCVI_14465	2.216	moderately similar to (207)AT5G23280| Symbols: | TCP family transcription factor, putative | chr5:7843020-7843772 FORWARD no or	
JCVI_8027	2.215	moderately similar to (449)AT1G05690| Symbols: BT3 | BT3 (BTB and TAZ domain protein 3); protein binding / transcription regulator	
JCVI_10630	2.213	weakly similar to (111)AT5G23130| Symbols: | peptidoglycan-binding LysM domain-containing protein | chr5:7781478-7783336 FORW	
JCVI_31620	2.213	weakly similar to (119)AT1G28640| Symbols: | GDSL-motif lipase, putative | chr1:10067549-10069095 REVERSE no original descriptio	2.389
EV054282	2.212	weakly similar to (101)AT2G45740| Symbols: PEX11D | PEX11D | chr2:18846939-18848176 FORWARD [21442]	
JCVI_4831	2.212	weakly similar to (179)AT3G46600| Symbols: | scarecrow transcription factor family protein | chr3:17169037-17170784 FORWARD no	
JCVI_10851	2.212	moderately similar to (315)AT4G18010| Symbols: IP5PII | IP5PII (INOSITOL POLYPHOSPHATE 5-PHOSPHATASE II); inositol-polyp	
EX085956	2.211	very weakly similar to (92.8)AT2G04790| Symbols: | similar to unnamed protein product [Vitis vinifera] (GB:CAO23994.1) | chr2:16795	1.933
JCVI_15429	2.211	weakly similar to (113)AT5G19875| Symbols: | similar to unknown protein [Arabidopsis thaliana] (TAIR:AT2G31940.1); similar to hypo	
JCVI_34442	2.211	weakly similar to (176)AT4G36500| Symbols: | similar to unknown protein [Arabidopsis thaliana] (TAIR:AT2G18210.1); similar to hypo	1.891
AM060665	2.210	no similarity	
EV211246	2.210	moderately similar to (276)AT4G37340| Symbols: CYP81D3 | CYP81D3 (cytochrome P450, family 81, subfamily D, polypeptide 3); oxy	1.896
DN964921	2.209	weakly similar to (136)AT3G12320| Symbols: | similar to unknown protein [Arabidopsis thaliana] (TAIR:AT5G06980.1); similar to hypo	
JCVI_14581	2.208	no original description	
JCVI_40058	2.208	weakly similar to (192)AT5G20710| Symbols: BGAL7 | BGAL7 (beta-galactosidase 7); beta-galactosidase | chr5:7010538-7013996 FORW	
JCVI_20780	2.206	weakly similar to (169)AT2G31990| Symbols: | exostosin family protein | chr2:13618577-13620628 REVERSEvery weakly similar to (9	
EE533169	2.206	no similarity	
EX039688	2.206	very weakly similar to (95.9)AT4G38960| Symbols: | zinc finger (B-box type) family protein | chr4:18162294-18163039 FORWARD [21	1.538
JCVI_24307	2.203	moderately similar to (256)AT5G60530| Symbols: | late embryogenesis abundant protein-related / LEA protein-related | chr5:24351423-2	
JCVI_5154	2.203	very weakly similar to (82.8)AT5G64310| Symbols: ATAGP1, AGP1 | AGP1 (ARABINOGALACTAN-PROTEIN 1) | chr5:25739244-257	2.287
JCVI_20847	2.202	moderately similar to (215)AT3G12560| Symbols: ATTBP2, TRFL9 | TRFL9 (TRF-LIKE 9); DNA binding | chr3:3982279-3984855 REV	
BG543935	2.202	weakly similar to (144)AT1G61340| Symbols: | F-box family protein | chr1:22632191-22633406 FORWARD [8791]	2.245
CD835918	2.201	weakly similar to (196)AT3G08040| Symbols: MAN1, FRD3 | FRD3 (FERRIC REDUCTASE DEFECTIVE 3); antiporter | chr3:256659	
RC_ES960556	2.198	no similarity	
JCVI_40054	2.197	no original description	
EX086329	2.196	very weakly similar to (82.8)AT2G40085| Symbols: | unknown protein | chr2:16743612-16743970 REVERSE [21823] 1 611 623	
JCVI_20213	2.195	no original description	
JCVI_20305	2.194	moderately similar to (305)AT3G23210| Symbols: | basic helix-loop-helix (bHLH) family protein | chr3:8283262-8284922 REVERSE no	
EE455190	2.194	weakly similar to (118)AT4G15430| Symbols: | similar to early-responsive to dehydration protein-related / ERD protein-related [Arabido	
AM391952	2.194	moderately similar to (333)AT1G07520| Symbols: | scarecrow transcription factor family protein | chr1:2309715-2311802 REVERSEEver	
EE504270	2.192	no similarity	
JCVI_41123	2.192	moderately similar to (445)AT1G55920| Symbols: SAT5, SAT1, AtSerat2;1 | AtSerat2;1 (SERINE ACETYLTRANSFERASE 1) | chr1:2(	
EE549186	2.191	no similarity	
JCVI_19123	2.191	weakly similar to (113)AT3G10930| Symbols: | similar to unknown protein [Arabidopsis thaliana] (TAIR:AT5G05300.1) | chr3:342047	2.140
EE560233	2.190	no similarity	
JCVI_26483	2.189	weakly similar to (172)AT1G78600| Symbols: | zinc finger (B-box type) family protein | chr1:29572263-29573555 FORWARD no origin	1.580
H74879	2.188	no similarity	
RC_EX015383	2.187	no similarity	
ES906388	2.186	moderately similar to (336)AT5G66210| Symbols: CPK28 | CPK28 (calcium-dependent protein kinase 28) | chr5:26474371-26476660 RE	
EV048397	2.185	weakly similar to (127)AT4G20880| Symbols: | ethylene-responsive nuclear protein / ethylene-regulated nuclear protein (ERT2) | chr4:11	
JCVI_16093	2.185	moderately similar to (450)AT1G30370| Symbols: | lipase class 3 family protein | chr1:10719151-10720740 REVERSE no original descr	
JCVI_37273	2.184	moderately similar to (273)AT1G70740| Symbols: | protein kinase family protein | chr1:26677509-26679349 REVERSE no original desc	
JCVI_19289	2.184	weakly similar to (181)AT1G44414| Symbols: | similar to unnamed protein product [Vitis vinifera] (GB:CAO61874.1) | chr1:16850221-1	
AM385180	2.184	no similarity	
JCVI_20441	2.182	highly similar to (737)AT3G28740| Symbols: CYP81D1 | cytochrome P450 family protein | chr3:10790001-10791789 REVERSEmodera	2.224
EV027089	2.179	weakly similar to (187)AT4G09820| Symbols: TT8 | TT8 (TRANSPARENT TESTA 8); DNA binding / transcription factor | chr4:618206	
JCVI_30628	2.178	weakly similar to (194)AT1G42550| Symbols: PMI1 | PMI1 (PLASTID MOVEMENT IMPAIRED1) | chr1:15979976-15982174 FORWA	1.304
JCVI_370	2.178	no original description	2.261
EX064352	2.176	no similarity	
EV033740	2.174	no similarity	
EX094943	2.173	very weakly similar to (81.6)AT1G19770| Symbols: ATPUP14 | ATPUP14 (Arabidopsis thaliana purine permease 14); purine transmembr	
EX077836	2.172	moderately similar to (401)AT2G38010| Symbols: | ceramidase family protein | chr2:15913940-15916945 FORWARD [21818]	
EE443822	2.171	weakly similar to (129)AT1G61960| Symbols: | mitochondrial transcription termination factor-related / mTERF-related | chr1:22905904-2	
DY022380	2.170	no similarity	
JCVI_6399	2.169	moderately similar to (269)AT1G19770| Symbols: ATPUP14 | ATPUP14 (Arabidopsis thaliana purine permease 14); purine transmembra	

JCVI_18534	2.168	weakly similar to (178)AT2G32530| Symbols: CSLB03, ATCSLB3, ATCSLB03 | ATCSLB03 (Cellulose synthase-like B3); transferase/ t	1.973
JCVI_17506	2.168	highly similar to (699)AT4G18010| Symbols: IP5PII | IP5PII (INOSITOL POLYPHOSPHATE 5-PHOSPHATASE II); inositol-polyphosp	
JCVI_784	2.168	highly similar to (506)AT1G13470| Symbols: | similar to unknown protein [Arabidopsis thaliana] (TAIR:AT1G13520.1); similar to unna	
EV192533	2.168	weakly similar to (123)AT3G06670| Symbols: | binding | chr3:2105914-2113142 REVERSE [21489] 1 742 757	
EX068443	2.167	weakly similar to (160)AT1G22630| Symbols: | heat shock protein binding / unfolded protein binding | chr1:8003476-8004156 FORWAI	
JCVI_6434	2.167	weakly similar to (187)AT2G20825| Symbols: ULT2 | ULT2 (ULTRAPETALA 2); DNA binding | chr2:8972926-8973876 REVERSE no	
ES986055	2.166	no similarity	2.674
RC_EE557313	2.165	no similarity	
JCVI_31600	2.164	moderately similar to (213)AT2G41290| Symbols: | strictosidine synthase family protein | chr2:17217677-17219982 REVERSE no origi	2.180
H07702	2.164	no similarity	
EX027590	2.163	no similarity	1.513
EX125453	2.162	weakly similar to (187)AT3G61190| Symbols: BAP1 | BAP1 (BON ASSOCIATION PROTEIN 1) | chr3:22661812-22662390 REVERSE	
JCVI_22687	2.161	no original description	
ES901454	2.159	no similarity	
EV123347	2.159	weakly similar to (120)AT4G30780| Symbols: | similar to unknown protein [Arabidopsis thaliana] (TAIR:AT2G24100.1); similar to unna	
ES944059	2.158	no similarity	
JCVI_19049	2.158	no original description	2.441
JCVI_26486	2.157	moderately similar to (313)AT1G26800| Symbols: | zinc finger (C3HC4-type RING finger) family protein | chr1:9285563-9286177 REV	
JCVI_33539	2.156	weakly similar to (119)AT3G10300| Symbols: | similar to unknown protein [Arabidopsis thaliana] (TAIR:AT3G04300.1); similar to unk	2.178
JCVI_22350	2.155	moderately similar to (327)AT5G12900| Symbols: | similar to unknown protein [Arabidopsis thaliana] (TAIR:AT1G12330.1); similar to	
JCVI_5957	2.154	weakly similar to (101)AT3G59400| Symbols: GUN4 | GUN4 (Genomes uncoupled 4) | chr3:21959858-21960655 REVERSE no original	2.197
JCVI_26589	2.152	moderately similar to (295)AT5G58660| Symbols: | oxidoreductase, 2OG-Fe(II) oxygenase family protein | chr5:23718735-23721028 FC	
EV148639	2.150	no similarity	
JCVI_8766	2.150	no original description	
JCVI_23299	2.150	moderately similar to (364)AT1G69540| Symbols: AGL94 | AGL94; DNA binding / transcription factor | chr1:26148969-26150822 REVI	1.666
EV136519	2.148	no similarity	
JCVI_22381	2.144	very weakly similar to (95.1)AT1G71000| Symbols: | DNAJ heat shock N-terminal domain-containing protein | chr1:26772998-26773773	
JCVI_1991	2.142	moderately similar to (416)AT2G39650| Symbols: | similar to unknown protein [Arabidopsis thaliana] (TAIR:AT4G14620.1); similar to	
JCVI_5473	2.139	moderately similar to (231)AT5G51460| Symbols: ATTPPA | ATTPPA (Arabidopsis thaliana trehalose-6-phosphate phosphatase); trehalo	2.227
EV218294	2.138	moderately similar to (322)AT4G37610| Symbols: BT5 | BT5 (BTB and TAZ domain protein 5); protein binding / transcription regulator	3.609
ES981144	2.138	weakly similar to (125)AT3G52820| Symbols: ATPAP22, PAP22 | ATPAP22/PAP22 (purple acid phosphatase 22); acid phosphatase/ pro	
EE466210	2.137	weakly similar to (181)AT4G25480| Symbols: CBF3, DREB1, DREB1A | DREB1A (DEHYDRATION RESPONSE ELEMENT B1A); I	2.463
EV142318	2.137	no similarity	
CV544654	2.135	no similarity	
JCVI_14524	2.135	moderately similar to (243)AT3G11410| Symbols: ATPP2CA, AHG3 | AHG3/ATPP2CA (ARABIDOPSIS THALIANA PROTEIN PHOS	
EE475848	2.133	very weakly similar to (91.7)AT2G22440| Symbols: | similar to reverse transcriptase, putative / RNA-dependent DNA polymerase, putativ	
CD837184	2.131	weakly similar to (186)AT1G21150| Symbols: | mitochondrial transcription termination factor family protein / mTERF family protein | ch	
JCVI_15245	2.130	moderately similar to (244)AT1G26800| Symbols: | zinc finger (C3HC4-type RING finger) family protein | chr1:9285563-9286177 REV	
EE419793	2.129	weakly similar to (110)AT3G58690| Symbols: | protein kinase family protein | chr3:21720346-21722223 FORWARD [20149]	
JCVI_38510	2.129	very weakly similar to (83.2)AT1G59910| Symbols: | formin homology 2 domain-containing protein / FH2 domain-containing protein | ch	
AM061459	2.126	no similarity	2.153
JCVI_39207	2.125	moderately similar to (210)AT3G29130| Symbols: | similar to unknown [Populus trichocarpa] (GB:ABK93868.1); contains domain PTHI	
EV091988	2.124	moderately similar to (360)AT2G32530| Symbols: CSLB03, ATCSLB3, ATCSLB03 | ATCSLB03 (Cellulose synthase-like B3); transfera	
JCVI_10987	2.122	weakly similar to (173)AT4G21870| Symbols: | 26.5 kDa class P-related heat shock protein (HSP26.5-P) | chr4:11603768-11604297 REV	1.933
ES930133	2.121	very weakly similar to (100)AT4G20880| Symbols: | ethylene-responsive nuclear protein / ethylene-regulated nuclear protein (ERT2) | ch	
CV432094	2.118	no similarity	
EV110864	2.117	no similarity	
EE565545	2.116	very weakly similar to (84.7)AT5G44720| Symbols: | molybdenum cofactor sulfurase family protein | chr5:18060313-18062502 FORWA	
JCVI_27077	2.114	no original description	
CV546383	2.111	very weakly similar to (88.6)AT1G27730| Symbols: ZAT10, STZ | STZ (SALT TOLERANCE ZINC FINGER); nucleic acid binding / tra	2.495
JCVI_13044	2.110	moderately similar to (288)AT2G28200| Symbols: | nucleic acid binding / transcription factor/ zinc ion binding | chr2:12031398-1203225	
JCVI_17264	2.110	moderately similar to (345)AT1G10010| Symbols: AAP8 | AAP8 (amino acid permease 8); amino acid transmembrane transporter | chr1:3	
EV049297	2.110	moderately similar to (320)AT3G55450| Symbols: | protein kinase, putative | chr3:20569106-20570940 FORWARDweakly similar to (1	1.498
JCVI_36422	2.109	no original description	
JCVI_18855	2.109	moderately similar to (383)AT3G21670| Symbols: | nitrate transporter (NTP3) | chr3:7626949-7628961 REVERSE no original descriptio	1.331
JCVI_20774	2.109	highly similar to (560)AT3G53230| Symbols: | cell division cycle protein 48, putative / CDC48, putative | chr3:19734394-19737467 FOF	
EE469173	2.107	weakly similar to (188)AT2G01870| Symbols: | similar to unnamed protein product [Vitis vinifera] (GB:CAO21902.1) | chr2:389845-39(	2.059
JCVI_26083	2.107	very weakly similar to (94.7)AT4G27310| Symbols: | zinc finger (B-box type) family protein | chr4:13675859-13676622 FORWARD no	2.570
JCVI_41701	2.106	no original description	
JCVI_10924	2.105	highly similar to (522)AT1G56600| Symbols: ATGOLS2 | ATGOLS2 (ARABIDOPSIS THALIANA GALACTINOL SYNTHASE 2); tra	
EV008996	2.105	no similarity	
JCVI_7992	2.104	very weakly similar to (87.8)AT4G19390| Symbols: | similar to unknown protein [Arabidopsis thaliana] (TAIR:AT5G13720.1); similar to	
JCVI_34217	2.104	weakly similar to (150)AT5G05900| Symbols: | UDP-glucoronosyl/UDP-glucosyl transferase family protein | chr5:1774514-1776382 FO	
EV215868	2.104	no similarity	
EV076981	2.103	no similarity	1.335
RC_JCVI_40485	2.102	no original description	
JCVI_38611	2.100	no original description	1.555
JCVI_8271	2.099	no original description	
JCVI_26710	2.097	moderately similar to (281)AT5G39670| Symbols: | calcium-binding EF hand family protein | chr5:15900498-15901112 FORWARD no	
JCVI_32367	2.097	no original description	
EE450080	2.097	weakly similar to (156)AT1G44100| Symbols: AAP5 | AAP5 (amino acid permease 5); amino acid transmembrane transporter | chr1:1676	
RC_EE522201	2.095	no similarity	
JCVI_11679	2.095	moderately similar to (352)AT1G73480| Symbols: | hydrolase, alpha/beta fold family protein | chr1:27632927-27636147 FORWARD no	
JCVI_14292	2.093	weakly similar to (117)AT4G11660| Symbols: HSFB2B, AT-HSFB2B | AT-HSFB2B (Arabidopsis thaliana heat shock transcription facto	
EX120516	2.093	no similarity	
EH429196	2.093	moderately similar to (226)AT2G21860| Symbols: | violaxanthin de-epoxidase-related | chr2:9325413-9327070 REVERSE [20767]	
JCVI_16378	2.093	moderately similar to (248)AT1G14480| Symbols: | protein binding | chr1:4956399-4957883 FORWARD no original description	
EV136896	2.093	no similarity	
JCVI_34264	2.092	no original description	
JCVI_25211	2.092	weakly similar to (125)AT5G62770| Symbols: | similar to unknown protein [Arabidopsis thaliana] (TAIR:AT3G27880.1); similar to hypo	
JCVI_40260	2.091	weakly similar to (151)AT4G12970| Symbols: | similar to unnamed protein product [Vitis vinifera] (GB:CAO17947.1) | chr4:7586241-75	
JCVI_14484	2.090	moderately similar to (266)AT4G30280| Symbols: ATXTH18, XTH18 | ATXTH18/XTH18 (XYLOGLUCAN ENDOTRANSGLUCOSY	3.028
RC_EH424469	2.089	no similarity	

EE417055	2.086	no similarity	
EV143922	2.085	no similarity	
EE479654	2.085	no similarity	
JCVI_13766	2.085	moderately similar to (261)AT5G67300| Symbols: ATMYBR1, ATMYB44, MYBR1 | ATMYB44/ATMYBR1/MYBR1 (MYB DOMAIN	1.687
JCVI_30808	2.085	moderately similar to (324)AT2G16430| Symbols: PAP10, ATPAP10 | ATPAP10/PAP10; acid phosphatase/ protein serine/threonine phos	2.019
DY027899	2.083	no similarity	
JCVI_38636	2.083	no original description	1.497
ES907374	2.082	moderately similar to (375)AT4G01450| Symbols: | nodulin MtN21 family protein | chr4:608586-610127 FORWARD [21429]	
JCVI_39109	2.081	moderately similar to (201)AT5G23130| Symbols: | peptidoglycan-binding LysM domain-containing protein | chr5:7781478-7783336 FO	1.452
ES906142	2.081	moderately similar to (228)AT5G06790| Symbols: | similar to unknown protein [Arabidopsis thaliana] (TAIR:AT3G57950.1); similar to	
EX133779	2.081	weakly similar to (196)AT1G49405| Symbols: | integral membrane protein, putative | chr1:18288003-18288615 REVERSE [21833] 1 68	
EV054296	2.080	weakly similar to (103)AT3G09740| Symbols: ATSYP71, SYP71 | SYP71 (SYNTAXIN OF PLANTS 71) | chr3:2989620-2991359 FOR	
JCVI_10530	2.080	moderately similar to (308)AT1G21670| Symbols: | similar to unknown protein [Arabidopsis thaliana] (TAIR:AT1G21680.1); similar to	2.177
JCVI_26872	2.078	moderately similar to (241)AT1G66160| Symbols: | U-box domain-containing protein | chr1:24640881-24642176 FORWARD no origina	2.128
JCVI_29680	2.078	moderately similar to (222)AT2G32530| Symbols: CSLB03, ATCSLB3, ATCSLB03 | ATCSLB03 (Cellulose synthase-like B3); transfera	1.320
ES945473	2.078	very weakly similar to (85.9)AT4G29900| Symbols: ATACA10, ACA10 | ACA10 (autoinhibited Ca2+ -ATPase 10); calcium-transporting	
JCVI_20208	2.077	moderately similar to (211)AT1G15260| Symbols: | similar to unknown protein [Arabidopsis thaliana] (TAIR:AT3G16070.1) | chr1:5249	1.275
JCVI_28406	2.076	weakly similar to (186)AT5G05220| Symbols: | similar to hypothetical protein [Vitis vinifera] (GB:CAN82940.1) | chr5:1550274-155082	
JCVI_12923	2.076	very weakly similar to (80.9)AT3G23170| Symbols: | similar to unknown protein [Arabidopsis thaliana] (TAIR:AT4G14450.1) | chr3:826	
JCVI_19551	2.076	weakly similar to (191)AT1G44414| Symbols: | similar to unnamed protein product [Vitis vinifera] (GB:CAO61874.1) | chr1:16850221-1	
JCVI_10486	2.074	weakly similar to (169)AT4G10300| Symbols: | similar to unknown protein [Arabidopsis thaliana] (TAIR:AT3G04300.1); similar to unkn	2.230
JCVI_19373	2.074	no original description	3.150
JCVI_37800	2.073	no original description	
ES919101	2.070	no similarity	
JCVI_11538	2.068	moderately similar to (224)AT4G20380| Symbols: LSD1 | LSD1 (LESION SIMULATING DISEASE) | chr4:11005023-11006449 FORW	
EV180758	2.067	weakly similar to (144)AT1G21890| Symbols: | nodulin MtN21 family protein | chr1:7682797-7685570 REVERSE [21487] 16 547 739	
EX100333	2.066	moderately similar to (360)AT1G07520| Symbols: | scarecrow transcription factor family protein | chr1:2309715-2311802 REVERSEwea	
JCVI_40192	2.066	weakly similar to (140)AT5G48990| Symbols: | kelch repeat-containing F-box family protein | chr5:19879566-19880684 FORWARD no	1.388
EV124296	2.065	no similarity	
JCVI_8448	2.065	moderately similar to (300)AT5G52420| Symbols: | similar to unknown protein [Arabidopsis thaliana] (TAIR:AT5G23920.1); similar to	
JCVI_19719	2.063	moderately similar to (299)AT3G28740| Symbols: CYP81D1 | cytochrome P450 family protein | chr3:10790001-10791789 REVERSEwe	2.183
EV165943	2.062	weakly similar to (146)AT2G32020| Symbols: | GCN5-related N-acetyltransferase (GNAT) family protein | chr2:13638270-13638821 RI	
EE501966	2.061	weakly similar to (117)AT5G56670| Symbols: | 40S ribosomal protein S30 (RPS30C) | chr5:22952639-22953207 REVERSE [20193] 35	
JCVI_19433	2.060	no original description	
EH429199	2.059	moderately similar to (304)AT2G21860| Symbols: | violaxanthin de-epoxidase-related | chr2:9325413-9327070 REVERSE [20767]	1.356
AM388297	2.058	weakly similar to (181)AT1G66500| Symbols: | zinc finger (C2H2-type) family protein | chr1:24814925-24816175 REVERSE [20118]	
EE490643	2.057	moderately similar to (225)AT5G05580| Symbols: SH1, FAD8 | FAD8 (FATTY ACID DESATURASE 8); omega-3 fatty acid desaturase	2.461
JCVI_16910	2.055	no original description	
EV197013	2.055	moderately similar to (204)AT4G19450| Symbols: | nodulin-related | chr4:10606549-10609229 FORWARD [21490]	1.634
EV055334	2.054	no similarity	
BQ792023	2.053	moderately similar to (214)AT3G28340| Symbols: GATL10 | GATL10 (Galacturonosyltransferase-like 10); polygalacturonate 4-alpha-gal	2.534
EX064182	2.053	very weakly similar to (83.2)AT4G13670| Symbols: PTAC5 | PTAC5 (PLASTID TRANSCRIPTIONALLY ACTIVE5); heat shock protei	1.754
JCVI_33411	2.052	moderately similar to (379)AT2G03550| Symbols: | hydrolase | chr2:1077077-1078015 FORWARDweakly similar to (115)GID1_ORYS	
JCVI_23724	2.052	moderately similar to (402)AT1G30860| Symbols: | protein binding / zinc ion binding | chr1:10986677-10989227 REVERSE no original	
JCVI_40415	2.052	moderately similar to (496)AT4G36870| Symbols: SAW1, BLH2 | BLH2 (BEL1-LIKE HOMEODOMAIN 2, SAWTOOTH 1); DNA binc	
JCVI_18054	2.052	weakly similar to (190)AT4G23570| Symbols: SGT1A | SGT1A (Suppressor of G2 (Two) 1A) | chr4:12300025-12302503 FORWARD nc	
JCVI_14672	2.049	no original description	
ES994226	2.049	weakly similar to (131)AT2G01180| Symbols: PAP1, LPP1, ATLPP1, ATPAP1 | ATPAP1 (PHOSPHATIDIC ACID PHOSPHATASE 1)	1.520
EE409683	2.048	no similarity	
JCVI_29302	2.048	moderately similar to (219)AT1G04380| Symbols: | 2-oxoglutarate-dependent dioxygenase, putative | chr1:1177141-1178383 REVERSE	
EV036222	2.047	weakly similar to (145)AT4G37480| Symbols: | DNAJ heat shock N-terminal domain-containing protein | chr4:17619255-17621396 FOR	
JCVI_15889	2.047	moderately similar to (306)AT1G65690| Symbols: | harpin-induced protein-related / HIN1-related / harpin-responsive protein-related | ch	
EV115465	2.046	moderately similar to (233)AT1G30370| Symbols: | lipase class 3 family protein | chr1:10719151-10720740 REVERSE [21479]	
JCVI_26544	2.045	moderately similar to (343)AT1G74650| Symbols: AtY13, AtMYB31 | AtMYB31/AtY13 (myb domain protein 31); DNA binding / transc	
EX078964	2.045	no similarity	
DY019163	2.043	moderately similar to (270)AT1G14480| Symbols: | protein binding | chr1:4956399-4957883 FORWARD [18966]	
JCVI_36695	2.041	moderately similar to (234)AT5G66310| Symbols: | kinesin motor family protein | chr5:26503012-26507530 REVERSE no original desc	
DN962793	2.040	weakly similar to (128)AT2G40000| Symbols: HSPRO2, ATHSPRO2 | similar to unknown protein [Arabidopsis thaliana] (TAIR:AT3G5	
JCVI_20460	2.040	very weakly similar to (96.3)AT3G57450| Symbols: | similar to unnamed protein product [Vitis vinifera] (GB:CAO40798.1) | chr3:21273	
EE457288	2.039	no similarity	1.669
EX036296	2.039	very weakly similar to (95.5)AT4G13690| Symbols: | similar to hypothetical protein MtrDRAFT_AC161864g11v2 [Medicago truncatula]	
EV135969	2.038	weakly similar to (191)AT1G18330| Symbols: EPR1 | EPR1 (EARLY-PHYTOCHROME-RESPONSIVE1) | chr1:6306189-6307711 REV	1.832
JCVI_41843	2.036	no original description	
EE540603	2.036	weakly similar to (137)AT4G37320| Symbols: CYP81D5 | CYP81D5 (cytochrome P450, family 81, subfamily D, polypeptide 5); oxygen	
JCVI_27566	2.035	moderately similar to (390)AT3G28960| Symbols: | amino acid transporter family protein | chr3:10985482-10987004 REVERSE no origi	
JCVI_30619	2.035	no original description	
JCVI_4805	2.035	moderately similar to (216)AT1G71000| Symbols: | DNAJ heat shock N-terminal domain-containing protein | chr1:26772998-26773773	
JCVI_32919	2.035	no original description	
ES962556	2.033	weakly similar to (104)AT2G20562| Symbols: | similar to unknown protein [Arabidopsis thaliana] (TAIR:AT2G31090.1); similar to unna	
AM394555	2.031	weakly similar to (143)AT2G31800| Symbols: | ankyrin protein kinase, putative | chr2:13527682-13530723 REVERSE [20346]	
JCVI_36638	2.030	no original description	
JCVI_7390	2.029	weakly similar to (194)AT5G24120| Symbols: SIG5, SIGE | SIGE (RNA polymerase sigma subunit E); DNA binding / DNA-directed RN	
RC_ES948251	2.028	no similarity	
JCVI_34303	2.027	weakly similar to (103)AT5G48820| Symbols: KRP3, ICK6 | ICK6/KRP3 (KIP-RELATED PROTEIN 3); cyclin binding / cyclin-depende	
JCVI_14969	2.027	highly similar to (853)AT3G61880| Symbols: CYP78A9 | CYP78A9 (CYTOCHROME P450 78A9); oxygen binding | chr3:22917089-22	1.584
RC_EV010845	2.026	no similarity	
JCVI_11608	2.026	moderately similar to (347)AT5G43730| Symbols: | disease resistance protein (CC-NBS-LRR class), putative | chr5:17577494-17580040	
JCVI_3621	2.025	highly similar to (593)AT4G26530| Symbols: | fructose-bisphosphate aldolase, putative | chr4:13391573-13392944 FORWARDhighly si	
JCVI_28980	2.025	weakly similar to (168)AT2G40080| Symbols: ELF4 | ELF4 (EARLY FLOWERING 4) | chr2:16741623-16741958 REVERSE no origina	
ES938920	2.023	no similarity	
EV041837	2.022	weakly similar to (144)AT5G17400| Symbols: ER-ANT1 | ADP, ATP carrier protein, mitochondrial, putative / ADP/ATP translocase, put	
EV194156	2.022	very weakly similar to (100)AT2G46830| Symbols: CCA1 | CCA1 (CIRCADIAN CLOCK ASSOCIATED 1); transcription factor | chr2:1	
EV068998	2.022	very weakly similar to (92.4)AT1G70740| Symbols: | protein kinase family protein | chr1:26677509-26679349 REVERSE [21443]	

EV088843	2.021	weakly similar to (124)AT1G70780| Symbols: | similar to unknown protein [Arabidopsis thaliana] (TAIR:AT1G23150.1); similar to unna	
EV107694	2.021	no similarity	1.890
JCVI_27977	2.019	moderately similar to (384)AT5G23130| Symbols: | peptidoglycan-binding LysM domain-containing protein | chr5:7781478-7783336 FO	1.466
JCVI_18453	2.019	moderately similar to (258)AT1G17620| Symbols: | similar to unknown protein [Arabidopsis thaliana] (TAIR:AT5G11890.1); similar to u	2.112
AT002133	2.019	no similarity	1.654
JCVI_21467	2.018	weakly similar to (190)AT3G14075| Symbols: | lipase class 3 family protein | chr3:4663826-4666345 REVERSE no original description	1.560
EL588022	2.016	no similarity	2.173
JCVI_41245	2.016	no original description	
EV123214	2.016	no similarity	
EV226929	2.015	no similarity	
JCVI_16337	2.013	weakly similar to (136)AT1G74370| Symbols: | zinc finger (C3HC4-type RING finger) family protein | chr1:27961861-27962646 REVE	
EE567498	2.013	no similarity	1.305
EX062935	2.012	no similarity	1.732
EV100364	2.010	moderately similar to (285)AT3G15354| Symbols: SPA3 | SPA3 (SPA1-RELATED 3); signal transducer | chr3:5169334-5172487 REVEI	
JCVI_18772	2.010	moderately similar to (499)AT5G11650| Symbols: | hydrolase, alpha/beta fold family protein | chr5:3745070-3746817 FORWARD no or	
JCVI_1443	2.009	moderately similar to (351)AT1G72030| Symbols: | GCN5-related N-acetyltransferase (GNAT) family protein | chr1:27114549-2711548	
EE508362	2.009	weakly similar to (102)AT2G39940| Symbols: COI1 | COI1 (CORONATINE INSENSITIVE 1); ubiquitin-protein ligase | chr2:16679926-	
CV544883	2.009	very weakly similar to (90.1)AT2G15480| Symbols: UGT73B5 | UGT73B5 (UDP-GLUCOSYL TRANSFERASE 73B5); UDP-glycosyltra	
JCVI_804	2.007	weakly similar to (135)AT1G28370| Symbols: ERF11, ATERF11 | ATERF11/ERF11 (ERF domain protein 11); DNA binding / transcript	
EE546123	2.005	very weakly similar to (93.2)AT3G45640| Symbols: MPK3, ATMPK3 | ATMPK3 (MITOGEN-ACTIVATED PROTEIN KINASE 3); MA	
AM391473	2.003	weakly similar to (103)AT1G25400| Symbols: | similar to unknown protein [Arabidopsis thaliana] (TAIR:AT1G68440.1); similar to unna	
EV205399	2.003	no similarity	
JCVI_28482	2.002	moderately similar to (433)AT1G49780| Symbols: | U-box domain-containing protein | chr1:18432692-18433957 REVERSE no original	
EV121574	2.001	very weakly similar to (100)AT2G26530| Symbols: AR781 | AR781 | chr2:11290114-11291067 REVERSE [21479]	1.847
JCVI_25449	2.000	moderately similar to (374)AT2G39920| Symbols: | acid phosphatase class B family protein | chr2:16670279-16671383 REVERSE no or	
JCVI_23523	2.000	moderately similar to (229)AT3G24190| Symbols: | ABC1 family protein | chr3:8743326-8747710 FORWARD no original description	
EE472897	1.998	weakly similar to (102)AT2G37460| Symbols: | nodulin MtN21 family protein | chr2:15733745-15736088 REVERSE [20163]	
JCVI_27098	1.996	no original description	
JCVI_14196	1.995	weakly similar to (112)AT2G17300| Symbols: | similar to unknown protein [Arabidopsis thaliana] (TAIR:AT4G35320.1) | chr2:7529547	2.112
ES931762	1.993	moderately similar to (283)AT3G04890| Symbols: | similar to unknown protein [Arabidopsis thaliana] (TAIR:AT2G46100.1); similar to u	1.829
EV177438	1.991	weakly similar to (132)AT4G15800| Symbols: RALFL33 | RALFL33 (RALF-LIKE 33) | chr4:8984923-8985273 FORWARD [21487] 1 1	1.978
AT000573	1.991	very weakly similar to (91.3)AT1G62300| Symbols: WRKY6 | WRKY6 (WRKY DNA-binding protein 6); transcription factor | chr1:2302	
JCVI_35371	1.989	moderately similar to (274)AT1G69900| Symbols: | similar to unknown protein [Arabidopsis thaliana] (TAIR:AT1G27100.1); similar to l	
JCVI_2940	1.989	moderately similar to (251)AT1G01250| Symbols: | AP2 domain-containing transcription factor, putative | chr1:104731-105309 REVERS	
JCVI_25076	1.988	weakly similar to (105)AT1G69490| Symbols: ANAC029, ATNAP, NAP | NAP (NAC-LIKE, ACTIVATED BY AP3/PI); transcription fa	
ES945435	1.987	weakly similar to (160)AT1G44100| Symbols: AAP5 | AAP5 (amino acid permease 5); amino acid transmembrane transporter | chr1:1676	
EV163061	1.987	weakly similar to (118)AT5G15770| Symbols: ATGNA1 | ATGNA1 (ARABIDOPSIS THALIANA GLUCOSE-6-PHOSPHATE ACETY	
JCVI_2292	1.987	no original description	
AM388881	1.987	moderately similar to (246)AT1G14780| Symbols: | similar to unknown protein [Arabidopsis thaliana] (TAIR:AT4G24290.2); similar to u	
JCVI_34173	1.986	moderately similar to (278)AT1G73480| Symbols: | hydrolase, alpha/beta fold family protein | chr1:27632927-27636147 FORWARD no	
JCVI_1247	1.986	weakly similar to (126)AT4G10340| Symbols: LHCB5 | LHCB5 (LIGHT HARVESTING COMPLEX OF PHOTOSYSTEM II 5); chloro	2.003
JCVI_13314	1.986	highly similar to (554)AT4G37610| Symbols: BT5 | BT5 (BTB and TAZ domain protein 5); protein binding / transcription regulator | chr4	3.479
AM056987	1.984	no similarity	
JCVI_6146	1.983	moderately similar to (319)AT4G23990| Symbols: CSLG3, ATCSLG3 | ATCSLG3 (Cellulose synthase-like G3); transferase/ transferase,	2.153
JCVI_30499	1.983	moderately similar to (366)AT2G17040| Symbols: ANAC036 | ANAC036 (Arabidopsis NAC domain containing protein 36); transcriptio	
DY026607	1.981	weakly similar to (190)AT3G08760| Symbols: ATSIK | ATSIK; kinase | chr3:2658135-2659990 REVERSE [18978]	
JCVI_38471	1.981	moderately similar to (263)AT3G05410| Symbols: | similar to hypothetical protein OsI_004967 [Oryza sativa (indica cultivar-group)] (G	1.611
EX105012	1.981	moderately similar to (297)AT3G05410| Symbols: | similar to hypothetical protein OsI_004967 [Oryza sativa (indica cultivar-group)] (G	1.662
JCVI_11039	1.980	moderately similar to (263)AT5G17230| Symbols: PSY | PSY (PHYTOENE SYNTHASE) | chr5:5659841-5662089 REVERSEweakly si	1.361
JCVI_28230	1.979	weakly similar to (134)AT2G47440| Symbols: | DNAJ heat shock N-terminal domain-containing protein | chr2:19476982-19478730 FOR	
JCVI_20526	1.979	weakly similar to (149)AT5G62200| Symbols: | embryo-specific protein-related | chr5:25001689-25002900 REVERSE no original descri	2.173
JCVI_22168	1.977	weakly similar to (101)AT1G67195| Symbols: MIR414 | MIR414 | chr1:25141041-25141307 REVERSE no original description	1.836
JCVI_19667	1.976	very weakly similar to (88.2)AT2G03830| Symbols: | unknown protein | chr2:1171223-1172144 FORWARD no original description	1.911
JCVI_8338	1.976	no original description	1.583
JCVI_4089	1.976	weakly similar to (120)AT3G27210| Symbols: | Identical to Uncharacterized protein At3g27210 (Y-2) [Arabidopsis Thaliana] (GB:Q9LK	
EX114202	1.976	moderately similar to (342)AT5G01540| Symbols: | lectin protein kinase, putative | chr5:211284-213332 REVERSEweakly similar to (1	
JCVI_22128	1.975	moderately similar to (383)AT4G35190| Symbols: | similar to unknown protein [Arabidopsis thaliana] (TAIR:AT2G37210.1); similar to u	
EV191421	1.974	weakly similar to (129)AT1G51480| Symbols: | disease resistance protein (CC-NBS-LRR class), putative | chr1:19094515-19097974 RE	
ES969031	1.973	no similarity	
EV076037	1.973	no similarity	
EV105899	1.973	no similarity	
JCVI_39178	1.970	moderately similar to (330)AT1G10460| Symbols: GLP7 | GLP7 (GERMIN-LIKE PROTEIN 7); manganese ion binding / metal ion bindi	
ES957548	1.969	no similarity	1.231
CD831313	1.969	weakly similar to (175)AT3G52740| Symbols: | similar to unknown protein [Arabidopsis thaliana] (TAIR:AT3G44450.1); similar to unna	2.105
EV108697	1.968	no similarity	
JCVI_18991	1.968	weakly similar to (101)AT1G67195| Symbols: MIR414 | MIR414 | chr1:25141041-25141307 REVERSE no original description	1.936
JCVI_2215	1.968	highly similar to (663)AT2G39800| Symbols: ATP5CS, P5CS1 | P5CS1 (DELTA1-PYRROLINE-5-CARBOXYLATE SYNTHASE 1) | c	1.485
EV153292	1.968	moderately similar to (271)AT2G42890| Symbols: AML2 | AML2 | chr2:17857709-17861282 FORWARD [21484] 61 1033 1033	
JCVI_20788	1.967	no original description	
JCVI_20267	1.967	weakly similar to (114)AT3G14395| Symbols: | unknown protein | chr3:4811051-4811278 FORWARD no original description	
EV185924	1.967	moderately similar to (343)AT1G27480| Symbols: | lecithin:cholesterol acyltransferase family protein / LACT family protein | chr1:9544	1.426
EE567412	1.967	no similarity	
EE561149	1.967	no similarity	
JCVI_36644	1.967	highly similar to (538)AT3G14850| Symbols: | similar to unknown protein [Arabidopsis thaliana] (TAIR:AT1G29050.1); similar to unna	
JCVI_33485	1.967	moderately similar to (258)AT1G26750| Symbols: | similar to unnamed protein product [Vitis vinifera] (GB:CAO68449.1); similar to hyp	
EV141196	1.966	moderately similar to (271)AT3G14950| Symbols: TTL2 | TTL2 (TETRATRICOPETIDE-REPEAT THIOREDOXIN-LIKE 2); binding |	
JCVI_17343	1.966	moderately similar to (275)AT1G60190| Symbols: | armadillo/beta-catenin repeat family protein / U-box domain-containing protein | chr1	
ES979962	1.965	no similarity	
JCVI_16357	1.964	no original description	
JCVI_40181	1.963	weakly similar to (171)AT5G42610| Symbols: | similar to unknown protein [Arabidopsis thaliana] (TAIR:AT2G23790.1); similar to hypo	
EV199111	1.961	moderately similar to (329)AT5G59580| Symbols: | UDP-glucoronosyl/UDP-glucosyl transferase family protein | chr5:24023465-240249	1.682
EE531069	1.961	weakly similar to (163)AT2G18460| Symbols: LCV3 | LCV3 (LIKE COV 3) | chr2:8008720-8009936 FORWARD [20175]	
ES949482	1.960	no similarity	

JCVI_21975	1.960	no original description	2.114
JCVI_33709	1.959	moderately similar to (229)AT2G29750| Symbols: | UDP-glucoronosyl/UDP-glucosyl transferase family protein | chr2:12716979-127184	1.856
EE496340	1.959	no similarity	
JCVI_10897	1.959	highly similar to (539)AT5G46800| Symbols: BOU | BOU (A BOUT DE SOUFFLE); binding | chr5:19006006-19007037 REVERSEvery	
JCVI_5926	1.958	moderately similar to (426)AT2G41290| Symbols: | strictosidine synthase family protein | chr2:17217677-17219982 REVERSEweakly si	2.276
EE405584	1.957	weakly similar to (115)AT5G44310| Symbols: | late embryogenesis abundant domain-containing protein / LEA domain-containing protei	
ES955034	1.955	weakly similar to (187)AT4G28703| Symbols: | similar to unknown protein [Arabidopsis thaliana] (TAIR:AT3G04300.1); similar to unna	1.415
EX133918	1.954	no similarity	
JCVI_263	1.954	moderately similar to (460)AT5G58770| Symbols: | dehydrodolichyl diphosphate synthase, putative / DEDOL-PP synthase, putative | chr	2.416
JCVI_3676	1.953	moderately similar to (274)AT3G22840| Symbols: ELIP, ELIP1 | ELIP1 (EARLY LIGHT-INDUCABLE PROTEIN); chlorophyll binding	
RC_ES968318	1.953	no similarity	
JCVI_18986	1.952	weakly similar to (192)AT3G16570| Symbols: RALFL23 | RALFL23 (RALF-LIKE 23) | chr3:5644754-5645170 FORWARD no original	
EV189797	1.951	moderately similar to (219)AT5G37260| Symbols: RVE2, CIR1 | CIR1/RVE2 (CIRCADIAN 1); DNA binding / transcription factor | chr5	2.320
EL587715	1.951	moderately similar to (384)AT2G26150| Symbols: HSFA2, ATHSFA2 | ATHSFA2 (Arabidopsis thaliana heat shock transcription factor A	
JCVI_37195	1.948	weakly similar to (131)AT5G18370| Symbols: | disease resistance protein (TIR-NBS-LRR class), putative | chr5:6085038-6088928 REVI	
EV189169	1.947	no similarity	
EV098127	1.946	no similarity	
JCVI_31983	1.946	no original description	1.483
JCVI_10332	1.945	weakly similar to (162)AT4G11390| Symbols: | DC1 domain-containing protein | chr4:6927887-6930373 FORWARD no original descrip	
EV201538	1.945	weakly similar to (186)AT1G01060| Symbols: LHY1, LHY | LHY (LATE ELONGATED HYPOCOTYL) | chr1:33992-37061 REVERSE	
DY026197	1.944	no similarity	
JCVI_8096	1.944	moderately similar to (488)AT1G48260| Symbols: SnRK3.21, CIPK17 | CIPK17 (SNF1-RELATED PROTEIN KINASE 3.21); kinase | cl	
EE473052	1.944	no similarity	
EV225634	1.943	weakly similar to (104)AT2G23810| Symbols: TET8 | TET8 (TETRASPANIN8) | chr2:10142939-10144432 REVERSE [21493] 69 740 7	1.454
ES907954	1.942	no similarity	
CV545835	1.942	no similarity	
JCVI_38191	1.942	no original description	
JCVI_30458	1.942	no original description	2.163
EV135810	1.940	weakly similar to (130)AT1G46768| Symbols: RAP2.1 | RAP2.1 (related to AP2 1); DNA binding / transcription factor | chr1:17268486-1	
CV544813	1.940	no similarity	
JCVI_8749	1.939	no original description	
EV038543	1.939	no similarity	
EX107105	1.939	very weakly similar to (98.2)AT4G01090| Symbols: | extra-large G-protein-related | chr4:470834-473248 REVERSE [21827]	
JCVI_26904	1.938	moderately similar to (301)AT3G61320| Symbols: | Identical to UPF0187 protein At3g61320, chloroplast precursor [Arabidopsis Thalian	1.528
EX096733	1.937	no similarity	
EV050576	1.936	weakly similar to (140)AT3G13340| Symbols: | WD-40 repeat family protein | chr3:4332377-4334610 FORWARD [21442]	1.863
RC_EX042891	1.936	no similarity	
EV107773	1.936	no similarity	1.601
JCVI_9043	1.935	moderately similar to (314)AT3G27540| Symbols: | glycosyl transferase family 17 protein | chr3:10206726-10208125 FORWARD no ori	
EV112327	1.934	no similarity	1.782
JCVI_27151	1.934	weakly similar to (187)AT5G59350| Symbols: | similar to unnamed protein product [Vitis vinifera] (GB:CAO21684.1) | chr5:23958358-2	1.902
EE412990	1.933	weakly similar to (175)AT4G21060| Symbols: | galactosyltransferase family protein | chr4:11240741-11244871 FORWARD [20145] 1 5	
JCVI_16826	1.933	moderately similar to (256)AT5G52870| Symbols: | similar to unknown protein [Arabidopsis thaliana] (TAIR:AT1G64080.1); similar to u	
EX051964	1.929	no similarity	
JCVI_41544	1.928	moderately similar to (452)AT5G61900| Symbols: CPN1, BON, BON1 | BON1 (BONZAI1); calcium-dependent phospholipid binding | c	
JCVI_23547	1.928	highly similar to (525)AT5G43630| Symbols: | zinc knuckle (CCHC-type) family protein | chr5:17544488-17547434 FORWARD no orig	
JCVI_28502	1.928	weakly similar to (125)AT1G80610| Symbols: | similar to unknown protein [Arabidopsis thaliana] (TAIR:AT1G15800.1); similar to hypo	
AM058972	1.927	weakly similar to (111)AT5G66630| Symbols: | LIM domain-containing protein | chr5:26609946-26612917 FORWARD [17712]	
EX043867	1.927	moderately similar to (316)AT5G62430| Symbols: CDF1 | CDF1 (CYCLING DOF FACTOR 1); DNA binding / protein binding / transcri	1.507
JCVI_39669	1.925	weakly similar to (112)AT1G22470| Symbols: | similar to unknown protein [Arabidopsis thaliana] (TAIR:AT1G72240.1) | chr1:7932897	
EX050276	1.925	moderately similar to (341)AT5G41890| Symbols: | GDSL-motif lipase/hydrolase family protein | chr5:16781520-16784128 REVERSE [	
EV218517	1.925	moderately similar to (248)AT4G37610| Symbols: BT5 | BT5 (BTB and TAZ domain protein 5); protein binding / transcription regulator	3.308
JCVI_14498	1.925	moderately similar to (498)AT4G28740| Symbols: | similar to LPA1 (LOW PSII ACCUMULATION1), binding [Arabidopsis thaliana] (T	
RC_EV086676	1.925	no similarity	
EE555777	1.924	weakly similar to (177)AT3G15990| Symbols: SULTR3;4 | SULTR3;4; sulfate transmembrane transporter | chr3:5427087-5430685 FORV	2.073
JCVI_36110	1.924	highly similar to (587)AT2G15880| Symbols: | leucine-rich repeat family protein / extensin family protein | chr2:6925121-6927401 REVI	
JCVI_19785	1.922	moderately similar to (276)AT5G43630| Symbols: | zinc knuckle (CCHC-type) family protein | chr5:17544488-17547434 FORWARD no	
EE462190	1.919	weakly similar to (176)AT1G75100| Symbols: JAC1 | JAC1 (J-DOMAIN PROTEIN REQUIRED FOR CHLOROPLAST ACCUMULAT	
JCVI_17512	1.918	moderately similar to (328)AT3G20050| Symbols: ATTCP-1 | ATTCP-1 (Arabidopsis thaliana T-complex protein 1 alpha subunit); ATP b	
JCVI_22790	1.918	very weakly similar to (88.2)AT1G60060| Symbols: | similar to unknown protein [Arabidopsis thaliana] (TAIR:AT5G53900.2); similar to	
EV199230	1.918	moderately similar to (248)AT4G18880| Symbols: HSFA4A, AT-HSFA4A | AT-HSFA4A (Arabidopsis thaliana heat shock transcription f	
AM389200	1.918	very weakly similar to (85.5)AT3G50810| Symbols: | integral membrane protein, putative | chr3:18898595-18900849 REVERSE [20118]	
JCVI_18514	1.916	weakly similar to (131)AT3G57450| Symbols: | similar to unnamed protein product [Vitis vinifera] (GB:CAO40798.1) | chr3:21273005-2	1.762
JCVI_38889	1.916	moderately similar to (264)AT1G17620| Symbols: | similar to unknown protein [Arabidopsis thaliana] (TAIR:AT5G11890.1); similar to u	2.118
JCVI_24738	1.916	highly similar to (965)AT5G08570| Symbols: | pyruvate kinase, putative | chr5:2778434-2780301 FORWARDhighly similar to (887)KP	
EX110829	1.915	weakly similar to (180)AT4G04830| Symbols: | methionine sulfoxide reductase domain-containing protein / SeIR domain-containing prot	
JCVI_15186	1.915	no original description	
CD833252	1.914	weakly similar to (115)AT4G30830| Symbols: | similar to unknown protein [Arabidopsis thaliana] (TAIR:AT2G24140.1); similar to unna	
EX016523	1.913	no similarity	
JCVI_35079	1.913	moderately similar to (278)AT3G54390| Symbols: | transcription factor | chr3:20148890-20149841 REVERSE no original description	
JCVI_30317	1.912	very weakly similar to (94.0)AT1G57820| Symbols: VIM1, ORTH2 | ORTH2/VIM1 (VARIANT IN METHYLATION 1); DNA binding |	
EV194015	1.910	very weakly similar to (88.6)AT1G69295| Symbols: | beta-1,3-glucanase-related | chr1:26054155-26055506 REVERSE [21489]	1.877
JCVI_7521	1.910	moderately similar to (261)AT4G01000| Symbols: | ubiquitin family protein | chr4:432186-433727 REVERSE no original description	
JCVI_34187	1.910	moderately similar to (357)AT2G34650| Symbols: ABR, PID | PID (PINOID); kinase | chr2:14597013-14598636 REVERSEweakly simil	
JCVI_207	1.909	moderately similar to (464)AT1G69490| Symbols: ANAC029, ATNAP, NAP | NAP (NAC-LIKE, ACTIVATED BY AP3/PI); transcriptio	
JCVI_23006	1.908	moderately similar to (271)AT5G19330| Symbols: | armadillo/beta-catenin repeat family protein / BTB/POZ domain-containing protein |	
JCVI_27007	1.907	moderately similar to (283)AT4G24660| Symbols: ATHB22, MEE68 | ATHB22/MEE68 (ARABIDOPSIS THALIANA HOMEOBOX PF	
ES966044	1.907	no similarity	
EE446682	1.907	no similarity	
EV085501	1.907	no similarity	
EV157418	1.906	moderately similar to (208)AT4G26850| Symbols: VTC2 | VTC2 (VITAMIN C DEFECTIVE 2) | chr4:13499268-13501151 REVERSE [	
JCVI_1705	1.904	moderately similar to (418)AT2G41250| Symbols: | haloacid dehalogenase-like hydrolase family protein | chr2:17207940-17209629 REV	1.371
JCVI_33161	1.903	weakly similar to (187)AT4G23530| Symbols: | similar to unknown protein [Arabidopsis thaliana] (TAIR:AT4G11300.1); similar to hypo	

ES961855	1.903	no similarity	
JCVI_21019	1.902	weakly similar to (159)AT1G11650| Symbols: ATRBP45B | ATRBP45B; RNA binding | chr1:3914895-3917301 FORWARD no original	
JCVI_36439	1.900	no original description	
EE456342	1.900	no similarity	
AM391559	1.898	no similarity	1.699
JCVI_33439	1.898	no original description	1.765
JCVI_15615	1.897	highly similar to (546)AT4G15430| Symbols: | similar to early-responsive to dehydration protein-related / ERD protein-related [Arabidop	1.694
JCVI_38650	1.897	very weakly similar to (84.7)AT5G62200| Symbols: | embryo-specific protein-related | chr5:25001689-25002900 REVERSE no original d	1.962
JCVI_34540	1.896	moderately similar to (244)AT4G28270| Symbols: | zinc finger (C3HC4-type RING finger) family protein | chr4:14007620-14008201 RE	
JCVI_12100	1.896	no original description	
AM388362	1.896	moderately similar to (263)AT1G13520| Symbols: | similar to unknown protein [Arabidopsis thaliana] (TAIR:AT1G13480.1); similar to u	
JCVI_24968	1.895	no original description	
ES950203	1.893	no similarity	1.851
JCVI_37535	1.892	weakly similar to (171)AT4G40020| Symbols: | similar to unknown protein [Arabidopsis thaliana] (TAIR:AT5G16730.1); similar to hypo	
JCVI_11196	1.891	weakly similar to (189)AT5G56710| Symbols: | 60S ribosomal protein L31 (RPL31C) | chr5:22961229-22961993 REVERSEweakly simi	2.449
EV168475	1.891	weakly similar to (120)AT2G46810| Symbols: | basic helix-loop-helix (bHLH) family protein | chr2:19246763-19249442 FORWARD [2	1.886
JCVI_28339	1.890	weakly similar to (154)AT4G28025| Symbols: | similar to unnamed protein product [Vitis vinifera] (GB:CAO66511.1); contains domain	2.040
EE514308	1.889	weakly similar to (194)AT5G09820| Symbols: | plastid-lipid associated protein PAP / fibrillin family protein | chr5:3056091-3057381 RE	
EV031987	1.888	moderately similar to (216)AT5G05190| Symbols: | Identical to Uncharacterized protein At5g05190 (Y-1) [Arabidopsis Thaliana] (GB:Q	
JCVI_20591	1.888	weakly similar to (133)AT5G57340| Symbols: | similar to hypothetical protein MtrDRAFT_AC155282g59v2 [Medicago truncatula] (GB	
JCVI_24343	1.887	moderately similar to (375)AT2G41835| Symbols: | zinc finger (C2H2 type, AN1-like) family protein | chr2:17465098-17466492 REVEI	
EV019429	1.884	no similarity	
JCVI_15416	1.883	very weakly similar to (92.0)AT4G16141| Symbols: | similar to zinc finger (GATA type) family protein [Arabidopsis thaliana] (TAIR:AT	
JCVI_33509	1.882	moderately similar to (322)AT1G48600| Symbols: | phosphoethanolamine N-methyltransferase 2, putative (NMT2) | chr1:17970116-1797	2.229
EX126783	1.880	highly similar to (503)AT1G63440| Symbols: HMA5 | HMA5 (HEAVY METAL ATPASE 5); ATPase, coupled to transmembrane mover	
JCVI_42293	1.880	no original description	
JCVI_8143	1.878	moderately similar to (209)AT1G72640| Symbols: | binding / catalytic | chr1:27350071-27351809 REVERSE no original description	
ES968415	1.878	no similarity	
JCVI_22085	1.877	weakly similar to (162)AT1G62250| Symbols: | similar to unnamed protein product [Vitis vinifera] (GB:CAO21221.1) | chr1:22999280-2	
EV170934	1.877	no similarity	
JCVI_654	1.877	moderately similar to (357)AT5G64840| Symbols: ATGCN5 | ATGCN5 (Arabidopsis thaliana general control non-repressible 5) | chr5:25	
JCVI_3170	1.876	no original description	
JCVI_27216	1.874	moderately similar to (309)AT2G26150| Symbols: HSFA2, ATHSFA2 | ATHSFA2 (Arabidopsis thaliana heat shock transcription factor A	
JCVI_23230	1.873	moderately similar to (253)AT1G50450| Symbols: | binding / catalytic | chr1:18691570-18694016 REVERSE no original description	1.963
JCVI_38623	1.873	highly similar to (556)AT3G55840| Symbols: | similar to unknown protein [Arabidopsis thaliana] (TAIR:AT2G40000.1); similar to unna	2.674
JCVI_33804	1.873	weakly similar to (129)AT3G61890| Symbols: ATHB12, ATHB-12 | ATHB-12 (ARABIDOPSIS THALIANA HOMEOBOX PROTEIN 1	
AM387827	1.872	weakly similar to (198)AT1G07520| Symbols: | scarecrow transcription factor family protein | chr1:2309715-2311802 REVERSE [20118	
JCVI_3138	1.872	moderately similar to (391)AT4G26850| Symbols: VTC2 | VTC2 (VITAMIN C DEFECTIVE 2) | chr4:13499268-13501151 REVERSE n	
EE564016	1.871	no similarity	
EX121234	1.870	very weakly similar to (94.7)AT5G05190| Symbols: | Identical to Uncharacterized protein At5g05190 (Y-1) [Arabidopsis Thaliana] (GB:(	
JCVI_29126	1.870	highly similar to (521)AT1G78510| Symbols: SPS1 | SPS1 (SOLANESYL DIPHOSPHATE SYNTHASE 1) | chr1:29540303-29541935 R	
JCVI_33053	1.870	no original description	1.518
JCVI_28035	1.868	weakly similar to (103)AT1G64490| Symbols: | similar to unknown protein [Arabidopsis thaliana] (TAIR:AT5G42060.1); contains doma	
JCVI_34203	1.868	moderately similar to (208)AT4G28025| Symbols: | similar to unnamed protein product [Vitis vinifera] (GB:CAO66511.1); contains dom	1.956
JCVI_41016	1.868	moderately similar to (241)AT1G63440| Symbols: HMA5 | HMA5 (HEAVY METAL ATPASE 5); ATPase, coupled to transmembrane m	
JCVI_22361	1.868	highly similar to (727)AT3G43600| Symbols: AAO2 | AAO2 (ALDEHYDE OXIDASE 2) | chr3:15523766-15528363 REVERSEhighly s	
DY028827	1.867	no similarity	
DN961498	1.866	weakly similar to (122)AT2G38170| Symbols: ATCAX1, RCI4, CAX1 | CAX1 (CATION EXCHANGER 1); calcium:hydrogen antiporte	
JCVI_16081	1.865	moderately similar to (207)AT1G77920| Symbols: | bZIP family transcription factor | chr1:29303853-29305501 FORWARD no original d	
EX093366	1.865	weakly similar to (149)AT1G07050| Symbols: | CONSTANS-like protein-related | chr1:2164326-2165132 REVERSE [21823]	2.502
EV064718	1.865	no similarity	
EV199927	1.863	weakly similar to (189)AT3G02380| Symbols: COL2 | COL2 (CONSTANS-LIKE 2); transcription factor/ zinc ion binding | chr3:487445-	2.496
JCVI_18620	1.862	no original description	1.654
EV140039	1.862	no similarity	
EE514837	1.862	no similarity	1.644
DY025326	1.861	moderately similar to (221)AT1G23030| Symbols: | armadillo/beta-catenin repeat family protein / U-box domain-containing protein | chr1	
JCVI_2320	1.860	moderately similar to (370)AT1G07450| Symbols: | tropinone reductase, putative / tropine dehydrogenase, putative | chr1:2288035-22892	
JCVI_17705	1.859	moderately similar to (474)AT4G12300| Symbols: CYP706A4 | CYP706A4 (cytochrome P450, family 706, subfamily A, polypeptide 4);	
ES942971	1.858	moderately similar to (213)AT5G52050| Symbols: | MATE efflux protein-related | chr5:21156159-21157676 FORWARD [21392]	2.034
EV117032	1.858	no similarity	1.238
JCVI_443	1.857	no original description	
JCVI_20777	1.857	weakly similar to (180)AT3G28940| Symbols: | avirulence-responsive protein, putative / avirulence induced gene (AIG) protein, putative	1.576
JCVI_30270	1.857	moderately similar to (365)AT3G52380| Symbols: PDE322, CP33 | CP33 (PIGMENT DEFECTIVE 322); RNA binding | chr3:19432597-	
EV203422	1.857	very weakly similar to (91.3)AT2G40400| Symbols: | similar to unknown protein [Arabidopsis thaliana] (TAIR:AT3G56140.1); similar to	2.125
EE483891	1.856	no similarity	1.478
JCVI_9783	1.855	highly similar to (633)AT5G04360| Symbols: ATPU1, ATLDA | ATLDA/ATPU1 (PULLULANASE 1); alpha-amylase/ limit dextrinase |	
EE409598	1.855	no similarity	
JCVI_33907	1.854	moderately similar to (417)AT5G65090| Symbols: MRH3, BST1, DER4 | BST1/DER4/MRH3 (BRISTLED1) | chr5:26022063-26023882	
RC_AM394013	1.853	no similarity	
JCVI_23811	1.853	no original description	
JCVI_7188	1.853	weakly similar to (164)AT2G46830| Symbols: CCA1 | CCA1 (CIRCADIAN CLOCK ASSOCIATED 1); transcription factor | chr2:19253	
JCVI_37905	1.853	no original description	
JCVI_29069	1.852	no original description	
DY023666	1.852	moderately similar to (207)AT1G61250| Symbols: SC3 | SC3 (SECRETORY CARRIER 3); transmembrane transporter | chr1:22589700-2	
EV027589	1.852	weakly similar to (157)AT5G62140| Symbols: | similar to unknown [Populus trichocarpa] (GB:ABK94834.1) | chr5:24971789-24972602	
JCVI_5451	1.851	moderately similar to (455)AT3G52060| Symbols: | similar to unknown protein [Arabidopsis thaliana] (TAIR:AT5G22070.1); similar to u	
JCVI_22078	1.850	weakly similar to (126)AT5G23210| Symbols: SCPL34 | SCPL34 | chr5:7811628-7814611 FORWARD no original description	2.571
JCVI_41488	1.850	no original description	
EV128075	1.850	no similarity	
EV122995	1.849	no similarity	
JCVI_27857	1.849	weakly similar to (162)AT1G33475| Symbols: | Identical to Probable VAMP-like protein At1g33475 [Arabidopsis Thaliana] (GB:Q84WI	
JCVI_16414	1.849	moderately similar to (437)AT1G80840| Symbols: ATWRKY40, WRKY40 | WRKY40 (WRKY DNA-binding protein 40); transcription f	
DY011268	1.848	weakly similar to (152)AT5G58320| Symbols: | kinase interacting protein-related | chr5:23595320-23596757 FORWARD [18980]	

JCVI_2663	1.848	moderately similar to (339)AT2G23580| Symbols: | hydrolase, alpha/beta fold family protein | chr2:10040440-10041390 REVERSEweak	1.575
EV092858	1.847	weakly similar to (175)AT2G15580| Symbols: | zinc finger (C3HC4-type RING finger) family protein | chr2:6804769-6805897 FORWA	1.581
ES937867	1.847	no similarity	
EH427320	1.845	moderately similar to (362)AT3G61320| Symbols: | Identical to UPF0187 protein At3g61320, chloroplast precursor [Arabidopsis Thalian	
CB686392	1.845	no similarity	
JCVI_30176	1.844	moderately similar to (246)AT5G17400| Symbols: ER-ANT1 | ADP, ATP carrier protein, mitochondrial, putative / ADP/ATP translocase	1.566
JCVI_38419	1.843	moderately similar to (469)AT2G03550| Symbols: | hydrolase | chr2:1077077-1078015 FORWARDweakly similar to (125)GID1_ORYS	
JCVI_5431	1.842	moderately similar to (449)AT4G37610| Symbols: BT5 | BT5 (BTB and TAZ domain protein 5); protein binding / transcription regulator	3.306
ES930275	1.842	weakly similar to (139)AT2G47300| Symbols: | ribonuclease P | chr2:19426403-19429146 FORWARD [20185]	
H07560	1.842	no similarity	
DY008536	1.842	weakly similar to (114)AT4G25980| Symbols: | cationic peroxidase, putative | chr4:13189402-13191516 FORWARD [18972]	
AM386883	1.841	weakly similar to (191)AT1G76600| Symbols: | similar to unknown protein [Arabidopsis thaliana] (TAIR:AT1G21010.1); similar to hypo	2.212
EV187751	1.840	no similarity	
DY005554	1.840	weakly similar to (118)AT3G17600| Symbols: IAA31 | IAA31 (indoleacetic acid-induced protein 31); transcription factor | chr3:6020287-	
EE412814	1.840	no similarity	
EV108800	1.840	no similarity	
JCVI_26215	1.839	moderately similar to (408)AT2G47180| Symbols: ATGOLS1 | ATGOLS1 (ARABIDOPSIS THALIANA GALACTINOL SYNTHASE 1)	
JCVI_35331	1.839	no original description	
JCVI_16334	1.838	no original description	
ES904772	1.838	moderately similar to (408)AT4G37260| Symbols: MYB73, AtMYB73 | AtMYB73/MYB73 (myb domain protein 73); DNA binding / tra	
EE568029	1.837	no similarity	
JCVI_32436	1.837	moderately similar to (400)AT5G62430| Symbols: CDF1 | CDF1 (CYCLING DOF FACTOR 1); DNA binding / protein binding / transcri	
EX043963	1.836	moderately similar to (341)AT4G04320| Symbols: | malonyl-CoA decarboxylase family protein | chr4:2113563-2116523 FORWARD [2	
CN829082	1.836	weakly similar to (102)AT2G41835| Symbols: | zinc finger (C2H2 type, AN1-like) family protein | chr2:17465098-17466492 REVERSE	
JCVI_36159	1.835	moderately similar to (250)AT5G39420| Symbols: CDC2CAT | CDC2CAT (ARABIDOPSIS THALIANA CDC2C); kinase | chr5:157894	
EV221069	1.835	weakly similar to (195)AT3G05200| Symbols: ATL6 | ATL6 (Arabidopsis T?xicos en Levadura 6); protein binding / zinc ion binding | ch	
JCVI_20797	1.834	moderately similar to (310)AT5G06320| Symbols: NHL3 | NHL3 (NDR1/HIN1-like 3) | chr5:1931017-1931712 REVERSE no original de	
EV128361	1.834	no similarity	
JCVI_4708	1.832	moderately similar to (335)AT3G51430| Symbols: YLS2 | YLS2 (yellow-leaf-specific gene 2); strictosidine synthase | chr3:19097527-190	
EX063012	1.832	no similarity	
JCVI_330	1.831	highly similar to (524)AT1G59725| Symbols: | DNAJ heat shock protein, putative | chr1:21954403-21955875 FORWARDweakly similar	1.802
ES922465	1.829	no similarity	
EV012124	1.829	no similarity	
EV102958	1.828	no similarity	
EV106031	1.827	weakly similar to (124)AT5G65300| Symbols: | unknown protein | chr5:26112492-26112944 REVERSE [21478] 39 606 735	
EX119983	1.827	no similarity	
EV164211	1.827	no similarity	
JCVI_37850	1.826	moderately similar to (429)AT4G25160| Symbols: | protein kinase family protein | chr4:12903370-12906679 REVERSEweakly similar to	
EV191999	1.826	moderately similar to (344)AT1G53090| Symbols: SPA4 | SPA4 (SPA1-RELATED 4); signal transducer | chr1:19787416-19790358 FOR	1.244
JCVI_13799	1.825	moderately similar to (243)AT5G05280| Symbols: | zinc finger (C3HC4-type RING finger) family protein | chr5:1565510-1566040 REV	
CD812196	1.824	no similarity	
JCVI_18896	1.824	moderately similar to (242)AT3G12860| Symbols: | nucleolar protein Nop56, putative | chr3:4091685-4093928 FORWARD no original d	
JCVI_28223	1.824	no original description	
EV161880	1.824	weakly similar to (138)AT2G40010| Symbols: | 60S acidic ribosomal protein P0 (RPP0A) | chr2:16715656-16717526 REVERSEweakly	
JCVI_17687	1.823	moderately similar to (227)AT3G62410| Symbols: CP12, CP12-2 | CP12-2 | chr3:23101982-23102377 FORWARD no original descriptio	
JCVI_3095	1.822	weakly similar to (186)AT3G54810| Symbols: BME3-ZF, BME3 | BME3/BME3-ZF (BLUE MICROPYLAR END3); transcription factor	2.170
JCVI_12784	1.822	weakly similar to (117)AT1G29690| Symbols: CAD1 | CAD1 (CONSTITUTIVELY ACTIVATED CELL DEATH 1) | chr1:10379296-10	
ES912977	1.820	moderately similar to (358)AT1G71530| Symbols: | protein kinase family protein | chr1:26943428-26945241 FORWARD [21430]	
JCVI_7443	1.819	moderately similar to (223)AT4G30270| Symbols: MERI-5, SEN4, MERI5B | MERI5B (MERISTEM-5); hydrolase, acting on glycosyl bo	
EV191697	1.819	weakly similar to (141)AT5G41100| Symbols: | DNA binding | chr5:16464657-16467914 FORWARD [21489]	
CN731264	1.815	moderately similar to (261)AT2G26150| Symbols: HSFA2, ATHSFA2 | ATHSFA2 (Arabidopsis thaliana heat shock transcription factor A	
JCVI_4553	1.814	moderately similar to (319)AT4G15550| Symbols: IAGLU | IAGLU (INDOLE-3-ACETATE BETA-D-GLUCOSYLTRANSFERASE); U	
DY015383	1.814	no similarity	1.475
JCVI_29181	1.813	weakly similar to (120)AT4G21740| Symbols: | unknown protein | chr4:11545938-11546408 FORWARD no original description	
JCVI_20427	1.813	very weakly similar to (87.4)AT1G33055| Symbols: | unknown protein | chr1:11972316-11972516 REVERSE no original description	
EL591285	1.811	moderately similar to (368)AT1G07540| Symbols: TRFL2 | TRFL2 (TRF-LIKE 2); DNA binding | chr1:2318430-2321045 REVERSE [20	
EV218940	1.810	no similarity	
JCVI_41262	1.809	no original description	
JCVI_16958	1.809	weakly similar to (195)AT1G61667| Symbols: | similar to unknown protein [Arabidopsis thaliana] (TAIR:AT5G54530.1); similar to unna	
EV106465	1.809	no similarity	
JCVI_22326	1.809	moderately similar to (347)AT1G44100| Symbols: AAP5 | AAP5 (amino acid permease 5); amino acid transmembrane transporter | chr1:1	
JCVI_39829	1.808	weakly similar to (140)AT3G15115| Symbols: | similar to unknown protein [Arabidopsis thaliana] (TAIR:AT1G53180.1) | chr3:5086226	
JCVI_28144	1.808	weakly similar to (185)AT1G52000| Symbols: | jacalin lectin family protein | chr1:19337021-19339369 REVERSE no original descriptio	
JCVI_5485	1.807	weakly similar to (172)AT3G16870| Symbols: | zinc finger (GATA type) family protein | chr3:5763758-5764582 REVERSE no original d	
EE524164	1.807	weakly similar to (104)AT4G18880| Symbols: HSFA4A, AT-HSFA4A | AT-HSFA4A (Arabidopsis thaliana heat shock transcription facto	
JCVI_30304	1.807	moderately similar to (350)AT5G06530| Symbols: | ABC transporter family protein | chr5:1990335-1994606 REVERSE no original desc	1.273
JCVI_41730	1.805	weakly similar to (130)AT5G48820| Symbols: KRP3, ICK6 | ICK6/KRP3 (KIP-RELATED PROTEIN 3); cyclin binding / cyclin-depende	1.230
JCVI_9800	1.802	moderately similar to (216)AT5G48655| Symbols: | zinc finger (C3HC4-type RING finger) family protein | chr5:19748801-19749712 RE	
JCVI_3082	1.801	no original description	
RC_JCVI_10503	1.801	no original description	
JCVI_18360	1.801	no original description	
JCVI_481	1.800	moderately similar to (250)AT4G32340| Symbols: | binding | chr4:15612747-15614225 REVERSE no original description	
JCVI_3716	1.800	moderately similar to (259)AT2G27100| Symbols: SE | SE (SERRATE); transcription factor | chr2:11579665-11583435 FORWARD no o	
ES941361	1.800	very weakly similar to (89.0)AT3G22970| Symbols: | similar to unknown protein [Arabidopsis thaliana] (TAIR:AT4G14620.1); similar to	
BQ791333	1.798	no similarity	2.173
JCVI_28525	1.796	moderately similar to (302)AT3G22850| Symbols: | similar to unknown protein [Arabidopsis thaliana] (TAIR:AT5G43830.1); similar to	
EV089082	1.795	moderately similar to (334)AT4G22790| Symbols: | MATE efflux family protein | chr4:11975164-11976639 REVERSE [21444]	
EV027628	1.795	moderately similar to (325)AT5G24430| Symbols: | calcium-dependent protein kinase, putative / CDPK, putative | chr5:8339393-834291	1.476
JCVI_39382	1.791	no original description	
JCVI_13401	1.790	moderately similar to (296)AT5G37740| Symbols: | C2 domain-containing protein | chr5:15009378-15010661 FORWARD no original de	
JCVI_39801	1.789	no original description	
JCVI_4356	1.788	moderately similar to (467)AT3G24190| Symbols: | ABC1 family protein | chr3:8743326-8747710 FORWARD no original description	
EV194073	1.787	weakly similar to (150)AT2G46830| Symbols: CCA1 | CCA1 (CIRCADIAN CLOCK ASSOCIATED 1); transcription factor | chr2:19253	1.332

EV123018	1.786	weakly similar to (132)AT2G03120| Symbols: | signal peptide peptidase family protein | chr2:937551-940080 FORWARD [21479]	
EV192295	1.785	no similarity	
RC_ES216592	1.784	no similarity	
CN725800	1.784	weakly similar to (126)AT1G16770| Symbols: | similar to unnamed protein product [Vitis vinifera] (GB:CAO41707.1) | chr1:5738128-57	
EE417839	1.783	weakly similar to (104)AT1G28360| Symbols: ERF12, ATERF12 | ATERF12/ERF12 (ERF domain protein 12); DNA binding / transcript	
AM392202	1.783	weakly similar to (105)AT1G25275| Symbols: | similar to unnamed protein product [Vitis vinifera] (GB:CAO43403.1) | chr1:8860706-88	
EV121615	1.782	no similarity	1.917
JCVI_161	1.781	very weakly similar to (93.2)AT3G12630| Symbols: | zinc finger (AN1-like) family protein | chr3:4012714-4013196 FORWARD no origi	1.321
EE552556	1.781	no similarity	
JCVI_28271	1.780	weakly similar to (172)AT3G50800| Symbols: | similar to unknown protein [Arabidopsis thaliana] (TAIR:AT5G66580.1); similar to unna	
EL590833	1.780	moderately similar to (224)AT1G04290| Symbols: | thioesterase family protein | chr1:1147720-1148351 REVERSE [20863]	
EX123200	1.780	moderately similar to (477)AT5G04360| Symbols: ATPU1, ATLDA | ATLDA/ATPU1 (PULLULANASE 1); alpha-amylase/ limit dextrin	
EV216677	1.780	weakly similar to (172)AT1G22770| Symbols: FB, GI | GI (GIGANTEA); binding | chr1:8062387-8067436 FORWARDweakly similar to	
CD817929	1.778	very weakly similar to (98.2)AT1G67195| Symbols: MIR414 | MIR414 | chr1:25141041-25141307 REVERSE [13978]	
JCVI_30050	1.778	moderately similar to (408)AT3G46130| Symbols: AtMYB48, MYB111 | MYB111 (myb domain protein 111) | chr3:16956450-16957361	
EV009199	1.778	very weakly similar to (99.8)AT5G60580| Symbols: | zinc finger (C3HC4-type RING finger) family protein | chr5:24371524-24373932 F	
EV205511	1.777	moderately similar to (286)AT3G27170| Symbols: ATCLC-B, CLC-B | CLC-B (chloride channel protein B); anion channel/ voltage-gatec	1.612
JCVI_40243	1.777	moderately similar to (340)AT2G18940| Symbols: | pentatricopeptide (PPR) repeat-containing protein | chr2:8210955-8213423 REVERS	
CD813571	1.777	moderately similar to (274)AT3G62260| Symbols: | protein phosphatase 2C, putative / PP2C, putative | chr3:23049491-23051366 REVE	
JCVI_24149	1.777	moderately similar to (310)AT3G24120| Symbols: | myb family transcription factor | chr3:8705932-8708155 REVERSE no original desc	1.501
JCVI_39719	1.777	moderately similar to (369)AT1G17690| Symbols: | similar to unnamed protein product [Vitis vinifera] (GB:CAO64063.1); contains Inte	
EX116646	1.775	moderately similar to (255)AT1G51940| Symbols: | protein kinase family protein / peptidoglycan-binding LysM domain-containing prote	
EV148845	1.775	weakly similar to (177)AT4G27595| Symbols: | protein transport protein-related | chr4:13772825-13776524 REVERSE [21483]	
JCVI_25290	1.775	weakly similar to (169)AT1G77090| Symbols: | thylakoid lumenal 29.8 kDa protein | chr1:28965470-28966769 REVERSE no original de	
JCVI_28307	1.775	weakly similar to (190)AT1G46768| Symbols: RAP2.1 | RAP2.1 (related to AP2 1); DNA binding / transcription factor | chr1:17268486-1	
EX124723	1.774	moderately similar to (270)AT1G14780| Symbols: | similar to unknown protein [Arabidopsis thaliana] (TAIR:AT4G24290.2); similar to u	
JCVI_29738	1.774	weakly similar to (182)AT4G16141| Symbols: | similar to zinc finger (GATA type) family protein [Arabidopsis thaliana] (TAIR:AT3G1	
JCVI_41470	1.774	moderately similar to (254)AT4G12680| Symbols: | similar to unknown protein [Arabidopsis thaliana] (TAIR:AT5G40640.1); similar to u	
JCVI_9412	1.773	highly similar to (551)AT4G14130| Symbols: XTR7 | XTR7 (XYLOGLUCAN ENDOTRANSGLYCOSYLASE 7); hydrolase, acting on	
JCVI_5794	1.773	moderately similar to (230)AT2G34720| Symbols: | CCAAT-binding transcription factor (CBF-B/NF-YA) family protein | chr2:1465709	
JCVI_36434	1.773	weakly similar to (119)AT5G50920| Symbols: ATHSP93-V, HSP93-V, CLPC, DCA1, CLPC1 | CLPC (HEAT SHOCK PROTEIN 93-V)	1.449
JCVI_13951	1.772	moderately similar to (306)AT5G44120| Symbols: ATCRA1, CRU1, CRA1 | CRA1 (CRUCIFERINA); nutrient reservoir | chr5:17773687	
JCVI_42056	1.770	weakly similar to (173)AT5G14880| Symbols: | potassium transporter, putative | chr5:4814247-4817670 FORWARDweakly similar to (	1.831
AT000965	1.769	no similarity	
EV096676	1.768	weakly similar to (104)AT1G69526| Symbols: | UbiE/COQ5 methyltransferase family protein | chr1:26135156-26136597 FORWARD [2	
EV015756	1.768	no similarity	
JCVI_20312	1.768	no original description	1.285
EV200593	1.768	weakly similar to (160)AT4G04850| Symbols: ATKEA3, KEA3 | KEA3 (K+ efflux antiporter 3); potassium:hydrogen antiporter | chr4:24	
AM394479	1.765	no similarity	1.571
EE554939	1.765	weakly similar to (165)AT3G04600| Symbols: | tRNA synthetase class I (W and Y) family protein | chr3:1243158-1245964 FORWARD	
JCVI_27935	1.764	moderately similar to (500)AT1G21450| Symbols: SCL1 | SCL1 (SCARECROW-LIKE 1); transcription factor | chr1:7509710-7511491 F	
CD816426	1.764	moderately similar to (236)AT3G14075| Symbols: | lipase class 3 family protein | chr3:4663826-4666345 REVERSE [13977]	
CX193407	1.763	no similarity	
JCVI_32636	1.761	highly similar to (589)AT5G62570| Symbols: | calmodulin-binding protein | chr5:25132214-25134193 FORWARD no original descriptio	
JCVI_15575	1.760	moderately similar to (369)AT1G22770| Symbols: FB, GI | GI (GIGANTEA); binding | chr1:8062387-8067436 FORWARDmoderately si	
JCVI_19462	1.760	weakly similar to (186)AT2G42530| Symbols: COR15B | COR15B | chr2:17716269-17716951 REVERSE no original description	
JCVI_35610	1.760	no original description	1.695
JCVI_36574	1.760	weakly similar to (177)AT1G50450| Symbols: | binding / catalytic | chr1:18691570-18694016 REVERSE no original description	1.721
JCVI_21676	1.759	highly similar to (676)AT5G19160| Symbols: | similar to unknown protein [Arabidopsis thaliana] (TAIR:AT3G06080.2); similar to unna	
AM059307	1.759	no similarity	
EE462092	1.759	no similarity	
CO750244	1.758	no similarity	
ES993037	1.756	very weakly similar to (94.7)AT1G33475| Symbols: | Identical to Probable VAMP-like protein At1g33475 [Arabidopsis Thaliana] (GB:Q	
JCVI_24713	1.756	weakly similar to (155)AT5G61380| Symbols: APRR1, PRR1, TOC1 | TOC1 (TIMING OF CAB EXPRESSION 1); transcription regulato	1.827
EV084988	1.754	no similarity	
EE403215	1.754	very weakly similar to (84.7)AT1G74370| Symbols: | zinc finger (C3HC4-type RING finger) family protein | chr1:27961861-27962646 R	
JCVI_29471	1.752	no original description	
JCVI_209	1.751	moderately similar to (366)AT3G56090| Symbols: ATFER3 | ATFER3 (FERRITIN 3); ferric iron binding | chr3:20825329-20826963 RE	
CO750168	1.751	no similarity	
JCVI_26327	1.751	no original description	
RC_ES966207	1.750	no similarity	1.573
DY021448	1.750	moderately similar to (416)AT5G14580| Symbols: | polyribonucleotide nucleotidyltransferase, putative | chr5:4697615-4703016 REVER	
ES969134	1.750	very weakly similar to (84.7)AT1G34010| Symbols: | similar to unknown protein [Arabidopsis thaliana] (TAIR:AT1G22790.2); similar to	1.855
JCVI_18933	1.749	weakly similar to (112)AT4G36550| Symbols: | binding / ubiquitin-protein ligase | chr4:17245403-17247721 REVERSE no original desc	
EV011579	1.749	no similarity	
ES955408	1.749	no similarity	
EE470530	1.749	moderately similar to (326)AT1G32640| Symbols: RD22BP1, JAI1, JIN1, MYC2, ZBF1, ATMYC2 | ATMYC2 (JASMONATE INSENS	
JCVI_37180	1.748	moderately similar to (224)AT1G75700| Symbols: HVA22G | HVA22G (HVA22-LIKE PROTEIN G) | chr1:28427737-28428592 FORW	
JCVI_21279	1.748	weakly similar to (128)AT1G73390| Symbols: | similar to unknown protein [Arabidopsis thaliana] (TAIR:AT1G17940.1); similar to unna	
EV176836	1.748	no similarity	
JCVI_27161	1.746	moderately similar to (209)AT4G27660| Symbols: | similar to RIN13 (RPM1 INTERACTING PROTEIN 13) [Arabidopsis thaliana] (TA	
JCVI_9	1.746	moderately similar to (411)AT2G31380| Symbols: STH | STH (salt tolerance homologue); transcription factor/ zinc ion binding | chr2:133	
JCVI_34904	1.746	weakly similar to (153)AT2G32620| Symbols: CSLB02, ATCSLB2, ATCSLB02 | ATCSLB02 (Cellulose synthase-like B2); transferase/ t	1.576
JCVI_18352	1.746	moderately similar to (422)AT5G66675| Symbols: | Identical to UPF0496 protein At5g66675 [Arabidopsis Thaliana] (GB:Q8GW16); sin	
JCVI_27874	1.744	moderately similar to (204)AT1G76070| Symbols: | Identical to Uncharacterized protein At1g76070 [Arabidopsis Thaliana] (GB:Q9SGS	1.487
EV031999	1.741	moderately similar to (354)AT5G01110| Symbols: | pentatricopeptide (PPR) repeat-containing protein | chr5:42113-44302 REVERSEwe	
JCVI_16079	1.740	highly similar to (651)AT5G64570| Symbols: ATBXL4, XYL4 | XYL4 (beta-xylosidase 4); hydrolase, hydrolyzing O-glycosyl compound	1.621
EX022680	1.740	weakly similar to (157)AT5G46510| Symbols: | disease resistance protein (TIR-NBS-LRR class), putative | chr5:18877678-18882437 FC	
JCVI_31680	1.740	no original description	
JCVI_36229	1.740	highly similar to (524)AT4G31390| Symbols: | ABC1 family protein | chr4:15233132-15236770 FORWARD no original description	
JCVI_29537	1.740	very weakly similar to (87.0)AT4G35090| Symbols: CAT2 | CAT2 (CATALASE 2); catalase | chr4:16701110-16703220 REVERSEvery v	
EE441525	1.740	weakly similar to (142)AT1G18570| Symbols: AtMYB51, BW51A, BW51B, MYB51 | MYB51 (MYB DOMAIN PROTEIN 51); DNA b	
EE479477	1.739	no similarity	2.103

JCVI_38469	1.739	no original description	
EV132933	1.736	no similarity	1.596
EV004763	1.736	no similarity	1.169
JCVI_24769	1.735	highly similar to (640)AT5G15950| Symbols: | adenosylmethionine decarboxylase family protein | chr5:5206709-5207797 FORWARDhi	
EV196161	1.734	moderately similar to (255)AT5G03555| Symbols: | permease, cytosine/purines, uracil, thiamine, allantoin family protein | chr5:898353-9	
JCVI_37170	1.734	moderately similar to (333)AT3G52080| Symbols: CHX28 | CHX28 (cation/hydrogen exchanger 28); monovalent cation:proton antiporte	
JCVI_42417	1.734	no original description	
JCVI_41570	1.733	moderately similar to (233)AT1G79040| Symbols: PSBR | PSBR (photosystem II subunit R) | chr1:29740978-29741674 FORWARDmod	
JCVI_980	1.732	moderately similar to (273)AT4G26850| Symbols: VTC2 | VTC2 (VITAMIN C DEFECTIVE 2) | chr4:13499268-13501151 REVERSE n	1.374
JCVI_15122	1.732	moderately similar to (212)AT3G12345| Symbols: | similar to Os06g0484500 [Oryza sativa (japonica cultivar-group)] (GB:NP_001057	1.804
JCVI_1201	1.731	moderately similar to (312)AT1G55210| Symbols: | disease resistance response | chr1:20601724-20602287 REVERSE no original descri	2.099
EV094783	1.730	moderately similar to (317)AT5G59750| Symbols: | riboflavin biosynthesis protein, putative | chr5:24090623-24092637 FORWARD [21	2.056
JCVI_30695	1.729	no original description	
CD835898	1.729	no similarity	
JCVI_7717	1.728	moderately similar to (206)AT1G78995| Symbols: | similar to unnamed protein product [Vitis vinifera] (GB:CAO41778.1) | chr1:297203	1.842
JCVI_26720	1.728	moderately similar to (281)AT2G37970| Symbols: SOUL-1 | SOUL-1; binding | chr2:15898105-15898782 FORWARD no original descri	
EV213654	1.727	no similarity	
JCVI_2083	1.726	moderately similar to (352)AT1G21460| Symbols: | nodulin MtN3 family protein | chr1:7512019-7513270 REVERSE no original descrip	2.354
EE553984	1.724	no similarity	2.437
JCVI_21128	1.724	no original description	3.025
JCVI_14346	1.723	moderately similar to (444)AT2G29630| Symbols: | thiamine biosynthesis family protein / thiC family protein | chr2:12674472-1267664	
JCVI_26965	1.722	highly similar to (851)AT2G41190| Symbols: | amino acid transporter family protein | chr2:17174639-17177223 REVERSE no original d	
EV109508	1.722	moderately similar to (306)AT2G37970| Symbols: SOUL-1 | SOUL-1; binding | chr2:15898105-15898782 FORWARD [21478]	
AM395389	1.721	no similarity	
EV091804	1.721	no similarity	
JCVI_29075	1.721	moderately similar to (268)AT5G08740| Symbols: NDC1 | NDC1 (NAD(P)H DEHYDROGENASE C1); NADH dehydrogenase | chr5:28	
JCVI_29628	1.721	moderately similar to (477)AT2G16365| Symbols: | F-box family protein | chr2:7082022-7086263 FORWARD no original description	2.626
JCVI_17269	1.720	moderately similar to (380)AT3G50240| Symbols: KICP-02 | KICP-02; microtubule motor | chr3:18634362-18639766 REVERSE no orig	
DT317711	1.720	moderately similar to (219)AT4G28270| Symbols: | zinc finger (C3HC4-type RING finger) family protein | chr4:14007620-14008201 RE	
JCVI_26583	1.720	moderately similar to (210)AT5G62200| Symbols: | embryo-specific protein-related | chr5:25001689-25002900 REVERSE no original de	1.819
JCVI_31554	1.720	very weakly similar to (92.4)AT3G27350| Symbols: | similar to unknown protein [Arabidopsis thaliana] (TAIR:AT5G40700.1); similar to	1.921
EV193817	1.720	weakly similar to (101)AT5G12440| Symbols: | zinc finger (CCCH-type) family protein | chr5:4035892-4038311 REVERSE [21489] 39	
EV130420	1.719	no similarity	
JCVI_32590	1.718	moderately similar to (300)AT4G04450| Symbols: ATWRKY42, WRKY42 | WRKY42 (WRKY DNA-binding protein 42); transcription	
EH419222	1.717	very weakly similar to (87.4)AT4G12300| Symbols: CYP706A4 | CYP706A4 (cytochrome P450, family 706, subfamily A, polypeptide 4)	
EV076440	1.716	moderately similar to (289)AT2G41835| Symbols: | zinc finger (C2H2 type, AN1-like) family protein | chr2:17465098-17466492 REVE	
EE564109	1.715	no similarity	1.452
AM394473	1.715	no similarity	
ES897894	1.714	weakly similar to (133)AT1G67370| Symbols: ASY1 | ASY1 (ASYNAPTIC 1); DNA binding | chr1:25243010-25247376 REVERSE [21	
ES935840	1.713	no similarity	
JCVI_35591	1.713	no original description	
EX134882	1.712	moderately similar to (266)AT1G27100| Symbols: | similar to unknown protein [Arabidopsis thaliana] (TAIR:AT1G69900.1); similar to	
JCVI_25817	1.711	no original description	
EX039908	1.711	moderately similar to (267)AT1G22770| Symbols: FB, GI | GI (GIGANTEA); binding | chr1:8062387-8067436 FORWARDweakly simil	
JCVI_37289	1.710	no original description	
JCVI_36476	1.710	highly similar to (525)AT1G78070| Symbols: | WD-40 repeat family protein | chr1:29360113-29363261 FORWARD no original descript	
JCVI_7459	1.708	weakly similar to (190)AT3G47650| Symbols: | bundle-sheath defective protein 2 family / bsd2 family | chr3:17580561-17581247 FORW	
JCVI_16417	1.708	moderately similar to (272)AT1G76500| Symbols: | DNA-binding family protein | chr1:28710426-28711334 FORWARD no original des	
EE477064	1.707	no similarity	
EE553905	1.707	moderately similar to (222)AT5G53080| Symbols: | kinesin light chain-related | chr5:21537850-21540384 FORWARD [20184] 16 488 5	
EV216760	1.707	no similarity	2.097
ES911257	1.705	weakly similar to (112)AT4G35090| Symbols: CAT2 | CAT2 (CATALASE 2); catalase | chr4:16701110-16703220 REVERSEweakly sim	
RC_ES965974	1.704	no similarity	
JCVI_927	1.704	moderately similar to (352)AT1G68410| Symbols: | protein phosphatase 2C-related / PP2C-related | chr1:25653925-25655918 REVERS	
EX062158	1.704	weakly similar to (164)AT5G49220| Symbols: | similar to unknown protein [Arabidopsis thaliana] (TAIR:AT2G01260.1); similar to unn	
JCVI_2263	1.704	weakly similar to (195)AT5G23750| Symbols: | remorin family protein | chr5:8010007-8011456 REVERSEweakly similar to (141)REM	
EV152128	1.703	weakly similar to (184)AT5G22020| Symbols: | strictosidine synthase family protein | chr5:7287881-7289360 REVERSE [21483]	
CN727916	1.703	weakly similar to (167)AT5G62680| Symbols: | proton-dependent oligopeptide transport (POT) family protein | chr5:25182656-2518504	
EX017231	1.703	weakly similar to (174)AT1G62720| Symbols: | binding | chr1:23231239-23232696 FORWARD [21809]	
EE566457	1.702	no similarity	
EV139849	1.702	moderately similar to (273)AT5G59750| Symbols: | riboflavin biosynthesis protein, putative | chr5:24090623-24092637 FORWARD [21	1.700
EE569137	1.700	no similarity	
JCVI_7479	1.700	moderately similar to (261)AT1G21010| Symbols: | similar to unknown protein [Arabidopsis thaliana] (TAIR:AT1G76600.1); similar to	
EV197639	1.697	moderately similar to (289)AT4G30780| Symbols: | similar to unknown protein [Arabidopsis thaliana] (TAIR:AT2G24100.1); similar to	1.324
JCVI_41409	1.697	weakly similar to (166)AT1G68570| Symbols: | proton-dependent oligopeptide transport (POT) family protein | chr1:25750474-2575377	
ES947980	1.697	no similarity	2.184
JCVI_2748	1.697	moderately similar to (364)AT4G35090| Symbols: CAT2 | CAT2 (CATALASE 2); catalase | chr4:16701110-16703220 REVERSEmodera	
ES923548	1.696	weakly similar to (130)AT1G34010| Symbols: | similar to unknown protein [Arabidopsis thaliana] (TAIR:AT1G22790.2); similar to unk	
JCVI_38973	1.696	weakly similar to (147)AT1G31460| Symbols: | similar to unknown protein [Arabidopsis thaliana] (TAIR:AT1G23270.1); similar to hyp	
EX052463	1.696	very weakly similar to (84.7)AT4G19645| Symbols: | similar to unknown protein [Arabidopsis thaliana] (TAIR:AT1G31300.2); similar to	
ES966048	1.695	no similarity	
JCVI_4522	1.694	moderately similar to (202)AT3G51780| Symbols: ATBAG4 | ATBAG4 (ARABIDOPSIS THALIANA BCL-2-ASSOCIATED ATHANO	
EX093176	1.693	weakly similar to (166)AT1G34010| Symbols: | similar to unknown protein [Arabidopsis thaliana] (TAIR:AT1G22790.2); similar to unk	
ES953802	1.692	no similarity	1.792
EV198684	1.691	very weakly similar to (89.7)AT2G46830| Symbols: CCA1 | CCA1 (CIRCADIAN CLOCK ASSOCIATED 1); transcription factor | chr2:1	
JCVI_38759	1.691	weakly similar to (124)AT1G18570| Symbols: AtMYB51, BW51A, BW51B, MYB51 | MYB51 (MYB DOMAIN PROTEIN 51); DNA b	
EE448817	1.690	very weakly similar to (94.4)AT5G66675| Symbols: | Identical to UPF0496 protein At5g66675 [Arabidopsis Thaliana] (GB:Q8GW16); si	1.048
EX099101	1.690	no similarity	
JCVI_27010	1.689	moderately similar to (201)AT3G02850| Symbols: SKOR | SKOR (stelar K+ outward rectifier); cyclic nucleotide binding / outward rectif	2.375
AM059909	1.688	weakly similar to (135)AT4G14560| Symbols: AXR5, IAA1 | IAA1 (INDOLE-3-ACETIC ACID INDUCIBLE); transcription factor | chr	2.260
JCVI_25793	1.687	no original description	
EV165005	1.687	no similarity	
JCVI_36555	1.685	weakly similar to (181)AT1G12220| Symbols: RPS5 | RPS5 (RESISTANT TO P. SYRINGAE 5) | chr1:4145009-4147678 FORWARD n	

JCVI_23095	1.685	highly similar to (505)AT4G04020| Symbols: FIB | FIB (FIBRILLIN); structural molecule | chr4:1932159-1933544 FORWARDmoderate	
EV167312	1.684	moderately similar to (288)AT5G27410| Symbols: | aminotransferase class IV family protein | chr5:9678824-9682471 FORWARD [214	
JCVI_14455	1.683	weakly similar to (181)AT4G37590| Symbols: | phototropic-responsive NPH3 family protein | chr4:17663074-17665293 REVERSEvery	
CX280442	1.683	weakly similar to (122)AT3G04080| Symbols: ATAPY1 | ATAPY1 (APYRASE 1); calmodulin binding | chr3:1068075-1070924 REVER	1.440
JCVI_24093	1.682	very weakly similar to (81.3)AT2G23550| Symbols: | hydrolase | chr2:10034893-10035847 REVERSE no original description	
EE535642	1.682	weakly similar to (124)AT5G04360| Symbols: ATPU1, ATLDA | ATLDA/ATPU1 (PULLULANASE 1); alpha-amylase/ limit dextrinase	
EV041581	1.680	weakly similar to (108)AT1G22050| Symbols: MUB6 | MUB6 (MEMBRANE-ANCHORED UBIQUITIN-FOLD PROTEIN 6 PRECUR	
JCVI_21086	1.680	no original description	
EV206346	1.680	very weakly similar to (86.3)AT5G08720| Symbols: | similar to unknown protein [Arabidopsis thaliana] (TAIR:AT4G01650.2); similar to	
JCVI_18429	1.679	moderately similar to (452)AT4G22980| Symbols: | similar to catalytic/ pyridoxal phosphate binding [Arabidopsis thaliana] (TAIR:AT5	
EX027736	1.678	moderately similar to (314)AT2G33060| Symbols: | leucine-rich repeat family protein | chr2:14032738-14035164 FORWARDweakly sin	
JCVI_12242	1.678	nearly identical (1075)AT4G34860| Symbols: | beta-fructofuranosidase, putative / invertase, putative / saccharase, putative / beta-fructosi	
JCVI_40140	1.677	weakly similar to (137)AT5G44005| Symbols: | unknown protein | chr5:17722436-17722720 REVERSE no original description	1.817
JCVI_12819	1.676	moderately similar to (477)AT2G38170| Symbols: ATCAX1, RCI4, CAX1 | CAX1 (CATION EXCHANGER 1); calcium:hydrogen antip	
ES965932	1.674	no similarity	
JCVI_35746	1.674	nearly identical (1019)AT1G73680| Symbols: | pathogen-responsive alpha-dioxygenase, putative | chr1:27707882-27711078 REVERSE n	
EE414947	1.673	very weakly similar to (89.0)AT2G18540| Symbols: | cupin family protein | chr2:8049464-8052090 REVERSE [20141]	
EE482292	1.672	very weakly similar to (80.9)AT1G07135| Symbols: | glycine-rich protein | chr1:2190156-2190623 REVERSE [20154]	1.850
JCVI_18868	1.672	weakly similar to (150)AT4G22780| Symbols: ACR7 | ACR7 (ACT Domain Repeat 7) | chr4:11968707-11970967 REVERSE no original	
EV052565	1.671	moderately similar to (279)AT3G48510| Symbols: | similar to unknown protein [Arabidopsis thaliana] (TAIR:AT5G63350.1); similar to	
EX030491	1.671	moderately similar to (206)AT1G29690| Symbols: CAD1 | CAD1 (CONSTITUTIVELY ACTIVATED CELL DEATH 1) | chr1:1037929	
EE414354	1.670	moderately similar to (345)AT1G74650| Symbols: AtY13, AtMYB31 | AtMYB31/AtY13 (myb domain protein 31); DNA binding / transc	
EX108966	1.670	moderately similar to (320)AT3G04890| Symbols: | similar to unknown protein [Arabidopsis thaliana] (TAIR:AT2G46100.1); similar to	1.689
EV108070	1.670	no similarity	
EE551684	1.668	no similarity	
EX111629	1.667	weakly similar to (115)AT5G08330| Symbols: | TCP family transcription factor, putative | chr5:2680829-2681548 FORWARD [21827] 1	
JCVI_30477	1.667	moderately similar to (284)AT5G62070| Symbols: IQD23 | IQD23 (IQ-domain 23); calmodulin binding | chr5:24947283-24949677 REV	
EV045136	1.667	no similarity	
JCVI_13341	1.667	moderately similar to (408)AT1G01050| Symbols: ATPPA1 | ATPPA1 (ARABIDOPSIS THALIANA PYROPHOSPHORYLASE 1); inor	
JCVI_24119	1.667	very weakly similar to (86.3)AT3G62260| Symbols: | protein phosphatase 2C, putative / PP2C, putative | chr3:23049491-23051366 REV	
EV177748	1.666	moderately similar to (399)AT1G44100| Symbols: AAP5 | AAP5 (amino acid permease 5); amino acid transmembrane transporter | chr1:1	
EV209272	1.666	no similarity	1.864
JCVI_19681	1.665	moderately similar to (314)AT1G08630| Symbols: THA1 | THA1 (THREONINE ALDOLASE 1) | chr1:2743951-2745688 REVERSE no	
EE535792	1.664	weakly similar to (144)AT3G52040| Symbols: | similar to unnamed protein product [Vitis vinifera] (GB:CAO40759.1) | chr3:19315346-1	
RC_CO749971	1.664	no similarity	
JCVI_28076	1.663	moderately similar to (335)AT4G22100| Symbols: | glycosyl hydrolase family 1 protein | chr4:11707382-11709944 REVERSEweakly sir	
RC_EX099573	1.663	no similarity	
DY024253	1.663	moderately similar to (344)AT4G34610| Symbols: BLH6 | BLH6 (BELL1-LIKE HOMEODOMAIN 5) | chr4:16530551-16532503 REVE	1.857
EV205067	1.662	weakly similar to (134)AT1G71810| Symbols: | ABC1 family protein | chr1:27006264-27011626 REVERSE [21491]	
EV042239	1.661	moderately similar to (232)AT4G37460| Symbols: | binding | chr4:17608617-17615528 REVERSE [21442]	1.262
JCVI_36201	1.661	no original description	
JCVI_37130	1.660	moderately similar to (293)AT1G45474| Symbols: LHCA5 | LHCA5 (Photosystem I light harvesting complex gene 5) | chr1:17181793-17	1.746
ES998075	1.659	very weakly similar to (87.4)AT2G41250| Symbols: | haloacid dehalogenase-like hydrolase family protein | chr2:17207940-17209629 RE	
EV128716	1.657	no similarity	
EV190021	1.657	no similarity	2.050
JCVI_38075	1.657	moderately similar to (214)AT3G19800| Symbols: | similar to unnamed protein product [Vitis vinifera] (GB:CAO15360.1) | chr3:687615	2.157
EE512033	1.657	very weakly similar to (85.9)AT2G34720| Symbols: | CCAAT-binding transcription factor (CBF-B/NF-YA) family protein | chr2:146570	
CN730708	1.657	no similarity	
JCVI_28058	1.657	highly similar to (560)AT1G75880| Symbols: | family II extracellular lipase 1 (EXL1) | chr1:28494225-28495959 FORWARDmoderately	
JCVI_27097	1.656	moderately similar to (313)AT3G21790| Symbols: | UDP-glucoronosyl/UDP-glucosyl transferase family protein | chr3:7676934-7678421	
EE532961	1.655	no similarity	
DY026490	1.654	weakly similar to (154)AT5G58660| Symbols: | oxidoreductase, 2OG-Fe(II) oxygenase family protein | chr5:23718735-23721028 FORW	
JCVI_17245	1.654	moderately similar to (273)AT5G50100| Symbols: | similar to PBng143 [Vigna radiata] (GB:BAB82450.1); contains InterPro domain Th	
EV047731	1.654	no similarity	
CX192067	1.654	weakly similar to (120)AT5G23750| Symbols: | remorin family protein | chr5:8010007-8011456 REVERSEvery weakly similar to (80.1)	
ES904856	1.653	weakly similar to (165)AT5G62130| Symbols: | Per1-like protein-related | chr5:24967911-24970144 REVERSE [21432] 1 605 650	
EV176230	1.653	no similarity	
JCVI_7918	1.653	moderately similar to (405)AT1G77090| Symbols: | thylakoid lumenal 29.8 kDa protein | chr1:28965470-28966769 REVERSE no origina	
CN729294	1.653	moderately similar to (207)AT4G19450| Symbols: | nodulin-related | chr4:10606549-10609229 FORWARD [15725]	
JCVI_33153	1.652	moderately similar to (268)AT5G15550| Symbols: | transducin family protein / WD-40 repeat family protein | chr5:5059318-5062006 RE	
JCVI_13868	1.652	weakly similar to (123)AT3G12320| Symbols: | similar to unknown protein [Arabidopsis thaliana] (TAIR:AT5G06980.1); similar to hyp	
JCVI_39183	1.649	very weakly similar to (98.2)AT5G13730| Symbols: SIGD, SIG4 | SIG4 (SIGMA FACTOR 4); DNA binding / DNA-directed RNA polym	1.219
JCVI_26722	1.648	moderately similar to (331)AT2G41120| Symbols: | similar to unnamed protein product [Vitis vinifera] (GB:CAO18305.1); contains Inte	
JCVI_5209	1.647	moderately similar to (374)AT3G26210| Symbols: CYP71B23 | CYP71B23 (cytochrome P450, family 71, subfamily B, polypeptide 23);	
JCVI_39350	1.647	moderately similar to (348)AT1G67370| Symbols: ASY1 | ASY1 (ASYNAPTIC 1); DNA binding | chr1:25243010-25247376 REVERSE	
JCVI_813	1.646	weakly similar to (147)AT3G56880| Symbols: | VQ motif-containing protein | chr3:21071023-21071760 FORWARD no original descrip	
EV035703	1.646	no similarity	
EE564648	1.645	no similarity	
JCVI_29438	1.644	weakly similar to (160)AT3G14680| Symbols: CYP72A14 | CYP72A14 (cytochrome P450, family 72, subfamily A, polypeptide 14); oxy	
JCVI_1254	1.644	weakly similar to (162)AT3G48240| Symbols: | octicosapeptide/Phox/Bem1p (PB1) domain-containing protein | chr3:17878366-178789	
ES266449	1.644	weakly similar to (161)AT3G62260| Symbols: | protein phosphatase 2C, putative / PP2C, putative | chr3:23049491-23051366 REVERSE	
JCVI_34519	1.643	weakly similar to (105)AT5G64350| Symbols: ATFKBP12, FKBP12 | FKBP12 (FK506-binding protein 12 kD); FK506 binding / peptidy	1.705
CO749481	1.643	weakly similar to (140)AT1G75100| Symbols: JAC1 | JAC1 (J-DOMAIN PROTEIN REQUIRED FOR CHLOROPLAST ACCUMULAT	
JCVI_20853	1.642	moderately similar to (278)AT1G08530| Symbols: | similar to unknown protein [Arabidopsis thaliana] (TAIR:AT5G09995.3); similar to	
JCVI_766	1.641	moderately similar to (374)AT4G09350| Symbols: | DNAJ heat shock N-terminal domain-containing protein | chr4:5931314-5932149 RE	1.735
JCVI_31156	1.641	weakly similar to (166)AT5G27600| Symbols: LACS7 | LACS7 (LONG-CHAIN ACYL-COA SYNTHETASE 7) | chr5:9742619-974679	
AM395056	1.640	weakly similar to (106)AT2G32120| Symbols: HSP70T-2 | HSP70T-2; ATP binding | chr2:13658797-13660488 REVERSE [20346]	
JCVI_6920	1.640	highly similar to (866)AT1G53140| Symbols: | dynamin family protein | chr1:19802939-19806109 FORWARD no original description	
EV008362	1.640	weakly similar to (122)AT1G63470| Symbols: | DNA-binding family protein | chr1:23540496-23542528 REVERSE [21427] 1 605 619	
EE519214	1.640	no similarity	
H74609	1.639	no similarity	
EE569260	1.639	no similarity	
EV108331	1.639	no similarity	

JCVI_11824	1.637	moderately similar to (459)AT3G05200| Symbols: ATL6 | ATL6 (Arabidopsis T?xicos en Levadura 6); protein binding / zinc ion binding	
EV146350	1.635	no similarity	
ES901040	1.635	weakly similar to (188)AT3G05165| Symbols: | sugar transporter, putative | chr3:1458293-1462743 REVERSE [21428]	0.995
CV546543	1.634	weakly similar to (111)AT1G17620| Symbols: | similar to unknown protein [Arabidopsis thaliana] (TAIR:AT5G11890.1); similar to unkn	1.919
JCVI_308	1.634	moderately similar to (279)AT3G52800| Symbols: | zinc finger (AN1-like) family protein | chr3:19580784-19581296 FORWARDweakly	
JCVI_30460	1.633	highly similar to (672)AT3G02875| Symbols: ILR1 | ILR1 (IAA-LEUCINE RESISTANT 1); metallopeptidase | chr3:632000-633866 FOR	
DW998864	1.632	weakly similar to (124)AT1G74450| Symbols: | similar to unknown protein [Arabidopsis thaliana] (TAIR:AT1G18740.1); similar to unna	
JCVI_7637	1.632	weakly similar to (147)AT3G25950| Symbols: | similar to DNA-binding storekeeper protein-related [Arabidopsis thaliana] (TAIR:AT5G1	
EV006195	1.631	moderately similar to (238)AT2G45870| Symbols: | Identical to UPF0187 protein At2g45870, chloroplast precursor [Arabidopsis Thalian	
EV099861	1.631	moderately similar to (320)AT1G78070| Symbols: | WD-40 repeat family protein | chr1:29360113-29363261 FORWARD [21477] 104 9	
EX098994	1.631	moderately similar to (226)AT4G37590| Symbols: | phototropic-responsive NPH3 family protein | chr4:17663074-17665293 REVERSEv	
EE462682	1.631	no similarity	
EE563089	1.631	no similarity	
JCVI_37924	1.630	weakly similar to (162)AT5G52750| Symbols: | heavy-metal-associated domain-containing protein | chr5:21401360-21402021 FORWAR	
JCVI_37564	1.628	weakly similar to (140)AT3G14420| Symbols: | (S)-2-hydroxy-acid oxidase, peroxisomal, putative / glycolate oxidase, putative / short ch	1.628
JCVI_301	1.628	moderately similar to (328)AT2G37220| Symbols: | 29 kDa ribonucleoprotein, chloroplast, putative / RNA-binding protein cp29, putative	
EV007990	1.627	moderately similar to (263)AT5G53920| Symbols: | ribosomal protein L11 methyltransferase-related | chr5:21909350-21911313 FORWA	
EV034359	1.627	no similarity	
EH422476	1.626	no similarity	
JCVI_6726	1.626	moderately similar to (273)AT3G21510| Symbols: AHP1 | AHP1 (HISTIDINE-CONTAINING PHOSPHOTRANSMITTER 3); histidine	
ES940059	1.626	no similarity	
JCVI_16321	1.625	moderately similar to (263)AT4G34150| Symbols: | C2 domain-containing protein | chr4:16355039-16356959 FORWARD no original de	
EE568740	1.625	no similarity	
DY004109	1.625	weakly similar to (144)AT3G62260| Symbols: | protein phosphatase 2C, putative / PP2C, putative | chr3:23049491-23051366 REVERSE	
JCVI_10451	1.625	no original description	
JCVI_41295	1.624	very weakly similar to (89.4)AT1G12280| Symbols: | disease resistance protein (CC-NBS-LRR class), putative | chr1:4174873-4177557 F	
JCVI_24647	1.624	no original description	
JCVI_11120	1.624	very weakly similar to (92.4)AT5G44510| Symbols: | disease resistance protein (TIR-NBS-LRR class), putative | chr5:17946900-1795141	
JCVI_6202	1.623	moderately similar to (253)AT1G76880| Symbols: | trihelix DNA-binding protein, putative | chr1:28870488-28872825 FORWARD no or	
JCVI_38446	1.622	no original description	
JCVI_25466	1.622	highly similar to (675)AT1G08230| Symbols: | amino acid transporter family protein | chr1:2583712-2586697 REVERSE no original des	
EX131001	1.621	weakly similar to (131)AT2G37530| Symbols: | similar to unknown protein [Arabidopsis thaliana] (TAIR:AT1G07795.1); similar to hypo	
EV077920	1.621	no similarity	
JCVI_10262	1.621	moderately similar to (291)AT1G21010| Symbols: | similar to unknown protein [Arabidopsis thaliana] (TAIR:AT1G76600.1); similar to u	
ES983579	1.620	no similarity	
JCVI_15812	1.620	moderately similar to (218)AT4G12980| Symbols: | auxin-responsive protein, putative | chr4:7589667-7591071 REVERSE no original de	1.650
ES964543	1.620	no similarity	
CX189998	1.620	very weakly similar to (96.3)AT5G45400| Symbols: | replication protein, putative | chr5:18416217-18418871 FORWARD [16807]	
JCVI_20165	1.619	moderately similar to (436)AT1G77990| Symbols: SULTR2;2, AST56 | AST56 (sulphate transporter 2;2); sulfate transmembrane transpor	
JCVI_20545	1.619	moderately similar to (435)AT4G25130| Symbols: | peptide methionine sulfoxide reductase, putative | chr4:12898812-12900008 REVER	
RC_ES979977	1.617	no similarity	
EV091132	1.617	no similarity	1.334
BQ704570	1.617	no similarity	2.578
JCVI_16188	1.616	moderately similar to (303)AT1G70070| Symbols: PDE317, ISE2, EMB25 | EMB25 (EMBRYO DEFECTIVE 25); ATP-dependent helica	
JCVI_31665	1.615	moderately similar to (443)AT2G40730| Symbols: | HEAT repeat-containing protein | chr2:16997161-17003150 REVERSE no original d	
JCVI_16811	1.613	weakly similar to (162)AT4G18740| Symbols: | similar to unnamed protein product [Vitis vinifera] (GB:CAO48031.1) | chr4:10303554-1	
JCVI_34572	1.613	moderately similar to (266)AT1G15760| Symbols: | similar to unknown protein [Arabidopsis thaliana] (TAIR:AT1G80520.1); similar to u	
JCVI_40795	1.613	moderately similar to (223)AT4G36050| Symbols: | endonuclease/exonuclease/phosphatase family protein | chr4:17052326-17055047 RE	
EV204270	1.613	no similarity	1.712
JCVI_18261	1.612	moderately similar to (418)AT1G21450| Symbols: SCL1 | SCL1 (SCARECROW-LIKE 1); transcription factor | chr1:7509710-7511491 F	
JCVI_19482	1.611	moderately similar to (488)AT1G76350| Symbols: | RWP-RK domain-containing protein | chr1:28645037-28647711 FORWARD no orig	
JCVI_1634	1.610	moderately similar to (312)AT1G23205| Symbols: | invertase/pectin methylesterase inhibitor family protein | chr1:8234223-8234840 REV	
JCVI_28588	1.610	highly similar to (646)AT5G47910| Symbols: ATRBOHD, RBOHD | RBOHD (RESPIRATORY BURST OXIDASE PROTEIN D) | chr5	
EX131487	1.609	moderately similar to (252)AT4G30850| Symbols: HHP2 | HHP2 (HEPTAHELICAL TRANSMEMBRANE PROTEIN2) | chr4:1502054(	
EV138048	1.608	no similarity	
AM394249	1.607	weakly similar to (104)AT4G06634| Symbols: | zinc finger (C2H2 type) family protein | chr4:3764496-3766174 REVERSE [20346] 1 42	
ES909645	1.606	weakly similar to (117)AT2G47710| Symbols: | universal stress protein (USP) family protein | chr2:19562115-19563026 REVERSE [214	
JCVI_32380	1.606	moderately similar to (428)AT3G53260| Symbols: PAL2 | PAL2 (phenylalanine ammonia-lyase 2); phenylalanine ammonia-lyase | chr3:1	
JCVI_9019	1.605	moderately similar to (361)AT4G31870| Symbols: ATGPX7 | ATGPX7 (GLUTATHIONE PEROXIDASE 7); glutathione peroxidase | ch	1.918
JCVI_28807	1.604	moderately similar to (420)AT3G44610| Symbols: | protein kinase family protein | chr3:16199253-16203094 REVERSEmoderately simil	
EV102803	1.604	moderately similar to (226)AT4G12310| Symbols: CYP706A5 | CYP706A5 (cytochrome P450, family 706, subfamily A, polypeptide 5);	
JCVI_26937	1.604	no original description	
JCVI_16857	1.603	no original description	
JCVI_27837	1.602	weakly similar to (139)AT1G45230| Symbols: | defective chloroplasts and leaves protein-related / DCL protein-related | chr1:17172314-1	
JCVI_10733	1.602	moderately similar to (325)AT2G33310| Symbols: IAA13 | IAA13 (indoleacetic acid-induced protein 13) | chr2:14121646-14122834 REV	
JCVI_1603	1.601	moderately similar to (480)AT1G32240| Symbols: KAN2 | KAN2 (KANADI 2); DNA binding / transcription factor | chr1:11625862-116	
EL588585	1.601	very weakly similar to (94.7)AT1G09660| Symbols: | KH domain-containing quaking protein, putative | chr1:3128207-3130793 REVERS	1.484
JCVI_2483	1.600	moderately similar to (352)AT3G12490| Symbols: | cysteine protease inhibitor, putative / cystatin, putative | chr3:3960530-3961784 REV	
EV109979	1.600	very weakly similar to (92.4)AT5G66570| Symbols: PSBO-1, OEE1, OEE33, OE33, PSBO1 | OE33/OEE1/OEE33/PSBO-1/PSBO1 (OXY	
EE462182	1.600	weakly similar to (176)AT1G72640| Symbols: | binding / catalytic | chr1:27350071-27351809 REVERSE [15722]	
EH423274	1.600	moderately similar to (259)AT4G23530| Symbols: | similar to unknown protein [Arabidopsis thaliana] (TAIR:AT4G11300.1); similar to l	
JCVI_28743	1.598	moderately similar to (314)AT1G66390| Symbols: ATMYB90, PAP2, MYB90 | PAP2 (PRODUCTION OF ANTHOCYANIN PIGMENT	
JCVI_15620	1.598	moderately similar to (416)AT4G32770| Symbols: ATSDX1, VTE1 | VTE1 (VITAMIN E DEFICIENT 1) | chr4:15804986-15807795 FO	
ES948420	1.597	no similarity	
JCVI_8733	1.594	moderately similar to (265)AT1G52880| Symbols: ANAC018, ATNAM, NAM | NAM (Arabidopsis NAC domain containing protein 18);	
JCVI_34439	1.594	moderately similar to (343)AT3G53830| Symbols: | regulator of chromosome condensation (RCC1) family protein / UVB-resistance prot	
JCVI_18541	1.593	highly similar to (504)AT1G13710| Symbols: CYP78A5 | CYP78A5 (cytochrome P450, family 78, subfamily A, polypeptide 5); oxygen l	
JCVI_23598	1.593	moderately similar to (332)AT1G75180| Symbols: | similar to unknown protein [Arabidopsis thaliana] (TAIR:AT1G19400.1); similar to u	
AM389654	1.593	no similarity	
JCVI_6904	1.592	moderately similar to (434)AT1G76110| Symbols: | high mobility group (HMG1/2) family protein / ARID/BRIGHT DNA-binding domai	
JCVI_9640	1.591	moderately similar to (251)AT1G66330| Symbols: | senescence-associated family protein | chr1:24733543-24735807 REVERSE no origi	
EV105665	1.591	no similarity	
AM056601	1.591	moderately similar to (220)AT1G14480| Symbols: | protein binding | chr1:4956399-4957883 FORWARD [17712]	

EV141915	1.589	no similarity	2.031
JCVI_29448	1.589	no original description	
JCVI_8974	1.589	highly similar to (510)AT2G40900| Symbols: | nodulin MtN21 family protein | chr2:17070474-17072592 REVERSE no original descript	
JCVI_34522	1.589	highly similar to (553)AT3G44290| Symbols: ANAC060 | ANAC060 (Arabidopsis NAC domain containing protein 60); transcription fact	
JCVI_33872	1.588	moderately similar to (289)AT5G64230| Symbols: | similar to unknown protein [Arabidopsis thaliana] (TAIR:AT3G19920.1); similar to l	
JCVI_14961	1.587	moderately similar to (232)AT1G10960| Symbols: ATFD1 | ATFD1 (FERREDOXIN 1); 2 iron, 2 sulfur cluster binding / electron carrier/	
EV210544	1.586	moderately similar to (221)AT4G36550| Symbols: | binding / ubiquitin-protein ligase | chr4:17245403-17247721 REVERSE [21491] 51	
EV071811	1.585	no similarity	
JCVI_3315	1.584	moderately similar to (330)AT5G65840| Symbols: | similar to antioxidant/ oxidoreductase [Arabidopsis thaliana] (TAIR:AT2G37240.1);	
JCVI_17301	1.583	moderately similar to (301)AT4G31390| Symbols: | ABC1 family protein | chr4:15233132-15236770 FORWARD no original description	
EV185414	1.583	no similarity	1.537
JCVI_3689	1.582	very weakly similar to (80.9)AT5G13090| Symbols: | similar to unknown protein [Arabidopsis thaliana] (TAIR:AT1G24270.1); similar to	
BG543396	1.581	no similarity	
CX192368	1.580	very weakly similar to (99.0)AT2G42190| Symbols: | similar to unknown protein [Arabidopsis thaliana] (TAIR:AT3G57930.1); similar to	1.301
EX063148	1.579	no similarity	
EV171189	1.578	weakly similar to (140)AT4G36550| Symbols: | binding / ubiquitin-protein ligase | chr4:17245403-17247721 REVERSE [21486] 96 905	
JCVI_39471	1.578	no original description	
JCVI_22248	1.577	weakly similar to (104)AT5G06520| Symbols: | SWAP (Suppressor-of-White-APricot)/surp domain-containing protein | chr5:1987251-1	
EX044469	1.575	moderately similar to (296)AT3G14690| Symbols: CYP72A15 | CYP72A15 (cytochrome P450, family 72, subfamily A, polypeptide 15);	
ES929102	1.575	moderately similar to (204)AT5G11260| Symbols: TED 5, HY5 | HY5 (ELONGATED HYPOCOTYL 5); DNA binding / transcription fa	
JCVI_16714	1.574	weakly similar to (156)AT3G14010| Symbols: CID4 | CID4 (CTC-Interacting Domain 4) | chr3:4637171-4640698 FORWARD no origina	
JCVI_42421	1.573	moderately similar to (246)AT2G27480| Symbols: | calcium ion binding | chr2:11753889-11754963 FORWARD no original description	1.497
DY020430	1.570	moderately similar to (316)AT4G22900| Symbols: | similar to unknown protein [Arabidopsis thaliana] (TAIR:AT4G11950.1); similar to	1.886
EV073235	1.570	weakly similar to (102)AT4G12130| Symbols: | aminomethyltransferase | chr4:7263634-7265419 FORWARD [21443]	
CN729023	1.569	no similarity	
JCVI_20564	1.569	weakly similar to (144)AT5G62520| Symbols: SRO5 | SRO5 (SIMILAR TO RCD ONE 5); NAD+ ADP-ribosyltransferase | chr5:251152	
EX123618	1.569	moderately similar to (371)AT5G40160| Symbols: EMB506 | EMB506 (EMBRYO DEFECTIVE 506); protein binding | chr5:16079954-1	1.547
JCVI_38632	1.568	moderately similar to (457)AT5G44110| Symbols: ATPOP1, ATNAP2, POP1 | POP1 | chr5:17771619-17772899 REVERSE no original	
JCVI_35560	1.568	no original description	1.809
EE424482	1.567	weakly similar to (168)AT1G17147| Symbols: | similar to VQ motif-containing protein [Arabidopsis thaliana] (TAIR:AT1G78410.1); sin	1.446
JCVI_6813	1.567	moderately similar to (425)AT1G36730| Symbols: | eukaryotic translation initiation factor 5, putative / eIF-5, putative | chr1:13899918-1	
ES961328	1.567	moderately similar to (337)AT3G18390| Symbols: EMB1865 | EMB1865 (EMBRYO DEFECTIVE 1865) | chr3:6313578-6317590 FOR	
JCVI_150	1.567	moderately similar to (243)AT2G33800| Symbols: | ribosomal protein S5 family protein | chr2:14308001-14309428 REVERSEweakly si	
EE533089	1.567	moderately similar to (208)AT2G44270| Symbols: | similar to unknown protein [Arabidopsis thaliana] (TAIR:AT1G76170.1); similar to	
JCVI_26539	1.567	weakly similar to (150)AT5G53590| Symbols: | auxin-responsive family protein | chr5:21789333-21789761 FORWARD no original desc	
JCVI_12051	1.566	weakly similar to (136)AT1G18170| Symbols: | immunophilin / FKBP-type peptidyl-prolyl cis-trans isomerase family protein | chr1:625	
JCVI_23675	1.565	moderately similar to (465)AT4G34830| Symbols: | binding | chr4:16599981-16605999 REVERSEvery weakly similar to (100)RF1_OR	
JCVI_22859	1.564	moderately similar to (236)AT1G42540| Symbols: GLR3.3, ATGLR3.3 | ATGLR3.3 (Arabidopsis thaliana glutamate receptor 3.3) | chr1:	
CB686317	1.564	no similarity	
JCVI_33299	1.562	weakly similar to (165)AT2G37530| Symbols: | similar to unknown protein [Arabidopsis thaliana] (TAIR:AT1G07795.1); similar to hyp	
JCVI_21849	1.562	moderately similar to (486)AT5G41080| Symbols: | glycerophosphoryl diester phosphodiesterase family protein | chr5:16459217-164610	2.002
JCVI_30588	1.562	no original description	
EX043068	1.560	no similarity	
JCVI_8753	1.560	moderately similar to (420)AT4G34680| Symbols: | GATA transcription factor 3, putative (GATA-3) | chr4:16553705-16554615 FORW	
JCVI_11117	1.560	moderately similar to (307)AT5G24460| Symbols: | hydrolase | chr5:8354818-8355720 FORWARD no original description	
RC_EX052706	1.559	no similarity	
JCVI_845	1.559	weakly similar to (178)AT2G01850| Symbols: XTH27, ATXTH27, EXGT-A3 | EXGT-A3 (endo-xyloglucan transferase A3); hydrolase,	
EE481286	1.559	no similarity	1.872
JCVI_22370	1.558	highly similar to (588)AT2G30840| Symbols: | 2-oxoglutarate-dependent dioxygenase, putative | chr2:13142658-13143910 REVERSEm	
JCVI_22481	1.558	moderately similar to (240)AT5G16170| Symbols: | similar to unknown protein [Arabidopsis thaliana] (TAIR:AT5G11730.1); similar to	
EE569362	1.558	no similarity	
EE475617	1.558	no similarity	
EV223721	1.557	moderately similar to (246)AT5G13820| Symbols: ATBP-1, ATBP1, ATTBP1, HPPBF-1, TBP1 | TBP1 (TELOMERIC DNA BINDING	
AM060154	1.557	no similarity	
JCVI_38745	1.555	weakly similar to (113)AT3G11170| Symbols: FADD, FAD7 | FAD7 (FATTY ACID DESATURASE 7); omega-3 fatty acid desaturase |	
JCVI_30892	1.555	weakly similar to (132)AT1G26230| Symbols: | chaperonin, putative | chr1:9072375-9075259 REVERSE no original description	1.539
EX071950	1.553	moderately similar to (229)AT5G43630| Symbols: | zinc knuckle (CCHC-type) family protein | chr5:17544488-17547434 FORWARD [2	
ES969062	1.551	no similarity	
EV084948	1.550	weakly similar to (105)AT5G01030| Symbols: | similar to unknown protein [Arabidopsis thaliana] (TAIR:AT2G37930.1); similar to unna	
CX188778	1.547	very weakly similar to (88.6)AT5G05550| Symbols: | transcription factor | chr5:1639530-1640551 REVERSE [16807]	
EE525877	1.547	no similarity	1.634
JCVI_4988	1.547	moderately similar to (372)AT4G12320| Symbols: CYP706A6 | CYP706A6 (cytochrome P450, family 706, subfamily A, polypeptide 6);	
JCVI_30505	1.545	weakly similar to (177)AT3G59080| Symbols: | aspartyl protease family protein | chr3:21847789-21849396 FORWARD no original desc	
JCVI_33360	1.545	moderately similar to (251)AT5G15950| Symbols: | adenosylmethionine decarboxylase family protein | chr5:5206709-5207797 FORWA	
JCVI_9672	1.544	moderately similar to (375)AT2G17230| Symbols: | phosphate-responsive 1 family protein | chr2:7501974-7503065 REVERSE no origin	1.481
JCVI_26976	1.544	no original description	
EV209077	1.543	weakly similar to (135)AT5G56860| Symbols: GNC | GNC (GATA, NITRATE-INDUCIBLE, CARBON METABOLISM-INVOLVED);	
JCVI_40453	1.543	weakly similar to (158)AT3G55646| Symbols: | similar to unknown protein [Arabidopsis thaliana] (TAIR:AT2G39855.2) | chr3:2065606	
EE567750	1.542	no similarity	
DY000135	1.541	no similarity	
EE435451	1.540	no similarity	
JCVI_38024	1.540	no original description	3.293
JCVI_5494	1.538	moderately similar to (449)AT4G10120| Symbols: ATSPS4F | ATSPS4F | chr4:6315029-6319781 FORWARDweakly similar to (196)SP	
JCVI_28818	1.537	moderately similar to (258)AT2G18245| Symbols: | similar to unknown protein [Arabidopsis thaliana] (TAIR:AT3G19970.1); similar to	
JCVI_28414	1.537	no original description	1.415
JCVI_30926	1.535	no original description	
ES983022	1.534	moderately similar to (236)AT2G18940| Symbols: | pentatricopeptide (PPR) repeat-containing protein | chr2:8210955-8213423 REVERS	
EE534158	1.534	very weakly similar to (89.4)AT1G76590| Symbols: | zinc-binding family protein | chr1:28745769-28746788 FORWARD [20150] 1 540	
EE448615	1.534	very weakly similar to (86.7)AT1G27210| Symbols: | binding | chr1:9455656-9457904 REVERSE [20172]	
JCVI_25642	1.534	moderately similar to (390)AT4G23570| Symbols: SGT1A | SGT1A (Suppressor of G2 (Two) 1A) | chr4:12300025-12302503 FORWAR	
EX020651	1.532	moderately similar to (261)AT5G25840| Symbols: | similar to unknown protein [Arabidopsis thaliana] (TAIR:AT1G79770.1); similar to	1.794
JCVI_15272	1.532	moderately similar to (228)AT4G24480| Symbols: | serine/threonine protein kinase, putative | chr4:12650420-12654765 FORWARD no	1.367
ES945788	1.532	no similarity	

EV102326	1.531	moderately similar to (342)AT4G22100| Symbols: | glycosyl hydrolase family 1 protein | chr4:11707382-11709944 REVERSEweakly si	
AM386056	1.531	very weakly similar to (92.0)AT5G63630| Symbols: | DEAD box RNA helicase, putative | chr5:25489824-25492422 REVERSE [20118]	
JCVI_10376	1.531	weakly similar to (148)AT5G23660| Symbols: MTN3 | MTN3 (ARABIDOPSIS HOMOLOG OF MEDICAGO TRUNCATULA MTN3) |	1.632
JCVI_41482	1.530	no original description	
JCVI_21042	1.530	no original description	1.454
JCVI_15695	1.529	highly similar to (888)AT5G08720| Symbols: | similar to unknown protein [Arabidopsis thaliana] (TAIR:AT4G01650.2); similar to unkn	
JCVI_3098	1.528	moderately similar to (490)AT4G24390| Symbols: | F-box family protein (FBX14) | chr4:12613919-12615976 REVERSE no original des	2.210
JCVI_38999	1.528	weakly similar to (193)AT4G01280| Symbols: | myb family transcription factor | chr4:535288-536854 FORWARD no original descriptio	
H06411	1.528	no similarity	
JCVI_15851	1.527	moderately similar to (411)AT1G29690| Symbols: CAD1 | CAD1 (CONSTITUTIVELY ACTIVATED CELL DEATH 1) | chr1:1037929	
JCVI_21951	1.527	no original description	
EV110700	1.527	no similarity	
EE542499	1.524	weakly similar to (172)AT4G36470| Symbols: | S-adenosyl-L-methionine:carboxyl methyltransferase family protein | chr4:17215131-172	
EV209810	1.524	very weakly similar to (92.8)AT5G64230| Symbols: | similar to unknown protein [Arabidopsis thaliana] (TAIR:AT3G19920.1); similar to	
JCVI_36108	1.524	moderately similar to (483)AT3G21360| Symbols: | oxidoreductase | chr3:7522871-7524042 FORWARD no original description	
EV004240	1.524	moderately similar to (297)AT4G25910| Symbols: ATCNFU3, NFU3 | NFU3 (NFU domain protein 3) | chr4:13164137-13165103 FORW	
JCVI_26218	1.523	weakly similar to (129)AT1G43730| Symbols: | similar to unknown protein [Arabidopsis thaliana] (TAIR:AT4G04650.1); similar to puta	
JCVI_31684	1.523	no original description	2.586
EH419365	1.522	weakly similar to (115)AT1G29720| Symbols: | protein kinase family protein | chr1:10393880-10395067 REVERSE [20767]	
EE545987	1.522	no similarity	
JCVI_6312	1.522	weakly similar to (193)AT1G21450| Symbols: SCL1 | SCL1 (SCARECROW-LIKE 1); transcription factor | chr1:7509710-7511491 FOR	
EV122754	1.522	weakly similar to (110)AT5G48560| Symbols: | basic helix-loop-helix (bHLH) family protein | chr5:19701386-19704097 FORWARD [2	
EE425009	1.520	no similarity	
JCVI_42520	1.520	moderately similar to (464)AT3G08760| Symbols: ATSIK | ATSIK; kinase | chr3:2658135-2659990 REVERSEvery weakly similar to (87	
EE548570	1.520	no similarity	
EV106908	1.519	weakly similar to (133)AT1G75880| Symbols: | family II extracellular lipase 1 (EXL1) | chr1:28494225-28495959 FORWARD [21478]	
EE564714	1.519	no similarity	
ES935268	1.518	weakly similar to (119)AT4G34680| Symbols: | GATA transcription factor 3, putative (GATA-3) | chr4:16553705-16554615 FORWARD	
JCVI_23284	1.516	weakly similar to (130)AT5G48545| Symbols: | histidine triad family protein / HIT family protein | chr5:19693454-19695171 FORWARD	
EV027655	1.516	moderately similar to (314)AT3G19553| Symbols: | amino acid permease family protein | chr3:6790994-6792513 REVERSE [21441] 1 6	
JCVI_20309	1.514	weakly similar to (169)AT2G31370| Symbols: | bZIP transcription factor (POSF21) | chr2:13386525-13388114 FORWARDweakly simil	
EX096590	1.513	moderately similar to (204)AT1G68440| Symbols: | similar to unknown protein [Arabidopsis thaliana] (TAIR:AT1G25400.1); similar to	
JCVI_32269	1.512	moderately similar to (447)AT1G06570| Symbols: HPD, PDS1 | PDS1 (PHYTOENE DESATURATION 1) | chr1:2012014-2013542 REV	
EX062659	1.512	weakly similar to (149)AT4G33100| Symbols: | Identical to Uncharacterized protein At4g33100 [Arabidopsis Thaliana] (GB:Q9SMZ9;G	
JCVI_17232	1.511	no original description	
EV009411	1.511	no similarity	
JCVI_34659	1.511	moderately similar to (238)AT1G28360| Symbols: ERF12, ATERF12 | ATERF12/ERF12 (ERF domain protein 12); DNA binding / transc	
EE430952	1.511	no similarity	
JCVI_12278	1.507	weakly similar to (182)AT5G53970| Symbols: | aminotransferase, putative | chr5:21927902-21929820 FORWARD no original descriptio	
JCVI_36423	1.507	no original description	1.550
CD831977	1.506	weakly similar to (170)AT5G63350| Symbols: | similar to unknown protein [Arabidopsis thaliana] (TAIR:AT3G48510.1); similar to unna	1.310
JCVI_27799	1.505	weakly similar to (137)AT1G29530| Symbols: | similar to unknown protein [Arabidopsis thaliana] (TAIR:AT2G34310.1); similar to unkr	
JCVI_36711	1.504	weakly similar to (164)AT3G22120| Symbols: CWLP | CWLP (CELL WALL-PLASMA MEMBRANE LINKER PROTEIN); lipid bindir	1.627
JCVI_23014	1.503	moderately similar to (306)AT4G35440| Symbols: ATCLC-E, CLC-E | CLC-E (CHLORIDE CHANNEL E); voltage-gated chloride chan	
JCVI_6422	1.503	no original description	
JCVI_17662	1.502	moderately similar to (475)AT4G32480| Symbols: | similar to unknown protein [Arabidopsis thaliana] (TAIR:AT2G20670.1); similar to	
RC_ES967834	1.502	no similarity	1.728
JCVI_35782	1.500	moderately similar to (311)AT1G70420| Symbols: | similar to unknown protein [Arabidopsis thaliana] (TAIR:AT1G23710.1); similar to	2.075
ES927039	1.499	no similarity	
EX114983	1.498	no similarity	
JCVI_40824	1.497	highly similar to (560)AT5G60730| Symbols: | anion-transporting ATPase family protein | chr5:24440064-24442578 FORWARD no orig	
JCVI_38617	1.497	no original description	
EV227037	1.496	weakly similar to (187)AT4G22980| Symbols: | similar to catalytic/ pyridoxal phosphate binding [Arabidopsis thaliana] (TAIR:AT5G51	
JCVI_41198	1.495	no original description	
JCVI_30263	1.495	moderately similar to (390)AT5G13630| Symbols: CCH, CHLH, CCH1, GUN5 | GUN5 (GENOMES UNCOUPLED 5) | chr5:4387923-4	
EV085499	1.495	no similarity	
JCVI_28528	1.495	moderately similar to (295)AT2G19640| Symbols: ASHR2 | ASHR2 (ASH1-RELATED PROTEIN 2) | chr2:8498483-8499679 FORWAR	
JCVI_6032	1.495	moderately similar to (388)AT3G01060| Symbols: | similar to unnamed protein product [Vitis vinifera] (GB:CAO15045.1); similar to unl	
EX050363	1.493	moderately similar to (486)AT3G29400| Symbols: ATEXO70E1 | ATEXO70E1 (exocyst subunit EXO70 family protein E1); protein binc	
JCVI_7388	1.493	moderately similar to (476)AT3G04650| Symbols: | oxidoreductase | chr3:1262023-1264349 FORWARD no original description	
EV063973	1.493	no similarity	
JCVI_25868	1.491	moderately similar to (433)AT5G22020| Symbols: | strictosidine synthase family protein | chr5:7287881-7289360 REVERSEweakly simi	
EV105427	1.491	weakly similar to (119)AT5G53970| Symbols: | aminotransferase, putative | chr5:21927902-21929820 FORWARD [21478]	
EV026824	1.491	moderately similar to (353)AT5G67385| Symbols: | signal transducer | chr5:26901980-26904309 FORWARDweakly similar to (142)NP	
EX092040	1.491	very weakly similar to (92.8)AT5G17780| Symbols: | hydrolase, alpha/beta fold family protein | chr5:5867431-5868978 REVERSE [2182	
CN727142	1.490	weakly similar to (120)AT1G07650| Symbols: | leucine-rich repeat transmembrane protein kinase, putative | chr1:2359814-2366420 REV	
EX134575	1.489	no similarity	
JCVI_25190	1.488	moderately similar to (219)AT1G20330| Symbols: CVP1, FRL1, SMT2 | SMT2 (STEROL METHYLTRANSFERASE 2) | chr1:7038958-	
JCVI_25349	1.488	weakly similar to (136)AT4G00050| Symbols: UNE10 | UNE10 (unfertilized embryo sac 10); DNA binding / transcription factor | chr4:1	1.847
JCVI_11590	1.487	moderately similar to (357)AT3G16770| Symbols: RAP2.3, ATEBP, ERF72 | ATEBP/ERF72/RAP2.3 (RELATED TO AP2 3); DNA bin	
ES984062	1.484	no similarity	
EX129024	1.484	moderately similar to (216)AT4G07960| Symbols: CSLC12, ATCSLC12 | ATCSLC12 (Cellulose synthase-like C12); transferase, transfer	
EE416669	1.484	moderately similar to (352)AT5G60540| Symbols: EMB2407, ATPDX2, PDX2 | ATPDX2/EMB2407/PDX2 (PYRIDOXINE BIOSYNTI	
JCVI_32316	1.483	no original description	
JCVI_32294	1.482	highly similar to (713)AT5G27600| Symbols: LACS7 | LACS7 (LONG-CHAIN ACYL-COA SYNTHETASE 7) | chr5:9742619-9746798	
JCVI_22871	1.481	weakly similar to (111)AT1G32060| Symbols: PRK | PRK (PHOSPHORIBULOKINASE); ATP binding / phosphoribulokinase/ protein b	1.256
EV199912	1.480	moderately similar to (219)AT3G27350| Symbols: | similar to unknown protein [Arabidopsis thaliana] (TAIR:AT5G40700.1); similar to	1.540
JCVI_12696	1.480	highly similar to (640)AT4G35090| Symbols: CAT2 | CAT2 (CATALASE 2); catalase | chr4:16701110-16703220 REVERSEhighly simil	
EE511843	1.479	moderately similar to (268)AT3G29400| Symbols: ATEXO70E1 | ATEXO70E1 (exocyst subunit EXO70 family protein E1); protein binc	
JCVI_6581	1.478	moderately similar to (233)AT4G24090| Symbols: | similar to hypothetical protein [Vitis vinifera] (GB:CAN62286.1) | chr4:12512752-1	
JCVI_415	1.478	moderately similar to (402)AT1G80760| Symbols: NIP6;1, NLM7, NIP6 | NIP6;1 (NOD26-like intrinsic protein 6;1); water channel | chr1	
EV216674	1.477	moderately similar to (327)AT3G48270| Symbols: CYP71A26 | CYP71A26 (cytochrome P450, family 71, subfamily A, polypeptide 26);	
JCVI_3671	1.477	moderately similar to (345)AT5G53970| Symbols: | aminotransferase, putative | chr5:21927902-21929820 FORWARD no original descri	

JCVI_40178	1.476	moderately similar to (321)AT5G24120| Symbols: SIG5, SIGE | SIGE (RNA polymerase sigma subunit E); DNA binding / DNA-directed	
DY023268	1.476	moderately similar to (243)AT3G16230| Symbols: | RNA binding / catalytic | chr3:5500569-5503309 FORWARD [18979]	
EH422740	1.475	moderately similar to (240)AT3G56630| Symbols: CYP94D2 | CYP94D2 (cytochrome P450, family 94, subfamily D, polypeptide 2); oxy	
JCVI_8758	1.474	moderately similar to (358)AT1G77280| Symbols: | protein kinase family protein | chr1:29036362-29040776 REVERSEvery weakly sim	
RC_ES966121	1.473	no similarity	
JCVI_29171	1.473	moderately similar to (298)AT4G17350| Symbols: | similar to unknown protein [Arabidopsis thaliana] (TAIR:AT5G47440.1); similar to	
JCVI_14303	1.472	moderately similar to (448)AT1G06430| Symbols: FTSH8 | FTSH8 (FtsH protease 8); ATP-dependent peptidase/ ATPase/ metallopeptida	
JCVI_18302	1.472	moderately similar to (215)AT4G13670| Symbols: PTAC5 | PTAC5 (PLASTID TRANSCRIPTIONALLY ACTIVE5); heat shock protein	1.645
EX138021	1.471	weakly similar to (154)AT2G35510| Symbols: SRO1 | SRO1 (SIMILAR TO RCD ONE 1); NAD+ ADP-ribosyltransferase | chr2:149239	
AM061598	1.471	no similarity	
CV432495	1.470	weakly similar to (130)AT5G16280| Symbols: | binding | chr5:5323380-5331348 REVERSE [16490]	
EV200952	1.470	no similarity	
EV177166	1.469	no similarity	
EV132699	1.467	no similarity	
JCVI_7418	1.467	no original description	
JCVI_37471	1.467	weakly similar to (122)AT2G23580| Symbols: | hydrolase, alpha/beta fold family protein | chr2:10040440-10041390 REVERSEvery wea	
JCVI_38688	1.467	moderately similar to (348)AT3G16560| Symbols: | protein phosphatase 2C-related / PP2C-related | chr3:5636057-5637708 REVERSE n	
RC_EV012604	1.466	no similarity	
JCVI_8318	1.465	moderately similar to (358)AT3G10420| Symbols: | sporulation protein-related | chr3:3239312-3241576 FORWARD no original descript	
JCVI_32498	1.465	moderately similar to (240)AT4G32340| Symbols: | binding | chr4:15612747-15614225 REVERSE no original description	
JCVI_40512	1.464	moderately similar to (289)AT4G15570| Symbols: | tRNA-splicing endonuclease positive effector-related | chr4:8893041-8898856 FORV	
EV155598	1.464	moderately similar to (310)AT3G21690| Symbols: | MATE efflux family protein | chr3:7638757-7641868 FORWARD [21484] 57 775 7	1.948
EX055263	1.464	no similarity	
EV033230	1.464	moderately similar to (203)AT1G20860| Symbols: | phosphate transporter family protein | chr1:7253996-7258666 REVERSE [21441]	
EE476140	1.462	weakly similar to (127)AT5G64800| Symbols: CLE21 | CLE21 (CLAVATA3/ESR-RELATED 21); receptor binding | chr5:25923309-259	
JCVI_26040	1.461	weakly similar to (142)AT4G09690| Symbols: | DC1 domain-containing protein | chr4:6124894-6126117 FORWARD no original descrip	1.643
DY015573	1.461	no similarity	
EE402507	1.460	weakly similar to (157)AT1G19490| Symbols: | bZIP transcription factor family protein | chr1:6751944-6753950 REVERSE [16820]	
JCVI_31136	1.460	no original description	
JCVI_27274	1.459	weakly similar to (187)AT5G43260| Symbols: | chaperone protein dnaJ-related | chr5:17374921-17375214 REVERSE no original descrip	1.239
JCVI_25059	1.458	highly similar to (729)AT2G42890| Symbols: AML2 | AML2 | chr2:17857709-17861282 FORWARDvery weakly similar to (98.2)PLA2_	
JCVI_36088	1.458	moderately similar to (481)AT2G01850| Symbols: XTH27, ATXTH27, EXGT-A3 | EXGT-A3 (endo-xyloglucan transferase A3); hydrola	
EV090755	1.458	weakly similar to (181)AT1G15760| Symbols: | similar to unknown protein [Arabidopsis thaliana] (TAIR:AT1G80520.1); similar to unna	
JCVI_7186	1.457	weakly similar to (179)AT5G18140| Symbols: | DNAJ heat shock N-terminal domain-containing protein | chr5:5998237-5999701 FORW	
JCVI_31769	1.456	weakly similar to (139)AT5G02120| Symbols: OHP | OHP (ONE HELIX PROTEIN) | chr5:419141-419630 FORWARD no original desc	1.402
DY006660	1.456	weakly similar to (197)AT2G41705| Symbols: | camphor resistance CrcB family protein | chr2:17405161-17406988 FORWARD [18976]	
EV066649	1.455	weakly similar to (158)AT4G16690| Symbols: | esterase/lipase/thioesterase family protein | chr4:9392427-9393446 REVERSE [21443]	
JCVI_26264	1.455	highly similar to (949)AT4G00370| Symbols: ANTR2 | ANTR2 (anion transporter 2); organic anion transmembrane transporter | chr4:163	
AM385820	1.455	weakly similar to (117)AT3G19760| Symbols: | eukaryotic translation initiation factor 4A, putative / eIF-4A, putative / DEAD box RNA	
JCVI_7963	1.454	weakly similar to (171)AT2G33250| Symbols: | similar to unnamed protein product [Vitis vinifera] (GB:CAO71510.1) | chr2:14104531-1	
JCVI_2616	1.454	moderately similar to (208)AT5G16830| Symbols: PEP12P, SYP21, ATSYP21, PEP12, ATPEP12 | SYP21 (syntaxin 21); SNAP receptor	
JCVI_23253	1.453	moderately similar to (455)AT5G18570| Symbols: | GTP1/OBG family protein | chr5:6171841-6174825 REVERSE no original descripti	
JCVI_16807	1.453	highly similar to (856)AT3G30775| Symbols: AT-POX, ATPOX, ATPDH, PRO1, PRODH, ERD5 | ERD5 (EARLY RESPONSIVE TO I	
JCVI_4776	1.453	moderately similar to (301)AT2G29630| Symbols: | thiamine biosynthesis family protein / thiC family protein | chr2:12674472-1267664	
EX101867	1.452	moderately similar to (211)AT1G10820| Symbols: | similar to unknown protein [Arabidopsis thaliana] (TAIR:AT1G60670.2); similar to	
JCVI_2640	1.451	moderately similar to (447)AT2G17230| Symbols: | phosphate-responsive 1 family protein | chr2:7501974-7503065 REVERSE no origin	
EX085756	1.451	moderately similar to (407)AT4G12830| Symbols: | hydrolase, alpha/beta fold family protein | chr4:7531186-7533324 FORWARD [218	1.298
ES977199	1.451	no similarity	
JCVI_5544	1.451	weakly similar to (159)AT5G59050| Symbols: | unknown protein | chr5:23859075-23859745 REVERSE no original description	
AT002171	1.450	no similarity	2.397
ES899115	1.449	no similarity	
EV100249	1.448	no similarity	1.738
JCVI_16858	1.448	no original description	
JCVI_30048	1.447	moderately similar to (273)AT2G46660| Symbols: CYP78A6 | CYP78A6 (cytochrome P450, family 78, subfamily A, polypeptide 6); oxy	
EV148028	1.446	no similarity	
JCVI_7403	1.446	no original description	
EE468391	1.446	no similarity	
JCVI_34406	1.446	weakly similar to (144)AT5G18370| Symbols: | disease resistance protein (TIR-NBS-LRR class), putative | chr5:6085038-6088928 REV	
JCVI_33180	1.446	highly similar to (575)AT2G02450| Symbols: ANAC034, ANAC035 | ANAC034/ANAC035 (Arabidopsis NAC domain containing protei	1.406
JCVI_35766	1.442	moderately similar to (259)AT1G26761| Symbols: | similar to unnamed protein product [Vitis vinifera] (GB:CAO68448.1); contains dom	
JCVI_13931	1.442	weakly similar to (138)AT4G27940| Symbols: | mitochondrial substrate carrier family protein | chr4:13904751-13907042 FORWARD no	
JCVI_26456	1.441	moderately similar to (362)AT1G09810| Symbols: ECT11 | ECT11 (evolutionarily conserved C-terminal region 11) | chr1:3181140-3183	1.263
DW998250	1.441	weakly similar to (110)AT1G54030| Symbols: | GDSL-motif lipase, putative | chr1:20171353-20173144 FORWARD [18977] 1 429 465	
EX086464	1.441	moderately similar to (395)AT5G52570| Symbols: B2, CHY2, BETA-OHASE 2 | BETA-OHASE 2 (BETA-CAROTENE HYDROXYLA	
EE490474	1.440	weakly similar to (103)AT4G28740| Symbols: | similar to LPA1 (LOW PSII ACCUMULATION1), binding [Arabidopsis thaliana] (TAII	
JCVI_23768	1.439	no original description	
ES914005	1.439	weakly similar to (122)AT1G08900| Symbols: | Identical to Sugar transporter ERD6-like 2 (SUGTL3) [Arabidopsis Thaliana] (GB:Q4F	1.370
EX093767	1.439	no similarity	
EH422975	1.438	weakly similar to (126)AT3G14420| Symbols: | (S)-2-hydroxy-acid oxidase, peroxisomal, putative / glycolate oxidase, putative / short ch	1.268
JCVI_1070	1.437	highly similar to (527)AT5G63140| Symbols: ATPAP29, PAP29 | ATPAP29/PAP29 (purple acid phosphatase 29); acid phosphatase/ prot	
JCVI_18202	1.437	highly similar to (599)AT5G46570| Symbols: | protein kinase family protein | chr5:18911914-18914425 FORWARD no original descript	
JCVI_41519	1.437	very weakly similar to (81.3)AT5G65840| Symbols: | similar to antioxidant/ oxidoreductase [Arabidopsis thaliana] (TAIR:AT2G37240.1)	
JCVI_19378	1.437	moderately similar to (232)AT1G17360| Symbols: | COP1-interacting protein-related | chr1:5947434-5951210 FORWARD no original de	
EV110855	1.436	very weakly similar to (99.4)AT4G02280| Symbols: SUS3 | SUS3; UDP-glycosyltransferase/ sucrose synthase/ transferase, transferring gly	
EV012135	1.435	no similarity	
JCVI_5414	1.433	weakly similar to (161)AT2G27290| Symbols: | similar to unnamed protein product [Vitis vinifera] (GB:CAO48682.1); contains InterPro	1.316
JCVI_21447	1.433	weakly similar to (122)AT4G33920| Symbols: | protein phosphatase 2C family protein / PP2C family protein | chr4:16260881-16262708	
JCVI_7513	1.432	moderately similar to (474)AT1G64860| Symbols: SIG1, SIG2, SIGB, RPOD1, SIGA | SIGA (SIGMA FACTOR A); DNA binding / DN	1.178
JCVI_25849	1.431	moderately similar to (231)AT5G15070| Symbols: | acid phosphatase/ oxidoreductase/ transition metal ion binding | chr5:4876901-48856	
JCVI_12283	1.431	weakly similar to (162)AT5G11250| Symbols: | disease resistance protein (TIR-NBS-LRR class), putative | chr5:3587979-3591961 REV	
EV124080	1.427	moderately similar to (353)AT5G41550| Symbols: | disease resistance protein (TIR-NBS-LRR class), putative | chr5:16634460-1663801	
JCVI_17925	1.426	weakly similar to (178)AT1G23980| Symbols: | zinc finger (C3HC4-type RING finger) family protein | chr1:8484868-8485977 REVERS	
JCVI_35729	1.426	highly similar to (651)AT1G56500| Symbols: | haloacid dehalogenase-like hydrolase family protein | chr1:21163440-21170757 FORWA	

RC_EV013364	1.425	no similarity	
JCVI_18395	1.425	weakly similar to (175)AT1G04770| Symbols: | male sterility MS5 family protein | chr1:1336563-1337766 REVERSE no original descrip	
EV089000	1.424	weakly similar to (111)AT1G49540| Symbols: | nucleotide binding | chr1:18337435-18341050 REVERSE [21444]	1.157
JCVI_9788	1.423	weakly similar to (161)AT4G15800| Symbols: RALFL33 | RALFL33 (RALF-LIKE 33) | chr4:8984923-8985273 FORWARD no original	1.551
JCVI_17262	1.423	weakly similar to (188)AT1G32710| Symbols: | cytochrome c oxidase subunit VIb family | chr1:11833093-11833686 FORWARD no ori	
JCVI_24163	1.423	no original description	
EE533139	1.423	moderately similar to (300)AT3G23670| Symbols: PAKRP1L, KINESIN-12B | KINESIN-12B/PAKRP1L; microtubule motor | chr3:8519	
JCVI_14557	1.423	moderately similar to (325)AT1G68890| Symbols: | 2-oxoglutarate decarboxylase/ hydro-lyase/ magnesium ion binding / thiamin pyroph	
JCVI_22845	1.422	weakly similar to (189)AT4G01090| Symbols: | extra-large G-protein-related | chr4:470834-473248 REVERSE no original description	
EX043605	1.422	moderately similar to (397)AT2G44280| Symbols: | similar to lactose permease-related [Arabidopsis thaliana] (TAIR:AT3G60070.1); sin	
EE564191	1.420	weakly similar to (186)AT5G15390| Symbols: | tRNA/rRNA methyltransferase (SpoU) family protein | chr5:4995336-4997258 FORWA	
JCVI_13282	1.419	moderately similar to (491)AT5G15180| Symbols: | peroxidase, putative | chr5:4930564-4932214 FORWARDmoderately similar to (27	1.483
JCVI_11489	1.419	moderately similar to (309)AT1G78110| Symbols: | similar to unknown protein [Arabidopsis thaliana] (TAIR:AT1G22230.1); similar to	
ES915455	1.419	moderately similar to (288)AT2G22790| Symbols: | similar to unknown protein [Arabidopsis thaliana] (TAIR:AT5G67020.1); similar to	
CB686250	1.419	no similarity	
JCVI_41612	1.419	no original description	
JCVI_12728	1.418	weakly similar to (152)AT1G26761| Symbols: | similar to unnamed protein product [Vitis vinifera] (GB:CAO68448.1); contains domain	
JCVI_4651	1.418	moderately similar to (369)AT3G28050| Symbols: | nodulin MtN21 family protein | chr3:10444221-10446453 FORWARD no original de	
JCVI_16543	1.418	weakly similar to (197)AT1G74950| Symbols: JAZ2, TIFY10B | JAZ2/TIFY10B (JASMONATE-ZIM-DOMAIN PROTEIN 2) | chr1:281	
RC_EV011386	1.417	no similarity	
JCVI_20276	1.417	moderately similar to (338)AT5G66070| Symbols: | zinc finger (C3HC4-type RING finger) family protein | chr5:26439149-26440259 FC	
EV223056	1.415	no similarity	
JCVI_24292	1.415	moderately similar to (490)AT1G30160| Symbols: | similar to unknown protein [Arabidopsis thaliana] (TAIR:AT1G05540.1); contains In	
EV092653	1.415	moderately similar to (288)AT1G59620| Symbols: CW9 | CW9; ATP binding | chr1:21906292-21909192 FORWARD [21476] 104 886 8	
EV192481	1.415	weakly similar to (147)AT3G15430| Symbols: | regulator of chromosome condensation (RCC1) family protein | chr3:5209415-5211569 F	
JCVI_17614	1.415	moderately similar to (211)AT1G11530| Symbols: ATCXXS1 | ATCXXS1 (C-TERMINAL CYSTEINE RESIDUE IS CHANGED TO A	
JCVI_41131	1.415	moderately similar to (452)AT3G10450| Symbols: SCPL7 | SCPL7; serine carboxypeptidase | chr3:3249775-3252325 FORWARDweakly	
JCVI_34874	1.414	highly similar to (592)AT1G12990| Symbols: | glycosyl transferase family 17 protein | chr1:4433971-4435550 FORWARD no original de	
JCVI_8732	1.413	moderately similar to (353)AT3G06430| Symbols: EMB2750 | EMB2750 (EMBRYO DEFECTIVE 2750) | chr3:1956664-1958246 REV	
CB686228	1.412	weakly similar to (187)AT5G19500| Symbols: | tryptophan/tyrosine permease family protein | chr5:6579021-6581701 FORWARD [1293	
JCVI_40452	1.412	no original description	
JCVI_19953	1.412	moderately similar to (358)AT3G20300| Symbols: | extracellular ligand-gated ion channel | chr3:7079838-7081815 REVERSE no origina	
JCVI_31510	1.411	moderately similar to (280)AT1G66130| Symbols: | oxidoreductase N-terminal domain-containing protein | chr1:24618703-24620405 FC	
JCVI_37245	1.411	moderately similar to (378)AT3G58160| Symbols: ATXIJ, ATMYOS3, MYA3, XIJ | XIJ (Myosin-like protein XIJ) | chr3:21545774-215	
EV158800	1.411	no similarity	
ES967436	1.410	no similarity	
CX281009	1.410	moderately similar to (227)AT4G25910| Symbols: ATCNFU3, NFU3 | NFU3 (NFU domain protein 3) | chr4:13164137-13165103 FORW	
JCVI_1695	1.409	very weakly similar to (91.7)AT4G23160| Symbols: | protein kinase family protein | chr4:12129496-12134097 FORWARDweakly similar	
EX061195	1.409	moderately similar to (213)AT4G21440| Symbols: ATMYB102, ATM4 | ATM4/ATMYB102 (ARABIDOPSIS MYB-LIKE 102); DNA b	
CD831581	1.408	no similarity	
CX188047	1.408	weakly similar to (161)AT1G47580| Symbols: | lipoyltransferase, putative | chr1:17487193-17488827 FORWARD [16807]	
EV106902	1.408	weakly similar to (127)AT1G50020| Symbols: | similar to unnamed protein product [Vitis vinifera] (GB:CAO49863.1) | chr1:18523812-1	
JCVI_10478	1.407	moderately similar to (331)AT4G18010| Symbols: IP5PII | IP5PII (INOSITOL POLYPHOSPHATE 5-PHOSPHATASE II); inositol-poly	
JCVI_5409	1.407	weakly similar to (178)AT5G11260| Symbols: TED 5, HY5 | HY5 (ELONGATED HYPOCOTYL 5); DNA binding / transcription factor	
JCVI_30076	1.407	highly similar to (713)AT3G07130| Symbols: ATPAP15, PAP15 | ATPAP15/PAP15 (purple acid phosphatase 15); acid phosphatase/ prot	
EV108959	1.406	no similarity	
EV182137	1.406	very weakly similar to (83.6)AT5G20140| Symbols: | SOUL heme-binding family protein | chr5:6799049-6800894 REVERSE [21487]	
JCVI_6057	1.404	weakly similar to (194)AT1G28270| Symbols: RALFL4 | RALFL4 (RALF-LIKE 4) | chr1:9883152-9883484 FORWARD no original des	
EE569939	1.404	no similarity	
JCVI_15193	1.403	highly similar to (562)AT1G20500| Symbols: | 4-coumarate--CoA ligase family / 4-coumaroyl-CoA synthase family | chr1:7101599-710	1.810
JCVI_41689	1.402	moderately similar to (256)AT5G20110| Symbols: | dynein light chain, putative | chr5:6791544-6793255 REVERSE no original descripti	1.517
EV118365	1.402	moderately similar to (218)AT1G29300| Symbols: UNE1 | UNE1 (unfertilized embryo sac 1) | chr1:10248106-10249485 REVERSE [214	1.625
EE561826	1.401	no similarity	
JCVI_13934	1.401	moderately similar to (446)AT4G13250| Symbols: | short-chain dehydrogenase/reductase (SDR) family protein | chr4:7684413-7686687	
EE471256	1.401	weakly similar to (140)AT2G32540| Symbols: CSLB04, ATCSLB4, ATCSLB04 | ATCSLB04 (Cellulose synthase-like B4); transferase/ t	
JCVI_14907	1.401	weakly similar to (157)AT5G37260| Symbols: RVE2, CIR1 | CIR1/RVE2 (CIRCADIAN 1); DNA binding / transcription factor | chr5:14	1.412
EE552941	1.400	no similarity	
ES941335	1.399	moderately similar to (219)AT5G47050| Symbols: | ATP binding / protein binding / shikimate kinase/ zinc ion binding | chr5:19123839-1	
RC_EX040707	1.397	no similarity	
EV092736	1.397	weakly similar to (132)AT2G16365| Symbols: | F-box family protein | chr2:7082022-7086263 FORWARD [21476] 45 496 896	1.955
EV139401	1.396	very weakly similar to (97.4)AT3G57370| Symbols: | transcription factor IIB (TFIIB) family protein | chr3:21237816-21239452 REVERS	
JCVI_12877	1.396	moderately similar to (465)AT5G02830| Symbols: | pentatricopeptide (PPR) repeat-containing protein | chr5:644456-648419 REVERSE	
JCVI_2120	1.396	moderately similar to (385)AT3G47730| Symbols: ATATH1 | ATATH1 (ABC2 homolog 1); ATPase, coupled to transmembrane moveme	
EV193364	1.395	no similarity	
EV085735	1.394	no similarity	
ES264797	1.394	weakly similar to (152)AT5G02020| Symbols: | similar to unknown protein [Arabidopsis thaliana] (TAIR:AT5G59080.1); similar to hyp	1.393
EE533182	1.393	moderately similar to (350)AT4G02050| Symbols: | sugar transporter, putative | chr4:898387-900095 REVERSEmoderately similar to (3	
AM395845	1.392	weakly similar to (120)AT4G22100| Symbols: | glycosyl hydrolase family 1 protein | chr4:11707382-11709944 REVERSE [20346]	
ES993667	1.390	weakly similar to (112)AT2G44190| Symbols: | similar to unknown protein [Arabidopsis thaliana] (TAIR:AT3G60000.2); similar to unna	
JCVI_41486	1.390	moderately similar to (243)AT4G28820| Symbols: | zinc finger (HIT type) family protein | chr4:14230821-14232293 REVERSE no origi	
JCVI_2570	1.388	weakly similar to (144)AT3G45050| Symbols: | heat shock protein binding / unfolded protein binding | chr3:16487008-16488092 FORW	
EV119723	1.388	no similarity	
JCVI_38381	1.388	moderately similar to (265)AT1G69310| Symbols: ATWRKY57, WRKY57 | WRKY57 (WRKY DNA-binding protein 57); transcription	
JCVI_33475	1.388	highly similar to (526)AT2G03750| Symbols: | sulfotransferase family protein | chr2:1147965-1149020 REVERSE no original descriptio	2.688
ES937747	1.386	weakly similar to (181)AT1G58290| Symbols: HEMA1 | HEMA1; glutamyl-tRNA reductase | chr1:21627693-21629716 REVERSEweak	
JCVI_2172	1.386	moderately similar to (379)AT3G14680| Symbols: CYP72A14 | CYP72A14 (cytochrome P450, family 72, subfamily A, polypeptide 14);	
JCVI_7843	1.386	highly similar to (537)AT2G46800| Symbols: ATMTP1, MTP1, ZAT1, ZAT | ATMTP1/MTP1/ZAT1 (ZINC TRANSPORTER OF ARA	
JCVI_7249	1.385	weakly similar to (200)AT3G16570| Symbols: RALFL23 | RALFL23 (RALF-LIKE 23) | chr3:5644754-5645170 FORWARD no original	
EV147259	1.385	no similarity	
JCVI_3183	1.385	highly similar to (889)AT1G57770| Symbols: | amine oxidase family | chr1:21398919-21401800 FORWARDweakly similar to (167)CR	
JCVI_25457	1.385	no original description	
JCVI_7433	1.385	moderately similar to (313)AT2G27680| Symbols: | aldo/keto reductase family protein | chr2:11811058-11813042 REVERSE no original	
JCVI_24218	1.384	moderately similar to (322)AT5G01710| Symbols: | similar to methyltransferase [Arabidopsis thaliana] (TAIR:AT4G24805.1); similar to	

JCVI_36738	1.384	weakly similar to (186)AT1G77120| Symbols: ADH, ATADH, ADH1 | ADH1 (ALCOHOL DEHYDROGENASE 1) | chr1:28980403-28	
JCVI_18041	1.384	moderately similar to (358)AT1G33970| Symbols: | avirulence-responsive protein, putative / avirulence induced gene protein, putative / A	
EV176450	1.384	no similarity	
JCVI_16914	1.383	highly similar to (598)AT4G02280| Symbols: SUS3 | SUS3; UDP-glycosyltransferase/ sucrose synthase/ transferase, transferring glycosyl	
JCVI_22978	1.383	moderately similar to (275)AT5G20830| Symbols: SUS1, ASUS1, ATSUS1 | SUS1 (SUCROSE SYNTHASE 1); UDP-glycosyltransfera	
EV135947	1.382	no similarity	
DN962661	1.382	no similarity	
EE534878	1.381	no similarity	
JCVI_17704	1.380	weakly similar to (150)AT3G55250| Symbols: | similar to unnamed protein product [Vitis vinifera] (GB:CAO14780.1) | chr3:20490279-2	
JCVI_15680	1.380	weakly similar to (193)AT5G67385| Symbols: | signal transducer | chr5:26901980-26904309 FORWARD no original description	
JCVI_7971	1.379	highly similar to (760)AT4G31210| Symbols: | DNA topoisomerase family protein | chr4:15165286-15172540 FORWARD no original de	
JCVI_8270	1.378	moderately similar to (393)AT5G10070| Symbols: | RNase L inhibitor protein-related | chr5:3148685-3150325 REVERSE no original de	
CV432490	1.378	no similarity	
EV047114	1.378	weakly similar to (112)AT4G29810| Symbols: MKK2, MK1, ATMKK2 | ATMKK2 (MAP KINASE KINASE 2) | chr4:14593485-14595	
EX057115	1.378	weakly similar to (198)AT3G45890| Symbols: | similar to unknown protein [Arabidopsis thaliana] (TAIR:AT1G13770.1); similar to unna	
EV209478	1.374	no similarity	
JCVI_9641	1.374	moderately similar to (395)AT2G12190| Symbols: | cytochrome P450, putative | chr2:4898889-4900427 REVERSEmoderately similar to	
EV056789	1.374	very weakly similar to (98.6)AT5G41010| Symbols: | DNA-directed RNA polymerases I, II, and III 7 kDa subunit, putative | chr5:164412	
JCVI_6728	1.374	very weakly similar to (98.2)AT1G49760| Symbols: PAB8 | PAB8 (POLY(A) BINDING PROTEIN 8); RNA binding / translation initiatio	
ES917488	1.373	weakly similar to (123)AT3G06190| Symbols: ATBPM2 | ATBPM2; protein binding | chr3:1874583-1876581 REVERSE [15718]	
ES931175	1.372	no similarity	
JCVI_30821	1.372	moderately similar to (397)AT5G27330| Symbols: | similar to unknown protein [Arabidopsis thaliana] (TAIR:AT3G05130.1); similar to	
EV140116	1.371	no similarity	
JCVI_27008	1.371	moderately similar to (204)AT2G22870| Symbols: EMB2001 | EMB2001 (EMBRYO DEFECTIVE 2001); GTP binding | chr2:9746556-9	
JCVI_28832	1.370	moderately similar to (375)AT1G80880| Symbols: | pentatricopeptide (PPR) repeat-containing protein | chr1:30400086-30401813 REVE	
JCVI_7117	1.370	no original description	
EV135593	1.370	no similarity	1.799
JCVI_34535	1.370	no original description	
EV076567	1.369	weakly similar to (162)AT4G16690| Symbols: | esterase/lipase/thioesterase family protein | chr4:9392427-9393446 REVERSE [21443]	
JCVI_12500	1.368	moderately similar to (212)AT5G08410| Symbols: FTRA2 | FTRA2 (ferredoxin/thioredoxin reductase subunit A (variable subunit) 2); fer	
JCVI_1451	1.367	moderately similar to (472)AT5G18650| Symbols: | zinc finger (C3HC4-type RING finger) family protein | chr5:6218262-6220376 FOR	
RC_EX119510	1.366	no similarity	
AM396151	1.366	weakly similar to (135)AT3G02990| Symbols: HSFA1E, ATHSFA1E | ATHSFA1E (Arabidopsis thaliana heat shock transcription factor	
ES265177	1.366	no similarity	
EV071876	1.366	moderately similar to (292)AT5G29000| Symbols: | myb family transcription factor | chr5:11023017-11024233 REVERSE [21443]	
DY023260	1.366	weakly similar to (172)AT1G68410| Symbols: | protein phosphatase 2C-related / PP2C-related | chr1:25653925-25655918 REVERSE [1	
JCVI_33246	1.365	highly similar to (526)AT2G02450| Symbols: ANAC034, ANAC035 | ANAC034/ANAC035 (Arabidopsis NAC domain containing protei	
CD841157	1.365	weakly similar to (138)AT4G30470| Symbols: | cinnamoyl-CoA reductase-related | chr4:14894269-14896512 FORWARD [13982]	
JCVI_985	1.365	highly similar to (514)AT1G80760| Symbols: NIP6;1, NLM7, NIP6 | NIP6;1 (NOD26-like intrinsic protein 6;1); water channel | chr1:303	
DN963563	1.364	no similarity	
JCVI_41966	1.364	very weakly similar to (88.2)AT2G39170| Symbols: | similar to unnamed protein product [Vitis vinifera] (GB:CAO68899.1); contains do	
JCVI_28850	1.363	very weakly similar to (80.1)AT3G57062| Symbols: | similar to hypothetical protein MtrDRAFT_AC153128g25v2 [Medicago truncatula]	
EV010880	1.363	no similarity	
EV040761	1.362	moderately similar to (228)AT3G23670| Symbols: PAKRP1L, KINESIN-12B | KINESIN-12B/PAKRP1L; microtubule motor | chr3:8519	
EE446340	1.362	weakly similar to (180)AT5G19470| Symbols: ATNUDT24 | ATNUDT24 (Arabidopsis thaliana Nudix hydrolase homolog 24); hydrolase	
JCVI_30315	1.361	moderately similar to (350)AT3G54740| Symbols: | similar to unknown protein [Arabidopsis thaliana] (TAIR:AT3G11850.1); similar to	
JCVI_33675	1.359	weakly similar to (138)AT1G27300| Symbols: | similar to unnamed protein product [Vitis vinifera] (GB:CAO66149.1) | chr1:9483311-9	
JCVI_28793	1.359	moderately similar to (430)AT3G06510| Symbols: SFR2 | SFR2 (SENSITIVE TO FREEZING 2) | chr3:2016456-2019539 FORWARD n	
EV075832	1.358	weakly similar to (149)AT4G22970| Symbols: AESP | AESP (ARABIDOPSIS HOMOLOG OF SEPARASE); peptidase | chr4:12033714-	
EE509065	1.357	moderately similar to (310)AT1G28350| Symbols: | ATP binding / aminoacyl-tRNA ligase | chr1:9944470-9949564 FORWARD [15718]	
EV098240	1.356	weakly similar to (115)AT2G29310| Symbols: | tropinone reductase, putative / tropine dehydrogenase, putative | chr2:12597145-1259833	1.467
EV167383	1.356	weakly similar to (190)AT3G57180| Symbols: | GTP binding | chr3:21174642-21176985 REVERSE [21486] 82 607 607	
EL592310	1.355	no similarity	
JCVI_8592	1.355	very weakly similar to (93.6)AT5G54585| Symbols: | unknown protein | chr5:22192914-22193735 FORWARD no original description	2.995
EE563126	1.355	no similarity	
CX191309	1.353	weakly similar to (140)AT3G22440| Symbols: | hydroxyproline-rich glycoprotein family protein | chr3:7959861-7961893 FORWARD [1	
JCVI_4785	1.352	moderately similar to (337)AT4G25910| Symbols: ATCNFU3, NFU3 | NFU3 (NFU domain protein 3) | chr4:13164137-13165103 FORW	
BQ704527	1.351	no similarity	
JCVI_81	1.351	moderately similar to (409)AT1G80840| Symbols: ATWRKY40, WRKY40 | WRKY40 (WRKY DNA-binding protein 40); transcription	1.783
JCVI_4100	1.351	moderately similar to (345)AT2G43235| Symbols: | sugar porter | chr2:17975987-17977899 REVERSE no original description	
EE532415	1.350	weakly similar to (169)AT2G41705| Symbols: | camphor resistance CrcB family protein | chr2:17405161-17406988 FORWARD [20175]	
JCVI_13397	1.350	moderately similar to (221)AT3G22550| Symbols: | senescence-associated protein-related | chr3:7991834-7992812 REVERSE no origina	1.816
EE440043	1.350	no similarity	
JCVI_27775	1.349	moderately similar to (201)AT3G52920| Symbols: | hematopoietin/interferon-class (D200-domain) cytokine receptor binding | chr3:1963	
ES913410	1.348	no similarity	
EV075063	1.347	weakly similar to (136)AT1G67510| Symbols: | leucine-rich repeat family protein | chr1:25301140-25303847 REVERSE [21443]	
ES968868	1.347	weakly similar to (184)AT5G25840| Symbols: | similar to unknown protein [Arabidopsis thaliana] (TAIR:AT1G79770.1); similar to unna	1.906
EX032230	1.346	no similarity	
JCVI_9453	1.345	moderately similar to (204)AT3G02310| Symbols: AGL4, SEP2 | SEP2 (SEPALLATA2); DNA binding / transcription factor | chr3:46456	
JCVI_3494	1.345	moderately similar to (236)AT5G24314| Symbols: PDE225, PTAC7 | PDE225/PTAC7 (PIGMENT DEFECTIVE 225) | chr5:8277753-82	
AM057156	1.345	no similarity	
JCVI_8704	1.344	highly similar to (585)AT5G63160| Symbols: BT1 | BT1 (BTB and TAZ domain protein 1); protein binding / transcription regulator | chr5	
JCVI_3182	1.343	weakly similar to (150)AT4G33300| Symbols: ADR1-L1 | ADR1-L1 (ADR1-LIKE 1); ATP binding / protein binding | chr4:16051166-16	
JCVI_14744	1.342	moderately similar to (373)AT1G09795| Symbols: HISN1B, ATATP-PRT2 | ATATP-PRT2 (ATP PHOSPHORIBOSYL TRANSFERASE	
JCVI_39164	1.341	no original description	
EV181486	1.341	moderately similar to (327)AT4G23990| Symbols: CSLG3, ATCSLG3 | ATCSLG3 (Cellulose synthase-like G3); transferase/ transferase,	1.253
JCVI_25822	1.341	moderately similar to (228)AT1G31460| Symbols: | similar to unknown protein [Arabidopsis thaliana] (TAIR:AT1G23270.1); similar to	
JCVI_11796	1.340	moderately similar to (271)AT5G19500| Symbols: | tryptophan/tyrosine permease family protein | chr5:6579021-6581701 FORWARD n	
JCVI_31186	1.339	no original description	
JCVI_35780	1.339	moderately similar to (211)AT5G10380| Symbols: | zinc finger (C3HC4-type RING finger) family protein | chr5:3267820-3268725 FOR	
EE569276	1.338	moderately similar to (422)AT2G26850| Symbols: | F-box family protein | chr2:11456447-11457967 REVERSE [20191] 43 821 821	
RC_EH427440	1.337	no similarity	
JCVI_8801	1.336	weakly similar to (179)AT5G64850| Symbols: | similar to unknown protein [Arabidopsis thaliana] (TAIR:AT5G09960.1); similar to 80C	

JCVI_2868	1.335	weakly similar to (168)AT5G23440| Symbols: FTRA1 | FTRA1 (ferredoxin/thioredoxin reductase subunit A (variable subunit) 1); ferredo	
RC_ES937978	1.334	no similarity	
EV084300	1.334	weakly similar to (152)AT3G59670| Symbols: | similar to unknown protein [Arabidopsis thaliana] (TAIR:AT4G37440.2); similar to unkn	
JCVI_1061	1.334	moderately similar to (330)AT1G80050| Symbols: ATAPT2, APT2 | APT2 (ADENINE PHOSPHORIBOSYL TRANSFERASE 2); adeni	
JCVI_8219	1.333	moderately similar to (254)AT5G66900| Symbols: | disease resistance protein (CC-NBS-LRR class), putative | chr5:26732157-26734983	
JCVI_26674	1.333	moderately similar to (275)AT3G61080| Symbols: | fructosamine kinase family protein | chr3:22618127-22619858 FORWARD no origin	
JCVI_18313	1.333	moderately similar to (452)AT2G37050| Symbols: | similar to leucine-rich repeat family protein / protein kinase family protein [Arabidop	1.672
BQ704168	1.332	no similarity	
EV037401	1.331	weakly similar to (182)AT2G43235| Symbols: | sugar porter | chr2:17975987-17977899 REVERSE [21441] 1 615 629	
ES922541	1.330	moderately similar to (208)AT5G16560| Symbols: KAN1, KAN | KAN (KANADI); transcription factor | chr5:5407368-5411095 REVER	
JCVI_31308	1.329	weakly similar to (124)AT1G29530| Symbols: | similar to unknown protein [Arabidopsis thaliana] (TAIR:AT2G34310.1); similar to unkn	
EV142036	1.327	no similarity	
JCVI_13418	1.326	highly similar to (818)AT5G48150| Symbols: PAT1 | PAT1 (PHYTOCHROME A SIGNAL TRANSDUCTION 1); transcription factor | c	
RC_AM385827	1.325	no similarity	
JCVI_28516	1.325	no original description	
EE417681	1.325	no similarity	
JCVI_413	1.325	moderately similar to (287)AT3G56400| Symbols: ATWRKY70, WRKY70 | WRKY70 (WRKY DNA-binding protein 70); transcription	
JCVI_10298	1.325	moderately similar to (246)AT1G76110| Symbols: | high mobility group (HMG1/2) family protein / ARID/BRIGHT DNA-binding domai	
JCVI_33247	1.325	moderately similar to (201)AT1G10657| Symbols: | similar to unknown protein [Arabidopsis thaliana] (TAIR:AT3G55240.1); similar to	
JCVI_19821	1.325	weakly similar to (158)AT1G67030| Symbols: ZFP6 | ZFP6 (ZINC FINGER PROTEIN 6); nucleic acid binding / transcription factor/ zin	
JCVI_16741	1.324	moderately similar to (218)AT5G57350| Symbols: AHA3 | AHA3 (Arabidopsis H(+)-ATPase 3); ATPase | chr5:23248434-23253607 RE	
JCVI_14157	1.324	moderately similar to (325)AT2G01180| Symbols: PAP1, LPP1, ATLPP1, ATPAP1 | ATPAP1 (PHOSPHATIDIC ACID PHOSPHATASE	
JCVI_14659	1.323	moderately similar to (296)AT1G56650| Symbols: PAP1, ATMYB75, MYB75, SIAA1 | PAP1 (PRODUCTION OF ANTHOCYANIN PI	
JCVI_2836	1.322	highly similar to (688)AT5G19220| Symbols: APL1, ADG2 | ADG2 (ADPG PYROPHOSPHORYLASE 2); glucose-1-phosphate adenyly	1.467
JCVI_30612	1.322	highly similar to (858)AT3G14630| Symbols: CYP72A9 | CYP72A9 (cytochrome P450, family 72, subfamily A, polypeptide 9); oxygen b	
AM059821	1.322	no similarity	
EV206429	1.321	no similarity	
JCVI_3083	1.320	moderately similar to (253)AT1G16320| Symbols: | similar to unknown protein [Arabidopsis thaliana] (TAIR:AT1G79510.2); similar to	
JCVI_39559	1.320	no original description	1.709
JCVI_15994	1.319	highly similar to (624)AT2G41670| Symbols: | GTP-binding family protein | chr2:17381196-17383255 FORWARD no original descripti	
JCVI_16626	1.318	weakly similar to (124)AT1G28540| Symbols: | similar to Os06g0524500 [Oryza sativa (japonica cultivar-group)] (GB:NP_001057759.1	
EV148049	1.318	no similarity	
JCVI_35838	1.318	no original description	
AM059112	1.317	no similarity	
JCVI_36796	1.317	highly similar to (898)AT1G31480| Symbols: SGR2 | SGR2 (SHOOT GRAVITROPISM 2) | chr1:11266206-11271508 FORWARD no o	
JCVI_31825	1.317	moderately similar to (381)AT4G26220| Symbols: | caffeoyl-CoA 3-O-methyltransferase, putative | chr4:13284188-13285155 FORWAR	
JCVI_5679	1.316	moderately similar to (498)AT1G48460| Symbols: | similar to unknown protein [Arabidopsis thaliana] (TAIR:AT5G63040.2); similar to	
CN726984	1.316	no similarity	
EE417743	1.316	weakly similar to (105)AT5G13650| Symbols: | elongation factor family protein | chr5:4397824-4402367 FORWARD [20146]	
JCVI_13970	1.315	highly similar to (678)AT1G07870| Symbols: | protein kinase family protein | chr1:2429930-2431840 REVERSEmoderately similar to (2	
EV209377	1.314	no similarity	
JCVI_28061	1.311	weakly similar to (199)AT1G11020| Symbols: | zinc finger (C3HC4-type RING finger) family protein | chr1:3676968-3678350 FORWA	
ES986386	1.311	moderately similar to (201)AT1G04920| Symbols: ATSPS3F | ATSPS3F (sucrose phosphate synthase 3F); sucrose-phosphate synthase/ tr	
JCVI_7936	1.311	weakly similar to (194)AT1G29690| Symbols: CAD1 | CAD1 (CONSTITUTIVELY ACTIVATED CELL DEATH 1) | chr1:10379296-10	
EX066713	1.310	weakly similar to (191)AT4G34680| Symbols: | GATA transcription factor 3, putative (GATA-3) | chr4:16553705-16554615 FORWARD	
DY001903	1.310	no similarity	
CD834806	1.309	weakly similar to (162)AT5G52990| Symbols: | vesicle-associated membrane protein-related | chr5:21501414-21502232 FORWARD [13	
EV139626	1.307	no similarity	
EV201808	1.307	moderately similar to (267)AT5G24120| Symbols: SIG5, SIGE | SIGE (RNA polymerase sigma subunit E); DNA binding / DNA-directed	
EV015083	1.306	no similarity	
CN735512	1.305	weakly similar to (167)AT1G69380| Symbols: | similar to unknown protein [Arabidopsis thaliana] (TAIR:AT5G13610.1); similar to unna	
JCVI_14434	1.305	weakly similar to (200)AT5G58760| Symbols: DDB2 | DDB2 (DAMAGED DNA-BINDING 2); nucleotide binding | chr5:23747967-237	
JCVI_2173	1.305	moderately similar to (406)AT5G08610| Symbols: | DEAD box RNA helicase (RH26) | chr5:2790342-2794060 FORWARD no original	
JCVI_36713	1.304	highly similar to (819)AT3G47090| Symbols: | leucine-rich repeat transmembrane protein kinase, putative | chr3:17352497-17355630 RE	
EX130945	1.303	weakly similar to (153)AT5G24430| Symbols: | calcium-dependent protein kinase, putative / CDPK, putative | chr5:8339393-8342916 R	
EV185303	1.301	moderately similar to (246)AT5G43870| Symbols: | similar to unknown protein [Arabidopsis thaliana] (TAIR:AT4G14740.1); similar to	1.118
CD827448	1.299	no similarity	
JCVI_29041	1.296	moderately similar to (315)AT2G44280| Symbols: | similar to lactose permease-related [Arabidopsis thaliana] (TAIR:AT3G60070.1); sin	
CX192117	1.294	no similarity	
JCVI_3911	1.293	moderately similar to (464)AT4G25700| Symbols: B1, CHY1, BETA-OHASE 1 | BETA-OHASE 1 (BETA-HYDROXYLASE 1) | chr4:1	
JCVI_10808	1.293	moderately similar to (255)AT4G18820| Symbols: | ATP binding / DNA binding / DNA-directed DNA polymerase/ nucleoside-triphosph	1.708
EV193644	1.293	weakly similar to (119)AT5G49730| Symbols: ATFRO6, FRO6 | ATFRO6/FRO6 (FERRIC REDUCTION OXIDASE 6); ferric-chelate re	
EV131436	1.293	no similarity	
JCVI_12423	1.292	no original description	
JCVI_4242	1.291	moderately similar to (302)AT4G15540| Symbols: | nodulin-related | chr4:8873392-8875184 FORWARD no original description	
RC_EE562412	1.291	no similarity	
JCVI_37200	1.291	highly similar to (616)AT3G14090| Symbols: ATEXO70D3 | ATEXO70D3 (exocyst subunit EXO70 family protein D3); protein binding	
JCVI_17677	1.290	moderately similar to (392)AT4G22780| Symbols: ACR7 | ACR7 (ACT Domain Repeat 7) | chr4:11968707-11970967 REVERSE no orig	
JCVI_20615	1.290	moderately similar to (349)AT1G79510| Symbols: | similar to unknown protein [Arabidopsis thaliana] (TAIR:AT1G16320.1); similar to	
ES913705	1.290	weakly similar to (160)AT4G36150| Symbols: | disease resistance protein (TIR-NBS-LRR class), putative | chr4:17104779-17108714 FC	
JCVI_16075	1.290	moderately similar to (283)AT2G46340| Symbols: SPA1 | SPA1 (SUPPRESSOR OF PHYA-105 1); signal transducer | chr2:19029645-19	
JCVI_4280	1.290	no original description	1.611
JCVI_40806	1.290	no original description	
EV102134	1.288	moderately similar to (201)AT1G55340| Symbols: | similar to unknown protein [Arabidopsis thaliana] (TAIR:AT3G03880.1); similar to	
JCVI_10872	1.288	weakly similar to (160)AT3G03920| Symbols: | Gar1 RNA-binding region family protein | chr3:1009130-1010386 REVERSE no origina	
RC_EX031510	1.288	no similarity	1.704
JCVI_38045	1.288	no original description	
JCVI_2171	1.288	moderately similar to (210)AT5G08130| Symbols: BIM1 | BIM1 (BES1-interacting Myc-like protein 1) | chr5:2606656-2609572 REVER	
EV036583	1.285	weakly similar to (140)AT3G27210| Symbols: | Identical to Uncharacterized protein At3g27210 (Y-2) [Arabidopsis Thaliana] (GB:Q9LK	
JCVI_18026	1.284	weakly similar to (168)AT4G32190| Symbols: | centromeric protein-related | chr4:15545058-15547695 FORWARD no original descripti	
JCVI_14865	1.283	moderately similar to (334)AT4G33770| Symbols: | inositol 1,3,4-trisphosphate 5/6-kinase family protein | chr4:16193591-16195240 RE	
ES949525	1.283	weakly similar to (183)AT3G06510| Symbols: SFR2 | SFR2 (SENSITIVE TO FREEZING 2) | chr3:2016456-2019539 FORWARD [2139	
JCVI_4413	1.283	weakly similar to (140)AT2G38230| Symbols: ATPDX1.1 | ATPDX1.1 (PYRIDOXINE BIOSYNTHESIS 1.1); protein heterodimerizatio	

JCVI_33105	1.282	moderately similar to (490)AT1G69800| Symbols: | CBS domain-containing protein | chr1:26278079-26279988 REVERSE no original de	
JCVI_27708	1.281	moderately similar to (467)AT1G55850| Symbols: CSLE1, ATCSLE1 | ATCSLE1 (Cellulose synthase-like E1); cellulose synthase/ transf	
CO750216	1.281	no similarity	
JCVI_8488	1.281	moderately similar to (394)AT3G14690| Symbols: CYP72A15 | CYP72A15 (cytochrome P450, family 72, subfamily A, polypeptide 15);	
EV202868	1.281	moderately similar to (273)AT2G33860| Symbols: ARF3, ETT | ETT (ETTIN); transcription factor | chr2:14332520-14335689 REVERSE	
EH430438	1.280	weakly similar to (102)AT4G20020| Symbols: | similar to unknown protein [Arabidopsis thaliana] (TAIR:AT5G44780.1); similar to Os1	
JCVI_5562	1.280	weakly similar to (139)AT1G29530| Symbols: | similar to unknown protein [Arabidopsis thaliana] (TAIR:AT2G34310.1); similar to unkn	
JCVI_15958	1.280	moderately similar to (313)AT3G01440| Symbols: | oxygen evolving enhancer 3 (PsbQ) family protein | chr3:168485-169414 FORWARD	
JCVI_13933	1.280	moderately similar to (437)AT3G01060| Symbols: | similar to unnamed protein product [Vitis vinifera] (GB:CAO15045.1); similar to unl	
EX099556	1.279	moderately similar to (349)AT3G23410| Symbols: | alcohol oxidase-related | chr3:8382867-8386031 FORWARD [21825]	
JCVI_10804	1.279	no original description	
DN961453	1.279	weakly similar to (130)AT1G11650| Symbols: ATRBP45B | ATRBP45B; RNA binding | chr1:3914895-3917301 FORWARD [17359]	
JCVI_22005	1.278	weakly similar to (148)AT4G30993| Symbols: | similar to hypothetical protein [Vitis vinifera] (GB:CAN67945.1); contains domain Meta	
ES948897	1.278	very weakly similar to (97.8)AT5G65300| Symbols: | unknown protein | chr5:26112492-26112944 REVERSE [21393]	
JCVI_15672	1.278	weakly similar to (166)AT3G49601| Symbols: | similar to unknown protein [Arabidopsis thaliana] (TAIR:AT4G37820.1); similar to unna	1.258
JCVI_42559	1.278	no original description	
EE539675	1.277	weakly similar to (179)AT3G53470| Symbols: | similar to unnamed protein product [Vitis vinifera] (GB:CAO18045.1) | chr3:19833791-1	
JCVI_20801	1.277	no original description	1.436
EV132438	1.277	no similarity	1.882
JCVI_11869	1.276	moderately similar to (284)AT5G65400| Symbols: | similar to unknown protein [Arabidopsis thaliana] (TAIR:AT4G24380.1); similar to u	1.131
JCVI_15073	1.275	moderately similar to (258)AT3G62910| Symbols: APG3 | APG3 (ALBINO AND PALE GREEN); translation release factor | chr3:23268	
JCVI_21970	1.275	weakly similar to (196)AT1G49890| Symbols: | similar to unknown protein [Arabidopsis thaliana] (TAIR:AT3G19570.1); similar to unkn	
JCVI_14017	1.275	very weakly similar to (85.1)AT3G09300| Symbols: | oxysterol-binding family protein | chr3:2858074-2860468 FORWARD no original d	
JCVI_15218	1.274	moderately similar to (332)AT3G10420| Symbols: | sporulation protein-related | chr3:3239312-3241576 FORWARD no original descript	
EX057896	1.274	moderately similar to (225)AT3G15354| Symbols: SPA3 | SPA3 (SPA1-RELATED 3); signal transducer | chr3:5169334-5172487 REVER	
DY012811	1.272	no similarity	
JCVI_35571	1.271	weakly similar to (164)AT1G23550| Symbols: SRO2 | SRO2 (SIMILAR TO RCD ONE 2); NAD+ ADP-ribosyltransferase | chr1:835090	
JCVI_20661	1.271	moderately similar to (283)AT3G48500| Symbols: PDE312, PTAC10 | PDE312/PTAC10 (PIGMENT DEFECTIVE 312); RNA binding |	
EE455964	1.269	very weakly similar to (89.4)AT5G44005| Symbols: | unknown protein | chr5:17722436-17722720 REVERSE [20178]	
EV068746	1.268	moderately similar to (235)AT2G20580| Symbols: RPN1A, AtRPN1a | AtRPN1a/RPN1A (26S proteasome regulatory subunit S2 1A); bir	
JCVI_37764	1.267	moderately similar to (257)AT1G06070| Symbols: | bZIP transcription factor, putative (bZIP69) | chr1:1835200-1837115 REVERSEweal	
EX051415	1.267	weakly similar to (171)AT4G15885| Symbols: | kinesin motor protein-related | chr4:9016466-9016993 REVERSE [21812]	
JCVI_27458	1.265	moderately similar to (396)AT5G08620| Symbols: STRS2 | STRS2 (STRESS RESPONSE SUPPRESSOR 2); ATP-dependent helicase | c	
EH428672	1.265	no similarity	
ES994891	1.265	no similarity	
JCVI_36678	1.264	no original description	
EX090991	1.264	weakly similar to (109)AT1G48175| Symbols: EMB2191 | EMB2191 (EMBRYO DEFECTIVE 2191); catalytic/ hydrolase/ zinc ion bind	
EX056312	1.263	weakly similar to (196)AT4G32770| Symbols: ATSDX1, VTE1 | VTE1 (VITAMIN E DEFICIENT 1) | chr4:15804986-15807795 FORW	
JCVI_7029	1.262	moderately similar to (310)AT5G53490| Symbols: | thylakoid lumenal 17.4 kDa protein, chloroplast | chr5:21740714-21741847 REVER	1.452
EE473850	1.262	weakly similar to (168)AT4G38060| Symbols: | similar to unknown protein [Arabidopsis thaliana] (TAIR:AT5G65480.1); similar to unkn	
EV089440	1.262	moderately similar to (206)AT5G61820| Symbols: | similar to MtN19-like protein [Pisum sativum] (GB:AAU14999.2); contains InterPro	
JCVI_38007	1.262	moderately similar to (279)AT4G31115| Symbols: | similar to unknown protein [Arabidopsis thaliana] (TAIR:AT5G04440.1); similar to p	
DN962534	1.262	no similarity	1.546
BG543306	1.261	weakly similar to (147)AT2G33430| Symbols: | plastid developmental protein DAG, putative | chr2:14169808-14171805 FORWARD [8	
JCVI_18962	1.259	weakly similar to (167)AT1G22160| Symbols: | senescence-associated protein-related | chr1:7823227-7823763 FORWARD no original d	
JCVI_28653	1.258	moderately similar to (393)AT2G01690| Symbols: | binding | chr2:309143-313498 REVERSE no original description	
CX195839	1.257	moderately similar to (438)AT1G74240| Symbols: | mitochondrial substrate carrier family protein | chr1:27921098-27923648 FORWARD	
ES944459	1.257	moderately similar to (236)AT2G01480| Symbols: | similar to unknown protein [Arabidopsis thaliana] (TAIR:AT1G14970.1); similar to	1.506
DY001451	1.257	weakly similar to (129)AT4G24480| Symbols: | serine/threonine protein kinase, putative | chr4:12650420-12654765 FORWARD [18968	1.467
JCVI_9625	1.256	moderately similar to (326)AT2G22680| Symbols: | zinc finger (C3HC4-type RING finger) family protein | chr2:9652513-9654564 FOR	
JCVI_26594	1.256	very weakly similar to (97.4)AT5G27320| Symbols: ATGID1C, GID1C | ATGID1C/GID1C (GA INSENSITIVE DWARF1C); hydrolase |	
JCVI_37296	1.256	moderately similar to (224)AT1G24400| Symbols: AATL2, LHT2 | LHT2 (LYSINE HISTIDINE TRANSPORTER 2); amino acid transm	1.777
EE453092	1.256	weakly similar to (103)AT5G02840| Symbols: LCL1 | LCL1 (LHY/CCA1-LIKE 1); DNA binding / transcription factor | chr5:648792-651	
JCVI_19277	1.255	moderately similar to (265)AT1G67480| Symbols: | kelch repeat-containing F-box family protein | chr1:25280957-25282192 FORWARD	
EV091175	1.255	no similarity	
JCVI_35468	1.255	no original description	
JCVI_28692	1.254	weakly similar to (181)AT5G51720| Symbols: | similar to Os07g0467200 [Oryza sativa (japonica cultivar-group)] (GB:NP_001059590.1	
JCVI_38581	1.254	very weakly similar to (95.5)AT5G07530| Symbols: ATGRP17, ATGRP-7, GRP17 | GRP17 (Glycine rich protein 17) | chr5:2382630-238	0.523
JCVI_7806	1.254	highly similar to (685)AT1G63180| Symbols: UGE3 | UGE3 (UDP-D-GLUCOSE/UDP-D-GALACTOSE 4-EPIMERASE 3); UDP-gluco	2.025
JCVI_19728	1.254	moderately similar to (281)AT1G29690| Symbols: CAD1 | CAD1 (CONSTITUTIVELY ACTIVATED CELL DEATH 1) | chr1:1037929	
JCVI_17250	1.253	moderately similar to (291)AT1G12370| Symbols: UVR2, PHR1 | PHR1 (PHOTOLYASE 1) | chr1:4206498-4208840 REVERSE no orig	
EV194149	1.253	moderately similar to (214)AT5G62430| Symbols: CDF1 | CDF1 (CYCLING DOF FACTOR 1); DNA binding / protein binding / transcri	
EV181448	1.252	moderately similar to (206)AT1G75020| Symbols: LPAT4 | LPAT4; acyltransferase | chr1:28175440-28177018 FORWARD [21487] 1 42	
JCVI_10494	1.252	moderately similar to (423)AT5G52450| Symbols: | MATE efflux protein-related | chr5:21306268-21308975 REVERSE no original desc	
RC_ES958250	1.251	no similarity	
EV105960	1.250	no similarity	
AM059731	1.250	moderately similar to (253)AT3G21760| Symbols: | UDP-glucoronosyl/UDP-glucosyl transferase family protein | chr3:7667106-7668563	
JCVI_5602	1.249	weakly similar to (170)AT2G04700| Symbols: | ferredoxin thioredoxin reductase catalytic beta chain family protein | chr2:1646958-1648	
JCVI_38340	1.249	very weakly similar to (92.8)AT5G48990| Symbols: | kelch repeat-containing F-box family protein | chr5:19879566-19880684 FORWAR	
JCVI_9135	1.248	weakly similar to (142)AT2G35060| Symbols: KUP11 | KUP11 (K+ uptake permease 11); potassium ion transmembrane transporter | chr2	
CX192609	1.247	weakly similar to (156)AT1G78140| Symbols: | methyltransferase-related | chr1:29406830-29408771 REVERSE [16807]	1.306
JCVI_19933	1.247	weakly similar to (165)AT3G52070| Symbols: | similar to unnamed protein product [Vitis vinifera] (GB:CAO40812.1) | chr3:19323731-1	
JCVI_17663	1.246	moderately similar to (204)AT2G34300| Symbols: | dehydration-responsive protein-related | chr2:14480995-14483890 REVERSE no orig	
JCVI_10431	1.246	highly similar to (808)AT5G54960| Symbols: PDC2 | PDC2 (PYRUVATE DECARBOXYLASE-2); pyruvate decarboxylase | chr5:22328	
EX022379	1.246	no similarity	1.400
JCVI_27830	1.245	no original description	
JCVI_31713	1.244	moderately similar to (337)AT4G03070| Symbols: AOP, AOP1.1, AOP1 | AOP1 (2-oxoglutarate?dependent dioxygenase 1.1); oxidoredu	1.824
EV191225	1.244	weakly similar to (144)AT1G20640| Symbols: | RWP-RK domain-containing protein | chr1:7155191-7157976 FORWARD [21489] 22 8	
JCVI_28169	1.244	weakly similar to (179)AT5G26594| Symbols: ARR24 | ARR24 (ARABIDOPSIS RESPONSE REGULATOR 24); two-component respon	
JCVI_23609	1.243	moderately similar to (253)AT5G60540| Symbols: EMB2407, ATPDX2, PDX2 | ATPDX2/EMB2407/PDX2 (PYRIDOXINE BIOSYNT	
JCVI_38952	1.243	moderately similar to (481)AT5G18820| Symbols: EMB3007 | EMB3007 (EMBRYO DEFECTIVE 3007); ATP binding / protein binding	
JCVI_35538	1.242	moderately similar to (352)AT5G60210| Symbols: | cytoplasmic linker protein-related | chr5:24260755-24262858 REVERSE no original	
JCVI_10958	1.241	moderately similar to (286)AT2G37450| Symbols: | nodulin MtN21 family protein | chr2:15729907-15731930 REVERSE no original des	

JCVI_7352	1.240	moderately similar to (258)AT4G03150| Symbols: | similar to unnamed protein product [Vitis vinifera] (GB:CAO22424.1) | chr4:139373	
JCVI_20992	1.236	weakly similar to (196)AT2G24280| Symbols: | serine carboxypeptidase S28 family protein | chr2:10341702-10344007 FORWARD no o	
EV193577	1.236	weakly similar to (190)AT1G42550| Symbols: PMI1 | PMI1 (PLASTID MOVEMENT IMPAIRED1) | chr1:15979976-15982174 FORWA	
ES950166	1.234	no similarity	
JCVI_38789	1.234	highly similar to (540)AT5G10170| Symbols: | inositol-3-phosphate synthase, putative / myo-inositol-1-phosphate synthase, putative / M	
JCVI_13684	1.234	weakly similar to (139)AT5G35970| Symbols: | DNA-binding protein, putative | chr5:14136290-14140308 REVERSE no original descrip	
CO750032	1.233	weakly similar to (187)AT1G74240| Symbols: | mitochondrial substrate carrier family protein | chr1:27921098-27923648 FORWARD [1	
JCVI_15644	1.232	highly similar to (660)AT1G44446| Symbols: ATCAO, CAO, CH1 | CH1 (CHLOROPHYLL B BIOSYNTHESIS); chlorophyllide a oxyg	1.579
JCVI_33968	1.231	moderately similar to (441)AT5G53080| Symbols: | kinesin light chain-related | chr5:21537850-21540384 FORWARD no original descri	
JCVI_22038	1.231	moderately similar to (421)AT5G51130| Symbols: | similar to unnamed protein product [Vitis vinifera] (GB:CAO65480.1); contains Inte	
EV015405	1.230	no similarity	
JCVI_699	1.228	moderately similar to (372)AT1G72150| Symbols: PATL1 | PATL1 (PATELLIN 1); transporter | chr1:27152220-27154314 FORWARD r	
JCVI_7811	1.225	moderately similar to (349)AT4G32770| Symbols: ATSDX1, VTE1 | VTE1 (VITAMIN E DEFICIENT 1) | chr4:15804986-15807795 FO	1.313
AM385827	1.224	no similarity	
JCVI_38390	1.224	moderately similar to (380)AT1G22850| Symbols: | similar to unknown protein [Arabidopsis thaliana] (TAIR:AT1G03260.1); similar to u	
JCVI_41430	1.224	weakly similar to (186)AT1G32870| Symbols: ANAC013 | ANAC013 (Arabidopsis NAC domain containing protein 13) | chr1:11911725-	
JCVI_19366	1.222	moderately similar to (307)AT5G57960| Symbols: | GTP-binding family protein | chr5:23480359-23483707 REVERSE no original descr	1.229
JCVI_18163	1.222	moderately similar to (350)AT5G57840| Symbols: | transferase family protein | chr5:23450171-23452387 REVERSEweakly similar to (	
JCVI_3516	1.221	moderately similar to (234)AT2G36835| Symbols: | similar to hypothetical protein [Vitis vinifera] (GB:CAN81061.1) | chr2:15456918-1	
ES978120	1.221	no similarity	
EV110266	1.220	no similarity	
JCVI_42226	1.220	moderately similar to (216)AT5G11020| Symbols: | kinase | chr5:3486440-3488984 REVERSE no original description	
JCVI_19175	1.220	moderately similar to (214)AT5G18140| Symbols: | DNAJ heat shock N-terminal domain-containing protein | chr5:5998237-5999701 FC	
JCVI_8920	1.219	moderately similar to (304)AT1G06650| Symbols: | 2-oxoglutarate-dependent dioxygenase, putative | chr1:2035908-2037185 FORWARI	
JCVI_41388	1.218	highly similar to (565)AT1G32170| Symbols: XTH30, XTR4 | XTR4 (XYLOGLUCAN ENDOTRANSGLYCOSYLASE 4); hydrolase, a	
CN731025	1.218	moderately similar to (364)AT5G12900| Symbols: | similar to unknown protein [Arabidopsis thaliana] (TAIR:AT1G12330.1); similar to u	
EX093931	1.217	no similarity	1.298
JCVI_31014	1.217	very weakly similar to (89.4)AT1G62120| Symbols: | mitochondrial transcription termination factor-related / mTERF-related | chr1:22963	
JCVI_27739	1.217	moderately similar to (202)AT5G62570| Symbols: | calmodulin-binding protein | chr5:25132214-25134193 FORWARD no original desc	
JCVI_34031	1.216	highly similar to (853)AT1G59990| Symbols: | DEAD/DEAH box helicase, putative (RH22) | chr1:22094034-22096550 REVERSE no o	
DY018222	1.215	moderately similar to (344)AT1G49580| Symbols: | calcium-dependent protein kinase, putative / CDPK, putative | chr1:18355279-18358	
JCVI_20522	1.215	no original description	
EE569140	1.215	no similarity	
EV180925	1.214	moderately similar to (263)AT4G21880| Symbols: | pentatricopeptide (PPR) repeat-containing protein | chr4:11605168-11611220 FORW	
JCVI_37411	1.214	moderately similar to (421)AT4G10120| Symbols: ATSPS4F | ATSPS4F | chr4:6315029-6319781 FORWARDmoderately similar to (247	
JCVI_9072	1.213	very weakly similar to (98.6)AT2G35120| Symbols: | glycine cleavage system H protein, mitochondrial, putative | chr2:14812992-148143	
EX041844	1.213	no similarity	
JCVI_33154	1.212	highly similar to (574)AT4G01100| Symbols: | mitochondrial substrate carrier family protein | chr4:477411-479590 FORWARDweakly s	
EV110599	1.212	weakly similar to (118)AT4G21910| Symbols: | MATE efflux family protein | chr4:11625833-11630976 REVERSE [21478] 1 367 755	
ES951500	1.211	no similarity	
JCVI_12973	1.211	moderately similar to (298)AT5G10150| Symbols: | similar to unknown protein [Arabidopsis thaliana] (TAIR:AT5G59790.1); similar to	
JCVI_37218	1.210	moderately similar to (360)AT5G62460| Symbols: | zinc finger (C3HC4-type RING finger) family protein | chr5:25092771-25094298 FC	
JCVI_12162	1.208	no original description	
ES999914	1.208	no similarity	
JCVI_7195	1.206	moderately similar to (405)AT1G10600| Symbols: | similar to mov34 family protein [Arabidopsis thaliana] (TAIR:AT1G48790.1); simila	
EV195567	1.205	moderately similar to (259)AT5G59700| Symbols: | protein kinase, putative | chr5:24069839-24072328 REVERSE [21490] 38 769 769	
JCVI_41782	1.205	moderately similar to (429)AT3G22550| Symbols: | senescence-associated protein-related | chr3:7991834-7992812 REVERSE no origina	
JCVI_6142	1.202	moderately similar to (439)AT3G61320| Symbols: | Identical to UPF0187 protein At3g61320, chloroplast precursor [Arabidopsis Thalian	
JCVI_27298	1.202	highly similar to (506)AT1G22180| Symbols: | SEC14 cytosolic factor family protein / phosphoglyceride transfer family protein | chr1:78	
JCVI_40618	1.202	moderately similar to (468)AT1G64280| Symbols: SAI1, NIM1, NPR1 | NPR1 (NONEXPRESSER OF PR GENES 1); protein binding | c	
JCVI_11359	1.201	moderately similar to (226)AT5G61820| Symbols: | similar to MtN19-like protein [Pisum sativum] (GB:AAU14999.2); contains InterPro	
ES900614	1.201	weakly similar to (186)AT1G60610| Symbols: | protein binding / zinc ion binding | chr1:22331664-22332851 REVERSE [21428] 1 481 4	
JCVI_20247	1.201	no original description	
JCVI_22076	1.200	weakly similar to (198)AT5G55710| Symbols: | similar to tic20 protein-related [Arabidopsis thaliana] (TAIR:AT2G47840.1); similar to u	
ES960310	1.200	no similarity	
EE558554	1.199	moderately similar to (207)AT5G08620| Symbols: STRS2 | STRS2 (STRESS RESPONSE SUPPRESSOR 2); ATP-dependent helicase | c	
JCVI_31759	1.199	weakly similar to (110)AT5G17270| Symbols: | tetratricopeptide repeat (TPR)-containing protein | chr5:5679997-5685599 FORWARD n	
EV132032	1.198	no similarity	1.685
JCVI_21570	1.196	moderately similar to (201)AT1G13080| Symbols: CYP71B2 | CYP71B2 (CYTOCHROME P450 71B2); oxygen binding | chr1:4459491-	1.760
JCVI_24422	1.194	moderately similar to (320)AT3G09650| Symbols: CRM3, HCF152 | HCF152 (HIGH CHLOROPHYLL FLUORESCENCE 152) | chr3:2	
EV148999	1.194	no similarity	
EX045717	1.193	weakly similar to (115)AT5G49730| Symbols: ATFRO6, FRO6 | ATFRO6/FRO6 (FERRIC REDUCTION OXIDASE 6); ferric-chelate re	
AM062443	1.193	moderately similar to (383)AT4G05090| Symbols: | inositol monophosphatase family protein | chr4:2609242-2611625 FORWARDweakl	
JCVI_9203	1.193	weakly similar to (137)AT4G16160| Symbols: ATOEP16-2, ATOEP16-S | ATOEP16-2/ATOEP16-S; P-P-bond-hydrolysis-driven protein	
JCVI_6153	1.191	highly similar to (677)AT3G10530| Symbols: | transducin family protein / WD-40 repeat family protein | chr3:3286282-3288675 FORWA	
JCVI_10350	1.190	moderately similar to (288)AT3G09050| Symbols: | similar to unknown [Populus trichocarpa x Populus deltoides] (GB:ABK96465.1) | cl	
JCVI_32540	1.189	moderately similar to (377)AT5G13650| Symbols: | elongation factor family protein | chr5:4397824-4402367 FORWARD no original de	
JCVI_14409	1.189	highly similar to (574)AT5G52820| Symbols: | WD-40 repeat family protein / notchless protein, putative | chr5:21418649-21421429 FOF	
EV150324	1.188	no similarity	
JCVI_29495	1.187	moderately similar to (360)AT1G18660| Symbols: | zinc finger (C3HC4-type RING finger) family protein | chr1:6421425-6425557 FOR	
JCVI_9648	1.186	weakly similar to (167)AT3G05900| Symbols: | neurofilament protein-related | chr3:1761414-1763860 REVERSE no original description	
EV090658	1.186	weakly similar to (107)AT3G14200| Symbols: | DNAJ heat shock N-terminal domain-containing protein | chr3:4712888-4714368 REVEI	
JCVI_27451	1.185	highly similar to (910)AT2G47390| Symbols: | serine-type endopeptidase/ serine-type peptidase | chr2:19449348-19453323 REVERSE n	
JCVI_18645	1.184	no original description	
JCVI_1073	1.182	moderately similar to (226)AT1G15890| Symbols: | disease resistance protein (CC-NBS-LRR class), putative | chr1:5461400-5463955 FC	
EV111535	1.181	weakly similar to (109)AT5G01410| Symbols: ATPDX1.3, RSR4, PDX1.3, PDX1 | PDX1 (PYRIDOXINE BIOSYNTHESIS 1.3); protein	
ES909677	1.181	moderately similar to (497)AT5G18650| Symbols: | zinc finger (C3HC4-type RING finger) family protein | chr5:6218262-6220376 FOR	
EX123017	1.179	no similarity	
JCVI_3995	1.177	moderately similar to (256)AT2G33860| Symbols: ARF3, ETT | ETT (ETTIN); transcription factor | chr2:14332520-14335689 REVERSI	
EV155653	1.176	moderately similar to (238)AT1G58290| Symbols: HEMA1 | HEMA1; glutamyl-tRNA reductase | chr1:21627693-21629716 REVERSEm	
CB617597	1.175	no similarity	
JCVI_17545	1.175	weakly similar to (119)AT2G32710| Symbols: ACK2, ICK7, KRP4 | KRP4 (KIP-RELATED PROTEIN 4) | chr2:13880573-13882427 FC	
EE549419	1.174	no similarity	

EX086887	1.173	weakly similar to (128)AT5G63820| Symbols: | similar to unknown protein [Arabidopsis thaliana] (TAIR:AT4G28920.1); contains InterP	1.976
EE551212	1.173	no similarity	
JCVI_30131	1.173	very weakly similar to (88.2)AT1G66330| Symbols: | senescence-associated family protein | chr1:24733543-24735807 REVERSE no orig	
JCVI_13106	1.172	highly similar to (529)AT5G24150| Symbols: SQP1 | SQP1 (Squalene monooxygenase 1) | chr5:8172673-8175398 REVERSEhighly simi	
JCVI_32002	1.171	moderately similar to (388)AT4G05090| Symbols: | inositol monophosphatase family protein | chr4:2609242-2611625 FORWARDvery w	
EX020842	1.171	no similarity	
JCVI_18535	1.171	highly similar to (552)AT2G45160| Symbols: | scarecrow transcription factor family protein | chr2:18625185-18627107 REVERSEweakl	
CO750309	1.170	no similarity	
JCVI_7739	1.168	no original description	
EV102078	1.168	very weakly similar to (94.7)AT2G21240| Symbols: BPC4, BBR/BPC4, ATBPC4 | ATBPC4/BBR/BPC4/BPC4 (BASIC PENTACYSTEI	
EV118501	1.168	moderately similar to (320)AT4G29900| Symbols: ATACA10, ACA10 | ACA10 (autoinhibited Ca2+ -ATPase 10); calcium-transporting A	
ES964175	1.167	weakly similar to (129)AT1G02560| Symbols: NCLPP1, NCLPP5, CLPP5 | CLPP5 (NUCLEAR ENCODED CLP PROTEASE 1); endop	
JCVI_5696	1.166	very weakly similar to (95.1)AT1G64140| Symbols: | similar to loricrin-related [Arabidopsis thaliana] (TAIR:AT5G64550.1); similar to u	
AM386177	1.165	no similarity	
JCVI_24080	1.165	highly similar to (567)AT5G49990| Symbols: | xanthine/uracil permease family protein | chr5:20355090-20358413 REVERSE no origina	
CO750316	1.164	no similarity	
JCVI_41681	1.164	no original description	
RC_EV029606	1.163	no similarity	
EX054690	1.161	moderately similar to (201)AT3G02030| Symbols: | hydrolase, alpha/beta fold family protein | chr3:345031-347820 FORWARD [21812]	
JCVI_12613	1.160	moderately similar to (334)AT4G26850| Symbols: VTC2 | VTC2 (VITAMIN C DEFECTIVE 2) | chr4:13499268-13501151 REVERSE n	
EE191543	1.159	no similarity	
JCVI_17664	1.158	highly similar to (798)AT2G01320| Symbols: | ABC transporter family protein | chr2:154668-158062 REVERSEweakly similar to (181)	
JCVI_41152	1.158	weakly similar to (106)AT1G19700| Symbols: BEL10, BLH10A | BEL10/BLH10A (BEL1-LIKE HOMEODOMAIN 10); DNA binding /	
JCVI_6882	1.158	weakly similar to (110)AT1G60950| Symbols: ATFD2, FED A | FED A (FERREDOXIN 2); 2 iron, 2 sulfur cluster binding / electron car	
JCVI_17691	1.157	no original description	
RC_ES985508	1.155	no similarity	
JCVI_21672	1.154	weakly similar to (123)AT3G17980| Symbols: | C2 domain-containing protein | chr3:6152423-6153121 FORWARD no original descripti	
JCVI_31309	1.153	highly similar to (823)AT4G14210| Symbols: PDS, PDE226, PDS3 | PDS3 (PHYTOENE DESATURASE) | chr4:8190421-8194764 REV	
JCVI_34514	1.153	highly similar to (641)AT2G04030| Symbols: EMB1956, CR88 | CR88 (EMBRYO DEFECTIVE 1956); ATP binding | chr2:1281980-128	
JCVI_41912	1.152	moderately similar to (284)AT3G03950| Symbols: ECT1 | ECT1 | chr3:1021509-1023774 FORWARD no original description	
JCVI_26776	1.152	moderately similar to (322)AT4G31800| Symbols: WRKY18 | WRKY18 (WRKY DNA-binding protein 18); transcription factor | chr4:15	
JCVI_33756	1.152	moderately similar to (354)AT4G18130| Symbols: PHYE | PHYE (PHYTOCHROME DEFECTIVE E); G-protein coupled photoreceptor	
JCVI_22223	1.151	moderately similar to (337)AT5G58870| Symbols: FTSH9 | FTSH9 (FtsH protease 9); ATP-dependent peptidase/ ATPase/ metallopeptida	
JCVI_1077	1.151	moderately similar to (343)AT4G09040| Symbols: | RNA recognition motif (RRM)-containing protein | chr4:5795072-5797190 REVERS	
EV096192	1.151	weakly similar to (103)AT5G67385| Symbols: | signal transducer | chr5:26901980-26904309 FORWARD [21476]	
JCVI_22000	1.150	moderately similar to (270)AT1G24490| Symbols: ALB4 | ALB4 (ALBINA 4) | chr1:8682352-8684954 FORWARDvery weakly similar t	
JCVI_28412	1.150	moderately similar to (427)AT5G49730| Symbols: ATFRO6, FRO6 | ATFRO6/FRO6 (FERRIC REDUCTION OXIDASE 6); ferric-chela	
JCVI_3692	1.149	moderately similar to (352)AT5G11150| Symbols: VAMP713, ATVAMP713 | ATVAMP713 (Arabidopsis thaliana vesicle-associated me	
JCVI_35010	1.148	highly similar to (680)AT5G63420| Symbols: EMB2746 | EMB2746 (EMBRYO DEFECTIVE 2746); catalytic | chr5:25417741-2542303	
CO749749	1.148	no similarity	
ES968003	1.147	weakly similar to (121)AT2G20585| Symbols: NFD6 | NFD6 (NUCLEAR FUSION DEFECTIVE 6) | chr2:8872303-8873178 FORWARI	
EE436595	1.147	moderately similar to (261)AT2G39940| Symbols: COI1 | COI1 (CORONATINE INSENSITIVE 1); ubiquitin-protein ligase | chr2:16679	
JCVI_5140	1.146	moderately similar to (426)AT5G61820| Symbols: | similar to MtN19-like protein [Pisum sativum] (GB:AAU14999.2); contains InterPro	
EE566306	1.145	moderately similar to (250)AT1G56230| Symbols: | similar to unknown protein [Arabidopsis thaliana] (TAIR:AT2G22660.2); similar to u	
JCVI_7373	1.144	moderately similar to (264)AT3G11930| Symbols: | universal stress protein (USP) family protein | chr3:3776377-3777399 FORWARD n	
JCVI_11755	1.144	no original description	
JCVI_5203	1.144	moderately similar to (308)AT1G64230| Symbols: UBC28 | UBC28; ubiquitin-protein ligase | chr1:23837455-23838883 FORWARDweal	
JCVI_22412	1.144	highly similar to (636)AT5G66770| Symbols: | scarecrow transcription factor family protein | chr5:26677949-26679703 FORWARDweal	
EV215008	1.143	no similarity	
JCVI_18292	1.143	moderately similar to (311)AT5G45370| Symbols: | nodulin-related / integral membrane family protein | chr5:18405638-18407509 FORV	
JCVI_21110	1.143	moderately similar to (280)AT2G41720| Symbols: EMB2654 | EMB2654 (EMBRYO DEFECTIVE 2654) | chr2:17410822-17414205 RE	1.274
JCVI_11000	1.141	weakly similar to (197)AT1G22630| Symbols: | heat shock protein binding / unfolded protein binding | chr1:8003476-8004156 FORWAI	1.374
JCVI_22009	1.140	highly similar to (664)ATCG00170| Symbols: RPOC2 | RNA polymerase beta' subunit-2 | chrC:15938-20068 REVERSEhighly similar to	
ES269966	1.139	no similarity	
EV100448	1.139	no similarity	
EE438735	1.138	moderately similar to (224)AT1G18660| Symbols: | zinc finger (C3HC4-type RING finger) family protein | chr1:6421425-6425557 FOR	
EE418244	1.136	weakly similar to (122)AT3G06530| Symbols: | BAP28-related | chr3:2022602-2033643 FORWARD [20146] 1 386 426	
JCVI_20033	1.135	highly similar to (524)AT3G06483| Symbols: ATPDHK, PDK | PDK (PYRUVATE DEHYDROGENASE KINASE); ATP binding / pyru	1.305
ES963435	1.135	weakly similar to (116)AT5G16420| Symbols: | pentatricopeptide (PPR) repeat-containing protein | chr5:5368037-5369644 FORWARD	
ES902623	1.134	moderately similar to (471)AT2G40390| Symbols: | similar to unknown protein [Arabidopsis thaliana] (TAIR:AT5G64190.1); similar to u	
JCVI_24089	1.134	weakly similar to (115)AT3G62250| Symbols: UBQ5 | UBQ5 (UBIQUITIN 5); protein binding | chr3:23048113-23048586 FORWARDw	
JCVI_22106	1.133	moderately similar to (303)AT2G03670| Symbols: CDC48B | CDC48B; ATPase | chr2:1117592-1120358 FORWARDweakly similar to (	
EE462415	1.133	weakly similar to (110)AT1G62250| Symbols: | similar to unnamed protein product [Vitis vinifera] (GB:CAO21221.1) | chr1:22999280-2	
JCVI_35316	1.131	moderately similar to (340)AT3G20270| Symbols: | lipid-binding serum glycoprotein family protein | chr3:7068885-7070872 FORWARI	
JCVI_31260	1.130	moderately similar to (247)AT3G16640| Symbols: TCTP | TCTP (TRANSLATIONALLY CONTROLLED TUMOR PROTEIN) | chr3:56	1.359
JCVI_38082	1.129	no original description	
EE475633	1.128	weakly similar to (140)AT1G73700| Symbols: | MATE efflux family protein | chr1:27721215-27723291 REVERSE [20134] 1 522 535	
JCVI_36969	1.125	moderately similar to (238)AT4G37550| Symbols: | formamidase, putative / formamide amidohydrolase, putative | chr4:17643678-17645	
JCVI_28047	1.124	moderately similar to (364)AT3G04550| Symbols: | similar to unknown protein [Arabidopsis thaliana] (TAIR:AT5G28500.1); similar to u	
JCVI_42166	1.124	weakly similar to (159)AT2G22490| Symbols: CYCD2;1 | CYCD2;1 (CYCLIN D2;1); cyclin-dependent protein kinase regulator/ protein	
ES946106	1.124	no similarity	
EE513355	1.123	weakly similar to (125)AT1G19870| Symbols: IQD32 | IQD32 (IQ-domain 32); calmodulin binding | chr1:6895391-6898530 REVERSE [	
EV110515	1.122	no similarity	
JCVI_4017	1.121	moderately similar to (213)AT2G17710| Symbols: | similar to unnamed protein product [Vitis vinifera] (GB:CAO42932.1) | chr2:770093	
JCVI_24276	1.121	moderately similar to (385)AT3G27925| Symbols: DEG1, DEGP1 | DEGP1 (DEGP PROTEASE 1); serine-type peptidase | chr3:1036789	
EV063703	1.120	moderately similar to (379)AT2G31340| Symbols: EMB1381 | EMB1381 (EMBRYO DEFECTIVE 1381) | chr2:13368691-13371710 FO	
JCVI_1151	1.120	moderately similar to (255)AT2G47450| Symbols: CAO | CAO (CHAOS); chromatin binding | chr2:19479851-19480972 FORWARDwea	
JCVI_21174	1.120	moderately similar to (360)AT2G28200| Symbols: | nucleic acid binding / transcription factor/ zinc ion binding | chr2:12031398-1203225	
ES952320	1.120	moderately similar to (269)AT4G31850| Symbols: PGR3 | PGR3 (PROTON GRADIENT REGULATION 3) | chr4:15403026-15406364 I	
JCVI_29162	1.120	highly similar to (594)AT1G24530| Symbols: | transducin family protein / WD-40 repeat family protein | chr1:8693274-8694530 FORW	2.110
JCVI_3146	1.117	moderately similar to (358)AT1G06650| Symbols: | 2-oxoglutarate-dependent dioxygenase, putative | chr1:2035908-2037185 FORWARI	
JCVI_7067	1.116	moderately similar to (439)AT3G06960| Symbols: PDE320 | PDE320 (PIGMENT DEFECTIVE 320) | chr3:2195222-2196510 REVERSI	1.174
EV111447	1.114	no similarity	

EX065546	1.114	very weakly similar to (80.5)ERG11_BRANA [21815]	
JCVI_4355	1.113	moderately similar to (340)AT2G42490| Symbols: | copper amine oxidase, putative | chr2:17698678-17702604 REVERSEvery weakly si	
JCVI_11025	1.113	no original description	
EV171250	1.111	weakly similar to (152)AT4G10080| Symbols: | similar to unknown protein [Arabidopsis thaliana] (TAIR:AT4G13530.1); similar to unna	
JCVI_28871	1.109	moderately similar to (273)AT3G57810| Symbols: | OTU-like cysteine protease family protein | chr3:21427313-21428786 FORWARD n	
JCVI_1532	1.107	moderately similar to (228)AT3G52800| Symbols: | zinc finger (AN1-like) family protein | chr3:19580784-19581296 FORWARDweakly	
EV056658	1.107	very weakly similar to (81.6)AT3G22942| Symbols: AGG2 | AGG2 (G-PROTEIN GAMMA SUBUNIT 2) | chr3:8134482-8135674 FORV	
JCVI_27351	1.106	moderately similar to (222)AT5G38990| Symbols: | protein kinase family protein | chr5:15626054-15628696 FORWARD no original des	
JCVI_22281	1.104	moderately similar to (420)AT2G38330| Symbols: | MATE efflux family protein | chr2:16071649-16074396 FORWARD no original des	
JCVI_5439	1.104	moderately similar to (326)AT4G16370| Symbols: OPT3, ATOPT3 | ATOPT3 (OLIGOPEPTIDE TRANSPORTER); oligopeptide transp	
JCVI_31427	1.102	moderately similar to (211)AT5G63860| Symbols: UVR8 | UVR8 (UVB-RESISTANCE 8) | chr5:25572047-25575813 REVERSE no orig	
ES952239	1.100	moderately similar to (298)AT5G07670| Symbols: | F-box family protein | chr5:2430422-2432066 FORWARD [21423]	
JCVI_28453	1.100	moderately similar to (211)AT3G08010| Symbols: ATAB2 | ATAB2; RNA binding | chr3:2556052-2557432 FORWARD no original desc	
JCVI_36393	1.099	no original description	
JCVI_15132	1.099	weakly similar to (173)AT1G51090| Symbols: | heavy-metal-associated domain-containing protein | chr1:18936653-18937415 FORWAR	
JCVI_8692	1.098	no original description	
JCVI_30678	1.096	moderately similar to (345)AT4G01037| Symbols: | similar to EMB1692 (EMBRYO DEFECTIVE 1692) [Arabidopsis thaliana] (TAIR:A	
CV432588	1.096	moderately similar to (215)AT5G20350| Symbols: TIP1 | TIP1 (TIP GROWTH DEFECTIVE 1) | chr5:6876774-6881104 FORWARD [1(	
JCVI_31822	1.095	no original description	
JCVI_40421	1.094	no original description	1.213
JCVI_23938	1.094	moderately similar to (320)AT2G03810| Symbols: | 18S pre-ribosomal assembly protein gar2-related | chr2:1162700-1164283 FORWAR	
JCVI_12922	1.093	weakly similar to (125)AT1G47500| Symbols: ATRBP47C' | ATRBP47C' (RNA-BINDING PROTEIN 47C'); RNA binding | chr1:174351	
JCVI_41907	1.093	highly similar to (538)AT5G51890| Symbols: | peroxidase | chr5:21108389-21109561 REVERSEmoderately similar to (241)PER1_ORY	
ES901726	1.091	no similarity	
CV544312	1.090	moderately similar to (209)AT3G27330| Symbols: | zinc finger (C3HC4-type RING finger) family protein | chr3:10118187-10121753 RE	
EX045943	1.089	no similarity	
JCVI_8063	1.086	moderately similar to (220)AT5G64300| Symbols: ATGCH | ATGCH (ARABIDOPSIS THALIANA GTP CYCLOHYDROLASE II); 3,4	
JCVI_41814	1.086	no original description	
EV217832	1.084	no similarity	
JCVI_12678	1.080	moderately similar to (479)AT2G03810| Symbols: | 18S pre-ribosomal assembly protein gar2-related | chr2:1162700-1164283 FORWAR	
EX090230	1.080	weakly similar to (110)AT1G35460| Symbols: | basic helix-loop-helix (bHLH) family protein | chr1:13040070-13041885 FORWARD [2	
JCVI_17892	1.079	moderately similar to (295)AT2G40460| Symbols: | proton-dependent oligopeptide transport (POT) family protein | chr2:16904201-1690	1.167
DY009503	1.078	weakly similar to (111)AT5G66470| Symbols: | GTP binding / RNA binding | chr5:26559212-26561528 REVERSE [18969] 1 378 455	
JCVI_11911	1.077	highly similar to (542)AT3G23700| Symbols: | S1 RNA-binding domain-containing protein | chr3:8531696-8533749 REVERSEweakly s	
JCVI_37793	1.077	no original description	
JCVI_42443	1.075	no original description	
JCVI_3872	1.074	moderately similar to (204)AT1G05870| Symbols: | similar to unknown protein [Arabidopsis thaliana] (TAIR:AT2G31560.1); similar to u	2.265
JCVI_1588	1.072	moderately similar to (406)AT4G27585| Symbols: | band 7 family protein | chr4:13766990-13769838 REVERSE no original description	
CV433633	1.071	weakly similar to (104)AT1G34780| Symbols: ATAPRL4 | ATAPRL4 (APR-LIKE 4) | chr1:12748813-12750102 REVERSE [16490] 1 4	
DY015167	1.070	moderately similar to (252)AT3G08010| Symbols: ATAB2 | ATAB2; RNA binding | chr3:2556052-2557432 FORWARD [18966]	
JCVI_5197	1.068	moderately similar to (239)AT3G58680| Symbols: MBF1B, ATMBF1B | ATMBF1B/MBF1B (MULTIPROTEIN BRIDGING FACTOR	
ES266193	1.066	moderately similar to (311)AT4G07960| Symbols: CSLC12, ATCSLC12 | ATCSLC12 (Cellulose synthase-like C12); transferase, transfer	
JCVI_38503	1.065	very weakly similar to (87.0)AT2G34720| Symbols: | CCAAT-binding transcription factor (CBF-B/NF-YA) family protein | chr2:146570	
JCVI_7350	1.064	highly similar to (640)AT1G21780| Symbols: | BTB/POZ domain-containing protein | chr1:7652465-7653855 FORWARD no original de	
JCVI_10417	1.062	weakly similar to (154)AT3G10410| Symbols: SCPL49 | SCPL49 (serine carboxypeptidase-like 49); serine carboxypeptidase | chr3:3235	
JCVI_934	1.060	moderately similar to (370)AT3G61440| Symbols: ARATH;BSAS3;1, ATCYSC1 | ATCYSC1 (BETA-SUBSTITUTED ALA SYNTHAS	
JCVI_18551	1.058	no original description	
JCVI_42045	1.058	weakly similar to (174)AT5G23050| Symbols: | acyl-activating enzyme 17 (AAE17) | chr5:7731516-7735402 REVERSE no original desc	
JCVI_37755	1.056	no original description	
EV167683	1.054	weakly similar to (135)AT1G50420| Symbols: SCL-3, SCL3 | SCL3 (SCARECROW-LIKE 3); transcription factor | chr1:18681845-1868	
JCVI_29481	1.053	moderately similar to (293)AT4G31390| Symbols: | ABC1 family protein | chr4:15233132-15236770 FORWARD no original description	
JCVI_4086	1.052	moderately similar to (401)AT1G49670| Symbols: | ARP protein (REF) | chr1:18385259-18389689 REVERSEvery weakly similar to (90	
JCVI_27019	1.051	weakly similar to (149)AT4G22770| Symbols: | DNA-binding family protein | chr4:11963890-11965450 REVERSE no original descripti	
JCVI_5858	1.050	highly similar to (635)AT1G53750| Symbols: RPT1A | RPT1A (regulatory particle triple-A 1A); ATPase | chr1:20069589-20071992 REV	
JCVI_10621	1.046	weakly similar to (159)ATCG00660| Symbols: RPL20 | encodes a chloroplast ribosomal protein L20, a constituent of the large subunit of	
AM394356	1.045	very weakly similar to (97.4)AT3G60070| Symbols: | lactose permease-related | chr3:22194554-22196917 REVERSE [20346] 1 372 400	
RC_H74686	1.045	no similarity	
JCVI_16561	1.034	moderately similar to (282)AT1G55140| Symbols: | RNA binding / ribonuclease III | chr1:20576455-20577999 REVERSE no original de	
JCVI_23123	1.031	very weakly similar to (85.9)AT5G66760| Symbols: SDH1-1 | SDH1-1 (Succinate dehydrogenase 1-1) | chr5:26671002-26674450 FORW.	
JCVI_21113	1.031	very weakly similar to (82.8)ATCG00905| Symbols: RPS12, RPS12C | chloroplast gene encoding ribosomal protein s12. The gene is locat	
BQ704934	1.029	weakly similar to (183)AT1G21780| Symbols: | BTB/POZ domain-containing protein | chr1:7652465-7653855 FORWARD [11009]	
EV156226	1.027	no similarity	
RC_CD838260	1.027	no similarity	
JCVI_14373	1.027	moderately similar to (245)AT1G63680| Symbols: ATMURE, PDE316 | ATMURE/PDE316 (PIGMENT DEFECTIVE EMBRYO); ATP	
JCVI_27050	1.024	moderately similar to (258)AT5G63790| Symbols: ANAC102 | ANAC102 (Arabidopsis NAC domain containing protein 102); transcripti	
JCVI_19595	1.023	moderately similar to (317)AT1G68070| Symbols: | zinc finger (C3HC4-type RING finger) family protein | chr1:25519075-25520430 RE	
EV169442	1.021	weakly similar to (134)AT5G02840| Symbols: LCL1 | LCL1 (LHY/CCA1-LIKE 1); DNA binding / transcription factor | chr5:648792-651	
JCVI_23769	1.019	weakly similar to (182)AT4G33980| Symbols: | similar to unknown protein [Arabidopsis thaliana] (TAIR:AT5G42900.2); similar to unkr	1.803
JCVI_4654	1.012	highly similar to (513)AT1G67800| Symbols: | copine-related | chr1:25424692-25426900 REVERSE no original description	
ES928296	1.009	no similarity	
JCVI_24666	1.005	weakly similar to (132)AT2G37130| Symbols: | peroxidase 21 (PER21) (P21) (PRXR5) | chr2:15605304-15606813 REVERSE no origina	1.084
JCVI_16888	1.004	moderately similar to (283)AT5G24120| Symbols: SIG5, SIGE | SIGE (RNA polymerase sigma subunit E); DNA binding / DNA-directed	
JCVI_7881	1.004	moderately similar to (424)AT4G08700| Symbols: ATPUP13 | ATPUP13 (Arabidopsis thaliana purine permease 13); purine transmembra	
JCVI_20818	1.001	moderately similar to (312)AT1G44446| Symbols: ATCAO, CAO, CH1 | CH1 (CHLOROPHYLL B BIOSYNTHESIS); chlorophyllide a	1.607
JCVI_5147	0.987	moderately similar to (335)AT3G08010| Symbols: ATAB2 | ATAB2; RNA binding | chr3:2556052-2557432 FORWARD no original desc	
JCVI_11350	0.969	moderately similar to (373)AT1G71870| Symbols: | MATE efflux family protein | chr1:27036118-27038557 REVERSE no original desc	
EV226857	0.963	weakly similar to (196)AT5G24470| Symbols: PRR5, APRR5 | APRR5 (PSEUDO-RESPONSE REGULATOR 5); transcription regulator	
EV174833	0.956	no similarity	
JCVI_38057	0.953	moderately similar to (377)AT5G20040| Symbols: ATIPT9 | ATIPT9 (Arabidopsis thaliana isopentenyltransferase 9); ATP binding / tRN.	
JCVI_18310	0.935	moderately similar to (281)AT5G02830| Symbols: | pentatricopeptide (PPR) repeat-containing protein | chr5:644456-648419 REVERSE	
EE535812	0.932	no similarity	
JCVI_33200	0.915	no original description	
CD844622	0.845	no similarity	

EV106399	0.843	no similarity	
EV125308	0.837	no similarity	
ES938100	0.814	moderately similar to (370)AT4G33170| Symbols: | pentatricopeptide (PPR) repeat-containing protein | chr4:15995704-15998676 REVE	
JCVI_16366	0.811	no original description	
CD824445	0.782	moderately similar to (232)AT3G06880| Symbols: | transducin family protein / WD-40 repeat family protein | chr3:2169862-2175692 RE	
EV207729	0.694	no similarity	
JCVI_35222	0.648	moderately similar to (276)AT5G51700| Symbols: ATRAR1, RPR2, RAR1, PBS2 | PBS2 (PPHB SUSCEPTIBLE 2); protein binding / zi	
RC_ES958174	0.630	no similarity	
EV047064	0.591	weakly similar to (145)AT2G42860| Symbols: | unknown protein | chr2:17840936-17841724 FORWARD [21442]	
ES927658	0.486	no similarity	
EE550774	0.480	very weakly similar to (83.2)AT2G43730| Symbols: | lectin-related | chr2:18131898-18132746 REVERSE [20184]	
EV087280	0.308	no similarity	1.730
JCVI_26638	0.049	no original description	

ɔn
