# Supplementary material for: Influence of Light and Temperature on Gene Expression Leading to Accumulation of Specific Flavonol Glycosides and Hydroxycinnamic Acid Derivatives in Kale (Brassica oleracea var. sabellica)
Source: Front Plant Sci. 2016 Mar 30;7:326. doi: 10.3389/fpls.2016.00326 (PMC4812050; doi:10.3389/fpls.2016.00326)
Supplement: Supplementary file 4 [file Table4.PDF]

Identifier	log2-fold HT induced	Description	log2-fold LL-HL
EV130558	8.415	moderately similar to (212)AT5G07010| Symbols: | sulfotransferase family protein | chr5:2174961-2176040 REVERSE [21481]	
EV098915	7.540	moderately similar to (238)AT3G21330| Symbols: | basic helix-loop-helix (bHLH) family protein | chr3:7507726-7508847 FORWARDv	
JCVI_25745	7.515	weakly similar to (157)AT4G37770| Symbols: ACS8 | ACS8 (1-Amino-cyclopropane-1-carboxylate synthase 8) | chr4:17752216-177539	
JCVI_17490	7.488	moderately similar to (304)AT4G29930| Symbols: | basic helix-loop-helix (bHLH) family protein | chr4:14644114-14645174 FORWAR	
EX108375	7.224	moderately similar to (425)AT5G07010| Symbols: | sulfotransferase family protein | chr5:2174961-2176040 REVERSE [21827]	-3.398
JCVI_15833	7.046	moderately similar to (218)AT4G19430| Symbols: | unknown protein | chr4:10598617-10599099 REVERSE no original description	
JCVI_36023	6.997	no original description	-3.797
CN831014	6.473	no similarity	-4.295
DW998114	6.414	moderately similar to (406)AT4G16590| Symbols: CSLA01, ATCSLA1, ATCSLA01 | ATCSLA01 (Cellulose synthase-like A1); glucosy	
JCVI_23517	6.392	weakly similar to (104)AT1G10585| Symbols: | transcription factor | chr1:3494116-3495105 REVERSE no original description	
EX121359	6.330	no similarity	
EE417789	6.325	moderately similar to (232)AT5G07200| Symbols: GA20OX3, ATGA20OX3 | YAP169 (GIBBERELLIN 20 OXIDASE 3); gibberellin 2	
JCVI_29231	6.274	moderately similar to (366)AT5G63450| Symbols: CYP94B1 | CYP94B1 (cytochrome P450, family 94, subfamily B, polypeptide 1); oxy	-3.231
JCVI_710	6.251	no original description	-3.192
EV171128	6.245	weakly similar to (129)AT1G72290| Symbols: | trypsin and protease inhibitor family protein / Kunitz family protein | chr1:27219514-272	
JCVI_11499	6.243	moderately similar to (278)AT5G64750| Symbols: ABR1 | ABR1 (ABA REPRESSOR1); DNA binding / transcription factor | chr5:2590	-2.347
JCVI_30902	6.237	highly similar to (503)AT5G05600| Symbols: | oxidoreductase, 2OG-Fe(II) oxygenase family protein | chr5:1672267-1674603 FORWA	
JCVI_34713	5.965	weakly similar to (115)AT5G50335| Symbols: | unknown protein | chr5:20506617-20506841 REVERSE no original description	
JCVI_22060	5.956	weakly similar to (135)AT5G02580| Symbols: | similar to unknown protein [Arabidopsis thaliana] (TAIR:AT3G55240.1); similar to hyp	-3.622
JCVI_19167	5.942	weakly similar to (182)AT5G28237| Symbols: | tryptophan synthase, beta subunit, putative | chr5:10207481-10213546 REVERSEweakl	
JCVI_1498	5.925	moderately similar to (452)AT5G05600| Symbols: | oxidoreductase, 2OG-Fe(II) oxygenase family protein | chr5:1672267-1674603 FOR	-2.475
JCVI_36990	5.924	weakly similar to (129)AT5G44110| Symbols: ATPOP1, ATNAP2, POP1 | POP1 | chr5:17771619-17772899 REVERSE no original des	
EV098037	5.903	weakly similar to (166)AT5G07200| Symbols: GA20OX3, ATGA20OX3 | YAP169 (GIBBERELLIN 20 OXIDASE 3); gibberellin 20-o	
EV217349	5.903	weakly similar to (167)AT1G14240| Symbols: | nucleoside phosphatase family protein / GDA1/CD39 family protein | chr1:4865154-486	
DY016454	5.852	weakly similar to (176)AT5G28237| Symbols: | tryptophan synthase, beta subunit, putative | chr5:10207481-10213546 REVERSEweakl	
EE446658	5.799	no similarity	
EV015464	5.790	very weakly similar to (96.3)AT4G27860| Symbols: | integral membrane family protein | chr4:13873814-13876246 FORWARD [21440]	
JCVI_941	5.581	moderately similar to (385)AT1G54020| Symbols: | myrosinase-associated protein, putative | chr1:20165473-20166591 REVERSE no o	
AM394020	5.531	very weakly similar to (85.5)AT5G28237| Symbols: | tryptophan synthase, beta subunit, putative | chr5:10207481-10213546 REVERSE |	
JCVI_20310	5.508	weakly similar to (150)AT5G02580| Symbols: | similar to unknown protein [Arabidopsis thaliana] (TAIR:AT3G55240.1); similar to hyp	-4.636
JCVI_11516	5.490	no original description	
JCVI_7069	5.488	highly similar to (816)AT2G46370| Symbols: JAR, FIN219, JAR1 | JAR1 (JASMONATE RESISTANT 1) | chr2:19041652-19043442 F	-1.832
JCVI_32135	5.418	highly similar to (660)AT3G63010| Symbols: ATGID1B, GID1B | ATGID1B/GID1B (GA INSENSITIVE DWARF1B); hydrolase | chr3	-3.608
JCVI_33618	5.398	moderately similar to (457)AT4G35160| Symbols: | O-methyltransferase family 2 protein | chr4:16730994-16732813 REVERSEweakly	
EG019365	5.364	weakly similar to (135)AT3G55240| Symbols: | Overexpression leads to PEL (Pseudo-Etiolation in Light) phenotype. | chr3:20484853-2	-3.669
EV168076	5.354	no similarity	-2.664
EV057320	5.337	weakly similar to (128)AT5G02580| Symbols: | similar to unknown protein [Arabidopsis thaliana] (TAIR:AT3G55240.1); similar to hyp	-4.060
JCVI_25738	5.268	no original description	-3.824
JCVI_24967	5.267	weakly similar to (112)AT2G43520| Symbols: ATTI2 | ATTI2 (ARABIDOPSIS THALIANA TRYPSIN INHIBITOR PROTEIN 2); tryp	
JCVI_1353	5.265	moderately similar to (437)AT1G54020| Symbols: | myrosinase-associated protein, putative | chr1:20165473-20166591 REVERSE no o	
EE465824	5.262	weakly similar to (193)AT1G62975| Symbols: | basic helix-loop-helix (bHLH) family protein (bHLH125) | chr1:23332566-23334187 FC	-3.209
JCVI_24500	5.237	moderately similar to (255)AT4G32030| Symbols: | unknown protein | chr4:15490909-15493013 FORWARD no original description	-2.013
EV142649	5.196	moderately similar to (238)AT3G28610| Symbols: | ATP binding / ATPase | chr3:10726227-10727651 FORWARD [21482]	-2.409
JCVI_13710	5.194	moderately similar to (218)AT2G34690| Symbols: ACD11 | ACD11 (ACCELERATED CELL DEATH 11) | chr2:14637504-14638858 I	-5.609
AM395348	5.164	weakly similar to (102)AT3G45140| Symbols: ATLOX2, LOX2 | LOX2 (LIPOXYGENASE 2) | chr3:16536422-16540218 FORWARD	
AM062272	5.154	no similarity	-3.528
JCVI_11797	5.151	highly similar to (577)AT2G39310| Symbols: | jacalin lectin family protein | chr2:16421340-16423401 REVERSE no original descriptio	
JCVI_32378	5.136	highly similar to (590)AT1G30040| Symbols: ATGA2OX2 | ATGA2OX2; gibberellin 2-beta-dioxygenase | chr1:10537753-10538765 FC	
EV100963	5.074	weakly similar to (141)AT5G44260| Symbols: | zinc finger (CCCH-type) family protein | chr5:17847201-17848346 REVERSE [21477]	
EE419603	5.058	moderately similar to (267)AT5G64750| Symbols: ABR1 | ABR1 (ABA REPRESSOR1); DNA binding / transcription factor | chr5:2590	
JCVI_1048	5.042	weakly similar to (108)AT5G25810| Symbols: TNY | TNY (TINY); DNA binding / transcription factor | chr5:8986979-8987635 REVEF	-2.031
JCVI_13226	5.036	highly similar to (665)AT4G21760| Symbols: BGLU47 | BGLU47 (Beta-glucosidase 47); hydrolase, hydrolyzing O-glycosyl compounds	-2.787
JCVI_20214	5.024	weakly similar to (199)AT3G12500| Symbols: PR3, PR-3, CHI-B, B-CHI, ATHCHIB | ATHCHIB (BASIC CHITINASE); chitinase | chr	
JCVI_4644	4.999	weakly similar to (187)AT1G72290| Symbols: | trypsin and protease inhibitor family protein / Kunitz family protein | chr1:27219514-272	
JCVI_27469	4.989	weakly similar to (150)AT1G11790| Symbols: ADT1 | ADT1 (AROGENATE DEHYDRATASE 1); prephenate dehydratase | chr1:3981	
JCVI_18858	4.972	moderately similar to (365)AT5G67210| Symbols: | nucleic acid binding / pancreatic ribonuclease | chr5:26836245-26837198 FORWAF	-2.752
H07729	4.914	no similarity	-3.515
EV203432	4.876	moderately similar to (242)AT4G28940| Symbols: | catalytic | chr4:14274420-14276926 FORWARD [21490]	
EL590539	4.862	very weakly similar to (99.4)AT5G53290| Symbols: CRF3 | CRF3 (CYTOKININ RESPONSE FACTOR 3); DNA binding / transcription	
EX123246	4.852	moderately similar to (360)AT5G47330| Symbols: | palmitoyl protein thioesterase family protein | chr5:19224397-19225938 FORWARI	-3.111
JCVI_9118	4.837	moderately similar to (241)AT4G32280| Symbols: IAA29 | IAA29 (indoleacetic acid-induced protein 29); transcription factor | chr4:155	
DN963281	4.816	weakly similar to (177)AT3G45140| Symbols: ATLOX2, LOX2 | LOX2 (LIPOXYGENASE 2) | chr3:16536422-16540218 FORWARD	
JCVI_15504	4.800	weakly similar to (154)AT3G55240| Symbols: | Overexpression leads to PEL (Pseudo-Etiolation in Light) phenotype. | chr3:20484853-2	-3.341
JCVI_23511	4.793	moderately similar to (388)AT5G24420| Symbols: | glucosamine/galactosamine-6-phosphate isomerase-related | chr5:8336946-8337882	
EH425128	4.791	no similarity	-3.638
JCVI_4290	4.779	moderately similar to (317)AT1G73325| Symbols: | trypsin and protease inhibitor family protein / Kunitz family protein | chr1:27571179	
EV124048	4.771	weakly similar to (128)AT4G35160| Symbols: | O-methyltransferase family 2 protein | chr4:16730994-16732813 REVERSE [21479] 1	
EX040782	4.727	weakly similar to (149)AT2G42870| Symbols: PAR1, HLH1 | HLH1/PAR1 (PHY RAPIDLY REGULATED 1); transcription regulator |	
JCVI_15469	4.726	no original description	
JCVI_652	4.704	moderately similar to (216)AT1G72290| Symbols: | trypsin and protease inhibitor family protein / Kunitz family protein | chr1:27219514	
EV186492	4.703	weakly similar to (138)AT2G32150| Symbols: | haloacid dehalogenase-like hydrolase family protein | chr2:13666172-13667608 FORW	-2.890
JCVI_21653	4.698	weakly similar to (142)AT1G10370| Symbols: GST30, ATGSTU17, GST30B, ERD9 | ATGSTU17/ERD9/GST30/GST30B (EARLY-R	
JCVI_7181	4.687	moderately similar to (202)AT3G05020| Symbols: ACP, ACP1 | ACP1 (ACYL CARRIER PROTEIN 1) | chr3:1391869-1392884 REVE	
AM059084	4.656	no similarity	-3.776
JCVI_12269	4.650	weakly similar to (174)AT3G05640| Symbols: | protein phosphatase 2C, putative / PP2C, putative | chr3:1640616-1642233 REVERSE r	
JCVI_342	4.634	moderately similar to (240)AT1G72290| Symbols: | trypsin and protease inhibitor family protein / Kunitz family protein | chr1:27219514	
JCVI_13739	4.611	moderately similar to (406)AT3G25770| Symbols: AOC2 | AOC2 (ALLENE OXIDE CYCLASE 2) | chr3:9408212-9409076 FORWAR	
EE545201	4.602	no similarity	-3.268

EX131577	4.597	no similarity	
JCVI_32365	4.592	moderately similar to (360)AT5G01380| Symbols: | transcription factor | chr5:155783-157450 REVERSE no original description	
JCVI_40394	4.574	moderately similar to (321)AT3G16350| Symbols: | myb family transcription factor | chr3:5547834-5549403 FORWARD no original de	-1.868
JCVI_7865	4.526	no original description	-2.783
JCVI_19310	4.509	weakly similar to (172)AT1G54120| Symbols: | similar to unknown protein [Arabidopsis thaliana] (TAIR:AT3G14060.1); similar to unn	
EV140916	4.507	very weakly similar to (91.7)AT5G53710| Symbols: | unknown protein | chr5:21822635-21822979 REVERSE [21482] 1 430 724	
JCVI_35883	4.497	very weakly similar to (91.7)AT5G53710| Symbols: | unknown protein | chr5:21822635-21822979 REVERSE no original description	
ES911771	4.481	weakly similar to (102)AT1G64405| Symbols: | unknown protein | chr1:23927463-23927819 FORWARD [21431] 1 383 459	
EV176695	4.480	weakly similar to (110)AT3G06070| Symbols: | similar to unknown protein [Arabidopsis thaliana] (TAIR:AT5G19190.1); similar to unn	
EE457196	4.479	no similarity	-2.417
JCVI_39155	4.467	no original description	
RC_EX077725	4.459	no similarity	
EX132242	4.454	weakly similar to (159)AT2G32150| Symbols: | haloacid dehalogenase-like hydrolase family protein | chr2:13666172-13667608 FORWA	-2.547
JCVI_22853	4.446	moderately similar to (215)AT5G53290| Symbols: CRF3 | CRF3 (CYTOKININ RESPONSE FACTOR 3); DNA binding / transcription	
EX081446	4.428	weakly similar to (106)AT5G52320| Symbols: CYP96A4 | CYP96A4 (cytochrome P450, family 96, subfamily A, polypeptide 4); oxygen	
JCVI_22253	4.415	moderately similar to (335)AT2G43590| Symbols: | chitinase, putative | chr2:18088669-18089826 REVERSEmoderately similar to (35	-4.375
EX103444	4.415	moderately similar to (303)AT5G09470| Symbols: | mitochondrial substrate carrier family protein | chr5:2949242-2950514 REVERSE [	
CX188694	4.413	moderately similar to (244)AT1G14240| Symbols: | nucleoside phosphatase family protein / GDA1/CD39 family protein | chr1:4865154	
JCVI_17220	4.403	moderately similar to (306)AT4G38580| Symbols: ATFP6 | ATFP6 (FARNESYLATED PROTEIN 6); metal ion binding | chr4:1803459	-1.852
EV200816	4.396	moderately similar to (400)AT1G65450| Symbols: | transferase family protein | chr1:24321698-24322558 FORWARDvery weakly simil	-2.155
DW999910	4.385	moderately similar to (255)AT5G49690| Symbols: | UDP-glucoronosyl/UDP-glucosyl transferase family protein | chr5:20207194-20208	
EV102840	4.375	weakly similar to (160)AT2G46970| Symbols: PIL1 | PIL1 (PHYTOCHROME INTERACTING FACTOR 3-LIKE 1); transcription facto	
JCVI_26899	4.364	weakly similar to (193)AT1G69760| Symbols: | similar to unknown protein [Arabidopsis thaliana] (TAIR:AT1G26920.1); similar to hyp	-2.687
JCVI_29146	4.349	moderately similar to (250)AT5G13220| Symbols: JAZ10, TIFY9, JAS1, AT5G13220 | JAS1/JAZ10/TIFY9 (JASMONATE-ZIM-DOM	
EV049094	4.339	weakly similar to (148)AT5G53290| Symbols: CRF3 | CRF3 (CYTOKININ RESPONSE FACTOR 3); DNA binding / transcription facto	
JCVI_7525	4.322	moderately similar to (215)AT3G27810| Symbols: AtMYB21, AtMYB3, MYB21, ATMYB21 | ATMYB21 (MYB DOMAIN PROTEIN	-1.571
ES910283	4.287	moderately similar to (212)AT3G06070| Symbols: | similar to unknown protein [Arabidopsis thaliana] (TAIR:AT5G19190.1); similar to	
JCVI_4395	4.287	moderately similar to (314)AT3G47380| Symbols: | invertase/pectin methylesterase inhibitor family protein | chr3:17468780-17469388 I	
CA991551	4.280	very weakly similar to (91.3)AT1G32190| Symbols: | similar to unknown protein [Arabidopsis thaliana] (TAIR:AT2G24320.1); similar to	
JCVI_7969	4.275	weakly similar to (189)AT3G12500| Symbols: PR3, PR-3, CHI-B, B-CHI, ATHCHIB | ATHCHIB (BASIC CHITINASE); chitinase | chr	
JCVI_26448	4.268	highly similar to (720)AT3G45140| Symbols: ATLOX2, LOX2 | LOX2 (LIPOXYGENASE 2) | chr3:16536422-16540218 FORWARDh	-2.547
JCVI_23070	4.256	very weakly similar to (97.8)AT1G70270| Symbols: | unknown protein | chr1:26468090-26468350 FORWARD no original description	
JCVI_22403	4.240	moderately similar to (302)AT2G46970| Symbols: PIL1 | PIL1 (PHYTOCHROME INTERACTING FACTOR 3-LIKE 1); transcription	
JCVI_22632	4.232	no original description	-2.303
JCVI_39721	4.224	very weakly similar to (91.7)AT4G33680| Symbols: AGD2 | AGD2 (ABERRANT GROWTH AND DEATH 2); transaminase | chr4:1617	
JCVI_9280	4.218	moderately similar to (214)AT5G25530| Symbols: | DNAJ heat shock protein, putative | chr5:8889668-8890957 REVERSEvery weakly	
JCVI_3732	4.217	weakly similar to (200)AT5G13220| Symbols: JAZ10, TIFY9, JAS1, AT5G13220 | JAS1/JAZ10/TIFY9 (JASMONATE-ZIM-DOMAIN	-2.996
JCVI_10293	4.205	moderately similar to (363)AT4G03400| Symbols: GH3-10, DFL2 | DFL2 (DWARF IN LIGHT 2) | chr4:1497674-1499728 REVERSEv	
JCVI_6435	4.188	weakly similar to (141)AT4G29905| Symbols: | similar to unknown protein [Arabidopsis thaliana] (TAIR:AT5G57123.1); similar to hyp	-2.895
EV092285	4.182	moderately similar to (300)AT4G32280| Symbols: IAA29 | IAA29 (indoleacetic acid-induced protein 29); transcription factor | chr4:155	
JCVI_4653	4.178	moderately similar to (218)AT5G36220| Symbols: CYP91A1, CYP81D1 | CYP81D1 (CYTOCHROME P450 91A1); oxygen binding | c	
JCVI_39565	4.166	weakly similar to (142)AT4G29905| Symbols: | similar to unknown protein [Arabidopsis thaliana] (TAIR:AT5G57123.1); similar to hyp	-2.491
EX037465	4.160	weakly similar to (123)AT3G49360| Symbols: | glucosamine/galactosamine-6-phosphate isomerase family protein | chr3:18314174-1831	
EV103971	4.137	no similarity	
EX044537	4.132	moderately similar to (280)AT1G66700| Symbols: PXMT1 | PXMT1; S-adenosylmethionine-dependent methyltransferase | chr1:24877	
JCVI_1330	4.125	moderately similar to (291)AT4G37990| Symbols: ELI3, ELI3-2 | ELI3-2 (ELICITOR-ACTIVATED GENE 3) | chr4:17855958-178573	
JCVI_20291	4.117	no original description	
JCVI_17865	4.110	highly similar to (633)AT1G06620| Symbols: | 2-oxoglutarate-dependent dioxygenase, putative | chr1:2025617-2027093 FORWARDmo	-1.845
JCVI_33787	4.102	moderately similar to (320)AT3G11340| Symbols: | UDP-glucoronosyl/UDP-glucosyl transferase family protein | chr3:3556734-355815	-4.679
JCVI_32805	4.084	moderately similar to (230)AT4G35720| Symbols: | similar to unknown protein [Arabidopsis thaliana] (TAIR:AT4G35690.1); similar to	
EV110827	4.069	no similarity	
EV144805	4.066	no similarity	
EE502449	4.050	no similarity	
JCVI_427	4.041	moderately similar to (371)AT2G43590| Symbols: | chitinase, putative | chr2:18088669-18089826 REVERSEmoderately similar to (37	-2.852
JCVI_38496	4.021	no original description	
JCVI_41466	4.019	no original description	
EV076295	4.018	weakly similar to (180)AT1G51790| Symbols: | kinase | chr1:19210527-19214243 REVERSE [21443]	-4.755
EV217600	4.017	moderately similar to (233)AT1G14250| Symbols: | nucleoside phosphatase family protein / GDA1/CD39 family protein | chr1:4868670	
JCVI_23685	4.004	no original description	-6.153
JCVI_1165	4.002	moderately similar to (219)AT3G16870| Symbols: | zinc finger (GATA type) family protein | chr3:5763758-5764582 REVERSE no orig	
JCVI_36190	3.997	weakly similar to (164)AT3G27880| Symbols: | similar to unknown protein [Arabidopsis thaliana] (TAIR:AT1G23710.1); similar to hyp	
JCVI_15319	3.991	highly similar to (570)AT1G19670| Symbols: CORI1, ATHCOR1 | ATCLH1 (CORONATINE-INDUCED PROTEIN 1) | chr1:6803787	
JCVI_25469	3.978	no original description	
JCVI_37398	3.971	no original description	-3.072
JCVI_6670	3.953	moderately similar to (382)AT2G02990| Symbols: RNS1 | RNS1 (RIBONUCLEASE 1); endoribonuclease | chr2:873713-874666 FORV	-3.569
JCVI_38091	3.952	no original description	-5.354
EX099259	3.932	moderately similar to (310)AT1G04180| Symbols: | flavin-containing monooxygenase family protein / FMO family protein | chr1:11046	
JCVI_2208	3.925	moderately similar to (270)AT1G75050| Symbols: | similar to ATLP-3 (Arabidopsis thaumatin-like protein 3) [Arabidopsis thaliana] (TA	-3.253
JCVI_24116	3.924	no original description	
EV110472	3.924	no similarity	
EX125506	3.921	moderately similar to (237)AT5G28050| Symbols: | cytidine/deoxycytidylate deaminase family protein | chr5:10044213-10045488 REV	-3.200
JCVI_20329	3.920	moderately similar to (232)AT1G72416| Symbols: | heat shock protein binding | chr1:27262657-27263771 REVERSE no original descri	-1.917
EV098486	3.914	moderately similar to (202)AT1G75450| Symbols: ATCKX5, ATCKX6, CKX5 | CKX5 (CYTOKININ OXIDASE 5); cytokinin dehydro	
JCVI_34461	3.908	highly similar to (515)AT4G36010| Symbols: | pathogenesis-related thaumatin family protein | chr4:17039475-17040979 REVERSEmo	
EE432166	3.905	weakly similar to (130)AT1G12890| Symbols: | AP2 domain-containing transcription factor, putative | chr1:4391732-4392391 FORWA	-3.571
EX106564	3.904	moderately similar to (232)AT5G20820| Symbols: | auxin-responsive protein-related | chr5:7046913-7047296 REVERSE [21827] 1 573	
JCVI_3093	3.903	moderately similar to (261)AT5G14120| Symbols: | nodulin family protein | chr5:4556310-4558449 FORWARD no original description	
JCVI_12176	3.895	no original description	-2.941
EE473027	3.892	very weakly similar to (91.3)AT1G02450| Symbols: NIMIN1, NIMIN-1 | NIMIN-1/NIMIN1; protein binding | chr1:498052-498480 REV	-4.437
JCVI_11913	3.891	highly similar to (742)AT2G26650| Symbols: AKT1 | AKT1 (ARABIDOPSIS K TRANSPORTER 1); cyclic nucleotide binding / inward	

JCVI_22820	3.888	moderately similar to (258)AT3G21230| Symbols: 4CL5 | 4CL5 (4-COUMARATE:COA LIGASE 5); 4-coumarate-CoA ligase | chr3:74	
JCVI_33417	3.881	weakly similar to (172)AT3G16870| Symbols: | zinc finger (GATA type) family protein | chr3:5763758-5764582 REVERSE no original	
JCVI_1912	3.880	moderately similar to (250)AT1G72290| Symbols: | trypsin and protease inhibitor family protein / Kunitz family protein | chr1:27219514	
EE408644	3.876	moderately similar to (232)AT1G75040| Symbols: PR-5, PR5 | PR5 (PATHOGENESIS-RELATED GENE 5) | chr1:28181415-2818239	-3.963
EV198220	3.875	no similarity	
EX087313	3.872	moderately similar to (216)AT2G31730| Symbols: | ethylene-responsive protein, putative | chr2:13494860-13495670 REVERSE [21823	-2.983
JCVI_17417	3.870	moderately similar to (216)AT2G21140| Symbols: ATPRP2 | ATPRP2 (PROLINE-RICH PROTEIN 2) | chr2:9067949-9069116 REVE	
JCVI_4502	3.869	highly similar to (560)AT1G80820| Symbols: CCR2 | CCR2 (CINNAMOYL COA REDUCTASE) | chr1:30375538-30377352 FORWA	-1.517
EX134821	3.867	moderately similar to (230)AT1G44130| Symbols: | nucellin protein, putative | chr1:16789948-16791758 REVERSEweakly similar to (	-3.293
JCVI_227	3.864	moderately similar to (333)AT2G38310| Symbols: | similar to unknown protein [Arabidopsis thaliana] (TAIR:AT5G05440.1); similar to	
JCVI_32435	3.854	moderately similar to (209)AT5G40210| Symbols: | nodulin MtN21 family protein | chr5:16090953-16093316 REVERSE no original de	-2.329
JCVI_15229	3.850	moderately similar to (202)AT1G75580| Symbols: | auxin-responsive protein, putative | chr1:28381191-28381517 FORWARDvery wea	
BQ704543	3.838	no similarity	-6.545
JCVI_37915	3.827	moderately similar to (421)AT5G19600| Symbols: SULTR3;5 | SULTR3;5; sulfate transmembrane transporter | chr5:6613115-6616893	
JCVI_10438	3.826	moderately similar to (251)AT5G57780| Symbols: | transcription regulator | chr5:23422731-23423234 REVERSE no original descriptio	
EX123908	3.819	moderately similar to (249)AT1G25340| Symbols: AtMYB116, MYB116 | MYB116 (myb domain protein 116); DNA binding / transcrip	-4.276
JCVI_33624	3.818	moderately similar to (301)AT3G30180| Symbols: CYP85A2, BR6OX2 | BR6OX2/CYP85A2 (BRASSINOSTEROID-6-OXIDASE 2);	
EV162033	3.814	moderately similar to (217)AT1G75050| Symbols: | similar to ATLP-3 (Arabidopsis thaumatin-like protein 3) [Arabidopsis thaliana] (TA	-3.071
JCVI_21124	3.798	moderately similar to (354)AT4G35160| Symbols: | O-methyltransferase family 2 protein | chr4:16730994-16732813 REVERSE no orig	
JCVI_40180	3.772	weakly similar to (140)AT4G23600| Symbols: JR2, CORI3 | CORI3 (CORONATINE INDUCED 1, JASMONIC ACID RESPONSIVE	
JCVI_3953	3.767	moderately similar to (241)AT3G06070| Symbols: | similar to unknown protein [Arabidopsis thaliana] (TAIR:AT5G19190.1); similar to	
ES903033	3.759	moderately similar to (270)AT5G03760| Symbols: CSLA09, ATCSLA9, CSLA9, RAT4, ATCSLA09 | ATCSLA09 (RESISTANT TO A	
JCVI_5021	3.748	moderately similar to (379)AT1G17170| Symbols: GST, ATGSTU24 | ATGSTU24 (ARABIDOPSIS THALIANA GLUTATHIONE S-T	-3.369
AM395252	3.747	moderately similar to (238)AT2G43590| Symbols: | chitinase, putative | chr2:18088669-18089826 REVERSEmoderately similar to (24	-3.647
EX120515	3.743	no similarity	-4.511
JCVI_14772	3.741	moderately similar to (228)AT5G67310| Symbols: CYP81G1 | CYP81G1 (cytochrome P450, family 81, subfamily G, polypeptide 1); oxy	
EV167713	3.734	moderately similar to (221)AT1G13080| Symbols: CYP71B2 | CYP71B2 (CYTOCHROME P450 71B2); oxygen binding | chr1:445949	-2.740
JCVI_35024	3.725	moderately similar to (226)AT1G72290| Symbols: | trypsin and protease inhibitor family protein / Kunitz family protein | chr1:27219514	
JCVI_25531	3.725	very weakly similar to (82.0)AT1G75940| Symbols: ATA27 | ATA27 (Arabidopsis thaliana anther 27); hydrolase, hydrolyzing O-glycosy	
JCVI_459	3.720	moderately similar to (446)AT2G32150| Symbols: | haloacid dehalogenase-like hydrolase family protein | chr2:13666172-13667608 FO	-2.109
JCVI_27673	3.719	moderately similar to (216)AT5G27200| Symbols: ACP5 | ACP5 (ACYL CARRIER PROTEIN 5); acyl carrier | chr5:9571188-9571992	
EE558777	3.719	no similarity	
EH420554	3.719	no similarity	-3.002
ES943003	3.712	moderately similar to (284)AT3G06550| Symbols: | similar to O-acetyltransferase family protein [Arabidopsis thaliana] (TAIR:AT2G34	
EV179210	3.710	weakly similar to (164)AT3G52370| Symbols: FLA15 | FLA15 (FASCICLIN-LIKE ARABINOGALACTAN PROTEIN 15 PRECURSO	
JCVI_15112	3.708	moderately similar to (378)AT3G08670| Symbols: | similar to unknown protein [Arabidopsis thaliana] (TAIR:AT3G51540.1); similar to	
EV110218	3.701	no similarity	
JCVI_8990	3.696	moderately similar to (422)AT5G36220| Symbols: CYP91A1, CYP81D1 | CYP81D1 (CYTOCHROME P450 91A1); oxygen binding | c	
EV018962	3.694	weakly similar to (187)AT5G12050| Symbols: | similar to unnamed protein product [Vitis vinifera] (GB:CAO45643.1) | chr5:3890214-3	
JCVI_19327	3.692	moderately similar to (490)AT3G57240| Symbols: BG3 | BG3 (BETA-1,3-GLUCANASE 3); hydrolase, hydrolyzing O-glycosyl compou	-3.747
JCVI_18861	3.691	no original description	
EX126494	3.681	weakly similar to (152)AT1G66700| Symbols: PXMT1 | PXMT1; S-adenosylmethionine-dependent methyltransferase | chr1:24877489-2	
EV048418	3.647	weakly similar to (102)AT4G32030| Symbols: | unknown protein | chr4:15490909-15493013 FORWARD [21442]	
JCVI_17170	3.644	weakly similar to (138)AT5G36220| Symbols: CYP91A1, CYP81D1 | CYP81D1 (CYTOCHROME P450 91A1); oxygen binding | chr5:	
JCVI_26408	3.636	moderately similar to (410)AT5G36220| Symbols: CYP91A1, CYP81D1 | CYP81D1 (CYTOCHROME P450 91A1); oxygen binding | c	
ES967164	3.631	no similarity	
JCVI_29799	3.628	weakly similar to (136)AT3G28857| Symbols: | transcription regulator | chr3:10857018-10857550 REVERSE no original description	
AI352922	3.626	very weakly similar to (83.2)AT2G39530| Symbols: | integral membrane protein, putative | chr2:16505737-16506479 REVERSE [1285]	-4.296
JCVI_33949	3.626	moderately similar to (348)AT2G13810| Symbols: ALD1 | ALD1 (AGD2-LIKE DEFENSE RESPONSE PROTEIN1); transaminase | chr	-4.664
JCVI_1001	3.620	moderately similar to (320)AT1G78170| Symbols: | similar to unknown protein [Arabidopsis thaliana] (TAIR:AT1G22250.1); similar to	-2.182
EX047564	3.618	no similarity	
EV189247	3.616	no similarity	
ES954834	3.611	no similarity	-7.439
JCVI_1195	3.603	moderately similar to (336)AT3G61460| Symbols: BRH1 | BRH1 (BRASSINOSTEROID-RESPONSIVE RING-H2); protein binding / z	-2.225
JCVI_17600	3.603	moderately similar to (499)AT2G19780| Symbols: | leucine-rich repeat family protein / extensin family protein | chr2:8529913-8531121	
EV171151	3.601	weakly similar to (179)AT3G08670| Symbols: | similar to unknown protein [Arabidopsis thaliana] (TAIR:AT3G51540.1); similar to hyp	
JCVI_39399	3.597	moderately similar to (234)AT1G13080| Symbols: CYP71B2 | CYP71B2 (CYTOCHROME P450 71B2); oxygen binding | chr1:445949	-2.442
JCVI_12575	3.590	no original description	
JCVI_34682	3.588	weakly similar to (121)AT1G12710| Symbols: ATPP2-A12 | ATPP2-A12 (PHLOEM PROTEIN 2-A12); carbohydrate binding | chr1:43	
JCVI_40432	3.587	no original description	
JCVI_28986	3.584	moderately similar to (356)AT4G03400| Symbols: GH3-10, DFL2 | DFL2 (DWARF IN LIGHT 2) | chr4:1497674-1499728 REVERSEm	
JCVI_2743	3.582	moderately similar to (292)AT5G62280| Symbols: | similar to unknown protein [Arabidopsis thaliana] (TAIR:AT2G45360.1); similar to	
EV132289	3.580	no similarity	
JCVI_5873	3.579	highly similar to (629)AT1G75450| Symbols: ATCKX5, ATCKX6, CKX5 | CKX5 (CYTOKININ OXIDASE 5); cytokinin dehydrogena	
JCVI_34350	3.578	moderately similar to (368)AT1G52410| Symbols: TSA1 | TSA1 (TSK-ASSOCIATING PROTEIN 1) | chr1:19524431-19529030 FORV	
JCVI_32604	3.562	highly similar to (856)AT2G18790| Symbols: HY3, OOP1, PHYB | PHYB (PHYTOCHROME B); G-protein coupled photoreceptor/ sig	
JCVI_3442	3.562	weakly similar to (137)AT4G15440| Symbols: CYP74B2, HPL1 | HPL1 (HYDROPEROXIDE LYASE 1); heme binding / iron ion bindi	
JCVI_19024	3.559	moderately similar to (369)AT4G27420| Symbols: | ABC transporter family protein | chr4:13712440-13714803 REVERSE no original d	
JCVI_41716	3.558	moderately similar to (318)AT5G01380| Symbols: | transcription factor | chr5:155783-157450 REVERSE no original description	-2.152
JCVI_17759	3.556	moderately similar to (243)AT1G29195| Symbols: | similar to unknown protein [Arabidopsis thaliana] (TAIR:AT2G30230.1); similar to	
ES953976	3.556	no similarity	
DW997085	3.550	moderately similar to (352)AT5G24420| Symbols: | glucosamine/galactosamine-6-phosphate isomerase-related | chr5:8336946-8337882	
EX135524	3.547	moderately similar to (234)AT5G28050| Symbols: | cytidine/deoxycytidylate deaminase family protein | chr5:10044213-10045488 REV	-2.993
JCVI_19367	3.546	moderately similar to (281)AT5G06860| Symbols: PGIP1 | PGIP1 (POLYGALACTURONASE INHIBITING PROTEIN 1); protein bin	
DY017774	3.543	no similarity	
JCVI_17750	3.539	moderately similar to (240)AT3G52370| Symbols: FLA15 | FLA15 (FASCICLIN-LIKE ARABINOGALACTAN PROTEIN 15 PRECU	
EV154298	3.536	no similarity	-3.259
JCVI_24170	3.536	weakly similar to (200)AT3G45140| Symbols: ATLOX2, LOX2 | LOX2 (LIPOXYGENASE 2) | chr3:16536422-16540218 FORWARD	-1.673
JCVI_18327	3.531	highly similar to (733)AT3G45140| Symbols: ATLOX2, LOX2 | LOX2 (LIPOXYGENASE 2) | chr3:16536422-16540218 FORWARDm	
JCVI_5211	3.528	weakly similar to (122)AT1G75580| Symbols: | auxin-responsive protein, putative | chr1:28381191-28381517 FORWARD no original d	

JCVI_4746	3.527	weakly similar to (172)AT1G52000| Symbols: | jacalin lectin family protein | chr1:19337021-19339369 REVERSE no original descriptio	
JCVI_14100	3.527	moderately similar to (340)AT5G07580| Symbols: | DNA binding / transcription factor | chr5:2399526-2400350 FORWARDweakly sim	
JCVI_28810	3.526	moderately similar to (225)AT4G16780| Symbols: HAT4, ATHB2, ATHB-2 | ATHB-2 (ARABIDOPSIS THALIANA HOMEOBOX PF	
EX028195	3.519	weakly similar to (121)AT2G44570| Symbols: ATGH9B12 | ATGH9B12 (ARABIDOPSIS THALIANA GLYCOSYL HYDROLASE 9F	
JCVI_3863	3.516	weakly similar to (182)AT1G58270| Symbols: ZW9 | ZW9 | chr1:21616059-21617754 REVERSE no original description	
EX103892	3.511	no similarity	-2.287
EE568090	3.503	moderately similar to (258)AT1G04180| Symbols: | flavin-containing monooxygenase family protein / FMO family protein | chr1:11046	
JCVI_18851	3.501	highly similar to (854)AT3G26170| Symbols: CYP71B19 | CYP71B19 (cytochrome P450, family 71, subfamily B, polypeptide 19); oxyg	-2.981
JCVI_40290	3.500	very weakly similar to (94.7)AT1G02340| Symbols: RSF1, FBI1, REP1, HFR1 | HFR1 (LONG HYPOCOTYL IN FAR-RED); DNA binc	
JCVI_37097	3.498	moderately similar to (309)AT1G66700| Symbols: PXMT1 | PXMT1; S-adenosylmethionine-dependent methyltransferase | chr1:248774	
JCVI_25824	3.498	moderately similar to (434)AT5G44050| Symbols: | MATE efflux family protein | chr5:17739711-17743436 FORWARD no original de	
JCVI_2645	3.485	no original description	
EX096357	3.485	no similarity	
JCVI_9773	3.480	moderately similar to (478)AT1G33811| Symbols: | GDSL-motif lipase/hydrolase family protein | chr1:12267898-12269670 FORWARI	
JCVI_28964	3.470	weakly similar to (139)AT1G75030| Symbols: ATLP-3 | ATLP-3 (Arabidopsis thaumatin-like protein 3) | chr1:28178079-28178916 FOF	-3.277
JCVI_11024	3.461	weakly similar to (185)AT5G22580| Symbols: | Identical to Uncharacterized protein At5g22580 [Arabidopsis Thaliana] (GB:Q9FK81);	
EV217644	3.455	no similarity	
EE468644	3.448	very weakly similar to (87.0)AT3G04300| Symbols: | similar to unknown protein [Arabidopsis thaliana] (TAIR:AT4G10300.1); similar to	-3.011
JCVI_22236	3.448	no original description	
ES969778	3.446	weakly similar to (101)AT4G03400| Symbols: GH3-10, DFL2 | DFL2 (DWARF IN LIGHT 2) | chr4:1497674-1499728 REVERSE [157	
EH429329	3.443	no similarity	-3.228
EV204109	3.441	weakly similar to (136)AT1G58270| Symbols: ZW9 | ZW9 | chr1:21616059-21617754 REVERSE [21491] 51 736 736	
JCVI_40375	3.439	no original description	-1.568
JCVI_9325	3.436	weakly similar to (141)AT3G05810| Symbols: | similar to unknown protein [Arabidopsis thaliana] (TAIR:AT5G26800.1); similar to unk	
EX095316	3.433	moderately similar to (292)AT3G04930| Symbols: | transcription regulator | chr3:1363035-1364405 FORWARD [21824] 1 764 778	
EV225738	3.424	moderately similar to (335)AT5G39110| Symbols: | germin-like protein, putative | chr5:15675030-15675812 REVERSEweakly similar t	-3.855
JCVI_806	3.421	moderately similar to (447)AT1G53070| Symbols: | legume lectin family protein | chr1:19782039-19782857 FORWARDvery weakly sir	
JCVI_26793	3.403	moderately similar to (471)AT3G07010| Symbols: | pectate lyase family protein | chr3:2212979-2216312 REVERSEmoderately similar	
H74760	3.401	no similarity	
DY019353	3.396	moderately similar to (345)AT4G11650| Symbols: ATOSM34 | ATOSM34 (OSMOTIN 34) | chr4:7025121-7026107 REVERSEmodera	
EE431428	3.394	weakly similar to (122)AT1G18710| Symbols: AtMYB47 | AtMYB47 (myb domain protein 47); DNA binding / transcription factor | chr	
JCVI_21470	3.386	moderately similar to (382)AT1G65680| Symbols: EXPB2, ATHEXP BETA 1.4, ATEXPB2 | ATEXPB2 (ARABIDOPSIS THALIANA	-3.428
JCVI_40779	3.381	no original description	-2.554
EE532339	3.378	weakly similar to (196)AT3G03520| Symbols: | phosphoesterase family protein | chr3:837979-840518 REVERSE [20175]	
JCVI_115	3.377	moderately similar to (381)AT1G58270| Symbols: ZW9 | ZW9 | chr1:21616059-21617754 REVERSE no original description	
JCVI_38983	3.377	moderately similar to (299)AT4G36410| Symbols: UBC17 | UBC17 (UBIQUITIN-CONJUGATING ENZYME 17); ubiquitin-protein lig	
JCVI_14980	3.364	moderately similar to (295)AT5G20630| Symbols: GLP3A, GLP3B, GLP3 | GLP3 (GERMIN-LIKE PROTEIN 3); manganese ion bindin	
JCVI_9028	3.354	weakly similar to (197)AT1G30320| Symbols: | remorin family protein | chr1:10680330-10682834 FORWARD no original description	-1.887
EH416910	3.350	no similarity	-7.078
JCVI_38481	3.349	moderately similar to (288)AT1G75280| Symbols: | isoflavone reductase, putative | chr1:28255691-28257016 FORWARDmoderately si	
JCVI_8333	3.347	moderately similar to (394)AT5G58310| Symbols: | hydrolase, alpha/beta fold family protein | chr5:23592353-23593233 REVERSEwea	
JCVI_778	3.345	highly similar to (978)AT1G20620| Symbols: SEN2, CAT3 | CAT3 (CATALASE 3); catalase | chr1:7143132-7146183 FORWARDhigh	-3.555
ES907698	3.341	moderately similar to (343)AT3G14370| Symbols: WAG2 | WAG2; kinase | chr3:4798033-4799475 REVERSEweakly similar to (152)C	
JCVI_25482	3.339	moderately similar to (279)AT2G38340| Symbols: | AP2 domain-containing transcription factor, putative (DRE2B) | chr2:16074525-16(	
JCVI_39501	3.338	moderately similar to (394)AT2G13810| Symbols: ALD1 | ALD1 (AGD2-LIKE DEFENSE RESPONSE PROTEIN1); transaminase | chı	-4.331
JCVI_8916	3.338	weakly similar to (120)AT5G04820| Symbols: ATOFP13, OFP13 | ATOFP13/OFP13 (Arabidopsis thaliana ovate family protein 13) | chı	
JCVI_28110	3.336	no original description	
JCVI_5907	3.336	highly similar to (558)AT1G12010| Symbols: | 1-aminocyclopropane-1-carboxylate oxidase, putative / ACC oxidase, putative | chr1:405(	
AM059719	3.336	no similarity	
JCVI_36485	3.334	moderately similar to (353)AT3G11340| Symbols: | UDP-glucoronosyl/UDP-glucosyl transferase family protein | chr3:3556734-355815	-3.794
EE443550	3.333	weakly similar to (116)AT1G72130| Symbols: | proton-dependent oligopeptide transport (POT) family protein | chr1:27141425-2714288	
JCVI_25735	3.332	highly similar to (853)AT1G32640| Symbols: RD22BP1, JAI1, JIN1, MYC2, ZBF1, ATMYC2 | ATMYC2 (JASMONATE INSENSITI	
JCVI_17673	3.331	moderately similar to (281)AT2G38090| Symbols: | myb family transcription factor | chr2:15952356-15953853 FORWARD no original	
JCVI_3277	3.330	moderately similar to (273)AT4G22010| Symbols: SKS4 | SKS4 (SKU5 Similar 4); copper ion binding / oxidoreductase | chr4:11663441	
JCVI_24991	3.330	moderately similar to (372)AT3G57260| Symbols: PR2, BG2, PR-2, BGL2 | BGL2 (PATHOGENESIS-RELATED PROTEIN 2); glucar	-3.611
JCVI_8821	3.329	weakly similar to (135)AT5G55450| Symbols: | protease inhibitor/seed storage/lipid transfer protein (LTP) family protein | chr5:2248478	-4.475
ES909993	3.328	moderately similar to (266)AT4G27950| Symbols: CRF4 | CRF4 (CYTOKININ RESPONSE FACTOR 4); DNA binding / transcription	-2.795
JCVI_5921	3.327	moderately similar to (332)AT2G22170| Symbols: | lipid-associated family protein | chr2:9434090-9434822 REVERSE no original desc	
EV179235	3.325	weakly similar to (146)AT1G75050| Symbols: | similar to ATLP-3 (Arabidopsis thaumatin-like protein 3) [Arabidopsis thaliana] (TAIR:	-2.765
EX065636	3.323	very weakly similar to (82.0)AT1G75450| Symbols: ATCKX5, ATCKX6, CKX5 | CKX5 (CYTOKININ OXIDASE 5); cytokinin dehydr	
JCVI_30218	3.319	no original description	-3.355
JCVI_37933	3.318	moderately similar to (234)AT4G32800| Symbols: | AP2 domain-containing transcription factor TINY, putative | chr4:15819816-158204	
CD811808	3.316	no similarity	-2.401
JCVI_36586	3.314	moderately similar to (351)AT1G52030| Symbols: MBP1.2, F-ATMBP, MBP2 | MBP2 (MYROSINASE-BINDING PROTEIN 2) | chr1	
EX134057	3.310	highly similar to (509)AT1G73340| Symbols: | oxygen binding | chr1:27576797-27578934 FORWARDweakly similar to (161)C90D2_	
JCVI_1186	3.305	weakly similar to (193)AT2G39530| Symbols: | integral membrane protein, putative | chr2:16505737-16506479 REVERSE no original c	-4.271
JCVI_15441	3.304	moderately similar to (360)AT2G32990| Symbols: ATGH9B8 | ATGH9B8 (ARABIDOPSIS THALIANA GLYCOSYL HYDROLASE 9	
EV013609	3.303	weakly similar to (144)AT2G32990| Symbols: ATGH9B8 | ATGH9B8 (ARABIDOPSIS THALIANA GLYCOSYL HYDROLASE 9B8)	
JCVI_32319	3.301	very weakly similar to (83.6)AT3G22910| Symbols: | calcium-transporting ATPase, plasma membrane-type, putative / Ca(2+)-ATPase, pı	-1.601
EV098450	3.300	weakly similar to (139)AT4G28940| Symbols: | catalytic | chr4:14274420-14276926 FORWARD [21477] 46 1101 1101	
EE525938	3.294	no similarity	-3.631
JCVI_33581	3.293	no original description	-7.465
CD829515	3.293	no similarity	
RC_ES964477	3.284	no similarity	-1.742
JCVI_8763	3.282	no original description	
JCVI_25528	3.275	moderately similar to (422)AT3G57260| Symbols: PR2, BG2, PR-2, BGL2 | BGL2 (PATHOGENESIS-RELATED PROTEIN 2); glucar	-4.548
JCVI_29830	3.273	weakly similar to (110)AT1G48330| Symbols: | similar to unknown protein [Arabidopsis thaliana] (TAIR:AT3G17580.1); similar to unk	
DN962854	3.266	moderately similar to (306)AT5G67360| Symbols: ARA12 | ARA12; subtilase | chr5:26889418-26891691 REVERSE [17359]	-3.480
ES264431	3.263	moderately similar to (283)AT1G78260| Symbols: | RNA recognition motif (RRM)-containing protein | chr1:29452210-29454139 FOR	-2.534
EV164885	3.263	weakly similar to (194)AT3G09560| Symbols: | lipin family protein | chr3:2934958-2938678 REVERSE [21485] 39 739 739	

JCVI_6928	3.261	moderately similar to (349)AT1G17745| Symbols: PGDH | PGDH (3-PHOSPHOGLYCERATE DEHYDROGENASE); phosphoglycera	-2.910
EX135752	3.261	moderately similar to (386)AT5G54170| Symbols: | similar to CP5 [Arabidopsis thaliana] (TAIR:AT1G64720.1); similar to putative nod	-1.794
JCVI_25797	3.257	moderately similar to (219)AT4G27860| Symbols: | integral membrane family protein | chr4:13873814-13876246 FORWARD no origin	
EV110906	3.246	no similarity	
JCVI_38243	3.239	no original description	
EX106705	3.235	moderately similar to (493)AT5G28237| Symbols: | tryptophan synthase, beta subunit, putative | chr5:10207481-10213546 REVERSEm	
JCVI_12384	3.233	weakly similar to (108)AT3G02885| Symbols: GASA5 | GASA5 (GAST1 PROTEIN HOMOLOG 5) | chr3:638330-639018 REVERSE	-3.631
CV544978	3.228	no similarity	
EV197431	3.224	no similarity	-3.524
EX141963	3.224	very weakly similar to (80.9)AT3G25717| Symbols: RTFL16, DVL6 | DVL6/RTFL16 (ROTUNDIFOLIA LIKE 16) | chr3:9376709-9376	
JCVI_31116	3.223	weakly similar to (188)AT5G67620| Symbols: | similar to unknown protein [Arabidopsis thaliana] (TAIR:AT5G62900.1); similar to unn	-2.208
JCVI_41333	3.219	weakly similar to (144)AT2G01340| Symbols: | similar to unknown protein [Arabidopsis thaliana] (TAIR:AT1G71015.1); similar to unk	
EE448877	3.216	moderately similar to (265)AT4G30410| Symbols: | transcription factor | chr4:14871315-14871857 REVERSE [20172]	
L46441	3.210	no similarity	-4.458
JCVI_17977	3.209	moderately similar to (289)AT5G66590| Symbols: | allergen V5/Tpx-1-related family protein | chr5:26591481-26592038 FORWARDwe	
EE566300	3.203	no similarity	-3.422
JCVI_10237	3.196	weakly similar to (192)AT5G04000| Symbols: | similar to hypothetical protein [Vitis vinifera] (GB:CAN76250.1) | chr5:1079304-10797	-3.409
RC_EX117010	3.195	no similarity	-3.835
JCVI_17120	3.194	moderately similar to (386)AT2G38310| Symbols: | similar to unknown protein [Arabidopsis thaliana] (TAIR:AT5G05440.1); similar to	
JCVI_33902	3.190	highly similar to (582)AT4G37980| Symbols: ELI3-1 | ELI3-1 (ELICITOR-ACTIVATED GENE 3); binding / catalytic/ oxidoreductase/	
EX128203	3.189	no similarity	
EV203397	3.184	moderately similar to (364)AT5G67360| Symbols: ARA12 | ARA12; subtilase | chr5:26889418-26891691 REVERSE [21490] 39 792 79	-2.962
ES966646	3.179	very weakly similar to (84.7)AT2G40330| Symbols: | Bet v I allergen family protein | chr2:16852255-16852902 REVERSE [20153]	
EE455499	3.178	no similarity	
JCVI_26920	3.172	highly similar to (724)AT2G34810| Symbols: | FAD-binding domain-containing protein | chr2:14692371-14693993 FORWARD no orig	
JCVI_33757	3.167	moderately similar to (218)AT1G69760| Symbols: | similar to unknown protein [Arabidopsis thaliana] (TAIR:AT1G26920.1); similar to	-2.463
EV203310	3.163	moderately similar to (386)AT5G67360| Symbols: ARA12 | ARA12; subtilase | chr5:26889418-26891691 REVERSE [21490]	-3.694
JCVI_35919	3.158	moderately similar to (323)AT1G44350| Symbols: ILL6 | ILL6 (IAA-leucine resistant (ILR)-like gene 6); metallopeptidase | chr1:168371	
EV017499	3.156	no similarity	-2.396
JCVI_16082	3.152	moderately similar to (362)AT5G57560| Symbols: XTH22, TCH4 | TCH4 (TOUCH 4); hydrolase, acting on glycosyl bonds / xyloglucan	
DY022165	3.149	moderately similar to (342)AT1G33260| Symbols: | protein kinase family protein | chr1:12064776-12066094 FORWARDvery weakly si	
JCVI_9163	3.147	moderately similar to (283)AT5G14120| Symbols: | nodulin family protein | chr5:4556310-4558449 FORWARD no original description	
EV052278	3.146	weakly similar to (164)AT5G40210| Symbols: | nodulin MtN21 family protein | chr5:16090953-16093316 REVERSE [21442]	-2.039
EX132647	3.142	moderately similar to (309)AT3G04510| Symbols: | similar to LSH1 (LIGHT-DEPENDENT SHORT HYPOCOTYLS 1) [Arabidopsis t	-3.067
EV064737	3.141	no similarity	
JCVI_22371	3.141	weakly similar to (119)AT1G07570| Symbols: APK1, APK1A | APK1A (Arabidopsis protein kinase 1A); kinase | chr1:2331366-233320	
JCVI_13432	3.141	moderately similar to (392)AT1G17180| Symbols: ATGSTU25 | ATGSTU25 (Arabidopsis thaliana Glutathione S-transferase (class tau)	-2.702
JCVI_34797	3.136	weakly similar to (198)AT3G03520| Symbols: | phosphoesterase family protein | chr3:837979-840518 REVERSE no original descriptio	-2.146
JCVI_40133	3.132	weakly similar to (178)AT3G16870| Symbols: | zinc finger (GATA type) family protein | chr3:5763758-5764582 REVERSE no original	
JCVI_16212	3.128	weakly similar to (147)AT3G04930| Symbols: | transcription regulator | chr3:1363035-1364405 FORWARD no original description	-1.990
JCVI_41053	3.124	moderately similar to (453)AT2G19070| Symbols: | transferase family protein | chr2:8267267-8269040 REVERSEweakly similar to (11	-2.779
EV143849	3.123	no similarity	
EV210392	3.120	no similarity	
DW998133	3.120	weakly similar to (199)AT5G24290| Symbols: | integral membrane family protein | chr5:8263274-8265621 REVERSE [18977]	
EE519591	3.119	no similarity	
JCVI_1576	3.116	moderately similar to (289)AT5G62320| Symbols: AtMYBCU15, AtMYB99, MYB99 | MYB99 (myb domain protein 99); DNA binding	
EX129137	3.115	moderately similar to (265)AT3G25480| Symbols: | rhodanese-like domain-containing protein | chr3:9236828-9237835 REVERSE [218	
JCVI_36587	3.114	no original description	
JCVI_40406	3.113	moderately similar to (380)AT3G57260| Symbols: PR2, BG2, PR-2, BGL2 | BGL2 (PATHOGENESIS-RELATED PROTEIN 2); glucar	-3.406
JCVI_37272	3.108	no original description	
EV200964	3.105	weakly similar to (133)AT5G16030| Symbols: | similar to unknown protein [Arabidopsis thaliana] (TAIR:AT3G02500.1); similar to hyp	
EE402061	3.103	very weakly similar to (85.9)AT2G45220| Symbols: | pectinesterase family protein | chr2:18651355-18653468 REVERSE [20197] 1 250	-3.032
EV198695	3.103	moderately similar to (286)AT5G16270| Symbols: ATRAD21.3, SYN4 | ATRAD21.3/SYN4 (ARABIDOPSIS HOMOLOG OF RAD21	
JCVI_41616	3.101	moderately similar to (210)AT5G17490| Symbols: RGL3 | RGL3 (RGA-LIKE 3); transcription factor | chr5:5764318-5765889 REVERS	
JCVI_33612	3.100	moderately similar to (390)AT4G34880| Symbols: | amidase family protein | chr4:16615554-16617429 FORWARD no original descript	-1.542
RC_ES968917	3.094	no similarity	-2.288
JCVI_37829	3.092	moderately similar to (271)AT1G72450| Symbols: JAZ6, TIFY11B | JAZ6/TIFY11B (JASMONATE-ZIM-DOMAIN PROTEIN 6) | chr	
JCVI_35502	3.092	moderately similar to (390)AT2G47130| Symbols: | short-chain dehydrogenase/reductase (SDR) family protein | chr2:19356697-193575	-3.656
JCVI_38850	3.090	weakly similar to (143)AT5G03380| Symbols: | heavy-metal-associated domain-containing protein | chr5:832399-834127 REVERSE no	-2.630
JCVI_3099	3.090	moderately similar to (236)AT1G52400| Symbols: BGL1 | BGL1 (BETA-GLUCOSIDASE HOMOLOG 1); hydrolase, hydrolyzing O-gly	
JCVI_18625	3.085	moderately similar to (231)AT5G28300| Symbols: | trihelix DNA-binding protein, putative | chr5:10292793-10295105 REVERSE no or	
DY000098	3.080	weakly similar to (171)AT1G45201| Symbols: | triacylglycerol lipase | chr1:17126329-17129937 FORWARD [18967] 1 408 422	-1.869
EV200903	3.080	moderately similar to (260)AT1G65445| Symbols: | transferase-related | chr1:24319538-24320154 FORWARDvery weakly similar to (9	
ES901375	3.078	no similarity	-2.089
JCVI_11067	3.077	moderately similar to (440)AT2G35860| Symbols: FLA16 | FLA16 (FASCICLIN-LIKE ARABINOGALACTAN PROTEIN 16 PRECUI	
EV092170	3.076	no similarity	
EH429905	3.076	moderately similar to (293)AT5G67360| Symbols: ARA12 | ARA12; subtilase | chr5:26889418-26891691 REVERSE [20767]	-2.831
DY017647	3.070	weakly similar to (135)AT3G17580| Symbols: | similar to unknown protein [Arabidopsis thaliana] (TAIR:AT1G48330.1); similar to unk	
EV076435	3.070	no similarity	
RC_DT317725	3.069	no similarity	
JCVI_41649	3.068	weakly similar to (103)AT4G23550| Symbols: WRKY29 | WRKY29 (WRKY DNA-binding protein 29); transcription factor | chr4:12291	
EX022987	3.063	weakly similar to (128)AT4G34710| Symbols: SPE2, ADC2 | ADC2 (ARGININE DECARBOXYLASE 2) | chr4:16560320-16562455 F	
EX043503	3.059	no similarity	
EE557546	3.059	no similarity	-2.085
JCVI_94	3.046	moderately similar to (424)AT1G02360| Symbols: | chitinase, putative | chr1:472138-473116 REVERSEmoderately similar to (316)CH	-4.385
ES906176	3.046	moderately similar to (254)AT3G08670| Symbols: | similar to unknown protein [Arabidopsis thaliana] (TAIR:AT3G51540.1); similar to	
JCVI_11581	3.046	weakly similar to (103)AT2G15960| Symbols: | unknown protein | chr2:6954435-6954668 FORWARD no original description	-1.966
JCVI_12417	3.044	moderately similar to (384)AT4G26760| Symbols: | microtubule associated protein (MAP65/ASE1) family protein | chr4:13478840-134	
AM061024	3.043	weakly similar to (192)AT4G34770| Symbols: | auxin-responsive family protein | chr4:16591357-16591671 FORWARDweakly similar t	
JCVI_39912	3.042	moderately similar to (271)AT5G57150| Symbols: | basic helix-loop-helix (bHLH) family protein | chr5:23169587-23170519 FORWAR	

JCVI_11016	3.041	weakly similar to (114)AT2G26010| Symbols: PDF1.3 | PDF1.3 (plant defensin 1.3) | chr2:11094489-11094833 FORWARDweakly simi	-3.875
CD828777	3.034	weakly similar to (181)AT5G65020| Symbols: ANNAT2 | ANNAT2 (ANNEXIN ARABIDOPSIS 2); calcium ion binding / calcium-depe	-2.399
JCVI_4539	3.027	moderately similar to (456)AT1G52400| Symbols: BGL1 | BGL1 (BETA-GLUCOSIDASE HOMOLOG 1); hydrolase, hydrolyzing O-gly	
JCVI_19012	3.026	no original description	
JCVI_10825	3.020	moderately similar to (251)AT1G30320| Symbols: | remorin family protein | chr1:10680330-10682834 FORWARD no original descripti	
EV151794	3.015	no similarity	
JCVI_15603	3.012	moderately similar to (367)AT1G20510| Symbols: OPCL1 | OPCL1 (OPC-8:0 COA LIGASE1); 4-coumarate-CoA ligase | chr1:7103929	-1.563
EV036453	3.012	moderately similar to (280)AT5G05260| Symbols: CYP79A2 | CYP79A2 (CYTOCHROME P450 79A2); oxygen binding | chr5:155977	
JCVI_24985	3.012	moderately similar to (222)AT2G31560| Symbols: | similar to unknown protein [Arabidopsis thaliana] (TAIR:AT1G05870.1); similar to	-2.929
EV108215	3.011	no similarity	
JCVI_34321	3.011	weakly similar to (168)AT4G16740| Symbols: ATTPS03 | ATTPS03 (Arabidopsis thaliana terpene synthase 03) | chr4:9407878-9409906	
EX100186	3.001	moderately similar to (355)AT1G15550| Symbols: ATGA3OX1, GA4 | GA4 (GA REQUIRING 4); gibberellin 3-beta-dioxygenase | chr1	
JCVI_33303	3.000	weakly similar to (137)AT2G24100| Symbols: | similar to unknown protein [Arabidopsis thaliana] (TAIR:AT4G30780.1); similar to unn	
JCVI_36934	2.997	highly similar to (523)AT1G08250| Symbols: ADT6 | ADT6 (AROGENATE DEHYDRATASE 6); arogenate dehydratase/ prephenate d	
JCVI_8269	2.996	weakly similar to (166)AT5G26170| Symbols: ATWRKY50, WRKY50 | WRKY50 (WRKY DNA-binding protein 50); transcription fact	-4.054
JCVI_1243	2.996	moderately similar to (329)AT3G20370| Symbols: | meprin and TRAF homology domain-containing protein / MATH domain-containing	
EV190171	2.995	very weakly similar to (97.8)AT3G06070| Symbols: | similar to unknown protein [Arabidopsis thaliana] (TAIR:AT5G19190.1); similar to	
AM388645	2.995	no similarity	-1.820
EV182056	2.995	moderately similar to (348)AT5G55180| Symbols: | glycosyl hydrolase family 17 protein | chr5:22406060-22407776 FORWARDweakly	
EE439069	2.992	no similarity	
JCVI_34923	2.991	no original description	-4.689
JCVI_17196	2.991	no original description	
AM390008	2.990	no similarity	-1.908
EV165776	2.987	very weakly similar to (84.3)AT5G48850| Symbols: | male sterility MS5 family protein | chr5:19822802-19824925 REVERSE [21486] 8	-1.712
JCVI_37265	2.986	highly similar to (744)AT5G17490| Symbols: RGL3 | RGL3 (RGA-LIKE 3); transcription factor | chr5:5764318-5765889 REVERSEhig	
JCVI_37747	2.984	moderately similar to (323)AT3G52370| Symbols: FLA15 | FLA15 (FASCICLIN-LIKE ARABINOGALACTAN PROTEIN 15 PRECUI	
RC_EE550419	2.983	no similarity	
EX122320	2.973	weakly similar to (109)AT2G38310| Symbols: | similar to unknown protein [Arabidopsis thaliana] (TAIR:AT5G05440.1); similar to unn	
EX099059	2.970	moderately similar to (259)AT1G02340| Symbols: RSF1, FBI1, REP1, HFR1 | HFR1 (LONG HYPOCOTYL IN FAR-RED); DNA bind	
JCVI_41947	2.968	no original description	-1.807
CV433909	2.967	no similarity	
EV147545	2.965	weakly similar to (120)AT2G19780| Symbols: | leucine-rich repeat family protein / extensin family protein | chr2:8529913-8531121 REV	
JCVI_32452	2.965	highly similar to (514)AT5G65020| Symbols: ANNAT2 | ANNAT2 (ANNEXIN ARABIDOPSIS 2); calcium ion binding / calcium-depe	-2.192
EX037042	2.964	moderately similar to (290)AT5G04660| Symbols: CYP77A4 | CYP77A4 (cytochrome P450, family 77, subfamily A, polypeptide 4); oxy	
JCVI_19038	2.964	moderately similar to (412)AT3G08030| Symbols: | similar to unknown protein [Arabidopsis thaliana] (TAIR:AT2G41800.1); similar to	-3.028
EV026425	2.964	no similarity	-2.533
ES969072	2.964	no similarity	
JCVI_29933	2.958	very weakly similar to (83.2)AT5G16030| Symbols: | similar to unknown protein [Arabidopsis thaliana] (TAIR:AT3G02500.1); similar to	
JCVI_27847	2.953	no original description	
EV108457	2.951	no similarity	
JCVI_27072	2.947	moderately similar to (264)AT4G34710| Symbols: SPE2, ADC2 | ADC2 (ARGININE DECARBOXYLASE 2) | chr4:16560320-165624	
JCVI_30141	2.947	highly similar to (666)AT2G24850| Symbols: TAT, TAT3 | TAT3 (TYROSINE AMINOTRANSFERASE 3); transaminase | chr2:10590	
JCVI_2695	2.946	weakly similar to (102)AT5G64080| Symbols: | protease inhibitor/seed storage/lipid transfer protein (LTP) family protein | chr5:256627(	-2.124
JCVI_24911	2.946	no original description	-1.944
JCVI_9609	2.945	moderately similar to (232)AT5G48890| Symbols: | transcription factor | chr5:19837579-19838100 FORWARD no original description	
JCVI_348	2.935	moderately similar to (451)AT5G05340| Symbols: | peroxidase, putative | chr5:1579143-1580820 REVERSEmoderately similar to (468	
EV052693	2.934	moderately similar to (207)AT3G12230| Symbols: SCPL14 | SCPL14 (serine carboxypeptidase-like 14); serine carboxypeptidase | chr3:3	
DY024947	2.928	moderately similar to (397)AT3G02170| Symbols: LNG2 | LNG2 (LONGIFOLIA2) | chr3:396215-399400 REVERSE [18971]	
EE418046	2.923	moderately similar to (288)AT5G17490| Symbols: RGL3 | RGL3 (RGA-LIKE 3); transcription factor | chr5:5764318-5765889 REVERS	
JCVI_24797	2.921	moderately similar to (211)AT3G09260| Symbols: PSR3.1, PYK10 | PYK10 (phosphate starvation-response 3.1); hydrolase, hydrolyzing	-4.009
EV203808	2.920	no similarity	
JCVI_8597	2.920	moderately similar to (266)AT2G43570| Symbols: | chitinase, putative | chr2:18083466-18084512 REVERSEmoderately similar to (239	-3.424
ES907950	2.919	weakly similar to (177)AT2G16700| Symbols: ADF5 | ADF5 (ACTIN DEPOLYMERIZING FACTOR 5); actin binding | chr2:7251799-	-2.643
EV152022	2.915	no similarity	
JCVI_2827	2.911	weakly similar to (181)AT4G37240| Symbols: | similar to unknown protein [Arabidopsis thaliana] (TAIR:AT2G23690.1); similar to unn	
EX096121	2.909	moderately similar to (221)AT1G71030| Symbols: ATMYBL2 | ATMYBL2 (Arabidopsis myb-like 2); DNA binding / transcription facto	
EV088366	2.903	moderately similar to (413)AT5G60700| Symbols: | glycosyltransferase family protein 2 | chr5:24419555-24421955 REVERSE [21444]	
EX094418	2.901	very weakly similar to (92.0)AT1G79700| Symbols: | ovule development protein, putative | chr1:29995333-29998551 REVERSE [21823	
CX188662	2.897	no similarity	
JCVI_27288	2.895	moderately similar to (352)AT4G36360| Symbols: BGAL3 | BGAL3 (beta-galactosidase 3); beta-galactosidase | chr4:17176843-1718114	
JCVI_25546	2.891	weakly similar to (161)AT3G13790| Symbols: ATCWINV1, ATBFRUCT1 | ATBFRUCT1/ATCWINV1 (ARABIDOPSIS THALIANA	-2.928
EV011577	2.890	no similarity	
EX127924	2.886	moderately similar to (255)AT5G12050| Symbols: | similar to unnamed protein product [Vitis vinifera] (GB:CAO45643.1) | chr5:38902	
JCVI_1544	2.881	very weakly similar to (83.2)AT1G52030| Symbols: MBP1.2, F-ATMBP, MBP2 | MBP2 (MYROSINASE-BINDING PROTEIN 2) | chr	
JCVI_1568	2.878	moderately similar to (385)AT5G15650| Symbols: RGP2 | RGP2 (REVERSIBLY GLYCOSYLATED POLYPEPTIDE 2); alpha-1,4-glu	
H74394	2.877	weakly similar to (115)AT1G12630| Symbols: | DNA binding / transcription activator/ transcription factor | chr1:4298895-4299473 FOR	
ES997334	2.876	weakly similar to (136)AT5G65020| Symbols: ANNAT2 | ANNAT2 (ANNEXIN ARABIDOPSIS 2); calcium ion binding / calcium-depe	
JCVI_7250	2.875	no original description	
JCVI_21352	2.873	moderately similar to (282)AT5G10130| Symbols: | pollen Ole e 1 allergen and extensin family protein | chr5:3171552-3172429 REVEF	-2.289
JCVI_12473	2.868	weakly similar to (119)AT2G18790| Symbols: HY3, OOP1, PHYB | PHYB (PHYTOCHROME B); G-protein coupled photoreceptor/ sig	
EV204309	2.864	moderately similar to (311)AT4G08850| Symbols: | leucine-rich repeat family protein / protein kinase family protein | chr4:5637464-564	-3.713
JCVI_14440	2.861	highly similar to (874)AT2G45220| Symbols: | pectinesterase family protein | chr2:18651355-18653468 REVERSEhighly similar to (62	-2.644
JCVI_37818	2.860	no original description	
JCVI_25345	2.857	highly similar to (726)AT3G09560| Symbols: | lipin family protein | chr3:2934958-2938678 REVERSE no original description	
BQ704241	2.856	moderately similar to (301)AT3G05580| Symbols: | serine/threonine protein phosphatase, putative | chr3:1618222-1619856 REVERSEn	-2.271
L37977	2.851	weakly similar to (109)AT4G39700| Symbols: | heavy-metal-associated domain-containing protein / copper chaperone (CCH)-related | ch	
JCVI_19085	2.850	moderately similar to (397)AT5G26280| Symbols: | meprin and TRAF homology domain-containing protein / MATH domain-containing	
JCVI_4867	2.849	moderately similar to (235)AT1G70690| Symbols: | kinase-related | chr1:26655762-26657044 FORWARD no original description	-2.670
CD835093	2.848	very weakly similar to (100)AT3G20960| Symbols: CYP705A33 | CYP705A33 (cytochrome P450, family 705, subfamily A, polypeptide	
JCVI_10532	2.848	no original description	-1.640

JCVI_24963	2.847	weakly similar to (113)AT1G79380| Symbols: | copine-related | chr1:29865705-29867916 FORWARD no original description	-1.955
JCVI_719	2.847	moderately similar to (415)AT4G39840| Symbols: | similar to unnamed protein product [Vitis vinifera] (GB:CAO21162.1); similar to un	-1.379
EV192767	2.844	weakly similar to (176)AT5G14120| Symbols: | nodulin family protein | chr5:4556310-4558449 FORWARD [21489]	
JCVI_90	2.841	moderately similar to (415)AT2G43590| Symbols: | chitinase, putative | chr2:18088669-18089826 REVERSEmoderately similar to (41	-2.804
CV546025	2.839	very weakly similar to (91.3)AT2G43590| Symbols: | chitinase, putative | chr2:18088669-18089826 REVERSEweakly similar to (106)C	-2.766
EV091684	2.836	moderately similar to (236)AT4G19810| Symbols: | glycosyl hydrolase family 18 protein | chr4:10764161-10765763 REVERSE [21476	-2.801
EV152290	2.835	no similarity	
CV650527	2.833	weakly similar to (103)AT5G53290| Symbols: CRF3 | CRF3 (CYTOKININ RESPONSE FACTOR 3); DNA binding / transcription facto	
EX121577	2.830	weakly similar to (190)AT1G70130| Symbols: | lectin protein kinase, putative | chr1:26413406-26415464 REVERSE [21829] 32 521 52	
CV545070	2.827	no similarity	
EE568494	2.827	no similarity	
JCVI_35958	2.826	highly similar to (683)AT1G13080| Symbols: CYP71B2 | CYP71B2 (CYTOCHROME P450 71B2); oxygen binding | chr1:4459491-44	
JCVI_40470	2.826	weakly similar to (115)AT1G58225| Symbols: | unknown protein | chr1:21567870-21568660 FORWARD no original description	
JCVI_10592	2.825	moderately similar to (377)AT1G29660| Symbols: | GDSL-motif lipase/hydrolase family protein | chr1:10371941-10373610 FORWARI	
EE459992	2.822	weakly similar to (138)AT4G23600| Symbols: JR2, CORI3 | CORI3 (CORONATINE INDUCED 1, JASMONIC ACID RESPONSIVE	
EE434960	2.815	no similarity	
CX192285	2.814	no similarity	
JCVI_34165	2.814	moderately similar to (417)AT5G06570| Symbols: | hydrolase | chr5:2008076-2011014 REVERSEweakly similar to (121)GID1_ORYS	-3.177
JCVI_26738	2.813	moderately similar to (342)AT5G06700| Symbols: | similar to unknown protein [Arabidopsis thaliana] (TAIR:AT3G12060.1); similar to	
JCVI_39138	2.809	weakly similar to (145)AT5G50760| Symbols: | auxin-responsive family protein | chr5:20662006-20662557 FORWARD no original des	-2.191
ES909147	2.809	moderately similar to (461)AT2G26650| Symbols: AKT1 | AKT1 (ARABIDOPSIS K TRANSPORTER 1); cyclic nucleotide binding / in	
JCVI_23917	2.809	weakly similar to (103)AT5G65020| Symbols: ANNAT2 | ANNAT2 (ANNEXIN ARABIDOPSIS 2); calcium ion binding / calcium-depe	-1.843
AM385928	2.807	weakly similar to (103)AT5G26600| Symbols: | catalytic/ pyridoxal phosphate binding | chr5:9377458-9378885 FORWARD [20118]	
EH423228	2.802	moderately similar to (296)AT1G29720| Symbols: | protein kinase family protein | chr1:10393880-10395067 REVERSEvery weakly sim	
EV182480	2.800	very weakly similar to (97.8)AT5G19600| Symbols: SULTR3;5 | SULTR3;5; sulfate transmembrane transporter | chr5:6613115-6616893	
EG019285	2.799	no similarity	
EV124150	2.797	very weakly similar to (87.8)AT5G42650| Symbols: CYP74A, AOS | AOS (ALLENE OXIDE SYNTHASE); hydro-lyase/ oxygen binding	
JCVI_11710	2.797	moderately similar to (356)AT1G11790| Symbols: ADT1 | ADT1 (AROGENATE DEHYDRATASE 1); prephenate dehydratase | chr1:3	
JCVI_32230	2.796	moderately similar to (361)AT1G76790| Symbols: | O-methyltransferase family 2 protein | chr1:28827249-28828524 REVERSEweakly	
JCVI_33924	2.794	moderately similar to (465)AT5G60890| Symbols: ATMYB34, ATR1, MYB34 | ATMYB34/ATR1/MYB34 (ALTERED TRYPTOPHA	
EV020611	2.793	no similarity	
JCVI_15854	2.791	weakly similar to (140)AT2G14610| Symbols: PR-1, PR1 | PR1 (PATHOGENESIS-RELATED GENE 1) | chr2:6249026-6249511 REV	-3.811
JCVI_17030	2.787	highly similar to (690)AT3G22910| Symbols: | calcium-transporting ATPase, plasma membrane-type, putative / Ca(2+)-ATPase, putative	-1.614
JCVI_22844	2.785	weakly similar to (156)AT1G70700| Symbols: JAZ9, TIFY7 | JAZ9/TIFY7 (JASMONATE-ZIM-DOMAIN PROTEIN 9) | chr1:266586	
JCVI_38817	2.782	moderately similar to (243)AT2G14100| Symbols: CYP705A13 | CYP705A13 (cytochrome P450, family 705, subfamily A, polypeptide	
ES904127	2.781	no similarity	
JCVI_33051	2.780	weakly similar to (158)AT2G24100| Symbols: | similar to unknown protein [Arabidopsis thaliana] (TAIR:AT4G30780.1); similar to unn	
JCVI_26368	2.780	moderately similar to (469)AT5G24530| Symbols: | oxidoreductase, 2OG-Fe(II) oxygenase family protein | chr5:8378967-8383157 FOR	-3.118
EE464285	2.777	no similarity	-2.383
JCVI_11780	2.775	moderately similar to (351)AT5G01210| Symbols: | transferase family protein | chr5:84553-85980 FORWARD no original description	
EV127161	2.774	no similarity	
EV227118	2.771	weakly similar to (115)AT2G45220| Symbols: | pectinesterase family protein | chr2:18651355-18653468 REVERSEvery weakly similar	-4.063
JCVI_9441	2.770	moderately similar to (455)AT3G57260| Symbols: PR2, BG2, PR-2, BGL2 | BGL2 (PATHOGENESIS-RELATED PROTEIN 2); glucar	-3.736
ES902099	2.769	moderately similar to (261)AT5G25160| Symbols: ZFP3 | ZFP3 (ZINC FINGER PROTEIN 3); nucleic acid binding / transcription factor	
ES938016	2.767	no similarity	-1.751
EE568940	2.766	weakly similar to (112)AT5G64570| Symbols: ATBXL4, XYL4 | XYL4 (beta-xylosidase 4); hydrolase, hydrolyzing O-glycosyl compour	
JCVI_29376	2.759	weakly similar to (126)AT5G50340| Symbols: | ATP binding / ATP-dependent peptidase/ damaged DNA binding / nucleoside-triphospha	
JCVI_53	2.755	moderately similar to (378)AT3G12920| Symbols: | protein binding / zinc ion binding | chr3:4122134-4123330 REVERSE no original d	-1.753
EV221600	2.750	no similarity	
CX194419	2.748	no similarity	
JCVI_33932	2.747	moderately similar to (326)AT5G48930| Symbols: HCT | transferase family protein | chr5:19853880-19855318 REVERSEweakly simila	
JCVI_11612	2.745	highly similar to (752)AT2G35020| Symbols: | UTP--glucose-1-phosphate uridylyltransferase family protein | chr2:14763882-14767556	
JCVI_33142	2.743	weakly similar to (104)AT2G40330| Symbols: | Bet v I allergen family protein | chr2:16852255-16852902 REVERSE no original descri	
JCVI_22851	2.743	moderately similar to (255)AT5G06860| Symbols: PGIP1 | PGIP1 (POLYGALACTURONASE INHIBITING PROTEIN 1); protein bin	
JCVI_1123	2.741	moderately similar to (408)AT1G04680| Symbols: | pectate lyase family protein | chr1:1304051-1307779 REVERSEmoderately similar	
ES906229	2.741	moderately similar to (357)AT1G08080| Symbols: | carbonic anhydrase family protein | chr1:2517019-2518543 REVERSE [21429]	
JCVI_18116	2.740	highly similar to (585)AT1G73600| Symbols: | phosphoethanolamine N-methyltransferase | chr1:27674486-27677061 FORWARDhighl	
EX108062	2.737	no similarity	-2.315
EV224402	2.736	no similarity	
EV101041	2.735	moderately similar to (313)AT5G44260| Symbols: | zinc finger (CCCH-type) family protein | chr5:17847201-17848346 REVERSE [214	
JCVI_40012	2.730	moderately similar to (437)AT1G26390| Symbols: | FAD-binding domain-containing protein | chr1:9130151-9131743 REVERSE no ori	-3.042
CO750295	2.730	weakly similar to (183)AT5G57800| Symbols: FLP1, YRE, CER3, WAX2 | CER3/FLP1/WAX2/YRE (ECERIFERUM 3); catalytic | chr	
CV432215	2.730	no similarity	
JCVI_34585	2.730	moderately similar to (306)AT3G10570| Symbols: CYP77A6 | CYP77A6 (cytochrome P450, family 77, subfamily A, polypeptide 6); ox	
EV160033	2.729	moderately similar to (346)AT1G11600| Symbols: CYP77B1 | CYP77B1 (cytochrome P450, family 77, subfamily B, polypeptide 1); ox	-1.903
EE444480	2.727	weakly similar to (171)AT3G52890| Symbols: KIPK | KIPK (KCBP-INTERACTING PROTEIN KINASE); kinase | chr3:19620128-196	
EE428698	2.727	weakly similar to (122)AT5G42650| Symbols: CYP74A, AOS | AOS (ALLENE OXIDE SYNTHASE); hydro-lyase/ oxygen binding | ch	
ES908931	2.726	no similarity	
JCVI_38452	2.724	weakly similar to (108)AT4G08850| Symbols: | leucine-rich repeat family protein / protein kinase family protein | chr4:5637464-564049	-3.320
JCVI_27967	2.722	moderately similar to (202)AT2G23610| Symbols: | esterase, putative | chr2:10051490-10053483 REVERSEweakly similar to (175)PIF	-3.473
EE567014	2.722	weakly similar to (106)AT2G17060| Symbols: | disease resistance protein (TIR-NBS-LRR class), putative | chr2:7429493-7433959 FOR	
JCVI_40281	2.722	moderately similar to (380)AT1G22360| Symbols: ATUGT85A2 | ATUGT85A2 (UDP-GLUCOSYL TRANSFERASE 85A2); UDP-gly	
CN727537	2.720	no similarity	-3.245
JCVI_20790	2.720	moderately similar to (216)AT1G49870| Symbols: | similar to MBD10 (methyl-CpG-binding domain 10), DNA binding [Arabidopsis tha	
JCVI_833	2.720	moderately similar to (499)AT1G78850| Symbols: | curculin-like (mannose-binding) lectin family protein | chr1:29646965-29648290 RI	-3.189
CX272791	2.719	no similarity	
JCVI_27218	2.719	moderately similar to (272)AT3G08670| Symbols: | similar to unknown protein [Arabidopsis thaliana] (TAIR:AT3G51540.1); similar to	
JCVI_3761	2.719	moderately similar to (276)AT3G30530| Symbols: ATBZIP42 | ATBZIP42 (ARABIDOPSIS THALIANA BASIC LEUCINE-ZIPPER);	
JCVI_13130	2.716	moderately similar to (447)AT2G24100| Symbols: | similar to unknown protein [Arabidopsis thaliana] (TAIR:AT4G30780.1); similar to	
JCVI_1219	2.712	no original description	-3.163

JCVI_649	2.710	highly similar to (693)AT3G01420| Symbols: DOX1, ALPHA-DOX1 | ALPHA-DOX1 (ALPHA-DIOXYGENASE 1) | chr3:159696-162	
EV170929	2.708	moderately similar to (292)AT3G28740| Symbols: CYP81D1 | cytochrome P450 family protein | chr3:10790001-10791789 REVERSEw	
JCVI_19080	2.708	no original description	-4.038
JCVI_3351	2.703	moderately similar to (306)AT4G37010| Symbols: | caltractin, putative / centrin, putative | chr4:17444309-17445374 FORWARDweakly	-1.853
JCVI_22812	2.702	moderately similar to (473)AT2G39640| Symbols: | glycosyl hydrolase family 17 protein | chr2:16532249-16534090 REVERSEmoderat	
RC_EX050641	2.701	no similarity	
JCVI_18475	2.700	moderately similar to (287)AT1G29330| Symbols: ATERD2, AERD2, ERD2 | ERD2 (ER lumen protein retaining receptor 2); receptor |	
EX053157	2.699	no similarity	
EX131086	2.692	no similarity	
ES903072	2.692	moderately similar to (211)AT4G00520| Symbols: | acyl-CoA thioesterase family protein | chr4:227726-231249 FORWARD [21432]	
JCVI_16208	2.690	no original description	
JCVI_9701	2.687	moderately similar to (427)AT2G29320| Symbols: | tropinone reductase, putative / tropine dehydrogenase, putative | chr2:12599257-126	
EE559228	2.685	no similarity	
JCVI_336	2.684	moderately similar to (426)AT3G26520| Symbols: SITIP, GAMMA-TIP2, TIP1;2, TIP2 | TIP2 (TONOPLAST INTRINSIC PROTEIN 2	
DY017708	2.680	weakly similar to (126)AT2G47240| Symbols: | long-chain-fatty-acid--CoA ligase family protein / long-chain acyl-CoA synthetase family	-1.557
JCVI_30822	2.677	weakly similar to (159)AT1G16860| Symbols: | merozoite surface protein-related | chr1:5768273-5770176 FORWARD no original desc	
JCVI_16583	2.674	weakly similar to (128)AT3G04720| Symbols: HEL, PR-4, PR4 | PR4 (PATHOGENESIS-RELATED 4) | chr3:1285697-1286537 REVE	-3.333
EV213225	2.673	no similarity	
JCVI_3342	2.673	moderately similar to (333)AT3G17390| Symbols: SAMS3, MAT4, MTO3 | MTO3 (S-adenosylmethionine synthase 3); methionine ader	
JCVI_41367	2.672	no original description	
JCVI_2523	2.672	highly similar to (532)AT3G03520| Symbols: | phosphoesterase family protein | chr3:837979-840518 REVERSE no original description	
JCVI_29895	2.670	no original description	
EV225720	2.669	weakly similar to (102)AT2G15960| Symbols: | unknown protein | chr2:6954435-6954668 FORWARD [21493] 1 612 807	
JCVI_40923	2.667	weakly similar to (112)AT2G46535| Symbols: | similar to unknown protein [Arabidopsis thaliana] (TAIR:AT3G61840.1) | chr2:1911677	
ES995767	2.657	very weakly similar to (94.0)AT2G04925| Symbols: | Encodes a defensin-like (DEFL) family protein. | chr2:1729762-1730138 REVERS	-3.421
EV177649	2.651	no similarity	
ES929854	2.649	moderately similar to (322)AT3G48770| Symbols: | ATP binding / DNA binding | chr3:18090246-18097802 REVERSE [20185] 1 626	
CN827085	2.646	moderately similar to (438)AT1G19300| Symbols: GATL1, PARVUS, GLZ1 | GATL1/GLZ1/PARVUS (GALACTURONOSYLTRANS	-1.584
JCVI_13559	2.644	moderately similar to (354)AT4G23590| Symbols: | aminotransferase class I and II family protein | chr4:12307205-12309455 FORWAR	
RC_H07804	2.640	no similarity	
EV035753	2.637	weakly similar to (108)AT1G29790| Symbols: | similar to (ARABIDOPSIS THALIANA RAS ASSOCIATED WITH DIABETES PRO	-1.753
EE404027	2.635	moderately similar to (276)AT5G28300| Symbols: | trihelix DNA-binding protein, putative | chr5:10292793-10295105 REVERSE [168	
JCVI_29684	2.630	moderately similar to (410)AT5G57830| Symbols: | similar to unknown protein [Arabidopsis thaliana] (TAIR:AT4G30830.1); similar to	-2.815
JCVI_1200	2.628	moderately similar to (431)AT5G16030| Symbols: | similar to unknown protein [Arabidopsis thaliana] (TAIR:AT3G02500.1); similar to	
JCVI_39389	2.628	moderately similar to (340)AT4G14950| Symbols: | similar to unknown protein [Arabidopsis thaliana] (TAIR:AT1G05360.1); similar to	-1.990
EV223461	2.628	moderately similar to (271)AT3G14840| Symbols: | leucine-rich repeat family protein / protein kinase family protein | chr3:4988278-499	-2.890
EE440908	2.624	moderately similar to (318)AT5G12010| Symbols: | similar to unknown protein [Arabidopsis thaliana] (TAIR:AT4G29780.1); similar to	
JCVI_40157	2.620	moderately similar to (281)AT1G53040| Symbols: | hydrolase, acting on carbon-nitrogen (but not peptide) bonds, in linear amides | chr1:	
JCVI_2678	2.619	moderately similar to (287)AT5G67180| Symbols: | AP2 domain-containing transcription factor, putative | chr5:26819345-26821160 RE	
CN728748	2.617	weakly similar to (103)AT1G06980| Symbols: | similar to unknown protein [Arabidopsis thaliana] (TAIR:AT2G30230.1); similar to unn	
JCVI_8980	2.616	moderately similar to (280)AT3G23820| Symbols: GAE6 | GAE6 (UDP-D-GLUCURONATE 4-EPIMERASE 6); catalytic | chr3:86036	-2.085
JCVI_4219	2.615	moderately similar to (373)AT4G17460| Symbols: HAT1 | HAT1 (homeobox-leucine zipper protein 1); DNA binding / transcription facto	
EV097689	2.614	weakly similar to (165)AT1G71030| Symbols: ATMYBL2 | ATMYBL2 (Arabidopsis myb-like 2); DNA binding / transcription factor | cl	
CD842525	2.613	very weakly similar to (94.7)AT4G18980| Symbols: | similar to unknown protein [Arabidopsis thaliana] (TAIR:AT5G45630.1); similar to	
EX094338	2.613	weakly similar to (122)AT5G52540| Symbols: | similar to unknown protein [Arabidopsis thaliana] (TAIR:AT5G24000.1); similar to unn	
JCVI_8581	2.612	moderately similar to (349)AT1G52400| Symbols: BGL1 | BGL1 (BETA-GLUCOSIDASE HOMOLOG 1); hydrolase, hydrolyzing O-gl	
JCVI_26682	2.610	moderately similar to (335)AT4G01230| Symbols: | reticulon family protein (RTNLB7) | chr4:516264-517408 REVERSE no original de	
JCVI_35521	2.610	moderately similar to (305)AT3G09270| Symbols: ATGSTU8 | ATGSTU8 (Arabidopsis thaliana Glutathione S-transferase (class tau) 8);	
EV205372	2.609	weakly similar to (196)AT5G04020| Symbols: | calmodulin-binding protein-related (PICBP) | chr5:1081979-1086545 REVERSE [2149	-2.505
JCVI_160	2.607	moderately similar to (363)AT2G38310| Symbols: | similar to unknown protein [Arabidopsis thaliana] (TAIR:AT5G05440.1); similar to	
JCVI_23115	2.606	no original description	-2.595
EX140350	2.606	no similarity	-2.420
JCVI_12573	2.604	moderately similar to (337)AT1G69930| Symbols: ATGSTU11 | ATGSTU11 (Arabidopsis thaliana Glutathione S-transferase (class tau)	
JCVI_18841	2.602	weakly similar to (170)AT5G20740| Symbols: | invertase/pectin methylesterase inhibitor family protein | chr5:7025869-7026486 REVEI	
EE546532	2.601	very weakly similar to (99.0)AT4G02390| Symbols: APP | APP (ARABIDOPSIS POLY(ADP-RIBOSE) POLYMERASE); NAD+ ADP-	
JCVI_19720	2.600	no original description	-1.986
JCVI_40667	2.600	moderately similar to (353)AT5G12340| Symbols: | similar to unknown protein [Arabidopsis thaliana] (TAIR:AT1G28190.1); similar to	
JCVI_11056	2.598	moderately similar to (256)AT3G07390| Symbols: AIR12 | AIR12 (Auxin-Induced in Root cultures 12); extracellular matrix structural co	
EV129942	2.596	no similarity	
EV110950	2.594	weakly similar to (146)AT1G12200| Symbols: | flavin-containing monooxygenase family protein / FMO family protein | chr1:4137625-4	-3.743
EE472763	2.592	no similarity	
AM388033	2.591	no similarity	
JCVI_3049	2.590	no original description	
JCVI_37660	2.589	nearly identical (1130)AT5G64740| Symbols: CESA6, IXR2, E112, PRC1 | CESA6 (CELLULOSE SYNTHASE 6); transferase, transfer	
EX085395	2.584	no similarity	
JCVI_29316	2.581	weakly similar to (155)AT2G15440| Symbols: | similar to unknown protein [Arabidopsis thaliana] (TAIR:AT5G67210.1); similar to unn	
JCVI_20557	2.580	moderately similar to (381)AT5G53660| Symbols: AtGRF7 | AtGRF7 (GROWTH-REGULATING FACTOR 7) | chr5:21811862-21813	-2.402
AM059003	2.579	weakly similar to (182)AT1G12890| Symbols: | AP2 domain-containing transcription factor, putative | chr1:4391732-4392391 FORWAI	-2.544
EV102843	2.575	weakly similar to (137)AT3G13360| Symbols: WIP3 | WIP3 (WPP-DOMAIN INTERACTING PROTEIN 3) | chr3:4338479-4339989 R	
JCVI_32893	2.575	moderately similar to (238)AT5G20670| Symbols: | similar to unknown protein [Arabidopsis thaliana] (TAIR:AT1G72510.1); similar to	
JCVI_1670	2.574	moderately similar to (452)AT1G09480| Symbols: | cinnamyl-alcohol dehydrogenase family / CAD family | chr1:3057977-3060663 FOI	
JCVI_16582	2.572	moderately similar to (230)AT5G58310| Symbols: | hydrolase, alpha/beta fold family protein | chr5:23592353-23593233 REVERSE no	
JCVI_380	2.572	moderately similar to (321)AT5G62350| Symbols: | invertase/pectin methylesterase inhibitor family protein / DC 1.2 homolog (FL5-2I22	
EV207596	2.572	no similarity	
EE524405	2.571	no similarity	
EX126359	2.568	moderately similar to (456)AT3G02170| Symbols: LNG2 | LNG2 (LONGIFOLIA2) | chr3:396215-399400 REVERSE [21831]	
EE561948	2.566	weakly similar to (130)AT2G41730| Symbols: | similar to unknown protein [Arabidopsis thaliana] (TAIR:AT5G24640.1); similar to unn	
EE560578	2.566	no similarity	-1.984
JCVI_11661	2.565	moderately similar to (281)AT1G20970| Symbols: | adhesin-related | chr1:7314327-7319235 FORWARD no original description	
JCVI_30017	2.564	no original description	-3.167

EV004616	2.564	weakly similar to (150)AT5G03700| Symbols: | PAN domain-containing protein | chr5:965873-967321 REVERSE [21427]	
JCVI_36228	2.559	weakly similar to (186)AT3G53850| Symbols: | Identical to UPF0497 membrane protein At3g53850 [Arabidopsis Thaliana] (GB:Q945M	
JCVI_1046	2.559	no original description	-2.318
JCVI_4075	2.547	moderately similar to (286)AT2G32280| Symbols: | similar to unknown protein [Arabidopsis thaliana] (TAIR:AT4G21310.1); similar to	
JCVI_28171	2.544	highly similar to (620)AT3G55350| Symbols: | similar to unknown protein [Arabidopsis thaliana] (TAIR:AT3G63270.1); similar to unna	
JCVI_1368	2.544	moderately similar to (408)AT3G25780| Symbols: AOC3 | AOC3 (ALLENE OXIDE CYCLASE 3) | chr3:9410599-9411646 FORWAR	-1.564
EX024132	2.541	moderately similar to (283)AT3G13790| Symbols: ATCWINV1, ATBFRUCT1 | ATBFRUCT1/ATCWINV1 (ARABIDOPSIS THALIA	
JCVI_4584	2.540	weakly similar to (197)AT4G28140| Symbols: | AP2 domain-containing transcription factor, putative | chr4:13974911-13975789 REVE	
JCVI_28312	2.540	no original description	-1.739
JCVI_24598	2.539	highly similar to (835)AT3G61280| Symbols: | similar to unknown protein [Arabidopsis thaliana] (TAIR:AT3G61290.1); similar to unna	-3.601
JCVI_1785	2.537	highly similar to (504)AT4G37870| Symbols: PCK1, PEPCK | PCK1/PEPCK (PHOSPHOENOLPYRUVATE CARBOXYKINASE 1);	-1.740
ES269414	2.536	moderately similar to (243)AT1G28030| Symbols: | oxidoreductase, 2OG-Fe(II) oxygenase family protein | chr1:9771780-9773332 FOR	
ES988944	2.533	weakly similar to (177)AT3G51740| Symbols: IMK2 | IMK2 (INFLORESCENCE MERISTEM RECEPTOR-LIKE KINASE 2); ATP bi	-2.190
H07491	2.530	no similarity	-3.507
EX043451	2.530	weakly similar to (136)AT3G21230| Symbols: 4CL5 | 4CL5 (4-COUMARATE:COA LIGASE 5); 4-coumarate-CoA ligase | chr3:744823	
JCVI_26437	2.529	moderately similar to (341)AT1G14130| Symbols: | 2-oxoglutarate-dependent dioxygenase, putative | chr1:4836038-4837037 REVERSI	
JCVI_4674	2.528	moderately similar to (384)AT1G76160| Symbols: SKS5 | SKS5 (SKU5 Similar 5); copper ion binding / oxidoreductase | chr1:28583105	
JCVI_16605	2.527	weakly similar to (194)AT3G17840| Symbols: RLK902 | RLK902 (receptor-like kinase 902); ATP binding / kinase/ protein serine/threon	
JCVI_3128	2.527	moderately similar to (310)AT4G15630| Symbols: | integral membrane family protein | chr4:8917525-8918681 FORWARD no original	
JCVI_16777	2.525	moderately similar to (226)AT1G28190| Symbols: | similar to unknown protein [Arabidopsis thaliana] (TAIR:AT5G12340.1); similar to	-3.147
JCVI_13020	2.523	weakly similar to (108)AT3G52360| Symbols: | similar to unknown protein [Arabidopsis thaliana] (TAIR:AT2G35850.1); similar to unn	
JCVI_27782	2.522	weakly similar to (112)AT5G04020| Symbols: | calmodulin-binding protein-related (PICBP) | chr5:1081979-1086545 REVERSE no ori	-2.417
JCVI_40837	2.521	very weakly similar to (91.7)AT2G14610| Symbols: PR-1, PR1 | PR1 (PATHOGENESIS-RELATED GENE 1) | chr2:6249026-6249511	-3.738
JCVI_22832	2.519	very weakly similar to (88.6)AT5G55450| Symbols: | protease inhibitor/seed storage/lipid transfer protein (LTP) family protein | chr5:224	-3.418
JCVI_18884	2.512	moderately similar to (363)AT2G38110| Symbols: ATGPAT6, GPAT6 | ATGPAT6/GPAT6 (GLYCEROL-3-PHOSPHATE ACYLTRA	
DY029163	2.510	no similarity	
EE447848	2.509	moderately similar to (205)AT3G08670| Symbols: | similar to unknown protein [Arabidopsis thaliana] (TAIR:AT3G51540.1); similar to	
JCVI_31226	2.509	weakly similar to (146)AT2G36220| Symbols: | similar to unknown protein [Arabidopsis thaliana] (TAIR:AT3G52710.1); similar to unn	
JCVI_37866	2.508	no original description	
JCVI_14560	2.508	moderately similar to (337)AT1G59700| Symbols: ATGSTU16 | ATGSTU16 (Arabidopsis thaliana Glutathione S-transferase (class tau)	-2.014
EX108655	2.503	no similarity	
JCVI_35751	2.501	moderately similar to (352)AT5G21950| Symbols: | hydrolase, alpha/beta fold family protein | chr5:7254070-7255869 REVERSE no ori	
JCVI_40979	2.500	very weakly similar to (100)AT3G20340| Symbols: | Expression of the gene is downregulated in the presence of paraquat, an inducer of	
JCVI_34965	2.494	no original description	-3.406
JCVI_37280	2.492	highly similar to (538)AT5G47240| Symbols: ATNUDT8 | ATNUDT8 (Arabidopsis thaliana Nudix hydrolase homolog 8); hydrolase | ch	
JCVI_39429	2.492	no original description	
JCVI_1235	2.491	moderately similar to (348)AT2G02930| Symbols: GST16, ATGSTF3 | ATGSTF3 (GLUTATHIONE S-TRANSFERASE 16); glutathion	
JCVI_10776	2.491	highly similar to (781)AT1G26420| Symbols: | FAD-binding domain-containing protein | chr1:9141702-9143291 REVERSE no original	-3.395
EH413811	2.490	moderately similar to (214)AT1G35710| Symbols: | leucine-rich repeat transmembrane protein kinase, putative | chr1:13222152-132225	-3.636
JCVI_18106	2.488	moderately similar to (251)AT4G37150| Symbols: | esterase, putative | chr4:17492979-17494051 REVERSEweakly similar to (113)PIF	
JCVI_25217	2.486	no original description	
EX131018	2.484	no similarity	
EE552684	2.482	moderately similar to (309)AT1G44130| Symbols: | nucellin protein, putative | chr1:16789948-16791758 REVERSEweakly similar to (	-2.325
JCVI_18242	2.482	moderately similar to (260)AT5G47240| Symbols: ATNUDT8 | ATNUDT8 (Arabidopsis thaliana Nudix hydrolase homolog 8); hydrolas	
JCVI_8504	2.480	moderately similar to (281)AT5G64250| Symbols: | 2-nitropropane dioxygenase family / NPD family | chr5:25714849-25716171 REVE	-3.904
JCVI_26529	2.478	moderately similar to (249)AT2G16660| Symbols: | nodulin family protein | chr2:7226012-7228674 REVERSE no original description	
AM060364	2.478	no similarity	-3.863
EE542279	2.478	no similarity	-2.198
EE472437	2.477	no similarity	
JCVI_19231	2.476	moderately similar to (218)AT5G01750| Symbols: | Identical to Uncharacterized protein At5g01750 [Arabidopsis Thaliana] (GB:Q9LZX	-2.498
JCVI_1570	2.475	moderately similar to (495)AT3G57010| Symbols: | strictosidine synthase family protein | chr3:21106833-21108295 REVERSEweakly s	
EV216530	2.474	no similarity	
JCVI_3798	2.474	no original description	-2.742
JCVI_42255	2.471	no original description	
EV162102	2.471	weakly similar to (190)AT1G17745| Symbols: PGDH | PGDH (3-PHOSPHOGLYCERATE DEHYDROGENASE); phosphoglycerate de	-2.960
H07394	2.469	no similarity	-1.755
AM394725	2.468	no similarity	
JCVI_9835	2.468	moderately similar to (227)AT5G20670| Symbols: | similar to unknown protein [Arabidopsis thaliana] (TAIR:AT1G72510.1); similar to	
JCVI_24589	2.465	moderately similar to (306)AT5G04820| Symbols: ATOFP13, OFP13 | ATOFP13/OFP13 (Arabidopsis thaliana ovate family protein 13)	
JCVI_25299	2.459	moderately similar to (291)AT2G42320| Symbols: | nucleolar protein gar2-related | chr2:17635180-17637735 FORWARD no original d	
ES912376	2.458	no similarity	
EV129295	2.458	no similarity	
EE532091	2.457	moderately similar to (221)AT1G22590| Symbols: AGL87 | AGL87 | chr1:7983500-7983991 FORWARD [20175] 1 562 590	
JCVI_12008	2.453	moderately similar to (248)AT1G11840| Symbols: ATGLX1 | ATGLX1 (GLYOXALASE I HOMOLOG); lactoylglutathione lyase | chr1	
JCVI_10096	2.453	weakly similar to (193)AT1G23730| Symbols: | carbonic anhydrase, putative / carbonate dehydratase, putative | chr1:8395954-8398003	-3.482
EV174240	2.452	no similarity	
JCVI_16332	2.450	highly similar to (516)AT2G24170| Symbols: | endomembrane protein 70, putative | chr2:10281387-10283974 REVERSE no original de	-2.053
JCVI_1850	2.450	moderately similar to (348)AT1G64980| Symbols: | similar to unnamed protein product [Vitis vinifera] (GB:CAO62125.1); contains dor	
EE420681	2.447	moderately similar to (201)AT4G19120| Symbols: ERD3 | ERD3 (EARLY-RESPONSIVE TO DEHYDRATION 3) | chr4:10460676-10	-1.958
JCVI_21734	2.446	highly similar to (965)AT3G48770| Symbols: | ATP binding / DNA binding | chr3:18090246-18097802 REVERSE no original descripti	
JCVI_33205	2.445	moderately similar to (286)AT5G65650| Symbols: | similar to unknown protein [Arabidopsis thaliana] (TAIR:AT4G36660.1); similar to	
JCVI_17147	2.444	weakly similar to (151)AT1G49760| Symbols: PAB8 | PAB8 (POLY(A) BINDING PROTEIN 8); RNA binding / translation initiation fa	
EE472129	2.442	no similarity	
EV219645	2.440	no similarity	
EV212790	2.438	weakly similar to (166)AT1G20970| Symbols: | adhesin-related | chr1:7314327-7319235 FORWARD [21491] 68 776 776	
AM061951	2.437	no similarity	-2.377
EX090893	2.436	moderately similar to (384)AT4G03140| Symbols: | oxidoreductase | chr4:1392191-1393662 FORWARDweakly similar to (186)TS2_N	-1.881
CV433145	2.430	weakly similar to (199)AT5G57150| Symbols: | basic helix-loop-helix (bHLH) family protein | chr5:23169587-23170519 FORWARD [1	
EV146737	2.430	weakly similar to (186)AT5G67180| Symbols: | AP2 domain-containing transcription factor, putative | chr5:26819345-26821160 REVE	
JCVI_25678	2.429	no original description	

EV085095	2.429	no similarity	
EX137648	2.429	moderately similar to (395)AT2G30470| Symbols: HSI2 | HSI2 (HIGH-LEVEL EXPRESSION OF SUGAR-INDUCIBLE GENE 2); tra	
JCVI_34404	2.428	weakly similar to (119)AT4G21920| Symbols: | unknown protein | chr4:11636096-11636485 REVERSE no original description	
JCVI_2917	2.428	moderately similar to (413)AT2G36400| Symbols: AtGRF3 | AtGRF3 (GROWTH-REGULATING FACTOR 3) | chr2:15277379-15279	
EV117829	2.426	weakly similar to (166)AT4G19960| Symbols: | potassium ion transmembrane transporter | chr4:10813817-10817007 FORWARDweakl	
EX021852	2.423	very weakly similar to (96.3)AT3G25720| Symbols: | similar to unknown protein [Arabidopsis thaliana] (TAIR:AT4G10613.1); similar to	
EV089414	2.422	no similarity	
JCVI_22300	2.418	moderately similar to (429)AT1G29720| Symbols: | protein kinase family protein | chr1:10393880-10395067 REVERSEweakly similar t	
JCVI_27410	2.417	moderately similar to (338)AT4G37520| Symbols: | peroxidase 50 (PER50) (P50) (PRXR2) | chr4:17631698-17633054 FORWARDwe	-2.977
EH430551	2.417	weakly similar to (189)AT3G56710| Symbols: SIB1 | SIB1 (SIGMA FACTOR BINDING PROTEIN 1); binding | chr3:21018032-21018	-3.014
ES967672	2.415	no similarity	
CD836329	2.414	no similarity	-2.126
EV155823	2.414	weakly similar to (101)AT3G10120| Symbols: | similar to unknown protein [Arabidopsis thaliana] (TAIR:AT5G03890.1); similar to hyp	
JCVI_21987	2.413	no original description	-2.368
JCVI_17459	2.409	weakly similar to (101)AT4G17670| Symbols: | senescence-associated protein-related | chr4:9833961-9834676 REVERSE no original d	-2.219
EX120674	2.407	weakly similar to (134)AT4G23160| Symbols: | protein kinase family protein | chr4:12129496-12134097 FORWARD [21829] 19 518 5	-3.292
AM062438	2.407	moderately similar to (269)AT5G64410| Symbols: ATOPT4 | ATOPT4 (oligopeptide transporter 4); oligopeptide transporter | chr5:2576	-2.624
JCVI_31593	2.406	weakly similar to (145)AT4G02390| Symbols: APP | APP (ARABIDOPSIS POLY(ADP-RIBOSE) POLYMERASE); NAD+ ADP-ribos	
JCVI_19564	2.406	weakly similar to (124)AT4G27740| Symbols: | Identical to Protein yippee-like At4g27740 [Arabidopsis Thaliana] (GB:Q2V3E2); simil	-2.127
JCVI_16377	2.402	weakly similar to (167)AT3G05920| Symbols: | heavy-metal-associated domain-containing protein | chr3:1768997-1769528 REVERSE	
DY016194	2.401	no similarity	
JCVI_14825	2.400	no original description	
JCVI_12181	2.397	moderately similar to (432)AT3G03220| Symbols: EXP13, ATEXP13, ATHEXP ALPHA 1.22, ATEXPA13 | ATEXPA13 (ARABIDOP	-1.824
JCVI_8307	2.395	moderately similar to (380)AT2G19800| Symbols: MIOX2 | MIOX2 (MYO-INOSITOL OXYGENASE 2) | chr2:8538188-8540436 RE	
EE454383	2.394	no similarity	
RC_AM386400	2.394	no similarity	-2.722
JCVI_25329	2.394	highly similar to (540)AT5G05340| Symbols: | peroxidase, putative | chr5:1579143-1580820 REVERSEhighly similar to (515)PERP7_	-2.831
JCVI_14668	2.393	moderately similar to (332)AT4G26970| Symbols: | aconitate hydratase, cytoplasmic, putative / citrate hydro-lyase/aconitase, putative | c	-2.849
JCVI_42611	2.390	highly similar to (506)AT1G53040| Symbols: | hydrolase, acting on carbon-nitrogen (but not peptide) bonds, in linear amides | chr1:1976	
JCVI_10699	2.389	moderately similar to (323)AT2G06050| Symbols: OPR3 | OPR3 (OPDA-REDUCTASE 3) | chr2:2359237-2361968 REVERSE no orig	
JCVI_38586	2.388	highly similar to (918)AT4G19120| Symbols: ERD3 | ERD3 (EARLY-RESPONSIVE TO DEHYDRATION 3) | chr4:10460676-104630	-1.825
RC_AM394725	2.387	no similarity	
EH423344	2.385	no similarity	-2.811
JCVI_3440	2.384	moderately similar to (246)AT1G52410| Symbols: TSA1 | TSA1 (TSK-ASSOCIATING PROTEIN 1) | chr1:19524431-19529030 FORV	
JCVI_39172	2.382	no original description	-2.070
JCVI_14671	2.379	moderately similar to (453)AT2G34930| Symbols: | disease resistance family protein | chr2:14744248-14746965 REVERSEvery weakly	
JCVI_23233	2.378	moderately similar to (376)AT2G36890| Symbols: RAX2, MYB38, ATMYB38 | ATMYB38/MYB38/RAX2 (myb domain protein 38);	
JCVI_11484	2.378	no original description	
JCVI_21028	2.376	moderately similar to (257)AT4G08770| Symbols: | peroxidase, putative | chr4:5598256-5600259 REVERSEweakly similar to (132)PE	
JCVI_13942	2.373	highly similar to (769)AT2G38940| Symbols: PHT1;4, ATPT2 | ATPT2 (PHOSPHATE TRANSPORTER 2); carbohydrate transmembra	-2.689
JCVI_13451	2.371	highly similar to (531)AT5G06570| Symbols: | hydrolase | chr5:2008076-2011014 REVERSEweakly similar to (131)GID1_ORYSA nc	-2.449
JCVI_9520	2.371	weakly similar to (136)AT1G80920| Symbols: J8 | J8; heat shock protein binding / unfolded protein binding | chr1:30408755-30409441 I	
JCVI_30500	2.369	moderately similar to (394)AT1G79720| Symbols: | aspartyl protease family protein | chr1:30002152-30003844 REVERSE no original c	
JCVI_34405	2.368	weakly similar to (109)AT2G39700| Symbols: ATEXP4, ATHEXP ALPHA 1.6, ATEXPA4 | ATEXPA4 (ARABIDOPSIS THALIANA	-2.314
EE420303	2.368	weakly similar to (190)AT5G25190| Symbols: | ethylene-responsive element-binding protein, putative | chr5:8707010-8707658 REVERS	
JCVI_42277	2.367	no original description	
CV546026	2.366	no similarity	-2.295
EV089490	2.366	no similarity	
EX136536	2.364	no similarity	
JCVI_96	2.364	highly similar to (531)AT3G47340| Symbols: DIN6, AT-ASN1, ASN1 | ASN1 (DARK INDUCIBLE 6) | chr3:17449430-17452028 RE	
JCVI_34222	2.363	moderately similar to (245)AT2G27420| Symbols: | cysteine proteinase, putative | chr2:11733389-11734597 REVERSEweakly similar t	-3.020
JCVI_16114	2.362	very weakly similar to (99.4)AT5G11930| Symbols: | glutaredoxin family protein | chr5:3845166-3845612 REVERSE no original descrip	-1.629
JCVI_3001	2.359	weakly similar to (168)AT5G66560| Symbols: | phototropic-responsive NPH3 family protein | chr5:26581594-26583888 FORWARD nc	
RC_CN726502	2.359	no similarity	
CD814689	2.358	moderately similar to (284)AT1G71880| Symbols: ATSUC1, SUC1 | SUC1 (SUCROSE-PROTON SYMPORTER 1); carbohydrate tran	-2.535
JCVI_15204	2.354	moderately similar to (413)AT5G64250| Symbols: | 2-nitropropane dioxygenase family / NPD family | chr5:25714849-25716171 REVE	-4.021
JCVI_746	2.344	moderately similar to (475)AT4G08770| Symbols: | peroxidase, putative | chr4:5598256-5600259 REVERSEmoderately similar to (237	
JCVI_4333	2.341	moderately similar to (330)AT4G27585| Symbols: | band 7 family protein | chr4:13766990-13769838 REVERSE no original descriptior	
EV100892	2.338	no similarity	-2.105
JCVI_24866	2.338	moderately similar to (336)AT1G61260| Symbols: | similar to unknown protein [Arabidopsis thaliana] (TAIR:AT1G11220.1); similar to	
JCVI_20579	2.334	moderately similar to (373)AT3G13790| Symbols: ATCWINV1, ATBFRUCT1 | ATBFRUCT1/ATCWINV1 (ARABIDOPSIS THALIA	-2.148
JCVI_25171	2.332	moderately similar to (236)AT5G61130| Symbols: | glycosyl hydrolase family protein 17 | chr5:24604664-24606595 REVERSEvery we	
DY005339	2.331	weakly similar to (130)AT2G34925| Symbols: CLE42 | CLE42 (CLAVATA3/ESR-RELATED 42) | chr2:14741349-14741615 FORWA	-3.464
AM395031	2.328	weakly similar to (137)AT1G11330| Symbols: | S-locus lectin protein kinase family protein | chr1:3810372-3813416 FORWARD [2034	-1.861
JCVI_16276	2.327	moderately similar to (397)AT3G54750| Symbols: | similar to unnamed protein product [Vitis vinifera] (GB:CAO48944.1); contains dor	
JCVI_24811	2.327	no original description	-2.646
EV110392	2.326	no similarity	-3.058
EV222392	2.326	no similarity	
JCVI_23509	2.326	weakly similar to (130)AT3G20340| Symbols: | Expression of the gene is downregulated in the presence of paraquat, an inducer of photo	
JCVI_13343	2.325	highly similar to (509)AT5G62680| Symbols: | proton-dependent oligopeptide transport (POT) family protein | chr5:25182656-2518504	
EV098206	2.324	weakly similar to (144)AT2G18790| Symbols: HY3, OOP1, PHYB | PHYB (PHYTOCHROME B); G-protein coupled photoreceptor/ sig	
ES265148	2.322	moderately similar to (300)AT1G09770| Symbols: ATMYBCDC5, ATCDC5, CDC5 | ATCDC5 (ARABIDOPSIS THALIANA HOMOI	-1.638
JCVI_37926	2.322	highly similar to (775)AT2G42320| Symbols: | nucleolar protein gar2-related | chr2:17635180-17637735 FORWARD no original descri	
JCVI_23976	2.322	moderately similar to (221)AT5G46800| Symbols: BOU | BOU (A BOUT DE SOUFFLE); binding | chr5:19006006-19007037 REVERS	
JCVI_35758	2.322	moderately similar to (286)AT1G80000| Symbols: | similar to glycine-rich protein [Arabidopsis thaliana] (TAIR:AT1G15280.2); similar	-2.359
EV156162	2.318	moderately similar to (271)AT2G30140| Symbols: | UDP-glucoronosyl/UDP-glucosyl transferase family protein | chr2:12879277-12880	-1.807
ES913153	2.318	moderately similar to (360)AT3G09560| Symbols: | lipin family protein | chr3:2934958-2938678 REVERSE [21430]	
JCVI_7526	2.315	moderately similar to (442)AT1G52400| Symbols: BGL1 | BGL1 (BETA-GLUCOSIDASE HOMOLOG 1); hydrolase, hydrolyzing O-gl	
JCVI_9156	2.315	moderately similar to (372)AT1G51760| Symbols: JR3, IAR3 | IAR3 (IAA-ALANINE RESISTANT 3); metallopeptidase | chr1:192032	
JCVI_21264	2.313	highly similar to (614)AT1G01610| Symbols: ATGPAT4, GPAT4 | ATGPAT4/GPAT4 (GLYCEROL-3-PHOSPHATE ACYLTRANSF	

JCVI_23994	2.312	weakly similar to (169)AT4G23550| Symbols: WRKY29 | WRKY29 (WRKY DNA-binding protein 29); transcription factor | chr4:1229	
JCVI_8045	2.311	highly similar to (515)AT1G28050| Symbols: | zinc finger (B-box type) family protein | chr1:9775755-9777644 REVERSE no original d	
EX136616	2.311	moderately similar to (303)AT5G66700| Symbols: HB-8, ATHB53, HB53 | HB53 (homeobox-8); DNA binding / transcription factor | ch	
JCVI_41998	2.310	weakly similar to (195)AT3G27400| Symbols: | pectate lyase family protein | chr3:10141560-10144260 FORWARDweakly similar to (1	-2.988
JCVI_27523	2.304	moderately similar to (262)AT1G79700| Symbols: | ovule development protein, putative | chr1:29995333-29998551 REVERSEweakly s	
JCVI_8697	2.302	moderately similar to (292)AT2G30140| Symbols: | UDP-glucoronosyl/UDP-glucosyl transferase family protein | chr2:12879277-12880	-1.882
JCVI_19403	2.301	very weakly similar to (93.2)AT5G24660| Symbols: | similar to unknown protein [Arabidopsis thaliana] (TAIR:AT5G24655.1); similar to	
JCVI_21122	2.301	moderately similar to (380)AT1G78700| Symbols: | brassinosteroid signalling positive regulator-related | chr1:29604747-29606432 FOR	
JCVI_17489	2.300	moderately similar to (320)AT1G17745| Symbols: PGDH | PGDH (3-PHOSPHOGLYCERATE DEHYDROGENASE); phosphoglycera	-2.459
JCVI_31440	2.298	no original description	-2.618
EX138385	2.297	no similarity	
JCVI_25800	2.297	weakly similar to (169)AT2G01150| Symbols: RHA2B | RHA2B (RING-H2 FINGER PROTEIN 2B); protein binding / zinc ion binding	-2.455
JCVI_17434	2.296	moderately similar to (379)AT1G14210| Symbols: | ribonuclease T2 family protein | chr1:4856897-4857936 REVERSEweakly similar to	
JCVI_19832	2.294	weakly similar to (164)AT1G78240| Symbols: QUA2, TSD2 | TSD2 (TUMOROUS SHOOT DEVELOPMENT 2); methyltransferase | c	-1.271
JCVI_23885	2.291	moderately similar to (337)AT3G60390| Symbols: HAT3 | HAT3 (homeobox-leucine zipper protein 3); transcription factor | chr3:22331	
JCVI_41539	2.289	weakly similar to (140)AT2G42570| Symbols: | similar to unknown protein [Arabidopsis thaliana] (TAIR:AT2G31110.2); similar to unk	-1.736
JCVI_42035	2.289	moderately similar to (271)AT1G73260| Symbols: | trypsin and protease inhibitor family protein / Kunitz family protein | chr1:27551071	-2.409
JCVI_28284	2.288	moderately similar to (431)AT1G11330| Symbols: | S-locus lectin protein kinase family protein | chr1:3810372-3813416 FORWARDwe	-2.091
JCVI_445	2.287	moderately similar to (435)AT2G38750| Symbols: ANNAT4 | ANNAT4 (ANNEXIN ARABIDOPSIS 4); calcium ion binding / calcium-	
EX099690	2.286	weakly similar to (152)AT3G29200| Symbols: ATCM1, CM1 | CM1 (chorismate mutase 1); chorismate mutase | chr3:11165819-111674	
JCVI_42051	2.284	moderately similar to (393)AT5G61190| Symbols: | zinc finger protein-related | chr5:24632706-24637112 FORWARD no original descr	
JCVI_28604	2.280	highly similar to (576)AT3G63380| Symbols: | calcium-transporting ATPase, plasma membrane-type, putative / Ca(2+)-ATPase, putative	-2.738
JCVI_6696	2.279	moderately similar to (469)AT3G20860| Symbols: ATNEK5 | ATNEK5; kinase | chr3:7306153-7308440 FORWARDvery weakly simila	
JCVI_38883	2.277	moderately similar to (335)AT5G23350| Symbols: | GRAM domain-containing protein / ABA-responsive protein-related | chr5:7858548	
JCVI_867	2.276	weakly similar to (114)AT1G76180| Symbols: ERD14 | ERD14 (EARLY RESPONSE TO DEHYDRATION 14) | chr1:28591907-2859	-1.955
JCVI_15337	2.275	moderately similar to (287)AT5G63490| Symbols: | CBS domain-containing protein / octicosapeptide/Phox/Bemp1 (PB1) domain-conta	-1.846
EV143177	2.272	weakly similar to (135)AT5G64410| Symbols: ATOPT4 | ATOPT4 (oligopeptide transporter 4); oligopeptide transporter | chr5:2576814	-2.320
CN732026	2.271	no similarity	-2.784
CD826853	2.271	no similarity	
EX050641	2.270	no similarity	
EX046111	2.270	moderately similar to (406)AT1G69780| Symbols: ATHB13 | ATHB13; DNA binding / transcription factor | chr1:26262829-26264128 F	
EV110607	2.268	no similarity	
JCVI_40683	2.268	no original description	
EV019254	2.266	no similarity	-2.324
JCVI_5467	2.265	moderately similar to (299)AT4G36990| Symbols: HSFB1, AT-HSFB1, ATHSF4, HSF4 | HSF4 (HEAT SHOCK FACTOR 4); DNA bi	-2.044
JCVI_8217	2.262	highly similar to (503)AT4G27585| Symbols: | band 7 family protein | chr4:13766990-13769838 REVERSE no original description	-1.682
EV070670	2.261	weakly similar to (147)AT2G33260| Symbols: | tryptophan/tyrosine permease family protein | chr2:14107917-14109329 REVERSE [21	-2.096
EX043287	2.260	weakly similar to (131)AT5G13180| Symbols: ANAC083 | ANAC083 (Arabidopsis NAC domain containing protein 83); transcription fa	-2.423
AM060217	2.259	no similarity	
JCVI_2517	2.258	moderately similar to (301)AT1G07090| Symbols: | similar to unknown protein [Arabidopsis thaliana] (TAIR:AT5G58500.1); similar to	
AY460110	2.257	weakly similar to (135)AT4G39260| Symbols: GR-RBP8, ATGRP8, CCR1 | ATGRP8/GR-RBP8 (COLD, CIRCADIAN RHYTHM, AN	
EX120389	2.257	weakly similar to (167)AT3G05360| Symbols: | disease resistance family protein / LRR family protein | chr3:1530906-1533266 REVER	-2.532
JCVI_37321	2.253	moderately similar to (428)AT5G67310| Symbols: CYP81G1 | CYP81G1 (cytochrome P450, family 81, subfamily G, polypeptide 1); ox	
ES919220	2.252	moderately similar to (275)AT1G79310| Symbols: ATMC7 | ATMC7 (METACASPASE 7); caspase | chr1:29838879-29840305 FORW	
JCVI_3196	2.251	moderately similar to (337)AT1G51780| Symbols: ILL5 | ILL5 (IAA-leucine resistant (ILR)-like gene 5); metallopeptidase | chr1:192082	
RC_ES953373	2.250	no similarity	
JCVI_17155	2.249	moderately similar to (446)AT3G59220| Symbols: PRN1, ATPIRIN1, PRN | PRN (PIRIN); calmodulin binding | chr3:21905182-219064	
EV103768	2.247	weakly similar to (102)AT3G30180| Symbols: CYP85A2, BR6OX2 | BR6OX2/CYP85A2 (BRASSINOSTEROID-6-OXIDASE 2); mo	
JCVI_13966	2.246	moderately similar to (285)AT5G06870| Symbols: PGIP2 | PGIP2 (POLYGALACTURONASE INHIBITING PROTEIN 2); protein bin	
JCVI_16693	2.245	moderately similar to (207)AT1G71880| Symbols: ATSUC1, SUC1 | SUC1 (SUCROSE-PROTON SYMPORTER 1); carbohydrate tran	
EE433315	2.243	very weakly similar to (94.0)AT3G02170| Symbols: LNG2 | LNG2 (LONGIFOLIA2) | chr3:396215-399400 REVERSE [15720] 1 298 3	
JCVI_14549	2.243	weakly similar to (160)AT1G52030| Symbols: MBP1.2, F-ATMBP, MBP2 | MBP2 (MYROSINASE-BINDING PROTEIN 2) | chr1:193	
JCVI_23496	2.243	no original description	
JCVI_39713	2.240	moderately similar to (286)AT4G39070| Symbols: | zinc finger (B-box type) family protein | chr4:18205055-18206415 REVERSE no o	
JCVI_18478	2.237	moderately similar to (400)AT1G53990| Symbols: GLIP3 | GLIP3 (GDSL-motif lipase 3); carboxylesterase/ lipase | chr1:20154684-2015	-3.702
JCVI_9521	2.232	moderately similar to (399)AT2G38010| Symbols: | ceramidase family protein | chr2:15913940-15916945 FORWARD no original descr	-1.901
EX032482	2.232	moderately similar to (239)AT5G03610| Symbols: | GDSL-motif lipase/hydrolase family protein | chr5:915649-918325 FORWARD [21	
JCVI_7399	2.232	moderately similar to (219)AT5G15350| Symbols: | plastocyanin-like domain-containing protein | chr5:4985187-4986157 REVERSE no	
ES943702	2.231	weakly similar to (200)AT4G02800| Symbols: | similar to unknown protein [Arabidopsis thaliana] (TAIR:AT1G30050.1); similar to Os0	
JCVI_25338	2.231	moderately similar to (277)AT5G15530| Symbols: BCCP2, CAC1-B | BCCP2 (biotin carboxyl carrier protein 2); biotin binding | chr5:50	
EE521952	2.227	weakly similar to (194)AT1G67850| Symbols: | similar to unknown protein [Arabidopsis thaliana] (TAIR:AT1G13000.2); similar to unk	-2.746
EX069739	2.223	no similarity	
EE523400	2.220	no similarity	
EX135089	2.220	moderately similar to (414)AT2G38240| Symbols: | oxidoreductase, 2OG-Fe(II) oxygenase family protein | chr2:16019801-16021744 R	
EE438830	2.220	no similarity	-2.365
JCVI_8843	2.219	moderately similar to (391)AT2G17120| Symbols: LYM2 | LYM2 (LYSM DOMAIN GPI-ANCHORED PROTEIN 2 PRECURSOR) | c	
H07446	2.216	no similarity	-3.274
JCVI_18750	2.211	moderately similar to (479)AT3G43960| Symbols: | cysteine proteinase, putative | chr3:15785109-15786615 REVERSEmoderately simi	
EE555164	2.210	no similarity	-1.791
JCVI_8111	2.208	moderately similar to (446)AT3G47340| Symbols: DIN6, AT-ASN1, ASN1 | ASN1 (DARK INDUCIBLE 6) | chr3:17449430-1745202	
JCVI_10286	2.205	moderately similar to (311)AT1G07090| Symbols: | similar to unknown protein [Arabidopsis thaliana] (TAIR:AT5G58500.1); similar to	
JCVI_26017	2.204	moderately similar to (337)AT4G19120| Symbols: ERD3 | ERD3 (EARLY-RESPONSIVE TO DEHYDRATION 3) | chr4:10460676-10	-1.726
CD812150	2.202	no similarity	
JCVI_24728	2.199	weakly similar to (107)AT3G52910| Symbols: AtGRF4 | AtGRF4 (GROWTH-REGULATING FACTOR 4) | chr3:19627155-19629246	
CN728982	2.198	moderately similar to (223)AT3G05360| Symbols: | disease resistance family protein / LRR family protein | chr3:1530906-1533266 REV	-2.697
JCVI_25365	2.197	moderately similar to (431)AT4G13810| Symbols: | disease resistance family protein / LRR family protein | chr4:8005058-8007283 REV	-2.123
EX049784	2.194	weakly similar to (196)AT1G30320| Symbols: | remorin family protein | chr1:10680330-10682834 FORWARD [21812]	
JCVI_31058	2.191	weakly similar to (181)AT2G24590| Symbols: | splicing factor, putative | chr2:10456916-10457939 FORWARD no original description	-2.174
EV184029	2.190	moderately similar to (245)AT5G05340| Symbols: | peroxidase, putative | chr5:1579143-1580820 REVERSEmoderately similar to (243	-2.597
JCVI_6556	2.190	weakly similar to (115)AT2G44080| Symbols: ARL | ARL (ARGOS-LIKE) | chr2:18244802-18245209 FORWARD no original descript	

AM058964	2.188	no similarity	-3.082
JCVI_1637	2.187	moderately similar to (289)AT4G23680| Symbols: | major latex protein-related / MLP-related | chr4:12336426-12337427 REVERSE no	
JCVI_19483	2.186	moderately similar to (379)AT2G30140| Symbols: | UDP-glucoronosyl/UDP-glucosyl transferase family protein | chr2:12879277-12880	-1.650
JCVI_38304	2.185	moderately similar to (201)AT5G43650| Symbols: | basic helix-loop-helix (bHLH) family protein | chr5:17550537-17551985 REVERSE	
EV221401	2.185	moderately similar to (238)AT5G55400| Symbols: | fimbrin-like protein, putative | chr5:22472761-22476176 REVERSE [21492]	
JCVI_35175	2.184	weakly similar to (107)AT1G67920| Symbols: | similar to unknown protein [Arabidopsis thaliana] (TAIR:AT1G24600.1) | chr1:2547739	
JCVI_10228	2.181	moderately similar to (375)AT2G38010| Symbols: | ceramidase family protein | chr2:15913940-15916945 FORWARD no original descr	-1.948
AT000634	2.180	no similarity	
JCVI_430	2.179	moderately similar to (263)AT1G09070| Symbols: SRC2, (AT)SRC2 | (AT)SRC2/SRC2 (SOYBEAN GENE REGULATED BY COLD-	-1.401
JCVI_8465	2.178	moderately similar to (397)AT1G11330| Symbols: | S-locus lectin protein kinase family protein | chr1:3810372-3813416 FORWARDwe	
JCVI_2824	2.177	moderately similar to (404)AT5G46600| Symbols: | similar to unknown protein [Arabidopsis thaliana] (TAIR:AT4G17970.1); similar to	
JCVI_8695	2.177	moderately similar to (356)AT1G60390| Symbols: | BURP domain-containing protein / polygalacturonase, putative | chr1:22251276-222	
JCVI_38661	2.174	moderately similar to (246)AT4G20780| Symbols: | calcium-binding protein, putative | chr4:11133320-11133895 REVERSEweakly sim	
EX091151	2.173	moderately similar to (342)AT5G22740| Symbols: CSLA02, ATCSLA2, ATCSLA02 | ATCSLA02 (Cellulose synthase-like A2); transfer	
EV165473	2.173	weakly similar to (174)AT5G60920| Symbols: COB | COB (COBRA) | chr5:24528692-24531158 REVERSEweakly similar to (134)CO	
JCVI_11255	2.172	moderately similar to (257)AT1G52410| Symbols: TSA1 | TSA1 (TSK-ASSOCIATING PROTEIN 1) | chr1:19524431-19529030 FORV	
JCVI_6342	2.172	weakly similar to (194)AT1G19380| Symbols: | similar to unknown protein [Arabidopsis thaliana] (TAIR:AT5G65650.1); similar to unk	-2.742
EX141948	2.167	very weakly similar to (92.8)AT3G56590| Symbols: | hydroxyproline-rich glycoprotein family protein | chr3:20976084-20978763 FORW	
EV197954	2.166	weakly similar to (179)AT2G37700| Symbols: | similar to CER1 protein, putative [Arabidopsis thaliana] (TAIR:AT1G02190.1); similar	
JCVI_2410	2.166	moderately similar to (293)AT4G16740| Symbols: ATTPS03 | ATTPS03 (Arabidopsis thaliana terpene synthase 03) | chr4:9407878-940	1.164
EV172076	2.166	no similarity	-1.930
JCVI_36814	2.164	moderately similar to (345)AT1G09480| Symbols: | cinnamyl-alcohol dehydrogenase family / CAD family | chr1:3057977-3060663 FOI	
ES899837	2.163	no similarity	
CO749888	2.162	no similarity	
RC_EE565735	2.161	no similarity	
AM060493	2.158	moderately similar to (375)AT5G42830| Symbols: | transferase family protein | chr5:17193612-17195134 FORWARD [17712]	
EE421897	2.158	no similarity	-2.266
JCVI_13395	2.157	moderately similar to (330)AT5G52390| Symbols: | photoassimilate-responsive protein, putative | chr5:21281507-21282399 REVERSE	-3.083
EH413915	2.153	moderately similar to (302)AT3G21760| Symbols: | UDP-glucoronosyl/UDP-glucosyl transferase family protein | chr3:7667106-766856	
JCVI_18164	2.152	moderately similar to (205)AT1G72360| Symbols: | ethylene-responsive element-binding protein, putative | chr1:27245566-27246439 FC	
ES912473	2.151	moderately similar to (261)AT4G36620| Symbols: | zinc finger (GATA type) family protein | chr4:17268912-17269668 REVERSE [214	
ES930241	2.150	no similarity	
JCVI_29606	2.150	moderately similar to (294)AT2G34930| Symbols: | disease resistance family protein | chr2:14744248-14746965 REVERSEweakly simi	
EV161212	2.149	moderately similar to (212)AT5G06860| Symbols: PGIP1 | PGIP1 (POLYGALACTURONASE INHIBITING PROTEIN 1); protein bin	
JCVI_38868	2.149	moderately similar to (343)AT2G38760| Symbols: ANNAT3 | ANNAT3 (ANNEXIN ARABIDOPSIS 3); calcium ion binding / calcium-	
EE429349	2.145	moderately similar to (233)AT1G10960| Symbols: ATFD1 | ATFD1 (FERREDOXIN 1); 2 iron, 2 sulfur cluster binding / electron carrier	
JCVI_36528	2.142	weakly similar to (120)AT4G35160| Symbols: | O-methyltransferase family 2 protein | chr4:16730994-16732813 REVERSE no original	
RC_EV080004	2.140	no similarity	-2.033
EE409666	2.140	very weakly similar to (89.4)AT5G67480| Symbols: BT4 | BT4 (BTB AND TAZ DOMAIN PROTEIN 4); protein binding | chr5:2694828	
JCVI_26789	2.138	highly similar to (520)AT5G62230| Symbols: ERL1 | ERL1 (ERECTA-LIKE 1); kinase | chr5:25013659-25019356 FORWARDmoderat	
JCVI_13368	2.138	moderately similar to (291)AT1G73260| Symbols: | trypsin and protease inhibitor family protein / Kunitz family protein | chr1:27551071	-2.241
JCVI_8511	2.138	highly similar to (569)AT5G67360| Symbols: ARA12 | ARA12; subtilase | chr5:26889418-26891691 REVERSE no original description	-2.067
AM390653	2.137	weakly similar to (104)AT4G33720| Symbols: | pathogenesis-related protein, putative | chr4:16182816-16183307 FORWARDvery weak	-2.453
EV167296	2.137	no similarity	-1.249
EV001540	2.136	weakly similar to (141)AT2G36910| Symbols: PGP1, ATMDR1 | ATPGP1 (ARABIDOPSIS THALIANA P GLYCOPROTEIN1); calm	
EE568594	2.136	weakly similar to (129)AT4G37180| Symbols: | myb family transcription factor | chr4:17504642-17506101 FORWARD [20153] 21 502	
RC_JCVI_41947	2.135	no original description	
JCVI_114	2.133	moderately similar to (311)AT1G79110| Symbols: | protein binding / zinc ion binding | chr1:29764238-29765479 FORWARD no origin	
EE479688	2.129	moderately similar to (206)AT1G16510| Symbols: | auxin-responsive family protein | chr1:5644777-5645220 REVERSE [20132] 1 567	-3.249
JCVI_18368	2.122	moderately similar to (227)AT3G06420| Symbols: ATG8H | ATG8H (AUTOPHAGY 8H); microtubule binding | chr3:1955225-195628(	-1.619
JCVI_41709	2.120	weakly similar to (180)AT2G01150| Symbols: RHA2B | RHA2B (RING-H2 FINGER PROTEIN 2B); protein binding / zinc ion binding	-2.549
JCVI_22726	2.120	moderately similar to (357)AT3G55640| Symbols: | mitochondrial substrate carrier family protein | chr3:20651027-20653390 FORWAR	
JCVI_8614	2.119	moderately similar to (366)AT4G39980| Symbols: DHS1 | DHS1 (3-DEOXY-D-ARABINO-HEPTULOSONATE 7-PHOSPHATE SYN	
EH430387	2.118	weakly similar to (153)AT1G29120| Symbols: | similar to unknown protein [Arabidopsis thaliana] (TAIR:AT4G25770.1); similar to unn	
EV059456	2.118	no similarity	
JCVI_33063	2.117	moderately similar to (289)AT1G69690| Symbols: | TCP family transcription factor, putative | chr1:26220112-26221089 FORWARD nc	
ES943828	2.116	weakly similar to (129)AT5G21280| Symbols: | hydroxyproline-rich glycoprotein family protein | chr5:7264097-7265517 REVERSE [2	
EV205296	2.115	no similarity	-2.205
JCVI_39827	2.115	no original description	
JCVI_5687	2.115	highly similar to (842)AT5G60920| Symbols: COB | COB (COBRA) | chr5:24528692-24531158 REVERSEhighly similar to (658)COB	
EV101700	2.114	moderately similar to (315)AT1G51805| Symbols: | leucine-rich repeat protein kinase, putative | chr1:19224856-19229259 REVERSE [2	-1.677
ES945125	2.111	weakly similar to (185)AT2G36400| Symbols: AtGRF3 | AtGRF3 (GROWTH-REGULATING FACTOR 3) | chr2:15277379-15279696	
JCVI_17087	2.110	moderately similar to (255)AT2G21340| Symbols: | enhanced disease susceptibility protein, putative / salicylic acid induction deficient p	
JCVI_39483	2.104	moderately similar to (296)AT3G18930| Symbols: | zinc finger (C3HC4-type RING finger) family protein | chr3:6524116-6525351 REV	-2.459
ES903435	2.103	moderately similar to (354)AT1G79720| Symbols: | aspartyl protease family protein | chr1:30002152-30003844 REVERSE [21432] 16	-2.523
JCVI_20525	2.103	nearly identical (1173)AT5G02500| Symbols: HSP70-1, AT-HSC70-1, HSC70, HSC70-1 | HSC70-1 (heat shock cognate 70 kDa protein	-1.395
JCVI_26326	2.100	moderately similar to (357)AT1G11260| Symbols: STP1 | STP1 (SUGAR TRANSPORTER 1); carbohydrate transmembrane transporter	-1.546
JCVI_10199	2.100	moderately similar to (209)AT1G05160| Symbols: ATKAO1, KAO1, CYP88A3 | CYP88A3 (ENT-KAURENOIC ACID HYDROXYLA	
RC_ES978926	2.099	no similarity	
JCVI_6514	2.099	moderately similar to (318)AT3G57010| Symbols: | strictosidine synthase family protein | chr3:21106833-21108295 REVERSEvery wea	
BQ704632	2.098	no similarity	
JCVI_2485	2.097	moderately similar to (499)AT3G17390| Symbols: SAMS3, MAT4, MTO3 | MTO3 (S-adenosylmethionine synthase 3); methionine ader	
AM391930	2.095	moderately similar to (265)AT3G14840| Symbols: | leucine-rich repeat family protein / protein kinase family protein | chr3:4988278-499	-2.071
ES953373	2.095	no similarity	
ES948968	2.090	weakly similar to (115)AT2G35160| Symbols: SUVH5 | SUVH5 (SU(VAR)3-9 HOMOLOG 5) | chr2:14830641-14833025 FORWARD	
JCVI_13144	2.087	moderately similar to (301)AT1G08450| Symbols: CRT3 | CRT3 (CALRETICULIN 3); calcium ion binding | chr1:2668005-2671797 RI	-2.160
EE462294	2.087	weakly similar to (169)AT1G75580| Symbols: | auxin-responsive protein, putative | chr1:28381191-28381517 FORWARD [15722]	
JCVI_11495	2.085	moderately similar to (404)AT4G24740| Symbols: AME1, AFC2 | AFC2 (ARABIDOPSIS FUS3-COMPLEMENTING GENE 1); kinase	-2.350
JCVI_2048	2.085	weakly similar to (199)AT1G79110| Symbols: | protein binding / zinc ion binding | chr1:29764238-29765479 FORWARD no original de	
EE516379	2.083	weakly similar to (157)AT5G11660| Symbols: | similar to unknown protein [Arabidopsis thaliana] (TAIR:AT5G05030.1); similar to unn	-2.216

JCVI_42188	2.080	no original description	
EV126769	2.079	no similarity	-1.401
AM058224	2.078	no similarity	-1.674
JCVI_26654	2.077	weakly similar to (128)AT1G27150| Symbols: | binding | chr1:9429164-9432055 FORWARD no original description	
DY024262	2.077	no similarity	
EE434731	2.075	moderately similar to (209)AT3G54950| Symbols: PLP7, PLA IIIA | PLA IIIA/PLP7 (Patatin-like protein 7) | chr3:20370053-20371751	
RC_ES968542	2.074	no similarity	
JCVI_1296	2.073	highly similar to (518)AT2G30490| Symbols: ATC4H, C4H, CYP73A5 | ATC4H/C4H/CYP73A5 (CINNAMATE 4-HYDROXYLASE,	
CN726502	2.072	no similarity	
JCVI_40451	2.071	weakly similar to (151)AT1G19180| Symbols: JAZ1, TIFY10A | JAZ1/TIFY10A (JASMONATE-ZIM-DOMAIN PROTEIN 1) | chr1:6	
JCVI_14532	2.071	moderately similar to (350)AT1G74440| Symbols: | similar to unknown protein [Arabidopsis thaliana] (TAIR:AT1G18720.1); similar to	-2.601
JCVI_34867	2.070	moderately similar to (363)AT5G48930| Symbols: HCT | transferase family protein | chr5:19853880-19855318 REVERSEweakly simila	
EV127727	2.070	weakly similar to (167)AT1G02640| Symbols: ATBXL2, BXL2 | BXL2 (BETA-XYLOSIDASE 2); hydrolase, hydrolyzing O-glycosyl c	
CX278039	2.068	weakly similar to (129)AT1G29980| Symbols: | similar to unknown protein [Arabidopsis thaliana] (TAIR:AT2G34510.1); similar to unk	
JCVI_17680	2.068	moderately similar to (251)AT2G35190| Symbols: ATNPSN11, NSPN11, NPSN11 | NPSN11 (NOVEL PLANT SNARE 11); protein tra	
ES903665	2.067	moderately similar to (226)AT3G20390| Symbols: | endoribonuclease L-PSP family protein | chr3:7110233-7111701 REVERSE [2142	-2.090
EV088958	2.066	no similarity	
EE529053	2.065	moderately similar to (287)AT3G30180| Symbols: CYP85A2, BR6OX2 | BR6OX2/CYP85A2 (BRASSINOSTEROID-6-OXIDASE 2);	
JCVI_26147	2.063	no original description	
EV059746	2.063	very weakly similar to (100)AT1G78390| Symbols: ATNCED9, NCED9 | NCED9 (NINE-CIS-EPOXYCAROTENOID DIOXYGENAS	
JCVI_32157	2.063	no original description	-1.792
EX015519	2.058	no similarity	
JCVI_17298	2.057	moderately similar to (455)AT4G12730| Symbols: FLA2 | FLA2 | chr4:7491595-7492806 REVERSE no original description	
JCVI_40830	2.055	very weakly similar to (86.7)AT5G53400| Symbols: | nuclear movement family protein | chr5:21678814-21680609 FORWARD no origi	
EX141390	2.055	very weakly similar to (82.0)AT5G52450| Symbols: | MATE efflux protein-related | chr5:21306268-21308975 REVERSE [21834]	
JCVI_31959	2.055	no original description	
CX194462	2.055	no similarity	-3.610
JCVI_27839	2.054	no original description	-1.471
CD813547	2.052	moderately similar to (254)AT3G26170| Symbols: CYP71B19 | CYP71B19 (cytochrome P450, family 71, subfamily B, polypeptide 19);	-2.203
AI352858	2.050	weakly similar to (112)AT2G14610| Symbols: PR-1, PR1 | PR1 (PATHOGENESIS-RELATED GENE 1) | chr2:6249026-6249511 REV	-3.040
EE439003	2.050	moderately similar to (279)AT2G15760| Symbols: | calmodulin-binding protein | chr2:6872889-6873836 REVERSE [20173]	
EV225508	2.049	no similarity	
EE419993	2.048	moderately similar to (216)AT4G37870| Symbols: PCK1, PEPCK | PCK1/PEPCK (PHOSPHOENOLPYRUVATE CARBOXYKINASE	
JCVI_30575	2.047	highly similar to (503)AT2G44590| Symbols: ADL1D | ADL1D; GTP binding / GTPase | chr2:18410932-18414037 REVERSE no origi	
JCVI_20714	2.044	very weakly similar to (99.0)AT1G21660| Symbols: | heat shock protein binding | chr1:7605913-7608824 FORWARD no original descri	
EV219618	2.043	moderately similar to (248)AT4G00970| Symbols: | protein kinase family protein | chr4:418437-421694 FORWARD [21492] 50 737 73	-2.587
AM391405	2.043	no similarity	
EX042619	2.042	no similarity	
JCVI_27760	2.039	highly similar to (553)AT4G15560| Symbols: DEF, CLA, DXS, DXPS2, CLA1 | CLA1 (CLOROPLASTOS ALTERADOS 1) | chr4:888	
EE557357	2.038	moderately similar to (268)AT1G13880| Symbols: | ELM2 domain-containing protein | chr1:4749600-4750964 FORWARD [20153] 21	
EH423349	2.038	very weakly similar to (93.2)AT1G14040| Symbols: | ATP binding / ATPase, coupled to transmembrane movement of substances | chr1:4	-2.300
ES930197	2.037	moderately similar to (216)AT4G05120| Symbols: ENT3, FUR1 | ENT3/FUR1 (FUDR RESISTANT 1); nucleoside transmembrane tran	-1.660
EX097653	2.036	moderately similar to (360)AT3G62780| Symbols: | C2 domain-containing protein | chr3:23233004-23233900 REVERSE [21824]	
JCVI_17503	2.034	moderately similar to (214)AT5G61440| Symbols: | thioredoxin family protein | chr5:24724884-24725910 FORWARD no original desc	
JCVI_33065	2.030	weakly similar to (131)AT3G10040| Symbols: | transcription factor | chr3:3096585-3097880 REVERSE no original description	-2.475
JCVI_15998	2.030	highly similar to (532)AT1G14590| Symbols: | similar to unknown protein [Arabidopsis thaliana] (TAIR:AT2G02061.1); similar to unna	
EV050608	2.029	weakly similar to (161)AT1G07280| Symbols: | binding | chr1:2238504-2240990 FORWARD [21442]	
JCVI_25884	2.029	moderately similar to (281)AT1G61960| Symbols: | mitochondrial transcription termination factor-related / mTERF-related | chr1:22905	
JCVI_29402	2.028	no original description	
JCVI_11788	2.028	moderately similar to (312)AT1G29280| Symbols: ATWRKY65, WRKY65 | WRKY65 (WRKY DNA-binding protein 65); transcription	-2.033
DY028164	2.026	moderately similar to (255)AT2G16900| Symbols: | similar to unknown protein [Arabidopsis thaliana] (TAIR:AT4G35110.2); similar to	
JCVI_22877	2.026	very weakly similar to (99.8)AT2G22795| Symbols: | similar to unknown protein [Arabidopsis thaliana] (TAIR:AT4G37820.1); similar to	
JCVI_34441	2.026	moderately similar to (340)AT2G42950| Symbols: | similar to unknown protein [Arabidopsis thaliana] (TAIR:AT1G29820.1); similar to	-1.495
JCVI_7713	2.025	moderately similar to (348)AT1G68470| Symbols: | exostosin family protein | chr1:25680208-25681930 REVERSEweakly similar to (1	
JCVI_11315	2.024	moderately similar to (422)AT5G54170| Symbols: | similar to CP5 [Arabidopsis thaliana] (TAIR:AT1G64720.1); similar to putative nod	
EE401805	2.019	moderately similar to (314)AT1G78390| Symbols: ATNCED9, NCED9 | NCED9 (NINE-CIS-EPOXYCAROTENOID DIOXYGENASE	
EV166483	2.018	very weakly similar to (83.6)AT2G24850| Symbols: TAT, TAT3 | TAT3 (TYROSINE AMINOTRANSFERASE 3); transaminase | chr2:	
JCVI_26062	2.017	moderately similar to (285)AT5G01370| Symbols: | unknown protein | chr5:152574-154294 FORWARD no original description	
JCVI_12882	2.016	moderately similar to (484)AT4G03420| Symbols: | similar to unknown protein [Arabidopsis thaliana] (TAIR:AT1G03610.1); similar to	
EE550165	2.014	no similarity	
JCVI_23661	2.013	weakly similar to (140)AT3G62860| Symbols: | esterase/lipase/thioesterase family protein | chr3:23250552-23253118 REVERSE no ori	
JCVI_8815	2.011	moderately similar to (251)AT1G67070| Symbols: DIN9 | DIN9 (DARK INDUCIBLE 9); mannose-6-phosphate isomerase | chr1:25045	-1.576
JCVI_27068	2.009	moderately similar to (250)AT4G20780| Symbols: | calcium-binding protein, putative | chr4:11133320-11133895 REVERSEweakly sim	
EX094798	2.008	very weakly similar to (85.9)AT3G30460| Symbols: | zinc finger (C3HC4-type RING finger) family protein | chr3:12106912-12107355 F	
JCVI_39241	2.006	weakly similar to (191)AT4G27520| Symbols: | plastocyanin-like domain-containing protein | chr4:13750674-13751825 REVERSE no	
JCVI_8252	2.005	weakly similar to (197)AT5G66170| Symbols: | similar to unknown protein [Arabidopsis thaliana] (TAIR:AT2G17850.1); similar to unn	
JCVI_31311	2.003	highly similar to (651)AT1G25320| Symbols: | leucine-rich repeat transmembrane protein kinase, putative | chr1:8877975-8880167 FOR	
JCVI_13503	2.002	moderately similar to (385)AT4G25310| Symbols: | oxidoreductase, 2OG-Fe(II) oxygenase family protein | chr4:12949763-12951148 F	-2.904
EV218573	2.000	no similarity	-2.175
JCVI_9022	2.000	moderately similar to (337)AT1G51860| Symbols: | leucine-rich repeat protein kinase, putative | chr1:19261303-19265148 REVERSEw	-2.992
JCVI_3272	1.999	moderately similar to (489)AT5G06720| Symbols: | peroxidase, putative | chr5:2077568-2078858 REVERSEmoderately similar to (340	
EX061630	1.999	moderately similar to (345)AT5G67360| Symbols: ARA12 | ARA12; subtilase | chr5:26889418-26891691 REVERSE [21813] 14 762 7	-1.709
JCVI_18334	1.998	moderately similar to (417)AT2G30490| Symbols: ATC4H, C4H, CYP73A5 | ATC4H/C4H/CYP73A5 (CINNAMATE 4-HYDROXYL	-2.356
JCVI_12958	1.998	moderately similar to (244)AT3G03440| Symbols: | armadillo/beta-catenin repeat family protein | chr3:815716-818575 FORWARD no	-2.582
JCVI_4554	1.997	highly similar to (663)AT5G48090| Symbols: ELP1 | ELP1 (EDM2-LIKE PROTEIN1); protein binding / zinc ion binding | chr5:195024	
EE426817	1.997	no similarity	-1.596
JCVI_36739	1.994	moderately similar to (329)AT1G22430| Symbols: | alcohol dehydrogenase, putative | chr1:7919224-7921583 FORWARDweakly simila	
JCVI_21039	1.994	no original description	-2.216
JCVI_38709	1.993	moderately similar to (317)AT1G52200| Symbols: | similar to unknown protein [Arabidopsis thaliana] (TAIR:AT3G18470.1); similar to	-2.553
JCVI_6699	1.992	weakly similar to (194)AT1G70700| Symbols: JAZ9, TIFY7 | JAZ9/TIFY7 (JASMONATE-ZIM-DOMAIN PROTEIN 9) | chr1:266586	

DY002583	1.991	weakly similar to (192)AT5G57580| Symbols: | calmodulin-binding protein | chr5:23332220-23334909 REVERSE [18968] 1 566 597	
EV197082	1.988	moderately similar to (267)AT1G29724| Symbols: | protein binding | chr1:10397726-10400438 REVERSEvery weakly similar to (90.1)	
DT317716	1.987	no similarity	-1.833
EE534833	1.986	weakly similar to (153)AT2G45010| Symbols: | similar to unknown protein [Arabidopsis thaliana] (TAIR:AT5G51400.1); similar to unk	-2.080
JCVI_34424	1.985	weakly similar to (116)AT5G58000| Symbols: | CPL4 (C-TERMINAL DOMAIN PHOSPHATASE-LIKE 4) | chr5:23494642-2349672	
ES966655	1.985	no similarity	
JCVI_6621	1.984	moderately similar to (361)AT5G22740| Symbols: CSLA02, ATCSLA2, ATCSLA02 | ATCSLA02 (Cellulose synthase-like A2); transfe	
EE432853	1.984	weakly similar to (141)AT3G15534| Symbols: | similar to unknown protein [Arabidopsis thaliana] (TAIR:AT1G52855.1); similar to unn	-1.468
JCVI_6507	1.984	moderately similar to (259)AT5G08770| Symbols: | similar to unnamed protein product [Vitis vinifera] (GB:CAO71661.1); similar to hy	-1.392
JCVI_23980	1.983	no original description	
JCVI_4291	1.982	weakly similar to (150)AT4G17890| Symbols: AGD8 | AGD8 (ARF-GAP DOMAIN 8); DNA binding | chr4:9937134-9939001 FORWA	
JCVI_840	1.982	moderately similar to (215)AT1G16840| Symbols: | similar to unknown protein [Arabidopsis thaliana] (TAIR:AT1G78890.1); similar to	
JCVI_15215	1.982	moderately similar to (473)AT5G64410| Symbols: ATOPT4 | ATOPT4 (oligopeptide transporter 4); oligopeptide transporter | chr5:2576	-1.940
JCVI_6730	1.982	moderately similar to (247)AT5G16230| Symbols: | acyl-(acyl-carrier-protein) desaturase, putative / stearoyl-ACP desaturase, putative | c	
EV166326	1.981	no similarity	
JCVI_41340	1.980	no original description	-2.576
EV202527	1.980	moderately similar to (304)AT5G09870| Symbols: CESA5 | CESA5 (CELLULOSE SYNTHASE 5); transferase, transferring glycosyl gr	
EV171174	1.979	very weakly similar to (87.4)AT5G64220| Symbols: | calmodulin-binding protein | chr5:25703660-25709129 FORWARD [21486] 89 10	-2.118
JCVI_10339	1.979	weakly similar to (185)AT4G21390| Symbols: B120 | B120; protein kinase/ sugar binding | chr4:11394469-11397485 REVERSE no orig	-1.615
EV085654	1.978	no similarity	
JCVI_38060	1.975	moderately similar to (340)AT3G25740| Symbols: MAP1C, MAP1B | MAP1B (METHIONINE AMINOPEPTIDASE 1C); metalloexop	
EE533226	1.975	moderately similar to (269)AT2G35160| Symbols: SUVH5 | SUVH5 (SU(VAR)3-9 HOMOLOG 5) | chr2:14830641-14833025 FORWA	
JCVI_35694	1.974	weakly similar to (186)AT4G18395| Symbols: | unknown protein | chr4:10169486-10169845 FORWARD no original description	
JCVI_31419	1.971	moderately similar to (377)AT4G03420| Symbols: | similar to unknown protein [Arabidopsis thaliana] (TAIR:AT1G03610.1); similar to	-1.442
JCVI_1644	1.971	moderately similar to (323)AT4G08780| Symbols: | peroxidase, putative | chr4:5604150-5608199 FORWARDweakly similar to (180)P	
JCVI_20846	1.970	no original description	-1.638
ES966314	1.969	no similarity	
JCVI_33773	1.968	moderately similar to (243)AT1G45207| Symbols: | remorin family protein | chr1:17133143-17135988 REVERSE no original descriptio	
JCVI_12874	1.968	weakly similar to (121)AT4G39795| Symbols: | senescence-associated protein-related | chr4:18466615-18467319 FORWARD no origin	
JCVI_22713	1.967	no original description	
EV140320	1.967	no similarity	
EV148338	1.967	very weakly similar to (82.4)AT1G78260| Symbols: | RNA recognition motif (RRM)-containing protein | chr1:29452210-29454139 FOR	
EX096295	1.964	no similarity	
JCVI_36284	1.964	weakly similar to (124)AT3G30460| Symbols: | zinc finger (C3HC4-type RING finger) family protein | chr3:12106912-12107355 FORV	
JCVI_20602	1.963	moderately similar to (454)AT2G44450| Symbols: | glycosyl hydrolase family 1 protein | chr2:18348042-18350820 FORWARDweakly	
JCVI_41898	1.963	moderately similar to (464)AT4G00970| Symbols: | protein kinase family protein | chr4:418437-421694 FORWARDweakly similar to (	-2.028
JCVI_74	1.961	moderately similar to (325)AT2G40330| Symbols: | Bet v I allergen family protein | chr2:16852255-16852902 REVERSE no original de	
EE513956	1.960	no similarity	
JCVI_14741	1.960	moderately similar to (389)AT1G10340| Symbols: | ankyrin repeat family protein | chr1:3390477-3392483 REVERSE no original descri	-3.153
JCVI_15381	1.959	moderately similar to (337)AT4G27540| Symbols: | prenylated rab acceptor (PRA1) protein-related | chr4:13753455-13754666 REVER	
JCVI_16066	1.958	moderately similar to (385)AT5G64640| Symbols: | pectinesterase family protein | chr5:25854046-25856279 FORWARD no original de	-1.890
JCVI_30744	1.957	no original description	
EE566454	1.955	weakly similar to (115)AT2G40435| Symbols: | transcription regulator | chr2:16894126-16895485 FORWARD [20153] 21 776 776	
CD817962	1.955	moderately similar to (254)AT3G59340| Symbols: | similar to unknown protein [Arabidopsis thaliana] (TAIR:AT3G59310.1); similar to	
EX088098	1.955	moderately similar to (310)AT5G48670| Symbols: FEM111, AGL80 | AGL80/FEM111 (AGAMOUS-LIKE80); DNA binding / transcrip	
EV008929	1.954	no similarity	
ES964477	1.952	no similarity	
RC_JCVI_31324	1.951	no original description	
AT000471	1.951	no similarity	-2.933
JCVI_21940	1.949	weakly similar to (191)AT2G38940| Symbols: PHT1;4, ATPT2 | ATPT2 (PHOSPHATE TRANSPORTER 2); carbohydrate transmembr	-2.562
CV544596	1.949	moderately similar to (228)AT2G17120| Symbols: LYM2 | LYM2 (LYSM DOMAIN GPI-ANCHORED PROTEIN 2 PRECURSOR) | c	
EV167051	1.949	moderately similar to (326)AT2G38010| Symbols: | ceramidase family protein | chr2:15913940-15916945 FORWARD [21486] 88 110	-1.726
EX126534	1.948	weakly similar to (118)AT3G55450| Symbols: | protein kinase, putative | chr3:20569106-20570940 FORWARD [21831]	-1.820
EV159250	1.948	weakly similar to (174)AT1G52040| Symbols: ATMBP, MBP1 | MBP1 (MYROSINASE-BINDING PROTEIN 1) | chr1:19354264-193	
JCVI_39557	1.948	weakly similar to (170)AT5G49300| Symbols: | zinc finger (GATA type) family protein | chr5:20002075-20002701 REVERSE no origin	
JCVI_11956	1.947	weakly similar to (183)AT1G26665| Symbols: | similar to RNA polymerase II mediator complex protein-related [Arabidopsis thaliana] (	-1.764
JCVI_17646	1.944	moderately similar to (243)AT4G23060| Symbols: IQD22 | IQD22 (IQ-domain 22); calmodulin binding | chr4:12087294-12090419 FOR	
EV148307	1.944	very weakly similar to (84.0)AT3G21055| Symbols: PSBTN | PSBTN (photosystem II subunit T) | chr3:7376767-7377078 REVERSE [2	
JCVI_21918	1.944	moderately similar to (320)AT4G27890| Symbols: | nuclear movement family protein | chr4:13886039-13887226 FORWARD no origina	
EE559032	1.939	no similarity	
EV130373	1.939	no similarity	
EV114502	1.939	moderately similar to (418)AT3G16940| Symbols: | calmodulin-binding protein | chr3:5781965-5785991 FORWARD [21479] 43 893 8	
DY029638	1.938	no similarity	
EV003450	1.936	no similarity	-1.798
JCVI_1508	1.935	moderately similar to (294)AT4G25260| Symbols: | invertase/pectin methylesterase inhibitor family protein | chr4:12936015-12936620 I	
EV182009	1.935	moderately similar to (273)AT3G12920| Symbols: | protein binding / zinc ion binding | chr3:4122134-4123330 REVERSE [21487]	
JCVI_13444	1.933	highly similar to (503)AT3G32980| Symbols: | peroxidase 32 (PER32) (P32) (PRXR3) | chr3:13530117-13533662 REVERSEmoderate	
JCVI_3880	1.932	moderately similar to (290)AT1G17860| Symbols: | trypsin and protease inhibitor family protein / Kunitz family protein | chr1:6149336-(	-1.763
JCVI_28644	1.931	moderately similar to (262)AT4G25240| Symbols: SKS1 | SKS1 (SKU5 SIMILAR 1); copper ion binding | chr4:12930549-12933573 FO	-1.833
ES948294	1.931	moderately similar to (358)AT3G06550| Symbols: | similar to O-acetyltransferase family protein [Arabidopsis thaliana] (TAIR:AT2G34	
JCVI_36533	1.929	moderately similar to (219)AT1G10960| Symbols: ATFD1 | ATFD1 (FERREDOXIN 1); 2 iron, 2 sulfur cluster binding / electron carrier	
DY016844	1.928	weakly similar to (147)AT1G52200| Symbols: | similar to unknown protein [Arabidopsis thaliana] (TAIR:AT3G18470.1); similar to unn	-2.420
JCVI_5662	1.928	no original description	
JCVI_18154	1.927	moderately similar to (240)AT1G11790| Symbols: ADT1 | ADT1 (AROGENATE DEHYDRATASE 1); prephenate dehydratase | chr1:3	
JCVI_32589	1.926	moderately similar to (461)AT2G43040| Symbols: NPG1 | NPG1 (NO POLLEN GERMINATION 1); calmodulin binding | chr2:179031	
JCVI_39690	1.926	moderately similar to (332)AT5G06250| Symbols: | similar to DNA-binding protein, putative [Arabidopsis thaliana] (TAIR:AT3G11580	
JCVI_33515	1.925	moderately similar to (354)AT5G13280| Symbols: AK, AK1, AK-LYS1 | AK-LYS1 (ASPARTATE KINASE 1) | chr5:4249519-425265	
JCVI_41206	1.925	moderately similar to (273)AT2G23200| Symbols: | protein kinase family protein | chr2:9886431-9888935 FORWARD no original desc	-1.818
JCVI_1837	1.922	moderately similar to (392)AT1G62740| Symbols: | stress-inducible protein, putative | chr1:23234691-23237045 FORWARDmoderately	-1.438
EE558876	1.922	no similarity	-2.588

JCVI_13113	1.921	moderately similar to (426)AT3G06490| Symbols: AtMYB108, BOS1, MYB108 | MYB108 (MYB DOMAIN PROTEIN 108); DNA bi	
JCVI_4349	1.921	highly similar to (590)AT3G21190| Symbols: | similar to unknown protein [Arabidopsis thaliana] (TAIR:AT1G51630.1); similar to unkr	
JCVI_8089	1.918	highly similar to (563)AT4G39980| Symbols: DHS1 | DHS1 (3-DEOXY-D-ARABINO-HEPTULOSONATE 7-PHOSPHATE SYNTHA	
JCVI_9044	1.918	weakly similar to (167)AT3G54880| Symbols: | similar to unknown protein [Arabidopsis thaliana] (TAIR:AT5G25360.1); similar to unn	
ES948539	1.917	no similarity	
JCVI_22117	1.915	moderately similar to (352)AT1G79720| Symbols: | aspartyl protease family protein | chr1:30002152-30003844 REVERSE no original c	-2.295
JCVI_2558	1.915	weakly similar to (189)AT2G28550| Symbols: RAP2.7, TOE1 | RAP2.7/TOE1 (TARGET OF EAT1 1); DNA binding / transcription fac	
EX042209	1.914	moderately similar to (426)AT4G19120| Symbols: ERD3 | ERD3 (EARLY-RESPONSIVE TO DEHYDRATION 3) | chr4:10460676-10	
JCVI_38987	1.911	weakly similar to (190)AT3G28220| Symbols: | meprin and TRAF homology domain-containing protein / MATH domain-containing pro	
JCVI_13699	1.911	moderately similar to (239)AT4G20780| Symbols: | calcium-binding protein, putative | chr4:11133320-11133895 REVERSEweakly sim	
JCVI_37556	1.908	no original description	
JCVI_37633	1.908	very weakly similar to (80.1)AT1G19180| Symbols: JAZ1, TIFY10A | JAZ1/TIFY10A (JASMONATE-ZIM-DOMAIN PROTEIN 1) | ch	
JCVI_17841	1.908	weakly similar to (182)AT3G11840| Symbols: | U-box domain-containing protein | chr3:3736584-3738256 REVERSE no original descr	-2.219
JCVI_22404	1.905	highly similar to (645)AT1G24320| Symbols: | alpha-glucosidase, putative | chr1:8626426-8630924 REVERSE no original description	-2.818
JCVI_4121	1.899	moderately similar to (241)AT1G15100| Symbols: RHA2A | RHA2A (RING-H2 finger A2A); protein binding / zinc ion binding | chr1:51	
JCVI_36466	1.896	weakly similar to (150)AT1G11260| Symbols: STP1 | STP1 (SUGAR TRANSPORTER 1); carbohydrate transmembrane transporter/ sug	-1.493
CX194935	1.896	weakly similar to (175)AT4G37180| Symbols: | myb family transcription factor | chr4:17504642-17506101 FORWARD [16807]	
JCVI_20063	1.893	highly similar to (890)AT1G30760| Symbols: | FAD-binding domain-containing protein | chr1:10918303-10920423 FORWARD no orig	
EV214299	1.892	no similarity	
EE563266	1.890	no similarity	
JCVI_3491	1.890	no original description	-1.900
JCVI_21306	1.890	moderately similar to (330)AT3G09035| Symbols: | legume lectin family protein | chr3:2759072-2760088 FORWARD no original descr	
JCVI_21710	1.889	moderately similar to (369)AT3G05120| Symbols: ATGID1A, GID1A | ATGID1A/GID1A (GA INSENSITIVE DWARF1A); hydrolase	-2.071
JCVI_35773	1.887	moderately similar to (257)AT1G07250| Symbols: | UDP-glucoronosyl/UDP-glucosyl transferase family protein | chr1:2225962-222740	
EE505319	1.887	very weakly similar to (96.3)AT1G52580| Symbols: | rhomboid family protein | chr1:19591306-19592627 FORWARD [20139]	-2.660
JCVI_30987	1.887	moderately similar to (425)AT1G21660| Symbols: | heat shock protein binding | chr1:7605913-7608824 FORWARD no original descrip	
JCVI_40644	1.884	moderately similar to (285)AT3G19850| Symbols: | phototropic-responsive NPH3 family protein | chr3:6898389-6901163 REVERSE no	
EV110630	1.884	no similarity	-1.536
JCVI_19759	1.883	weakly similar to (173)AT4G02390| Symbols: APP | APP (ARABIDOPSIS POLY(ADP-RIBOSE) POLYMERASE); NAD+ ADP-ribos	
JCVI_17966	1.883	moderately similar to (224)AT4G11360| Symbols: RHA1B | RHA1B (RING-H2 finger A1B); protein binding / zinc ion binding | chr4:69	-1.720
EX042932	1.883	weakly similar to (166)AT5G48930| Symbols: HCT | transferase family protein | chr5:19853880-19855318 REVERSE [21811]	
ES937277	1.881	very weakly similar to (98.2)AT2G22795| Symbols: | similar to unknown protein [Arabidopsis thaliana] (TAIR:AT4G37820.1); similar to	
JCVI_1861	1.881	moderately similar to (351)AT3G04720| Symbols: HEL, PR-4, PR4 | PR4 (PATHOGENESIS-RELATED 4) | chr3:1285697-1286537 R	
JCVI_13092	1.880	moderately similar to (352)AT1G72450| Symbols: JAZ6, TIFY11B | JAZ6/TIFY11B (JASMONATE-ZIM-DOMAIN PROTEIN 6) | chr	
JCVI_28995	1.879	weakly similar to (164)AT4G24340| Symbols: | phosphorylase family protein | chr4:12607485-12609158 FORWARD no original descri	
JCVI_6159	1.873	moderately similar to (427)AT3G23820| Symbols: GAE6 | GAE6 (UDP-D-GLUCURONATE 4-EPIMERASE 6); catalytic | chr3:860365	-1.362
JCVI_10067	1.872	moderately similar to (492)AT1G52410| Symbols: TSA1 | TSA1 (TSK-ASSOCIATING PROTEIN 1) | chr1:19524431-19529030 FORV	
EX100282	1.871	moderately similar to (290)AT3G61460| Symbols: BRH1 | BRH1 (BRASSINOSTEROID-RESPONSIVE RING-H2); protein binding / z	-1.741
EX100783	1.870	weakly similar to (146)AT3G05510| Symbols: | phospholipid/glycerol acyltransferase family protein | chr3:1596139-1598080 FORWAR	
EX090309	1.869	weakly similar to (198)AT1G61260| Symbols: | similar to unknown protein [Arabidopsis thaliana] (TAIR:AT1G11220.1); similar to unk	
L47851	1.868	weakly similar to (142)AT4G34200| Symbols: EDA9 | EDA9 (embryo sac development arrest 9); NAD binding / amino acid binding / bi	
JCVI_18216	1.868	moderately similar to (233)AT2G37630| Symbols: ATPHAN, AS1, ATMYB91, MYB91 | AS1/ATMYB91/ATPHAN/MYB91 (ASYMI	
JCVI_36942	1.868	very weakly similar to (84.0)AT5G54170| Symbols: | similar to CP5 [Arabidopsis thaliana] (TAIR:AT1G64720.1); similar to putative no	
JCVI_21308	1.867	moderately similar to (496)AT1G64760| Symbols: | glycosyl hydrolase family 17 protein | chr1:24057883-24059857 REVERSEweakly	
JCVI_16013	1.866	very weakly similar to (94.7)AT3G13480| Symbols: | similar to unknown protein [Arabidopsis thaliana] (TAIR:AT1G55475.1); similar to	
DY022790	1.866	weakly similar to (192)AT1G08940| Symbols: | phosphoglycerate/bisphosphoglycerate mutase family protein | chr1:2877697-2879107 F	
JCVI_33527	1.866	weakly similar to (140)AT2G44670| Symbols: | senescence-associated protein-related | chr2:18432354-18432748 FORWARD no origin	
JCVI_2647	1.866	moderately similar to (315)AT3G02230| Symbols: ATRGP1, ATRGP, RGP1 | RGP1 (REVERSIBLY GLYCOSYLATED POLYPEPTI	-2.433
EV124681	1.865	moderately similar to (349)AT5G10720| Symbols: CKI2, AHK5 | AHK5 (CYTOKININ INDEPENDENT 2) | chr5:3386836-3390542 F	-2.123
JCVI_16498	1.864	moderately similar to (218)AT2G35860| Symbols: FLA16 | FLA16 (FASCICLIN-LIKE ARABINOGALACTAN PROTEIN 16 PRECUI	
JCVI_36550	1.864	moderately similar to (347)AT5G06730| Symbols: | peroxidase, putative | chr5:2080208-2081622 REVERSEmoderately similar to (223	-2.204
AM056911	1.863	no similarity	
EX135288	1.863	very weakly similar to (89.4)AT5G06860| Symbols: PGIP1 | PGIP1 (POLYGALACTURONASE INHIBITING PROTEIN 1); protein bir	
EV130246	1.863	no similarity	
EX134666	1.862	weakly similar to (167)AT1G71400| Symbols: | disease resistance family protein / LRR family protein | chr1:26913567-26916110 FORV	-2.394
JCVI_39785	1.862	weakly similar to (129)AT5G47550| Symbols: | cysteine protease inhibitor, putative / cystatin, putative | chr5:19303822-19304190 REV	
JCVI_27	1.861	moderately similar to (370)AT3G17860| Symbols: JAZ3, JAI3, TIFY6B | JAI3/JAZ3/TIFY6B (JASMONATE-ZIM-DOMAIN PROTEI	
JCVI_28627	1.861	moderately similar to (208)AT1G27360| Symbols: | squamosa promoter-binding protein-like 11 (SPL11) | chr1:9502126-9503702 FORV	
JCVI_28416	1.861	moderately similar to (249)AT3G03160| Symbols: | similar to unknown protein [Arabidopsis thaliana] (TAIR:AT5G17190.1); similar to	
JCVI_25733	1.861	moderately similar to (289)AT5G20680| Symbols: | similar to unknown protein [Arabidopsis thaliana] (TAIR:AT5G64020.1); similar to	-2.882
JCVI_726	1.858	moderately similar to (440)AT5G06860| Symbols: PGIP1 | PGIP1 (POLYGALACTURONASE INHIBITING PROTEIN 1); protein bin	
AM395963	1.857	very weakly similar to (99.4)AT3G54870| Symbols: MRH2 | MRH2 (morphogenesis of root hair 2); microtubule motor | chr3:20341784-2	
JCVI_23384	1.857	moderately similar to (407)AT2G33170| Symbols: | leucine-rich repeat transmembrane protein kinase, putative | chr2:14063448-140669	-1.611
EE512835	1.855	no similarity	-1.958
JCVI_31269	1.855	moderately similar to (253)AT3G51080| Symbols: | zinc finger (GATA type) family protein | chr3:18984619-18985648 FORWARD no	-1.884
JCVI_8519	1.854	no original description	
CD827548	1.854	very weakly similar to (94.4)AT5G04560| Symbols: DME1, DME | DME (DEMETER) | chr5:1309787-1318092 FORWARD [13979]	
EX051456	1.853	no similarity	-1.895
JCVI_25700	1.851	highly similar to (503)AT3G47340| Symbols: DIN6, AT-ASN1, ASN1 | ASN1 (DARK INDUCIBLE 6) | chr3:17449430-17452028 REV	
JCVI_35588	1.851	weakly similar to (140)AT3G49120| Symbols: ATPERX34, PERX34, PRXCB, ATPCB | ATPCB/ATPERX34/PERX34/PRXCB (PERC	
JCVI_42447	1.850	moderately similar to (356)AT4G00500| Symbols: | lipase class 3 family protein / calmodulin-binding heat-shock protein-related | chr4:2	
JCVI_8314	1.849	moderately similar to (402)AT4G34200| Symbols: EDA9 | EDA9 (embryo sac development arrest 9); NAD binding / amino acid binding	-1.613
EE559065	1.848	no similarity	
EX114325	1.847	weakly similar to (132)AT1G14010| Symbols: | emp24/gp25L/p24 family protein | chr1:4800382-4801787 REVERSE [21827]	-2.265
JCVI_1332	1.846	moderately similar to (314)AT2G22170| Symbols: | lipid-associated family protein | chr2:9434090-9434822 REVERSE no original desc	
JCVI_33298	1.845	moderately similar to (432)AT1G07220| Symbols: | similar to unknown protein [Arabidopsis thaliana] (TAIR:AT5G23850.1); similar to	
JCVI_8259	1.844	weakly similar to (194)AT2G35860| Symbols: FLA16 | FLA16 (FASCICLIN-LIKE ARABINOGALACTAN PROTEIN 16 PRECURSO	
ES913294	1.844	moderately similar to (315)AT1G51860| Symbols: | leucine-rich repeat protein kinase, putative | chr1:19261303-19265148 REVERSE [2	-2.290
JCVI_20639	1.840	moderately similar to (229)AT4G36920| Symbols: FLO2, FL1, AP2 | AP2 (APETALA 2); transcription factor | chr4:17400995-1740313	
JCVI_20101	1.840	weakly similar to (130)AT1G65960| Symbols: GAD2 | GAD2 (GLUTAMATE DECARBOXYLASE 2) | chr1:24555757-24560916 FOR	-1.652

JCVI_33169	1.838	moderately similar to (485)AT3G02590| Symbols: | delta 7-sterol-C5-desaturase, putative | chr3:549347-550560 FORWARDmoderately	-1.432
JCVI_4772	1.838	moderately similar to (263)AT4G35480| Symbols: RHA3B | RHA3B (RING-H2 finger A3B); protein binding / zinc ion binding | chr4:16	
JCVI_36728	1.838	moderately similar to (267)AT2G40230| Symbols: | transferase family protein | chr2:16810363-16811664 REVERSE no original descrip	-1.816
EV011511	1.838	no similarity	
EE561659	1.836	no similarity	
EV168965	1.836	no similarity	
JCVI_29802	1.833	highly similar to (603)AT4G26970| Symbols: | aconitate hydratase, cytoplasmic, putative / citrate hydro-lyase/aconitase, putative | chr4:1	-3.400
JCVI_19333	1.832	weakly similar to (162)AT4G35380| Symbols: | guanine nucleotide exchange family protein | chr4:16819888-16825965 FORWARD no	-2.471
CV434041	1.832	weakly similar to (140)AT1G72280| Symbols: AERO1 | AERO1 (ARABIDOPSIS ENDOPLASMIC RETICULUM OXIDOREDUCTIN	-1.796
JCVI_37512	1.830	moderately similar to (379)AT3G53620| Symbols: ATPPA4 | ATPPA4 (ARABIDOPSIS THALIANA PYROPHOSPHORYLASE 4); in	
JCVI_17933	1.827	weakly similar to (128)AT2G42580| Symbols: TTL3 | TTL3 (TETRATRICOPETIDE-REPEAT THIOREDOXIN-LIKE 3); protein bind	
JCVI_34669	1.827	moderately similar to (248)AT2G04240| Symbols: XERICO | XERICO; protein binding / zinc ion binding | chr2:1461813-1462301 REV	
JCVI_10188	1.827	moderately similar to (209)AT3G07510| Symbols: | similar to unknown protein [Arabidopsis thaliana] (TAIR:AT2G01580.1); similar to	-1.552
JCVI_18443	1.826	moderately similar to (382)AT2G38400| Symbols: AGT3 | AGT3 (ALANINE:GLYOXYLATE AMINOTRANSFERASE 3); alanine-gly	-2.191
JCVI_10347	1.825	moderately similar to (348)AT2G22900| Symbols: | galactosyl transferase GMA12/MNN10 family protein | chr2:9751439-9753273 RE	
EV148141	1.824	no similarity	
JCVI_39469	1.824	moderately similar to (286)AT1G80510| Symbols: | amino acid transporter family protein | chr1:30277992-30279461 FORWARD no or	-1.914
EV072164	1.823	very weakly similar to (81.3)AT4G00440| Symbols: | similar to unknown protein [Arabidopsis thaliana] (TAIR:AT2G45900.1); similar to	
JCVI_21863	1.822	moderately similar to (377)AT1G51850| Symbols: | leucine-rich repeat protein kinase, putative | chr1:19256633-19260452 REVERSEve	-1.743
JCVI_37620	1.822	moderately similar to (330)AT2G40280| Symbols: | dehydration-responsive family protein | chr2:16832785-16835378 REVERSE no ori	
EV121562	1.822	weakly similar to (155)AT5G15470| Symbols: GAUT14 | GAUT14 (Galacturonosyltransferase 14); polygalacturonate 4-alpha-galacturon	-1.675
EX069519	1.821	weakly similar to (191)AT3G13480| Symbols: | similar to unknown protein [Arabidopsis thaliana] (TAIR:AT1G55475.1); similar to unn	
JCVI_17559	1.820	moderately similar to (217)AT2G46650| Symbols: B5 #1 | B5 #1 (cytochrome b5 family protein #1); heme binding / transition metal ion	
EE526435	1.819	moderately similar to (268)AT3G52890| Symbols: KIPK | KIPK (KCBP-INTERACTING PROTEIN KINASE); kinase | chr3:19620128-	
JCVI_26255	1.819	highly similar to (647)AT4G02330| Symbols: ATPMEPCRB | ATPMEPCRB; pectinesterase | chr4:1032479-1034928 FORWARDmode	-2.725
JCVI_377	1.818	moderately similar to (403)AT5G06860| Symbols: PGIP1 | PGIP1 (POLYGALACTURONASE INHIBITING PROTEIN 1); protein bind	
JCVI_7437	1.818	moderately similar to (234)AT1G48320| Symbols: | thioesterase family protein | chr1:17858692-17859245 REVERSE no original descri	-2.946
EX029032	1.817	weakly similar to (181)AT2G37678| Symbols: PAT3, FRY1, FHY1 | FHY1 (FAR-RED ELONGATED HYPOCOTYL 1) | chr2:158087	
EE533233	1.817	moderately similar to (209)AT2G43820| Symbols: GT, UGT74F2 | GT/UGT74F2 (UDP-GLUCOSYLTRANSFERASE 74F2); UDP-glu	-2.082
EV176160	1.815	weakly similar to (114)AT5G06860| Symbols: PGIP1 | PGIP1 (POLYGALACTURONASE INHIBITING PROTEIN 1); protein binding	
JCVI_30299	1.815	moderately similar to (280)AT1G01120| Symbols: KCS1 | KCS1 (3-KETOACYL-COA SYNTHASE 1); acyltransferase | chr1:57392-58	
EV105785	1.814	moderately similar to (274)AT5G04540| Symbols: | inositol or phosphatidylinositol phosphatase/ phosphoric monoester hydrolase/ prote	
JCVI_35041	1.814	highly similar to (640)AT5G25880| Symbols: ATNADP-ME3 | ATNADP-ME3 (NADP-MALIC ENZYME 3); malate dehydrogenase (o	
JCVI_8424	1.812	moderately similar to (416)AT1G08940| Symbols: | phosphoglycerate/bisphosphoglycerate mutase family protein | chr1:2877697-28791	
JCVI_18243	1.812	moderately similar to (286)AT1G50420| Symbols: SCL-3, SCL3 | SCL3 (SCARECROW-LIKE 3); transcription factor | chr1:18681845-	-1.525
JCVI_41655	1.809	moderately similar to (297)AT4G35160| Symbols: | O-methyltransferase family 2 protein | chr4:16730994-16732813 REVERSE no orig	
EE544434	1.809	weakly similar to (194)AT1G75030| Symbols: ATLP-3 | ATLP-3 (Arabidopsis thaumatin-like protein 3) | chr1:28178079-28178916 FOR	-2.406
JCVI_35217	1.807	moderately similar to (358)AT3G16220| Symbols: | similar to RNA binding / catalytic [Arabidopsis thaliana] (TAIR:AT3G16230.1); sin	-2.055
JCVI_34749	1.806	moderately similar to (233)AT5G11890| Symbols: | similar to unknown protein [Arabidopsis thaliana] (TAIR:AT1G17620.1); similar to	
EE449986	1.806	weakly similar to (129)AT2G29420| Symbols: GST25, ATGSTU7 | ATGSTU7 (GLUTATHIONE S-TRANSFERASE 25); glutathione t	-2.153
JCVI_41002	1.804	moderately similar to (446)AT3G53810| Symbols: | lectin protein kinase, putative | chr3:19944131-19946164 REVERSE no original des	
JCVI_8040	1.804	weakly similar to (187)AT5G66040| Symbols: STR16 | STR16 (SULFURTRANSFERASE PROTEIN 16) | chr5:26427783-26428365 F	
JCVI_13125	1.803	no original description	-2.061
JCVI_8286	1.803	highly similar to (569)AT5G09870| Symbols: CESA5 | CESA5 (CELLULOSE SYNTHASE 5); transferase, transferring glycosyl groups	
JCVI_17890	1.802	moderately similar to (225)AT4G27830| Symbols: | glycosyl hydrolase family 1 protein | chr4:13861800-13864495 REVERSE no origin	
EX042007	1.802	moderately similar to (291)AT1G56670| Symbols: | GDSL-motif lipase/hydrolase family protein | chr1:21245353-21247630 FORWARD	
JCVI_21613	1.800	moderately similar to (215)AT4G26690| Symbols: MRH5, SHV3 | MRH5/SHV3 (morphogenesis of root hair 5); glycerophosphodiester	-1.934
EV125354	1.800	weakly similar to (188)AT2G43240| Symbols: | similar to ATUTR6/UTR6 (UDP-GALACTOSE TRANSPORTER 6), nucleotide-sugar	
EE548031	1.800	no similarity	-1.607
EX040023	1.799	no similarity	
DY000659	1.799	moderately similar to (240)AT3G16330| Symbols: | similar to unknown protein [Arabidopsis thaliana] (TAIR:AT1G52140.1); similar to	
JCVI_37161	1.799	moderately similar to (410)AT1G15520| Symbols: PDR12, ATPDR12 | ATPDR12/PDR12 (PLEIOTROPIC DRUG RESISTANCE 12);	-2.696
JCVI_27846	1.794	weakly similar to (154)AT4G27870| Symbols: | integral membrane family protein | chr4:13878989-13882685 FORWARD no original de	
JCVI_21437	1.794	weakly similar to (124)AT1G25220| Symbols: TRP4, ASB1 | ASB1 (ANTHRANILATE SYNTHASE BETA SUBUNIT 1); anthranilate	
CD837078	1.794	no similarity	
EE438909	1.794	no similarity	
JCVI_15879	1.793	moderately similar to (281)AT2G39360| Symbols: | protein kinase family protein | chr2:16444670-16447117 REVERSEvery weakly sim	
JCVI_13873	1.793	highly similar to (675)AT1G10700| Symbols: | ribose-phosphate pyrophosphokinase 3 / phosphoribosyl diphosphate synthetase 3 (PRS3	
JCVI_10992	1.793	no original description	
JCVI_7198	1.793	moderately similar to (357)AT3G16940| Symbols: | calmodulin-binding protein | chr3:5781965-5785991 FORWARD no original descrip	
EV117919	1.792	moderately similar to (317)AT4G19960| Symbols: | potassium ion transmembrane transporter | chr4:10813817-10817007 FORWARDm	
JCVI_34836	1.791	highly similar to (838)AT2G24240| Symbols: | potassium channel tetramerisation domain-containing protein | chr2:10317918-10319243	
JCVI_4168	1.789	moderately similar to (290)AT3G12920| Symbols: | protein binding / zinc ion binding | chr3:4122134-4123330 REVERSE no original de	-2.100
JCVI_41757	1.789	moderately similar to (291)AT1G14010| Symbols: | emp24/gp25L/p24 family protein | chr1:4800382-4801787 REVERSE no original de	-2.633
JCVI_37890	1.789	highly similar to (610)AT5G64640| Symbols: | pectinesterase family protein | chr5:25854046-25856279 FORWARDmoderately similar	
EV021806	1.788	weakly similar to (147)AT2G40095| Symbols: | similar to unknown protein [Arabidopsis thaliana] (TAIR:AT3G55880.2); similar to unn	
JCVI_25914	1.787	no original description	
EE562954	1.787	moderately similar to (204)AT1G15520| Symbols: PDR12, ATPDR12 | ATPDR12/PDR12 (PLEIOTROPIC DRUG RESISTANCE 12);	
JCVI_2257	1.787	moderately similar to (239)AT1G09310| Symbols: | similar to unknown protein [Arabidopsis thaliana] (TAIR:AT1G56580.1); similar to	
EE561336	1.786	no similarity	
EV010170	1.786	no similarity	
EV030996	1.786	no similarity	
JCVI_39513	1.785	weakly similar to (114)AT5G13320| Symbols: GDG1, WIN3, PBS3 | PBS3 (AVRPPHB SUSCEPTIBLE 3) | chr5:4268905-4270899 FO	-2.280
JCVI_15377	1.785	highly similar to (547)AT2G33070| Symbols: | jacalin lectin family protein | chr2:14036427-14038011 REVERSE no original descriptio	
EE443421	1.785	weakly similar to (190)AT1G07570| Symbols: APK1, APK1A | APK1A (Arabidopsis protein kinase 1A); kinase | chr1:2331366-2333207	
JCVI_6901	1.784	moderately similar to (419)AT2G02950| Symbols: PKS1 | PKS1 (PHYTOCHROME KINASE SUBSTRATE 1) | chr2:855148-856467 F	
EV091475	1.784	moderately similar to (329)AT2G42580| Symbols: TTL3 | TTL3 (TETRATRICOPETIDE-REPEAT THIOREDOXIN-LIKE 3); protein b	
EX017039	1.783	weakly similar to (148)AT1G80790| Symbols: | XH/XS domain-containing protein / XS zinc finger domain-containing protein | chr1:303	
EX063822	1.782	no similarity	
JCVI_5865	1.782	moderately similar to (396)AT5G17760| Symbols: | AAA-type ATPase family protein | chr5:5861278-5862303 REVERSE no original d	-1.887

EX036279	1.780	no similarity	-1.916
JCVI_15667	1.778	moderately similar to (256)AT1G07050| Symbols: | CONSTANS-like protein-related | chr1:2164326-2165132 REVERSE no original de	
CX191033	1.778	very weakly similar to (98.6)AT3G13910| Symbols: | similar to unknown protein [Arabidopsis thaliana] (TAIR:AT2G19460.1); similar to	
DN961805	1.777	very weakly similar to (85.5)AT1G08450| Symbols: CRT3 | CRT3 (CALRETICULIN 3); calcium ion binding | chr1:2668005-2671797 R	-1.634
EV218670	1.773	moderately similar to (282)AT5G46330| Symbols: FLS2 | FLS2 (FLAGELLIN-SENSITIVE 2); ATP binding / kinase/ protein binding / p	
JCVI_30864	1.773	weakly similar to (156)AT5G55890| Symbols: | similar to unknown protein [Arabidopsis thaliana] (TAIR:AT5G55880.1); contains Inter	-1.858
JCVI_3139	1.772	moderately similar to (320)AT4G34131| Symbols: UGT73B3 | UGT73B3 (UDP-GLUCOSYL TRANSFERASE 73B3); UDP-glycosyltra	
CX190718	1.772	weakly similar to (132)AT1G25370| Symbols: | similar to unknown protein [Arabidopsis thaliana] (TAIR:AT1G68340.1); similar to unn	-1.778
JCVI_1766	1.772	weakly similar to (147)AT3G23820| Symbols: GAE6 | GAE6 (UDP-D-GLUCURONATE 4-EPIMERASE 6); catalytic | chr3:8603652-8	
JCVI_4621	1.769	weakly similar to (153)AT4G39260| Symbols: GR-RBP8, ATGRP8, CCR1 | ATGRP8/GR-RBP8 (COLD, CIRCADIAN RHYTHM, AN	-1.444
JCVI_40346	1.768	weakly similar to (101)AT5G39865| Symbols: | glutaredoxin family protein | chr5:15982788-15983960 REVERSE no original descriptio	
JCVI_19587	1.768	moderately similar to (363)AT1G66620| Symbols: | seven in absentia (SINA) protein, putative | chr1:24856469-24857707 REVERSEve	-1.856
EX127400	1.767	moderately similar to (367)AT1G11330| Symbols: | S-locus lectin protein kinase family protein | chr1:3810372-3813416 FORWARDwe	-1.870
EV159729	1.767	moderately similar to (280)AT2G44210| Symbols: | similar to unknown protein [Arabidopsis thaliana] (TAIR:AT1G55360.1); similar to	
JCVI_1817	1.763	moderately similar to (438)AT2G06050| Symbols: OPR3 | OPR3 (OPDA-REDUCTASE 3) | chr2:2359237-2361968 REVERSE no orig	
EV105581	1.763	no similarity	-1.920
EV006132	1.763	no similarity	-2.969
JCVI_2291	1.762	very weakly similar to (94.4)AT1G28330| Symbols: DRM1 | DRM1 (DORMANCY-ASSOCIATED PROTEIN 1) | chr1:9934487-99352	
JCVI_18578	1.761	highly similar to (602)AT5G18470| Symbols: | curculin-like (mannose-binding) lectin family protein | chr5:6127954-6129195 FORWAR	-2.152
DY023750	1.760	no similarity	-1.297
JCVI_41019	1.760	no original description	
JCVI_21249	1.760	highly similar to (666)AT2G38290| Symbols: ATAMT2 | ATAMT2 (AMMONIUM TRANSPORTER 2); ammonium transmembrane tra	
JCVI_2487	1.759	moderately similar to (262)AT4G37180| Symbols: | myb family transcription factor | chr4:17504642-17506101 FORWARD no original	
JCVI_9196	1.756	highly similar to (731)AT5G26600| Symbols: | catalytic/ pyridoxal phosphate binding | chr5:9377458-9378885 FORWARD no original	-1.493
JCVI_1514	1.755	moderately similar to (479)AT2G36880| Symbols: MAT3 | MAT3 (METHIONINE ADENOSYLTRANSFERASE 3) | chr2:15486800-1	
JCVI_21065	1.754	highly similar to (521)AT1G13080| Symbols: CYP71B2 | CYP71B2 (CYTOCHROME P450 71B2); oxygen binding | chr1:4459491-44	
JCVI_12003	1.753	weakly similar to (143)AT2G01670| Symbols: ATNUDT17 | ATNUDT17 (Arabidopsis thaliana Nudix hydrolase homolog 17); hydrolas	
JCVI_7925	1.752	weakly similar to (166)AT1G03170| Symbols: | similar to unknown protein [Arabidopsis thaliana] (TAIR:AT4G02810.1); similar to unn	
EX032983	1.751	weakly similar to (160)AT1G69520| Symbols: | methyltransferase-related | chr1:26131951-26133265 FORWARD [21810]	-2.722
EV126123	1.751	weakly similar to (138)AT1G77760| Symbols: GNR1, NR1, NIA1 | NIA1 (NITRATE REDUCTASE 1) | chr1:29240899-29244261 REV	
CX272527	1.749	weakly similar to (171)AT5G19960| Symbols: | RNA recognition motif (RRM)-containing protein | chr5:6744031-6746135 FORWARD	-1.627
AM387983	1.749	no similarity	-1.464
CX193178	1.749	moderately similar to (260)AT5G22740| Symbols: CSLA02, ATCSLA2, ATCSLA02 | ATCSLA02 (Cellulose synthase-like A2); transfer	
EH413958	1.749	weakly similar to (114)AT3G21760| Symbols: | UDP-glucoronosyl/UDP-glucosyl transferase family protein | chr3:7667106-7668563 FC	
ES265763	1.748	moderately similar to (301)AT1G80510| Symbols: | amino acid transporter family protein | chr1:30277992-30279461 FORWARD [210	-2.118
JCVI_34174	1.747	weakly similar to (187)AT4G37730| Symbols: ATBZIP7 | ATBZIP7 (ARABIDOPSIS THALIANA BASIC LEUCINE-ZIPPER 7); DNA	
JCVI_8973	1.746	moderately similar to (462)AT5G44070| Symbols: ARA8, ATPCS1, PCS1, CAD1 | CAD1 (CADMIUM SENSITIVE 1) | chr5:177521	-2.110
EE569212	1.744	weakly similar to (157)AT5G27430| Symbols: | signal peptidase subunit family protein | chr5:9687473-9689188 FORWARDweakly sim	-1.880
EE568131	1.744	no similarity	
JCVI_16546	1.742	moderately similar to (398)AT1G69780| Symbols: ATHB13 | ATHB13; DNA binding / transcription factor | chr1:26262829-26264128 F	-2.235
ES992845	1.741	moderately similar to (241)AT5G66840| Symbols: | SAP domain-containing protein | chr5:26710148-26712522 REVERSE [21427] 14	
EE416328	1.740	no similarity	
JCVI_2854	1.739	moderately similar to (201)AT4G14420| Symbols: | lesion inducing protein-related | chr4:8302167-8303734 REVERSE no original desc	
JCVI_20059	1.738	highly similar to (537)AT4G30080| Symbols: ARF16 | ARF16 (AUXIN RESPONSE FACTOR 16); miRNA binding / transcription facto	-1.146
CD826017	1.737	moderately similar to (239)AT2G02080| Symbols: ATIDD4 | ATIDD4 (ARABIDOPSIS THALIANA INDETERMINATE(ID)-DOMAI	-1.884
EE449610	1.736	weakly similar to (176)AT2G23380| Symbols: ICU1, SDG1, SET1, CLF | CLF (CURLY LEAF); transcription factor | chr2:9962650-996	
JCVI_10491	1.736	no original description	
JCVI_22897	1.735	moderately similar to (377)AT5G57150| Symbols: | basic helix-loop-helix (bHLH) family protein | chr5:23169587-23170519 FORWAR	
EV064753	1.735	moderately similar to (297)AT5G13670| Symbols: | nodulin MtN21 family protein | chr5:4407208-4408958 REVERSE [21443]	
JCVI_5561	1.734	moderately similar to (259)AT2G28400| Symbols: | similar to unknown protein [Arabidopsis thaliana] (TAIR:AT5G60680.1); similar to	-2.069
RC_AM386974	1.733	no similarity	
DY021602	1.733	weakly similar to (134)AT3G14350| Symbols: SRF7 | SRF7 (STRUBBELIG-RECEPTOR FAMILY 7); ATP binding / protein serine/thr	
JCVI_8371	1.732	moderately similar to (207)AT5G03040| Symbols: IQD2 | IQD2 (IQ-domain 2); calmodulin binding | chr5:710378-712405 REVERSE no	
EE448614	1.732	weakly similar to (152)AT2G28080| Symbols: | glycosyltransferase family protein | chr2:11967851-11970304 REVERSE [20172]	
EV166752	1.730	weakly similar to (199)AT5G02320| Symbols: ATMYB3R5, MYB3R-5 | MYB3R-5 (myb domain protein 3R-5); DNA binding / transcri	-1.008
AM056965	1.730	weakly similar to (109)AT4G15990| Symbols: | similar to unknown protein [Arabidopsis thaliana] (TAIR:AT4G16024.1) | chr4:9061281	
JCVI_30551	1.724	weakly similar to (166)AT4G37180| Symbols: | myb family transcription factor | chr4:17504642-17506101 FORWARD no original desc	
CX280967	1.724	no similarity	
JCVI_39056	1.723	moderately similar to (284)AT4G36010| Symbols: | pathogenesis-related thaumatin family protein | chr4:17039475-17040979 REVERS	
JCVI_15684	1.723	moderately similar to (306)AT4G34950| Symbols: | nodulin family protein | chr4:16642549-16644764 REVERSE no original descriptio	
EX135068	1.723	moderately similar to (292)AT4G24340| Symbols: | phosphorylase family protein | chr4:12607485-12609158 FORWARDvery weakly si	
ES964329	1.722	no similarity	
EV221410	1.722	no similarity	
JCVI_2551	1.721	moderately similar to (434)AT4G32410| Symbols: CESA1, RSW1 | CESA1 (CELLULOSE SYNTHASE 1); transferase, transferring gly	
JCVI_26471	1.720	weakly similar to (133)AT1G20970| Symbols: | adhesin-related | chr1:7314327-7319235 FORWARD no original description	
JCVI_109	1.719	moderately similar to (493)AT4G31500| Symbols: SUR2, RNT1, RED1, ATR4, CYP83B1 | CYP83B1 (CYTOCHROME P450 MONO	-2.171
EV183450	1.718	moderately similar to (246)AT4G34980| Symbols: SLP2 | SLP2 (subtilisin-like serine protease 2); subtilase | chr4:16656934-16659228 F	-4.696
JCVI_20187	1.717	moderately similar to (464)AT5G18760| Symbols: | zinc finger (C3HC4-type RING finger) family protein | chr5:6258510-6260830 REV	-1.598
EV126740	1.717	no similarity	
JCVI_14824	1.716	moderately similar to (395)AT3G11280| Symbols: | myb family transcription factor | chr3:3533483-3534399 REVERSE no original desc	-2.307
DY014824	1.716	weakly similar to (123)AT3G61150| Symbols: HDG1 | HDG1 (HOMEODOMAIN GLABROUS1); DNA binding / transcription factor | c	-1.667
JCVI_9898	1.715	moderately similar to (279)AT1G70830| Symbols: MLP28 | MLP28 (MLP-LIKE PROTEIN 28) | chr1:26713865-26715057 REVERSE	
JCVI_35822	1.715	weakly similar to (170)AT2G25180| Symbols: ARR12 | ARR12 (ARABIDOPSIS RESPONSE REGULATOR 12); transcription factor/ t	
JCVI_3764	1.715	no original description	
JCVI_15353	1.714	moderately similar to (338)AT3G02230| Symbols: ATRGP1, ATRGP, RGP1 | RGP1 (REVERSIBLY GLYCOSYLATED POLYPEPTI	-2.025
JCVI_36329	1.714	weakly similar to (169)AT2G25930| Symbols: PYK20, ELF3 | ELF3 (EARLY FLOWERING 3) | chr2:11066537-11070256 FORWARD	
JCVI_41376	1.713	moderately similar to (366)AT1G07390| Symbols: | protein binding | chr1:2269891-2274651 FORWARDweakly similar to (109)PSKR_	
CD833051	1.712	weakly similar to (200)AT5G11790| Symbols: | Ndr family protein | chr5:3799683-3802497 FORWARDweakly similar to (130)SF21_I	
JCVI_35461	1.712	moderately similar to (364)AT5G05870| Symbols: UGT76C1 | UGT76C1 (UDP-GLUCOSYL TRANSFERASE 76C1); UDP-glycosyltra	
EV069575	1.710	no similarity	-1.613

JCVI_15867	1.707	weakly similar to (130)AT2G38360| Symbols: | prenylated rab acceptor (PRA1) family protein | chr2:16076918-16077580 REVERSE n	
RC_ES968958	1.706	no similarity	
EE556817	1.706	no similarity	
JCVI_1450	1.706	highly similar to (770)AT3G14310| Symbols: ATPME3 | ATPME3 (Arabidopsis thaliana pectin methylesterase 3) | chr3:4772221-47751	
EE441797	1.704	moderately similar to (206)AT1G21740| Symbols: | similar to unknown protein [Arabidopsis thaliana] (TAIR:AT1G77500.1); similar to	-1.280
JCVI_7772	1.703	moderately similar to (294)AT5G64740| Symbols: CESA6, IXR2, E112, PRC1 | CESA6 (CELLULOSE SYNTHASE 6); transferase, tra	
JCVI_1834	1.702	very weakly similar to (98.2)AT2G46550| Symbols: | similar to unknown protein [Arabidopsis thaliana] (TAIR:AT1G01240.3); similar to	-1.447
ES906980	1.702	moderately similar to (481)AT1G31070| Symbols: | UDP-N-acetylglucosamine pyrophosphorylase-related | chr1:11084932-11088342 F	
EV120117	1.702	weakly similar to (179)AT5G17330| Symbols: GAD1, GAD | GAD (Glutamate decarboxylase 1); calmodulin binding | chr5:5711143-571	-1.989
EV165517	1.702	no similarity	
ES968089	1.701	no similarity	-1.471
JCVI_4446	1.701	very weakly similar to (96.7)AT4G36900| Symbols: RAP2.10 | RAP2.10 (related to AP2 10); DNA binding / transcription factor | chr4:17	
EE567753	1.701	no similarity	
EV165259	1.700	weakly similar to (143)AT4G36040| Symbols: | DNAJ heat shock N-terminal domain-containing protein (J11) | chr4:17049711-1705019	
EV185605	1.698	moderately similar to (270)AT5G08630| Symbols: | DDT domain-containing protein | chr5:2798576-2802139 REVERSE [21488]	-1.563
EV043098	1.697	no similarity	-1.829
EV109814	1.696	weakly similar to (130)AT3G20770| Symbols: EIN3 | EIN3 (ETHYLENE-INSENSITIVE3); transcription factor | chr3:7260708-726259	
JCVI_11224	1.696	highly similar to (653)AT1G74210| Symbols: | glycerophosphoryl diester phosphodiesterase family protein | chr1:27914057-27916446 F	-1.471
EV127372	1.695	no similarity	
EX096865	1.694	weakly similar to (172)AT5G13460| Symbols: IQD11 | IQD11 (IQ-domain 11); calmodulin binding | chr5:4316326-4318250 FORWARD	
EV123291	1.693	moderately similar to (279)AT4G00440| Symbols: | similar to unknown protein [Arabidopsis thaliana] (TAIR:AT2G45900.1); similar to	
JCVI_28162	1.693	moderately similar to (261)AT1G53035| Symbols: | similar to unknown protein [Arabidopsis thaliana] (TAIR:AT3G15358.1); similar to	
JCVI_24157	1.692	no original description	
EX023787	1.692	no similarity	
JCVI_25544	1.689	highly similar to (574)AT4G00440| Symbols: | similar to unknown protein [Arabidopsis thaliana] (TAIR:AT2G45900.1); similar to unna	
JCVI_29277	1.689	moderately similar to (246)AT5G58900| Symbols: | myb family transcription factor | chr5:23800501-23801893 REVERSE no original d	
EV184037	1.689	no similarity	-1.959
JCVI_36721	1.687	moderately similar to (285)AT1G78090| Symbols: ATTPPB | ATTPPB (TREHALOSE-6-PHOSPHATE PHOSPHATASE) | chr1:29378	
BG544700	1.685	no similarity	
EE563185	1.685	no similarity	
JCVI_26632	1.685	moderately similar to (269)AT1G72470| Symbols: ATEXO70D1 | ATEXO70D1 (exocyst subunit EXO70 family protein D1); protein bi	-1.409
JCVI_1652	1.684	no original description	
ES267678	1.684	weakly similar to (121)AT3G27040| Symbols: | meprin and TRAF homology domain-containing protein / MATH domain-containing pro	
EE545575	1.683	weakly similar to (123)AT5G65170| Symbols: | VQ motif-containing protein | chr5:26058444-26059532 FORWARD [20124] 15 524 6	
JCVI_23805	1.680	no original description	
EE528866	1.679	no similarity	
JCVI_24993	1.679	weakly similar to (154)AT3G21190| Symbols: | similar to unknown protein [Arabidopsis thaliana] (TAIR:AT1G51630.1); similar to unk	
JCVI_14315	1.678	moderately similar to (233)AT5G47920| Symbols: | similar to unknown protein [Arabidopsis thaliana] (TAIR:AT5G13880.1); similar to	
JCVI_16171	1.677	moderately similar to (408)AT4G14680| Symbols: APS3 | APS3 (ATP sulfurylase 2); sulfate adenylyltransferase (ATP) | chr4:8413438-8	
JCVI_25374	1.677	highly similar to (647)AT4G39090| Symbols: RD19A, RD19 | RD19 (RESPONSIVE TO DEHYDRATION 19); cysteine-type peptidase	-1.702
JCVI_29371	1.677	very weakly similar to (97.8)AT5G15470| Symbols: GAUT14 | GAUT14 (Galacturonosyltransferase 14); polygalacturonate 4-alpha-galac	-1.649
RC_ES969151	1.676	no similarity	
JCVI_28335	1.676	very weakly similar to (98.6)AT5G65660| Symbols: | hydroxyproline-rich glycoprotein family protein | chr5:26262670-26263471 REVE	
JCVI_27590	1.676	highly similar to (816)AT5G18500| Symbols: | protein kinase family protein | chr5:6139265-6141285 FORWARDmoderately similar to	
JCVI_3416	1.676	moderately similar to (238)AT5G13880| Symbols: | similar to unknown protein [Arabidopsis thaliana] (TAIR:AT5G47920.1); similar to	
JCVI_4431	1.674	highly similar to (610)AT2G43020| Symbols: ATPAO2 | ATPAO2 (POLYAMINE OXIDASE 2); amine oxidase | chr2:17899022-17901	-1.207
EE506233	1.674	weakly similar to (149)AT3G57980| Symbols: | DNA-binding bromodomain-containing protein | chr3:21477896-21480125 REVERSE [	-1.776
EX018778	1.673	weakly similar to (154)AT4G02390| Symbols: APP | APP (ARABIDOPSIS POLY(ADP-RIBOSE) POLYMERASE); NAD+ ADP-ribos	-1.526
EV171717	1.673	no similarity	
EV019887	1.672	moderately similar to (305)AT4G28890| Symbols: | protein binding / ubiquitin-protein ligase/ zinc ion binding | chr4:14256443-1425774	-2.165
JCVI_9231	1.672	weakly similar to (108)AT5G47550| Symbols: | cysteine protease inhibitor, putative / cystatin, putative | chr5:19303822-19304190 REV	
EG019871	1.672	weakly similar to (111)ATMG00310| Symbols: ORF154 | hypothetical protein | chrM:90883-91347 REVERSE [20440]	
JCVI_14161	1.671	moderately similar to (308)AT4G24080| Symbols: ALL1 | ALL1 (ALDOLASE LIKE); carbon-carbon lyase/ catalytic | chr4:12511394-12	-1.719
EX140431	1.671	no similarity	
EH424303	1.671	moderately similar to (218)AT4G08500| Symbols: ATMEKK1, MAPKKK8, MEKK1 | MEKK1 (MYTOGEN ACTIVATED PROTEIN	-2.085
RC_EE558426	1.671	no similarity	-2.027
EV073144	1.670	moderately similar to (280)AT1G21010| Symbols: | similar to unknown protein [Arabidopsis thaliana] (TAIR:AT1G76600.1); similar to	
JCVI_11792	1.669	moderately similar to (261)AT5G24580| Symbols: | copper-binding family protein | chr5:8410397-8412090 REVERSE no original descr	
JCVI_6790	1.669	highly similar to (685)AT3G59530| Symbols: | strictosidine synthase family protein | chr3:22004687-22006141 FORWARDweakly simi	
DW999835	1.669	no similarity	
JCVI_40903	1.668	weakly similar to (125)AT2G37678| Symbols: PAT3, FRY1, FHY1 | FHY1 (FAR-RED ELONGATED HYPOCOTYL 1) | chr2:1580870	
JCVI_39107	1.668	moderately similar to (480)AT3G03440| Symbols: | armadillo/beta-catenin repeat family protein | chr3:815716-818575 FORWARD no c	-1.466
EE568504	1.667	no similarity	
EV003770	1.667	no similarity	
EV173169	1.666	moderately similar to (281)AT3G28740| Symbols: CYP81D1 | cytochrome P450 family protein | chr3:10790001-10791789 REVERSEw	
JCVI_21189	1.666	very weakly similar to (82.8)AT3G02190| Symbols: | 60S ribosomal protein L39 (RPL39B) | chr3:406012-406349 REVERSE no origina	-2.240
ES947032	1.666	weakly similar to (144)AT1G21740| Symbols: | similar to unknown protein [Arabidopsis thaliana] (TAIR:AT1G77500.1); similar to hyp	-1.520
JCVI_32209	1.666	highly similar to (810)AT1G19440| Symbols: | very-long-chain fatty acid condensing enzyme, putative | chr1:6729110-6730660 FORWA	
JCVI_17171	1.665	no original description	
CD826046	1.665	weakly similar to (131)AT3G06550| Symbols: | similar to O-acetyltransferase family protein [Arabidopsis thaliana] (TAIR:AT2G34410.	
EX096464	1.664	no similarity	
JCVI_8556	1.660	weakly similar to (200)AT2G36400| Symbols: AtGRF3 | AtGRF3 (GROWTH-REGULATING FACTOR 3) | chr2:15277379-15279696	
JCVI_4297	1.660	weakly similar to (135)AT2G21660| Symbols: GR-RBP7, GRP7, CCR2, ATGRP7 | ATGRP7 (COLD, CIRCADIAN RHYTHM, AND F	
ES267205	1.659	weakly similar to (190)AT3G04630| Symbols: WDL1 | WDL1 (WVD2-LIKE 1) | chr3:1259237-1260658 FORWARD [21032]	
JCVI_21963	1.658	highly similar to (585)AT3G62110| Symbols: | glycoside hydrolase family 28 protein / polygalacturonase (pectinase) family protein | chr3	
EX025411	1.657	weakly similar to (190)AT1G59700| Symbols: ATGSTU16 | ATGSTU16 (Arabidopsis thaliana Glutathione S-transferase (class tau) 16);	
JCVI_6494	1.657	moderately similar to (338)AT1G53500| Symbols: RHM2, MUM4 | MUM4 (MUCILAGE-MODIFIED 4); catalytic | chr1:19970825-19	
JCVI_17728	1.656	moderately similar to (347)AT1G19940| Symbols: ATGH9B5 | ATGH9B5 (ARABIDOPSIS THALIANA GLYCOSYL HYDROLASE 9	
EV060903	1.656	no similarity	
AM394399	1.653	no similarity	-1.566

ES950693	1.652	no similarity	
JCVI_21496	1.651	highly similar to (862)AT4G34200| Symbols: EDA9 | EDA9 (embryo sac development arrest 9); NAD binding / amino acid binding / bin	
JCVI_818	1.651	moderately similar to (211)AT2G01570| Symbols: RGA, RGA1 | RGA1 (REPRESSOR OF GA1-3 1); transcription factor | chr2:255580	
EV072499	1.650	moderately similar to (399)AT1G19940| Symbols: ATGH9B5 | ATGH9B5 (ARABIDOPSIS THALIANA GLYCOSYL HYDROLASE 9	
JCVI_27052	1.650	moderately similar to (495)AT2G26980| Symbols: SnRK3.17, CIPK3 | CIPK3 (CBL-INTERACTING PROTEIN KINASE 3); kinase | cl	
JCVI_41510	1.650	moderately similar to (270)AT5G19980| Symbols: | integral membrane family protein | chr5:6749909-6750934 REVERSE no original d	-1.903
JCVI_22986	1.649	moderately similar to (339)AT3G02740| Symbols: | aspartyl protease family protein | chr3:590568-593096 FORWARD no original desc	
JCVI_20813	1.649	moderately similar to (436)AT1G56130| Symbols: | leucine-rich repeat family protein / protein kinase family protein | chr1:20998596-21	
JCVI_14667	1.649	moderately similar to (236)AT2G46370| Symbols: JAR, FIN219, JAR1 | JAR1 (JASMONATE RESISTANT 1) | chr2:19041652-190434	
JCVI_39527	1.648	moderately similar to (222)AT1G13880| Symbols: | ELM2 domain-containing protein | chr1:4749600-4750964 FORWARD no original	
EV178458	1.647	weakly similar to (159)AT4G36250| Symbols: ALDH3F1 | ALDH3F1 (ALDEHYDE DEHYDROGENASE 3F1); 3-chloroallyl aldehyde	
JCVI_31062	1.646	moderately similar to (461)AT3G58620| Symbols: TTL4 | TTL4 (TETRATRICOPETIDE-REPEAT THIOREDOXIN-LIKE 4); binding	
JCVI_22962	1.644	no original description	-2.431
EV092801	1.643	weakly similar to (108)AT1G66620| Symbols: | seven in absentia (SINA) protein, putative | chr1:24856469-24857707 REVERSE [2147	-1.497
EV048763	1.640	moderately similar to (249)AT5G58190| Symbols: ECT10 | ECT10 | chr5:23563660-23566589 FORWARD [21442]	-1.517
EV147055	1.639	no similarity	
JCVI_41433	1.639	moderately similar to (419)AT4G25110| Symbols: ATMC2 | ATMC2 (METACASPASE 2); caspase | chr4:12887748-12889963 REVEI	-2.105
JCVI_40291	1.638	moderately similar to (252)AT5G11850| Symbols: | protein kinase family protein | chr5:3816633-3821025 REVERSE no original descri	
EV133325	1.637	no similarity	
JCVI_1922	1.636	moderately similar to (496)AT1G20270| Symbols: | oxidoreductase, 2OG-Fe(II) oxygenase family protein | chr1:7021373-7022913 REV	-1.475
JCVI_37852	1.636	no original description	
ES992152	1.635	no similarity	
JCVI_16239	1.635	no original description	-2.351
JCVI_10883	1.634	moderately similar to (246)AT1G08510| Symbols: FATB | FATB (FATTY ACYL-ACP THIOESTERASES B); acyl carrier/ acyl-ACP tl	
EX130367	1.634	no similarity	
JCVI_39148	1.633	moderately similar to (280)AT5G57070| Symbols: | hydroxyproline-rich glycoprotein family protein | chr5:23113447-23115174 FORWA	
JCVI_15992	1.632	moderately similar to (370)AT1G08650| Symbols: PPCK1 | PPCK1 (PHOSPHOENOLPYRUVATE CARBOXYLASE KINASE); kinas	-2.010
JCVI_11386	1.632	no original description	
JCVI_5867	1.632	moderately similar to (487)AT1G04430| Symbols: | dehydration-responsive protein-related | chr1:1198859-1201300 FORWARD no orig	
JCVI_29957	1.630	highly similar to (777)AT1G66880| Symbols: | serine/threonine protein kinase family protein | chr1:24950591-24959101 FORWARDmo	-2.373
ES969277	1.628	no similarity	-2.159
JCVI_21143	1.627	moderately similar to (385)AT2G45590| Symbols: | protein kinase family protein | chr2:18793799-18795850 FORWARDweakly similar	
JCVI_22475	1.626	moderately similar to (203)AT5G59960| Symbols: | similar to unnamed protein product [Vitis vinifera] (GB:CAO21698.1) | chr5:24159	-1.613
JCVI_25357	1.624	very weakly similar to (97.8)AT4G27657| Symbols: | similar to unknown protein [Arabidopsis thaliana] (TAIR:AT4G27652.1) | chr4:138	
JCVI_12810	1.624	moderately similar to (454)AT5G11960| Symbols: | similar to hypothetical protein [Vitis vinifera] (GB:CAN81798.1); contains InterPro	-1.099
JCVI_11133	1.624	moderately similar to (259)AT1G76980| Symbols: | similar to EMB2170 (EMBRYO DEFECTIVE 2170) [Arabidopsis thaliana] (TAIR	-2.094
JCVI_38316	1.623	very weakly similar to (93.2)AT1G12110| Symbols: CHL1-1, NRT1, B-1, ATNRT1, CHL1, NRT1.1 | NRT1.1 (NITRATE TRANSPORT	
JCVI_21679	1.621	very weakly similar to (92.8)AT2G30870| Symbols: ERD13, ATGSTF4, ATGSTF10 | ATGSTF10 (EARLY DEHYDRATION-INDUCE	-1.914
JCVI_1447	1.620	highly similar to (533)AT3G08030| Symbols: | similar to unknown protein [Arabidopsis thaliana] (TAIR:AT2G41800.1); similar to unna	
JCVI_28413	1.619	weakly similar to (115)AT3G24255| Symbols: | similar to unknown protein [Arabidopsis thaliana] (TAIR:AT3G23910.1); similar to unn	-1.846
CD821313	1.616	weakly similar to (103)AT1G11270| Symbols: | F-box family protein | chr1:3785833-3786653 REVERSE [13979]	
EV216595	1.616	moderately similar to (322)AT3G56060| Symbols: | glucose-methanol-choline (GMC) oxidoreductase family protein | chr3:20814307-20	
EE519322	1.616	no similarity	-1.933
EE489734	1.615	weakly similar to (172)AT5G55400| Symbols: | fimbrin-like protein, putative | chr5:22472761-22476176 REVERSE [15724]	
JCVI_40283	1.614	moderately similar to (424)AT1G14670| Symbols: | endomembrane protein 70, putative | chr1:5037664-5040194 FORWARD no origina	-1.418
JCVI_5101	1.612	weakly similar to (116)AT1G17340| Symbols: | phosphoinositide phosphatase family protein | chr1:5934122-5938384 FORWARD no o	-1.874
EV087433	1.612	no similarity	
JCVI_13481	1.612	moderately similar to (296)AT5G66280| Symbols: GMD1 | GMD1 (GDP-D-MANNOSE 4,6-DEHYDRATASE 1); GDP-mannose 4,6-d	-1.820
JCVI_35447	1.610	moderately similar to (364)AT5G25070| Symbols: | similar to unnamed protein product [Vitis vinifera] (GB:CAO66326.1) | chr5:86412	-1.551
JCVI_32561	1.610	highly similar to (571)AT3G50340| Symbols: | similar to unknown protein [Arabidopsis thaliana] (TAIR:AT5G67020.1); similar to unkn	
JCVI_19791	1.610	moderately similar to (484)AT1G29750| Symbols: RKF1 | RKF1 (RECEPTOR-LIKE KINASE IN FLOWERS 1); ATP binding / protein	
DY024116	1.610	weakly similar to (102)AT4G37740| Symbols: AtGRF2 | AtGRF2 (GROWTHREGULATING FACTOR 2) | chr4:17725527-17727603 F	
JCVI_2631	1.609	moderately similar to (202)AT5G54530| Symbols: | similar to unknown protein [Arabidopsis thaliana] (TAIR:AT1G61667.1); similar to	
JCVI_31887	1.608	moderately similar to (215)AT5G59220| Symbols: | protein phosphatase 2C, putative / PP2C, putative | chr5:23911898-23913723 REVE	
JCVI_5433	1.608	moderately similar to (244)AT1G01140| Symbols: PKS6, SnRK3.12, CIPK9 | CIPK9 (CBL-INTERACTING PROTEIN KINASE 9); kir	
EE555870	1.608	very weakly similar to (94.7)AT5G61190| Symbols: | zinc finger protein-related | chr5:24632706-24637112 FORWARD [20184] 1 413 4	
DY006582	1.608	weakly similar to (123)AT2G34925| Symbols: CLE42 | CLE42 (CLAVATA3/ESR-RELATED 42) | chr2:14741349-14741615 FORWA	-2.670
EV086876	1.607	no similarity	-1.627
JCVI_18366	1.607	moderately similar to (482)AT1G67850| Symbols: | similar to unknown protein [Arabidopsis thaliana] (TAIR:AT1G13000.2); similar to	-2.093
JCVI_4938	1.605	highly similar to (703)AT3G52850| Symbols: VSR1, BP-80, ATELP, VSR-1, BP80, BP80B, ATELP1 | ATELP/ATELP1/BP-80/BP80/I	
JCVI_22121	1.605	weakly similar to (137)AT2G36880| Symbols: MAT3 | MAT3 (METHIONINE ADENOSYLTRANSFERASE 3) | chr2:15486800-1548	
JCVI_13436	1.604	weakly similar to (168)AT1G27360| Symbols: | squamosa promoter-binding protein-like 11 (SPL11) | chr1:9502126-9503702 FORWAI	
JCVI_488	1.603	moderately similar to (426)AT3G17390| Symbols: SAMS3, MAT4, MTO3 | MTO3 (S-adenosylmethionine synthase 3); methionine ader	
ES903284	1.602	weakly similar to (114)AT3G57230| Symbols: AGL16 | AGL16 (AGAMOUS-LIKE 16) | chr3:21188689-21191650 FORWARDweakly	-1.456
JCVI_998	1.602	no original description	
JCVI_19846	1.600	moderately similar to (395)AT3G01400| Symbols: | armadillo/beta-catenin repeat family protein | chr3:151927-152994 FORWARDweal	
JCVI_16672	1.600	moderately similar to (441)AT5G10830| Symbols: | embryo-abundant protein-related | chr5:3423732-3424650 FORWARD no original c	
EE523769	1.599	moderately similar to (265)AT5G61780| Symbols: | tudor domain-containing protein / nuclease family protein | chr5:24839238-2484386	-1.693
EE420526	1.597	moderately similar to (328)AT5G13750| Symbols: ZIFL1 | ZIFL1 (ZINC INDUCED FACILITATOR-LIKE 1); tetracycline:hydrogen an	
JCVI_29163	1.595	moderately similar to (289)AT5G46760| Symbols: | basic helix-loop-helix (bHLH) family protein | chr5:18991458-18993236 FORWAR	
JCVI_12401	1.595	moderately similar to (372)AT2G01570| Symbols: RGA, RGA1 | RGA1 (REPRESSOR OF GA1-3 1); transcription factor | chr2:255580	
ES965817	1.593	very weakly similar to (84.0)AT3G06500| Symbols: | beta-fructofuranosidase, putative / invertase, putative / saccharase, putative / beta-fr	
ES961043	1.589	weakly similar to (140)AT1G19440| Symbols: | very-long-chain fatty acid condensing enzyme, putative | chr1:6729110-6730660 FORW	
EX110103	1.589	weakly similar to (198)AT5G22570| Symbols: ATWRKY38, WRKY38 | WRKY38 (WRKY DNA-binding protein 38); transcription fact	-2.150
JCVI_28091	1.588	moderately similar to (275)AT3G15970| Symbols: | Ran-binding protein 1 domain-containing protein / RanBP1 domain-containing prote	
AM386090	1.588	no similarity	-1.512
JCVI_36726	1.587	moderately similar to (254)AT1G73740| Symbols: | glycosyl transferase family 28 protein | chr1:27738112-27739669 FORWARD no or	-1.731
JCVI_28409	1.587	moderately similar to (218)AT1G01260| Symbols: | basic helix-loop-helix (bHLH) family protein | chr1:109595-111367 FORWARD no	
DY027627	1.586	no similarity	
JCVI_2809	1.586	moderately similar to (456)AT4G22530| Symbols: | embryo-abundant protein-related | chr4:11859258-11860140 REVERSE no original	

AM390427	1.586	moderately similar to (283)AT2G17120| Symbols: LYM2 | LYM2 (LYSM DOMAIN GPI-ANCHORED PROTEIN 2 PRECURSOR) | c	-1.721
JCVI_37967	1.584	no original description	
EX057876	1.583	weakly similar to (124)AT1G29470| Symbols: | dehydration-responsive protein-related | chr1:10310410-10313355 REVERSE [21813]	
JCVI_3901	1.582	highly similar to (650)AT4G32410| Symbols: CESA1, RSW1 | CESA1 (CELLULOSE SYNTHASE 1); transferase, transferring glycosyl	
EV111184	1.582	no similarity	
JCVI_31441	1.580	moderately similar to (322)AT5G05090| Symbols: | myb family transcription factor | chr5:1503394-1504194 FORWARD no original de	-1.325
EG021105	1.580	no similarity	
JCVI_10940	1.578	moderately similar to (354)AT5G26850| Symbols: | binding | chr5:9445953-9450587 FORWARD no original description	-1.591
EV124029	1.578	moderately similar to (369)AT4G21390| Symbols: B120 | B120; protein kinase/ sugar binding | chr4:11394469-11397485 REVERSEwe	
JCVI_39639	1.578	moderately similar to (408)AT5G04885| Symbols: | glycosyl hydrolase family 3 protein | chr5:1423370-1426629 FORWARD no origina	
JCVI_37777	1.577	moderately similar to (347)AT1G64200| Symbols: VHA-E3 | VHA-E3 (VACUOLAR H+-ATPASE SUBUNIT E ISOFORM 3); hydrog	
JCVI_17761	1.576	moderately similar to (273)AT2G46250| Symbols: | myosin heavy chain-related | chr2:18998459-19000274 FORWARD no original desc	-1.711
JCVI_29646	1.576	highly similar to (544)AT1G14040| Symbols: | ATP binding / ATPase, coupled to transmembrane movement of substances | chr1:481048	
JCVI_10069	1.575	moderately similar to (315)AT5G19980| Symbols: | integral membrane family protein | chr5:6749909-6750934 REVERSE no original de	-1.797
EE448341	1.575	no similarity	-1.355
JCVI_16812	1.574	very weakly similar to (85.1)AT1G51355| Symbols: | similar to hypothetical protein [Vitis vinifera] (GB:CAN73276.1); similar to unnam	
ES969151	1.574	no similarity	
JCVI_28212	1.574	weakly similar to (140)AT2G37630| Symbols: ATPHAN, AS1, ATMYB91, MYB91 | AS1/ATMYB91/ATPHAN/MYB91 (ASYMMET	
JCVI_9503	1.573	weakly similar to (105)AT1G74840| Symbols: | myb family transcription factor | chr1:28119862-28120978 REVERSE no original descr	
JCVI_13386	1.572	moderately similar to (233)AT5G62360| Symbols: | invertase/pectin methylesterase inhibitor family protein | chr5:25057925-25058536 I	
AM386107	1.572	moderately similar to (201)AT4G13420| Symbols: HAK5 | HAK5 (High affinity K+ transporter 5); potassium ion transmembrane transpo	
JCVI_4650	1.572	moderately similar to (459)AT4G31500| Symbols: SUR2, RNT1, RED1, ATR4, CYP83B1 | CYP83B1 (CYTOCHROME P450 MONO	-1.980
JCVI_5508	1.571	moderately similar to (411)AT4G18800| Symbols: AthSGBP, AtRab11B, AtRABA1d | AtRABA1d/AtRab11B/AthSGBP (Arabidopsis F	-1.438
JCVI_12677	1.571	moderately similar to (365)AT5G64700| Symbols: | nodulin MtN21 family protein | chr5:25882416-25884071 REVERSE no original de	
JCVI_18252	1.570	moderately similar to (260)AT5G07580| Symbols: | DNA binding / transcription factor | chr5:2399526-2400350 FORWARDweakly sim	
AM231597	1.566	weakly similar to (185)AT1G73880| Symbols: | UDP-glucoronosyl/UDP-glucosyl transferase family protein | chr1:27788804-27790225	
EV036657	1.566	weakly similar to (189)AT5G45750| Symbols: AtRABA1c | AtRABA1c (Arabidopsis Rab GTPase homolog A1c); GTP binding | chr5:18	
JCVI_28117	1.565	moderately similar to (258)AT1G60960| Symbols: IRT3 | IRT3 (Iron regulated transporter 3); cation transmembrane transporter/ metal ic	-1.391
EX133984	1.565	very weakly similar to (89.0)AT2G21620| Symbols: RD2 | RD2 (RESPONSIVE TO DESSICATION 2) | chr2:9255829-9257066 FORW	-1.626
EE444995	1.565	weakly similar to (104)AT4G35120| Symbols: | kelch repeat-containing F-box family protein | chr4:16716811-16718022 FORWARD [2	-1.461
JCVI_22346	1.564	moderately similar to (461)AT5G18520| Symbols: | similar to unknown protein [Arabidopsis thaliana] (TAIR:AT3G09570.1); similar to	-1.404
JCVI_798	1.564	moderately similar to (297)AT3G44840| Symbols: | S-adenosyl-L-methionine:carboxyl methyltransferase family protein | chr3:16383484	
JCVI_8838	1.563	moderately similar to (448)AT5G24530| Symbols: | oxidoreductase, 2OG-Fe(II) oxygenase family protein | chr5:8378967-8383157 FOR	-1.788
JCVI_23946	1.563	weakly similar to (105)AT3G05270| Symbols: | similar to myosin heavy chain-related [Arabidopsis thaliana] (TAIR:AT1G77580.2); sim	
JCVI_14322	1.563	moderately similar to (439)AT3G03990| Symbols: | esterase/lipase/thioesterase family protein | chr3:1033795-1034598 FORWARD no	
JCVI_37934	1.562	highly similar to (641)AT5G25100| Symbols: | endomembrane protein 70, putative | chr5:8648377-8651018 REVERSE no original desc	
EE450290	1.561	weakly similar to (196)AT3G02340| Symbols: | zinc finger (C3HC4-type RING finger) family protein | chr3:477039-478268 FORWARI	
JCVI_22729	1.560	weakly similar to (139)AT3G03440| Symbols: | armadillo/beta-catenin repeat family protein | chr3:815716-818575 FORWARD no origi	
JCVI_7805	1.559	moderately similar to (331)AT5G53160| Symbols: | similar to unknown protein [Arabidopsis thaliana] (TAIR:AT1G01360.1); similar to	
AM386396	1.559	moderately similar to (350)AT5G16230| Symbols: | acyl-(acyl-carrier-protein) desaturase, putative / stearoyl-ACP desaturase, putative | c	-1.643
JCVI_9082	1.559	moderately similar to (325)AT3G03870| Symbols: | similar to unknown protein [Arabidopsis thaliana] (TAIR:AT5G18130.2) | chr3:995	
AM395628	1.559	moderately similar to (242)AT1G66620| Symbols: | seven in absentia (SINA) protein, putative | chr1:24856469-24857707 REVERSE [2	-1.636
ES907461	1.559	moderately similar to (210)AT1G14710| Symbols: | hydroxyproline-rich glycoprotein family protein | chr1:5062163-5064692 REVERSI	
EX044124	1.559	moderately similar to (286)AT3G11040| Symbols: | glycosyl hydrolase family 85 protein | chr3:3460149-3463318 FORWARD [21811]	-2.199
JCVI_24035	1.558	moderately similar to (263)AT4G26690| Symbols: MRH5, SHV3 | MRH5/SHV3 (morphogenesis of root hair 5); glycerophosphodiester	-1.678
JCVI_18818	1.558	moderately similar to (330)AT4G37180| Symbols: | myb family transcription factor | chr4:17504642-17506101 FORWARD no original	
JCVI_35374	1.558	moderately similar to (477)AT2G31970| Symbols: ATRAD50, RAD50 | RAD50; ATP binding / nuclease/ zinc ion binding | chr2:136077	-1.901
JCVI_6436	1.557	nearly identical (1053)AT1G77140| Symbols: ATVPS45, VPS45 | VPS45 (VACUOLAR PROTEIN SORTING 45); protein transporter |	-1.179
JCVI_3250	1.557	highly similar to (682)AT2G16230| Symbols: | glycosyl hydrolase family 17 protein | chr2:7043103-7045408 REVERSEmoderately sim	-1.388
JCVI_20281	1.556	moderately similar to (223)AT1G13880| Symbols: | ELM2 domain-containing protein | chr1:4749600-4750964 FORWARD no original	
AM385662	1.556	no similarity	
JCVI_24691	1.554	no original description	
JCVI_4959	1.554	weakly similar to (123)AT4G33355| Symbols: | lipid binding | chr4:16067101-16067454 FORWARDvery weakly similar to (81.3)NLTF	
RC_EE550738	1.553	no similarity	-1.542
JCVI_31730	1.553	moderately similar to (285)AT3G23670| Symbols: PAKRP1L, KINESIN-12B | KINESIN-12B/PAKRP1L; microtubule motor | chr3:851	
JCVI_3230	1.552	weakly similar to (124)AT5G45500| Symbols: | similar to unknown protein [Arabidopsis thaliana] (TAIR:AT5G45520.1); similar to unn	-1.758
EE555957	1.551	no similarity	-1.334
JCVI_39734	1.551	moderately similar to (282)AT1G22410| Symbols: | 2-dehydro-3-deoxyphosphoheptonate aldolase, putative / 3-deoxy-D-arabino-heptulo	-2.309
JCVI_7077	1.551	highly similar to (586)AT3G02570| Symbols: MEE31 | MEE31 (maternal effect embryo arrest 31); mannose-6-phosphate isomerase | chr	
JCVI_34862	1.551	very weakly similar to (90.5)AT3G19680| Symbols: | similar to unknown protein [Arabidopsis thaliana] (TAIR:AT1G50040.1); similar to	
JCVI_41982	1.550	weakly similar to (139)AT1G75540| Symbols: STH2 | STH2 (SALT TOLERANCE HOMOLOG2); transcription factor/ zinc ion binding	
AM061937	1.550	no similarity	-1.683
JCVI_2802	1.549	moderately similar to (469)AT3G15450| Symbols: | similar to unknown protein [Arabidopsis thaliana] (TAIR:AT4G27450.1); similar to	
JCVI_38803	1.548	moderately similar to (265)AT3G58620| Symbols: TTL4 | TTL4 (TETRATRICOPETIDE-REPEAT THIOREDOXIN-LIKE 4); binding	
JCVI_5585	1.548	moderately similar to (423)AT2G40280| Symbols: | dehydration-responsive family protein | chr2:16832785-16835378 REVERSE no ori	
RC_H74760	1.546	no similarity	
JCVI_33936	1.546	highly similar to (511)AT2G28890| Symbols: PLL4 | PLL4 (POLTERGEIST LIKE 4); protein serine/threonine phosphatase | chr2:12412	-1.890
EE412700	1.545	weakly similar to (165)AT1G04350| Symbols: | 2-oxoglutarate-dependent dioxygenase, putative | chr1:1165295-1166537 FORWARDve	
JCVI_29816	1.545	moderately similar to (470)AT5G10610| Symbols: CYP81K1 | CYP81K1 (cytochrome P450, family 81, subfamily K, polypeptide 1); oxy	
JCVI_12890	1.544	no original description	
JCVI_14458	1.544	moderately similar to (477)AT1G71880| Symbols: ATSUC1, SUC1 | SUC1 (SUCROSE-PROTON SYMPORTER 1); carbohydrate tran	-2.422
JCVI_21140	1.543	moderately similar to (207)AT5G18310| Symbols: | similar to unknown protein [Arabidopsis thaliana] (TAIR:AT5G48500.1); similar to	
EE560243	1.543	no similarity	
JCVI_35358	1.542	no original description	
JCVI_25339	1.542	moderately similar to (266)AT3G52890| Symbols: KIPK | KIPK (KCBP-INTERACTING PROTEIN KINASE); kinase | chr3:19620128-	
JCVI_13273	1.540	moderately similar to (218)AT2G02860| Symbols: ATSUC3, SUC3, SUT2 | SUT2 (sucrose transporter 3); carbohydrate transmembrane	-1.582
EV085687	1.538	no similarity	-1.422
JCVI_3471	1.538	moderately similar to (417)AT2G47140| Symbols: | short-chain dehydrogenase/reductase (SDR) family protein | chr2:19358040-193591	
JCVI_18389	1.537	weakly similar to (124)AT3G29785| Symbols: | similar to hypothetical protein [Vitis vinifera] (GB:CAN67882.1) | chr3:11614836-1161	
EE564149	1.537	no similarity	

EE554421	1.535	no similarity	
JCVI_4880	1.533	moderately similar to (320)AT4G40060| Symbols: ATHB16, ATHB-16 | ATHB-16/ATHB16 (ARABIDOPSIS THALIANA HOMEOB	
EE563660	1.532	no similarity	-1.586
EX036771	1.532	moderately similar to (425)AT1G56130| Symbols: | leucine-rich repeat family protein / protein kinase family protein | chr1:20998596-21	
JCVI_4963	1.531	moderately similar to (347)AT2G27920| Symbols: SCPL51 | SCPL51; serine carboxypeptidase | chr2:11892854-11895809 REVERSE n	
JCVI_14530	1.531	weakly similar to (193)AT5G45500| Symbols: | similar to unknown protein [Arabidopsis thaliana] (TAIR:AT5G45520.1); similar to unn	-1.642
JCVI_5626	1.530	highly similar to (668)AT4G36920| Symbols: FLO2, FL1, AP2 | AP2 (APETALA 2); transcription factor | chr4:17400995-17403137 FO	
CD813940	1.529	moderately similar to (216)AT5G11250| Symbols: | disease resistance protein (TIR-NBS-LRR class), putative | chr5:3587979-3591961	
EX130657	1.529	moderately similar to (300)AT4G37730| Symbols: ATBZIP7 | ATBZIP7 (ARABIDOPSIS THALIANA BASIC LEUCINE-ZIPPER 7);	
EV008883	1.528	very weakly similar to (86.3)AT3G07810| Symbols: | heterogeneous nuclear ribonucleoprotein, putative / hnRNP, putative | chr3:249288	
JCVI_31035	1.527	weakly similar to (101)AT1G10950| Symbols: | endomembrane protein 70, putative | chr1:3659322-3663622 FORWARD no original de	
JCVI_33685	1.526	moderately similar to (372)AT5G62680| Symbols: | proton-dependent oligopeptide transport (POT) family protein | chr5:25182656-2518	
EV155694	1.524	moderately similar to (461)AT5G05170| Symbols: CESA3, IXR1, ATCESA3, ATH-B, CEV1 | CESA3 (CELLULOSE SYNTHASE 3);	
EV176066	1.523	very weakly similar to (99.4)AT1G02500| Symbols: SAM-1, MAT1, SAM1 | SAM1 (S-adenosylmethionine synthetase 1); methionine ad	-2.142
JCVI_7698	1.521	moderately similar to (208)AT4G31170| Symbols: | protein kinase family protein | chr4:15153505-15154852 REVERSE no original des	
JCVI_36594	1.519	moderately similar to (496)AT1G48280| Symbols: | hydroxyproline-rich glycoprotein family protein | chr1:17838864-17841221 FORWA	-1.487
JCVI_18354	1.519	moderately similar to (254)AT2G38940| Symbols: PHT1;4, ATPT2 | ATPT2 (PHOSPHATE TRANSPORTER 2); carbohydrate transme	-2.399
JCVI_27112	1.519	weakly similar to (112)AT1G21740| Symbols: | similar to unknown protein [Arabidopsis thaliana] (TAIR:AT1G77500.1); similar to hyp	-1.507
JCVI_12983	1.518	weakly similar to (107)AT1G45145| Symbols: ATH5, LIV1, ATTRX5 | ATTRX5 (thioredoxin H-type 5); thiol-disulfide exchange intern	-1.778
CX194992	1.518	moderately similar to (254)AT3G50340| Symbols: | similar to unknown protein [Arabidopsis thaliana] (TAIR:AT5G67020.1); similar to	
JCVI_31019	1.518	moderately similar to (261)AT5G25610| Symbols: RD22 | RD22 (RESPONSIVE TO DESSICATION 22) | chr5:8914501-8916687 REV	
JCVI_13219	1.517	moderately similar to (334)AT4G35830| Symbols: | aconitate hydratase, cytoplasmic / citrate hydro-lyase / aconitase (ACO) | chr4:16973	-3.024
JCVI_38713	1.515	highly similar to (504)AT3G62590| Symbols: | lipase class 3 family protein | chr3:23158949-23161145 REVERSE no original descriptic	
JCVI_6454	1.515	very weakly similar to (85.5)AT5G33393| Symbols: | unknown protein | chr5:12673660-12675818 REVERSE no original description	
EX019210	1.515	weakly similar to (184)AT2G44420| Symbols: | protein N-terminal asparagine amidohydrolase family protein | chr2:18337644-18339772	-1.374
DW999055	1.514	weakly similar to (182)AT1G11260| Symbols: STP1 | STP1 (SUGAR TRANSPORTER 1); carbohydrate transmembrane transporter/ sug	
JCVI_25550	1.514	moderately similar to (427)AT4G28150| Symbols: | similar to unknown protein [Arabidopsis thaliana] (TAIR:AT4G03420.1); similar to	-2.084
JCVI_32904	1.513	no original description	-1.896
JCVI_16363	1.513	moderately similar to (236)AT1G12200| Symbols: | flavin-containing monooxygenase family protein / FMO family protein | chr1:413762	-2.516
JCVI_3151	1.512	moderately similar to (402)AT3G08030| Symbols: | similar to unknown protein [Arabidopsis thaliana] (TAIR:AT2G41800.1); similar to	
JCVI_700	1.510	moderately similar to (222)AT3G16420| Symbols: PBP1 | PBP1 (PYK10-BINDING PROTEIN 1) | chr3:5579566-5580680 FORWARD	
ES966125	1.509	no similarity	
JCVI_5747	1.509	weakly similar to (110)AT4G35500| Symbols: | protein kinase family protein | chr4:16857480-16859412 FORWARD no original descrip	
JCVI_27137	1.508	moderately similar to (330)AT5G18520| Symbols: | similar to unknown protein [Arabidopsis thaliana] (TAIR:AT3G09570.1); similar to	-1.302
JCVI_17810	1.507	moderately similar to (216)AT2G35290| Symbols: | similar to unnamed protein product [Vitis vinifera] (GB:CAO63442.1) | chr2:14868	
JCVI_26344	1.506	moderately similar to (341)AT2G28470| Symbols: BGAL8 | BGAL8 (BETA-GALACTOSIDASE 8); beta-galactosidase | chr2:12176124	
JCVI_41853	1.505	no original description	
EE544205	1.505	weakly similar to (142)AT5G65810| Symbols: | similar to unknown protein [Arabidopsis thaliana] (TAIR:AT3G49720.1); similar to unk	
JCVI_37263	1.504	highly similar to (675)AT5G57580| Symbols: | calmodulin-binding protein | chr5:23332220-23334909 REVERSE no original descriptio	
JCVI_31800	1.503	weakly similar to (127)AT1G73250| Symbols: ATFX, GER1 | ATFX/GER1 (GDP-4-KETO-6-DEOXYMANNOSE-3,5-EPIMERASE-4	-1.938
ES903000	1.502	moderately similar to (471)AT4G30080| Symbols: ARF16 | ARF16 (AUXIN RESPONSE FACTOR 16); miRNA binding / transcription	
JCVI_13260	1.501	moderately similar to (459)AT2G45790| Symbols: ATPMM | ATPMM; phosphomannomutase | chr2:18862950-18864827 FORWARD	-1.579
JCVI_30075	1.498	very weakly similar to (92.4)AT1G01260| Symbols: | basic helix-loop-helix (bHLH) family protein | chr1:109595-111367 FORWARD no	
JCVI_1768	1.498	moderately similar to (280)AT3G17390| Symbols: SAMS3, MAT4, MTO3 | MTO3 (S-adenosylmethionine synthase 3); methionine ader	
JCVI_23881	1.498	highly similar to (504)AT5G39380| Symbols: | calmodulin-binding protein-related | chr5:15776391-15777914 REVERSE no original de	
RC_ES899586	1.498	no similarity	
JCVI_3321	1.498	moderately similar to (234)AT3G04670| Symbols: ATWRKY39, WRKY39 | WRKY39 (WRKY DNA-binding protein 39); transcription	-1.474
JCVI_3199	1.496	highly similar to (557)AT5G25980| Symbols: TGG2 | TGG2 (GLUCOSIDE GLUCOHYDROLASE 2); hydrolase, hydrolyzing O-glycos	
JCVI_19205	1.496	weakly similar to (164)AT1G49710| Symbols: ATFUT12, FUCTB, FUCT2, FUT12 | FUT12 (fucosyltransferase 12); fucosyltransferase/	-2.390
JCVI_16410	1.494	moderately similar to (256)AT3G06590| Symbols: | similar to transcription factor [Arabidopsis thaliana] (TAIR:AT3G17100.2); similar	
JCVI_40372	1.494	moderately similar to (214)AT3G54750| Symbols: | similar to unnamed protein product [Vitis vinifera] (GB:CAO48944.1); contains dor	-1.519
JCVI_22188	1.493	very weakly similar to (82.8)AT3G17820| Symbols: ATGSKB6 | ATGSKB6 (Arabidopsis thaliana glutamine synthase clone KB6); glutar	-1.619
JCVI_16310	1.492	no original description	
EX097213	1.491	moderately similar to (305)AT4G23470| Symbols: | hydroxyproline-rich glycoprotein family protein | chr4:12249299-12251089 FORWA	-2.154
EE557837	1.491	no similarity	
JCVI_11618	1.491	highly similar to (591)AT1G60890| Symbols: | phosphatidylinositol-4-phosphate 5-kinase family protein | chr1:22416117-22420003 RE	-1.399
EE559078	1.491	no similarity	-1.503
AI352900	1.491	weakly similar to (193)AT4G27870| Symbols: | integral membrane family protein | chr4:13878989-13882685 FORWARD [1285] 18 71	
EX040816	1.490	very weakly similar to (85.1)AT5G24580| Symbols: | copper-binding family protein | chr5:8410397-8412090 REVERSE [21811]	
JCVI_257	1.490	moderately similar to (341)AT4G01026| Symbols: | similar to unknown protein [Arabidopsis thaliana] (TAIR:AT1G01360.1); similar to	
CX272464	1.489	very weakly similar to (95.1)AT4G27500| Symbols: PPI1 | PPI1 (PROTON PUMP INTERACTOR 1) | chr4:13743620-13745906 FORW	
JCVI_10746	1.489	weakly similar to (126)AT4G16500| Symbols: | cysteine protease inhibitor family protein / cystatin family protein | chr4:9301553-93019	
JCVI_23915	1.488	weakly similar to (176)AT2G37630| Symbols: ATPHAN, AS1, ATMYB91, MYB91 | AS1/ATMYB91/ATPHAN/MYB91 (ASYMMET	
ES931524	1.488	no similarity	
JCVI_3084	1.487	moderately similar to (352)AT5G11670| Symbols: ATNADP-ME2 | ATNADP-ME2 (NADP-MALIC ENZYME 2); malate dehydrogena	
EE567964	1.487	no similarity	
JCVI_5796	1.485	moderately similar to (362)AT2G28830| Symbols: | armadillo/beta-catenin repeat family protein / U-box domain-containing protein | chr	-1.469
EH428695	1.482	moderately similar to (278)AT1G56130| Symbols: | leucine-rich repeat family protein / protein kinase family protein | chr1:20998596-21	
JCVI_33355	1.482	moderately similar to (397)AT1G22410| Symbols: | 2-dehydro-3-deoxyphosphoheptonate aldolase, putative / 3-deoxy-D-arabino-heptulo	
JCVI_18718	1.481	moderately similar to (416)AT2G43080| Symbols: AT-P4H-1 | AT-P4H-1 (A. THALIANA P4H ISOFORM 1); oxidoreductase, acting o	-1.346
JCVI_20898	1.481	very weakly similar to (95.5)AT3G53800| Symbols: | armadillo/beta-catenin repeat family protein | chr3:19941920-19943697 FORWAR	
EE436640	1.481	no similarity	
JCVI_21131	1.480	highly similar to (593)AT3G55640| Symbols: | mitochondrial substrate carrier family protein | chr3:20651027-20653390 FORWARDwe	-1.236
EV160117	1.479	moderately similar to (218)AT3G62590| Symbols: | lipase class 3 family protein | chr3:23158949-23161145 REVERSE [21484]	
JCVI_31672	1.475	no original description	
ES910271	1.474	moderately similar to (377)AT3G02570| Symbols: MEE31 | MEE31 (maternal effect embryo arrest 31); mannose-6-phosphate isomerase	
EV027964	1.474	weakly similar to (200)AT5G45750| Symbols: AtRABA1c | AtRABA1c (Arabidopsis Rab GTPase homolog A1c); GTP binding | chr5:18	
EV115177	1.474	moderately similar to (213)AT3G11570| Symbols: | similar to unknown protein [Arabidopsis thaliana] (TAIR:AT5G06230.1); similar to	
JCVI_24065	1.474	moderately similar to (447)AT5G03330| Symbols: | OTU-like cysteine protease family protein | chr5:807727-809607 FORWARD no or	-1.252
DY029033	1.474	weakly similar to (129)AT2G34670| Symbols: | proline-rich family protein | chr2:14620318-14622310 REVERSE [18978]	

EE476722	1.473	no similarity	
CV547080	1.473	weakly similar to (178)AT5G67360| Symbols: ARA12 | ARA12; subtilase | chr5:26889418-26891691 REVERSE [16551]	-2.130
EV226569	1.472	no similarity	
JCVI_24505	1.471	moderately similar to (355)AT5G26667| Symbols: | uridylate kinase / uridine monophosphate kinase / UMP kinase (PYR6) | chr5:92766	
JCVI_7544	1.471	moderately similar to (397)AT5G10840| Symbols: | endomembrane protein 70, putative | chr5:3424911-3427798 REVERSE no original	
JCVI_36974	1.469	moderately similar to (271)AT4G02370| Symbols: | similar to unknown protein [Arabidopsis thaliana] (TAIR:AT1G02816.1); similar to	
EV058678	1.467	no similarity	-1.475
JCVI_40539	1.467	moderately similar to (400)AT5G16300| Symbols: | similar to hypothetical protein OsI_003871 [Oryza sativa (indica cultivar-group)] (G	
EV024340	1.467	weakly similar to (157)AT4G16790| Symbols: | hydroxyproline-rich glycoprotein family protein | chr4:9451766-9453187 REVERSE [2	
JCVI_5654	1.467	highly similar to (632)AT5G03610| Symbols: | GDSL-motif lipase/hydrolase family protein | chr5:915649-918325 FORWARDweakly si	
JCVI_14195	1.467	moderately similar to (235)AT4G26690| Symbols: MRH5, SHV3 | MRH5/SHV3 (morphogenesis of root hair 5); glycerophosphodiester	-1.516
JCVI_4499	1.466	moderately similar to (369)AT4G35630| Symbols: PSAT | PSAT (phosphoserine aminotransferase); phosphoserine transaminase | chr4:1	-1.539
EE561850	1.462	no similarity	
JCVI_40636	1.462	moderately similar to (285)AT4G18170| Symbols: ATWRKY28, WRKY28 | WRKY28 (WRKY DNA-binding protein 28); transcription	-1.416
EV112173	1.462	very weakly similar to (98.6)AT1G13260| Symbols: RAV1 | RAV1 (Related to ABI3/VP1 1); DNA binding / transcription factor | chr1:4	
JCVI_9741	1.461	highly similar to (714)AT2G43790| Symbols: MPK6, MAPK6, ATMPK6 | ATMPK6 (MAP KINASE 6); MAP kinase/ kinase | chr2:181	
JCVI_1371	1.461	weakly similar to (150)AT1G11210| Symbols: | similar to unknown protein [Arabidopsis thaliana] (TAIR:AT1G11220.1); similar to cot	
DY026020	1.459	very weakly similar to (94.7)AT3G61960| Symbols: | protein kinase family protein | chr3:22952941-22955971 REVERSE [18978]	
JCVI_36650	1.459	highly similar to (695)AT1G07720| Symbols: | beta-ketoacyl-CoA synthase family protein | chr1:2390967-2392403 REVERSE no origi	-1.514
JCVI_7919	1.458	moderately similar to (236)AT3G05120| Symbols: ATGID1A, GID1A | ATGID1A/GID1A (GA INSENSITIVE DWARF1A); hydrolase	
EX113549	1.458	moderately similar to (226)AT2G35910| Symbols: | zinc finger (C3HC4-type RING finger) family protein | chr2:15080304-15080957 R	-1.525
EV112621	1.457	very weakly similar to (98.6)AT1G13260| Symbols: RAV1 | RAV1 (Related to ABI3/VP1 1); DNA binding / transcription factor | chr1:4	
JCVI_38509	1.456	moderately similar to (328)AT3G14590| Symbols: NTMC2TYPE6.2, NTMC2T6.2 | NTMC2T6.2/NTMC2TYPE6.2 | chr3:4904455-49	
JCVI_7251	1.455	highly similar to (523)AT2G46370| Symbols: JAR, FIN219, JAR1 | JAR1 (JASMONATE RESISTANT 1) | chr2:19041652-19043442 F	
AM390711	1.454	moderately similar to (260)AT1G09970| Symbols: | leucine-rich repeat transmembrane protein kinase, putative | chr1:3252410-3255430	
EV145507	1.452	no similarity	
JCVI_6551	1.452	moderately similar to (399)AT5G05870| Symbols: UGT76C1 | UGT76C1 (UDP-GLUCOSYL TRANSFERASE 76C1); UDP-glycosyltr	
JCVI_32523	1.451	highly similar to (507)AT3G51160| Symbols: MUR_1, GMD2, MUR1 | MUR1 (MURUS 1) | chr3:19018211-19019332 REVERSE no	
JCVI_24890	1.449	moderately similar to (226)AT4G36990| Symbols: HSFB1, AT-HSFB1, ATHSF4, HSF4 | HSF4 (HEAT SHOCK FACTOR 4); DNA bi	-1.659
EV136303	1.449	moderately similar to (234)AT1G69780| Symbols: ATHB13 | ATHB13; DNA binding / transcription factor | chr1:26262829-26264128 F	-2.100
EE474190	1.448	weakly similar to (197)AT5G16280| Symbols: | binding | chr5:5323380-5331348 REVERSE [20134] 1 314 356	-1.304
JCVI_28274	1.448	weakly similar to (179)AT1G10040| Symbols: | similar to unknown protein [Arabidopsis thaliana] (TAIR:AT4G25770.1); similar to unn	
JCVI_1421	1.445	moderately similar to (412)AT3G23820| Symbols: GAE6 | GAE6 (UDP-D-GLUCURONATE 4-EPIMERASE 6); catalytic | chr3:86036	
JCVI_24862	1.444	highly similar to (583)AT5G48930| Symbols: HCT | transferase family protein | chr5:19853880-19855318 REVERSEmoderately similar	
JCVI_1137	1.443	moderately similar to (452)AT1G08450| Symbols: CRT3 | CRT3 (CALRETICULIN 3); calcium ion binding | chr1:2668005-2671797 R	
EE568976	1.442	no similarity	
JCVI_11966	1.442	highly similar to (664)AT3G18830| Symbols: ATPLT5 | ATPLT5 (POLYOL TRANSPORTER 5); D-ribose transmembrane transporter/	-2.013
JCVI_14306	1.442	moderately similar to (276)AT5G63490| Symbols: | CBS domain-containing protein / octicosapeptide/Phox/Bemp1 (PB1) domain-conta	
JCVI_6082	1.441	moderately similar to (278)AT1G01470| Symbols: LSR3, LEA14 | LEA14 (LATE EMBRYOGENESIS ABUNDANT 14) | chr1:172295	
JCVI_4155	1.439	moderately similar to (249)AT4G34950| Symbols: | nodulin family protein | chr4:16642549-16644764 REVERSE no original descriptio	
JCVI_36674	1.439	no original description	
JCVI_42161	1.438	highly similar to (751)AT3G61580| Symbols: | delta-8 sphingolipid desaturase (SLD1) | chr3:22797228-22798577 FORWARD no origi	
EG020785	1.438	weakly similar to (152)AT3G13130| Symbols: | similar to hypothetical protein [Vitis vinifera] (GB:CAN76025.1) | chr3:4223015-42236	-1.006
CN729991	1.438	weakly similar to (199)AT5G25070| Symbols: | similar to unnamed protein product [Vitis vinifera] (GB:CAO66326.1) | chr5:8641264-8	
EE567941	1.437	no similarity	-1.356
EE535335	1.436	no similarity	-1.616
JCVI_37503	1.435	moderately similar to (384)AT3G53800| Symbols: | armadillo/beta-catenin repeat family protein | chr3:19941920-19943697 FORWARD	
JCVI_11734	1.433	highly similar to (602)AT5G60360| Symbols: AALP | AALP (ARABIDOPSIS ALEURAIN-LIKE PROTEASE) | chr5:24297270-24299	-2.365
AM395087	1.432	no similarity	
EE474678	1.430	weakly similar to (195)AT2G40650| Symbols: | pre-mRNA splicing factor PRP38 family protein | chr2:16970666-16972674 REVERSE	-1.267
JCVI_34635	1.429	highly similar to (597)AT3G22400| Symbols: LOX5 | LOX5; lipoxygenase | chr3:7927018-7931174 FORWARDmoderately similar to (	
EE563777	1.429	weakly similar to (194)AT1G14360| Symbols: ATUTR3, UTR3 | ATUTR3/UTR3 (UDP-GALACTOSE TRANSPORTER 3); pyrimidin	-1.680
EV110650	1.428	no similarity	-2.005
JCVI_2074	1.427	moderately similar to (320)AT1G62740| Symbols: | stress-inducible protein, putative | chr1:23234691-23237045 FORWARDweakly sim	
AM395366	1.426	no similarity	-1.416
JCVI_26121	1.426	moderately similar to (361)AT4G02030| Symbols: | similar to unknown protein [Arabidopsis thaliana] (TAIR:AT1G21170.1); similar to	
JCVI_11676	1.426	moderately similar to (298)AT5G56030| Symbols: ERD8, HSP81-2 | HSP81-2 (EARLY-RESPONSIVE TO DEHYDRATION 8); ATP	
JCVI_1867	1.425	moderately similar to (429)AT1G22410| Symbols: | 2-dehydro-3-deoxyphosphoheptonate aldolase, putative / 3-deoxy-D-arabino-heptulo	
EV165827	1.425	no similarity	
JCVI_29886	1.424	weakly similar to (172)AT1G15880| Symbols: ATGOS11, GOS11 | GOS11 (GOLGI SNARE 11); SNARE binding | chr1:5458712-5460	
JCVI_2510	1.424	moderately similar to (376)AT5G45370| Symbols: | nodulin-related / integral membrane family protein | chr5:18405638-18407509 FOR	
JCVI_30714	1.423	no original description	
JCVI_25413	1.422	weakly similar to (199)AT2G43020| Symbols: ATPAO2 | ATPAO2 (POLYAMINE OXIDASE 2); amine oxidase | chr2:17899022-1790	
CD827824	1.422	weakly similar to (121)AT2G22720| Symbols: | similar to unknown protein [Arabidopsis thaliana] (TAIR:AT4G37860.1); similar to unn	
JCVI_1866	1.421	weakly similar to (169)AT5G09870| Symbols: CESA5 | CESA5 (CELLULOSE SYNTHASE 5); transferase, transferring glycosyl groups	
JCVI_23064	1.421	no original description	
EV002627	1.419	moderately similar to (282)AT4G27760| Symbols: FEY3, FEY | FEY (FOREVER YOUNG); oxidoreductase | chr4:13844157-1384656	-1.830
JCVI_35577	1.419	no original description	-1.746
JCVI_10589	1.418	moderately similar to (301)AT1G19310| Symbols: | zinc finger (C3HC4-type RING finger) family protein | chr1:6676415-6677095 REV	-1.310
JCVI_12952	1.417	moderately similar to (258)AT2G36490| Symbols: DML1, ROS1 | DML1/ROS1 (REPRESSOR OF SILENCING1); DNA N-glycosylase	
EX021109	1.415	weakly similar to (196)AT2G35620| Symbols: | leucine-rich repeat transmembrane protein kinase, putative | chr2:14968266-14971719 R	
EV151519	1.413	moderately similar to (327)AT2G16660| Symbols: | nodulin family protein | chr2:7226012-7228674 REVERSE [21483]	
JCVI_5437	1.413	moderately similar to (337)AT5G27430| Symbols: | signal peptidase subunit family protein | chr5:9687473-9689188 FORWARDweakly	-1.408
EV221239	1.411	no similarity	
JCVI_37516	1.410	highly similar to (613)AT5G55400| Symbols: | fimbrin-like protein, putative | chr5:22472761-22476176 REVERSE no original descripti	
JCVI_29562	1.410	highly similar to (763)AT4G02390| Symbols: APP | APP (ARABIDOPSIS POLY(ADP-RIBOSE) POLYMERASE); NAD+ ADP-ribosy	
ES991052	1.408	weakly similar to (120)AT5G54440| Symbols: | binding | chr5:22120813-22124921 FORWARD [21425]	-1.421
JCVI_31676	1.408	weakly similar to (114)AT1G68500| Symbols: | similar to hypothetical protein [Vitis vinifera] (GB:CAN66643.1) | chr1:25705704-2570	
JCVI_9424	1.403	moderately similar to (421)AT3G25140| Symbols: GAUT8, QUA1 | GAUT8/QUA1 (GALACTURONOSYLTRANSFERASE 8, QUAS	
CD818482	1.403	weakly similar to (108)AT2G30360| Symbols: SIP4, SNRK3.22, PKS5, CIPK11 | CIPK11 (SOS3-INTERACTING PROTEIN 4); kinase	-1.245

EV218513	1.402	moderately similar to (394)AT1G76850| Symbols: SEC5A | SEC5A (EXOCYST COMPLEX COMPONENT SEC5) | chr1:28852907-28	-1.602
JCVI_32965	1.402	moderately similar to (394)AT5G57035| Symbols: | kinase | chr5:23097969-23101045 FORWARD no original description	
JCVI_27754	1.400	no original description	
JCVI_17820	1.400	moderately similar to (500)AT4G00350| Symbols: | MATE efflux family protein | chr4:151978-153988 FORWARD no original descript	-1.406
EV012438	1.399	no similarity	-1.544
EV123231	1.398	weakly similar to (120)AT5G11790| Symbols: | Ndr family protein | chr5:3799683-3802497 FORWARDvery weakly similar to (80.5)SF	
EV075965	1.397	weakly similar to (140)AT3G13910| Symbols: | similar to unknown protein [Arabidopsis thaliana] (TAIR:AT2G19460.1); similar to unk	-1.431
JCVI_15728	1.396	weakly similar to (150)AT5G40170| Symbols: | disease resistance family protein | chr5:16082407-16084785 REVERSE no original desc	
EX089495	1.396	moderately similar to (257)AT4G08500| Symbols: ATMEKK1, MAPKKK8, MEKK1 | MEKK1 (MYTOGEN ACTIVATED PROTEIN	
AM392180	1.395	moderately similar to (249)AT3G26670| Symbols: | similar to permease-related [Arabidopsis thaliana] (TAIR:AT3G23870.1); similar to	
JCVI_31982	1.395	moderately similar to (367)AT5G19960| Symbols: | RNA recognition motif (RRM)-containing protein | chr5:6744031-6746135 FORWA	
DW997567	1.395	weakly similar to (126)AT3G25140| Symbols: GAUT8, QUA1 | GAUT8/QUA1 (GALACTURONOSYLTRANSFERASE 8, QUASIMC	
JCVI_16159	1.394	highly similar to (513)AT5G52060| Symbols: ATBAG1 | ATBAG1 (ARABIDOPSIS THALIANA BCL-2-ASSOCIATED ATHANOGE	
JCVI_9278	1.394	moderately similar to (209)AT3G54620| Symbols: ATBZIP25, BZO2H4 | ATBZIP25/BZO2H4 (ARABIDOPSIS THALIANA BASIC L	
JCVI_21350	1.394	moderately similar to (276)AT3G56590| Symbols: | hydroxyproline-rich glycoprotein family protein | chr3:20976084-20978763 FORWA	
JCVI_13309	1.393	highly similar to (836)AT5G27380| Symbols: GSH2, GSHB | GSH2/GSHB (GLUTATHIONE SYNTHETASE 2); glutathione synthase |	
JCVI_19485	1.392	highly similar to (687)AT3G20770| Symbols: EIN3 | EIN3 (ETHYLENE-INSENSITIVE3); transcription factor | chr3:7260708-7262594	
EE424791	1.391	moderately similar to (271)AT3G06450| Symbols: | anion exchange family protein | chr3:1976091-1979309 REVERSE [20158]	-1.683
JCVI_26445	1.388	moderately similar to (366)AT1G62740| Symbols: | stress-inducible protein, putative | chr1:23234691-23237045 FORWARDmoderately	
JCVI_41490	1.388	highly similar to (671)AT1G78280| Symbols: | transcription factor jumonji (jmjC) domain-containing protein | chr1:29457716-29462011	
JCVI_31108	1.388	highly similar to (603)AT5G13710| Symbols: CPH, SMT1 | SMT1 (STEROL METHYLTRANSFERASE 1) | chr5:4424051-4426869 R	
JCVI_36484	1.387	weakly similar to (179)AT1G35720| Symbols: OXY5, ATOXY5, ANNAT1 | ANNAT1 (ANNEXIN ARABIDOPSIS 1); calcium ion bin	
EV213570	1.385	weakly similar to (104)AT1G04430| Symbols: | dehydration-responsive protein-related | chr1:1198859-1201300 FORWARD [21491] 13	
ES998935	1.385	moderately similar to (232)AT1G61210| Symbols: | WD-40 repeat family protein / katanin p80 subunit, putative | chr1:22568450-22575	
JCVI_12446	1.383	moderately similar to (269)AT2G31230| Symbols: ATERF15 | ATERF15 (ETHYLENE-RESPONSIVE ELEMENT BINDING FACTO	-1.418
JCVI_2650	1.382	weakly similar to (194)AT4G11820| Symbols: HMGS, EMB2778, MVA1 | MVA1 (HYDROXYMETHYLGLUTARYL-COA SYNTHA	
JCVI_1756	1.379	highly similar to (531)AT2G36880| Symbols: MAT3 | MAT3 (METHIONINE ADENOSYLTRANSFERASE 3) | chr2:15486800-15487	
JCVI_22767	1.379	moderately similar to (370)AT1G05810| Symbols: ARA, Ara-1, AtRab11D, AtRABA5e | ARA/Ara-1/AtRABA5e/AtRab11D (Arabidops	
JCVI_585	1.378	moderately similar to (298)AT2G15970| Symbols: WCOR413, WCOR413-LIKE, ATCOR413-PM1, FL3-5A3, COR413-PM1 | COR413	-1.441
EE464302	1.378	moderately similar to (215)AT4G38940| Symbols: | kelch repeat-containing F-box family protein | chr4:18152842-18153954 FORWAR	-1.811
JCVI_4854	1.377	highly similar to (694)AT5G05170| Symbols: CESA3, IXR1, ATCESA3, ATH-B, CEV1 | CESA3 (CELLULOSE SYNTHASE 3); cellu	
EV195324	1.377	moderately similar to (228)AT1G73920| Symbols: | lipase family protein | chr1:27795428-27798359 FORWARD [21489]	
JCVI_5952	1.377	moderately similar to (251)AT1G35720| Symbols: OXY5, ATOXY5, ANNAT1 | ANNAT1 (ANNEXIN ARABIDOPSIS 1); calcium ion	
JCVI_15700	1.376	moderately similar to (385)AT4G26070| Symbols: NMAPKK, ATMEK1, MKK1, MEK1 | MEK1 (mitogen-activated protein kinase kina	-1.272
JCVI_16959	1.375	highly similar to (529)AT5G64700| Symbols: | nodulin MtN21 family protein | chr5:25882416-25884071 REVERSE no original descrip	
JCVI_27012	1.374	moderately similar to (217)AT2G01450| Symbols: ATMPK17 | ATMPK17 (Arabidopsis thaliana MAP kinase 17); MAP kinase | chr2:19	
EV112896	1.374	moderately similar to (296)AT3G11570| Symbols: | similar to unknown protein [Arabidopsis thaliana] (TAIR:AT5G06230.1); similar to	
JCVI_23007	1.373	moderately similar to (471)AT2G25180| Symbols: ARR12 | ARR12 (ARABIDOPSIS RESPONSE REGULATOR 12); transcription fact	
JCVI_30128	1.373	moderately similar to (446)AT2G32440| Symbols: CYP88A4, KAO2 | KAO2 (ENT-KAURENOIC ACID HYDROXYLASE 2); oxygen	
JCVI_12068	1.373	moderately similar to (374)AT4G01850| Symbols: SAM-2, MAT2 | MAT2/SAM-2 (S-adenosylmethionine synthetase 2) | chr4:796298-7	
JCVI_21472	1.372	moderately similar to (213)AT3G53380| Symbols: | lectin protein kinase family protein | chr3:19800182-19802329 REVERSE no origin	
EV219697	1.372	moderately similar to (405)AT5G64740| Symbols: CESA6, IXR2, E112, PRC1 | CESA6 (CELLULOSE SYNTHASE 6); transferase, tra	
EV156807	1.372	moderately similar to (220)AT4G17890| Symbols: AGD8 | AGD8 (ARF-GAP DOMAIN 8); DNA binding | chr4:9937134-9939001 FOR	
ES967879	1.371	no similarity	
ES939368	1.370	weakly similar to (116)AT1G52260| Symbols: ATPDIL1-5 | ATPDIL1-5 (PDI-LIKE 1-5); thiol-disulfide exchange intermediate | chr1:19	
JCVI_9360	1.368	moderately similar to (231)AT2G40890| Symbols: CYP98A3 | CYP98A3 (CYTOCHROME P450, FAMILY 98, SUBFAMILY A, POL	
EV148147	1.367	no similarity	
BG544859	1.367	no similarity	
JCVI_1044	1.366	highly similar to (857)AT5G02500| Symbols: HSP70-1, AT-HSC70-1, HSC70, HSC70-1 | HSC70-1 (heat shock cognate 70 kDa protein	
ES999057	1.366	no similarity	-1.694
EV000942	1.366	no similarity	-1.569
EE408187	1.366	moderately similar to (206)AT1G27290| Symbols: | similar to unknown [Populus trichocarpa] (GB:ABK95086.1) | chr1:9481775-94825	
JCVI_32576	1.365	moderately similar to (451)AT4G01700| Symbols: | chitinase, putative | chr4:732413-733487 REVERSEmoderately similar to (313)CH	-1.647
JCVI_12962	1.365	moderately similar to (374)AT1G15130| Symbols: | hydroxyproline-rich glycoprotein family protein | chr1:5206212-5209843 REVERSI	
JCVI_12021	1.364	moderately similar to (294)AT1G01640| Symbols: | speckle-type POZ protein-related | chr1:231164-231915 REVERSE no original desc	
EV179644	1.364	moderately similar to (369)AT4G35500| Symbols: | protein kinase family protein | chr4:16857480-16859412 FORWARD [21487]	
EX042042	1.363	moderately similar to (243)AT4G22910| Symbols: | signal transducer | chr4:12012754-12015674 FORWARD [21811] 1 638 656	
JCVI_7604	1.362	weakly similar to (155)AT5G25540| Symbols: CID6 | CID6 (CTC-Interacting Domain 6); protein binding | chr5:8891773-8892365 REV	-1.617
EV125716	1.360	no similarity	
ES969275	1.360	no similarity	
EE562704	1.360	no similarity	
EE439049	1.359	no similarity	-1.437
EE423832	1.358	moderately similar to (225)AT5G32470| Symbols: | similar to unnamed protein product [Vitis vinifera] (GB:CAO22231.1); similar to un	
JCVI_9192	1.358	weakly similar to (164)AT5G08130| Symbols: BIM1 | BIM1 (BES1-interacting Myc-like protein 1) | chr5:2606656-2609572 REVERSE	
CX194769	1.357	no similarity	
JCVI_31980	1.356	weakly similar to (142)AT5G04560| Symbols: DME1, DME | DME (DEMETER) | chr5:1309787-1318092 FORWARD no original desc	
ES978320	1.355	no similarity	
EV217231	1.354	moderately similar to (243)AT5G65500| Symbols: | protein kinase family protein | chr5:26198319-26201223 REVERSE [21492] 54 73	-1.582
JCVI_14238	1.354	moderately similar to (273)AT5G19980| Symbols: | integral membrane family protein | chr5:6749909-6750934 REVERSE no original d	-2.005
JCVI_4038	1.354	moderately similar to (220)AT5G19250| Symbols: | Identical to Uncharacterized GPI-anchored protein At5g19250 precursor [Arabidops	-1.330
DY001166	1.353	weakly similar to (155)AT5G67360| Symbols: ARA12 | ARA12; subtilase | chr5:26889418-26891691 REVERSE [18967]	-1.181
JCVI_34579	1.353	highly similar to (711)AT1G30620| Symbols: HSR8, MUR4, UXE1 | HSR8/MUR4/UXE1 (MURUS 4) | chr1:10855478-10857952 FOR	
JCVI_30132	1.351	moderately similar to (364)AT4G00970| Symbols: | protein kinase family protein | chr4:418437-421694 FORWARD no original descrip	
JCVI_12285	1.351	weakly similar to (126)AT2G30360| Symbols: SIP4, SNRK3.22, PKS5, CIPK11 | CIPK11 (SOS3-INTERACTING PROTEIN 4); kinase	-1.292
JCVI_17549	1.351	highly similar to (508)AT5G05170| Symbols: CESA3, IXR1, ATCESA3, ATH-B, CEV1 | CESA3 (CELLULOSE SYNTHASE 3); cellu	
ES963657	1.350	no similarity	-2.367
JCVI_9882	1.349	weakly similar to (121)AT4G35890| Symbols: | La domain-containing protein | chr4:16997436-17000413 FORWARD no original descr	-1.321
EE566920	1.349	no similarity	-1.498
JCVI_16439	1.349	moderately similar to (461)AT1G80840| Symbols: ATWRKY40, WRKY40 | WRKY40 (WRKY DNA-binding protein 40); transcription	
JCVI_24918	1.349	moderately similar to (282)AT2G18790| Symbols: HY3, OOP1, PHYB | PHYB (PHYTOCHROME B); G-protein coupled photorecepto	

JCVI_20841	1.348	highly similar to (758)AT5G54860| Symbols: | integral membrane transporter family protein | chr5:22301947-22304251 FORWARD no	-1.757
JCVI_33208	1.348	weakly similar to (120)AT2G35430| Symbols: | zinc finger (CCCH-type) family protein | chr2:14908093-14909336 REVERSE no origi	
EV050684	1.348	weakly similar to (144)AT4G18800| Symbols: AthSGBP, AtRab11B, AtRABA1d | AtRABA1d/AtRab11B/AthSGBP (Arabidopsis Rab (	-1.721
RC_CX192824	1.347	no similarity	
JCVI_27918	1.347	moderately similar to (451)AT5G18520| Symbols: | similar to unknown protein [Arabidopsis thaliana] (TAIR:AT3G09570.1); similar to	
JCVI_9978	1.345	moderately similar to (226)AT3G18030| Symbols: HAL3A, HAL3, ATHAL3, ATHAL3A | ATHAL3A (Arabidopsis thaliana Hal3-like p	
JCVI_20355	1.344	no original description	-1.930
JCVI_37648	1.344	moderately similar to (340)AT2G45590| Symbols: | protein kinase family protein | chr2:18793799-18795850 FORWARDweakly similar	
AM057911	1.343	weakly similar to (151)AT5G16820| Symbols: HSFA1B, ATHSFA1B, HSF3 | HSF3 (HEAT SHOCK FACTOR 3); DNA binding / trans	
EX095267	1.339	moderately similar to (303)AT5G64360| Symbols: | DNAJ heat shock N-terminal domain-containing protein | chr5:25754434-25755828	
JCVI_22297	1.339	moderately similar to (486)AT5G07350| Symbols: | tudor domain-containing protein / nuclease family protein | chr5:2320345-2324893 I	
EX071143	1.338	no similarity	
JCVI_11858	1.338	moderately similar to (471)AT2G44450| Symbols: | glycosyl hydrolase family 1 protein | chr2:18348042-18350820 FORWARDmoderat	
DY023408	1.337	no similarity	
EV064976	1.337	no similarity	-1.250
ES901208	1.337	no similarity	
JCVI_18640	1.335	very weakly similar to (91.3)AT1G08910| Symbols: EMB3001 | EMB3001 (EMBRYO DEFECTIVE 3001); zinc ion binding | chr1:2856	
CV432194	1.335	weakly similar to (127)AT4G21910| Symbols: | MATE efflux family protein | chr4:11625833-11630976 REVERSE [16490]	-1.515
EV034881	1.335	weakly similar to (150)AT1G78110| Symbols: | similar to unknown protein [Arabidopsis thaliana] (TAIR:AT1G22230.1); similar to unn	
JCVI_21772	1.334	moderately similar to (242)AT5G15770| Symbols: ATGNA1 | ATGNA1 (ARABIDOPSIS THALIANA GLUCOSE-6-PHOSPHATE AC	
JCVI_29084	1.332	moderately similar to (236)AT5G16300| Symbols: | similar to hypothetical protein OsI_003871 [Oryza sativa (indica cultivar-group)] (G	-1.353
JCVI_33550	1.331	no original description	-1.450
JCVI_37751	1.331	moderately similar to (215)AT4G12090| Symbols: | cornichon family protein | chr4:7242159-7242918 REVERSE no original descriptio	
EE447474	1.329	no similarity	
JCVI_11377	1.329	no original description	-2.033
RC_EE558293	1.326	no similarity	
EE569403	1.324	no similarity	
JCVI_973	1.323	highly similar to (505)AT4G21570| Symbols: | similar to unknown protein [Arabidopsis thaliana] (TAIR:AT1G11200.1); similar to hypo	
EV214277	1.322	weakly similar to (198)AT2G01670| Symbols: ATNUDT17 | ATNUDT17 (Arabidopsis thaliana Nudix hydrolase homolog 17); hydrolas	
JCVI_39140	1.321	weakly similar to (163)AT5G59960| Symbols: | similar to unnamed protein product [Vitis vinifera] (GB:CAO21698.1) | chr5:24159578-	
JCVI_6035	1.320	weakly similar to (175)AT5G53560| Symbols: B5 #2, ATB5-A | ATB5-A (Cytochrome b5 A) | chr5:21776854-21777579 FORWARDw	
JCVI_16610	1.319	no original description	
EX104820	1.318	very weakly similar to (89.4)AT5G62090| Symbols: | similar to SLK1 (SEUSS-LIKE 1), transcription regulator [Arabidopsis thaliana] (T	
JCVI_17944	1.318	moderately similar to (278)AT3G13650| Symbols: | disease resistance response | chr3:4463063-4463623 FORWARD no original descrip	
JCVI_33755	1.318	moderately similar to (457)AT5G44370| Symbols: | transporter-related | chr5:17892374-17893672 REVERSE no original description	-1.349
DY012174	1.317	no similarity	
JCVI_19944	1.317	moderately similar to (226)AT2G18700| Symbols: TPS11, ATTPSB, ATTPS11 | ATTPS11 (Arabidopsis thaliana trehalose phosphatase/	
JCVI_26254	1.314	moderately similar to (239)AT1G28510| Symbols: | similar to unknown protein [Arabidopsis thaliana] (TAIR:AT3G58150.1); similar to	
JCVI_750	1.314	weakly similar to (196)AT2G45820| Symbols: | DNA-binding protein, putative | chr2:18870221-18871650 REVERSEweakly similar to	
ES968542	1.313	no similarity	-1.099
EE561370	1.313	moderately similar to (256)AT3G51160| Symbols: MUR_1, GMD2, MUR1 | MUR1 (MURUS 1) | chr3:19018211-19019332 REVERSI	-1.402
JCVI_8580	1.313	no original description	-1.821
ES938497	1.312	moderately similar to (243)AT5G61390| Symbols: | exonuclease family protein | chr5:24696028-24698083 REVERSE [21390]	
CX269309	1.309	very weakly similar to (81.3)GRP2_SINAL [16816]	
JCVI_3465	1.308	moderately similar to (437)AT5G22250| Symbols: | CCR4-NOT transcription complex protein, putative | chr5:7365608-7366444 REVE	
CX190307	1.308	moderately similar to (462)AT3G53180| Symbols: | glutamate-ammonia ligase | chr3:19718046-19722166 FORWARD [16807]	-1.490
JCVI_16123	1.308	moderately similar to (316)AT4G36920| Symbols: FLO2, FL1, AP2 | AP2 (APETALA 2); transcription factor | chr4:17400995-1740313	
JCVI_22631	1.307	weakly similar to (104)AT1G74950| Symbols: JAZ2, TIFY10B | JAZ2/TIFY10B (JASMONATE-ZIM-DOMAIN PROTEIN 2) | chr1:28	
JCVI_29757	1.307	no original description	
JCVI_31644	1.307	highly similar to (568)AT5G61780| Symbols: | tudor domain-containing protein / nuclease family protein | chr5:24839238-24843867 FO	
DY014723	1.306	moderately similar to (320)AT2G41790| Symbols: | peptidase M16 family protein / insulinase family protein | chr2:17436531-17443188	-1.333
JCVI_38583	1.304	moderately similar to (431)AT3G21230| Symbols: 4CL5 | 4CL5 (4-COUMARATE:COA LIGASE 5); 4-coumarate-CoA ligase | chr3:74	
JCVI_32787	1.304	moderately similar to (323)AT5G13460| Symbols: IQD11 | IQD11 (IQ-domain 11); calmodulin binding | chr5:4316326-4318250 FORW	
JCVI_28827	1.302	weakly similar to (101)AT1G16510| Symbols: | auxin-responsive family protein | chr1:5644777-5645220 REVERSE no original descrip	-2.826
JCVI_33686	1.301	moderately similar to (215)AT5G59670| Symbols: | leucine-rich repeat protein kinase, putative | chr5:24058764-24062704 FORWARD	-1.470
JCVI_34578	1.301	weakly similar to (114)AT1G15415| Symbols: | The protein encoded by this gene was identified as a part of pollen proteome by mass sp	
JCVI_37998	1.300	highly similar to (518)AT1G08800| Symbols: | similar to unknown protein [Arabidopsis thaliana] (TAIR:AT2G30690.1); similar to unna	
JCVI_38497	1.300	no original description	
JCVI_4012	1.298	moderately similar to (204)AT4G35840| Symbols: | zinc finger (C3HC4-type RING finger) family protein | chr4:16981087-16982269 F	
JCVI_35904	1.298	moderately similar to (261)AT1G60960| Symbols: IRT3 | IRT3 (Iron regulated transporter 3); cation transmembrane transporter/ metal ic	-1.675
JCVI_24898	1.297	moderately similar to (468)AT5G14790| Symbols: | binding | chr5:4784061-4785511 FORWARD no original description	
ES992852	1.296	no similarity	
EV118219	1.295	moderately similar to (405)AT5G06400| Symbols: | pentatricopeptide (PPR) repeat-containing protein | chr5:1955960-1959052 FORWA	-1.394
JCVI_30493	1.294	very weakly similar to (81.3)AT5G12170| Symbols: | similar to unknown protein [Arabidopsis thaliana] (TAIR:AT5G19380.1); similar to	
JCVI_25092	1.294	moderately similar to (402)AT5G51180| Symbols: | similar to unknown protein [Arabidopsis thaliana] (TAIR:AT4G25770.1); similar to	-1.283
EV019393	1.292	moderately similar to (232)AT3G13780| Symbols: | similar to SMAD/FHA [Medicago truncatula] (GB:ABN05826.1); contains InterPro	
EV156277	1.290	weakly similar to (186)AT2G21390| Symbols: | coatomer protein complex, subunit alpha, putative | chr2:9159508-9163657 FORWARD	-1.351
JCVI_21127	1.289	moderately similar to (447)AT3G01220| Symbols: ATHB20 | ATHB20 (ARABIDOPSIS THALIANA HOMEOBOX PROTEIN 20); DI	
JCVI_35562	1.288	weakly similar to (124)AT2G20370| Symbols: KAM1, MUR3 | KAM1/MUR3 (MURUS 3); catalytic/ transferase, transferring glycosyl g	
JCVI_16292	1.288	no original description	
JCVI_1386	1.287	moderately similar to (342)AT2G39900| Symbols: | LIM domain-containing protein | chr2:16666214-16667345 FORWARDmoderately	
JCVI_7798	1.287	moderately similar to (251)AT3G03160| Symbols: | similar to unknown protein [Arabidopsis thaliana] (TAIR:AT5G17190.1); similar to	-1.208
JCVI_18102	1.286	moderately similar to (497)AT1G07040| Symbols: | similar to unknown protein [Arabidopsis thaliana] (TAIR:AT1G27030.1); similar to	-1.482
JCVI_12120	1.285	weakly similar to (177)AT3G18610| Symbols: ATRANGAP1 | ATRANGAP1 (RAN GTPASE-ACTIVATING PROTEIN 1); nucleic aci	-1.662
JCVI_29598	1.285	highly similar to (895)AT1G74030| Symbols: | enolase, putative | chr1:27843126-27845562 REVERSEhighly similar to (580)ENO2_M	-1.533
L38152	1.285	weakly similar to (102)AT1G63000| Symbols: UER1, NRS/ER | NRS/ER (NUCLEOTIDE-RHAMNOSE SYNTHASE/EPIMERASE-R	
DN964507	1.285	weakly similar to (129)AT5G27380| Symbols: GSH2, GSHB | GSH2/GSHB (GLUTATHIONE SYNTHETASE 2); glutathione synthase	
EX119284	1.284	no similarity	-1.310
EV039252	1.284	weakly similar to (165)AT5G60820| Symbols: | zinc finger (C3HC4-type RING finger) family protein | chr5:24486862-24488121 FORW	
JCVI_41218	1.281	highly similar to (573)AT5G05460| Symbols: | hydrolase, acting on glycosyl bonds | chr5:1615616-1618772 FORWARD no original des	-1.576

JCVI_41530	1.281	moderately similar to (233)AT4G33220| Symbols: | pectinesterase family protein | chr4:16024449-16026134 FORWARDweakly similar	
JCVI_36191	1.280	weakly similar to (175)AT3G12560| Symbols: ATTBP2, TRFL9 | TRFL9 (TRF-LIKE 9); DNA binding | chr3:3982279-3984855 REVEI	-1.472
JCVI_8000	1.280	moderately similar to (308)AT3G14830| Symbols: | similar to unknown protein [Arabidopsis thaliana] (TAIR:AT1G53450.2); similar to	
EE527348	1.279	no similarity	
JCVI_28265	1.279	moderately similar to (243)AT3G60800| Symbols: | zinc finger (DHHC type) family protein | chr3:22478461-22480248 REVERSE no c	
EV119008	1.279	moderately similar to (438)AT4G10590| Symbols: | ubiquitin carboxyl-terminal hydrolase family protein | chr4:6538915-6543268 REVI	
EE551077	1.277	no similarity	
EV138431	1.276	weakly similar to (185)AT5G11790| Symbols: | Ndr family protein | chr5:3799683-3802497 FORWARDweakly similar to (117)SF21_I	
ES901120	1.276	weakly similar to (122)AT2G33170| Symbols: | leucine-rich repeat transmembrane protein kinase, putative | chr2:14063448-14066906 R	-1.851
EV072419	1.274	weakly similar to (196)AT3G08530| Symbols: | clathrin heavy chain, putative | chr3:2587177-2595417 REVERSE [21443]	
JCVI_41949	1.274	no original description	
ES980563	1.272	moderately similar to (351)AT1G12600| Symbols: | similar to ATUTR2/UTR2 (UDP-GALACTOSE TRANSPORTER 2) [Arabidopsis	
JCVI_22668	1.272	weakly similar to (169)AT2G01720| Symbols: | ribophorin I family protein | chr2:317192-320015 REVERSE no original description	
JCVI_32343	1.271	moderately similar to (374)AT5G19000| Symbols: ATBPM1 | ATBPM1 (BTB-POZ AND MATH DOMAIN 1); protein binding | chr5:6	
JCVI_11485	1.270	moderately similar to (351)AT5G05460| Symbols: | hydrolase, acting on glycosyl bonds | chr5:1615616-1618772 FORWARD no origin	-1.590
AM390423	1.270	moderately similar to (254)AT2G45910| Symbols: | protein kinase family protein / U-box domain-containing protein | chr2:18901593-18	
JCVI_1934	1.268	moderately similar to (226)AT2G36320| Symbols: | zinc finger (AN1-like) family protein | chr2:15236467-15236952 FORWARDweakl	-1.776
JCVI_34718	1.267	weakly similar to (187)AT5G11060| Symbols: KNAT4 | KNAT4 (KNOTTED1-LIKE HOMEOBOX GENE 4); transcription factor | chr5	-2.093
EV058221	1.267	moderately similar to (250)AT5G50020| Symbols: | zinc finger (DHHC type) family protein | chr5:20368817-20371350 FORWARD [21	
EE559503	1.266	weakly similar to (132)AT2G39260| Symbols: | RNA binding | chr2:16399366-16406666 REVERSE [20153] 21 630 630	
JCVI_24203	1.266	moderately similar to (350)AT4G39550| Symbols: | kelch repeat-containing F-box family protein | chr4:18380675-18381853 REVERSE	-1.109
ES264889	1.266	weakly similar to (135)AT5G63260| Symbols: | zinc finger (CCCH-type) family protein | chr5:25379126-25381679 FORWARD [15723	-1.299
JCVI_9352	1.265	no original description	-1.517
JCVI_25355	1.265	no original description	
RC_EV173417	1.262	no similarity	
CB686302	1.262	weakly similar to (167)AT5G22070| Symbols: | similar to unknown protein [Arabidopsis thaliana] (TAIR:AT3G52060.2); similar to unk	-1.285
JCVI_25178	1.261	highly similar to (554)AT3G17860| Symbols: JAZ3, JAI3, TIFY6B | JAI3/JAZ3/TIFY6B (JASMONATE-ZIM-DOMAIN PROTEIN 3)	
EX098535	1.261	moderately similar to (393)AT3G28740| Symbols: CYP81D1 | cytochrome P450 family protein | chr3:10790001-10791789 REVERSEw	
EE550466	1.261	no similarity	-1.205
JCVI_26431	1.260	weakly similar to (163)AT5G08130| Symbols: BIM1 | BIM1 (BES1-interacting Myc-like protein 1) | chr5:2606656-2609572 REVERSE	
CB617683	1.258	no similarity	
BQ791460	1.258	weakly similar to (131)AT3G13330| Symbols: | binding | chr3:4319811-4330068 REVERSE [8791]	-1.379
DY001867	1.258	weakly similar to (197)AT5G57580| Symbols: | calmodulin-binding protein | chr5:23332220-23334909 REVERSE [18968] 1 585 621	
JCVI_17831	1.256	weakly similar to (144)AT5G56170| Symbols: | similar to unknown protein [Arabidopsis thaliana] (TAIR:AT4G26466.1); similar to unn	-1.898
EX126541	1.255	highly similar to (520)AT1G66830| Symbols: | leucine-rich repeat transmembrane protein kinase, putative | chr1:24934363-24936497 RI	-1.801
ES908955	1.254	moderately similar to (398)AT3G20170| Symbols: | armadillo/beta-catenin repeat family protein | chr3:7041786-7043213 FORWARD [	-1.307
JCVI_6367	1.252	weakly similar to (182)AT1G76490| Symbols: HMGR1, HMG1 | HMG1 (3-HYDROXY-3-METHYLGLUTARYL COA REDUCTASE	
JCVI_9802	1.251	moderately similar to (211)AT1G08450| Symbols: CRT3 | CRT3 (CALRETICULIN 3); calcium ion binding | chr1:2668005-2671797 RI	
AM062056	1.249	weakly similar to (166)AT2G37710| Symbols: RLK | RLK (RECEPTOR LECTIN KINASE); kinase | chr2:15822012-15824039 REVER	
EV178105	1.249	weakly similar to (118)AT3G14230| Symbols: RAP2.2 | RAP2.2; DNA binding / transcription factor | chr3:4737623-4739007 REVERSI	
ES947935	1.249	very weakly similar to (81.6)AT2G16230| Symbols: | glycosyl hydrolase family 17 protein | chr2:7043103-7045408 REVERSE [21393]	
JCVI_29047	1.249	no original description	-1.401
JCVI_37690	1.247	moderately similar to (208)AT5G17910| Symbols: | similar to unknown protein [Arabidopsis thaliana] (TAIR:AT2G29620.1); similar to	-1.468
AM386037	1.247	moderately similar to (210)AT3G09910| Symbols: AtRab18C, AtRABC2b | AtRABC2b/AtRab18C (Arabidopsis Rab GTPase homolog (	
JCVI_36464	1.246	highly similar to (568)AT1G78570| Symbols: RHM1, ROL1 | RHM1/ROL1 (RHAMNOSE BIOSYNTHESIS1); UDP-L-rhamnose synth	
DY023978	1.246	moderately similar to (220)AT3G27010| Symbols: PCF1, AT-TCP20 | AT-TCP20 (ARABIDOPSIS THALIANA TEOSINTE BRANCH	-1.196
EX023486	1.245	moderately similar to (351)AT3G06450| Symbols: | anion exchange family protein | chr3:1976091-1979309 REVERSE [21809]	-1.297
EX093322	1.244	no similarity	
JCVI_12964	1.244	weakly similar to (191)AT1G30360| Symbols: ERD4 | ERD4 (EARLY-RESPONSIVE TO DEHYDRATION 4) | chr1:10715874-10718	
JCVI_17105	1.244	very weakly similar to (97.1)AT5G52300| Symbols: RD29B, LTI65 | LTI65/RD29B (RESPONSIVE TO DESSICATION 29B) | chr5:21	
EE557314	1.244	no similarity	-1.071
JCVI_35990	1.241	moderately similar to (397)AT4G16480| Symbols: ATINT4 | ATINT4 (INOSITOL TRANSPORTER 4); carbohydrate transmembrane tr	
EX129699	1.240	moderately similar to (299)AT1G12620| Symbols: | pentatricopeptide (PPR) repeat-containing protein | chr1:4294881-4296746 REVER	
EE405085	1.240	moderately similar to (297)AT5G57110| Symbols: AT-ACA8 | ACA8 (AUTOINHIBITED CA2+ -ATPASE, ISOFORM 8); calmodulin	
JCVI_9146	1.237	weakly similar to (127)AT5G64740| Symbols: CESA6, IXR2, E112, PRC1 | CESA6 (CELLULOSE SYNTHASE 6); transferase, transfe	
JCVI_35894	1.236	weakly similar to (186)AT2G44940| Symbols: | AP2 domain-containing transcription factor TINY, putative | chr2:18544369-18545256	
EX087317	1.235	moderately similar to (204)AT2G38290| Symbols: ATAMT2 | ATAMT2 (AMMONIUM TRANSPORTER 2); ammonium transmembra	
L38201	1.235	weakly similar to (135)AT1G10040| Symbols: | similar to unknown protein [Arabidopsis thaliana] (TAIR:AT4G25770.1); similar to unn	
JCVI_34343	1.235	weakly similar to (140)AT4G09890| Symbols: | similar to unknown protein [Arabidopsis thaliana] (TAIR:AT2G47480.1); similar to unk	
JCVI_18766	1.235	moderately similar to (245)AT3G54630| Symbols: | similar to kinetochore protein [Capsella rubella] (GB:BAF63163.1); similar to kinet	-1.290
JCVI_14327	1.234	highly similar to (511)AT5G14950| Symbols: GMII, ATGMII | ATGMII/GMII (GOLGI ALPHA-MANNOSIDASE II); alpha-mannosida	
CD840195	1.233	no similarity	-1.656
JCVI_25000	1.232	weakly similar to (188)AT3G19130| Symbols: ATRBP47B | ATRBP47B (RNA-BINDING PROTEIN 47B); RNA binding | chr3:66114(	
EV030709	1.232	no similarity	-1.727
JCVI_11916	1.231	moderately similar to (269)AT4G38940| Symbols: | kelch repeat-containing F-box family protein | chr4:18152842-18153954 FORWAR	-1.612
EV182815	1.230	weakly similar to (155)AT5G63980| Symbols: ATSAL1, HOS2, FRY1, SAL1 | SAL1 (FIERY1); 3'(2'),5'-bisphosphate nucleotidase/ ino:	-1.289
EV179488	1.228	very weakly similar to (99.0)AT4G17890| Symbols: AGD8 | AGD8 (ARF-GAP DOMAIN 8); DNA binding | chr4:9937134-9939001 FO	
EV058877	1.228	no similarity	
EX060560	1.226	weakly similar to (135)AT5G23450| Symbols: ATLCBK1 | ATLCBK1 (A. THALIANA LONG-CHAIN BASE (LCB) KINASE 1); diac	
JCVI_17945	1.225	moderately similar to (221)AT2G30250| Symbols: ATWRKY25, WRKY25 | WRKY25 (WRKY DNA-binding protein 25); transcription	-1.584
EE473009	1.224	weakly similar to (126)AT4G13020| Symbols: MHK | MHK | chr4:7603944-7606729 FORWARD [20163]	-1.289
JCVI_33851	1.223	moderately similar to (265)AT1G77680| Symbols: | ribonuclease II family protein | chr1:29197082-29200857 REVERSE no original de	
JCVI_30684	1.221	moderately similar to (268)AT3G15820| Symbols: | phosphatidic acid phosphatase-related / PAP2-related | chr3:5351224-5353580 FOR	
EE531941	1.221	moderately similar to (387)AT5G63770| Symbols: ATDGK2 | ATDGK2 (DIACYLGLYCEROL KINASE 2) | chr5:25536981-2553963(	
JCVI_36735	1.221	no original description	
JCVI_33095	1.219	moderately similar to (301)AT1G01240| Symbols: | similar to unknown protein [Arabidopsis thaliana] (TAIR:AT2G46550.1); similar to	-1.709
ES992760	1.218	weakly similar to (149)AT4G18150| Symbols: | similar to unknown protein [Arabidopsis thaliana] (TAIR:AT1G29350.1); similar to unn	
RC_EE560578	1.217	no similarity	
JCVI_10727	1.216	moderately similar to (327)AT3G26950| Symbols: | binding | chr3:9942439-9944648 REVERSE no original description	
JCVI_42409	1.215	no original description	

EE442388	1.214	weakly similar to (107)AT5G06110| Symbols: | DNAJ heat shock N-terminal domain-containing protein / cell division protein-related | c	-1.161
JCVI_235	1.214	moderately similar to (403)AT5G17920| Symbols: ATMETS, ATMS1, ATCIMS | ATCIMS (COBALAMIN-INDEPENDENT METHIO	
JCVI_24369	1.213	very weakly similar to (84.0)AT4G33690| Symbols: | similar to hypothetical protein [Vitis vinifera] (GB:CAN61243.1) | chr4:16175201-	
JCVI_7062	1.213	moderately similar to (415)AT3G16500| Symbols: IAA26, PAP1 | PAP1 (PHYTOCHROME-ASSOCIATED PROTEIN 1); transcriptior	
JCVI_24537	1.212	weakly similar to (158)AT3G51090| Symbols: | similar to unknown protein [Arabidopsis thaliana] (TAIR:AT2G16460.1); similar to unn	
JCVI_36606	1.211	very weakly similar to (80.9)AT2G39260| Symbols: | RNA binding | chr2:16399366-16406666 REVERSE no original description	
EH420964	1.211	no similarity	-1.318
JCVI_28969	1.210	highly similar to (740)AT3G26890| Symbols: | similar to unknown protein [Arabidopsis thaliana] (TAIR:AT5G41110.1); similar to unna	
EX056479	1.210	moderately similar to (303)AT1G75140| Symbols: | Identical to Uncharacterized membrane protein At1g75140 [Arabidopsis Thaliana] (	-1.662
AT000535	1.209	no similarity	-1.886
ES987983	1.209	moderately similar to (318)AT5G19000| Symbols: ATBPM1 | ATBPM1 (BTB-POZ AND MATH DOMAIN 1); protein binding | chr5:6	
EE541543	1.208	moderately similar to (372)AT3G52870| Symbols: | calmodulin-binding family protein | chr3:19604343-19606664 REVERSE [20124]	
JCVI_3734	1.207	moderately similar to (404)AT5G27380| Symbols: GSH2, GSHB | GSH2/GSHB (GLUTATHIONE SYNTHETASE 2); glutathione syntl	
DY025083	1.206	very weakly similar to (80.9)AT1G63000| Symbols: UER1, NRS/ER | NRS/ER (NUCLEOTIDE-RHAMNOSE SYNTHASE/EPIMERAS	
JCVI_8231	1.205	moderately similar to (219)AT5G46760| Symbols: | basic helix-loop-helix (bHLH) family protein | chr5:18991458-18993236 FORWAR	
CX265776	1.204	moderately similar to (278)AT1G79990| Symbols: | coatomer protein complex, subunit beta 2 (beta prime), putative | chr1:30090803-30	
EV111198	1.199	no similarity	
JCVI_28138	1.199	weakly similar to (172)AT1G08180| Symbols: | similar to unknown protein [Arabidopsis thaliana] (TAIR:AT5G02420.1) | chr1:2564738	-1.839
ES269450	1.197	no similarity	-1.251
EV207932	1.197	moderately similar to (317)AT3G63070| Symbols: | PWWP domain-containing protein | chr3:23313642-23320550 FORWARD [21491]	
JCVI_3075	1.194	moderately similar to (292)AT1G52140| Symbols: | similar to unknown protein [Arabidopsis thaliana] (TAIR:AT3G16330.1); similar to	
EX135587	1.194	moderately similar to (233)AT5G13860| Symbols: ELC-LIKE | ATELC-LIKE/ELC-LIKE; small conjugating protein ligase | chr5:447321	-1.626
EE422801	1.194	moderately similar to (245)AT5G46760| Symbols: | basic helix-loop-helix (bHLH) family protein | chr5:18991458-18993236 FORWAR	
EE477773	1.193	moderately similar to (221)AT1G12950| Symbols: | MATE efflux family protein | chr1:4419847-4422460 FORWARD [20157]	
JCVI_25334	1.193	moderately similar to (256)AT1G80510| Symbols: | amino acid transporter family protein | chr1:30277992-30279461 FORWARD no or	-1.340
EV182507	1.193	weakly similar to (110)AT3G57480| Symbols: | zinc finger (C2H2 type, AN1-like) family protein | chr3:21289060-21290096 REVERSI	-1.443
JCVI_16533	1.191	moderately similar to (338)AT1G80630| Symbols: | leucine-rich repeat family protein | chr1:30313771-30315507 REVERSE no original	-1.439
EV120366	1.191	no similarity	
JCVI_22799	1.190	weakly similar to (119)AT5G18680| Symbols: AtTLP11 | AtTLP11 (TUBBY LIKE PROTEIN 11); phosphoric diester hydrolase/ transcr	
JCVI_13158	1.190	moderately similar to (296)AT1G67310| Symbols: | calmodulin binding / transcription regulator | chr1:25201845-25206789 REVERSE	
EV143691	1.190	no similarity	-1.191
JCVI_42055	1.189	moderately similar to (342)AT1G05540| Symbols: | similar to unknown protein [Arabidopsis thaliana] (TAIR:AT1G30160.2); contains I	-1.530
EX127270	1.189	moderately similar to (206)AT5G22860| Symbols: | serine carboxypeptidase S28 family protein | chr5:7639910-7642948 REVERSE [21	
JCVI_32496	1.189	moderately similar to (364)AT1G80510| Symbols: | amino acid transporter family protein | chr1:30277992-30279461 FORWARD no or	-1.385
JCVI_8607	1.188	weakly similar to (184)AT5G61260| Symbols: | chromosome scaffold protein-related | chr5:24654335-24655825 FORWARD no origina	
JCVI_16828	1.188	weakly similar to (176)AT2G26980| Symbols: SnRK3.17, CIPK3 | CIPK3 (CBL-INTERACTING PROTEIN KINASE 3); kinase | chr2:1	-1.026
CX266927	1.188	moderately similar to (357)AT2G28520| Symbols: VHA-A1 | VHA-A1 (VACUOLAR PROTON ATPASE A 1); ATPase | chr2:1221710	
JCVI_37957	1.187	moderately similar to (326)AT5G11850| Symbols: | protein kinase family protein | chr5:3816633-3821025 REVERSE no original descri	-1.380
JCVI_23563	1.186	very weakly similar to (100)AT5G58090| Symbols: | glycosyl hydrolase family 17 protein | chr5:23522782-23524419 REVERSE no orig	
EE560299	1.186	no similarity	
BQ791279	1.186	weakly similar to (114)AT5G06700| Symbols: | similar to unknown protein [Arabidopsis thaliana] (TAIR:AT3G12060.1); similar to unn	
JCVI_4797	1.185	moderately similar to (290)AT5G62360| Symbols: | invertase/pectin methylesterase inhibitor family protein | chr5:25057925-25058536 I	1.583
H74706	1.183	no similarity	
JCVI_32989	1.181	moderately similar to (251)AT1G14870| Symbols: | Identical to Uncharacterized protein At1g14870 [Arabidopsis Thaliana] (GB:Q9LQU	
CD816471	1.179	moderately similar to (211)AT1G16710| Symbols: HAC12 | HAC12 (HISTONE ACETYLTRANSFERASE OF THE CBP FAMILY 12)	
EE556982	1.179	no similarity	
CN829977	1.178	weakly similar to (193)AT1G74030| Symbols: | enolase, putative | chr1:27843126-27845562 REVERSEweakly similar to (159)ENO_C	
JCVI_19351	1.178	moderately similar to (201)AT1G27170| Symbols: | ATP binding / protein binding / transmembrane receptor | chr1:9434705-9439206 FO	-1.645
JCVI_34006	1.177	weakly similar to (102)AT3G13222| Symbols: GIP1 | GIP1 (GBF-INTERACTING PROTEIN 1) | chr3:4251294-4254213 REVERSE no	
EV126591	1.177	weakly similar to (191)AT4G11840| Symbols: PLDGAMMA3 | PLDGAMMA3 (phospholipase D gamma 3); phospholipase D | chr4:712	
CD819066	1.177	weakly similar to (140)AT5G19530| Symbols: ACL5 | ACL5 (ACAULIS 5) | chr5:6589178-6591072 REVERSE [13978]	
JCVI_41350	1.176	no original description	
ES996625	1.176	weakly similar to (131)AT5G20030| Symbols: | agenet domain-containing protein | chr5:6764973-6766038 REVERSE [21427]	
EV085251	1.175	weakly similar to (111)AT1G01240| Symbols: | similar to unknown protein [Arabidopsis thaliana] (TAIR:AT2G46550.1); similar to unn	-1.726
EV216944	1.174	moderately similar to (250)AT2G39435| Symbols: | similar to unknown protein [Arabidopsis thaliana] (TAIR:AT3G53540.1); similar to	
EX118978	1.173	no similarity	
JCVI_37548	1.172	no original description	-2.137
EE557757	1.171	no similarity	
JCVI_39954	1.171	no original description	-1.149
EE477104	1.171	weakly similar to (112)AT1G48770| Symbols: | similar to unknown protein [Arabidopsis thaliana] (TAIR:AT3G18295.1); similar to unn	
DY009607	1.171	weakly similar to (194)AT1G60780| Symbols: | clathrin adaptor complexes medium subunit family protein | chr1:22372954-22375550 R	-1.202
ES917232	1.170	weakly similar to (163)AT1G10950| Symbols: | endomembrane protein 70, putative | chr1:3659322-3663622 FORWARD [15718]	-1.160
EE409212	1.170	weakly similar to (185)AT2G15560| Symbols: | similar to unknown protein [Arabidopsis thaliana] (TAIR:AT3G62200.1); similar to ED.	
ES911692	1.169	moderately similar to (226)AT4G31150| Symbols: | endonuclease V family protein | chr4:15143913-15145475 REVERSE [21431]	
JCVI_6058	1.165	moderately similar to (353)AT4G17720| Symbols: | RNA recognition motif (RRM)-containing protein | chr4:9862673-9864511 REVER	-1.367
JCVI_10766	1.164	no original description	
ES932050	1.164	weakly similar to (163)AT5G41950| Symbols: | binding | chr5:16803053-16806588 FORWARD [20143]	-1.533
EV016096	1.163	moderately similar to (330)AT2G28520| Symbols: VHA-A1 | VHA-A1 (VACUOLAR PROTON ATPASE A 1); ATPase | chr2:1221710	
JCVI_14585	1.163	moderately similar to (449)AT2G41740| Symbols: VLN2 | VLN2 (VILLIN 2); actin binding | chr2:17418040-17423956 REVERSE no o	
JCVI_40920	1.162	highly similar to (681)AT3G01780| Symbols: TPLATE | TPLATE; binding | chr3:279178-283406 FORWARD no original description	
JCVI_20764	1.161	weakly similar to (103)AT2G22720| Symbols: | similar to unknown protein [Arabidopsis thaliana] (TAIR:AT4G37860.1); similar to unn	
EE566139	1.154	very weakly similar to (84.7)AT3G63400| Symbols: | peptidyl-prolyl cis-trans isomerase cyclophilin-type family protein | chr3:23423424-	-1.428
JCVI_13887	1.154	moderately similar to (226)AT2G01190| Symbols: | octicosapeptide/Phox/Bem1p (PB1) domain-containing protein | chr2:115022-1172	
EV090632	1.153	weakly similar to (136)AT1G07310| Symbols: | C2 domain-containing protein | chr1:2247774-2248832 REVERSE [21444]	
EE482913	1.153	weakly similar to (139)AT4G22760| Symbols: | pentatricopeptide (PPR) repeat-containing protein | chr4:11958488-11962881 FORWAI	-1.291
EV143107	1.152	moderately similar to (214)AT5G63640| Symbols: | VHS domain-containing protein / GAT domain-containing protein | chr5:25496161-	
JCVI_22892	1.149	weakly similar to (198)AT3G05280| Symbols: | integral membrane Yip1 family protein | chr3:1503998-1505560 REVERSE no original	
JCVI_2353	1.148	moderately similar to (214)AT1G04340| Symbols: | lesion inducing protein-related | chr1:1163344-1164736 REVERSE no original desc	
JCVI_16490	1.147	moderately similar to (202)AT4G24990| Symbols: ATGP4 | ATGP4 (Arabidopsis thaliana geranylgeranylated protein) | chr4:12849983-1	
JCVI_9459	1.147	very weakly similar to (99.8)AT4G30660| Symbols: | hydrophobic protein, putative / low temperature and salt responsive protein, putative	

JCVI_31185	1.146	moderately similar to (228)AT3G13870| Symbols: RHD3 | RHD3 (ROOT HAIR DEFECTIVE 3) | chr3:4565769-4571116 REVERSE m	
ES898531	1.145	moderately similar to (309)AT4G34430| Symbols: ATSWI3D, CHB3 | CHB3 (Arabidopsis thaliana switch 3D); DNA binding / transcrip	
JCVI_37230	1.142	no original description	
JCVI_32624	1.141	highly similar to (561)AT1G49750| Symbols: | leucine-rich repeat family protein | chr1:18414845-18416447 REVERSEweakly similar to	-1.414
JCVI_23180	1.139	moderately similar to (232)AT3G14830| Symbols: | similar to unknown protein [Arabidopsis thaliana] (TAIR:AT1G53450.2); similar to	
JCVI_26795	1.137	weakly similar to (198)AT1G49720| Symbols: ABF1 | ABF1 (ABSCISIC ACID RESPONSIVE ELEMENT-BINDING FACTOR 1); DN	
JCVI_22858	1.136	no original description	
EV134888	1.134	no similarity	
EV195457	1.130	no similarity	
ES999886	1.130	moderately similar to (208)AT3G05170| Symbols: | phosphoglycerate/bisphosphoglycerate mutase family protein | chr3:1466744-14682	
CD819009	1.129	no similarity	
JCVI_35057	1.127	highly similar to (568)AT1G22650| Symbols: | beta-fructofuranosidase, putative / invertase, putative / saccharase, putative / beta-fructosi	
EX127869	1.127	weakly similar to (154)AT1G48960| Symbols: | universal stress protein (USP) family protein | chr1:18116220-18117218 FORWARD [2	-1.472
JCVI_30494	1.125	no original description	-1.577
EE530664	1.123	moderately similar to (201)AT1G17500| Symbols: | ATPase, coupled to transmembrane movement of ions, phosphorylative mechanism |	-1.814
JCVI_32563	1.123	no original description	
JCVI_11382	1.123	moderately similar to (226)AT2G41475| Symbols: | similar to embryo-specific protein-related [Arabidopsis thaliana] (TAIR:AT5G6220(	
JCVI_35949	1.121	no original description	
EV182586	1.120	moderately similar to (239)AT3G57480| Symbols: | zinc finger (C2H2 type, AN1-like) family protein | chr3:21289060-21290096 REVE	-1.462
EX015789	1.119	no similarity	-1.227
JCVI_994	1.119	moderately similar to (439)AT5G63770| Symbols: ATDGK2 | ATDGK2 (DIACYLGLYCEROL KINASE 2) | chr5:25536981-25539630	
JCVI_42337	1.117	no original description	
EV012934	1.116	no similarity	
JCVI_9429	1.116	moderately similar to (354)AT1G71940| Symbols: | similar to unknown protein [Arabidopsis thaliana] (TAIR:AT4G09580.1); similar to	-1.501
JCVI_19356	1.115	moderately similar to (298)AT4G30996| Symbols: | similar to unknown protein [Arabidopsis thaliana] (TAIR:AT2G24290.1); similar to	
JCVI_39128	1.115	moderately similar to (324)AT1G61820| Symbols: BGLU46 | BGLU46; hydrolase, hydrolyzing O-glycosyl compounds | chr1:22840372-	
ES944527	1.114	weakly similar to (178)AT5G22770| Symbols: ALPHA-ADR | ALPHA-ADR (ALPHA-ADAPTIN); binding / protein binding / protein tra	
JCVI_3682	1.114	moderately similar to (295)AT1G05720| Symbols: | selenoprotein family protein | chr1:1717676-1718848 REVERSE no original descrip	-1.199
EV212519	1.114	moderately similar to (383)AT5G54670| Symbols: KATC, ATK3 | ATK3 (ARABIDOPSIS THALIANA KINESIN 3); microtubule moto	-1.252
EE469538	1.113	no similarity	
JCVI_1008	1.110	weakly similar to (133)AT5G05060| Symbols: | similar to cysteine protease inhibitor [Arabidopsis thaliana] (TAIR:AT5G05040.1); cont	
JCVI_19869	1.109	very weakly similar to (82.4)AT1G15970| Symbols: | methyladenine glycosylase family protein | chr1:5486538-5488488 REVERSE no c	
JCVI_10993	1.108	moderately similar to (212)AT1G21370| Symbols: | similar to unnamed protein product [Vitis vinifera] (GB:CAO60871.1) | chr1:74842	-1.158
EE548698	1.106	no similarity	
EE517454	1.101	weakly similar to (157)AT1G76850| Symbols: SEC5A | SEC5A (EXOCYST COMPLEX COMPONENT SEC5) | chr1:28852907-28859	-1.351
JCVI_28779	1.101	no original description	
JCVI_20123	1.101	highly similar to (666)AT2G46260| Symbols: | BTB/POZ domain-containing protein | chr2:19003184-19005536 FORWARD no origina	
JCVI_7667	1.100	weakly similar to (147)AT4G32030| Symbols: | unknown protein | chr4:15490909-15493013 FORWARD no original description	
JCVI_23139	1.100	moderately similar to (273)AT4G26400| Symbols: | zinc finger (C3HC4-type RING finger) family protein | chr4:13344962-13346032 R	
JCVI_7262	1.100	moderately similar to (278)AT3G50910| Symbols: | similar to unknown protein [Arabidopsis thaliana] (TAIR:AT5G66480.1); similar to	-1.176
JCVI_13781	1.097	moderately similar to (389)AT2G29890| Symbols: ATVLN1, VLN1 | VLN1 (VILLIN 1); actin binding | chr2:12751674-12756551 FOR	
JCVI_24820	1.097	moderately similar to (315)AT3G55020| Symbols: | RabGAP/TBC domain-containing protein | chr3:20400255-20405690 REVERSE no	
JCVI_28421	1.094	weakly similar to (199)AT2G24220| Symbols: ATPUP5 | ATPUP5 (Arabidopsis thaliana purine permease 5); purine transmembrane trans	
CD828258	1.094	weakly similar to (160)AT3G22520| Symbols: | similar to unknown protein [Arabidopsis thaliana] (TAIR:AT4G14840.1); similar to unn	
JCVI_18474	1.091	moderately similar to (430)AT5G14790| Symbols: | binding | chr5:4784061-4785511 FORWARD no original description	
JCVI_8668	1.091	highly similar to (787)AT2G01720| Symbols: | ribophorin I family protein | chr2:317192-320015 REVERSE no original description	
EE424994	1.084	weakly similar to (144)AT3G57330| Symbols: ACA11 | ACA11 (AUTOINHIBITED CA2+-ATPASE 11); calcium-transporting ATPase	-1.221
JCVI_28702	1.083	moderately similar to (384)AT1G07310| Symbols: | C2 domain-containing protein | chr1:2247774-2248832 REVERSE no original desc	
JCVI_30407	1.081	no original description	-1.566
JCVI_25436	1.081	weakly similar to (106)AT2G31305| Symbols: | similar to Protein phosphatase inhibitor [Medicago truncatula] (GB:ABN09808.1); conta	
CD820097	1.080	no similarity	
JCVI_1143	1.080	highly similar to (503)AT5G02500| Symbols: HSP70-1, AT-HSC70-1, HSC70, HSC70-1 | HSC70-1 (heat shock cognate 70 kDa protein	
JCVI_20520	1.080	highly similar to (506)AT5G18280| Symbols: APY2, ATAPY2 | ATAPY2 (APYRASE 2) | chr5:6050801-6054025 REVERSEmoderate	
JCVI_35507	1.080	no original description	-1.199
JCVI_22642	1.074	moderately similar to (466)AT1G52360| Symbols: | coatomer protein complex, subunit beta 2 (beta prime), putative | chr1:19502951-195	
CN727625	1.073	weakly similar to (181)AT3G52240| Symbols: | similar to unnamed protein product [Vitis vinifera] (GB:CAO46543.1) | chr3:19383880-	-1.327
JCVI_19130	1.072	highly similar to (701)AT2G38000| Symbols: | chaperone protein dnaJ-related | chr2:15910393-15912354 REVERSE no original descrip	
JCVI_17785	1.071	moderately similar to (294)AT4G17060| Symbols: | unknown protein | chr4:9593740-9594672 REVERSE no original description	
EX037128	1.065	weakly similar to (172)AT2G03480| Symbols: | dehydration-responsive protein-related | chr2:1051506-1054087 FORWARD [21811]	
JCVI_2718	1.065	highly similar to (614)AT1G30900| Symbols: | vacuolar sorting receptor, putative | chr1:10997256-11000524 FORWARDmoderately sin	
EV088313	1.063	moderately similar to (215)AT5G44240| Symbols: | haloacid dehalogenase-like hydrolase family protein | chr5:17834846-17840825 FOR	
EV043421	1.057	moderately similar to (240)AT4G17483| Symbols: | palmitoyl protein thioesterase family protein | chr4:9747387-9748904 REVERSE [2	
ES907481	1.057	weakly similar to (153)AT5G64440| Symbols: ATFAAH | ATFAAH (ARABIDOPSIS THALIANA FATTY ACID AMIDE HYDROLA	
EV100393	1.057	no similarity	
JCVI_28120	1.056	moderately similar to (244)AT5G56230| Symbols: | prenylated rab acceptor (PRA1) family protein | chr5:22775994-22776554 REVERS	-1.656
JCVI_24114	1.055	no original description	-1.570
BG543778	1.053	weakly similar to (140)NIA1_BRANA [8791]	
EX108549	1.052	weakly similar to (123)AT4G33690| Symbols: | similar to hypothetical protein [Vitis vinifera] (GB:CAN61243.1) | chr4:16175201-1617	
JCVI_38513	1.052	no original description	-1.397
JCVI_21706	1.051	weakly similar to (147)AT2G37035| Symbols: | similar to unnamed protein product [Vitis vinifera] (GB:CAO18172.1) | chr2:15562453-	
EX100581	1.050	moderately similar to (321)AT1G60890| Symbols: | phosphatidylinositol-4-phosphate 5-kinase family protein | chr1:22416117-22420003	-1.063
EE533363	1.048	moderately similar to (298)AT4G31150| Symbols: | endonuclease V family protein | chr4:15143913-15145475 REVERSE [20175]	
EE472468	1.048	weakly similar to (140)AT4G13700| Symbols: ATPAP23, PAP23 | ATPAP23/PAP23 (purple acid phosphatase 23); acid phosphatase/ pr	
CD825020	1.047	very weakly similar to (95.1)AT1G24764| Symbols: ATMAP70-2 | ATMAP70-2 (microtubule-associated proteins 70-2); microtubule bin	
JCVI_6591	1.046	highly similar to (652)AT2G01970| Symbols: | endomembrane protein 70, putative | chr2:452196-454818 REVERSE no original descrip	
JCVI_16049	1.042	moderately similar to (442)AT1G49710| Symbols: ATFUT12, FUCTB, FUCT2, FUT12 | FUT12 (fucosyltransferase 12); fucosyltransfer	-1.167
JCVI_17675	1.040	moderately similar to (416)AT1G73250| Symbols: ATFX, GER1 | ATFX/GER1 (GDP-4-KETO-6-DEOXYMANNOSE-3,5-EPIMERAS	-1.279
JCVI_18062	1.036	highly similar to (589)AT5G65950| Symbols: | binding | chr5:26397516-26402186 FORWARD no original description	-1.128
JCVI_31425	1.036	no original description	
JCVI_33008	1.034	moderately similar to (202)AT5G11710| Symbols: | (EPSIN1); binding | chr5:3772982-3776317 FORWARD no original description	

JCVI_7141	1.032	very weakly similar to (89.0)AT3G27100| Symbols: | similar to unknown [Picea sitchensis] (GB:ABK23311.1) | chr3:9995969-9997160	-1.499
JCVI_39658	1.032	weakly similar to (126)AT5G22770| Symbols: ALPHA-ADR | ALPHA-ADR (ALPHA-ADAPTIN); binding / protein binding / protein tra	
JCVI_3778	1.029	very weakly similar to (87.0)AT2G37980| Symbols: | similar to unknown protein [Arabidopsis thaliana] (TAIR:AT5G01100.1); similar to	
EE548097	1.029	weakly similar to (143)AT5G64880| Symbols: | unknown protein | chr5:25949579-25951100 FORWARD [20128] 45 770 770	
JCVI_41652	1.028	weakly similar to (104)AT5G63990| Symbols: | 3'(2'),5'-bisphosphate nucleotidase, putative / inositol polyphosphate 1-phosphatase, puta	-1.201
JCVI_12190	1.027	moderately similar to (275)AT5G25790| Symbols: | transcription factor | chr5:8977236-8979184 REVERSE no original description	
JCVI_25085	1.026	highly similar to (729)AT5G05170| Symbols: CESA3, IXR1, ATCESA3, ATH-B, CEV1 | CESA3 (CELLULOSE SYNTHASE 3); cellu	
EX111290	1.022	moderately similar to (325)AT5G55990| Symbols: ATCBL2, CBL2 | CBL2 (calcineurin B-like protein 2); calcium ion binding | chr5:226	
JCVI_18190	1.021	moderately similar to (238)AT5G11950| Symbols: | Encodes a protein of unknown function. It has been crystallized and shown to be str	-1.142
JCVI_9090	1.009	moderately similar to (213)AT3G60520| Symbols: | zinc ion binding | chr3:22372726-22373442 REVERSE no original description	-1.020
JCVI_20486	1.007	moderately similar to (474)AT4G18030| Symbols: | dehydration-responsive family protein | chr4:10012862-10015279 REVERSE no ori	
EV069203	1.007	moderately similar to (260)AT3G63460| Symbols: | WD-40 repeat family protein | chr3:23441984-23448216 REVERSE [21443]	
DY014787	1.004	weakly similar to (170)AT1G68720| Symbols: | cytidine/deoxycytidylate deaminase family protein | chr1:25808210-25812483 FORWAI	
JCVI_16871	1.004	moderately similar to (403)AT5G11890| Symbols: | similar to unknown protein [Arabidopsis thaliana] (TAIR:AT1G17620.1); similar to	-1.658
EV134955	1.001	no similarity	
EE440296	1.000	moderately similar to (340)AT1G60780| Symbols: | clathrin adaptor complexes medium subunit family protein | chr1:22372954-2237555	-1.067
JCVI_42211	0.996	moderately similar to (340)AT1G02970| Symbols: WEE1 | WEE1 (ARABIDOPSIS WEE1 KINASE HOMOLOG); kinase/ protein kinas	-1.226
EE496526	0.964	no similarity	-0.678
JCVI_24744	0.963	highly similar to (810)AT4G13460| Symbols: SUVH9, SDG22, SET22 | SUVH9 (SU(VAR)3-9 HOMOLOG 9); histone-lysine N-methy	-1.054
ES997552	0.959	weakly similar to (155)AT1G49340| Symbols: ATPI4K ALPHA | ATPI4K ALPHA (Arabidopsis thaliana phosphatidylinositol 4-kinase a	-1.163
EV206682	0.948	moderately similar to (209)AT2G41900| Symbols: | zinc finger (CCCH-type) family protein | chr2:17498430-17500580 FORWARD [21	-1.079
ES959683	0.938	no similarity	
JCVI_4034	0.938	moderately similar to (424)AT1G20780| Symbols: | armadillo/beta-catenin repeat protein-related / U-box domain-containing protein | chr	
JCVI_15705	0.930	moderately similar to (405)AT3G11130| Symbols: | clathrin heavy chain, putative | chr3:3482581-3491673 REVERSE no original descr	
EL591273	0.878	weakly similar to (122)AT2G27880| Symbols: | argonaute protein, putative / AGO, putative | chr2:11878565-11883789 FORWARD [20	
JCVI_30185	0.809	no original description	
CX191119	0.772	no similarity	-0.992
JCVI_25041	0.524	no original description	-1.622
