# Supplementary material for: Influence of Light and Temperature on Gene Expression Leading to Accumulation of Specific Flavonol Glycosides and Hydroxycinnamic Acid Derivatives in Kale (Brassica oleracea var. sabellica)
Source: Front Plant Sci. 2016 Mar 30;7:326. doi: 10.3389/fpls.2016.00326 (PMC4812050; doi:10.3389/fpls.2016.00326)
Supplement: Supplementary file 5 [file Table5.PDF]

Supplementary Table 5. Main mass peaks of the selected compounds from kale leaves.

Compound	RT	Main mass peaks in *m/z*		
		MS	MS^2	MS^3
Quercetin glycosides as quercetin-3-O-glucoside equivalents				
quercetin-3-*O*-sophoroside-7-*O*-D-glucoside	**18,8**	**787**	**625**	**301**
quercetin-3-*O*-feruloyl-sophoroside-7-*O*-D-glucoside	**32,9**	**963**	**801**	**625**
quercetin-3-*O*-hydoxyferuloyl-sophoroside-7-*O*-D-glucoside	**22,6**	**979**	**817**	**625**
quercetin-3-*O*-sinapoyl-sophoroside-7-*O*-D-glucoside quercetin-3-*O*-sophoroside-7-*O*-sinapoyl-diglucoside	**31,2**	**993/ 1155**	**831/ 949**	**625**
quercetin-3-*O*-disinapoyl-triglucoside-7-*O*-D-glucoside	**97,7**	**1361**	**1199**	**993**
Kaempferol glycosides as kaempferol-3-O-glucoside equivalents				
kaempferol-feruloyl-3-*O*-sophoroside	**85,2**	**785**	**609**	**285**
kaempferol-hydroxyferuloyl-3-*O*-sophoroside	**58,8**	**801**	**609**	**285**
kaempferol-sinapoyl-3-*O*-sophoroside	**72,5**	**815**	**609**	**285**
kaempferol-3-*O*-sophoroside-7-*O*-D-glucoside	**22,9**	**771**	**609**	**285**
kaempferol-3-*O*-caffeoyl-sophoroside-7-*O*-D-glucoside	**30,1**	**933**	**771**	**609**
kaempferol-3-*O*-feruloyl-sophoroside-7-*O*-D-glucoside	**38,3**	**947**	**785**	**609**
kaempferol-3-*O*-hydoxyferuloyl-sophoroside-7-*O*-D-glucoside	**27,1**	**963**	**801**	**609**
kaempferol-3-*O*-sinaopyl-sophoroside-7-*O*-D-glucoside	**36,2**	**977**	**815**	**609**
kaempferol-3-*O*-hydoxyferuloyl-sophoroside-7-*O*-diglucoside	**29,0**	**1125**	**801**	**609**
kaempferol-3-*O*-sinapoyl-sophoroside-7-*O*-diglucoside	**36,8**	**1139**	**815**	**609**
kaempferol-3-*O*-disinapoyl-triglucoside-7-*O*-D-glucoside	**108,0**	**1345**	**1183**	**977**
Caffeoylquinic acid and hydroxycinnamic acid glycosides as caffeoylquinic acid equivalents				
Caffeoylquinic acid	**11,9**	**353**	**191**	
Disinapoyl-gentiobiose	**127,3**	**753**	**529**	**223**
Sinapoyl-Feruloyl-gentiobiose	**146,2**	**723**	**499**	**193**

Main mass peaks in *m/z* of the selected compounds from kale extracted from the paper Schmidt, S., Zietz, M., Schreiner, M., Rohn, S., Kroh, L.W., and Krumbein, A. (2010b). Identification of complex, naturally occurring flavonoid glycosides in kale (Brassica oleracea var. sabellica) by high-performance liquid chromatography diode-array detection/electrospray ionization multi-stage mass spectrometry. *Rapid Communications in Mass Spectrometry* 24, 2009-2022.
